# Identification of Novel Choroidal Neovascularization-Related Genes Using Laplacian Heat Diffusion Algorithm

**DOI:** 10.1155/2021/2295412

**Published:** 2021-09-06

**Authors:** Minjie Sheng, Haiying Cai, Ming Cheng, Jing Li, Jian Zhang, Lihua Liu

**Affiliations:** ^1^Department of Ophthalmology, Yangpu Hospital, Tongji University School of Medicine, 450 Tengyue Road, Shanghai 200090, China; ^2^Department of Ophthalmology, Shanghai General Hospital, Shanghai Jiao Tong University School of Medicine, shanghai 200080, China; ^3^Shanghai Key Laboratory of Ocular Fundus Diseases, Shanghai 200080, China; ^4^Shanghai Engineering Center for Visual Science and Photomedicine, Shanghai 200080, China; ^5^National Clinical Research Center for Eye Diseases, Shanghai 20080, China; ^6^Shanghai Engineering Center for Precise Diagnosis and Treatment of Eye Diseases, Shanghai 20080, China

## Abstract

Choroidal neovascularization (CNV) is a type of eye disease that can cause vision loss. In recent years, many studies have attempted to investigate the major pathological processes and molecular pathogenic mechanisms of CNV. Because many diseases are related to genes, the genes associated with CNV need to be identified. In this study, we proposed a network-based approach for identifying novel CNV-associated genes. To execute such method, we first employed a protein-protein interaction network reported in STRING. Then, we applied a network diffusion algorithm, Laplacian heat diffusion, on this network by selecting validated CNV-related genes as the seed nodes. As a result, some novel genes that had unknown but strong relationships with validated genes were identified. Furthermore, we used a screening procedure to extract the most essential genes. Eleven latent CNV-related genes were finally obtained. Extensive analyses were performed to confirm that these genes are novel CNV-related genes.

## 1. Introduction

Choroidal neovascularization (CNV) is a typical pathogenic process that refers to the abnormal creation of blood vessels specifically in the choroid layer of the eye. As a severe pathogenesis of one subtype of age-related macular degeneration (AMD), CNV can be clinically concomitant with various ocular symptoms such as extreme myopia and malignant myopic degeneration. According to the recent epidemiological statistics provided by *Lancet*, more than 6 million people around the world suffered from AMD in 2015 [1]. Based on another independent survey, the prevalence of CNV-associated AMD was found to be 1.2% of all adults aged 43–86 years [2], indicating that CNV may be one of the major causes of vision loss.

As mentioned above, CNV is a major threat to visual health, especially in elderly people around the world. Therefore, for centuries, scientists have attempted to determine the major pathological processes and molecular pathogenic mechanisms of CNV [3, 4]. However, the detailed and comprehensive mechanisms of CNV have not been fully elucidated. According to existing literatures, the major pathogenic mechanisms of CNV can be attributed to the imbalance of antiangiogenic factors and angiogenic factors [5, 6]. The imbalance of these factors in the choroid may promote vasculogenesis and angiogenesis pathologically related to CNV [6]. In terms of regulators, PEDF (pigment epithelium-derived factor) [6] and VEGF (vascular endothelial growth factor), which are antiangiogenic and typical angiogenic factors, respectively) [5] have both been confirmed to contribute to the initiation and progression of CNV. However, the factors or initiators that drive the abnormal biological functions of PEDF and VEGF have not been confirmed. Hypoxia [7], high glucose [8], protein kinase C activation [9], advanced glycation end products [10], reactive oxygen species [11], activated oncogenes [12], and abnormal cytokine production [13] may all contribute to the pathogenesis and clinical symptoms of CNV.

Although the pathogenesis of the diseases we have discussed is complicated, we can still simply cluster all the potential pathogenic factors into two groups: genetic factors and environmental factors [3, 4]. In this study, we computationally investigated the genetic pathogenesis of CNV. According to recent publications, various genetic factors have been confirmed to contribute to CNV. Abnormal angiogenesis and antiangiogenesis are two major pathogenic processes in such disease [5, 6]. Recent publications revealed that various genes related to angiogenesis and antiangiogenesis may directly participate in the pathogenesis of CNV. *VEGF* [14] and *FGF2* [15] are two typical genes associated with angiogenesis. In 2009, these genes have been confirmed to be related to CNV and regulate its rate of progression [16]. Besides these genes, another functional gene called *CFI*, which is related to extreme myopia, has also been reported to contribute to CNV pathogenesis [17, 18], revealing the complicated genetic basis of CNV. Other functional genes associated with cell proliferation, such as *RELA* [19], *NFKB1* [20, 21], and *RELB* [19], have all been reported to promote abnormal angiogenesis during the initiation and progression of CNV.

For decades, scientists have attempted to reveal the comprehensive genetic background of CNV. However, identifying and validating CNV-associated genes one by one is quite expensive and time consuming. In recent years, with the development of high-throughput sequencing, bioinformatics algorithms have provided us a novel and more effective approach for identifying CNV-associated genes. In 2016, a systematic prediction [22] based on all the identified CNV-related genes, protein–protein interaction (PPI) network, and shortest path algorithm identified various genes associated with CNV, including *ANK1*, *ITGA4*, and *CD44*. Most of these genes have already been identified to contribute to abnormal angiogenesis or antiangiogenesis in the choroid [22], validating the efficacy and accuracy of computational prediction on disease-associated genes. Therefore, in this study, we introduced a novel computational method called *Laplacian heat diffusion* (LHD) [23] to further explore the pathogenic factors of CNV. This study not only identified potential CNV-associated genes but also revealed the detailed pathogenesis of CNV.

## 2. Materials and Methods

### 2.1. CNV-Associated Genes

Genes associated with AMD were first obtained from a previous study [24]. In detail, we downloaded the “Additional file 3” in such study, which contained these genes. Then, according to “Additional file 5”, genes in CNV up or CNV down modules were picked up, accessing 37 CNV-associated genes (Table S1). These genes were further converted to Ensembl gene IDs to be consistent with the protein IDs in the PPI network from the STRING database [25]. These genes comprised a seed gene set *S*.

### 2.2. PPIs

In general, proteins interact with each other to regulate biological process; thus, they share similar biological functions. Based on this assumption, many studies have been devoted to infer protein functions. Therefore, potential CNV-associated genes can be identified from the known CNV-associated genes and their interaction network.

We downloaded 4,274,001 human protein-protein interactions (PPIs) for 19,247 proteins from STRING (https://www.string-db.org/, version 10) [26]. These interactions were derived from genomic context predictions, high-throughput lab experiments, (conserved) coexpression, automated text mining, and previous knowledge in databases. Thus, PPIs reported in STRING can widely measure the associations of proteins compared with those in some other databases [27, 28], in which PPIs were only determined by solid experiments. For each PPI, both proteins are represented by Ensembl IDs, and a score ranging from 150 to 999 is assigned. A high score indicates that the corresponding interaction is supported by high-quality evidence. The interaction score between two proteins (*P*_1_ and *P*_2_) was denoted as *I*(*P*_1_, *P*_2_). Using the abovementioned data, we can construct a PPI network consisting of 19,247 nodes and 4,274,001 edges, which connects two nodes with interaction score as the weight if and only if two proteins interact. The PPI network is denoted as *G*. Such PPI network has been widely used in many researches [29–37].

### 2.3. Laplacian Heat Diffusion

Nowadays, network methods are more and more popular to deal with different biological and medical problems [30, 32, 36, 38–41]. This study also adopted a powerful network method, LHD algorithm. As a type of network diffusion method, heat diffusion follows some rules to transmit heat on the seed nodes to surrounding nodes in the network. The heat on a node indicates its connections to seed nodes. In this study, the LHD algorithm [23] was applied to search for novel CNV-related genes, which was a heat diffusion process on a Laplacian matrix constructed from protein-protein network.

Given a PPI network *G*, we can first construct its adjacent matrix *A* based on the edge weights. Then, we normalize it column wisely as follows:

(1) $$\begin{array}{c} A^{\prime }\left[i,j\right]=\frac{A\left[i,j\right]}{\sum_{k=1}^{n}A\left[k,j\right]}, \end{array}$$

where *i* is the column index of 19,247 nodes in *G* and *j* is the index of CNV-related genes. Each column in *A*′ was a 19,247-dimensional vector. Each element was the heat of a node in the network *G*. Initially, the component in *A*′ corresponding to 37 CNV-related genes was configured to be 1/37; other components were set to 0. Then, the values of each vector were updated as follows:

(2) $$\begin{array}{c} H_{t}\left[i\right]=H_{0}\left[i\right]\exp\left(-\lambda_{i}t\right), \end{array}$$

where *H*_*t*_ is the heat distribution at time *t* and *λ*_*i*_ is the *i*th eigenvalue of matrix *A*′. We updated the vectors until the heat distribution vectors at two consecutive time points change as small as a defined threshold. After the diffusion process, each node was assigned a heat value. A larger heat value indicates that the node is more important. Thus, we selected nodes with heat values greater than the defined cutoff and mapped those nodes back to the corresponding genes.

In this study, we used the LHD algorithm (https://CRAN.R-project.org/package=diffusr) to perform the analysis with default parameters on the PPI network *G*.

### 2.4. Postprocessing of CNV-Related Candidate Genes

According to the LHD-based method, we can obtain a large number of candidate CNV-related genes. However, some of them are essential genes, while others are nonessential genes. A three-stage method was applied to select the essential genes by integrating other biological information: (1) *Z*-score based on permutation test to exclude false positives, (2) maximum interaction score (MIS) based on PPI information to exclude genes with few connections to the validated CNV-related genes, and (3) maximum function score (MFS) based on biological function annotation information to filter functional genes.

#### 2.4.1. *Z*-Score

To evaluate the significance of the produced heat values, we randomly sampled 1000 gene sets and calculated the mean and standard deviation of these heat values. Then, we calculated the *Z*-score for all CNV-related candidate genes. In detail, 1000 gene sets with a size of 37 were randomly generated. For each gene set, we performed the LHD algorithm on the PPI network *G* by using it as the seed set. Then, each gene *g* was assigned a heat value. The above process was run for the produced 1000 gene sets. Each *g* received 1000 heat values and a real heat value based on 37 validated CNV-related genes. We calculated the measurement *Z*-score as follows:

(3) $$\begin{array}{c} Z‐\text{score}\left(g\right)=\frac{h-\bar{h}}{\text{sd}}, \end{array}$$

where *h* is the real heat value of gene *g* and $\bar{h}$ and sd are the mean and standard deviation of 1000 heat values of the 1000 randomly produced gene sets, respectively. The higher the *Z*-score of one gene is, the more likely it is a real CNV-related gene. In this study, we selected genes with *Z*-score greater than 1.96.

#### 2.4.2. MIS

After the permutation test, some CNV-related candidate genes were further verified to have strong associations with the validated CNV-related genes. In general, interacting proteins always exhibit similar functions. Based on this observation, we calculated MIS as follows:

(4) $$\begin{array}{c} \text{MIS}\left(g\right)=\max\left\{I\left(g,g^{\prime }\right)\;|\;g^{\prime }\text{ is a validated CNV}‐\text{related gene}\right\}, \end{array}$$

where *I*(*g*, *g*′) is the interaction score between two genes from the STRING database. A high MIS value indicates that this gene is strongly connected to the validated CNV-related genes; thus, it is more likely to be true CNV-associated gene. Here, we set a threshold of 900 (the highest confidence score in the STRING database) to filter out genes with low MIS values.

#### 2.4.3. MFS

To be CNV-related genes, they must highly contribute to certain biological processes involved in CNV. To further select more reliable CNV-related candidate genes, Gene Ontology (GO) terms and Kyoto Encyclopedia of Genes and Genomes (KEGG) [42] pathways were used. We extracted important candidate CNV-related genes with similar GO terms and KEGG pathways to validate CNV-related genes. The enrichment theory [43, 44] was applied to estimate the relationships between genes and GO/KEGG pathways. It encodes a gene as a vector. The relationship between two genes can be calculated as follows:

(5) $$\begin{array}{c} Q\left(g,g^{\prime }\right)=\frac{E\left(g\right)\cdot E\left(g^{\prime }\right)}{\left\|E\left(g\right)\right\|\cdot \left\|E\left(g^{\prime }\right)\right\|}, \end{array}$$

where *E*(*g*) is the column vector obtained according to enrichment theory.

Similarly, for each gene, MFS(*g*) was calculated as follows:

(6) $$\begin{array}{c} \text{MFS}\left(g\right)=\max\left\{Q\left(g,g^{\prime }\right)\;|\;g^{\prime }\text{ is a validated CNV}‐\text{related gene}\right\}. \end{array}$$

The higher the MFS of one gene is, the more GO/KEGG pathways it shares with the validated CNV-related genes. The final candidate CNV-related genes were extracted with an MFS value greater than a defined cutoff of 0.9.

## 3. Results

**Figure 1:**
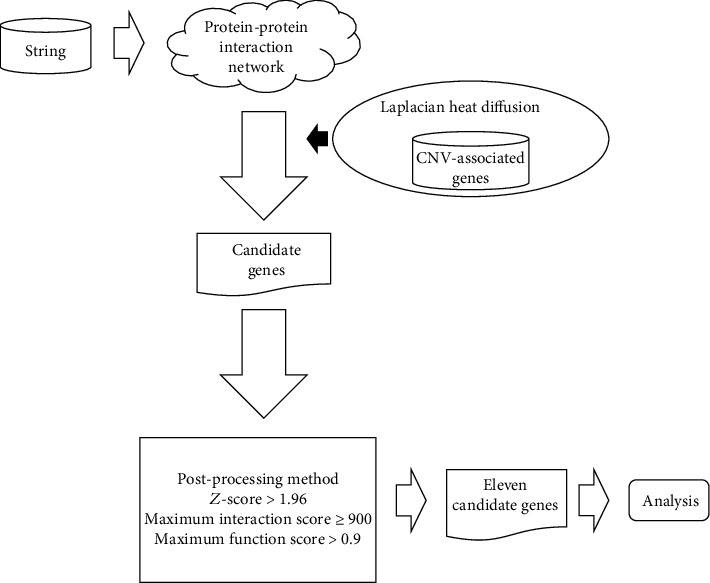
Entire procedures to identify novel choroidal neovascularization- (CNV-) related genes. A protein-protein interaction network reported in STRING is employed. Laplacian heat diffusion (LHD) with validated CNV-related genes as seed nodes is applied to such network for extracting raw candidate genes. They are further filtered by a postprocessing method, resulting in eleven candidate genes. These genes were extensively analyzed.

In this study, we presented a computational approach to infer novel CNV-associated genes using the LHD-based method. The entire procedures are illustrated in Figure 1. This approach collected verified CNV-related genes, which were extrapolated to identify novel candidate genes on the PPI network using Laplacian heat diffusion. Next, these identified candidate genes were further screened to filter out false positive genes that are not associated with any CNV-related biological process.

**Figure 2:**
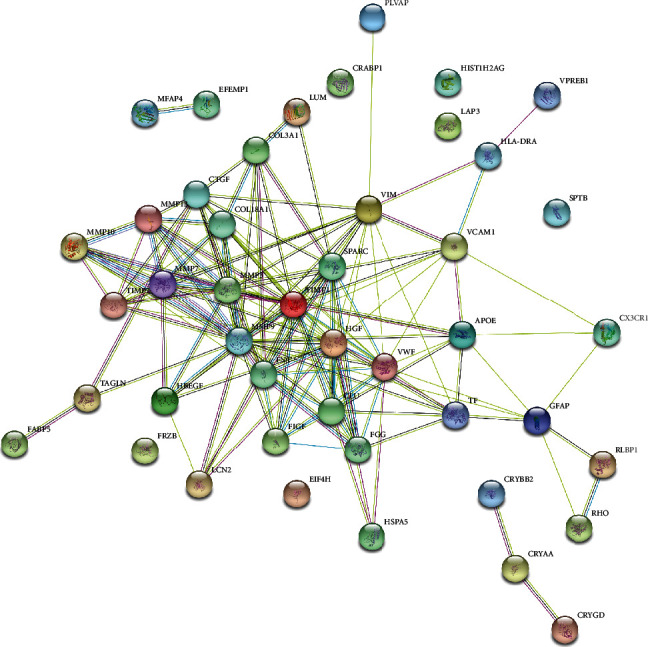
Interaction network between the 11 candidate genes and verified 37 CNV-related genes.

We first selected genes with a heat value > *e* − 10, and a total of 19,218 genes were obtained. Then, these genes were evaluated using the permutation test with 1000 randomly generated sets. We selected genes with a *Z*-score greater than 1.96 and obtained a list of 153 genes. We further filtered out genes with fewer connections to the validated CNV-related genes by MIS score. We kept genes with MIS value greater than 900 and obtained 27 genes. Finally, for each of the 27 genes, we calculated the MFS and selected genes with an MFS value greater than 0.9, resulting in a final list of 11 CNV-related genes, which is totally different from previous discoveries [22]. The selected numbers of putative genes in different steps of LHD are shown in Table 1, and the detailed information of the 11 final candidate CNV-associated genes is listed in Table 2. The interaction network between the 11 candidate genes and 37 verified CNV-related genes is shown in Figure 2. All measurements mentioned above are listed in sheets 1–4 of Table S2.

## 4. Discussion

**Figure 3:**
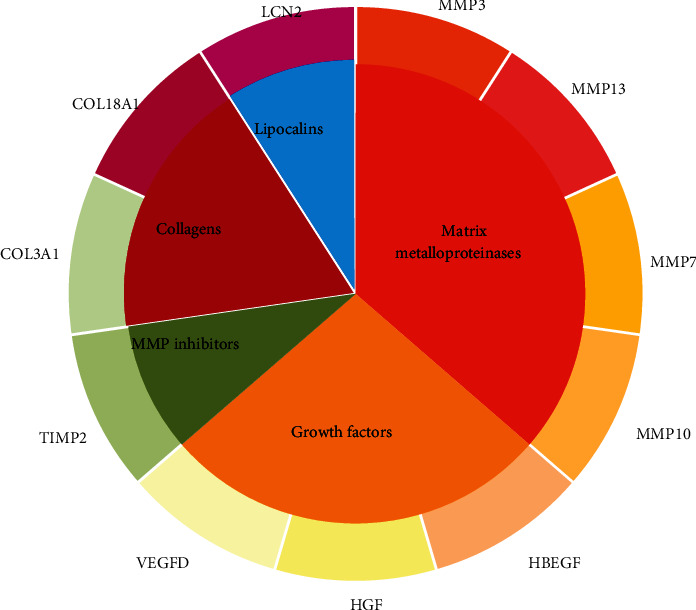
Classification of eleven candidate CNV-associated genes.

As we have analyzed above, we applied a novel computational method named *Laplacian heat diffusion* [45] to identify potential CNV-related genes based on the existing PPI network provided in STRING [25]. According to such algorithm and the database, we screened out eleven functional genes that may directly or indirectly participate in the pathogenesis of CNV. To validate the efficacy and accuracy of our newly applied computational method, we performed a systematic datamining on the biological functions and CNV relevance of all predicted genes. The predicted genes have been validated by recent publications. The detailed analysis on each gene can be seen below. For a clear description, we classified these genes into some classes, which is illustrated in Figure 3.

### 4.1. Matrix Metalloproteinases (MMPs)

*MMP3* (ENSP00000299855), which ranks the highest in the prediction list, has been predicted to be related to the pathogenesis of CNV. Generally, it has been widely reported to contribute to the activation of procollagenase [46] and matrix remodeling [47]. In terms of its potential pathogenic functions in CNV, this gene has been confirmed to act abnormally in the choroidal neovascular membranes, implying its pathogenic potential [48]. Further studies on the contribution of hypoxia to CNV confirmed that our predicted gene *MMP3* may contribute to hypoxia-induced apoptosis and secretion of proangiogenic factors in the choroid under hypoxia microenvironment, which further initiates CNV [49]. Therefore, our predicted gene *MMP3* may functionally be a potential driving factor for CNV, demonstrating the accuracy of our prediction result. Apart from *MMP3*, three other components of the MMP family, namely, *MMP13* (ENSP00000260302), *MMP7* (ENSP00000260227), and *MMP10* (ENSP00000279441), have also been predicted to contribute to the pathogenesis of CNV in our prediction list with a high rank. With similar biological functions as *MMP3*, all of such three genes (*MMP13*, *MMP7*, and *MMP10*) have been reported to participate in the abnormal angiogenesis of choroidal tissues, validating their specific contribution to CNV. In 2011, a study [50] on CNV in a mouse model confirmed that the deficiency of *MMP13* contributed to the impairment of neovascularization formation in choroid tissues, and such pathogenesis could be restored by injecting mesenchymal cells secreting *MMP13*, validating the specific role of this gene during CNV initiation and progression. As for *MMP7*, basal laminar and linear deposits are typical complications of CNV, contributing to the constitution of the CNV microenvironment [51, 52]. A recent study [53] on the typical basal laminar and linear deposits of CNV confirmed that *MMP7* together with its homologue *MMP13* may contribute to CNV by regulating the inflammatory processes in the microenvironment of choroidal tissues. Furthermore, *MMP10* has also been validated by recent publications. Although no reports connected *MMP10* and CNV directly, the specific contribution of all metalloproteinases including *MMP10* on choroidal microenvironment remodeling and inflammation mediation implies the specific biological function of MMP10 during the progression of CNV [54].

### 4.2. Growth Factors

*HBEGF* (ENSP00000230990) has also been predicted to contribute to the progression of CNV. As a typical growth factor, *HBEGF* participates in the ERBB2 signaling pathway and interacts with functional genes such as EGFR and ERBB4 [55, 56]. A recent study confirmed that *HBEGF* may affect the production and biological functions of *VEGF* in CNV [57]. Therefore, although no direct reports confirmed the detailed biological function of *HBEGF* in CNV, this gene may interact with *VEGF* and play a crucial pathogenic role during the progression of CNV. Another functional growth factor encoding gene *HGF* (ENSP00000222390) has also been predicted to contribute to the pathogenesis of CNV. Generally, the binding of *HGF* to its target receptor (hepatocyte growth factor) contributes to the regulation of cell growth, cell motility, and morphogenesis in various cell and tissue subtypes [58, 59]. As for its unique pathogenic contribution to CNV, a paired experimental study [60] on CNV confirmed that compared with normal tissues, the pathogenic tissues of the choroid during CNV initiation and progression have different expression profiling of growth factors including *VEGF*, *HGF*, and *FGF*, implying the potential pathogenic role of *HGF* in such disease. In 2011, a specific study on the biological and pathogenic functions of cytokines in CNV confirmed that *HGF* has a mitogenic effect on choroidal cells, promoting neovascularization processes [61]. Therefore, such gene may be a potential CNV-associated gene. As the next predicted growth factor in the predicted list of genes, *VEGFD* (ENSP00000297904) has been widely reported to be a member of the platelet-derived growth factor family. This gene has been reported to promote angiogenesis [62], lymph angiogenesis [62], and endothelial cell growth [63]. As the homologue of the identified key driver gene of CNV (*VEGF*) generated by differential alternative splicing, *VEGFD* directly participates in the pathogenesis of CNV, regulating the same biological processes of *VEGF* [64]. Recent clinical studies [65] confirmed that *VEGFD* may also be a candidate marker for the diagnosis and treatment of CNV, and drugs that target *VEGF* to relieve symptoms may also target the products of *VEGFD*.

### 4.3. MMP Inhibitors

*TIMP2* (ENSP00000262768) has been widely reported to act as a natural inhibitor for MMPs [66]. With a specific expression pattern in vitreous and subretinal fluid, this gene has been found to be expressed in choroid tissues [67] and directly contribute to the activation of the hypoxia-induced VEGF signaling pathway and MMP regulation [68]. Considering the irreplaceable role of VEGF in CNV, *TIMP2* may be a potential CNV-associated gene.

### 4.4. Collagens

Based on our newly presented computational methods, we also obtained two collagen coding genes that may contribute to the pathogenesis of CNV, namely, *COL3A1* (ENSP00000304408) and *COL18A1* (ENSP00000347665). *COL3A1* encodes the pro-alpha1 chain of type III collagen, a fibrillary collagen. Based on existing literatures, this gene contributes to the regulation of cortical development together with type I collagen in soft connective tissues [69, 70]. As for its specific pathogenic contribution to CNV, a specific study [71] confirmed that *COL3A1* may contribute to actin cytoskeleton remodeling and affect the specific lesion size and fibrosis of CNV. Similarly, the next predicted gene (*COL18A1*) has also been reported to participate in collagen-associated CNV pathogenesis [72]. Currently, no direct pathogenic experiment has confirmed that *COL18A1* can induce the progression of CNV independently. Other studies on collagen families including collagen XVIII [48, 73, 74] in CNV and their respective angiogenic functions have validated the potential pathogenic role of our predicted collagen encoding genes.

### 4.5. Lipocalins

Apart from MMPs, collagen, and growth factor-associated genes, we also obtained a specific lipocalin encoding gene, namely, *LCN2* (ENSP00000277480). Generally, this gene has been identified in the lung, breast [75], and eye secretions [76] and contribute to the transport of hydrophobic ligands [77]. As for its specific contribution to CNV, this gene may promote angiogenesis and neovascularization under pathogenic conditions [78, 79]. With a high-expression pattern in choroid tissues [80] and its interaction with MMPs [81], *LCN2* has been confirmed to participate in the pathogenic activation of the *AKT2*–*NF-κB*–*lipocalin-2* axis in CNV [82].

Taken together, the predicted functional genes are enriched in MMP-, growth factor-, collagen-, and lipocalin-related genes, implying the specific role of such components during the initiation and progression of CNV. The predicted genes have all been confirmed by recent publications as we have described above. Therefore, the computational approach in this study may be quite effective and accurate for identifying CNV-associated genes. This study not only identified a group of functional CNV-associated genes and potential related biological processes but also contributed to the improvement of current computational prediction approaches on the genetic background of diseases.

## 5. Conclusions

This study employed a powerful network diffusion method to identify possible CNV-related genes in a PPI network. To obtain reliable genes, a three-stage method followed to screen out key latent CNV-related genes. The analysis on final obtained genes indicate that they can be novel CNV-related genes with high likelihood. It is hopeful that the new findings reported in this study can provide new insights for investigating CNV.

## Figures and Tables

**Table 1:** Number of candidate CNV-related genes in different stages of LHD-based method.

Method	Network diffusion algorithm	*Z*-score	MIS	MFS
LHD-based method	19,218	153	27	11

**Table 2:** Eleven candidate genes yielded by LHD-based method.

Ensemble ID	Gene symbol	Description	Heat	*Z*-score	MIS	MES
ENSP00000299855	MMP3	Matrix metallopeptidase 3	8.79*E* − 05	2.0806	999	0.9761
ENSP00000230990	HBEGF	Heparin-binding EGF-like growth factor	9.01*E* − 05	3.0964	989	0.9608
ENSP00000260302	MMP13	Matrix metallopeptidase 13	1.54*E* − 04	3.9922	964	0.9600
ENSP00000260227	MMP7	Matrix metallopeptidase 7	1.25*E* − 04	3.8042	975	0.9580
ENSP00000262768	TIMP2	TIMP metallopeptidase inhibitor 2	1.20*E* − 04	3.2461	994	0.9569
ENSP00000222390	HGF	Hepatocyte growth factor	1.09*E* − 04	4.0224	922	0.9487
ENSP00000304408	COL3A1	Collagen type III alpha 1 chain	1.40*E* − 04	2.6753	951	0.9461
ENSP00000279441	MMP10	Matrix metallopeptidase 10	1.31*E* − 04	3.2940	977	0.9186
ENSP00000347665	COL18A1	Collagen type XVIII alpha 1 chain	1.25*E* − 04	2.5902	991	0.9183
ENSP00000277480	LCN2	Lipocalin 2	9.96*E* − 05	2.1251	985	0.9141
ENSP00000297904	VEGFD	Vascular endothelial growth factor D	2.13*E* − 04	6.6122	939	0.9078

## Data Availability

The data used to support the findings of this study are included within the supplementary information files.

## Supplementary Materials

Supplementary Materials Table S1: curated CNV-associated genes. Table S2: measurements of genes in each step.
