# Supplementary material for: Identification of Novel Choroidal Neovascularization-Related Genes Using Laplacian Heat Diffusion Algorithm
Source: Biomed Res Int. 2021 Sep 6;2021:2295412. doi: 10.1155/2021/2295412 (PMC8440095; doi:10.1155/2021/2295412)
Supplement: Supplementary Materials — Table S1: curated CNV-associated genes. Table S2: measurements of genes in each step. [file 2295412.f1.zip › 2295412.f1/Table S1.pdf]

Table S1: Curated CNV-associated genes

1	APOE
2	CLU
3	CRABP1
4	CRYAA
5	CRYBB2
6	CRYGD
7	CTGF
8	CX3CR1
9	EFEMP1
10	EIF4H
11	FABP5
12	FGG
13	FN1
14	FRZB
15	GFAP
16	HIST1H2AG
17	HLA-DRA
18	HSPA5
19	IG@
20	LAP3
21	LUM
22	LUM
23	MFAP4
24	MMP9
25	PLVAP
26	RHO
27	RLBP1
28	RLBP1
29	SEMA3B
30	SPARC
31	SPTB
32	TAGLN
33	TF
34	TIMP1
35	VCAM1
36	VIM
37	VWF
