# Supplementary material for: Identification of Novel Choroidal Neovascularization-Related Genes Using Laplacian Heat Diffusion Algorithm
Source: Biomed Res Int. 2021 Sep 6;2021:2295412. doi: 10.1155/2021/2295412 (PMC8440095; doi:10.1155/2021/2295412)
Supplement: Supplementary Materials — Table S1: curated CNV-associated genes. Table S2: measurements of genes in each step. [file 2295412.f1.zip › 2295412.f1/Table S2.pdf]

**Table S2:** Measurements of genes in each step

| Ensembl ID | Heat | Z-score | MIS | MFS |
|---|---|---|---|---|
| ENSP00000472293 | 9.21E-05 | 11.8844 | 241 | 0.000 |
| ENSP00000328631 | 1.98E-04 | 11.2781 | 192 | 0.218 |
| ENSP00000363976 | 1.28E-03 | 10.1062 | 671 | 0.739 |
| ENSP00000410815 | 1.65E-04 | 8.4500 | 190 | 0.000 |
| ENSP00000263408 | 1.47E-03 | 7.9368 | 696 | 0.339 |
| ENSP00000360841 | 5.65E-04 | 7.9107 | 235 | 0.168 |
| ENSP00000251296 | 1.97E-03 | 7.5888 | 325 | 0.118 |
| ENSP00000232003 | 2.97E-04 | 6.9968 | 943 | 0.650 |
| ENSP00000297904 | 2.13E-04 | 6.6122 | 939 | 0.908 |
| ENSP00000037502 | 3.46E-04 | 6.1745 | 828 | 0.679 |
| ENSP00000216286 | 4.56E-04 | 6.1405 | 323 | 0.483 |
| ENSP00000215743 | 2.15E-04 | 5.8568 | 920 | 0.878 |
| ENSP00000279396 | 3.79E-03 | 5.7100 | 250 | 0.160 |
| ENSP00000282141 | 7.34E-04 | 5.6639 | 978 | 0.404 |
| ENSP00000351473 | 8.65E-04 | 5.3798 | 380 | 0.434 |
| ENSP00000226355 | 4.14E-04 | 5.0547 | 329 | 0.544 |
| ENSP00000233997 | 1.52E-04 | 4.9718 | 459 | 0.696 |
| ENSP00000251643 | 4.48E-04 | 4.6313 | 329 | 0.198 |
| ENSP00000223061 | 4.28E-04 | 4.5966 | 328 | 0.554 |
| ENSP00000314299 | 2.73E-04 | 4.5186 | 284 | 0.483 |
| ENSP00000261336 | 3.24E-04 | 4.4961 | 618 | 0.442 |
| ENSP00000320396 | 7.27E-04 | 4.3772 | 407 | 0.000 |
| ENSP00000342445 | 7.83E-05 | 4.2668 | 152 | 0.197 |
| ENSP00000259526 | 1.66E-04 | 4.2498 | 595 | 0.832 |
| ENSP00000412457 | 5.38E-04 | 4.1939 | 225 | 0.000 |
| ENSP00000269593 | 2.56E-04 | 4.1244 | 323 | 0.827 |
| ENSP00000215727 | 1.73E-04 | 4.0784 | 558 | 0.589 |
| ENSP00000232217 | 2.91E-04 | 4.0342 | 355 | 0.643 |
| ENSP00000222390 | 1.09E-04 | 4.0224 | 922 | 0.949 |
| ENSP00000193391 | 2.89E-04 | 4.0033 | 291 | 0.414 |
| ENSP00000260302 | 1.54E-04 | 3.9922 | 964 | 0.960 |
| ENSP00000302105 | 4.82E-04 | 3.9687 | 752 | 0.346 |
| ENSP00000263100 | 1.54E-04 | 3.8824 | 235 | 0.742 |
| ENSP00000260227 | 1.25E-04 | 3.8042 | 975 | 0.958 |
| ENSP00000240986 | 3.48E-04 | 3.7918 | 757 | 0.000 |
| ENSP00000330808 | 6.08E-04 | 3.7886 | 288 | 0.136 |
| ENSP00000246006 | 1.89E-04 | 3.7603 | 311 | 0.579 |
| ENSP00000222381 | 1.18E-04 | 3.7228 | 793 | 0.688 |
| ENSP00000260988 | 4.01E-04 | 3.6571 | 563 | 0.302 |
| ENSP00000408132 | 1.98E-04 | 3.6529 | 301 | 0.171 |
| ENSP00000341442 | 2.64E-04 | 3.6397 | 288 | 0.116 |
| ENSP00000168148 | 7.50E-04 | 3.5870 | 244 | 0.281 |
| ENSP00000390020 | 2.89E-04 | 3.5689 | 265 | 0.572 |

| | | | | |
|---|---|---|---|---|
| ENSP00000301464 | 2.37E-04 | 3.5200 | 250 | 0.644 |
| ENSP00000347933 | 4.55E-04 | 3.4350 | 206 | 0.304 |
| ENSP00000286657 | 1.47E-04 | 3.3985 | 562 | 0.682 |
| ENSP00000307164 | 2.34E-04 | 3.3695 | 198 | 0.175 |
| ENSP00000304945 | 1.84E-04 | 3.2999 | 802 | 0.510 |
| ENSP00000279441 | 1.31E-04 | 3.2940 | 977 | 0.919 |
| ENSP00000299367 | 1.73E-04 | 3.2938 | 197 | 0.586 |
| ENSP00000311313 | 2.88E-04 | 3.2890 | 151 | 0.306 |
| ENSP00000274432 | 4.42E-04 | 3.2736 | 169 | 0.213 |
| ENSP00000220809 | 1.47E-04 | 3.2711 | 685 | 0.789 |
| ENSP00000258168 | 1.65E-04 | 3.2693 | 364 | 0.000 |
| ENSP00000258214 | 6.13E-04 | 3.2689 | 150 | 0.118 |
| ENSP00000262768 | 1.20E-04 | 3.2461 | 994 | 0.957 |
| ENSP00000256951 | 2.36E-04 | 3.1702 | 191 | 0.314 |
| ENSP00000294489 | 1.86E-04 | 3.1590 | 471 | 0.796 |
| ENSP00000257359 | 3.27E-04 | 3.1102 | 265 | 0.349 |
| ENSP00000278407 | 1.10E-04 | 3.1088 | 925 | 0.573 |
| ENSP00000230990 | 9.01E-05 | 3.0964 | 989 | 0.961 |
| ENSP00000216336 | 9.05E-05 | 3.0702 | 740 | 0.873 |
| ENSP00000256733 | 2.63E-04 | 3.0621 | 217 | 0.457 |
| ENSP00000282588 | 1.22E-04 | 3.0503 | 826 | 0.805 |
| ENSP00000226524 | 3.21E-04 | 3.0213 | 284 | 0.282 |
| ENSP00000376793 | 2.20E-04 | 2.9873 | 632 | 0.000 |
| ENSP00000367952 | 2.92E-04 | 2.9765 | 159 | 0.084 |
| ENSP00000256412 | 2.50E-04 | 2.9344 | 199 | 0.324 |
| ENSP00000009180 | 1.12E-04 | 2.9071 | 408 | 0.886 |
| ENSP00000362001 | 2.46E-04 | 2.8902 | 213 | 0.000 |
| ENSP00000456337 | 1.15E-04 | 2.8592 | 213 | 0.000 |
| ENSP00000004982 | 2.46E-04 | 2.8588 | 299 | 0.315 |
| ENSP00000295633 | 1.96E-04 | 2.8504 | 257 | 0.636 |
| ENSP00000250160 | 2.98E-04 | 2.8382 | 806 | 0.548 |
| ENSP00000249330 | 2.18E-04 | 2.8333 | 159 | 0.408 |
| ENSP00000222271 | 1.09E-04 | 2.8161 | 633 | 0.786 |
| ENSP00000304234 | 3.26E-04 | 2.8078 | 188 | 0.099 |
| ENSP00000293922 | 1.14E-03 | 2.7898 | 150 | 0.093 |
| ENSP00000167588 | 2.84E-04 | 2.7661 | 196 | 0.107 |
| ENSP00000225387 | 1.89E-04 | 2.7644 | 890 | 0.644 |
| ENSP00000390431 | 2.89E-04 | 2.7497 | 260 | 0.586 |
| ENSP00000215855 | 2.64E-04 | 2.7362 | 896 | 0.328 |
| ENSP00000242465 | 2.33E-04 | 2.6923 | 916 | 0.447 |
| ENSP00000225245 | 9.12E-05 | 2.6906 | 398 | 0.000 |
| ENSP00000304408 | 1.40E-04 | 2.6753 | 951 | 0.946 |
| ENSP00000454261 | 2.23E-04 | 2.6650 | 537 | 0.357 |
| ENSP00000264870 | 1.30E-04 | 2.6627 | 969 | 0.641 |
| ENSP00000267485 | 4.39E-04 | 2.6450 | 187 | 0.139 |
| ENSP00000347665 | 1.25E-04 | 2.5902 | 991 | 0.918 |

| | | | | |
|---|---|---|---|---|
| ENSP00000271651 | 1.02E-04 | 2.5801 | 826 | 0.896 |
| ENSP00000359596 | 1.19E-04 | 2.5432 | 360 | 0.230 |
| ENSP00000287814 | 1.63E-04 | 2.5395 | 815 | 0.572 |
| ENSP00000159060 | 1.13E-04 | 2.5248 | 914 | 0.777 |
| ENSP00000264187 | 1.01E-04 | 2.5212 | 949 | 0.881 |
| ENSP00000217407 | 2.13E-04 | 2.5143 | 330 | 0.695 |
| ENSP00000310631 | 2.66E-04 | 2.4950 | 200 | 0.292 |
| ENSP00000244336 | 1.34E-04 | 2.4781 | 282 | 0.505 |
| ENSP00000253796 | 3.93E-04 | 2.4746 | 267 | 0.174 |
| ENSP00000156499 | 1.68E-04 | 2.4611 | 566 | 0.151 |
| ENSP00000280193 | 1.35E-04 | 2.4524 | 936 | 0.000 |
| ENSP00000352458 | 1.37E-04 | 2.4483 | 702 | 0.458 |
| ENSP00000250378 | 8.97E-05 | 2.4306 | 534 | 0.759 |
| ENSP00000263326 | 2.40E-04 | 2.4292 | 357 | 0.739 |
| ENSP00000261978 | 2.52E-04 | 2.4270 | 571 | 0.436 |
| ENSP00000304987 | 1.89E-04 | 2.4231 | 619 | 0.255 |
| ENSP00000316152 | 1.42E-04 | 2.4128 | 622 | 0.826 |
| ENSP00000314810 | 1.21E-04 | 2.4081 | 359 | 0.883 |
| ENSP00000218099 | 9.45E-05 | 2.4025 | 976 | 0.590 |
| ENSP00000226218 | 8.76E-05 | 2.3950 | 925 | 0.000 |
| ENSP00000258229 | 3.57E-04 | 2.3897 | 378 | 0.112 |
| ENSP00000204615 | 9.45E-05 | 2.3680 | 581 | 0.942 |
| ENSP00000224181 | 2.53E-04 | 2.3631 | 163 | 0.272 |
| ENSP00000371811 | 4.19E-04 | 2.3595 | 157 | 0.165 |
| ENSP00000246949 | 9.17E-05 | 2.3427 | 216 | 0.842 |
| ENSP00000234347 | 7.65E-05 | 2.3362 | 472 | 0.783 |
| ENSP00000252229 | 1.44E-03 | 2.2945 | 165 | 0.140 |
| ENSP00000228438 | 9.75E-05 | 2.2919 | 198 | 0.458 |
| ENSP00000236850 | 6.94E-05 | 2.2687 | 991 | 0.874 |
| ENSP00000260653 | 8.68E-05 | 2.2648 | 398 | 0.847 |
| ENSP00000250092 | 7.92E-05 | 2.2639 | 670 | 0.950 |
| ENSP00000264952 | 3.04E-04 | 2.2390 | 971 | 0.438 |
| ENSP00000280190 | 2.35E-04 | 2.2293 | 176 | 0.122 |
| ENSP00000408146 | 2.13E-04 | 2.2254 | 280 | 0.542 |
| ENSP00000221891 | 1.13E-04 | 2.1981 | 472 | 0.606 |
| ENSP00000314116 | 2.52E-04 | 2.1965 | 169 | 0.135 |
| ENSP00000286614 | 9.99E-05 | 2.1608 | 862 | 0.840 |
| ENSP00000242994 | 1.61E-04 | 2.1582 | 414 | 0.781 |
| ENSP00000277480 | 9.96E-05 | 2.1251 | 985 | 0.914 |
| ENSP00000230381 | 1.67E-04 | 2.1243 | 749 | 0.489 |
| ENSP00000259396 | 1.74E-04 | 2.1178 | 202 | 0.486 |
| ENSP00000261921 | 1.55E-04 | 2.1148 | 270 | 0.540 |
| ENSP00000339260 | 1.16E-04 | 2.0863 | 457 | 0.230 |
| ENSP00000299855 | 8.79E-05 | 2.0806 | 999 | 0.976 |
| ENSP00000221804 | 2.97E-04 | 2.0769 | 150 | 0.181 |
| ENSP00000382434 | 2.51E-04 | 2.0645 | 154 | 0.102 |

| | | | | |
|---|---|---|---|---|
| ENSP00000295550 | 1.08E-04 | 2.0539 | 420 | 0.685 |
| ENSP00000344115 | 1.65E-04 | 2.0426 | 810 | 0.956 |
| ENSP00000223357 | 2.05E-04 | 2.0394 | 414 | 0.213 |
| ENSP00000230588 | 1.90E-04 | 2.0321 | 584 | 0.157 |
| ENSP00000357068 | 9.50E-05 | 2.0227 | 284 | 0.356 |
| ENSP00000225844 | 1.68E-04 | 2.0196 | 261 | 0.634 |
| ENSP00000324532 | 1.38E-04 | 2.0124 | 721 | 0.713 |
| ENSP00000264790 | 7.08E-05 | 2.0101 | 959 | 0.479 |
| ENSP00000242338 | 1.01E-04 | 2.0081 | 265 | 0.758 |
| ENSP00000252085 | 2.18E-04 | 2.0064 | 420 | 0.128 |
| ENSP00000341640 | 1.17E-04 | 1.9964 | 930 | 0.470 |
| ENSP00000229771 | 1.18E-04 | 1.9933 | 645 | 0.553 |
| ENSP00000222543 | 1.56E-04 | 1.9929 | 866 | 0.704 |
| ENSP00000332371 | 1.05E-04 | 1.9659 | 828 | 0.653 |
| ENSP00000313967 | 1.53E-04 | 1.9657 | 579 | 0.533 |
| ENSP00000252034 | 3.58E-04 | 1.9641 | 576 | 0.222 |
| ENSP00000415481 | 1.38E-04 | 1.9613 | 700 | 0.953 |
| ENSP00000387261 | 2.54E-04 | 1.9205 | 328 | 0.249 |
| ENSP00000323455 | 1.78E-04 | 1.9192 | 167 | 0.499 |
| ENSP00000223271 | 2.35E-04 | 1.9137 | 209 | 0.418 |
| ENSP00000311545 | 9.54E-05 | 1.9068 | 225 | 0.000 |
| ENSP00000341815 | 9.20E-05 | 1.8858 | 158 | 0.821 |
| ENSP00000228463 | 8.34E-05 | 1.8856 | 345 | 0.664 |
| ENSP00000317997 | 7.89E-05 | 1.8819 | 358 | 0.755 |
| ENSP00000231454 | 7.83E-05 | 1.8764 | 564 | 0.907 |
| ENSP00000215637 | 9.29E-05 | 1.8723 | 319 | 0.787 |
| ENSP00000313437 | 9.08E-05 | 1.8605 | 919 | 0.821 |
| ENSP00000244869 | 7.88E-05 | 1.8489 | 300 | 0.922 |
| ENSP00000278175 | 9.46E-05 | 1.8477 | 866 | 0.852 |
| ENSP00000341032 | 9.57E-05 | 1.8466 | 656 | 0.931 |
| ENSP00000345008 | 2.18E-04 | 1.8184 | 904 | 0.692 |
| ENSP00000221742 | 7.17E-05 | 1.8158 | 563 | 0.116 |
| ENSP00000332809 | 2.32E-04 | 1.8107 | 459 | 0.475 |
| ENSP00000255040 | 1.71E-04 | 1.8086 | 602 | 0.488 |
| ENSP00000255409 | 9.07E-05 | 1.8045 | 890 | 0.707 |
| ENSP00000248598 | 2.04E-04 | 1.8021 | 771 | 0.298 |
| ENSP00000291744 | 1.39E-04 | 1.7992 | 439 | 0.267 |
| ENSP00000263413 | 2.17E-04 | 1.7989 | 222 | 0.219 |
| ENSP00000277508 | 1.04E-04 | 1.7876 | 240 | 0.752 |
| ENSP00000222002 | 7.61E-05 | 1.7851 | 277 | 0.518 |
| ENSP00000377549 | 1.37E-04 | 1.7839 | 557 | 0.405 |
| ENSP00000334848 | 3.16E-04 | 1.7768 | 197 | 0.156 |
| ENSP00000264595 | 9.81E-05 | 1.7717 | 267 | 0.268 |
| ENSP00000343577 | 1.21E-04 | 1.7670 | 166 | 0.144 |
| ENSP00000268704 | 6.45E-05 | 1.7670 | 456 | 0.702 |
| ENSP00000221973 | 1.54E-04 | 1.7616 | 688 | 0.297 |

| | | | | |
|---|---|---|---|---|
| ENSP00000257626 | 1.84E-04 | 1.7587 | 150 | 0.084 |
| ENSP00000259206 | 7.13E-05 | 1.7507 | 252 | 0.804 |
| ENSP00000426070 | 8.25E-05 | 1.7497 | 225 | 0.401 |
| ENSP00000011292 | 1.10E-04 | 1.7347 | 264 | 0.226 |
| ENSP00000265627 | 1.63E-04 | 1.7316 | 495 | 0.280 |
| ENSP00000215939 | 1.92E-04 | 1.7201 | 774 | 0.393 |
| ENSP00000291670 | 1.07E-04 | 1.7151 | 265 | 0.226 |
| ENSP00000278927 | 9.27E-05 | 1.7128 | 812 | 0.933 |
| ENSP00000298966 | 2.51E-04 | 1.7111 | 406 | 0.115 |
| ENSP00000300182 | 1.63E-04 | 1.6926 | 416 | 0.165 |
| ENSP00000271331 | 9.09E-05 | 1.6921 | 822 | 0.569 |
| ENSP00000259633 | 1.06E-04 | 1.6903 | 196 | 0.637 |
| ENSP00000248076 | 1.07E-04 | 1.6881 | 242 | 0.493 |
| ENSP00000357156 | 1.35E-04 | 1.6834 | 562 | 0.362 |
| ENSP00000228938 | 5.52E-05 | 1.6822 | 562 | 0.871 |
| ENSP00000296280 | 1.18E-04 | 1.6759 | 192 | 0.355 |
| ENSP00000238667 | 1.56E-04 | 1.6725 | 150 | 0.212 |
| ENSP00000305714 | 9.12E-05 | 1.6677 | 358 | 0.917 |
| ENSP00000296029 | 9.68E-05 | 1.6513 | 974 | 0.884 |
| ENSP00000261304 | 9.98E-05 | 1.6498 | 472 | 0.575 |
| ENSP00000331544 | 1.86E-04 | 1.6355 | 914 | 0.693 |
| ENSP00000321455 | 2.01E-04 | 1.6341 | 157 | 0.164 |
| ENSP00000280527 | 1.06E-04 | 1.6298 | 368 | 0.244 |
| ENSP00000265012 | 9.57E-05 | 1.6266 | 728 | 0.198 |
| ENSP00000267803 | 7.46E-05 | 1.6242 | 235 | 0.495 |
| ENSP00000410926 | 1.51E-04 | 1.6098 | 216 | 0.116 |
| ENSP00000242208 | 7.38E-05 | 1.6082 | 325 | 0.932 |
| ENSP00000355180 | 1.64E-04 | 1.6027 | 504 | 0.815 |
| ENSP00000327075 | 1.56E-04 | 1.5930 | 191 | 0.082 |
| ENSP00000296130 | 8.74E-05 | 1.5930 | 325 | 0.779 |
| ENSP00000296420 | 1.53E-04 | 1.5926 | 242 | 0.386 |
| ENSP00000271348 | 6.55E-05 | 1.5870 | 309 | 0.000 |
| ENSP00000260049 | 1.27E-04 | 1.5793 | 856 | 0.653 |
| ENSP00000219334 | 1.48E-04 | 1.5707 | 560 | 0.121 |
| ENSP00000270631 | 1.43E-04 | 1.5619 | 159 | 0.455 |
| ENSP00000345633 | 9.21E-05 | 1.5617 | 297 | 0.930 |
| ENSP00000311165 | 9.61E-05 | 1.5568 | 297 | 0.403 |
| ENSP00000380897 | 6.33E-04 | 1.5498 | 268 | 0.279 |
| ENSP00000361366 | 1.24E-04 | 1.5478 | 404 | 0.607 |
| ENSP00000364697 | 7.58E-05 | 1.5418 | 298 | 0.453 |
| ENSP00000455469 | 9.02E-05 | 1.5382 | 170 | 0.252 |
| ENSP00000357726 | 1.44E-04 | 1.5364 | 325 | 0.422 |
| ENSP00000360269 | 1.03E-04 | 1.5324 | 240 | 0.297 |
| ENSP00000225371 | 7.15E-05 | 1.5309 | 360 | 0.629 |
| ENSP00000216139 | 5.10E-05 | 1.5294 | 191 | 0.245 |
| ENSP00000256178 | 8.18E-05 | 1.5187 | 361 | 0.570 |

| | | | | |
|---|---|---|---|---|
| ENSP00000226413 | 5.80E-05 | 1.5168 | 659 | 0.800 |
| ENSP00000257979 | 6.43E-05 | 1.5135 | 943 | 0.231 |
| ENSP00000216361 | 1.69E-04 | 1.5128 | 359 | 0.238 |
| ENSP00000273430 | 6.78E-05 | 1.5103 | 537 | 0.872 |
| ENSP00000377783 | 1.04E-04 | 1.5059 | 971 | 0.665 |
| ENSP00000353427 | 5.79E-04 | 1.5003 | 265 | 0.879 |
| ENSP00000269202 | 1.31E-04 | 1.4962 | 577 | 0.124 |
| ENSP00000278833 | 7.50E-05 | 1.4939 | 700 | 0.567 |
| ENSP00000254868 | 6.95E-05 | 1.4897 | 202 | 0.456 |
| ENSP00000309953 | 1.79E-04 | 1.4793 | 902 | 0.325 |
| ENSP00000006053 | 4.90E-05 | 1.4792 | 854 | 0.720 |
| ENSP00000345179 | 1.48E-04 | 1.4758 | 702 | 0.643 |
| ENSP00000305464 | 6.62E-05 | 1.4733 | 242 | 0.000 |
| ENSP00000256785 | 3.64E-04 | 1.4678 | 266 | 0.202 |
| ENSP00000328173 | 9.01E-05 | 1.4637 | 392 | 0.572 |
| ENSP00000366314 | 6.30E-04 | 1.4564 | 0 | 0.185 |
| ENSP00000222481 | 9.29E-05 | 1.4474 | 248 | 0.063 |
| ENSP00000245983 | 8.62E-05 | 1.4424 | 216 | 0.536 |
| ENSP00000366307 | 7.00E-05 | 1.4416 | 400 | 0.585 |
| ENSP00000296422 | 8.09E-05 | 1.4409 | 191 | 0.116 |
| ENSP00000231009 | 7.61E-05 | 1.4378 | 198 | 0.266 |
| ENSP00000330218 | 7.28E-05 | 1.4344 | 297 | 0.865 |
| ENSP00000310335 | 1.80E-04 | 1.4344 | 214 | 0.262 |
| ENSP00000360549 | 8.42E-05 | 1.4330 | 272 | 0.143 |
| ENSP00000373948 | 7.29E-05 | 1.4292 | 323 | 0.000 |
| ENSP00000359013 | 1.22E-04 | 1.4239 | 159 | 0.603 |
| ENSP00000368402 | 1.38E-04 | 1.4184 | 179 | 0.086 |
| ENSP00000221347 | 1.90E-04 | 1.4123 | 159 | 0.000 |
| ENSP00000200676 | 9.47E-05 | 1.4119 | 752 | 0.470 |
| ENSP00000245457 | 1.04E-04 | 1.4049 | 208 | 0.705 |
| ENSP00000357103 | 1.10E-04 | 1.4044 | 353 | 0.000 |
| ENSP00000337224 | 1.00E-04 | 1.4040 | 700 | 0.483 |
| ENSP00000256119 | 4.87E-05 | 1.4035 | 284 | 0.000 |
| ENSP00000221980 | 1.59E-04 | 1.3991 | 265 | 0.581 |
| ENSP00000327048 | 1.47E-04 | 1.3931 | 824 | 0.887 |
| ENSP00000221496 | 5.44E-05 | 1.3913 | 265 | 0.920 |
| ENSP00000293280 | 8.09E-05 | 1.3866 | 359 | 0.000 |
| ENSP00000261751 | 1.33E-04 | 1.3844 | 473 | 0.334 |
| ENSP00000319308 | 5.48E-05 | 1.3794 | 238 | 0.745 |
| ENSP00000296097 | 6.80E-05 | 1.3756 | 776 | 0.200 |
| ENSP00000351059 | 9.07E-05 | 1.3752 | 440 | 0.722 |
| ENSP00000264741 | 7.47E-05 | 1.3734 | 923 | 0.605 |
| ENSP00000227756 | 1.13E-04 | 1.3731 | 202 | 0.090 |
| ENSP00000226279 | 5.00E-05 | 1.3694 | 360 | 0.854 |
| ENSP00000349805 | 1.56E-04 | 1.3642 | 206 | 0.118 |
| ENSP00000447173 | 5.04E-04 | 1.3636 | 471 | 0.897 |

| | | | | |
|---|---|---|---|---|
| ENSP00000310111 | 1.07E-04 | 1.3585 | 472 | 0.141 |
| ENSP00000257267 | 1.78E-04 | 1.3575 | 206 | 0.000 |
| ENSP00000253673 | 8.88E-05 | 1.3558 | 151 | 0.000 |
| ENSP00000270879 | 1.85E-04 | 1.3557 | 510 | 0.144 |
| ENSP00000251102 | 9.12E-05 | 1.3552 | 499 | 0.465 |
| ENSP00000296953 | 2.30E-04 | 1.3446 | 183 | 0.168 |
| ENSP00000221992 | 9.69E-05 | 1.3445 | 281 | 0.198 |
| ENSP00000346294 | 1.40E-04 | 1.3445 | 437 | 0.868 |
| ENSP00000439985 | 6.89E-05 | 1.3439 | 191 | 0.144 |
| ENSP00000253255 | 1.09E-04 | 1.3361 | 313 | 0.181 |
| ENSP00000299440 | 1.09E-04 | 1.3310 | 356 | 0.884 |
| ENSP00000353094 | 1.28E-04 | 1.3304 | 461 | 0.696 |
| ENSP00000290271 | 6.72E-05 | 1.3289 | 330 | 0.684 |
| ENSP00000286688 | 1.35E-04 | 1.3272 | 252 | 0.307 |
| ENSP00000265498 | 1.19E-04 | 1.3271 | 200 | 0.195 |
| ENSP00000323929 | 1.35E-04 | 1.3269 | 930 | 0.826 |
| ENSP00000281081 | 7.66E-05 | 1.3219 | 451 | 0.242 |
| ENSP00000274979 | 1.58E-04 | 1.3198 | 247 | 0.073 |
| ENSP00000414321 | 1.79E-04 | 1.3184 | 181 | 0.833 |
| ENSP00000456609 | 1.82E-04 | 1.3116 | 286 | 0.230 |
| ENSP00000222304 | 1.31E-04 | 1.3102 | 340 | 0.640 |
| ENSP00000416330 | 1.51E-04 | 1.3083 | 872 | 0.800 |
| ENSP00000219299 | 3.02E-04 | 1.3046 | 198 | 0.119 |
| ENSP00000265769 | 7.82E-05 | 1.3042 | 328 | 0.222 |
| ENSP00000334962 | 8.98E-05 | 1.3041 | 589 | 0.323 |
| ENSP00000330959 | 1.41E-04 | 1.3040 | 213 | 0.564 |
| ENSP00000356771 | 1.17E-04 | 1.3012 | 980 | 0.789 |
| ENSP00000356037 | 8.69E-05 | 1.2957 | 325 | 0.416 |
| ENSP00000258704 | 2.73E-04 | 1.2935 | 159 | 0.139 |
| ENSP00000272847 | 9.63E-05 | 1.2922 | 422 | 0.480 |
| ENSP00000369320 | 1.14E-04 | 1.2890 | 461 | 0.419 |
| ENSP00000359699 | 1.35E-04 | 1.2864 | 533 | 0.379 |
| ENSP00000296140 | 7.16E-05 | 1.2791 | 499 | 0.813 |
| ENSP00000296145 | 7.69E-05 | 1.2779 | 895 | 0.877 |
| ENSP00000255030 | 1.48E-04 | 1.2689 | 593 | 0.354 |
| ENSP00000349709 | 7.61E-05 | 1.2674 | 349 | 0.272 |
| ENSP00000370473 | 6.08E-04 | 1.2616 | 890 | 0.959 |
| ENSP00000259883 | 1.41E-04 | 1.2604 | 169 | 0.139 |
| ENSP00000387356 | 9.96E-05 | 1.2575 | 996 | 0.894 |
| ENSP00000260229 | 1.50E-04 | 1.2536 | 548 | 0.544 |
| ENSP00000386881 | 2.11E-04 | 1.2522 | 165 | 0.279 |
| ENSP00000264828 | 7.48E-05 | 1.2511 | 905 | 0.750 |
| ENSP00000239849 | 6.66E-05 | 1.2460 | 501 | 0.941 |
| ENSP00000264563 | 9.77E-05 | 1.2445 | 360 | 0.891 |
| ENSP00000220772 | 6.99E-05 | 1.2441 | 301 | 0.965 |
| ENSP00000340864 | 1.59E-04 | 1.2405 | 214 | 0.090 |

| | | | | |
|---|---|---|---|---|
| ENSP00000226317 | 8.15E-05 | 1.2372 | 731 | 0.626 |
| ENSP00000360899 | 1.26E-04 | 1.2372 | 325 | 0.197 |
| ENSP00000297435 | 9.75E-05 | 1.2355 | 221 | 0.126 |
| ENSP00000274625 | 6.53E-05 | 1.2329 | 257 | 0.931 |
| ENSP00000223293 | 2.39E-04 | 1.2316 | 311 | 0.117 |
| ENSP00000215781 | 1.12E-04 | 1.2266 | 890 | 0.860 |
| ENSP00000361400 | 9.18E-05 | 1.2251 | 198 | 0.499 |
| ENSP00000326948 | 6.55E-05 | 1.2231 | 274 | 0.773 |
| ENSP00000237837 | 5.94E-05 | 1.2172 | 558 | 0.920 |
| ENSP00000263851 | 5.86E-05 | 1.2171 | 485 | 0.886 |
| ENSP00000363988 | 1.15E-04 | 1.2115 | 295 | 0.364 |
| ENSP00000343445 | 7.14E-05 | 1.2069 | 196 | 0.614 |
| ENSP00000158166 | 5.56E-05 | 1.2012 | 203 | 0.653 |
| ENSP00000247866 | 9.23E-05 | 1.1959 | 288 | 0.185 |
| ENSP00000436450 | 2.60E-05 | 1.1928 | 215 | 0.158 |
| ENSP00000252519 | 1.11E-04 | 1.1927 | 570 | 0.563 |
| ENSP00000218867 | 4.49E-05 | 1.1921 | 301 | 0.633 |
| ENSP00000297350 | 9.31E-05 | 1.1905 | 708 | 0.956 |
| ENSP00000362776 | 7.73E-05 | 1.1884 | 909 | 0.439 |
| ENSP00000357798 | 1.21E-04 | 1.1827 | 632 | 0.321 |
| ENSP00000319486 | 8.94E-05 | 1.1820 | 252 | 0.129 |
| ENSP00000353582 | 8.24E-05 | 1.1809 | 216 | 0.787 |
| ENSP00000346227 | 3.23E-04 | 1.1764 | 309 | 0.373 |
| ENSP00000357461 | 9.30E-05 | 1.1732 | 473 | 0.393 |
| ENSP00000246532 | 1.36E-04 | 1.1729 | 150 | 0.151 |
| ENSP00000290438 | 1.19E-04 | 1.1666 | 252 | 0.152 |
| ENSP00000257254 | 6.21E-05 | 1.1657 | 198 | 0.764 |
| ENSP00000273590 | 1.22E-04 | 1.1649 | 513 | 0.212 |
| ENSP00000408288 | 6.99E-05 | 1.1604 | 191 | 0.109 |
| ENSP00000303149 | 9.24E-05 | 1.1595 | 150 | 0.275 |
| ENSP00000267102 | 2.54E-04 | 1.1591 | 311 | 0.351 |
| ENSP00000314910 | 1.90E-04 | 1.1585 | 510 | 0.878 |
| ENSP00000243167 | 5.50E-05 | 1.1547 | 814 | 0.257 |
| ENSP00000367104 | 2.26E-04 | 1.1545 | 829 | 0.305 |
| ENSP00000216106 | 1.95E-04 | 1.1496 | 208 | 0.501 |
| ENSP00000300762 | 9.31E-05 | 1.1447 | 726 | 0.601 |
| ENSP00000382034 | 1.80E-04 | 1.1428 | 301 | 0.724 |
| ENSP00000369071 | 1.13E-04 | 1.1359 | 517 | 0.919 |
| ENSP00000256104 | 6.20E-05 | 1.1349 | 456 | 0.761 |
| ENSP00000204604 | 5.49E-05 | 1.1318 | 552 | 0.902 |
| ENSP00000388548 | 6.60E-05 | 1.1315 | 471 | 0.780 |
| ENSP00000362381 | 8.98E-05 | 1.1314 | 167 | 0.120 |
| ENSP00000338343 | 8.74E-05 | 1.1292 | 806 | 0.307 |
| ENSP00000275216 | 9.72E-05 | 1.1240 | 404 | 0.499 |
| ENSP00000199936 | 1.01E-04 | 1.1086 | 163 | 0.188 |
| ENSP00000326159 | 6.52E-05 | 1.1082 | 700 | 0.770 |

| | | | | |
|---|---|---|---|---|
| ENSP00000276410 | 1.01E-04 | 1.1026 | 605 | 0.241 |
| ENSP00000211122 | 9.28E-05 | 1.0992 | 341 | 0.180 |
| ENSP00000274721 | 1.28E-04 | 1.0989 | 313 | 0.151 |
| ENSP00000350734 | 2.01E-04 | 1.0982 | 177 | 0.089 |
| ENSP00000254661 | 1.81E-04 | 1.0972 | 254 | 0.315 |
| ENSP00000364407 | 1.43E-04 | 1.0939 | 191 | 0.163 |
| ENSP00000225474 | 4.86E-05 | 1.0913 | 471 | 0.932 |
| ENSP00000219244 | 8.16E-05 | 1.0878 | 675 | 0.687 |
| ENSP00000309148 | 1.10E-04 | 1.0868 | 514 | 0.437 |
| ENSP00000345395 | 1.14E-04 | 1.0847 | 736 | 0.284 |
| ENSP00000294800 | 1.04E-04 | 1.0839 | 300 | 0.431 |
| ENSP00000231121 | 5.39E-05 | 1.0825 | 295 | 0.868 |
| ENSP00000355370 | 6.57E-05 | 1.0796 | 930 | 0.922 |
| ENSP00000291700 | 6.31E-05 | 1.0763 | 981 | 0.877 |
| ENSP00000355751 | 1.06E-04 | 1.0731 | 958 | 0.916 |
| ENSP00000248444 | 4.33E-05 | 1.0715 | 341 | 0.772 |
| ENSP00000294671 | 2.13E-04 | 1.0669 | 403 | 0.197 |
| ENSP00000356016 | 1.27E-04 | 1.0664 | 636 | 0.870 |
| ENSP00000348308 | 1.16E-04 | 1.0597 | 596 | 0.414 |
| ENSP00000337816 | 6.69E-05 | 1.0594 | 802 | 0.689 |
| ENSP00000296575 | 6.77E-05 | 1.0565 | 216 | 0.774 |
| ENSP00000200691 | 3.01E-04 | 1.0547 | 192 | 0.091 |
| ENSP00000341838 | 6.35E-05 | 1.0505 | 902 | 0.487 |
| ENSP00000324553 | 1.09E-04 | 1.0459 | 167 | 0.179 |
| ENSP00000356791 | 2.03E-04 | 1.0371 | 436 | 0.497 |
| ENSP00000226193 | 4.31E-05 | 1.0348 | 997 | 0.550 |
| ENSP00000360687 | 1.26E-04 | 1.0226 | 493 | 0.694 |
| ENSP00000364814 | 1.17E-04 | 1.0225 | 203 | 0.225 |
| ENSP00000304930 | 8.42E-05 | 1.0206 | 402 | 0.736 |
| ENSP00000220616 | 6.33E-05 | 1.0203 | 872 | 0.852 |
| ENSP00000398890 | 2.74E-04 | 1.0189 | 228 | 0.560 |
| ENSP00000265132 | 5.85E-05 | 1.0183 | 685 | 0.701 |
| ENSP00000252491 | 1.25E-04 | 1.0173 | 731 | 0.000 |
| ENSP00000359691 | 1.11E-04 | 1.0093 | 317 | 0.419 |
| ENSP00000273183 | 1.80E-04 | 1.0089 | 177 | 0.241 |
| ENSP00000277903 | 7.55E-05 | 1.0088 | 751 | 0.315 |
| ENSP00000259607 | 5.20E-05 | 1.0033 | 456 | 0.862 |
| ENSP00000257248 | 5.83E-05 | 1.0013 | 398 | 0.385 |
| ENSP00000260128 | 8.45E-05 | 1.0012 | 212 | 0.565 |
| ENSP00000430690 | 1.32E-04 | 0.9967 | 261 | 0.706 |
| ENSP00000307259 | 5.50E-05 | 0.9946 | 658 | 0.663 |
| ENSP00000359245 | 9.51E-05 | 0.9923 | 706 | 0.564 |
| ENSP00000259711 | 6.58E-05 | 0.9921 | 159 | 0.200 |
| ENSP00000360272 | 2.71E-05 | 0.9912 | 0 | 0.000 |
| ENSP00000254122 | 5.95E-05 | 0.9906 | 313 | 0.837 |
| ENSP00000261454 | 9.58E-05 | 0.9889 | 280 | 0.848 |

| | | | | |
|---|---|---|---|---|
| ENSP00000284885 | 1.01E-04 | 0.9885 | 297 | 0.164 |
| ENSP00000289672 | 9.96E-05 | 0.9819 | 161 | 0.098 |
| ENSP00000296027 | 5.51E-05 | 0.9753 | 879 | 0.651 |
| ENSP00000296028 | 7.99E-05 | 0.9744 | 964 | 0.712 |
| ENSP00000019103 | 4.58E-05 | 0.9735 | 634 | 0.603 |
| ENSP00000243222 | 1.13E-04 | 0.9735 | 303 | 0.672 |
| ENSP00000344012 | 6.52E-05 | 0.9716 | 345 | 0.000 |
| ENSP00000221459 | 7.86E-05 | 0.9702 | 210 | 0.138 |
| ENSP00000211076 | 9.36E-05 | 0.9638 | 264 | 0.183 |
| ENSP00000311746 | 9.87E-05 | 0.9584 | 418 | 0.225 |
| ENSP00000027335 | 4.30E-05 | 0.9583 | 585 | 0.961 |
| ENSP00000261994 | 8.13E-05 | 0.9562 | 204 | 0.302 |
| ENSP00000378577 | 6.92E-05 | 0.9526 | 152 | 0.190 |
| ENSP00000309463 | 7.99E-05 | 0.9485 | 179 | 0.119 |
| ENSP00000282701 | 4.57E-05 | 0.9484 | 404 | 0.910 |
| ENSP00000299022 | 6.64E-05 | 0.9484 | 960 | 0.407 |
| ENSP00000399709 | 8.57E-05 | 0.9480 | 523 | 0.526 |
| ENSP00000355627 | 9.90E-05 | 0.9378 | 861 | 0.873 |
| ENSP00000257632 | 1.09E-04 | 0.9373 | 150 | 0.188 |
| ENSP00000263369 | 9.48E-05 | 0.9372 | 602 | 0.000 |
| ENSP00000411004 | 7.19E-05 | 0.9360 | 345 | 0.103 |
| ENSP00000301455 | 8.14E-05 | 0.9338 | 357 | 0.814 |
| ENSP00000351905 | 1.47E-04 | 0.9325 | 940 | 0.949 |
| ENSP00000219454 | 7.12E-05 | 0.9215 | 195 | 0.742 |
| ENSP00000283249 | 8.96E-05 | 0.9210 | 963 | 0.823 |
| ENSP00000378130 | 5.74E-05 | 0.9194 | 313 | 0.286 |
| ENSP00000472280 | 1.17E-04 | 0.9184 | 159 | 0.000 |
| ENSP00000413479 | 1.63E-04 | 0.9180 | 203 | 0.000 |
| ENSP00000216540 | 6.07E-05 | 0.9170 | 192 | 0.324 |
| ENSP00000364114 | 1.20E-04 | 0.9166 | 900 | 0.579 |
| ENSP00000308117 | 1.34E-04 | 0.9121 | 626 | 0.179 |
| ENSP00000299320 | 2.38E-04 | 0.9058 | 479 | 0.307 |
| ENSP00000261918 | 6.59E-05 | 0.9028 | 311 | 0.573 |
| ENSP00000219235 | 8.47E-05 | 0.9028 | 619 | 0.726 |
| ENSP00000362643 | 1.55E-04 | 0.9028 | 295 | 0.325 |
| ENSP00000356671 | 1.19E-04 | 0.9014 | 953 | 0.763 |
| ENSP00000299213 | 8.88E-05 | 0.8941 | 301 | 0.469 |
| ENSP00000348068 | 1.39E-04 | 0.8938 | 944 | 0.863 |
| ENSP00000300127 | 5.46E-05 | 0.8938 | 171 | 0.000 |
| ENSP00000406381 | 1.00E-04 | 0.8919 | 514 | 0.000 |
| ENSP00000329374 | 8.50E-05 | 0.8907 | 285 | 0.425 |
| ENSP00000273145 | 7.52E-05 | 0.8899 | 284 | 0.000 |
| ENSP00000386341 | 7.81E-05 | 0.8879 | 191 | 0.000 |
| ENSP00000331736 | 6.68E-05 | 0.8870 | 813 | 0.907 |
| ENSP00000258400 | 4.70E-05 | 0.8867 | 216 | 0.612 |
| ENSP00000261037 | 2.18E-04 | 0.8839 | 329 | 0.361 |

| | | | | |
|---|---|---|---|---|
| ENSP00000252854 | 7.35E-05 | 0.8837 | 150 | 0.105 |
| ENSP00000319730 | 4.68E-05 | 0.8789 | 392 | 0.224 |
| ENSP00000347213 | 5.32E-05 | 0.8788 | 407 | 0.320 |
| ENSP00000190983 | 1.71E-04 | 0.8733 | 356 | 0.429 |
| ENSP00000216341 | 4.42E-05 | 0.8732 | 642 | 0.902 |
| ENSP00000359019 | 9.30E-05 | 0.8729 | 586 | 0.000 |
| ENSP00000336661 | 2.20E-04 | 0.8723 | 179 | 0.105 |
| ENSP00000320089 | 7.25E-05 | 0.8696 | 286 | 0.857 |
| ENSP00000372654 | 6.56E-05 | 0.8682 | 418 | 0.433 |
| ENSP00000247161 | 2.23E-04 | 0.8628 | 900 | 0.727 |
| ENSP00000278060 | 8.17E-05 | 0.8614 | 203 | 0.151 |
| ENSP00000298569 | 2.88E-04 | 0.8605 | 159 | 0.179 |
| ENSP00000319179 | 8.71E-05 | 0.8600 | 179 | 0.000 |
| ENSP00000312946 | 9.27E-05 | 0.8589 | 915 | 0.323 |
| ENSP00000295108 | 4.68E-05 | 0.8564 | 872 | 0.872 |
| ENSP00000287590 | 1.01E-04 | 0.8564 | 913 | 0.151 |
| ENSP00000262018 | 5.17E-05 | 0.8560 | 226 | 0.419 |
| ENSP00000339067 | 1.32E-04 | 0.8540 | 150 | 0.158 |
| ENSP00000350387 | 6.93E-05 | 0.8535 | 163 | 0.221 |
| ENSP00000292513 | 4.50E-05 | 0.8522 | 209 | 0.654 |
| ENSP00000362684 | 8.73E-05 | 0.8518 | 171 | 0.161 |
| ENSP00000316737 | 8.21E-05 | 0.8515 | 159 | 0.165 |
| ENSP00000358106 | 7.21E-05 | 0.8502 | 788 | 0.350 |
| ENSP00000246186 | 6.47E-05 | 0.8500 | 850 | 0.768 |
| ENSP00000444736 | 1.84E-07 | 0.8497 | 0 | 0.203 |
| ENSP00000441624 | 1.84E-07 | 0.8497 | 0 | 0.203 |
| ENSP00000362029 | 1.54E-04 | 0.8478 | 261 | 0.184 |
| ENSP00000380352 | 1.95E-04 | 0.8470 | 302 | 0.275 |
| ENSP00000331741 | 5.28E-05 | 0.8453 | 418 | 0.836 |
| ENSP00000250144 | 8.09E-05 | 0.8444 | 726 | 0.000 |
| ENSP00000228534 | 6.61E-05 | 0.8406 | 845 | 0.718 |
| ENSP00000293272 | 6.24E-05 | 0.8374 | 540 | 0.000 |
| ENSP00000394624 | 5.17E-04 | 0.8350 | 201 | 0.710 |
| ENSP00000254488 | 7.19E-05 | 0.8338 | 457 | 0.233 |
| ENSP00000381273 | 8.72E-05 | 0.8335 | 980 | 0.338 |
| ENSP00000334657 | 7.90E-05 | 0.8278 | 162 | 0.097 |
| ENSP00000225512 | 3.88E-05 | 0.8277 | 648 | 0.926 |
| ENSP00000289166 | 1.08E-04 | 0.8267 | 228 | 0.115 |
| ENSP00000345785 | 9.14E-05 | 0.8256 | 290 | 0.843 |
| ENSP00000157600 | 1.74E-04 | 0.8199 | 174 | 0.098 |
| ENSP00000274024 | 6.07E-05 | 0.8198 | 390 | 0.560 |
| ENSP00000397297 | 5.24E-05 | 0.8120 | 242 | 0.886 |
| ENSP00000344413 | 1.22E-04 | 0.8096 | 157 | 0.507 |
| ENSP00000258034 | 6.91E-05 | 0.8095 | 216 | 0.329 |
| ENSP00000342868 | 6.75E-05 | 0.8092 | 183 | 0.000 |
| ENSP00000267291 | 9.52E-05 | 0.8064 | 242 | 0.658 |

| | | | | |
|---|---|---|---|---|
| ENSP00000295256 | 5.19E-05 | 0.8053 | 252 | 0.461 |
| ENSP00000384957 | 6.88E-05 | 0.8015 | 636 | 0.488 |
| ENSP00000264162 | 4.04E-05 | 0.7972 | 668 | 0.586 |
| ENSP00000349790 | 7.09E-05 | 0.7960 | 909 | 0.630 |
| ENSP00000268605 | 3.94E-05 | 0.7943 | 252 | 0.701 |
| ENSP00000346805 | 6.80E-05 | 0.7913 | 763 | 0.283 |
| ENSP00000289473 | 8.57E-05 | 0.7902 | 601 | 0.918 |
| ENSP00000227251 | 1.35E-04 | 0.7891 | 701 | 0.750 |
| ENSP00000434034 | 1.04E-04 | 0.7867 | 875 | 0.933 |
| ENSP00000357470 | 5.51E-05 | 0.7837 | 354 | 0.899 |
| ENSP00000242152 | 3.61E-05 | 0.7823 | 416 | 0.857 |
| ENSP00000323584 | 1.04E-04 | 0.7817 | 382 | 0.417 |
| ENSP00000287196 | 1.83E-04 | 0.7799 | 351 | 0.119 |
| ENSP00000249005 | 1.00E-04 | 0.7798 | 205 | 0.069 |
| ENSP00000338010 | 5.94E-05 | 0.7792 | 197 | 0.662 |
| ENSP00000387230 | 1.22E-04 | 0.7757 | 576 | 0.000 |
| ENSP00000353767 | 7.02E-05 | 0.7752 | 802 | 0.697 |
| ENSP00000273283 | 6.35E-05 | 0.7747 | 490 | 0.405 |
| ENSP00000356382 | 8.33E-05 | 0.7742 | 971 | 0.377 |
| ENSP00000454071 | 9.67E-05 | 0.7708 | 997 | 0.929 |
| ENSP00000343339 | 7.20E-05 | 0.7708 | 227 | 0.530 |
| ENSP00000303550 | 9.59E-05 | 0.7699 | 195 | 0.106 |
| ENSP00000382867 | 4.66E-05 | 0.7681 | 165 | 0.181 |
| ENSP00000239316 | 1.01E-04 | 0.7671 | 216 | 0.171 |
| ENSP00000262418 | 6.66E-05 | 0.7670 | 748 | 0.319 |
| ENSP00000274063 | 8.18E-05 | 0.7658 | 286 | 0.953 |
| ENSP00000252891 | 5.03E-05 | 0.7655 | 317 | 0.826 |
| ENSP00000307875 | 6.59E-05 | 0.7646 | 349 | 0.707 |
| ENSP00000308165 | 6.88E-05 | 0.7632 | 729 | 0.944 |
| ENSP00000393566 | 9.99E-05 | 0.7614 | 0 | 0.843 |
| ENSP00000257981 | 6.79E-05 | 0.7612 | 170 | 0.080 |
| ENSP00000360997 | 9.24E-05 | 0.7605 | 996 | 0.623 |
| ENSP00000298841 | 5.45E-05 | 0.7601 | 265 | 0.704 |
| ENSP00000286428 | 5.42E-05 | 0.7598 | 246 | 0.309 |
| ENSP00000359998 | 6.10E-05 | 0.7573 | 352 | 0.215 |
| ENSP00000328181 | 5.83E-05 | 0.7567 | 888 | 0.931 |
| ENSP00000234701 | 5.90E-05 | 0.7508 | 171 | 0.402 |
| ENSP00000293502 | 9.13E-05 | 0.7485 | 765 | 0.293 |
| ENSP00000262430 | 6.00E-05 | 0.7468 | 234 | 0.496 |
| ENSP00000355140 | 5.00E-05 | 0.7443 | 536 | 0.857 |
| ENSP00000426964 | 5.15E-05 | 0.7442 | 288 | 0.000 |
| ENSP00000358470 | 1.02E-04 | 0.7427 | 198 | 0.426 |
| ENSP00000233826 | 6.31E-05 | 0.7419 | 265 | 0.175 |
| ENSP00000263621 | 5.04E-05 | 0.7414 | 933 | 0.873 |
| ENSP00000296238 | 1.03E-04 | 0.7404 | 800 | 0.101 |
| ENSP00000263062 | 6.63E-05 | 0.7402 | 295 | 0.894 |

| | | | | |
|---|---|---|---|---|
| ENSP00000331210 | 1.36E-04 | 0.7395 | 302 | 0.339 |
| ENSP00000218316 | 4.15E-05 | 0.7386 | 407 | 0.619 |
| ENSP00000375147 | 8.34E-05 | 0.7381 | 212 | 0.142 |
| ENSP00000304767 | 5.11E-05 | 0.7296 | 507 | 0.632 |
| ENSP00000335076 | 8.57E-05 | 0.7289 | 197 | 0.790 |
| ENSP00000357206 | 1.23E-04 | 0.7266 | 382 | 0.948 |
| ENSP00000341762 | 1.03E-04 | 0.7260 | 396 | 0.155 |
| ENSP00000263816 | 8.75E-05 | 0.7258 | 993 | 0.771 |
| ENSP00000241808 | 6.60E-05 | 0.7251 | 581 | 0.306 |
| ENSP00000333519 | 8.41E-05 | 0.7245 | 165 | 0.126 |
| ENSP00000272462 | 8.59E-05 | 0.7241 | 167 | 0.145 |
| ENSP00000369274 | 6.88E-05 | 0.7235 | 163 | 0.261 |
| ENSP00000356399 | 8.60E-05 | 0.7208 | 414 | 0.798 |
| ENSP00000334187 | 7.77E-05 | 0.7191 | 158 | 0.149 |
| ENSP00000246891 | 5.65E-05 | 0.7183 | 358 | 0.279 |
| ENSP00000160262 | 8.31E-05 | 0.7163 | 563 | 0.779 |
| ENSP00000240364 | 1.33E-04 | 0.7161 | 161 | 0.185 |
| ENSP00000359234 | 8.61E-05 | 0.7114 | 150 | 0.132 |
| ENSP00000393887 | 9.79E-05 | 0.7114 | 361 | 0.607 |
| ENSP00000264664 | 5.84E-05 | 0.7100 | 324 | 0.934 |
| ENSP00000175238 | 5.54E-05 | 0.7093 | 242 | 0.179 |
| ENSP00000258530 | 6.04E-05 | 0.7093 | 186 | 0.226 |
| ENSP00000281030 | 1.24E-04 | 0.7085 | 202 | 0.508 |
| ENSP00000287020 | 5.06E-05 | 0.7071 | 206 | 0.895 |
| ENSP00000264382 | 4.90E-05 | 0.7056 | 471 | 0.303 |
| ENSP00000354826 | 1.15E-04 | 0.7045 | 473 | 0.490 |
| ENSP00000231656 | 3.48E-05 | 0.7042 | 189 | 0.889 |
| ENSP00000265983 | 1.55E-04 | 0.7042 | 811 | 0.790 |
| ENSP00000353099 | 6.77E-05 | 0.7036 | 916 | 0.559 |
| ENSP00000384169 | 1.16E-04 | 0.7035 | 910 | 0.521 |
| ENSP00000221204 | 1.94E-04 | 0.6992 | 744 | 0.000 |
| ENSP00000351602 | 6.33E-05 | 0.6982 | 472 | 0.898 |
| ENSP00000373854 | 6.19E-05 | 0.6957 | 970 | 0.546 |
| ENSP00000199764 | 5.07E-05 | 0.6946 | 181 | 0.544 |
| ENSP00000291560 | 7.86E-05 | 0.6927 | 232 | 0.129 |
| ENSP00000292169 | 6.12E-05 | 0.6916 | 664 | 0.404 |
| ENSP00000241124 | 3.86E-05 | 0.6907 | 177 | 0.455 |
| ENSP00000222747 | 7.72E-05 | 0.6905 | 204 | 0.163 |
| ENSP00000221421 | 9.44E-05 | 0.6900 | 224 | 0.765 |
| ENSP00000225992 | 3.63E-05 | 0.6867 | 208 | 0.728 |
| ENSP00000334458 | 6.24E-05 | 0.6831 | 555 | 0.000 |
| ENSP00000269142 | 6.80E-05 | 0.6826 | 191 | 0.773 |
| ENSP00000365081 | 1.22E-04 | 0.6813 | 514 | 0.399 |
| ENSP00000334456 | 5.76E-05 | 0.6813 | 181 | 0.000 |
| ENSP00000293549 | 5.25E-05 | 0.6803 | 696 | 0.929 |
| ENSP00000339730 | 7.33E-05 | 0.6802 | 410 | 0.707 |

| | | | | |
|---|---|---|---|---|
| ENSP00000346627 | 1.16E-04 | 0.6788 | 215 | 0.179 |
| ENSP00000351395 | 6.60E-05 | 0.6780 | 242 | 0.653 |
| ENSP00000257863 | 6.08E-05 | 0.6774 | 190 | 0.830 |
| ENSP00000469538 | 2.28E-06 | 0.6763 | 0 | 0.000 |
| ENSP00000233840 | 6.48E-05 | 0.6740 | 302 | 0.127 |
| ENSP00000288988 | 6.35E-05 | 0.6718 | 387 | 0.081 |
| ENSP00000355896 | 7.72E-05 | 0.6715 | 937 | 0.954 |
| ENSP00000291582 | 4.69E-05 | 0.6714 | 667 | 0.761 |
| ENSP00000243786 | 4.17E-05 | 0.6708 | 204 | 0.885 |
| ENSP00000296589 | 5.76E-05 | 0.6667 | 201 | 0.574 |
| ENSP00000265310 | 4.69E-05 | 0.6660 | 174 | 0.319 |
| ENSP00000225428 | 5.81E-05 | 0.6657 | 382 | 0.000 |
| ENSP00000231188 | 3.39E-05 | 0.6650 | 591 | 0.609 |
| ENSP00000364979 | 1.18E-04 | 0.6640 | 735 | 0.941 |
| ENSP00000302251 | 4.53E-05 | 0.6619 | 239 | 0.864 |
| ENSP00000456711 | 6.96E-05 | 0.6605 | 859 | 0.000 |
| ENSP00000394316 | 2.40E-04 | 0.6584 | 157 | 0.337 |
| ENSP00000305005 | 1.49E-04 | 0.6554 | 151 | 0.145 |
| ENSP00000228434 | 3.25E-05 | 0.6535 | 406 | 0.899 |
| ENSP00000417451 | 6.94E-05 | 0.6533 | 157 | 0.494 |
| ENSP00000350833 | 6.40E-05 | 0.6512 | 223 | 0.150 |
| ENSP00000417677 | 1.33E-04 | 0.6509 | 302 | 0.335 |
| ENSP00000337065 | 7.52E-05 | 0.6429 | 300 | 0.753 |
| ENSP00000254323 | 2.20E-04 | 0.6423 | 224 | 0.147 |
| ENSP00000286955 | 6.62E-05 | 0.6422 | 345 | 0.081 |
| ENSP00000295683 | 5.26E-05 | 0.6415 | 252 | 0.662 |
| ENSP00000370074 | 9.82E-05 | 0.6392 | 223 | 0.599 |
| ENSP00000305906 | 5.95E-05 | 0.6386 | 158 | 0.769 |
| ENSP00000252482 | 5.12E-05 | 0.6374 | 472 | 0.269 |
| ENSP00000249440 | 4.99E-05 | 0.6358 | 258 | 0.848 |
| ENSP00000279028 | 2.34E-04 | 0.6335 | 194 | 0.105 |
| ENSP00000216029 | 1.51E-04 | 0.6329 | 598 | 0.433 |
| ENSP00000274520 | 7.61E-05 | 0.6320 | 205 | 0.749 |
| ENSP00000246080 | 4.93E-05 | 0.6293 | 503 | 0.797 |
| ENSP00000347178 | 6.29E-05 | 0.6290 | 531 | 0.000 |
| ENSP00000369889 | 6.95E-05 | 0.6272 | 846 | 0.946 |
| ENSP00000342850 | 5.68E-05 | 0.6246 | 242 | 0.492 |
| ENSP00000340983 | 5.44E-05 | 0.6213 | 459 | 0.320 |
| ENSP00000354294 | 5.86E-05 | 0.6203 | 288 | 0.208 |
| ENSP00000360882 | 1.11E-04 | 0.6195 | 858 | 0.878 |
| ENSP00000366246 | 5.71E-05 | 0.6185 | 900 | 0.572 |
| ENSP00000369018 | 9.17E-05 | 0.6184 | 220 | 0.000 |
| ENSP00000425493 | 1.18E-04 | 0.6171 | 561 | 0.724 |
| ENSP00000209668 | 3.50E-05 | 0.6141 | 434 | 0.295 |
| ENSP00000452347 | 2.86E-05 | 0.6137 | 396 | 0.000 |
| ENSP00000246895 | 3.67E-05 | 0.6119 | 349 | 0.359 |

| | | | | |
|---|---|---|---|---|
| ENSP00000265441 | 3.88E-05 | 0.6087 | 618 | 0.944 |
| ENSP00000263208 | 5.91E-05 | 0.6078 | 257 | 0.918 |
| ENSP00000363812 | 5.95E-05 | 0.6077 | 238 | 0.792 |
| ENSP00000419945 | 1.34E-04 | 0.6069 | 394 | 0.900 |
| ENSP00000326022 | 6.49E-05 | 0.6048 | 270 | 0.609 |
| ENSP00000416561 | 1.31E-04 | 0.6040 | 337 | 0.773 |
| ENSP00000363689 | 5.61E-05 | 0.6016 | 806 | 0.779 |
| ENSP00000387100 | 1.02E-04 | 0.6014 | 167 | 0.093 |
| ENSP00000435550 | 7.00E-05 | 0.6012 | 227 | 0.164 |
| ENSP00000252505 | 3.72E-05 | 0.5984 | 357 | 0.536 |
| ENSP00000421725 | 9.25E-05 | 0.5979 | 355 | 0.610 |
| ENSP00000312099 | 8.92E-05 | 0.5979 | 712 | 0.297 |
| ENSP00000315013 | 4.55E-05 | 0.5969 | 199 | 0.457 |
| ENSP00000432163 | 9.11E-05 | 0.5955 | 171 | 0.173 |
| ENSP00000306754 | 6.00E-05 | 0.5950 | 165 | 0.791 |
| ENSP00000320217 | 6.18E-05 | 0.5940 | 155 | 0.077 |
| ENSP00000260843 | 9.10E-05 | 0.5939 | 230 | 0.181 |
| ENSP00000250156 | 5.36E-05 | 0.5915 | 158 | 0.000 |
| ENSP00000278282 | 7.17E-05 | 0.5912 | 865 | 0.760 |
| ENSP00000463376 | 6.58E-05 | 0.5878 | 175 | 0.000 |
| ENSP00000285018 | 9.86E-05 | 0.5866 | 695 | 0.934 |
| ENSP00000247933 | 7.01E-05 | 0.5858 | 208 | 0.134 |
| ENSP00000455951 | 2.00E-04 | 0.5851 | 0 | 0.124 |
| ENSP00000437133 | 2.59E-05 | 0.5805 | 338 | 0.000 |
| ENSP00000261892 | 1.07E-04 | 0.5803 | 396 | 0.297 |
| ENSP00000300179 | 8.26E-05 | 0.5798 | 167 | 0.157 |
| ENSP00000431814 | 2.60E-05 | 0.5781 | 338 | 0.000 |
| ENSP00000432029 | 2.60E-05 | 0.5781 | 338 | 0.000 |
| ENSP00000430853 | 2.60E-05 | 0.5781 | 338 | 0.000 |
| ENSP00000433597 | 2.60E-05 | 0.5781 | 338 | 0.000 |
| ENSP00000316328 | 5.17E-05 | 0.5780 | 911 | 0.804 |
| ENSP00000435325 | 2.59E-05 | 0.5772 | 338 | 0.000 |
| ENSP00000377409 | 9.75E-05 | 0.5759 | 216 | 0.767 |
| ENSP00000364076 | 1.01E-04 | 0.5752 | 0 | 0.780 |
| ENSP00000359114 | 6.66E-05 | 0.5745 | 922 | 0.733 |
| ENSP00000225831 | 6.25E-05 | 0.5743 | 961 | 0.959 |
| ENSP00000290953 | 5.06E-05 | 0.5739 | 206 | 0.739 |
| ENSP00000368767 | 8.70E-05 | 0.5735 | 159 | 0.316 |
| ENSP00000310036 | 6.97E-05 | 0.5735 | 659 | 0.940 |
| ENSP00000365394 | 3.82E-05 | 0.5693 | 165 | 0.000 |
| ENSP00000260228 | 8.25E-05 | 0.5685 | 670 | 0.532 |
| ENSP00000309681 | 8.43E-05 | 0.5679 | 167 | 0.141 |
| ENSP00000263339 | 6.11E-05 | 0.5635 | 898 | 0.943 |
| ENSP00000341905 | 8.22E-05 | 0.5616 | 191 | 0.236 |
| ENSP00000270328 | 9.40E-05 | 0.5611 | 214 | 0.194 |
| ENSP00000292596 | 1.21E-04 | 0.5607 | 167 | 0.400 |

| | | | | |
|---|---|---|---|---|
| ENSP00000206423 | 6.71E-05 | 0.5602 | 165 | 0.074 |
| ENSP00000356063 | 7.89E-05 | 0.5601 | 815 | 0.449 |
| ENSP00000237527 | 3.17E-05 | 0.5591 | 268 | 0.732 |
| ENSP00000265417 | 7.90E-05 | 0.5533 | 162 | 0.000 |
| ENSP00000223167 | 2.97E-05 | 0.5529 | 173 | 0.403 |
| ENSP00000302234 | 8.17E-05 | 0.5528 | 563 | 0.884 |
| ENSP00000352427 | 5.25E-05 | 0.5521 | 649 | 0.563 |
| ENSP00000258457 | 6.79E-05 | 0.5482 | 214 | 0.154 |
| ENSP00000357757 | 7.43E-05 | 0.5459 | 150 | 0.000 |
| ENSP00000291707 | 8.64E-05 | 0.5453 | 307 | 0.147 |
| ENSP00000316675 | 9.99E-05 | 0.5451 | 198 | 0.196 |
| ENSP00000269919 | 1.51E-04 | 0.5448 | 759 | 0.146 |
| ENSP00000254605 | 3.29E-05 | 0.5434 | 286 | 0.725 |
| ENSP00000363489 | 6.71E-05 | 0.5422 | 547 | 0.925 |
| ENSP00000299164 | 7.67E-05 | 0.5418 | 265 | 0.211 |
| ENSP00000285402 | 1.03E-04 | 0.5414 | 343 | 0.075 |
| ENSP00000371420 | 1.19E-04 | 0.5396 | 414 | 0.909 |
| ENSP00000222212 | 3.66E-05 | 0.5368 | 156 | 0.106 |
| ENSP00000373363 | 1.31E-04 | 0.5343 | 211 | 0.117 |
| ENSP00000013222 | 6.73E-05 | 0.5330 | 213 | 0.118 |
| ENSP00000353475 | 9.88E-05 | 0.5327 | 185 | 0.498 |
| ENSP00000414287 | 9.70E-05 | 0.5323 | 321 | 0.262 |
| ENSP00000287957 | 9.96E-05 | 0.5307 | 250 | 0.338 |
| ENSP00000376337 | 7.84E-05 | 0.5283 | 0 | 0.157 |
| ENSP00000388320 | 7.97E-05 | 0.5283 | 151 | 0.577 |
| ENSP00000290295 | 6.02E-05 | 0.5279 | 158 | 0.782 |
| ENSP00000221466 | 5.53E-05 | 0.5246 | 197 | 0.208 |
| ENSP00000299721 | 6.25E-05 | 0.5239 | 179 | 0.232 |
| ENSP00000404439 | 2.64E-05 | 0.5205 | 338 | 0.000 |
| ENSP00000321804 | 6.48E-05 | 0.5171 | 165 | 0.138 |
| ENSP00000217381 | 4.67E-05 | 0.5156 | 290 | 0.487 |
| ENSP00000312189 | 3.51E-05 | 0.5152 | 172 | 0.476 |
| ENSP00000302548 | 5.35E-05 | 0.5148 | 357 | 0.826 |
| ENSP00000365105 | 4.77E-05 | 0.5129 | 159 | 0.112 |
| ENSP00000265517 | 5.51E-05 | 0.5094 | 658 | 0.576 |
| ENSP00000259254 | 6.16E-05 | 0.5075 | 159 | 0.319 |
| ENSP00000261615 | 4.33E-05 | 0.5041 | 284 | 0.085 |
| ENSP00000360493 | 8.92E-05 | 0.5027 | 896 | 0.909 |
| ENSP00000358512 | 7.81E-05 | 0.5018 | 167 | 0.149 |
| ENSP00000375482 | 9.24E-05 | 0.5010 | 241 | 0.128 |
| ENSP00000361508 | 6.32E-05 | 0.5008 | 879 | 0.483 |
| ENSP00000337572 | 7.43E-05 | 0.4977 | 330 | 0.086 |
| ENSP00000218436 | 4.98E-05 | 0.4974 | 194 | 0.459 |
| ENSP00000354045 | 8.63E-05 | 0.4964 | 519 | 0.438 |
| ENSP00000321797 | 6.32E-05 | 0.4963 | 372 | 0.912 |
| ENSP00000233809 | 1.02E-04 | 0.4962 | 390 | 0.925 |

| | | | | |
|---|---|---|---|---|
| ENSP00000337022 | 5.48E-05 | 0.4947 | 900 | 0.273 |
| ENSP00000342343 | 4.98E-05 | 0.4927 | 323 | 0.653 |
| ENSP00000254090 | 9.90E-05 | 0.4921 | 197 | 0.113 |
| ENSP00000261883 | 6.49E-05 | 0.4899 | 313 | 0.251 |
| ENSP00000352608 | 8.25E-05 | 0.4892 | 227 | 0.735 |
| ENSP00000357255 | 8.52E-05 | 0.4883 | 816 | 0.910 |
| ENSP00000369699 | 4.37E-05 | 0.4877 | 197 | 0.086 |
| ENSP00000053469 | 2.73E-05 | 0.4867 | 660 | 0.393 |
| ENSP00000292599 | 6.98E-05 | 0.4862 | 295 | 0.821 |
| ENSP00000407375 | 1.03E-04 | 0.4861 | 503 | 0.634 |
| ENSP00000361895 | 7.89E-05 | 0.4860 | 163 | 0.085 |
| ENSP00000222248 | 2.79E-05 | 0.4855 | 244 | 0.329 |
| ENSP00000295728 | 5.49E-05 | 0.4828 | 685 | 0.165 |
| ENSP00000258534 | 7.88E-05 | 0.4826 | 216 | 0.297 |
| ENSP00000243911 | 4.10E-05 | 0.4805 | 345 | 0.595 |
| ENSP00000347117 | 4.31E-05 | 0.4802 | 808 | 0.447 |
| ENSP00000349436 | 7.43E-05 | 0.4788 | 428 | 0.640 |
| ENSP00000300900 | 1.43E-04 | 0.4778 | 360 | 0.510 |
| ENSP00000240328 | 3.31E-05 | 0.4745 | 254 | 0.863 |
| ENSP00000290472 | 5.59E-05 | 0.4739 | 154 | 0.154 |
| ENSP00000236979 | 6.82E-05 | 0.4739 | 150 | 0.324 |
| ENSP00000228644 | 2.59E-05 | 0.4738 | 850 | 0.873 |
| ENSP00000262101 | 5.50E-05 | 0.4721 | 931 | 0.725 |
| ENSP00000356395 | 8.24E-05 | 0.4720 | 239 | 0.430 |
| ENSP00000306606 | 3.91E-05 | 0.4720 | 382 | 0.457 |
| ENSP00000255192 | 6.39E-05 | 0.4713 | 181 | 0.116 |
| ENSP00000329654 | 1.05E-04 | 0.4696 | 280 | 0.159 |
| ENSP00000230036 | 6.40E-05 | 0.4674 | 202 | 0.194 |
| ENSP00000358045 | 1.28E-04 | 0.4660 | 923 | 0.515 |
| ENSP00000354003 | 6.51E-05 | 0.4659 | 358 | 0.778 |
| ENSP00000395359 | 6.78E-05 | 0.4643 | 459 | 0.858 |
| ENSP00000441691 | 1.32E-04 | 0.4635 | 959 | 0.964 |
| ENSP00000381206 | 7.24E-05 | 0.4619 | 206 | 0.145 |
| ENSP00000364709 | 9.18E-05 | 0.4598 | 923 | 0.853 |
| ENSP00000254466 | 5.26E-05 | 0.4583 | 338 | 0.000 |
| ENSP00000229447 | 4.36E-05 | 0.4578 | 388 | 0.163 |
| ENSP00000369400 | 4.92E-05 | 0.4568 | 357 | 0.402 |
| ENSP00000236495 | 6.10E-05 | 0.4566 | 167 | 0.069 |
| ENSP00000348912 | 5.95E-05 | 0.4559 | 349 | 0.566 |
| ENSP00000261292 | 4.58E-05 | 0.4550 | 403 | 0.327 |
| ENSP00000342144 | 4.30E-05 | 0.4507 | 218 | 0.210 |
| ENSP00000288040 | 6.58E-05 | 0.4494 | 151 | 0.086 |
| ENSP00000223368 | 7.52E-05 | 0.4493 | 173 | 0.301 |
| ENSP00000353608 | 6.03E-05 | 0.4491 | 284 | 0.577 |
| ENSP00000371234 | 2.70E-05 | 0.4483 | 362 | 0.376 |
| ENSP00000361355 | 8.27E-05 | 0.4479 | 304 | 0.146 |

| | | | | |
|---|---|---|---|---|
| ENSP00000201586 | 2.74E-05 | 0.4477 | 313 | 0.882 |
| ENSP00000286574 | 1.05E-04 | 0.4473 | 833 | 0.897 |
| ENSP00000369547 | 5.48E-05 | 0.4462 | 159 | 0.147 |
| ENSP00000351671 | 5.60E-05 | 0.4457 | 323 | 0.724 |
| ENSP00000336571 | 4.58E-05 | 0.4450 | 167 | 0.447 |
| ENSP00000331664 | 7.27E-05 | 0.4449 | 192 | 0.000 |
| ENSP00000378517 | 7.12E-05 | 0.4446 | 902 | 0.966 |
| ENSP00000313309 | 4.23E-05 | 0.4440 | 335 | 0.590 |
| ENSP00000264260 | 5.60E-05 | 0.4406 | 212 | 0.602 |
| ENSP00000320376 | 4.36E-05 | 0.4398 | 231 | 0.501 |
| ENSP00000364621 | 9.54E-05 | 0.4397 | 159 | 0.178 |
| ENSP00000314992 | 4.56E-05 | 0.4377 | 263 | 0.209 |
| ENSP00000312029 | 4.45E-05 | 0.4377 | 418 | 0.812 |
| ENSP00000327290 | 4.77E-05 | 0.4377 | 841 | 0.421 |
| ENSP00000233813 | 1.84E-04 | 0.4373 | 652 | 0.920 |
| ENSP00000281321 | 3.68E-05 | 0.4355 | 505 | 0.805 |
| ENSP00000367064 | 5.78E-05 | 0.4345 | 361 | 0.445 |
| ENSP00000282478 | 5.20E-05 | 0.4340 | 519 | 0.783 |
| ENSP00000008938 | 5.06E-05 | 0.4339 | 169 | 0.385 |
| ENSP00000275169 | 5.57E-05 | 0.4319 | 418 | 0.305 |
| ENSP00000327943 | 5.14E-05 | 0.4317 | 236 | 0.142 |
| ENSP00000352565 | 9.29E-05 | 0.4307 | 360 | 0.893 |
| ENSP00000372199 | 7.25E-05 | 0.4303 | 222 | 0.642 |
| ENSP00000228841 | 2.36E-05 | 0.4296 | 907 | 0.639 |
| ENSP00000240093 | 5.00E-05 | 0.4286 | 173 | 0.903 |
| ENSP00000371155 | 6.96E-05 | 0.4286 | 161 | 0.000 |
| ENSP00000333751 | 6.27E-05 | 0.4268 | 0 | 0.095 |
| ENSP00000363708 | 3.38E-05 | 0.4266 | 443 | 0.933 |
| ENSP00000366673 | 9.52E-05 | 0.4247 | 210 | 0.100 |
| ENSP00000359629 | 5.33E-05 | 0.4244 | 159 | 0.265 |
| ENSP00000287766 | 4.30E-05 | 0.4241 | 280 | 0.281 |
| ENSP00000357721 | 1.29E-04 | 0.4234 | 322 | 0.891 |
| ENSP00000262629 | 3.38E-05 | 0.4230 | 215 | 0.678 |
| ENSP00000372313 | 7.94E-05 | 0.4225 | 217 | 0.511 |
| ENSP00000360817 | 8.99E-05 | 0.4210 | 406 | 0.743 |
| ENSP00000448403 | 7.67E-05 | 0.4205 | 158 | 0.000 |
| ENSP00000368066 | 9.55E-05 | 0.4200 | 420 | 0.328 |
| ENSP00000264677 | 1.02E-04 | 0.4181 | 167 | 0.250 |
| ENSP00000242505 | 3.40E-04 | 0.4177 | 157 | 0.124 |
| ENSP00000219660 | 3.38E-05 | 0.4175 | 193 | 0.201 |
| ENSP00000379434 | 7.01E-05 | 0.4174 | 602 | 0.150 |
| ENSP00000313070 | 7.31E-05 | 0.4173 | 388 | 0.151 |
| ENSP00000339086 | 6.91E-05 | 0.4171 | 787 | 0.578 |
| ENSP00000220812 | 2.02E-04 | 0.4165 | 480 | 0.716 |
| ENSP00000269299 | 1.06E-04 | 0.4164 | 802 | 0.140 |
| ENSP00000263593 | 4.05E-05 | 0.4163 | 394 | 0.495 |

| | | | | |
|---|---|---|---|---|
| ENSP00000240652 | 3.63E-05 | 0.4159 | 360 | 0.673 |
| ENSP00000264381 | 3.83E-05 | 0.4134 | 537 | 0.513 |
| ENSP00000292641 | 1.12E-04 | 0.4132 | 499 | 0.111 |
| ENSP00000334122 | 5.16E-05 | 0.4128 | 300 | 0.929 |
| ENSP00000291574 | 4.54E-05 | 0.4121 | 172 | 0.130 |
| ENSP00000240662 | 4.48E-05 | 0.4118 | 157 | 0.282 |
| ENSP00000370989 | 5.85E-05 | 0.4115 | 201 | 0.827 |
| ENSP00000301924 | 6.59E-05 | 0.4115 | 230 | 0.115 |
| ENSP00000322061 | 6.99E-05 | 0.4114 | 576 | 0.141 |
| ENSP00000301258 | 8.05E-05 | 0.4107 | 167 | 0.340 |
| ENSP00000253799 | 3.21E-05 | 0.4107 | 410 | 0.360 |
| ENSP00000257905 | 5.61E-05 | 0.4105 | 225 | 0.457 |
| ENSP00000262968 | 4.84E-05 | 0.4104 | 216 | 0.000 |
| ENSP00000469960 | 1.23E-06 | 0.4101 | 0 | 0.000 |
| ENSP00000282406 | 5.88E-05 | 0.4100 | 254 | 0.157 |
| ENSP00000266304 | 7.66E-05 | 0.4097 | 323 | 0.643 |
| ENSP00000365227 | 6.02E-05 | 0.4085 | 582 | 0.753 |
| ENSP00000354721 | 6.33E-05 | 0.4085 | 157 | 0.068 |
| ENSP00000382101 | 2.71E-05 | 0.4054 | 338 | 0.000 |
| ENSP00000306884 | 3.76E-05 | 0.4044 | 394 | 0.748 |
| ENSP00000411734 | 3.02E-05 | 0.4029 | 165 | 0.000 |
| ENSP00000357362 | 5.58E-05 | 0.4027 | 362 | 0.606 |
| ENSP00000379701 | 7.36E-05 | 0.4025 | 705 | 0.669 |
| ENSP00000353452 | 6.22E-05 | 0.4022 | 608 | 0.865 |
| ENSP00000215376 | 9.24E-05 | 0.4016 | 181 | 0.000 |
| ENSP00000465932 | 6.75E-05 | 0.4015 | 632 | 0.492 |
| ENSP00000353679 | 6.59E-05 | 0.4010 | 802 | 0.912 |
| ENSP00000265800 | 4.83E-05 | 0.3995 | 740 | 0.000 |
| ENSP00000285381 | 1.25E-04 | 0.3995 | 418 | 0.388 |
| ENSP00000395742 | 7.15E-05 | 0.3976 | 390 | 0.528 |
| ENSP00000221498 | 5.46E-05 | 0.3974 | 258 | 0.096 |
| ENSP00000200453 | 6.47E-05 | 0.3969 | 501 | 0.726 |
| ENSP00000342392 | 3.90E-05 | 0.3967 | 271 | 0.866 |
| ENSP00000262017 | 5.75E-05 | 0.3958 | 996 | 0.000 |
| ENSP00000359866 | 4.19E-05 | 0.3958 | 553 | 0.891 |
| ENSP00000219599 | 4.25E-05 | 0.3956 | 337 | 0.120 |
| ENSP00000398979 | 3.23E-05 | 0.3946 | 163 | 0.115 |
| ENSP00000364685 | 1.26E-04 | 0.3944 | 927 | 0.420 |
| ENSP00000244728 | 6.23E-05 | 0.3940 | 282 | 0.175 |
| ENSP00000310216 | 1.01E-04 | 0.3938 | 0 | 0.194 |
| ENSP00000302476 | 5.50E-05 | 0.3937 | 481 | 0.545 |
| ENSP00000357711 | 7.95E-05 | 0.3935 | 971 | 0.316 |
| ENSP00000344829 | 1.14E-04 | 0.3932 | 306 | 0.706 |
| ENSP00000221996 | 5.76E-05 | 0.3931 | 963 | 0.863 |
| ENSP00000341987 | 6.10E-05 | 0.3924 | 207 | 0.000 |
| ENSP00000280097 | 4.13E-05 | 0.3923 | 206 | 0.399 |

| | | | | |
|---|---|---|---|---|
| ENSP00000308597 | 6.41E-05 | 0.3911 | 195 | 0.416 |
| ENSP00000372221 | 9.70E-05 | 0.3900 | 396 | 0.928 |
| ENSP00000254616 | 3.87E-05 | 0.3894 | 150 | 0.620 |
| ENSP00000343930 | 5.78E-05 | 0.3886 | 380 | 0.507 |
| ENSP00000241305 | 5.39E-05 | 0.3883 | 167 | 0.105 |
| ENSP00000360281 | 5.94E-05 | 0.3870 | 576 | 0.272 |
| ENSP00000316938 | 7.86E-05 | 0.3866 | 150 | 0.113 |
| ENSP00000263686 | 7.39E-05 | 0.3856 | 754 | 0.821 |
| ENSP00000346537 | 8.92E-05 | 0.3855 | 411 | 0.094 |
| ENSP00000292303 | 5.91E-05 | 0.3854 | 358 | 0.866 |
| ENSP00000371532 | 8.32E-05 | 0.3838 | 988 | 0.744 |
| ENSP00000345060 | 5.37E-05 | 0.3834 | 198 | 0.000 |
| ENSP00000353512 | 8.08E-05 | 0.3833 | 166 | 0.177 |
| ENSP00000353654 | 1.30E-04 | 0.3821 | 721 | 0.898 |
| ENSP00000348069 | 8.58E-05 | 0.3821 | 889 | 0.789 |
| ENSP00000360305 | 7.76E-05 | 0.3809 | 529 | 0.462 |
| ENSP00000471857 | 1.42E-07 | 0.3805 | 0 | 0.000 |
| ENSP00000357412 | 5.90E-05 | 0.3800 | 195 | 0.124 |
| ENSP00000355785 | 6.22E-05 | 0.3799 | 913 | 0.886 |
| ENSP00000054668 | 3.59E-05 | 0.3798 | 195 | 0.466 |
| ENSP00000318820 | 4.16E-05 | 0.3785 | 205 | 0.716 |
| ENSP00000268122 | 4.72E-05 | 0.3779 | 260 | 0.156 |
| ENSP00000307093 | 4.73E-05 | 0.3778 | 258 | 0.108 |
| ENSP00000417289 | 6.07E-05 | 0.3775 | 186 | 0.131 |
| ENSP00000259631 | 6.56E-05 | 0.3761 | 270 | 0.704 |
| ENSP00000337133 | 6.57E-05 | 0.3753 | 157 | 0.197 |
| ENSP00000265071 | 5.68E-05 | 0.3748 | 288 | 0.398 |
| ENSP00000249504 | 3.56E-05 | 0.3741 | 201 | 0.835 |
| ENSP00000360561 | 3.40E-05 | 0.3723 | 171 | 0.299 |
| ENSP00000346901 | 4.90E-05 | 0.3721 | 150 | 0.126 |
| ENSP00000278550 | 7.35E-05 | 0.3709 | 0 | 0.134 |
| ENSP00000225648 | 3.53E-05 | 0.3701 | 210 | 0.857 |
| ENSP00000332296 | 5.36E-05 | 0.3680 | 505 | 0.887 |
| ENSP00000357348 | 3.85E-05 | 0.3680 | 448 | 0.872 |
| ENSP00000368678 | 7.34E-05 | 0.3671 | 900 | 0.285 |
| ENSP00000349616 | 8.21E-05 | 0.3655 | 390 | 0.552 |
| ENSP00000361554 | 8.38E-05 | 0.3650 | 506 | 0.682 |
| ENSP00000295755 | 5.56E-05 | 0.3635 | 356 | 0.310 |
| ENSP00000228641 | 2.65E-05 | 0.3630 | 179 | 0.867 |
| ENSP00000379138 | 9.46E-05 | 0.3599 | 216 | 0.875 |
| ENSP00000268070 | 8.40E-05 | 0.3594 | 325 | 0.170 |
| ENSP00000293761 | 3.38E-05 | 0.3591 | 216 | 0.356 |
| ENSP00000293599 | 3.45E-05 | 0.3564 | 211 | 0.295 |
| ENSP00000360250 | 5.42E-05 | 0.3563 | 361 | 0.537 |
| ENSP00000355110 | 8.68E-05 | 0.3558 | 512 | 0.624 |
| ENSP00000229304 | 4.44E-05 | 0.3557 | 241 | 0.323 |

| | | | | |
|---|---|---|---|---|
| ENSP00000310447 | 3.35E-05 | 0.3552 | 181 | 0.320 |
| ENSP00000344285 | 5.76E-05 | 0.3537 | 311 | 0.385 |
| ENSP00000298542 | 6.17E-05 | 0.3536 | 158 | 0.144 |
| ENSP00000391668 | 5.70E-05 | 0.3530 | 287 | 0.131 |
| ENSP00000450694 | 2.69E-05 | 0.3529 | 246 | 0.000 |
| ENSP00000320318 | 8.45E-05 | 0.3526 | 812 | 0.091 |
| ENSP00000329312 | 3.25E-05 | 0.3518 | 343 | 0.449 |
| ENSP00000262031 | 5.91E-05 | 0.3511 | 187 | 0.120 |
| ENSP00000299767 | 9.59E-05 | 0.3490 | 999 | 0.837 |
| ENSP00000246553 | 3.95E-05 | 0.3489 | 252 | 0.520 |
| ENSP00000352358 | 5.46E-05 | 0.3466 | 161 | 0.350 |
| ENSP00000364794 | 7.16E-05 | 0.3459 | 397 | 0.730 |
| ENSP00000220597 | 6.88E-05 | 0.3458 | 195 | 0.491 |
| ENSP00000391664 | 8.34E-05 | 0.3453 | 655 | 0.661 |
| ENSP00000287641 | 3.93E-05 | 0.3441 | 499 | 0.903 |
| ENSP00000366942 | 7.96E-05 | 0.3435 | 0 | 0.120 |
| ENSP00000381499 | 4.33E-05 | 0.3410 | 347 | 0.859 |
| ENSP00000258302 | 4.25E-05 | 0.3401 | 189 | 0.380 |
| ENSP00000366623 | 4.78E-05 | 0.3400 | 282 | 0.000 |
| ENSP00000416037 | 4.93E-05 | 0.3393 | 264 | 0.145 |
| ENSP00000422464 | 4.23E-05 | 0.3379 | 859 | 0.862 |
| ENSP00000397082 | 7.31E-05 | 0.3334 | 211 | 0.483 |
| ENSP00000282020 | 5.33E-05 | 0.3334 | 0 | 0.207 |
| ENSP00000365944 | 8.69E-05 | 0.3326 | 173 | 0.150 |
| ENSP00000219593 | 3.88E-05 | 0.3319 | 242 | 0.000 |
| ENSP00000369442 | 4.88E-05 | 0.3298 | 416 | 0.786 |
| ENSP00000420976 | 5.74E-05 | 0.3298 | 325 | 0.144 |
| ENSP00000243924 | 2.17E-05 | 0.3290 | 357 | 0.922 |
| ENSP00000373570 | 5.04E-05 | 0.3289 | 822 | 0.803 |
| ENSP00000350467 | 5.20E-05 | 0.3276 | 345 | 0.908 |
| ENSP00000269216 | 2.70E-05 | 0.3265 | 940 | 0.000 |
| ENSP00000300055 | 4.88E-05 | 0.3260 | 392 | 0.679 |
| ENSP00000275525 | 6.08E-05 | 0.3259 | 412 | 0.906 |
| ENSP00000251582 | 1.06E-04 | 0.3241 | 361 | 0.621 |
| ENSP00000351310 | 7.16E-05 | 0.3232 | 161 | 0.208 |
| ENSP00000224605 | 3.30E-05 | 0.3221 | 218 | 0.000 |
| ENSP00000345259 | 5.88E-05 | 0.3219 | 858 | 0.367 |
| ENSP00000256857 | 3.02E-05 | 0.3219 | 398 | 0.817 |
| ENSP00000364635 | 6.69E-05 | 0.3210 | 286 | 0.170 |
| ENSP00000362463 | 5.09E-05 | 0.3210 | 244 | 0.486 |
| ENSP00000331871 | 6.85E-05 | 0.3195 | 745 | 0.566 |
| ENSP00000377947 | 5.62E-05 | 0.3182 | 390 | 0.858 |
| ENSP00000256010 | 3.20E-05 | 0.3180 | 822 | 0.742 |
| ENSP00000316339 | 7.96E-05 | 0.3162 | 377 | 0.341 |
| ENSP00000375109 | 3.83E-05 | 0.3161 | 171 | 0.129 |
| ENSP00000310998 | 3.11E-05 | 0.3159 | 288 | 0.350 |

| | | | | |
|---|---|---|---|---|
| ENSP00000335156 | 8.44E-05 | 0.3156 | 181 | 0.000 |
| ENSP00000216180 | 4.03E-05 | 0.3152 | 179 | 0.486 |
| ENSP00000307445 | 3.33E-05 | 0.3149 | 501 | 0.699 |
| ENSP00000004921 | 1.60E-04 | 0.3136 | 359 | 0.000 |
| ENSP00000253270 | 4.28E-05 | 0.3129 | 900 | 0.070 |
| ENSP00000319635 | 4.88E-05 | 0.3128 | 505 | 0.860 |
| ENSP00000355812 | 6.48E-05 | 0.3100 | 576 | 0.077 |
| ENSP00000369252 | 4.66E-05 | 0.3099 | 254 | 0.000 |
| ENSP00000316228 | 4.71E-05 | 0.3089 | 209 | 0.492 |
| ENSP00000164024 | 3.46E-05 | 0.3085 | 323 | 0.527 |
| ENSP00000252575 | 9.59E-05 | 0.3084 | 510 | 0.515 |
| ENSP00000267953 | 3.68E-05 | 0.3080 | 355 | 0.902 |
| ENSP00000366244 | 6.35E-05 | 0.3078 | 659 | 0.875 |
| ENSP00000286744 | 8.59E-05 | 0.3072 | 381 | 0.161 |
| ENSP00000299847 | 3.75E-05 | 0.3071 | 270 | 0.665 |
| ENSP00000315383 | 4.50E-05 | 0.3064 | 250 | 0.256 |
| ENSP00000342070 | 1.61E-04 | 0.3061 | 921 | 0.908 |
| ENSP00000240333 | 6.67E-05 | 0.3056 | 170 | 0.082 |
| ENSP00000363868 | 5.93E-05 | 0.3040 | 973 | 0.850 |
| ENSP00000385879 | 6.34E-05 | 0.3034 | 264 | 0.133 |
| ENSP00000405455 | 6.26E-05 | 0.3034 | 488 | 0.585 |
| ENSP00000369399 | 5.50E-05 | 0.3029 | 376 | 0.138 |
| ENSP00000334876 | 4.93E-05 | 0.3024 | 999 | 0.632 |
| ENSP00000312027 | 4.39E-05 | 0.3023 | 240 | 0.631 |
| ENSP00000355512 | 3.70E-05 | 0.3020 | 284 | 0.493 |
| ENSP00000408581 | 5.22E-05 | 0.3017 | 826 | 0.547 |
| ENSP00000352835 | 8.71E-05 | 0.3016 | 836 | 0.710 |
| ENSP00000302079 | 4.25E-05 | 0.3006 | 198 | 0.611 |
| ENSP00000307879 | 1.33E-04 | 0.2999 | 216 | 0.119 |
| ENSP00000301691 | 8.70E-05 | 0.2993 | 499 | 0.893 |
| ENSP00000324570 | 9.10E-05 | 0.2983 | 0 | 0.089 |
| ENSP00000318032 | 5.82E-05 | 0.2982 | 165 | 0.098 |
| ENSP00000344489 | 5.30E-05 | 0.2982 | 177 | 0.104 |
| ENSP00000360541 | 4.47E-05 | 0.2975 | 229 | 0.574 |
| ENSP00000257408 | 3.45E-05 | 0.2962 | 201 | 0.275 |
| ENSP00000337383 | 4.55E-05 | 0.2960 | 284 | 0.862 |
| ENSP00000365343 | 6.32E-05 | 0.2947 | 167 | 0.000 |
| ENSP00000357668 | 6.38E-05 | 0.2939 | 501 | 0.799 |
| ENSP00000327545 | 5.32E-05 | 0.2934 | 242 | 0.347 |
| ENSP00000354734 | 3.66E-05 | 0.2930 | 398 | 0.432 |
| ENSP00000370588 | 4.60E-05 | 0.2927 | 585 | 0.645 |
| ENSP00000287706 | 4.25E-05 | 0.2923 | 499 | 0.252 |
| ENSP00000225538 | 3.21E-05 | 0.2913 | 356 | 0.489 |
| ENSP00000229030 | 4.10E-05 | 0.2912 | 290 | 0.856 |
| ENSP00000367891 | 5.87E-05 | 0.2904 | 906 | 0.348 |
| ENSP00000353720 | 5.36E-05 | 0.2899 | 842 | 0.584 |

| | | | | |
|---|---|---|---|---|
| ENSP00000363157 | 4.22E-05 | 0.2896 | 211 | 0.681 |
| ENSP00000351190 | 4.90E-05 | 0.2878 | 356 | 0.443 |
| ENSP00000399679 | 3.78E-05 | 0.2876 | 487 | 0.742 |
| ENSP00000382237 | 5.34E-05 | 0.2862 | 226 | 0.092 |
| ENSP00000386043 | 6.39E-05 | 0.2829 | 910 | 0.669 |
| ENSP00000353331 | 4.89E-05 | 0.2818 | 386 | 0.201 |
| ENSP00000205143 | 3.66E-05 | 0.2815 | 241 | 0.843 |
| ENSP00000349547 | 8.12E-05 | 0.2798 | 245 | 0.863 |
| ENSP00000354053 | 8.02E-05 | 0.2798 | 159 | 0.137 |
| ENSP00000356213 | 4.03E-05 | 0.2787 | 471 | 0.834 |
| ENSP00000233957 | 4.55E-05 | 0.2780 | 204 | 0.608 |
| ENSP00000268164 | 3.84E-05 | 0.2774 | 360 | 0.240 |
| ENSP00000217386 | 4.19E-05 | 0.2754 | 463 | 0.817 |
| ENSP00000331358 | 5.57E-05 | 0.2753 | 842 | 0.885 |
| ENSP00000180166 | 2.87E-05 | 0.2742 | 184 | 0.944 |
| ENSP00000391397 | 6.05E-05 | 0.2742 | 451 | 0.150 |
| ENSP00000358071 | 5.72E-05 | 0.2742 | 165 | 0.559 |
| ENSP00000372295 | 5.02E-05 | 0.2732 | 213 | 0.456 |
| ENSP00000345023 | 5.62E-05 | 0.2728 | 235 | 0.345 |
| ENSP00000303424 | 4.66E-05 | 0.2715 | 302 | 0.663 |
| ENSP00000357981 | 1.01E-04 | 0.2714 | 716 | 0.904 |
| ENSP00000161559 | 3.17E-05 | 0.2712 | 242 | 0.740 |
| ENSP00000364000 | 5.61E-05 | 0.2710 | 835 | 0.722 |
| ENSP00000352900 | 5.05E-05 | 0.2709 | 322 | 0.822 |
| ENSP00000332659 | 3.76E-05 | 0.2708 | 288 | 0.629 |
| ENSP00000358525 | 7.30E-05 | 0.2707 | 307 | 0.950 |
| ENSP00000376423 | 4.71E-05 | 0.2705 | 193 | 0.379 |
| ENSP00000334003 | 4.23E-05 | 0.2701 | 227 | 0.498 |
| ENSP00000346829 | 5.28E-05 | 0.2688 | 269 | 0.614 |
| ENSP00000321684 | 5.07E-05 | 0.2685 | 206 | 0.842 |
| ENSP00000328412 | 4.50E-05 | 0.2685 | 564 | 0.402 |
| ENSP00000272224 | 3.36E-05 | 0.2684 | 162 | 0.872 |
| ENSP00000217270 | 3.24E-05 | 0.2681 | 296 | 0.725 |
| ENSP00000265708 | 2.72E-05 | 0.2665 | 161 | 0.467 |
| ENSP00000262653 | 3.30E-05 | 0.2662 | 349 | 0.584 |
| ENSP00000430487 | 1.22E-04 | 0.2659 | 614 | 0.263 |
| ENSP00000330774 | 8.31E-05 | 0.2658 | 159 | 0.000 |
| ENSP00000355609 | 7.28E-05 | 0.2652 | 219 | 0.116 |
| ENSP00000422846 | 7.40E-05 | 0.2649 | 181 | 0.402 |
| ENSP00000357998 | 6.55E-05 | 0.2647 | 851 | 0.347 |
| ENSP00000369962 | 6.32E-05 | 0.2637 | 150 | 0.094 |
| ENSP00000349003 | 4.15E-05 | 0.2631 | 864 | 0.782 |
| ENSP00000393099 | 8.54E-05 | 0.2626 | 159 | 0.173 |
| ENSP00000357727 | 9.40E-05 | 0.2616 | 483 | 0.798 |
| ENSP00000298861 | 1.14E-04 | 0.2615 | 216 | 0.000 |
| ENSP00000217961 | 3.25E-05 | 0.2613 | 301 | 0.723 |

| | | | | |
|---|---|---|---|---|
| ENSP00000352561 | 7.90E-05 | 0.2612 | 414 | 0.786 |
| ENSP00000262352 | 2.63E-05 | 0.2600 | 211 | 0.214 |
| ENSP00000410452 | 7.47E-05 | 0.2592 | 657 | 0.547 |
| ENSP00000263525 | 3.30E-05 | 0.2592 | 216 | 0.586 |
| ENSP00000361232 | 4.40E-05 | 0.2586 | 280 | 0.869 |
| ENSP00000372224 | 3.10E-05 | 0.2586 | 286 | 0.482 |
| ENSP00000287097 | 1.06E-04 | 0.2584 | 156 | 0.126 |
| ENSP00000371886 | 4.45E-05 | 0.2580 | 374 | 0.136 |
| ENSP00000358966 | 5.74E-05 | 0.2564 | 241 | 0.182 |
| ENSP00000220562 | 2.37E-05 | 0.2559 | 390 | 0.458 |
| ENSP00000254442 | 5.21E-05 | 0.2557 | 561 | 0.104 |
| ENSP00000354130 | 6.75E-05 | 0.2553 | 242 | 0.893 |
| ENSP00000297161 | 5.73E-05 | 0.2546 | 191 | 0.747 |
| ENSP00000349677 | 5.56E-05 | 0.2544 | 760 | 0.115 |
| ENSP00000301653 | 8.66E-05 | 0.2537 | 418 | 0.959 |
| ENSP00000177648 | 1.34E-04 | 0.2534 | 198 | 0.190 |
| ENSP00000296875 | 3.30E-05 | 0.2530 | 203 | 0.879 |
| ENSP00000257189 | 4.48E-05 | 0.2528 | 151 | 0.384 |
| ENSP00000287387 | 5.71E-05 | 0.2495 | 197 | 0.073 |
| ENSP00000352540 | 5.41E-05 | 0.2494 | 204 | 0.166 |
| ENSP00000235628 | 2.54E-05 | 0.2480 | 286 | 0.240 |
| ENSP00000389275 | 2.39E-05 | 0.2479 | 338 | 0.000 |
| ENSP00000350425 | 6.80E-05 | 0.2472 | 927 | 0.600 |
| ENSP00000345873 | 4.31E-05 | 0.2469 | 381 | 0.585 |
| ENSP00000309007 | 9.14E-05 | 0.2468 | 194 | 0.174 |
| ENSP00000319799 | 4.34E-05 | 0.2466 | 479 | 0.847 |
| ENSP00000331514 | 7.24E-05 | 0.2453 | 543 | 0.715 |
| ENSP00000405718 | 5.46E-05 | 0.2453 | 191 | 0.000 |
| ENSP00000357452 | 5.44E-05 | 0.2434 | 169 | 0.362 |
| ENSP00000267889 | 6.33E-05 | 0.2418 | 181 | 0.314 |
| ENSP00000258888 | 5.65E-05 | 0.2417 | 252 | 0.097 |
| ENSP00000257209 | 4.08E-05 | 0.2413 | 153 | 0.126 |
| ENSP00000251645 | 2.48E-05 | 0.2410 | 360 | 0.285 |
| ENSP00000356584 | 8.49E-05 | 0.2399 | 150 | 0.117 |
| ENSP00000352011 | 4.11E-05 | 0.2395 | 658 | 0.400 |
| ENSP00000337340 | 3.97E-05 | 0.2387 | 328 | 0.681 |
| ENSP00000229402 | 2.91E-05 | 0.2386 | 179 | 0.604 |
| ENSP00000428230 | 1.78E-05 | 0.2385 | 156 | 0.000 |
| ENSP00000441828 | 5.72E-05 | 0.2376 | 252 | 0.415 |
| ENSP00000354458 | 3.57E-05 | 0.2374 | 0 | 0.289 |
| ENSP00000270800 | 5.37E-05 | 0.2370 | 158 | 0.598 |
| ENSP00000378400 | 5.68E-05 | 0.2364 | 164 | 0.250 |
| ENSP00000273968 | 3.90E-05 | 0.2361 | 216 | 0.285 |
| ENSP00000278949 | 8.47E-05 | 0.2360 | 150 | 0.135 |
| ENSP00000273550 | 1.11E-04 | 0.2355 | 383 | 0.605 |
| ENSP00000381895 | 3.26E-02 | 0.2353 | 0 | 0.198 |

| | | | | |
|---|---|---|---|---|
| ENSP00000265162 | 1.37E-04 | 0.2351 | 747 | 0.538 |
| ENSP00000389338 | 3.26E-02 | 0.2346 | 739 | 0.949 |
| ENSP00000305924 | 6.06E-05 | 0.2344 | 309 | 0.074 |
| ENSP00000377176 | 5.29E-05 | 0.2338 | 222 | 0.502 |
| ENSP00000257312 | 5.12E-05 | 0.2328 | 153 | 0.237 |
| ENSP00000262999 | 2.79E-05 | 0.2326 | 390 | 0.823 |
| ENSP00000352702 | 1.48E-04 | 0.2309 | 212 | 0.098 |
| ENSP00000312326 | 8.34E-05 | 0.2305 | 408 | 0.591 |
| ENSP00000252338 | 2.46E-05 | 0.2305 | 217 | 0.410 |
| ENSP00000356024 | 2.81E-05 | 0.2296 | 340 | 0.877 |
| ENSP00000352717 | 5.60E-05 | 0.2293 | 159 | 0.000 |
| ENSP00000356370 | 3.98E-05 | 0.2290 | 580 | 0.472 |
| ENSP00000337128 | 3.07E-05 | 0.2279 | 254 | 0.700 |
| ENSP00000270288 | 4.04E-05 | 0.2266 | 159 | 0.000 |
| ENSP00000233954 | 3.65E-05 | 0.2261 | 173 | 0.638 |
| ENSP00000352047 | 3.97E-05 | 0.2261 | 357 | 0.522 |
| ENSP00000410910 | 9.36E-05 | 0.2260 | 193 | 0.085 |
| ENSP00000444565 | 7.92E-05 | 0.2245 | 150 | 0.131 |
| ENSP00000306245 | 9.81E-05 | 0.2237 | 954 | 0.000 |
| ENSP00000353847 | 4.42E-05 | 0.2234 | 983 | 0.734 |
| ENSP00000295834 | 7.88E-05 | 0.2230 | 714 | 0.608 |
| ENSP00000265052 | 4.10E-05 | 0.2226 | 343 | 0.489 |
| ENSP00000398350 | 7.77E-05 | 0.2226 | 796 | 0.144 |
| ENSP00000294413 | 2.74E-05 | 0.2219 | 571 | 0.350 |
| ENSP00000370808 | 7.73E-05 | 0.2215 | 358 | 0.430 |
| ENSP00000358945 | 3.38E-05 | 0.2214 | 155 | 0.412 |
| ENSP00000357753 | 8.60E-05 | 0.2213 | 345 | 0.796 |
| ENSP00000006275 | 4.00E-05 | 0.2192 | 230 | 0.104 |
| ENSP00000345344 | 6.57E-05 | 0.2192 | 350 | 0.000 |
| ENSP00000251535 | 4.26E-05 | 0.2191 | 215 | 0.362 |
| ENSP00000409007 | 4.59E-05 | 0.2174 | 254 | 0.925 |
| ENSP00000291232 | 4.36E-05 | 0.2167 | 163 | 0.754 |
| ENSP00000371785 | 3.05E-05 | 0.2160 | 252 | 0.816 |
| ENSP00000247020 | 4.66E-05 | 0.2158 | 160 | 0.126 |
| ENSP00000221485 | 2.70E-05 | 0.2151 | 216 | 0.494 |
| ENSP00000398736 | 1.26E-04 | 0.2146 | 702 | 0.953 |
| ENSP00000252825 | 6.13E-05 | 0.2138 | 158 | 0.244 |
| ENSP00000308541 | 1.20E-04 | 0.2136 | 944 | 0.972 |
| ENSP00000285311 | 4.28E-05 | 0.2135 | 531 | 0.693 |
| ENSP00000318916 | 3.64E-05 | 0.2131 | 505 | 0.270 |
| ENSP00000369129 | 5.07E-05 | 0.2131 | 676 | 0.747 |
| ENSP00000239374 | 3.73E-05 | 0.2104 | 240 | 0.220 |
| ENSP00000362299 | 7.25E-05 | 0.2102 | 507 | 0.890 |
| ENSP00000290866 | 7.96E-05 | 0.2100 | 725 | 0.909 |
| ENSP00000354219 | 5.21E-05 | 0.2070 | 912 | 0.648 |
| ENSP00000362410 | 5.20E-05 | 0.2064 | 598 | 0.813 |

| | | | | |
|---|---|---|---|---|
| ENSP00000371110 | 7.00E-05 | 0.2058 | 340 | 0.397 |
| ENSP00000363680 | 6.18E-05 | 0.2024 | 293 | 0.806 |
| ENSP00000334472 | 9.81E-05 | 0.2023 | 502 | 0.000 |
| ENSP00000344822 | 7.01E-05 | 0.2010 | 150 | 0.134 |
| ENSP00000341550 | 4.37E-05 | 0.2009 | 208 | 0.231 |
| ENSP00000267838 | 9.77E-05 | 0.2007 | 216 | 0.126 |
| ENSP00000457031 | 4.50E-05 | 0.2004 | 292 | 0.156 |
| ENSP00000171757 | 3.39E-05 | 0.2002 | 221 | 0.412 |
| ENSP00000307369 | 3.27E-05 | 0.2000 | 150 | 0.116 |
| ENSP00000341170 | 6.87E-05 | 0.1993 | 360 | 0.904 |
| ENSP00000346791 | 2.30E-04 | 0.1991 | 416 | 0.323 |
| ENSP00000254908 | 7.27E-05 | 0.1984 | 165 | 0.111 |
| ENSP00000400365 | 5.06E-05 | 0.1981 | 270 | 0.774 |
| ENSP00000395498 | 4.66E-05 | 0.1980 | 173 | 0.883 |
| ENSP00000262487 | 6.59E-05 | 0.1974 | 179 | 0.087 |
| ENSP00000291536 | 4.74E-05 | 0.1972 | 171 | 0.092 |
| ENSP00000381693 | 6.07E-05 | 0.1961 | 150 | 0.105 |
| ENSP00000348384 | 3.84E-05 | 0.1958 | 234 | 0.643 |
| ENSP00000414302 | 4.10E-05 | 0.1954 | 204 | 0.726 |
| ENSP00000307900 | 9.05E-05 | 0.1952 | 562 | 0.704 |
| ENSP00000389026 | 1.26E-04 | 0.1951 | 182 | 0.150 |
| ENSP00000362159 | 4.71E-05 | 0.1950 | 830 | 0.100 |
| ENSP00000252506 | 3.78E-05 | 0.1947 | 150 | 0.682 |
| ENSP00000262041 | 6.19E-05 | 0.1947 | 309 | 0.859 |
| ENSP00000327850 | 3.85E-05 | 0.1946 | 284 | 0.912 |
| ENSP00000344789 | 8.36E-05 | 0.1943 | 466 | 0.000 |
| ENSP00000388666 | 4.63E-05 | 0.1942 | 636 | 0.747 |
| ENSP00000359864 | 7.28E-05 | 0.1942 | 905 | 0.687 |
| ENSP00000418491 | 2.99E-05 | 0.1924 | 0 | 0.142 |
| ENSP00000282018 | 4.83E-05 | 0.1905 | 198 | 0.458 |
| ENSP00000308252 | 4.52E-05 | 0.1904 | 262 | 0.841 |
| ENSP00000337212 | 5.53E-05 | 0.1896 | 256 | 0.585 |
| ENSP00000379204 | 9.49E-05 | 0.1890 | 913 | 0.929 |
| ENSP00000358831 | 7.53E-05 | 0.1886 | 541 | 0.331 |
| ENSP00000261917 | 2.90E-05 | 0.1876 | 154 | 0.406 |
| ENSP00000290294 | 3.97E-05 | 0.1868 | 198 | 0.000 |
| ENSP00000360502 | 5.03E-05 | 0.1865 | 580 | 0.650 |
| ENSP00000420295 | 6.08E-05 | 0.1863 | 725 | 0.665 |
| ENSP00000349252 | 4.35E-05 | 0.1858 | 725 | 0.864 |
| ENSP00000311291 | 3.73E-05 | 0.1855 | 159 | 0.123 |
| ENSP00000358951 | 3.37E-05 | 0.1847 | 156 | 0.000 |
| ENSP00000349274 | 3.33E-05 | 0.1845 | 197 | 0.524 |
| ENSP00000401678 | 5.74E-05 | 0.1843 | 995 | 0.918 |
| ENSP00000385834 | 1.67E-04 | 0.1838 | 579 | 0.948 |
| ENSP00000278200 | 2.62E-05 | 0.1837 | 507 | 0.336 |
| ENSP00000379823 | 4.50E-05 | 0.1836 | 696 | 0.690 |

| | | | | |
|---|---|---|---|---|
| ENSP00000222718 | 6.57E-05 | 0.1832 | 800 | 0.854 |
| ENSP00000290167 | 5.62E-05 | 0.1830 | 679 | 0.930 |
| ENSP00000370115 | 5.21E-05 | 0.1825 | 300 | 0.759 |
| ENSP00000441600 | 5.57E-05 | 0.1824 | 242 | 0.678 |
| ENSP00000430236 | 6.88E-05 | 0.1820 | 193 | 0.387 |
| ENSP00000274545 | 4.14E-05 | 0.1819 | 168 | 0.347 |
| ENSP00000297316 | 2.32E-05 | 0.1817 | 193 | 0.877 |
| ENSP00000282561 | 9.10E-05 | 0.1813 | 479 | 0.944 |
| ENSP00000258411 | 2.83E-05 | 0.1803 | 544 | 0.912 |
| ENSP00000308107 | 9.00E-05 | 0.1799 | 509 | 0.801 |
| ENSP00000332170 | 5.77E-05 | 0.1798 | 900 | 0.081 |
| ENSP00000341327 | 4.56E-05 | 0.1792 | 158 | 0.646 |
| ENSP00000262407 | 6.71E-05 | 0.1771 | 991 | 0.926 |
| ENSP00000382086 | 2.24E-05 | 0.1769 | 338 | 0.493 |
| ENSP00000221554 | 2.94E-05 | 0.1768 | 286 | 0.466 |
| ENSP00000264426 | 4.59E-05 | 0.1765 | 341 | 0.536 |
| ENSP00000295731 | 3.09E-05 | 0.1752 | 583 | 0.942 |
| ENSP00000373574 | 4.30E-05 | 0.1744 | 407 | 0.928 |
| ENSP00000351593 | 7.30E-05 | 0.1738 | 793 | 0.609 |
| ENSP00000305941 | 6.20E-05 | 0.1734 | 472 | 0.513 |
| ENSP00000313833 | 7.21E-05 | 0.1734 | 0 | 0.177 |
| ENSP00000354919 | 7.94E-05 | 0.1731 | 0 | 0.072 |
| ENSP00000258317 | 3.47E-05 | 0.1730 | 345 | 0.472 |
| ENSP00000406367 | 6.63E-05 | 0.1729 | 206 | 0.000 |
| ENSP00000347931 | 5.11E-05 | 0.1729 | 549 | 0.188 |
| ENSP00000355629 | 5.56E-05 | 0.1721 | 812 | 0.590 |
| ENSP00000340913 | 3.14E-05 | 0.1721 | 428 | 0.688 |
| ENSP00000361125 | 1.38E-04 | 0.1719 | 994 | 0.000 |
| ENSP00000455434 | 3.32E-05 | 0.1715 | 157 | 0.111 |
| ENSP00000353660 | 4.97E-05 | 0.1714 | 238 | 0.723 |
| ENSP00000263233 | 3.60E-05 | 0.1713 | 394 | 0.385 |
| ENSP00000317331 | 6.17E-05 | 0.1711 | 216 | 0.173 |
| ENSP00000354207 | 4.87E-05 | 0.1703 | 229 | 0.891 |
| ENSP00000438833 | 8.73E-05 | 0.1701 | 321 | 0.000 |
| ENSP00000302639 | 3.15E-05 | 0.1694 | 300 | 0.000 |
| ENSP00000303111 | 3.15E-05 | 0.1693 | 325 | 0.000 |
| ENSP00000281923 | 3.06E-05 | 0.1692 | 212 | 0.211 |
| ENSP00000264497 | 3.91E-05 | 0.1692 | 270 | 0.735 |
| ENSP00000355317 | 9.36E-05 | 0.1686 | 949 | 0.796 |
| ENSP00000429734 | 2.39E-05 | 0.1678 | 338 | 0.000 |
| ENSP00000384534 | 4.64E-05 | 0.1675 | 250 | 0.300 |
| ENSP00000418648 | 6.83E-05 | 0.1675 | 0 | 0.118 |
| ENSP00000246012 | 3.34E-05 | 0.1649 | 157 | 0.582 |
| ENSP00000359686 | 6.57E-05 | 0.1647 | 0 | 0.000 |
| ENSP00000372193 | 6.20E-05 | 0.1646 | 211 | 0.164 |
| ENSP00000362734 | 3.70E-05 | 0.1641 | 189 | 0.654 |

| | | | | |
|---|---|---|---|---|
| ENSP00000357863 | 4.97E-05 | 0.1639 | 167 | 0.078 |
| ENSP00000381768 | 4.68E-05 | 0.1636 | 205 | 0.664 |
| ENSP00000363965 | 5.26E-05 | 0.1631 | 370 | 0.608 |
| ENSP00000361189 | 4.62E-05 | 0.1618 | 203 | 0.374 |
| ENSP00000355124 | 1.01E-04 | 0.1616 | 383 | 0.903 |
| ENSP00000398971 | 6.47E-05 | 0.1613 | 321 | 0.000 |
| ENSP00000342434 | 3.25E-05 | 0.1609 | 443 | 0.938 |
| ENSP00000253392 | 1.37E-04 | 0.1607 | 227 | 0.832 |
| ENSP00000431840 | 7.87E-05 | 0.1605 | 173 | 0.097 |
| ENSP00000373487 | 8.09E-05 | 0.1597 | 637 | 0.832 |
| ENSP00000352177 | 1.47E-04 | 0.1596 | 0 | 0.107 |
| ENSP00000337432 | 3.75E-05 | 0.1581 | 239 | 0.873 |
| ENSP00000357789 | 6.48E-05 | 0.1578 | 562 | 0.688 |
| ENSP00000301825 | 3.07E-05 | 0.1577 | 158 | 0.269 |
| ENSP00000360519 | 6.18E-05 | 0.1576 | 247 | 0.224 |
| ENSP00000449598 | 1.97E-05 | 0.1573 | 202 | 0.102 |
| ENSP00000353393 | 2.94E-05 | 0.1571 | 577 | 0.216 |
| ENSP00000367151 | 6.50E-05 | 0.1554 | 0 | 0.103 |
| ENSP00000358223 | 4.21E-05 | 0.1542 | 472 | 0.394 |
| ENSP00000261652 | 4.68E-05 | 0.1540 | 159 | 0.737 |
| ENSP00000339191 | 1.03E-04 | 0.1538 | 820 | 0.905 |
| ENSP00000392423 | 4.89E-05 | 0.1537 | 923 | 0.883 |
| ENSP00000296099 | 2.85E-05 | 0.1528 | 169 | 0.597 |
| ENSP00000368966 | 3.12E-05 | 0.1520 | 374 | 0.650 |
| ENSP00000218721 | 3.44E-05 | 0.1519 | 252 | 0.492 |
| ENSP00000265023 | 6.45E-05 | 0.1510 | 961 | 0.845 |
| ENSP00000295619 | 3.03E-05 | 0.1505 | 179 | 0.646 |
| ENSP00000276414 | 4.57E-05 | 0.1497 | 234 | 0.802 |
| ENSP00000347649 | 3.43E-04 | 0.1493 | 156 | 0.000 |
| ENSP00000227665 | 3.52E-05 | 0.1491 | 969 | 0.396 |
| ENSP00000258955 | 4.01E-05 | 0.1487 | 284 | 0.074 |
| ENSP00000268603 | 9.13E-05 | 0.1481 | 311 | 0.652 |
| ENSP00000300177 | 4.74E-05 | 0.1481 | 471 | 0.933 |
| ENSP00000244513 | 2.85E-05 | 0.1476 | 254 | 0.238 |
| ENSP00000339804 | 4.56E-05 | 0.1472 | 240 | 0.820 |
| ENSP00000358677 | 2.76E-05 | 0.1461 | 212 | 0.839 |
| ENSP00000379804 | 5.51E-05 | 0.1457 | 427 | 0.131 |
| ENSP00000457706 | 1.57E-04 | 0.1452 | 931 | 0.908 |
| ENSP00000329158 | 3.97E-05 | 0.1449 | 242 | 0.313 |
| ENSP00000327758 | 2.92E-05 | 0.1448 | 613 | 0.879 |
| ENSP00000380903 | 4.25E-05 | 0.1441 | 256 | 0.612 |
| ENSP00000234739 | 4.71E-05 | 0.1435 | 216 | 0.589 |
| ENSP00000258443 | 6.89E-05 | 0.1429 | 258 | 0.863 |
| ENSP00000361027 | 4.35E-05 | 0.1420 | 181 | 0.478 |
| ENSP00000328973 | 1.16E-04 | 0.1406 | 949 | 0.000 |
| ENSP00000362205 | 4.28E-05 | 0.1402 | 234 | 0.404 |

| | | | | |
|---|---|---|---|---|
| ENSP00000262345 | 3.31E-05 | 0.1402 | 201 | 0.768 |
| ENSP00000374566 | 3.95E-05 | 0.1401 | 305 | 0.239 |
| ENSP00000346572 | 2.97E-05 | 0.1400 | 338 | 0.000 |
| ENSP00000295743 | 2.65E-05 | 0.1399 | 159 | 0.864 |
| ENSP00000257770 | 6.57E-05 | 0.1399 | 652 | 0.769 |
| ENSP00000371875 | 2.99E-05 | 0.1398 | 298 | 0.858 |
| ENSP00000369461 | 5.53E-05 | 0.1395 | 210 | 0.331 |
| ENSP00000346987 | 1.17E-04 | 0.1393 | 319 | 0.373 |
| ENSP00000309757 | 9.18E-05 | 0.1392 | 997 | 0.854 |
| ENSP00000053867 | 1.66E-04 | 0.1392 | 428 | 0.777 |
| ENSP00000263390 | 3.16E-05 | 0.1381 | 242 | 0.000 |
| ENSP00000359446 | 3.37E-05 | 0.1380 | 499 | 0.323 |
| ENSP00000420321 | 4.68E-05 | 0.1378 | 306 | 0.690 |
| ENSP00000386741 | 4.13E-05 | 0.1372 | 239 | 0.560 |
| ENSP00000371514 | 3.22E-05 | 0.1369 | 212 | 0.252 |
| ENSP00000369460 | 7.42E-05 | 0.1369 | 301 | 0.888 |
| ENSP00000382356 | 1.70E-05 | 0.1368 | 587 | 0.235 |
| ENSP00000383894 | 5.21E-05 | 0.1367 | 736 | 0.457 |
| ENSP00000358730 | 4.10E-05 | 0.1365 | 439 | 0.000 |
| ENSP00000342681 | 4.16E-05 | 0.1362 | 302 | 0.889 |
| ENSP00000351727 | 4.31E-05 | 0.1361 | 360 | 0.123 |
| ENSP00000367828 | 7.16E-05 | 0.1354 | 384 | 0.537 |
| ENSP00000262946 | 3.15E-05 | 0.1352 | 165 | 0.156 |
| ENSP00000362924 | 1.19E-04 | 0.1347 | 752 | 0.910 |
| ENSP00000420405 | 5.64E-05 | 0.1343 | 165 | 0.110 |
| ENSP00000368190 | 3.92E-05 | 0.1337 | 457 | 0.877 |
| ENSP00000262915 | 3.10E-05 | 0.1335 | 900 | 0.133 |
| ENSP00000361141 | 3.16E-05 | 0.1334 | 357 | 0.644 |
| ENSP00000295897 | 8.17E-05 | 0.1331 | 980 | 0.956 |
| ENSP00000252744 | 1.72E-04 | 0.1331 | 374 | 0.111 |
| ENSP00000356162 | 2.90E-05 | 0.1326 | 939 | 0.660 |
| ENSP00000442563 | 4.55E-05 | 0.1311 | 170 | 0.722 |
| ENSP00000399985 | 5.94E-05 | 0.1304 | 359 | 0.837 |
| ENSP00000355195 | 4.67E-05 | 0.1303 | 307 | 0.660 |
| ENSP00000239144 | 2.66E-05 | 0.1303 | 176 | 0.857 |
| ENSP00000376865 | 3.49E-05 | 0.1301 | 858 | 0.661 |
| ENSP00000381034 | 4.22E-05 | 0.1296 | 158 | 0.530 |
| ENSP00000373827 | 2.42E-05 | 0.1296 | 150 | 0.118 |
| ENSP00000347409 | 3.17E-05 | 0.1288 | 312 | 0.311 |
| ENSP00000359958 | 9.35E-05 | 0.1273 | 216 | 0.432 |
| ENSP00000358327 | 4.52E-05 | 0.1271 | 968 | 0.863 |
| ENSP00000433773 | 6.92E-05 | 0.1270 | 191 | 0.000 |
| ENSP00000261654 | 4.15E-05 | 0.1268 | 191 | 0.000 |
| ENSP00000313681 | 5.76E-04 | 0.1266 | 842 | 0.722 |
| ENSP00000294312 | 2.59E-05 | 0.1257 | 210 | 0.916 |
| ENSP00000436682 | 8.06E-05 | 0.1253 | 564 | 0.500 |

| | | | | |
|---|---|---|---|---|
| ENSP00000227266 | 9.98E-05 | 0.1251 | 457 | 0.803 |
| ENSP00000368798 | 9.28E-05 | 0.1248 | 624 | 0.535 |
| ENSP00000296695 | 2.73E-05 | 0.1247 | 390 | 0.538 |
| ENSP00000356940 | 3.54E-05 | 0.1247 | 298 | 0.234 |
| ENSP00000347197 | 3.49E-05 | 0.1247 | 167 | 0.604 |
| ENSP00000266579 | 9.10E-05 | 0.1243 | 356 | 0.154 |
| ENSP00000293860 | 2.68E-05 | 0.1242 | 196 | 0.807 |
| ENSP00000392762 | 3.13E-05 | 0.1242 | 218 | 0.899 |
| ENSP00000302630 | 2.53E-05 | 0.1237 | 196 | 0.863 |
| ENSP00000276571 | 3.71E-05 | 0.1231 | 233 | 0.814 |
| ENSP00000396586 | 2.85E-05 | 0.1227 | 160 | 0.052 |
| ENSP00000377252 | 6.12E-05 | 0.1223 | 156 | 0.000 |
| ENSP00000254301 | 7.74E-05 | 0.1222 | 896 | 0.944 |
| ENSP00000349078 | 6.96E-05 | 0.1222 | 242 | 0.472 |
| ENSP00000365775 | 5.70E-05 | 0.1218 | 720 | 0.781 |
| ENSP00000419362 | 6.32E-05 | 0.1213 | 0 | 0.793 |
| ENSP00000344468 | 4.74E-05 | 0.1209 | 918 | 0.662 |
| ENSP00000365280 | 4.28E-05 | 0.1208 | 239 | 0.904 |
| ENSP00000393312 | 7.73E-05 | 0.1199 | 424 | 0.000 |
| ENSP00000233242 | 6.05E-05 | 0.1194 | 997 | 0.877 |
| ENSP00000367034 | 5.54E-05 | 0.1193 | 877 | 0.947 |
| ENSP00000407685 | 1.36E-05 | 0.1192 | 0 | 0.245 |
| ENSP00000367714 | 3.71E-05 | 0.1192 | 256 | 0.834 |
| ENSP00000311697 | 4.99E-05 | 0.1189 | 222 | 0.928 |
| ENSP00000262738 | 3.85E-05 | 0.1189 | 421 | 0.359 |
| ENSP00000308549 | 3.48E-05 | 0.1187 | 412 | 0.702 |
| ENSP00000435466 | 4.78E-05 | 0.1186 | 382 | 0.238 |
| ENSP00000264613 | 1.05E-04 | 0.1185 | 508 | 0.656 |
| ENSP00000219746 | 4.57E-05 | 0.1177 | 269 | 0.897 |
| ENSP00000299752 | 5.57E-05 | 0.1174 | 187 | 0.182 |
| ENSP00000327569 | 2.72E-05 | 0.1171 | 160 | 0.055 |
| ENSP00000364683 | 4.18E-05 | 0.1169 | 215 | 0.759 |
| ENSP00000309913 | 2.47E-05 | 0.1162 | 301 | 0.875 |
| ENSP00000341108 | 4.32E-05 | 0.1159 | 241 | 0.835 |
| ENSP00000215909 | 1.55E-04 | 0.1152 | 734 | 0.881 |
| ENSP00000353508 | 6.00E-05 | 0.1139 | 717 | 0.825 |
| ENSP00000172229 | 7.48E-05 | 0.1139 | 323 | 0.963 |
| ENSP00000315602 | 3.88E-05 | 0.1137 | 605 | 0.478 |
| ENSP00000011653 | 2.48E-05 | 0.1135 | 564 | 0.950 |
| ENSP00000424838 | 1.04E-04 | 0.1130 | 565 | 0.692 |
| ENSP00000367766 | 8.25E-05 | 0.1125 | 648 | 0.389 |
| ENSP00000307443 | 2.89E-05 | 0.1123 | 160 | 0.055 |
| ENSP00000224337 | 3.63E-05 | 0.1121 | 158 | 0.710 |
| ENSP00000357380 | 4.92E-05 | 0.1117 | 284 | 0.000 |
| ENSP00000265077 | 9.35E-05 | 0.1115 | 795 | 0.942 |
| ENSP00000367198 | 4.13E-05 | 0.1113 | 300 | 0.528 |

| | | | | |
|---|---|---|---|---|
| ENSP00000301891 | 2.56E-05 | 0.1104 | 160 | 0.054 |
| ENSP00000365931 | 7.51E-05 | 0.1102 | 151 | 0.746 |
| ENSP00000231461 | 2.87E-05 | 0.1101 | 441 | 0.107 |
| ENSP00000456533 | 3.88E-05 | 0.1099 | 198 | 0.000 |
| ENSP00000336762 | 3.91E-05 | 0.1096 | 196 | 0.615 |
| ENSP00000352455 | 7.68E-05 | 0.1092 | 922 | 0.353 |
| ENSP00000362888 | 3.88E-05 | 0.1086 | 184 | 0.301 |
| ENSP00000244527 | 3.93E-05 | 0.1086 | 341 | 0.086 |
| ENSP00000180173 | 4.87E-05 | 0.1083 | 414 | 0.096 |
| ENSP00000236671 | 7.41E-05 | 0.1078 | 701 | 0.876 |
| ENSP00000248572 | 2.07E-05 | 0.1077 | 974 | 0.000 |
| ENSP00000346316 | 5.08E-05 | 0.1076 | 884 | 0.000 |
| ENSP00000261267 | 7.09E-05 | 0.1075 | 715 | 0.858 |
| ENSP00000249373 | 3.00E-05 | 0.1074 | 171 | 0.923 |
| ENSP00000257555 | 2.84E-05 | 0.1066 | 575 | 0.822 |
| ENSP00000234590 | 3.43E-05 | 0.1065 | 834 | 0.858 |
| ENSP00000456548 | 3.58E-05 | 0.1063 | 181 | 0.156 |
| ENSP00000217026 | 1.85E-05 | 0.1059 | 284 | 0.000 |
| ENSP00000364694 | 7.27E-05 | 0.1057 | 623 | 0.849 |
| ENSP00000311837 | 8.37E-05 | 0.1054 | 268 | 0.111 |
| ENSP00000215885 | 2.93E-05 | 0.1053 | 181 | 0.162 |
| ENSP00000371833 | 3.81E-05 | 0.1051 | 154 | 0.000 |
| ENSP00000351314 | 6.12E-05 | 0.1051 | 330 | 0.585 |
| ENSP00000380193 | 8.37E-05 | 0.1051 | 942 | 0.720 |
| ENSP00000347041 | 6.71E-05 | 0.1047 | 916 | 0.909 |
| ENSP00000340191 | 2.87E-05 | 0.1047 | 301 | 0.637 |
| ENSP00000375777 | 5.02E-05 | 0.1046 | 163 | 0.235 |
| ENSP00000366898 | 8.89E-05 | 0.1044 | 440 | 0.769 |
| ENSP00000357113 | 1.26E-04 | 0.1043 | 173 | 0.514 |
| ENSP00000262958 | 5.51E-05 | 0.1042 | 607 | 0.854 |
| ENSP00000251808 | 2.98E-05 | 0.1041 | 266 | 0.781 |
| ENSP00000468772 | 5.26E-05 | 0.1040 | 224 | 0.000 |
| ENSP00000263577 | 2.79E-05 | 0.1039 | 188 | 0.621 |
| ENSP00000231004 | 1.24E-04 | 0.1038 | 696 | 0.872 |
| ENSP00000359991 | 5.36E-05 | 0.1038 | 412 | 0.605 |
| ENSP00000291527 | 4.97E-05 | 0.1037 | 206 | 0.758 |
| ENSP00000351682 | 5.22E-05 | 0.1036 | 837 | 0.277 |
| ENSP00000236826 | 1.23E-04 | 0.1029 | 930 | 0.953 |
| ENSP00000271324 | 6.66E-05 | 0.1029 | 239 | 0.559 |
| ENSP00000254351 | 9.92E-05 | 0.1028 | 951 | 0.925 |
| ENSP00000270202 | 5.46E-05 | 0.1026 | 969 | 0.000 |
| ENSP00000357429 | 3.44E-05 | 0.1025 | 359 | 0.825 |
| ENSP00000364252 | 4.74E-05 | 0.1018 | 589 | 0.729 |
| ENSP00000371798 | 5.07E-05 | 0.1017 | 280 | 0.676 |
| ENSP00000450687 | 6.74E-05 | 0.1013 | 357 | 0.760 |
| ENSP00000240874 | 2.95E-05 | 0.1012 | 272 | 0.741 |

| | | | | |
|---|---|---|---|---|
| ENSP00000160740 | 1.54E-04 | 0.1011 | 216 | 0.617 |
| ENSP00000338345 | 8.66E-05 | 0.1008 | 645 | 0.946 |
| ENSP00000357980 | 7.75E-05 | 0.1003 | 431 | 0.623 |
| ENSP00000215754 | 1.57E-04 | 0.1001 | 899 | 0.917 |
| ENSP00000466140 | 1.13E-05 | 0.0998 | 0 | 0.369 |
| ENSP00000344192 | 3.77E-05 | 0.0995 | 897 | 0.925 |
| ENSP00000325527 | 1.05E-04 | 0.0993 | 898 | 0.862 |
| ENSP00000362993 | 8.66E-05 | 0.0989 | 393 | 0.452 |
| ENSP00000356906 | 3.92E-05 | 0.0989 | 250 | 0.274 |
| ENSP00000360777 | 3.45E-05 | 0.0976 | 160 | 0.262 |
| ENSP00000344648 | 4.39E-05 | 0.0974 | 210 | 0.098 |
| ENSP00000261584 | 3.78E-05 | 0.0974 | 195 | 0.554 |
| ENSP00000271628 | 3.39E-05 | 0.0972 | 378 | 0.691 |
| ENSP00000319377 | 2.54E-05 | 0.0970 | 345 | 0.494 |
| ENSP00000375921 | 4.55E-05 | 0.0967 | 305 | 0.872 |
| ENSP00000261023 | 1.36E-04 | 0.0961 | 997 | 0.904 |
| ENSP00000300181 | 4.62E-05 | 0.0958 | 392 | 0.458 |
| ENSP00000374309 | 4.07E-05 | 0.0955 | 258 | 0.673 |
| ENSP00000348429 | 3.55E-05 | 0.0955 | 344 | 0.538 |
| ENSP00000321853 | 1.53E-04 | 0.0953 | 954 | 0.636 |
| ENSP00000355325 | 4.07E-05 | 0.0947 | 644 | 0.467 |
| ENSP00000297268 | 7.96E-05 | 0.0945 | 976 | 0.960 |
| ENSP00000382178 | 5.06E-05 | 0.0945 | 241 | 0.094 |
| ENSP00000344223 | 8.66E-05 | 0.0942 | 835 | 0.771 |
| ENSP00000324277 | 4.48E-05 | 0.0941 | 191 | 0.263 |
| ENSP00000336927 | 1.14E-04 | 0.0934 | 912 | 0.432 |
| ENSP00000435835 | 4.17E-05 | 0.0933 | 233 | 0.828 |
| ENSP00000200557 | 6.23E-05 | 0.0932 | 150 | 0.231 |
| ENSP00000351342 | 3.89E-05 | 0.0930 | 199 | 0.694 |
| ENSP00000365131 | 4.60E-05 | 0.0924 | 686 | 0.228 |
| ENSP00000332771 | 7.32E-05 | 0.0923 | 216 | 0.124 |
| ENSP00000233948 | 2.68E-05 | 0.0921 | 602 | 0.891 |
| ENSP00000356969 | 3.44E-05 | 0.0917 | 991 | 0.538 |
| ENSP00000358162 | 5.21E-05 | 0.0916 | 889 | 0.000 |
| ENSP00000333363 | 8.83E-05 | 0.0914 | 593 | 0.882 |
| ENSP00000351113 | 6.79E-05 | 0.0913 | 483 | 0.142 |
| ENSP00000221972 | 3.58E-05 | 0.0912 | 809 | 0.937 |
| ENSP00000373477 | 6.13E-05 | 0.0907 | 340 | 0.416 |
| ENSP00000440403 | 4.64E-05 | 0.0906 | 187 | 0.693 |
| ENSP00000294304 | 1.20E-04 | 0.0906 | 876 | 0.944 |
| ENSP00000264245 | 3.19E-05 | 0.0900 | 200 | 0.471 |
| ENSP00000366525 | 2.02E-04 | 0.0900 | 440 | 0.411 |
| ENSP00000368698 | 6.64E-05 | 0.0899 | 373 | 0.771 |
| ENSP00000452746 | 5.61E-05 | 0.0888 | 481 | 0.766 |
| ENSP00000348089 | 4.87E-05 | 0.0888 | 284 | 0.881 |
| ENSP00000368349 | 6.54E-05 | 0.0885 | 509 | 0.795 |

| | | | | |
|---|---|---|---|---|
| ENSP00000422554 | 3.51E-05 | 0.0881 | 263 | 0.237 |
| ENSP00000348307 | 4.51E-05 | 0.0880 | 919 | 0.595 |
| ENSP00000353624 | 4.99E-05 | 0.0870 | 884 | 0.000 |
| ENSP00000261799 | 6.60E-05 | 0.0870 | 897 | 0.000 |
| ENSP00000415222 | 1.95E-05 | 0.0867 | 471 | 0.000 |
| ENSP00000406012 | 6.07E-05 | 0.0861 | 0 | 0.072 |
| ENSP00000434364 | 5.28E-05 | 0.0857 | 216 | 0.150 |
| ENSP00000296026 | 2.77E-05 | 0.0856 | 163 | 0.638 |
| ENSP00000262464 | 2.24E-04 | 0.0854 | 614 | 0.620 |
| ENSP00000377914 | 4.79E-05 | 0.0853 | 153 | 0.171 |
| ENSP00000334267 | 2.61E-05 | 0.0852 | 343 | 0.411 |
| ENSP00000358549 | 3.64E-05 | 0.0851 | 363 | 0.609 |
| ENSP00000009530 | 1.37E-04 | 0.0847 | 744 | 0.776 |
| ENSP00000296585 | 7.08E-05 | 0.0847 | 948 | 0.949 |
| ENSP00000455329 | 2.89E-05 | 0.0845 | 518 | 0.000 |
| ENSP00000285379 | 9.40E-05 | 0.0843 | 416 | 0.931 |
| ENSP00000367202 | 3.41E-05 | 0.0843 | 968 | 0.757 |
| ENSP00000243077 | 1.17E-04 | 0.0841 | 998 | 0.876 |
| ENSP00000361834 | 3.92E-05 | 0.0837 | 926 | 0.443 |
| ENSP00000331411 | 4.43E-05 | 0.0835 | 203 | 0.092 |
| ENSP00000279178 | 2.55E-05 | 0.0827 | 160 | 0.053 |
| ENSP00000446100 | 4.81E-05 | 0.0826 | 150 | 0.142 |
| ENSP00000353010 | 4.77E-05 | 0.0822 | 183 | 0.060 |
| ENSP00000305603 | 3.00E-05 | 0.0819 | 418 | 0.371 |
| ENSP00000346173 | 3.89E-05 | 0.0818 | 300 | 0.259 |
| ENSP00000360124 | 4.25E-05 | 0.0817 | 288 | 0.508 |
| ENSP00000446714 | 3.47E-05 | 0.0806 | 278 | 0.466 |
| ENSP00000308620 | 9.63E-05 | 0.0805 | 297 | 0.911 |
| ENSP00000315167 | 4.26E-05 | 0.0800 | 167 | 0.191 |
| ENSP00000352295 | 7.17E-05 | 0.0798 | 0 | 0.660 |
| ENSP00000428417 | 3.29E-05 | 0.0793 | 196 | 0.352 |
| ENSP00000352614 | 1.15E-04 | 0.0789 | 422 | 0.095 |
| ENSP00000321326 | 8.52E-05 | 0.0789 | 704 | 0.847 |
| ENSP00000368401 | 1.49E-04 | 0.0788 | 838 | 0.893 |
| ENSP00000342011 | 3.13E-04 | 0.0787 | 0 | 0.664 |
| ENSP00000268459 | 3.56E-05 | 0.0785 | 398 | 0.868 |
| ENSP00000407262 | 4.69E-05 | 0.0783 | 173 | 0.691 |
| ENSP00000205948 | 2.26E-04 | 0.0781 | 581 | 0.575 |
| ENSP00000388658 | 7.07E-05 | 0.0774 | 201 | 0.059 |
| ENSP00000323155 | 3.24E-05 | 0.0764 | 414 | 0.545 |
| ENSP00000358414 | 3.88E-05 | 0.0764 | 594 | 0.575 |
| ENSP00000367595 | 1.46E-05 | 0.0761 | 183 | 0.206 |
| ENSP00000366581 | 5.30E-05 | 0.0761 | 884 | 0.946 |
| ENSP00000365116 | 4.38E-05 | 0.0760 | 300 | 0.204 |
| ENSP00000355645 | 5.42E-05 | 0.0753 | 876 | 0.702 |
| ENSP00000415941 | 5.13E-05 | 0.0750 | 459 | 0.708 |

| | | | | |
|---|---|---|---|---|
| ENSP00000257497 | 7.41E-05 | 0.0749 | 366 | 0.786 |
| ENSP00000386951 | 3.34E-05 | 0.0749 | 242 | 0.831 |
| ENSP00000368664 | 3.82E-05 | 0.0748 | 351 | 0.816 |
| ENSP00000362817 | 3.55E-05 | 0.0747 | 345 | 0.648 |
| ENSP00000370880 | 3.40E-05 | 0.0746 | 342 | 0.697 |
| ENSP00000332258 | 3.94E-05 | 0.0745 | 512 | 0.566 |
| ENSP00000266041 | 1.53E-04 | 0.0745 | 478 | 0.908 |
| ENSP00000262424 | 3.52E-05 | 0.0741 | 156 | 0.105 |
| ENSP00000226284 | 1.23E-04 | 0.0740 | 836 | 0.886 |
| ENSP00000384400 | 3.84E-05 | 0.0734 | 292 | 0.137 |
| ENSP00000239165 | 2.29E-05 | 0.0734 | 198 | 0.855 |
| ENSP00000365759 | 3.36E-05 | 0.0732 | 609 | 0.537 |
| ENSP00000262178 | 3.42E-05 | 0.0732 | 346 | 0.567 |
| ENSP00000052754 | 3.83E-05 | 0.0731 | 927 | 0.943 |
| ENSP00000348258 | 4.66E-05 | 0.0730 | 885 | 0.000 |
| ENSP00000272371 | 2.81E-05 | 0.0726 | 340 | 0.284 |
| ENSP00000300098 | 3.14E-05 | 0.0723 | 408 | 0.424 |
| ENSP00000361548 | 2.83E-05 | 0.0722 | 414 | 0.865 |
| ENSP00000365899 | 1.29E-04 | 0.0721 | 313 | 0.383 |
| ENSP00000264832 | 9.21E-05 | 0.0719 | 998 | 0.978 |
| ENSP00000356811 | 3.89E-05 | 0.0717 | 170 | 0.807 |
| ENSP00000363840 | 2.97E-05 | 0.0717 | 568 | 0.795 |
| ENSP00000307156 | 1.12E-04 | 0.0714 | 533 | 0.926 |
| ENSP00000257290 | 3.97E-05 | 0.0709 | 264 | 0.701 |
| ENSP00000343246 | 3.87E-05 | 0.0708 | 467 | 0.867 |
| ENSP00000447378 | 1.04E-04 | 0.0708 | 400 | 0.723 |
| ENSP00000347168 | 4.73E-05 | 0.0701 | 884 | 0.946 |
| ENSP00000309845 | 7.50E-05 | 0.0700 | 923 | 0.000 |
| ENSP00000350159 | 4.68E-05 | 0.0700 | 884 | 0.000 |
| ENSP00000309629 | 7.97E-05 | 0.0695 | 518 | 0.635 |
| ENSP00000267843 | 5.54E-05 | 0.0692 | 906 | 0.975 |
| ENSP00000256078 | 4.37E-05 | 0.0690 | 907 | 0.749 |
| ENSP00000321106 | 6.85E-05 | 0.0690 | 600 | 0.803 |
| ENSP00000302150 | 3.91E-05 | 0.0686 | 225 | 0.903 |
| ENSP00000370007 | 4.97E-05 | 0.0680 | 912 | 0.303 |
| ENSP00000381726 | 4.21E-05 | 0.0680 | 359 | 0.694 |
| ENSP00000316329 | 8.79E-05 | 0.0678 | 297 | 0.491 |
| ENSP00000435150 | 1.22E-04 | 0.0678 | 625 | 0.175 |
| ENSP00000354280 | 4.14E-05 | 0.0676 | 387 | 0.428 |
| ENSP00000361562 | 6.69E-05 | 0.0674 | 361 | 0.337 |
| ENSP00000377778 | 4.78E-05 | 0.0673 | 150 | 0.096 |
| ENSP00000368752 | 1.12E-04 | 0.0673 | 983 | 0.824 |
| ENSP00000374280 | 5.71E-05 | 0.0670 | 358 | 0.881 |
| ENSP00000276373 | 9.52E-05 | 0.0669 | 328 | 0.205 |
| ENSP00000378702 | 4.02E-05 | 0.0666 | 246 | 0.930 |
| ENSP00000366015 | 3.29E-05 | 0.0665 | 216 | 0.167 |

| | | | | |
|---|---|---|---|---|
| ENSP00000441875 | 8.03E-05 | 0.0665 | 581 | 0.697 |
| ENSP00000389792 | 3.47E-05 | 0.0665 | 224 | 0.483 |
| ENSP00000358727 | 5.57E-05 | 0.0662 | 284 | 0.459 |
| ENSP00000310565 | 3.58E-05 | 0.0660 | 254 | 0.103 |
| ENSP00000358153 | 4.92E-05 | 0.0659 | 884 | 0.000 |
| ENSP00000282326 | 8.26E-05 | 0.0658 | 661 | 0.796 |
| ENSP00000283635 | 6.59E-05 | 0.0656 | 633 | 0.000 |
| ENSP00000321334 | 1.29E-04 | 0.0652 | 905 | 0.571 |
| ENSP00000322775 | 6.40E-05 | 0.0651 | 270 | 0.838 |
| ENSP00000255380 | 2.22E-05 | 0.0650 | 185 | 0.593 |
| ENSP00000282397 | 6.85E-05 | 0.0643 | 926 | 0.745 |
| ENSP00000328928 | 4.56E-05 | 0.0643 | 335 | 0.871 |
| ENSP00000351686 | 1.27E-04 | 0.0642 | 704 | 0.000 |
| ENSP00000252898 | 5.63E-05 | 0.0641 | 900 | 0.295 |
| ENSP00000301420 | 6.23E-05 | 0.0640 | 584 | 0.280 |
| ENSP00000216492 | 1.35E-04 | 0.0624 | 416 | 0.848 |
| ENSP00000181383 | 1.97E-04 | 0.0624 | 531 | 0.599 |
| ENSP00000230732 | 2.58E-05 | 0.0622 | 159 | 0.783 |
| ENSP00000446916 | 3.07E-05 | 0.0610 | 216 | 0.312 |
| ENSP00000310978 | 3.01E-05 | 0.0610 | 183 | 0.000 |
| ENSP00000219833 | 2.75E-05 | 0.0610 | 235 | 0.347 |
| ENSP00000315477 | 4.80E-05 | 0.0609 | 916 | 0.782 |
| ENSP00000356581 | 4.92E-05 | 0.0609 | 234 | 0.000 |
| ENSP00000393840 | 2.06E-05 | 0.0608 | 242 | 0.134 |
| ENSP00000300060 | 5.46E-05 | 0.0608 | 980 | 0.793 |
| ENSP00000365963 | 4.87E-05 | 0.0607 | 236 | 0.177 |
| ENSP00000383256 | 2.09E-05 | 0.0604 | 158 | 0.471 |
| ENSP00000411552 | 1.03E-04 | 0.0603 | 222 | 0.906 |
| ENSP00000313740 | 2.68E-05 | 0.0602 | 212 | 0.000 |
| ENSP00000352208 | 8.16E-05 | 0.0601 | 199 | 0.153 |
| ENSP00000369677 | 6.04E-05 | 0.0600 | 290 | 0.451 |
| ENSP00000386184 | 4.49E-05 | 0.0597 | 206 | 0.000 |
| ENSP00000402584 | 4.72E-05 | 0.0588 | 153 | 0.079 |
| ENSP00000357708 | 6.31E-05 | 0.0587 | 441 | 0.490 |
| ENSP00000426359 | 1.75E-05 | 0.0586 | 156 | 0.157 |
| ENSP00000245907 | 7.00E-05 | 0.0579 | 472 | 0.717 |
| ENSP00000261707 | 2.68E-05 | 0.0577 | 473 | 0.698 |
| ENSP00000340019 | 9.17E-05 | 0.0577 | 978 | 0.810 |
| ENSP00000362795 | 3.72E-05 | 0.0577 | 504 | 0.871 |
| ENSP00000468236 | 2.65E-05 | 0.0572 | 555 | 0.329 |
| ENSP00000386104 | 3.65E-05 | 0.0565 | 345 | 0.585 |
| ENSP00000020945 | 5.16E-05 | 0.0565 | 912 | 0.926 |
| ENSP00000355601 | 4.41E-05 | 0.0564 | 270 | 0.768 |
| ENSP00000295228 | 2.41E-05 | 0.0563 | 205 | 0.887 |
| ENSP00000386170 | 2.88E-05 | 0.0557 | 227 | 0.850 |
| ENSP00000297512 | 2.79E-05 | 0.0556 | 615 | 0.260 |

| | | | | |
|---|---|---|---|---|
| ENSP00000318944 | 6.45E-05 | 0.0556 | 173 | 0.690 |
| ENSP00000342026 | 7.72E-05 | 0.0552 | 512 | 0.620 |
| ENSP00000464149 | 6.25E-05 | 0.0551 | 700 | 0.000 |
| ENSP00000361120 | 4.49E-05 | 0.0548 | 626 | 0.828 |
| ENSP00000396163 | 2.74E-05 | 0.0546 | 418 | 0.508 |
| ENSP00000211998 | 5.36E-05 | 0.0546 | 695 | 0.892 |
| ENSP00000369654 | 6.00E-05 | 0.0545 | 250 | 0.747 |
| ENSP00000262865 | 3.56E-05 | 0.0542 | 198 | 0.602 |
| ENSP00000357731 | 3.17E-05 | 0.0539 | 214 | 0.724 |
| ENSP00000370521 | 4.23E-05 | 0.0533 | 540 | 0.522 |
| ENSP00000385721 | 1.09E-04 | 0.0530 | 720 | 0.902 |
| ENSP00000387946 | 4.33E-05 | 0.0529 | 581 | 0.724 |
| ENSP00000358878 | 5.03E-05 | 0.0527 | 151 | 0.185 |
| ENSP00000360170 | 4.77E-05 | 0.0524 | 470 | 0.421 |
| ENSP00000380129 | 3.81E-05 | 0.0524 | 496 | 0.236 |
| ENSP00000371040 | 1.85E-05 | 0.0524 | 213 | 0.126 |
| ENSP00000367343 | 3.76E-05 | 0.0523 | 233 | 0.294 |
| ENSP00000357066 | 3.62E-05 | 0.0523 | 360 | 0.678 |
| ENSP00000297785 | 5.50E-05 | 0.0522 | 416 | 0.583 |
| ENSP00000312624 | 2.88E-05 | 0.0521 | 903 | 0.441 |
| ENSP00000377527 | 4.76E-05 | 0.0519 | 216 | 0.498 |
| ENSP00000413575 | 4.53E-05 | 0.0516 | 218 | 0.506 |
| ENSP00000300289 | 4.84E-05 | 0.0514 | 955 | 0.674 |
| ENSP00000350767 | 4.49E-05 | 0.0512 | 884 | 0.950 |
| ENSP00000299882 | 3.78E-05 | 0.0506 | 459 | 0.107 |
| ENSP00000367992 | 5.20E-05 | 0.0505 | 429 | 0.553 |
| ENSP00000347890 | 8.71E-05 | 0.0499 | 372 | 0.822 |
| ENSP00000298125 | 7.20E-05 | 0.0494 | 507 | 0.956 |
| ENSP00000217305 | 3.94E-05 | 0.0489 | 509 | 0.753 |
| ENSP00000007722 | 1.46E-04 | 0.0476 | 940 | 0.745 |
| ENSP00000272252 | 2.76E-05 | 0.0474 | 211 | 0.158 |
| ENSP00000339155 | 3.89E-05 | 0.0474 | 175 | 0.094 |
| ENSP00000325708 | 5.70E-05 | 0.0473 | 202 | 0.542 |
| ENSP00000242248 | 2.41E-05 | 0.0473 | 161 | 0.469 |
| ENSP00000365858 | 5.18E-05 | 0.0472 | 844 | 0.000 |
| ENSP00000347847 | 7.02E-05 | 0.0470 | 789 | 0.830 |
| ENSP00000471024 | 2.77E-05 | 0.0465 | 201 | 0.000 |
| ENSP00000322087 | 5.19E-05 | 0.0459 | 313 | 0.764 |
| ENSP00000316605 | 4.28E-05 | 0.0450 | 566 | 0.463 |
| ENSP00000384264 | 3.85E-05 | 0.0446 | 581 | 0.450 |
| ENSP00000257857 | 1.01E-04 | 0.0442 | 939 | 0.701 |
| ENSP00000262340 | 1.40E-04 | 0.0441 | 918 | 0.575 |
| ENSP00000355689 | 5.84E-05 | 0.0438 | 374 | 0.539 |
| ENSP00000380227 | 3.89E-05 | 0.0437 | 969 | 0.822 |
| ENSP00000465194 | 2.49E-05 | 0.0436 | 252 | 0.258 |
| ENSP00000228280 | 6.87E-05 | 0.0434 | 585 | 0.947 |

| | | | | |
|---|---|---|---|---|
| ENSP00000379258 | 9.84E-05 | 0.0430 | 282 | 0.852 |
| ENSP00000343819 | 6.80E-05 | 0.0430 | 528 | 0.862 |
| ENSP00000278590 | 5.40E-05 | 0.0429 | 487 | 0.165 |
| ENSP00000370842 | 4.22E-05 | 0.0428 | 566 | 0.558 |
| ENSP00000290363 | 2.86E-04 | 0.0426 | 183 | 0.000 |
| ENSP00000310928 | 8.87E-05 | 0.0424 | 392 | 0.867 |
| ENSP00000377502 | 3.70E-05 | 0.0422 | 171 | 0.090 |
| ENSP00000338548 | 6.65E-05 | 0.0422 | 855 | 0.972 |
| ENSP00000283147 | 7.09E-05 | 0.0420 | 852 | 0.940 |
| ENSP00000252321 | 3.49E-05 | 0.0420 | 459 | 0.407 |
| ENSP00000411217 | 2.03E-05 | 0.0419 | 193 | 0.000 |
| ENSP00000269143 | 7.32E-05 | 0.0416 | 373 | 0.586 |
| ENSP00000452120 | 2.86E-05 | 0.0416 | 847 | 0.672 |
| ENSP00000419692 | 1.15E-04 | 0.0416 | 486 | 0.851 |
| ENSP00000364475 | 3.58E-05 | 0.0415 | 198 | 0.670 |
| ENSP00000277541 | 4.87E-05 | 0.0414 | 940 | 0.000 |
| ENSP00000307508 | 3.76E-05 | 0.0411 | 572 | 0.663 |
| ENSP00000379330 | 3.68E-05 | 0.0411 | 201 | 0.905 |
| ENSP00000303242 | 7.31E-05 | 0.0410 | 968 | 0.910 |
| ENSP00000265165 | 4.42E-05 | 0.0408 | 937 | 0.884 |
| ENSP00000381657 | 6.09E-05 | 0.0406 | 808 | 0.893 |
| ENSP00000320924 | 4.19E-05 | 0.0406 | 191 | 0.501 |
| ENSP00000420514 | 4.01E-05 | 0.0402 | 542 | 0.806 |
| ENSP00000029410 | 3.94E-05 | 0.0400 | 200 | 0.173 |
| ENSP00000300527 | 1.82E-04 | 0.0399 | 694 | 0.557 |
| ENSP00000284984 | 1.27E-04 | 0.0399 | 533 | 0.869 |
| ENSP00000242576 | 7.24E-05 | 0.0398 | 269 | 0.727 |
| ENSP00000368952 | 7.71E-05 | 0.0393 | 216 | 0.233 |
| ENSP00000216629 | 2.38E-05 | 0.0393 | 244 | 0.556 |
| ENSP00000359635 | 1.08E-04 | 0.0391 | 613 | 0.000 |
| ENSP00000435300 | 4.52E-05 | 0.0387 | 154 | 0.089 |
| ENSP00000361306 | 3.64E-05 | 0.0386 | 177 | 0.096 |
| ENSP00000356346 | 9.18E-05 | 0.0386 | 705 | 0.000 |
| ENSP00000354040 | 3.85E-05 | 0.0385 | 633 | 0.801 |
| ENSP00000262551 | 4.55E-05 | 0.0382 | 929 | 0.891 |
| ENSP00000269097 | 4.77E-05 | 0.0378 | 159 | 0.081 |
| ENSP00000260649 | 2.04E-05 | 0.0376 | 358 | 0.225 |
| ENSP00000264009 | 2.13E-05 | 0.0376 | 734 | 0.000 |
| ENSP00000278765 | 2.20E-05 | 0.0376 | 165 | 0.309 |
| ENSP00000469315 | 3.51E-05 | 0.0376 | 562 | 0.159 |
| ENSP00000363804 | 9.62E-05 | 0.0375 | 897 | 0.866 |
| ENSP00000363603 | 5.25E-05 | 0.0372 | 206 | 0.071 |
| ENSP00000377836 | 5.76E-05 | 0.0372 | 912 | 0.914 |
| ENSP00000300215 | 1.02E-04 | 0.0371 | 681 | 0.364 |
| ENSP00000350132 | 5.24E-05 | 0.0370 | 212 | 0.613 |
| ENSP00000262383 | 7.22E-05 | 0.0363 | 208 | 0.821 |

| | | | | |
|---|---|---|---|---|
| ENSP00000227667 | 1.08E-04 | 0.0350 | 997 | 0.743 |
| ENSP00000361803 | 9.46E-05 | 0.0350 | 168 | 0.163 |
| ENSP00000372169 | 3.49E-05 | 0.0349 | 218 | 0.260 |
| ENSP00000357905 | 7.43E-05 | 0.0341 | 396 | 0.562 |
| ENSP00000268296 | 4.83E-05 | 0.0341 | 900 | 0.899 |
| ENSP00000293379 | 1.05E-04 | 0.0340 | 998 | 0.852 |
| ENSP00000218758 | 9.15E-05 | 0.0340 | 586 | 0.885 |
| ENSP00000415900 | 2.30E-05 | 0.0339 | 242 | 0.531 |
| ENSP00000346063 | 4.34E-05 | 0.0332 | 254 | 0.904 |
| ENSP00000355060 | 6.24E-05 | 0.0331 | 0 | 0.166 |
| ENSP00000369081 | 3.63E-05 | 0.0329 | 518 | 0.532 |
| ENSP00000387088 | 1.29E-04 | 0.0329 | 193 | 0.000 |
| ENSP00000343925 | 6.91E-05 | 0.0328 | 396 | 0.904 |
| ENSP00000014935 | 3.86E-04 | 0.0326 | 159 | 0.168 |
| ENSP00000226253 | 8.86E-05 | 0.0325 | 202 | 0.271 |
| ENSP00000371835 | 8.72E-05 | 0.0323 | 0 | 0.137 |
| ENSP00000365431 | 1.16E-04 | 0.0323 | 165 | 0.207 |
| ENSP00000368226 | 3.65E-05 | 0.0322 | 314 | 0.555 |
| ENSP00000351755 | 3.20E-05 | 0.0321 | 360 | 0.674 |
| ENSP00000283290 | 1.10E-04 | 0.0320 | 359 | 0.338 |
| ENSP00000418397 | 3.31E-05 | 0.0319 | 254 | 0.341 |
| ENSP00000307188 | 1.18E-04 | 0.0319 | 513 | 0.551 |
| ENSP00000377941 | 5.76E-05 | 0.0318 | 955 | 0.617 |
| ENSP00000356545 | 2.93E-05 | 0.0315 | 234 | 0.703 |
| ENSP00000348936 | 2.44E-05 | 0.0314 | 0 | 0.127 |
| ENSP00000353575 | 3.41E-05 | 0.0314 | 400 | 0.374 |
| ENSP00000284202 | 9.87E-05 | 0.0312 | 263 | 0.338 |
| ENSP00000302846 | 7.87E-05 | 0.0312 | 416 | 0.895 |
| ENSP00000430633 | 2.00E-05 | 0.0310 | 338 | 0.000 |
| ENSP00000363985 | 4.72E-05 | 0.0306 | 200 | 0.108 |
| ENSP00000282091 | 9.00E-05 | 0.0304 | 859 | 0.948 |
| ENSP00000263923 | 6.06E-05 | 0.0302 | 810 | 0.755 |
| ENSP00000363641 | 9.02E-05 | 0.0301 | 866 | 0.876 |
| ENSP00000321259 | 9.48E-05 | 0.0300 | 211 | 0.449 |
| ENSP00000309968 | 6.93E-05 | 0.0299 | 655 | 0.946 |
| ENSP00000369784 | 3.10E-05 | 0.0298 | 234 | 0.468 |
| ENSP00000288955 | 2.49E-05 | 0.0298 | 206 | 0.718 |
| ENSP00000349259 | 6.65E-05 | 0.0298 | 902 | 0.725 |
| ENSP00000292301 | 3.08E-05 | 0.0297 | 459 | 0.848 |
| ENSP00000387282 | 6.86E-05 | 0.0297 | 612 | 0.502 |
| ENSP00000263735 | 9.54E-05 | 0.0297 | 330 | 0.898 |
| ENSP00000420686 | 2.84E-05 | 0.0296 | 152 | 0.112 |
| ENSP00000291526 | 8.67E-05 | 0.0294 | 160 | 0.269 |
| ENSP00000431418 | 4.45E-05 | 0.0293 | 850 | 0.928 |
| ENSP00000393847 | 4.04E-05 | 0.0293 | 470 | 0.409 |
| ENSP00000356481 | 5.13E-05 | 0.0292 | 200 | 0.096 |

| | | | | |
|---|---|---|---|---|
| ENSP00000368079 | 4.54E-05 | 0.0290 | 275 | 0.308 |
| ENSP00000350314 | 4.19E-05 | 0.0288 | 248 | 0.323 |
| ENSP00000359022 | 4.34E-05 | 0.0288 | 264 | 0.494 |
| ENSP00000386378 | 3.02E-05 | 0.0287 | 271 | 0.708 |
| ENSP00000245323 | 7.08E-05 | 0.0284 | 357 | 0.933 |
| ENSP00000321856 | 4.25E-05 | 0.0282 | 457 | 0.381 |
| ENSP00000239243 | 6.07E-05 | 0.0280 | 403 | 0.882 |
| ENSP00000220676 | 2.68E-05 | 0.0278 | 420 | 0.600 |
| ENSP00000419446 | 5.12E-05 | 0.0276 | 448 | 0.742 |
| ENSP00000372170 | 6.61E-05 | 0.0275 | 345 | 0.884 |
| ENSP00000261980 | 9.40E-05 | 0.0274 | 632 | 0.834 |
| ENSP00000411822 | 2.60E-05 | 0.0273 | 458 | 0.598 |
| ENSP00000334072 | 3.82E-05 | 0.0272 | 215 | 0.130 |
| ENSP00000290575 | 1.33E-04 | 0.0270 | 394 | 0.000 |
| ENSP00000271657 | 5.32E-05 | 0.0269 | 417 | 0.000 |
| ENSP00000239761 | 4.77E-05 | 0.0267 | 290 | 0.000 |
| ENSP00000393953 | 8.13E-05 | 0.0267 | 426 | 0.834 |
| ENSP00000339299 | 4.37E-05 | 0.0263 | 214 | 0.634 |
| ENSP00000271640 | 6.18E-05 | 0.0261 | 244 | 0.882 |
| ENSP00000350785 | 3.51E-05 | 0.0261 | 270 | 0.682 |
| ENSP00000355330 | 1.43E-04 | 0.0261 | 970 | 0.906 |
| ENSP00000290378 | 3.30E-05 | 0.0261 | 570 | 0.709 |
| ENSP00000005226 | 2.33E-05 | 0.0259 | 313 | 0.342 |
| ENSP00000284440 | 7.31E-05 | 0.0259 | 414 | 0.835 |
| ENSP00000465500 | 3.71E-05 | 0.0256 | 347 | 0.683 |
| ENSP00000329623 | 7.95E-05 | 0.0255 | 940 | 0.000 |
| ENSP00000272644 | 2.32E-05 | 0.0254 | 235 | 0.533 |
| ENSP00000396722 | 3.14E-05 | 0.0253 | 302 | 0.453 |
| ENSP00000246657 | 4.66E-05 | 0.0251 | 470 | 0.898 |
| ENSP00000410294 | 9.08E-05 | 0.0251 | 674 | 0.000 |
| ENSP00000360753 | 2.72E-05 | 0.0250 | 241 | 0.111 |
| ENSP00000343477 | 7.68E-05 | 0.0250 | 829 | 0.882 |
| ENSP00000333994 | 6.76E-05 | 0.0248 | 301 | 0.831 |
| ENSP00000227155 | 6.63E-05 | 0.0242 | 834 | 0.912 |
| ENSP00000305988 | 1.59E-04 | 0.0240 | 358 | 0.885 |
| ENSP00000292377 | 3.38E-05 | 0.0239 | 900 | 0.535 |
| ENSP00000289013 | 5.53E-05 | 0.0236 | 159 | 0.000 |
| ENSP00000369375 | 8.89E-05 | 0.0236 | 915 | 0.701 |
| ENSP00000367030 | 4.63E-05 | 0.0233 | 195 | 0.779 |
| ENSP00000296511 | 8.70E-05 | 0.0229 | 433 | 0.844 |
| ENSP00000328422 | 3.18E-05 | 0.0225 | 0 | 0.000 |
| ENSP00000259486 | 1.01E-04 | 0.0225 | 207 | 0.399 |
| ENSP00000416250 | 3.05E-05 | 0.0225 | 207 | 0.558 |
| ENSP00000356920 | 2.82E-05 | 0.0218 | 284 | 0.770 |
| ENSP00000322323 | 2.23E-05 | 0.0217 | 250 | 0.303 |
| ENSP00000359225 | 4.70E-05 | 0.0217 | 368 | 0.389 |

| | | | | |
|---|---|---|---|---|
| ENSP00000274938 | 4.17E-05 | 0.0211 | 575 | 0.085 |
| ENSP00000356170 | 2.91E-05 | 0.0210 | 218 | 0.108 |
| ENSP00000360216 | 1.26E-04 | 0.0209 | 505 | 0.936 |
| ENSP00000332353 | 5.70E-05 | 0.0208 | 490 | 0.939 |
| ENSP00000462106 | 1.74E-06 | 0.0206 | 0 | 0.212 |
| ENSP00000303942 | 7.26E-05 | 0.0206 | 980 | 0.784 |
| ENSP00000373518 | 2.46E-05 | 0.0205 | 262 | 0.708 |
| ENSP00000311183 | 6.67E-05 | 0.0204 | 179 | 0.678 |
| ENSP00000231751 | 1.05E-04 | 0.0204 | 874 | 0.615 |
| ENSP00000163416 | 2.34E-05 | 0.0201 | 197 | 0.117 |
| ENSP00000272602 | 5.18E-05 | 0.0200 | 584 | 0.470 |
| ENSP00000359526 | 4.94E-05 | 0.0197 | 805 | 0.097 |
| ENSP00000217131 | 1.35E-04 | 0.0197 | 341 | 0.259 |
| ENSP00000356438 | 1.18E-04 | 0.0194 | 854 | 0.952 |
| ENSP00000319286 | 8.09E-05 | 0.0191 | 974 | 0.000 |
| ENSP00000367177 | 2.25E-05 | 0.0191 | 841 | 0.509 |
| ENSP00000349022 | 9.48E-05 | 0.0189 | 375 | 0.391 |
| ENSP00000306361 | 1.32E-04 | 0.0186 | 999 | 0.736 |
| ENSP00000364934 | 2.91E-05 | 0.0185 | 297 | 0.224 |
| ENSP00000302324 | 9.91E-05 | 0.0182 | 420 | 0.827 |
| ENSP00000303507 | 4.50E-05 | 0.0180 | 349 | 0.942 |
| ENSP00000360991 | 4.30E-05 | 0.0177 | 150 | 0.272 |
| ENSP00000277120 | 4.99E-05 | 0.0177 | 342 | 0.884 |
| ENSP00000332139 | 1.19E-04 | 0.0177 | 920 | 0.782 |
| ENSP00000350549 | 6.11E-05 | 0.0168 | 271 | 0.821 |
| ENSP00000264039 | 8.66E-05 | 0.0167 | 917 | 0.879 |
| ENSP00000368438 | 8.52E-05 | 0.0165 | 580 | 0.781 |
| ENSP00000369554 | 3.52E-05 | 0.0163 | 214 | 0.806 |
| ENSP00000250151 | 6.71E-05 | 0.0162 | 513 | 0.000 |
| ENSP00000373657 | 6.35E-05 | 0.0161 | 300 | 0.000 |
| ENSP00000242592 | 2.15E-05 | 0.0161 | 169 | 0.420 |
| ENSP00000225698 | 3.34E-05 | 0.0160 | 293 | 0.835 |
| ENSP00000352071 | 8.54E-05 | 0.0160 | 242 | 0.597 |
| ENSP00000346342 | 5.70E-05 | 0.0159 | 198 | 0.356 |
| ENSP00000260408 | 7.19E-05 | 0.0159 | 591 | 0.903 |
| ENSP00000355930 | 2.43E-05 | 0.0158 | 160 | 0.098 |
| ENSP00000361668 | 2.82E-05 | 0.0157 | 173 | 0.573 |
| ENSP00000264346 | 5.96E-05 | 0.0157 | 192 | 0.639 |
| ENSP00000442633 | 2.30E-05 | 0.0154 | 338 | 0.000 |
| ENSP00000410715 | 4.19E-05 | 0.0153 | 218 | 0.937 |
| ENSP00000382895 | 2.62E-05 | 0.0152 | 480 | 0.416 |
| ENSP00000330862 | 2.72E-05 | 0.0152 | 242 | 0.526 |
| ENSP00000263645 | 8.00E-05 | 0.0152 | 384 | 0.827 |
| ENSP00000302665 | 7.82E-05 | 0.0149 | 948 | 0.975 |
| ENSP00000359215 | 4.22E-05 | 0.0145 | 170 | 0.856 |
| ENSP00000422496 | 4.73E-05 | 0.0143 | 179 | 0.176 |

| | | | | |
|---|---|---|---|---|
| ENSP00000370812 | 3.36E-05 | 0.0142 | 254 | 0.480 |
| ENSP00000243347 | 6.98E-05 | 0.0141 | 656 | 0.820 |
| ENSP00000297873 | 3.41E-05 | 0.0135 | 225 | 0.682 |
| ENSP00000306697 | 6.91E-05 | 0.0135 | 325 | 0.895 |
| ENSP00000358511 | 3.13E-05 | 0.0134 | 301 | 0.829 |
| ENSP00000368682 | 5.82E-05 | 0.0134 | 426 | 0.723 |
| ENSP00000359497 | 6.36E-05 | 0.0132 | 907 | 0.542 |
| ENSP00000409937 | 3.33E-06 | 0.0131 | 0 | 0.195 |
| ENSP00000329384 | 3.25E-05 | 0.0130 | 239 | 0.737 |
| ENSP00000442050 | 2.91E-05 | 0.0127 | 281 | 0.233 |
| ENSP00000301522 | 5.10E-05 | 0.0126 | 581 | 0.779 |
| ENSP00000321584 | 4.53E-05 | 0.0125 | 649 | 0.712 |
| ENSP00000349114 | 3.42E-05 | 0.0121 | 0 | 0.000 |
| ENSP00000305631 | 4.30E-05 | 0.0118 | 390 | 0.253 |
| ENSP00000346725 | 4.78E-05 | 0.0111 | 271 | 0.813 |
| ENSP00000360855 | 4.70E-05 | 0.0110 | 359 | 0.092 |
| ENSP00000230882 | 6.27E-05 | 0.0110 | 216 | 0.901 |
| ENSP00000224764 | 5.73E-05 | 0.0107 | 252 | 0.000 |
| ENSP00000241014 | 4.11E-05 | 0.0106 | 325 | 0.826 |
| ENSP00000303599 | 3.43E-05 | 0.0103 | 558 | 0.000 |
| ENSP00000410076 | 1.10E-04 | 0.0103 | 487 | 0.930 |
| ENSP00000196061 | 1.12E-04 | 0.0098 | 313 | 0.538 |
| ENSP00000304236 | 4.06E-05 | 0.0098 | 265 | 0.673 |
| ENSP00000403609 | 7.66E-07 | 0.0095 | 0 | 0.000 |
| ENSP00000385638 | 4.79E-05 | 0.0094 | 328 | 0.516 |
| ENSP00000371321 | 4.70E-05 | 0.0093 | 282 | 0.822 |
| ENSP00000352980 | 4.54E-05 | 0.0092 | 884 | 0.000 |
| ENSP00000333203 | 6.87E-05 | 0.0092 | 647 | 0.854 |
| ENSP00000306549 | 3.53E-05 | 0.0091 | 558 | 0.000 |
| ENSP00000361007 | 4.78E-05 | 0.0088 | 196 | 0.220 |
| ENSP00000371294 | 4.84E-05 | 0.0088 | 196 | 0.115 |
| ENSP00000377470 | 3.51E-05 | 0.0085 | 439 | 0.249 |
| ENSP00000351363 | 1.60E-03 | 0.0085 | 196 | 0.000 |
| ENSP00000377298 | 5.18E-05 | 0.0083 | 356 | 0.563 |
| ENSP00000332931 | 8.38E-05 | 0.0080 | 191 | 0.253 |
| ENSP00000222399 | 1.27E-04 | 0.0080 | 392 | 0.593 |
| ENSP00000429214 | 2.60E-05 | 0.0080 | 269 | 0.936 |
| ENSP00000295137 | 3.20E-05 | 0.0079 | 539 | 0.705 |
| ENSP00000401177 | 2.30E-05 | 0.0077 | 205 | 0.522 |
| ENSP00000398632 | 7.78E-05 | 0.0076 | 995 | 0.962 |
| ENSP00000261693 | 6.02E-05 | 0.0075 | 983 | 0.739 |
| ENSP00000444408 | 8.35E-05 | 0.0074 | 277 | 0.181 |
| ENSP00000215631 | 1.01E-04 | 0.0073 | 221 | 0.864 |
| ENSP00000461413 | 4.22E-05 | 0.0073 | 163 | 0.694 |
| ENSP00000245414 | 4.87E-05 | 0.0072 | 988 | 0.889 |
| ENSP00000234091 | 5.95E-05 | 0.0068 | 282 | 0.879 |

| | | | | |
|---|---|---|---|---|
| ENSP00000278317 | 4.38E-05 | 0.0067 | 904 | 0.496 |
| ENSP00000273368 | 1.09E-04 | 0.0067 | 352 | 0.367 |
| ENSP00000305692 | 1.06E-04 | 0.0066 | 426 | 0.269 |
| ENSP00000366974 | 5.42E-05 | 0.0065 | 884 | 0.964 |
| ENSP00000378332 | 4.06E-05 | 0.0061 | 228 | 0.813 |
| ENSP00000262158 | 5.58E-05 | 0.0061 | 937 | 0.903 |
| ENSP00000252595 | 8.70E-05 | 0.0061 | 389 | 0.584 |
| ENSP00000298085 | 7.64E-05 | 0.0061 | 301 | 0.167 |
| ENSP00000294954 | 2.23E-05 | 0.0060 | 219 | 0.722 |
| ENSP00000305839 | 1.16E-04 | 0.0060 | 150 | 0.222 |
| ENSP00000264036 | 1.00E-04 | 0.0056 | 660 | 0.910 |
| ENSP00000356591 | 4.35E-05 | 0.0056 | 658 | 0.504 |
| ENSP00000356505 | 6.71E-05 | 0.0055 | 932 | 0.844 |
| ENSP00000345230 | 4.06E-05 | 0.0055 | 915 | 0.437 |
| ENSP00000354394 | 8.71E-05 | 0.0054 | 899 | 0.000 |
| ENSP00000397900 | 1.89E-05 | 0.0054 | 210 | 0.369 |
| ENSP00000310515 | 4.51E-05 | 0.0051 | 261 | 0.245 |
| ENSP00000357650 | 7.68E-05 | 0.0050 | 192 | 0.310 |
| ENSP00000340210 | 8.59E-05 | 0.0049 | 343 | 0.766 |
| ENSP00000280357 | 9.06E-05 | 0.0049 | 898 | 0.915 |
| ENSP00000408632 | 3.92E-05 | 0.0048 | 223 | 0.896 |
| ENSP00000278616 | 6.37E-05 | 0.0048 | 287 | 0.796 |
| ENSP00000396402 | 4.72E-05 | 0.0047 | 310 | 0.688 |
| ENSP00000233946 | 1.25E-04 | 0.0045 | 202 | 0.000 |
| ENSP00000324648 | 6.17E-05 | 0.0043 | 328 | 0.691 |
| ENSP00000261937 | 7.15E-05 | 0.0042 | 908 | 0.669 |
| ENSP00000245552 | 4.96E-05 | 0.0038 | 305 | 0.438 |
| ENSP00000393762 | 4.84E-05 | 0.0037 | 157 | 0.496 |
| ENSP00000352138 | 5.87E-05 | 0.0037 | 400 | 0.186 |
| ENSP00000318631 | 9.61E-05 | 0.0037 | 459 | 0.853 |
| ENSP00000472225 | 7.53E-06 | 0.0034 | 0 | 0.000 |
| ENSP00000264350 | 5.57E-05 | 0.0034 | 169 | 0.662 |
| ENSP00000299339 | 2.99E-05 | 0.0033 | 159 | 0.095 |
| ENSP00000209873 | 2.36E-04 | 0.0032 | 640 | 0.412 |
| ENSP00000312741 | 6.44E-05 | 0.0031 | 250 | 0.827 |
| ENSP00000293308 | 7.84E-05 | 0.0030 | 675 | 0.838 |
| ENSP00000398698 | 9.24E-05 | 0.0029 | 984 | 0.000 |
| ENSP00000383563 | 8.88E-05 | 0.0028 | 254 | 0.085 |
| ENSP00000230124 | 6.73E-05 | 0.0028 | 212 | 0.227 |
| ENSP00000236147 | 4.75E-05 | 0.0028 | 509 | 0.887 |
| ENSP00000250457 | 7.23E-05 | 0.0025 | 213 | 0.752 |
| ENSP00000302812 | 4.90E-05 | 0.0020 | 565 | 0.918 |
| ENSP00000231572 | 3.58E-05 | 0.0019 | 771 | 0.785 |
| ENSP00000421799 | 4.16E-05 | 0.0017 | 349 | 0.000 |
| ENSP00000360576 | 6.10E-05 | 0.0016 | 178 | 0.154 |
| ENSP00000251363 | 3.62E-05 | 0.0016 | 213 | 0.491 |

| | | | | |
|---|---|---|---|---|
| ENSP00000300406 | 3.31E-05 | 0.0013 | 385 | 0.463 |
| ENSP00000379866 | 3.65E-05 | 0.0013 | 645 | 0.602 |
| ENSP00000364293 | 4.52E-05 | 0.0013 | 301 | 0.791 |
| ENSP00000167586 | 8.07E-05 | 0.0012 | 309 | 0.920 |
| ENSP00000359380 | 1.15E-04 | 0.0012 | 560 | 0.635 |
| ENSP00000366124 | 1.09E-04 | 0.0011 | 581 | 0.832 |
| ENSP00000241125 | 2.09E-04 | 0.0011 | 757 | 0.355 |
| ENSP00000228682 | 2.88E-05 | 0.0011 | 346 | 0.912 |
| ENSP00000328521 | 3.83E-05 | 0.0011 | 510 | 0.180 |
| ENSP00000306099 | 1.18E-04 | 0.0010 | 999 | 0.756 |
| ENSP00000373783 | 6.10E-05 | 0.0009 | 263 | 0.602 |
| ENSP00000264908 | 1.49E-04 | 0.0006 | 215 | 0.367 |
| ENSP00000202831 | 9.23E-05 | 0.0003 | 323 | 0.000 |
| ENSP00000354341 | 7.66E-05 | 0.0003 | 539 | 0.523 |
| ENSP00000216968 | 2.80E-04 | 0.0003 | 210 | 0.269 |
| ENSP00000325355 | 6.55E-05 | 0.0002 | 201 | 0.560 |
| ENSP00000236877 | 4.00E-05 | 0.0001 | 313 | 0.174 |
| ENSP00000396688 | 4.48E-05 | 0.0000 | 361 | 0.584 |
| ENSP00000220166 | 1.21E-04 | -0.0001 | 496 | 0.333 |
| ENSP00000284601 | 3.80E-05 | -0.0002 | 161 | 0.491 |
| ENSP00000354923 | 8.59E-05 | -0.0003 | 905 | 0.876 |
| ENSP00000349324 | 2.65E-05 | -0.0004 | 205 | 0.586 |
| ENSP00000337949 | 4.93E-05 | -0.0004 | 359 | 0.832 |
| ENSP00000246912 | 1.24E-04 | -0.0005 | 227 | 0.808 |
| ENSP00000264025 | 8.18E-05 | -0.0005 | 329 | 0.803 |
| ENSP00000351642 | 6.58E-05 | -0.0006 | 0 | 0.097 |
| ENSP00000357555 | 4.81E-05 | -0.0007 | 212 | 0.751 |
| ENSP00000222693 | 1.30E-04 | -0.0008 | 284 | 0.764 |
| ENSP00000292823 | 1.06E-04 | -0.0008 | 420 | 0.258 |
| ENSP00000327025 | 3.29E-05 | -0.0008 | 224 | 0.859 |
| ENSP00000336630 | 6.24E-05 | -0.0017 | 298 | 0.784 |
| ENSP00000303394 | 1.50E-04 | -0.0019 | 873 | 0.891 |
| ENSP00000361014 | 4.03E-05 | -0.0020 | 242 | 0.552 |
| ENSP00000350256 | 2.13E-05 | -0.0020 | 305 | 0.655 |
| ENSP00000328674 | 2.09E-05 | -0.0020 | 152 | 0.093 |
| ENSP00000252674 | 3.31E-05 | -0.0022 | 598 | 0.822 |
| ENSP00000000412 | 8.52E-05 | -0.0022 | 241 | 0.346 |
| ENSP00000252137 | 9.00E-05 | -0.0024 | 249 | 0.427 |
| ENSP00000409555 | 3.30E-05 | -0.0024 | 452 | 0.633 |
| ENSP00000351664 | 3.40E-05 | -0.0026 | 576 | 0.491 |
| ENSP00000217423 | 1.74E-04 | -0.0030 | 581 | 0.305 |
| ENSP00000350630 | 5.73E-05 | -0.0030 | 388 | 0.296 |
| ENSP00000434480 | 3.29E-05 | -0.0030 | 151 | 0.141 |
| ENSP00000360217 | 1.00E-04 | -0.0030 | 416 | 0.175 |
| ENSP00000353224 | 8.85E-05 | -0.0031 | 594 | 0.902 |
| ENSP00000331727 | 2.27E-05 | -0.0033 | 186 | 0.116 |

| | | | | |
|---|---|---|---|---|
| ENSP00000345096 | 2.93E-05 | -0.0033 | 511 | 0.541 |
| ENSP00000379110 | 4.68E-05 | -0.0035 | 766 | 0.891 |
| ENSP00000348099 | 4.44E-05 | -0.0037 | 850 | 0.870 |
| ENSP00000300408 | 5.16E-05 | -0.0037 | 434 | 0.768 |
| ENSP00000230361 | 3.23E-05 | -0.0037 | 515 | 0.353 |
| ENSP00000264498 | 5.84E-05 | -0.0038 | 948 | 0.000 |
| ENSP00000265361 | 5.70E-05 | -0.0038 | 151 | 0.705 |
| ENSP00000356286 | 3.26E-05 | -0.0038 | 908 | 0.731 |
| ENSP00000315379 | 3.73E-05 | -0.0041 | 357 | 0.753 |
| ENSP00000298295 | 2.17E-05 | -0.0043 | 191 | 0.704 |
| ENSP00000343657 | 3.42E-05 | -0.0044 | 176 | 0.252 |
| ENSP00000356825 | 7.05E-05 | -0.0048 | 472 | 0.702 |
| ENSP00000316786 | 2.68E-05 | -0.0049 | 216 | 0.769 |
| ENSP00000284523 | 3.55E-05 | -0.0049 | 761 | 0.944 |
| ENSP00000334665 | 4.11E-05 | -0.0050 | 452 | 0.335 |
| ENSP00000234371 | 2.25E-05 | -0.0051 | 280 | 0.655 |
| ENSP00000356599 | 5.35E-05 | -0.0052 | 343 | 0.180 |
| ENSP00000271638 | 1.74E-04 | -0.0053 | 282 | 0.436 |
| ENSP00000325713 | 2.21E-04 | -0.0055 | 0 | 0.178 |
| ENSP00000260630 | 6.43E-05 | -0.0055 | 242 | 0.000 |
| ENSP00000242480 | 3.30E-05 | -0.0058 | 407 | 0.868 |
| ENSP00000219271 | 1.57E-04 | -0.0058 | 995 | 0.836 |
| ENSP00000295408 | 3.85E-05 | -0.0058 | 598 | 0.680 |
| ENSP00000356953 | 6.88E-05 | -0.0059 | 457 | 0.843 |
| ENSP00000325905 | 9.66E-05 | -0.0059 | 361 | 0.842 |
| ENSP00000287936 | 4.30E-05 | -0.0060 | 720 | 0.785 |
| ENSP00000323856 | 6.72E-05 | -0.0060 | 781 | 0.538 |
| ENSP00000340292 | 4.44E-05 | -0.0060 | 833 | 0.912 |
| ENSP00000321746 | 3.31E-05 | -0.0060 | 497 | 0.852 |
| ENSP00000252456 | 8.37E-05 | -0.0061 | 368 | 0.722 |
| ENSP00000354476 | 6.10E-05 | -0.0062 | 581 | 0.748 |
| ENSP00000368104 | 9.44E-05 | -0.0062 | 859 | 0.000 |
| ENSP00000366853 | 4.22E-05 | -0.0064 | 179 | 0.000 |
| ENSP00000295666 | 1.30E-04 | -0.0066 | 757 | 0.742 |
| ENSP00000343924 | 6.11E-05 | -0.0066 | 914 | 0.772 |
| ENSP00000464890 | 2.02E-05 | -0.0066 | 0 | 0.404 |
| ENSP00000222573 | 1.12E-04 | -0.0068 | 814 | 0.709 |
| ENSP00000308938 | 1.11E-04 | -0.0068 | 998 | 0.909 |
| ENSP00000352673 | 4.54E-05 | -0.0068 | 820 | 0.869 |
| ENSP00000365651 | 3.18E-05 | -0.0068 | 197 | 0.556 |
| ENSP00000362308 | 4.65E-05 | -0.0069 | 197 | 0.081 |
| ENSP00000219700 | 3.75E-05 | -0.0070 | 330 | 0.912 |
| ENSP00000284987 | 1.76E-04 | -0.0070 | 560 | 0.788 |
| ENSP00000275428 | 1.41E-04 | -0.0071 | 903 | 0.388 |
| ENSP00000296518 | 6.70E-05 | -0.0073 | 151 | 0.436 |
| ENSP00000311997 | 4.95E-05 | -0.0073 | 377 | 0.700 |

| | | | | |
|---|---|---|---|---|
| ENSP00000369756 | 2.93E-05 | -0.0076 | 900 | 0.723 |
| ENSP00000342609 | 3.35E-05 | -0.0077 | 151 | 0.106 |
| ENSP00000379616 | 4.76E-05 | -0.0078 | 814 | 0.863 |
| ENSP00000225893 | 2.03E-05 | -0.0079 | 177 | 0.000 |
| ENSP00000303070 | 2.90E-05 | -0.0079 | 198 | 0.134 |
| ENSP00000232219 | 1.15E-04 | -0.0081 | 910 | 0.778 |
| ENSP00000358283 | 4.55E-05 | -0.0082 | 916 | 0.086 |
| ENSP00000261349 | 7.59E-05 | -0.0083 | 615 | 0.928 |
| ENSP00000264487 | 6.31E-05 | -0.0084 | 778 | 0.000 |
| ENSP00000253303 | 4.62E-05 | -0.0084 | 266 | 0.284 |
| ENSP00000379282 | 3.51E-05 | -0.0085 | 0 | 0.186 |
| ENSP00000334052 | 1.26E-04 | -0.0085 | 506 | 0.202 |
| ENSP00000216181 | 2.94E-05 | -0.0085 | 600 | 0.763 |
| ENSP00000229330 | 2.53E-05 | -0.0085 | 199 | 0.692 |
| ENSP00000243349 | 2.89E-05 | -0.0086 | 239 | 0.835 |
| ENSP00000356385 | 3.00E-05 | -0.0089 | 187 | 0.222 |
| ENSP00000222266 | 9.11E-05 | -0.0090 | 407 | 0.647 |
| ENSP00000369634 | 8.61E-05 | -0.0090 | 574 | 0.780 |
| ENSP00000265195 | 7.50E-05 | -0.0090 | 781 | 0.627 |
| ENSP00000380450 | 1.84E-05 | -0.0092 | 0 | 0.059 |
| ENSP00000219789 | 3.85E-05 | -0.0093 | 340 | 0.523 |
| ENSP00000216780 | 5.12E-05 | -0.0094 | 329 | 0.734 |
| ENSP00000402203 | 5.40E-07 | -0.0094 | 0 | 0.000 |
| ENSP00000471397 | 2.54E-05 | -0.0094 | 201 | 0.000 |
| ENSP00000367301 | 1.10E-04 | -0.0094 | 361 | 0.657 |
| ENSP00000381412 | 5.23E-05 | -0.0097 | 625 | 0.677 |
| ENSP00000309503 | 6.41E-05 | -0.0097 | 927 | 0.713 |
| ENSP00000166139 | 1.35E-04 | -0.0099 | 607 | 0.795 |
| ENSP00000206765 | 9.07E-05 | -0.0102 | 212 | 0.726 |
| ENSP00000353915 | 2.47E-05 | -0.0103 | 806 | 0.629 |
| ENSP00000262776 | 1.28E-04 | -0.0103 | 629 | 0.458 |
| ENSP00000286332 | 6.63E-05 | -0.0104 | 900 | 0.000 |
| ENSP00000359285 | 2.98E-05 | -0.0104 | 864 | 0.654 |
| ENSP00000325239 | 2.14E-05 | -0.0104 | 310 | 0.470 |
| ENSP00000261735 | 1.70E-04 | -0.0105 | 782 | 0.291 |
| ENSP00000199280 | 3.65E-05 | -0.0109 | 310 | 0.331 |
| ENSP00000314407 | 1.17E-04 | -0.0109 | 254 | 0.201 |
| ENSP00000315757 | 5.04E-05 | -0.0109 | 686 | 0.531 |
| ENSP00000261769 | 4.77E-05 | -0.0111 | 976 | 0.973 |
| ENSP00000390475 | 3.81E-05 | -0.0111 | 203 | 0.886 |
| ENSP00000237014 | 6.36E-05 | -0.0112 | 792 | 0.913 |
| ENSP00000266085 | 1.32E-04 | -0.0112 | 989 | 0.959 |
| ENSP00000324205 | 1.38E-04 | -0.0114 | 414 | 0.123 |
| ENSP00000422131 | 8.10E-05 | -0.0114 | 167 | 0.133 |
| ENSP00000282470 | 1.21E-04 | -0.0114 | 224 | 0.533 |
| ENSP00000421280 | 4.85E-05 | -0.0114 | 244 | 0.441 |

| | | | | |
|---|---|---|---|---|
| ENSP00000265070 | 8.04E-05 | -0.0115 | 538 | 0.651 |
| ENSP00000382513 | 4.19E-05 | -0.0117 | 158 | 0.689 |
| ENSP00000347088 | 9.53E-05 | -0.0117 | 240 | 0.660 |
| ENSP00000470555 | 1.68E-05 | -0.0118 | 472 | 0.192 |
| ENSP00000254801 | 1.21E-04 | -0.0119 | 608 | 0.000 |
| ENSP00000291568 | 6.03E-05 | -0.0121 | 511 | 0.454 |
| ENSP00000222572 | 2.14E-04 | -0.0121 | 499 | 0.416 |
| ENSP00000381293 | 4.68E-05 | -0.0123 | 396 | 0.503 |
| ENSP00000410024 | 8.73E-07 | -0.0124 | 0 | 0.215 |
| ENSP00000324101 | 1.30E-04 | -0.0126 | 864 | 0.646 |
| ENSP00000301634 | 5.45E-05 | -0.0127 | 215 | 0.664 |
| ENSP00000386169 | 1.21E-05 | -0.0127 | 0 | 0.000 |
| ENSP00000318799 | 3.45E-05 | -0.0128 | 195 | 0.805 |
| ENSP00000351947 | 3.38E-05 | -0.0129 | 264 | 0.899 |
| ENSP00000367086 | 5.24E-05 | -0.0129 | 260 | 0.208 |
| ENSP00000367888 | 1.98E-05 | -0.0132 | 398 | 0.487 |
| ENSP00000353731 | 5.42E-05 | -0.0133 | 827 | 0.782 |
| ENSP00000358866 | 3.63E-05 | -0.0135 | 822 | 0.608 |
| ENSP00000071281 | 2.18E-04 | -0.0136 | 200 | 0.629 |
| ENSP00000259953 | 2.56E-05 | -0.0136 | 345 | 0.314 |
| ENSP00000320430 | 2.76E-05 | -0.0137 | 0 | 0.152 |
| ENSP00000054950 | 5.16E-05 | -0.0139 | 381 | 0.348 |
| ENSP00000325146 | 1.88E-04 | -0.0140 | 911 | 0.470 |
| ENSP00000428340 | 7.28E-05 | -0.0140 | 912 | 0.975 |
| ENSP00000348170 | 1.49E-04 | -0.0141 | 959 | 0.807 |
| ENSP00000265171 | 6.28E-05 | -0.0142 | 915 | 0.719 |
| ENSP00000357075 | 9.33E-05 | -0.0143 | 338 | 0.395 |
| ENSP00000354519 | 2.80E-05 | -0.0145 | 212 | 0.153 |
| ENSP00000378957 | 6.07E-05 | -0.0145 | 905 | 0.440 |
| ENSP00000364119 | 3.77E-05 | -0.0145 | 157 | 0.841 |
| ENSP00000296387 | 3.37E-05 | -0.0146 | 191 | 0.096 |
| ENSP00000438284 | 4.24E-05 | -0.0147 | 511 | 0.654 |
| ENSP00000244137 | 2.66E-05 | -0.0148 | 683 | 0.786 |
| ENSP00000357440 | 3.69E-05 | -0.0149 | 803 | 0.000 |
| ENSP00000305056 | 3.41E-05 | -0.0149 | 550 | 0.000 |
| ENSP00000361943 | 2.93E-05 | -0.0150 | 323 | 0.835 |
| ENSP00000346827 | 3.02E-05 | -0.0152 | 216 | 0.299 |
| ENSP00000323588 | 4.40E-05 | -0.0154 | 492 | 0.852 |
| ENSP00000234071 | 6.50E-05 | -0.0155 | 819 | 0.928 |
| ENSP00000232014 | 2.66E-05 | -0.0156 | 482 | 0.836 |
| ENSP00000328169 | 5.27E-05 | -0.0157 | 216 | 0.904 |
| ENSP00000392513 | 6.32E-05 | -0.0158 | 201 | 0.196 |
| ENSP00000451112 | 3.49E-05 | -0.0158 | 394 | 0.376 |
| ENSP00000237612 | 3.85E-05 | -0.0158 | 349 | 0.840 |
| ENSP00000261623 | 1.20E-04 | -0.0161 | 944 | 0.844 |
| ENSP00000304447 | 6.99E-05 | -0.0161 | 292 | 0.874 |

| | | | | |
|---|---|---|---|---|
| ENSP00000402855 | 1.80E-05 | -0.0162 | 209 | 0.115 |
| ENSP00000280362 | 7.23E-05 | -0.0163 | 211 | 0.740 |
| ENSP00000301974 | 7.37E-05 | -0.0164 | 927 | 0.755 |
| ENSP00000376322 | 3.28E-05 | -0.0164 | 200 | 0.866 |
| ENSP00000335153 | 7.40E-05 | -0.0164 | 992 | 0.848 |
| ENSP00000286713 | 9.11E-05 | -0.0164 | 203 | 0.416 |
| ENSP00000359485 | 5.77E-05 | -0.0165 | 403 | 0.180 |
| ENSP00000225728 | 7.42E-05 | -0.0167 | 281 | 0.730 |
| ENSP00000274341 | 1.17E-04 | -0.0167 | 628 | 0.523 |
| ENSP00000295206 | 3.16E-05 | -0.0168 | 504 | 0.866 |
| ENSP00000298171 | 5.83E-05 | -0.0168 | 600 | 0.759 |
| ENSP00000296946 | 1.91E-05 | -0.0169 | 273 | 0.856 |
| ENSP00000215773 | 1.09E-04 | -0.0170 | 282 | 0.831 |
| ENSP00000274793 | 8.86E-05 | -0.0170 | 501 | 0.418 |
| ENSP00000406955 | 4.64E-05 | -0.0172 | 242 | 0.718 |
| ENSP00000267082 | 7.07E-05 | -0.0172 | 967 | 0.809 |
| ENSP00000241052 | 2.94E-05 | -0.0173 | 641 | 0.683 |
| ENSP00000367208 | 4.05E-05 | -0.0176 | 847 | 0.323 |
| ENSP00000440698 | 5.21E-05 | -0.0176 | 480 | 0.378 |
| ENSP00000260356 | 7.57E-05 | -0.0177 | 997 | 0.971 |
| ENSP00000279022 | 3.57E-05 | -0.0178 | 463 | 0.660 |
| ENSP00000301873 | 2.35E-04 | -0.0178 | 454 | 0.451 |
| ENSP00000252660 | 6.10E-05 | -0.0178 | 181 | 0.165 |
| ENSP00000262269 | 3.75E-05 | -0.0179 | 752 | 0.928 |
| ENSP00000254079 | 6.98E-05 | -0.0181 | 226 | 0.714 |
| ENSP00000290401 | 1.35E-04 | -0.0181 | 462 | 0.412 |
| ENSP00000405222 | 2.07E-05 | -0.0181 | 156 | 0.136 |
| ENSP00000327246 | 2.79E-05 | -0.0181 | 429 | 0.666 |
| ENSP00000349960 | 6.67E-05 | -0.0182 | 648 | 0.000 |
| ENSP00000304283 | 4.77E-05 | -0.0183 | 910 | 0.759 |
| ENSP00000383517 | 6.31E-07 | -0.0183 | 0 | 0.000 |
| ENSP00000242786 | 9.77E-05 | -0.0183 | 301 | 0.000 |
| ENSP00000373674 | 2.71E-05 | -0.0186 | 282 | 0.189 |
| ENSP00000264896 | 7.46E-05 | -0.0187 | 414 | 0.473 |
| ENSP00000361536 | 4.02E-05 | -0.0187 | 157 | 0.326 |
| ENSP00000234198 | 4.55E-05 | -0.0188 | 282 | 0.869 |
| ENSP00000217182 | 2.41E-05 | -0.0189 | 349 | 0.763 |
| ENSP00000313886 | 3.06E-05 | -0.0189 | 168 | 0.000 |
| ENSP00000377545 | 2.60E-05 | -0.0189 | 313 | 0.176 |
| ENSP00000417235 | 2.52E-05 | -0.0189 | 370 | 0.558 |
| ENSP00000301490 | 2.13E-05 | -0.0190 | 195 | 0.261 |
| ENSP00000234170 | 5.05E-05 | -0.0190 | 405 | 0.876 |
| ENSP00000363506 | 4.18E-05 | -0.0191 | 297 | 0.000 |
| ENSP00000263321 | 3.59E-05 | -0.0191 | 349 | 0.909 |
| ENSP00000354238 | 3.29E-05 | -0.0191 | 301 | 0.440 |
| ENSP00000262623 | 3.04E-05 | -0.0192 | 634 | 0.680 |

| | | | | |
|---|---|---|---|---|
| ENSP00000227495 | 1.08E-04 | -0.0195 | 900 | 0.158 |
| ENSP00000333664 | 7.54E-05 | -0.0195 | 309 | 0.445 |
| ENSP00000350024 | 2.99E-05 | -0.0195 | 197 | 0.112 |
| ENSP00000216951 | 7.66E-05 | -0.0197 | 922 | 0.407 |
| ENSP00000269260 | 3.00E-05 | -0.0199 | 930 | 0.000 |
| ENSP00000318977 | 3.38E-05 | -0.0199 | 565 | 0.747 |
| ENSP00000308815 | 4.27E-05 | -0.0199 | 501 | 0.770 |
| ENSP00000375067 | 9.16E-05 | -0.0200 | 576 | 0.237 |
| ENSP00000405926 | 7.91E-05 | -0.0200 | 204 | 0.163 |
| ENSP00000336666 | 9.10E-05 | -0.0200 | 175 | 0.210 |
| ENSP00000345464 | 4.28E-05 | -0.0202 | 585 | 0.491 |
| ENSP00000168216 | 4.45E-05 | -0.0202 | 170 | 0.181 |
| ENSP00000434442 | 4.67E-05 | -0.0202 | 203 | 0.189 |
| ENSP00000410943 | 4.29E-05 | -0.0204 | 153 | 0.000 |
| ENSP00000246635 | 2.21E-04 | -0.0204 | 193 | 0.137 |
| ENSP00000337040 | 3.38E-05 | -0.0205 | 418 | 0.414 |
| ENSP00000252999 | 9.87E-05 | -0.0206 | 220 | 0.547 |
| ENSP00000269886 | 3.03E-05 | -0.0207 | 720 | 0.753 |
| ENSP00000296464 | 3.17E-05 | -0.0207 | 515 | 0.760 |
| ENSP00000370997 | 8.21E-07 | -0.0208 | 0 | 0.214 |
| ENSP00000415452 | 2.17E-05 | -0.0208 | 154 | 0.000 |
| ENSP00000376652 | 4.95E-05 | -0.0208 | 323 | 0.415 |
| ENSP00000386284 | 9.60E-05 | -0.0209 | 567 | 0.302 |
| ENSP00000265394 | 5.17E-05 | -0.0210 | 351 | 0.146 |
| ENSP00000025008 | 6.27E-05 | -0.0211 | 308 | 0.633 |
| ENSP00000387662 | 7.63E-05 | -0.0213 | 472 | 0.916 |
| ENSP00000244364 | 4.60E-05 | -0.0213 | 309 | 0.593 |
| ENSP00000302895 | 4.82E-05 | -0.0214 | 311 | 0.286 |
| ENSP00000354526 | 5.19E-05 | -0.0214 | 181 | 0.597 |
| ENSP00000460682 | 3.20E-05 | -0.0216 | 398 | 0.000 |
| ENSP00000379086 | 2.76E-05 | -0.0216 | 312 | 0.520 |
| ENSP00000316333 | 6.32E-05 | -0.0217 | 288 | 0.592 |
| ENSP00000262626 | 8.52E-05 | -0.0217 | 210 | 0.448 |
| ENSP00000302486 | 6.36E-05 | -0.0217 | 377 | 0.657 |
| ENSP00000005558 | 1.09E-04 | -0.0217 | 321 | 0.507 |
| ENSP00000292401 | 1.11E-04 | -0.0218 | 176 | 0.396 |
| ENSP00000364163 | 7.91E-05 | -0.0220 | 451 | 0.923 |
| ENSP00000285930 | 4.90E-05 | -0.0221 | 364 | 0.483 |
| ENSP00000333950 | 2.68E-05 | -0.0221 | 193 | 0.756 |
| ENSP00000456648 | 3.27E-05 | -0.0223 | 584 | 0.113 |
| ENSP00000302088 | 8.72E-05 | -0.0224 | 274 | 0.212 |
| ENSP00000359484 | 6.08E-05 | -0.0225 | 168 | 0.213 |
| ENSP00000261254 | 4.71E-05 | -0.0227 | 564 | 0.890 |
| ENSP00000355988 | 2.86E-05 | -0.0228 | 900 | 0.845 |
| ENSP00000264657 | 4.39E-05 | -0.0229 | 989 | 0.000 |
| ENSP00000358202 | 2.34E-05 | -0.0231 | 238 | 0.000 |

| | | | | |
|---|---|---|---|---|
| ENSP00000456163 | 2.16E-05 | -0.0231 | 159 | 0.000 |
| ENSP00000229277 | 2.29E-05 | -0.0231 | 488 | 0.732 |
| ENSP00000064724 | 3.77E-05 | -0.0231 | 301 | 0.505 |
| ENSP00000228918 | 7.76E-05 | -0.0232 | 235 | 0.842 |
| ENSP00000339824 | 3.87E-05 | -0.0232 | 665 | 0.701 |
| ENSP00000262406 | 7.61E-05 | -0.0233 | 416 | 0.433 |
| ENSP00000357392 | 4.57E-05 | -0.0234 | 264 | 0.860 |
| ENSP00000177694 | 3.24E-05 | -0.0234 | 266 | 0.904 |
| ENSP00000352264 | 3.51E-05 | -0.0235 | 457 | 0.776 |
| ENSP00000338606 | 6.70E-05 | -0.0235 | 472 | 0.677 |
| ENSP00000266066 | 3.33E-05 | -0.0235 | 239 | 0.892 |
| ENSP00000264552 | 2.93E-05 | -0.0235 | 224 | 0.721 |
| ENSP00000241453 | 2.26E-05 | -0.0237 | 345 | 0.691 |
| ENSP00000314620 | 1.43E-04 | -0.0238 | 778 | 0.601 |
| ENSP00000268097 | 2.01E-05 | -0.0238 | 379 | 0.301 |
| ENSP00000339027 | 4.25E-05 | -0.0239 | 497 | 0.861 |
| ENSP00000312652 | 6.36E-05 | -0.0239 | 937 | 0.957 |
| ENSP00000264568 | 6.18E-05 | -0.0239 | 202 | 0.899 |
| ENSP00000356713 | 4.32E-05 | -0.0240 | 356 | 0.871 |
| ENSP00000263093 | 8.02E-05 | -0.0240 | 283 | 0.426 |
| ENSP00000352510 | 4.21E-05 | -0.0240 | 221 | 0.108 |
| ENSP00000176763 | 4.73E-04 | -0.0240 | 191 | 0.216 |
| ENSP00000421736 | 6.82E-06 | -0.0240 | 0 | 0.120 |
| ENSP00000241261 | 3.43E-05 | -0.0241 | 836 | 0.905 |
| ENSP00000300456 | 7.71E-05 | -0.0241 | 336 | 0.499 |
| ENSP00000219782 | 3.57E-05 | -0.0242 | 865 | 0.860 |
| ENSP00000341828 | 4.00E-05 | -0.0242 | 208 | 0.345 |
| ENSP00000287934 | 5.02E-05 | -0.0243 | 357 | 0.960 |
| ENSP00000368439 | 2.77E-05 | -0.0243 | 420 | 0.183 |
| ENSP00000322421 | 6.58E-05 | -0.0243 | 603 | 0.500 |
| ENSP00000317272 | 5.64E-05 | -0.0244 | 839 | 0.720 |
| ENSP00000307280 | 4.23E-05 | -0.0244 | 906 | 0.481 |
| ENSP00000414303 | 7.73E-05 | -0.0245 | 889 | 0.937 |
| ENSP00000296370 | 7.60E-05 | -0.0245 | 211 | 0.583 |
| ENSP00000337255 | 3.18E-05 | -0.0246 | 192 | 0.275 |
| ENSP00000321049 | 5.90E-05 | -0.0246 | 378 | 0.681 |
| ENSP00000244458 | 5.47E-05 | -0.0247 | 183 | 0.369 |
| ENSP00000269141 | 7.59E-05 | -0.0247 | 990 | 0.958 |
| ENSP00000298687 | 2.68E-05 | -0.0248 | 240 | 0.181 |
| ENSP00000170630 | 5.39E-05 | -0.0248 | 357 | 0.855 |
| ENSP00000254066 | 3.53E-05 | -0.0248 | 829 | 0.873 |
| ENSP00000264265 | 2.41E-05 | -0.0249 | 351 | 0.519 |
| ENSP00000265029 | 2.23E-04 | -0.0252 | 196 | 0.447 |
| ENSP00000248923 | 4.57E-05 | -0.0255 | 932 | 0.909 |
| ENSP00000305682 | 3.38E-05 | -0.0257 | 405 | 0.691 |
| ENSP00000412673 | 3.25E-05 | -0.0257 | 167 | 0.330 |

| | | | | |
|---|---|---|---|---|
| ENSP00000358994 | 4.31E-05 | -0.0257 | 258 | 0.593 |
| ENSP00000276914 | 6.21E-05 | -0.0258 | 589 | 0.565 |
| ENSP00000361290 | 4.16E-05 | -0.0258 | 639 | 0.456 |
| ENSP00000274353 | 2.34E-05 | -0.0258 | 235 | 0.250 |
| ENSP00000229239 | 2.10E-05 | -0.0259 | 823 | 0.000 |
| ENSP00000324122 | 3.47E-05 | -0.0260 | 543 | 0.942 |
| ENSP00000271588 | 1.01E-04 | -0.0261 | 359 | 0.467 |
| ENSP00000362409 | 4.86E-05 | -0.0261 | 213 | 0.642 |
| ENSP00000436691 | 6.66E-05 | -0.0261 | 216 | 0.709 |
| ENSP00000257527 | 1.20E-04 | -0.0262 | 301 | 0.481 |
| ENSP00000388519 | 7.06E-07 | -0.0262 | 0 | 0.000 |
| ENSP00000248114 | 3.61E-05 | -0.0262 | 292 | 0.422 |
| ENSP00000252971 | 5.08E-05 | -0.0263 | 479 | 0.879 |
| ENSP00000384432 | 3.14E-05 | -0.0265 | 472 | 0.472 |
| ENSP00000453302 | 2.48E-05 | -0.0266 | 732 | 0.386 |
| ENSP00000245451 | 4.90E-05 | -0.0269 | 889 | 0.000 |
| ENSP00000229270 | 3.17E-05 | -0.0269 | 564 | 0.610 |
| ENSP00000367528 | 2.46E-05 | -0.0270 | 266 | 0.153 |
| ENSP00000260950 | 4.34E-05 | -0.0271 | 220 | 0.915 |
| ENSP00000355890 | 4.85E-05 | -0.0271 | 558 | 0.767 |
| ENSP00000329468 | 4.62E-05 | -0.0271 | 168 | 0.212 |
| ENSP00000355996 | 6.61E-05 | -0.0271 | 192 | 0.148 |
| ENSP00000362465 | 7.03E-05 | -0.0273 | 0 | 0.065 |
| ENSP00000403649 | 2.15E-05 | -0.0273 | 203 | 0.647 |
| ENSP00000230431 | 5.98E-05 | -0.0273 | 329 | 0.369 |
| ENSP00000373261 | 2.83E-05 | -0.0273 | 242 | 0.089 |
| ENSP00000356814 | 4.34E-05 | -0.0274 | 403 | 0.166 |
| ENSP00000310596 | 4.24E-05 | -0.0274 | 301 | 0.783 |
| ENSP00000263036 | 6.32E-05 | -0.0274 | 284 | 0.710 |
| ENSP00000348888 | 2.23E-05 | -0.0275 | 198 | 0.393 |
| ENSP00000327191 | 4.33E-05 | -0.0275 | 605 | 0.739 |
| ENSP00000348842 | 5.53E-05 | -0.0276 | 406 | 0.536 |
| ENSP00000364016 | 3.15E-05 | -0.0276 | 254 | 0.344 |
| ENSP00000225402 | 2.88E-05 | -0.0277 | 250 | 0.000 |
| ENSP00000316746 | 3.15E-05 | -0.0278 | 0 | 0.000 |
| ENSP00000254584 | 6.90E-05 | -0.0278 | 457 | 0.443 |
| ENSP00000265986 | 5.52E-05 | -0.0278 | 631 | 0.666 |
| ENSP00000262304 | 4.57E-05 | -0.0278 | 654 | 0.791 |
| ENSP00000362777 | 3.23E-05 | -0.0278 | 476 | 0.811 |
| ENSP00000284240 | 5.78E-05 | -0.0279 | 507 | 0.844 |
| ENSP00000299299 | 9.71E-05 | -0.0279 | 401 | 0.214 |
| ENSP00000357442 | 2.85E-05 | -0.0279 | 191 | 0.604 |
| ENSP00000264553 | 6.63E-05 | -0.0279 | 208 | 0.532 |
| ENSP00000264005 | 4.12E-05 | -0.0279 | 925 | 0.449 |
| ENSP00000350894 | 1.69E-04 | -0.0280 | 776 | 0.791 |
| ENSP00000258743 | 4.14E-05 | -0.0280 | 949 | 0.000 |

| | | | | |
|---|---|---|---|---|
| ENSP00000370748 | 4.44E-05 | -0.0281 | 222 | 0.373 |
| ENSP00000325120 | 7.58E-05 | -0.0282 | 854 | 0.907 |
| ENSP00000359490 | 5.24E-05 | -0.0282 | 403 | 0.128 |
| ENSP00000247470 | 6.63E-05 | -0.0283 | 330 | 0.771 |
| ENSP00000370543 | 2.98E-05 | -0.0284 | 230 | 0.206 |
| ENSP00000322276 | 2.93E-05 | -0.0284 | 198 | 0.131 |
| ENSP00000394932 | 8.61E-05 | -0.0285 | 455 | 0.000 |
| ENSP00000367527 | 2.38E-05 | -0.0285 | 153 | 0.638 |
| ENSP00000061240 | 1.17E-04 | -0.0285 | 407 | 0.573 |
| ENSP00000305651 | 3.12E-05 | -0.0286 | 472 | 0.859 |
| ENSP00000307940 | 3.32E-05 | -0.0286 | 666 | 0.841 |
| ENSP00000358490 | 3.70E-05 | -0.0286 | 360 | 0.849 |
| ENSP00000394869 | 2.43E-05 | -0.0287 | 493 | 0.556 |
| ENSP00000346916 | 4.38E-05 | -0.0288 | 154 | 0.232 |
| ENSP00000005995 | 1.22E-04 | -0.0289 | 198 | 0.234 |
| ENSP00000297373 | 2.65E-05 | -0.0289 | 0 | 0.280 |
| ENSP00000274056 | 9.21E-05 | -0.0290 | 576 | 0.279 |
| ENSP00000349593 | 1.99E-05 | -0.0290 | 0 | 0.000 |
| ENSP00000371905 | 7.20E-05 | -0.0291 | 0 | 0.078 |
| ENSP00000298139 | 3.03E-05 | -0.0291 | 305 | 0.787 |
| ENSP00000289081 | 5.16E-05 | -0.0291 | 424 | 0.687 |
| ENSP00000335371 | 1.54E-04 | -0.0291 | 328 | 0.128 |
| ENSP00000348385 | 2.93E-05 | -0.0293 | 527 | 0.672 |
| ENSP00000343282 | 4.73E-05 | -0.0294 | 883 | 0.000 |
| ENSP00000260197 | 4.87E-05 | -0.0294 | 834 | 0.325 |
| ENSP00000303208 | 3.59E-05 | -0.0295 | 585 | 0.454 |
| ENSP00000282728 | 3.80E-05 | -0.0295 | 265 | 0.860 |
| ENSP00000248272 | 1.34E-04 | -0.0296 | 681 | 0.000 |
| ENSP00000367449 | 1.76E-05 | -0.0296 | 0 | 0.000 |
| ENSP00000260129 | 1.15E-04 | -0.0297 | 900 | 0.733 |
| ENSP00000205061 | 1.16E-04 | -0.0299 | 282 | 0.378 |
| ENSP00000295522 | 6.30E-05 | -0.0300 | 221 | 0.607 |
| ENSP00000289228 | 2.77E-05 | -0.0300 | 449 | 0.694 |
| ENSP00000268251 | 2.05E-05 | -0.0301 | 215 | 0.111 |
| ENSP00000278070 | 7.33E-05 | -0.0301 | 181 | 0.746 |
| ENSP00000419027 | 4.06E-05 | -0.0301 | 223 | 0.298 |
| ENSP00000372025 | 1.06E-04 | -0.0302 | 587 | 0.948 |
| ENSP00000020673 | 6.13E-05 | -0.0302 | 290 | 0.227 |
| ENSP00000317780 | 3.88E-05 | -0.0303 | 563 | 0.382 |
| ENSP00000363081 | 7.32E-05 | -0.0303 | 787 | 0.958 |
| ENSP00000360560 | 3.92E-05 | -0.0304 | 192 | 0.686 |
| ENSP00000339740 | 7.04E-05 | -0.0306 | 576 | 0.600 |
| ENSP00000379964 | 2.77E-05 | -0.0307 | 163 | 0.534 |
| ENSP00000293829 | 2.59E-05 | -0.0307 | 152 | 0.903 |
| ENSP00000270349 | 2.68E-05 | -0.0308 | 414 | 0.723 |
| ENSP00000357674 | 3.89E-05 | -0.0309 | 153 | 0.149 |

| | | | | |
|---|---|---|---|---|
| ENSP00000304350 | 3.73E-05 | -0.0310 | 472 | 0.891 |
| ENSP00000327801 | 4.19E-05 | -0.0311 | 961 | 0.759 |
| ENSP00000225737 | 2.69E-05 | -0.0311 | 271 | 0.228 |
| ENSP00000231790 | 2.67E-05 | -0.0312 | 509 | 0.761 |
| ENSP00000221452 | 4.29E-05 | -0.0314 | 408 | 0.865 |
| ENSP00000317379 | 1.10E-04 | -0.0314 | 407 | 0.650 |
| ENSP00000284981 | 5.01E-05 | -0.0314 | 994 | 0.936 |
| ENSP00000324884 | 4.54E-05 | -0.0315 | 325 | 0.841 |
| ENSP00000298545 | 2.28E-05 | -0.0315 | 200 | 0.403 |
| ENSP00000244289 | 5.67E-05 | -0.0315 | 213 | 0.358 |
| ENSP00000251595 | 6.00E-05 | -0.0315 | 226 | 0.438 |
| ENSP00000225614 | 3.34E-05 | -0.0316 | 476 | 0.367 |
| ENSP00000249389 | 1.82E-04 | -0.0316 | 922 | 0.592 |
| ENSP00000330032 | 7.27E-05 | -0.0318 | 583 | 0.354 |
| ENSP00000267814 | 7.11E-05 | -0.0318 | 227 | 0.353 |
| ENSP00000320084 | 1.19E-04 | -0.0318 | 198 | 0.517 |
| ENSP00000220325 | 1.16E-04 | -0.0319 | 212 | 0.148 |
| ENSP00000382274 | 2.64E-05 | -0.0319 | 214 | 0.137 |
| ENSP00000348019 | 2.99E-05 | -0.0319 | 235 | 0.542 |
| ENSP00000343782 | 3.34E-05 | -0.0319 | 282 | 0.602 |
| ENSP00000363168 | 4.82E-05 | -0.0319 | 154 | 0.212 |
| ENSP00000310935 | 3.20E-05 | -0.0319 | 444 | 0.735 |
| ENSP00000291294 | 8.71E-05 | -0.0320 | 472 | 0.682 |
| ENSP00000300692 | 6.38E-05 | -0.0320 | 163 | 0.692 |
| ENSP00000299162 | 2.46E-05 | -0.0320 | 281 | 0.000 |
| ENSP00000320754 | 3.29E-05 | -0.0320 | 152 | 0.000 |
| ENSP00000219431 | 6.27E-05 | -0.0320 | 235 | 0.219 |
| ENSP00000469799 | 4.00E-07 | -0.0320 | 0 | 0.000 |
| ENSP00000290200 | 6.87E-05 | -0.0321 | 197 | 0.763 |
| ENSP00000363208 | 4.96E-05 | -0.0321 | 154 | 0.000 |
| ENSP00000370713 | 4.56E-05 | -0.0321 | 198 | 0.269 |
| ENSP00000246069 | 2.59E-05 | -0.0321 | 362 | 0.580 |
| ENSP00000450527 | 3.55E-05 | -0.0321 | 239 | 0.852 |
| ENSP00000388241 | 3.61E-05 | -0.0322 | 159 | 0.189 |
| ENSP00000264167 | 3.97E-05 | -0.0322 | 343 | 0.223 |
| ENSP00000337838 | 4.30E-05 | -0.0323 | 203 | 0.760 |
| ENSP00000252945 | 4.77E-05 | -0.0324 | 300 | 0.588 |
| ENSP00000268171 | 4.00E-05 | -0.0324 | 814 | 0.639 |
| ENSP00000251241 | 4.03E-05 | -0.0324 | 306 | 0.915 |
| ENSP00000039007 | 2.16E-05 | -0.0324 | 348 | 0.531 |
| ENSP00000252242 | 4.05E-05 | -0.0325 | 210 | 0.885 |
| ENSP00000296181 | 5.65E-05 | -0.0326 | 845 | 0.748 |
| ENSP00000261313 | 4.48E-05 | -0.0326 | 211 | 0.676 |
| ENSP00000408342 | 4.45E-05 | -0.0326 | 156 | 0.104 |
| ENSP00000292494 | 1.10E-04 | -0.0327 | 301 | 0.203 |
| ENSP00000295640 | 4.78E-05 | -0.0327 | 594 | 0.405 |

| | | | | |
|---|---|---|---|---|
| ENSP00000263094 | 6.65E-05 | -0.0328 | 563 | 0.241 |
| ENSP00000348471 | 2.89E-05 | -0.0328 | 565 | 0.115 |
| ENSP00000364731 | 1.25E-04 | -0.0328 | 963 | 0.875 |
| ENSP00000367316 | 2.65E-05 | -0.0329 | 961 | 0.541 |
| ENSP00000363873 | 8.81E-06 | -0.0330 | 0 | 0.120 |
| ENSP00000330523 | 1.49E-04 | -0.0331 | 810 | 0.223 |
| ENSP00000297848 | 4.09E-05 | -0.0331 | 918 | 0.790 |
| ENSP00000376855 | 4.86E-05 | -0.0332 | 296 | 0.153 |
| ENSP00000298556 | 4.41E-05 | -0.0332 | 293 | 0.689 |
| ENSP00000437781 | 4.02E-07 | -0.0332 | 0 | 0.000 |
| ENSP00000282903 | 6.81E-05 | -0.0333 | 212 | 0.256 |
| ENSP00000337724 | 4.67E-05 | -0.0333 | 282 | 0.736 |
| ENSP00000300026 | 5.69E-05 | -0.0333 | 946 | 0.377 |
| ENSP00000353920 | 4.43E-05 | -0.0333 | 381 | 0.240 |
| ENSP00000229319 | 2.65E-05 | -0.0334 | 427 | 0.605 |
| ENSP00000326324 | 1.11E-04 | -0.0334 | 0 | 0.136 |
| ENSP00000295057 | 4.61E-05 | -0.0336 | 0 | 0.663 |
| ENSP00000366797 | 3.40E-05 | -0.0336 | 360 | 0.319 |
| ENSP00000261558 | 3.41E-05 | -0.0336 | 240 | 0.403 |
| ENSP00000245206 | 4.70E-05 | -0.0336 | 862 | 0.424 |
| ENSP00000357917 | 5.36E-05 | -0.0336 | 190 | 0.093 |
| ENSP00000304915 | 1.38E-04 | -0.0336 | 954 | 0.890 |
| ENSP00000318197 | 2.83E-05 | -0.0337 | 260 | 0.573 |
| ENSP00000377372 | 3.60E-05 | -0.0337 | 267 | 0.814 |
| ENSP00000384259 | 4.18E-05 | -0.0337 | 169 | 0.202 |
| ENSP00000238682 | 1.01E-04 | -0.0338 | 937 | 0.949 |
| ENSP00000338769 | 5.61E-05 | -0.0340 | 242 | 0.311 |
| ENSP00000422753 | 8.10E-05 | -0.0340 | 877 | 0.961 |
| ENSP00000278856 | 4.69E-05 | -0.0341 | 199 | 0.842 |
| ENSP00000293371 | 7.37E-05 | -0.0342 | 217 | 0.358 |
| ENSP00000299402 | 3.90E-05 | -0.0342 | 499 | 0.863 |
| ENSP00000293255 | 2.39E-05 | -0.0342 | 601 | 0.654 |
| ENSP00000306185 | 4.29E-05 | -0.0342 | 390 | 0.825 |
| ENSP00000297579 | 4.39E-05 | -0.0342 | 0 | 0.846 |
| ENSP00000419485 | 1.56E-05 | -0.0342 | 156 | 0.580 |
| ENSP00000401310 | 1.37E-05 | -0.0343 | 0 | 0.115 |
| ENSP00000359910 | 5.18E-05 | -0.0343 | 357 | 0.622 |
| ENSP00000252593 | 9.42E-05 | -0.0344 | 742 | 0.513 |
| ENSP00000217893 | 3.88E-05 | -0.0344 | 234 | 0.819 |
| ENSP00000256509 | 6.61E-05 | -0.0345 | 323 | 0.900 |
| ENSP00000348163 | 3.05E-05 | -0.0345 | 638 | 0.526 |
| ENSP00000417711 | 8.42E-07 | -0.0345 | 0 | 0.203 |
| ENSP00000415034 | 2.76E-05 | -0.0345 | 560 | 0.227 |
| ENSP00000304592 | 1.02E-04 | -0.0345 | 580 | 0.757 |
| ENSP00000329243 | 6.04E-05 | -0.0346 | 242 | 0.683 |
| ENSP00000222800 | 4.63E-05 | -0.0346 | 165 | 0.081 |

| | | | | |
|---|---|---|---|---|
| ENSP00000273783 | 6.02E-05 | -0.0347 | 485 | 0.389 |
| ENSP00000230538 | 7.64E-05 | -0.0347 | 242 | 0.464 |
| ENSP00000186436 | 1.05E-04 | -0.0349 | 266 | 0.216 |
| ENSP00000262477 | 5.08E-05 | -0.0351 | 336 | 0.000 |
| ENSP00000399968 | 7.69E-05 | -0.0352 | 900 | 0.825 |
| ENSP00000275162 | 7.99E-05 | -0.0353 | 361 | 0.520 |
| ENSP00000335612 | 2.90E-05 | -0.0353 | 153 | 0.640 |
| ENSP00000243213 | 1.18E-04 | -0.0353 | 0 | 0.112 |
| ENSP00000367123 | 3.74E-05 | -0.0353 | 420 | 0.744 |
| ENSP00000228027 | 5.26E-05 | -0.0354 | 343 | 0.580 |
| ENSP00000352936 | 3.92E-05 | -0.0354 | 670 | 0.647 |
| ENSP00000361867 | 3.50E-05 | -0.0354 | 254 | 0.344 |
| ENSP00000324742 | 1.20E-04 | -0.0355 | 304 | 0.792 |
| ENSP00000222598 | 3.94E-05 | -0.0355 | 297 | 0.883 |
| ENSP00000436786 | 3.75E-05 | -0.0355 | 281 | 0.000 |
| ENSP00000326305 | 8.98E-05 | -0.0355 | 260 | 0.376 |
| ENSP00000263598 | 1.23E-04 | -0.0355 | 215 | 0.254 |
| ENSP00000413572 | 4.36E-05 | -0.0356 | 325 | 0.752 |
| ENSP00000280187 | 7.18E-05 | -0.0356 | 162 | 0.084 |
| ENSP00000255078 | 2.01E-05 | -0.0356 | 157 | 0.764 |
| ENSP00000078445 | 2.13E-05 | -0.0357 | 472 | 0.528 |
| ENSP00000224140 | 4.71E-05 | -0.0358 | 384 | 0.762 |
| ENSP00000276202 | 9.25E-05 | -0.0359 | 214 | 0.267 |
| ENSP00000311905 | 2.71E-04 | -0.0359 | 216 | 0.382 |
| ENSP00000357130 | 3.99E-05 | -0.0360 | 999 | 0.000 |
| ENSP00000010404 | 6.51E-05 | -0.0360 | 198 | 0.552 |
| ENSP00000301776 | 1.31E-04 | -0.0360 | 249 | 0.110 |
| ENSP00000257915 | 3.03E-05 | -0.0360 | 808 | 0.767 |
| ENSP00000215904 | 2.64E-05 | -0.0361 | 359 | 0.493 |
| ENSP00000383900 | 3.14E-05 | -0.0361 | 390 | 0.323 |
| ENSP00000363779 | 4.39E-05 | -0.0362 | 284 | 0.687 |
| ENSP00000363193 | 2.20E-05 | -0.0363 | 254 | 0.790 |
| ENSP00000256398 | 2.84E-05 | -0.0363 | 311 | 0.819 |
| ENSP00000231420 | 3.71E-05 | -0.0364 | 160 | 0.083 |
| ENSP00000216252 | 3.67E-05 | -0.0365 | 302 | 0.690 |
| ENSP00000307607 | 6.96E-05 | -0.0365 | 193 | 0.187 |
| ENSP00000317674 | 2.39E-05 | -0.0365 | 465 | 0.466 |
| ENSP00000223095 | 5.36E-05 | -0.0365 | 980 | 0.979 |
| ENSP00000219070 | 4.89E-05 | -0.0366 | 998 | 0.991 |
| ENSP00000260570 | 5.50E-05 | -0.0367 | 201 | 0.287 |
| ENSP00000366081 | 1.29E-07 | -0.0367 | 0 | 0.000 |
| ENSP00000225964 | 7.68E-05 | -0.0368 | 957 | 0.970 |
| ENSP00000278742 | 7.23E-05 | -0.0369 | 750 | 0.432 |
| ENSP00000204679 | 9.20E-05 | -0.0371 | 374 | 0.276 |
| ENSP00000459028 | 3.51E-06 | -0.0371 | 0 | 0.157 |
| ENSP00000312987 | 9.59E-05 | -0.0371 | 723 | 0.889 |

| | | | | |
|---|---|---|---|---|
| ENSP00000221957 | 1.54E-04 | -0.0372 | 178 | 0.188 |
| ENSP00000263043 | 2.06E-05 | -0.0372 | 286 | 0.721 |
| ENSP00000306512 | 5.94E-05 | -0.0373 | 977 | 0.000 |
| ENSP00000221166 | 6.89E-05 | -0.0373 | 650 | 0.643 |
| ENSP00000338358 | 5.02E-05 | -0.0374 | 286 | 0.652 |
| ENSP00000301061 | 3.93E-05 | -0.0374 | 836 | 0.889 |
| ENSP00000373610 | 2.55E-05 | -0.0374 | 159 | 0.112 |
| ENSP00000363207 | 4.68E-05 | -0.0375 | 154 | 0.226 |
| ENSP00000318472 | 5.77E-05 | -0.0376 | 962 | 0.944 |
| ENSP00000328570 | 2.67E-05 | -0.0377 | 221 | 0.206 |
| ENSP00000273063 | 5.24E-05 | -0.0377 | 535 | 0.464 |
| ENSP00000357811 | 8.27E-06 | -0.0378 | 0 | 0.222 |
| ENSP00000240488 | 5.84E-05 | -0.0379 | 307 | 0.340 |
| ENSP00000262186 | 3.92E-05 | -0.0381 | 195 | 0.561 |
| ENSP00000261479 | 3.76E-05 | -0.0381 | 374 | 0.614 |
| ENSP00000262290 | 4.40E-05 | -0.0381 | 435 | 0.575 |
| ENSP00000354558 | 6.31E-05 | -0.0381 | 549 | 0.892 |
| ENSP00000327890 | 2.20E-05 | -0.0382 | 209 | 0.834 |
| ENSP00000297157 | 5.38E-05 | -0.0383 | 472 | 0.416 |
| ENSP00000281092 | 3.75E-05 | -0.0383 | 258 | 0.744 |
| ENSP00000282957 | 4.08E-05 | -0.0384 | 237 | 0.447 |
| ENSP00000311733 | 3.66E-05 | -0.0384 | 193 | 0.375 |
| ENSP00000258484 | 7.11E-05 | -0.0384 | 295 | 0.870 |
| ENSP00000381150 | 8.35E-06 | -0.0384 | 0 | 0.159 |
| ENSP00000318687 | 3.32E-05 | -0.0384 | 553 | 0.804 |
| ENSP00000276431 | 4.61E-05 | -0.0384 | 258 | 0.817 |
| ENSP00000346067 | 3.46E-05 | -0.0385 | 812 | 0.768 |
| ENSP00000278888 | 7.70E-05 | -0.0386 | 197 | 0.698 |
| ENSP00000381250 | 1.48E-05 | -0.0386 | 407 | 0.342 |
| ENSP00000311290 | 2.83E-05 | -0.0387 | 570 | 0.818 |
| ENSP00000253332 | 8.42E-05 | -0.0388 | 828 | 0.626 |
| ENSP00000368199 | 2.43E-05 | -0.0388 | 163 | 0.128 |
| ENSP00000293217 | 4.48E-05 | -0.0388 | 244 | 0.549 |
| ENSP00000294338 | 4.85E-05 | -0.0388 | 196 | 0.362 |
| ENSP00000233156 | 1.02E-04 | -0.0388 | 720 | 0.737 |
| ENSP00000310448 | 4.77E-05 | -0.0389 | 592 | 0.830 |
| ENSP00000269445 | 8.69E-05 | -0.0389 | 181 | 0.575 |
| ENSP00000253496 | 3.55E-05 | -0.0389 | 246 | 0.546 |
| ENSP00000365812 | 2.48E-05 | -0.0390 | 196 | 0.000 |
| ENSP00000346012 | 3.41E-05 | -0.0390 | 151 | 0.802 |
| ENSP00000304311 | 1.02E-04 | -0.0391 | 602 | 0.631 |
| ENSP00000274276 | 7.76E-05 | -0.0391 | 321 | 0.753 |
| ENSP00000306100 | 8.56E-05 | -0.0391 | 351 | 0.476 |
| ENSP00000261590 | 7.58E-05 | -0.0392 | 183 | 0.562 |
| ENSP00000263121 | 3.51E-05 | -0.0393 | 330 | 0.835 |
| ENSP00000366746 | 4.08E-05 | -0.0393 | 161 | 0.297 |

| | | | | |
|---|---|---|---|---|
| ENSP00000250971 | 2.62E-05 | -0.0394 | 926 | 0.000 |
| ENSP00000272430 | 8.29E-05 | -0.0394 | 181 | 0.743 |
| ENSP00000222254 | 8.13E-05 | -0.0394 | 193 | 0.813 |
| ENSP00000388910 | 1.94E-05 | -0.0394 | 815 | 0.856 |
| ENSP00000308720 | 6.07E-05 | -0.0395 | 389 | 0.402 |
| ENSP00000279242 | 3.51E-05 | -0.0395 | 345 | 0.342 |
| ENSP00000310226 | 3.33E-05 | -0.0396 | 404 | 0.243 |
| ENSP00000296474 | 3.58E-05 | -0.0396 | 199 | 0.901 |
| ENSP00000326737 | 2.05E-04 | -0.0396 | 239 | 0.417 |
| ENSP00000376150 | 3.85E-05 | -0.0396 | 165 | 0.178 |
| ENSP00000319814 | 4.57E-05 | -0.0397 | 357 | 0.670 |
| ENSP00000355537 | 4.34E-05 | -0.0397 | 915 | 0.643 |
| ENSP00000295688 | 5.08E-05 | -0.0397 | 786 | 0.757 |
| ENSP00000313875 | 1.10E-04 | -0.0397 | 227 | 0.561 |
| ENSP00000254037 | 2.15E-05 | -0.0397 | 297 | 0.920 |
| ENSP00000360266 | 8.72E-05 | -0.0398 | 996 | 0.000 |
| ENSP00000295704 | 1.83E-04 | -0.0398 | 165 | 0.225 |
| ENSP00000276198 | 2.56E-05 | -0.0398 | 175 | 0.622 |
| ENSP00000322956 | 5.80E-04 | -0.0399 | 900 | 0.224 |
| ENSP00000472724 | 1.65E-06 | -0.0399 | 0 | 0.000 |
| ENSP00000307077 | 1.34E-04 | -0.0400 | 452 | 0.353 |
| ENSP00000235382 | 3.07E-05 | -0.0400 | 221 | 0.795 |
| ENSP00000313021 | 4.38E-05 | -0.0400 | 167 | 0.426 |
| ENSP00000325506 | 5.60E-05 | -0.0400 | 361 | 0.514 |
| ENSP00000272771 | 8.12E-05 | -0.0401 | 309 | 0.512 |
| ENSP00000228476 | 3.09E-05 | -0.0401 | 209 | 0.321 |
| ENSP00000199708 | 7.10E-05 | -0.0401 | 211 | 0.182 |
| ENSP00000338934 | 7.13E-05 | -0.0401 | 935 | 0.874 |
| ENSP00000313921 | 7.48E-05 | -0.0401 | 234 | 0.133 |
| ENSP00000265131 | 9.75E-05 | -0.0403 | 915 | 0.957 |
| ENSP00000164227 | 2.26E-05 | -0.0403 | 220 | 0.861 |
| ENSP00000370288 | 3.48E-05 | -0.0403 | 264 | 0.000 |
| ENSP00000267842 | 7.32E-05 | -0.0403 | 329 | 0.509 |
| ENSP00000262262 | 5.89E-05 | -0.0403 | 284 | 0.729 |
| ENSP00000312134 | 2.35E-04 | -0.0403 | 150 | 0.111 |
| ENSP00000363827 | 8.66E-05 | -0.0404 | 989 | 0.917 |
| ENSP00000219473 | 3.28E-05 | -0.0405 | 319 | 0.728 |
| ENSP00000417404 | 4.08E-05 | -0.0405 | 570 | 0.826 |
| ENSP00000323901 | 4.83E-05 | -0.0405 | 219 | 0.933 |
| ENSP00000256592 | 3.31E-05 | -0.0406 | 163 | 0.656 |
| ENSP00000366095 | 1.14E-05 | -0.0406 | 0 | 0.100 |
| ENSP00000253719 | 5.90E-05 | -0.0406 | 314 | 0.142 |
| ENSP00000387123 | 3.43E-05 | -0.0407 | 224 | 0.268 |
| ENSP00000220764 | 2.57E-05 | -0.0407 | 929 | 0.882 |
| ENSP00000297261 | 6.48E-05 | -0.0407 | 514 | 0.000 |
| ENSP00000263087 | 4.02E-05 | -0.0408 | 242 | 0.883 |

| | | | | |
|---|---|---|---|---|
| ENSP00000349156 | 3.97E-05 | -0.0408 | 343 | 0.806 |
| ENSP00000263088 | 2.89E-05 | -0.0408 | 547 | 0.627 |
| ENSP00000316664 | 1.09E-04 | -0.0409 | 177 | 0.166 |
| ENSP00000354791 | 2.68E-05 | -0.0409 | 576 | 0.301 |
| ENSP00000293330 | 2.77E-05 | -0.0409 | 163 | 0.769 |
| ENSP00000233146 | 2.63E-05 | -0.0410 | 337 | 0.773 |
| ENSP00000215793 | 3.74E-05 | -0.0410 | 331 | 0.813 |
| ENSP00000263409 | 6.02E-05 | -0.0410 | 194 | 0.847 |
| ENSP00000450980 | 6.12E-05 | -0.0410 | 193 | 0.301 |
| ENSP00000379290 | 1.65E-05 | -0.0411 | 0 | 0.097 |
| ENSP00000226382 | 3.89E-05 | -0.0411 | 196 | 0.806 |
| ENSP00000309649 | 1.09E-04 | -0.0411 | 188 | 0.126 |
| ENSP00000264059 | 7.20E-05 | -0.0411 | 403 | 0.177 |
| ENSP00000365261 | 1.06E-06 | -0.0412 | 0 | 0.210 |
| ENSP00000362401 | 3.02E-05 | -0.0412 | 288 | 0.560 |
| ENSP00000384193 | 2.71E-05 | -0.0412 | 150 | 0.114 |
| ENSP00000302830 | 4.37E-05 | -0.0413 | 330 | 0.511 |
| ENSP00000245185 | 8.10E-05 | -0.0413 | 900 | 0.308 |
| ENSP00000368683 | 6.75E-05 | -0.0413 | 941 | 0.961 |
| ENSP00000466775 | 2.85E-05 | -0.0414 | 983 | 0.401 |
| ENSP00000290902 | 1.27E-04 | -0.0414 | 206 | 0.169 |
| ENSP00000062104 | 1.27E-04 | -0.0414 | 167 | 0.279 |
| ENSP00000342033 | 4.12E-05 | -0.0415 | 941 | 0.000 |
| ENSP00000415528 | 2.01E-07 | -0.0415 | 0 | 0.213 |
| ENSP00000362435 | 3.46E-04 | -0.0415 | 0 | 0.183 |
| ENSP00000288135 | 4.01E-05 | -0.0415 | 569 | 0.000 |
| ENSP00000263867 | 8.23E-05 | -0.0415 | 309 | 0.332 |
| ENSP00000270586 | 4.45E-05 | -0.0417 | 799 | 0.511 |
| ENSP00000284322 | 2.63E-04 | -0.0417 | 150 | 0.089 |
| ENSP00000265512 | 3.61E-05 | -0.0417 | 422 | 0.296 |
| ENSP00000262919 | 8.91E-05 | -0.0417 | 159 | 0.116 |
| ENSP00000309439 | 5.69E-05 | -0.0418 | 271 | 0.805 |
| ENSP00000299872 | 8.67E-05 | -0.0418 | 241 | 0.000 |
| ENSP00000166534 | 7.71E-05 | -0.0418 | 900 | 0.072 |
| ENSP00000281129 | 3.73E-04 | -0.0419 | 150 | 0.127 |
| ENSP00000368727 | 3.28E-05 | -0.0419 | 260 | 0.490 |
| ENSP00000330219 | 4.83E-05 | -0.0419 | 401 | 0.514 |
| ENSP00000353408 | 7.38E-05 | -0.0420 | 975 | 0.836 |
| ENSP00000264144 | 7.01E-05 | -0.0421 | 210 | 0.517 |
| ENSP00000387699 | 8.13E-05 | -0.0421 | 880 | 0.000 |
| ENSP00000264276 | 4.24E-05 | -0.0422 | 475 | 0.451 |
| ENSP00000226574 | 2.30E-05 | -0.0422 | 994 | 0.000 |
| ENSP00000366208 | 1.11E-04 | -0.0422 | 204 | 0.084 |
| ENSP00000292327 | 3.13E-05 | -0.0422 | 900 | 0.396 |
| ENSP00000279386 | 2.68E-05 | -0.0423 | 162 | 0.851 |
| ENSP00000310723 | 6.16E-05 | -0.0423 | 259 | 0.899 |

| | | | | |
|---|---|---|---|---|
| ENSP00000394352 | 5.12E-05 | -0.0423 | 483 | 0.085 |
| ENSP00000303634 | 7.02E-05 | -0.0424 | 999 | 0.738 |
| ENSP00000466933 | 2.14E-05 | -0.0424 | 808 | 0.669 |
| ENSP00000445175 | 5.60E-05 | -0.0424 | 476 | 0.763 |
| ENSP00000308887 | 3.39E-05 | -0.0424 | 614 | 0.932 |
| ENSP00000304501 | 5.25E-05 | -0.0425 | 313 | 0.716 |
| ENSP00000361915 | 2.60E-05 | -0.0425 | 212 | 0.886 |
| ENSP00000338082 | 5.61E-05 | -0.0425 | 315 | 0.661 |
| ENSP00000463533 | 2.14E-05 | -0.0426 | 186 | 0.147 |
| ENSP00000357040 | 3.46E-05 | -0.0426 | 475 | 0.881 |
| ENSP00000257552 | 7.07E-05 | -0.0426 | 335 | 0.645 |
| ENSP00000363079 | 2.59E-05 | -0.0426 | 196 | 0.580 |
| ENSP00000316674 | 2.95E-05 | -0.0426 | 449 | 0.689 |
| ENSP00000327323 | 4.91E-05 | -0.0428 | 294 | 0.483 |
| ENSP00000331211 | 1.32E-04 | -0.0428 | 479 | 0.826 |
| ENSP00000463047 | 9.98E-07 | -0.0428 | 0 | 0.000 |
| ENSP00000229135 | 2.72E-05 | -0.0428 | 313 | 0.000 |
| ENSP00000322706 | 4.74E-05 | -0.0429 | 436 | 0.573 |
| ENSP00000263640 | 3.83E-05 | -0.0429 | 230 | 0.878 |
| ENSP00000326519 | 5.29E-05 | -0.0429 | 227 | 0.697 |
| ENSP00000256379 | 5.48E-05 | -0.0430 | 317 | 0.800 |
| ENSP00000342307 | 6.86E-05 | -0.0431 | 851 | 0.000 |
| ENSP00000351209 | 5.42E-05 | -0.0431 | 390 | 0.665 |
| ENSP00000218340 | 1.32E-04 | -0.0431 | 303 | 0.198 |
| ENSP00000259512 | 4.09E-05 | -0.0431 | 600 | 0.553 |
| ENSP00000332979 | 5.36E-05 | -0.0432 | 356 | 0.222 |
| ENSP00000307252 | 4.89E-05 | -0.0432 | 330 | 0.192 |
| ENSP00000331831 | 1.38E-04 | -0.0433 | 916 | 0.671 |
| ENSP00000224356 | 5.05E-05 | -0.0433 | 652 | 0.671 |
| ENSP00000375977 | 3.20E-05 | -0.0433 | 823 | 0.867 |
| ENSP00000265970 | 2.66E-05 | -0.0433 | 261 | 0.760 |
| ENSP00000315417 | 2.77E-05 | -0.0434 | 155 | 0.720 |
| ENSP00000264663 | 2.77E-05 | -0.0435 | 464 | 0.439 |
| ENSP00000299353 | 1.87E-04 | -0.0435 | 200 | 0.000 |
| ENSP00000310170 | 3.14E-05 | -0.0435 | 993 | 0.000 |
| ENSP00000256194 | 4.82E-05 | -0.0435 | 387 | 0.459 |
| ENSP00000451258 | 8.19E-08 | -0.0435 | 0 | 0.000 |
| ENSP00000330382 | 6.88E-05 | -0.0436 | 889 | 0.969 |
| ENSP00000283882 | 3.59E-05 | -0.0437 | 344 | 0.861 |
| ENSP00000252530 | 8.00E-05 | -0.0437 | 181 | 0.143 |
| ENSP00000176183 | 2.76E-05 | -0.0437 | 330 | 0.684 |
| ENSP00000278937 | 1.09E-04 | -0.0437 | 309 | 0.154 |
| ENSP00000362153 | 2.50E-05 | -0.0438 | 267 | 0.893 |
| ENSP00000326095 | 1.17E-04 | -0.0438 | 157 | 0.128 |
| ENSP00000301046 | 2.46E-04 | -0.0439 | 576 | 0.085 |
| ENSP00000241337 | 9.04E-05 | -0.0440 | 803 | 0.263 |

| | | | | |
|---|---|---|---|---|
| ENSP00000257724 | 3.59E-05 | -0.0441 | 165 | 0.434 |
| ENSP00000248553 | 8.46E-05 | -0.0441 | 875 | 0.910 |
| ENSP00000302564 | 5.38E-05 | -0.0442 | 880 | 0.945 |
| ENSP00000400101 | 7.91E-05 | -0.0442 | 201 | 0.066 |
| ENSP00000396211 | 3.35E-05 | -0.0443 | 227 | 0.000 |
| ENSP00000246554 | 4.26E-05 | -0.0443 | 416 | 0.378 |
| ENSP00000366413 | 2.58E-05 | -0.0443 | 357 | 0.813 |
| ENSP00000304192 | 1.35E-04 | -0.0443 | 554 | 0.141 |
| ENSP00000220966 | 3.33E-05 | -0.0443 | 908 | 0.301 |
| ENSP00000227507 | 3.00E-05 | -0.0444 | 860 | 0.000 |
| ENSP00000268182 | 3.67E-05 | -0.0444 | 645 | 0.704 |
| ENSP00000329647 | 2.98E-05 | -0.0444 | 449 | 0.693 |
| ENSP00000293288 | 6.38E-05 | -0.0444 | 988 | 0.907 |
| ENSP00000258080 | 2.61E-05 | -0.0445 | 636 | 0.699 |
| ENSP00000282928 | 3.04E-05 | -0.0445 | 396 | 0.867 |
| ENSP00000470694 | 2.50E-06 | -0.0445 | 0 | 0.000 |
| ENSP00000381949 | 2.45E-05 | -0.0445 | 591 | 0.180 |
| ENSP00000265565 | 8.83E-05 | -0.0446 | 489 | 0.551 |
| ENSP00000311121 | 4.54E-05 | -0.0446 | 644 | 0.383 |
| ENSP00000262962 | 4.72E-05 | -0.0446 | 304 | 0.408 |
| ENSP00000296955 | 1.52E-04 | -0.0446 | 150 | 0.110 |
| ENSP00000248594 | 3.42E-05 | -0.0446 | 380 | 0.765 |
| ENSP00000264434 | 3.13E-03 | -0.0447 | 0 | 0.207 |
| ENSP00000309259 | 3.13E-03 | -0.0447 | 256 | 0.605 |
| ENSP00000459439 | 2.14E-07 | -0.0447 | 0 | 0.000 |
| ENSP00000347792 | 2.83E-05 | -0.0447 | 414 | 0.384 |
| ENSP00000237500 | 2.70E-05 | -0.0447 | 291 | 0.544 |
| ENSP00000306477 | 5.69E-05 | -0.0448 | 168 | 0.140 |
| ENSP00000285848 | 2.62E-05 | -0.0448 | 457 | 0.401 |
| ENSP00000327145 | 3.39E-05 | -0.0448 | 406 | 0.580 |
| ENSP00000447679 | 9.80E-06 | -0.0449 | 242 | 0.000 |
| ENSP00000299964 | 6.90E-05 | -0.0449 | 256 | 0.258 |
| ENSP00000280614 | 3.21E-05 | -0.0449 | 213 | 0.000 |
| ENSP00000303452 | 2.36E-05 | -0.0449 | 459 | 0.812 |
| ENSP00000340507 | 4.58E-05 | -0.0450 | 313 | 0.836 |
| ENSP00000346634 | 4.45E-05 | -0.0450 | 156 | 0.689 |
| ENSP00000284292 | 6.31E-05 | -0.0450 | 223 | 0.489 |
| ENSP00000321070 | 3.58E-05 | -0.0450 | 216 | 0.575 |
| ENSP00000303129 | 6.36E-05 | -0.0450 | 218 | 0.109 |
| ENSP00000216294 | 1.27E-04 | -0.0451 | 160 | 0.481 |
| ENSP00000295962 | 7.59E-05 | -0.0451 | 212 | 0.053 |
| ENSP00000242067 | 3.67E-05 | -0.0451 | 196 | 0.200 |
| ENSP00000265112 | 2.48E-05 | -0.0451 | 424 | 0.733 |
| ENSP00000296641 | 5.61E-05 | -0.0451 | 281 | 0.564 |
| ENSP00000261172 | 3.43E-05 | -0.0452 | 263 | 0.770 |
| ENSP00000332116 | 2.99E-05 | -0.0452 | 511 | 0.660 |

| | | | | |
|---|---|---|---|---|
| ENSP00000360158 | 3.87E-05 | -0.0453 | 270 | 0.620 |
| ENSP00000205402 | 1.70E-05 | -0.0453 | 369 | 0.550 |
| ENSP00000307634 | 2.60E-05 | -0.0454 | 154 | 0.208 |
| ENSP00000420588 | 6.80E-05 | -0.0454 | 299 | 0.856 |
| ENSP00000262613 | 3.21E-05 | -0.0455 | 922 | 0.665 |
| ENSP00000332816 | 3.12E-05 | -0.0455 | 925 | 0.879 |
| ENSP00000365725 | 4.00E-05 | -0.0455 | 154 | 0.071 |
| ENSP00000304854 | 4.94E-05 | -0.0455 | 364 | 0.205 |
| ENSP00000311502 | 8.13E-05 | -0.0456 | 330 | 0.749 |
| ENSP00000394863 | 4.32E-05 | -0.0456 | 329 | 0.417 |
| ENSP00000294973 | 3.72E-05 | -0.0457 | 195 | 0.302 |
| ENSP00000369963 | 1.02E-03 | -0.0457 | 0 | 0.200 |
| ENSP00000261503 | 4.58E-05 | -0.0457 | 338 | 0.457 |
| ENSP00000206542 | 3.63E-05 | -0.0457 | 290 | 0.323 |
| ENSP00000370031 | 4.22E-05 | -0.0458 | 282 | 0.315 |
| ENSP00000261416 | 4.48E-05 | -0.0458 | 345 | 0.203 |
| ENSP00000322396 | 4.13E-05 | -0.0458 | 252 | 0.887 |
| ENSP00000346032 | 9.83E-05 | -0.0458 | 421 | 0.860 |
| ENSP00000258884 | 1.23E-04 | -0.0458 | 206 | 0.096 |
| ENSP00000288266 | 4.74E-05 | -0.0458 | 259 | 0.943 |
| ENSP00000297307 | 3.70E-05 | -0.0459 | 0 | 0.189 |
| ENSP00000386439 | 1.86E-05 | -0.0459 | 208 | 0.133 |
| ENSP00000371724 | 1.48E-03 | -0.0459 | 0 | 0.141 |
| ENSP00000296452 | 4.44E-05 | -0.0459 | 396 | 0.432 |
| ENSP00000263997 | 1.05E-04 | -0.0460 | 173 | 0.306 |
| ENSP00000242108 | 9.79E-05 | -0.0460 | 191 | 0.076 |
| ENSP00000271450 | 3.29E-05 | -0.0460 | 282 | 0.792 |
| ENSP00000265857 | 3.35E-05 | -0.0460 | 294 | 0.424 |
| ENSP00000377833 | 5.07E-05 | -0.0460 | 439 | 0.426 |
| ENSP00000286201 | 5.21E-05 | -0.0461 | 178 | 0.901 |
| ENSP00000245479 | 5.47E-05 | -0.0461 | 874 | 0.886 |
| ENSP00000265849 | 2.30E-05 | -0.0462 | 357 | 0.719 |
| ENSP00000316950 | 6.71E-05 | -0.0462 | 347 | 0.863 |
| ENSP00000259455 | 2.39E-05 | -0.0462 | 370 | 0.500 |
| ENSP00000266079 | 2.92E-05 | -0.0462 | 316 | 0.829 |
| ENSP00000331040 | 2.10E-05 | -0.0462 | 539 | 0.872 |
| ENSP00000367830 | 2.97E-05 | -0.0462 | 900 | 0.863 |
| ENSP00000363512 | 6.13E-05 | -0.0462 | 477 | 0.788 |
| ENSP00000343115 | 1.96E-04 | -0.0463 | 0 | 0.213 |
| ENSP00000379003 | 2.65E-05 | -0.0464 | 189 | 0.113 |
| ENSP00000256594 | 8.81E-05 | -0.0465 | 151 | 0.281 |
| ENSP00000219255 | 2.70E-05 | -0.0465 | 206 | 0.832 |
| ENSP00000233575 | 1.33E-04 | -0.0465 | 440 | 0.124 |
| ENSP00000292211 | 1.20E-04 | -0.0465 | 193 | 0.206 |
| ENSP00000253063 | 7.34E-05 | -0.0466 | 157 | 0.567 |
| ENSP00000356311 | 4.69E-05 | -0.0466 | 152 | 0.087 |

| | | | | |
|---|---|---|---|---|
| ENSP00000265724 | 2.79E-05 | -0.0466 | 323 | 0.821 |
| ENSP00000247226 | 3.06E-04 | -0.0466 | 505 | 0.216 |
| ENSP00000304736 | 3.53E-05 | -0.0467 | 241 | 0.516 |
| ENSP00000247153 | 1.59E-04 | -0.0467 | 361 | 0.588 |
| ENSP00000281631 | 2.85E-04 | -0.0467 | 351 | 0.116 |
| ENSP00000329662 | 6.40E-05 | -0.0467 | 479 | 0.878 |
| ENSP00000005178 | 3.00E-05 | -0.0467 | 267 | 0.589 |
| ENSP00000343645 | 3.86E-05 | -0.0467 | 499 | 0.407 |
| ENSP00000265641 | 3.29E-05 | -0.0467 | 301 | 0.594 |
| ENSP00000302051 | 5.21E-05 | -0.0468 | 297 | 0.219 |
| ENSP00000267229 | 8.69E-05 | -0.0468 | 217 | 0.304 |
| ENSP00000363753 | 3.38E-05 | -0.0468 | 212 | 0.085 |
| ENSP00000298942 | 3.62E-05 | -0.0468 | 256 | 0.850 |
| ENSP00000258105 | 2.68E-04 | -0.0468 | 644 | 0.380 |
| ENSP00000363799 | 3.76E-05 | -0.0468 | 449 | 0.691 |
| ENSP00000255266 | 1.73E-05 | -0.0468 | 632 | 0.649 |
| ENSP00000272163 | 2.76E-05 | -0.0469 | 217 | 0.571 |
| ENSP00000236273 | 4.30E-05 | -0.0470 | 431 | 0.475 |
| ENSP00000264637 | 4.56E-05 | -0.0470 | 185 | 0.850 |
| ENSP00000262507 | 7.44E-05 | -0.0470 | 221 | 0.054 |
| ENSP00000256644 | 1.08E-04 | -0.0470 | 656 | 0.433 |
| ENSP00000281537 | 6.18E-05 | -0.0470 | 854 | 0.885 |
| ENSP00000415786 | 6.19E-05 | -0.0471 | 838 | 0.875 |
| ENSP00000318374 | 1.21E-05 | -0.0472 | 0 | 0.194 |
| ENSP00000219439 | 7.73E-05 | -0.0472 | 150 | 0.179 |
| ENSP00000368831 | 7.23E-05 | -0.0472 | 0 | 0.152 |
| ENSP00000299198 | 5.87E-05 | -0.0472 | 383 | 0.305 |
| ENSP00000382026 | 3.14E-07 | -0.0472 | 0 | 0.195 |
| ENSP00000436337 | 3.21E-05 | -0.0472 | 338 | 0.000 |
| ENSP00000457962 | 5.82E-07 | -0.0473 | 0 | 0.206 |
| ENSP00000220514 | 7.16E-05 | -0.0473 | 194 | 0.395 |
| ENSP00000296930 | 6.40E-05 | -0.0473 | 812 | 0.818 |
| ENSP00000297469 | 4.50E-05 | -0.0474 | 284 | 0.000 |
| ENSP00000415299 | 2.58E-05 | -0.0474 | 0 | 0.207 |
| ENSP00000244534 | 6.30E-05 | -0.0474 | 525 | 0.855 |
| ENSP00000259119 | 4.60E-05 | -0.0475 | 326 | 0.823 |
| ENSP00000085219 | 3.78E-05 | -0.0475 | 357 | 0.797 |
| ENSP00000221930 | 2.47E-05 | -0.0476 | 993 | 0.000 |
| ENSP00000272286 | 3.25E-05 | -0.0476 | 634 | 0.510 |
| ENSP00000284719 | 2.72E-05 | -0.0476 | 334 | 0.718 |
| ENSP00000222792 | 1.80E-05 | -0.0476 | 329 | 0.464 |
| ENSP00000321999 | 4.20E-05 | -0.0477 | 374 | 0.738 |
| ENSP00000371968 | 4.39E-02 | -0.0477 | 0 | 0.000 |
| ENSP00000265968 | 5.69E-05 | -0.0477 | 576 | 0.475 |
| ENSP00000258418 | 4.22E-05 | -0.0477 | 588 | 0.461 |
| ENSP00000262188 | 3.96E-05 | -0.0478 | 900 | 0.841 |

| | | | | |
|---|---|---|---|---|
| ENSP00000365435 | 3.67E-05 | -0.0478 | 361 | 0.883 |
| ENSP00000259457 | 3.25E-05 | -0.0478 | 223 | 0.522 |
| ENSP00000262990 | 8.46E-05 | -0.0478 | 232 | 0.142 |
| ENSP00000367894 | 2.13E-05 | -0.0478 | 0 | 0.123 |
| ENSP00000351157 | 3.44E-04 | -0.0479 | 0 | 0.188 |
| ENSP00000357660 | 9.52E-05 | -0.0479 | 0 | 0.161 |
| ENSP00000390750 | 1.18E-05 | -0.0479 | 167 | 0.139 |
| ENSP00000217159 | 7.30E-05 | -0.0479 | 166 | 0.072 |
| ENSP00000366376 | 1.32E-06 | -0.0480 | 0 | 0.216 |
| ENSP00000344504 | 7.06E-05 | -0.0480 | 479 | 0.805 |
| ENSP00000370391 | 8.22E-05 | -0.0480 | 0 | 0.187 |
| ENSP00000343764 | 5.18E-05 | -0.0480 | 915 | 0.684 |
| ENSP00000283905 | 6.77E-06 | -0.0481 | 0 | 0.214 |
| ENSP00000265729 | 3.00E-05 | -0.0481 | 485 | 0.281 |
| ENSP00000235372 | 4.97E-05 | -0.0481 | 150 | 0.809 |
| ENSP00000304669 | 4.15E-05 | -0.0482 | 520 | 0.828 |
| ENSP00000277657 | 3.50E-04 | -0.0482 | 0 | 0.155 |
| ENSP00000358865 | 3.18E-05 | -0.0482 | 363 | 0.302 |
| ENSP00000361850 | 9.75E-05 | -0.0482 | 987 | 0.925 |
| ENSP00000261208 | 3.80E-05 | -0.0482 | 284 | 0.378 |
| ENSP00000295589 | 2.12E-05 | -0.0482 | 152 | 0.605 |
| ENSP00000263688 | 5.47E-05 | -0.0482 | 204 | 0.098 |
| ENSP00000296412 | 2.98E-05 | -0.0483 | 305 | 0.322 |
| ENSP00000379140 | 7.25E-05 | -0.0483 | 942 | 0.967 |
| ENSP00000265371 | 1.42E-04 | -0.0483 | 634 | 0.913 |
| ENSP00000240851 | 6.20E-05 | -0.0483 | 334 | 0.724 |
| ENSP00000327916 | 3.64E-05 | -0.0483 | 183 | 0.348 |
| ENSP00000220584 | 3.99E-05 | -0.0483 | 357 | 0.504 |
| ENSP00000185150 | 4.94E-05 | -0.0484 | 893 | 0.292 |
| ENSP00000276033 | 6.21E-05 | -0.0484 | 181 | 0.000 |
| ENSP00000380467 | 6.62E-05 | -0.0485 | 0 | 0.100 |
| ENSP00000332118 | 3.14E-05 | -0.0485 | 216 | 0.672 |
| ENSP00000268124 | 3.00E-05 | -0.0485 | 192 | 0.561 |
| ENSP00000307705 | 7.46E-05 | -0.0486 | 535 | 0.888 |
| ENSP00000366336 | 1.90E-07 | -0.0486 | 0 | 0.228 |
| ENSP00000254043 | 5.46E-05 | -0.0486 | 153 | 0.562 |
| ENSP00000254722 | 1.49E-04 | -0.0486 | 539 | 0.893 |
| ENSP00000268719 | 7.50E-05 | -0.0486 | 150 | 0.090 |
| ENSP00000340937 | 4.88E-05 | -0.0486 | 356 | 0.661 |
| ENSP00000373136 | 1.90E-07 | -0.0486 | 0 | 0.228 |
| ENSP00000230050 | 2.72E-05 | -0.0487 | 213 | 0.905 |
| ENSP00000369840 | 1.61E-05 | -0.0488 | 0 | 0.196 |
| ENSP00000217971 | 3.65E-05 | -0.0488 | 216 | 0.250 |
| ENSP00000318884 | 2.77E-05 | -0.0488 | 196 | 0.459 |
| ENSP00000265276 | 4.79E-05 | -0.0488 | 270 | 0.577 |
| ENSP00000209884 | 6.52E-05 | -0.0489 | 551 | 0.592 |

| | | | | |
|---|---|---|---|---|
| ENSP00000314897 | 1.48E-04 | -0.0489 | 581 | 0.967 |
| ENSP00000250101 | 1.32E-04 | -0.0489 | 165 | 0.090 |
| ENSP00000302918 | 2.06E-04 | -0.0490 | 414 | 0.148 |
| ENSP00000251607 | 3.86E-05 | -0.0490 | 814 | 0.676 |
| ENSP00000386394 | 3.51E-05 | -0.0490 | 514 | 0.131 |
| ENSP00000318094 | 4.56E-05 | -0.0491 | 234 | 0.756 |
| ENSP00000371872 | 2.31E-05 | -0.0491 | 300 | 0.399 |
| ENSP00000259874 | 1.23E-04 | -0.0491 | 343 | 0.426 |
| ENSP00000325526 | 3.16E-05 | -0.0491 | 698 | 0.927 |
| ENSP00000330720 | 4.06E-05 | -0.0491 | 0 | 0.169 |
| ENSP00000344967 | 3.21E-05 | -0.0492 | 216 | 0.524 |
| ENSP00000297494 | 4.66E-05 | -0.0492 | 943 | 0.961 |
| ENSP00000046794 | 4.29E-05 | -0.0492 | 329 | 0.853 |
| ENSP00000309334 | 2.94E-05 | -0.0492 | 214 | 0.860 |
| ENSP00000055077 | 1.88E-05 | -0.0492 | 259 | 0.719 |
| ENSP00000058691 | 1.91E-04 | -0.0493 | 182 | 0.000 |
| ENSP00000222115 | 2.61E-05 | -0.0493 | 266 | 0.657 |
| ENSP00000362457 | 3.54E-05 | -0.0493 | 0 | 0.194 |
| ENSP00000371554 | 2.79E-05 | -0.0494 | 418 | 0.634 |
| ENSP00000366326 | 2.75E-05 | -0.0494 | 150 | 0.330 |
| ENSP00000225171 | 3.69E-05 | -0.0494 | 761 | 0.172 |
| ENSP00000386896 | 7.80E-05 | -0.0494 | 864 | 0.934 |
| ENSP00000270233 | 7.62E-05 | -0.0494 | 408 | 0.264 |
| ENSP00000262160 | 5.17E-05 | -0.0495 | 942 | 0.917 |
| ENSP00000222219 | 4.88E-05 | -0.0495 | 500 | 0.442 |
| ENSP00000370223 | 2.97E-05 | -0.0495 | 389 | 0.476 |
| ENSP00000282251 | 3.10E-05 | -0.0495 | 234 | 0.380 |
| ENSP00000357304 | 4.91E-05 | -0.0495 | 0 | 0.149 |
| ENSP00000275493 | 3.92E-05 | -0.0495 | 952 | 0.000 |
| ENSP00000451040 | 5.99E-05 | -0.0495 | 909 | 0.916 |
| ENSP00000431512 | 5.44E-05 | -0.0496 | 854 | 0.000 |
| ENSP00000290299 | 2.69E-05 | -0.0496 | 399 | 0.515 |
| ENSP00000327716 | 5.53E-05 | -0.0496 | 179 | 0.294 |
| ENSP00000256319 | 7.42E-05 | -0.0496 | 151 | 0.066 |
| ENSP00000300056 | 4.52E-05 | -0.0496 | 169 | 0.320 |
| ENSP00000296871 | 5.52E-05 | -0.0497 | 582 | 0.960 |
| ENSP00000279488 | 3.72E-05 | -0.0497 | 311 | 0.894 |
| ENSP00000235332 | 6.79E-05 | -0.0497 | 0 | 0.103 |
| ENSP00000264202 | 3.95E-05 | -0.0497 | 474 | 0.501 |
| ENSP00000284262 | 2.59E-05 | -0.0497 | 196 | 0.496 |
| ENSP00000263092 | 1.13E-04 | -0.0497 | 157 | 0.267 |
| ENSP00000216117 | 5.19E-05 | -0.0497 | 912 | 0.949 |
| ENSP00000307843 | 6.93E-05 | -0.0497 | 241 | 0.465 |
| ENSP00000403270 | 2.35E-05 | -0.0498 | 154 | 0.000 |
| ENSP00000350012 | 3.58E-05 | -0.0498 | 194 | 0.502 |
| ENSP00000170564 | 1.15E-04 | -0.0499 | 186 | 0.430 |

| | | | | |
|---|---|---|---|---|
| ENSP00000314036 | 3.07E-05 | -0.0499 | 156 | 0.297 |
| ENSP00000302967 | 4.75E-05 | -0.0499 | 465 | 0.000 |
| ENSP00000260643 | 3.88E-05 | -0.0499 | 343 | 0.408 |
| ENSP00000327513 | 5.12E-05 | -0.0499 | 354 | 0.924 |
| ENSP00000346127 | 2.98E-05 | -0.0500 | 0 | 0.165 |
| ENSP00000222145 | 2.79E-05 | -0.0500 | 250 | 0.367 |
| ENSP00000361669 | 2.54E-05 | -0.0500 | 270 | 0.762 |
| ENSP00000244043 | 5.41E-05 | -0.0500 | 264 | 0.364 |
| ENSP00000244573 | 5.39E-05 | -0.0500 | 787 | 0.852 |
| ENSP00000254958 | 6.49E-05 | -0.0500 | 392 | 0.956 |
| ENSP00000380413 | 2.27E-05 | -0.0500 | 154 | 0.169 |
| ENSP00000265437 | 1.07E-04 | -0.0501 | 177 | 0.087 |
| ENSP00000327336 | 4.03E-05 | -0.0501 | 242 | 0.740 |
| ENSP00000248420 | 6.83E-05 | -0.0501 | 202 | 0.412 |
| ENSP00000296233 | 3.28E-05 | -0.0501 | 841 | 0.795 |
| ENSP00000228811 | 4.64E-04 | -0.0502 | 206 | 0.126 |
| ENSP00000253792 | 1.78E-05 | -0.0502 | 672 | 0.685 |
| ENSP00000339511 | 3.71E-05 | -0.0502 | 0 | 0.000 |
| ENSP00000265271 | 9.16E-05 | -0.0502 | 974 | 0.256 |
| ENSP00000340660 | 4.66E-05 | -0.0502 | 444 | 0.708 |
| ENSP00000276651 | 2.98E-05 | -0.0502 | 178 | 0.455 |
| ENSP00000391121 | 3.00E-05 | -0.0503 | 391 | 0.181 |
| ENSP00000301149 | 3.41E-05 | -0.0503 | 167 | 0.416 |
| ENSP00000291532 | 4.34E-05 | -0.0503 | 288 | 0.095 |
| ENSP00000261168 | 1.56E-05 | -0.0503 | 0 | 0.602 |
| ENSP00000056217 | 3.49E-05 | -0.0503 | 199 | 0.684 |
| ENSP00000296805 | 2.35E-05 | -0.0503 | 403 | 0.832 |
| ENSP00000307208 | 2.26E-05 | -0.0504 | 246 | 0.899 |
| ENSP00000363089 | 6.72E-05 | -0.0504 | 913 | 0.000 |
| ENSP00000292174 | 3.93E-05 | -0.0504 | 270 | 0.667 |
| ENSP00000359534 | 1.84E-08 | -0.0505 | 0 | 0.214 |
| ENSP00000358861 | 3.44E-05 | -0.0505 | 309 | 0.432 |
| ENSP00000312506 | 4.75E-05 | -0.0505 | 168 | 0.399 |
| ENSP00000359119 | 2.34E-05 | -0.0506 | 440 | 0.607 |
| ENSP00000247843 | 2.71E-05 | -0.0506 | 644 | 0.913 |
| ENSP00000249042 | 2.28E-05 | -0.0506 | 392 | 0.365 |
| ENSP00000337701 | 4.14E-05 | -0.0506 | 235 | 0.574 |
| ENSP00000219150 | 2.37E-05 | -0.0507 | 357 | 0.425 |
| ENSP00000238714 | 4.49E-05 | -0.0507 | 168 | 0.804 |
| ENSP00000228468 | 2.57E-05 | -0.0507 | 784 | 0.369 |
| ENSP00000438757 | 1.42E-08 | -0.0507 | 0 | 0.215 |
| ENSP00000341214 | 4.95E-05 | -0.0508 | 479 | 0.831 |
| ENSP00000362978 | 2.47E-05 | -0.0509 | 0 | 0.483 |
| ENSP00000314879 | 7.01E-05 | -0.0509 | 165 | 0.161 |
| ENSP00000229195 | 2.83E-05 | -0.0509 | 286 | 0.532 |
| ENSP00000364720 | 4.40E-08 | -0.0509 | 0 | 0.000 |

| | | | | |
|---|---|---|---|---|
| ENSP00000264126 | 4.93E-05 | -0.0509 | 802 | 0.246 |
| ENSP00000235329 | 2.34E-05 | -0.0509 | 293 | 0.450 |
| ENSP00000216911 | 2.04E-05 | -0.0509 | 433 | 0.647 |
| ENSP00000296632 | 8.13E-05 | -0.0509 | 239 | 0.341 |
| ENSP00000222823 | 3.64E-05 | -0.0510 | 196 | 0.789 |
| ENSP00000292896 | 3.24E-05 | -0.0510 | 228 | 0.488 |
| ENSP00000383873 | 1.65E-08 | -0.0510 | 0 | 0.207 |
| ENSP00000323696 | 1.51E-04 | -0.0511 | 168 | 0.127 |
| ENSP00000317790 | 4.32E-05 | -0.0511 | 900 | 0.764 |
| ENSP00000053243 | 3.46E-05 | -0.0511 | 196 | 0.690 |
| ENSP00000262890 | 7.09E-05 | -0.0511 | 153 | 0.084 |
| ENSP00000296577 | 2.65E-05 | -0.0512 | 242 | 0.786 |
| ENSP00000307850 | 6.88E-05 | -0.0512 | 155 | 0.789 |
| ENSP00000360031 | 2.93E-05 | -0.0512 | 275 | 0.902 |
| ENSP00000382982 | 4.09E-05 | -0.0513 | 250 | 0.546 |
| ENSP00000351244 | 2.17E-05 | -0.0513 | 198 | 0.137 |
| ENSP00000277010 | 5.17E-05 | -0.0513 | 232 | 0.142 |
| ENSP00000288350 | 3.62E-05 | -0.0513 | 0 | 0.105 |
| ENSP00000309572 | 3.10E-05 | -0.0513 | 349 | 0.863 |
| ENSP00000284061 | 4.40E-05 | -0.0513 | 280 | 0.102 |
| ENSP00000312370 | 3.46E-05 | -0.0513 | 200 | 0.368 |
| ENSP00000378710 | 2.01E-05 | -0.0514 | 0 | 0.110 |
| ENSP00000454370 | 1.02E-06 | -0.0514 | 0 | 0.184 |
| ENSP00000269980 | 2.71E-05 | -0.0514 | 804 | 0.602 |
| ENSP00000400803 | 1.98E-05 | -0.0515 | 500 | 0.671 |
| ENSP00000261879 | 7.59E-05 | -0.0515 | 270 | 0.532 |
| ENSP00000239461 | 1.78E-05 | -0.0515 | 356 | 0.819 |
| ENSP00000217260 | 2.17E-05 | -0.0515 | 302 | 0.279 |
| ENSP00000349244 | 2.75E-05 | -0.0515 | 0 | 0.000 |
| ENSP00000323805 | 3.65E-05 | -0.0516 | 0 | 0.124 |
| ENSP00000294952 | 2.47E-05 | -0.0516 | 0 | 0.109 |
| ENSP00000225842 | 3.41E-04 | -0.0516 | 658 | 0.403 |
| ENSP00000322788 | 8.35E-05 | -0.0517 | 997 | 0.979 |
| ENSP00000242261 | 6.16E-05 | -0.0517 | 841 | 0.901 |
| ENSP00000269159 | 3.91E-05 | -0.0517 | 195 | 0.219 |
| ENSP00000168712 | 5.83E-05 | -0.0517 | 366 | 0.923 |
| ENSP00000257861 | 4.46E-05 | -0.0517 | 239 | 0.546 |
| ENSP00000343619 | 3.96E-05 | -0.0517 | 328 | 0.845 |
| ENSP00000303366 | 8.07E-05 | -0.0517 | 830 | 0.102 |
| ENSP00000285407 | 3.27E-05 | -0.0518 | 309 | 0.827 |
| ENSP00000342557 | 2.81E-05 | -0.0518 | 328 | 0.342 |
| ENSP00000328729 | 7.91E-05 | -0.0518 | 165 | 0.000 |
| ENSP00000324842 | 3.49E-05 | -0.0518 | 191 | 0.346 |
| ENSP00000337439 | 4.81E-05 | -0.0518 | 183 | 0.790 |
| ENSP00000371829 | 9.78E-03 | -0.0519 | 0 | 0.145 |
| ENSP00000266719 | 3.25E-05 | -0.0519 | 910 | 0.804 |

| | | | | |
|---|---|---|---|---|
| ENSP00000238112 | 3.08E-05 | -0.0519 | 225 | 0.824 |
| ENSP00000187397 | 3.26E-05 | -0.0519 | 207 | 0.366 |
| ENSP00000228741 | 1.23E-04 | -0.0519 | 151 | 0.538 |
| ENSP00000216392 | 2.96E-05 | -0.0519 | 173 | 0.428 |
| ENSP00000234961 | 4.51E-05 | -0.0520 | 196 | 0.656 |
| ENSP00000375562 | 1.67E-08 | -0.0520 | 0 | 0.000 |
| ENSP00000368572 | 5.81E-05 | -0.0520 | 256 | 0.507 |
| ENSP00000298316 | 3.54E-05 | -0.0520 | 905 | 0.755 |
| ENSP00000216367 | 4.70E-05 | -0.0520 | 223 | 0.667 |
| ENSP00000263635 | 2.29E-05 | -0.0520 | 284 | 0.875 |
| ENSP00000222547 | 2.91E-05 | -0.0520 | 172 | 0.239 |
| ENSP00000291009 | 7.94E-05 | -0.0521 | 284 | 0.260 |
| ENSP00000335605 | 1.08E-04 | -0.0521 | 190 | 0.181 |
| ENSP00000312625 | 5.44E-05 | -0.0521 | 479 | 0.796 |
| ENSP00000386186 | 1.78E-05 | -0.0521 | 219 | 0.186 |
| ENSP00000242159 | 3.22E-05 | -0.0521 | 159 | 0.842 |
| ENSP00000310800 | 5.53E-05 | -0.0522 | 372 | 0.792 |
| ENSP00000266517 | 4.04E-05 | -0.0522 | 218 | 0.311 |
| ENSP00000399627 | 3.33E-08 | -0.0522 | 0 | 0.077 |
| ENSP00000265333 | 2.13E-05 | -0.0522 | 815 | 0.613 |
| ENSP00000381045 | 9.91E-03 | -0.0523 | 241 | 0.783 |
| ENSP00000267012 | 1.57E-04 | -0.0523 | 720 | 0.000 |
| ENSP00000301452 | 2.49E-05 | -0.0523 | 151 | 0.090 |
| ENSP00000216027 | 2.38E-05 | -0.0523 | 658 | 0.178 |
| ENSP00000376024 | 3.98E-05 | -0.0523 | 509 | 0.268 |
| ENSP00000265689 | 3.27E-05 | -0.0523 | 292 | 0.723 |
| ENSP00000381198 | 1.78E-05 | -0.0524 | 0 | 0.108 |
| ENSP00000261435 | 5.36E-05 | -0.0524 | 158 | 0.190 |
| ENSP00000343847 | 8.30E-05 | -0.0524 | 207 | 0.486 |
| ENSP00000236957 | 2.67E-05 | -0.0524 | 320 | 0.835 |
| ENSP00000301732 | 4.21E-05 | -0.0524 | 242 | 0.204 |
| ENSP00000370883 | 4.03E-05 | -0.0524 | 214 | 0.145 |
| ENSP00000311538 | 5.03E-05 | -0.0524 | 824 | 0.556 |
| ENSP00000310094 | 6.38E-05 | -0.0525 | 183 | 0.305 |
| ENSP00000217455 | 2.30E-05 | -0.0525 | 347 | 0.300 |
| ENSP00000329357 | 3.19E-05 | -0.0525 | 864 | 0.852 |
| ENSP00000361592 | 3.57E-05 | -0.0525 | 216 | 0.160 |
| ENSP00000373600 | 9.13E-06 | -0.0526 | 0 | 0.687 |
| ENSP00000358212 | 1.89E-08 | -0.0526 | 0 | 0.194 |
| ENSP00000261714 | 4.90E-05 | -0.0526 | 472 | 0.451 |
| ENSP00000257860 | 1.93E-04 | -0.0526 | 572 | 0.247 |
| ENSP00000262120 | 4.38E-05 | -0.0526 | 268 | 0.726 |
| ENSP00000318222 | 7.97E-05 | -0.0526 | 156 | 0.119 |
| ENSP00000349678 | 1.99E-05 | -0.0527 | 0 | 0.098 |
| ENSP00000267415 | 7.28E-05 | -0.0527 | 311 | 0.383 |
| ENSP00000265969 | 1.94E-05 | -0.0527 | 270 | 0.264 |

| | | | | |
|---|---|---|---|---|
| ENSP00000264033 | 3.14E-05 | -0.0527 | 778 | 0.917 |
| ENSP00000370767 | 2.60E-05 | -0.0527 | 517 | 0.504 |
| ENSP00000335029 | 4.90E-05 | -0.0527 | 644 | 0.570 |
| ENSP00000331897 | 2.76E-05 | -0.0527 | 321 | 0.626 |
| ENSP00000250498 | 4.53E-05 | -0.0528 | 215 | 0.377 |
| ENSP00000303174 | 2.92E-05 | -0.0528 | 241 | 0.274 |
| ENSP00000234160 | 3.07E-05 | -0.0528 | 296 | 0.130 |
| ENSP00000307513 | 1.30E-04 | -0.0528 | 196 | 0.157 |
| ENSP00000372390 | 2.60E-05 | -0.0529 | 174 | 0.076 |
| ENSP00000261716 | 4.20E-05 | -0.0529 | 183 | 0.501 |
| ENSP00000221566 | 1.85E-05 | -0.0529 | 550 | 0.692 |
| ENSP00000216602 | 2.17E-04 | -0.0530 | 483 | 0.103 |
| ENSP00000265866 | 4.82E-05 | -0.0530 | 231 | 0.707 |
| ENSP00000309181 | 4.74E-05 | -0.0530 | 185 | 0.748 |
| ENSP00000328671 | 3.50E-05 | -0.0530 | 550 | 0.782 |
| ENSP00000386331 | 3.78E-05 | -0.0530 | 340 | 0.388 |
| ENSP00000217402 | 2.08E-05 | -0.0531 | 459 | 0.000 |
| ENSP00000324105 | 3.60E-05 | -0.0531 | 499 | 0.730 |
| ENSP00000375682 | 2.62E-05 | -0.0531 | 414 | 0.197 |
| ENSP00000376684 | 3.14E-05 | -0.0531 | 205 | 0.000 |
| ENSP00000388731 | 1.80E-05 | -0.0531 | 0 | 0.109 |
| ENSP00000281243 | 4.54E-05 | -0.0532 | 254 | 0.204 |
| ENSP00000244709 | 1.31E-04 | -0.0532 | 181 | 0.503 |
| ENSP00000337125 | 4.71E-05 | -0.0532 | 349 | 0.000 |
| ENSP00000337733 | 5.08E-05 | -0.0532 | 568 | 0.249 |
| ENSP00000343706 | 5.83E-05 | -0.0532 | 207 | 0.247 |
| ENSP00000325612 | 5.59E-05 | -0.0532 | 185 | 0.712 |
| ENSP00000254719 | 2.46E-05 | -0.0533 | 284 | 0.852 |
| ENSP00000252487 | 2.63E-05 | -0.0533 | 560 | 0.385 |
| ENSP00000328228 | 5.64E-05 | -0.0533 | 169 | 0.325 |
| ENSP00000258201 | 7.30E-05 | -0.0533 | 153 | 0.527 |
| ENSP00000301645 | 4.03E-05 | -0.0533 | 580 | 0.542 |
| ENSP00000252244 | 3.53E-05 | -0.0533 | 162 | 0.680 |
| ENSP00000159087 | 5.57E-05 | -0.0533 | 230 | 0.161 |
| ENSP00000272937 | 3.78E-05 | -0.0534 | 154 | 0.809 |
| ENSP00000217652 | 2.22E-05 | -0.0534 | 185 | 0.472 |
| ENSP00000328915 | 3.67E-05 | -0.0534 | 0 | 0.142 |
| ENSP00000284476 | 7.29E-05 | -0.0534 | 181 | 0.550 |
| ENSP00000295802 | 3.64E-05 | -0.0535 | 206 | 0.124 |
| ENSP00000299421 | 5.39E-05 | -0.0535 | 896 | 0.954 |
| ENSP00000357973 | 1.16E-05 | -0.0535 | 0 | 0.169 |
| ENSP00000295981 | 8.94E-05 | -0.0535 | 216 | 0.536 |
| ENSP00000368547 | 2.25E-05 | -0.0536 | 169 | 0.166 |
| ENSP00000456026 | 1.55E-08 | -0.0536 | 0 | 0.217 |
| ENSP00000278836 | 5.45E-05 | -0.0536 | 150 | 0.680 |
| ENSP00000265094 | 2.90E-05 | -0.0536 | 289 | 0.715 |

| | | | | |
|---|---|---|---|---|
| ENSP00000333769 | 5.09E-05 | -0.0536 | 913 | 0.688 |
| ENSP00000364482 | 5.69E-06 | -0.0536 | 0 | 0.153 |
| ENSP00000252725 | 5.85E-05 | -0.0537 | 217 | 0.367 |
| ENSP00000078429 | 3.78E-05 | -0.0538 | 607 | 0.879 |
| ENSP00000378548 | 1.82E-05 | -0.0538 | 234 | 0.320 |
| ENSP00000360821 | 2.53E-05 | -0.0538 | 815 | 0.144 |
| ENSP00000394107 | 3.58E-05 | -0.0538 | 221 | 0.000 |
| ENSP00000330634 | 2.07E-05 | -0.0538 | 338 | 0.000 |
| ENSP00000282030 | 1.96E-05 | -0.0538 | 244 | 0.908 |
| ENSP00000472130 | 3.99E-08 | -0.0538 | 0 | 0.000 |
| ENSP00000296677 | 3.82E-05 | -0.0539 | 823 | 0.604 |
| ENSP00000339769 | 4.40E-05 | -0.0539 | 349 | 0.000 |
| ENSP00000265686 | 2.94E-05 | -0.0539 | 192 | 0.189 |
| ENSP00000289746 | 4.73E-05 | -0.0539 | 198 | 0.557 |
| ENSP00000263373 | 4.29E-05 | -0.0539 | 900 | 0.703 |
| ENSP00000225972 | 8.21E-05 | -0.0539 | 497 | 0.129 |
| ENSP00000269571 | 3.91E-05 | -0.0539 | 922 | 0.967 |
| ENSP00000359820 | 1.55E-05 | -0.0540 | 0 | 0.151 |
| ENSP00000294724 | 3.12E-05 | -0.0540 | 250 | 0.290 |
| ENSP00000361699 | 3.74E-05 | -0.0540 | 377 | 0.710 |
| ENSP00000245564 | 8.28E-05 | -0.0540 | 380 | 0.074 |
| ENSP00000294064 | 9.63E-05 | -0.0540 | 831 | 0.070 |
| ENSP00000266025 | 1.62E-04 | -0.0540 | 164 | 0.181 |
| ENSP00000364858 | 2.08E-05 | -0.0540 | 158 | 0.334 |
| ENSP00000225823 | 2.26E-05 | -0.0540 | 690 | 0.328 |
| ENSP00000310688 | 4.61E-05 | -0.0541 | 191 | 0.213 |
| ENSP00000275603 | 2.48E-05 | -0.0541 | 644 | 0.797 |
| ENSP00000361465 | 3.38E-05 | -0.0541 | 424 | 0.827 |
| ENSP00000308820 | 1.57E-04 | -0.0541 | 200 | 0.084 |
| ENSP00000396301 | 2.92E-05 | -0.0541 | 329 | 0.430 |
| ENSP00000429399 | 1.31E-06 | -0.0541 | 0 | 0.218 |
| ENSP00000240687 | 4.48E-05 | -0.0542 | 0 | 0.209 |
| ENSP00000215057 | 2.71E-05 | -0.0542 | 659 | 0.804 |
| ENSP00000199389 | 3.05E-05 | -0.0543 | 407 | 0.300 |
| ENSP00000237596 | 2.86E-05 | -0.0543 | 270 | 0.378 |
| ENSP00000265572 | 4.29E-05 | -0.0543 | 202 | 0.620 |
| ENSP00000254806 | 1.74E-04 | -0.0543 | 230 | 0.127 |
| ENSP00000245541 | 2.56E-05 | -0.0543 | 457 | 0.000 |
| ENSP00000278919 | 7.29E-05 | -0.0543 | 388 | 0.100 |
| ENSP00000358413 | 1.39E-05 | -0.0544 | 0 | 0.075 |
| ENSP00000283752 | 4.15E-05 | -0.0544 | 194 | 0.514 |
| ENSP00000278865 | 3.83E-05 | -0.0544 | 0 | 0.110 |
| ENSP00000339161 | 1.51E-05 | -0.0544 | 0 | 0.121 |
| ENSP00000231668 | 7.07E-05 | -0.0544 | 167 | 0.206 |
| ENSP00000256759 | 8.16E-05 | -0.0544 | 637 | 0.943 |
| ENSP00000332413 | 1.28E-05 | -0.0544 | 0 | 0.085 |

| | | | | |
|---|---|---|---|---|
| ENSP00000301396 | 6.99E-05 | -0.0544 | 204 | 0.702 |
| ENSP00000215957 | 4.33E-05 | -0.0544 | 338 | 0.450 |
| ENSP00000366416 | 6.76E-05 | -0.0544 | 865 | 0.871 |
| ENSP00000284637 | 9.27E-05 | -0.0545 | 161 | 0.410 |
| ENSP00000361359 | 6.46E-05 | -0.0545 | 630 | 0.949 |
| ENSP00000320324 | 3.03E-05 | -0.0545 | 779 | 0.598 |
| ENSP00000298159 | 2.62E-05 | -0.0545 | 362 | 0.502 |
| ENSP00000295872 | 4.00E-05 | -0.0545 | 394 | 0.736 |
| ENSP00000336528 | 4.07E-05 | -0.0545 | 412 | 0.722 |
| ENSP00000070846 | 2.77E-05 | -0.0545 | 545 | 0.587 |
| ENSP00000341466 | 3.54E-05 | -0.0546 | 165 | 0.000 |
| ENSP00000261868 | 5.03E-05 | -0.0546 | 439 | 0.744 |
| ENSP00000327440 | 2.45E-05 | -0.0546 | 192 | 0.873 |
| ENSP00000276659 | 4.95E-05 | -0.0546 | 173 | 0.734 |
| ENSP00000386791 | 6.67E-06 | -0.0546 | 0 | 0.000 |
| ENSP00000276218 | 7.04E-05 | -0.0546 | 662 | 0.314 |
| ENSP00000317333 | 4.83E-05 | -0.0546 | 208 | 0.848 |
| ENSP00000318615 | 7.38E-05 | -0.0547 | 295 | 0.275 |
| ENSP00000367658 | 3.78E-05 | -0.0547 | 449 | 0.691 |
| ENSP00000269321 | 2.36E-05 | -0.0547 | 226 | 0.718 |
| ENSP00000263932 | 4.81E-05 | -0.0547 | 261 | 0.842 |
| ENSP00000289902 | 4.87E-05 | -0.0547 | 196 | 0.550 |
| ENSP00000285735 | 3.61E-05 | -0.0547 | 685 | 0.589 |
| ENSP00000321735 | 2.82E-05 | -0.0547 | 334 | 0.093 |
| ENSP00000352852 | 6.24E-05 | -0.0547 | 795 | 0.957 |
| ENSP00000300482 | 4.63E-05 | -0.0547 | 816 | 0.143 |
| ENSP00000398597 | 5.30E-05 | -0.0548 | 0 | 0.862 |
| ENSP00000368017 | 2.60E-05 | -0.0548 | 0 | 0.404 |
| ENSP00000359663 | 7.88E-05 | -0.0548 | 895 | 0.949 |
| ENSP00000303351 | 6.97E-05 | -0.0548 | 988 | 0.946 |
| ENSP00000302111 | 5.44E-05 | -0.0548 | 347 | 0.673 |
| ENSP00000327652 | 2.74E-05 | -0.0548 | 299 | 0.000 |
| ENSP00000241416 | 4.03E-05 | -0.0548 | 249 | 0.891 |
| ENSP00000366330 | 7.53E-05 | -0.0548 | 402 | 0.795 |
| ENSP00000385479 | 2.17E-05 | -0.0548 | 702 | 0.960 |
| ENSP00000333275 | 5.26E-05 | -0.0548 | 301 | 0.739 |
| ENSP00000267017 | 1.31E-05 | -0.0549 | 0 | 0.482 |
| ENSP00000257867 | 8.83E-05 | -0.0549 | 574 | 0.161 |
| ENSP00000263980 | 2.35E-05 | -0.0549 | 171 | 0.540 |
| ENSP00000306670 | 4.90E-05 | -0.0549 | 252 | 0.248 |
| ENSP00000335074 | 2.42E-05 | -0.0549 | 824 | 0.664 |
| ENSP00000251849 | 4.22E-05 | -0.0549 | 914 | 0.956 |
| ENSP00000303315 | 2.87E-05 | -0.0549 | 936 | 0.000 |
| ENSP00000317271 | 6.99E-05 | -0.0550 | 204 | 0.097 |
| ENSP00000259154 | 5.38E-05 | -0.0550 | 161 | 0.100 |
| ENSP00000298396 | 3.46E-05 | -0.0550 | 0 | 0.209 |

| | | | | |
|---|---|---|---|---|
| ENSP00000298772 | 6.42E-05 | -0.0550 | 457 | 0.000 |
| ENSP00000220763 | 5.97E-05 | -0.0550 | 161 | 0.244 |
| ENSP00000250831 | 1.97E-05 | -0.0550 | 0 | 0.215 |
| ENSP00000361667 | 2.74E-05 | -0.0550 | 161 | 0.108 |
| ENSP00000259406 | 4.66E-05 | -0.0550 | 254 | 0.649 |
| ENSP00000246785 | 9.05E-05 | -0.0551 | 167 | 0.188 |
| ENSP00000374049 | 9.41E-06 | -0.0551 | 0 | 0.201 |
| ENSP00000328777 | 2.51E-05 | -0.0551 | 195 | 0.853 |
| ENSP00000324270 | 3.32E-05 | -0.0551 | 242 | 0.731 |
| ENSP00000268854 | 2.58E-05 | -0.0551 | 404 | 0.000 |
| ENSP00000272065 | 3.01E-05 | -0.0551 | 214 | 0.677 |
| ENSP00000344322 | 3.65E-05 | -0.0552 | 213 | 0.078 |
| ENSP00000269122 | 2.27E-05 | -0.0552 | 281 | 0.621 |
| ENSP00000216122 | 1.41E-05 | -0.0552 | 252 | 0.000 |
| ENSP00000298352 | 3.36E-05 | -0.0552 | 212 | 0.414 |
| ENSP00000249776 | 2.05E-05 | -0.0552 | 202 | 0.189 |
| ENSP00000233893 | 1.74E-05 | -0.0552 | 724 | 0.722 |
| ENSP00000327116 | 4.83E-05 | -0.0553 | 177 | 0.131 |
| ENSP00000260762 | 3.94E-05 | -0.0553 | 175 | 0.254 |
| ENSP00000368759 | 3.82E-05 | -0.0553 | 319 | 0.858 |
| ENSP00000371483 | 2.85E-05 | -0.0553 | 0 | 0.111 |
| ENSP00000354560 | 2.06E-05 | -0.0554 | 359 | 0.345 |
| ENSP00000302647 | 4.34E-05 | -0.0554 | 349 | 0.234 |
| ENSP00000259477 | 4.80E-05 | -0.0554 | 373 | 0.470 |
| ENSP00000306997 | 9.77E-06 | -0.0554 | 0 | 0.280 |
| ENSP00000256999 | 3.32E-05 | -0.0554 | 394 | 0.345 |
| ENSP00000374558 | 1.31E-05 | -0.0554 | 0 | 0.116 |
| ENSP00000287667 | 4.15E-05 | -0.0554 | 211 | 0.148 |
| ENSP00000272298 | 1.92E-05 | -0.0554 | 625 | 0.660 |
| ENSP00000339399 | 5.12E-05 | -0.0554 | 593 | 0.158 |
| ENSP00000343885 | 3.06E-05 | -0.0555 | 214 | 0.671 |
| ENSP00000300305 | 5.44E-05 | -0.0555 | 267 | 0.870 |
| ENSP00000346986 | 1.57E-05 | -0.0555 | 0 | 0.462 |
| ENSP00000353483 | 5.67E-05 | -0.0555 | 967 | 0.961 |
| ENSP00000360688 | 1.54E-05 | -0.0555 | 0 | 0.110 |
| ENSP00000263846 | 1.38E-05 | -0.0555 | 0 | 0.226 |
| ENSP00000254227 | 2.39E-05 | -0.0555 | 395 | 0.858 |
| ENSP00000002165 | 6.14E-05 | -0.0556 | 205 | 0.079 |
| ENSP00000345892 | 2.97E-05 | -0.0556 | 406 | 0.191 |
| ENSP00000294168 | 4.14E-05 | -0.0556 | 200 | 0.876 |
| ENSP00000429344 | 2.10E-05 | -0.0556 | 216 | 0.344 |
| ENSP00000293230 | 3.01E-05 | -0.0556 | 216 | 0.467 |
| ENSP00000260210 | 3.08E-05 | -0.0556 | 460 | 0.764 |
| ENSP00000247001 | 4.25E-05 | -0.0556 | 908 | 0.794 |
| ENSP00000337088 | 3.13E-05 | -0.0556 | 610 | 0.853 |
| ENSP00000317123 | 3.61E-05 | -0.0557 | 368 | 0.904 |

| | | | | |
|---|---|---|---|---|
| ENSP00000431284 | 3.64E-05 | -0.0557 | 727 | 0.666 |
| ENSP00000226730 | 3.86E-05 | -0.0557 | 534 | 0.952 |
| ENSP00000281453 | 5.21E-05 | -0.0557 | 150 | 0.000 |
| ENSP00000359444 | 3.50E-05 | -0.0558 | 558 | 0.000 |
| ENSP00000306662 | 1.95E-05 | -0.0558 | 197 | 0.580 |
| ENSP00000320516 | 3.26E-05 | -0.0558 | 194 | 0.285 |
| ENSP00000398704 | 6.48E-05 | -0.0558 | 0 | 0.000 |
| ENSP00000239940 | 2.01E-05 | -0.0558 | 213 | 0.483 |
| ENSP00000262746 | 2.44E-05 | -0.0558 | 579 | 0.786 |
| ENSP00000391669 | 2.87E-05 | -0.0558 | 911 | 0.887 |
| ENSP00000273317 | 4.84E-05 | -0.0558 | 152 | 0.349 |
| ENSP00000222969 | 2.57E-05 | -0.0559 | 421 | 0.641 |
| ENSP00000292035 | 4.54E-05 | -0.0559 | 214 | 0.705 |
| ENSP00000352162 | 3.41E-05 | -0.0559 | 309 | 0.869 |
| ENSP00000227752 | 4.40E-05 | -0.0559 | 177 | 0.683 |
| ENSP00000264220 | 2.64E-05 | -0.0559 | 203 | 0.550 |
| ENSP00000178638 | 4.82E-05 | -0.0560 | 165 | 0.275 |
| ENSP00000260264 | 3.33E-05 | -0.0560 | 499 | 0.640 |
| ENSP00000287038 | 2.68E-05 | -0.0560 | 198 | 0.824 |
| ENSP00000253754 | 2.33E-05 | -0.0560 | 287 | 0.648 |
| ENSP00000222305 | 3.21E-05 | -0.0560 | 585 | 0.828 |
| ENSP00000295934 | 3.02E-05 | -0.0560 | 530 | 0.842 |
| ENSP00000227378 | 2.76E-05 | -0.0561 | 608 | 0.959 |
| ENSP00000204961 | 2.46E-05 | -0.0561 | 161 | 0.919 |
| ENSP00000234420 | 1.99E-05 | -0.0561 | 319 | 0.744 |
| ENSP00000227868 | 1.93E-05 | -0.0561 | 404 | 0.640 |
| ENSP00000279227 | 5.32E-05 | -0.0561 | 739 | 0.191 |
| ENSP00000283027 | 3.07E-05 | -0.0561 | 158 | 0.277 |
| ENSP00000299335 | 3.91E-05 | -0.0561 | 199 | 0.292 |
| ENSP00000296869 | 3.26E-05 | -0.0562 | 154 | 0.405 |
| ENSP00000331019 | 3.01E-05 | -0.0562 | 202 | 0.744 |
| ENSP00000345405 | 1.08E-05 | -0.0562 | 0 | 0.412 |
| ENSP00000251496 | 3.55E-05 | -0.0562 | 212 | 0.470 |
| ENSP00000289371 | 2.34E-05 | -0.0563 | 653 | 0.817 |
| ENSP00000291577 | 4.01E-05 | -0.0563 | 206 | 0.081 |
| ENSP00000222482 | 4.03E-05 | -0.0563 | 185 | 0.055 |
| ENSP00000258787 | 2.70E-05 | -0.0563 | 244 | 0.389 |
| ENSP00000319279 | 4.11E-05 | -0.0563 | 210 | 0.839 |
| ENSP00000313571 | 3.44E-05 | -0.0563 | 608 | 0.256 |
| ENSP00000224784 | 2.25E-05 | -0.0563 | 674 | 0.705 |
| ENSP00000366950 | 2.05E-05 | -0.0564 | 0 | 0.209 |
| ENSP00000333915 | 6.48E-06 | -0.0564 | 0 | 0.000 |
| ENSP00000253382 | 2.43E-05 | -0.0564 | 235 | 0.416 |
| ENSP00000239151 | 3.56E-05 | -0.0564 | 525 | 0.855 |
| ENSP00000376177 | 2.68E-05 | -0.0565 | 413 | 0.586 |
| ENSP00000250018 | 2.23E-05 | -0.0565 | 268 | 0.599 |

| | | | | |
|---|---|---|---|---|
| ENSP00000329106 | 3.19E-05 | -0.0565 | 0 | 0.191 |
| ENSP00000319977 | 3.25E-05 | -0.0565 | 426 | 0.696 |
| ENSP00000199814 | 2.61E-05 | -0.0565 | 429 | 0.700 |
| ENSP00000257192 | 3.84E-05 | -0.0565 | 439 | 0.324 |
| ENSP00000354111 | 2.81E-05 | -0.0566 | 934 | 0.427 |
| ENSP00000244741 | 3.60E-05 | -0.0566 | 503 | 0.000 |
| ENSP00000303145 | 3.07E-05 | -0.0566 | 492 | 0.276 |
| ENSP00000302599 | 1.48E-05 | -0.0566 | 361 | 0.087 |
| ENSP00000249883 | 6.41E-05 | -0.0566 | 223 | 0.167 |
| ENSP00000308576 | 2.50E-05 | -0.0566 | 515 | 0.540 |
| ENSP00000253968 | 6.74E-05 | -0.0566 | 179 | 0.837 |
| ENSP00000308540 | 3.51E-05 | -0.0567 | 516 | 0.000 |
| ENSP00000318351 | 2.52E-05 | -0.0567 | 319 | 0.649 |
| ENSP00000229329 | 2.30E-05 | -0.0567 | 345 | 0.222 |
| ENSP00000221700 | 5.30E-05 | -0.0567 | 282 | 0.304 |
| ENSP00000234454 | 9.98E-05 | -0.0567 | 159 | 0.113 |
| ENSP00000251287 | 3.91E-05 | -0.0567 | 158 | 0.299 |
| ENSP00000290349 | 3.12E-05 | -0.0567 | 194 | 0.262 |
| ENSP00000255764 | 3.89E-05 | -0.0568 | 234 | 0.721 |
| ENSP00000355797 | 1.47E-07 | -0.0568 | 0 | 0.215 |
| ENSP00000005286 | 7.93E-05 | -0.0568 | 613 | 0.128 |
| ENSP00000267119 | 4.24E-05 | -0.0568 | 173 | 0.427 |
| ENSP00000319781 | 2.37E-05 | -0.0568 | 284 | 0.254 |
| ENSP00000240050 | 1.73E-05 | -0.0568 | 0 | 0.000 |
| ENSP00000221740 | 6.15E-05 | -0.0569 | 222 | 0.000 |
| ENSP00000262839 | 5.59E-05 | -0.0569 | 292 | 0.477 |
| ENSP00000382004 | 2.72E-05 | -0.0569 | 295 | 0.811 |
| ENSP00000368959 | 4.24E-05 | -0.0569 | 396 | 0.285 |
| ENSP00000302935 | 4.21E-05 | -0.0569 | 240 | 0.630 |
| ENSP00000334300 | 2.93E-05 | -0.0569 | 449 | 0.689 |
| ENSP00000308548 | 5.12E-05 | -0.0569 | 215 | 0.141 |
| ENSP00000349693 | 9.02E-06 | -0.0569 | 0 | 0.000 |
| ENSP00000228872 | 4.23E-05 | -0.0569 | 878 | 0.912 |
| ENSP00000354901 | 4.09E-05 | -0.0569 | 347 | 0.720 |
| ENSP00000300404 | 2.07E-05 | -0.0569 | 609 | 0.256 |
| ENSP00000356236 | 9.78E-06 | -0.0570 | 0 | 0.249 |
| ENSP00000338799 | 3.83E-05 | -0.0570 | 208 | 0.843 |
| ENSP00000221515 | 1.20E-04 | -0.0570 | 891 | 0.862 |
| ENSP00000251871 | 3.94E-05 | -0.0570 | 284 | 0.757 |
| ENSP00000246071 | 2.63E-05 | -0.0571 | 599 | 0.872 |
| ENSP00000330074 | 4.37E-05 | -0.0571 | 737 | 0.868 |
| ENSP00000164139 | 2.03E-05 | -0.0571 | 176 | 0.441 |
| ENSP00000188790 | 6.64E-05 | -0.0571 | 553 | 0.290 |
| ENSP00000005587 | 4.47E-05 | -0.0571 | 191 | 0.168 |
| ENSP00000271636 | 3.64E-05 | -0.0572 | 151 | 0.431 |
| ENSP00000278483 | 7.81E-05 | -0.0572 | 248 | 0.148 |

| | | | | |
|---|---|---|---|---|
| ENSP00000265560 | 3.79E-05 | -0.0572 | 259 | 0.267 |
| ENSP00000260442 | 5.72E-05 | -0.0572 | 150 | 0.426 |
| ENSP00000327431 | 3.08E-05 | -0.0572 | 157 | 0.353 |
| ENSP00000266556 | 5.60E-05 | -0.0572 | 439 | 0.489 |
| ENSP00000261731 | 1.61E-05 | -0.0572 | 219 | 0.719 |
| ENSP00000305343 | 1.42E-05 | -0.0572 | 0 | 0.506 |
| ENSP00000249007 | 6.85E-05 | -0.0572 | 235 | 0.403 |
| ENSP00000280333 | 3.01E-05 | -0.0573 | 209 | 0.748 |
| ENSP00000230449 | 2.85E-05 | -0.0573 | 202 | 0.248 |
| ENSP00000326627 | 1.11E-05 | -0.0573 | 504 | 0.107 |
| ENSP00000005905 | 8.52E-05 | -0.0573 | 197 | 0.000 |
| ENSP00000323036 | 4.33E-05 | -0.0573 | 159 | 0.170 |
| ENSP00000320171 | 3.15E-05 | -0.0574 | 499 | 0.870 |
| ENSP00000284320 | 3.02E-05 | -0.0574 | 350 | 0.202 |
| ENSP00000307241 | 2.75E-05 | -0.0574 | 397 | 0.492 |
| ENSP00000345206 | 5.94E-05 | -0.0574 | 459 | 0.895 |
| ENSP00000210633 | 1.39E-05 | -0.0574 | 0 | 0.402 |
| ENSP00000325875 | 4.14E-05 | -0.0574 | 988 | 0.878 |
| ENSP00000458537 | 5.11E-05 | -0.0574 | 646 | 0.883 |
| ENSP00000334134 | 2.44E-05 | -0.0575 | 554 | 0.443 |
| ENSP00000054650 | 1.25E-04 | -0.0575 | 222 | 0.199 |
| ENSP00000261637 | 3.40E-05 | -0.0575 | 271 | 0.885 |
| ENSP00000365233 | 3.15E-05 | -0.0575 | 712 | 0.418 |
| ENSP00000290650 | 3.89E-05 | -0.0575 | 311 | 0.400 |
| ENSP00000239938 | 3.24E-05 | -0.0575 | 896 | 0.925 |
| ENSP00000342793 | 2.73E-05 | -0.0576 | 547 | 0.434 |
| ENSP00000351697 | 2.19E-05 | -0.0576 | 266 | 0.745 |
| ENSP00000321475 | 2.11E-05 | -0.0576 | 150 | 0.196 |
| ENSP00000350052 | 4.88E-05 | -0.0576 | 626 | 0.000 |
| ENSP00000365411 | 2.06E-05 | -0.0576 | 912 | 0.447 |
| ENSP00000252934 | 6.54E-05 | -0.0576 | 570 | 0.176 |
| ENSP00000281455 | 3.42E-05 | -0.0576 | 309 | 0.611 |
| ENSP00000347379 | 9.13E-05 | -0.0576 | 567 | 0.878 |
| ENSP00000329933 | 2.86E-05 | -0.0576 | 265 | 0.905 |
| ENSP00000260810 | 3.56E-05 | -0.0577 | 437 | 0.804 |
| ENSP00000322542 | 3.87E-05 | -0.0577 | 859 | 0.000 |
| ENSP00000262109 | 1.87E-02 | -0.0577 | 0 | 0.213 |
| ENSP00000251547 | 4.51E-05 | -0.0577 | 171 | 0.116 |
| ENSP00000265471 | 6.12E-05 | -0.0577 | 175 | 0.058 |
| ENSP00000357901 | 1.13E-05 | -0.0578 | 0 | 0.077 |
| ENSP00000262096 | 6.77E-05 | -0.0578 | 0 | 0.103 |
| ENSP00000334928 | 2.37E-05 | -0.0578 | 195 | 0.634 |
| ENSP00000369154 | 3.03E-05 | -0.0578 | 568 | 0.876 |
| ENSP00000294008 | 4.57E-05 | -0.0578 | 306 | 0.485 |
| ENSP00000262713 | 2.94E-05 | -0.0578 | 152 | 0.613 |
| ENSP00000303908 | 3.72E-05 | -0.0578 | 150 | 0.259 |

| | | | | |
|---|---|---|---|---|
| ENSP00000251636 | 2.21E-05 | -0.0578 | 556 | 0.923 |
| ENSP00000261772 | 2.59E-05 | -0.0579 | 404 | 0.723 |
| ENSP00000264690 | 5.17E-05 | -0.0579 | 538 | 0.683 |
| ENSP00000296456 | 2.85E-05 | -0.0579 | 498 | 0.433 |
| ENSP00000354609 | 8.90E-06 | -0.0579 | 0 | 0.412 |
| ENSP00000384708 | 3.14E-05 | -0.0579 | 307 | 0.751 |
| ENSP00000040663 | 5.82E-05 | -0.0579 | 386 | 0.231 |
| ENSP00000292779 | 2.64E-05 | -0.0579 | 0 | 0.210 |
| ENSP00000369136 | 7.06E-06 | -0.0580 | 0 | 0.512 |
| ENSP00000156471 | 3.67E-05 | -0.0580 | 186 | 0.814 |
| ENSP00000252483 | 5.65E-05 | -0.0581 | 225 | 0.186 |
| ENSP00000287727 | 4.16E-05 | -0.0581 | 564 | 0.265 |
| ENSP00000308208 | 9.58E-05 | -0.0581 | 997 | 0.960 |
| ENSP00000334872 | 9.55E-05 | -0.0581 | 193 | 0.144 |
| ENSP00000225504 | 2.89E-05 | -0.0581 | 307 | 0.910 |
| ENSP00000202773 | 2.28E-05 | -0.0581 | 195 | 0.861 |
| ENSP00000206595 | 6.52E-05 | -0.0581 | 274 | 0.327 |
| ENSP00000273130 | 4.40E-05 | -0.0581 | 159 | 0.058 |
| ENSP00000301956 | 2.68E-05 | -0.0582 | 414 | 0.445 |
| ENSP00000303830 | 4.08E-05 | -0.0582 | 535 | 0.715 |
| ENSP00000308466 | 4.00E-05 | -0.0582 | 181 | 0.061 |
| ENSP00000248935 | 1.70E-05 | -0.0582 | 336 | 0.000 |
| ENSP00000258428 | 2.41E-05 | -0.0582 | 206 | 0.651 |
| ENSP00000275233 | 2.53E-05 | -0.0582 | 160 | 0.944 |
| ENSP00000300108 | 3.03E-05 | -0.0582 | 432 | 0.686 |
| ENSP00000057513 | 6.31E-05 | -0.0583 | 159 | 0.331 |
| ENSP00000251127 | 2.67E-05 | -0.0583 | 380 | 0.216 |
| ENSP00000278612 | 2.65E-05 | -0.0583 | 390 | 0.764 |
| ENSP00000258874 | 4.95E-05 | -0.0583 | 200 | 0.148 |
| ENSP00000366962 | 2.50E-05 | -0.0584 | 890 | 0.948 |
| ENSP00000357794 | 2.92E-05 | -0.0584 | 317 | 0.594 |
| ENSP00000264080 | 6.66E-05 | -0.0584 | 157 | 0.143 |
| ENSP00000250111 | 3.69E-05 | -0.0584 | 225 | 0.078 |
| ENSP00000312185 | 3.72E-05 | -0.0585 | 224 | 0.504 |
| ENSP00000295736 | 2.91E-05 | -0.0585 | 197 | 0.257 |
| ENSP00000307567 | 2.50E-05 | -0.0585 | 367 | 0.823 |
| ENSP00000243108 | 3.56E-05 | -0.0585 | 194 | 0.832 |
| ENSP00000307599 | 2.55E-05 | -0.0585 | 250 | 0.496 |
| ENSP00000233468 | 4.06E-05 | -0.0585 | 185 | 0.000 |
| ENSP00000313581 | 5.74E-05 | -0.0586 | 505 | 0.254 |
| ENSP00000336775 | 2.86E-05 | -0.0586 | 309 | 0.369 |
| ENSP00000305675 | 3.59E-05 | -0.0586 | 329 | 0.391 |
| ENSP00000233838 | 6.83E-05 | -0.0586 | 359 | 0.204 |
| ENSP00000339328 | 1.28E-04 | -0.0586 | 918 | 0.855 |
| ENSP00000158762 | 5.26E-05 | -0.0586 | 242 | 0.270 |
| ENSP00000207549 | 4.06E-05 | -0.0586 | 157 | 0.175 |

| | | | | |
|---|---|---|---|---|
| ENSP00000332326 | 2.65E-05 | -0.0586 | 242 | 0.433 |
| ENSP00000216115 | 7.67E-05 | -0.0586 | 311 | 0.344 |
| ENSP00000344314 | 3.42E-05 | -0.0587 | 216 | 0.452 |
| ENSP00000427211 | 1.70E-05 | -0.0587 | 250 | 0.551 |
| ENSP00000263826 | 1.72E-05 | -0.0587 | 699 | 0.679 |
| ENSP00000264895 | 2.21E-05 | -0.0587 | 438 | 0.000 |
| ENSP00000281938 | 4.14E-05 | -0.0587 | 276 | 0.573 |
| ENSP00000313670 | 5.00E-05 | -0.0588 | 200 | 0.117 |
| ENSP00000206249 | 1.86E-05 | -0.0588 | 876 | 0.000 |
| ENSP00000263372 | 2.54E-05 | -0.0588 | 203 | 0.240 |
| ENSP00000338481 | 3.28E-05 | -0.0588 | 232 | 0.300 |
| ENSP00000305152 | 2.71E-05 | -0.0588 | 177 | 0.000 |
| ENSP00000267970 | 4.64E-05 | -0.0588 | 690 | 0.113 |
| ENSP00000370104 | 1.31E-05 | -0.0588 | 0 | 0.605 |
| ENSP00000443399 | 1.97E-07 | -0.0588 | 0 | 0.000 |
| ENSP00000280605 | 2.67E-05 | -0.0588 | 408 | 0.480 |
| ENSP00000424711 | 1.86E-06 | -0.0588 | 0 | 0.142 |
| ENSP00000340211 | 3.46E-05 | -0.0589 | 335 | 0.376 |
| ENSP00000267460 | 8.25E-05 | -0.0589 | 158 | 0.300 |
| ENSP00000320936 | 5.79E-05 | -0.0589 | 240 | 0.717 |
| ENSP00000315557 | 2.06E-06 | -0.0589 | 0 | 0.215 |
| ENSP00000248958 | 6.15E-05 | -0.0590 | 276 | 0.131 |
| ENSP00000303920 | 5.90E-05 | -0.0590 | 751 | 0.116 |
| ENSP00000295050 | 3.36E-05 | -0.0590 | 360 | 0.344 |
| ENSP00000225426 | 3.51E-05 | -0.0590 | 331 | 0.000 |
| ENSP00000223127 | 4.22E-05 | -0.0590 | 190 | 0.116 |
| ENSP00000316476 | 3.98E-05 | -0.0590 | 158 | 0.302 |
| ENSP00000449241 | 2.53E-05 | -0.0590 | 205 | 0.252 |
| ENSP00000338235 | 3.34E-05 | -0.0591 | 842 | 0.696 |
| ENSP00000331504 | 2.28E-05 | -0.0591 | 270 | 0.765 |
| ENSP00000295770 | 3.64E-05 | -0.0591 | 368 | 0.254 |
| ENSP00000280200 | 3.16E-05 | -0.0591 | 214 | 0.516 |
| ENSP00000240423 | 2.34E-05 | -0.0591 | 192 | 0.547 |
| ENSP00000314615 | 5.67E-05 | -0.0591 | 324 | 0.256 |
| ENSP00000299328 | 2.77E-05 | -0.0592 | 381 | 0.000 |
| ENSP00000329932 | 8.44E-05 | -0.0592 | 181 | 0.159 |
| ENSP00000263025 | 2.80E-05 | -0.0592 | 467 | 0.000 |
| ENSP00000260867 | 2.89E-05 | -0.0592 | 261 | 0.000 |
| ENSP00000427279 | 3.08E-05 | -0.0592 | 295 | 0.778 |
| ENSP00000307235 | 5.51E-05 | -0.0592 | 999 | 0.936 |
| ENSP00000462745 | 1.89E-05 | -0.0592 | 156 | 0.115 |
| ENSP00000243253 | 1.65E-05 | -0.0592 | 890 | 0.652 |
| ENSP00000363397 | 3.67E-05 | -0.0592 | 233 | 0.292 |
| ENSP00000287600 | 5.02E-05 | -0.0593 | 211 | 0.269 |
| ENSP00000362849 | 2.43E-05 | -0.0593 | 520 | 0.000 |
| ENSP00000262457 | 2.12E-05 | -0.0593 | 202 | 0.797 |

| | | | | |
|---|---|---|---|---|
| ENSP00000225724 | 2.72E-05 | -0.0593 | 179 | 0.189 |
| ENSP00000249066 | 5.77E-05 | -0.0593 | 163 | 0.246 |
| ENSP00000315693 | 4.02E-05 | -0.0593 | 152 | 0.079 |
| ENSP00000266095 | 3.65E-05 | -0.0593 | 158 | 0.148 |
| ENSP00000307859 | 3.41E-05 | -0.0594 | 234 | 0.598 |
| ENSP00000312697 | 3.42E-05 | -0.0594 | 356 | 0.866 |
| ENSP00000262450 | 2.59E-05 | -0.0594 | 209 | 0.916 |
| ENSP00000341390 | 2.55E-05 | -0.0594 | 254 | 0.767 |
| ENSP00000263268 | 3.16E-05 | -0.0594 | 264 | 0.289 |
| ENSP00000225174 | 1.69E-05 | -0.0594 | 516 | 0.734 |
| ENSP00000385328 | 2.24E-05 | -0.0594 | 156 | 0.513 |
| ENSP00000365076 | 7.75E-06 | -0.0595 | 0 | 0.186 |
| ENSP00000314151 | 6.13E-05 | -0.0595 | 893 | 0.758 |
| ENSP00000228307 | 4.97E-05 | -0.0595 | 962 | 0.911 |
| ENSP00000335055 | 4.13E-05 | -0.0595 | 195 | 0.388 |
| ENSP00000228945 | 2.34E-05 | -0.0595 | 211 | 0.534 |
| ENSP00000219548 | 2.37E-05 | -0.0595 | 524 | 0.821 |
| ENSP00000311032 | 5.91E-05 | -0.0595 | 973 | 0.930 |
| ENSP00000385158 | 3.91E-05 | -0.0595 | 398 | 0.367 |
| ENSP00000240304 | 4.06E-05 | -0.0596 | 496 | 0.781 |
| ENSP00000311135 | 2.30E-05 | -0.0596 | 275 | 0.920 |
| ENSP00000218075 | 2.01E-05 | -0.0596 | 156 | 0.309 |
| ENSP00000253861 | 2.26E-05 | -0.0596 | 298 | 0.286 |
| ENSP00000373648 | 2.63E-05 | -0.0597 | 900 | 0.265 |
| ENSP00000306279 | 3.44E-05 | -0.0597 | 215 | 0.341 |
| ENSP00000338157 | 3.89E-05 | -0.0597 | 606 | 0.845 |
| ENSP00000262441 | 3.62E-05 | -0.0597 | 164 | 0.521 |
| ENSP00000239462 | 1.87E-05 | -0.0597 | 242 | 0.404 |
| ENSP00000301785 | 4.91E-05 | -0.0597 | 644 | 0.401 |
| ENSP00000290246 | 3.68E-05 | -0.0598 | 227 | 0.381 |
| ENSP00000272895 | 3.73E-05 | -0.0598 | 804 | 0.187 |
| ENSP00000386230 | 1.75E-07 | -0.0598 | 0 | 0.214 |
| ENSP00000318227 | 6.31E-05 | -0.0599 | 191 | 0.079 |
| ENSP00000269881 | 1.75E-05 | -0.0599 | 451 | 0.211 |
| ENSP00000345282 | 9.31E-06 | -0.0599 | 0 | 0.313 |
| ENSP00000360455 | 7.00E-06 | -0.0600 | 0 | 0.706 |
| ENSP00000250535 | 6.25E-05 | -0.0600 | 800 | 0.085 |
| ENSP00000260505 | 8.79E-06 | -0.0600 | 0 | 0.076 |
| ENSP00000364784 | 6.43E-08 | -0.0600 | 0 | 0.170 |
| ENSP00000445831 | 2.16E-05 | -0.0600 | 819 | 0.953 |
| ENSP00000318585 | 5.62E-05 | -0.0600 | 633 | 0.000 |
| ENSP00000233143 | 7.83E-05 | -0.0600 | 203 | 0.133 |
| ENSP00000354794 | 7.14E-05 | -0.0600 | 222 | 0.754 |
| ENSP00000206262 | 3.96E-05 | -0.0601 | 188 | 0.414 |
| ENSP00000307240 | 3.16E-05 | -0.0601 | 182 | 0.449 |
| ENSP00000252455 | 4.21E-05 | -0.0601 | 384 | 0.000 |

| | | | | |
|---|---|---|---|---|
| ENSP00000221132 | 4.59E-05 | -0.0601 | 242 | 0.850 |
| ENSP00000263383 | 2.66E-05 | -0.0601 | 229 | 0.417 |
| ENSP00000316948 | 2.32E-05 | -0.0601 | 254 | 0.606 |
| ENSP00000325301 | 8.24E-08 | -0.0601 | 0 | 0.211 |
| ENSP00000343273 | 3.94E-05 | -0.0602 | 309 | 0.360 |
| ENSP00000288985 | 2.62E-05 | -0.0602 | 284 | 0.916 |
| ENSP00000322450 | 2.55E-05 | -0.0602 | 357 | 0.413 |
| ENSP00000343164 | 2.60E-05 | -0.0602 | 253 | 0.000 |
| ENSP00000290399 | 3.82E-05 | -0.0602 | 158 | 0.716 |
| ENSP00000309771 | 3.44E-05 | -0.0602 | 225 | 0.527 |
| ENSP00000322142 | 2.72E-05 | -0.0602 | 203 | 0.933 |
| ENSP00000156626 | 2.92E-05 | -0.0602 | 171 | 0.238 |
| ENSP00000222329 | 4.76E-05 | -0.0603 | 181 | 0.744 |
| ENSP00000250024 | 9.52E-06 | -0.0603 | 0 | 0.597 |
| ENSP00000270625 | 2.04E-05 | -0.0604 | 356 | 0.825 |
| ENSP00000269127 | 2.20E-05 | -0.0604 | 160 | 0.944 |
| ENSP00000345599 | 1.00E-04 | -0.0604 | 261 | 0.000 |
| ENSP00000351384 | 8.44E-07 | -0.0604 | 0 | 0.000 |
| ENSP00000216044 | 3.21E-05 | -0.0604 | 216 | 0.189 |
| ENSP00000322909 | 4.47E-05 | -0.0604 | 302 | 0.855 |
| ENSP00000364700 | 3.15E-05 | -0.0605 | 906 | 0.793 |
| ENSP00000361556 | 2.31E-06 | -0.0605 | 0 | 0.169 |
| ENSP00000261024 | 6.07E-05 | -0.0605 | 192 | 0.335 |
| ENSP00000006015 | 3.05E-05 | -0.0605 | 165 | 0.834 |
| ENSP00000350941 | 6.99E-05 | -0.0605 | 993 | 0.000 |
| ENSP00000320866 | 5.95E-05 | -0.0605 | 931 | 0.880 |
| ENSP00000234179 | 2.93E-05 | -0.0606 | 282 | 0.437 |
| ENSP00000218249 | 1.98E-05 | -0.0606 | 207 | 0.261 |
| ENSP00000386452 | 2.15E-06 | -0.0606 | 0 | 0.199 |
| ENSP00000330289 | 4.05E-05 | -0.0606 | 230 | 0.113 |
| ENSP00000245046 | 3.71E-05 | -0.0606 | 191 | 0.084 |
| ENSP00000444196 | 2.54E-05 | -0.0606 | 680 | 0.000 |
| ENSP00000255084 | 1.83E-05 | -0.0606 | 216 | 0.276 |
| ENSP00000419249 | 1.08E-05 | -0.0606 | 0 | 0.170 |
| ENSP00000339566 | 4.62E-05 | -0.0606 | 595 | 0.877 |
| ENSP00000358323 | 1.09E-04 | -0.0606 | 371 | 0.000 |
| ENSP00000216465 | 1.74E-05 | -0.0606 | 328 | 0.553 |
| ENSP00000222511 | 2.33E-05 | -0.0606 | 340 | 0.631 |
| ENSP00000348784 | 3.23E-05 | -0.0606 | 610 | 0.875 |
| ENSP00000324248 | 3.37E-05 | -0.0606 | 349 | 0.736 |
| ENSP00000275015 | 2.08E-05 | -0.0607 | 275 | 0.811 |
| ENSP00000358310 | 1.73E-05 | -0.0607 | 288 | 0.219 |
| ENSP00000217133 | 1.49E-05 | -0.0607 | 402 | 0.575 |
| ENSP00000215742 | 2.05E-05 | -0.0607 | 0 | 0.302 |
| ENSP00000342056 | 3.60E-05 | -0.0607 | 581 | 0.641 |
| ENSP00000338018 | 4.83E-05 | -0.0607 | 887 | 0.934 |

| | | | | |
|---|---|---|---|---|
| ENSP00000449002 | 4.73E-06 | -0.0607 | 0 | 0.156 |
| ENSP00000307183 | 2.69E-05 | -0.0607 | 203 | 0.910 |
| ENSP00000336831 | 3.03E-05 | -0.0607 | 638 | 0.530 |
| ENSP00000308383 | 6.09E-05 | -0.0607 | 199 | 0.562 |
| ENSP00000314048 | 3.82E-05 | -0.0607 | 359 | 0.083 |
| ENSP00000284690 | 2.48E-05 | -0.0607 | 268 | 0.928 |
| ENSP00000221283 | 2.20E-05 | -0.0607 | 204 | 0.247 |
| ENSP00000289416 | 2.33E-05 | -0.0608 | 183 | 0.342 |
| ENSP00000256935 | 2.84E-05 | -0.0608 | 214 | 0.567 |
| ENSP00000254810 | 1.71E-05 | -0.0608 | 702 | 0.935 |
| ENSP00000307305 | 3.79E-05 | -0.0608 | 191 | 0.786 |
| ENSP00000256593 | 3.81E-05 | -0.0608 | 164 | 0.095 |
| ENSP00000278886 | 2.58E-05 | -0.0608 | 216 | 0.167 |
| ENSP00000266643 | 3.53E-05 | -0.0609 | 205 | 0.246 |
| ENSP00000261783 | 2.32E-05 | -0.0609 | 242 | 0.351 |
| ENSP00000247306 | 4.92E-03 | -0.0609 | 0 | 0.184 |
| ENSP00000223029 | 3.52E-05 | -0.0609 | 311 | 0.433 |
| ENSP00000328236 | 4.20E-05 | -0.0609 | 491 | 0.333 |
| ENSP00000268058 | 2.72E-05 | -0.0609 | 900 | 0.766 |
| ENSP00000307234 | 1.67E-05 | -0.0609 | 0 | 0.121 |
| ENSP00000217426 | 1.77E-05 | -0.0609 | 343 | 0.567 |
| ENSP00000294179 | 2.68E-05 | -0.0609 | 490 | 0.246 |
| ENSP00000253814 | 5.35E-05 | -0.0609 | 203 | 0.073 |
| ENSP00000337261 | 5.15E-05 | -0.0609 | 234 | 0.779 |
| ENSP00000346015 | 7.59E-05 | -0.0610 | 469 | 0.799 |
| ENSP00000303554 | 3.59E-05 | -0.0610 | 303 | 0.396 |
| ENSP00000265896 | 3.39E-05 | -0.0610 | 284 | 0.479 |
| ENSP00000245312 | 2.99E-05 | -0.0610 | 242 | 0.303 |
| ENSP00000242577 | 2.25E-05 | -0.0610 | 197 | 0.278 |
| ENSP00000302621 | 3.21E-05 | -0.0610 | 600 | 0.723 |
| ENSP00000298937 | 3.69E-05 | -0.0610 | 213 | 0.601 |
| ENSP00000301286 | 6.29E-05 | -0.0610 | 151 | 0.099 |
| ENSP00000344562 | 3.48E-05 | -0.0610 | 335 | 0.394 |
| ENSP00000294816 | 1.45E-05 | -0.0611 | 174 | 0.835 |
| ENSP00000384223 | 2.82E-05 | -0.0611 | 479 | 0.299 |
| ENSP00000275815 | 2.13E-05 | -0.0611 | 349 | 0.664 |
| ENSP00000389948 | 7.20E-06 | -0.0611 | 0 | 0.061 |
| ENSP00000267436 | 3.27E-05 | -0.0611 | 199 | 0.194 |
| ENSP00000265846 | 4.24E-05 | -0.0611 | 168 | 0.204 |
| ENSP00000255631 | 3.03E-05 | -0.0611 | 782 | 0.253 |
| ENSP00000265447 | 6.80E-05 | -0.0611 | 499 | 0.332 |
| ENSP00000354687 | 2.53E-05 | -0.0611 | 806 | 0.725 |
| ENSP00000302728 | 2.96E-05 | -0.0611 | 251 | 0.441 |
| ENSP00000169298 | 2.50E-05 | -0.0611 | 204 | 0.149 |
| ENSP00000359573 | 3.42E-05 | -0.0612 | 182 | 0.711 |
| ENSP00000229307 | 6.08E-05 | -0.0612 | 352 | 0.864 |

| | | | | |
|---|---|---|---|---|
| ENSP00000302232 | 1.84E-05 | -0.0612 | 0 | 0.301 |
| ENSP00000338487 | 4.72E-05 | -0.0612 | 159 | 0.053 |
| ENSP00000360116 | 4.60E-05 | -0.0612 | 223 | 0.835 |
| ENSP00000363431 | 1.18E-05 | -0.0613 | 190 | 0.138 |
| ENSP00000251588 | 2.41E-05 | -0.0613 | 150 | 0.286 |
| ENSP00000417185 | 2.95E-05 | -0.0613 | 242 | 0.722 |
| ENSP00000304593 | 2.60E-05 | -0.0613 | 387 | 0.866 |
| ENSP00000330753 | 2.71E-05 | -0.0613 | 246 | 0.930 |
| ENSP00000200457 | 2.87E-05 | -0.0613 | 542 | 0.663 |
| ENSP00000258412 | 3.10E-05 | -0.0613 | 483 | 0.084 |
| ENSP00000370648 | 2.57E-05 | -0.0613 | 300 | 0.160 |
| ENSP00000332973 | 5.74E-05 | -0.0613 | 942 | 0.909 |
| ENSP00000261180 | 2.11E-05 | -0.0613 | 658 | 0.357 |
| ENSP00000257017 | 1.81E-05 | -0.0613 | 284 | 0.248 |
| ENSP00000311596 | 2.70E-05 | -0.0613 | 280 | 0.808 |
| ENSP00000290846 | 2.71E-05 | -0.0613 | 334 | 0.490 |
| ENSP00000285083 | 2.95E-05 | -0.0614 | 193 | 0.234 |
| ENSP00000308227 | 2.19E-05 | -0.0614 | 575 | 0.757 |
| ENSP00000317955 | 2.97E-05 | -0.0614 | 594 | 0.383 |
| ENSP00000291688 | 3.25E-05 | -0.0614 | 165 | 0.550 |
| ENSP00000262030 | 1.81E-05 | -0.0614 | 496 | 0.639 |
| ENSP00000257622 | 4.51E-05 | -0.0614 | 173 | 0.000 |
| ENSP00000297625 | 2.93E-05 | -0.0614 | 173 | 0.086 |
| ENSP00000270139 | 2.97E-05 | -0.0615 | 163 | 0.806 |
| ENSP00000244537 | 2.14E-05 | -0.0615 | 774 | 0.000 |
| ENSP00000347581 | 1.56E-05 | -0.0615 | 0 | 0.094 |
| ENSP00000381089 | 9.88E-06 | -0.0615 | 0 | 0.062 |
| ENSP00000216807 | 3.91E-05 | -0.0616 | 242 | 0.770 |
| ENSP00000287820 | 5.43E-05 | -0.0616 | 942 | 0.916 |
| ENSP00000334319 | 3.33E-05 | -0.0616 | 396 | 0.164 |
| ENSP00000393262 | 5.92E-05 | -0.0616 | 700 | 0.000 |
| ENSP00000389658 | 4.56E-06 | -0.0616 | 0 | 0.000 |
| ENSP00000223051 | 3.65E-05 | -0.0616 | 396 | 0.415 |
| ENSP00000240189 | 2.29E-03 | -0.0616 | 0 | 0.199 |
| ENSP00000235310 | 3.13E-05 | -0.0616 | 201 | 0.624 |
| ENSP00000307197 | 2.31E-05 | -0.0616 | 894 | 0.431 |
| ENSP00000325290 | 3.42E-05 | -0.0616 | 223 | 0.409 |
| ENSP00000409950 | 1.38E-07 | -0.0617 | 0 | 0.208 |
| ENSP00000272902 | 2.47E-05 | -0.0617 | 157 | 0.523 |
| ENSP00000287908 | 4.21E-05 | -0.0617 | 176 | 0.071 |
| ENSP00000272190 | 4.52E-05 | -0.0617 | 581 | 0.924 |
| ENSP00000318486 | 2.82E-05 | -0.0617 | 234 | 0.733 |
| ENSP00000271332 | 3.38E-05 | -0.0617 | 321 | 0.386 |
| ENSP00000268206 | 1.79E-05 | -0.0617 | 399 | 0.859 |
| ENSP00000263274 | 2.09E-05 | -0.0618 | 171 | 0.797 |
| ENSP00000328269 | 3.32E-05 | -0.0618 | 649 | 0.927 |

| | | | | |
|---|---|---|---|---|
| ENSP00000361502 | 1.62E-05 | -0.0618 | 158 | 0.076 |
| ENSP00000262878 | 3.86E-05 | -0.0618 | 403 | 0.306 |
| ENSP00000285900 | 3.64E-05 | -0.0618 | 589 | 0.690 |
| ENSP00000326767 | 3.77E-05 | -0.0618 | 313 | 0.672 |
| ENSP00000345684 | 1.41E-05 | -0.0619 | 0 | 0.000 |
| ENSP00000223321 | 1.99E-05 | -0.0619 | 656 | 0.517 |
| ENSP00000368667 | 3.92E-05 | -0.0619 | 330 | 0.702 |
| ENSP00000215780 | 1.71E-05 | -0.0619 | 336 | 0.000 |
| ENSP00000286788 | 2.41E-05 | -0.0620 | 385 | 0.747 |
| ENSP00000310901 | 4.75E-05 | -0.0620 | 360 | 0.416 |
| ENSP00000427941 | 1.76E-05 | -0.0620 | 0 | 0.055 |
| ENSP00000219409 | 2.35E-05 | -0.0620 | 178 | 0.387 |
| ENSP00000339398 | 5.30E-05 | -0.0620 | 0 | 0.772 |
| ENSP00000301790 | 1.64E-05 | -0.0620 | 0 | 0.074 |
| ENSP00000361748 | 1.18E-05 | -0.0620 | 0 | 0.092 |
| ENSP00000269305 | 5.07E-05 | -0.0621 | 942 | 0.000 |
| ENSP00000379383 | 3.92E-05 | -0.0621 | 416 | 0.361 |
| ENSP00000262644 | 3.35E-05 | -0.0621 | 223 | 0.119 |
| ENSP00000301729 | 2.13E-05 | -0.0621 | 300 | 0.380 |
| ENSP00000361768 | 8.64E-06 | -0.0621 | 0 | 0.069 |
| ENSP00000252011 | 1.88E-05 | -0.0621 | 272 | 0.921 |
| ENSP00000467396 | 1.88E-05 | -0.0621 | 244 | 0.000 |
| ENSP00000258774 | 2.89E-05 | -0.0621 | 165 | 0.679 |
| ENSP00000305416 | 4.24E-05 | -0.0622 | 329 | 0.765 |
| ENSP00000317891 | 3.35E-05 | -0.0622 | 202 | 0.594 |
| ENSP00000292069 | 1.87E-05 | -0.0622 | 210 | 0.674 |
| ENSP00000415769 | 4.75E-05 | -0.0622 | 363 | 0.548 |
| ENSP00000264634 | 5.22E-05 | -0.0622 | 951 | 0.000 |
| ENSP00000366787 | 9.77E-06 | -0.0623 | 0 | 0.000 |
| ENSP00000263991 | 2.35E-05 | -0.0623 | 527 | 0.491 |
| ENSP00000310338 | 2.43E-05 | -0.0623 | 0 | 0.161 |
| ENSP00000311489 | 3.83E-05 | -0.0623 | 902 | 0.716 |
| ENSP00000013807 | 1.98E-05 | -0.0624 | 181 | 0.733 |
| ENSP00000334148 | 1.23E-05 | -0.0624 | 0 | 0.127 |
| ENSP00000257904 | 2.47E-05 | -0.0624 | 420 | 0.000 |
| ENSP00000310491 | 3.24E-05 | -0.0624 | 199 | 0.616 |
| ENSP00000296388 | 1.10E-04 | -0.0624 | 185 | 0.000 |
| ENSP00000231357 | 2.43E-05 | -0.0624 | 270 | 0.791 |
| ENSP00000318142 | 3.22E-05 | -0.0625 | 470 | 0.245 |
| ENSP00000374152 | 2.11E-05 | -0.0625 | 246 | 0.944 |
| ENSP00000262061 | 2.56E-05 | -0.0625 | 179 | 0.109 |
| ENSP00000246533 | 2.26E-05 | -0.0625 | 165 | 0.279 |
| ENSP00000259803 | 4.53E-05 | -0.0625 | 213 | 0.779 |
| ENSP00000265379 | 4.56E-05 | -0.0625 | 204 | 0.238 |
| ENSP00000324948 | 2.91E-05 | -0.0626 | 240 | 0.866 |
| ENSP00000278829 | 8.45E-05 | -0.0626 | 235 | 0.294 |

| | | | | |
|---|---|---|---|---|
| ENSP00000329380 | 2.99E-05 | -0.0626 | 999 | 0.816 |
| ENSP00000274764 | 1.94E-05 | -0.0627 | 837 | 0.959 |
| ENSP00000221455 | 2.23E-05 | -0.0627 | 179 | 0.294 |
| ENSP00000318602 | 2.24E-05 | -0.0627 | 212 | 0.178 |
| ENSP00000370750 | 2.99E-05 | -0.0627 | 975 | 0.241 |
| ENSP00000332449 | 6.33E-05 | -0.0627 | 518 | 0.217 |
| ENSP00000358565 | 8.74E-06 | -0.0628 | 0 | 0.064 |
| ENSP00000324573 | 2.18E-05 | -0.0628 | 810 | 0.805 |
| ENSP00000417653 | 3.81E-05 | -0.0628 | 779 | 0.462 |
| ENSP00000296486 | 3.09E-05 | -0.0629 | 183 | 0.000 |
| ENSP00000324287 | 4.93E-05 | -0.0629 | 242 | 0.266 |
| ENSP00000324191 | 9.96E-06 | -0.0629 | 0 | 0.076 |
| ENSP00000256707 | 2.24E-05 | -0.0629 | 254 | 0.777 |
| ENSP00000340328 | 1.85E-05 | -0.0629 | 396 | 0.704 |
| ENSP00000340278 | 3.29E-05 | -0.0630 | 408 | 0.758 |
| ENSP00000328596 | 3.61E-05 | -0.0630 | 261 | 0.824 |
| ENSP00000382318 | 1.62E-07 | -0.0630 | 0 | 0.000 |
| ENSP00000251020 | 2.41E-05 | -0.0630 | 150 | 0.822 |
| ENSP00000301904 | 2.64E-05 | -0.0631 | 156 | 0.255 |
| ENSP00000254035 | 2.59E-05 | -0.0631 | 347 | 0.171 |
| ENSP00000226209 | 2.11E-05 | -0.0631 | 901 | 0.000 |
| ENSP00000309031 | 5.72E-05 | -0.0631 | 317 | 0.084 |
| ENSP00000306772 | 3.30E-05 | -0.0631 | 227 | 0.791 |
| ENSP00000413196 | 9.78E-08 | -0.0632 | 0 | 0.198 |
| ENSP00000294244 | 1.65E-03 | -0.0632 | 0 | 0.188 |
| ENSP00000345470 | 1.11E-05 | -0.0632 | 0 | 0.128 |
| ENSP00000375234 | 4.38E-07 | -0.0632 | 0 | 0.209 |
| ENSP00000312309 | 7.09E-05 | -0.0632 | 214 | 0.000 |
| ENSP00000299238 | 5.53E-05 | -0.0632 | 198 | 0.076 |
| ENSP00000252723 | 1.03E-04 | -0.0632 | 294 | 0.899 |
| ENSP00000252951 | 2.45E-05 | -0.0632 | 153 | 0.504 |
| ENSP00000354742 | 3.09E-05 | -0.0633 | 400 | 0.494 |
| ENSP00000244496 | 2.35E-05 | -0.0633 | 174 | 0.849 |
| ENSP00000286827 | 2.26E-05 | -0.0633 | 264 | 0.793 |
| ENSP00000181839 | 1.70E-05 | -0.0633 | 172 | 0.859 |
| ENSP00000207437 | 2.04E-05 | -0.0633 | 212 | 0.000 |
| ENSP00000346402 | 3.02E-05 | -0.0633 | 358 | 0.631 |
| ENSP00000259253 | 3.12E-05 | -0.0634 | 377 | 0.206 |
| ENSP00000377686 | 2.18E-05 | -0.0634 | 561 | 0.691 |
| ENSP00000264160 | 1.06E-05 | -0.0634 | 0 | 0.159 |
| ENSP00000275766 | 8.17E-04 | -0.0634 | 0 | 0.176 |
| ENSP00000331368 | 1.91E-05 | -0.0634 | 284 | 0.493 |
| ENSP00000328335 | 7.65E-02 | -0.0634 | 0 | 0.216 |
| ENSP00000328768 | 7.65E-02 | -0.0634 | 0 | 0.216 |
| ENSP00000341333 | 7.65E-02 | -0.0634 | 0 | 0.000 |
| ENSP00000341988 | 7.65E-02 | -0.0634 | 0 | 0.216 |

| | | | | |
|---|---|---|---|---|
| ENSP00000347211 | 7.05E-06 | -0.0634 | 0 | 0.000 |
| ENSP00000358228 | 7.05E-06 | -0.0634 | 0 | 0.000 |
| ENSP00000358372 | 7.05E-06 | -0.0634 | 0 | 0.000 |
| ENSP00000365266 | 7.05E-06 | -0.0634 | 0 | 0.211 |
| ENSP00000365289 | 7.05E-06 | -0.0634 | 0 | 0.000 |
| ENSP00000394705 | 7.05E-06 | -0.0634 | 0 | 0.000 |
| ENSP00000421868 | 7.05E-06 | -0.0634 | 0 | 0.216 |
| ENSP00000431179 | 7.05E-06 | -0.0634 | 0 | 0.000 |
| ENSP00000335004 | 2.54E-04 | -0.0634 | 150 | 0.248 |
| ENSP00000315614 | 2.87E-03 | -0.0634 | 0 | 0.000 |
| ENSP00000235347 | 7.20E-04 | -0.0634 | 0 | 0.201 |
| ENSP00000295658 | 4.44E-04 | -0.0635 | 0 | 0.183 |
| ENSP00000263431 | 1.69E-05 | -0.0635 | 900 | 0.658 |
| ENSP00000320232 | 6.58E-04 | -0.0635 | 0 | 0.197 |
| ENSP00000306991 | 4.03E-05 | -0.0635 | 846 | 0.710 |
| ENSP00000360682 | 7.05E-06 | -0.0635 | 0 | 0.211 |
| ENSP00000403130 | 7.05E-06 | -0.0635 | 0 | 0.216 |
| ENSP00000409009 | 7.05E-06 | -0.0635 | 0 | 0.217 |
| ENSP00000369986 | 1.93E-06 | -0.0635 | 0 | 0.000 |
| ENSP00000363529 | 7.05E-06 | -0.0635 | 0 | 0.216 |
| ENSP00000361765 | 7.05E-06 | -0.0635 | 0 | 0.212 |
| ENSP00000207870 | 2.92E-05 | -0.0635 | 159 | 0.119 |
| ENSP00000366347 | 2.57E-05 | -0.0635 | 180 | 0.863 |
| ENSP00000326411 | 3.36E-05 | -0.0635 | 317 | 0.562 |
| ENSP00000381857 | 7.05E-06 | -0.0635 | 0 | 0.216 |
| ENSP00000268679 | 2.98E-05 | -0.0635 | 185 | 0.790 |
| ENSP00000365302 | 7.05E-06 | -0.0635 | 0 | 0.000 |
| ENSP00000219919 | 1.72E-05 | -0.0636 | 193 | 0.287 |
| ENSP00000218439 | 2.90E-05 | -0.0636 | 181 | 0.474 |
| ENSP00000402301 | 3.61E-04 | -0.0636 | 201 | 0.265 |
| ENSP00000355778 | 2.57E-05 | -0.0636 | 702 | 0.939 |
| ENSP00000270310 | 4.91E-05 | -0.0636 | 562 | 0.140 |
| ENSP00000342075 | 1.73E-04 | -0.0636 | 0 | 0.256 |
| ENSP00000221671 | 1.81E-03 | -0.0636 | 0 | 0.208 |
| ENSP00000335041 | 1.06E-04 | -0.0637 | 0 | 0.137 |
| ENSP00000340935 | 3.05E-05 | -0.0637 | 242 | 0.554 |
| ENSP00000300035 | 8.46E-05 | -0.0637 | 457 | 0.800 |
| ENSP00000355437 | 3.24E-05 | -0.0637 | 163 | 0.145 |
| ENSP00000233025 | 2.16E-05 | -0.0637 | 172 | 0.516 |
| ENSP00000306106 | 5.94E-05 | -0.0637 | 186 | 0.118 |
| ENSP00000339324 | 1.51E-04 | -0.0637 | 0 | 0.182 |
| ENSP00000305426 | 2.61E-05 | -0.0638 | 282 | 0.588 |
| ENSP00000363516 | 7.08E-06 | -0.0638 | 0 | 0.189 |
| ENSP00000316527 | 1.01E-04 | -0.0638 | 0 | 0.251 |
| ENSP00000323199 | 3.74E-04 | -0.0638 | 0 | 0.180 |
| ENSP00000306320 | 2.16E-04 | -0.0638 | 0 | 0.157 |

| | | | | |
|---|---|---|---|---|
| ENSP00000305263 | 2.07E-04 | -0.0638 | 0 | 0.163 |
| ENSP00000332134 | 2.08E-04 | -0.0639 | 0 | 0.261 |
| ENSP00000354842 | 3.04E-05 | -0.0639 | 269 | 0.869 |
| ENSP00000365576 | 6.71E-04 | -0.0639 | 577 | 0.795 |
| ENSP00000386444 | 7.50E-05 | -0.0639 | 999 | 0.893 |
| ENSP00000283351 | 3.19E-05 | -0.0639 | 305 | 0.119 |
| ENSP00000361596 | 8.67E-06 | -0.0639 | 0 | 0.213 |
| ENSP00000215531 | 1.42E-04 | -0.0639 | 0 | 0.000 |
| ENSP00000264229 | 3.46E-04 | -0.0639 | 0 | 0.130 |
| ENSP00000405965 | 6.82E-03 | -0.0639 | 600 | 0.000 |
| ENSP00000338641 | 1.25E-04 | -0.0639 | 0 | 0.197 |
| ENSP00000300650 | 1.27E-04 | -0.0639 | 0 | 0.141 |
| ENSP00000168977 | 2.60E-05 | -0.0639 | 277 | 0.167 |
| ENSP00000331363 | 1.55E-04 | -0.0639 | 0 | 0.166 |
| ENSP00000282074 | 3.01E-05 | -0.0639 | 268 | 0.238 |
| ENSP00000266126 | 2.31E-05 | -0.0639 | 459 | 0.539 |
| ENSP00000222005 | 1.67E-04 | -0.0639 | 377 | 0.644 |
| ENSP00000342938 | 2.25E-04 | -0.0640 | 0 | 0.134 |
| ENSP00000339374 | 6.89E-05 | -0.0640 | 0 | 0.112 |
| ENSP00000330237 | 2.38E-05 | -0.0640 | 577 | 0.871 |
| ENSP00000276416 | 5.17E-05 | -0.0640 | 258 | 0.118 |
| ENSP00000367787 | 3.58E-05 | -0.0640 | 315 | 0.663 |
| ENSP00000416387 | 2.34E-05 | -0.0640 | 152 | 0.412 |
| ENSP00000288087 | 7.82E-05 | -0.0640 | 0 | 0.100 |
| ENSP00000342098 | 2.76E-05 | -0.0640 | 342 | 0.147 |
| ENSP00000267502 | 3.78E-05 | -0.0640 | 625 | 0.343 |
| ENSP00000328364 | 4.91E-05 | -0.0640 | 0 | 0.618 |
| ENSP00000339186 | 2.61E-05 | -0.0640 | 167 | 0.836 |
| ENSP00000262812 | 2.90E-05 | -0.0640 | 259 | 0.159 |
| ENSP00000318075 | 8.68E-05 | -0.0640 | 0 | 0.132 |
| ENSP00000265016 | 2.94E-05 | -0.0640 | 563 | 0.180 |
| ENSP00000366280 | 1.33E-05 | -0.0640 | 214 | 0.102 |
| ENSP00000289269 | 8.72E-05 | -0.0640 | 0 | 0.106 |
| ENSP00000343891 | 6.66E-05 | -0.0641 | 0 | 0.114 |
| ENSP00000226225 | 1.10E-05 | -0.0641 | 0 | 0.093 |
| ENSP00000311398 | 7.92E-06 | -0.0641 | 0 | 0.116 |
| ENSP00000321691 | 9.34E-05 | -0.0641 | 0 | 0.131 |
| ENSP00000315474 | 6.88E-05 | -0.0641 | 0 | 0.176 |
| ENSP00000270538 | 2.82E-05 | -0.0641 | 358 | 0.282 |
| ENSP00000263073 | 3.10E-05 | -0.0641 | 237 | 0.815 |
| ENSP00000295079 | 8.30E-05 | -0.0641 | 0 | 0.181 |
| ENSP00000299705 | 2.17E-05 | -0.0641 | 155 | 0.177 |
| ENSP00000267996 | 6.15E-05 | -0.0641 | 916 | 0.721 |
| ENSP00000264031 | 7.97E-05 | -0.0641 | 0 | 0.212 |
| ENSP00000280704 | 1.86E-05 | -0.0641 | 397 | 0.551 |
| ENSP00000309907 | 1.17E-04 | -0.0641 | 0 | 0.000 |

| | | | | |
|---|---|---|---|---|
| ENSP00000463159 | 5.99E-06 | -0.0641 | 0 | 0.203 |
| ENSP00000310057 | 1.06E-04 | -0.0642 | 0 | 0.203 |
| ENSP00000230419 | 1.82E-05 | -0.0642 | 273 | 0.662 |
| ENSP00000293662 | 2.14E-05 | -0.0642 | 284 | 0.403 |
| ENSP00000262982 | 2.53E-05 | -0.0642 | 159 | 0.700 |
| ENSP00000259470 | 2.62E-05 | -0.0642 | 403 | 0.000 |
| ENSP00000286031 | 1.30E-04 | -0.0642 | 0 | 0.155 |
| ENSP00000326706 | 4.49E-05 | -0.0642 | 0 | 0.212 |
| ENSP00000259608 | 5.23E-05 | -0.0642 | 0 | 0.132 |
| ENSP00000383407 | 1.33E-06 | -0.0643 | 0 | 0.000 |
| ENSP00000295124 | 1.60E-04 | -0.0643 | 0 | 0.096 |
| ENSP00000301284 | 3.82E-05 | -0.0643 | 151 | 0.000 |
| ENSP00000261434 | 2.47E-05 | -0.0643 | 394 | 0.359 |
| ENSP00000298552 | 2.91E-05 | -0.0643 | 163 | 0.819 |
| ENSP00000300283 | 2.31E-05 | -0.0643 | 347 | 0.177 |
| ENSP00000303356 | 2.55E-05 | -0.0643 | 194 | 0.310 |
| ENSP00000314619 | 2.98E-05 | -0.0643 | 215 | 0.399 |
| ENSP00000309189 | 2.09E-04 | -0.0643 | 0 | 0.121 |
| ENSP00000299518 | 1.99E-05 | -0.0643 | 197 | 0.554 |
| ENSP00000295641 | 5.00E-05 | -0.0644 | 482 | 0.343 |
| ENSP00000303427 | 2.33E-05 | -0.0644 | 203 | 0.576 |
| ENSP00000441954 | 4.88E-05 | -0.0644 | 525 | 0.891 |
| ENSP00000279206 | 7.72E-05 | -0.0644 | 0 | 0.093 |
| ENSP00000366999 | 2.11E-05 | -0.0644 | 800 | 0.955 |
| ENSP00000217999 | 6.56E-06 | -0.0644 | 0 | 0.691 |
| ENSP00000342222 | 2.57E-05 | -0.0644 | 302 | 0.455 |
| ENSP00000333568 | 2.23E-05 | -0.0644 | 282 | 0.815 |
| ENSP00000292090 | 5.90E-05 | -0.0644 | 0 | 0.103 |
| ENSP00000086933 | 7.51E-06 | -0.0644 | 0 | 0.701 |
| ENSP00000350378 | 1.23E-05 | -0.0644 | 0 | 0.092 |
| ENSP00000312367 | 4.29E-05 | -0.0644 | 0 | 0.346 |
| ENSP00000338288 | 7.72E-05 | -0.0644 | 0 | 0.000 |
| ENSP00000385045 | 9.95E-05 | -0.0645 | 908 | 0.769 |
| ENSP00000416015 | 4.37E-05 | -0.0645 | 338 | 0.478 |
| ENSP00000435096 | 4.18E-05 | -0.0645 | 171 | 0.757 |
| ENSP00000233616 | 2.31E-05 | -0.0645 | 445 | 0.295 |
| ENSP00000264708 | 3.93E-05 | -0.0645 | 873 | 0.919 |
| ENSP00000314518 | 3.32E-05 | -0.0645 | 0 | 0.094 |
| ENSP00000311496 | 2.76E-05 | -0.0645 | 205 | 0.240 |
| ENSP00000296679 | 5.06E-05 | -0.0645 | 0 | 0.145 |
| ENSP00000395505 | 2.96E-05 | -0.0645 | 191 | 0.156 |
| ENSP00000330825 | 3.63E-05 | -0.0645 | 252 | 0.718 |
| ENSP00000432561 | 4.55E-05 | -0.0645 | 282 | 0.698 |
| ENSP00000349931 | 2.19E-06 | -0.0645 | 0 | 0.189 |
| ENSP00000331397 | 4.70E-05 | -0.0645 | 0 | 0.172 |
| ENSP00000310573 | 7.15E-05 | -0.0646 | 0 | 0.139 |

| | | | | |
|---|---|---|---|---|
| ENSP00000382323 | 1.80E-05 | -0.0646 | 0 | 0.131 |
| ENSP00000245934 | 4.11E-05 | -0.0646 | 167 | 0.536 |
| ENSP00000286067 | 8.71E-05 | -0.0646 | 0 | 0.314 |
| ENSP00000292524 | 7.21E-05 | -0.0646 | 0 | 0.154 |
| ENSP00000321029 | 4.54E-05 | -0.0646 | 0 | 0.134 |
| ENSP00000217420 | 2.82E-05 | -0.0646 | 195 | 0.340 |
| ENSP00000301678 | 4.76E-05 | -0.0646 | 0 | 0.000 |
| ENSP00000327691 | 2.20E-05 | -0.0647 | 288 | 0.000 |
| ENSP00000280083 | 8.13E-05 | -0.0647 | 0 | 0.182 |
| ENSP00000451575 | 1.39E-04 | -0.0647 | 324 | 0.690 |
| ENSP00000265465 | 2.39E-05 | -0.0647 | 154 | 0.665 |
| ENSP00000224073 | 2.22E-05 | -0.0647 | 152 | 0.490 |
| ENSP00000196371 | 1.85E-05 | -0.0647 | 480 | 0.256 |
| ENSP00000274605 | 1.16E-04 | -0.0647 | 0 | 0.153 |
| ENSP00000265978 | 5.41E-05 | -0.0647 | 0 | 0.158 |
| ENSP00000324404 | 3.78E-05 | -0.0647 | 186 | 0.100 |
| ENSP00000266022 | 4.61E-05 | -0.0647 | 0 | 0.109 |
| ENSP00000341957 | 3.61E-05 | -0.0647 | 810 | 0.729 |
| ENSP00000262177 | 1.72E-05 | -0.0647 | 904 | 0.475 |
| ENSP00000264448 | 2.27E-05 | -0.0647 | 606 | 0.000 |
| ENSP00000331845 | 3.93E-05 | -0.0647 | 0 | 0.140 |
| ENSP00000339637 | 5.20E-05 | -0.0647 | 0 | 0.244 |
| ENSP00000250340 | 5.02E-05 | -0.0647 | 0 | 0.138 |
| ENSP00000261250 | 6.24E-05 | -0.0647 | 0 | 0.135 |
| ENSP00000270502 | 4.49E-05 | -0.0647 | 0 | 0.081 |
| ENSP00000322469 | 8.21E-05 | -0.0648 | 0 | 0.171 |
| ENSP00000339634 | 3.09E-05 | -0.0648 | 0 | 0.132 |
| ENSP00000340668 | 4.42E-05 | -0.0648 | 0 | 0.138 |
| ENSP00000358038 | 2.29E-05 | -0.0648 | 167 | 0.211 |
| ENSP00000313226 | 5.64E-05 | -0.0648 | 0 | 0.172 |
| ENSP00000299191 | 4.85E-03 | -0.0648 | 0 | 0.204 |
| ENSP00000322229 | 4.29E-05 | -0.0648 | 328 | 0.582 |
| ENSP00000244050 | 4.40E-05 | -0.0648 | 921 | 0.932 |
| ENSP00000263256 | 1.08E-04 | -0.0648 | 0 | 0.139 |
| ENSP00000251343 | 9.26E-05 | -0.0648 | 0 | 0.144 |
| ENSP00000329360 | 3.21E-05 | -0.0648 | 0 | 0.086 |
| ENSP00000316681 | 5.13E-05 | -0.0648 | 0 | 0.000 |
| ENSP00000297205 | 4.63E-05 | -0.0649 | 0 | 0.110 |
| ENSP00000278426 | 4.87E-05 | -0.0649 | 0 | 0.160 |
| ENSP00000357516 | 5.95E-05 | -0.0649 | 915 | 0.729 |
| ENSP00000342499 | 5.68E-05 | -0.0649 | 0 | 0.089 |
| ENSP00000288602 | 2.01E-05 | -0.0649 | 277 | 0.848 |
| ENSP00000197268 | 4.51E-05 | -0.0649 | 0 | 0.000 |
| ENSP00000307887 | 4.94E-05 | -0.0649 | 182 | 0.145 |
| ENSP00000268314 | 4.30E-05 | -0.0649 | 0 | 0.244 |
| ENSP00000309233 | 1.76E-04 | -0.0649 | 0 | 0.170 |

| | | | | |
|---|---|---|---|---|
| ENSP00000301659 | 4.35E-05 | -0.0649 | 0 | 0.110 |
| ENSP00000310260 | 2.03E-05 | -0.0649 | 561 | 0.219 |
| ENSP00000344655 | 4.04E-05 | -0.0649 | 0 | 0.000 |
| ENSP00000239367 | 5.20E-05 | -0.0649 | 0 | 0.196 |
| ENSP00000249014 | 6.73E-05 | -0.0649 | 0 | 0.121 |
| ENSP00000253108 | 2.02E-05 | -0.0649 | 567 | 0.896 |
| ENSP00000303987 | 4.77E-05 | -0.0650 | 0 | 0.116 |
| ENSP00000293845 | 4.33E-05 | -0.0650 | 0 | 0.119 |
| ENSP00000327994 | 3.19E-05 | -0.0650 | 0 | 0.122 |
| ENSP00000374037 | 2.24E-05 | -0.0650 | 215 | 0.203 |
| ENSP00000297532 | 5.63E-05 | -0.0650 | 0 | 0.189 |
| ENSP00000341324 | 3.63E-05 | -0.0650 | 0 | 0.095 |
| ENSP00000448789 | 1.24E-07 | -0.0650 | 0 | 0.219 |
| ENSP00000319006 | 2.95E-05 | -0.0650 | 0 | 0.419 |
| ENSP00000303490 | 5.52E-05 | -0.0650 | 0 | 0.161 |
| ENSP00000309576 | 2.97E-05 | -0.0650 | 396 | 0.098 |
| ENSP00000321427 | 2.59E-05 | -0.0650 | 175 | 0.138 |
| ENSP00000313046 | 3.58E-05 | -0.0650 | 241 | 0.000 |
| ENSP00000343990 | 3.25E-05 | -0.0651 | 0 | 0.121 |
| ENSP00000341565 | 3.20E-05 | -0.0651 | 0 | 0.141 |
| ENSP00000229340 | 3.28E-05 | -0.0651 | 451 | 0.148 |
| ENSP00000328478 | 3.77E-05 | -0.0651 | 302 | 0.385 |
| ENSP00000362446 | 1.45E-05 | -0.0651 | 242 | 0.740 |
| ENSP00000309561 | 3.81E-05 | -0.0651 | 0 | 0.121 |
| ENSP00000312158 | 3.79E-05 | -0.0651 | 0 | 0.144 |
| ENSP00000338989 | 3.30E-05 | -0.0651 | 576 | 0.000 |
| ENSP00000317175 | 4.19E-05 | -0.0651 | 0 | 0.157 |
| ENSP00000221859 | 1.82E-05 | -0.0651 | 272 | 0.825 |
| ENSP00000340896 | 3.00E-05 | -0.0651 | 265 | 0.884 |
| ENSP00000316029 | 4.63E-05 | -0.0651 | 927 | 0.703 |
| ENSP00000269053 | 3.62E-05 | -0.0651 | 0 | 0.097 |
| ENSP00000337632 | 3.03E-05 | -0.0652 | 670 | 0.452 |
| ENSP00000310861 | 2.48E-05 | -0.0652 | 0 | 0.136 |
| ENSP00000341422 | 2.57E-05 | -0.0652 | 305 | 0.332 |
| ENSP00000232888 | 1.74E-05 | -0.0652 | 644 | 0.888 |
| ENSP00000360538 | 2.42E-05 | -0.0652 | 221 | 0.453 |
| ENSP00000242784 | 5.08E-05 | -0.0652 | 0 | 0.240 |
| ENSP00000244926 | 4.69E-05 | -0.0652 | 0 | 0.173 |
| ENSP00000259229 | 6.55E-05 | -0.0652 | 0 | 0.163 |
| ENSP00000292246 | 2.44E-05 | -0.0652 | 230 | 0.176 |
| ENSP00000274364 | 3.77E-05 | -0.0652 | 292 | 0.353 |
| ENSP00000326884 | 2.78E-05 | -0.0652 | 300 | 0.000 |
| ENSP00000230658 | 4.74E-05 | -0.0652 | 323 | 0.861 |
| ENSP00000267199 | 2.55E-05 | -0.0652 | 644 | 0.215 |
| ENSP00000258145 | 2.09E-05 | -0.0652 | 198 | 0.316 |
| ENSP00000300976 | 3.90E-05 | -0.0652 | 0 | 0.158 |

| | | | | |
|---|---|---|---|---|
| ENSP00000330587 | 3.31E-05 | -0.0652 | 0 | 0.091 |
| ENSP00000264474 | 7.76E-05 | -0.0652 | 254 | 0.192 |
| ENSP00000229633 | 6.09E-05 | -0.0652 | 0 | 0.163 |
| ENSP00000328426 | 3.74E-05 | -0.0652 | 0 | 0.152 |
| ENSP00000366098 | 9.73E-06 | -0.0652 | 0 | 0.106 |
| ENSP00000301242 | 2.23E-05 | -0.0652 | 207 | 0.538 |
| ENSP00000346508 | 1.78E-04 | -0.0652 | 644 | 0.925 |
| ENSP00000372651 | 5.70E-06 | -0.0652 | 0 | 0.000 |
| ENSP00000276603 | 5.00E-05 | -0.0652 | 159 | 0.703 |
| ENSP00000318185 | 4.43E-05 | -0.0652 | 0 | 0.117 |
| ENSP00000282633 | 3.93E-05 | -0.0652 | 0 | 0.132 |
| ENSP00000364739 | 1.08E-06 | -0.0652 | 0 | 0.000 |
| ENSP00000231173 | 4.71E-05 | -0.0652 | 0 | 0.129 |
| ENSP00000298784 | 4.44E-05 | -0.0652 | 0 | 0.241 |
| ENSP00000325414 | 6.86E-05 | -0.0652 | 0 | 0.177 |
| ENSP00000258969 | 2.47E-05 | -0.0653 | 363 | 0.752 |
| ENSP00000289805 | 3.31E-05 | -0.0653 | 0 | 0.136 |
| ENSP00000223336 | 6.69E-05 | -0.0653 | 0 | 0.144 |
| ENSP00000302077 | 9.42E-05 | -0.0653 | 0 | 0.198 |
| ENSP00000299427 | 2.35E-05 | -0.0653 | 418 | 0.165 |
| ENSP00000322170 | 2.12E-05 | -0.0653 | 213 | 0.458 |
| ENSP00000333193 | 2.18E-05 | -0.0653 | 163 | 0.294 |
| ENSP00000262764 | 1.93E-05 | -0.0653 | 298 | 0.371 |
| ENSP00000309230 | 3.49E-05 | -0.0653 | 151 | 0.155 |
| ENSP00000301219 | 1.12E-05 | -0.0653 | 0 | 0.000 |
| ENSP00000261888 | 3.52E-05 | -0.0653 | 0 | 0.178 |
| ENSP00000294517 | 2.68E-05 | -0.0653 | 316 | 0.000 |
| ENSP00000244601 | 1.60E-05 | -0.0653 | 898 | 0.000 |
| ENSP00000471878 | 8.35E-08 | -0.0653 | 0 | 0.000 |
| ENSP00000309096 | 2.27E-05 | -0.0653 | 901 | 0.000 |
| ENSP00000251166 | 3.90E-05 | -0.0653 | 0 | 0.105 |
| ENSP00000348799 | 3.79E-05 | -0.0653 | 0 | 0.402 |
| ENSP00000305892 | 3.39E-05 | -0.0653 | 0 | 0.133 |
| ENSP00000305502 | 3.16E-05 | -0.0654 | 312 | 0.198 |
| ENSP00000314396 | 4.27E-05 | -0.0654 | 0 | 0.000 |
| ENSP00000361855 | 2.83E-05 | -0.0654 | 252 | 0.185 |
| ENSP00000265342 | 3.99E-05 | -0.0654 | 0 | 0.134 |
| ENSP00000251038 | 2.14E-05 | -0.0654 | 173 | 0.761 |
| ENSP00000220509 | 2.39E-05 | -0.0654 | 192 | 0.169 |
| ENSP00000262055 | 6.33E-05 | -0.0654 | 0 | 0.108 |
| ENSP00000398273 | 2.06E-05 | -0.0654 | 212 | 0.852 |
| ENSP00000258083 | 6.00E-05 | -0.0654 | 0 | 0.125 |
| ENSP00000296096 | 2.09E-05 | -0.0654 | 242 | 0.651 |
| ENSP00000263341 | 4.18E-05 | -0.0654 | 939 | 0.960 |
| ENSP00000161863 | 1.33E-05 | -0.0654 | 345 | 0.930 |
| ENSP00000316109 | 2.50E-05 | -0.0654 | 250 | 0.283 |

| | | | | |
|---|---|---|---|---|
| ENSP00000307889 | 2.18E-05 | -0.0655 | 203 | 0.851 |
| ENSP00000339007 | 9.63E-05 | -0.0655 | 580 | 0.940 |
| ENSP00000310117 | 3.01E-05 | -0.0655 | 0 | 0.081 |
| ENSP00000194152 | 4.06E-05 | -0.0655 | 0 | 0.128 |
| ENSP00000288873 | 3.36E-05 | -0.0655 | 0 | 0.154 |
| ENSP00000340200 | 2.05E-05 | -0.0655 | 150 | 0.209 |
| ENSP00000313391 | 2.30E-05 | -0.0655 | 184 | 0.668 |
| ENSP00000367926 | 1.17E-07 | -0.0655 | 0 | 0.000 |
| ENSP00000350005 | 2.41E-05 | -0.0655 | 239 | 0.680 |
| ENSP00000286186 | 1.97E-05 | -0.0655 | 181 | 0.797 |
| ENSP00000342604 | 2.94E-05 | -0.0655 | 0 | 0.124 |
| ENSP00000265018 | 3.49E-05 | -0.0655 | 0 | 0.161 |
| ENSP00000295453 | 1.85E-05 | -0.0655 | 157 | 0.215 |
| ENSP00000288757 | 3.04E-05 | -0.0655 | 0 | 0.143 |
| ENSP00000299977 | 2.28E-05 | -0.0655 | 0 | 0.092 |
| ENSP00000412733 | 2.87E-05 | -0.0655 | 427 | 0.141 |
| ENSP00000325785 | 2.18E-05 | -0.0656 | 338 | 0.411 |
| ENSP00000339207 | 2.59E-05 | -0.0656 | 0 | 0.142 |
| ENSP00000231130 | 4.23E-05 | -0.0656 | 0 | 0.123 |
| ENSP00000433511 | 1.91E-05 | -0.0656 | 727 | 0.587 |
| ENSP00000315011 | 7.11E-05 | -0.0656 | 345 | 0.816 |
| ENSP00000303398 | 3.09E-05 | -0.0656 | 0 | 0.464 |
| ENSP00000331258 | 1.73E-05 | -0.0656 | 0 | 0.000 |
| ENSP00000320359 | 5.17E-05 | -0.0656 | 0 | 0.000 |
| ENSP00000330361 | 1.41E-05 | -0.0656 | 0 | 0.215 |
| ENSP00000281871 | 3.17E-05 | -0.0656 | 0 | 0.075 |
| ENSP00000303570 | 3.22E-05 | -0.0656 | 0 | 0.173 |
| ENSP00000240651 | 3.04E-05 | -0.0656 | 0 | 0.096 |
| ENSP00000315386 | 1.95E-05 | -0.0656 | 0 | 0.098 |
| ENSP00000263401 | 5.23E-05 | -0.0656 | 0 | 0.167 |
| ENSP00000287859 | 3.15E-05 | -0.0656 | 0 | 0.129 |
| ENSP00000254691 | 6.49E-05 | -0.0656 | 0 | 0.182 |
| ENSP00000273541 | 2.39E-05 | -0.0656 | 458 | 0.605 |
| ENSP00000333539 | 2.11E-05 | -0.0656 | 0 | 0.627 |
| ENSP00000257901 | 1.55E-05 | -0.0656 | 0 | 0.116 |
| ENSP00000254742 | 5.55E-05 | -0.0656 | 0 | 0.113 |
| ENSP00000222812 | 2.29E-05 | -0.0656 | 377 | 0.257 |
| ENSP00000297533 | 2.90E-05 | -0.0656 | 0 | 0.104 |
| ENSP00000276654 | 2.92E-05 | -0.0656 | 0 | 0.122 |
| ENSP00000339835 | 1.90E-05 | -0.0656 | 702 | 0.960 |
| ENSP00000299952 | 3.21E-05 | -0.0657 | 0 | 0.117 |
| ENSP00000358674 | 7.76E-05 | -0.0657 | 489 | 0.673 |
| ENSP00000297613 | 3.26E-05 | -0.0657 | 0 | 0.471 |
| ENSP00000321737 | 2.62E-05 | -0.0657 | 0 | 0.151 |
| ENSP00000255174 | 5.45E-05 | -0.0657 | 0 | 0.000 |
| ENSP00000321606 | 2.25E-05 | -0.0657 | 836 | 0.397 |

| | | | | |
|---|---|---|---|---|
| ENSP00000326379 | 2.95E-05 | -0.0657 | 0 | 0.432 |
| ENSP00000308137 | 2.08E-05 | -0.0657 | 0 | 0.053 |
| ENSP00000332602 | 2.70E-05 | -0.0657 | 0 | 0.469 |
| ENSP00000377520 | 1.05E-05 | -0.0657 | 0 | 0.081 |
| ENSP00000338706 | 2.94E-05 | -0.0658 | 0 | 0.129 |
| ENSP00000233712 | 4.47E-05 | -0.0658 | 0 | 0.165 |
| ENSP00000268138 | 2.88E-05 | -0.0658 | 0 | 0.507 |
| ENSP00000299633 | 2.96E-05 | -0.0658 | 0 | 0.000 |
| ENSP00000341543 | 2.01E-05 | -0.0658 | 380 | 0.120 |
| ENSP00000264834 | 2.50E-04 | -0.0658 | 214 | 0.816 |
| ENSP00000292140 | 3.12E-05 | -0.0658 | 0 | 0.102 |
| ENSP00000171887 | 2.26E-05 | -0.0658 | 435 | 0.894 |
| ENSP00000460371 | 1.34E-05 | -0.0658 | 227 | 0.174 |
| ENSP00000246672 | 5.71E-05 | -0.0658 | 201 | 0.791 |
| ENSP00000320251 | 2.40E-05 | -0.0658 | 0 | 0.105 |
| ENSP00000340969 | 2.24E-05 | -0.0658 | 0 | 0.124 |
| ENSP00000340761 | 2.50E-05 | -0.0658 | 0 | 0.167 |
| ENSP00000263563 | 2.79E-05 | -0.0658 | 0 | 0.064 |
| ENSP00000216420 | 3.56E-05 | -0.0658 | 0 | 0.139 |
| ENSP00000282884 | 2.56E-05 | -0.0658 | 0 | 0.130 |
| ENSP00000353732 | 7.79E-06 | -0.0658 | 0 | 0.081 |
| ENSP00000344446 | 2.12E-05 | -0.0658 | 0 | 0.181 |
| ENSP00000353735 | 3.25E-05 | -0.0659 | 270 | 0.374 |
| ENSP00000299413 | 2.09E-05 | -0.0659 | 0 | 0.119 |
| ENSP00000338838 | 2.92E-05 | -0.0659 | 0 | 0.000 |
| ENSP00000395465 | 2.58E-05 | -0.0659 | 0 | 0.000 |
| ENSP00000343223 | 2.39E-05 | -0.0659 | 0 | 0.117 |
| ENSP00000216446 | 4.09E-05 | -0.0659 | 0 | 0.120 |
| ENSP00000221418 | 2.08E-05 | -0.0659 | 189 | 0.443 |
| ENSP00000306496 | 2.37E-05 | -0.0659 | 284 | 0.243 |
| ENSP00000309751 | 1.86E-05 | -0.0659 | 0 | 0.090 |
| ENSP00000248098 | 3.87E-05 | -0.0659 | 0 | 0.154 |
| ENSP00000225567 | 1.60E-05 | -0.0659 | 167 | 0.229 |
| ENSP00000337950 | 8.38E-06 | -0.0659 | 0 | 0.000 |
| ENSP00000313140 | 2.50E-05 | -0.0659 | 0 | 0.327 |
| ENSP00000321370 | 2.17E-05 | -0.0659 | 0 | 0.141 |
| ENSP00000277746 | 2.36E-05 | -0.0659 | 0 | 0.109 |
| ENSP00000386935 | 1.06E-05 | -0.0659 | 0 | 0.095 |
| ENSP00000262809 | 2.56E-04 | -0.0659 | 503 | 0.707 |
| ENSP00000376076 | 1.10E-04 | -0.0659 | 627 | 0.845 |
| ENSP00000266736 | 2.21E-05 | -0.0660 | 0 | 0.132 |
| ENSP00000216190 | 1.66E-05 | -0.0660 | 549 | 0.881 |
| ENSP00000228510 | 2.56E-05 | -0.0660 | 254 | 0.427 |
| ENSP00000206474 | 3.09E-05 | -0.0660 | 0 | 0.108 |
| ENSP00000338013 | 2.02E-05 | -0.0660 | 0 | 0.000 |
| ENSP00000239449 | 3.90E-05 | -0.0660 | 0 | 0.100 |

| | | | | |
|---|---|---|---|---|
| ENSP00000334958 | 2.21E-05 | -0.0660 | 0 | 0.118 |
| ENSP00000216802 | 2.63E-05 | -0.0660 | 198 | 0.421 |
| ENSP00000242210 | 2.23E-05 | -0.0660 | 236 | 0.146 |
| ENSP00000325681 | 1.95E-05 | -0.0660 | 0 | 0.125 |
| ENSP00000339992 | 4.66E-05 | -0.0660 | 826 | 0.000 |
| ENSP00000271843 | 2.13E-05 | -0.0661 | 193 | 0.177 |
| ENSP00000335595 | 2.70E-05 | -0.0661 | 0 | 0.094 |
| ENSP00000311500 | 2.56E-05 | -0.0661 | 0 | 0.062 |
| ENSP00000303769 | 2.52E-05 | -0.0661 | 152 | 0.104 |
| ENSP00000298317 | 2.43E-05 | -0.0661 | 236 | 0.718 |
| ENSP00000322249 | 1.97E-05 | -0.0661 | 0 | 0.162 |
| ENSP00000325822 | 2.36E-05 | -0.0661 | 181 | 0.586 |
| ENSP00000302569 | 1.83E-05 | -0.0661 | 177 | 0.479 |
| ENSP00000312606 | 2.38E-05 | -0.0661 | 253 | 0.280 |
| ENSP00000305069 | 2.29E-05 | -0.0661 | 0 | 0.112 |
| ENSP00000307292 | 3.28E-05 | -0.0661 | 0 | 0.226 |
| ENSP00000331843 | 1.73E-05 | -0.0661 | 338 | 0.000 |
| ENSP00000328216 | 1.97E-05 | -0.0661 | 197 | 0.000 |
| ENSP00000194155 | 3.47E-05 | -0.0661 | 0 | 0.139 |
| ENSP00000239446 | 2.70E-05 | -0.0661 | 0 | 0.120 |
| ENSP00000302833 | 3.26E-05 | -0.0661 | 0 | 0.131 |
| ENSP00000288228 | 2.18E-05 | -0.0661 | 0 | 0.121 |
| ENSP00000366374 | 1.32E-06 | -0.0661 | 0 | 0.216 |
| ENSP00000296129 | 2.20E-05 | -0.0661 | 0 | 0.265 |
| ENSP00000298690 | 2.67E-05 | -0.0661 | 0 | 0.101 |
| ENSP00000333656 | 1.66E-05 | -0.0662 | 0 | 0.522 |
| ENSP00000259056 | 2.40E-05 | -0.0662 | 0 | 0.102 |
| ENSP00000261244 | 2.51E-05 | -0.0662 | 0 | 0.112 |
| ENSP00000289953 | 2.10E-05 | -0.0662 | 0 | 0.133 |
| ENSP00000054666 | 2.43E-05 | -0.0662 | 346 | 0.246 |
| ENSP00000338050 | 1.41E-05 | -0.0662 | 0 | 0.252 |
| ENSP00000205194 | 3.41E-05 | -0.0662 | 0 | 0.117 |
| ENSP00000269389 | 2.76E-05 | -0.0662 | 0 | 0.213 |
| ENSP00000237858 | 1.63E-05 | -0.0662 | 240 | 0.388 |
| ENSP00000306396 | 3.05E-05 | -0.0662 | 0 | 0.115 |
| ENSP00000215770 | 2.58E-05 | -0.0662 | 0 | 0.078 |
| ENSP00000311320 | 1.54E-05 | -0.0662 | 0 | 0.137 |
| ENSP00000395538 | 1.19E-05 | -0.0662 | 0 | 0.289 |
| ENSP00000301939 | 3.97E-05 | -0.0662 | 0 | 0.125 |
| ENSP00000296824 | 3.42E-05 | -0.0662 | 0 | 0.133 |
| ENSP00000274458 | 3.18E-05 | -0.0663 | 0 | 0.164 |
| ENSP00000258052 | 3.52E-05 | -0.0663 | 313 | 0.390 |
| ENSP00000247712 | 2.95E-05 | -0.0663 | 0 | 0.000 |
| ENSP00000011473 | 3.82E-05 | -0.0663 | 0 | 0.165 |
| ENSP00000365906 | 2.98E-05 | -0.0663 | 0 | 0.684 |
| ENSP00000312304 | 2.80E-05 | -0.0663 | 198 | 0.115 |

| | | | | |
|---|---|---|---|---|
| ENSP00000341213 | 1.31E-05 | -0.0663 | 0 | 0.134 |
| ENSP00000262947 | 3.03E-05 | -0.0663 | 502 | 0.000 |
| ENSP00000225328 | 2.48E-05 | -0.0663 | 0 | 0.099 |
| ENSP00000272139 | 4.13E-05 | -0.0663 | 186 | 0.431 |
| ENSP00000376445 | 1.98E-05 | -0.0663 | 163 | 0.762 |
| ENSP00000314458 | 4.07E-05 | -0.0663 | 891 | 0.752 |
| ENSP00000292778 | 2.08E-05 | -0.0664 | 0 | 0.077 |
| ENSP00000265806 | 3.37E-05 | -0.0664 | 0 | 0.133 |
| ENSP00000313422 | 4.12E-05 | -0.0664 | 0 | 0.162 |
| ENSP00000291900 | 2.61E-05 | -0.0664 | 201 | 0.111 |
| ENSP00000248248 | 2.00E-05 | -0.0664 | 158 | 0.087 |
| ENSP00000261740 | 1.61E-05 | -0.0664 | 234 | 0.590 |
| ENSP00000407509 | 1.62E-04 | -0.0664 | 471 | 0.629 |
| ENSP00000262415 | 1.77E-05 | -0.0664 | 725 | 0.951 |
| ENSP00000358131 | 7.69E-06 | -0.0664 | 0 | 0.000 |
| ENSP00000223073 | 1.86E-05 | -0.0664 | 372 | 0.898 |
| ENSP00000264012 | 2.06E-05 | -0.0664 | 196 | 0.694 |
| ENSP00000216185 | 1.29E-05 | -0.0664 | 415 | 0.630 |
| ENSP00000334216 | 1.39E-05 | -0.0664 | 0 | 0.094 |
| ENSP00000341657 | 2.19E-05 | -0.0664 | 0 | 0.089 |
| ENSP00000334037 | 1.79E-05 | -0.0664 | 0 | 0.162 |
| ENSP00000254051 | 3.02E-05 | -0.0664 | 0 | 0.190 |
| ENSP00000255674 | 3.10E-05 | -0.0664 | 0 | 0.107 |
| ENSP00000316284 | 1.74E-05 | -0.0664 | 0 | 0.218 |
| ENSP00000332148 | 2.05E-05 | -0.0664 | 0 | 0.113 |
| ENSP00000317542 | 2.83E-05 | -0.0664 | 461 | 0.000 |
| ENSP00000293406 | 2.45E-05 | -0.0664 | 207 | 0.705 |
| ENSP00000319831 | 2.61E-05 | -0.0664 | 0 | 0.149 |
| ENSP00000280800 | 2.47E-05 | -0.0664 | 0 | 0.086 |
| ENSP00000319412 | 3.17E-05 | -0.0664 | 0 | 0.093 |
| ENSP00000299088 | 3.52E-05 | -0.0664 | 0 | 0.125 |
| ENSP00000264712 | 2.11E-05 | -0.0664 | 196 | 0.308 |
| ENSP00000322238 | 2.38E-05 | -0.0664 | 0 | 0.106 |
| ENSP00000316244 | 2.24E-05 | -0.0665 | 414 | 0.709 |
| ENSP00000342023 | 1.77E-05 | -0.0665 | 0 | 0.176 |
| ENSP00000337991 | 1.86E-05 | -0.0665 | 0 | 0.114 |
| ENSP00000263126 | 2.31E-05 | -0.0665 | 243 | 0.225 |
| ENSP00000280987 | 2.05E-05 | -0.0665 | 0 | 0.162 |
| ENSP00000270645 | 2.95E-05 | -0.0665 | 0 | 0.150 |
| ENSP00000312649 | 3.14E-05 | -0.0665 | 211 | 0.679 |
| ENSP00000219315 | 3.62E-05 | -0.0665 | 0 | 0.357 |
| ENSP00000249269 | 1.67E-05 | -0.0665 | 204 | 0.436 |
| ENSP00000290155 | 2.13E-05 | -0.0665 | 0 | 0.120 |
| ENSP00000363257 | 8.66E-06 | -0.0665 | 0 | 0.085 |
| ENSP00000312550 | 1.83E-05 | -0.0665 | 0 | 0.211 |
| ENSP00000206544 | 2.80E-05 | -0.0665 | 0 | 0.112 |

| | | | | |
|---|---|---|---|---|
| ENSP00000306105 | 1.96E-05 | -0.0665 | 0 | 0.104 |
| ENSP00000256186 | 3.13E-05 | -0.0665 | 0 | 0.108 |
| ENSP00000269491 | 2.57E-05 | -0.0665 | 0 | 0.207 |
| ENSP00000308330 | 1.91E-05 | -0.0665 | 0 | 0.113 |
| ENSP00000340093 | 2.16E-05 | -0.0665 | 215 | 0.115 |
| ENSP00000344503 | 2.48E-05 | -0.0665 | 201 | 0.151 |
| ENSP00000221130 | 3.80E-05 | -0.0665 | 429 | 0.727 |
| ENSP00000262518 | 1.73E-05 | -0.0665 | 196 | 0.944 |
| ENSP00000279907 | 4.06E-05 | -0.0665 | 0 | 0.122 |
| ENSP00000272732 | 1.90E-05 | -0.0665 | 0 | 0.172 |
| ENSP00000295746 | 3.35E-05 | -0.0665 | 0 | 0.215 |
| ENSP00000344683 | 6.89E-05 | -0.0665 | 581 | 0.247 |
| ENSP00000327487 | 1.60E-05 | -0.0665 | 0 | 0.266 |
| ENSP00000008391 | 2.25E-05 | -0.0665 | 0 | 0.427 |
| ENSP00000257013 | 2.35E-05 | -0.0665 | 0 | 0.131 |
| ENSP00000293443 | 2.07E-05 | -0.0666 | 0 | 0.119 |
| ENSP00000291416 | 2.12E-05 | -0.0666 | 0 | 0.121 |
| ENSP00000315387 | 2.70E-05 | -0.0666 | 0 | 0.150 |
| ENSP00000327506 | 3.40E-05 | -0.0666 | 0 | 0.098 |
| ENSP00000343298 | 1.57E-05 | -0.0666 | 0 | 0.142 |
| ENSP00000363344 | 2.05E-05 | -0.0666 | 175 | 0.680 |
| ENSP00000272424 | 2.19E-05 | -0.0666 | 526 | 0.116 |
| ENSP00000288699 | 1.83E-05 | -0.0666 | 194 | 0.330 |
| ENSP00000265140 | 2.11E-05 | -0.0666 | 157 | 0.000 |
| ENSP00000363348 | 1.38E-05 | -0.0666 | 0 | 0.000 |
| ENSP00000265807 | 1.84E-05 | -0.0666 | 0 | 0.094 |
| ENSP00000253669 | 2.93E-05 | -0.0666 | 0 | 0.107 |
| ENSP00000417257 | 2.07E-04 | -0.0666 | 200 | 0.608 |
| ENSP00000264501 | 3.86E-05 | -0.0667 | 167 | 0.290 |
| ENSP00000264108 | 1.88E-05 | -0.0667 | 264 | 0.856 |
| ENSP00000295600 | 3.48E-05 | -0.0667 | 286 | 0.000 |
| ENSP00000323645 | 2.33E-05 | -0.0667 | 0 | 0.105 |
| ENSP00000301021 | 2.16E-05 | -0.0667 | 591 | 0.083 |
| ENSP00000219139 | 2.36E-05 | -0.0667 | 0 | 0.111 |
| ENSP00000344724 | 2.63E-05 | -0.0667 | 269 | 0.915 |
| ENSP00000396505 | 1.10E-07 | -0.0667 | 0 | 0.000 |
| ENSP00000259006 | 2.28E-05 | -0.0667 | 0 | 0.217 |
| ENSP00000254900 | 2.81E-05 | -0.0667 | 0 | 0.760 |
| ENSP00000295304 | 1.83E-05 | -0.0667 | 0 | 0.116 |
| ENSP00000162044 | 3.02E-05 | -0.0667 | 0 | 0.163 |
| ENSP00000303158 | 2.63E-05 | -0.0667 | 0 | 0.083 |
| ENSP00000338387 | 3.32E-05 | -0.0667 | 179 | 0.243 |
| ENSP00000274507 | 2.71E-05 | -0.0667 | 0 | 0.152 |
| ENSP00000356985 | 2.15E-05 | -0.0667 | 179 | 0.377 |
| ENSP00000222574 | 2.07E-05 | -0.0667 | 0 | 0.485 |
| ENSP00000333283 | 1.85E-05 | -0.0667 | 0 | 0.000 |

| | | | | |
|---|---|---|---|---|
| ENSP00000320239 | 4.82E-05 | -0.0667 | 271 | 0.443 |
| ENSP00000295924 | 1.96E-05 | -0.0667 | 0 | 0.105 |
| ENSP00000316782 | 1.99E-05 | -0.0668 | 0 | 0.167 |
| ENSP00000310803 | 1.38E-05 | -0.0668 | 0 | 0.148 |
| ENSP00000344254 | 1.95E-05 | -0.0668 | 160 | 0.485 |
| ENSP00000252655 | 2.15E-05 | -0.0668 | 0 | 0.133 |
| ENSP00000315564 | 1.71E-05 | -0.0668 | 0 | 0.123 |
| ENSP00000011619 | 3.02E-05 | -0.0668 | 150 | 0.389 |
| ENSP00000231134 | 2.56E-05 | -0.0668 | 0 | 0.120 |
| ENSP00000272342 | 4.03E-05 | -0.0668 | 0 | 0.158 |
| ENSP00000265523 | 1.20E-05 | -0.0668 | 0 | 0.158 |
| ENSP00000289361 | 2.45E-05 | -0.0668 | 0 | 0.155 |
| ENSP00000296292 | 2.72E-05 | -0.0668 | 0 | 0.091 |
| ENSP00000283303 | 2.04E-05 | -0.0668 | 0 | 0.000 |
| ENSP00000263379 | 2.22E-05 | -0.0668 | 0 | 0.491 |
| ENSP00000312017 | 2.17E-05 | -0.0668 | 0 | 0.092 |
| ENSP00000258417 | 2.17E-05 | -0.0668 | 0 | 0.123 |
| ENSP00000225525 | 2.49E-05 | -0.0668 | 218 | 0.104 |
| ENSP00000261758 | 3.98E-05 | -0.0668 | 167 | 0.266 |
| ENSP00000006526 | 2.11E-05 | -0.0668 | 0 | 0.179 |
| ENSP00000257604 | 1.94E-05 | -0.0668 | 0 | 0.295 |
| ENSP00000322532 | 1.94E-05 | -0.0669 | 0 | 0.380 |
| ENSP00000215582 | 2.08E-05 | -0.0669 | 0 | 0.000 |
| ENSP00000306887 | 1.59E-05 | -0.0669 | 0 | 0.140 |
| ENSP00000239027 | 1.58E-05 | -0.0669 | 161 | 0.925 |
| ENSP00000295082 | 2.06E-05 | -0.0669 | 195 | 0.177 |
| ENSP00000320815 | 2.25E-05 | -0.0669 | 215 | 0.100 |
| ENSP00000300030 | 2.43E-05 | -0.0669 | 0 | 0.107 |
| ENSP00000338389 | 1.17E-05 | -0.0669 | 0 | 0.228 |
| ENSP00000231137 | 1.76E-05 | -0.0669 | 0 | 0.101 |
| ENSP00000261796 | 2.53E-05 | -0.0669 | 0 | 0.339 |
| ENSP00000253047 | 1.78E-05 | -0.0669 | 0 | 0.109 |
| ENSP00000297283 | 2.00E-05 | -0.0669 | 368 | 0.471 |
| ENSP00000333456 | 2.10E-05 | -0.0669 | 0 | 0.142 |
| ENSP00000304903 | 2.33E-05 | -0.0669 | 200 | 0.699 |
| ENSP00000314544 | 1.49E-05 | -0.0669 | 0 | 0.096 |
| ENSP00000320650 | 1.88E-05 | -0.0669 | 776 | 0.317 |
| ENSP00000310305 | 2.02E-05 | -0.0669 | 850 | 0.603 |
| ENSP00000473092 | 1.35E-05 | -0.0669 | 300 | 0.000 |
| ENSP00000312250 | 1.93E-05 | -0.0669 | 0 | 0.072 |
| ENSP00000264313 | 2.84E-05 | -0.0669 | 0 | 0.132 |
| ENSP00000273375 | 1.91E-05 | -0.0669 | 0 | 0.112 |
| ENSP00000231368 | 1.69E-05 | -0.0670 | 750 | 0.527 |
| ENSP00000251250 | 6.31E-05 | -0.0670 | 0 | 0.175 |
| ENSP00000241527 | 2.19E-05 | -0.0670 | 0 | 0.000 |
| ENSP00000338796 | 2.38E-05 | -0.0670 | 0 | 0.209 |

| | | | | |
|---|---|---|---|---|
| ENSP00000301244 | 1.66E-05 | -0.0670 | 0 | 0.179 |
| ENSP00000317431 | 1.30E-05 | -0.0670 | 0 | 0.113 |
| ENSP00000453854 | 2.45E-05 | -0.0670 | 194 | 0.128 |
| ENSP00000279477 | 3.03E-05 | -0.0670 | 0 | 0.134 |
| ENSP00000274030 | 2.33E-05 | -0.0670 | 0 | 0.122 |
| ENSP00000383690 | 2.06E-05 | -0.0670 | 167 | 0.263 |
| ENSP00000268720 | 1.45E-05 | -0.0670 | 0 | 0.135 |
| ENSP00000244930 | 2.99E-05 | -0.0670 | 0 | 0.148 |
| ENSP00000319009 | 1.81E-05 | -0.0670 | 0 | 0.104 |
| ENSP00000281437 | 1.90E-05 | -0.0670 | 0 | 0.587 |
| ENSP00000263461 | 2.38E-05 | -0.0670 | 0 | 0.527 |
| ENSP00000245908 | 2.37E-05 | -0.0670 | 0 | 0.380 |
| ENSP00000290536 | 1.31E-05 | -0.0670 | 0 | 0.154 |
| ENSP00000332757 | 1.53E-05 | -0.0670 | 0 | 0.089 |
| ENSP00000261944 | 1.53E-05 | -0.0670 | 0 | 0.089 |
| ENSP00000318883 | 2.18E-05 | -0.0671 | 0 | 0.093 |
| ENSP00000307765 | 2.20E-05 | -0.0671 | 0 | 0.170 |
| ENSP00000303077 | 2.35E-05 | -0.0671 | 186 | 0.325 |
| ENSP00000359221 | 2.61E-05 | -0.0671 | 153 | 0.204 |
| ENSP00000263650 | 3.47E-05 | -0.0671 | 427 | 0.175 |
| ENSP00000240922 | 1.68E-05 | -0.0671 | 213 | 0.507 |
| ENSP00000256366 | 2.77E-05 | -0.0671 | 0 | 0.109 |
| ENSP00000295117 | 1.39E-05 | -0.0671 | 0 | 0.000 |
| ENSP00000361547 | 5.33E-06 | -0.0671 | 0 | 0.744 |
| ENSP00000331342 | 2.01E-05 | -0.0671 | 0 | 0.117 |
| ENSP00000384554 | 1.15E-04 | -0.0671 | 501 | 0.697 |
| ENSP00000337313 | 2.11E-05 | -0.0671 | 0 | 0.000 |
| ENSP00000321544 | 2.12E-05 | -0.0671 | 0 | 0.086 |
| ENSP00000260118 | 2.10E-05 | -0.0671 | 215 | 0.148 |
| ENSP00000300917 | 1.98E-05 | -0.0671 | 0 | 0.695 |
| ENSP00000310726 | 1.40E-05 | -0.0671 | 0 | 0.000 |
| ENSP00000349145 | 1.98E-05 | -0.0671 | 244 | 0.322 |
| ENSP00000262715 | 1.61E-05 | -0.0671 | 181 | 0.833 |
| ENSP00000284694 | 2.17E-05 | -0.0671 | 341 | 0.320 |
| ENSP00000220420 | 2.14E-05 | -0.0671 | 0 | 0.176 |
| ENSP00000343635 | 2.19E-05 | -0.0671 | 0 | 0.136 |
| ENSP00000327468 | 1.53E-05 | -0.0671 | 0 | 0.102 |
| ENSP00000318406 | 2.26E-05 | -0.0672 | 0 | 0.104 |
| ENSP00000286070 | 2.61E-05 | -0.0672 | 0 | 0.128 |
| ENSP00000306561 | 2.08E-05 | -0.0672 | 181 | 0.730 |
| ENSP00000245817 | 2.23E-05 | -0.0672 | 0 | 0.561 |
| ENSP00000348634 | 3.21E-05 | -0.0672 | 910 | 0.810 |
| ENSP00000269221 | 4.60E-05 | -0.0672 | 0 | 0.166 |
| ENSP00000262293 | 1.81E-05 | -0.0672 | 0 | 0.102 |
| ENSP00000312070 | 1.82E-05 | -0.0672 | 0 | 0.114 |
| ENSP00000315371 | 1.97E-05 | -0.0672 | 0 | 0.306 |

| | | | | |
|---|---|---|---|---|
| ENSP00000260247 | 2.82E-05 | -0.0672 | 0 | 0.216 |
| ENSP00000261778 | 1.94E-05 | -0.0672 | 0 | 0.117 |
| ENSP00000304290 | 1.64E-05 | -0.0672 | 0 | 0.375 |
| ENSP00000296849 | 2.74E-05 | -0.0672 | 179 | 0.607 |
| ENSP00000241651 | 3.55E-05 | -0.0672 | 309 | 0.875 |
| ENSP00000337773 | 1.15E-05 | -0.0672 | 0 | 0.108 |
| ENSP00000295702 | 1.96E-05 | -0.0672 | 176 | 0.412 |
| ENSP00000258381 | 4.41E-05 | -0.0672 | 204 | 0.331 |
| ENSP00000248121 | 1.73E-05 | -0.0672 | 0 | 0.086 |
| ENSP00000324810 | 1.64E-05 | -0.0672 | 0 | 0.074 |
| ENSP00000336724 | 2.53E-05 | -0.0672 | 300 | 0.170 |
| ENSP00000270357 | 2.27E-05 | -0.0672 | 336 | 0.329 |
| ENSP00000312663 | 1.49E-05 | -0.0672 | 0 | 0.306 |
| ENSP00000227638 | 1.86E-05 | -0.0672 | 0 | 0.336 |
| ENSP00000324960 | 2.23E-05 | -0.0672 | 414 | 0.646 |
| ENSP00000296440 | 8.85E-06 | -0.0673 | 0 | 0.709 |
| ENSP00000310356 | 1.44E-05 | -0.0673 | 0 | 0.559 |
| ENSP00000312702 | 1.62E-05 | -0.0673 | 0 | 0.174 |
| ENSP00000317579 | 1.58E-05 | -0.0673 | 181 | 0.191 |
| ENSP00000327459 | 1.40E-05 | -0.0673 | 0 | 0.134 |
| ENSP00000332359 | 2.75E-05 | -0.0673 | 0 | 0.089 |
| ENSP00000299638 | 2.38E-05 | -0.0673 | 0 | 0.339 |
| ENSP00000334849 | 1.59E-05 | -0.0673 | 0 | 0.096 |
| ENSP00000326227 | 1.67E-05 | -0.0673 | 433 | 0.193 |
| ENSP00000256538 | 2.10E-05 | -0.0673 | 0 | 0.000 |
| ENSP00000293745 | 1.50E-05 | -0.0673 | 0 | 0.258 |
| ENSP00000313699 | 2.82E-05 | -0.0673 | 173 | 0.142 |
| ENSP00000329918 | 1.42E-05 | -0.0673 | 0 | 0.118 |
| ENSP00000331327 | 5.82E-05 | -0.0673 | 865 | 0.893 |
| ENSP00000295148 | 3.18E-05 | -0.0673 | 0 | 0.467 |
| ENSP00000326924 | 1.75E-05 | -0.0673 | 0 | 0.124 |
| ENSP00000288912 | 1.46E-05 | -0.0673 | 0 | 0.089 |
| ENSP00000287996 | 1.86E-05 | -0.0673 | 0 | 0.066 |
| ENSP00000240617 | 2.48E-05 | -0.0673 | 0 | 0.107 |
| ENSP00000269228 | 2.27E-05 | -0.0673 | 509 | 0.407 |
| ENSP00000269499 | 1.81E-05 | -0.0673 | 0 | 0.129 |
| ENSP00000237536 | 2.16E-05 | -0.0674 | 0 | 0.157 |
| ENSP00000299886 | 1.63E-05 | -0.0674 | 343 | 0.181 |
| ENSP00000262146 | 2.26E-05 | -0.0674 | 0 | 0.101 |
| ENSP00000288014 | 1.43E-05 | -0.0674 | 0 | 0.069 |
| ENSP00000234195 | 3.11E-05 | -0.0674 | 0 | 0.112 |
| ENSP00000222120 | 2.31E-05 | -0.0674 | 242 | 0.209 |
| ENSP00000298546 | 1.40E-05 | -0.0674 | 0 | 0.066 |
| ENSP00000296581 | 2.08E-05 | -0.0674 | 262 | 0.790 |
| ENSP00000294507 | 1.94E-05 | -0.0674 | 0 | 0.143 |
| ENSP00000301011 | 2.26E-05 | -0.0674 | 0 | 0.544 |

| | | | | |
|---|---|---|---|---|
| ENSP00000296380 | 2.62E-05 | -0.0674 | 0 | 0.106 |
| ENSP00000305524 | 1.35E-05 | -0.0674 | 0 | 0.317 |
| ENSP00000325941 | 7.27E-05 | -0.0674 | 543 | 0.183 |
| ENSP00000305161 | 1.59E-05 | -0.0674 | 0 | 0.280 |
| ENSP00000301295 | 1.79E-05 | -0.0674 | 0 | 0.172 |
| ENSP00000215591 | 2.23E-05 | -0.0674 | 168 | 0.000 |
| ENSP00000329471 | 2.28E-05 | -0.0674 | 426 | 0.196 |
| ENSP00000256151 | 2.17E-05 | -0.0674 | 0 | 0.176 |
| ENSP00000382794 | 8.39E-06 | -0.0674 | 0 | 0.000 |
| ENSP00000292566 | 1.70E-05 | -0.0675 | 0 | 0.128 |
| ENSP00000325958 | 1.34E-05 | -0.0675 | 0 | 0.069 |
| ENSP00000312262 | 2.19E-05 | -0.0675 | 598 | 0.617 |
| ENSP00000307706 | 1.90E-05 | -0.0675 | 0 | 0.178 |
| ENSP00000338742 | 1.31E-05 | -0.0675 | 0 | 0.070 |
| ENSP00000256997 | 2.15E-05 | -0.0675 | 303 | 0.224 |
| ENSP00000267615 | 1.61E-05 | -0.0675 | 0 | 0.101 |
| ENSP00000219345 | 1.83E-05 | -0.0675 | 193 | 0.198 |
| ENSP00000344299 | 1.45E-05 | -0.0675 | 0 | 0.096 |
| ENSP00000244096 | 1.37E-05 | -0.0675 | 0 | 0.156 |
| ENSP00000332013 | 1.54E-05 | -0.0675 | 0 | 0.273 |
| ENSP00000332103 | 1.76E-05 | -0.0675 | 0 | 0.000 |
| ENSP00000273920 | 1.47E-05 | -0.0675 | 0 | 0.105 |
| ENSP00000296333 | 1.55E-05 | -0.0675 | 0 | 0.093 |
| ENSP00000254816 | 3.47E-05 | -0.0675 | 0 | 0.118 |
| ENSP00000351591 | 3.97E-05 | -0.0675 | 252 | 0.210 |
| ENSP00000262102 | 1.94E-05 | -0.0675 | 0 | 0.147 |
| ENSP00000305926 | 1.97E-05 | -0.0675 | 0 | 0.101 |
| ENSP00000239944 | 2.09E-05 | -0.0675 | 427 | 0.244 |
| ENSP00000322579 | 1.65E-05 | -0.0675 | 0 | 0.504 |
| ENSP00000238146 | 1.97E-05 | -0.0675 | 186 | 0.893 |
| ENSP00000210444 | 2.14E-05 | -0.0675 | 278 | 0.100 |
| ENSP00000264596 | 1.94E-05 | -0.0675 | 0 | 0.149 |
| ENSP00000244227 | 2.06E-05 | -0.0675 | 0 | 0.280 |
| ENSP00000343746 | 3.24E-05 | -0.0676 | 335 | 0.371 |
| ENSP00000256015 | 2.08E-05 | -0.0676 | 0 | 0.465 |
| ENSP00000309542 | 2.40E-05 | -0.0676 | 240 | 0.199 |
| ENSP00000360060 | 1.87E-05 | -0.0676 | 206 | 0.625 |
| ENSP00000252936 | 1.73E-05 | -0.0676 | 212 | 0.133 |
| ENSP00000258243 | 2.04E-05 | -0.0676 | 0 | 0.179 |
| ENSP00000337512 | 1.35E-05 | -0.0676 | 169 | 0.000 |
| ENSP00000357082 | 6.81E-06 | -0.0676 | 0 | 0.179 |
| ENSP00000328287 | 1.81E-05 | -0.0676 | 582 | 0.446 |
| ENSP00000273075 | 1.86E-05 | -0.0676 | 625 | 0.119 |
| ENSP00000341963 | 1.78E-05 | -0.0676 | 0 | 0.098 |
| ENSP00000194118 | 2.35E-05 | -0.0676 | 247 | 0.677 |
| ENSP00000321184 | 2.02E-05 | -0.0676 | 0 | 0.138 |

| | | | | |
|---|---|---|---|---|
| ENSP00000307199 | 1.31E-05 | -0.0676 | 0 | 0.146 |
| ENSP00000321260 | 1.88E-05 | -0.0676 | 302 | 0.474 |
| ENSP00000260187 | 2.17E-05 | -0.0676 | 228 | 0.405 |
| ENSP00000222374 | 1.80E-05 | -0.0676 | 0 | 0.080 |
| ENSP00000225777 | 1.79E-05 | -0.0677 | 0 | 0.251 |
| ENSP00000222968 | 1.90E-05 | -0.0677 | 0 | 0.122 |
| ENSP00000262510 | 1.85E-05 | -0.0677 | 0 | 0.189 |
| ENSP00000265000 | 1.56E-05 | -0.0677 | 0 | 0.103 |
| ENSP00000314807 | 1.94E-05 | -0.0677 | 163 | 0.526 |
| ENSP00000231449 | 4.08E-05 | -0.0677 | 926 | 0.959 |
| ENSP00000254770 | 1.70E-05 | -0.0677 | 0 | 0.087 |
| ENSP00000318852 | 1.98E-05 | -0.0677 | 0 | 0.079 |
| ENSP00000293925 | 2.87E-05 | -0.0677 | 0 | 0.000 |
| ENSP00000231198 | 1.70E-05 | -0.0677 | 0 | 0.174 |
| ENSP00000253004 | 1.74E-05 | -0.0677 | 254 | 0.515 |
| ENSP00000278980 | 2.07E-05 | -0.0677 | 0 | 0.270 |
| ENSP00000335657 | 2.41E-05 | -0.0677 | 472 | 0.815 |
| ENSP00000369965 | 1.77E-05 | -0.0677 | 329 | 0.754 |
| ENSP00000246112 | 2.43E-05 | -0.0677 | 188 | 0.822 |
| ENSP00000265963 | 2.27E-05 | -0.0677 | 396 | 0.763 |
| ENSP00000278071 | 2.10E-05 | -0.0677 | 0 | 0.114 |
| ENSP00000285850 | 2.75E-05 | -0.0677 | 191 | 0.112 |
| ENSP00000298694 | 1.31E-05 | -0.0677 | 0 | 0.111 |
| ENSP00000351015 | 5.11E-05 | -0.0677 | 318 | 0.905 |
| ENSP00000290913 | 1.32E-05 | -0.0677 | 0 | 0.082 |
| ENSP00000300006 | 1.99E-05 | -0.0677 | 0 | 0.128 |
| ENSP00000301458 | 1.57E-05 | -0.0677 | 598 | 0.442 |
| ENSP00000216214 | 2.15E-05 | -0.0677 | 0 | 0.083 |
| ENSP00000340237 | 1.82E-05 | -0.0677 | 299 | 0.082 |
| ENSP00000359498 | 1.96E-08 | -0.0677 | 0 | 0.214 |
| ENSP00000254663 | 1.66E-05 | -0.0677 | 413 | 0.342 |
| ENSP00000227163 | 4.27E-05 | -0.0677 | 325 | 0.870 |
| ENSP00000296149 | 1.59E-05 | -0.0678 | 0 | 0.099 |
| ENSP00000321271 | 1.49E-05 | -0.0678 | 0 | 0.062 |
| ENSP00000337353 | 1.45E-05 | -0.0678 | 0 | 0.519 |
| ENSP00000338814 | 1.93E-05 | -0.0678 | 296 | 0.399 |
| ENSP00000326371 | 2.23E-05 | -0.0678 | 242 | 0.000 |
| ENSP00000367878 | 7.10E-06 | -0.0678 | 0 | 0.105 |
| ENSP00000218364 | 2.17E-05 | -0.0678 | 165 | 0.641 |
| ENSP00000244519 | 1.87E-05 | -0.0678 | 0 | 0.108 |
| ENSP00000314971 | 1.44E-05 | -0.0678 | 0 | 0.434 |
| ENSP00000290705 | 1.93E-05 | -0.0678 | 0 | 0.130 |
| ENSP00000292539 | 1.81E-05 | -0.0678 | 280 | 0.689 |
| ENSP00000238618 | 1.97E-05 | -0.0678 | 0 | 0.079 |
| ENSP00000261017 | 1.72E-05 | -0.0678 | 224 | 0.544 |
| ENSP00000262384 | 2.64E-05 | -0.0678 | 0 | 0.164 |

| | | | | |
|---|---|---|---|---|
| ENSP00000333350 | 1.55E-05 | -0.0678 | 0 | 0.290 |
| ENSP00000334813 | 1.28E-05 | -0.0678 | 207 | 0.755 |
| ENSP00000342805 | 1.54E-05 | -0.0678 | 0 | 0.064 |
| ENSP00000308727 | 1.88E-05 | -0.0678 | 0 | 0.120 |
| ENSP00000332204 | 1.29E-05 | -0.0678 | 0 | 0.306 |
| ENSP00000353887 | 7.39E-06 | -0.0678 | 0 | 0.132 |
| ENSP00000329033 | 1.86E-05 | -0.0678 | 317 | 0.127 |
| ENSP00000318086 | 1.39E-05 | -0.0678 | 0 | 0.155 |
| ENSP00000334130 | 1.13E-05 | -0.0678 | 0 | 0.607 |
| ENSP00000304467 | 1.83E-05 | -0.0678 | 775 | 0.390 |
| ENSP00000261789 | 1.62E-05 | -0.0678 | 0 | 0.108 |
| ENSP00000267935 | 2.27E-05 | -0.0678 | 0 | 0.115 |
| ENSP00000273861 | 1.49E-05 | -0.0678 | 0 | 0.082 |
| ENSP00000301717 | 1.97E-05 | -0.0678 | 0 | 0.146 |
| ENSP00000328325 | 1.55E-05 | -0.0679 | 0 | 0.226 |
| ENSP00000264718 | 1.81E-05 | -0.0679 | 193 | 0.637 |
| ENSP00000240587 | 1.86E-05 | -0.0679 | 0 | 0.417 |
| ENSP00000341267 | 3.02E-05 | -0.0679 | 0 | 0.206 |
| ENSP00000306117 | 1.88E-05 | -0.0679 | 203 | 0.795 |
| ENSP00000261191 | 1.80E-05 | -0.0679 | 0 | 0.086 |
| ENSP00000244007 | 1.70E-05 | -0.0679 | 179 | 0.890 |
| ENSP00000401653 | 5.61E-06 | -0.0679 | 0 | 0.776 |
| ENSP00000336800 | 1.42E-05 | -0.0679 | 0 | 0.128 |
| ENSP00000016171 | 1.78E-05 | -0.0679 | 199 | 0.238 |
| ENSP00000263119 | 1.20E-05 | -0.0679 | 0 | 0.728 |
| ENSP00000405202 | 1.10E-05 | -0.0679 | 0 | 0.000 |
| ENSP00000286317 | 2.07E-05 | -0.0679 | 233 | 0.749 |
| ENSP00000233121 | 1.55E-05 | -0.0679 | 164 | 0.400 |
| ENSP00000226760 | 2.04E-05 | -0.0679 | 325 | 0.343 |
| ENSP00000313759 | 1.81E-05 | -0.0679 | 0 | 0.141 |
| ENSP00000340402 | 1.19E-05 | -0.0679 | 0 | 0.078 |
| ENSP00000316699 | 1.55E-05 | -0.0680 | 0 | 0.116 |
| ENSP00000261658 | 1.49E-05 | -0.0680 | 0 | 0.180 |
| ENSP00000297142 | 1.42E-05 | -0.0680 | 0 | 0.566 |
| ENSP00000332806 | 3.42E-05 | -0.0680 | 0 | 0.161 |
| ENSP00000382064 | 2.56E-05 | -0.0680 | 341 | 0.396 |
| ENSP00000338190 | 2.92E-05 | -0.0680 | 200 | 0.000 |
| ENSP00000290354 | 3.20E-05 | -0.0680 | 159 | 0.199 |
| ENSP00000320493 | 2.44E-05 | -0.0680 | 191 | 0.499 |
| ENSP00000335091 | 1.48E-05 | -0.0680 | 0 | 0.101 |
| ENSP00000266263 | 1.39E-05 | -0.0680 | 0 | 0.071 |
| ENSP00000317905 | 2.52E-05 | -0.0680 | 0 | 0.000 |
| ENSP00000309644 | 1.76E-05 | -0.0680 | 0 | 0.210 |
| ENSP00000462172 | 5.59E-08 | -0.0680 | 0 | 0.209 |
| ENSP00000256339 | 1.37E-05 | -0.0680 | 0 | 0.124 |
| ENSP00000370303 | 6.40E-06 | -0.0680 | 0 | 0.076 |

| | | | | |
|---|---|---|---|---|
| ENSP00000265026 | 2.20E-05 | -0.0680 | 0 | 0.642 |
| ENSP00000258062 | 2.20E-05 | -0.0680 | 0 | 0.098 |
| ENSP00000297239 | 1.42E-05 | -0.0680 | 0 | 0.081 |
| ENSP00000337889 | 1.44E-05 | -0.0680 | 0 | 0.464 |
| ENSP00000281543 | 1.72E-05 | -0.0680 | 507 | 0.473 |
| ENSP00000243611 | 1.84E-05 | -0.0680 | 0 | 0.247 |
| ENSP00000324890 | 1.41E-05 | -0.0681 | 0 | 0.595 |
| ENSP00000339626 | 1.47E-05 | -0.0681 | 0 | 0.064 |
| ENSP00000308921 | 1.51E-05 | -0.0681 | 0 | 0.074 |
| ENSP00000419923 | 3.44E-05 | -0.0681 | 284 | 0.839 |
| ENSP00000317327 | 1.34E-05 | -0.0681 | 0 | 0.177 |
| ENSP00000350937 | 5.48E-05 | -0.0681 | 349 | 0.745 |
| ENSP00000411459 | 2.09E-05 | -0.0681 | 169 | 0.542 |
| ENSP00000326649 | 1.28E-05 | -0.0681 | 0 | 0.071 |
| ENSP00000308717 | 1.01E-05 | -0.0681 | 0 | 0.267 |
| ENSP00000263579 | 2.26E-05 | -0.0681 | 426 | 0.257 |
| ENSP00000220496 | 1.79E-05 | -0.0681 | 742 | 0.481 |
| ENSP00000316881 | 1.22E-05 | -0.0681 | 0 | 0.000 |
| ENSP00000337335 | 2.65E-05 | -0.0681 | 160 | 0.067 |
| ENSP00000310375 | 1.45E-05 | -0.0681 | 0 | 0.144 |
| ENSP00000308695 | 1.29E-05 | -0.0681 | 0 | 0.083 |
| ENSP00000330075 | 1.85E-05 | -0.0681 | 0 | 0.116 |
| ENSP00000323348 | 1.35E-05 | -0.0681 | 0 | 0.154 |
| ENSP00000246190 | 1.61E-05 | -0.0681 | 0 | 0.080 |
| ENSP00000248342 | 1.66E-05 | -0.0681 | 169 | 0.811 |
| ENSP00000023939 | 2.38E-05 | -0.0681 | 0 | 0.143 |
| ENSP00000282041 | 1.41E-05 | -0.0681 | 0 | 0.120 |
| ENSP00000267859 | 1.47E-05 | -0.0681 | 0 | 0.292 |
| ENSP00000344820 | 2.20E-05 | -0.0681 | 173 | 0.127 |
| ENSP00000254878 | 9.60E-06 | -0.0681 | 0 | 0.120 |
| ENSP00000333487 | 1.17E-05 | -0.0681 | 0 | 0.499 |
| ENSP00000276925 | 1.78E-05 | -0.0681 | 211 | 0.867 |
| ENSP00000278903 | 1.74E-05 | -0.0681 | 0 | 0.194 |
| ENSP00000270708 | 1.57E-05 | -0.0682 | 0 | 0.074 |
| ENSP00000327541 | 1.28E-05 | -0.0682 | 0 | 0.133 |
| ENSP00000263663 | 2.37E-05 | -0.0682 | 0 | 0.727 |
| ENSP00000339529 | 1.10E-05 | -0.0682 | 0 | 0.585 |
| ENSP00000332444 | 2.56E-05 | -0.0682 | 239 | 0.876 |
| ENSP00000221856 | 1.81E-05 | -0.0682 | 0 | 0.112 |
| ENSP00000295984 | 1.51E-05 | -0.0682 | 0 | 0.201 |
| ENSP00000333938 | 2.01E-05 | -0.0682 | 215 | 0.487 |
| ENSP00000317144 | 1.45E-05 | -0.0682 | 0 | 0.080 |
| ENSP00000303408 | 1.76E-05 | -0.0682 | 819 | 0.000 |
| ENSP00000359660 | 3.44E-05 | -0.0682 | 156 | 0.103 |
| ENSP00000270792 | 1.66E-05 | -0.0682 | 0 | 0.136 |
| ENSP00000255688 | 2.27E-05 | -0.0682 | 0 | 0.579 |

| | | | | |
|---|---|---|---|---|
| ENSP00000239666 | 1.86E-05 | -0.0682 | 0 | 0.085 |
| ENSP00000298596 | 1.84E-05 | -0.0682 | 0 | 0.192 |
| ENSP00000263384 | 1.79E-05 | -0.0682 | 0 | 0.314 |
| ENSP00000341684 | 4.16E-05 | -0.0682 | 0 | 0.178 |
| ENSP00000228136 | 2.25E-05 | -0.0682 | 0 | 0.095 |
| ENSP00000357914 | 1.12E-05 | -0.0682 | 201 | 0.664 |
| ENSP00000217195 | 2.13E-05 | -0.0682 | 0 | 0.127 |
| ENSP00000307181 | 1.83E-05 | -0.0682 | 0 | 0.289 |
| ENSP00000331791 | 7.23E-05 | -0.0682 | 360 | 0.915 |
| ENSP00000243346 | 1.60E-05 | -0.0682 | 0 | 0.430 |
| ENSP00000271417 | 1.80E-05 | -0.0682 | 0 | 0.122 |
| ENSP00000247182 | 1.79E-05 | -0.0683 | 158 | 0.865 |
| ENSP00000312679 | 2.53E-05 | -0.0683 | 0 | 0.142 |
| ENSP00000297770 | 1.33E-05 | -0.0683 | 0 | 0.086 |
| ENSP00000290208 | 1.25E-05 | -0.0683 | 0 | 0.238 |
| ENSP00000237172 | 1.48E-05 | -0.0683 | 0 | 0.093 |
| ENSP00000350094 | 1.98E-05 | -0.0683 | 0 | 0.237 |
| ENSP00000330393 | 2.26E-05 | -0.0683 | 566 | 0.542 |
| ENSP00000025301 | 2.25E-05 | -0.0683 | 0 | 0.120 |
| ENSP00000311300 | 1.83E-05 | -0.0683 | 0 | 0.085 |
| ENSP00000311768 | 1.28E-05 | -0.0683 | 0 | 0.511 |
| ENSP00000026218 | 3.64E-05 | -0.0683 | 187 | 0.141 |
| ENSP00000241051 | 1.21E-05 | -0.0683 | 0 | 0.131 |
| ENSP00000313674 | 1.96E-05 | -0.0683 | 284 | 0.618 |
| ENSP00000340578 | 1.69E-05 | -0.0683 | 0 | 0.191 |
| ENSP00000295131 | 2.81E-05 | -0.0683 | 173 | 0.424 |
| ENSP00000218147 | 1.44E-05 | -0.0683 | 0 | 0.427 |
| ENSP00000245932 | 7.15E-05 | -0.0683 | 414 | 0.835 |
| ENSP00000261167 | 2.32E-05 | -0.0683 | 564 | 0.460 |
| ENSP00000253457 | 1.50E-05 | -0.0683 | 0 | 0.258 |
| ENSP00000331152 | 1.61E-05 | -0.0684 | 177 | 0.708 |
| ENSP00000339587 | 1.24E-05 | -0.0684 | 0 | 0.084 |
| ENSP00000294409 | 1.75E-05 | -0.0684 | 0 | 0.367 |
| ENSP00000313601 | 1.07E-05 | -0.0684 | 0 | 0.177 |
| ENSP00000238823 | 1.69E-05 | -0.0684 | 0 | 0.227 |
| ENSP00000321546 | 1.51E-05 | -0.0684 | 0 | 0.000 |
| ENSP00000298310 | 2.37E-05 | -0.0684 | 173 | 0.118 |
| ENSP00000335321 | 2.08E-05 | -0.0684 | 377 | 0.878 |
| ENSP00000302586 | 1.59E-05 | -0.0684 | 0 | 0.116 |
| ENSP00000326819 | 1.24E-05 | -0.0684 | 0 | 0.361 |
| ENSP00000371595 | 2.45E-06 | -0.0684 | 0 | 0.124 |
| ENSP00000258424 | 1.83E-05 | -0.0684 | 436 | 0.398 |
| ENSP00000265651 | 1.53E-05 | -0.0684 | 0 | 0.180 |
| ENSP00000260702 | 2.75E-05 | -0.0684 | 162 | 0.123 |
| ENSP00000360829 | 1.57E-05 | -0.0684 | 0 | 0.108 |
| ENSP00000216099 | 1.73E-05 | -0.0684 | 0 | 0.093 |

| | | | | |
|---|---|---|---|---|
| ENSP00000262365 | 1.06E-05 | -0.0684 | 0 | 0.091 |
| ENSP00000300571 | 1.25E-05 | -0.0684 | 0 | 0.124 |
| ENSP00000315630 | 1.96E-05 | -0.0684 | 0 | 0.213 |
| ENSP00000270066 | 1.13E-05 | -0.0685 | 0 | 0.671 |
| ENSP00000222902 | 4.34E-04 | -0.0685 | 0 | 0.153 |
| ENSP00000337513 | 1.41E-05 | -0.0685 | 0 | 0.661 |
| ENSP00000258770 | 1.53E-05 | -0.0685 | 0 | 0.118 |
| ENSP00000344055 | 2.33E-05 | -0.0685 | 564 | 0.144 |
| ENSP00000285419 | 1.61E-05 | -0.0685 | 0 | 0.084 |
| ENSP00000176195 | 2.06E-05 | -0.0685 | 0 | 0.425 |
| ENSP00000229708 | 1.62E-05 | -0.0685 | 0 | 0.227 |
| ENSP00000261407 | 2.02E-05 | -0.0685 | 0 | 0.200 |
| ENSP00000278840 | 4.23E-05 | -0.0685 | 300 | 0.570 |
| ENSP00000277632 | 1.24E-05 | -0.0685 | 0 | 0.178 |
| ENSP00000295491 | 1.34E-05 | -0.0685 | 302 | 0.631 |
| ENSP00000313885 | 1.12E-05 | -0.0685 | 0 | 0.000 |
| ENSP00000341179 | 1.53E-05 | -0.0685 | 0 | 0.000 |
| ENSP00000281513 | 1.64E-05 | -0.0685 | 0 | 0.127 |
| ENSP00000336801 | 2.20E-05 | -0.0685 | 0 | 0.098 |
| ENSP00000169551 | 1.42E-05 | -0.0686 | 0 | 0.086 |
| ENSP00000263713 | 1.72E-05 | -0.0686 | 0 | 0.198 |
| ENSP00000309336 | 1.56E-05 | -0.0686 | 158 | 0.785 |
| ENSP00000295571 | 1.58E-05 | -0.0686 | 0 | 0.073 |
| ENSP00000296603 | 1.97E-05 | -0.0686 | 0 | 0.139 |
| ENSP00000263033 | 1.33E-05 | -0.0686 | 0 | 0.146 |
| ENSP00000290597 | 1.90E-05 | -0.0686 | 216 | 0.266 |
| ENSP00000357494 | 2.75E-05 | -0.0686 | 323 | 0.594 |
| ENSP00000311513 | 1.22E-05 | -0.0686 | 0 | 0.730 |
| ENSP00000321203 | 1.64E-05 | -0.0686 | 0 | 0.729 |
| ENSP00000308193 | 2.01E-05 | -0.0686 | 0 | 0.120 |
| ENSP00000233627 | 1.68E-05 | -0.0686 | 193 | 0.295 |
| ENSP00000264720 | 1.65E-05 | -0.0686 | 0 | 0.494 |
| ENSP00000256545 | 1.75E-05 | -0.0686 | 0 | 0.086 |
| ENSP00000299821 | 1.07E-05 | -0.0686 | 0 | 0.222 |
| ENSP00000214869 | 1.50E-05 | -0.0686 | 0 | 0.095 |
| ENSP00000294129 | 1.30E-05 | -0.0686 | 0 | 0.223 |
| ENSP00000330730 | 1.01E-05 | -0.0686 | 0 | 0.098 |
| ENSP00000206020 | 2.20E-05 | -0.0686 | 0 | 0.072 |
| ENSP00000267568 | 1.79E-05 | -0.0686 | 0 | 0.063 |
| ENSP00000276344 | 1.19E-05 | -0.0686 | 0 | 0.390 |
| ENSP00000404833 | 4.99E-05 | -0.0686 | 398 | 0.433 |
| ENSP00000264938 | 1.87E-05 | -0.0686 | 201 | 0.414 |
| ENSP00000272643 | 1.79E-05 | -0.0686 | 0 | 0.154 |
| ENSP00000014914 | 1.91E-05 | -0.0686 | 0 | 0.137 |
| ENSP00000261308 | 1.49E-05 | -0.0686 | 0 | 0.469 |
| ENSP00000264001 | 2.06E-05 | -0.0686 | 0 | 0.612 |

| | | | | |
|---|---|---|---|---|
| ENSP00000256441 | 1.21E-05 | -0.0686 | 0 | 0.318 |
| ENSP00000297156 | 1.68E-05 | -0.0686 | 0 | 0.665 |
| ENSP00000221922 | 1.65E-05 | -0.0686 | 0 | 0.095 |
| ENSP00000317842 | 1.87E-05 | -0.0686 | 193 | 0.381 |
| ENSP00000309142 | 2.20E-05 | -0.0687 | 154 | 0.645 |
| ENSP00000243706 | 1.49E-05 | -0.0687 | 0 | 0.177 |
| ENSP00000325266 | 1.66E-05 | -0.0687 | 0 | 0.118 |
| ENSP00000221167 | 1.61E-05 | -0.0687 | 170 | 0.000 |
| ENSP00000304133 | 3.27E-05 | -0.0687 | 207 | 0.556 |
| ENSP00000246117 | 2.02E-05 | -0.0687 | 163 | 0.098 |
| ENSP00000326070 | 1.23E-05 | -0.0687 | 0 | 0.116 |
| ENSP00000227520 | 1.61E-05 | -0.0687 | 0 | 0.527 |
| ENSP00000308925 | 1.20E-05 | -0.0687 | 169 | 0.765 |
| ENSP00000329040 | 1.22E-05 | -0.0687 | 0 | 0.115 |
| ENSP00000263904 | 3.59E-05 | -0.0687 | 215 | 0.266 |
| ENSP00000364041 | 1.62E-05 | -0.0687 | 0 | 0.394 |
| ENSP00000257198 | 1.41E-05 | -0.0687 | 0 | 0.147 |
| ENSP00000263433 | 1.88E-05 | -0.0687 | 282 | 0.753 |
| ENSP00000310263 | 1.73E-05 | -0.0687 | 0 | 0.751 |
| ENSP00000329448 | 1.59E-05 | -0.0687 | 0 | 0.167 |
| ENSP00000337850 | 4.30E-05 | -0.0687 | 230 | 0.366 |
| ENSP00000286758 | 3.00E-04 | -0.0687 | 343 | 0.700 |
| ENSP00000308236 | 4.03E-05 | -0.0687 | 822 | 0.452 |
| ENSP00000313159 | 1.62E-05 | -0.0688 | 0 | 0.156 |
| ENSP00000264454 | 3.84E-05 | -0.0688 | 201 | 0.241 |
| ENSP00000271889 | 2.55E-05 | -0.0688 | 224 | 0.549 |
| ENSP00000324318 | 1.14E-05 | -0.0688 | 0 | 0.111 |
| ENSP00000271526 | 1.76E-05 | -0.0688 | 360 | 0.301 |
| ENSP00000251413 | 1.37E-05 | -0.0688 | 293 | 0.493 |
| ENSP00000246662 | 1.56E-05 | -0.0688 | 0 | 0.122 |
| ENSP00000262210 | 1.36E-05 | -0.0688 | 0 | 0.096 |
| ENSP00000037869 | 1.42E-05 | -0.0688 | 0 | 0.303 |
| ENSP00000349689 | 7.40E-06 | -0.0688 | 0 | 0.069 |
| ENSP00000296252 | 1.25E-05 | -0.0688 | 0 | 0.141 |
| ENSP00000004531 | 1.50E-05 | -0.0688 | 197 | 0.068 |
| ENSP00000249700 | 1.75E-05 | -0.0688 | 0 | 0.114 |
| ENSP00000320935 | 4.86E-05 | -0.0688 | 472 | 0.883 |
| ENSP00000296003 | 1.49E-05 | -0.0688 | 0 | 0.139 |
| ENSP00000244661 | 1.45E-05 | -0.0688 | 829 | 0.000 |
| ENSP00000282369 | 1.18E-05 | -0.0688 | 0 | 0.161 |
| ENSP00000262193 | 2.01E-05 | -0.0688 | 358 | 0.586 |
| ENSP00000228705 | 1.05E-05 | -0.0688 | 0 | 0.126 |
| ENSP00000343557 | 3.29E-05 | -0.0688 | 319 | 0.850 |
| ENSP00000278422 | 2.31E-05 | -0.0689 | 0 | 0.157 |
| ENSP00000298097 | 1.68E-05 | -0.0689 | 0 | 0.112 |
| ENSP00000307265 | 1.55E-05 | -0.0689 | 0 | 0.163 |

| | | | | |
|---|---|---|---|---|
| ENSP00000285398 | 2.28E-05 | -0.0689 | 284 | 0.851 |
| ENSP00000182377 | 1.17E-05 | -0.0689 | 0 | 0.072 |
| ENSP00000286353 | 1.54E-05 | -0.0689 | 0 | 0.000 |
| ENSP00000296600 | 1.61E-05 | -0.0689 | 0 | 0.107 |
| ENSP00000446880 | 2.30E-05 | -0.0689 | 246 | 0.922 |
| ENSP00000271375 | 1.06E-05 | -0.0689 | 0 | 0.124 |
| ENSP00000225726 | 1.27E-05 | -0.0689 | 0 | 0.111 |
| ENSP00000261296 | 1.94E-05 | -0.0689 | 0 | 0.067 |
| ENSP00000290776 | 1.07E-05 | -0.0689 | 0 | 0.152 |
| ENSP00000370737 | 3.52E-05 | -0.0689 | 187 | 0.322 |
| ENSP00000250974 | 1.85E-05 | -0.0690 | 0 | 0.095 |
| ENSP00000424007 | 2.06E-07 | -0.0690 | 0 | 0.000 |
| ENSP00000225729 | 1.97E-05 | -0.0690 | 174 | 0.543 |
| ENSP00000292782 | 2.07E-05 | -0.0690 | 153 | 0.502 |
| ENSP00000040738 | 1.43E-05 | -0.0690 | 0 | 0.132 |
| ENSP00000276704 | 2.18E-05 | -0.0690 | 0 | 0.246 |
| ENSP00000303580 | 9.67E-06 | -0.0690 | 0 | 0.130 |
| ENSP00000319574 | 2.16E-05 | -0.0690 | 355 | 0.225 |
| ENSP00000343742 | 1.00E-05 | -0.0690 | 0 | 0.099 |
| ENSP00000201979 | 1.06E-05 | -0.0690 | 585 | 0.619 |
| ENSP00000376048 | 1.56E-05 | -0.0690 | 583 | 0.740 |
| ENSP00000311318 | 2.10E-05 | -0.0690 | 211 | 0.111 |
| ENSP00000337396 | 8.85E-06 | -0.0690 | 0 | 0.360 |
| ENSP00000241502 | 1.56E-05 | -0.0690 | 0 | 0.380 |
| ENSP00000307014 | 1.49E-05 | -0.0690 | 0 | 0.206 |
| ENSP00000340297 | 1.37E-05 | -0.0690 | 0 | 0.079 |
| ENSP00000232375 | 1.66E-05 | -0.0690 | 174 | 0.231 |
| ENSP00000237822 | 1.71E-05 | -0.0690 | 0 | 0.000 |
| ENSP00000272610 | 1.32E-05 | -0.0690 | 0 | 0.072 |
| ENSP00000376213 | 1.12E-06 | -0.0690 | 0 | 0.113 |
| ENSP00000237316 | 2.33E-05 | -0.0690 | 153 | 0.815 |
| ENSP00000290429 | 1.60E-05 | -0.0690 | 408 | 0.520 |
| ENSP00000260270 | 1.59E-05 | -0.0690 | 696 | 0.379 |
| ENSP00000258383 | 1.16E-05 | -0.0690 | 0 | 0.293 |
| ENSP00000304360 | 1.52E-05 | -0.0690 | 244 | 0.942 |
| ENSP00000263243 | 1.90E-05 | -0.0691 | 0 | 0.000 |
| ENSP00000243389 | 1.48E-05 | -0.0691 | 159 | 0.104 |
| ENSP00000360635 | 1.83E-05 | -0.0691 | 508 | 0.104 |
| ENSP00000337289 | 1.26E-05 | -0.0691 | 0 | 0.307 |
| ENSP00000315397 | 2.67E-05 | -0.0691 | 0 | 0.226 |
| ENSP00000396441 | 1.01E-05 | -0.0691 | 0 | 0.171 |
| ENSP00000342710 | 1.45E-05 | -0.0691 | 0 | 0.123 |
| ENSP00000293777 | 1.36E-05 | -0.0691 | 0 | 0.675 |
| ENSP00000363826 | 2.95E-05 | -0.0691 | 185 | 0.916 |
| ENSP00000263773 | 1.29E-05 | -0.0691 | 0 | 0.106 |
| ENSP00000275034 | 1.44E-05 | -0.0691 | 0 | 0.297 |

| | | | | |
|---|---|---|---|---|
| ENSP00000307423 | 1.24E-05 | -0.0691 | 0 | 0.116 |
| ENSP00000305702 | 1.85E-05 | -0.0691 | 305 | 0.928 |
| ENSP00000007264 | 1.92E-05 | -0.0691 | 236 | 0.636 |
| ENSP00000265881 | 2.19E-05 | -0.0691 | 235 | 0.474 |
| ENSP00000467176 | 5.67E-05 | -0.0691 | 906 | 0.869 |
| ENSP00000293273 | 1.92E-05 | -0.0691 | 0 | 0.000 |
| ENSP00000297508 | 1.90E-05 | -0.0691 | 338 | 0.458 |
| ENSP00000306275 | 2.10E-05 | -0.0691 | 214 | 0.320 |
| ENSP00000365536 | 5.84E-06 | -0.0691 | 0 | 0.000 |
| ENSP00000321988 | 1.55E-05 | -0.0691 | 165 | 0.073 |
| ENSP00000336914 | 1.05E-05 | -0.0692 | 0 | 0.184 |
| ENSP00000344307 | 1.55E-05 | -0.0692 | 0 | 0.203 |
| ENSP00000264917 | 1.31E-05 | -0.0692 | 0 | 0.450 |
| ENSP00000302640 | 2.05E-05 | -0.0692 | 269 | 0.893 |
| ENSP00000296591 | 7.37E-05 | -0.0692 | 512 | 0.251 |
| ENSP00000326238 | 2.16E-05 | -0.0692 | 253 | 0.385 |
| ENSP00000326858 | 1.37E-05 | -0.0692 | 0 | 0.190 |
| ENSP00000258772 | 1.84E-05 | -0.0692 | 178 | 0.899 |
| ENSP00000257600 | 3.46E-05 | -0.0692 | 292 | 0.796 |
| ENSP00000258098 | 1.77E-05 | -0.0692 | 0 | 0.114 |
| ENSP00000267023 | 1.29E-05 | -0.0692 | 0 | 0.289 |
| ENSP00000247026 | 1.80E-05 | -0.0692 | 0 | 0.329 |
| ENSP00000343021 | 1.03E-05 | -0.0692 | 0 | 0.234 |
| ENSP00000247270 | 1.39E-05 | -0.0692 | 0 | 0.251 |
| ENSP00000252992 | 1.61E-05 | -0.0692 | 0 | 0.112 |
| ENSP00000242607 | 1.40E-05 | -0.0692 | 0 | 0.144 |
| ENSP00000300061 | 1.71E-05 | -0.0692 | 392 | 0.538 |
| ENSP00000304544 | 1.45E-05 | -0.0692 | 0 | 0.532 |
| ENSP00000260818 | 2.05E-05 | -0.0692 | 242 | 0.166 |
| ENSP00000386884 | 3.22E-05 | -0.0692 | 412 | 0.744 |
| ENSP00000335044 | 2.70E-05 | -0.0692 | 193 | 0.000 |
| ENSP00000381302 | 9.24E-06 | -0.0692 | 0 | 0.000 |
| ENSP00000239830 | 1.91E-05 | -0.0692 | 0 | 0.141 |
| ENSP00000268766 | 1.53E-05 | -0.0693 | 359 | 0.382 |
| ENSP00000311677 | 2.11E-05 | -0.0693 | 261 | 0.639 |
| ENSP00000233557 | 1.55E-05 | -0.0693 | 0 | 0.623 |
| ENSP00000282382 | 1.21E-05 | -0.0693 | 0 | 0.114 |
| ENSP00000242591 | 1.44E-05 | -0.0693 | 0 | 0.075 |
| ENSP00000335203 | 8.45E-06 | -0.0693 | 0 | 0.196 |
| ENSP00000311453 | 1.07E-05 | -0.0693 | 0 | 0.138 |
| ENSP00000323714 | 2.25E-05 | -0.0693 | 352 | 0.895 |
| ENSP00000312634 | 2.28E-05 | -0.0693 | 394 | 0.553 |
| ENSP00000303585 | 1.80E-05 | -0.0693 | 288 | 0.438 |
| ENSP00000261187 | 1.73E-05 | -0.0693 | 0 | 0.123 |
| ENSP00000336627 | 1.31E-05 | -0.0693 | 0 | 0.118 |
| ENSP00000325423 | 1.16E-05 | -0.0693 | 0 | 0.106 |

| | | | | |
|---|---|---|---|---|
| ENSP00000337159 | 1.16E-05 | -0.0693 | 0 | 0.328 |
| ENSP00000288462 | 1.44E-05 | -0.0693 | 0 | 0.121 |
| ENSP00000295899 | 3.00E-05 | -0.0693 | 897 | 0.572 |
| ENSP00000302037 | 1.39E-05 | -0.0693 | 0 | 0.135 |
| ENSP00000304139 | 1.34E-05 | -0.0693 | 0 | 0.433 |
| ENSP00000325827 | 9.38E-06 | -0.0693 | 0 | 0.099 |
| ENSP00000262753 | 1.26E-05 | -0.0693 | 0 | 0.117 |
| ENSP00000261700 | 1.44E-05 | -0.0693 | 0 | 0.445 |
| ENSP00000271883 | 1.55E-05 | -0.0693 | 0 | 0.307 |
| ENSP00000296597 | 1.16E-05 | -0.0693 | 0 | 0.088 |
| ENSP00000281282 | 9.50E-06 | -0.0693 | 0 | 0.139 |
| ENSP00000276055 | 1.24E-05 | -0.0694 | 0 | 0.060 |
| ENSP00000391402 | 1.79E-05 | -0.0694 | 196 | 0.000 |
| ENSP00000308546 | 2.14E-05 | -0.0694 | 357 | 0.691 |
| ENSP00000268638 | 2.53E-05 | -0.0694 | 912 | 0.863 |
| ENSP00000314132 | 2.20E-05 | -0.0694 | 163 | 0.711 |
| ENSP00000264866 | 2.09E-05 | -0.0694 | 0 | 0.120 |
| ENSP00000256255 | 2.18E-05 | -0.0694 | 0 | 0.000 |
| ENSP00000302393 | 1.65E-05 | -0.0694 | 329 | 0.482 |
| ENSP00000315680 | 9.84E-06 | -0.0694 | 0 | 0.079 |
| ENSP00000322594 | 1.20E-05 | -0.0694 | 0 | 0.071 |
| ENSP00000419712 | 2.04E-07 | -0.0694 | 0 | 0.178 |
| ENSP00000348381 | 3.62E-05 | -0.0694 | 433 | 0.193 |
| ENSP00000313050 | 1.96E-05 | -0.0694 | 157 | 0.116 |
| ENSP00000279281 | 1.22E-05 | -0.0694 | 0 | 0.119 |
| ENSP00000304353 | 1.11E-05 | -0.0694 | 0 | 0.186 |
| ENSP00000310796 | 2.09E-05 | -0.0694 | 0 | 0.217 |
| ENSP00000233379 | 1.25E-05 | -0.0695 | 0 | 0.079 |
| ENSP00000249806 | 1.44E-05 | -0.0695 | 0 | 0.082 |
| ENSP00000311493 | 1.07E-05 | -0.0695 | 0 | 0.000 |
| ENSP00000335106 | 1.63E-05 | -0.0695 | 564 | 0.247 |
| ENSP00000304783 | 1.82E-05 | -0.0695 | 242 | 0.605 |
| ENSP00000262731 | 1.72E-05 | -0.0695 | 0 | 0.081 |
| ENSP00000270172 | 1.25E-05 | -0.0695 | 0 | 0.733 |
| ENSP00000264689 | 1.60E-05 | -0.0695 | 0 | 0.088 |
| ENSP00000338990 | 1.01E-05 | -0.0695 | 0 | 0.000 |
| ENSP00000216733 | 1.63E-05 | -0.0695 | 0 | 0.430 |
| ENSP00000268711 | 1.60E-05 | -0.0695 | 0 | 0.630 |
| ENSP00000365962 | 5.77E-06 | -0.0695 | 0 | 0.201 |
| ENSP00000318318 | 2.10E-05 | -0.0695 | 358 | 0.354 |
| ENSP00000288078 | 1.09E-05 | -0.0695 | 0 | 0.237 |
| ENSP00000341342 | 1.16E-05 | -0.0695 | 0 | 0.175 |
| ENSP00000235835 | 1.27E-05 | -0.0695 | 0 | 0.075 |
| ENSP00000304843 | 1.32E-05 | -0.0695 | 0 | 0.000 |
| ENSP00000246174 | 1.07E-05 | -0.0695 | 0 | 0.141 |
| ENSP00000312244 | 2.43E-05 | -0.0695 | 339 | 0.937 |

| | | | | |
|---|---|---|---|---|
| ENSP00000296564 | 2.71E-05 | -0.0695 | 0 | 0.000 |
| ENSP00000309749 | 1.28E-05 | -0.0695 | 0 | 0.077 |
| ENSP00000256429 | 1.84E-05 | -0.0695 | 0 | 0.815 |
| ENSP00000261386 | 1.76E-05 | -0.0695 | 215 | 0.131 |
| ENSP00000325929 | 1.98E-05 | -0.0695 | 0 | 0.133 |
| ENSP00000362068 | 8.47E-06 | -0.0696 | 0 | 0.811 |
| ENSP00000297562 | 1.37E-05 | -0.0696 | 0 | 0.130 |
| ENSP00000369682 | 1.22E-05 | -0.0696 | 0 | 0.447 |
| ENSP00000216452 | 1.56E-05 | -0.0696 | 0 | 0.060 |
| ENSP00000262633 | 1.28E-05 | -0.0696 | 0 | 0.483 |
| ENSP00000260598 | 1.27E-05 | -0.0696 | 0 | 0.195 |
| ENSP00000330875 | 9.85E-06 | -0.0696 | 0 | 0.675 |
| ENSP00000330945 | 1.34E-05 | -0.0696 | 201 | 0.110 |
| ENSP00000313752 | 1.79E-05 | -0.0696 | 201 | 0.092 |
| ENSP00000216129 | 1.07E-05 | -0.0696 | 0 | 0.289 |
| ENSP00000220853 | 1.33E-05 | -0.0696 | 0 | 0.087 |
| ENSP00000332034 | 1.34E-05 | -0.0697 | 0 | 0.163 |
| ENSP00000305769 | 4.12E-05 | -0.0697 | 500 | 0.847 |
| ENSP00000265462 | 1.78E-05 | -0.0697 | 285 | 0.341 |
| ENSP00000294360 | 9.36E-06 | -0.0697 | 0 | 0.272 |
| ENSP00000286371 | 1.09E-05 | -0.0697 | 0 | 0.095 |
| ENSP00000244061 | 1.29E-05 | -0.0697 | 0 | 0.211 |
| ENSP00000341117 | 3.14E-05 | -0.0697 | 171 | 0.392 |
| ENSP00000303758 | 1.29E-05 | -0.0697 | 0 | 0.100 |
| ENSP00000318131 | 1.31E-05 | -0.0697 | 0 | 0.109 |
| ENSP00000310406 | 1.09E-05 | -0.0697 | 0 | 0.685 |
| ENSP00000343398 | 1.72E-06 | -0.0697 | 0 | 0.187 |
| ENSP00000268896 | 1.22E-05 | -0.0697 | 0 | 0.217 |
| ENSP00000329915 | 1.12E-05 | -0.0697 | 0 | 0.183 |
| ENSP00000215739 | 1.14E-05 | -0.0697 | 0 | 0.446 |
| ENSP00000323288 | 1.78E-05 | -0.0697 | 236 | 0.661 |
| ENSP00000085068 | 1.52E-05 | -0.0697 | 0 | 0.103 |
| ENSP00000290291 | 1.06E-05 | -0.0697 | 0 | 0.098 |
| ENSP00000264436 | 2.33E-05 | -0.0698 | 470 | 0.234 |
| ENSP00000011691 | 1.35E-05 | -0.0698 | 0 | 0.376 |
| ENSP00000301765 | 1.03E-05 | -0.0698 | 0 | 0.067 |
| ENSP00000258711 | 1.63E-05 | -0.0698 | 0 | 0.078 |
| ENSP00000238379 | 9.71E-06 | -0.0698 | 0 | 0.103 |
| ENSP00000225927 | 1.23E-05 | -0.0698 | 0 | 0.056 |
| ENSP00000243918 | 1.30E-05 | -0.0698 | 0 | 0.121 |
| ENSP00000315118 | 8.63E-06 | -0.0698 | 0 | 0.060 |
| ENSP00000309532 | 1.33E-05 | -0.0698 | 0 | 0.081 |
| ENSP00000457957 | 9.34E-07 | -0.0698 | 0 | 0.000 |
| ENSP00000341848 | 1.15E-05 | -0.0698 | 169 | 0.238 |
| ENSP00000324491 | 9.68E-06 | -0.0698 | 0 | 0.062 |
| ENSP00000238831 | 1.33E-05 | -0.0698 | 0 | 0.207 |

| | | | | |
|---|---|---|---|---|
| ENSP00000259698 | 9.95E-06 | -0.0698 | 0 | 0.123 |
| ENSP00000217121 | 1.37E-05 | -0.0698 | 0 | 0.108 |
| ENSP00000330488 | 8.19E-06 | -0.0698 | 0 | 0.108 |
| ENSP00000319678 | 1.27E-05 | -0.0698 | 0 | 0.080 |
| ENSP00000269346 | 1.20E-05 | -0.0698 | 0 | 0.069 |
| ENSP00000263150 | 1.35E-05 | -0.0698 | 0 | 0.133 |
| ENSP00000342905 | 1.70E-05 | -0.0698 | 0 | 0.485 |
| ENSP00000312734 | 2.17E-05 | -0.0698 | 687 | 0.272 |
| ENSP00000284154 | 2.37E-05 | -0.0698 | 320 | 0.815 |
| ENSP00000315568 | 9.31E-06 | -0.0699 | 0 | 0.121 |
| ENSP00000263702 | 1.92E-05 | -0.0699 | 165 | 0.185 |
| ENSP00000284669 | 8.69E-06 | -0.0699 | 0 | 0.087 |
| ENSP00000202017 | 1.57E-05 | -0.0699 | 0 | 0.109 |
| ENSP00000326699 | 1.88E-05 | -0.0699 | 721 | 0.179 |
| ENSP00000323782 | 1.59E-05 | -0.0699 | 0 | 0.084 |
| ENSP00000333947 | 1.62E-05 | -0.0699 | 0 | 0.057 |
| ENSP00000300147 | 1.13E-05 | -0.0699 | 0 | 0.000 |
| ENSP00000285393 | 1.86E-05 | -0.0699 | 178 | 0.084 |
| ENSP00000272198 | 1.18E-05 | -0.0699 | 0 | 0.098 |
| ENSP00000365838 | 6.63E-06 | -0.0699 | 0 | 0.687 |
| ENSP00000300213 | 1.00E-05 | -0.0699 | 0 | 0.382 |
| ENSP00000259199 | 1.14E-05 | -0.0699 | 0 | 0.221 |
| ENSP00000268676 | 1.63E-05 | -0.0699 | 0 | 0.087 |
| ENSP00000194214 | 1.33E-05 | -0.0699 | 0 | 0.084 |
| ENSP00000336994 | 9.82E-06 | -0.0699 | 0 | 0.090 |
| ENSP00000309166 | 1.21E-05 | -0.0699 | 0 | 0.536 |
| ENSP00000039989 | 1.46E-05 | -0.0700 | 0 | 0.145 |
| ENSP00000301178 | 5.34E-05 | -0.0700 | 288 | 0.679 |
| ENSP00000380349 | 3.57E-05 | -0.0700 | 346 | 0.673 |
| ENSP00000329213 | 1.15E-05 | -0.0700 | 0 | 0.218 |
| ENSP00000325663 | 1.94E-05 | -0.0700 | 212 | 0.838 |
| ENSP00000295324 | 1.19E-05 | -0.0700 | 0 | 0.151 |
| ENSP00000289316 | 1.69E-05 | -0.0700 | 852 | 0.957 |
| ENSP00000260746 | 1.64E-05 | -0.0700 | 216 | 0.065 |
| ENSP00000320508 | 8.95E-06 | -0.0700 | 0 | 0.177 |
| ENSP00000266604 | 1.20E-05 | -0.0700 | 0 | 0.519 |
| ENSP00000261813 | 1.04E-05 | -0.0700 | 0 | 0.285 |
| ENSP00000262319 | 1.46E-05 | -0.0700 | 0 | 0.251 |
| ENSP00000297056 | 1.06E-05 | -0.0700 | 0 | 0.081 |
| ENSP00000296658 | 1.28E-05 | -0.0700 | 0 | 0.050 |
| ENSP00000278174 | 1.31E-05 | -0.0700 | 0 | 0.087 |
| ENSP00000371661 | 4.21E-05 | -0.0700 | 162 | 0.236 |
| ENSP00000265967 | 1.34E-05 | -0.0700 | 0 | 0.093 |
| ENSP00000301015 | 1.26E-05 | -0.0700 | 0 | 0.085 |
| ENSP00000219400 | 1.26E-05 | -0.0700 | 0 | 0.115 |
| ENSP00000216455 | 1.28E-05 | -0.0700 | 247 | 0.507 |

| | | | | |
|---|---|---|---|---|
| ENSP00000295500 | 8.97E-06 | -0.0700 | 0 | 0.140 |
| ENSP00000262817 | 1.29E-05 | -0.0700 | 0 | 0.168 |
| ENSP00000299563 | 1.56E-05 | -0.0700 | 0 | 0.361 |
| ENSP00000274376 | 1.75E-05 | -0.0700 | 210 | 0.833 |
| ENSP00000333821 | 1.69E-05 | -0.0700 | 323 | 0.559 |
| ENSP00000255784 | 1.57E-05 | -0.0700 | 0 | 0.096 |
| ENSP00000343966 | 8.82E-06 | -0.0700 | 0 | 0.568 |
| ENSP00000264605 | 1.21E-05 | -0.0700 | 0 | 0.126 |
| ENSP00000328088 | 1.56E-05 | -0.0700 | 925 | 0.412 |
| ENSP00000362057 | 7.65E-05 | -0.0701 | 939 | 0.889 |
| ENSP00000348944 | 3.01E-05 | -0.0701 | 302 | 0.565 |
| ENSP00000325594 | 9.54E-06 | -0.0701 | 0 | 0.086 |
| ENSP00000223642 | 1.61E-05 | -0.0701 | 0 | 0.487 |
| ENSP00000341743 | 1.13E-05 | -0.0701 | 0 | 0.243 |
| ENSP00000338885 | 1.06E-05 | -0.0701 | 0 | 0.093 |
| ENSP00000261844 | 1.26E-05 | -0.0701 | 0 | 0.122 |
| ENSP00000219054 | 1.49E-05 | -0.0701 | 183 | 0.384 |
| ENSP00000268099 | 1.01E-05 | -0.0701 | 0 | 0.158 |
| ENSP00000295727 | 1.02E-05 | -0.0701 | 0 | 0.664 |
| ENSP00000300087 | 9.17E-06 | -0.0701 | 0 | 0.250 |
| ENSP00000269373 | 1.02E-05 | -0.0701 | 0 | 0.105 |
| ENSP00000323659 | 1.94E-05 | -0.0701 | 153 | 0.782 |
| ENSP00000262843 | 1.23E-05 | -0.0701 | 0 | 0.176 |
| ENSP00000312235 | 1.68E-05 | -0.0701 | 196 | 0.000 |
| ENSP00000306344 | 9.98E-06 | -0.0701 | 0 | 0.130 |
| ENSP00000298894 | 1.36E-05 | -0.0701 | 0 | 0.321 |
| ENSP00000257696 | 1.43E-05 | -0.0702 | 0 | 0.086 |
| ENSP00000258169 | 1.08E-05 | -0.0702 | 0 | 0.280 |
| ENSP00000344903 | 8.17E-06 | -0.0702 | 0 | 0.655 |
| ENSP00000299601 | 3.44E-05 | -0.0702 | 317 | 0.900 |
| ENSP00000339428 | 2.29E-05 | -0.0702 | 214 | 0.886 |
| ENSP00000255613 | 1.05E-05 | -0.0702 | 0 | 0.425 |
| ENSP00000259075 | 3.55E-05 | -0.0702 | 178 | 0.719 |
| ENSP00000285599 | 1.00E-05 | -0.0702 | 0 | 0.081 |
| ENSP00000343435 | 1.15E-05 | -0.0702 | 176 | 0.097 |
| ENSP00000303423 | 9.00E-06 | -0.0702 | 0 | 0.000 |
| ENSP00000261600 | 1.21E-05 | -0.0702 | 0 | 0.495 |
| ENSP00000292385 | 1.33E-05 | -0.0702 | 0 | 0.227 |
| ENSP00000322020 | 1.07E-05 | -0.0702 | 0 | 0.094 |
| ENSP00000262215 | 1.95E-05 | -0.0702 | 191 | 0.221 |
| ENSP00000349087 | 9.59E-06 | -0.0702 | 0 | 0.139 |
| ENSP00000353701 | 2.05E-05 | -0.0702 | 408 | 0.000 |
| ENSP00000262580 | 1.70E-05 | -0.0702 | 0 | 0.134 |
| ENSP00000294454 | 1.25E-05 | -0.0702 | 0 | 0.072 |
| ENSP00000258398 | 9.15E-06 | -0.0703 | 0 | 0.079 |
| ENSP00000323339 | 9.68E-06 | -0.0703 | 0 | 0.086 |

| | | | | |
|---|---|---|---|---|
| ENSP00000261866 | 1.30E-05 | -0.0703 | 0 | 0.082 |
| ENSP00000329554 | 1.76E-05 | -0.0703 | 772 | 0.000 |
| ENSP00000380998 | 5.51E-05 | -0.0703 | 0 | 0.060 |
| ENSP00000377262 | 9.13E-04 | -0.0703 | 440 | 0.790 |
| ENSP00000263681 | 1.16E-05 | -0.0703 | 0 | 0.518 |
| ENSP00000322316 | 1.08E-05 | -0.0703 | 0 | 0.097 |
| ENSP00000323780 | 1.16E-05 | -0.0703 | 0 | 0.046 |
| ENSP00000321347 | 1.09E-05 | -0.0703 | 0 | 0.391 |
| ENSP00000279593 | 2.29E-05 | -0.0703 | 325 | 0.000 |
| ENSP00000314606 | 1.17E-05 | -0.0703 | 0 | 0.104 |
| ENSP00000267202 | 1.06E-05 | -0.0703 | 0 | 0.110 |
| ENSP00000286548 | 2.47E-05 | -0.0703 | 450 | 0.840 |
| ENSP00000298902 | 1.08E-05 | -0.0703 | 0 | 0.000 |
| ENSP00000316854 | 1.24E-05 | -0.0704 | 0 | 0.055 |
| ENSP00000329102 | 8.83E-06 | -0.0704 | 0 | 0.652 |
| ENSP00000334329 | 1.43E-05 | -0.0704 | 0 | 0.631 |
| ENSP00000247881 | 2.73E-05 | -0.0704 | 151 | 0.479 |
| ENSP00000287218 | 1.42E-05 | -0.0704 | 0 | 0.111 |
| ENSP00000344609 | 1.13E-05 | -0.0704 | 0 | 0.562 |
| ENSP00000323837 | 1.29E-05 | -0.0704 | 0 | 0.207 |
| ENSP00000258947 | 1.49E-05 | -0.0704 | 172 | 0.441 |
| ENSP00000329858 | 8.77E-06 | -0.0704 | 0 | 0.366 |
| ENSP00000295240 | 2.99E-05 | -0.0704 | 381 | 0.218 |
| ENSP00000320917 | 1.63E-05 | -0.0704 | 154 | 0.891 |
| ENSP00000258390 | 1.10E-05 | -0.0704 | 0 | 0.157 |
| ENSP00000323927 | 2.41E-05 | -0.0704 | 204 | 0.150 |
| ENSP00000217429 | 9.79E-06 | -0.0704 | 0 | 0.196 |
| ENSP00000342109 | 1.68E-05 | -0.0705 | 819 | 0.315 |
| ENSP00000315035 | 1.40E-05 | -0.0705 | 309 | 0.781 |
| ENSP00000342015 | 7.60E-06 | -0.0705 | 0 | 0.064 |
| ENSP00000259050 | 1.54E-05 | -0.0705 | 205 | 0.279 |
| ENSP00000358067 | 9.92E-06 | -0.0705 | 0 | 0.000 |
| ENSP00000285071 | 1.22E-05 | -0.0705 | 0 | 0.000 |
| ENSP00000261811 | 1.09E-05 | -0.0705 | 0 | 0.110 |
| ENSP00000337675 | 1.26E-05 | -0.0705 | 0 | 0.372 |
| ENSP00000328203 | 9.01E-06 | -0.0705 | 0 | 0.339 |
| ENSP00000267750 | 1.04E-05 | -0.0705 | 0 | 0.160 |
| ENSP00000306261 | 1.26E-05 | -0.0705 | 0 | 0.102 |
| ENSP00000251289 | 1.04E-05 | -0.0705 | 0 | 0.412 |
| ENSP00000238341 | 1.11E-05 | -0.0705 | 0 | 0.066 |
| ENSP00000262482 | 1.01E-05 | -0.0705 | 0 | 0.089 |
| ENSP00000246868 | 2.17E-05 | -0.0705 | 352 | 0.631 |
| ENSP00000303992 | 1.32E-05 | -0.0705 | 0 | 0.072 |
| ENSP00000318822 | 1.62E-05 | -0.0705 | 191 | 0.480 |
| ENSP00000258991 | 8.47E-06 | -0.0705 | 0 | 0.133 |
| ENSP00000222247 | 1.61E-05 | -0.0705 | 177 | 0.851 |

| | | | | |
|---|---|---|---|---|
| ENSP00000246747 | 1.29E-05 | -0.0706 | 0 | 0.127 |
| ENSP00000222284 | 1.22E-05 | -0.0706 | 0 | 0.094 |
| ENSP00000307701 | 1.13E-05 | -0.0706 | 0 | 0.094 |
| ENSP00000277540 | 1.30E-05 | -0.0706 | 0 | 0.000 |
| ENSP00000264538 | 3.64E-05 | -0.0706 | 266 | 0.508 |
| ENSP00000305529 | 1.88E-05 | -0.0706 | 917 | 0.164 |
| ENSP00000263253 | 3.11E-05 | -0.0706 | 657 | 0.000 |
| ENSP00000341843 | 1.47E-05 | -0.0706 | 0 | 0.806 |
| ENSP00000340505 | 2.30E-05 | -0.0706 | 165 | 0.341 |
| ENSP00000267540 | 1.11E-05 | -0.0706 | 0 | 0.107 |
| ENSP00000271373 | 1.17E-05 | -0.0706 | 0 | 0.086 |
| ENSP00000328358 | 1.16E-05 | -0.0706 | 0 | 0.564 |
| ENSP00000347119 | 2.12E-05 | -0.0706 | 819 | 0.955 |
| ENSP00000233668 | 1.88E-05 | -0.0706 | 0 | 0.615 |
| ENSP00000304710 | 1.20E-05 | -0.0706 | 0 | 0.120 |
| ENSP00000344666 | 3.30E-05 | -0.0706 | 197 | 0.879 |
| ENSP00000072869 | 1.13E-05 | -0.0706 | 0 | 0.069 |
| ENSP00000369372 | 6.40E-06 | -0.0706 | 0 | 0.165 |
| ENSP00000279036 | 1.08E-05 | -0.0706 | 0 | 0.103 |
| ENSP00000264779 | 1.18E-05 | -0.0706 | 0 | 0.248 |
| ENSP00000258123 | 2.73E-05 | -0.0706 | 173 | 0.345 |
| ENSP00000293872 | 1.89E-05 | -0.0706 | 481 | 0.843 |
| ENSP00000260645 | 1.72E-05 | -0.0706 | 700 | 0.490 |
| ENSP00000261655 | 9.99E-06 | -0.0706 | 0 | 0.114 |
| ENSP00000341364 | 1.84E-05 | -0.0707 | 0 | 0.130 |
| ENSP00000392395 | 4.19E-05 | -0.0707 | 545 | 0.599 |
| ENSP00000224862 | 1.29E-05 | -0.0707 | 0 | 0.120 |
| ENSP00000356590 | 6.01E-07 | -0.0707 | 0 | 0.198 |
| ENSP00000318373 | 3.12E-05 | -0.0707 | 400 | 0.696 |
| ENSP00000081029 | 7.92E-06 | -0.0707 | 0 | 0.435 |
| ENSP00000297347 | 1.15E-05 | -0.0707 | 0 | 0.624 |
| ENSP00000339532 | 9.15E-06 | -0.0707 | 0 | 0.452 |
| ENSP00000263780 | 1.18E-05 | -0.0707 | 212 | 0.164 |
| ENSP00000243344 | 1.07E-05 | -0.0707 | 0 | 0.198 |
| ENSP00000340797 | 1.14E-05 | -0.0707 | 207 | 0.126 |
| ENSP00000297668 | 9.47E-06 | -0.0707 | 0 | 0.086 |
| ENSP00000341351 | 1.68E-05 | -0.0707 | 0 | 0.122 |
| ENSP00000252603 | 1.55E-05 | -0.0707 | 200 | 0.389 |
| ENSP00000310686 | 1.18E-05 | -0.0707 | 0 | 0.393 |
| ENSP00000307479 | 1.01E-05 | -0.0707 | 0 | 0.522 |
| ENSP00000303482 | 9.45E-06 | -0.0707 | 0 | 0.082 |
| ENSP00000239690 | 1.22E-05 | -0.0707 | 0 | 0.155 |
| ENSP00000310208 | 1.48E-05 | -0.0707 | 173 | 0.076 |
| ENSP00000257262 | 1.15E-05 | -0.0707 | 0 | 0.128 |
| ENSP00000270776 | 1.71E-05 | -0.0708 | 430 | 0.483 |
| ENSP00000253856 | 1.56E-05 | -0.0708 | 197 | 0.133 |

| | | | | |
|---|---|---|---|---|
| ENSP00000005260 | 1.31E-05 | -0.0708 | 0 | 0.128 |
| ENSP00000295685 | 1.95E-05 | -0.0708 | 461 | 0.427 |
| ENSP00000300589 | 2.97E-05 | -0.0708 | 567 | 0.813 |
| ENSP00000272217 | 9.50E-06 | -0.0708 | 0 | 0.089 |
| ENSP00000268231 | 7.84E-06 | -0.0708 | 0 | 0.202 |
| ENSP00000281017 | 1.49E-05 | -0.0708 | 195 | 0.277 |
| ENSP00000339952 | 1.27E-05 | -0.0708 | 0 | 0.613 |
| ENSP00000296557 | 1.21E-05 | -0.0708 | 0 | 0.086 |
| ENSP00000288774 | 1.17E-05 | -0.0708 | 0 | 0.164 |
| ENSP00000296490 | 1.72E-05 | -0.0708 | 258 | 0.745 |
| ENSP00000273308 | 9.28E-06 | -0.0708 | 0 | 0.000 |
| ENSP00000400591 | 3.68E-05 | -0.0708 | 561 | 0.779 |
| ENSP00000264169 | 9.17E-06 | -0.0708 | 0 | 0.626 |
| ENSP00000335579 | 8.09E-06 | -0.0708 | 0 | 0.000 |
| ENSP00000262960 | 2.01E-05 | -0.0708 | 623 | 0.249 |
| ENSP00000268661 | 1.66E-05 | -0.0708 | 437 | 0.838 |
| ENSP00000340409 | 8.94E-05 | -0.0708 | 359 | 0.707 |
| ENSP00000184266 | 9.03E-06 | -0.0709 | 0 | 0.184 |
| ENSP00000215862 | 1.07E-05 | -0.0709 | 0 | 0.148 |
| ENSP00000332287 | 1.40E-05 | -0.0709 | 226 | 0.092 |
| ENSP00000303222 | 1.04E-05 | -0.0709 | 256 | 0.137 |
| ENSP00000311399 | 9.23E-06 | -0.0709 | 0 | 0.092 |
| ENSP00000251453 | 1.39E-05 | -0.0709 | 763 | 0.782 |
| ENSP00000309430 | 1.00E-05 | -0.0709 | 0 | 0.118 |
| ENSP00000333277 | 1.78E-05 | -0.0709 | 702 | 0.956 |
| ENSP00000369003 | 3.76E-05 | -0.0709 | 312 | 0.000 |
| ENSP00000216479 | 1.57E-05 | -0.0709 | 406 | 0.352 |
| ENSP00000305442 | 8.30E-06 | -0.0709 | 0 | 0.079 |
| ENSP00000262461 | 1.50E-05 | -0.0709 | 181 | 0.314 |
| ENSP00000217740 | 1.09E-05 | -0.0709 | 0 | 0.086 |
| ENSP00000440586 | 2.34E-05 | -0.0709 | 194 | 0.000 |
| ENSP00000253048 | 1.17E-05 | -0.0709 | 0 | 0.632 |
| ENSP00000369419 | 1.16E-05 | -0.0709 | 176 | 0.135 |
| ENSP00000270176 | 1.25E-05 | -0.0710 | 0 | 0.089 |
| ENSP00000311083 | 1.57E-05 | -0.0710 | 206 | 0.586 |
| ENSP00000227135 | 9.67E-06 | -0.0710 | 0 | 0.089 |
| ENSP00000354960 | 5.29E-05 | -0.0710 | 259 | 0.079 |
| ENSP00000221265 | 1.47E-05 | -0.0710 | 173 | 0.609 |
| ENSP00000232603 | 1.08E-05 | -0.0710 | 0 | 0.122 |
| ENSP00000299314 | 1.32E-05 | -0.0710 | 302 | 0.316 |
| ENSP00000298292 | 8.16E-06 | -0.0710 | 0 | 0.072 |
| ENSP00000366404 | 2.62E-05 | -0.0710 | 171 | 0.658 |
| ENSP00000326604 | 1.07E-05 | -0.0710 | 0 | 0.696 |
| ENSP00000307833 | 8.41E-06 | -0.0710 | 0 | 0.069 |
| ENSP00000329418 | 7.74E-05 | -0.0710 | 499 | 0.919 |
| ENSP00000285279 | 1.02E-05 | -0.0710 | 0 | 0.069 |

| | | | | |
|---|---|---|---|---|
| ENSP00000266682 | 9.20E-06 | -0.0710 | 0 | 0.066 |
| ENSP00000361177 | 2.37E-05 | -0.0710 | 204 | 0.583 |
| ENSP00000330060 | 8.92E-06 | -0.0710 | 0 | 0.333 |
| ENSP00000405610 | 4.43E-06 | -0.0710 | 0 | 0.000 |
| ENSP00000286364 | 1.01E-05 | -0.0710 | 0 | 0.575 |
| ENSP00000331682 | 7.26E-06 | -0.0711 | 0 | 0.144 |
| ENSP00000384026 | 7.89E-05 | -0.0711 | 362 | 0.857 |
| ENSP00000329452 | 8.67E-06 | -0.0711 | 0 | 0.071 |
| ENSP00000296015 | 1.50E-05 | -0.0711 | 0 | 0.125 |
| ENSP00000313034 | 9.51E-06 | -0.0711 | 0 | 0.234 |
| ENSP00000310332 | 9.42E-06 | -0.0711 | 0 | 0.240 |
| ENSP00000236980 | 1.28E-05 | -0.0711 | 0 | 0.133 |
| ENSP00000328062 | 9.43E-06 | -0.0711 | 0 | 0.136 |
| ENSP00000217456 | 9.99E-06 | -0.0711 | 0 | 0.053 |
| ENSP00000344779 | 1.22E-05 | -0.0711 | 0 | 0.124 |
| ENSP00000216832 | 1.31E-05 | -0.0711 | 0 | 0.588 |
| ENSP00000380334 | 1.90E-05 | -0.0711 | 920 | 0.606 |
| ENSP00000266970 | 3.14E-05 | -0.0711 | 879 | 0.000 |
| ENSP00000264935 | 9.04E-06 | -0.0711 | 0 | 0.055 |
| ENSP00000239223 | 4.34E-05 | -0.0711 | 846 | 0.909 |
| ENSP00000248378 | 1.05E-05 | -0.0711 | 0 | 0.117 |
| ENSP00000322730 | 1.71E-05 | -0.0711 | 265 | 0.263 |
| ENSP00000296043 | 7.82E-06 | -0.0711 | 0 | 0.104 |
| ENSP00000245787 | 2.79E-05 | -0.0711 | 511 | 0.541 |
| ENSP00000320688 | 1.04E-05 | -0.0711 | 0 | 0.066 |
| ENSP00000252445 | 1.30E-05 | -0.0711 | 0 | 0.376 |
| ENSP00000255945 | 2.14E-05 | -0.0711 | 0 | 0.169 |
| ENSP00000222307 | 1.10E-05 | -0.0712 | 0 | 0.068 |
| ENSP00000249442 | 8.75E-06 | -0.0712 | 0 | 0.291 |
| ENSP00000326342 | 1.43E-05 | -0.0712 | 0 | 0.129 |
| ENSP00000276533 | 8.80E-06 | -0.0712 | 0 | 0.554 |
| ENSP00000361818 | 9.56E-05 | -0.0712 | 937 | 0.858 |
| ENSP00000268482 | 1.89E-05 | -0.0712 | 430 | 0.934 |
| ENSP00000358778 | 1.70E-05 | -0.0712 | 0 | 0.345 |
| ENSP00000262126 | 1.01E-05 | -0.0712 | 0 | 0.111 |
| ENSP00000286800 | 3.36E-05 | -0.0712 | 0 | 0.478 |
| ENSP00000252699 | 6.50E-05 | -0.0712 | 932 | 0.693 |
| ENSP00000332656 | 1.14E-05 | -0.0712 | 0 | 0.060 |
| ENSP00000317992 | 2.29E-05 | -0.0712 | 337 | 0.817 |
| ENSP00000364964 | 4.41E-06 | -0.0712 | 0 | 0.127 |
| ENSP00000395929 | 4.84E-05 | -0.0712 | 309 | 0.817 |
| ENSP00000252512 | 1.35E-05 | -0.0712 | 0 | 0.178 |
| ENSP00000266732 | 9.91E-06 | -0.0712 | 0 | 0.474 |
| ENSP00000227471 | 1.08E-05 | -0.0712 | 0 | 0.347 |
| ENSP00000265107 | 9.66E-06 | -0.0712 | 0 | 0.451 |
| ENSP00000290942 | 1.21E-05 | -0.0713 | 163 | 0.542 |

| | | | | |
|---|---|---|---|---|
| ENSP00000220822 | 1.04E-05 | -0.0713 | 0 | 0.092 |
| ENSP00000269187 | 8.09E-06 | -0.0713 | 0 | 0.095 |
| ENSP00000305654 | 1.11E-05 | -0.0713 | 0 | 0.089 |
| ENSP00000292147 | 9.08E-06 | -0.0713 | 0 | 0.112 |
| ENSP00000333433 | 7.27E-06 | -0.0713 | 0 | 0.309 |
| ENSP00000358640 | 3.44E-05 | -0.0713 | 193 | 0.149 |
| ENSP00000216330 | 1.20E-05 | -0.0713 | 246 | 0.752 |
| ENSP00000263578 | 1.03E-05 | -0.0713 | 0 | 0.166 |
| ENSP00000260282 | 9.14E-06 | -0.0713 | 0 | 0.118 |
| ENSP00000307321 | 1.08E-05 | -0.0713 | 0 | 0.767 |
| ENSP00000299565 | 9.80E-06 | -0.0713 | 0 | 0.456 |
| ENSP00000333401 | 7.18E-06 | -0.0713 | 0 | 0.468 |
| ENSP00000265870 | 1.33E-05 | -0.0714 | 197 | 0.000 |
| ENSP00000005386 | 1.05E-05 | -0.0714 | 0 | 0.228 |
| ENSP00000360441 | 6.41E-06 | -0.0714 | 0 | 0.715 |
| ENSP00000344572 | 1.32E-05 | -0.0714 | 0 | 0.174 |
| ENSP00000311609 | 8.03E-06 | -0.0714 | 0 | 0.201 |
| ENSP00000221480 | 1.07E-05 | -0.0714 | 0 | 0.129 |
| ENSP00000262498 | 1.85E-05 | -0.0714 | 0 | 0.000 |
| ENSP00000282026 | 2.27E-05 | -0.0714 | 274 | 0.504 |
| ENSP00000219905 | 1.10E-05 | -0.0714 | 0 | 0.779 |
| ENSP00000235932 | 1.00E-05 | -0.0714 | 0 | 0.000 |
| ENSP00000261488 | 1.30E-05 | -0.0714 | 0 | 0.287 |
| ENSP00000219172 | 9.03E-06 | -0.0714 | 0 | 0.000 |
| ENSP00000276420 | 1.02E-05 | -0.0714 | 0 | 0.780 |
| ENSP00000285879 | 7.96E-06 | -0.0714 | 0 | 0.184 |
| ENSP00000335246 | 7.94E-06 | -0.0714 | 0 | 0.118 |
| ENSP00000309163 | 7.72E-06 | -0.0714 | 0 | 0.185 |
| ENSP00000312671 | 2.14E-05 | -0.0714 | 527 | 0.522 |
| ENSP00000254654 | 1.29E-05 | -0.0714 | 395 | 0.592 |
| ENSP00000319778 | 1.83E-05 | -0.0714 | 0 | 0.116 |
| ENSP00000354159 | 2.05E-05 | -0.0714 | 702 | 0.539 |
| ENSP00000309899 | 8.31E-06 | -0.0714 | 0 | 0.236 |
| ENSP00000199706 | 9.16E-06 | -0.0714 | 0 | 0.272 |
| ENSP00000231509 | 4.94E-05 | -0.0714 | 391 | 0.870 |
| ENSP00000337926 | 1.06E-05 | -0.0714 | 0 | 0.544 |
| ENSP00000384224 | 7.50E-07 | -0.0714 | 0 | 0.000 |
| ENSP00000164305 | 1.09E-05 | -0.0714 | 0 | 0.071 |
| ENSP00000372910 | 1.22E-07 | -0.0714 | 0 | 0.214 |
| ENSP00000321826 | 1.06E-05 | -0.0714 | 0 | 0.201 |
| ENSP00000244751 | 8.78E-06 | -0.0715 | 0 | 0.101 |
| ENSP00000317714 | 2.89E-05 | -0.0715 | 377 | 0.278 |
| ENSP00000266581 | 1.40E-05 | -0.0715 | 0 | 0.084 |
| ENSP00000343610 | 1.27E-05 | -0.0715 | 0 | 0.000 |
| ENSP00000321330 | 8.91E-06 | -0.0715 | 0 | 0.167 |
| ENSP00000243326 | 1.02E-05 | -0.0715 | 0 | 0.600 |

| | | | | |
|---|---|---|---|---|
| ENSP00000336725 | 2.68E-05 | -0.0715 | 235 | 0.841 |
| ENSP00000355718 | 5.36E-05 | -0.0715 | 281 | 0.924 |
| ENSP00000271234 | 9.07E-06 | -0.0715 | 0 | 0.249 |
| ENSP00000216085 | 1.14E-05 | -0.0715 | 0 | 0.080 |
| ENSP00000305699 | 9.20E-06 | -0.0715 | 0 | 0.067 |
| ENSP00000252250 | 1.21E-05 | -0.0715 | 0 | 0.153 |
| ENSP00000289547 | 1.24E-05 | -0.0715 | 564 | 0.436 |
| ENSP00000380177 | 2.11E-05 | -0.0715 | 866 | 0.955 |
| ENSP00000323076 | 1.02E-05 | -0.0715 | 302 | 0.143 |
| ENSP00000318147 | 3.93E-05 | -0.0715 | 378 | 0.190 |
| ENSP00000307214 | 1.02E-05 | -0.0715 | 0 | 0.191 |
| ENSP00000317469 | 1.41E-05 | -0.0715 | 457 | 0.000 |
| ENSP00000275053 | 8.91E-06 | -0.0715 | 0 | 0.213 |
| ENSP00000202556 | 1.15E-05 | -0.0715 | 0 | 0.649 |
| ENSP00000318986 | 1.91E-05 | -0.0715 | 178 | 0.857 |
| ENSP00000339381 | 1.68E-05 | -0.0716 | 188 | 0.448 |
| ENSP00000272797 | 8.65E-06 | -0.0716 | 0 | 0.132 |
| ENSP00000297293 | 1.09E-05 | -0.0716 | 0 | 0.359 |
| ENSP00000334808 | 8.62E-06 | -0.0716 | 0 | 0.417 |
| ENSP00000007390 | 1.36E-05 | -0.0716 | 0 | 0.154 |
| ENSP00000203556 | 1.01E-05 | -0.0716 | 0 | 0.167 |
| ENSP00000338019 | 3.18E-05 | -0.0716 | 926 | 0.496 |
| ENSP00000321845 | 1.90E-05 | -0.0716 | 270 | 0.186 |
| ENSP00000310088 | 1.02E-05 | -0.0716 | 0 | 0.421 |
| ENSP00000231948 | 1.11E-05 | -0.0716 | 0 | 0.132 |
| ENSP00000314363 | 1.95E-05 | -0.0716 | 166 | 0.659 |
| ENSP00000327104 | 1.35E-05 | -0.0716 | 0 | 0.118 |
| ENSP00000222728 | 1.02E-05 | -0.0716 | 0 | 0.760 |
| ENSP00000331532 | 5.65E-06 | -0.0716 | 157 | 0.155 |
| ENSP00000024061 | 1.14E-05 | -0.0716 | 0 | 0.069 |
| ENSP00000260662 | 9.22E-06 | -0.0716 | 0 | 0.306 |
| ENSP00000285805 | 1.10E-05 | -0.0716 | 0 | 0.263 |
| ENSP00000309092 | 1.89E-05 | -0.0716 | 202 | 0.204 |
| ENSP00000321744 | 1.79E-05 | -0.0717 | 899 | 0.948 |
| ENSP00000290039 | 7.12E-06 | -0.0717 | 0 | 0.095 |
| ENSP00000266014 | 1.24E-05 | -0.0717 | 0 | 0.116 |
| ENSP00000398017 | 1.89E-05 | -0.0717 | 152 | 0.109 |
| ENSP00000284116 | 2.02E-05 | -0.0717 | 215 | 0.098 |
| ENSP00000274853 | 1.45E-06 | -0.0717 | 0 | 0.218 |
| ENSP00000260563 | 1.70E-05 | -0.0717 | 336 | 0.877 |
| ENSP00000359674 | 9.30E-07 | -0.0717 | 0 | 0.196 |
| ENSP00000262219 | 1.29E-05 | -0.0717 | 153 | 0.176 |
| ENSP00000238018 | 1.03E-05 | -0.0717 | 0 | 0.106 |
| ENSP00000340466 | 1.57E-05 | -0.0717 | 451 | 0.287 |
| ENSP00000298717 | 9.21E-06 | -0.0717 | 0 | 0.404 |
| ENSP00000290765 | 1.57E-05 | -0.0717 | 336 | 0.609 |

| | | | | |
|---|---|---|---|---|
| ENSP00000323580 | 2.03E-05 | -0.0717 | 456 | 0.470 |
| ENSP00000298351 | 9.37E-06 | -0.0717 | 0 | 0.088 |
| ENSP00000307746 | 8.67E-06 | -0.0717 | 0 | 0.187 |
| ENSP00000319254 | 8.74E-06 | -0.0717 | 0 | 0.328 |
| ENSP00000384593 | 8.02E-06 | -0.0717 | 0 | 0.187 |
| ENSP00000258416 | 1.81E-05 | -0.0717 | 336 | 0.874 |
| ENSP00000339906 | 2.23E-05 | -0.0717 | 207 | 0.850 |
| ENSP00000251089 | 1.08E-05 | -0.0717 | 0 | 0.404 |
| ENSP00000263774 | 1.30E-05 | -0.0717 | 198 | 0.397 |
| ENSP00000257700 | 7.30E-06 | -0.0717 | 0 | 0.112 |
| ENSP00000307342 | 8.50E-06 | -0.0717 | 0 | 0.281 |
| ENSP00000331983 | 9.01E-06 | -0.0717 | 0 | 0.271 |
| ENSP00000292314 | 1.69E-05 | -0.0717 | 186 | 0.662 |
| ENSP00000317087 | 2.49E-05 | -0.0717 | 216 | 0.391 |
| ENSP00000216489 | 1.16E-05 | -0.0717 | 0 | 0.089 |
| ENSP00000381282 | 2.44E-05 | -0.0717 | 242 | 0.412 |
| ENSP00000254579 | 8.64E-06 | -0.0717 | 0 | 0.088 |
| ENSP00000260045 | 1.25E-05 | -0.0717 | 0 | 0.189 |
| ENSP00000326063 | 1.19E-05 | -0.0718 | 0 | 0.370 |
| ENSP00000311360 | 1.14E-05 | -0.0718 | 163 | 0.676 |
| ENSP00000263795 | 1.26E-05 | -0.0718 | 0 | 0.705 |
| ENSP00000343818 | 7.29E-06 | -0.0718 | 0 | 0.099 |
| ENSP00000282058 | 8.40E-06 | -0.0718 | 0 | 0.127 |
| ENSP00000263368 | 9.44E-06 | -0.0718 | 0 | 0.189 |
| ENSP00000431063 | 3.53E-05 | -0.0718 | 558 | 0.000 |
| ENSP00000339382 | 8.19E-06 | -0.0718 | 0 | 0.000 |
| ENSP00000297440 | 8.13E-06 | -0.0718 | 0 | 0.000 |
| ENSP00000260605 | 8.95E-06 | -0.0718 | 0 | 0.133 |
| ENSP00000277517 | 9.99E-06 | -0.0718 | 0 | 0.192 |
| ENSP00000295448 | 1.62E-05 | -0.0718 | 254 | 0.261 |
| ENSP00000273666 | 9.59E-06 | -0.0718 | 0 | 0.177 |
| ENSP00000300605 | 8.72E-06 | -0.0718 | 0 | 0.106 |
| ENSP00000279146 | 1.28E-05 | -0.0718 | 0 | 0.362 |
| ENSP00000265343 | 8.80E-06 | -0.0718 | 0 | 0.423 |
| ENSP00000441927 | 4.89E-05 | -0.0718 | 170 | 0.269 |
| ENSP00000265295 | 7.05E-06 | -0.0718 | 0 | 0.287 |
| ENSP00000216223 | 1.49E-05 | -0.0718 | 213 | 0.899 |
| ENSP00000239730 | 8.72E-06 | -0.0718 | 0 | 0.000 |
| ENSP00000263674 | 1.58E-05 | -0.0718 | 295 | 0.176 |
| ENSP00000239882 | 1.82E-05 | -0.0718 | 0 | 0.818 |
| ENSP00000286298 | 1.24E-05 | -0.0718 | 179 | 0.078 |
| ENSP00000265594 | 1.29E-05 | -0.0718 | 242 | 0.247 |
| ENSP00000223641 | 1.41E-05 | -0.0719 | 364 | 0.427 |
| ENSP00000377854 | 5.65E-06 | -0.0719 | 0 | 0.818 |
| ENSP00000279392 | 1.58E-05 | -0.0719 | 0 | 0.435 |
| ENSP00000344460 | 8.37E-05 | -0.0719 | 490 | 0.782 |

| | | | | |
|---|---|---|---|---|
| ENSP00000264042 | 1.18E-05 | -0.0719 | 0 | 0.249 |
| ENSP00000268797 | 8.38E-06 | -0.0719 | 0 | 0.143 |
| ENSP00000313121 | 1.24E-05 | -0.0719 | 0 | 0.102 |
| ENSP00000299178 | 2.42E-05 | -0.0719 | 303 | 0.573 |
| ENSP00000307854 | 2.01E-05 | -0.0719 | 190 | 0.468 |
| ENSP00000263864 | 2.71E-05 | -0.0719 | 346 | 0.252 |
| ENSP00000344155 | 1.71E-05 | -0.0719 | 293 | 0.255 |
| ENSP00000415998 | 1.82E-05 | -0.0719 | 0 | 0.075 |
| ENSP00000276585 | 8.79E-06 | -0.0719 | 0 | 0.355 |
| ENSP00000297151 | 1.62E-05 | -0.0719 | 278 | 0.888 |
| ENSP00000262094 | 1.27E-05 | -0.0719 | 159 | 0.141 |
| ENSP00000261523 | 4.38E-05 | -0.0719 | 490 | 0.816 |
| ENSP00000262585 | 1.07E-05 | -0.0719 | 0 | 0.079 |
| ENSP00000367102 | 2.61E-05 | -0.0719 | 160 | 0.072 |
| ENSP00000336888 | 1.12E-05 | -0.0719 | 0 | 0.115 |
| ENSP00000364865 | 9.41E-07 | -0.0719 | 0 | 0.000 |
| ENSP00000250056 | 8.14E-06 | -0.0719 | 0 | 0.122 |
| ENSP00000319713 | 8.99E-06 | -0.0720 | 0 | 0.079 |
| ENSP00000336923 | 7.41E-06 | -0.0720 | 0 | 0.492 |
| ENSP00000318115 | 1.67E-05 | -0.0720 | 164 | 0.583 |
| ENSP00000365851 | 7.06E-05 | -0.0720 | 844 | 0.799 |
| ENSP00000260324 | 1.34E-05 | -0.0720 | 318 | 0.000 |
| ENSP00000263956 | 9.65E-06 | -0.0720 | 0 | 0.677 |
| ENSP00000338165 | 1.60E-05 | -0.0720 | 0 | 0.137 |
| ENSP00000327072 | 9.55E-06 | -0.0720 | 0 | 0.667 |
| ENSP00000326477 | 1.65E-05 | -0.0720 | 244 | 0.929 |
| ENSP00000448220 | 2.86E-05 | -0.0720 | 150 | 0.263 |
| ENSP00000310521 | 3.60E-05 | -0.0720 | 201 | 0.240 |
| ENSP00000261755 | 1.10E-05 | -0.0720 | 0 | 0.222 |
| ENSP00000311368 | 1.16E-05 | -0.0720 | 0 | 0.409 |
| ENSP00000337500 | 7.39E-06 | -0.0720 | 0 | 0.616 |
| ENSP00000347433 | 1.63E-05 | -0.0720 | 159 | 0.409 |
| ENSP00000306253 | 2.89E-05 | -0.0720 | 345 | 0.773 |
| ENSP00000287008 | 1.39E-05 | -0.0720 | 0 | 0.144 |
| ENSP00000247003 | 1.64E-05 | -0.0720 | 228 | 0.903 |
| ENSP00000262265 | 9.36E-06 | -0.0720 | 0 | 0.328 |
| ENSP00000304422 | 1.25E-05 | -0.0720 | 345 | 0.518 |
| ENSP00000241436 | 1.27E-05 | -0.0720 | 165 | 0.632 |
| ENSP00000234256 | 1.22E-05 | -0.0721 | 396 | 0.107 |
| ENSP00000317159 | 1.44E-05 | -0.0721 | 159 | 0.361 |
| ENSP00000313500 | 8.80E-06 | -0.0721 | 0 | 0.125 |
| ENSP00000227525 | 1.31E-05 | -0.0721 | 0 | 0.109 |
| ENSP00000249910 | 9.33E-06 | -0.0721 | 0 | 0.770 |
| ENSP00000254835 | 1.53E-05 | -0.0721 | 202 | 0.000 |
| ENSP00000268220 | 1.60E-05 | -0.0721 | 446 | 0.446 |
| ENSP00000261368 | 1.82E-05 | -0.0721 | 287 | 0.740 |

| | | | | |
|---|---|---|---|---|
| ENSP00000299543 | 1.63E-05 | -0.0721 | 268 | 0.754 |
| ENSP00000310933 | 2.16E-05 | -0.0721 | 410 | 0.308 |
| ENSP00000302562 | 1.22E-05 | -0.0721 | 0 | 0.277 |
| ENSP00000336616 | 9.69E-06 | -0.0721 | 0 | 0.108 |
| ENSP00000251900 | 9.02E-06 | -0.0721 | 0 | 0.778 |
| ENSP00000322376 | 1.07E-05 | -0.0721 | 0 | 0.000 |
| ENSP00000306070 | 9.98E-06 | -0.0721 | 0 | 0.081 |
| ENSP00000260056 | 8.72E-06 | -0.0721 | 0 | 0.137 |
| ENSP00000239926 | 1.11E-05 | -0.0721 | 0 | 0.212 |
| ENSP00000331574 | 9.43E-06 | -0.0722 | 0 | 0.000 |
| ENSP00000297195 | 7.79E-06 | -0.0722 | 0 | 0.071 |
| ENSP00000340510 | 2.40E-05 | -0.0722 | 903 | 0.239 |
| ENSP00000266458 | 2.10E-05 | -0.0722 | 347 | 0.473 |
| ENSP00000303511 | 8.68E-06 | -0.0722 | 0 | 0.115 |
| ENSP00000317128 | 1.01E-05 | -0.0722 | 0 | 0.545 |
| ENSP00000263277 | 9.31E-06 | -0.0722 | 0 | 0.107 |
| ENSP00000260372 | 6.89E-06 | -0.0722 | 0 | 0.096 |
| ENSP00000272427 | 2.80E-05 | -0.0722 | 158 | 0.200 |
| ENSP00000253237 | 1.54E-05 | -0.0722 | 275 | 0.877 |
| ENSP00000286234 | 9.21E-06 | -0.0722 | 0 | 0.484 |
| ENSP00000264047 | 1.38E-05 | -0.0722 | 187 | 0.086 |
| ENSP00000260184 | 1.45E-05 | -0.0722 | 173 | 0.891 |
| ENSP00000290974 | 9.93E-06 | -0.0722 | 0 | 0.106 |
| ENSP00000337561 | 1.03E-05 | -0.0722 | 0 | 0.075 |
| ENSP00000339057 | 6.35E-06 | -0.0722 | 0 | 0.067 |
| ENSP00000283206 | 1.06E-05 | -0.0722 | 0 | 0.089 |
| ENSP00000324549 | 2.34E-05 | -0.0722 | 223 | 0.000 |
| ENSP00000002596 | 1.09E-05 | -0.0722 | 0 | 0.068 |
| ENSP00000315702 | 9.14E-06 | -0.0722 | 0 | 0.119 |
| ENSP00000264694 | 1.34E-05 | -0.0722 | 0 | 0.097 |
| ENSP00000267812 | 6.93E-05 | -0.0722 | 921 | 0.527 |
| ENSP00000312856 | 1.24E-05 | -0.0722 | 401 | 0.782 |
| ENSP00000262065 | 5.92E-05 | -0.0723 | 265 | 0.481 |
| ENSP00000238256 | 8.44E-06 | -0.0723 | 0 | 0.110 |
| ENSP00000300086 | 1.18E-05 | -0.0723 | 0 | 0.386 |
| ENSP00000411948 | 1.19E-06 | -0.0723 | 0 | 0.193 |
| ENSP00000283429 | 8.17E-06 | -0.0723 | 0 | 0.116 |
| ENSP00000265742 | 1.49E-05 | -0.0723 | 0 | 0.174 |
| ENSP00000263187 | 1.29E-05 | -0.0723 | 317 | 0.564 |
| ENSP00000245539 | 1.23E-05 | -0.0723 | 390 | 0.751 |
| ENSP00000341361 | 1.69E-05 | -0.0723 | 0 | 0.092 |
| ENSP00000354487 | 6.49E-05 | -0.0723 | 546 | 0.901 |
| ENSP00000263181 | 1.55E-05 | -0.0723 | 165 | 0.266 |
| ENSP00000318900 | 1.08E-05 | -0.0723 | 0 | 0.128 |
| ENSP00000230056 | 9.25E-06 | -0.0723 | 0 | 0.558 |
| ENSP00000297135 | 7.96E-06 | -0.0724 | 0 | 0.273 |

| | | | | |
|---|---|---|---|---|
| ENSP00000300255 | 1.05E-05 | -0.0724 | 0 | 0.108 |
| ENSP00000288050 | 1.71E-05 | -0.0724 | 229 | 0.322 |
| ENSP00000263925 | 2.42E-05 | -0.0724 | 190 | 0.119 |
| ENSP00000292852 | 1.48E-05 | -0.0724 | 0 | 0.104 |
| ENSP00000301364 | 1.83E-05 | -0.0724 | 167 | 0.891 |
| ENSP00000408527 | 1.11E-07 | -0.0724 | 0 | 0.195 |
| ENSP00000266483 | 7.98E-06 | -0.0724 | 0 | 0.049 |
| ENSP00000262577 | 8.80E-06 | -0.0724 | 0 | 0.659 |
| ENSP00000319918 | 1.04E-05 | -0.0724 | 0 | 0.103 |
| ENSP00000253686 | 7.64E-06 | -0.0724 | 0 | 0.328 |
| ENSP00000418813 | 1.77E-05 | -0.0724 | 0 | 0.000 |
| ENSP00000282032 | 1.13E-05 | -0.0724 | 0 | 0.301 |
| ENSP00000352273 | 8.51E-06 | -0.0724 | 0 | 0.000 |
| ENSP00000268668 | 1.75E-05 | -0.0724 | 152 | 0.262 |
| ENSP00000309141 | 3.02E-05 | -0.0725 | 205 | 0.208 |
| ENSP00000225740 | 1.25E-05 | -0.0725 | 225 | 0.336 |
| ENSP00000259008 | 1.29E-05 | -0.0725 | 201 | 0.726 |
| ENSP00000311857 | 2.31E-05 | -0.0725 | 181 | 0.855 |
| ENSP00000319992 | 1.18E-05 | -0.0725 | 181 | 0.235 |
| ENSP00000345107 | 3.87E-06 | -0.0725 | 0 | 0.120 |
| ENSP00000341737 | 8.73E-06 | -0.0725 | 0 | 0.110 |
| ENSP00000240100 | 1.42E-05 | -0.0725 | 197 | 0.843 |
| ENSP00000337697 | 5.36E-05 | -0.0725 | 533 | 0.909 |
| ENSP00000246914 | 9.29E-06 | -0.0725 | 0 | 0.279 |
| ENSP00000265175 | 1.56E-05 | -0.0725 | 275 | 0.177 |
| ENSP00000289119 | 9.62E-06 | -0.0725 | 0 | 0.089 |
| ENSP00000305632 | 6.78E-06 | -0.0725 | 0 | 0.735 |
| ENSP00000257951 | 8.76E-06 | -0.0725 | 0 | 0.160 |
| ENSP00000318629 | 1.42E-05 | -0.0725 | 0 | 0.061 |
| ENSP00000322991 | 2.54E-05 | -0.0725 | 291 | 0.502 |
| ENSP00000330199 | 1.58E-05 | -0.0726 | 0 | 0.106 |
| ENSP00000262879 | 9.69E-06 | -0.0726 | 0 | 0.144 |
| ENSP00000012049 | 1.13E-05 | -0.0726 | 0 | 0.076 |
| ENSP00000337194 | 8.57E-06 | -0.0726 | 0 | 0.589 |
| ENSP00000336940 | 1.19E-05 | -0.0726 | 0 | 0.280 |
| ENSP00000302114 | 9.12E-06 | -0.0726 | 0 | 0.160 |
| ENSP00000339479 | 2.15E-05 | -0.0726 | 242 | 0.166 |
| ENSP00000277165 | 1.08E-05 | -0.0726 | 0 | 0.184 |
| ENSP00000384015 | 9.33E-06 | -0.0726 | 0 | 0.279 |
| ENSP00000223129 | 7.41E-06 | -0.0726 | 0 | 0.654 |
| ENSP00000329219 | 1.04E-05 | -0.0726 | 351 | 0.195 |
| ENSP00000451451 | 2.63E-05 | -0.0726 | 824 | 0.000 |
| ENSP00000316990 | 6.56E-06 | -0.0726 | 0 | 0.086 |
| ENSP00000320623 | 8.24E-06 | -0.0726 | 0 | 0.140 |
| ENSP00000305204 | 6.80E-06 | -0.0726 | 0 | 0.195 |
| ENSP00000348128 | 2.85E-05 | -0.0726 | 720 | 0.282 |

| | | | | |
|---|---|---|---|---|
| ENSP00000270061 | 8.51E-06 | -0.0726 | 0 | 0.433 |
| ENSP00000344566 | 7.01E-06 | -0.0727 | 0 | 0.071 |
| ENSP00000233630 | 8.07E-06 | -0.0727 | 0 | 0.712 |
| ENSP00000254730 | 8.43E-06 | -0.0727 | 0 | 0.411 |
| ENSP00000303088 | 7.15E-06 | -0.0727 | 0 | 0.749 |
| ENSP00000293970 | 7.59E-06 | -0.0727 | 0 | 0.078 |
| ENSP00000072644 | 1.06E-05 | -0.0727 | 0 | 0.635 |
| ENSP00000283632 | 1.28E-05 | -0.0727 | 0 | 0.132 |
| ENSP00000312458 | 9.21E-06 | -0.0727 | 0 | 0.092 |
| ENSP00000367315 | 9.26E-06 | -0.0727 | 476 | 0.663 |
| ENSP00000248633 | 1.21E-05 | -0.0727 | 272 | 0.339 |
| ENSP00000343943 | 4.63E-05 | -0.0727 | 197 | 0.501 |
| ENSP00000252015 | 6.42E-06 | -0.0727 | 0 | 0.111 |
| ENSP00000253788 | 1.28E-05 | -0.0727 | 236 | 0.853 |
| ENSP00000292180 | 1.53E-05 | -0.0727 | 156 | 0.443 |
| ENSP00000224652 | 1.00E-05 | -0.0727 | 0 | 0.242 |
| ENSP00000237283 | 1.54E-05 | -0.0727 | 404 | 0.454 |
| ENSP00000229251 | 9.97E-06 | -0.0727 | 0 | 0.329 |
| ENSP00000232501 | 9.07E-06 | -0.0727 | 0 | 0.130 |
| ENSP00000305133 | 8.48E-06 | -0.0727 | 0 | 0.586 |
| ENSP00000322419 | 2.06E-05 | -0.0727 | 198 | 0.776 |
| ENSP00000311984 | 7.63E-06 | -0.0727 | 0 | 0.075 |
| ENSP00000237937 | 8.60E-06 | -0.0728 | 0 | 0.158 |
| ENSP00000251973 | 1.44E-05 | -0.0728 | 302 | 0.501 |
| ENSP00000245157 | 3.20E-05 | -0.0728 | 450 | 0.671 |
| ENSP00000216187 | 1.05E-05 | -0.0728 | 0 | 0.126 |
| ENSP00000313169 | 1.32E-05 | -0.0728 | 181 | 0.458 |
| ENSP00000011684 | 7.91E-06 | -0.0728 | 0 | 0.110 |
| ENSP00000074304 | 9.96E-06 | -0.0728 | 0 | 0.112 |
| ENSP00000261499 | 1.04E-05 | -0.0728 | 0 | 0.095 |
| ENSP00000210187 | 9.11E-06 | -0.0728 | 0 | 0.091 |
| ENSP00000301030 | 1.15E-05 | -0.0728 | 0 | 0.399 |
| ENSP00000322192 | 2.79E-05 | -0.0728 | 238 | 0.203 |
| ENSP00000272348 | 1.77E-05 | -0.0728 | 404 | 0.814 |
| ENSP00000219097 | 7.16E-06 | -0.0728 | 0 | 0.619 |
| ENSP00000276440 | 1.09E-05 | -0.0728 | 0 | 0.547 |
| ENSP00000282276 | 1.25E-05 | -0.0728 | 315 | 0.648 |
| ENSP00000306015 | 8.27E-06 | -0.0728 | 0 | 0.141 |
| ENSP00000328773 | 7.73E-06 | -0.0728 | 0 | 0.722 |
| ENSP00000365160 | 1.21E-05 | -0.0728 | 195 | 0.386 |
| ENSP00000221114 | 7.42E-06 | -0.0728 | 0 | 0.209 |
| ENSP00000342032 | 1.55E-05 | -0.0728 | 264 | 0.451 |
| ENSP00000307298 | 2.01E-05 | -0.0728 | 201 | 0.629 |
| ENSP00000002829 | 1.17E-05 | -0.0729 | 0 | 0.670 |
| ENSP00000263202 | 2.25E-05 | -0.0729 | 347 | 0.538 |
| ENSP00000324463 | 8.90E-06 | -0.0729 | 0 | 0.460 |

| | | | | |
|---|---|---|---|---|
| ENSP00000323099 | 7.38E-06 | -0.0729 | 0 | 0.701 |
| ENSP00000299736 | 1.13E-05 | -0.0729 | 0 | 0.450 |
| ENSP00000252826 | 9.50E-06 | -0.0729 | 0 | 0.262 |
| ENSP00000310120 | 7.81E-06 | -0.0729 | 0 | 0.046 |
| ENSP00000398003 | 4.56E-06 | -0.0729 | 0 | 0.000 |
| ENSP00000295121 | 9.83E-06 | -0.0729 | 0 | 0.122 |
| ENSP00000313432 | 7.48E-06 | -0.0729 | 0 | 0.218 |
| ENSP00000216259 | 1.49E-05 | -0.0729 | 192 | 0.368 |
| ENSP00000386165 | 1.05E-04 | -0.0729 | 250 | 0.846 |
| ENSP00000325919 | 9.33E-06 | -0.0730 | 0 | 0.285 |
| ENSP00000351783 | 5.47E-06 | -0.0730 | 0 | 0.111 |
| ENSP00000249344 | 7.04E-06 | -0.0730 | 0 | 0.090 |
| ENSP00000263946 | 1.30E-05 | -0.0730 | 545 | 0.577 |
| ENSP00000230640 | 1.34E-05 | -0.0730 | 364 | 0.912 |
| ENSP00000267978 | 1.48E-05 | -0.0730 | 270 | 0.174 |
| ENSP00000300452 | 1.12E-05 | -0.0730 | 0 | 0.064 |
| ENSP00000280551 | 1.64E-05 | -0.0730 | 280 | 0.186 |
| ENSP00000429430 | 2.97E-05 | -0.0730 | 188 | 0.122 |
| ENSP00000217246 | 9.13E-06 | -0.0730 | 0 | 0.109 |
| ENSP00000272238 | 6.81E-06 | -0.0730 | 0 | 0.097 |
| ENSP00000313385 | 2.25E-05 | -0.0730 | 0 | 0.122 |
| ENSP00000245680 | 8.01E-06 | -0.0730 | 0 | 0.046 |
| ENSP00000320434 | 8.35E-06 | -0.0730 | 0 | 0.269 |
| ENSP00000318313 | 8.85E-06 | -0.0730 | 0 | 0.224 |
| ENSP00000264057 | 1.13E-05 | -0.0730 | 0 | 0.091 |
| ENSP00000273258 | 1.24E-05 | -0.0730 | 210 | 0.131 |
| ENSP00000272418 | 1.38E-05 | -0.0730 | 390 | 0.814 |
| ENSP00000229829 | 3.72E-05 | -0.0730 | 0 | 0.546 |
| ENSP00000311888 | 1.01E-05 | -0.0730 | 191 | 0.233 |
| ENSP00000378464 | 1.45E-05 | -0.0730 | 211 | 0.207 |
| ENSP00000296741 | 8.33E-06 | -0.0730 | 0 | 0.081 |
| ENSP00000376258 | 2.03E-05 | -0.0730 | 214 | 0.253 |
| ENSP00000288199 | 2.19E-05 | -0.0730 | 347 | 0.557 |
| ENSP00000254630 | 8.59E-06 | -0.0731 | 0 | 0.466 |
| ENSP00000288071 | 1.60E-05 | -0.0731 | 367 | 0.812 |
| ENSP00000261534 | 9.86E-06 | -0.0731 | 0 | 0.185 |
| ENSP00000295030 | 9.87E-06 | -0.0731 | 0 | 0.161 |
| ENSP00000259469 | 1.25E-05 | -0.0731 | 248 | 0.795 |
| ENSP00000263388 | 3.35E-05 | -0.0731 | 553 | 0.930 |
| ENSP00000352401 | 2.19E-05 | -0.0731 | 267 | 0.572 |
| ENSP00000302843 | 1.60E-05 | -0.0731 | 876 | 0.289 |
| ENSP00000274170 | 8.53E-06 | -0.0731 | 0 | 0.118 |
| ENSP00000309431 | 1.51E-05 | -0.0731 | 402 | 0.000 |
| ENSP00000234111 | 2.15E-05 | -0.0731 | 320 | 0.581 |
| ENSP00000271277 | 9.14E-06 | -0.0731 | 0 | 0.138 |
| ENSP00000284073 | 1.90E-05 | -0.0731 | 335 | 0.697 |

| | | | | |
|---|---|---|---|---|
| ENSP00000317141 | 1.30E-05 | -0.0731 | 0 | 0.000 |
| ENSP00000056233 | 1.02E-05 | -0.0731 | 0 | 0.725 |
| ENSP00000225576 | 9.43E-06 | -0.0731 | 0 | 0.091 |
| ENSP00000261800 | 2.17E-05 | -0.0732 | 151 | 0.718 |
| ENSP00000326491 | 9.17E-06 | -0.0732 | 0 | 0.087 |
| ENSP00000230895 | 1.32E-05 | -0.0732 | 0 | 0.724 |
| ENSP00000339393 | 2.43E-05 | -0.0732 | 333 | 0.533 |
| ENSP00000403936 | 2.81E-05 | -0.0732 | 460 | 0.000 |
| ENSP00000265433 | 8.29E-06 | -0.0732 | 0 | 0.672 |
| ENSP00000301012 | 1.16E-05 | -0.0732 | 0 | 0.439 |
| ENSP00000233623 | 1.27E-05 | -0.0732 | 671 | 0.301 |
| ENSP00000320447 | 9.94E-06 | -0.0732 | 0 | 0.815 |
| ENSP00000242375 | 1.51E-05 | -0.0732 | 243 | 0.221 |
| ENSP00000300574 | 2.56E-05 | -0.0732 | 908 | 0.872 |
| ENSP00000229758 | 8.71E-06 | -0.0732 | 0 | 0.436 |
| ENSP00000333920 | 1.98E-05 | -0.0732 | 250 | 0.837 |
| ENSP00000312318 | 9.75E-06 | -0.0732 | 0 | 0.153 |
| ENSP00000244217 | 7.84E-06 | -0.0732 | 0 | 0.155 |
| ENSP00000340698 | 3.10E-05 | -0.0732 | 208 | 0.652 |
| ENSP00000251527 | 9.48E-06 | -0.0733 | 0 | 0.109 |
| ENSP00000257287 | 9.06E-06 | -0.0733 | 0 | 0.070 |
| ENSP00000226840 | 7.33E-06 | -0.0733 | 0 | 0.091 |
| ENSP00000263245 | 3.65E-05 | -0.0733 | 203 | 0.206 |
| ENSP00000261884 | 8.06E-06 | -0.0733 | 0 | 0.405 |
| ENSP00000219313 | 1.42E-05 | -0.0733 | 208 | 0.555 |
| ENSP00000199320 | 1.17E-05 | -0.0733 | 388 | 0.843 |
| ENSP00000356480 | 4.24E-05 | -0.0733 | 321 | 0.829 |
| ENSP00000306968 | 7.56E-06 | -0.0733 | 0 | 0.523 |
| ENSP00000322845 | 7.62E-06 | -0.0733 | 0 | 0.746 |
| ENSP00000276449 | 3.86E-05 | -0.0733 | 263 | 0.774 |
| ENSP00000312311 | 6.60E-06 | -0.0733 | 0 | 0.568 |
| ENSP00000261247 | 1.05E-05 | -0.0733 | 0 | 0.100 |
| ENSP00000219837 | 1.68E-05 | -0.0733 | 0 | 0.154 |
| ENSP00000225519 | 1.17E-05 | -0.0733 | 305 | 0.301 |
| ENSP00000251993 | 7.83E-06 | -0.0733 | 0 | 0.084 |
| ENSP00000203664 | 1.01E-05 | -0.0733 | 0 | 0.105 |
| ENSP00000334223 | 7.44E-06 | -0.0733 | 0 | 0.079 |
| ENSP00000263697 | 1.03E-05 | -0.0733 | 0 | 0.796 |
| ENSP00000261681 | 1.38E-05 | -0.0733 | 302 | 0.471 |
| ENSP00000264717 | 1.31E-05 | -0.0733 | 198 | 0.475 |
| ENSP00000306920 | 2.48E-05 | -0.0733 | 242 | 0.198 |
| ENSP00000326603 | 7.07E-06 | -0.0733 | 0 | 0.204 |
| ENSP00000284995 | 9.35E-06 | -0.0733 | 0 | 0.239 |
| ENSP00000321445 | 2.43E-05 | -0.0733 | 311 | 0.789 |
| ENSP00000394248 | 3.02E-06 | -0.0733 | 0 | 0.697 |
| ENSP00000395629 | 3.02E-06 | -0.0733 | 0 | 0.699 |

| | | | | |
|---|---|---|---|---|
| ENSP00000394047 | 3.02E-06 | -0.0733 | 0 | 0.000 |
| ENSP00000248244 | 1.07E-05 | -0.0734 | 0 | 0.713 |
| ENSP00000264198 | 1.08E-05 | -0.0734 | 0 | 0.176 |
| ENSP00000359978 | 1.14E-05 | -0.0734 | 0 | 0.171 |
| ENSP00000254457 | 1.11E-05 | -0.0734 | 250 | 0.000 |
| ENSP00000216373 | 1.46E-05 | -0.0734 | 242 | 0.711 |
| ENSP00000314343 | 8.50E-06 | -0.0734 | 0 | 0.666 |
| ENSP00000295321 | 8.47E-06 | -0.0734 | 0 | 0.794 |
| ENSP00000304185 | 8.04E-06 | -0.0734 | 0 | 0.131 |
| ENSP00000321348 | 1.41E-05 | -0.0734 | 202 | 0.434 |
| ENSP00000251312 | 7.82E-06 | -0.0734 | 0 | 0.000 |
| ENSP00000372335 | 1.29E-05 | -0.0734 | 186 | 0.644 |
| ENSP00000264716 | 1.59E-05 | -0.0734 | 458 | 0.000 |
| ENSP00000246024 | 1.41E-05 | -0.0734 | 0 | 0.100 |
| ENSP00000308948 | 7.00E-06 | -0.0734 | 0 | 0.192 |
| ENSP00000364766 | 1.57E-06 | -0.0734 | 0 | 0.199 |
| ENSP00000014930 | 1.02E-05 | -0.0734 | 0 | 0.459 |
| ENSP00000002125 | 8.48E-06 | -0.0734 | 0 | 0.092 |
| ENSP00000249749 | 1.16E-04 | -0.0734 | 860 | 0.933 |
| ENSP00000269397 | 8.31E-06 | -0.0734 | 0 | 0.788 |
| ENSP00000336127 | 7.76E-06 | -0.0735 | 0 | 0.102 |
| ENSP00000318635 | 1.65E-05 | -0.0735 | 281 | 0.865 |
| ENSP00000252542 | 8.99E-06 | -0.0735 | 0 | 0.734 |
| ENSP00000330721 | 9.27E-06 | -0.0735 | 199 | 0.195 |
| ENSP00000255608 | 8.77E-06 | -0.0735 | 0 | 0.215 |
| ENSP00000290130 | 8.80E-06 | -0.0735 | 150 | 0.307 |
| ENSP00000337998 | 1.09E-04 | -0.0735 | 0 | 0.133 |
| ENSP00000216121 | 8.35E-06 | -0.0735 | 0 | 0.094 |
| ENSP00000312778 | 5.51E-06 | -0.0735 | 0 | 0.268 |
| ENSP00000265421 | 7.40E-06 | -0.0735 | 0 | 0.449 |
| ENSP00000340463 | 9.09E-06 | -0.0735 | 0 | 0.093 |
| ENSP00000329002 | 1.19E-05 | -0.0735 | 670 | 0.531 |
| ENSP00000228843 | 7.64E-06 | -0.0735 | 0 | 0.370 |
| ENSP00000222388 | 1.17E-05 | -0.0735 | 157 | 0.665 |
| ENSP00000396937 | 4.00E-05 | -0.0735 | 153 | 0.367 |
| ENSP00000320413 | 9.83E-06 | -0.0735 | 150 | 0.164 |
| ENSP00000319255 | 1.20E-05 | -0.0735 | 0 | 0.105 |
| ENSP00000300291 | 1.31E-05 | -0.0736 | 644 | 0.835 |
| ENSP00000264658 | 9.56E-06 | -0.0736 | 227 | 0.322 |
| ENSP00000327704 | 2.95E-05 | -0.0736 | 167 | 0.366 |
| ENSP00000226432 | 1.02E-05 | -0.0736 | 0 | 0.114 |
| ENSP00000294258 | 6.08E-06 | -0.0736 | 0 | 0.241 |
| ENSP00000331103 | 8.76E-06 | -0.0736 | 0 | 0.405 |
| ENSP00000299134 | 8.03E-06 | -0.0736 | 0 | 0.000 |
| ENSP00000270583 | 6.75E-06 | -0.0736 | 0 | 0.718 |
| ENSP00000289528 | 1.37E-05 | -0.0736 | 215 | 0.412 |

| | | | | |
|---|---|---|---|---|
| ENSP00000329867 | 1.21E-05 | -0.0736 | 159 | 0.102 |
| ENSP00000315615 | 1.36E-05 | -0.0736 | 0 | 0.425 |
| ENSP00000335615 | 7.19E-06 | -0.0736 | 0 | 0.089 |
| ENSP00000342481 | 4.85E-05 | -0.0736 | 174 | 0.063 |
| ENSP00000290101 | 2.56E-05 | -0.0737 | 185 | 0.742 |
| ENSP00000312395 | 1.15E-05 | -0.0737 | 0 | 0.000 |
| ENSP00000250263 | 8.12E-06 | -0.0737 | 0 | 0.642 |
| ENSP00000235345 | 6.98E-06 | -0.0737 | 0 | 0.031 |
| ENSP00000304689 | 1.22E-05 | -0.0737 | 0 | 0.470 |
| ENSP00000389841 | 3.04E-06 | -0.0737 | 0 | 0.694 |
| ENSP00000378394 | 2.98E-05 | -0.0737 | 284 | 0.345 |
| ENSP00000288937 | 1.18E-05 | -0.0737 | 309 | 0.703 |
| ENSP00000333326 | 2.22E-05 | -0.0737 | 234 | 0.486 |
| ENSP00000357643 | 1.99E-05 | -0.0737 | 249 | 0.723 |
| ENSP00000264670 | 1.31E-05 | -0.0737 | 178 | 0.858 |
| ENSP00000339881 | 8.32E-06 | -0.0737 | 0 | 0.083 |
| ENSP00000341805 | 6.71E-06 | -0.0737 | 0 | 0.704 |
| ENSP00000308583 | 6.54E-06 | -0.0737 | 0 | 0.514 |
| ENSP00000382697 | 5.79E-05 | -0.0737 | 610 | 0.892 |
| ENSP00000298229 | 2.19E-05 | -0.0737 | 471 | 0.591 |
| ENSP00000334382 | 8.29E-06 | -0.0737 | 0 | 0.680 |
| ENSP00000331087 | 4.95E-05 | -0.0737 | 0 | 0.229 |
| ENSP00000305230 | 2.44E-05 | -0.0737 | 168 | 0.625 |
| ENSP00000315112 | 9.91E-06 | -0.0737 | 0 | 0.313 |
| ENSP00000246166 | 6.74E-06 | -0.0737 | 0 | 0.158 |
| ENSP00000305810 | 7.68E-06 | -0.0738 | 0 | 0.210 |
| ENSP00000359382 | 6.05E-06 | -0.0738 | 0 | 0.127 |
| ENSP00000340796 | 8.75E-06 | -0.0738 | 0 | 0.445 |
| ENSP00000380480 | 3.07E-06 | -0.0738 | 0 | 0.000 |
| ENSP00000253099 | 1.30E-05 | -0.0738 | 364 | 0.811 |
| ENSP00000329411 | 1.55E-05 | -0.0738 | 912 | 0.840 |
| ENSP00000323065 | 8.15E-06 | -0.0738 | 0 | 0.336 |
| ENSP00000306565 | 1.83E-05 | -0.0738 | 226 | 0.731 |
| ENSP00000254695 | 7.38E-06 | -0.0738 | 0 | 0.070 |
| ENSP00000305595 | 1.04E-05 | -0.0738 | 900 | 0.161 |
| ENSP00000260665 | 1.91E-05 | -0.0738 | 172 | 0.413 |
| ENSP00000228495 | 7.81E-06 | -0.0738 | 0 | 0.073 |
| ENSP00000247977 | 1.43E-05 | -0.0738 | 0 | 0.109 |
| ENSP00000225688 | 1.23E-05 | -0.0738 | 478 | 0.609 |
| ENSP00000243776 | 8.14E-06 | -0.0738 | 0 | 0.071 |
| ENSP00000288986 | 2.32E-05 | -0.0738 | 650 | 0.845 |
| ENSP00000321732 | 6.60E-06 | -0.0738 | 0 | 0.387 |
| ENSP00000234313 | 3.31E-05 | -0.0738 | 407 | 0.868 |
| ENSP00000312402 | 1.53E-05 | -0.0738 | 0 | 0.185 |
| ENSP00000313854 | 8.69E-06 | -0.0739 | 0 | 0.344 |
| ENSP00000307143 | 9.18E-06 | -0.0739 | 0 | 0.341 |

| | | | | |
|---|---|---|---|---|
| ENSP00000238651 | 9.07E-06 | -0.0739 | 0 | 0.365 |
| ENSP00000265354 | 2.69E-05 | -0.0739 | 993 | 0.000 |
| ENSP00000254508 | 9.09E-06 | -0.0739 | 0 | 0.553 |
| ENSP00000356789 | 1.11E-04 | -0.0739 | 499 | 0.257 |
| ENSP00000379458 | 3.08E-06 | -0.0739 | 0 | 0.697 |
| ENSP00000215980 | 6.02E-06 | -0.0739 | 0 | 0.221 |
| ENSP00000410758 | 4.24E-05 | -0.0739 | 644 | 0.718 |
| ENSP00000314444 | 8.10E-06 | -0.0739 | 0 | 0.092 |
| ENSP00000303997 | 9.68E-06 | -0.0739 | 0 | 0.119 |
| ENSP00000347684 | 1.54E-05 | -0.0739 | 227 | 0.842 |
| ENSP00000266087 | 5.65E-06 | -0.0739 | 0 | 0.276 |
| ENSP00000274963 | 8.48E-06 | -0.0739 | 0 | 0.153 |
| ENSP00000414359 | 3.53E-05 | -0.0739 | 264 | 0.565 |
| ENSP00000166345 | 1.05E-05 | -0.0740 | 272 | 0.542 |
| ENSP00000357323 | 8.56E-06 | -0.0740 | 0 | 0.318 |
| ENSP00000281038 | 6.78E-06 | -0.0740 | 0 | 0.455 |
| ENSP00000252245 | 9.99E-06 | -0.0740 | 0 | 0.183 |
| ENSP00000335557 | 1.01E-05 | -0.0740 | 0 | 0.196 |
| ENSP00000359403 | 1.33E-06 | -0.0740 | 0 | 0.211 |
| ENSP00000256578 | 7.69E-06 | -0.0740 | 0 | 0.192 |
| ENSP00000254803 | 1.46E-05 | -0.0740 | 659 | 0.855 |
| ENSP00000159111 | 8.61E-06 | -0.0740 | 0 | 0.841 |
| ENSP00000338352 | 1.92E-05 | -0.0740 | 0 | 0.067 |
| ENSP00000215812 | 9.34E-06 | -0.0740 | 0 | 0.483 |
| ENSP00000405268 | 9.90E-06 | -0.0740 | 0 | 0.567 |
| ENSP00000203630 | 7.91E-06 | -0.0740 | 0 | 0.233 |
| ENSP00000268261 | 1.48E-05 | -0.0740 | 200 | 0.344 |
| ENSP00000262850 | 8.15E-06 | -0.0740 | 0 | 0.457 |
| ENSP00000262394 | 8.69E-06 | -0.0740 | 213 | 0.347 |
| ENSP00000383096 | 2.63E-05 | -0.0741 | 175 | 0.355 |
| ENSP00000006101 | 8.31E-06 | -0.0741 | 0 | 0.000 |
| ENSP00000295400 | 7.79E-05 | -0.0741 | 355 | 0.949 |
| ENSP00000232461 | 1.65E-05 | -0.0741 | 999 | 0.644 |
| ENSP00000309052 | 7.78E-06 | -0.0741 | 0 | 0.149 |
| ENSP00000332604 | 1.55E-05 | -0.0741 | 355 | 0.912 |
| ENSP00000217446 | 7.39E-06 | -0.0741 | 0 | 0.049 |
| ENSP00000418287 | 9.87E-06 | -0.0741 | 0 | 0.620 |
| ENSP00000261721 | 8.46E-06 | -0.0741 | 0 | 0.139 |
| ENSP00000223114 | 1.23E-05 | -0.0741 | 0 | 0.222 |
| ENSP00000247665 | 7.72E-06 | -0.0741 | 0 | 0.135 |
| ENSP00000391372 | 1.67E-05 | -0.0741 | 335 | 0.585 |
| ENSP00000262043 | 8.53E-06 | -0.0741 | 0 | 0.123 |
| ENSP00000311127 | 2.08E-04 | -0.0741 | 920 | 0.817 |
| ENSP00000271227 | 1.10E-05 | -0.0741 | 0 | 0.162 |
| ENSP00000252252 | 8.95E-06 | -0.0741 | 0 | 0.175 |
| ENSP00000309830 | 1.75E-05 | -0.0741 | 210 | 0.823 |

| | | | | |
|---|---|---|---|---|
| ENSP00000225665 | 7.09E-06 | -0.0741 | 0 | 0.167 |
| ENSP00000230321 | 6.46E-06 | -0.0741 | 0 | 0.216 |
| ENSP00000262127 | 8.15E-06 | -0.0741 | 0 | 0.083 |
| ENSP00000342136 | 1.49E-05 | -0.0741 | 215 | 0.557 |
| ENSP00000311876 | 1.87E-05 | -0.0741 | 261 | 0.318 |
| ENSP00000317332 | 1.03E-05 | -0.0741 | 0 | 0.097 |
| ENSP00000309772 | 1.03E-05 | -0.0742 | 0 | 0.078 |
| ENSP00000343412 | 2.03E-05 | -0.0742 | 265 | 0.705 |
| ENSP00000330408 | 9.89E-06 | -0.0742 | 644 | 0.000 |
| ENSP00000319343 | 6.28E-06 | -0.0742 | 0 | 0.379 |
| ENSP00000339495 | 7.50E-06 | -0.0742 | 0 | 0.734 |
| ENSP00000264638 | 8.12E-06 | -0.0742 | 0 | 0.546 |
| ENSP00000293695 | 2.55E-05 | -0.0742 | 156 | 0.215 |
| ENSP00000316598 | 1.59E-05 | -0.0743 | 201 | 0.240 |
| ENSP00000266503 | 4.89E-05 | -0.0743 | 165 | 0.557 |
| ENSP00000268035 | 3.08E-05 | -0.0743 | 918 | 0.000 |
| ENSP00000217372 | 1.23E-05 | -0.0743 | 359 | 0.530 |
| ENSP00000272928 | 1.36E-05 | -0.0743 | 0 | 0.000 |
| ENSP00000283426 | 8.39E-06 | -0.0743 | 0 | 0.293 |
| ENSP00000322628 | 2.70E-05 | -0.0743 | 274 | 0.513 |
| ENSP00000297564 | 6.38E-06 | -0.0743 | 0 | 0.276 |
| ENSP00000361185 | 5.76E-06 | -0.0743 | 0 | 0.662 |
| ENSP00000324302 | 1.18E-05 | -0.0743 | 0 | 0.142 |
| ENSP00000392024 | 2.99E-06 | -0.0743 | 0 | 0.692 |
| ENSP00000372276 | 2.16E-07 | -0.0743 | 0 | 0.203 |
| ENSP00000267113 | 1.00E-05 | -0.0743 | 0 | 0.186 |
| ENSP00000231524 | 1.45E-05 | -0.0743 | 274 | 0.180 |
| ENSP00000230048 | 6.90E-06 | -0.0743 | 0 | 0.120 |
| ENSP00000295767 | 7.34E-06 | -0.0743 | 0 | 0.092 |
| ENSP00000264122 | 1.04E-05 | -0.0744 | 0 | 0.681 |
| ENSP00000255499 | 9.20E-05 | -0.0744 | 270 | 0.854 |
| ENSP00000234488 | 7.24E-06 | -0.0744 | 0 | 0.057 |
| ENSP00000373254 | 3.12E-06 | -0.0744 | 0 | 0.697 |
| ENSP00000295156 | 1.83E-05 | -0.0744 | 341 | 0.325 |
| ENSP00000261249 | 6.82E-06 | -0.0744 | 0 | 0.622 |
| ENSP00000295065 | 7.16E-06 | -0.0744 | 0 | 0.114 |
| ENSP00000330572 | 1.31E-05 | -0.0744 | 0 | 0.459 |
| ENSP00000307684 | 2.71E-05 | -0.0745 | 216 | 0.869 |
| ENSP00000339122 | 7.48E-06 | -0.0745 | 0 | 0.092 |
| ENSP00000267522 | 6.35E-06 | -0.0745 | 0 | 0.445 |
| ENSP00000289968 | 1.37E-05 | -0.0745 | 0 | 0.539 |
| ENSP00000310561 | 1.05E-05 | -0.0745 | 0 | 0.511 |
| ENSP00000341489 | 7.00E-06 | -0.0745 | 0 | 0.086 |
| ENSP00000378195 | 5.63E-05 | -0.0745 | 330 | 0.205 |
| ENSP00000313171 | 7.37E-06 | -0.0745 | 0 | 0.714 |
| ENSP00000311224 | 1.03E-05 | -0.0745 | 150 | 0.409 |

| | | | | |
|---|---|---|---|---|
| ENSP00000262940 | 7.00E-06 | -0.0745 | 0 | 0.393 |
| ENSP00000356191 | 1.28E-05 | -0.0745 | 203 | 0.703 |
| ENSP00000268624 | 1.31E-05 | -0.0745 | 0 | 0.194 |
| ENSP00000367157 | 4.57E-06 | -0.0746 | 0 | 0.706 |
| ENSP00000317382 | 7.71E-06 | -0.0746 | 0 | 0.168 |
| ENSP00000273853 | 7.13E-06 | -0.0746 | 0 | 0.000 |
| ENSP00000341551 | 5.72E-05 | -0.0746 | 943 | 0.912 |
| ENSP00000261674 | 9.36E-06 | -0.0746 | 0 | 0.232 |
| ENSP00000179259 | 7.54E-06 | -0.0746 | 0 | 0.000 |
| ENSP00000257575 | 1.40E-05 | -0.0746 | 236 | 0.124 |
| ENSP00000262888 | 9.52E-06 | -0.0746 | 223 | 0.425 |
| ENSP00000301633 | 4.09E-05 | -0.0746 | 582 | 0.886 |
| ENSP00000372959 | 9.19E-06 | -0.0746 | 487 | 0.658 |
| ENSP00000373340 | 2.49E-05 | -0.0746 | 613 | 0.866 |
| ENSP00000257895 | 1.01E-04 | -0.0746 | 992 | 0.431 |
| ENSP00000244711 | 8.55E-06 | -0.0746 | 0 | 0.324 |
| ENSP00000334801 | 8.37E-06 | -0.0746 | 0 | 0.239 |
| ENSP00000335628 | 5.88E-06 | -0.0746 | 0 | 0.076 |
| ENSP00000351137 | 2.12E-05 | -0.0746 | 580 | 0.757 |
| ENSP00000395733 | 3.18E-06 | -0.0746 | 0 | 0.695 |
| ENSP00000273067 | 2.30E-05 | -0.0747 | 205 | 0.205 |
| ENSP00000343126 | 7.00E-06 | -0.0747 | 0 | 0.832 |
| ENSP00000293373 | 7.97E-06 | -0.0747 | 0 | 0.186 |
| ENSP00000306522 | 6.88E-06 | -0.0747 | 0 | 0.215 |
| ENSP00000266214 | 7.27E-06 | -0.0747 | 0 | 0.000 |
| ENSP00000332646 | 8.20E-06 | -0.0747 | 0 | 0.062 |
| ENSP00000228837 | 8.42E-05 | -0.0747 | 257 | 0.920 |
| ENSP00000265036 | 9.00E-06 | -0.0747 | 0 | 0.104 |
| ENSP00000296543 | 8.38E-06 | -0.0747 | 0 | 0.427 |
| ENSP00000298746 | 1.93E-05 | -0.0747 | 443 | 0.915 |
| ENSP00000219596 | 1.97E-05 | -0.0747 | 203 | 0.361 |
| ENSP00000337688 | 9.19E-06 | -0.0747 | 423 | 0.183 |
| ENSP00000408695 | 5.55E-05 | -0.0747 | 908 | 0.665 |
| ENSP00000260116 | 1.04E-05 | -0.0747 | 270 | 0.422 |
| ENSP00000353698 | 6.39E-06 | -0.0748 | 0 | 0.231 |
| ENSP00000299853 | 8.89E-06 | -0.0748 | 0 | 0.553 |
| ENSP00000262460 | 1.47E-05 | -0.0748 | 186 | 0.347 |
| ENSP00000310620 | 1.16E-05 | -0.0748 | 0 | 0.086 |
| ENSP00000320184 | 6.07E-06 | -0.0748 | 0 | 0.339 |
| ENSP00000310873 | 6.55E-06 | -0.0748 | 0 | 0.090 |
| ENSP00000361666 | 2.56E-05 | -0.0748 | 160 | 0.095 |
| ENSP00000231498 | 7.57E-06 | -0.0748 | 0 | 0.553 |
| ENSP00000262134 | 1.05E-05 | -0.0748 | 236 | 0.191 |
| ENSP00000318821 | 6.14E-06 | -0.0748 | 0 | 0.090 |
| ENSP00000267163 | 3.42E-05 | -0.0748 | 288 | 0.827 |
| ENSP00000261475 | 7.55E-06 | -0.0748 | 0 | 0.155 |

| | | | | |
|---|---|---|---|---|
| ENSP00000301738 | 6.51E-06 | -0.0748 | 0 | 0.083 |
| ENSP00000231238 | 7.85E-06 | -0.0748 | 0 | 0.247 |
| ENSP00000255390 | 8.17E-06 | -0.0748 | 0 | 0.297 |
| ENSP00000234827 | 1.68E-05 | -0.0748 | 168 | 0.875 |
| ENSP00000269601 | 7.78E-06 | -0.0748 | 0 | 0.829 |
| ENSP00000281273 | 5.91E-06 | -0.0749 | 0 | 0.538 |
| ENSP00000281172 | 2.11E-05 | -0.0749 | 212 | 0.599 |
| ENSP00000364073 | 6.44E-05 | -0.0749 | 650 | 0.000 |
| ENSP00000264230 | 1.43E-05 | -0.0749 | 240 | 0.847 |
| ENSP00000354676 | 3.56E-05 | -0.0749 | 179 | 0.059 |
| ENSP00000331901 | 2.59E-05 | -0.0749 | 500 | 0.780 |
| ENSP00000266971 | 6.41E-06 | -0.0749 | 0 | 0.177 |
| ENSP00000330918 | 2.06E-05 | -0.0749 | 197 | 0.258 |
| ENSP00000243903 | 8.07E-06 | -0.0749 | 0 | 0.451 |
| ENSP00000278379 | 5.36E-05 | -0.0749 | 512 | 0.414 |
| ENSP00000269586 | 2.34E-05 | -0.0749 | 274 | 0.494 |
| ENSP00000311245 | 7.30E-06 | -0.0749 | 0 | 0.117 |
| ENSP00000294119 | 3.54E-05 | -0.0749 | 424 | 0.165 |
| ENSP00000279804 | 1.15E-04 | -0.0749 | 309 | 0.831 |
| ENSP00000245838 | 1.29E-05 | -0.0749 | 869 | 0.794 |
| ENSP00000216962 | 1.19E-05 | -0.0749 | 158 | 0.355 |
| ENSP00000289352 | 1.38E-05 | -0.0749 | 882 | 0.000 |
| ENSP00000262419 | 7.48E-06 | -0.0750 | 0 | 0.401 |
| ENSP00000368502 | 3.79E-06 | -0.0750 | 0 | 0.698 |
| ENSP00000322977 | 7.80E-06 | -0.0750 | 0 | 0.612 |
| ENSP00000291281 | 7.41E-06 | -0.0750 | 0 | 0.205 |
| ENSP00000344546 | 7.22E-06 | -0.0750 | 0 | 0.756 |
| ENSP00000265224 | 1.33E-05 | -0.0750 | 202 | 0.670 |
| ENSP00000282841 | 2.76E-05 | -0.0750 | 150 | 0.305 |
| ENSP00000331827 | 9.66E-06 | -0.0750 | 0 | 0.151 |
| ENSP00000295269 | 1.29E-05 | -0.0750 | 197 | 0.246 |
| ENSP00000244766 | 5.35E-05 | -0.0750 | 282 | 0.334 |
| ENSP00000341044 | 4.90E-05 | -0.0750 | 436 | 0.708 |
| ENSP00000360025 | 5.54E-05 | -0.0751 | 860 | 0.857 |
| ENSP00000264279 | 1.44E-05 | -0.0751 | 243 | 0.000 |
| ENSP00000296473 | 1.01E-05 | -0.0751 | 192 | 0.085 |
| ENSP00000251337 | 1.77E-05 | -0.0751 | 986 | 0.648 |
| ENSP00000253462 | 5.24E-06 | -0.0751 | 0 | 0.443 |
| ENSP00000216194 | 1.75E-05 | -0.0751 | 216 | 0.446 |
| ENSP00000243662 | 7.32E-06 | -0.0751 | 0 | 0.146 |
| ENSP00000303058 | 8.35E-06 | -0.0751 | 0 | 0.053 |
| ENSP00000303211 | 3.98E-05 | -0.0751 | 562 | 0.714 |
| ENSP00000319622 | 8.51E-06 | -0.0751 | 0 | 0.093 |
| ENSP00000216038 | 9.31E-06 | -0.0751 | 0 | 0.000 |
| ENSP00000250066 | 1.06E-05 | -0.0752 | 808 | 0.270 |
| ENSP00000364555 | 3.70E-05 | -0.0752 | 449 | 0.699 |

| | | | | |
|---|---|---|---|---|
| ENSP00000262027 | 1.28E-05 | -0.0752 | 449 | 0.786 |
| ENSP00000318016 | 7.36E-06 | -0.0752 | 0 | 0.119 |
| ENSP00000306138 | 2.26E-05 | -0.0752 | 484 | 0.740 |
| ENSP00000262887 | 1.86E-05 | -0.0752 | 153 | 0.714 |
| ENSP00000253115 | 7.02E-05 | -0.0752 | 158 | 0.699 |
| ENSP00000229238 | 4.96E-06 | -0.0752 | 0 | 0.257 |
| ENSP00000357799 | 1.11E-04 | -0.0752 | 225 | 0.597 |
| ENSP00000255108 | 5.33E-06 | -0.0752 | 0 | 0.616 |
| ENSP00000355396 | 5.76E-06 | -0.0752 | 0 | 0.590 |
| ENSP00000262953 | 1.87E-05 | -0.0752 | 202 | 0.820 |
| ENSP00000281141 | 1.48E-05 | -0.0752 | 0 | 0.145 |
| ENSP00000216200 | 1.13E-05 | -0.0752 | 330 | 0.650 |
| ENSP00000305958 | 1.40E-05 | -0.0752 | 866 | 0.583 |
| ENSP00000393631 | 1.10E-05 | -0.0752 | 0 | 0.098 |
| ENSP00000271915 | 9.19E-06 | -0.0752 | 186 | 0.421 |
| ENSP00000217169 | 1.16E-05 | -0.0752 | 0 | 0.514 |
| ENSP00000262570 | 5.11E-06 | -0.0753 | 0 | 0.103 |
| ENSP00000328036 | 8.41E-06 | -0.0753 | 0 | 0.164 |
| ENSP00000299441 | 1.40E-05 | -0.0753 | 151 | 0.747 |
| ENSP00000251472 | 7.82E-06 | -0.0753 | 0 | 0.526 |
| ENSP00000305664 | 6.58E-06 | -0.0753 | 0 | 0.103 |
| ENSP00000351608 | 9.28E-06 | -0.0753 | 0 | 0.096 |
| ENSP00000219066 | 1.04E-05 | -0.0753 | 221 | 0.623 |
| ENSP00000333666 | 1.04E-05 | -0.0753 | 0 | 0.095 |
| ENSP00000293894 | 8.05E-06 | -0.0753 | 0 | 0.810 |
| ENSP00000344659 | 1.10E-05 | -0.0753 | 0 | 0.073 |
| ENSP00000221476 | 8.79E-05 | -0.0753 | 373 | 0.567 |
| ENSP00000331902 | 6.23E-05 | -0.0753 | 705 | 0.843 |
| ENSP00000312356 | 5.91E-06 | -0.0753 | 0 | 0.797 |
| ENSP00000320679 | 2.59E-05 | -0.0753 | 377 | 0.271 |
| ENSP00000355623 | 1.78E-04 | -0.0753 | 158 | 0.246 |
| ENSP00000344352 | 4.86E-05 | -0.0754 | 555 | 0.000 |
| ENSP00000312981 | 7.82E-06 | -0.0754 | 0 | 0.734 |
| ENSP00000360905 | 1.34E-05 | -0.0754 | 0 | 0.090 |
| ENSP00000317257 | 6.01E-06 | -0.0754 | 0 | 0.119 |
| ENSP00000264758 | 2.96E-05 | -0.0754 | 725 | 0.217 |
| ENSP00000251074 | 6.85E-06 | -0.0754 | 0 | 0.412 |
| ENSP00000238788 | 7.03E-06 | -0.0754 | 0 | 0.069 |
| ENSP00000287675 | 8.62E-06 | -0.0755 | 0 | 0.230 |
| ENSP00000306356 | 1.11E-05 | -0.0755 | 0 | 0.383 |
| ENSP00000274137 | 1.18E-05 | -0.0755 | 158 | 0.282 |
| ENSP00000472412 | 6.02E-07 | -0.0755 | 0 | 0.000 |
| ENSP00000299626 | 7.06E-06 | -0.0755 | 0 | 0.159 |
| ENSP00000247815 | 6.93E-06 | -0.0755 | 0 | 0.625 |
| ENSP00000323050 | 1.51E-05 | -0.0755 | 181 | 0.783 |
| ENSP00000399075 | 9.67E-07 | -0.0755 | 0 | 0.202 |

| | | | | |
|---|---|---|---|---|
| ENSP00000306844 | 7.20E-06 | -0.0755 | 0 | 0.394 |
| ENSP00000313851 | 5.68E-06 | -0.0755 | 0 | 0.508 |
| ENSP00000257118 | 6.51E-06 | -0.0755 | 0 | 0.618 |
| ENSP00000263798 | 1.79E-05 | -0.0755 | 467 | 0.678 |
| ENSP00000306473 | 5.80E-06 | -0.0755 | 0 | 0.262 |
| ENSP00000259037 | 5.85E-06 | -0.0755 | 0 | 0.212 |
| ENSP00000278618 | 8.93E-06 | -0.0755 | 0 | 0.514 |
| ENSP00000368138 | 8.40E-06 | -0.0755 | 0 | 0.216 |
| ENSP00000285968 | 6.89E-06 | -0.0755 | 0 | 0.569 |
| ENSP00000263657 | 1.64E-05 | -0.0755 | 195 | 0.916 |
| ENSP00000330460 | 1.58E-05 | -0.0756 | 436 | 0.930 |
| ENSP00000306999 | 1.49E-05 | -0.0756 | 183 | 0.684 |
| ENSP00000378426 | 3.54E-04 | -0.0756 | 328 | 0.000 |
| ENSP00000200652 | 3.09E-05 | -0.0756 | 360 | 0.515 |
| ENSP00000304845 | 2.79E-05 | -0.0756 | 245 | 0.272 |
| ENSP00000216410 | 1.27E-05 | -0.0756 | 160 | 0.155 |
| ENSP00000263035 | 1.20E-05 | -0.0756 | 224 | 0.438 |
| ENSP00000384000 | 3.26E-06 | -0.0756 | 0 | 0.695 |
| ENSP00000293897 | 1.30E-05 | -0.0756 | 270 | 0.723 |
| ENSP00000252711 | 6.07E-06 | -0.0756 | 0 | 0.233 |
| ENSP00000215730 | 7.41E-06 | -0.0756 | 0 | 0.248 |
| ENSP00000276893 | 7.08E-06 | -0.0756 | 0 | 0.763 |
| ENSP00000337103 | 4.35E-05 | -0.0756 | 472 | 0.757 |
| ENSP00000277575 | 1.47E-05 | -0.0756 | 223 | 0.384 |
| ENSP00000216366 | 6.91E-06 | -0.0756 | 0 | 0.121 |
| ENSP00000339389 | 6.79E-06 | -0.0757 | 0 | 0.280 |
| ENSP00000318861 | 1.91E-05 | -0.0757 | 551 | 0.872 |
| ENSP00000266771 | 8.76E-06 | -0.0757 | 0 | 0.092 |
| ENSP00000292476 | 1.78E-05 | -0.0757 | 300 | 0.430 |
| ENSP00000304891 | 5.76E-06 | -0.0757 | 0 | 0.078 |
| ENSP00000253233 | 1.37E-05 | -0.0757 | 290 | 0.562 |
| ENSP00000344106 | 9.47E-06 | -0.0757 | 168 | 0.096 |
| ENSP00000242872 | 6.67E-06 | -0.0757 | 0 | 0.318 |
| ENSP00000357301 | 1.31E-05 | -0.0757 | 563 | 0.486 |
| ENSP00000274498 | 7.53E-06 | -0.0757 | 0 | 0.573 |
| ENSP00000158771 | 1.59E-05 | -0.0757 | 801 | 0.512 |
| ENSP00000305653 | 7.43E-06 | -0.0757 | 0 | 0.077 |
| ENSP00000282050 | 2.07E-05 | -0.0757 | 747 | 0.681 |
| ENSP00000262209 | 1.41E-05 | -0.0757 | 205 | 0.615 |
| ENSP00000265537 | 1.53E-05 | -0.0757 | 340 | 0.625 |
| ENSP00000270747 | 5.93E-06 | -0.0757 | 0 | 0.584 |
| ENSP00000300107 | 2.43E-05 | -0.0757 | 359 | 0.516 |
| ENSP00000246535 | 6.71E-06 | -0.0757 | 0 | 0.623 |
| ENSP00000363318 | 2.41E-05 | -0.0758 | 200 | 0.877 |
| ENSP00000369857 | 1.47E-05 | -0.0758 | 177 | 0.659 |
| ENSP00000217109 | 7.18E-06 | -0.0758 | 0 | 0.769 |

| | | | | |
|---|---|---|---|---|
| ENSP00000346414 | 1.30E-05 | -0.0758 | 159 | 0.495 |
| ENSP00000311572 | 9.94E-06 | -0.0758 | 0 | 0.123 |
| ENSP00000261183 | 3.26E-05 | -0.0758 | 427 | 0.160 |
| ENSP00000340138 | 6.45E-06 | -0.0758 | 0 | 0.000 |
| ENSP00000311401 | 9.01E-06 | -0.0758 | 0 | 0.092 |
| ENSP00000253188 | 8.38E-06 | -0.0758 | 0 | 0.077 |
| ENSP00000285949 | 4.41E-05 | -0.0758 | 421 | 0.236 |
| ENSP00000263382 | 1.31E-05 | -0.0758 | 427 | 0.940 |
| ENSP00000319788 | 8.83E-06 | -0.0758 | 0 | 0.378 |
| ENSP00000329093 | 6.26E-06 | -0.0758 | 0 | 0.214 |
| ENSP00000261741 | 1.22E-05 | -0.0758 | 229 | 0.855 |
| ENSP00000337623 | 9.33E-06 | -0.0758 | 0 | 0.543 |
| ENSP00000295926 | 7.05E-06 | -0.0758 | 0 | 0.604 |
| ENSP00000310785 | 7.03E-06 | -0.0758 | 0 | 0.421 |
| ENSP00000302814 | 5.49E-06 | -0.0758 | 0 | 0.759 |
| ENSP00000362304 | 2.58E-05 | -0.0758 | 400 | 0.139 |
| ENSP00000233607 | 8.03E-06 | -0.0758 | 0 | 0.805 |
| ENSP00000311202 | 9.20E-06 | -0.0758 | 0 | 0.137 |
| ENSP00000216500 | 8.45E-06 | -0.0758 | 0 | 0.111 |
| ENSP00000296215 | 7.42E-06 | -0.0758 | 0 | 0.729 |
| ENSP00000338864 | 1.73E-05 | -0.0759 | 349 | 0.724 |
| ENSP00000295225 | 1.23E-05 | -0.0759 | 169 | 0.427 |
| ENSP00000255389 | 9.73E-06 | -0.0759 | 304 | 0.392 |
| ENSP00000320337 | 1.18E-05 | -0.0759 | 0 | 0.141 |
| ENSP00000258399 | 2.17E-05 | -0.0759 | 223 | 0.262 |
| ENSP00000316114 | 7.23E-06 | -0.0759 | 0 | 0.000 |
| ENSP00000366702 | 9.95E-05 | -0.0759 | 394 | 0.390 |
| ENSP00000263205 | 7.36E-06 | -0.0759 | 0 | 0.691 |
| ENSP00000252172 | 1.74E-05 | -0.0759 | 244 | 0.376 |
| ENSP00000322915 | 7.69E-06 | -0.0759 | 0 | 0.633 |
| ENSP00000319231 | 2.68E-05 | -0.0759 | 0 | 0.112 |
| ENSP00000227880 | 8.26E-06 | -0.0759 | 0 | 0.098 |
| ENSP00000306157 | 8.80E-06 | -0.0760 | 0 | 0.804 |
| ENSP00000263897 | 8.50E-06 | -0.0760 | 0 | 0.194 |
| ENSP00000333735 | 7.20E-06 | -0.0760 | 0 | 0.132 |
| ENSP00000354723 | 2.15E-05 | -0.0760 | 819 | 0.959 |
| ENSP00000244221 | 7.15E-06 | -0.0760 | 0 | 0.102 |
| ENSP00000342858 | 9.59E-06 | -0.0760 | 0 | 0.304 |
| ENSP00000040584 | 8.05E-06 | -0.0760 | 0 | 0.815 |
| ENSP00000310623 | 1.71E-05 | -0.0760 | 762 | 0.343 |
| ENSP00000316454 | 1.29E-05 | -0.0760 | 0 | 0.193 |
| ENSP00000341692 | 5.64E-06 | -0.0760 | 0 | 0.703 |
| ENSP00000295930 | 1.30E-05 | -0.0760 | 165 | 0.934 |
| ENSP00000274242 | 1.40E-05 | -0.0760 | 159 | 0.759 |
| ENSP00000268876 | 6.01E-06 | -0.0760 | 0 | 0.191 |
| ENSP00000264773 | 6.98E-06 | -0.0761 | 0 | 0.158 |

| | | | | |
|---|---|---|---|---|
| ENSP00000287022 | 5.33E-06 | -0.0761 | 0 | 0.250 |
| ENSP00000299498 | 1.60E-05 | -0.0761 | 193 | 0.288 |
| ENSP00000286794 | 5.74E-06 | -0.0761 | 0 | 0.411 |
| ENSP00000379464 | 3.10E-06 | -0.0761 | 0 | 0.697 |
| ENSP00000274496 | 6.16E-06 | -0.0761 | 0 | 0.131 |
| ENSP00000230565 | 7.97E-06 | -0.0761 | 0 | 0.063 |
| ENSP00000315775 | 6.15E-06 | -0.0761 | 0 | 0.126 |
| ENSP00000264982 | 2.61E-05 | -0.0761 | 549 | 0.170 |
| ENSP00000215587 | 1.03E-05 | -0.0761 | 250 | 0.817 |
| ENSP00000315064 | 1.91E-05 | -0.0761 | 0 | 0.224 |
| ENSP00000323074 | 1.94E-05 | -0.0761 | 0 | 0.159 |
| ENSP00000264499 | 4.03E-05 | -0.0761 | 455 | 0.235 |
| ENSP00000338562 | 2.65E-05 | -0.0761 | 377 | 0.301 |
| ENSP00000339245 | 8.73E-06 | -0.0761 | 0 | 0.769 |
| ENSP00000385122 | 6.03E-06 | -0.0761 | 0 | 0.112 |
| ENSP00000310722 | 1.69E-05 | -0.0761 | 554 | 0.681 |
| ENSP00000338057 | 5.57E-06 | -0.0762 | 0 | 0.313 |
| ENSP00000402835 | 1.41E-05 | -0.0762 | 428 | 0.000 |
| ENSP00000322524 | 1.34E-05 | -0.0762 | 0 | 0.147 |
| ENSP00000365465 | 6.76E-06 | -0.0762 | 0 | 0.184 |
| ENSP00000229214 | 1.12E-05 | -0.0762 | 367 | 0.920 |
| ENSP00000330694 | 1.93E-05 | -0.0762 | 0 | 0.199 |
| ENSP00000276297 | 8.60E-06 | -0.0762 | 0 | 0.499 |
| ENSP00000295119 | 7.03E-06 | -0.0762 | 0 | 0.512 |
| ENSP00000222402 | 1.67E-05 | -0.0762 | 187 | 0.589 |
| ENSP00000326671 | 6.45E-06 | -0.0762 | 0 | 0.062 |
| ENSP00000262735 | 4.19E-05 | -0.0762 | 943 | 0.890 |
| ENSP00000463817 | 4.55E-08 | -0.0762 | 0 | 0.000 |
| ENSP00000331376 | 9.75E-06 | -0.0763 | 0 | 0.096 |
| ENSP00000362803 | 1.75E-05 | -0.0763 | 244 | 0.796 |
| ENSP00000296684 | 6.39E-06 | -0.0763 | 0 | 0.187 |
| ENSP00000311862 | 1.20E-05 | -0.0763 | 0 | 0.189 |
| ENSP00000301408 | 2.60E-05 | -0.0763 | 0 | 0.462 |
| ENSP00000258499 | 7.43E-06 | -0.0763 | 0 | 0.856 |
| ENSP00000262316 | 6.63E-06 | -0.0763 | 0 | 0.143 |
| ENSP00000310842 | 5.45E-06 | -0.0763 | 0 | 0.627 |
| ENSP00000335655 | 1.02E-05 | -0.0763 | 0 | 0.112 |
| ENSP00000341538 | 7.55E-06 | -0.0763 | 160 | 0.567 |
| ENSP00000296266 | 2.46E-05 | -0.0763 | 223 | 0.428 |
| ENSP00000299206 | 6.26E-06 | -0.0763 | 0 | 0.629 |
| ENSP00000264389 | 6.74E-06 | -0.0763 | 0 | 0.360 |
| ENSP00000225916 | 1.20E-05 | -0.0763 | 399 | 0.000 |
| ENSP00000391453 | 7.58E-06 | -0.0763 | 0 | 0.155 |
| ENSP00000263985 | 6.11E-06 | -0.0763 | 0 | 0.000 |
| ENSP00000253329 | 1.08E-05 | -0.0763 | 244 | 0.737 |
| ENSP00000335614 | 6.40E-06 | -0.0763 | 0 | 0.083 |

| | | | | |
|---|---|---|---|---|
| ENSP00000302783 | 6.77E-06 | -0.0763 | 0 | 0.159 |
| ENSP00000431872 | 2.23E-05 | -0.0763 | 310 | 0.673 |
| ENSP00000316596 | 7.98E-06 | -0.0763 | 0 | 0.046 |
| ENSP00000298472 | 8.85E-06 | -0.0764 | 0 | 0.427 |
| ENSP00000331907 | 1.72E-05 | -0.0764 | 306 | 0.933 |
| ENSP00000318355 | 1.33E-05 | -0.0764 | 193 | 0.261 |
| ENSP00000274813 | 2.02E-05 | -0.0764 | 190 | 0.635 |
| ENSP00000320176 | 6.91E-06 | -0.0764 | 0 | 0.389 |
| ENSP00000310275 | 1.73E-05 | -0.0764 | 207 | 0.824 |
| ENSP00000288422 | 1.08E-05 | -0.0764 | 0 | 0.630 |
| ENSP00000265164 | 2.41E-05 | -0.0764 | 954 | 0.750 |
| ENSP00000237530 | 5.82E-06 | -0.0764 | 0 | 0.494 |
| ENSP00000233596 | 7.39E-06 | -0.0764 | 0 | 0.094 |
| ENSP00000296137 | 7.35E-06 | -0.0764 | 0 | 0.073 |
| ENSP00000365443 | 3.27E-06 | -0.0764 | 0 | 0.000 |
| ENSP00000288670 | 5.68E-06 | -0.0764 | 0 | 0.115 |
| ENSP00000340925 | 8.20E-06 | -0.0764 | 349 | 0.000 |
| ENSP00000247655 | 5.94E-06 | -0.0765 | 0 | 0.301 |
| ENSP00000250237 | 6.24E-06 | -0.0765 | 0 | 0.559 |
| ENSP00000324392 | 7.61E-06 | -0.0765 | 0 | 0.143 |
| ENSP00000229854 | 1.18E-05 | -0.0765 | 197 | 0.788 |
| ENSP00000322343 | 5.23E-06 | -0.0765 | 0 | 0.731 |
| ENSP00000303434 | 6.87E-06 | -0.0765 | 0 | 0.133 |
| ENSP00000298355 | 9.05E-06 | -0.0765 | 0 | 0.171 |
| ENSP00000327009 | 6.80E-06 | -0.0765 | 0 | 0.082 |
| ENSP00000333037 | 2.88E-05 | -0.0765 | 449 | 0.693 |
| ENSP00000363822 | 6.31E-05 | -0.0765 | 843 | 0.893 |
| ENSP00000233714 | 8.02E-06 | -0.0765 | 0 | 0.088 |
| ENSP00000307939 | 8.06E-06 | -0.0765 | 0 | 0.084 |
| ENSP00000250784 | 1.04E-05 | -0.0766 | 179 | 0.866 |
| ENSP00000174618 | 3.82E-05 | -0.0766 | 301 | 0.714 |
| ENSP00000243056 | 5.09E-06 | -0.0766 | 0 | 0.760 |
| ENSP00000356135 | 2.40E-07 | -0.0766 | 0 | 0.172 |
| ENSP00000340737 | 7.99E-06 | -0.0766 | 0 | 0.704 |
| ENSP00000265044 | 6.34E-06 | -0.0766 | 0 | 0.394 |
| ENSP00000265715 | 6.99E-06 | -0.0766 | 0 | 0.287 |
| ENSP00000334128 | 7.73E-06 | -0.0766 | 0 | 0.177 |
| ENSP00000228825 | 6.31E-06 | -0.0766 | 0 | 0.398 |
| ENSP00000310040 | 1.74E-05 | -0.0766 | 307 | 0.773 |
| ENSP00000352516 | 4.75E-05 | -0.0767 | 593 | 0.793 |
| ENSP00000348918 | 3.28E-05 | -0.0767 | 473 | 0.731 |
| ENSP00000217073 | 2.17E-05 | -0.0767 | 522 | 0.921 |
| ENSP00000249750 | 1.40E-05 | -0.0767 | 548 | 0.616 |
| ENSP00000294904 | 1.32E-05 | -0.0767 | 0 | 0.401 |
| ENSP00000299502 | 5.85E-05 | -0.0767 | 472 | 0.871 |
| ENSP00000299293 | 2.67E-05 | -0.0767 | 286 | 0.000 |

| | | | | |
|---|---|---|---|---|
| ENSP00000361207 | 2.40E-05 | -0.0767 | 198 | 0.414 |
| ENSP00000012443 | 9.96E-06 | -0.0767 | 283 | 0.673 |
| ENSP00000315925 | 1.97E-05 | -0.0767 | 186 | 0.178 |
| ENSP00000312837 | 1.27E-05 | -0.0767 | 0 | 0.000 |
| ENSP00000256190 | 7.06E-06 | -0.0768 | 0 | 0.085 |
| ENSP00000285046 | 1.00E-05 | -0.0768 | 0 | 0.095 |
| ENSP00000275517 | 4.92E-06 | -0.0768 | 0 | 0.377 |
| ENSP00000296438 | 2.58E-06 | -0.0768 | 0 | 0.210 |
| ENSP00000263354 | 6.63E-06 | -0.0768 | 0 | 0.231 |
| ENSP00000261749 | 8.13E-06 | -0.0768 | 0 | 0.111 |
| ENSP00000359368 | 7.66E-06 | -0.0768 | 0 | 0.000 |
| ENSP00000387006 | 2.55E-05 | -0.0768 | 296 | 0.721 |
| ENSP00000222008 | 7.84E-06 | -0.0768 | 0 | 0.183 |
| ENSP00000327821 | 7.09E-06 | -0.0768 | 0 | 0.659 |
| ENSP00000204566 | 8.90E-06 | -0.0768 | 0 | 0.465 |
| ENSP00000327077 | 7.62E-06 | -0.0768 | 0 | 0.154 |
| ENSP00000357356 | 3.36E-06 | -0.0768 | 0 | 0.000 |
| ENSP00000330737 | 5.72E-06 | -0.0768 | 0 | 0.126 |
| ENSP00000296754 | 1.74E-05 | -0.0768 | 766 | 0.460 |
| ENSP00000282223 | 8.66E-05 | -0.0768 | 900 | 0.279 |
| ENSP00000314420 | 7.32E-06 | -0.0768 | 0 | 0.424 |
| ENSP00000287295 | 1.48E-05 | -0.0768 | 383 | 0.647 |
| ENSP00000262173 | 5.93E-06 | -0.0768 | 0 | 0.549 |
| ENSP00000386393 | 3.08E-06 | -0.0768 | 0 | 0.695 |
| ENSP00000248437 | 3.27E-05 | -0.0768 | 557 | 0.609 |
| ENSP00000263346 | 7.14E-06 | -0.0768 | 0 | 0.140 |
| ENSP00000318066 | 9.41E-06 | -0.0768 | 0 | 0.063 |
| ENSP00000333813 | 7.71E-06 | -0.0768 | 249 | 0.120 |
| ENSP00000333551 | 9.47E-06 | -0.0768 | 0 | 0.110 |
| ENSP00000326170 | 7.70E-06 | -0.0768 | 0 | 0.059 |
| ENSP00000188312 | 1.52E-05 | -0.0768 | 319 | 0.685 |
| ENSP00000243997 | 5.26E-06 | -0.0769 | 0 | 0.163 |
| ENSP00000219481 | 7.84E-06 | -0.0769 | 0 | 0.298 |
| ENSP00000356694 | 5.04E-05 | -0.0769 | 883 | 0.930 |
| ENSP00000296277 | 5.72E-06 | -0.0769 | 0 | 0.763 |
| ENSP00000322180 | 4.99E-06 | -0.0769 | 0 | 0.594 |
| ENSP00000339503 | 7.97E-06 | -0.0769 | 195 | 0.385 |
| ENSP00000278353 | 7.48E-06 | -0.0769 | 0 | 0.259 |
| ENSP00000312599 | 6.71E-06 | -0.0769 | 0 | 0.092 |
| ENSP00000200181 | 1.12E-04 | -0.0769 | 847 | 0.858 |
| ENSP00000315351 | 9.01E-06 | -0.0769 | 0 | 0.096 |
| ENSP00000300403 | 1.04E-05 | -0.0769 | 0 | 0.517 |
| ENSP00000301607 | 1.79E-05 | -0.0770 | 0 | 0.199 |
| ENSP00000297423 | 8.36E-05 | -0.0770 | 0 | 0.000 |
| ENSP00000342181 | 6.35E-06 | -0.0770 | 0 | 0.709 |
| ENSP00000402758 | 2.52E-05 | -0.0770 | 206 | 0.692 |

| | | | | |
|---|---|---|---|---|
| ENSP00000312066 | 6.49E-06 | -0.0770 | 0 | 0.667 |
| ENSP00000339353 | 6.30E-06 | -0.0770 | 0 | 0.000 |
| ENSP00000338207 | 2.19E-05 | -0.0770 | 462 | 0.818 |
| ENSP00000259667 | 1.47E-05 | -0.0770 | 301 | 0.413 |
| ENSP00000280871 | 7.24E-06 | -0.0771 | 0 | 0.076 |
| ENSP00000435289 | 2.05E-05 | -0.0771 | 222 | 0.082 |
| ENSP00000321703 | 5.77E-06 | -0.0771 | 0 | 0.241 |
| ENSP00000326572 | 9.60E-06 | -0.0771 | 202 | 0.000 |
| ENSP00000354694 | 3.40E-06 | -0.0771 | 0 | 0.697 |
| ENSP00000339958 | 1.06E-05 | -0.0771 | 261 | 0.592 |
| ENSP00000280886 | 6.59E-06 | -0.0771 | 0 | 0.189 |
| ENSP00000309186 | 2.61E-05 | -0.0771 | 161 | 0.156 |
| ENSP00000362576 | 3.42E-05 | -0.0771 | 608 | 0.705 |
| ENSP00000250003 | 2.94E-05 | -0.0772 | 948 | 0.000 |
| ENSP00000337761 | 1.42E-05 | -0.0772 | 307 | 0.255 |
| ENSP00000318085 | 2.45E-05 | -0.0772 | 196 | 0.746 |
| ENSP00000313377 | 7.24E-06 | -0.0772 | 0 | 0.202 |
| ENSP00000328405 | 6.52E-06 | -0.0772 | 0 | 0.248 |
| ENSP00000220849 | 2.69E-05 | -0.0772 | 370 | 0.888 |
| ENSP00000230792 | 5.49E-06 | -0.0772 | 0 | 0.162 |
| ENSP00000341082 | 5.75E-06 | -0.0772 | 332 | 0.664 |
| ENSP00000283254 | 6.86E-06 | -0.0772 | 0 | 0.348 |
| ENSP00000261867 | 8.22E-06 | -0.0772 | 0 | 0.078 |
| ENSP00000312814 | 5.69E-06 | -0.0772 | 0 | 0.102 |
| ENSP00000305459 | 6.48E-06 | -0.0772 | 0 | 0.142 |
| ENSP00000354932 | 2.04E-05 | -0.0772 | 284 | 0.880 |
| ENSP00000251968 | 1.94E-05 | -0.0773 | 270 | 0.464 |
| ENSP00000222214 | 6.06E-06 | -0.0773 | 0 | 0.288 |
| ENSP00000291442 | 7.89E-06 | -0.0773 | 0 | 0.842 |
| ENSP00000249071 | 1.31E-05 | -0.0773 | 433 | 0.560 |
| ENSP00000330930 | 1.35E-05 | -0.0773 | 0 | 0.000 |
| ENSP00000225603 | 7.37E-06 | -0.0773 | 0 | 0.832 |
| ENSP00000287394 | 2.04E-05 | -0.0773 | 272 | 0.789 |
| ENSP00000301962 | 6.22E-06 | -0.0773 | 0 | 0.624 |
| ENSP00000301587 | 1.28E-05 | -0.0773 | 205 | 0.369 |
| ENSP00000007516 | 9.80E-06 | -0.0773 | 191 | 0.449 |
| ENSP00000362931 | 9.51E-04 | -0.0773 | 496 | 0.788 |
| ENSP00000332549 | 2.82E-05 | -0.0773 | 252 | 0.761 |
| ENSP00000261497 | 9.59E-06 | -0.0773 | 607 | 0.893 |
| ENSP00000346733 | 4.26E-05 | -0.0773 | 242 | 0.208 |
| ENSP00000358918 | 3.41E-05 | -0.0774 | 233 | 0.819 |
| ENSP00000419718 | 3.10E-06 | -0.0774 | 0 | 0.075 |
| ENSP00000336747 | 2.33E-05 | -0.0774 | 359 | 0.300 |
| ENSP00000216714 | 1.27E-05 | -0.0774 | 215 | 0.735 |
| ENSP00000333537 | 9.50E-06 | -0.0774 | 0 | 0.000 |
| ENSP00000254759 | 5.64E-06 | -0.0774 | 0 | 0.198 |

| | | | | |
|---|---|---|---|---|
| ENSP00000323687 | 5.65E-06 | -0.0774 | 0 | 0.206 |
| ENSP00000265593 | 6.23E-06 | -0.0774 | 0 | 0.172 |
| ENSP00000258975 | 6.12E-06 | -0.0774 | 0 | 0.314 |
| ENSP00000266037 | 9.25E-06 | -0.0774 | 250 | 0.390 |
| ENSP00000013070 | 2.11E-05 | -0.0774 | 0 | 0.187 |
| ENSP00000019317 | 5.78E-06 | -0.0774 | 0 | 0.415 |
| ENSP00000254928 | 5.80E-06 | -0.0774 | 0 | 0.332 |
| ENSP00000221486 | 1.08E-05 | -0.0774 | 185 | 0.599 |
| ENSP00000301463 | 1.59E-05 | -0.0774 | 0 | 0.152 |
| ENSP00000382030 | 1.19E-05 | -0.0774 | 0 | 0.164 |
| ENSP00000266505 | 6.03E-06 | -0.0774 | 0 | 0.225 |
| ENSP00000311219 | 1.69E-05 | -0.0775 | 0 | 0.000 |
| ENSP00000184956 | 2.38E-05 | -0.0775 | 0 | 0.737 |
| ENSP00000407436 | 2.57E-05 | -0.0775 | 326 | 0.000 |
| ENSP00000306003 | 4.65E-06 | -0.0775 | 0 | 0.261 |
| ENSP00000223054 | 2.73E-05 | -0.0775 | 187 | 0.132 |
| ENSP00000302936 | 8.25E-06 | -0.0775 | 0 | 0.063 |
| ENSP00000371271 | 2.82E-07 | -0.0775 | 0 | 0.187 |
| ENSP00000224950 | 3.00E-05 | -0.0775 | 194 | 0.723 |
| ENSP00000258455 | 1.07E-05 | -0.0775 | 365 | 0.787 |
| ENSP00000346509 | 8.69E-06 | -0.0775 | 0 | 0.996 |
| ENSP00000238558 | 6.14E-06 | -0.0775 | 0 | 0.788 |
| ENSP00000174653 | 6.30E-06 | -0.0776 | 0 | 0.105 |
| ENSP00000319104 | 1.79E-05 | -0.0776 | 181 | 0.888 |
| ENSP00000040877 | 5.97E-06 | -0.0776 | 0 | 0.661 |
| ENSP00000357204 | 3.14E-05 | -0.0776 | 198 | 0.860 |
| ENSP00000227474 | 1.01E-05 | -0.0776 | 317 | 0.688 |
| ENSP00000235349 | 7.58E-05 | -0.0776 | 0 | 0.184 |
| ENSP00000238721 | 6.08E-06 | -0.0776 | 0 | 0.206 |
| ENSP00000251412 | 1.14E-05 | -0.0776 | 229 | 0.423 |
| ENSP00000342300 | 1.19E-05 | -0.0776 | 0 | 0.644 |
| ENSP00000343464 | 2.82E-05 | -0.0776 | 562 | 0.770 |
| ENSP00000334280 | 6.40E-06 | -0.0777 | 0 | 0.323 |
| ENSP00000350848 | 5.43E-06 | -0.0777 | 0 | 0.146 |
| ENSP00000329715 | 7.31E-06 | -0.0777 | 405 | 0.820 |
| ENSP00000306449 | 1.64E-05 | -0.0777 | 271 | 0.428 |
| ENSP00000299505 | 2.16E-05 | -0.0777 | 0 | 0.104 |
| ENSP00000324527 | 2.60E-05 | -0.0777 | 244 | 0.384 |
| ENSP00000252771 | 5.43E-06 | -0.0777 | 0 | 0.257 |
| ENSP00000276062 | 5.96E-06 | -0.0777 | 0 | 0.332 |
| ENSP00000376110 | 3.23E-06 | -0.0777 | 0 | 0.693 |
| ENSP00000292807 | 1.46E-05 | -0.0777 | 190 | 0.307 |
| ENSP00000347210 | 3.48E-06 | -0.0777 | 0 | 0.697 |
| ENSP00000317039 | 1.52E-05 | -0.0777 | 159 | 0.511 |
| ENSP00000338728 | 9.15E-06 | -0.0777 | 0 | 0.133 |
| ENSP00000222644 | 1.00E-05 | -0.0777 | 302 | 0.505 |

| | | | | |
|---|---|---|---|---|
| ENSP00000262302 | 1.33E-05 | -0.0777 | 185 | 0.231 |
| ENSP00000216484 | 1.92E-05 | -0.0778 | 229 | 0.377 |
| ENSP00000300417 | 2.31E-05 | -0.0778 | 372 | 0.698 |
| ENSP00000357810 | 4.90E-06 | -0.0778 | 0 | 0.667 |
| ENSP00000292475 | 5.08E-06 | -0.0778 | 0 | 0.256 |
| ENSP00000308275 | 8.14E-06 | -0.0778 | 0 | 0.374 |
| ENSP00000356655 | 2.91E-06 | -0.0778 | 0 | 0.191 |
| ENSP00000274335 | 2.22E-05 | -0.0778 | 901 | 0.000 |
| ENSP00000346348 | 3.34E-06 | -0.0779 | 0 | 0.697 |
| ENSP00000343081 | 4.98E-06 | -0.0779 | 0 | 0.827 |
| ENSP00000234626 | 1.13E-05 | -0.0779 | 163 | 0.732 |
| ENSP00000318604 | 5.62E-06 | -0.0779 | 0 | 0.551 |
| ENSP00000363095 | 1.03E-05 | -0.0779 | 329 | 0.377 |
| ENSP00000199447 | 1.21E-05 | -0.0779 | 398 | 0.583 |
| ENSP00000222553 | 8.26E-05 | -0.0779 | 345 | 0.720 |
| ENSP00000397881 | 1.51E-06 | -0.0779 | 0 | 0.118 |
| ENSP00000285298 | 6.18E-06 | -0.0779 | 0 | 0.000 |
| ENSP00000310127 | 2.61E-05 | -0.0779 | 907 | 0.865 |
| ENSP00000219794 | 1.21E-05 | -0.0779 | 186 | 0.438 |
| ENSP00000377721 | 5.86E-05 | -0.0779 | 231 | 0.879 |
| ENSP00000248929 | 6.06E-06 | -0.0779 | 0 | 0.245 |
| ENSP00000290231 | 6.23E-06 | -0.0779 | 0 | 0.400 |
| ENSP00000337452 | 1.08E-05 | -0.0779 | 0 | 0.103 |
| ENSP00000297954 | 8.17E-06 | -0.0779 | 0 | 0.070 |
| ENSP00000253413 | 5.65E-06 | -0.0779 | 0 | 0.189 |
| ENSP00000260102 | 9.34E-06 | -0.0780 | 183 | 0.800 |
| ENSP00000307260 | 4.87E-06 | -0.0780 | 0 | 0.452 |
| ENSP00000328808 | 1.39E-05 | -0.0780 | 402 | 0.000 |
| ENSP00000171111 | 6.24E-05 | -0.0780 | 349 | 0.739 |
| ENSP00000341268 | 2.28E-05 | -0.0780 | 155 | 0.768 |
| ENSP00000445677 | 3.60E-05 | -0.0780 | 258 | 0.000 |
| ENSP00000348349 | 2.01E-05 | -0.0780 | 244 | 0.438 |
| ENSP00000304292 | 1.50E-05 | -0.0780 | 260 | 0.615 |
| ENSP00000296870 | 4.02E-05 | -0.0780 | 459 | 0.943 |
| ENSP00000321320 | 5.70E-06 | -0.0780 | 0 | 0.000 |
| ENSP00000312107 | 1.18E-05 | -0.0781 | 154 | 0.085 |
| ENSP00000295266 | 2.32E-05 | -0.0781 | 262 | 0.392 |
| ENSP00000320646 | 6.42E-06 | -0.0781 | 0 | 0.281 |
| ENSP00000379092 | 1.61E-05 | -0.0781 | 665 | 0.841 |
| ENSP00000291386 | 9.17E-06 | -0.0781 | 0 | 0.651 |
| ENSP00000266070 | 7.07E-06 | -0.0781 | 0 | 0.830 |
| ENSP00000332791 | 1.09E-05 | -0.0781 | 0 | 0.141 |
| ENSP00000264930 | 5.55E-06 | -0.0781 | 0 | 0.101 |
| ENSP00000378349 | 1.03E-05 | -0.0781 | 352 | 0.119 |
| ENSP00000218348 | 5.28E-06 | -0.0781 | 0 | 0.424 |
| ENSP00000262144 | 2.15E-05 | -0.0781 | 202 | 0.923 |

| | | | | |
|---|---|---|---|---|
| ENSP00000250405 | 5.20E-05 | -0.0781 | 186 | 0.691 |
| ENSP00000323377 | 5.57E-06 | -0.0781 | 0 | 0.283 |
| ENSP00000301761 | 3.93E-05 | -0.0781 | 195 | 0.469 |
| ENSP00000253925 | 4.37E-06 | -0.0782 | 0 | 0.132 |
| ENSP00000363869 | 2.14E-05 | -0.0782 | 252 | 0.225 |
| ENSP00000383042 | 4.24E-05 | -0.0782 | 487 | 0.000 |
| ENSP00000264028 | 1.21E-05 | -0.0782 | 306 | 0.190 |
| ENSP00000300747 | 1.86E-05 | -0.0782 | 570 | 0.296 |
| ENSP00000327124 | 1.03E-05 | -0.0782 | 0 | 0.000 |
| ENSP00000275230 | 7.40E-06 | -0.0782 | 0 | 0.115 |
| ENSP00000330216 | 1.17E-05 | -0.0782 | 0 | 0.269 |
| ENSP00000215574 | 1.23E-05 | -0.0782 | 178 | 0.747 |
| ENSP00000333633 | 1.16E-04 | -0.0782 | 906 | 0.775 |
| ENSP00000351894 | 3.18E-05 | -0.0782 | 900 | 0.781 |
| ENSP00000254181 | 1.88E-05 | -0.0782 | 223 | 0.252 |
| ENSP00000216442 | 6.36E-06 | -0.0782 | 0 | 0.074 |
| ENSP00000295709 | 1.13E-05 | -0.0782 | 194 | 0.251 |
| ENSP00000265245 | 1.09E-05 | -0.0782 | 240 | 0.901 |
| ENSP00000360683 | 3.35E-05 | -0.0782 | 913 | 0.916 |
| ENSP00000329097 | 5.94E-06 | -0.0782 | 0 | 0.590 |
| ENSP00000287156 | 1.97E-05 | -0.0783 | 256 | 0.645 |
| ENSP00000341562 | 3.98E-05 | -0.0783 | 197 | 0.154 |
| ENSP00000262584 | 1.17E-05 | -0.0783 | 760 | 0.832 |
| ENSP00000233336 | 4.31E-05 | -0.0783 | 242 | 0.313 |
| ENSP00000323967 | 7.51E-06 | -0.0783 | 0 | 0.816 |
| ENSP00000250617 | 6.07E-06 | -0.0783 | 0 | 0.420 |
| ENSP00000265097 | 3.52E-05 | -0.0783 | 904 | 0.519 |
| ENSP00000313869 | 1.40E-05 | -0.0783 | 240 | 0.140 |
| ENSP00000299443 | 1.29E-05 | -0.0783 | 266 | 0.679 |
| ENSP00000222753 | 8.33E-06 | -0.0783 | 0 | 0.833 |
| ENSP00000256031 | 6.57E-06 | -0.0783 | 0 | 0.141 |
| ENSP00000281187 | 6.36E-06 | -0.0783 | 0 | 0.147 |
| ENSP00000265038 | 5.54E-06 | -0.0783 | 0 | 0.599 |
| ENSP00000267103 | 5.70E-06 | -0.0783 | 0 | 0.736 |
| ENSP00000270570 | 5.66E-06 | -0.0784 | 0 | 0.072 |
| ENSP00000310568 | 6.55E-06 | -0.0784 | 0 | 0.176 |
| ENSP00000280700 | 6.60E-06 | -0.0784 | 0 | 0.105 |
| ENSP00000295902 | 1.12E-05 | -0.0784 | 270 | 0.597 |
| ENSP00000362110 | 1.96E-05 | -0.0784 | 443 | 0.883 |
| ENSP00000356674 | 3.32E-06 | -0.0784 | 0 | 0.699 |
| ENSP00000333253 | 8.71E-06 | -0.0784 | 0 | 0.379 |
| ENSP00000335463 | 4.71E-06 | -0.0784 | 0 | 0.333 |
| ENSP00000377789 | 1.89E-05 | -0.0784 | 262 | 0.574 |
| ENSP00000274712 | 9.82E-06 | -0.0784 | 165 | 0.430 |
| ENSP00000215095 | 1.96E-05 | -0.0784 | 377 | 0.258 |
| ENSP00000255381 | 1.76E-05 | -0.0785 | 434 | 0.511 |

| | | | | |
|---|---|---|---|---|
| ENSP00000261245 | 6.31E-06 | -0.0785 | 0 | 0.776 |
| ENSP00000462986 | 1.99E-05 | -0.0785 | 502 | 0.000 |
| ENSP00000310966 | 7.73E-06 | -0.0785 | 0 | 0.430 |
| ENSP00000337946 | 1.48E-05 | -0.0785 | 311 | 0.235 |
| ENSP00000332969 | 9.71E-06 | -0.0785 | 0 | 0.252 |
| ENSP00000282344 | 6.90E-06 | -0.0785 | 0 | 0.170 |
| ENSP00000335082 | 1.95E-05 | -0.0785 | 0 | 0.126 |
| ENSP00000311095 | 8.50E-06 | -0.0785 | 0 | 0.361 |
| ENSP00000282007 | 6.83E-06 | -0.0785 | 0 | 0.702 |
| ENSP00000324127 | 6.70E-06 | -0.0785 | 0 | 0.135 |
| ENSP00000266376 | 1.67E-05 | -0.0785 | 239 | 0.574 |
| ENSP00000253024 | 6.42E-06 | -0.0785 | 0 | 0.799 |
| ENSP00000245816 | 1.59E-05 | -0.0786 | 508 | 0.715 |
| ENSP00000308000 | 2.06E-05 | -0.0786 | 159 | 0.821 |
| ENSP00000365370 | 3.62E-05 | -0.0786 | 195 | 0.977 |
| ENSP00000408994 | 2.12E-05 | -0.0786 | 175 | 0.491 |
| ENSP00000308012 | 2.14E-05 | -0.0786 | 502 | 0.888 |
| ENSP00000366934 | 7.71E-06 | -0.0786 | 0 | 0.570 |
| ENSP00000245503 | 1.63E-05 | -0.0786 | 900 | 0.562 |
| ENSP00000257548 | 5.65E-06 | -0.0786 | 0 | 0.792 |
| ENSP00000323300 | 6.54E-06 | -0.0786 | 0 | 0.254 |
| ENSP00000412292 | 4.01E-05 | -0.0786 | 150 | 0.195 |
| ENSP00000295066 | 5.71E-06 | -0.0786 | 0 | 0.612 |
| ENSP00000376930 | 8.42E-06 | -0.0786 | 0 | 0.069 |
| ENSP00000280346 | 1.52E-05 | -0.0786 | 350 | 0.555 |
| ENSP00000212355 | 9.97E-05 | -0.0786 | 915 | 0.683 |
| ENSP00000276689 | 5.24E-06 | -0.0786 | 0 | 0.203 |
| ENSP00000260433 | 3.40E-05 | -0.0786 | 358 | 0.892 |
| ENSP00000261726 | 6.19E-06 | -0.0786 | 0 | 0.524 |
| ENSP00000228284 | 7.01E-06 | -0.0786 | 0 | 0.825 |
| ENSP00000454021 | 1.77E-05 | -0.0786 | 154 | 0.687 |
| ENSP00000372484 | 1.78E-07 | -0.0787 | 0 | 0.215 |
| ENSP00000294618 | 7.98E-06 | -0.0787 | 0 | 0.193 |
| ENSP00000318176 | 6.50E-06 | -0.0787 | 0 | 0.542 |
| ENSP00000306190 | 1.02E-05 | -0.0787 | 0 | 0.857 |
| ENSP00000276066 | 2.21E-05 | -0.0787 | 196 | 0.241 |
| ENSP00000320672 | 5.05E-06 | -0.0787 | 0 | 0.109 |
| ENSP00000320567 | 5.86E-06 | -0.0787 | 0 | 0.255 |
| ENSP00000171214 | 1.07E-04 | -0.0787 | 769 | 0.000 |
| ENSP00000270474 | 1.70E-05 | -0.0787 | 514 | 0.000 |
| ENSP00000367972 | 2.46E-05 | -0.0787 | 209 | 0.867 |
| ENSP00000360183 | 2.73E-05 | -0.0788 | 327 | 0.241 |
| ENSP00000380840 | 4.08E-06 | -0.0788 | 0 | 0.699 |
| ENSP00000420298 | 2.00E-05 | -0.0788 | 159 | 0.279 |
| ENSP00000305847 | 6.02E-05 | -0.0788 | 0 | 0.174 |
| ENSP00000376250 | 1.44E-05 | -0.0788 | 150 | 0.094 |

| | | | | |
|---|---|---|---|---|
| ENSP00000252050 | 1.41E-05 | -0.0788 | 0 | 0.252 |
| ENSP00000258873 | 1.99E-05 | -0.0788 | 167 | 0.476 |
| ENSP00000360165 | 3.67E-05 | -0.0788 | 449 | 0.688 |
| ENSP00000346050 | 5.13E-05 | -0.0788 | 294 | 0.767 |
| ENSP00000248846 | 5.82E-06 | -0.0788 | 0 | 0.106 |
| ENSP00000287652 | 2.48E-05 | -0.0788 | 201 | 0.243 |
| ENSP00000262544 | 1.21E-05 | -0.0788 | 415 | 0.282 |
| ENSP00000375229 | 1.04E-05 | -0.0788 | 0 | 0.000 |
| ENSP00000245544 | 1.07E-05 | -0.0789 | 576 | 0.550 |
| ENSP00000304440 | 3.10E-05 | -0.0789 | 215 | 0.345 |
| ENSP00000309415 | 6.60E-06 | -0.0789 | 0 | 0.145 |
| ENSP00000237853 | 1.17E-05 | -0.0789 | 0 | 0.661 |
| ENSP00000362618 | 1.87E-05 | -0.0789 | 508 | 0.918 |
| ENSP00000281701 | 1.39E-05 | -0.0789 | 289 | 0.617 |
| ENSP00000362621 | 1.87E-05 | -0.0789 | 508 | 0.918 |
| ENSP00000327604 | 1.12E-05 | -0.0789 | 0 | 0.278 |
| ENSP00000295887 | 1.40E-05 | -0.0789 | 200 | 0.323 |
| ENSP00000262315 | 4.80E-06 | -0.0789 | 0 | 0.667 |
| ENSP00000160382 | 1.27E-05 | -0.0789 | 316 | 0.735 |
| ENSP00000341071 | 1.88E-05 | -0.0789 | 184 | 0.508 |
| ENSP00000360034 | 8.67E-05 | -0.0789 | 198 | 0.789 |
| ENSP00000263269 | 6.15E-06 | -0.0789 | 0 | 0.580 |
| ENSP00000339720 | 5.37E-06 | -0.0790 | 0 | 0.175 |
| ENSP00000264161 | 1.26E-05 | -0.0790 | 258 | 0.679 |
| ENSP00000390179 | 5.17E-06 | -0.0790 | 0 | 0.690 |
| ENSP00000219343 | 6.82E-06 | -0.0790 | 0 | 0.068 |
| ENSP00000361373 | 1.10E-05 | -0.0790 | 163 | 0.742 |
| ENSP00000396259 | 5.20E-05 | -0.0790 | 214 | 0.879 |
| ENSP00000203407 | 8.35E-06 | -0.0790 | 282 | 0.491 |
| ENSP00000315247 | 3.71E-06 | -0.0790 | 0 | 0.217 |
| ENSP00000262432 | 1.31E-05 | -0.0791 | 0 | 0.112 |
| ENSP00000302886 | 1.90E-05 | -0.0791 | 601 | 0.000 |
| ENSP00000287713 | 1.77E-05 | -0.0791 | 252 | 0.351 |
| ENSP00000252136 | 5.05E-06 | -0.0791 | 0 | 0.714 |
| ENSP00000377233 | 1.97E-05 | -0.0791 | 159 | 0.337 |
| ENSP00000354920 | 1.93E-05 | -0.0791 | 214 | 0.208 |
| ENSP00000263246 | 3.10E-05 | -0.0791 | 175 | 0.509 |
| ENSP00000297405 | 7.51E-06 | -0.0791 | 0 | 0.055 |
| ENSP00000353132 | 5.36E-06 | -0.0791 | 0 | 0.763 |
| ENSP00000377700 | 3.99E-06 | -0.0792 | 0 | 0.127 |
| ENSP00000352572 | 2.76E-05 | -0.0792 | 240 | 0.561 |
| ENSP00000219611 | 1.15E-05 | -0.0792 | 193 | 0.000 |
| ENSP00000372124 | 1.78E-07 | -0.0792 | 0 | 0.000 |
| ENSP00000263083 | 1.92E-05 | -0.0792 | 576 | 0.630 |
| ENSP00000349275 | 4.11E-05 | -0.0792 | 264 | 0.917 |
| ENSP00000302719 | 5.86E-06 | -0.0792 | 0 | 0.099 |

| | | | | |
|---|---|---|---|---|
| ENSP00000245796 | 6.32E-06 | -0.0792 | 0 | 0.145 |
| ENSP00000311847 | 1.51E-05 | -0.0792 | 302 | 0.656 |
| ENSP00000357234 | 1.25E-05 | -0.0792 | 0 | 0.194 |
| ENSP00000326550 | 5.80E-06 | -0.0792 | 0 | 0.359 |
| ENSP00000338272 | 5.52E-05 | -0.0792 | 847 | 0.880 |
| ENSP00000279263 | 6.31E-06 | -0.0792 | 0 | 0.240 |
| ENSP00000307046 | 8.11E-05 | -0.0793 | 965 | 0.810 |
| ENSP00000268129 | 6.50E-06 | -0.0793 | 0 | 0.090 |
| ENSP00000293414 | 1.49E-05 | -0.0793 | 307 | 0.647 |
| ENSP00000280612 | 1.34E-05 | -0.0793 | 260 | 0.278 |
| ENSP00000319240 | 1.70E-05 | -0.0793 | 232 | 0.852 |
| ENSP00000334941 | 7.93E-06 | -0.0793 | 0 | 0.636 |
| ENSP00000366372 | 1.32E-06 | -0.0793 | 0 | 0.216 |
| ENSP00000351480 | 2.00E-05 | -0.0793 | 338 | 0.000 |
| ENSP00000340296 | 7.04E-06 | -0.0793 | 183 | 0.000 |
| ENSP00000375748 | 1.79E-05 | -0.0793 | 271 | 0.240 |
| ENSP00000333380 | 1.37E-05 | -0.0793 | 0 | 0.000 |
| ENSP00000261326 | 6.39E-06 | -0.0794 | 0 | 0.070 |
| ENSP00000325485 | 1.36E-05 | -0.0794 | 0 | 0.499 |
| ENSP00000347338 | 4.45E-06 | -0.0794 | 0 | 0.000 |
| ENSP00000447057 | 5.70E-06 | -0.0794 | 0 | 0.192 |
| ENSP00000340454 | 8.30E-06 | -0.0794 | 428 | 0.298 |
| ENSP00000297229 | 5.22E-06 | -0.0794 | 0 | 0.000 |
| ENSP00000340279 | 4.59E-05 | -0.0794 | 0 | 0.302 |
| ENSP00000316377 | 4.01E-06 | -0.0794 | 0 | 0.121 |
| ENSP00000298832 | 5.24E-06 | -0.0794 | 0 | 0.142 |
| ENSP00000343144 | 5.55E-06 | -0.0794 | 0 | 0.271 |
| ENSP00000327738 | 1.10E-05 | -0.0794 | 0 | 0.321 |
| ENSP00000304370 | 1.83E-05 | -0.0794 | 402 | 0.828 |
| ENSP00000239440 | 2.47E-05 | -0.0794 | 154 | 0.336 |
| ENSP00000233969 | 6.16E-06 | -0.0794 | 0 | 0.222 |
| ENSP00000363746 | 2.30E-05 | -0.0794 | 220 | 0.880 |
| ENSP00000334840 | 6.36E-06 | -0.0794 | 0 | 0.000 |
| ENSP00000219479 | 1.42E-05 | -0.0794 | 324 | 0.613 |
| ENSP00000302625 | 2.14E-05 | -0.0794 | 494 | 0.936 |
| ENSP00000312442 | 2.16E-05 | -0.0795 | 184 | 0.171 |
| ENSP00000362082 | 4.79E-05 | -0.0795 | 207 | 0.861 |
| ENSP00000261776 | 5.61E-06 | -0.0795 | 0 | 0.211 |
| ENSP00000265748 | 9.15E-06 | -0.0795 | 0 | 0.239 |
| ENSP00000223215 | 1.68E-05 | -0.0795 | 517 | 0.330 |
| ENSP00000377751 | 2.77E-06 | -0.0795 | 0 | 0.104 |
| ENSP00000316203 | 8.62E-06 | -0.0795 | 0 | 0.000 |
| ENSP00000316021 | 8.27E-06 | -0.0795 | 0 | 0.000 |
| ENSP00000392995 | 2.17E-05 | -0.0795 | 168 | 0.498 |
| ENSP00000332818 | 2.26E-05 | -0.0795 | 161 | 0.098 |
| ENSP00000290541 | 1.89E-05 | -0.0795 | 245 | 0.513 |

| | | | | |
|---|---|---|---|---|
| ENSP00000320543 | 4.97E-06 | -0.0795 | 0 | 0.185 |
| ENSP00000309116 | 8.34E-06 | -0.0795 | 0 | 0.692 |
| ENSP00000215567 | 1.44E-05 | -0.0795 | 242 | 0.230 |
| ENSP00000472367 | 8.30E-08 | -0.0796 | 0 | 0.000 |
| ENSP00000257749 | 9.52E-06 | -0.0796 | 0 | 0.694 |
| ENSP00000313603 | 1.27E-05 | -0.0796 | 157 | 0.711 |
| ENSP00000340900 | 5.99E-06 | -0.0796 | 0 | 0.088 |
| ENSP00000372547 | 5.97E-05 | -0.0796 | 282 | 0.880 |
| ENSP00000347314 | 1.65E-05 | -0.0796 | 556 | 0.253 |
| ENSP00000386126 | 1.42E-05 | -0.0796 | 150 | 0.147 |
| ENSP00000379602 | 2.63E-05 | -0.0796 | 346 | 0.246 |
| ENSP00000297837 | 1.11E-05 | -0.0796 | 202 | 0.624 |
| ENSP00000264883 | 6.94E-06 | -0.0796 | 0 | 0.494 |
| ENSP00000332679 | 1.20E-05 | -0.0796 | 207 | 0.301 |
| ENSP00000251630 | 1.41E-05 | -0.0796 | 217 | 0.672 |
| ENSP00000215071 | 5.53E-06 | -0.0797 | 0 | 0.420 |
| ENSP00000350418 | 3.53E-06 | -0.0797 | 0 | 0.693 |
| ENSP00000369976 | 1.26E-05 | -0.0797 | 158 | 0.092 |
| ENSP00000205214 | 5.96E-06 | -0.0797 | 0 | 0.484 |
| ENSP00000307786 | 2.99E-05 | -0.0797 | 284 | 0.732 |
| ENSP00000349161 | 3.57E-06 | -0.0797 | 0 | 0.692 |
| ENSP00000298838 | 3.32E-05 | -0.0797 | 185 | 0.423 |
| ENSP00000398076 | 6.80E-06 | -0.0797 | 0 | 0.066 |
| ENSP00000349823 | 2.07E-05 | -0.0797 | 160 | 0.920 |
| ENSP00000311873 | 1.84E-05 | -0.0797 | 376 | 0.730 |
| ENSP00000315774 | 1.61E-05 | -0.0797 | 201 | 0.328 |
| ENSP00000338777 | 8.13E-06 | -0.0797 | 0 | 0.122 |
| ENSP00000252622 | 5.52E-06 | -0.0798 | 0 | 0.812 |
| ENSP00000258742 | 5.99E-06 | -0.0798 | 0 | 0.347 |
| ENSP00000242285 | 5.75E-06 | -0.0798 | 0 | 0.248 |
| ENSP00000324074 | 8.77E-06 | -0.0798 | 0 | 0.000 |
| ENSP00000254521 | 7.98E-06 | -0.0798 | 0 | 0.289 |
| ENSP00000469669 | 6.56E-08 | -0.0798 | 0 | 0.000 |
| ENSP00000288111 | 6.90E-06 | -0.0798 | 0 | 0.248 |
| ENSP00000339850 | 5.27E-06 | -0.0798 | 0 | 0.306 |
| ENSP00000308344 | 6.72E-06 | -0.0798 | 0 | 0.000 |
| ENSP00000343366 | 5.97E-07 | -0.0798 | 0 | 0.206 |
| ENSP00000348461 | 5.19E-05 | -0.0798 | 947 | 0.770 |
| ENSP00000472039 | 2.71E-05 | -0.0799 | 202 | 0.000 |
| ENSP00000257694 | 1.22E-05 | -0.0799 | 0 | 0.146 |
| ENSP00000256854 | 5.35E-06 | -0.0799 | 0 | 0.646 |
| ENSP00000326514 | 7.30E-06 | -0.0799 | 284 | 0.237 |
| ENSP00000302160 | 5.98E-06 | -0.0799 | 0 | 0.716 |
| ENSP00000258449 | 5.88E-06 | -0.0799 | 0 | 0.132 |
| ENSP00000267622 | 1.24E-05 | -0.0799 | 0 | 0.358 |
| ENSP00000334246 | 6.65E-06 | -0.0799 | 0 | 0.177 |

| | | | | |
|---|---|---|---|---|
| ENSP00000233535 | 6.75E-06 | -0.0800 | 0 | 0.083 |
| ENSP00000412267 | 1.34E-05 | -0.0800 | 157 | 0.000 |
| ENSP00000342344 | 8.37E-06 | -0.0800 | 0 | 0.104 |
| ENSP00000272167 | 1.98E-05 | -0.0800 | 206 | 0.346 |
| ENSP00000351248 | 5.33E-06 | -0.0800 | 0 | 0.000 |
| ENSP00000316053 | 1.58E-05 | -0.0800 | 891 | 0.252 |
| ENSP00000298281 | 6.85E-06 | -0.0800 | 0 | 0.770 |
| ENSP00000264933 | 6.72E-06 | -0.0800 | 0 | 0.070 |
| ENSP00000353581 | 1.96E-05 | -0.0800 | 724 | 0.000 |
| ENSP00000333194 | 1.44E-05 | -0.0800 | 211 | 0.648 |
| ENSP00000327650 | 1.19E-05 | -0.0800 | 208 | 0.202 |
| ENSP00000275820 | 5.62E-06 | -0.0800 | 0 | 0.773 |
| ENSP00000290524 | 1.11E-05 | -0.0800 | 163 | 0.744 |
| ENSP00000353854 | 1.08E-05 | -0.0800 | 0 | 0.141 |
| ENSP00000248901 | 6.40E-06 | -0.0800 | 0 | 0.136 |
| ENSP00000347712 | 8.64E-06 | -0.0801 | 0 | 0.116 |
| ENSP00000286621 | 1.23E-05 | -0.0801 | 218 | 0.410 |
| ENSP00000271764 | 1.26E-05 | -0.0801 | 0 | 0.400 |
| ENSP00000223369 | 1.86E-05 | -0.0801 | 201 | 0.242 |
| ENSP00000279068 | 6.85E-06 | -0.0801 | 0 | 0.620 |
| ENSP00000222990 | 5.86E-06 | -0.0801 | 0 | 0.126 |
| ENSP00000319341 | 2.11E-05 | -0.0801 | 438 | 0.756 |
| ENSP00000223122 | 1.57E-05 | -0.0801 | 213 | 0.224 |
| ENSP00000291576 | 2.29E-05 | -0.0802 | 189 | 0.879 |
| ENSP00000321821 | 8.42E-06 | -0.0802 | 0 | 0.192 |
| ENSP00000306760 | 9.50E-05 | -0.0802 | 0 | 0.108 |
| ENSP00000297534 | 1.45E-05 | -0.0802 | 0 | 0.107 |
| ENSP00000264159 | 2.27E-05 | -0.0802 | 160 | 0.917 |
| ENSP00000340171 | 7.34E-05 | -0.0802 | 191 | 0.693 |
| ENSP00000361597 | 8.28E-07 | -0.0802 | 0 | 0.159 |
| ENSP00000366352 | 6.37E-07 | -0.0802 | 0 | 0.212 |
| ENSP00000340742 | 8.01E-06 | -0.0802 | 162 | 0.212 |
| ENSP00000272395 | 1.03E-05 | -0.0802 | 0 | 0.310 |
| ENSP00000409316 | 1.19E-07 | -0.0802 | 0 | 0.189 |
| ENSP00000352172 | 3.71E-02 | -0.0802 | 0 | 0.212 |
| ENSP00000252669 | 7.63E-06 | -0.0803 | 0 | 0.000 |
| ENSP00000216254 | 1.25E-05 | -0.0803 | 370 | 0.604 |
| ENSP00000328854 | 1.01E-05 | -0.0803 | 191 | 0.867 |
| ENSP00000302227 | 4.99E-06 | -0.0803 | 0 | 0.219 |
| ENSP00000227349 | 3.07E-04 | -0.0803 | 0 | 0.190 |
| ENSP00000454404 | 1.15E-06 | -0.0803 | 0 | 0.186 |
| ENSP00000296102 | 6.27E-06 | -0.0803 | 0 | 0.589 |
| ENSP00000216840 | 1.14E-05 | -0.0803 | 0 | 0.087 |
| ENSP00000296411 | 1.09E-05 | -0.0803 | 305 | 0.796 |
| ENSP00000341159 | 3.49E-05 | -0.0803 | 560 | 0.322 |
| ENSP00000252818 | 2.48E-05 | -0.0803 | 927 | 0.000 |

| | | | | |
|---|---|---|---|---|
| ENSP00000282466 | 5.50E-06 | -0.0803 | 0 | 0.445 |
| ENSP00000254108 | 3.48E-05 | -0.0804 | 571 | 0.792 |
| ENSP00000275730 | 5.80E-06 | -0.0804 | 0 | 0.072 |
| ENSP00000405218 | 2.21E-05 | -0.0804 | 358 | 0.716 |
| ENSP00000447300 | 1.58E-05 | -0.0804 | 323 | 0.216 |
| ENSP00000356975 | 8.40E-05 | -0.0804 | 598 | 0.644 |
| ENSP00000339795 | 2.06E-05 | -0.0804 | 301 | 0.896 |
| ENSP00000318867 | 7.75E-06 | -0.0805 | 0 | 0.268 |
| ENSP00000342262 | 7.67E-06 | -0.0805 | 0 | 0.469 |
| ENSP00000341189 | 5.46E-05 | -0.0805 | 939 | 0.932 |
| ENSP00000358397 | 3.94E-06 | -0.0805 | 0 | 0.163 |
| ENSP00000364813 | 2.07E-05 | -0.0805 | 262 | 0.761 |
| ENSP00000324956 | 2.26E-05 | -0.0805 | 930 | 0.633 |
| ENSP00000396749 | 7.32E-06 | -0.0805 | 175 | 0.355 |
| ENSP00000256906 | 1.85E-05 | -0.0805 | 325 | 0.482 |
| ENSP00000358452 | 7.79E-06 | -0.0805 | 0 | 0.000 |
| ENSP00000265564 | 5.77E-06 | -0.0805 | 0 | 0.845 |
| ENSP00000257789 | 8.14E-06 | -0.0805 | 0 | 0.641 |
| ENSP00000343867 | 8.46E-06 | -0.0805 | 307 | 0.672 |
| ENSP00000202816 | 4.82E-06 | -0.0805 | 0 | 0.881 |
| ENSP00000356070 | 8.14E-05 | -0.0806 | 214 | 0.832 |
| ENSP00000366288 | 2.37E-05 | -0.0806 | 810 | 0.302 |
| ENSP00000351391 | 3.98E-06 | -0.0806 | 0 | 0.703 |
| ENSP00000311429 | 4.33E-06 | -0.0806 | 0 | 0.416 |
| ENSP00000317473 | 5.48E-06 | -0.0806 | 0 | 0.144 |
| ENSP00000301764 | 1.94E-05 | -0.0806 | 216 | 0.830 |
| ENSP00000297591 | 6.21E-06 | -0.0806 | 0 | 0.336 |
| ENSP00000364561 | 8.23E-02 | -0.0806 | 0 | 0.210 |
| ENSP00000297988 | 1.06E-05 | -0.0806 | 196 | 0.301 |
| ENSP00000261514 | 6.20E-06 | -0.0806 | 0 | 0.210 |
| ENSP00000356898 | 2.46E-04 | -0.0806 | 574 | 0.654 |
| ENSP00000215941 | 1.22E-05 | -0.0806 | 218 | 0.714 |
| ENSP00000244745 | 7.44E-05 | -0.0806 | 294 | 0.872 |
| ENSP00000306080 | 5.27E-06 | -0.0806 | 0 | 0.536 |
| ENSP00000433712 | 1.62E-05 | -0.0806 | 280 | 0.187 |
| ENSP00000330836 | 7.06E-06 | -0.0806 | 222 | 0.273 |
| ENSP00000252115 | 1.81E-05 | -0.0806 | 399 | 0.876 |
| ENSP00000023064 | 6.50E-06 | -0.0806 | 0 | 0.074 |
| ENSP00000338673 | 5.43E-06 | -0.0806 | 0 | 0.485 |
| ENSP00000270279 | 9.88E-06 | -0.0806 | 0 | 0.570 |
| ENSP00000337510 | 1.38E-05 | -0.0807 | 0 | 0.095 |
| ENSP00000252809 | 6.02E-05 | -0.0807 | 312 | 0.910 |
| ENSP00000343040 | 5.16E-05 | -0.0807 | 889 | 0.798 |
| ENSP00000331640 | 6.58E-05 | -0.0807 | 0 | 0.159 |
| ENSP00000337358 | 4.61E-06 | -0.0807 | 0 | 0.065 |
| ENSP00000306983 | 1.97E-05 | -0.0807 | 206 | 0.924 |

| | | | | |
|---|---|---|---|---|
| ENSP00000442304 | 6.30E-06 | -0.0807 | 0 | 0.152 |
| ENSP00000254667 | 5.25E-06 | -0.0807 | 0 | 0.614 |
| ENSP00000263672 | 6.21E-06 | -0.0807 | 0 | 0.396 |
| ENSP00000367300 | 3.08E-06 | -0.0807 | 0 | 0.697 |
| ENSP00000320604 | 5.85E-06 | -0.0807 | 0 | 0.182 |
| ENSP00000309501 | 5.54E-06 | -0.0808 | 0 | 0.000 |
| ENSP00000306579 | 1.99E-05 | -0.0808 | 292 | 0.526 |
| ENSP00000231706 | 5.46E-06 | -0.0808 | 0 | 0.079 |
| ENSP00000299824 | 1.61E-05 | -0.0808 | 280 | 0.743 |
| ENSP00000309690 | 2.43E-05 | -0.0808 | 242 | 0.177 |
| ENSP00000263317 | 1.02E-04 | -0.0808 | 516 | 0.878 |
| ENSP00000281928 | 8.54E-06 | -0.0808 | 0 | 0.692 |
| ENSP00000345797 | 3.34E-06 | -0.0808 | 0 | 0.695 |
| ENSP00000257347 | 9.44E-06 | -0.0808 | 362 | 0.716 |
| ENSP00000007969 | 1.44E-05 | -0.0808 | 412 | 0.480 |
| ENSP00000303754 | 1.18E-05 | -0.0808 | 571 | 0.665 |
| ENSP00000361824 | 3.84E-05 | -0.0808 | 984 | 0.696 |
| ENSP00000223136 | 5.75E-06 | -0.0809 | 0 | 0.303 |
| ENSP00000315212 | 8.67E-06 | -0.0809 | 0 | 0.735 |
| ENSP00000218089 | 4.29E-06 | -0.0809 | 0 | 0.633 |
| ENSP00000315700 | 2.34E-05 | -0.0809 | 150 | 0.823 |
| ENSP00000386110 | 2.59E-08 | -0.0809 | 0 | 0.000 |
| ENSP00000404583 | 2.59E-08 | -0.0809 | 0 | 0.000 |
| ENSP00000309429 | 3.95E-05 | -0.0809 | 0 | 0.095 |
| ENSP00000313995 | 4.36E-06 | -0.0809 | 0 | 0.302 |
| ENSP00000324422 | 7.71E-05 | -0.0809 | 670 | 0.822 |
| ENSP00000318154 | 5.25E-05 | -0.0809 | 0 | 0.169 |
| ENSP00000253778 | 1.03E-05 | -0.0809 | 317 | 0.550 |
| ENSP00000268933 | 4.41E-06 | -0.0809 | 0 | 0.105 |
| ENSP00000228799 | 8.20E-06 | -0.0809 | 0 | 0.148 |
| ENSP00000248450 | 4.96E-06 | -0.0809 | 0 | 0.663 |
| ENSP00000305790 | 1.91E-05 | -0.0809 | 401 | 0.897 |
| ENSP00000326869 | 6.44E-06 | -0.0810 | 0 | 0.168 |
| ENSP00000295095 | 9.99E-06 | -0.0810 | 0 | 0.264 |
| ENSP00000358456 | 5.11E-06 | -0.0810 | 0 | 0.000 |
| ENSP00000262866 | 1.53E-05 | -0.0810 | 0 | 0.238 |
| ENSP00000243045 | 1.59E-05 | -0.0810 | 0 | 0.204 |
| ENSP00000376792 | 6.16E-06 | -0.0810 | 0 | 0.207 |
| ENSP00000221232 | 4.23E-06 | -0.0810 | 0 | 0.545 |
| ENSP00000300896 | 2.58E-05 | -0.0810 | 183 | 0.060 |
| ENSP00000359603 | 2.27E-05 | -0.0810 | 527 | 0.649 |
| ENSP00000328690 | 1.48E-05 | -0.0810 | 396 | 0.911 |
| ENSP00000459962 | 2.81E-05 | -0.0810 | 814 | 0.000 |
| ENSP00000320130 | 6.35E-06 | -0.0810 | 0 | 0.116 |
| ENSP00000334188 | 2.02E-05 | -0.0810 | 277 | 0.626 |
| ENSP00000340610 | 7.78E-06 | -0.0811 | 202 | 0.657 |

| | | | | |
|---|---|---|---|---|
| ENSP00000343023 | 2.36E-05 | -0.0811 | 900 | 0.698 |
| ENSP00000200135 | 4.30E-06 | -0.0811 | 0 | 0.313 |
| ENSP00000265872 | 7.07E-06 | -0.0811 | 0 | 0.672 |
| ENSP00000303476 | 7.39E-06 | -0.0811 | 0 | 0.434 |
| ENSP00000301280 | 5.35E-06 | -0.0811 | 0 | 0.872 |
| ENSP00000233154 | 9.86E-06 | -0.0811 | 0 | 0.720 |
| ENSP00000230771 | 9.06E-06 | -0.0811 | 348 | 0.749 |
| ENSP00000083182 | 1.23E-05 | -0.0811 | 316 | 0.525 |
| ENSP00000305721 | 1.05E-05 | -0.0811 | 0 | 0.382 |
| ENSP00000273062 | 7.63E-06 | -0.0811 | 0 | 0.440 |
| ENSP00000364822 | 1.20E-07 | -0.0811 | 0 | 0.000 |
| ENSP00000341911 | 4.28E-05 | -0.0812 | 0 | 0.093 |
| ENSP00000278855 | 5.85E-06 | -0.0812 | 0 | 0.000 |
| ENSP00000311665 | 2.69E-05 | -0.0812 | 302 | 0.298 |
| ENSP00000319464 | 1.79E-05 | -0.0812 | 237 | 0.670 |
| ENSP00000469019 | 7.93E-07 | -0.0812 | 0 | 0.622 |
| ENSP00000302961 | 3.75E-05 | -0.0812 | 825 | 0.953 |
| ENSP00000215565 | 4.26E-06 | -0.0812 | 0 | 0.235 |
| ENSP00000382241 | 4.49E-06 | -0.0812 | 0 | 0.148 |
| ENSP00000298705 | 8.78E-06 | -0.0812 | 0 | 0.177 |
| ENSP00000240343 | 4.98E-06 | -0.0812 | 0 | 0.000 |
| ENSP00000392410 | 4.87E-06 | -0.0812 | 0 | 0.699 |
| ENSP00000255175 | 3.60E-05 | -0.0812 | 407 | 0.366 |
| ENSP00000409612 | 3.62E-05 | -0.0813 | 224 | 0.485 |
| ENSP00000362230 | 1.12E-05 | -0.0813 | 0 | 0.000 |
| ENSP00000078527 | 5.89E-06 | -0.0813 | 0 | 0.104 |
| ENSP00000314004 | 5.44E-06 | -0.0813 | 0 | 0.455 |
| ENSP00000252102 | 5.41E-06 | -0.0813 | 0 | 0.179 |
| ENSP00000316338 | 3.34E-05 | -0.0813 | 248 | 0.556 |
| ENSP00000260526 | 5.75E-06 | -0.0813 | 0 | 0.458 |
| ENSP00000264246 | 3.27E-05 | -0.0813 | 325 | 0.914 |
| ENSP00000251170 | 6.43E-06 | -0.0813 | 0 | 0.454 |
| ENSP00000375660 | 3.19E-06 | -0.0813 | 0 | 0.695 |
| ENSP00000407521 | 7.59E-07 | -0.0813 | 0 | 0.218 |
| ENSP00000297313 | 9.46E-06 | -0.0813 | 188 | 0.564 |
| ENSP00000305193 | 6.48E-05 | -0.0813 | 0 | 0.072 |
| ENSP00000422188 | 4.58E-06 | -0.0813 | 0 | 0.236 |
| ENSP00000170447 | 1.20E-05 | -0.0813 | 0 | 0.330 |
| ENSP00000315950 | 1.07E-05 | -0.0814 | 0 | 0.386 |
| ENSP00000355517 | 5.58E-06 | -0.0814 | 0 | 0.140 |
| ENSP00000262013 | 8.26E-06 | -0.0814 | 213 | 0.393 |
| ENSP00000381891 | 8.05E-05 | -0.0814 | 450 | 0.595 |
| ENSP00000392095 | 8.28E-06 | -0.0814 | 162 | 0.687 |
| ENSP00000281589 | 2.05E-05 | -0.0814 | 516 | 0.932 |
| ENSP00000335538 | 8.91E-06 | -0.0815 | 0 | 0.197 |
| ENSP00000437910 | 2.73E-06 | -0.0815 | 0 | 0.299 |

| | | | | |
|---|---|---|---|---|
| ENSP00000334100 | 5.64E-06 | -0.0815 | 0 | 0.312 |
| ENSP00000379496 | 1.78E-05 | -0.0815 | 0 | 0.261 |
| ENSP00000333836 | 2.00E-05 | -0.0815 | 272 | 0.366 |
| ENSP00000317578 | 9.46E-06 | -0.0815 | 273 | 0.000 |
| ENSP00000246841 | 5.09E-06 | -0.0815 | 0 | 0.448 |
| ENSP00000420854 | 2.03E-07 | -0.0815 | 0 | 0.202 |
| ENSP00000353058 | 3.39E-06 | -0.0815 | 0 | 0.693 |
| ENSP00000222673 | 9.50E-06 | -0.0815 | 235 | 0.542 |
| ENSP00000442795 | 1.34E-05 | -0.0816 | 900 | 0.273 |
| ENSP00000359804 | 6.52E-05 | -0.0816 | 205 | 0.666 |
| ENSP00000216862 | 1.78E-05 | -0.0816 | 150 | 0.560 |
| ENSP00000268053 | 3.22E-05 | -0.0816 | 227 | 0.659 |
| ENSP00000345175 | 5.09E-06 | -0.0816 | 0 | 0.653 |
| ENSP00000325017 | 5.02E-06 | -0.0816 | 0 | 0.502 |
| ENSP00000349873 | 1.05E-05 | -0.0816 | 0 | 0.000 |
| ENSP00000341680 | 8.60E-06 | -0.0816 | 202 | 0.318 |
| ENSP00000295713 | 4.73E-06 | -0.0816 | 0 | 0.000 |
| ENSP00000332887 | 9.02E-06 | -0.0816 | 202 | 0.207 |
| ENSP00000261881 | 4.12E-06 | -0.0816 | 0 | 0.666 |
| ENSP00000304364 | 1.70E-05 | -0.0816 | 209 | 0.168 |
| ENSP00000323889 | 1.07E-05 | -0.0816 | 163 | 0.545 |
| ENSP00000369816 | 3.16E-05 | -0.0817 | 502 | 0.772 |
| ENSP00000322159 | 3.25E-05 | -0.0817 | 502 | 0.319 |
| ENSP00000347717 | 3.00E-05 | -0.0817 | 358 | 0.466 |
| ENSP00000344758 | 2.32E-05 | -0.0817 | 480 | 0.664 |
| ENSP00000352920 | 5.97E-06 | -0.0817 | 0 | 0.495 |
| ENSP00000358454 | 8.72E-06 | -0.0817 | 0 | 0.000 |
| ENSP00000322885 | 1.09E-05 | -0.0817 | 0 | 0.416 |
| ENSP00000320658 | 1.20E-05 | -0.0817 | 315 | 0.597 |
| ENSP00000291634 | 5.20E-05 | -0.0817 | 0 | 0.349 |
| ENSP00000295888 | 2.19E-05 | -0.0817 | 293 | 0.281 |
| ENSP00000346450 | 8.07E-06 | -0.0817 | 0 | 0.000 |
| ENSP00000359707 | 2.86E-06 | -0.0817 | 0 | 0.184 |
| ENSP00000221307 | 1.87E-05 | -0.0817 | 250 | 0.331 |
| ENSP00000333934 | 4.03E-06 | -0.0818 | 0 | 0.778 |
| ENSP00000377225 | 5.62E-06 | -0.0818 | 0 | 0.526 |
| ENSP00000419370 | 1.86E-05 | -0.0818 | 211 | 0.445 |
| ENSP00000369625 | 2.31E-05 | -0.0818 | 231 | 0.376 |
| ENSP00000329757 | 5.99E-06 | -0.0818 | 0 | 0.365 |
| ENSP00000251775 | 5.62E-06 | -0.0818 | 0 | 0.143 |
| ENSP00000298307 | 5.53E-06 | -0.0818 | 0 | 0.300 |
| ENSP00000343515 | 1.74E-05 | -0.0818 | 168 | 0.876 |
| ENSP00000334040 | 3.99E-05 | -0.0818 | 0 | 0.077 |
| ENSP00000194130 | 4.87E-06 | -0.0818 | 0 | 0.064 |
| ENSP00000396052 | 9.42E-06 | -0.0818 | 263 | 0.761 |
| ENSP00000330054 | 5.16E-05 | -0.0819 | 422 | 0.802 |

| | | | | |
|---|---|---|---|---|
| ENSP00000286301 | 3.31E-05 | -0.0819 | 877 | 0.674 |
| ENSP00000252785 | 7.64E-06 | -0.0819 | 0 | 0.556 |
| ENSP00000306614 | 1.04E-05 | -0.0819 | 524 | 0.791 |
| ENSP00000306881 | 1.20E-05 | -0.0819 | 307 | 0.296 |
| ENSP00000259632 | 7.55E-06 | -0.0819 | 0 | 0.165 |
| ENSP00000309474 | 1.57E-05 | -0.0819 | 635 | 0.546 |
| ENSP00000310590 | 8.53E-06 | -0.0820 | 0 | 0.615 |
| ENSP00000339844 | 3.72E-06 | -0.0820 | 0 | 0.746 |
| ENSP00000267973 | 5.09E-06 | -0.0820 | 0 | 0.666 |
| ENSP00000356348 | 7.21E-06 | -0.0820 | 194 | 0.507 |
| ENSP00000209875 | 5.80E-06 | -0.0820 | 0 | 0.834 |
| ENSP00000283928 | 4.75E-06 | -0.0820 | 0 | 0.616 |
| ENSP00000261438 | 1.65E-05 | -0.0820 | 179 | 0.762 |
| ENSP00000338397 | 7.21E-06 | -0.0820 | 195 | 0.000 |
| ENSP00000382856 | 5.11E-06 | -0.0821 | 0 | 0.106 |
| ENSP00000389103 | 1.74E-05 | -0.0821 | 295 | 0.870 |
| ENSP00000257245 | 3.71E-06 | -0.0821 | 0 | 0.654 |
| ENSP00000266744 | 3.64E-05 | -0.0821 | 282 | 0.863 |
| ENSP00000313079 | 6.94E-06 | -0.0821 | 0 | 0.093 |
| ENSP00000310621 | 5.26E-06 | -0.0821 | 0 | 0.284 |
| ENSP00000368648 | 5.69E-06 | -0.0821 | 0 | 0.045 |
| ENSP00000227524 | 1.36E-05 | -0.0821 | 546 | 0.907 |
| ENSP00000312455 | 5.22E-05 | -0.0821 | 432 | 0.911 |
| ENSP00000308310 | 8.77E-06 | -0.0821 | 0 | 0.372 |
| ENSP00000289865 | 1.68E-05 | -0.0821 | 223 | 0.454 |
| ENSP00000331471 | 3.08E-05 | -0.0821 | 237 | 0.000 |
| ENSP00000247087 | 7.20E-05 | -0.0822 | 0 | 0.101 |
| ENSP00000435010 | 3.02E-07 | -0.0822 | 0 | 0.148 |
| ENSP00000471817 | 9.92E-07 | -0.0822 | 0 | 0.000 |
| ENSP00000345083 | 4.11E-05 | -0.0822 | 304 | 0.899 |
| ENSP00000351040 | 8.23E-02 | -0.0822 | 0 | 0.210 |
| ENSP00000322295 | 8.57E-06 | -0.0822 | 0 | 0.000 |
| ENSP00000361021 | 6.72E-05 | -0.0822 | 873 | 0.938 |
| ENSP00000327675 | 5.08E-06 | -0.0822 | 0 | 0.211 |
| ENSP00000318352 | 3.27E-05 | -0.0823 | 0 | 0.208 |
| ENSP00000219368 | 1.50E-05 | -0.0823 | 183 | 0.239 |
| ENSP00000342502 | 5.62E-06 | -0.0823 | 0 | 0.367 |
| ENSP00000273859 | 7.36E-06 | -0.0823 | 0 | 0.099 |
| ENSP00000265838 | 1.09E-05 | -0.0823 | 298 | 0.437 |
| ENSP00000294964 | 1.89E-05 | -0.0823 | 205 | 0.676 |
| ENSP00000242257 | 1.00E-05 | -0.0824 | 274 | 0.827 |
| ENSP00000244360 | 1.03E-05 | -0.0824 | 0 | 0.252 |
| ENSP00000259021 | 1.17E-05 | -0.0824 | 320 | 0.000 |
| ENSP00000371729 | 6.29E-06 | -0.0824 | 0 | 0.102 |
| ENSP00000236192 | 2.15E-05 | -0.0824 | 346 | 0.247 |
| ENSP00000385467 | 1.26E-07 | -0.0824 | 0 | 0.215 |

| | | | | |
|---|---|---|---|---|
| ENSP00000250244 | 5.23E-06 | -0.0824 | 0 | 0.179 |
| ENSP00000353104 | 5.08E-05 | -0.0824 | 291 | 0.585 |
| ENSP00000376010 | 5.01E-06 | -0.0824 | 0 | 0.709 |
| ENSP00000343674 | 2.10E-05 | -0.0824 | 652 | 0.081 |
| ENSP00000229379 | 3.98E-06 | -0.0824 | 0 | 0.383 |
| ENSP00000303279 | 1.15E-05 | -0.0824 | 317 | 0.000 |
| ENSP00000351939 | 3.27E-06 | -0.0825 | 0 | 0.692 |
| ENSP00000297282 | 5.72E-06 | -0.0825 | 0 | 0.062 |
| ENSP00000280904 | 1.68E-04 | -0.0825 | 225 | 0.304 |
| ENSP00000367001 | 1.42E-05 | -0.0825 | 364 | 0.134 |
| ENSP00000388421 | 3.98E-06 | -0.0825 | 0 | 0.000 |
| ENSP00000263207 | 5.60E-06 | -0.0825 | 0 | 0.603 |
| ENSP00000342071 | 7.00E-06 | -0.0825 | 177 | 0.000 |
| ENSP00000391101 | 2.52E-07 | -0.0825 | 0 | 0.121 |
| ENSP00000290943 | 8.54E-06 | -0.0825 | 202 | 0.642 |
| ENSP00000384515 | 7.56E-06 | -0.0825 | 0 | 0.333 |
| ENSP00000471094 | 8.32E-07 | -0.0825 | 0 | 0.000 |
| ENSP00000309262 | 7.27E-06 | -0.0826 | 0 | 0.248 |
| ENSP00000311977 | 5.84E-06 | -0.0826 | 0 | 0.734 |
| ENSP00000329189 | 6.16E-06 | -0.0826 | 0 | 0.499 |
| ENSP00000367263 | 9.93E-06 | -0.0826 | 163 | 0.500 |
| ENSP00000371307 | 6.19E-06 | -0.0826 | 0 | 0.000 |
| ENSP00000342667 | 2.94E-05 | -0.0826 | 271 | 0.399 |
| ENSP00000356405 | 2.47E-05 | -0.0826 | 283 | 0.816 |
| ENSP00000235090 | 5.09E-06 | -0.0826 | 0 | 0.637 |
| ENSP00000365397 | 6.40E-05 | -0.0826 | 314 | 0.000 |
| ENSP00000264156 | 8.59E-06 | -0.0827 | 176 | 0.782 |
| ENSP00000361784 | 2.76E-06 | -0.0827 | 0 | 0.689 |
| ENSP00000343765 | 9.09E-06 | -0.0827 | 0 | 0.306 |
| ENSP00000312675 | 9.11E-06 | -0.0827 | 0 | 0.345 |
| ENSP00000335651 | 7.92E-06 | -0.0827 | 0 | 0.260 |
| ENSP00000293275 | 1.95E-05 | -0.0827 | 216 | 0.000 |
| ENSP00000365147 | 1.05E-04 | -0.0827 | 198 | 0.215 |
| ENSP00000223366 | 9.78E-06 | -0.0827 | 349 | 0.675 |
| ENSP00000328182 | 8.37E-06 | -0.0827 | 0 | 0.093 |
| ENSP00000210060 | 1.28E-05 | -0.0827 | 240 | 0.377 |
| ENSP00000262462 | 1.22E-04 | -0.0827 | 287 | 0.558 |
| ENSP00000368140 | 7.68E-06 | -0.0827 | 197 | 0.330 |
| ENSP00000375820 | 3.32E-06 | -0.0828 | 0 | 0.694 |
| ENSP00000326888 | 2.45E-05 | -0.0828 | 0 | 0.180 |
| ENSP00000260952 | 5.21E-06 | -0.0828 | 0 | 0.354 |
| ENSP00000173898 | 5.80E-05 | -0.0828 | 361 | 0.735 |
| ENSP00000427463 | 8.11E-06 | -0.0828 | 189 | 0.491 |
| ENSP00000240327 | 5.32E-06 | -0.0828 | 0 | 0.686 |
| ENSP00000332123 | 1.88E-05 | -0.0828 | 164 | 0.000 |
| ENSP00000288139 | 1.13E-05 | -0.0828 | 217 | 0.529 |

| | | | | |
|---|---|---|---|---|
| ENSP00000368332 | 1.64E-05 | -0.0829 | 200 | 0.791 |
| ENSP00000318775 | 5.35E-06 | -0.0829 | 0 | 0.382 |
| ENSP00000342828 | 2.10E-05 | -0.0829 | 227 | 0.815 |
| ENSP00000331065 | 7.96E-06 | -0.0829 | 201 | 0.704 |
| ENSP00000253023 | 8.04E-06 | -0.0829 | 0 | 0.357 |
| ENSP00000262861 | 1.59E-05 | -0.0830 | 0 | 0.103 |
| ENSP00000271452 | 5.17E-06 | -0.0830 | 0 | 0.439 |
| ENSP00000302867 | 2.57E-06 | -0.0830 | 0 | 0.000 |
| ENSP00000321971 | 5.10E-06 | -0.0830 | 0 | 0.214 |
| ENSP00000267884 | 5.97E-06 | -0.0830 | 0 | 0.599 |
| ENSP00000305647 | 2.33E-05 | -0.0830 | 346 | 0.238 |
| ENSP00000353344 | 4.40E-05 | -0.0830 | 418 | 0.890 |
| ENSP00000320309 | 1.06E-05 | -0.0830 | 340 | 0.664 |
| ENSP00000325677 | 2.36E-05 | -0.0830 | 302 | 0.905 |
| ENSP00000305785 | 8.89E-06 | -0.0830 | 0 | 0.633 |
| ENSP00000372286 | 6.72E-07 | -0.0830 | 0 | 0.192 |
| ENSP00000272322 | 7.33E-06 | -0.0830 | 0 | 0.144 |
| ENSP00000312709 | 7.97E-06 | -0.0830 | 0 | 0.810 |
| ENSP00000281043 | 5.26E-05 | -0.0830 | 281 | 0.905 |
| ENSP00000278412 | 1.77E-05 | -0.0831 | 414 | 0.925 |
| ENSP00000221419 | 9.60E-05 | -0.0831 | 629 | 0.727 |
| ENSP00000345151 | 6.13E-06 | -0.0831 | 0 | 0.306 |
| ENSP00000323280 | 2.76E-05 | -0.0831 | 208 | 0.645 |
| ENSP00000339109 | 7.59E-06 | -0.0831 | 0 | 0.498 |
| ENSP00000366513 | 3.78E-05 | -0.0831 | 151 | 0.221 |
| ENSP00000268717 | 7.68E-06 | -0.0831 | 0 | 0.416 |
| ENSP00000339917 | 2.70E-05 | -0.0831 | 165 | 0.776 |
| ENSP00000329967 | 1.00E-05 | -0.0831 | 296 | 0.742 |
| ENSP00000288828 | 5.19E-06 | -0.0831 | 0 | 0.148 |
| ENSP00000296859 | 2.53E-05 | -0.0831 | 0 | 0.376 |
| ENSP00000260508 | 4.22E-06 | -0.0832 | 0 | 0.180 |
| ENSP00000382511 | 3.12E-06 | -0.0832 | 0 | 0.686 |
| ENSP00000373539 | 1.57E-05 | -0.0832 | 161 | 0.364 |
| ENSP00000340823 | 5.80E-06 | -0.0832 | 0 | 0.773 |
| ENSP00000336752 | 6.93E-06 | -0.0832 | 0 | 0.507 |
| ENSP00000451229 | 1.29E-07 | -0.0832 | 0 | 0.214 |
| ENSP00000399441 | 4.62E-06 | -0.0832 | 0 | 0.666 |
| ENSP00000406485 | 2.53E-05 | -0.0832 | 338 | 0.000 |
| ENSP00000297044 | 5.55E-06 | -0.0833 | 0 | 0.196 |
| ENSP00000359310 | 5.01E-04 | -0.0833 | 0 | 0.201 |
| ENSP00000330812 | 8.49E-06 | -0.0833 | 198 | 0.000 |
| ENSP00000276692 | 1.79E-05 | -0.0833 | 201 | 0.336 |
| ENSP00000349393 | 2.80E-05 | -0.0833 | 262 | 0.768 |
| ENSP00000275016 | 1.00E-05 | -0.0833 | 0 | 0.257 |
| ENSP00000264431 | 2.34E-05 | -0.0833 | 333 | 0.548 |
| ENSP00000359802 | 3.11E-06 | -0.0834 | 0 | 0.698 |

| | | | | |
|---|---|---|---|---|
| ENSP00000352248 | 7.86E-06 | -0.0834 | 0 | 0.089 |
| ENSP00000262375 | 1.18E-05 | -0.0834 | 920 | 0.496 |
| ENSP00000344411 | 1.20E-05 | -0.0834 | 426 | 0.428 |
| ENSP00000296424 | 8.04E-06 | -0.0834 | 0 | 0.228 |
| ENSP00000229824 | 3.98E-06 | -0.0834 | 0 | 0.246 |
| ENSP00000226105 | 2.15E-05 | -0.0834 | 0 | 0.088 |
| ENSP00000297990 | 4.85E-06 | -0.0834 | 0 | 0.869 |
| ENSP00000300954 | 2.15E-05 | -0.0834 | 467 | 0.633 |
| ENSP00000398619 | 1.40E-07 | -0.0834 | 0 | 0.200 |
| ENSP00000319979 | 1.70E-05 | -0.0834 | 224 | 0.397 |
| ENSP00000219302 | 1.21E-05 | -0.0834 | 315 | 0.636 |
| ENSP00000284951 | 1.21E-05 | -0.0835 | 299 | 0.509 |
| ENSP00000364133 | 6.61E-05 | -0.0835 | 909 | 0.947 |
| ENSP00000359634 | 5.11E-06 | -0.0835 | 0 | 0.196 |
| ENSP00000372361 | 4.31E-06 | -0.0835 | 0 | 0.105 |
| ENSP00000388684 | 6.25E-06 | -0.0835 | 0 | 0.117 |
| ENSP00000334474 | 5.66E-06 | -0.0835 | 0 | 0.774 |
| ENSP00000260985 | 4.00E-05 | -0.0835 | 330 | 0.727 |
| ENSP00000347755 | 6.99E-06 | -0.0835 | 0 | 0.686 |
| ENSP00000391490 | 2.44E-05 | -0.0835 | 396 | 0.071 |
| ENSP00000318966 | 6.31E-06 | -0.0835 | 0 | 0.783 |
| ENSP00000332284 | 8.25E-06 | -0.0835 | 0 | 0.263 |
| ENSP00000297689 | 1.13E-05 | -0.0835 | 0 | 0.821 |
| ENSP00000360730 | 7.77E-05 | -0.0835 | 214 | 0.652 |
| ENSP00000291547 | 6.03E-06 | -0.0835 | 0 | 0.785 |
| ENSP00000358518 | 8.77E-06 | -0.0835 | 0 | 0.111 |
| ENSP00000218032 | 1.43E-05 | -0.0835 | 195 | 0.810 |
| ENSP00000316924 | 1.19E-05 | -0.0836 | 183 | 0.425 |
| ENSP00000264867 | 5.59E-05 | -0.0836 | 224 | 0.861 |
| ENSP00000261396 | 4.65E-06 | -0.0836 | 0 | 0.514 |
| ENSP00000317636 | 5.03E-06 | -0.0836 | 0 | 0.735 |
| ENSP00000299218 | 9.03E-06 | -0.0836 | 0 | 0.000 |
| ENSP00000237889 | 7.73E-06 | -0.0836 | 0 | 0.203 |
| ENSP00000296326 | 1.80E-05 | -0.0836 | 0 | 0.104 |
| ENSP00000282516 | 6.63E-06 | -0.0837 | 0 | 0.763 |
| ENSP00000273482 | 4.40E-05 | -0.0837 | 265 | 0.605 |
| ENSP00000262966 | 5.39E-06 | -0.0837 | 0 | 0.417 |
| ENSP00000299166 | 5.89E-06 | -0.0837 | 0 | 0.000 |
| ENSP00000396936 | 1.04E-06 | -0.0837 | 0 | 0.000 |
| ENSP00000304670 | 1.27E-05 | -0.0837 | 255 | 0.180 |
| ENSP00000261439 | 6.42E-06 | -0.0837 | 0 | 0.210 |
| ENSP00000379157 | 7.61E-05 | -0.0838 | 300 | 0.712 |
| ENSP00000303015 | 9.31E-06 | -0.0838 | 0 | 0.738 |
| ENSP00000274773 | 8.00E-06 | -0.0838 | 0 | 0.257 |
| ENSP00000262320 | 3.75E-05 | -0.0838 | 607 | 0.949 |
| ENSP00000262306 | 5.50E-06 | -0.0838 | 0 | 0.578 |

| | | | | |
|---|---|---|---|---|
| ENSP00000296328 | 1.56E-05 | -0.0838 | 161 | 0.798 |
| ENSP00000330101 | 1.80E-05 | -0.0838 | 165 | 0.000 |
| ENSP00000218006 | 1.05E-05 | -0.0838 | 509 | 0.663 |
| ENSP00000370372 | 2.14E-05 | -0.0838 | 725 | 0.384 |
| ENSP00000308772 | 1.37E-05 | -0.0838 | 816 | 0.674 |
| ENSP00000327268 | 4.44E-06 | -0.0838 | 0 | 0.334 |
| ENSP00000373274 | 3.22E-06 | -0.0839 | 0 | 0.695 |
| ENSP00000347858 | 4.14E-05 | -0.0839 | 480 | 0.886 |
| ENSP00000317580 | 3.80E-05 | -0.0839 | 635 | 0.860 |
| ENSP00000364677 | 3.22E-06 | -0.0839 | 0 | 0.676 |
| ENSP00000340089 | 2.11E-05 | -0.0839 | 252 | 0.000 |
| ENSP00000452780 | 4.66E-05 | -0.0839 | 923 | 0.910 |
| ENSP00000284509 | 1.33E-05 | -0.0839 | 151 | 0.388 |
| ENSP00000209728 | 1.05E-05 | -0.0839 | 326 | 0.873 |
| ENSP00000259400 | 1.87E-05 | -0.0839 | 151 | 0.259 |
| ENSP00000331944 | 2.03E-05 | -0.0839 | 356 | 0.728 |
| ENSP00000217515 | 7.93E-06 | -0.0840 | 0 | 0.369 |
| ENSP00000249299 | 4.79E-06 | -0.0840 | 0 | 0.085 |
| ENSP00000352442 | 1.97E-05 | -0.0840 | 857 | 0.000 |
| ENSP00000298282 | 5.80E-06 | -0.0840 | 0 | 0.702 |
| ENSP00000332288 | 1.06E-05 | -0.0840 | 0 | 0.377 |
| ENSP00000372088 | 6.76E-05 | -0.0840 | 586 | 0.000 |
| ENSP00000267273 | 1.97E-05 | -0.0840 | 384 | 0.579 |
| ENSP00000383630 | 7.61E-06 | -0.0841 | 0 | 0.257 |
| ENSP00000313408 | 2.38E-05 | -0.0841 | 0 | 0.183 |
| ENSP00000283441 | 1.76E-05 | -0.0841 | 0 | 0.081 |
| ENSP00000237449 | 4.11E-06 | -0.0841 | 0 | 0.115 |
| ENSP00000366894 | 1.60E-05 | -0.0841 | 150 | 0.727 |
| ENSP00000293328 | 4.37E-05 | -0.0841 | 361 | 0.937 |
| ENSP00000394380 | 4.92E-06 | -0.0841 | 0 | 0.000 |
| ENSP00000251119 | 8.46E-06 | -0.0842 | 0 | 0.106 |
| ENSP00000269439 | 7.81E-06 | -0.0842 | 0 | 0.478 |
| ENSP00000386450 | 8.78E-07 | -0.0842 | 0 | 0.167 |
| ENSP00000407182 | 3.71E-05 | -0.0842 | 271 | 0.614 |
| ENSP00000354612 | 3.68E-05 | -0.0842 | 255 | 0.828 |
| ENSP00000359055 | 1.01E-05 | -0.0842 | 0 | 0.000 |
| ENSP00000344479 | 4.76E-05 | -0.0842 | 815 | 0.866 |
| ENSP00000326630 | 1.84E-05 | -0.0842 | 254 | 0.795 |
| ENSP00000164133 | 3.92E-06 | -0.0842 | 0 | 0.529 |
| ENSP00000290949 | 6.06E-06 | -0.0842 | 0 | 0.098 |
| ENSP00000358921 | 3.63E-05 | -0.0843 | 463 | 0.694 |
| ENSP00000281623 | 1.52E-04 | -0.0843 | 222 | 0.282 |
| ENSP00000343690 | 5.90E-06 | -0.0843 | 0 | 0.372 |
| ENSP00000307666 | 7.72E-06 | -0.0843 | 0 | 0.000 |
| ENSP00000293778 | 4.21E-05 | -0.0843 | 581 | 0.621 |
| ENSP00000327213 | 4.34E-05 | -0.0843 | 254 | 0.795 |

| | | | | |
|---|---|---|---|---|
| ENSP00000311273 | 4.03E-06 | -0.0843 | 0 | 0.110 |
| ENSP00000354964 | 4.26E-05 | -0.0843 | 0 | 0.171 |
| ENSP00000235180 | 7.15E-06 | -0.0843 | 0 | 0.228 |
| ENSP00000257829 | 8.76E-06 | -0.0843 | 169 | 0.879 |
| ENSP00000314946 | 1.37E-04 | -0.0843 | 179 | 0.281 |
| ENSP00000314508 | 2.29E-05 | -0.0843 | 196 | 0.696 |
| ENSP00000262965 | 3.79E-05 | -0.0844 | 235 | 0.863 |
| ENSP00000229268 | 6.02E-06 | -0.0844 | 0 | 0.315 |
| ENSP00000442373 | 3.99E-06 | -0.0844 | 0 | 0.096 |
| ENSP00000340523 | 1.77E-05 | -0.0844 | 252 | 0.341 |
| ENSP00000358022 | 4.00E-05 | -0.0844 | 442 | 0.940 |
| ENSP00000244230 | 4.13E-06 | -0.0844 | 0 | 0.874 |
| ENSP00000285199 | 1.79E-05 | -0.0844 | 229 | 0.275 |
| ENSP00000386773 | 2.89E-06 | -0.0844 | 0 | 0.000 |
| ENSP00000387081 | 2.89E-06 | -0.0844 | 0 | 0.000 |
| ENSP00000326219 | 6.13E-06 | -0.0844 | 0 | 0.266 |
| ENSP00000368924 | 6.00E-05 | -0.0845 | 242 | 0.000 |
| ENSP00000307525 | 1.64E-05 | -0.0845 | 188 | 0.916 |
| ENSP00000318914 | 9.79E-06 | -0.0845 | 0 | 0.277 |
| ENSP00000328835 | 2.42E-05 | -0.0845 | 171 | 0.833 |
| ENSP00000378118 | 1.57E-05 | -0.0845 | 263 | 0.672 |
| ENSP00000292853 | 9.50E-05 | -0.0845 | 150 | 0.146 |
| ENSP00000320563 | 7.23E-06 | -0.0845 | 0 | 0.075 |
| ENSP00000263270 | 5.71E-06 | -0.0845 | 0 | 0.226 |
| ENSP00000354504 | 2.72E-05 | -0.0845 | 158 | 0.056 |
| ENSP00000262395 | 6.85E-06 | -0.0845 | 0 | 0.796 |
| ENSP00000287482 | 7.89E-06 | -0.0845 | 0 | 0.153 |
| ENSP00000284551 | 7.32E-06 | -0.0846 | 0 | 0.314 |
| ENSP00000333181 | 8.99E-06 | -0.0846 | 205 | 0.205 |
| ENSP00000258538 | 1.86E-05 | -0.0846 | 0 | 0.116 |
| ENSP00000333982 | 7.26E-06 | -0.0846 | 0 | 0.344 |
| ENSP00000267425 | 6.15E-06 | -0.0846 | 0 | 0.848 |
| ENSP00000361927 | 7.71E-05 | -0.0846 | 231 | 0.673 |
| ENSP00000349882 | 2.68E-06 | -0.0846 | 0 | 0.691 |
| ENSP00000330485 | 1.67E-05 | -0.0846 | 0 | 0.055 |
| ENSP00000265605 | 8.71E-06 | -0.0846 | 216 | 0.289 |
| ENSP00000368152 | 2.25E-05 | -0.0846 | 201 | 0.089 |
| ENSP00000227451 | 1.05E-05 | -0.0846 | 0 | 0.257 |
| ENSP00000331369 | 6.89E-06 | -0.0846 | 0 | 0.134 |
| ENSP00000366679 | 1.48E-05 | -0.0847 | 0 | 0.994 |
| ENSP00000331288 | 4.89E-06 | -0.0847 | 0 | 0.520 |
| ENSP00000318057 | 2.26E-05 | -0.0847 | 197 | 0.870 |
| ENSP00000320893 | 9.32E-06 | -0.0847 | 202 | 0.663 |
| ENSP00000261835 | 1.00E-05 | -0.0847 | 0 | 0.227 |
| ENSP00000300190 | 9.27E-06 | -0.0848 | 0 | 0.177 |
| ENSP00000369293 | 3.25E-05 | -0.0848 | 292 | 0.878 |

| | | | | |
|---|---|---|---|---|
| ENSP00000383382 | 1.06E-05 | -0.0848 | 966 | 0.760 |
| ENSP00000417246 | 6.75E-07 | -0.0848 | 0 | 0.178 |
| ENSP00000265081 | 1.39E-05 | -0.0848 | 317 | 0.696 |
| ENSP00000323853 | 2.63E-06 | -0.0848 | 0 | 0.000 |
| ENSP00000456039 | 1.66E-07 | -0.0848 | 0 | 0.077 |
| ENSP00000299259 | 5.96E-06 | -0.0848 | 0 | 0.703 |
| ENSP00000380252 | 5.90E-05 | -0.0848 | 850 | 0.771 |
| ENSP00000265339 | 1.38E-05 | -0.0848 | 223 | 0.872 |
| ENSP00000173229 | 3.03E-05 | -0.0848 | 247 | 0.901 |
| ENSP00000218548 | 2.52E-05 | -0.0848 | 634 | 0.713 |
| ENSP00000268379 | 1.11E-05 | -0.0849 | 224 | 0.569 |
| ENSP00000382815 | 4.76E-06 | -0.0849 | 0 | 0.161 |
| ENSP00000354947 | 4.58E-05 | -0.0849 | 374 | 0.385 |
| ENSP00000334002 | 3.13E-05 | -0.0849 | 198 | 0.638 |
| ENSP00000300648 | 1.17E-05 | -0.0849 | 237 | 0.000 |
| ENSP00000325240 | 1.99E-05 | -0.0849 | 169 | 0.437 |
| ENSP00000302176 | 1.38E-05 | -0.0849 | 227 | 0.601 |
| ENSP00000338572 | 3.72E-06 | -0.0849 | 0 | 0.699 |
| ENSP00000326366 | 4.95E-05 | -0.0850 | 826 | 0.928 |
| ENSP00000370966 | 2.56E-05 | -0.0850 | 605 | 0.254 |
| ENSP00000248041 | 9.24E-06 | -0.0850 | 0 | 0.208 |
| ENSP00000296522 | 3.82E-05 | -0.0850 | 835 | 0.369 |
| ENSP00000293670 | 1.91E-05 | -0.0851 | 0 | 0.124 |
| ENSP00000420093 | 3.42E-06 | -0.0851 | 0 | 0.000 |
| ENSP00000335144 | 1.40E-05 | -0.0851 | 0 | 0.088 |
| ENSP00000285093 | 1.11E-05 | -0.0851 | 306 | 0.426 |
| ENSP00000043402 | 1.30E-05 | -0.0851 | 197 | 0.832 |
| ENSP00000269214 | 5.63E-06 | -0.0851 | 0 | 0.697 |
| ENSP00000382791 | 3.05E-05 | -0.0851 | 270 | 0.417 |
| ENSP00000351524 | 4.41E-05 | -0.0851 | 191 | 0.767 |
| ENSP00000354361 | 1.97E-05 | -0.0851 | 0 | 0.384 |
| ENSP00000257818 | 4.35E-05 | -0.0851 | 495 | 0.832 |
| ENSP00000350639 | 5.92E-05 | -0.0851 | 165 | 0.720 |
| ENSP00000334800 | 3.57E-06 | -0.0851 | 0 | 0.680 |
| ENSP00000274008 | 1.19E-05 | -0.0852 | 410 | 0.563 |
| ENSP00000283645 | 6.64E-06 | -0.0852 | 0 | 0.000 |
| ENSP00000264649 | 4.43E-06 | -0.0852 | 0 | 0.112 |
| ENSP00000386458 | 3.77E-05 | -0.0852 | 415 | 0.857 |
| ENSP00000300091 | 1.12E-04 | -0.0852 | 472 | 0.153 |
| ENSP00000397843 | 2.16E-05 | -0.0852 | 0 | 0.083 |
| ENSP00000373853 | 4.39E-06 | -0.0852 | 0 | 0.687 |
| ENSP00000257934 | 9.55E-06 | -0.0852 | 192 | 0.457 |
| ENSP00000351407 | 4.78E-05 | -0.0853 | 290 | 0.843 |
| ENSP00000320347 | 1.42E-05 | -0.0853 | 515 | 0.708 |
| ENSP00000356737 | 1.70E-04 | -0.0853 | 187 | 0.091 |
| ENSP00000310557 | 6.03E-06 | -0.0853 | 0 | 0.152 |

| | | | | |
|---|---|---|---|---|
| ENSP00000410769 | 1.97E-05 | -0.0853 | 301 | 0.931 |
| ENSP00000355459 | 2.81E-06 | -0.0853 | 0 | 0.696 |
| ENSP00000294189 | 4.92E-06 | -0.0853 | 0 | 0.777 |
| ENSP00000335304 | 1.30E-05 | -0.0853 | 353 | 0.562 |
| ENSP00000288235 | 1.70E-05 | -0.0853 | 244 | 0.476 |
| ENSP00000196551 | 6.72E-06 | -0.0853 | 391 | 0.827 |
| ENSP00000357047 | 5.45E-05 | -0.0854 | 307 | 0.770 |
| ENSP00000248566 | 6.75E-06 | -0.0854 | 0 | 0.619 |
| ENSP00000340009 | 4.65E-06 | -0.0854 | 0 | 0.386 |
| ENSP00000374357 | 1.00E-04 | -0.0854 | 256 | 0.813 |
| ENSP00000311280 | 1.33E-05 | -0.0854 | 0 | 0.825 |
| ENSP00000331912 | 4.51E-06 | -0.0854 | 0 | 0.119 |
| ENSP00000354305 | 5.33E-06 | -0.0854 | 0 | 0.673 |
| ENSP00000367832 | 4.57E-05 | -0.0854 | 414 | 0.822 |
| ENSP00000305071 | 1.13E-05 | -0.0854 | 211 | 0.703 |
| ENSP00000301072 | 3.74E-05 | -0.0854 | 343 | 0.656 |
| ENSP00000433998 | 8.10E-06 | -0.0855 | 0 | 0.129 |
| ENSP00000381339 | 3.95E-05 | -0.0855 | 952 | 0.612 |
| ENSP00000336552 | 6.15E-06 | -0.0855 | 0 | 0.115 |
| ENSP00000318480 | 2.18E-05 | -0.0855 | 459 | 0.758 |
| ENSP00000323184 | 2.94E-05 | -0.0855 | 0 | 0.094 |
| ENSP00000282111 | 1.40E-05 | -0.0855 | 197 | 0.851 |
| ENSP00000341885 | 1.62E-05 | -0.0855 | 479 | 0.829 |
| ENSP00000244571 | 1.12E-05 | -0.0855 | 192 | 0.667 |
| ENSP00000370839 | 1.81E-05 | -0.0855 | 250 | 0.157 |
| ENSP00000299424 | 4.08E-05 | -0.0855 | 250 | 0.882 |
| ENSP00000219252 | 8.49E-06 | -0.0856 | 379 | 0.817 |
| ENSP00000044462 | 9.86E-06 | -0.0856 | 164 | 0.594 |
| ENSP00000313059 | 6.29E-06 | -0.0856 | 0 | 0.273 |
| ENSP00000344674 | 7.04E-06 | -0.0856 | 0 | 0.264 |
| ENSP00000329697 | 5.79E-06 | -0.0856 | 0 | 0.000 |
| ENSP00000342886 | 1.50E-05 | -0.0856 | 852 | 0.954 |
| ENSP00000308258 | 9.75E-06 | -0.0856 | 0 | 0.127 |
| ENSP00000162749 | 3.37E-05 | -0.0856 | 521 | 0.933 |
| ENSP00000275699 | 1.39E-05 | -0.0856 | 307 | 0.583 |
| ENSP00000363308 | 2.44E-05 | -0.0856 | 391 | 0.748 |
| ENSP00000261517 | 6.15E-06 | -0.0856 | 0 | 0.146 |
| ENSP00000215375 | 3.81E-06 | -0.0856 | 0 | 0.477 |
| ENSP00000268042 | 7.89E-06 | -0.0856 | 0 | 0.115 |
| ENSP00000273353 | 1.86E-05 | -0.0857 | 244 | 0.371 |
| ENSP00000309457 | 4.85E-06 | -0.0857 | 0 | 0.117 |
| ENSP00000308895 | 1.45E-05 | -0.0857 | 0 | 0.076 |
| ENSP00000373454 | 2.22E-05 | -0.0857 | 309 | 0.000 |
| ENSP00000275605 | 1.32E-05 | -0.0857 | 158 | 0.311 |
| ENSP00000314499 | 2.59E-05 | -0.0858 | 563 | 0.698 |
| ENSP00000302100 | 3.53E-05 | -0.0858 | 361 | 0.851 |

| | | | | |
|---|---|---|---|---|
| ENSP00000222286 | 1.13E-05 | -0.0858 | 550 | 0.586 |
| ENSP00000397178 | 3.61E-06 | -0.0858 | 0 | 0.697 |
| ENSP00000336655 | 2.64E-05 | -0.0858 | 333 | 0.675 |
| ENSP00000362186 | 2.88E-05 | -0.0858 | 286 | 0.115 |
| ENSP00000340299 | 1.52E-05 | -0.0858 | 0 | 0.069 |
| ENSP00000238855 | 1.68E-05 | -0.0858 | 0 | 0.119 |
| ENSP00000309714 | 1.62E-05 | -0.0858 | 720 | 0.304 |
| ENSP00000329419 | 6.34E-06 | -0.0858 | 240 | 0.211 |
| ENSP00000264607 | 9.93E-06 | -0.0858 | 202 | 0.625 |
| ENSP00000229794 | 1.60E-05 | -0.0858 | 822 | 0.000 |
| ENSP00000343103 | 5.51E-06 | -0.0859 | 0 | 0.618 |
| ENSP00000366729 | 1.91E-05 | -0.0859 | 188 | 0.796 |
| ENSP00000274306 | 3.45E-05 | -0.0859 | 205 | 0.729 |
| ENSP00000304586 | 7.81E-06 | -0.0859 | 202 | 0.615 |
| ENSP00000391536 | 8.32E-06 | -0.0859 | 0 | 0.000 |
| ENSP00000248071 | 4.43E-05 | -0.0859 | 886 | 0.874 |
| ENSP00000333799 | 9.27E-06 | -0.0859 | 0 | 0.000 |
| ENSP00000253452 | 1.31E-05 | -0.0860 | 282 | 0.586 |
| ENSP00000351530 | 3.23E-06 | -0.0860 | 0 | 0.685 |
| ENSP00000308452 | 1.27E-05 | -0.0860 | 0 | 0.634 |
| ENSP00000238616 | 1.07E-05 | -0.0860 | 457 | 0.189 |
| ENSP00000348550 | 2.97E-05 | -0.0861 | 405 | 0.314 |
| ENSP00000358807 | 7.63E-06 | -0.0861 | 0 | 0.785 |
| ENSP00000347043 | 3.15E-06 | -0.0861 | 0 | 0.694 |
| ENSP00000264463 | 5.89E-06 | -0.0861 | 0 | 0.167 |
| ENSP00000309548 | 5.61E-06 | -0.0861 | 0 | 0.584 |
| ENSP00000295373 | 1.11E-05 | -0.0861 | 150 | 0.000 |
| ENSP00000287169 | 1.40E-05 | -0.0861 | 0 | 0.085 |
| ENSP00000233202 | 4.86E-05 | -0.0861 | 238 | 0.575 |
| ENSP00000281828 | 4.21E-06 | -0.0861 | 0 | 0.714 |
| ENSP00000366283 | 6.28E-06 | -0.0861 | 0 | 0.140 |
| ENSP00000308032 | 9.07E-06 | -0.0861 | 0 | 0.151 |
| ENSP00000297431 | 4.08E-06 | -0.0861 | 0 | 0.784 |
| ENSP00000402257 | 4.46E-05 | -0.0861 | 386 | 0.853 |
| ENSP00000373772 | 2.18E-05 | -0.0861 | 302 | 0.917 |
| ENSP00000402914 | 1.96E-06 | -0.0861 | 0 | 0.000 |
| ENSP00000252543 | 4.33E-06 | -0.0862 | 0 | 0.832 |
| ENSP00000318697 | 1.61E-05 | -0.0862 | 545 | 0.623 |
| ENSP00000420194 | 3.93E-05 | -0.0862 | 516 | 0.821 |
| ENSP00000368189 | 4.56E-06 | -0.0862 | 0 | 0.845 |
| ENSP00000403397 | 4.39E-02 | -0.0862 | 338 | 0.688 |
| ENSP00000260010 | 3.06E-05 | -0.0862 | 909 | 0.948 |
| ENSP00000357750 | 7.51E-05 | -0.0863 | 394 | 0.662 |
| ENSP00000269703 | 7.47E-06 | -0.0863 | 0 | 0.252 |
| ENSP00000294638 | 5.65E-06 | -0.0863 | 0 | 0.831 |
| ENSP00000384670 | 1.53E-05 | -0.0863 | 0 | 0.196 |

| | | | | |
|---|---|---|---|---|
| ENSP00000316772 | 5.34E-06 | -0.0863 | 0 | 0.362 |
| ENSP00000308782 | 1.49E-06 | -0.0863 | 0 | 0.288 |
| ENSP00000237455 | 6.54E-06 | -0.0864 | 0 | 0.390 |
| ENSP00000375656 | 3.21E-06 | -0.0864 | 0 | 0.000 |
| ENSP00000262033 | 1.36E-05 | -0.0864 | 346 | 0.628 |
| ENSP00000246802 | 4.98E-06 | -0.0864 | 0 | 0.828 |
| ENSP00000228347 | 9.59E-06 | -0.0864 | 381 | 0.805 |
| ENSP00000305648 | 3.91E-06 | -0.0864 | 0 | 0.253 |
| ENSP00000413418 | 4.55E-05 | -0.0864 | 239 | 0.266 |
| ENSP00000384716 | 1.01E-05 | -0.0864 | 211 | 0.176 |
| ENSP00000253577 | 7.47E-06 | -0.0864 | 0 | 0.113 |
| ENSP00000262607 | 4.37E-06 | -0.0864 | 0 | 0.610 |
| ENSP00000304713 | 6.95E-06 | -0.0864 | 0 | 0.120 |
| ENSP00000339467 | 2.88E-05 | -0.0865 | 239 | 0.564 |
| ENSP00000250937 | 6.33E-06 | -0.0865 | 0 | 0.834 |
| ENSP00000314214 | 2.33E-05 | -0.0865 | 346 | 0.301 |
| ENSP00000358622 | 4.30E-05 | -0.0865 | 298 | 0.000 |
| ENSP00000354707 | 4.15E-06 | -0.0865 | 0 | 0.129 |
| ENSP00000340004 | 1.41E-05 | -0.0865 | 211 | 0.704 |
| ENSP00000383333 | 5.96E-06 | -0.0865 | 0 | 0.282 |
| ENSP00000246194 | 5.93E-06 | -0.0865 | 0 | 0.971 |
| ENSP00000297012 | 1.65E-05 | -0.0866 | 158 | 0.990 |
| ENSP00000378418 | 3.91E-06 | -0.0866 | 0 | 0.720 |
| ENSP00000320869 | 7.42E-06 | -0.0866 | 0 | 0.415 |
| ENSP00000374552 | 4.64E-06 | -0.0866 | 0 | 0.210 |
| ENSP00000372272 | 2.16E-07 | -0.0866 | 0 | 0.203 |
| ENSP00000372278 | 2.16E-07 | -0.0866 | 0 | 0.203 |
| ENSP00000240499 | 1.81E-05 | -0.0866 | 197 | 0.777 |
| ENSP00000376148 | 7.94E-06 | -0.0866 | 0 | 0.089 |
| ENSP00000241600 | 7.78E-06 | -0.0866 | 416 | 0.735 |
| ENSP00000265080 | 6.18E-06 | -0.0866 | 0 | 0.465 |
| ENSP00000266557 | 4.13E-05 | -0.0867 | 163 | 0.823 |
| ENSP00000216774 | 7.10E-06 | -0.0867 | 424 | 0.680 |
| ENSP00000273153 | 8.78E-05 | -0.0867 | 150 | 0.140 |
| ENSP00000319417 | 1.39E-05 | -0.0867 | 0 | 0.230 |
| ENSP00000367471 | 7.38E-05 | -0.0867 | 0 | 0.100 |
| ENSP00000366557 | 2.57E-05 | -0.0867 | 564 | 0.729 |
| ENSP00000400997 | 3.35E-06 | -0.0867 | 0 | 0.708 |
| ENSP00000256646 | 2.58E-05 | -0.0867 | 904 | 0.910 |
| ENSP00000325038 | 1.36E-05 | -0.0867 | 211 | 0.690 |
| ENSP00000253571 | 7.38E-06 | -0.0867 | 0 | 0.606 |
| ENSP00000268835 | 9.01E-06 | -0.0867 | 201 | 0.578 |
| ENSP00000282412 | 1.08E-05 | -0.0867 | 317 | 0.676 |
| ENSP00000384018 | 4.83E-05 | -0.0867 | 300 | 0.822 |
| ENSP00000262640 | 1.77E-05 | -0.0867 | 201 | 0.242 |
| ENSP00000304102 | 1.65E-05 | -0.0867 | 192 | 0.517 |

| | | | | |
|---|---|---|---|---|
| ENSP00000295033 | 5.95E-06 | -0.0868 | 0 | 0.706 |
| ENSP00000352918 | 4.32E-05 | -0.0868 | 389 | 0.470 |
| ENSP00000305465 | 1.15E-05 | -0.0868 | 422 | 0.497 |
| ENSP00000346483 | 5.34E-06 | -0.0868 | 0 | 0.096 |
| ENSP00000325548 | 1.85E-05 | -0.0868 | 684 | 0.350 |
| ENSP00000359664 | 2.11E-04 | -0.0868 | 169 | 0.170 |
| ENSP00000306382 | 1.89E-05 | -0.0869 | 275 | 0.406 |
| ENSP00000250615 | 1.79E-05 | -0.0869 | 418 | 0.252 |
| ENSP00000400836 | 3.11E-06 | -0.0869 | 0 | 0.000 |
| ENSP00000263754 | 2.55E-05 | -0.0869 | 831 | 0.000 |
| ENSP00000306866 | 1.85E-05 | -0.0869 | 210 | 0.329 |
| ENSP00000361598 | 5.07E-03 | -0.0869 | 0 | 0.213 |
| ENSP00000219240 | 7.18E-06 | -0.0869 | 312 | 0.647 |
| ENSP00000342812 | 9.58E-06 | -0.0869 | 0 | 0.518 |
| ENSP00000270142 | 4.26E-05 | -0.0869 | 399 | 0.797 |
| ENSP00000349923 | 1.13E-05 | -0.0869 | 0 | 0.139 |
| ENSP00000264029 | 1.48E-05 | -0.0869 | 377 | 0.348 |
| ENSP00000256658 | 3.64E-06 | -0.0869 | 0 | 0.220 |
| ENSP00000373301 | 4.86E-05 | -0.0869 | 263 | 0.771 |
| ENSP00000279247 | 1.14E-05 | -0.0869 | 163 | 0.682 |
| ENSP00000368645 | 5.85E-06 | -0.0869 | 0 | 0.789 |
| ENSP00000263966 | 5.68E-06 | -0.0870 | 0 | 0.290 |
| ENSP00000300504 | 1.04E-05 | -0.0870 | 0 | 0.197 |
| ENSP00000295822 | 1.60E-05 | -0.0870 | 266 | 0.000 |
| ENSP00000268712 | 3.70E-05 | -0.0870 | 900 | 0.806 |
| ENSP00000261875 | 7.17E-06 | -0.0870 | 0 | 0.000 |
| ENSP00000215832 | 1.34E-05 | -0.0870 | 367 | 0.000 |
| ENSP00000217173 | 7.04E-06 | -0.0870 | 0 | 0.336 |
| ENSP00000308149 | 2.08E-05 | -0.0870 | 216 | 0.122 |
| ENSP00000366157 | 3.51E-05 | -0.0870 | 561 | 0.523 |
| ENSP00000266497 | 1.03E-05 | -0.0870 | 165 | 0.660 |
| ENSP00000312995 | 9.77E-06 | -0.0870 | 270 | 0.660 |
| ENSP00000298852 | 2.29E-05 | -0.0870 | 369 | 0.567 |
| ENSP00000298532 | 1.48E-05 | -0.0870 | 250 | 0.000 |
| ENSP00000368872 | 5.36E-06 | -0.0870 | 0 | 0.169 |
| ENSP00000357986 | 1.20E-04 | -0.0870 | 564 | 0.479 |
| ENSP00000337056 | 1.35E-05 | -0.0870 | 273 | 0.835 |
| ENSP00000340736 | 9.34E-06 | -0.0871 | 0 | 0.094 |
| ENSP00000272520 | 2.76E-05 | -0.0871 | 0 | 0.197 |
| ENSP00000356257 | 4.71E-05 | -0.0871 | 292 | 0.610 |
| ENSP00000222726 | 6.44E-05 | -0.0871 | 246 | 0.860 |
| ENSP00000264192 | 1.28E-05 | -0.0871 | 0 | 0.170 |
| ENSP00000239891 | 1.29E-05 | -0.0871 | 231 | 0.381 |
| ENSP00000234453 | 9.74E-06 | -0.0871 | 0 | 0.097 |
| ENSP00000247191 | 5.63E-06 | -0.0871 | 0 | 0.288 |
| ENSP00000258415 | 1.08E-05 | -0.0871 | 323 | 0.382 |

| | | | | |
|---|---|---|---|---|
| ENSP00000268383 | 7.37E-06 | -0.0871 | 0 | 0.284 |
| ENSP00000226444 | 1.83E-05 | -0.0871 | 300 | 0.382 |
| ENSP00000301921 | 1.78E-05 | -0.0872 | 181 | 0.819 |
| ENSP00000366509 | 4.49E-06 | -0.0872 | 0 | 0.701 |
| ENSP00000307078 | 4.00E-05 | -0.0872 | 359 | 0.472 |
| ENSP00000373691 | 4.03E-05 | -0.0872 | 343 | 0.738 |
| ENSP00000325312 | 1.12E-05 | -0.0872 | 265 | 0.860 |
| ENSP00000364190 | 4.35E-05 | -0.0872 | 165 | 0.842 |
| ENSP00000222330 | 1.16E-05 | -0.0872 | 343 | 0.686 |
| ENSP00000338788 | 6.57E-06 | -0.0872 | 179 | 0.844 |
| ENSP00000243896 | 1.27E-05 | -0.0873 | 0 | 0.067 |
| ENSP00000295454 | 6.89E-06 | -0.0873 | 0 | 0.337 |
| ENSP00000302913 | 7.03E-05 | -0.0873 | 225 | 0.600 |
| ENSP00000300441 | 7.05E-06 | -0.0873 | 0 | 0.246 |
| ENSP00000285208 | 1.71E-05 | -0.0873 | 196 | 0.229 |
| ENSP00000358155 | 9.21E-06 | -0.0873 | 158 | 0.000 |
| ENSP00000266000 | 3.87E-05 | -0.0873 | 270 | 0.853 |
| ENSP00000283558 | 1.42E-05 | -0.0873 | 155 | 0.000 |
| ENSP00000268802 | 4.03E-06 | -0.0874 | 0 | 0.864 |
| ENSP00000219406 | 9.26E-06 | -0.0874 | 724 | 0.563 |
| ENSP00000314348 | 1.63E-05 | -0.0874 | 404 | 0.926 |
| ENSP00000314505 | 4.42E-06 | -0.0874 | 0 | 0.687 |
| ENSP00000295901 | 1.79E-05 | -0.0874 | 188 | 0.446 |
| ENSP00000310721 | 7.48E-06 | -0.0874 | 0 | 0.269 |
| ENSP00000217939 | 1.34E-05 | -0.0874 | 159 | 0.651 |
| ENSP00000340465 | 1.57E-05 | -0.0874 | 152 | 0.082 |
| ENSP00000155840 | 9.40E-06 | -0.0874 | 213 | 0.540 |
| ENSP00000306627 | 6.42E-06 | -0.0874 | 0 | 0.181 |
| ENSP00000262139 | 5.47E-06 | -0.0875 | 0 | 0.107 |
| ENSP00000195654 | 6.15E-06 | -0.0875 | 0 | 0.080 |
| ENSP00000288840 | 8.04E-05 | -0.0875 | 665 | 0.882 |
| ENSP00000320898 | 7.82E-06 | -0.0875 | 0 | 0.725 |
| ENSP00000332151 | 2.26E-05 | -0.0875 | 349 | 0.331 |
| ENSP00000296861 | 4.14E-05 | -0.0875 | 163 | 0.749 |
| ENSP00000262077 | 6.74E-06 | -0.0875 | 0 | 0.710 |
| ENSP00000261537 | 3.33E-05 | -0.0875 | 416 | 0.855 |
| ENSP00000326534 | 5.88E-06 | -0.0875 | 0 | 0.164 |
| ENSP00000437812 | 5.42E-06 | -0.0875 | 0 | 0.189 |
| ENSP00000327214 | 1.20E-05 | -0.0875 | 418 | 0.336 |
| ENSP00000358151 | 2.20E-05 | -0.0875 | 885 | 0.953 |
| ENSP00000284629 | 6.22E-06 | -0.0876 | 202 | 0.625 |
| ENSP00000313490 | 1.34E-05 | -0.0876 | 428 | 0.562 |
| ENSP00000302456 | 1.51E-05 | -0.0876 | 324 | 0.754 |
| ENSP00000260402 | 6.96E-06 | -0.0876 | 208 | 0.692 |
| ENSP00000332225 | 7.98E-06 | -0.0876 | 0 | 0.541 |
| ENSP00000376886 | 3.85E-05 | -0.0876 | 389 | 0.836 |

| | | | | |
|---|---|---|---|---|
| ENSP00000228289 | 1.27E-05 | -0.0876 | 181 | 0.695 |
| ENSP00000216264 | 3.07E-05 | -0.0876 | 165 | 0.309 |
| ENSP00000351482 | 5.68E-06 | -0.0876 | 0 | 0.680 |
| ENSP00000307134 | 7.52E-06 | -0.0876 | 0 | 0.549 |
| ENSP00000352846 | 3.53E-06 | -0.0876 | 0 | 0.694 |
| ENSP00000360569 | 4.76E-05 | -0.0876 | 430 | 0.414 |
| ENSP00000416706 | 4.27E-05 | -0.0876 | 483 | 0.707 |
| ENSP00000303939 | 2.76E-05 | -0.0876 | 859 | 0.890 |
| ENSP00000233638 | 6.30E-05 | -0.0877 | 193 | 0.668 |
| ENSP00000267101 | 3.13E-05 | -0.0877 | 890 | 0.960 |
| ENSP00000281821 | 1.70E-05 | -0.0877 | 220 | 0.670 |
| ENSP00000254976 | 1.08E-05 | -0.0877 | 0 | 0.241 |
| ENSP00000292363 | 6.69E-06 | -0.0877 | 0 | 0.417 |
| ENSP00000400142 | 3.55E-05 | -0.0878 | 305 | 0.809 |
| ENSP00000437532 | 9.53E-08 | -0.0878 | 0 | 0.000 |
| ENSP00000339095 | 4.54E-06 | -0.0878 | 0 | 0.857 |
| ENSP00000424243 | 3.33E-05 | -0.0878 | 270 | 0.641 |
| ENSP00000402153 | 1.49E-06 | -0.0878 | 0 | 0.143 |
| ENSP00000386992 | 2.91E-05 | -0.0878 | 159 | 0.435 |
| ENSP00000350757 | 5.25E-05 | -0.0878 | 294 | 0.248 |
| ENSP00000370571 | 4.65E-05 | -0.0878 | 349 | 0.882 |
| ENSP00000276201 | 4.83E-06 | -0.0878 | 0 | 0.840 |
| ENSP00000451560 | 4.64E-05 | -0.0878 | 439 | 0.738 |
| ENSP00000283268 | 1.59E-05 | -0.0878 | 339 | 0.843 |
| ENSP00000259791 | 1.33E-05 | -0.0878 | 158 | 0.000 |
| ENSP00000288502 | 3.04E-03 | -0.0878 | 0 | 0.209 |
| ENSP00000335636 | 5.35E-06 | -0.0878 | 0 | 0.286 |
| ENSP00000240055 | 9.40E-06 | -0.0878 | 911 | 0.000 |
| ENSP00000264170 | 1.14E-05 | -0.0878 | 0 | 0.097 |
| ENSP00000418603 | 8.11E-06 | -0.0879 | 0 | 0.000 |
| ENSP00000292095 | 6.41E-05 | -0.0879 | 650 | 0.918 |
| ENSP00000216037 | 5.43E-05 | -0.0879 | 966 | 0.856 |
| ENSP00000276927 | 2.37E-05 | -0.0879 | 268 | 0.918 |
| ENSP00000355141 | 4.86E-05 | -0.0879 | 177 | 0.193 |
| ENSP00000315674 | 1.61E-05 | -0.0879 | 157 | 0.875 |
| ENSP00000270509 | 7.61E-05 | -0.0879 | 566 | 0.278 |
| ENSP00000305503 | 6.85E-06 | -0.0879 | 0 | 0.582 |
| ENSP00000350199 | 4.54E-06 | -0.0880 | 0 | 0.223 |
| ENSP00000309376 | 1.27E-04 | -0.0880 | 440 | 0.322 |
| ENSP00000380557 | 6.11E-06 | -0.0880 | 0 | 0.508 |
| ENSP00000367608 | 4.75E-05 | -0.0880 | 455 | 0.859 |
| ENSP00000342188 | 1.13E-05 | -0.0880 | 508 | 0.660 |
| ENSP00000266397 | 7.33E-06 | -0.0880 | 403 | 0.492 |
| ENSP00000279230 | 7.78E-06 | -0.0880 | 258 | 0.718 |
| ENSP00000359518 | 5.62E-05 | -0.0880 | 360 | 0.846 |
| ENSP00000223023 | 2.57E-05 | -0.0880 | 364 | 0.688 |

| | | | | |
|---|---|---|---|---|
| ENSP00000328640 | 2.17E-05 | -0.0880 | 0 | 0.000 |
| ENSP00000343479 | 1.32E-05 | -0.0880 | 200 | 0.462 |
| ENSP00000305494 | 9.40E-06 | -0.0880 | 294 | 0.484 |
| ENSP00000254190 | 5.91E-05 | -0.0880 | 159 | 0.168 |
| ENSP00000306397 | 1.01E-05 | -0.0881 | 210 | 0.483 |
| ENSP00000334145 | 9.87E-05 | -0.0881 | 316 | 0.840 |
| ENSP00000381105 | 4.61E-05 | -0.0881 | 702 | 0.853 |
| ENSP00000160827 | 3.27E-06 | -0.0881 | 0 | 0.256 |
| ENSP00000281456 | 1.01E-05 | -0.0881 | 225 | 0.453 |
| ENSP00000354006 | 1.71E-05 | -0.0881 | 210 | 0.388 |
| ENSP00000256079 | 1.01E-05 | -0.0881 | 563 | 0.000 |
| ENSP00000380174 | 3.15E-06 | -0.0882 | 0 | 0.701 |
| ENSP00000338703 | 7.52E-06 | -0.0882 | 0 | 0.344 |
| ENSP00000225298 | 3.47E-06 | -0.0882 | 0 | 0.883 |
| ENSP00000345339 | 3.11E-06 | -0.0882 | 0 | 0.692 |
| ENSP00000345392 | 3.71E-06 | -0.0882 | 0 | 0.000 |
| ENSP00000310129 | 1.04E-05 | -0.0882 | 178 | 0.604 |
| ENSP00000302501 | 1.03E-04 | -0.0882 | 609 | 0.880 |
| ENSP00000315659 | 1.11E-05 | -0.0883 | 241 | 0.156 |
| ENSP00000378642 | 3.67E-06 | -0.0883 | 0 | 0.697 |
| ENSP00000372210 | 2.55E-05 | -0.0883 | 428 | 0.232 |
| ENSP00000351883 | 3.33E-06 | -0.0883 | 0 | 0.688 |
| ENSP00000260766 | 5.74E-06 | -0.0883 | 0 | 0.648 |
| ENSP00000344989 | 6.35E-06 | -0.0884 | 363 | 0.597 |
| ENSP00000342848 | 1.12E-05 | -0.0884 | 151 | 0.422 |
| ENSP00000304762 | 6.02E-05 | -0.0884 | 0 | 0.174 |
| ENSP00000337445 | 5.13E-06 | -0.0884 | 0 | 0.753 |
| ENSP00000338030 | 7.19E-06 | -0.0884 | 0 | 0.063 |
| ENSP00000300935 | 3.51E-05 | -0.0884 | 463 | 0.443 |
| ENSP00000351589 | 1.11E-05 | -0.0885 | 0 | 0.996 |
| ENSP00000403304 | 2.05E-05 | -0.0885 | 153 | 0.000 |
| ENSP00000357079 | 1.59E-04 | -0.0885 | 198 | 0.000 |
| ENSP00000379213 | 3.68E-05 | -0.0885 | 859 | 0.867 |
| ENSP00000220429 | 1.49E-05 | -0.0885 | 201 | 0.719 |
| ENSP00000233710 | 7.35E-06 | -0.0885 | 0 | 0.474 |
| ENSP00000342962 | 1.70E-05 | -0.0885 | 199 | 0.127 |
| ENSP00000254193 | 1.34E-05 | -0.0885 | 393 | 0.880 |
| ENSP00000402065 | 5.77E-06 | -0.0885 | 0 | 0.000 |
| ENSP00000395369 | 9.02E-07 | -0.0885 | 0 | 0.000 |
| ENSP00000265138 | 8.20E-06 | -0.0885 | 0 | 0.097 |
| ENSP00000375904 | 3.49E-06 | -0.0885 | 0 | 0.100 |
| ENSP00000258646 | 7.16E-06 | -0.0885 | 0 | 0.715 |
| ENSP00000311344 | 4.90E-06 | -0.0886 | 0 | 0.549 |
| ENSP00000370990 | 1.38E-05 | -0.0886 | 286 | 0.776 |
| ENSP00000359364 | 6.09E-05 | -0.0886 | 200 | 0.195 |
| ENSP00000332152 | 7.56E-06 | -0.0886 | 0 | 0.000 |

| | | | | |
|---|---|---|---|---|
| ENSP00000314736 | 5.86E-06 | -0.0886 | 0 | 0.160 |
| ENSP00000267845 | 1.63E-05 | -0.0886 | 270 | 0.337 |
| ENSP00000242776 | 8.61E-06 | -0.0886 | 394 | 0.940 |
| ENSP00000265944 | 1.78E-05 | -0.0887 | 244 | 0.408 |
| ENSP00000284727 | 4.55E-06 | -0.0887 | 0 | 0.421 |
| ENSP00000352836 | 3.63E-06 | -0.0887 | 0 | 0.711 |
| ENSP00000328340 | 6.13E-06 | -0.0887 | 0 | 0.385 |
| ENSP00000317334 | 2.63E-05 | -0.0887 | 800 | 0.769 |
| ENSP00000300249 | 4.84E-06 | -0.0887 | 0 | 0.468 |
| ENSP00000324693 | 9.45E-06 | -0.0887 | 0 | 0.756 |
| ENSP00000286918 | 7.77E-06 | -0.0887 | 202 | 0.570 |
| ENSP00000225969 | 3.02E-06 | -0.0887 | 0 | 0.528 |
| ENSP00000309689 | 4.24E-06 | -0.0887 | 0 | 0.566 |
| ENSP00000254950 | 1.33E-05 | -0.0888 | 202 | 0.299 |
| ENSP00000263464 | 4.16E-05 | -0.0888 | 416 | 0.896 |
| ENSP00000310219 | 3.15E-05 | -0.0888 | 876 | 0.881 |
| ENSP00000205890 | 1.17E-05 | -0.0888 | 244 | 0.367 |
| ENSP00000263209 | 4.16E-05 | -0.0888 | 573 | 0.755 |
| ENSP00000220751 | 1.28E-05 | -0.0888 | 210 | 0.689 |
| ENSP00000364895 | 1.16E-05 | -0.0888 | 905 | 0.748 |
| ENSP00000350295 | 3.37E-06 | -0.0888 | 0 | 0.694 |
| ENSP00000330671 | 1.08E-05 | -0.0888 | 208 | 0.154 |
| ENSP00000222584 | 1.22E-05 | -0.0889 | 191 | 0.779 |
| ENSP00000353794 | 6.14E-06 | -0.0889 | 0 | 0.173 |
| ENSP00000359074 | 4.10E-05 | -0.0889 | 177 | 0.000 |
| ENSP00000382518 | 2.29E-05 | -0.0889 | 213 | 0.181 |
| ENSP00000267584 | 4.63E-06 | -0.0889 | 0 | 0.272 |
| ENSP00000357051 | 5.25E-05 | -0.0889 | 200 | 0.201 |
| ENSP00000263734 | 4.91E-05 | -0.0889 | 438 | 0.879 |
| ENSP00000355361 | 5.53E-05 | -0.0889 | 803 | 0.666 |
| ENSP00000350049 | 5.25E-06 | -0.0889 | 0 | 0.663 |
| ENSP00000315505 | 1.22E-05 | -0.0889 | 288 | 0.701 |
| ENSP00000215956 | 6.25E-06 | -0.0890 | 172 | 0.000 |
| ENSP00000316240 | 5.41E-05 | -0.0890 | 609 | 0.093 |
| ENSP00000228606 | 1.51E-05 | -0.0890 | 158 | 0.645 |
| ENSP00000407353 | 4.50E-06 | -0.0890 | 0 | 0.000 |
| ENSP00000328720 | 1.74E-05 | -0.0890 | 543 | 0.000 |
| ENSP00000256474 | 6.37E-05 | -0.0890 | 992 | 0.872 |
| ENSP00000346453 | 2.00E-05 | -0.0890 | 298 | 0.691 |
| ENSP00000263228 | 1.14E-05 | -0.0890 | 178 | 0.661 |
| ENSP00000261900 | 4.70E-06 | -0.0890 | 0 | 0.801 |
| ENSP00000265602 | 3.51E-05 | -0.0890 | 440 | 0.342 |
| ENSP00000262374 | 4.06E-06 | -0.0890 | 0 | 0.402 |
| ENSP00000318165 | 2.73E-05 | -0.0890 | 990 | 0.406 |
| ENSP00000344235 | 1.70E-05 | -0.0890 | 202 | 0.657 |
| ENSP00000324792 | 1.70E-05 | -0.0890 | 281 | 0.875 |

| | | | | |
|---|---|---|---|---|
| ENSP00000287078 | 8.94E-06 | -0.0891 | 340 | 0.329 |
| ENSP00000309565 | 5.27E-06 | -0.0891 | 0 | 0.278 |
| ENSP00000334675 | 2.26E-05 | -0.0891 | 284 | 0.913 |
| ENSP00000320188 | 1.35E-05 | -0.0891 | 195 | 0.689 |
| ENSP00000261015 | 8.95E-06 | -0.0891 | 343 | 0.888 |
| ENSP00000381928 | 1.04E-05 | -0.0891 | 0 | 0.097 |
| ENSP00000261601 | 1.96E-05 | -0.0891 | 163 | 0.514 |
| ENSP00000274811 | 6.38E-06 | -0.0891 | 0 | 0.280 |
| ENSP00000274289 | 1.76E-05 | -0.0891 | 201 | 0.564 |
| ENSP00000306682 | 1.88E-05 | -0.0891 | 177 | 0.790 |
| ENSP00000261708 | 3.54E-06 | -0.0891 | 0 | 0.852 |
| ENSP00000357565 | 3.46E-06 | -0.0891 | 0 | 0.185 |
| ENSP00000313600 | 6.83E-06 | -0.0892 | 0 | 0.095 |
| ENSP00000263257 | 4.42E-05 | -0.0892 | 181 | 0.602 |
| ENSP00000371789 | 2.03E-03 | -0.0892 | 0 | 0.000 |
| ENSP00000007414 | 6.28E-06 | -0.0892 | 0 | 0.086 |
| ENSP00000222250 | 5.45E-06 | -0.0892 | 0 | 0.076 |
| ENSP00000360262 | 2.70E-05 | -0.0892 | 228 | 0.115 |
| ENSP00000322457 | 7.81E-06 | -0.0892 | 0 | 0.738 |
| ENSP00000251047 | 7.05E-05 | -0.0892 | 493 | 0.220 |
| ENSP00000322652 | 1.01E-04 | -0.0892 | 193 | 0.079 |
| ENSP00000354568 | 2.35E-05 | -0.0892 | 246 | 0.933 |
| ENSP00000398401 | 4.20E-06 | -0.0893 | 0 | 0.141 |
| ENSP00000424598 | 4.80E-07 | -0.0893 | 0 | 0.135 |
| ENSP00000266646 | 3.25E-05 | -0.0893 | 214 | 0.854 |
| ENSP00000320378 | 7.31E-06 | -0.0893 | 0 | 0.081 |
| ENSP00000359258 | 3.85E-05 | -0.0893 | 903 | 0.427 |
| ENSP00000261574 | 4.49E-05 | -0.0893 | 349 | 0.836 |
| ENSP00000391826 | 7.29E-05 | -0.0893 | 929 | 0.947 |
| ENSP00000222157 | 2.97E-05 | -0.0894 | 242 | 0.872 |
| ENSP00000295777 | 4.79E-05 | -0.0894 | 300 | 0.289 |
| ENSP00000296792 | 3.93E-06 | -0.0894 | 0 | 0.856 |
| ENSP00000311492 | 1.24E-05 | -0.0894 | 0 | 0.691 |
| ENSP00000228740 | 6.59E-05 | -0.0894 | 506 | 0.441 |
| ENSP00000323047 | 2.28E-05 | -0.0894 | 240 | 0.000 |
| ENSP00000261733 | 9.04E-06 | -0.0894 | 323 | 0.466 |
| ENSP00000395340 | 3.28E-07 | -0.0894 | 0 | 0.214 |
| ENSP00000264377 | 4.96E-05 | -0.0894 | 151 | 0.201 |
| ENSP00000254436 | 7.66E-06 | -0.0894 | 163 | 0.550 |
| ENSP00000244426 | 7.73E-06 | -0.0894 | 0 | 0.136 |
| ENSP00000371800 | 5.76E-06 | -0.0894 | 0 | 0.474 |
| ENSP00000352257 | 4.16E-05 | -0.0895 | 997 | 0.868 |
| ENSP00000345079 | 3.25E-05 | -0.0895 | 440 | 0.304 |
| ENSP00000319664 | 7.82E-06 | -0.0895 | 0 | 0.319 |
| ENSP00000261712 | 5.32E-06 | -0.0895 | 0 | 0.541 |
| ENSP00000232975 | 8.93E-06 | -0.0895 | 906 | 0.658 |

| | | | | |
|---|---|---|---|---|
| ENSP00000396320 | 9.41E-06 | -0.0895 | 0 | 0.409 |
| ENSP00000325817 | 9.77E-06 | -0.0895 | 224 | 0.000 |
| ENSP00000357881 | 3.39E-06 | -0.0895 | 0 | 0.695 |
| ENSP00000365806 | 2.59E-05 | -0.0895 | 240 | 0.883 |
| ENSP00000363284 | 2.12E-05 | -0.0896 | 218 | 0.904 |
| ENSP00000385720 | 1.65E-05 | -0.0896 | 214 | 0.804 |
| ENSP00000317376 | 9.83E-06 | -0.0896 | 363 | 0.705 |
| ENSP00000366915 | 3.59E-05 | -0.0896 | 522 | 0.843 |
| ENSP00000355304 | 5.18E-06 | -0.0896 | 0 | 0.554 |
| ENSP00000248139 | 3.63E-06 | -0.0896 | 0 | 0.243 |
| ENSP00000313811 | 1.51E-05 | -0.0896 | 159 | 0.176 |
| ENSP00000296525 | 7.83E-06 | -0.0896 | 206 | 0.685 |
| ENSP00000271308 | 1.08E-05 | -0.0896 | 174 | 0.548 |
| ENSP00000264954 | 7.58E-06 | -0.0897 | 987 | 0.632 |
| ENSP00000330307 | 1.23E-05 | -0.0897 | 158 | 0.000 |
| ENSP00000265643 | 2.67E-05 | -0.0897 | 342 | 0.716 |
| ENSP00000310440 | 6.55E-06 | -0.0897 | 0 | 0.229 |
| ENSP00000356630 | 4.63E-05 | -0.0897 | 305 | 0.195 |
| ENSP00000350896 | 3.37E-05 | -0.0897 | 309 | 0.675 |
| ENSP00000310071 | 5.18E-06 | -0.0897 | 0 | 0.446 |
| ENSP00000357736 | 1.14E-05 | -0.0897 | 0 | 0.161 |
| ENSP00000341382 | 1.58E-05 | -0.0897 | 180 | 0.782 |
| ENSP00000264926 | 6.94E-06 | -0.0898 | 0 | 0.784 |
| ENSP00000302189 | 3.18E-05 | -0.0898 | 200 | 0.835 |
| ENSP00000280701 | 6.30E-06 | -0.0898 | 0 | 0.315 |
| ENSP00000263665 | 2.75E-05 | -0.0898 | 224 | 0.672 |
| ENSP00000295379 | 1.95E-05 | -0.0898 | 181 | 0.893 |
| ENSP00000369592 | 1.45E-06 | -0.0898 | 0 | 0.342 |
| ENSP00000439660 | 2.59E-05 | -0.0898 | 751 | 0.000 |
| ENSP00000358458 | 4.97E-05 | -0.0899 | 150 | 0.129 |
| ENSP00000221399 | 5.37E-05 | -0.0899 | 328 | 0.274 |
| ENSP00000372720 | 2.25E-04 | -0.0899 | 216 | 0.104 |
| ENSP00000333329 | 4.69E-06 | -0.0899 | 0 | 0.598 |
| ENSP00000393333 | 2.38E-05 | -0.0899 | 471 | 0.689 |
| ENSP00000296504 | 1.43E-05 | -0.0899 | 0 | 0.771 |
| ENSP00000281154 | 1.40E-05 | -0.0899 | 214 | 0.355 |
| ENSP00000452773 | 1.69E-06 | -0.0899 | 0 | 0.124 |
| ENSP00000303709 | 1.72E-05 | -0.0900 | 185 | 0.625 |
| ENSP00000381549 | 4.15E-05 | -0.0900 | 203 | 0.143 |
| ENSP00000391266 | 2.04E-05 | -0.0900 | 281 | 0.000 |
| ENSP00000364140 | 5.39E-05 | -0.0900 | 263 | 0.415 |
| ENSP00000369853 | 5.15E-05 | -0.0900 | 212 | 0.104 |
| ENSP00000321389 | 1.43E-05 | -0.0900 | 158 | 0.990 |
| ENSP00000337393 | 6.85E-06 | -0.0900 | 0 | 0.422 |
| ENSP00000325917 | 3.15E-05 | -0.0900 | 737 | 0.642 |
| ENSP00000301327 | 5.83E-06 | -0.0901 | 0 | 0.103 |

| | | | | |
|---|---|---|---|---|
| ENSP00000358548 | 5.15E-05 | -0.0901 | 910 | 0.735 |
| ENSP00000329825 | 2.48E-06 | -0.0901 | 0 | 0.214 |
| ENSP00000342121 | 6.76E-06 | -0.0901 | 0 | 0.567 |
| ENSP00000359483 | 5.08E-06 | -0.0901 | 0 | 0.652 |
| ENSP00000366899 | 4.05E-06 | -0.0901 | 0 | 0.690 |
| ENSP00000261837 | 3.15E-05 | -0.0901 | 220 | 0.492 |
| ENSP00000318423 | 1.32E-04 | -0.0901 | 210 | 0.000 |
| ENSP00000314193 | 3.63E-06 | -0.0901 | 0 | 0.821 |
| ENSP00000362107 | 8.94E-06 | -0.0901 | 270 | 0.560 |
| ENSP00000366317 | 8.40E-04 | -0.0902 | 0 | 0.000 |
| ENSP00000373828 | 3.79E-06 | -0.0902 | 0 | 0.133 |
| ENSP00000381129 | 1.85E-05 | -0.0902 | 360 | 0.492 |
| ENSP00000391249 | 1.81E-05 | -0.0902 | 265 | 0.423 |
| ENSP00000262367 | 2.84E-05 | -0.0902 | 921 | 0.000 |
| ENSP00000226319 | 5.41E-06 | -0.0902 | 0 | 0.000 |
| ENSP00000229903 | 6.29E-04 | -0.0902 | 0 | 0.209 |
| ENSP00000332790 | 1.05E-05 | -0.0902 | 158 | 0.993 |
| ENSP00000364702 | 6.54E-06 | -0.0902 | 0 | 0.000 |
| ENSP00000259239 | 4.99E-06 | -0.0903 | 0 | 0.885 |
| ENSP00000263116 | 1.76E-05 | -0.0903 | 196 | 0.240 |
| ENSP00000377582 | 1.37E-06 | -0.0903 | 0 | 0.151 |
| ENSP00000360913 | 5.15E-05 | -0.0903 | 241 | 0.154 |
| ENSP00000347055 | 2.20E-05 | -0.0903 | 907 | 0.447 |
| ENSP00000235521 | 1.26E-05 | -0.0903 | 271 | 0.541 |
| ENSP00000322640 | 2.48E-06 | -0.0903 | 0 | 0.214 |
| ENSP00000285679 | 4.89E-06 | -0.0903 | 0 | 0.253 |
| ENSP00000240285 | 9.02E-05 | -0.0903 | 958 | 0.206 |
| ENSP00000377446 | 3.01E-05 | -0.0903 | 259 | 0.691 |
| ENSP00000323811 | 1.33E-05 | -0.0903 | 229 | 0.385 |
| ENSP00000321449 | 3.93E-06 | -0.0903 | 0 | 0.829 |
| ENSP00000221543 | 8.09E-06 | -0.0903 | 0 | 0.186 |
| ENSP00000469038 | 2.41E-07 | -0.0903 | 0 | 0.000 |
| ENSP00000216036 | 8.18E-06 | -0.0903 | 0 | 0.000 |
| ENSP00000381711 | 1.72E-06 | -0.0904 | 0 | 0.000 |
| ENSP00000370414 | 2.00E-05 | -0.0904 | 209 | 0.157 |
| ENSP00000343745 | 3.56E-05 | -0.0904 | 825 | 0.758 |
| ENSP00000294785 | 7.36E-05 | -0.0904 | 502 | 0.684 |
| ENSP00000369927 | 8.12E-06 | -0.0904 | 243 | 0.247 |
| ENSP00000359504 | 3.40E-05 | -0.0904 | 911 | 0.691 |
| ENSP00000353874 | 2.68E-05 | -0.0904 | 873 | 0.906 |
| ENSP00000288532 | 7.43E-06 | -0.0904 | 0 | 0.262 |
| ENSP00000358770 | 3.49E-05 | -0.0904 | 386 | 0.752 |
| ENSP00000333394 | 9.71E-04 | -0.0904 | 0 | 0.211 |
| ENSP00000257566 | 1.89E-05 | -0.0904 | 192 | 0.875 |
| ENSP00000368396 | 6.27E-04 | -0.0904 | 0 | 0.000 |
| ENSP00000341208 | 4.25E-05 | -0.0904 | 364 | 0.944 |

| | | | | |
|---|---|---|---|---|
| ENSP00000442163 | 2.67E-04 | -0.0904 | 211 | 0.000 |
| ENSP00000361001 | 4.86E-05 | -0.0905 | 0 | 0.129 |
| ENSP00000221801 | 2.40E-05 | -0.0905 | 374 | 0.914 |
| ENSP00000337915 | 1.61E-05 | -0.0905 | 242 | 0.446 |
| ENSP00000391806 | 6.13E-06 | -0.0905 | 160 | 0.380 |
| ENSP00000353646 | 6.22E-06 | -0.0905 | 0 | 0.213 |
| ENSP00000355319 | 6.36E-04 | -0.0905 | 0 | 0.000 |
| ENSP00000261461 | 3.93E-06 | -0.0905 | 0 | 0.642 |
| ENSP00000381204 | 1.72E-04 | -0.0905 | 0 | 0.169 |
| ENSP00000344456 | 6.46E-05 | -0.0905 | 957 | 0.000 |
| ENSP00000246957 | 8.63E-06 | -0.0905 | 929 | 0.690 |
| ENSP00000225655 | 4.06E-05 | -0.0905 | 213 | 0.535 |
| ENSP00000379394 | 3.28E-06 | -0.0905 | 0 | 0.100 |
| ENSP00000412237 | 4.80E-05 | -0.0905 | 923 | 0.966 |
| ENSP00000371221 | 5.54E-05 | -0.0905 | 200 | 0.133 |
| ENSP00000290551 | 9.27E-05 | -0.0906 | 281 | 0.775 |
| ENSP00000297063 | 8.23E-02 | -0.0906 | 0 | 0.210 |
| ENSP00000350260 | 8.23E-02 | -0.0906 | 0 | 0.210 |
| ENSP00000385269 | 1.69E-01 | -0.0906 | 407 | 0.770 |
| ENSP00000262662 | 8.79E-06 | -0.0906 | 202 | 0.851 |
| ENSP00000381672 | 1.99E-03 | -0.0906 | 0 | 0.191 |
| ENSP00000346981 | 8.53E-04 | -0.0906 | 0 | 0.210 |
| ENSP00000304604 | 1.11E-05 | -0.0906 | 159 | 0.550 |
| ENSP00000261667 | 1.37E-05 | -0.0906 | 154 | 0.709 |
| ENSP00000332407 | 7.61E-06 | -0.0906 | 198 | 0.282 |
| ENSP00000380116 | 2.96E-04 | -0.0906 | 0 | 0.000 |
| ENSP00000291572 | 4.97E-06 | -0.0906 | 0 | 0.303 |
| ENSP00000371061 | 3.66E-04 | -0.0906 | 0 | 0.143 |
| ENSP00000348043 | 1.17E-05 | -0.0906 | 448 | 0.069 |
| ENSP00000273596 | 5.39E-06 | -0.0907 | 0 | 0.099 |
| ENSP00000364943 | 5.64E-05 | -0.0907 | 207 | 0.588 |
| ENSP00000306906 | 6.66E-06 | -0.0907 | 0 | 0.336 |
| ENSP00000367755 | 3.15E-05 | -0.0907 | 353 | 0.465 |
| ENSP00000324203 | 5.93E-06 | -0.0907 | 0 | 0.573 |
| ENSP00000369431 | 1.04E-04 | -0.0907 | 0 | 0.148 |
| ENSP00000216127 | 9.54E-06 | -0.0907 | 470 | 0.607 |
| ENSP00000265316 | 8.20E-06 | -0.0907 | 0 | 0.127 |
| ENSP00000349687 | 5.20E-05 | -0.0907 | 215 | 0.160 |
| ENSP00000343526 | 5.60E-06 | -0.0907 | 0 | 0.598 |
| ENSP00000363441 | 1.38E-04 | -0.0907 | 0 | 0.181 |
| ENSP00000383866 | 1.92E-04 | -0.0907 | 0 | 0.130 |
| ENSP00000343505 | 1.30E-04 | -0.0907 | 0 | 0.129 |
| ENSP00000323511 | 5.69E-06 | -0.0907 | 0 | 0.372 |
| ENSP00000398495 | 1.18E-04 | -0.0908 | 0 | 0.672 |
| ENSP00000363836 | 3.44E-05 | -0.0908 | 232 | 0.403 |
| ENSP00000374260 | 1.25E-06 | -0.0908 | 0 | 0.124 |

| | | | | |
|---|---|---|---|---|
| ENSP00000366843 | 6.00E-05 | -0.0908 | 292 | 0.606 |
| ENSP00000348611 | 2.51E-05 | -0.0908 | 235 | 0.000 |
| ENSP00000355051 | 5.52E-06 | -0.0908 | 0 | 0.734 |
| ENSP00000383515 | 6.68E-07 | -0.0908 | 0 | 0.000 |
| ENSP00000261318 | 2.67E-04 | -0.0908 | 0 | 0.142 |
| ENSP00000339912 | 4.58E-06 | -0.0908 | 0 | 0.566 |
| ENSP00000344218 | 1.21E-04 | -0.0908 | 0 | 0.081 |
| ENSP00000311336 | 1.17E-05 | -0.0908 | 0 | 0.059 |
| ENSP00000368405 | 1.30E-04 | -0.0908 | 0 | 0.077 |
| ENSP00000269243 | 1.57E-05 | -0.0908 | 644 | 0.537 |
| ENSP00000397157 | 1.01E-04 | -0.0908 | 167 | 0.579 |
| ENSP00000377461 | 3.49E-06 | -0.0908 | 0 | 0.703 |
| ENSP00000362372 | 6.32E-06 | -0.0909 | 0 | 0.488 |
| ENSP00000462879 | 1.04E-05 | -0.0909 | 0 | 0.176 |
| ENSP00000301740 | 6.12E-05 | -0.0909 | 591 | 0.775 |
| ENSP00000369636 | 3.47E-05 | -0.0909 | 0 | 0.118 |
| ENSP00000340083 | 2.16E-04 | -0.0909 | 0 | 0.163 |
| ENSP00000346206 | 6.15E-06 | -0.0909 | 160 | 0.428 |
| ENSP00000355979 | 1.23E-04 | -0.0909 | 0 | 0.124 |
| ENSP00000364028 | 4.05E-05 | -0.0909 | 471 | 0.703 |
| ENSP00000310729 | 1.27E-04 | -0.0909 | 0 | 0.123 |
| ENSP00000226207 | 1.21E-05 | -0.0909 | 434 | 0.580 |
| ENSP00000382231 | 7.95E-05 | -0.0909 | 0 | 0.000 |
| ENSP00000256544 | 2.31E-04 | -0.0909 | 0 | 0.138 |
| ENSP00000332433 | 1.35E-05 | -0.0909 | 288 | 0.704 |
| ENSP00000352928 | 6.02E-05 | -0.0909 | 628 | 0.167 |
| ENSP00000332721 | 6.36E-05 | -0.0910 | 0 | 0.121 |
| ENSP00000364382 | 6.98E-05 | -0.0910 | 0 | 0.111 |
| ENSP00000357842 | 1.14E-04 | -0.0910 | 0 | 0.122 |
| ENSP00000347495 | 1.76E-05 | -0.0910 | 404 | 0.663 |
| ENSP00000321636 | 1.12E-05 | -0.0910 | 256 | 0.684 |
| ENSP00000333724 | 2.50E-06 | -0.0910 | 0 | 0.000 |
| ENSP00000414330 | 1.52E-05 | -0.0910 | 221 | 0.161 |
| ENSP00000355759 | 3.42E-05 | -0.0910 | 623 | 0.782 |
| ENSP00000380156 | 2.93E-05 | -0.0910 | 194 | 0.808 |
| ENSP00000359837 | 5.29E-06 | -0.0910 | 0 | 0.721 |
| ENSP00000319984 | 2.66E-05 | -0.0910 | 270 | 0.591 |
| ENSP00000355649 | 3.27E-06 | -0.0910 | 0 | 0.391 |
| ENSP00000353093 | 1.22E-04 | -0.0910 | 158 | 0.134 |
| ENSP00000450144 | 9.42E-06 | -0.0910 | 0 | 0.145 |
| ENSP00000298585 | 7.00E-05 | -0.0910 | 456 | 0.147 |
| ENSP00000316861 | 6.78E-05 | -0.0911 | 152 | 0.101 |
| ENSP00000378996 | 5.98E-05 | -0.0911 | 0 | 0.122 |
| ENSP00000327344 | 9.76E-05 | -0.0911 | 0 | 0.170 |
| ENSP00000337907 | 6.46E-06 | -0.0911 | 302 | 0.459 |
| ENSP00000327608 | 6.38E-05 | -0.0911 | 0 | 0.161 |

| | | | | |
|---|---|---|---|---|
| ENSP00000262904 | 2.24E-05 | -0.0911 | 196 | 0.771 |
| ENSP00000363416 | 7.37E-05 | -0.0911 | 0 | 0.151 |
| ENSP00000344616 | 7.89E-05 | -0.0911 | 0 | 0.146 |
| ENSP00000354849 | 1.82E-05 | -0.0911 | 902 | 0.173 |
| ENSP00000345789 | 5.80E-05 | -0.0911 | 0 | 0.000 |
| ENSP00000345492 | 2.70E-05 | -0.0911 | 810 | 0.558 |
| ENSP00000302620 | 2.48E-05 | -0.0911 | 158 | 0.283 |
| ENSP00000262483 | 1.65E-05 | -0.0911 | 376 | 0.000 |
| ENSP00000339157 | 3.30E-05 | -0.0911 | 504 | 0.173 |
| ENSP00000254854 | 1.05E-05 | -0.0911 | 635 | 0.658 |
| ENSP00000264093 | 1.21E-05 | -0.0911 | 195 | 0.881 |
| ENSP00000378935 | 4.65E-05 | -0.0911 | 192 | 0.143 |
| ENSP00000348778 | 2.09E-05 | -0.0911 | 284 | 0.262 |
| ENSP00000367207 | 7.75E-05 | -0.0911 | 957 | 0.000 |
| ENSP00000299957 | 4.25E-05 | -0.0912 | 0 | 0.130 |
| ENSP00000369519 | 3.75E-05 | -0.0912 | 218 | 0.308 |
| ENSP00000253407 | 9.25E-05 | -0.0912 | 0 | 0.129 |
| ENSP00000322175 | 4.93E-05 | -0.0912 | 281 | 0.451 |
| ENSP00000269095 | 8.43E-06 | -0.0912 | 302 | 0.437 |
| ENSP00000354680 | 3.45E-06 | -0.0912 | 0 | 0.000 |
| ENSP00000242057 | 4.59E-05 | -0.0912 | 303 | 0.849 |
| ENSP00000367265 | 5.23E-05 | -0.0912 | 0 | 0.078 |
| ENSP00000367808 | 5.80E-05 | -0.0912 | 0 | 0.000 |
| ENSP00000305372 | 4.49E-05 | -0.0912 | 644 | 0.849 |
| ENSP00000363520 | 5.51E-05 | -0.0912 | 0 | 0.114 |
| ENSP00000220931 | 3.66E-06 | -0.0912 | 0 | 0.239 |
| ENSP00000310459 | 8.54E-05 | -0.0912 | 0 | 0.105 |
| ENSP00000249861 | 7.82E-05 | -0.0912 | 0 | 0.142 |
| ENSP00000265056 | 4.23E-06 | -0.0912 | 0 | 0.787 |
| ENSP00000352513 | 7.01E-05 | -0.0912 | 0 | 0.186 |
| ENSP00000327453 | 6.79E-06 | -0.0912 | 183 | 0.350 |
| ENSP00000357199 | 5.35E-05 | -0.0912 | 0 | 0.150 |
| ENSP00000267430 | 4.05E-06 | -0.0913 | 0 | 0.764 |
| ENSP00000367359 | 6.46E-05 | -0.0913 | 0 | 0.174 |
| ENSP00000356150 | 2.45E-05 | -0.0913 | 163 | 0.813 |
| ENSP00000349575 | 4.72E-05 | -0.0913 | 0 | 0.141 |
| ENSP00000379689 | 3.02E-06 | -0.0913 | 0 | 0.699 |
| ENSP00000341652 | 5.81E-05 | -0.0913 | 0 | 0.125 |
| ENSP00000331305 | 4.24E-05 | -0.0913 | 0 | 0.234 |
| ENSP00000297679 | 1.33E-05 | -0.0913 | 198 | 0.280 |
| ENSP00000361066 | 3.99E-05 | -0.0913 | 302 | 0.815 |
| ENSP00000394086 | 3.13E-06 | -0.0913 | 0 | 0.688 |
| ENSP00000348714 | 1.93E-04 | -0.0913 | 0 | 0.249 |
| ENSP00000370439 | 4.91E-05 | -0.0913 | 0 | 0.000 |
| ENSP00000337972 | 6.49E-06 | -0.0913 | 215 | 0.086 |
| ENSP00000350773 | 3.17E-06 | -0.0913 | 0 | 0.690 |

| | | | | |
|---|---|---|---|---|
| ENSP00000335229 | 2.25E-05 | -0.0913 | 0 | 0.000 |
| ENSP00000247461 | 2.70E-05 | -0.0913 | 937 | 0.650 |
| ENSP00000285021 | 5.81E-06 | -0.0914 | 0 | 0.748 |
| ENSP00000261597 | 3.30E-05 | -0.0914 | 343 | 0.588 |
| ENSP00000348933 | 3.34E-05 | -0.0914 | 412 | 0.089 |
| ENSP00000345997 | 3.12E-05 | -0.0914 | 325 | 0.301 |
| ENSP00000373474 | 3.47E-05 | -0.0914 | 0 | 0.461 |
| ENSP00000315915 | 4.74E-05 | -0.0914 | 0 | 0.093 |
| ENSP00000370889 | 3.25E-05 | -0.0914 | 271 | 0.089 |
| ENSP00000351490 | 3.46E-05 | -0.0914 | 903 | 0.728 |
| ENSP00000262547 | 5.60E-05 | -0.0914 | 0 | 0.125 |
| ENSP00000352841 | 6.85E-05 | -0.0914 | 0 | 0.149 |
| ENSP00000353971 | 4.14E-05 | -0.0914 | 0 | 0.117 |
| ENSP00000332198 | 5.70E-06 | -0.0914 | 0 | 0.872 |
| ENSP00000232458 | 4.87E-06 | -0.0914 | 0 | 0.338 |
| ENSP00000257572 | 4.24E-05 | -0.0914 | 0 | 0.240 |
| ENSP00000351035 | 2.94E-05 | -0.0914 | 168 | 0.219 |
| ENSP00000317903 | 7.21E-05 | -0.0914 | 0 | 0.182 |
| ENSP00000349465 | 1.97E-05 | -0.0914 | 271 | 0.451 |
| ENSP00000338072 | 2.06E-05 | -0.0915 | 458 | 0.394 |
| ENSP00000405575 | 2.64E-05 | -0.0915 | 359 | 0.000 |
| ENSP00000297991 | 3.30E-05 | -0.0915 | 229 | 0.473 |
| ENSP00000381607 | 5.42E-05 | -0.0915 | 301 | 0.762 |
| ENSP00000295025 | 1.47E-04 | -0.0915 | 589 | 0.927 |
| ENSP00000368805 | 2.31E-05 | -0.0915 | 214 | 0.733 |
| ENSP00000359827 | 4.14E-05 | -0.0915 | 0 | 0.084 |
| ENSP00000265960 | 7.77E-06 | -0.0915 | 0 | 0.684 |
| ENSP00000379294 | 3.81E-05 | -0.0915 | 0 | 0.153 |
| ENSP00000320291 | 7.08E-06 | -0.0915 | 0 | 0.141 |
| ENSP00000377942 | 3.80E-05 | -0.0915 | 0 | 0.133 |
| ENSP00000263969 | 1.19E-05 | -0.0915 | 194 | 0.354 |
| ENSP00000340494 | 3.28E-05 | -0.0915 | 0 | 0.229 |
| ENSP00000304410 | 4.68E-05 | -0.0915 | 0 | 0.153 |
| ENSP00000267512 | 3.63E-06 | -0.0915 | 0 | 0.249 |
| ENSP00000308897 | 8.54E-06 | -0.0915 | 361 | 0.726 |
| ENSP00000326817 | 1.00E-05 | -0.0915 | 489 | 0.660 |
| ENSP00000393719 | 4.09E-06 | -0.0916 | 0 | 0.150 |
| ENSP00000323847 | 2.32E-05 | -0.0916 | 274 | 0.503 |
| ENSP00000362638 | 4.39E-05 | -0.0916 | 0 | 0.115 |
| ENSP00000368450 | 3.13E-05 | -0.0916 | 242 | 0.833 |
| ENSP00000333376 | 1.13E-05 | -0.0916 | 0 | 0.240 |
| ENSP00000347444 | 3.40E-05 | -0.0916 | 0 | 0.221 |
| ENSP00000342012 | 3.84E-05 | -0.0916 | 583 | 0.137 |
| ENSP00000263097 | 1.17E-04 | -0.0916 | 376 | 0.401 |
| ENSP00000379669 | 2.84E-05 | -0.0916 | 0 | 0.000 |
| ENSP00000358576 | 2.31E-05 | -0.0916 | 153 | 0.601 |

| | | | | |
|---|---|---|---|---|
| ENSP00000354859 | 2.73E-05 | -0.0917 | 347 | 0.791 |
| ENSP00000221665 | 1.17E-05 | -0.0917 | 514 | 0.719 |
| ENSP00000314099 | 2.79E-05 | -0.0917 | 183 | 0.119 |
| ENSP00000353998 | 5.81E-05 | -0.0917 | 0 | 0.077 |
| ENSP00000219281 | 5.41E-05 | -0.0917 | 0 | 0.136 |
| ENSP00000310244 | 2.29E-05 | -0.0917 | 252 | 0.796 |
| ENSP00000362942 | 3.42E-05 | -0.0917 | 0 | 0.200 |
| ENSP00000362707 | 3.94E-05 | -0.0917 | 0 | 0.090 |
| ENSP00000271688 | 1.34E-05 | -0.0917 | 151 | 0.537 |
| ENSP00000358599 | 3.66E-06 | -0.0917 | 0 | 0.000 |
| ENSP00000352271 | 2.37E-05 | -0.0917 | 177 | 0.749 |
| ENSP00000361245 | 4.71E-06 | -0.0917 | 0 | 0.322 |
| ENSP00000260682 | 9.56E-06 | -0.0917 | 394 | 0.422 |
| ENSP00000472934 | 1.09E-06 | -0.0917 | 0 | 0.000 |
| ENSP00000298530 | 5.06E-05 | -0.0917 | 0 | 0.138 |
| ENSP00000362574 | 3.31E-05 | -0.0917 | 0 | 0.143 |
| ENSP00000263733 | 3.44E-05 | -0.0918 | 0 | 0.101 |
| ENSP00000349541 | 3.10E-05 | -0.0918 | 167 | 0.673 |
| ENSP00000290795 | 5.05E-05 | -0.0918 | 0 | 0.142 |
| ENSP00000358251 | 3.01E-05 | -0.0918 | 0 | 0.118 |
| ENSP00000228506 | 2.90E-04 | -0.0918 | 357 | 0.204 |
| ENSP00000258301 | 1.40E-05 | -0.0918 | 208 | 0.230 |
| ENSP00000255198 | 5.62E-05 | -0.0918 | 0 | 0.148 |
| ENSP00000353706 | 4.23E-05 | -0.0918 | 0 | 0.173 |
| ENSP00000359171 | 3.05E-05 | -0.0918 | 0 | 0.099 |
| ENSP00000371073 | 2.42E-05 | -0.0918 | 0 | 0.079 |
| ENSP00000376609 | 1.59E-05 | -0.0918 | 935 | 0.645 |
| ENSP00000356552 | 5.95E-05 | -0.0918 | 0 | 0.231 |
| ENSP00000360104 | 2.26E-05 | -0.0918 | 0 | 0.074 |
| ENSP00000295951 | 3.41E-06 | -0.0918 | 0 | 0.485 |
| ENSP00000263576 | 2.04E-05 | -0.0918 | 367 | 0.761 |
| ENSP00000387077 | 2.04E-05 | -0.0918 | 349 | 0.820 |
| ENSP00000350716 | 3.38E-05 | -0.0918 | 0 | 0.128 |
| ENSP00000308533 | 1.39E-05 | -0.0918 | 0 | 0.703 |
| ENSP00000257336 | 6.96E-05 | -0.0918 | 0 | 0.141 |
| ENSP00000375730 | 3.56E-05 | -0.0919 | 509 | 0.799 |
| ENSP00000303525 | 7.58E-05 | -0.0919 | 150 | 0.188 |
| ENSP00000392909 | 2.05E-06 | -0.0919 | 0 | 0.000 |
| ENSP00000457230 | 8.03E-05 | -0.0919 | 509 | 0.898 |
| ENSP00000344277 | 2.56E-05 | -0.0919 | 0 | 0.156 |
| ENSP00000382091 | 3.96E-05 | -0.0919 | 0 | 0.132 |
| ENSP00000363694 | 3.84E-05 | -0.0919 | 158 | 0.105 |
| ENSP00000345528 | 2.90E-05 | -0.0919 | 0 | 0.058 |
| ENSP00000397026 | 3.40E-05 | -0.0919 | 256 | 0.556 |
| ENSP00000343871 | 2.71E-05 | -0.0919 | 0 | 0.133 |
| ENSP00000358147 | 1.54E-04 | -0.0919 | 0 | 0.701 |

| | | | | |
|---|---|---|---|---|
| ENSP00000365550 | 1.33E-05 | -0.0919 | 512 | 0.101 |
| ENSP00000286692 | 3.33E-05 | -0.0919 | 0 | 0.096 |
| ENSP00000415030 | 3.24E-06 | -0.0919 | 0 | 0.182 |
| ENSP00000331172 | 3.21E-05 | -0.0920 | 0 | 0.462 |
| ENSP00000297164 | 6.06E-06 | -0.0920 | 0 | 0.121 |
| ENSP00000316891 | 2.28E-05 | -0.0920 | 0 | 0.111 |
| ENSP00000371278 | 1.78E-05 | -0.0920 | 159 | 0.424 |
| ENSP00000263512 | 3.03E-05 | -0.0920 | 0 | 0.089 |
| ENSP00000408617 | 5.57E-05 | -0.0920 | 394 | 0.000 |
| ENSP00000264852 | 3.34E-05 | -0.0920 | 0 | 0.108 |
| ENSP00000373360 | 2.05E-05 | -0.0920 | 0 | 0.199 |
| ENSP00000370345 | 2.19E-05 | -0.0920 | 0 | 0.418 |
| ENSP00000341368 | 5.41E-06 | -0.0920 | 0 | 0.162 |
| ENSP00000308461 | 2.23E-05 | -0.0920 | 204 | 0.562 |
| ENSP00000302397 | 9.66E-06 | -0.0920 | 240 | 0.308 |
| ENSP00000359052 | 2.68E-05 | -0.0920 | 0 | 0.122 |
| ENSP00000261692 | 2.47E-05 | -0.0920 | 0 | 0.292 |
| ENSP00000350333 | 5.25E-06 | -0.0920 | 0 | 0.786 |
| ENSP00000355114 | 2.62E-05 | -0.0920 | 360 | 0.113 |
| ENSP00000305059 | 1.47E-05 | -0.0920 | 193 | 0.000 |
| ENSP00000364034 | 6.23E-06 | -0.0920 | 160 | 0.000 |
| ENSP00000370055 | 4.92E-05 | -0.0920 | 0 | 0.161 |
| ENSP00000264775 | 7.44E-06 | -0.0920 | 0 | 0.000 |
| ENSP00000312122 | 1.05E-05 | -0.0920 | 312 | 0.352 |
| ENSP00000266673 | 5.38E-05 | -0.0920 | 0 | 0.113 |
| ENSP00000287202 | 2.40E-05 | -0.0920 | 0 | 0.242 |
| ENSP00000381216 | 3.21E-05 | -0.0921 | 176 | 0.757 |
| ENSP00000345374 | 1.48E-05 | -0.0921 | 0 | 0.000 |
| ENSP00000368503 | 5.29E-05 | -0.0921 | 0 | 0.117 |
| ENSP00000359768 | 4.29E-06 | -0.0921 | 0 | 0.483 |
| ENSP00000457299 | 1.97E-05 | -0.0921 | 224 | 0.000 |
| ENSP00000290219 | 2.35E-05 | -0.0921 | 0 | 0.498 |
| ENSP00000217909 | 3.10E-05 | -0.0921 | 0 | 0.090 |
| ENSP00000347896 | 2.67E-05 | -0.0921 | 0 | 0.488 |
| ENSP00000365756 | 2.38E-05 | -0.0921 | 0 | 0.149 |
| ENSP00000325660 | 2.66E-05 | -0.0921 | 300 | 0.641 |
| ENSP00000265085 | 2.34E-05 | -0.0921 | 0 | 0.275 |
| ENSP00000379451 | 3.04E-06 | -0.0921 | 0 | 0.685 |
| ENSP00000347184 | 3.74E-05 | -0.0921 | 448 | 0.893 |
| ENSP00000340989 | 3.87E-05 | -0.0922 | 322 | 0.696 |
| ENSP00000343190 | 3.88E-06 | -0.0922 | 0 | 0.517 |
| ENSP00000301585 | 9.21E-06 | -0.0922 | 225 | 0.644 |
| ENSP00000464803 | 1.85E-07 | -0.0922 | 0 | 0.000 |
| ENSP00000211314 | 6.09E-05 | -0.0922 | 0 | 0.138 |
| ENSP00000355340 | 4.76E-05 | -0.0922 | 0 | 0.145 |
| ENSP00000367196 | 2.43E-05 | -0.0922 | 0 | 0.151 |

| | | | | |
|---|---|---|---|---|
| ENSP00000356110 | 2.96E-05 | -0.0922 | 0 | 0.110 |
| ENSP00000259748 | 5.08E-05 | -0.0922 | 0 | 0.530 |
| ENSP00000353538 | 4.94E-06 | -0.0922 | 0 | 0.471 |
| ENSP00000297273 | 2.70E-05 | -0.0922 | 0 | 0.077 |
| ENSP00000379368 | 3.45E-06 | -0.0922 | 0 | 0.716 |
| ENSP00000313933 | 2.73E-05 | -0.0922 | 0 | 0.138 |
| ENSP00000364054 | 2.03E-05 | -0.0922 | 0 | 0.122 |
| ENSP00000361383 | 3.20E-05 | -0.0922 | 0 | 0.147 |
| ENSP00000256720 | 9.97E-06 | -0.0922 | 198 | 0.668 |
| ENSP00000348848 | 5.80E-06 | -0.0922 | 0 | 0.092 |
| ENSP00000344871 | 1.53E-05 | -0.0922 | 244 | 0.368 |
| ENSP00000350009 | 2.04E-05 | -0.0923 | 0 | 0.162 |
| ENSP00000290583 | 2.31E-05 | -0.0923 | 0 | 0.359 |
| ENSP00000466214 | 3.57E-06 | -0.0923 | 0 | 0.479 |
| ENSP00000320076 | 8.54E-06 | -0.0923 | 202 | 0.000 |
| ENSP00000356849 | 2.68E-05 | -0.0923 | 0 | 0.178 |
| ENSP00000381428 | 3.65E-05 | -0.0923 | 0 | 0.113 |
| ENSP00000366243 | 2.14E-05 | -0.0923 | 0 | 0.065 |
| ENSP00000365884 | 1.52E-05 | -0.0923 | 213 | 0.330 |
| ENSP00000349929 | 1.92E-05 | -0.0923 | 197 | 0.343 |
| ENSP00000339659 | 3.09E-05 | -0.0923 | 0 | 0.135 |
| ENSP00000266754 | 2.25E-05 | -0.0923 | 0 | 0.123 |
| ENSP00000261593 | 4.13E-05 | -0.0923 | 0 | 0.100 |
| ENSP00000313350 | 2.19E-05 | -0.0923 | 202 | 0.572 |
| ENSP00000312615 | 3.85E-05 | -0.0923 | 0 | 0.126 |
| ENSP00000360181 | 3.13E-05 | -0.0923 | 200 | 0.812 |
| ENSP00000354158 | 3.25E-05 | -0.0923 | 379 | 0.809 |
| ENSP00000182527 | 2.07E-05 | -0.0923 | 0 | 0.119 |
| ENSP00000306548 | 8.87E-06 | -0.0923 | 409 | 0.699 |
| ENSP00000315554 | 4.70E-06 | -0.0923 | 0 | 0.088 |
| ENSP00000361795 | 7.25E-07 | -0.0923 | 0 | 0.188 |
| ENSP00000301905 | 2.28E-05 | -0.0924 | 439 | 0.711 |
| ENSP00000276052 | 2.46E-05 | -0.0924 | 363 | 0.000 |
| ENSP00000325421 | 1.59E-05 | -0.0924 | 488 | 0.240 |
| ENSP00000356602 | 1.98E-05 | -0.0924 | 0 | 0.043 |
| ENSP00000369647 | 3.43E-05 | -0.0924 | 459 | 0.767 |
| ENSP00000311766 | 1.03E-05 | -0.0924 | 362 | 0.535 |
| ENSP00000222982 | 1.74E-05 | -0.0924 | 159 | 0.347 |
| ENSP00000376071 | 3.50E-05 | -0.0924 | 0 | 0.109 |
| ENSP00000363136 | 2.20E-05 | -0.0924 | 288 | 0.369 |
| ENSP00000328938 | 1.91E-05 | -0.0924 | 0 | 0.077 |
| ENSP00000256442 | 2.59E-05 | -0.0924 | 357 | 0.756 |
| ENSP00000349955 | 4.77E-05 | -0.0924 | 800 | 0.380 |
| ENSP00000284031 | 1.80E-05 | -0.0924 | 150 | 0.129 |
| ENSP00000343199 | 2.58E-05 | -0.0924 | 192 | 0.091 |
| ENSP00000347045 | 3.07E-06 | -0.0924 | 0 | 0.685 |

| | | | | |
|---|---|---|---|---|
| ENSP00000349773 | 2.23E-05 | -0.0924 | 0 | 0.076 |
| ENSP00000369402 | 2.05E-05 | -0.0924 | 0 | 0.077 |
| ENSP00000359160 | 3.87E-05 | -0.0925 | 0 | 0.194 |
| ENSP00000360184 | 2.61E-05 | -0.0925 | 0 | 0.123 |
| ENSP00000265981 | 3.59E-05 | -0.0925 | 0 | 0.127 |
| ENSP00000343000 | 2.28E-05 | -0.0925 | 0 | 0.392 |
| ENSP00000368314 | 2.94E-05 | -0.0925 | 914 | 0.670 |
| ENSP00000312988 | 1.21E-05 | -0.0925 | 394 | 0.854 |
| ENSP00000303437 | 2.17E-05 | -0.0925 | 151 | 0.212 |
| ENSP00000310520 | 4.52E-06 | -0.0925 | 0 | 0.769 |
| ENSP00000267116 | 7.12E-06 | -0.0925 | 202 | 0.635 |
| ENSP00000267984 | 1.68E-05 | -0.0925 | 0 | 0.234 |
| ENSP00000331720 | 3.07E-05 | -0.0925 | 0 | 0.112 |
| ENSP00000360776 | 1.81E-05 | -0.0925 | 0 | 0.083 |
| ENSP00000227322 | 5.98E-05 | -0.0925 | 474 | 0.000 |
| ENSP00000380054 | 1.94E-05 | -0.0925 | 0 | 0.542 |
| ENSP00000307340 | 2.28E-05 | -0.0925 | 0 | 0.000 |
| ENSP00000305742 | 1.80E-05 | -0.0925 | 0 | 0.055 |
| ENSP00000383909 | 1.86E-05 | -0.0925 | 0 | 0.682 |
| ENSP00000247170 | 8.08E-06 | -0.0925 | 299 | 0.000 |
| ENSP00000356265 | 2.03E-05 | -0.0925 | 0 | 0.121 |
| ENSP00000364219 | 1.84E-05 | -0.0925 | 0 | 0.119 |
| ENSP00000333837 | 6.35E-06 | -0.0925 | 353 | 0.641 |
| ENSP00000380073 | 2.09E-05 | -0.0925 | 900 | 0.697 |
| ENSP00000262067 | 2.16E-05 | -0.0925 | 0 | 0.158 |
| ENSP00000337618 | 1.68E-05 | -0.0926 | 0 | 0.080 |
| ENSP00000406482 | 2.75E-05 | -0.0926 | 0 | 0.098 |
| ENSP00000246421 | 2.55E-05 | -0.0926 | 0 | 0.058 |
| ENSP00000293350 | 8.34E-06 | -0.0926 | 216 | 0.299 |
| ENSP00000352655 | 1.56E-05 | -0.0926 | 0 | 0.093 |
| ENSP00000301141 | 9.60E-06 | -0.0926 | 197 | 0.405 |
| ENSP00000349783 | 1.79E-05 | -0.0926 | 0 | 0.367 |
| ENSP00000272233 | 2.45E-05 | -0.0926 | 546 | 0.602 |
| ENSP00000373472 | 2.57E-05 | -0.0926 | 309 | 0.253 |
| ENSP00000348020 | 6.63E-06 | -0.0926 | 353 | 0.635 |
| ENSP00000456743 | 1.30E-06 | -0.0926 | 0 | 0.193 |
| ENSP00000243644 | 1.05E-05 | -0.0926 | 808 | 0.731 |
| ENSP00000264914 | 2.07E-05 | -0.0926 | 0 | 0.123 |
| ENSP00000355809 | 2.21E-05 | -0.0926 | 181 | 0.586 |
| ENSP00000338348 | 7.26E-06 | -0.0926 | 0 | 0.328 |
| ENSP00000283131 | 1.05E-05 | -0.0926 | 268 | 0.926 |
| ENSP00000369380 | 2.63E-05 | -0.0926 | 0 | 0.238 |
| ENSP00000262445 | 5.34E-05 | -0.0926 | 203 | 0.886 |
| ENSP00000335592 | 5.67E-06 | -0.0927 | 0 | 0.242 |
| ENSP00000391404 | 2.31E-06 | -0.0927 | 0 | 0.202 |
| ENSP00000345341 | 6.54E-06 | -0.0927 | 0 | 0.229 |

| | | | | |
|---|---|---|---|---|
| ENSP00000362460 | 2.34E-05 | -0.0927 | 185 | 0.174 |
| ENSP00000309477 | 4.49E-05 | -0.0927 | 336 | 0.599 |
| ENSP00000359339 | 2.25E-05 | -0.0927 | 0 | 0.119 |
| ENSP00000304923 | 1.02E-05 | -0.0927 | 479 | 0.657 |
| ENSP00000356919 | 3.12E-05 | -0.0927 | 997 | 0.842 |
| ENSP00000347714 | 8.21E-06 | -0.0927 | 0 | 0.182 |
| ENSP00000339813 | 2.33E-05 | -0.0927 | 165 | 0.296 |
| ENSP00000377280 | 3.01E-05 | -0.0927 | 191 | 0.189 |
| ENSP00000438244 | 8.80E-07 | -0.0927 | 0 | 0.121 |
| ENSP00000261207 | 2.96E-05 | -0.0927 | 295 | 0.839 |
| ENSP00000303909 | 1.38E-05 | -0.0927 | 150 | 0.493 |
| ENSP00000377665 | 1.74E-05 | -0.0927 | 0 | 0.099 |
| ENSP00000080059 | 9.04E-06 | -0.0928 | 563 | 0.000 |
| ENSP00000282486 | 4.05E-05 | -0.0928 | 198 | 0.670 |
| ENSP00000404503 | 2.82E-05 | -0.0928 | 207 | 0.834 |
| ENSP00000365522 | 2.23E-05 | -0.0928 | 0 | 0.193 |
| ENSP00000363824 | 2.66E-05 | -0.0928 | 0 | 0.105 |
| ENSP00000300119 | 1.45E-05 | -0.0928 | 244 | 0.428 |
| ENSP00000350215 | 2.64E-05 | -0.0928 | 0 | 0.426 |
| ENSP00000362711 | 2.14E-05 | -0.0928 | 0 | 0.091 |
| ENSP00000347560 | 2.18E-05 | -0.0928 | 0 | 0.000 |
| ENSP00000371790 | 4.02E-05 | -0.0928 | 282 | 0.952 |
| ENSP00000357880 | 2.71E-05 | -0.0928 | 211 | 0.248 |
| ENSP00000324020 | 3.82E-06 | -0.0928 | 0 | 0.217 |
| ENSP00000361043 | 1.58E-05 | -0.0928 | 160 | 0.000 |
| ENSP00000381104 | 1.67E-05 | -0.0928 | 0 | 0.103 |
| ENSP00000336587 | 2.28E-05 | -0.0928 | 0 | 0.072 |
| ENSP00000265997 | 1.71E-05 | -0.0928 | 0 | 0.225 |
| ENSP00000345333 | 3.47E-06 | -0.0929 | 0 | 0.701 |
| ENSP00000355747 | 3.88E-05 | -0.0929 | 725 | 0.876 |
| ENSP00000373476 | 1.62E-05 | -0.0929 | 0 | 0.000 |
| ENSP00000348578 | 3.38E-05 | -0.0929 | 453 | 0.879 |
| ENSP00000362590 | 2.55E-05 | -0.0929 | 300 | 0.163 |
| ENSP00000348108 | 2.78E-05 | -0.0929 | 334 | 0.685 |
| ENSP00000238789 | 1.12E-05 | -0.0929 | 338 | 0.737 |
| ENSP00000303710 | 1.67E-05 | -0.0929 | 0 | 0.000 |
| ENSP00000320709 | 5.68E-05 | -0.0929 | 912 | 0.910 |
| ENSP00000333946 | 4.82E-06 | -0.0929 | 0 | 0.124 |
| ENSP00000380310 | 3.07E-06 | -0.0929 | 0 | 0.684 |
| ENSP00000269848 | 1.08E-05 | -0.0929 | 331 | 0.652 |
| ENSP00000310749 | 3.72E-06 | -0.0929 | 0 | 0.298 |
| ENSP00000336931 | 3.07E-05 | -0.0929 | 188 | 0.630 |
| ENSP00000306410 | 7.65E-06 | -0.0929 | 0 | 0.083 |
| ENSP00000302108 | 1.54E-05 | -0.0929 | 0 | 0.130 |
| ENSP00000216797 | 2.12E-05 | -0.0929 | 583 | 0.889 |
| ENSP00000264344 | 6.67E-06 | -0.0929 | 0 | 0.292 |

| | | | | |
|---|---|---|---|---|
| ENSP00000367355 | 1.12E-05 | -0.0929 | 0 | 0.550 |
| ENSP00000285947 | 1.69E-05 | -0.0929 | 0 | 0.130 |
| ENSP00000359292 | 2.21E-05 | -0.0929 | 0 | 0.000 |
| ENSP00000257765 | 5.71E-06 | -0.0929 | 0 | 0.198 |
| ENSP00000228865 | 1.90E-05 | -0.0930 | 0 | 0.318 |
| ENSP00000379654 | 1.91E-05 | -0.0930 | 0 | 0.075 |
| ENSP00000372459 | 3.02E-05 | -0.0930 | 0 | 0.539 |
| ENSP00000270861 | 1.02E-05 | -0.0930 | 205 | 0.447 |
| ENSP00000259870 | 5.24E-06 | -0.0930 | 0 | 0.190 |
| ENSP00000254846 | 1.01E-05 | -0.0930 | 183 | 0.000 |
| ENSP00000331044 | 1.84E-05 | -0.0930 | 0 | 0.090 |
| ENSP00000363852 | 1.97E-05 | -0.0930 | 0 | 0.105 |
| ENSP00000359474 | 2.38E-05 | -0.0930 | 0 | 0.666 |
| ENSP00000350170 | 3.62E-05 | -0.0930 | 177 | 0.596 |
| ENSP00000358363 | 1.69E-05 | -0.0930 | 0 | 0.101 |
| ENSP00000295628 | 9.99E-06 | -0.0930 | 482 | 0.634 |
| ENSP00000300651 | 7.05E-05 | -0.0930 | 900 | 0.795 |
| ENSP00000348050 | 2.20E-05 | -0.0930 | 0 | 0.141 |
| ENSP00000322242 | 5.88E-06 | -0.0930 | 0 | 0.442 |
| ENSP00000375767 | 2.28E-05 | -0.0931 | 0 | 0.608 |
| ENSP00000337405 | 4.79E-06 | -0.0931 | 0 | 0.577 |
| ENSP00000364493 | 2.82E-05 | -0.0931 | 0 | 0.107 |
| ENSP00000255194 | 9.50E-06 | -0.0931 | 207 | 0.217 |
| ENSP00000304111 | 3.38E-05 | -0.0931 | 480 | 0.000 |
| ENSP00000312618 | 4.87E-06 | -0.0931 | 0 | 0.229 |
| ENSP00000386213 | 1.82E-05 | -0.0931 | 244 | 0.360 |
| ENSP00000175506 | 4.25E-05 | -0.0931 | 359 | 0.586 |
| ENSP00000360626 | 2.22E-05 | -0.0931 | 187 | 0.159 |
| ENSP00000216267 | 1.02E-05 | -0.0931 | 430 | 0.000 |
| ENSP00000376615 | 2.13E-05 | -0.0931 | 0 | 0.107 |
| ENSP00000013034 | 2.28E-05 | -0.0931 | 874 | 0.828 |
| ENSP00000206380 | 2.18E-05 | -0.0932 | 0 | 0.147 |
| ENSP00000265723 | 1.29E-05 | -0.0932 | 398 | 0.560 |
| ENSP00000258494 | 7.72E-06 | -0.0932 | 216 | 0.278 |
| ENSP00000262709 | 3.29E-05 | -0.0932 | 269 | 0.000 |
| ENSP00000262244 | 2.05E-05 | -0.0932 | 0 | 0.071 |
| ENSP00000220531 | 1.75E-05 | -0.0932 | 0 | 0.079 |
| ENSP00000383851 | 7.97E-06 | -0.0932 | 0 | 0.123 |
| ENSP00000351141 | 1.32E-05 | -0.0932 | 0 | 0.350 |
| ENSP00000302707 | 2.26E-05 | -0.0932 | 234 | 0.585 |
| ENSP00000357389 | 2.06E-05 | -0.0932 | 0 | 0.104 |
| ENSP00000297632 | 1.48E-05 | -0.0932 | 0 | 0.113 |
| ENSP00000342513 | 1.92E-05 | -0.0932 | 0 | 0.328 |
| ENSP00000323516 | 1.41E-05 | -0.0932 | 0 | 0.615 |
| ENSP00000357683 | 2.56E-05 | -0.0932 | 0 | 0.406 |
| ENSP00000360597 | 7.05E-06 | -0.0933 | 0 | 0.656 |

| | | | | |
|---|---|---|---|---|
| ENSP00000234677 | 2.94E-05 | -0.0933 | 347 | 0.694 |
| ENSP00000346959 | 1.55E-05 | -0.0933 | 170 | 0.047 |
| ENSP00000262901 | 1.25E-05 | -0.0933 | 568 | 0.812 |
| ENSP00000309124 | 9.22E-05 | -0.0933 | 700 | 0.857 |
| ENSP00000376544 | 1.63E-05 | -0.0933 | 0 | 0.643 |
| ENSP00000379051 | 1.56E-05 | -0.0933 | 0 | 0.580 |
| ENSP00000428331 | 2.30E-05 | -0.0933 | 0 | 0.109 |
| ENSP00000375881 | 2.37E-05 | -0.0933 | 325 | 0.441 |
| ENSP00000403524 | 8.67E-06 | -0.0933 | 169 | 0.674 |
| ENSP00000360231 | 1.49E-05 | -0.0933 | 0 | 0.632 |
| ENSP00000333122 | 2.06E-05 | -0.0933 | 167 | 0.836 |
| ENSP00000217958 | 9.88E-06 | -0.0933 | 201 | 0.693 |
| ENSP00000203001 | 4.73E-06 | -0.0933 | 0 | 0.836 |
| ENSP00000285814 | 1.51E-05 | -0.0933 | 362 | 0.000 |
| ENSP00000283109 | 4.69E-06 | -0.0934 | 0 | 0.853 |
| ENSP00000349168 | 3.59E-05 | -0.0934 | 231 | 0.876 |
| ENSP00000247005 | 1.72E-05 | -0.0934 | 159 | 0.874 |
| ENSP00000376800 | 1.29E-05 | -0.0934 | 0 | 0.297 |
| ENSP00000368623 | 7.04E-07 | -0.0934 | 0 | 0.176 |
| ENSP00000259708 | 1.20E-05 | -0.0934 | 316 | 0.551 |
| ENSP00000304895 | 3.39E-05 | -0.0934 | 905 | 0.945 |
| ENSP00000302216 | 1.77E-05 | -0.0934 | 216 | 0.863 |
| ENSP00000357836 | 1.97E-05 | -0.0934 | 0 | 0.124 |
| ENSP00000406463 | 2.21E-05 | -0.0934 | 776 | 0.430 |
| ENSP00000328858 | 4.29E-05 | -0.0934 | 929 | 0.701 |
| ENSP00000455607 | 2.79E-05 | -0.0934 | 0 | 0.279 |
| ENSP00000302120 | 1.54E-05 | -0.0934 | 0 | 0.332 |
| ENSP00000355217 | 3.43E-05 | -0.0935 | 227 | 0.167 |
| ENSP00000386520 | 3.12E-05 | -0.0935 | 238 | 0.923 |
| ENSP00000244051 | 1.30E-05 | -0.0935 | 496 | 0.426 |
| ENSP00000443459 | 3.30E-05 | -0.0935 | 254 | 0.458 |
| ENSP00000359222 | 1.73E-05 | -0.0935 | 0 | 0.131 |
| ENSP00000323439 | 4.08E-06 | -0.0935 | 0 | 0.771 |
| ENSP00000359890 | 2.59E-05 | -0.0935 | 152 | 0.389 |
| ENSP00000272117 | 1.64E-05 | -0.0935 | 0 | 0.228 |
| ENSP00000316137 | 5.67E-08 | -0.0935 | 0 | 0.000 |
| ENSP00000355306 | 1.86E-05 | -0.0935 | 0 | 0.142 |
| ENSP00000376436 | 3.19E-05 | -0.0935 | 836 | 0.868 |
| ENSP00000300584 | 3.58E-06 | -0.0935 | 0 | 0.182 |
| ENSP00000243298 | 3.45E-06 | -0.0935 | 0 | 0.186 |
| ENSP00000356795 | 1.58E-05 | -0.0936 | 218 | 0.857 |
| ENSP00000347198 | 1.25E-05 | -0.0936 | 0 | 0.314 |
| ENSP00000220058 | 1.76E-05 | -0.0936 | 317 | 0.603 |
| ENSP00000282570 | 8.49E-06 | -0.0936 | 0 | 0.151 |
| ENSP00000262942 | 5.33E-05 | -0.0936 | 382 | 0.698 |
| ENSP00000361162 | 2.81E-05 | -0.0936 | 218 | 0.279 |

| | | | | |
|---|---|---|---|---|
| ENSP00000349098 | 3.48E-06 | -0.0936 | 0 | 0.715 |
| ENSP00000237380 | 9.11E-05 | -0.0936 | 397 | 0.735 |
| ENSP00000350681 | 1.44E-05 | -0.0936 | 0 | 0.745 |
| ENSP00000253083 | 1.75E-05 | -0.0936 | 359 | 0.350 |
| ENSP00000401363 | 1.21E-05 | -0.0936 | 0 | 0.970 |
| ENSP00000379168 | 3.18E-06 | -0.0936 | 0 | 0.692 |
| ENSP00000352497 | 2.48E-05 | -0.0936 | 0 | 0.094 |
| ENSP00000369339 | 2.29E-05 | -0.0936 | 300 | 0.310 |
| ENSP00000302578 | 2.11E-05 | -0.0936 | 0 | 0.103 |
| ENSP00000376889 | 4.43E-05 | -0.0936 | 846 | 0.767 |
| ENSP00000387435 | 1.58E-05 | -0.0936 | 311 | 0.530 |
| ENSP00000356468 | 1.31E-05 | -0.0937 | 0 | 0.535 |
| ENSP00000341681 | 9.77E-06 | -0.0937 | 0 | 0.066 |
| ENSP00000362166 | 3.39E-05 | -0.0937 | 167 | 0.842 |
| ENSP00000310006 | 1.30E-05 | -0.0937 | 198 | 0.689 |
| ENSP00000384312 | 1.27E-05 | -0.0937 | 0 | 0.089 |
| ENSP00000229416 | 6.60E-05 | -0.0937 | 905 | 0.548 |
| ENSP00000318607 | 2.38E-05 | -0.0937 | 0 | 0.156 |
| ENSP00000382083 | 2.07E-05 | -0.0937 | 208 | 0.446 |
| ENSP00000327975 | 1.21E-05 | -0.0937 | 0 | 0.198 |
| ENSP00000258654 | 9.18E-06 | -0.0937 | 0 | 0.173 |
| ENSP00000274192 | 1.67E-05 | -0.0937 | 0 | 0.188 |
| ENSP00000291565 | 7.84E-05 | -0.0937 | 488 | 0.559 |
| ENSP00000368341 | 1.76E-05 | -0.0937 | 0 | 0.343 |
| ENSP00000311196 | 1.42E-05 | -0.0937 | 0 | 0.424 |
| ENSP00000221561 | 4.07E-05 | -0.0937 | 273 | 0.825 |
| ENSP00000355089 | 1.15E-05 | -0.0937 | 0 | 0.303 |
| ENSP00000242719 | 5.20E-06 | -0.0937 | 0 | 0.520 |
| ENSP00000351811 | 1.72E-05 | -0.0937 | 0 | 0.595 |
| ENSP00000352575 | 1.70E-05 | -0.0937 | 165 | 0.660 |
| ENSP00000247271 | 1.04E-05 | -0.0938 | 151 | 0.803 |
| ENSP00000333298 | 4.42E-05 | -0.0938 | 509 | 0.659 |
| ENSP00000359539 | 1.90E-05 | -0.0938 | 866 | 0.391 |
| ENSP00000170168 | 1.28E-05 | -0.0938 | 205 | 0.806 |
| ENSP00000366466 | 2.36E-05 | -0.0938 | 242 | 0.698 |
| ENSP00000351605 | 2.32E-05 | -0.0938 | 158 | 0.906 |
| ENSP00000365159 | 1.08E-05 | -0.0938 | 398 | 0.179 |
| ENSP00000381109 | 4.03E-06 | -0.0938 | 0 | 0.209 |
| ENSP00000345292 | 1.36E-05 | -0.0938 | 0 | 0.073 |
| ENSP00000419425 | 6.50E-05 | -0.0938 | 733 | 0.809 |
| ENSP00000298130 | 1.65E-05 | -0.0938 | 0 | 0.076 |
| ENSP00000366173 | 3.76E-06 | -0.0938 | 0 | 0.162 |
| ENSP00000354633 | 1.41E-05 | -0.0938 | 0 | 0.804 |
| ENSP00000388311 | 3.63E-06 | -0.0938 | 0 | 0.696 |
| ENSP00000362773 | 1.58E-05 | -0.0938 | 309 | 0.491 |
| ENSP00000321239 | 1.17E-05 | -0.0938 | 305 | 0.501 |

| | | | | |
|---|---|---|---|---|
| ENSP00000269576 | 5.87E-05 | -0.0939 | 266 | 0.666 |
| ENSP00000308080 | 9.48E-06 | -0.0939 | 0 | 0.000 |
| ENSP00000308022 | 9.80E-06 | -0.0939 | 368 | 0.000 |
| ENSP00000264027 | 8.64E-06 | -0.0939 | 163 | 0.406 |
| ENSP00000377995 | 2.18E-05 | -0.0939 | 0 | 0.130 |
| ENSP00000294635 | 6.32E-06 | -0.0939 | 0 | 0.663 |
| ENSP00000361522 | 2.70E-05 | -0.0939 | 0 | 0.206 |
| ENSP00000371200 | 4.44E-06 | -0.0939 | 0 | 0.090 |
| ENSP00000355204 | 1.32E-05 | -0.0939 | 0 | 0.119 |
| ENSP00000362360 | 3.07E-06 | -0.0939 | 0 | 0.000 |
| ENSP00000358605 | 1.60E-05 | -0.0939 | 0 | 0.738 |
| ENSP00000270223 | 8.88E-06 | -0.0939 | 195 | 0.559 |
| ENSP00000355556 | 1.60E-05 | -0.0940 | 0 | 0.434 |
| ENSP00000305422 | 9.39E-05 | -0.0940 | 349 | 0.887 |
| ENSP00000355656 | 1.14E-05 | -0.0940 | 158 | 0.991 |
| ENSP00000368538 | 1.14E-05 | -0.0940 | 197 | 0.852 |
| ENSP00000263610 | 1.92E-05 | -0.0940 | 0 | 0.684 |
| ENSP00000252029 | 7.46E-05 | -0.0940 | 361 | 0.585 |
| ENSP00000222803 | 1.34E-05 | -0.0940 | 260 | 0.398 |
| ENSP00000300176 | 7.33E-06 | -0.0940 | 0 | 0.128 |
| ENSP00000332389 | 5.44E-06 | -0.0941 | 0 | 0.142 |
| ENSP00000364986 | 1.10E-05 | -0.0941 | 0 | 0.142 |
| ENSP00000383168 | 1.99E-05 | -0.0941 | 333 | 0.652 |
| ENSP00000283646 | 3.47E-05 | -0.0941 | 300 | 0.610 |
| ENSP00000313731 | 6.63E-06 | -0.0941 | 0 | 0.000 |
| ENSP00000318868 | 1.83E-05 | -0.0941 | 847 | 0.646 |
| ENSP00000394623 | 7.24E-06 | -0.0941 | 0 | 0.229 |
| ENSP00000346065 | 1.08E-05 | -0.0941 | 0 | 0.521 |
| ENSP00000262133 | 4.14E-05 | -0.0941 | 296 | 0.823 |
| ENSP00000311962 | 4.59E-06 | -0.0942 | 0 | 0.166 |
| ENSP00000315173 | 1.21E-05 | -0.0942 | 606 | 0.694 |
| ENSP00000370616 | 1.60E-05 | -0.0942 | 165 | 0.430 |
| ENSP00000334538 | 1.58E-05 | -0.0942 | 0 | 0.867 |
| ENSP00000369871 | 1.37E-05 | -0.0942 | 0 | 0.134 |
| ENSP00000370256 | 3.73E-05 | -0.0942 | 191 | 0.000 |
| ENSP00000357069 | 1.33E-05 | -0.0942 | 0 | 0.089 |
| ENSP00000279147 | 1.46E-05 | -0.0942 | 0 | 0.099 |
| ENSP00000306817 | 1.55E-05 | -0.0942 | 150 | 0.262 |
| ENSP00000371376 | 1.09E-05 | -0.0942 | 282 | 0.800 |
| ENSP00000294753 | 1.63E-05 | -0.0942 | 224 | 0.695 |
| ENSP00000366225 | 1.05E-05 | -0.0943 | 0 | 0.218 |
| ENSP00000362207 | 1.85E-05 | -0.0943 | 608 | 0.112 |
| ENSP00000274680 | 8.18E-06 | -0.0943 | 388 | 0.653 |
| ENSP00000331745 | 2.20E-05 | -0.0943 | 194 | 0.869 |
| ENSP00000301019 | 6.02E-06 | -0.0943 | 0 | 0.621 |
| ENSP00000356623 | 1.73E-05 | -0.0943 | 0 | 0.699 |

| | | | | |
|---|---|---|---|---|
| ENSP00000323858 | 7.08E-06 | -0.0943 | 0 | 0.903 |
| ENSP00000366482 | 1.96E-05 | -0.0943 | 283 | 0.630 |
| ENSP00000304429 | 5.41E-06 | -0.0943 | 0 | 0.125 |
| ENSP00000262873 | 1.34E-05 | -0.0943 | 434 | 0.479 |
| ENSP00000367893 | 1.26E-05 | -0.0943 | 0 | 0.228 |
| ENSP00000266058 | 6.05E-06 | -0.0943 | 163 | 0.824 |
| ENSP00000312224 | 1.38E-05 | -0.0943 | 0 | 0.140 |
| ENSP00000344215 | 2.13E-05 | -0.0943 | 0 | 0.705 |
| ENSP00000362217 | 1.09E-05 | -0.0943 | 0 | 0.785 |
| ENSP00000225577 | 1.85E-05 | -0.0943 | 874 | 0.000 |
| ENSP00000419494 | 2.43E-05 | -0.0943 | 566 | 0.711 |
| ENSP00000362314 | 2.07E-05 | -0.0943 | 375 | 0.706 |
| ENSP00000356125 | 1.61E-05 | -0.0944 | 0 | 0.500 |
| ENSP00000327070 | 1.23E-05 | -0.0944 | 356 | 0.508 |
| ENSP00000342295 | 7.56E-06 | -0.0944 | 202 | 0.618 |
| ENSP00000345024 | 5.58E-06 | -0.0944 | 271 | 0.723 |
| ENSP00000367096 | 1.19E-05 | -0.0944 | 0 | 0.074 |
| ENSP00000337459 | 3.84E-05 | -0.0944 | 856 | 0.891 |
| ENSP00000279027 | 5.43E-06 | -0.0944 | 0 | 0.072 |
| ENSP00000363414 | 1.79E-05 | -0.0944 | 0 | 0.083 |
| ENSP00000294053 | 1.03E-05 | -0.0944 | 964 | 0.684 |
| ENSP00000317686 | 1.18E-05 | -0.0945 | 197 | 0.781 |
| ENSP00000294485 | 2.08E-05 | -0.0945 | 0 | 0.129 |
| ENSP00000352956 | 2.59E-05 | -0.0945 | 221 | 0.805 |
| ENSP00000296802 | 3.93E-06 | -0.0945 | 0 | 0.904 |
| ENSP00000320768 | 5.21E-05 | -0.0945 | 185 | 0.820 |
| ENSP00000328875 | 1.88E-05 | -0.0945 | 0 | 0.077 |
| ENSP00000304286 | 9.93E-06 | -0.0945 | 319 | 0.000 |
| ENSP00000346139 | 1.03E-05 | -0.0945 | 0 | 0.097 |
| ENSP00000308937 | 1.11E-05 | -0.0946 | 163 | 0.359 |
| ENSP00000340954 | 2.24E-05 | -0.0946 | 760 | 0.646 |
| ENSP00000263974 | 9.76E-06 | -0.0946 | 0 | 0.845 |
| ENSP00000230671 | 1.23E-05 | -0.0946 | 0 | 0.467 |
| ENSP00000393725 | 8.69E-06 | -0.0946 | 250 | 0.734 |
| ENSP00000350297 | 4.69E-05 | -0.0946 | 263 | 0.391 |
| ENSP00000362690 | 2.72E-05 | -0.0946 | 206 | 0.865 |
| ENSP00000251757 | 1.69E-05 | -0.0946 | 0 | 0.000 |
| ENSP00000262493 | 1.37E-05 | -0.0946 | 508 | 0.652 |
| ENSP00000356425 | 1.41E-05 | -0.0946 | 318 | 0.553 |
| ENSP00000357624 | 2.86E-05 | -0.0946 | 817 | 0.000 |
| ENSP00000288666 | 1.08E-05 | -0.0946 | 176 | 0.000 |
| ENSP00000360222 | 1.85E-05 | -0.0946 | 0 | 0.080 |
| ENSP00000238156 | 7.67E-05 | -0.0946 | 0 | 0.132 |
| ENSP00000379144 | 2.12E-05 | -0.0946 | 0 | 0.573 |
| ENSP00000342021 | 4.56E-06 | -0.0946 | 0 | 0.000 |
| ENSP00000359290 | 5.89E-05 | -0.0946 | 290 | 0.757 |

| | | | | |
|---|---|---|---|---|
| ENSP00000381066 | 1.63E-05 | -0.0947 | 210 | 0.856 |
| ENSP00000284811 | 5.71E-06 | -0.0947 | 0 | 0.687 |
| ENSP00000364246 | 1.33E-05 | -0.0947 | 296 | 0.194 |
| ENSP00000228318 | 1.02E-05 | -0.0947 | 154 | 0.283 |
| ENSP00000384048 | 2.38E-05 | -0.0947 | 333 | 0.863 |
| ENSP00000318177 | 8.81E-05 | -0.0947 | 270 | 0.647 |
| ENSP00000352738 | 3.82E-05 | -0.0947 | 364 | 0.856 |
| ENSP00000348538 | 1.67E-05 | -0.0947 | 0 | 0.515 |
| ENSP00000361625 | 1.34E-05 | -0.0947 | 0 | 0.040 |
| ENSP00000383303 | 1.63E-05 | -0.0947 | 0 | 0.373 |
| ENSP00000322427 | 9.43E-06 | -0.0948 | 189 | 0.696 |
| ENSP00000306425 | 1.44E-05 | -0.0948 | 208 | 0.259 |
| ENSP00000283628 | 2.54E-05 | -0.0948 | 0 | 0.623 |
| ENSP00000359990 | 8.70E-06 | -0.0948 | 214 | 0.527 |
| ENSP00000262643 | 3.85E-05 | -0.0948 | 270 | 0.797 |
| ENSP00000369989 | 1.17E-05 | -0.0949 | 0 | 0.217 |
| ENSP00000269033 | 1.52E-05 | -0.0949 | 257 | 0.665 |
| ENSP00000331462 | 1.35E-05 | -0.0949 | 588 | 0.777 |
| ENSP00000360320 | 1.05E-05 | -0.0949 | 0 | 0.000 |
| ENSP00000369860 | 3.26E-05 | -0.0949 | 413 | 0.130 |
| ENSP00000348206 | 2.07E-05 | -0.0949 | 0 | 0.000 |
| ENSP00000280155 | 2.20E-05 | -0.0949 | 407 | 0.571 |
| ENSP00000364609 | 1.37E-05 | -0.0949 | 512 | 0.067 |
| ENSP00000365745 | 3.21E-05 | -0.0949 | 444 | 0.817 |
| ENSP00000261722 | 3.91E-06 | -0.0949 | 0 | 0.205 |
| ENSP00000351250 | 3.89E-05 | -0.0949 | 325 | 0.846 |
| ENSP00000382423 | 2.52E-05 | -0.0950 | 263 | 0.854 |
| ENSP00000305919 | 9.28E-06 | -0.0950 | 340 | 0.387 |
| ENSP00000302239 | 1.02E-05 | -0.0950 | 223 | 0.402 |
| ENSP00000215555 | 5.37E-05 | -0.0950 | 214 | 0.400 |
| ENSP00000370803 | 2.01E-05 | -0.0950 | 407 | 0.128 |
| ENSP00000354850 | 1.27E-05 | -0.0950 | 0 | 0.485 |
| ENSP00000399518 | 1.15E-05 | -0.0950 | 151 | 0.800 |
| ENSP00000326042 | 3.03E-05 | -0.0950 | 328 | 0.643 |
| ENSP00000251473 | 6.25E-06 | -0.0950 | 0 | 0.000 |
| ENSP00000349696 | 2.75E-05 | -0.0950 | 183 | 0.154 |
| ENSP00000341344 | 6.42E-06 | -0.0950 | 156 | 0.213 |
| ENSP00000330658 | 1.15E-04 | -0.0950 | 349 | 0.622 |
| ENSP00000304401 | 1.29E-05 | -0.0950 | 0 | 0.194 |
| ENSP00000337008 | 8.91E-06 | -0.0951 | 150 | 0.666 |
| ENSP00000234038 | 1.12E-05 | -0.0951 | 498 | 0.470 |
| ENSP00000357622 | 1.11E-05 | -0.0951 | 0 | 0.790 |
| ENSP00000348527 | 1.97E-05 | -0.0951 | 0 | 0.275 |
| ENSP00000345719 | 1.64E-05 | -0.0951 | 208 | 0.395 |
| ENSP00000314480 | 6.93E-06 | -0.0951 | 0 | 0.807 |
| ENSP00000212015 | 1.63E-05 | -0.0952 | 877 | 0.784 |

| | | | | |
|---|---|---|---|---|
| ENSP00000336894 | 1.88E-05 | -0.0952 | 0 | 0.685 |
| ENSP00000355343 | 1.73E-05 | -0.0952 | 0 | 0.197 |
| ENSP00000294339 | 4.12E-05 | -0.0952 | 228 | 0.851 |
| ENSP00000384160 | 2.84E-05 | -0.0952 | 0 | 0.116 |
| ENSP00000347005 | 2.11E-05 | -0.0952 | 481 | 0.000 |
| ENSP00000380747 | 1.16E-05 | -0.0952 | 0 | 0.071 |
| ENSP00000216923 | 8.65E-06 | -0.0952 | 177 | 0.772 |
| ENSP00000320797 | 1.39E-05 | -0.0952 | 192 | 0.440 |
| ENSP00000292408 | 1.74E-05 | -0.0952 | 216 | 0.685 |
| ENSP00000335042 | 4.62E-06 | -0.0952 | 0 | 0.695 |
| ENSP00000267328 | 5.09E-06 | -0.0952 | 0 | 0.222 |
| ENSP00000362979 | 8.82E-06 | -0.0952 | 0 | 0.692 |
| ENSP00000290810 | 1.48E-05 | -0.0952 | 266 | 0.497 |
| ENSP00000376822 | 8.86E-06 | -0.0953 | 181 | 0.118 |
| ENSP00000359356 | 9.99E-06 | -0.0953 | 0 | 0.161 |
| ENSP00000255324 | 5.40E-06 | -0.0953 | 0 | 0.178 |
| ENSP00000333802 | 4.51E-06 | -0.0953 | 0 | 0.702 |
| ENSP00000337555 | 4.74E-06 | -0.0953 | 0 | 0.696 |
| ENSP00000371230 | 4.26E-05 | -0.0953 | 206 | 0.437 |
| ENSP00000357982 | 3.10E-05 | -0.0953 | 359 | 0.785 |
| ENSP00000470965 | 2.85E-06 | -0.0954 | 0 | 0.000 |
| ENSP00000266659 | 1.60E-05 | -0.0954 | 0 | 0.136 |
| ENSP00000332274 | 1.72E-05 | -0.0954 | 0 | 0.090 |
| ENSP00000425257 | 2.07E-06 | -0.0954 | 0 | 0.000 |
| ENSP00000382779 | 2.34E-05 | -0.0954 | 416 | 0.218 |
| ENSP00000329365 | 4.67E-06 | -0.0954 | 0 | 0.000 |
| ENSP00000358794 | 2.64E-05 | -0.0954 | 0 | 0.180 |
| ENSP00000350071 | 1.59E-05 | -0.0954 | 163 | 0.224 |
| ENSP00000216160 | 2.68E-05 | -0.0954 | 348 | 0.835 |
| ENSP00000371362 | 1.11E-05 | -0.0954 | 0 | 0.000 |
| ENSP00000358862 | 1.17E-05 | -0.0954 | 0 | 0.800 |
| ENSP00000385083 | 1.28E-05 | -0.0955 | 0 | 0.090 |
| ENSP00000259569 | 4.20E-05 | -0.0955 | 320 | 0.809 |
| ENSP00000348298 | 1.02E-05 | -0.0955 | 186 | 0.682 |
| ENSP00000324025 | 9.68E-06 | -0.0955 | 0 | 0.126 |
| ENSP00000355929 | 1.94E-05 | -0.0955 | 157 | 0.691 |
| ENSP00000464743 | 6.87E-06 | -0.0955 | 0 | 0.000 |
| ENSP00000350369 | 6.49E-06 | -0.0955 | 0 | 0.659 |
| ENSP00000373864 | 2.13E-05 | -0.0955 | 207 | 0.815 |
| ENSP00000369755 | 1.02E-05 | -0.0955 | 0 | 0.179 |
| ENSP00000373594 | 4.22E-06 | -0.0955 | 0 | 0.000 |
| ENSP00000320147 | 4.13E-05 | -0.0955 | 325 | 0.810 |
| ENSP00000303508 | 6.33E-06 | -0.0955 | 0 | 0.086 |
| ENSP00000294383 | 5.31E-06 | -0.0956 | 0 | 0.379 |
| ENSP00000307697 | 5.92E-06 | -0.0956 | 0 | 0.414 |
| ENSP00000362918 | 1.56E-05 | -0.0956 | 226 | 0.401 |

| | | | | |
|---|---|---|---|---|
| ENSP00000350198 | 1.47E-05 | -0.0956 | 227 | 0.704 |
| ENSP00000362013 | 2.12E-05 | -0.0956 | 0 | 0.586 |
| ENSP00000362637 | 1.62E-05 | -0.0956 | 0 | 0.154 |
| ENSP00000377504 | 3.73E-06 | -0.0956 | 0 | 0.686 |
| ENSP00000375975 | 1.40E-05 | -0.0956 | 0 | 0.156 |
| ENSP00000359606 | 1.78E-05 | -0.0956 | 0 | 0.594 |
| ENSP00000284268 | 1.20E-04 | -0.0956 | 206 | 0.712 |
| ENSP00000261647 | 1.12E-05 | -0.0956 | 316 | 0.491 |
| ENSP00000257068 | 1.40E-05 | -0.0956 | 304 | 0.688 |
| ENSP00000360473 | 2.29E-05 | -0.0956 | 277 | 0.499 |
| ENSP00000321812 | 4.73E-06 | -0.0956 | 0 | 0.697 |
| ENSP00000376139 | 2.29E-05 | -0.0956 | 0 | 0.176 |
| ENSP00000346440 | 2.70E-05 | -0.0956 | 240 | 0.870 |
| ENSP00000348886 | 1.00E-05 | -0.0957 | 689 | 0.331 |
| ENSP00000247668 | 6.01E-05 | -0.0957 | 409 | 0.765 |
| ENSP00000370408 | 2.76E-05 | -0.0957 | 181 | 0.887 |
| ENSP00000306163 | 8.29E-06 | -0.0957 | 202 | 0.741 |
| ENSP00000322899 | 4.74E-06 | -0.0957 | 0 | 0.700 |
| ENSP00000340132 | 4.42E-06 | -0.0957 | 0 | 0.697 |
| ENSP00000279463 | 1.01E-05 | -0.0957 | 0 | 0.660 |
| ENSP00000322265 | 4.72E-06 | -0.0957 | 0 | 0.697 |
| ENSP00000358903 | 2.66E-05 | -0.0957 | 242 | 0.772 |
| ENSP00000403476 | 5.61E-06 | -0.0957 | 0 | 0.187 |
| ENSP00000274026 | 3.09E-05 | -0.0957 | 313 | 0.764 |
| ENSP00000336799 | 3.37E-05 | -0.0957 | 335 | 0.662 |
| ENSP00000384823 | 1.15E-05 | -0.0958 | 0 | 0.464 |
| ENSP00000360157 | 1.62E-05 | -0.0958 | 159 | 0.000 |
| ENSP00000380087 | 3.51E-06 | -0.0958 | 0 | 0.685 |
| ENSP00000341165 | 4.28E-06 | -0.0958 | 0 | 0.692 |
| ENSP00000375736 | 2.12E-05 | -0.0958 | 849 | 0.000 |
| ENSP00000340347 | 2.37E-05 | -0.0958 | 354 | 0.860 |
| ENSP00000350708 | 4.88E-05 | -0.0958 | 286 | 0.793 |
| ENSP00000358092 | 1.97E-05 | -0.0958 | 215 | 0.853 |
| ENSP00000272091 | 8.03E-05 | -0.0958 | 509 | 0.417 |
| ENSP00000340684 | 1.71E-05 | -0.0959 | 439 | 0.000 |
| ENSP00000265372 | 2.28E-05 | -0.0959 | 226 | 0.897 |
| ENSP00000352627 | 8.23E-06 | -0.0959 | 0 | 0.996 |
| ENSP00000382595 | 1.26E-05 | -0.0959 | 243 | 0.607 |
| ENSP00000365019 | 3.10E-05 | -0.0959 | 349 | 0.466 |
| ENSP00000401477 | 3.53E-06 | -0.0959 | 0 | 0.115 |
| ENSP00000253339 | 1.18E-05 | -0.0959 | 490 | 0.676 |
| ENSP00000341882 | 1.17E-05 | -0.0959 | 195 | 0.424 |
| ENSP00000269740 | 2.69E-06 | -0.0959 | 0 | 0.000 |
| ENSP00000386653 | 4.96E-06 | -0.0959 | 0 | 0.000 |
| ENSP00000363970 | 2.82E-05 | -0.0959 | 786 | 0.601 |
| ENSP00000300134 | 3.54E-05 | -0.0959 | 871 | 0.939 |

| | | | | |
|---|---|---|---|---|
| ENSP00000266987 | 1.11E-05 | -0.0960 | 0 | 0.672 |
| ENSP00000313443 | 4.77E-06 | -0.0960 | 0 | 0.697 |
| ENSP00000311221 | 2.36E-05 | -0.0960 | 0 | 0.152 |
| ENSP00000331313 | 6.76E-06 | -0.0960 | 0 | 0.598 |
| ENSP00000362850 | 3.59E-06 | -0.0960 | 0 | 0.000 |
| ENSP00000365130 | 1.89E-05 | -0.0960 | 159 | 0.754 |
| ENSP00000003100 | 5.59E-05 | -0.0960 | 242 | 0.487 |
| ENSP00000360500 | 1.37E-05 | -0.0960 | 0 | 0.348 |
| ENSP00000388864 | 3.61E-06 | -0.0960 | 0 | 0.689 |
| ENSP00000345102 | 4.12E-06 | -0.0960 | 0 | 0.196 |
| ENSP00000258341 | 9.03E-05 | -0.0960 | 319 | 0.539 |
| ENSP00000373713 | 3.19E-05 | -0.0960 | 575 | 0.198 |
| ENSP00000347169 | 2.33E-05 | -0.0960 | 586 | 0.900 |
| ENSP00000370340 | 1.86E-05 | -0.0960 | 0 | 0.757 |
| ENSP00000288466 | 1.75E-05 | -0.0960 | 0 | 0.135 |
| ENSP00000287538 | 1.21E-05 | -0.0961 | 159 | 0.848 |
| ENSP00000356146 | 1.73E-05 | -0.0961 | 270 | 0.596 |
| ENSP00000352802 | 3.23E-05 | -0.0961 | 195 | 0.484 |
| ENSP00000354649 | 8.09E-06 | -0.0961 | 174 | 0.084 |
| ENSP00000315870 | 4.76E-06 | -0.0961 | 0 | 0.698 |
| ENSP00000351052 | 6.03E-06 | -0.0961 | 0 | 0.116 |
| ENSP00000351552 | 1.80E-05 | -0.0961 | 0 | 0.733 |
| ENSP00000308332 | 3.91E-06 | -0.0961 | 0 | 0.803 |
| ENSP00000261366 | 3.11E-05 | -0.0961 | 591 | 0.716 |
| ENSP00000351684 | 1.30E-05 | -0.0961 | 0 | 0.095 |
| ENSP00000222139 | 3.63E-05 | -0.0961 | 282 | 0.922 |
| ENSP00000343443 | 1.51E-05 | -0.0961 | 167 | 0.669 |
| ENSP00000355777 | 1.71E-05 | -0.0961 | 0 | 0.059 |
| ENSP00000406407 | 2.02E-05 | -0.0962 | 153 | 0.000 |
| ENSP00000400036 | 5.13E-06 | -0.0962 | 0 | 0.000 |
| ENSP00000316426 | 9.07E-06 | -0.0962 | 0 | 0.000 |
| ENSP00000282499 | 2.27E-05 | -0.0962 | 197 | 0.438 |
| ENSP00000331748 | 4.09E-06 | -0.0962 | 0 | 0.235 |
| ENSP00000333980 | 4.56E-06 | -0.0962 | 0 | 0.699 |
| ENSP00000333640 | 1.19E-05 | -0.0962 | 0 | 0.841 |
| ENSP00000364448 | 1.79E-05 | -0.0962 | 205 | 0.835 |
| ENSP00000360166 | 1.45E-05 | -0.0962 | 0 | 0.000 |
| ENSP00000012134 | 8.49E-06 | -0.0962 | 195 | 0.769 |
| ENSP00000306894 | 3.62E-05 | -0.0962 | 342 | 0.880 |
| ENSP00000361229 | 1.31E-05 | -0.0962 | 0 | 0.126 |
| ENSP00000309330 | 4.80E-06 | -0.0962 | 0 | 0.697 |
| ENSP00000303373 | 8.09E-06 | -0.0962 | 0 | 0.992 |
| ENSP00000288943 | 4.98E-06 | -0.0962 | 0 | 0.718 |
| ENSP00000016946 | 6.09E-06 | -0.0962 | 179 | 0.711 |
| ENSP00000355924 | 1.91E-05 | -0.0962 | 244 | 0.924 |
| ENSP00000316357 | 1.16E-05 | -0.0962 | 220 | 0.545 |

| | | | | |
|---|---|---|---|---|
| ENSP00000328251 | 4.42E-05 | -0.0962 | 325 | 0.835 |
| ENSP00000353415 | 3.23E-05 | -0.0963 | 901 | 0.660 |
| ENSP00000348708 | 2.03E-05 | -0.0963 | 195 | 0.832 |
| ENSP00000350278 | 8.19E-06 | -0.0963 | 0 | 0.064 |
| ENSP00000305288 | 1.80E-05 | -0.0963 | 0 | 0.174 |
| ENSP00000355536 | 1.82E-05 | -0.0963 | 262 | 0.257 |
| ENSP00000346300 | 2.22E-05 | -0.0963 | 175 | 0.896 |
| ENSP00000337463 | 9.49E-06 | -0.0963 | 300 | 0.325 |
| ENSP00000351650 | 2.28E-05 | -0.0963 | 0 | 0.000 |
| ENSP00000305221 | 6.55E-06 | -0.0963 | 0 | 0.164 |
| ENSP00000299798 | 8.89E-06 | -0.0963 | 0 | 0.167 |
| ENSP00000384490 | 1.04E-05 | -0.0963 | 0 | 0.806 |
| ENSP00000316589 | 5.19E-06 | -0.0964 | 0 | 0.179 |
| ENSP00000464833 | 1.70E-07 | -0.0964 | 0 | 0.000 |
| ENSP00000324056 | 4.65E-06 | -0.0964 | 0 | 0.694 |
| ENSP00000458238 | 3.18E-05 | -0.0964 | 226 | 0.000 |
| ENSP00000374185 | 4.61E-06 | -0.0964 | 0 | 0.679 |
| ENSP00000363135 | 1.62E-05 | -0.0964 | 155 | 0.075 |
| ENSP00000377428 | 1.98E-05 | -0.0964 | 0 | 0.000 |
| ENSP00000339957 | 7.01E-06 | -0.0964 | 0 | 0.319 |
| ENSP00000378504 | 2.02E-05 | -0.0965 | 237 | 0.750 |
| ENSP00000282286 | 1.00E-05 | -0.0965 | 806 | 0.679 |
| ENSP00000257745 | 2.44E-05 | -0.0965 | 516 | 0.000 |
| ENSP00000258761 | 1.58E-04 | -0.0965 | 157 | 0.271 |
| ENSP00000300151 | 7.55E-06 | -0.0965 | 388 | 0.762 |
| ENSP00000364519 | 2.15E-05 | -0.0965 | 163 | 0.794 |
| ENSP00000327718 | 1.57E-05 | -0.0965 | 0 | 0.139 |
| ENSP00000382584 | 1.04E-06 | -0.0965 | 0 | 0.178 |
| ENSP00000339115 | 8.92E-06 | -0.0965 | 202 | 0.690 |
| ENSP00000371432 | 2.10E-05 | -0.0965 | 167 | 0.000 |
| ENSP00000314441 | 1.84E-05 | -0.0965 | 208 | 0.796 |
| ENSP00000334308 | 1.99E-05 | -0.0965 | 338 | 0.400 |
| ENSP00000216241 | 6.08E-06 | -0.0965 | 0 | 0.669 |
| ENSP00000290418 | 2.27E-05 | -0.0965 | 0 | 0.194 |
| ENSP00000358081 | 4.82E-05 | -0.0965 | 586 | 0.591 |
| ENSP00000328708 | 4.70E-06 | -0.0966 | 0 | 0.491 |
| ENSP00000383516 | 1.90E-05 | -0.0966 | 169 | 0.863 |
| ENSP00000377390 | 8.83E-06 | -0.0966 | 0 | 0.331 |
| ENSP00000265382 | 3.51E-06 | -0.0966 | 0 | 0.439 |
| ENSP00000275364 | 1.24E-05 | -0.0966 | 508 | 0.647 |
| ENSP00000352252 | 1.97E-05 | -0.0966 | 837 | 0.939 |
| ENSP00000392466 | 2.38E-05 | -0.0966 | 240 | 0.785 |
| ENSP00000342385 | 9.61E-05 | -0.0966 | 824 | 0.761 |
| ENSP00000356641 | 1.81E-05 | -0.0966 | 527 | 0.441 |
| ENSP00000230340 | 3.30E-06 | -0.0966 | 0 | 0.891 |
| ENSP00000399753 | 1.73E-05 | -0.0966 | 238 | 0.000 |

| | | | | |
|---|---|---|---|---|
| ENSP00000323148 | 1.64E-05 | -0.0966 | 165 | 0.723 |
| ENSP00000351333 | 1.27E-05 | -0.0966 | 0 | 0.173 |
| ENSP00000364840 | 1.48E-05 | -0.0966 | 165 | 0.155 |
| ENSP00000380414 | 1.50E-05 | -0.0966 | 0 | 0.751 |
| ENSP00000359151 | 2.10E-05 | -0.0966 | 328 | 0.536 |
| ENSP00000405987 | 9.09E-06 | -0.0966 | 203 | 0.667 |
| ENSP00000339726 | 5.22E-06 | -0.0966 | 0 | 0.000 |
| ENSP00000387286 | 3.82E-05 | -0.0966 | 507 | 0.300 |
| ENSP00000222270 | 1.08E-05 | -0.0967 | 244 | 0.000 |
| ENSP00000352678 | 1.37E-05 | -0.0967 | 0 | 0.646 |
| ENSP00000305255 | 8.19E-06 | -0.0967 | 0 | 0.208 |
| ENSP00000341151 | 8.66E-06 | -0.0967 | 340 | 0.700 |
| ENSP00000299300 | 1.95E-05 | -0.0967 | 776 | 0.722 |
| ENSP00000353878 | 4.98E-05 | -0.0967 | 554 | 0.923 |
| ENSP00000401947 | 3.69E-06 | -0.0967 | 0 | 0.000 |
| ENSP00000354033 | 8.90E-06 | -0.0967 | 0 | 0.000 |
| ENSP00000375822 | 3.75E-06 | -0.0967 | 0 | 0.687 |
| ENSP00000347153 | 6.66E-06 | -0.0967 | 0 | 0.000 |
| ENSP00000225396 | 1.36E-05 | -0.0967 | 368 | 0.000 |
| ENSP00000359506 | 2.59E-05 | -0.0967 | 299 | 0.614 |
| ENSP00000353564 | 9.39E-06 | -0.0968 | 0 | 0.000 |
| ENSP00000325819 | 4.67E-05 | -0.0968 | 213 | 0.871 |
| ENSP00000033079 | 1.78E-05 | -0.0968 | 0 | 0.354 |
| ENSP00000356859 | 2.16E-05 | -0.0968 | 215 | 0.166 |
| ENSP00000314556 | 9.18E-06 | -0.0968 | 201 | 0.573 |
| ENSP00000350261 | 9.38E-06 | -0.0968 | 0 | 0.000 |
| ENSP00000339585 | 4.53E-06 | -0.0968 | 0 | 0.682 |
| ENSP00000333660 | 4.53E-06 | -0.0968 | 0 | 0.694 |
| ENSP00000298283 | 8.11E-06 | -0.0968 | 430 | 0.751 |
| ENSP00000367299 | 8.54E-06 | -0.0968 | 0 | 0.097 |
| ENSP00000287322 | 7.09E-05 | -0.0968 | 564 | 0.663 |
| ENSP00000302485 | 1.41E-05 | -0.0968 | 478 | 0.611 |
| ENSP00000225719 | 2.52E-04 | -0.0968 | 543 | 0.085 |
| ENSP00000341785 | 7.80E-05 | -0.0968 | 398 | 0.667 |
| ENSP00000362650 | 1.50E-05 | -0.0969 | 165 | 0.236 |
| ENSP00000293771 | 5.35E-06 | -0.0969 | 0 | 0.716 |
| ENSP00000262435 | 1.14E-05 | -0.0969 | 263 | 0.745 |
| ENSP00000380876 | 1.40E-05 | -0.0969 | 263 | 0.396 |
| ENSP00000263574 | 5.32E-05 | -0.0969 | 361 | 0.769 |
| ENSP00000290759 | 7.15E-06 | -0.0969 | 0 | 0.808 |
| ENSP00000361463 | 5.24E-06 | -0.0969 | 0 | 0.000 |
| ENSP00000351631 | 1.14E-05 | -0.0969 | 0 | 0.175 |
| ENSP00000316729 | 2.36E-05 | -0.0969 | 389 | 0.593 |
| ENSP00000358799 | 2.41E-05 | -0.0969 | 487 | 0.524 |
| ENSP00000375107 | 5.98E-06 | -0.0969 | 0 | 0.157 |
| ENSP00000329738 | 4.58E-06 | -0.0969 | 0 | 0.688 |

| | | | | |
|---|---|---|---|---|
| ENSP00000256001 | 1.27E-05 | -0.0969 | 316 | 0.667 |
| ENSP00000317125 | 4.67E-06 | -0.0969 | 0 | 0.694 |
| ENSP00000356989 | 2.36E-05 | -0.0970 | 189 | 0.385 |
| ENSP00000371689 | 1.44E-05 | -0.0970 | 0 | 0.094 |
| ENSP00000242104 | 7.50E-06 | -0.0970 | 211 | 0.000 |
| ENSP00000333595 | 4.47E-06 | -0.0970 | 0 | 0.000 |
| ENSP00000357887 | 2.33E-05 | -0.0970 | 423 | 0.877 |
| ENSP00000315713 | 1.65E-05 | -0.0970 | 185 | 0.689 |
| ENSP00000261465 | 4.11E-05 | -0.0970 | 634 | 0.457 |
| ENSP00000321679 | 7.89E-06 | -0.0970 | 202 | 0.660 |
| ENSP00000363071 | 1.96E-05 | -0.0970 | 900 | 0.283 |
| ENSP00000248673 | 7.16E-05 | -0.0970 | 361 | 0.000 |
| ENSP00000331540 | 4.78E-06 | -0.0971 | 0 | 0.698 |
| ENSP00000364946 | 1.32E-05 | -0.0971 | 213 | 0.774 |
| ENSP00000366410 | 2.24E-05 | -0.0971 | 252 | 0.466 |
| ENSP00000442308 | 2.60E-05 | -0.0971 | 266 | 0.815 |
| ENSP00000380293 | 3.50E-06 | -0.0971 | 0 | 0.693 |
| ENSP00000398637 | 4.07E-06 | -0.0971 | 0 | 0.156 |
| ENSP00000381148 | 2.49E-05 | -0.0971 | 511 | 0.447 |
| ENSP00000302478 | 4.79E-06 | -0.0971 | 0 | 0.794 |
| ENSP00000376306 | 8.40E-06 | -0.0971 | 0 | 0.223 |
| ENSP00000317872 | 1.20E-05 | -0.0971 | 0 | 0.486 |
| ENSP00000325002 | 6.46E-06 | -0.0972 | 171 | 0.189 |
| ENSP00000340841 | 4.41E-06 | -0.0972 | 0 | 0.698 |
| ENSP00000358158 | 6.76E-06 | -0.0972 | 0 | 0.988 |
| ENSP00000357873 | 6.23E-06 | -0.0972 | 0 | 0.306 |
| ENSP00000264731 | 2.76E-05 | -0.0972 | 364 | 0.879 |
| ENSP00000354995 | 2.89E-05 | -0.0972 | 811 | 0.667 |
| ENSP00000344393 | 1.84E-05 | -0.0972 | 359 | 0.795 |
| ENSP00000354681 | 3.20E-05 | -0.0972 | 670 | 0.464 |
| ENSP00000327959 | 8.92E-06 | -0.0972 | 0 | 0.764 |
| ENSP00000282282 | 4.98E-06 | -0.0972 | 0 | 0.697 |
| ENSP00000320246 | 5.21E-06 | -0.0972 | 0 | 0.210 |
| ENSP00000345848 | 1.26E-05 | -0.0973 | 0 | 0.479 |
| ENSP00000348216 | 4.33E-06 | -0.0973 | 0 | 0.247 |
| ENSP00000381740 | 2.03E-05 | -0.0973 | 204 | 0.641 |
| ENSP00000332643 | 1.09E-05 | -0.0973 | 204 | 0.511 |
| ENSP00000298910 | 2.58E-05 | -0.0973 | 845 | 0.663 |
| ENSP00000230859 | 1.23E-05 | -0.0973 | 178 | 0.830 |
| ENSP00000366875 | 3.32E-06 | -0.0973 | 0 | 0.216 |
| ENSP00000262630 | 1.26E-05 | -0.0973 | 354 | 0.823 |
| ENSP00000252071 | 1.21E-05 | -0.0974 | 316 | 0.667 |
| ENSP00000379836 | 1.07E-05 | -0.0974 | 0 | 0.094 |
| ENSP00000293471 | 4.69E-06 | -0.0974 | 0 | 0.694 |
| ENSP00000250896 | 1.22E-04 | -0.0974 | 198 | 0.616 |
| ENSP00000349131 | 3.16E-05 | -0.0974 | 302 | 0.288 |

| | | | | |
|---|---|---|---|---|
| ENSP00000339314 | 4.54E-06 | -0.0974 | 0 | 0.699 |
| ENSP00000348510 | 1.33E-05 | -0.0974 | 0 | 0.767 |
| ENSP00000259806 | 7.61E-06 | -0.0974 | 204 | 0.000 |
| ENSP00000260970 | 1.02E-05 | -0.0974 | 531 | 0.686 |
| ENSP00000379042 | 1.22E-05 | -0.0975 | 621 | 0.000 |
| ENSP00000458149 | 2.88E-05 | -0.0975 | 301 | 0.000 |
| ENSP00000262554 | 9.63E-06 | -0.0975 | 238 | 0.395 |
| ENSP00000299267 | 5.83E-06 | -0.0975 | 0 | 0.561 |
| ENSP00000374332 | 1.57E-05 | -0.0975 | 193 | 0.333 |
| ENSP00000267197 | 1.14E-05 | -0.0975 | 244 | 0.928 |
| ENSP00000446121 | 1.51E-05 | -0.0975 | 211 | 0.217 |
| ENSP00000308315 | 6.91E-06 | -0.0975 | 0 | 0.643 |
| ENSP00000256447 | 1.24E-05 | -0.0975 | 293 | 0.681 |
| ENSP00000320096 | 4.66E-06 | -0.0975 | 0 | 0.698 |
| ENSP00000447803 | 3.81E-05 | -0.0975 | 845 | 0.878 |
| ENSP00000355568 | 1.37E-05 | -0.0975 | 0 | 0.489 |
| ENSP00000298743 | 1.10E-04 | -0.0975 | 193 | 0.812 |
| ENSP00000264157 | 6.63E-06 | -0.0976 | 0 | 0.783 |
| ENSP00000278193 | 8.27E-05 | -0.0976 | 254 | 0.219 |
| ENSP00000202788 | 1.34E-04 | -0.0976 | 208 | 0.000 |
| ENSP00000280258 | 1.62E-04 | -0.0976 | 237 | 0.101 |
| ENSP00000407724 | 2.15E-05 | -0.0976 | 302 | 0.679 |
| ENSP00000298510 | 2.04E-05 | -0.0976 | 512 | 0.780 |
| ENSP00000281741 | 7.24E-06 | -0.0976 | 355 | 0.503 |
| ENSP00000254322 | 3.64E-05 | -0.0976 | 955 | 0.779 |
| ENSP00000380351 | 3.03E-06 | -0.0976 | 0 | 0.683 |
| ENSP00000254231 | 2.38E-05 | -0.0976 | 302 | 0.726 |
| ENSP00000310472 | 4.65E-06 | -0.0976 | 0 | 0.692 |
| ENSP00000286398 | 3.42E-06 | -0.0976 | 0 | 0.771 |
| ENSP00000349576 | 8.10E-06 | -0.0976 | 0 | 0.281 |
| ENSP00000322270 | 1.01E-05 | -0.0977 | 0 | 0.000 |
| ENSP00000264995 | 7.66E-06 | -0.0977 | 371 | 0.825 |
| ENSP00000339918 | 6.18E-06 | -0.0977 | 0 | 0.655 |
| ENSP00000380254 | 1.06E-06 | -0.0977 | 0 | 0.000 |
| ENSP00000264255 | 8.39E-06 | -0.0977 | 355 | 0.510 |
| ENSP00000289788 | 4.86E-06 | -0.0977 | 0 | 0.700 |
| ENSP00000256383 | 3.06E-05 | -0.0977 | 636 | 0.775 |
| ENSP00000381272 | 9.71E-06 | -0.0977 | 0 | 0.182 |
| ENSP00000355208 | 7.09E-06 | -0.0977 | 0 | 0.000 |
| ENSP00000376534 | 2.57E-05 | -0.0977 | 246 | 0.927 |
| ENSP00000303766 | 9.65E-06 | -0.0977 | 340 | 0.360 |
| ENSP00000361544 | 1.42E-05 | -0.0977 | 0 | 0.376 |
| ENSP00000341524 | 6.18E-06 | -0.0977 | 0 | 0.456 |
| ENSP00000318690 | 5.06E-06 | -0.0977 | 0 | 0.112 |
| ENSP00000360871 | 1.93E-05 | -0.0977 | 440 | 0.163 |
| ENSP00000240185 | 3.52E-05 | -0.0977 | 412 | 0.808 |

| | | | | |
|---|---|---|---|---|
| ENSP00000246041 | 1.66E-05 | -0.0977 | 0 | 0.139 |
| ENSP00000261200 | 9.10E-06 | -0.0977 | 160 | 0.359 |
| ENSP00000354451 | 3.71E-05 | -0.0977 | 292 | 0.351 |
| ENSP00000246062 | 1.24E-05 | -0.0977 | 757 | 0.803 |
| ENSP00000357402 | 3.99E-06 | -0.0978 | 0 | 0.129 |
| ENSP00000231021 | 1.47E-05 | -0.0978 | 0 | 0.133 |
| ENSP00000217964 | 1.50E-05 | -0.0978 | 903 | 0.749 |
| ENSP00000301838 | 4.12E-05 | -0.0978 | 236 | 0.857 |
| ENSP00000299194 | 6.22E-06 | -0.0978 | 0 | 0.658 |
| ENSP00000359976 | 1.67E-05 | -0.0978 | 812 | 0.330 |
| ENSP00000237289 | 4.35E-05 | -0.0978 | 241 | 0.856 |
| ENSP00000368414 | 1.22E-05 | -0.0978 | 0 | 0.107 |
| ENSP00000397435 | 1.60E-05 | -0.0978 | 192 | 0.657 |
| ENSP00000428541 | 3.77E-06 | -0.0978 | 0 | 0.145 |
| ENSP00000288207 | 1.59E-05 | -0.0979 | 191 | 0.685 |
| ENSP00000257430 | 1.39E-05 | -0.0979 | 167 | 0.820 |
| ENSP00000353073 | 1.60E-05 | -0.0979 | 158 | 0.000 |
| ENSP00000287380 | 9.38E-05 | -0.0979 | 242 | 0.000 |
| ENSP00000354911 | 1.47E-05 | -0.0979 | 0 | 0.291 |
| ENSP00000376910 | 5.41E-06 | -0.0979 | 0 | 0.489 |
| ENSP00000417517 | 2.66E-05 | -0.0979 | 329 | 0.000 |
| ENSP00000373700 | 3.89E-05 | -0.0979 | 309 | 0.944 |
| ENSP00000356567 | 3.88E-06 | -0.0979 | 0 | 0.100 |
| ENSP00000362010 | 2.12E-05 | -0.0979 | 489 | 0.561 |
| ENSP00000075120 | 2.83E-05 | -0.0979 | 179 | 0.488 |
| ENSP00000246529 | 5.28E-06 | -0.0979 | 0 | 0.655 |
| ENSP00000270014 | 4.67E-06 | -0.0979 | 0 | 0.698 |
| ENSP00000264709 | 7.64E-06 | -0.0980 | 163 | 0.769 |
| ENSP00000380941 | 1.86E-06 | -0.0980 | 0 | 0.000 |
| ENSP00000416892 | 2.00E-05 | -0.0980 | 211 | 0.621 |
| ENSP00000328494 | 3.82E-06 | -0.0980 | 0 | 0.233 |
| ENSP00000371634 | 1.19E-05 | -0.0980 | 163 | 0.598 |
| ENSP00000360922 | 2.44E-05 | -0.0980 | 348 | 0.835 |
| ENSP00000302896 | 7.68E-06 | -0.0980 | 450 | 0.865 |
| ENSP00000276390 | 4.90E-06 | -0.0980 | 0 | 0.300 |
| ENSP00000328245 | 4.44E-06 | -0.0981 | 0 | 0.689 |
| ENSP00000302770 | 6.59E-06 | -0.0981 | 224 | 0.722 |
| ENSP00000341992 | 8.94E-06 | -0.0981 | 0 | 0.536 |
| ENSP00000369268 | 1.52E-05 | -0.0981 | 165 | 0.289 |
| ENSP00000249842 | 5.95E-06 | -0.0981 | 0 | 0.683 |
| ENSP00000341528 | 4.40E-06 | -0.0981 | 0 | 0.692 |
| ENSP00000381780 | 7.40E-06 | -0.0981 | 0 | 0.186 |
| ENSP00000369055 | 1.06E-05 | -0.0981 | 0 | 0.143 |
| ENSP00000345681 | 3.12E-05 | -0.0981 | 866 | 0.000 |
| ENSP00000353210 | 2.23E-05 | -0.0981 | 274 | 0.488 |
| ENSP00000219169 | 3.05E-05 | -0.0982 | 163 | 0.618 |

| | | | | |
|---|---|---|---|---|
| ENSP00000442656 | 2.85E-05 | -0.0982 | 325 | 0.895 |
| ENSP00000345752 | 9.20E-06 | -0.0982 | 0 | 0.529 |
| ENSP00000366306 | 4.58E-05 | -0.0982 | 457 | 0.929 |
| ENSP00000292431 | 6.63E-06 | -0.0982 | 0 | 0.725 |
| ENSP00000385057 | 1.12E-05 | -0.0982 | 163 | 0.598 |
| ENSP00000341662 | 6.86E-06 | -0.0982 | 0 | 0.220 |
| ENSP00000369996 | 4.42E-06 | -0.0982 | 0 | 0.125 |
| ENSP00000306351 | 5.37E-06 | -0.0982 | 0 | 0.702 |
| ENSP00000378887 | 8.84E-06 | -0.0982 | 0 | 0.154 |
| ENSP00000314792 | 1.82E-05 | -0.0982 | 200 | 0.952 |
| ENSP00000222567 | 4.70E-06 | -0.0982 | 0 | 0.794 |
| ENSP00000280154 | 4.59E-05 | -0.0982 | 351 | 0.843 |
| ENSP00000323795 | 4.17E-06 | -0.0982 | 0 | 0.204 |
| ENSP00000294543 | 2.26E-06 | -0.0982 | 0 | 0.000 |
| ENSP00000355884 | 1.48E-05 | -0.0982 | 0 | 0.696 |
| ENSP00000360939 | 6.99E-06 | -0.0982 | 0 | 0.478 |
| ENSP00000296783 | 2.08E-05 | -0.0983 | 159 | 0.748 |
| ENSP00000296795 | 3.60E-05 | -0.0983 | 388 | 0.905 |
| ENSP00000369681 | 6.86E-06 | -0.0983 | 0 | 0.843 |
| ENSP00000366365 | 8.12E-07 | -0.0983 | 0 | 0.216 |
| ENSP00000219476 | 3.13E-05 | -0.0983 | 252 | 0.903 |
| ENSP00000298119 | 5.05E-06 | -0.0983 | 0 | 0.655 |
| ENSP00000420419 | 1.36E-05 | -0.0983 | 254 | 0.227 |
| ENSP00000333001 | 9.95E-06 | -0.0983 | 0 | 0.000 |
| ENSP00000383059 | 1.43E-05 | -0.0983 | 0 | 0.139 |
| ENSP00000314311 | 1.83E-05 | -0.0983 | 240 | 0.929 |
| ENSP00000390224 | 3.31E-06 | -0.0983 | 0 | 0.000 |
| ENSP00000364929 | 2.56E-05 | -0.0983 | 203 | 0.910 |
| ENSP00000362298 | 9.53E-06 | -0.0983 | 0 | 0.178 |
| ENSP00000359685 | 1.25E-05 | -0.0984 | 0 | 0.070 |
| ENSP00000365198 | 1.90E-05 | -0.0984 | 0 | 0.142 |
| ENSP00000296257 | 1.08E-05 | -0.0984 | 0 | 0.560 |
| ENSP00000338160 | 3.75E-05 | -0.0984 | 351 | 0.000 |
| ENSP00000369858 | 2.51E-05 | -0.0984 | 392 | 0.458 |
| ENSP00000329793 | 6.00E-06 | -0.0984 | 0 | 0.698 |
| ENSP00000334853 | 5.33E-06 | -0.0984 | 0 | 0.687 |
| ENSP00000310668 | 6.87E-05 | -0.0984 | 266 | 0.479 |
| ENSP00000356999 | 2.66E-05 | -0.0984 | 414 | 0.843 |
| ENSP00000278715 | 1.43E-05 | -0.0984 | 192 | 0.407 |
| ENSP00000264424 | 4.88E-06 | -0.0984 | 0 | 0.506 |
| ENSP00000265990 | 4.07E-05 | -0.0984 | 284 | 0.901 |
| ENSP00000328968 | 8.45E-06 | -0.0984 | 151 | 0.494 |
| ENSP00000407425 | 8.56E-08 | -0.0985 | 0 | 0.000 |
| ENSP00000276079 | 4.19E-05 | -0.0985 | 539 | 0.743 |
| ENSP00000353098 | 4.03E-06 | -0.0985 | 0 | 0.567 |
| ENSP00000330341 | 3.68E-05 | -0.0985 | 421 | 0.948 |

| | | | | |
|---|---|---|---|---|
| ENSP00000368666 | 4.71E-06 | -0.0985 | 0 | 0.149 |
| ENSP00000291890 | 1.42E-05 | -0.0985 | 0 | 0.115 |
| ENSP00000222308 | 7.29E-06 | -0.0985 | 326 | 0.715 |
| ENSP00000327427 | 5.51E-06 | -0.0986 | 0 | 0.703 |
| ENSP00000221403 | 4.99E-06 | -0.0986 | 183 | 0.000 |
| ENSP00000378338 | 4.13E-05 | -0.0986 | 902 | 0.698 |
| ENSP00000263168 | 3.81E-05 | -0.0986 | 253 | 0.389 |
| ENSP00000307272 | 9.49E-06 | -0.0986 | 296 | 0.810 |
| ENSP00000354837 | 9.00E-06 | -0.0986 | 0 | 0.449 |
| ENSP00000383911 | 5.24E-06 | -0.0986 | 0 | 0.119 |
| ENSP00000334563 | 2.00E-05 | -0.0986 | 244 | 0.414 |
| ENSP00000264714 | 3.98E-06 | -0.0987 | 0 | 0.000 |
| ENSP00000285039 | 1.47E-05 | -0.0987 | 434 | 0.565 |
| ENSP00000311957 | 4.97E-06 | -0.0987 | 0 | 0.690 |
| ENSP00000362335 | 1.11E-05 | -0.0987 | 159 | 0.203 |
| ENSP00000341280 | 1.13E-05 | -0.0987 | 0 | 0.756 |
| ENSP00000320050 | 4.42E-06 | -0.0987 | 0 | 0.709 |
| ENSP00000420075 | 6.64E-06 | -0.0987 | 0 | 0.000 |
| ENSP00000216237 | 7.12E-06 | -0.0987 | 0 | 0.718 |
| ENSP00000251269 | 5.13E-06 | -0.0987 | 0 | 0.695 |
| ENSP00000324827 | 7.86E-06 | -0.0987 | 0 | 0.411 |
| ENSP00000364235 | 1.11E-05 | -0.0987 | 227 | 0.468 |
| ENSP00000254657 | 5.63E-05 | -0.0988 | 260 | 0.822 |
| ENSP00000366847 | 7.50E-08 | -0.0988 | 0 | 0.000 |
| ENSP00000348822 | 4.59E-05 | -0.0988 | 158 | 0.668 |
| ENSP00000354206 | 3.07E-06 | -0.0988 | 0 | 0.694 |
| ENSP00000406957 | 7.50E-08 | -0.0988 | 0 | 0.000 |
| ENSP00000264345 | 1.67E-05 | -0.0988 | 177 | 0.616 |
| ENSP00000301093 | 3.95E-06 | -0.0988 | 0 | 0.686 |
| ENSP00000261560 | 5.12E-06 | -0.0988 | 0 | 0.694 |
| ENSP00000358154 | 2.06E-05 | -0.0988 | 702 | 0.959 |
| ENSP00000340330 | 2.55E-05 | -0.0988 | 320 | 0.000 |
| ENSP00000368523 | 9.33E-06 | -0.0989 | 718 | 0.261 |
| ENSP00000262948 | 1.65E-05 | -0.0989 | 250 | 0.660 |
| ENSP00000245919 | 2.05E-05 | -0.0989 | 945 | 0.000 |
| ENSP00000356562 | 9.25E-06 | -0.0989 | 0 | 0.083 |
| ENSP00000362441 | 2.82E-05 | -0.0989 | 297 | 0.897 |
| ENSP00000264065 | 9.88E-06 | -0.0989 | 999 | 0.530 |
| ENSP00000300870 | 4.46E-06 | -0.0989 | 0 | 0.687 |
| ENSP00000291715 | 1.34E-05 | -0.0989 | 0 | 0.118 |
| ENSP00000324729 | 3.88E-05 | -0.0989 | 196 | 0.279 |
| ENSP00000320821 | 4.02E-06 | -0.0989 | 0 | 0.214 |
| ENSP00000262891 | 1.14E-05 | -0.0989 | 165 | 0.672 |
| ENSP00000342007 | 9.33E-06 | -0.0989 | 205 | 0.477 |
| ENSP00000264183 | 8.29E-06 | -0.0989 | 195 | 0.000 |
| ENSP00000295208 | 4.65E-06 | -0.0989 | 0 | 0.687 |

| | | | | |
|---|---|---|---|---|
| ENSP00000319060 | 1.16E-05 | -0.0990 | 181 | 0.615 |
| ENSP00000296091 | 5.74E-06 | -0.0990 | 0 | 0.698 |
| ENSP00000367139 | 1.26E-05 | -0.0990 | 215 | 0.100 |
| ENSP00000261819 | 5.83E-06 | -0.0990 | 0 | 0.666 |
| ENSP00000359018 | 2.15E-06 | -0.0990 | 0 | 0.206 |
| ENSP00000336790 | 3.70E-05 | -0.0990 | 987 | 0.878 |
| ENSP00000359095 | 2.75E-05 | -0.0990 | 205 | 0.843 |
| ENSP00000363149 | 2.06E-05 | -0.0990 | 814 | 0.572 |
| ENSP00000366731 | 7.50E-08 | -0.0990 | 0 | 0.000 |
| ENSP00000355249 | 6.88E-06 | -0.0990 | 0 | 0.779 |
| ENSP00000259727 | 8.07E-06 | -0.0990 | 330 | 0.625 |
| ENSP00000255746 | 4.99E-06 | -0.0991 | 0 | 0.692 |
| ENSP00000268150 | 1.49E-05 | -0.0991 | 0 | 0.437 |
| ENSP00000401303 | 4.15E-05 | -0.0991 | 400 | 0.956 |
| ENSP00000358795 | 3.92E-05 | -0.0991 | 560 | 0.000 |
| ENSP00000396673 | 3.28E-05 | -0.0991 | 274 | 0.863 |
| ENSP00000384886 | 2.97E-05 | -0.0991 | 901 | 0.581 |
| ENSP00000353458 | 2.47E-05 | -0.0991 | 246 | 0.928 |
| ENSP00000265113 | 4.89E-05 | -0.0991 | 489 | 0.598 |
| ENSP00000455948 | 3.27E-06 | -0.0991 | 0 | 0.098 |
| ENSP00000298299 | 8.34E-06 | -0.0991 | 159 | 0.692 |
| ENSP00000323720 | 1.16E-05 | -0.0991 | 563 | 0.000 |
| ENSP00000259335 | 1.08E-05 | -0.0991 | 233 | 0.444 |
| ENSP00000362994 | 2.20E-05 | -0.0991 | 359 | 0.860 |
| ENSP00000035383 | 4.86E-06 | -0.0991 | 0 | 0.620 |
| ENSP00000385099 | 4.66E-06 | -0.0991 | 0 | 0.690 |
| ENSP00000377900 | 2.40E-05 | -0.0991 | 211 | 0.361 |
| ENSP00000344741 | 5.32E-05 | -0.0991 | 300 | 0.617 |
| ENSP00000320081 | 5.72E-06 | -0.0992 | 0 | 0.071 |
| ENSP00000305244 | 7.06E-06 | -0.0992 | 0 | 0.797 |
| ENSP00000347988 | 2.34E-05 | -0.0992 | 191 | 0.303 |
| ENSP00000308578 | 4.57E-06 | -0.0992 | 0 | 0.697 |
| ENSP00000291598 | 4.56E-06 | -0.0992 | 0 | 0.704 |
| ENSP00000355187 | 6.47E-06 | -0.0992 | 198 | 0.681 |
| ENSP00000263967 | 2.13E-05 | -0.0992 | 337 | 0.923 |
| ENSP00000354346 | 1.08E-05 | -0.0992 | 0 | 0.000 |
| ENSP00000366828 | 6.92E-06 | -0.0992 | 0 | 0.740 |
| ENSP00000229179 | 5.00E-06 | -0.0992 | 151 | 0.000 |
| ENSP00000296509 | 1.41E-05 | -0.0992 | 177 | 0.583 |
| ENSP00000345809 | 3.82E-06 | -0.0992 | 0 | 0.693 |
| ENSP00000262428 | 1.68E-04 | -0.0992 | 292 | 0.098 |
| ENSP00000365946 | 9.65E-06 | -0.0992 | 0 | 0.983 |
| ENSP00000362728 | 1.79E-05 | -0.0992 | 154 | 0.639 |
| ENSP00000365016 | 1.65E-05 | -0.0992 | 329 | 0.896 |
| ENSP00000346603 | 5.67E-06 | -0.0993 | 0 | 0.677 |
| ENSP00000201031 | 5.92E-05 | -0.0993 | 304 | 0.832 |

| | | | | |
|---|---|---|---|---|
| ENSP00000338807 | 4.57E-06 | -0.0993 | 0 | 0.683 |
| ENSP00000381588 | 2.05E-05 | -0.0993 | 216 | 0.686 |
| ENSP00000436812 | 3.15E-05 | -0.0993 | 854 | 0.666 |
| ENSP00000324124 | 4.89E-06 | -0.0993 | 0 | 0.769 |
| ENSP00000269829 | 5.03E-06 | -0.0993 | 0 | 0.696 |
| ENSP00000302502 | 4.50E-06 | -0.0993 | 0 | 0.692 |
| ENSP00000359301 | 5.75E-06 | -0.0993 | 0 | 0.535 |
| ENSP00000410041 | 8.86E-06 | -0.0993 | 0 | 0.146 |
| ENSP00000281156 | 6.05E-05 | -0.0993 | 386 | 0.804 |
| ENSP00000369217 | 1.56E-05 | -0.0993 | 353 | 0.421 |
| ENSP00000368589 | 9.55E-06 | -0.0993 | 0 | 0.062 |
| ENSP00000429276 | 3.45E-05 | -0.0993 | 154 | 0.109 |
| ENSP00000342215 | 2.51E-05 | -0.0993 | 0 | 0.202 |
| ENSP00000334105 | 6.88E-06 | -0.0994 | 0 | 0.000 |
| ENSP00000357177 | 2.49E-05 | -0.0994 | 631 | 0.646 |
| ENSP00000352834 | 2.29E-05 | -0.0994 | 244 | 0.500 |
| ENSP00000290158 | 3.78E-05 | -0.0994 | 675 | 0.808 |
| ENSP00000296144 | 8.20E-06 | -0.0994 | 480 | 0.644 |
| ENSP00000223459 | 5.11E-06 | -0.0994 | 0 | 0.700 |
| ENSP00000232424 | 5.14E-05 | -0.0994 | 232 | 0.839 |
| ENSP00000270649 | 4.17E-06 | -0.0994 | 0 | 0.689 |
| ENSP00000292841 | 4.87E-06 | -0.0994 | 0 | 0.693 |
| ENSP00000270451 | 4.41E-06 | -0.0994 | 0 | 0.694 |
| ENSP00000425421 | 8.98E-06 | -0.0994 | 160 | 0.145 |
| ENSP00000263805 | 4.52E-06 | -0.0995 | 0 | 0.688 |
| ENSP00000385795 | 3.05E-06 | -0.0995 | 0 | 0.770 |
| ENSP00000350580 | 1.71E-05 | -0.0995 | 852 | 0.000 |
| ENSP00000351108 | 2.11E-05 | -0.0995 | 473 | 0.979 |
| ENSP00000384640 | 7.08E-08 | -0.0995 | 0 | 0.000 |
| ENSP00000408984 | 2.10E-05 | -0.0995 | 286 | 0.000 |
| ENSP00000454437 | 5.72E-07 | -0.0995 | 0 | 0.000 |
| ENSP00000380552 | 3.05E-05 | -0.0995 | 198 | 0.421 |
| ENSP00000330374 | 4.47E-06 | -0.0995 | 0 | 0.211 |
| ENSP00000361868 | 3.98E-05 | -0.0995 | 0 | 0.354 |
| ENSP00000288177 | 4.25E-06 | -0.0995 | 0 | 0.000 |
| ENSP00000344431 | 6.39E-06 | -0.0995 | 887 | 0.298 |
| ENSP00000268154 | 4.46E-06 | -0.0995 | 0 | 0.698 |
| ENSP00000315664 | 4.35E-06 | -0.0995 | 0 | 0.684 |
| ENSP00000369643 | 1.19E-05 | -0.0996 | 157 | 0.637 |
| ENSP00000342626 | 5.23E-05 | -0.0996 | 229 | 0.866 |
| ENSP00000340749 | 4.13E-06 | -0.0996 | 0 | 0.686 |
| ENSP00000361608 | 6.75E-06 | -0.0996 | 161 | 0.367 |
| ENSP00000162023 | 8.67E-06 | -0.0996 | 214 | 0.000 |
| ENSP00000258962 | 2.29E-05 | -0.0996 | 370 | 0.864 |
| ENSP00000291842 | 7.23E-05 | -0.0996 | 165 | 0.112 |
| ENSP00000243052 | 7.13E-06 | -0.0996 | 0 | 0.211 |

| | | | | |
|---|---|---|---|---|
| ENSP00000328547 | 4.52E-05 | -0.0996 | 188 | 0.795 |
| ENSP00000364883 | 6.84E-06 | -0.0996 | 0 | 0.265 |
| ENSP00000263056 | 1.39E-05 | -0.0997 | 520 | 0.656 |
| ENSP00000254166 | 5.31E-06 | -0.0997 | 0 | 0.699 |
| ENSP00000178640 | 8.75E-06 | -0.0997 | 335 | 0.661 |
| ENSP00000301071 | 2.65E-05 | -0.0997 | 335 | 0.641 |
| ENSP00000322570 | 5.23E-06 | -0.0997 | 0 | 0.719 |
| ENSP00000342356 | 1.48E-05 | -0.0997 | 158 | 0.185 |
| ENSP00000314649 | 1.05E-05 | -0.0997 | 216 | 0.276 |
| ENSP00000362751 | 6.96E-06 | -0.0997 | 0 | 0.244 |
| ENSP00000417464 | 3.30E-05 | -0.0997 | 175 | 0.892 |
| ENSP00000323945 | 4.42E-06 | -0.0998 | 0 | 0.696 |
| ENSP00000362820 | 1.96E-05 | -0.0998 | 274 | 0.899 |
| ENSP00000321848 | 4.26E-06 | -0.0998 | 0 | 0.695 |
| ENSP00000365503 | 3.81E-06 | -0.0998 | 0 | 0.201 |
| ENSP00000306869 | 4.72E-06 | -0.0998 | 0 | 0.671 |
| ENSP00000266671 | 7.35E-05 | -0.0998 | 311 | 0.589 |
| ENSP00000296223 | 4.23E-06 | -0.0998 | 0 | 0.000 |
| ENSP00000343054 | 7.78E-06 | -0.0998 | 0 | 0.860 |
| ENSP00000332049 | 3.88E-05 | -0.0998 | 343 | 0.909 |
| ENSP00000341236 | 4.04E-06 | -0.0999 | 0 | 0.700 |
| ENSP00000311469 | 3.68E-05 | -0.0999 | 342 | 0.738 |
| ENSP00000319305 | 4.19E-06 | -0.0999 | 0 | 0.696 |
| ENSP00000337368 | 4.69E-06 | -0.0999 | 0 | 0.702 |
| ENSP00000279968 | 1.74E-05 | -0.1000 | 0 | 0.166 |
| ENSP00000408598 | 2.00E-05 | -0.1000 | 186 | 0.905 |
| ENSP00000316032 | 6.96E-06 | -0.1000 | 0 | 0.756 |
| ENSP00000221327 | 4.99E-06 | -0.1000 | 0 | 0.695 |
| ENSP00000331626 | 4.60E-06 | -0.1000 | 0 | 0.696 |
| ENSP00000234396 | 4.49E-06 | -0.1000 | 0 | 0.236 |
| ENSP00000299237 | 5.18E-06 | -0.1000 | 0 | 0.690 |
| ENSP00000282493 | 8.74E-06 | -0.1000 | 190 | 0.000 |
| ENSP00000356198 | 3.02E-05 | -0.1000 | 165 | 0.107 |
| ENSP00000248668 | 5.01E-06 | -0.1000 | 0 | 0.650 |
| ENSP00000274547 | 6.61E-06 | -0.1000 | 196 | 0.577 |
| ENSP00000262294 | 3.81E-05 | -0.1000 | 191 | 0.489 |
| ENSP00000289779 | 5.05E-05 | -0.1001 | 311 | 0.000 |
| ENSP00000332256 | 3.50E-05 | -0.1001 | 472 | 0.404 |
| ENSP00000220659 | 5.40E-06 | -0.1001 | 230 | 0.000 |
| ENSP00000292928 | 4.20E-06 | -0.1001 | 0 | 0.685 |
| ENSP00000374014 | 5.47E-06 | -0.1001 | 0 | 0.117 |
| ENSP00000353007 | 8.05E-06 | -0.1001 | 0 | 0.194 |
| ENSP00000385806 | 3.08E-05 | -0.1001 | 711 | 0.738 |
| ENSP00000243563 | 2.50E-05 | -0.1001 | 599 | 0.701 |
| ENSP00000366902 | 5.69E-06 | -0.1001 | 290 | 0.713 |
| ENSP00000064571 | 6.66E-06 | -0.1001 | 0 | 0.221 |

| | | | | |
|---|---|---|---|---|
| ENSP00000329264 | 4.25E-06 | -0.1001 | 0 | 0.696 |
| ENSP00000316054 | 9.46E-06 | -0.1001 | 640 | 0.890 |
| ENSP00000242804 | 4.82E-06 | -0.1001 | 0 | 0.692 |
| ENSP00000274054 | 8.01E-05 | -0.1001 | 216 | 0.869 |
| ENSP00000337354 | 4.03E-05 | -0.1001 | 349 | 0.327 |
| ENSP00000196489 | 4.72E-06 | -0.1001 | 0 | 0.691 |
| ENSP00000261622 | 3.17E-05 | -0.1001 | 325 | 0.365 |
| ENSP00000450114 | 3.05E-06 | -0.1001 | 0 | 0.184 |
| ENSP00000287701 | 8.43E-06 | -0.1002 | 0 | 0.275 |
| ENSP00000371394 | 1.20E-05 | -0.1002 | 0 | 0.115 |
| ENSP00000411198 | 4.50E-07 | -0.1002 | 0 | 0.189 |
| ENSP00000264360 | 6.23E-06 | -0.1002 | 0 | 0.153 |
| ENSP00000302222 | 8.25E-06 | -0.1002 | 151 | 0.675 |
| ENSP00000348564 | 1.82E-05 | -0.1002 | 284 | 0.348 |
| ENSP00000324605 | 4.05E-06 | -0.1002 | 0 | 0.699 |
| ENSP00000308928 | 3.24E-05 | -0.1002 | 357 | 0.810 |
| ENSP00000345479 | 3.20E-06 | -0.1002 | 0 | 0.683 |
| ENSP00000345985 | 4.22E-06 | -0.1002 | 0 | 0.630 |
| ENSP00000404151 | 1.05E-06 | -0.1002 | 0 | 0.147 |
| ENSP00000362144 | 3.66E-05 | -0.1002 | 195 | 0.433 |
| ENSP00000240731 | 4.38E-06 | -0.1002 | 0 | 0.698 |
| ENSP00000369218 | 2.72E-05 | -0.1003 | 183 | 0.692 |
| ENSP00000336712 | 3.59E-05 | -0.1003 | 282 | 0.582 |
| ENSP00000352400 | 1.85E-05 | -0.1003 | 213 | 0.648 |
| ENSP00000247970 | 1.26E-05 | -0.1003 | 316 | 0.708 |
| ENSP00000378506 | 1.12E-04 | -0.1003 | 0 | 0.127 |
| ENSP00000409126 | 1.44E-05 | -0.1003 | 361 | 0.215 |
| ENSP00000269554 | 4.39E-06 | -0.1003 | 0 | 0.000 |
| ENSP00000358033 | 7.27E-06 | -0.1003 | 0 | 0.340 |
| ENSP00000253003 | 7.11E-06 | -0.1003 | 0 | 0.308 |
| ENSP00000359321 | 9.84E-06 | -0.1003 | 0 | 0.769 |
| ENSP00000317985 | 4.21E-05 | -0.1003 | 280 | 0.832 |
| ENSP00000309871 | 9.59E-06 | -0.1003 | 191 | 0.675 |
| ENSP00000260585 | 5.69E-05 | -0.1003 | 183 | 0.124 |
| ENSP00000383820 | 1.40E-05 | -0.1003 | 150 | 0.619 |
| ENSP00000293981 | 2.59E-04 | -0.1004 | 150 | 0.170 |
| ENSP00000381590 | 4.08E-05 | -0.1004 | 405 | 0.000 |
| ENSP00000273480 | 4.02E-06 | -0.1004 | 0 | 0.425 |
| ENSP00000373884 | 2.50E-05 | -0.1004 | 270 | 0.394 |
| ENSP00000384880 | 1.44E-05 | -0.1004 | 0 | 0.068 |
| ENSP00000286648 | 2.18E-05 | -0.1004 | 218 | 0.885 |
| ENSP00000321835 | 4.02E-05 | -0.1004 | 951 | 0.892 |
| ENSP00000404251 | 2.47E-06 | -0.1004 | 0 | 0.177 |
| ENSP00000325074 | 3.23E-05 | -0.1004 | 278 | 0.596 |
| ENSP00000283141 | 1.12E-05 | -0.1004 | 0 | 0.158 |
| ENSP00000337081 | 4.61E-06 | -0.1004 | 0 | 0.708 |

| | | | | |
|---|---|---|---|---|
| ENSP00000295006 | 9.53E-06 | -0.1005 | 214 | 0.000 |
| ENSP00000308622 | 6.26E-05 | -0.1005 | 663 | 0.665 |
| ENSP00000347719 | 2.81E-05 | -0.1005 | 199 | 0.275 |
| ENSP00000347906 | 2.29E-05 | -0.1005 | 205 | 0.866 |
| ENSP00000455744 | 7.84E-06 | -0.1005 | 173 | 0.000 |
| ENSP00000254029 | 1.25E-04 | -0.1005 | 445 | 0.119 |
| ENSP00000223428 | 4.67E-06 | -0.1005 | 0 | 0.691 |
| ENSP00000310033 | 4.16E-06 | -0.1005 | 0 | 0.701 |
| ENSP00000379133 | 9.21E-06 | -0.1005 | 0 | 0.062 |
| ENSP00000255977 | 1.05E-05 | -0.1005 | 0 | 0.283 |
| ENSP00000255129 | 4.30E-06 | -0.1005 | 0 | 0.693 |
| ENSP00000292579 | 4.59E-06 | -0.1005 | 0 | 0.685 |
| ENSP00000369736 | 1.79E-05 | -0.1005 | 150 | 0.158 |
| ENSP00000350616 | 7.94E-06 | -0.1005 | 0 | 0.302 |
| ENSP00000369395 | 4.72E-06 | -0.1005 | 0 | 0.642 |
| ENSP00000255120 | 4.73E-06 | -0.1005 | 0 | 0.703 |
| ENSP00000362334 | 1.25E-05 | -0.1006 | 545 | 0.653 |
| ENSP00000342818 | 4.46E-06 | -0.1006 | 0 | 0.698 |
| ENSP00000301995 | 5.37E-06 | -0.1006 | 0 | 0.684 |
| ENSP00000263629 | 7.31E-06 | -0.1006 | 376 | 0.698 |
| ENSP00000349428 | 2.13E-05 | -0.1006 | 950 | 0.785 |
| ENSP00000336750 | 6.10E-06 | -0.1006 | 0 | 0.542 |
| ENSP00000339404 | 2.33E-04 | -0.1006 | 171 | 0.121 |
| ENSP00000290691 | 7.65E-06 | -0.1006 | 0 | 0.420 |
| ENSP00000262894 | 5.41E-06 | -0.1006 | 0 | 0.693 |
| ENSP00000282892 | 4.06E-05 | -0.1006 | 216 | 0.726 |
| ENSP00000336543 | 5.40E-06 | -0.1006 | 0 | 0.000 |
| ENSP00000358089 | 2.30E-05 | -0.1006 | 572 | 0.833 |
| ENSP00000364263 | 5.01E-06 | -0.1006 | 0 | 0.194 |
| ENSP00000247930 | 4.39E-06 | -0.1007 | 0 | 0.691 |
| ENSP00000354554 | 2.87E-05 | -0.1007 | 361 | 0.613 |
| ENSP00000454783 | 3.41E-05 | -0.1007 | 335 | 0.378 |
| ENSP00000338093 | 5.02E-06 | -0.1007 | 424 | 0.699 |
| ENSP00000245222 | 9.02E-06 | -0.1007 | 0 | 0.441 |
| ENSP00000355511 | 3.31E-05 | -0.1007 | 267 | 0.372 |
| ENSP00000366563 | 2.87E-05 | -0.1007 | 576 | 0.855 |
| ENSP00000373153 | 9.13E-06 | -0.1007 | 0 | 0.000 |
| ENSP00000362361 | 4.05E-05 | -0.1007 | 485 | 0.791 |
| ENSP00000298288 | 9.01E-06 | -0.1007 | 193 | 0.644 |
| ENSP00000308741 | 5.14E-05 | -0.1007 | 293 | 0.798 |
| ENSP00000379612 | 1.74E-05 | -0.1007 | 163 | 0.727 |
| ENSP00000341094 | 9.99E-06 | -0.1008 | 158 | 0.991 |
| ENSP00000357465 | 5.74E-07 | -0.1008 | 0 | 0.157 |
| ENSP00000326967 | 4.31E-06 | -0.1008 | 0 | 0.688 |
| ENSP00000275423 | 4.48E-06 | -0.1008 | 0 | 0.697 |
| ENSP00000304376 | 4.32E-06 | -0.1008 | 0 | 0.213 |

| | | | | |
|---|---|---|---|---|
| ENSP00000319829 | 4.05E-06 | -0.1008 | 0 | 0.726 |
| ENSP00000358815 | 3.95E-05 | -0.1008 | 560 | 0.748 |
| ENSP00000221494 | 2.86E-05 | -0.1008 | 456 | 0.849 |
| ENSP00000263273 | 5.23E-05 | -0.1008 | 454 | 0.000 |
| ENSP00000278772 | 5.87E-06 | -0.1008 | 0 | 0.712 |
| ENSP00000301547 | 4.03E-06 | -0.1008 | 0 | 0.685 |
| ENSP00000310301 | 1.24E-05 | -0.1008 | 185 | 0.831 |
| ENSP00000264234 | 1.12E-05 | -0.1009 | 0 | 0.122 |
| ENSP00000352438 | 1.69E-05 | -0.1009 | 354 | 0.871 |
| ENSP00000306756 | 5.05E-06 | -0.1009 | 0 | 0.681 |
| ENSP00000356211 | 3.67E-06 | -0.1009 | 0 | 0.138 |
| ENSP00000322872 | 4.50E-06 | -0.1009 | 0 | 0.691 |
| ENSP00000304908 | 7.25E-06 | -0.1009 | 355 | 0.491 |
| ENSP00000271015 | 3.78E-05 | -0.1009 | 208 | 0.000 |
| ENSP00000252211 | 4.63E-06 | -0.1009 | 0 | 0.713 |
| ENSP00000342156 | 2.87E-06 | -0.1009 | 0 | 0.125 |
| ENSP00000437940 | 2.02E-05 | -0.1010 | 301 | 0.880 |
| ENSP00000301215 | 4.45E-06 | -0.1010 | 0 | 0.679 |
| ENSP00000358820 | 4.66E-06 | -0.1010 | 0 | 0.162 |
| ENSP00000249396 | 2.26E-05 | -0.1010 | 234 | 0.825 |
| ENSP00000343785 | 6.60E-05 | -0.1010 | 607 | 0.847 |
| ENSP00000258960 | 5.24E-06 | -0.1010 | 0 | 0.453 |
| ENSP00000379054 | 3.51E-06 | -0.1010 | 0 | 0.000 |
| ENSP00000252840 | 4.34E-06 | -0.1010 | 0 | 0.685 |
| ENSP00000260061 | 5.51E-06 | -0.1010 | 0 | 0.636 |
| ENSP00000377617 | 1.50E-05 | -0.1011 | 243 | 0.516 |
| ENSP00000419126 | 1.58E-06 | -0.1011 | 0 | 0.132 |
| ENSP00000260130 | 5.44E-05 | -0.1011 | 174 | 0.220 |
| ENSP00000310697 | 4.34E-06 | -0.1011 | 0 | 0.699 |
| ENSP00000383398 | 4.72E-06 | -0.1011 | 0 | 0.189 |
| ENSP00000273047 | 2.71E-05 | -0.1011 | 323 | 0.570 |
| ENSP00000356386 | 2.37E-05 | -0.1011 | 211 | 0.164 |
| ENSP00000366384 | 3.65E-06 | -0.1011 | 0 | 0.691 |
| ENSP00000324897 | 2.36E-05 | -0.1012 | 331 | 0.823 |
| ENSP00000332681 | 6.47E-06 | -0.1012 | 247 | 0.518 |
| ENSP00000330320 | 5.10E-06 | -0.1012 | 0 | 0.130 |
| ENSP00000296674 | 7.19E-06 | -0.1012 | 390 | 0.688 |
| ENSP00000356433 | 2.21E-05 | -0.1012 | 325 | 0.484 |
| ENSP00000381490 | 8.49E-06 | -0.1012 | 0 | 0.367 |
| ENSP00000452126 | 1.76E-05 | -0.1012 | 491 | 0.719 |
| ENSP00000297071 | 9.87E-05 | -0.1012 | 216 | 0.693 |
| ENSP00000230354 | 1.79E-05 | -0.1012 | 548 | 0.000 |
| ENSP00000428518 | 1.12E-05 | -0.1012 | 0 | 0.145 |
| ENSP00000349959 | 2.82E-05 | -0.1012 | 252 | 0.877 |
| ENSP00000221722 | 4.31E-06 | -0.1013 | 0 | 0.000 |
| ENSP00000341730 | 5.63E-06 | -0.1013 | 430 | 0.743 |

| | | | | |
|---|---|---|---|---|
| ENSP00000335333 | 7.83E-06 | -0.1013 | 0 | 0.434 |
| ENSP00000317912 | 9.89E-06 | -0.1013 | 166 | 0.000 |
| ENSP00000220592 | 1.76E-05 | -0.1013 | 197 | 0.727 |
| ENSP00000263817 | 9.18E-06 | -0.1013 | 360 | 0.467 |
| ENSP00000294818 | 5.64E-06 | -0.1013 | 0 | 0.659 |
| ENSP00000293813 | 2.72E-05 | -0.1013 | 316 | 0.000 |
| ENSP00000357283 | 2.55E-05 | -0.1014 | 412 | 0.836 |
| ENSP00000196482 | 4.25E-06 | -0.1014 | 0 | 0.691 |
| ENSP00000348215 | 2.88E-05 | -0.1014 | 720 | 0.360 |
| ENSP00000311028 | 7.84E-06 | -0.1014 | 395 | 0.802 |
| ENSP00000375872 | 1.43E-05 | -0.1014 | 195 | 0.515 |
| ENSP00000359212 | 9.28E-06 | -0.1014 | 0 | 0.854 |
| ENSP00000338887 | 5.31E-06 | -0.1014 | 0 | 0.660 |
| ENSP00000382590 | 3.21E-06 | -0.1014 | 0 | 0.192 |
| ENSP00000363921 | 1.78E-05 | -0.1014 | 202 | 0.743 |
| ENSP00000304169 | 1.62E-05 | -0.1014 | 291 | 0.000 |
| ENSP00000321963 | 3.98E-06 | -0.1014 | 0 | 0.702 |
| ENSP00000359424 | 2.56E-05 | -0.1014 | 585 | 0.852 |
| ENSP00000347046 | 2.86E-05 | -0.1015 | 523 | 0.651 |
| ENSP00000255641 | 5.54E-06 | -0.1015 | 0 | 0.395 |
| ENSP00000363556 | 2.81E-06 | -0.1015 | 0 | 0.689 |
| ENSP00000382673 | 2.85E-06 | -0.1015 | 0 | 0.000 |
| ENSP00000365048 | 2.78E-05 | -0.1015 | 328 | 0.919 |
| ENSP00000465021 | 3.13E-07 | -0.1015 | 0 | 0.000 |
| ENSP00000301146 | 9.20E-06 | -0.1015 | 207 | 0.254 |
| ENSP00000351273 | 4.32E-05 | -0.1016 | 961 | 0.917 |
| ENSP00000309710 | 5.26E-06 | -0.1016 | 0 | 0.000 |
| ENSP00000328484 | 9.21E-06 | -0.1016 | 158 | 0.990 |
| ENSP00000333725 | 4.10E-06 | -0.1016 | 0 | 0.687 |
| ENSP00000300128 | 8.65E-05 | -0.1016 | 242 | 0.000 |
| ENSP00000339435 | 1.10E-05 | -0.1016 | 242 | 0.456 |
| ENSP00000355566 | 1.63E-05 | -0.1016 | 212 | 0.310 |
| ENSP00000427550 | 5.29E-06 | -0.1016 | 0 | 0.068 |
| ENSP00000355011 | 1.97E-05 | -0.1017 | 652 | 0.874 |
| ENSP00000196548 | 4.51E-06 | -0.1017 | 0 | 0.000 |
| ENSP00000320566 | 3.68E-05 | -0.1017 | 203 | 0.898 |
| ENSP00000332750 | 4.20E-06 | -0.1017 | 0 | 0.686 |
| ENSP00000254286 | 1.15E-05 | -0.1017 | 449 | 0.691 |
| ENSP00000318898 | 5.18E-06 | -0.1017 | 0 | 0.697 |
| ENSP00000335437 | 4.07E-06 | -0.1017 | 0 | 0.691 |
| ENSP00000250173 | 4.66E-06 | -0.1017 | 0 | 0.462 |
| ENSP00000312141 | 3.88E-06 | -0.1017 | 0 | 0.677 |
| ENSP00000265192 | 2.30E-04 | -0.1017 | 202 | 0.657 |
| ENSP00000261636 | 1.06E-05 | -0.1017 | 286 | 0.465 |
| ENSP00000344782 | 1.07E-05 | -0.1017 | 200 | 0.789 |
| ENSP00000387122 | 1.30E-05 | -0.1017 | 201 | 0.422 |

| | | | | |
|---|---|---|---|---|
| ENSP00000332171 | 2.06E-05 | -0.1017 | 250 | 0.798 |
| ENSP00000351153 | 8.32E-06 | -0.1018 | 195 | 0.000 |
| ENSP00000373637 | 2.10E-05 | -0.1018 | 811 | 0.000 |
| ENSP00000319756 | 5.64E-06 | -0.1018 | 0 | 0.000 |
| ENSP00000438095 | 3.82E-05 | -0.1018 | 267 | 0.000 |
| ENSP00000301073 | 4.53E-06 | -0.1018 | 0 | 0.699 |
| ENSP00000256433 | 6.06E-05 | -0.1018 | 250 | 0.000 |
| ENSP00000399982 | 2.22E-05 | -0.1018 | 200 | 0.867 |
| ENSP00000338770 | 4.17E-06 | -0.1018 | 0 | 0.699 |
| ENSP00000354775 | 9.57E-06 | -0.1019 | 0 | 0.301 |
| ENSP00000312081 | 5.01E-06 | -0.1019 | 0 | 0.681 |
| ENSP00000319914 | 6.11E-05 | -0.1019 | 270 | 0.582 |
| ENSP00000369100 | 1.01E-05 | -0.1019 | 203 | 0.480 |
| ENSP00000364645 | 4.08E-06 | -0.1019 | 0 | 0.700 |
| ENSP00000380872 | 5.35E-06 | -0.1019 | 0 | 0.145 |
| ENSP00000338185 | 8.49E-06 | -0.1019 | 0 | 0.715 |
| ENSP00000248211 | 4.52E-06 | -0.1019 | 0 | 0.697 |
| ENSP00000393876 | 1.07E-05 | -0.1019 | 167 | 0.708 |
| ENSP00000355279 | 1.14E-05 | -0.1019 | 284 | 0.413 |
| ENSP00000312435 | 3.38E-05 | -0.1019 | 503 | 0.774 |
| ENSP00000257910 | 2.70E-05 | -0.1019 | 0 | 0.097 |
| ENSP00000379217 | 1.95E-05 | -0.1019 | 163 | 0.465 |
| ENSP00000364212 | 1.28E-05 | -0.1020 | 179 | 0.604 |
| ENSP00000365693 | 2.27E-05 | -0.1020 | 207 | 0.119 |
| ENSP00000175091 | 6.47E-05 | -0.1020 | 373 | 0.121 |
| ENSP00000327538 | 4.47E-06 | -0.1020 | 0 | 0.696 |
| ENSP00000371472 | 1.94E-05 | -0.1020 | 282 | 0.502 |
| ENSP00000283415 | 4.28E-05 | -0.1020 | 272 | 0.263 |
| ENSP00000257627 | 7.61E-06 | -0.1020 | 242 | 0.000 |
| ENSP00000312042 | 4.04E-05 | -0.1020 | 311 | 0.528 |
| ENSP00000315791 | 5.72E-06 | -0.1020 | 0 | 0.824 |
| ENSP00000236959 | 2.47E-05 | -0.1020 | 387 | 0.764 |
| ENSP00000348753 | 2.45E-05 | -0.1020 | 0 | 0.541 |
| ENSP00000362063 | 1.98E-05 | -0.1020 | 239 | 0.900 |
| ENSP00000296084 | 4.94E-05 | -0.1021 | 421 | 0.000 |
| ENSP00000431905 | 1.03E-06 | -0.1021 | 0 | 0.152 |
| ENSP00000354125 | 1.71E-05 | -0.1021 | 330 | 0.933 |
| ENSP00000356155 | 1.76E-05 | -0.1021 | 165 | 0.656 |
| ENSP00000337473 | 4.59E-06 | -0.1021 | 0 | 0.700 |
| ENSP00000328551 | 4.63E-06 | -0.1021 | 0 | 0.238 |
| ENSP00000314080 | 4.12E-05 | -0.1021 | 301 | 0.802 |
| ENSP00000285238 | 9.43E-06 | -0.1021 | 160 | 0.434 |
| ENSP00000351100 | 6.87E-06 | -0.1021 | 0 | 0.721 |
| ENSP00000472919 | 1.90E-06 | -0.1022 | 0 | 0.191 |
| ENSP00000436496 | 3.31E-06 | -0.1022 | 0 | 0.000 |
| ENSP00000361433 | 6.52E-06 | -0.1022 | 0 | 0.861 |

| | | | | |
|---|---|---|---|---|
| ENSP00000319222 | 5.25E-06 | -0.1022 | 0 | 0.674 |
| ENSP00000380150 | 2.04E-05 | -0.1022 | 208 | 0.849 |
| ENSP00000225573 | 6.79E-05 | -0.1022 | 198 | 0.125 |
| ENSP00000225296 | 3.31E-05 | -0.1022 | 352 | 0.922 |
| ENSP00000311319 | 4.13E-06 | -0.1022 | 0 | 0.000 |
| ENSP00000310649 | 4.46E-06 | -0.1022 | 0 | 0.691 |
| ENSP00000377080 | 8.46E-06 | -0.1022 | 177 | 0.764 |
| ENSP00000297183 | 6.84E-06 | -0.1023 | 202 | 0.654 |
| ENSP00000332861 | 4.05E-06 | -0.1023 | 0 | 0.696 |
| ENSP00000227758 | 2.69E-05 | -0.1023 | 388 | 0.847 |
| ENSP00000264606 | 2.07E-05 | -0.1023 | 319 | 0.000 |
| ENSP00000254321 | 3.96E-06 | -0.1023 | 0 | 0.678 |
| ENSP00000340305 | 5.98E-06 | -0.1023 | 0 | 0.666 |
| ENSP00000253693 | 2.41E-06 | -0.1023 | 0 | 0.000 |
| ENSP00000452571 | 2.12E-06 | -0.1023 | 0 | 0.000 |
| ENSP00000346236 | 9.70E-06 | -0.1023 | 0 | 0.912 |
| ENSP00000378856 | 1.29E-05 | -0.1023 | 151 | 0.548 |
| ENSP00000351908 | 3.79E-05 | -0.1023 | 526 | 0.893 |
| ENSP00000369325 | 5.66E-06 | -0.1024 | 0 | 0.243 |
| ENSP00000324598 | 6.00E-06 | -0.1024 | 0 | 0.703 |
| ENSP00000356064 | 3.10E-05 | -0.1024 | 213 | 0.088 |
| ENSP00000304151 | 3.50E-06 | -0.1024 | 0 | 0.000 |
| ENSP00000296839 | 2.78E-06 | -0.1024 | 0 | 0.000 |
| ENSP00000307142 | 9.86E-06 | -0.1024 | 0 | 0.149 |
| ENSP00000415027 | 2.52E-07 | -0.1024 | 0 | 0.213 |
| ENSP00000264639 | 6.83E-06 | -0.1024 | 0 | 0.457 |
| ENSP00000262032 | 4.13E-06 | -0.1024 | 0 | 0.707 |
| ENSP00000250416 | 2.26E-05 | -0.1024 | 568 | 0.761 |
| ENSP00000317177 | 3.37E-05 | -0.1025 | 298 | 0.469 |
| ENSP00000238081 | 1.69E-05 | -0.1025 | 428 | 0.691 |
| ENSP00000259271 | 2.80E-05 | -0.1025 | 227 | 0.700 |
| ENSP00000337853 | 4.42E-05 | -0.1025 | 418 | 0.902 |
| ENSP00000370891 | 8.02E-06 | -0.1025 | 0 | 0.152 |
| ENSP00000359353 | 5.33E-06 | -0.1025 | 0 | 0.076 |
| ENSP00000402060 | 2.16E-05 | -0.1025 | 163 | 0.623 |
| ENSP00000252463 | 5.00E-06 | -0.1025 | 0 | 0.716 |
| ENSP00000468354 | 9.45E-06 | -0.1025 | 202 | 0.589 |
| ENSP00000358140 | 1.83E-05 | -0.1025 | 266 | 0.899 |
| ENSP00000311030 | 1.06E-05 | -0.1025 | 160 | 0.363 |
| ENSP00000350698 | 1.84E-05 | -0.1025 | 334 | 0.895 |
| ENSP00000453144 | 3.85E-05 | -0.1025 | 252 | 0.489 |
| ENSP00000305777 | 6.77E-06 | -0.1025 | 0 | 0.321 |
| ENSP00000345964 | 1.80E-05 | -0.1026 | 330 | 0.168 |
| ENSP00000317817 | 2.35E-05 | -0.1026 | 191 | 0.264 |
| ENSP00000262039 | 1.13E-05 | -0.1026 | 662 | 0.705 |
| ENSP00000304229 | 9.41E-06 | -0.1026 | 301 | 0.645 |

| | | | | |
|---|---|---|---|---|
| ENSP00000396045 | 1.88E-05 | -0.1026 | 154 | 0.163 |
| ENSP00000306528 | 4.17E-05 | -0.1026 | 382 | 0.295 |
| ENSP00000243643 | 4.35E-06 | -0.1026 | 0 | 0.692 |
| ENSP00000360069 | 3.79E-06 | -0.1026 | 0 | 0.692 |
| ENSP00000251810 | 6.25E-06 | -0.1026 | 292 | 0.649 |
| ENSP00000375416 | 4.09E-06 | -0.1026 | 0 | 0.000 |
| ENSP00000324441 | 4.13E-06 | -0.1026 | 0 | 0.695 |
| ENSP00000270225 | 9.32E-06 | -0.1026 | 266 | 0.669 |
| ENSP00000223500 | 1.78E-06 | -0.1026 | 0 | 0.000 |
| ENSP00000347549 | 1.64E-05 | -0.1027 | 252 | 0.000 |
| ENSP00000297185 | 2.29E-05 | -0.1027 | 256 | 0.887 |
| ENSP00000369887 | 6.25E-06 | -0.1027 | 0 | 0.671 |
| ENSP00000347810 | 2.32E-07 | -0.1027 | 0 | 0.122 |
| ENSP00000393835 | 5.21E-06 | -0.1027 | 0 | 0.688 |
| ENSP00000366273 | 7.49E-06 | -0.1027 | 609 | 0.690 |
| ENSP00000355580 | 7.56E-06 | -0.1027 | 0 | 0.207 |
| ENSP00000304277 | 8.24E-05 | -0.1027 | 151 | 0.159 |
| ENSP00000369695 | 2.48E-05 | -0.1027 | 789 | 0.860 |
| ENSP00000276282 | 5.01E-06 | -0.1027 | 0 | 0.642 |
| ENSP00000301475 | 5.64E-06 | -0.1027 | 0 | 0.675 |
| ENSP00000300737 | 3.57E-05 | -0.1027 | 211 | 0.546 |
| ENSP00000216034 | 4.17E-05 | -0.1027 | 196 | 0.308 |
| ENSP00000309812 | 4.72E-06 | -0.1027 | 0 | 0.710 |
| ENSP00000339823 | 6.51E-06 | -0.1027 | 0 | 0.690 |
| ENSP00000222224 | 4.49E-05 | -0.1028 | 0 | 0.302 |
| ENSP00000229266 | 4.19E-05 | -0.1028 | 183 | 0.179 |
| ENSP00000217086 | 4.42E-05 | -0.1028 | 221 | 0.821 |
| ENSP00000364582 | 1.51E-05 | -0.1028 | 644 | 0.394 |
| ENSP00000341940 | 1.83E-05 | -0.1028 | 313 | 0.595 |
| ENSP00000317904 | 1.17E-05 | -0.1028 | 264 | 0.647 |
| ENSP00000296503 | 3.16E-05 | -0.1028 | 269 | 0.894 |
| ENSP00000248070 | 5.63E-06 | -0.1028 | 0 | 0.350 |
| ENSP00000264734 | 7.98E-06 | -0.1029 | 0 | 0.149 |
| ENSP00000348722 | 7.11E-06 | -0.1029 | 0 | 0.825 |
| ENSP00000363621 | 2.94E-05 | -0.1029 | 212 | 0.348 |
| ENSP00000325519 | 7.84E-06 | -0.1029 | 0 | 0.000 |
| ENSP00000336719 | 4.48E-06 | -0.1029 | 0 | 0.683 |
| ENSP00000382659 | 2.60E-05 | -0.1029 | 814 | 0.757 |
| ENSP00000319281 | 2.90E-04 | -0.1029 | 652 | 0.092 |
| ENSP00000334785 | 2.93E-05 | -0.1029 | 210 | 0.000 |
| ENSP00000376176 | 2.80E-05 | -0.1029 | 346 | 0.755 |
| ENSP00000292176 | 4.48E-06 | -0.1029 | 0 | 0.766 |
| ENSP00000396080 | 4.71E-07 | -0.1029 | 0 | 0.183 |
| ENSP00000356540 | 2.56E-05 | -0.1029 | 377 | 0.254 |
| ENSP00000465742 | 3.54E-06 | -0.1029 | 0 | 0.505 |
| ENSP00000434142 | 5.88E-06 | -0.1030 | 0 | 0.077 |

| | | | | |
|---|---|---|---|---|
| ENSP00000339145 | 3.42E-05 | -0.1030 | 162 | 0.892 |
| ENSP00000463013 | 7.11E-06 | -0.1030 | 0 | 0.183 |
| ENSP00000232974 | 4.33E-06 | -0.1030 | 0 | 0.698 |
| ENSP00000233057 | 4.96E-05 | -0.1030 | 336 | 0.722 |
| ENSP00000157812 | 9.48E-06 | -0.1030 | 214 | 0.599 |
| ENSP00000261195 | 3.63E-06 | -0.1030 | 0 | 0.581 |
| ENSP00000238628 | 6.14E-05 | -0.1030 | 256 | 0.837 |
| ENSP00000296582 | 4.84E-06 | -0.1030 | 0 | 0.148 |
| ENSP00000278671 | 1.40E-04 | -0.1030 | 804 | 0.093 |
| ENSP00000362306 | 8.58E-06 | -0.1031 | 467 | 0.707 |
| ENSP00000330343 | 1.66E-05 | -0.1031 | 281 | 0.857 |
| ENSP00000000442 | 4.50E-05 | -0.1031 | 195 | 0.822 |
| ENSP00000358596 | 1.55E-05 | -0.1031 | 201 | 0.806 |
| ENSP00000340596 | 5.85E-06 | -0.1031 | 0 | 0.265 |
| ENSP00000376178 | 3.04E-05 | -0.1031 | 377 | 0.266 |
| ENSP00000465978 | 6.95E-09 | -0.1031 | 0 | 0.000 |
| ENSP00000362127 | 5.57E-06 | -0.1031 | 0 | 0.696 |
| ENSP00000327984 | 1.00E-05 | -0.1031 | 153 | 0.000 |
| ENSP00000353957 | 3.62E-06 | -0.1031 | 0 | 0.685 |
| ENSP00000229264 | 9.25E-06 | -0.1031 | 397 | 0.648 |
| ENSP00000350028 | 8.44E-06 | -0.1031 | 0 | 0.830 |
| ENSP00000265854 | 4.04E-06 | -0.1032 | 0 | 0.571 |
| ENSP00000313199 | 2.83E-05 | -0.1032 | 822 | 0.931 |
| ENSP00000307491 | 4.20E-06 | -0.1032 | 0 | 0.643 |
| ENSP00000472847 | 2.66E-05 | -0.1032 | 336 | 0.668 |
| ENSP00000408526 | 1.82E-05 | -0.1032 | 185 | 0.252 |
| ENSP00000445366 | 7.04E-06 | -0.1032 | 0 | 0.586 |
| ENSP00000282308 | 3.69E-06 | -0.1032 | 0 | 0.689 |
| ENSP00000403980 | 8.98E-07 | -0.1032 | 0 | 0.000 |
| ENSP00000268957 | 3.98E-05 | -0.1032 | 284 | 0.533 |
| ENSP00000256497 | 3.58E-05 | -0.1032 | 923 | 0.348 |
| ENSP00000384965 | 3.69E-06 | -0.1032 | 0 | 0.163 |
| ENSP00000261401 | 3.29E-05 | -0.1032 | 335 | 0.423 |
| ENSP00000260363 | 3.14E-06 | -0.1033 | 0 | 0.358 |
| ENSP00000358297 | 8.70E-06 | -0.1033 | 0 | 0.000 |
| ENSP00000313258 | 3.09E-06 | -0.1033 | 0 | 0.714 |
| ENSP00000263921 | 5.50E-05 | -0.1033 | 156 | 0.180 |
| ENSP00000468311 | 3.23E-06 | -0.1033 | 0 | 0.000 |
| ENSP00000351280 | 3.67E-06 | -0.1033 | 0 | 0.693 |
| ENSP00000260385 | 1.86E-04 | -0.1033 | 644 | 0.085 |
| ENSP00000245903 | 8.09E-05 | -0.1033 | 224 | 0.659 |
| ENSP00000305077 | 3.80E-06 | -0.1033 | 0 | 0.684 |
| ENSP00000250076 | 4.30E-06 | -0.1033 | 0 | 0.692 |
| ENSP00000309606 | 4.53E-06 | -0.1033 | 0 | 0.686 |
| ENSP00000355166 | 8.11E-06 | -0.1033 | 484 | 0.638 |
| ENSP00000328023 | 5.96E-06 | -0.1033 | 398 | 0.621 |

| | | | | |
|---|---|---|---|---|
| ENSP00000303889 | 4.32E-06 | -0.1034 | 0 | 0.693 |
| ENSP00000360277 | 1.23E-05 | -0.1034 | 218 | 0.000 |
| ENSP00000337168 | 3.94E-05 | -0.1034 | 404 | 0.336 |
| ENSP00000360916 | 9.61E-06 | -0.1034 | 0 | 0.745 |
| ENSP00000247584 | 4.55E-06 | -0.1034 | 0 | 0.717 |
| ENSP00000242796 | 5.35E-05 | -0.1034 | 186 | 0.000 |
| ENSP00000420381 | 3.42E-05 | -0.1035 | 517 | 0.647 |
| ENSP00000403103 | 2.30E-05 | -0.1035 | 207 | 0.789 |
| ENSP00000355094 | 1.86E-05 | -0.1035 | 334 | 0.767 |
| ENSP00000347134 | 6.60E-06 | -0.1035 | 0 | 0.539 |
| ENSP00000356105 | 1.74E-05 | -0.1035 | 0 | 0.098 |
| ENSP00000263381 | 3.87E-06 | -0.1035 | 0 | 0.716 |
| ENSP00000320236 | 4.19E-05 | -0.1035 | 167 | 0.193 |
| ENSP00000376952 | 4.63E-06 | -0.1035 | 0 | 0.476 |
| ENSP00000320627 | 5.22E-06 | -0.1035 | 0 | 0.000 |
| ENSP00000386759 | 3.89E-05 | -0.1035 | 244 | 0.906 |
| ENSP00000352121 | 2.92E-05 | -0.1036 | 310 | 0.921 |
| ENSP00000325326 | 4.50E-06 | -0.1036 | 0 | 0.706 |
| ENSP00000263556 | 4.01E-05 | -0.1036 | 900 | 0.566 |
| ENSP00000218004 | 9.30E-05 | -0.1036 | 221 | 0.428 |
| ENSP00000282292 | 4.25E-06 | -0.1036 | 0 | 0.681 |
| ENSP00000341944 | 7.12E-06 | -0.1036 | 0 | 0.663 |
| ENSP00000356548 | 4.70E-06 | -0.1036 | 0 | 0.818 |
| ENSP00000350075 | 3.26E-05 | -0.1036 | 0 | 0.000 |
| ENSP00000302805 | 1.26E-05 | -0.1036 | 216 | 0.000 |
| ENSP00000331927 | 3.79E-06 | -0.1036 | 0 | 0.675 |
| ENSP00000397693 | 3.57E-06 | -0.1036 | 0 | 0.691 |
| ENSP00000347032 | 6.25E-06 | -0.1036 | 0 | 0.499 |
| ENSP00000341625 | 1.92E-05 | -0.1036 | 270 | 0.500 |
| ENSP00000339787 | 3.80E-05 | -0.1036 | 328 | 0.539 |
| ENSP00000292530 | 3.94E-06 | -0.1037 | 0 | 0.685 |
| ENSP00000267287 | 5.00E-05 | -0.1037 | 154 | 0.521 |
| ENSP00000372322 | 2.13E-05 | -0.1037 | 219 | 0.000 |
| ENSP00000371127 | 1.44E-07 | -0.1037 | 0 | 0.000 |
| ENSP00000294288 | 8.62E-06 | -0.1037 | 439 | 0.000 |
| ENSP00000252979 | 3.71E-06 | -0.1037 | 0 | 0.682 |
| ENSP00000268793 | 1.65E-05 | -0.1037 | 194 | 0.109 |
| ENSP00000349494 | 4.23E-06 | -0.1037 | 0 | 0.691 |
| ENSP00000261507 | 3.65E-05 | -0.1037 | 193 | 0.440 |
| ENSP00000355599 | 3.49E-06 | -0.1037 | 0 | 0.187 |
| ENSP00000369538 | 2.16E-05 | -0.1037 | 293 | 0.310 |
| ENSP00000249636 | 2.82E-05 | -0.1037 | 181 | 0.791 |
| ENSP00000264645 | 4.78E-05 | -0.1037 | 292 | 0.818 |
| ENSP00000371606 | 4.33E-06 | -0.1037 | 0 | 0.202 |
| ENSP00000362300 | 2.13E-05 | -0.1037 | 207 | 0.722 |
| ENSP00000247219 | 9.45E-06 | -0.1037 | 315 | 0.000 |

| | | | | |
|---|---|---|---|---|
| ENSP00000309945 | 2.12E-05 | -0.1038 | 242 | 0.626 |
| ENSP00000353362 | 2.47E-05 | -0.1038 | 282 | 0.676 |
| ENSP00000302756 | 2.64E-06 | -0.1038 | 0 | 0.000 |
| ENSP00000326870 | 4.86E-06 | -0.1038 | 0 | 0.000 |
| ENSP00000375863 | 1.36E-05 | -0.1038 | 549 | 0.725 |
| ENSP00000327143 | 4.83E-06 | -0.1038 | 0 | 0.702 |
| ENSP00000344791 | 4.08E-06 | -0.1038 | 0 | 0.681 |
| ENSP00000202967 | 2.73E-05 | -0.1038 | 185 | 0.848 |
| ENSP00000244763 | 5.34E-05 | -0.1038 | 609 | 0.519 |
| ENSP00000301399 | 3.70E-06 | -0.1038 | 0 | 0.685 |
| ENSP00000345281 | 5.29E-06 | -0.1038 | 0 | 0.654 |
| ENSP00000291552 | 3.28E-05 | -0.1038 | 230 | 0.875 |
| ENSP00000261965 | 9.28E-06 | -0.1038 | 0 | 0.153 |
| ENSP00000316465 | 2.30E-06 | -0.1039 | 0 | 0.000 |
| ENSP00000385597 | 5.06E-06 | -0.1039 | 0 | 0.094 |
| ENSP00000311899 | 2.03E-05 | -0.1039 | 175 | 0.799 |
| ENSP00000225388 | 1.06E-04 | -0.1039 | 197 | 0.360 |
| ENSP00000350195 | 7.79E-06 | -0.1039 | 199 | 0.672 |
| ENSP00000396445 | 3.47E-06 | -0.1039 | 0 | 0.259 |
| ENSP00000270115 | 5.47E-06 | -0.1039 | 0 | 0.462 |
| ENSP00000344308 | 3.92E-06 | -0.1040 | 0 | 0.689 |
| ENSP00000194530 | 1.84E-05 | -0.1040 | 0 | 0.269 |
| ENSP00000277458 | 2.46E-05 | -0.1040 | 307 | 0.699 |
| ENSP00000353444 | 6.19E-06 | -0.1040 | 0 | 0.227 |
| ENSP00000273077 | 3.22E-06 | -0.1040 | 0 | 0.000 |
| ENSP00000308496 | 7.79E-06 | -0.1040 | 204 | 0.548 |
| ENSP00000221315 | 4.33E-06 | -0.1041 | 0 | 0.703 |
| ENSP00000344129 | 4.32E-06 | -0.1041 | 0 | 0.687 |
| ENSP00000351422 | 3.06E-06 | -0.1041 | 0 | 0.167 |
| ENSP00000369213 | 2.06E-05 | -0.1041 | 188 | 0.693 |
| ENSP00000307604 | 4.30E-06 | -0.1041 | 0 | 0.685 |
| ENSP00000289952 | 4.93E-05 | -0.1041 | 177 | 0.176 |
| ENSP00000333071 | 7.74E-05 | -0.1041 | 0 | 0.119 |
| ENSP00000298681 | 2.85E-06 | -0.1041 | 0 | 0.000 |
| ENSP00000300161 | 2.62E-05 | -0.1041 | 791 | 0.679 |
| ENSP00000358737 | 4.47E-06 | -0.1041 | 0 | 0.108 |
| ENSP00000380671 | 2.20E-07 | -0.1041 | 0 | 0.208 |
| ENSP00000343171 | 7.65E-06 | -0.1041 | 0 | 0.209 |
| ENSP00000259467 | 5.51E-05 | -0.1041 | 866 | 0.094 |
| ENSP00000219091 | 4.01E-06 | -0.1042 | 0 | 0.702 |
| ENSP00000338477 | 3.86E-05 | -0.1042 | 615 | 0.872 |
| ENSP00000439128 | 9.30E-06 | -0.1042 | 347 | 0.236 |
| ENSP00000282869 | 3.75E-06 | -0.1042 | 0 | 0.676 |
| ENSP00000250916 | 3.82E-06 | -0.1042 | 0 | 0.719 |
| ENSP00000332668 | 1.37E-05 | -0.1042 | 271 | 0.775 |
| ENSP00000308219 | 2.31E-05 | -0.1042 | 216 | 0.589 |

| | | | | |
|---|---|---|---|---|
| ENSP00000246548 | 8.63E-06 | -0.1042 | 276 | 0.648 |
| ENSP00000274311 | 3.87E-05 | -0.1042 | 167 | 0.219 |
| ENSP00000369162 | 8.44E-06 | -0.1042 | 0 | 0.820 |
| ENSP00000369371 | 5.89E-06 | -0.1042 | 0 | 0.094 |
| ENSP00000349977 | 5.22E-06 | -0.1042 | 0 | 0.441 |
| ENSP00000314153 | 4.05E-06 | -0.1042 | 0 | 0.718 |
| ENSP00000362244 | 5.23E-06 | -0.1043 | 0 | 0.073 |
| ENSP00000348924 | 1.77E-05 | -0.1043 | 900 | 0.000 |
| ENSP00000284957 | 4.27E-06 | -0.1043 | 0 | 0.156 |
| ENSP00000301480 | 4.99E-06 | -0.1043 | 0 | 0.709 |
| ENSP00000295463 | 2.59E-05 | -0.1043 | 357 | 0.378 |
| ENSP00000226247 | 3.11E-06 | -0.1043 | 0 | 0.000 |
| ENSP00000263791 | 2.11E-05 | -0.1043 | 658 | 0.748 |
| ENSP00000313953 | 4.57E-06 | -0.1043 | 0 | 0.820 |
| ENSP00000155093 | 3.79E-06 | -0.1043 | 0 | 0.717 |
| ENSP00000312535 | 5.00E-06 | -0.1043 | 0 | 0.659 |
| ENSP00000266269 | 4.02E-06 | -0.1043 | 0 | 0.760 |
| ENSP00000361845 | 1.90E-05 | -0.1043 | 340 | 0.419 |
| ENSP00000359393 | 2.34E-05 | -0.1044 | 269 | 0.917 |
| ENSP00000395590 | 4.57E-06 | -0.1044 | 0 | 0.561 |
| ENSP00000362717 | 2.86E-05 | -0.1044 | 250 | 0.849 |
| ENSP00000356903 | 1.52E-05 | -0.1044 | 0 | 0.152 |
| ENSP00000419005 | 8.70E-06 | -0.1044 | 0 | 0.722 |
| ENSP00000337466 | 1.12E-05 | -0.1044 | 0 | 0.144 |
| ENSP00000313877 | 1.03E-05 | -0.1044 | 378 | 0.646 |
| ENSP00000415151 | 8.24E-06 | -0.1045 | 0 | 0.739 |
| ENSP00000372191 | 3.05E-05 | -0.1045 | 407 | 0.809 |
| ENSP00000417063 | 1.16E-06 | -0.1045 | 0 | 0.172 |
| ENSP00000369571 | 4.94E-06 | -0.1045 | 0 | 0.336 |
| ENSP00000333776 | 5.00E-06 | -0.1045 | 0 | 0.685 |
| ENSP00000339834 | 1.19E-04 | -0.1045 | 0 | 0.300 |
| ENSP00000455099 | 1.01E-06 | -0.1045 | 0 | 0.145 |
| ENSP00000246229 | 4.03E-06 | -0.1045 | 0 | 0.734 |
| ENSP00000277225 | 7.06E-06 | -0.1045 | 0 | 0.693 |
| ENSP00000318195 | 3.36E-05 | -0.1045 | 262 | 0.753 |
| ENSP00000223210 | 3.54E-06 | -0.1045 | 0 | 0.640 |
| ENSP00000391641 | 5.78E-06 | -0.1045 | 0 | 0.708 |
| ENSP00000292427 | 1.82E-05 | -0.1045 | 265 | 0.544 |
| ENSP00000315768 | 2.95E-05 | -0.1045 | 233 | 0.894 |
| ENSP00000354878 | 4.69E-06 | -0.1046 | 0 | 0.248 |
| ENSP00000337722 | 2.48E-05 | -0.1046 | 390 | 0.549 |
| ENSP00000276072 | 1.30E-05 | -0.1046 | 429 | 0.000 |
| ENSP00000288221 | 4.04E-05 | -0.1046 | 372 | 0.126 |
| ENSP00000219478 | 3.97E-06 | -0.1046 | 0 | 0.696 |
| ENSP00000365924 | 4.19E-06 | -0.1046 | 0 | 0.299 |
| ENSP00000303057 | 4.12E-05 | -0.1046 | 424 | 0.209 |

| | | | | |
|---|---|---|---|---|
| ENSP00000337332 | 2.61E-05 | -0.1046 | 185 | 0.864 |
| ENSP00000359398 | 6.31E-06 | -0.1046 | 0 | 0.148 |
| ENSP00000349147 | 6.97E-06 | -0.1046 | 189 | 0.154 |
| ENSP00000348706 | 1.68E-05 | -0.1046 | 845 | 0.000 |
| ENSP00000249363 | 4.30E-06 | -0.1046 | 0 | 0.659 |
| ENSP00000382000 | 1.26E-06 | -0.1046 | 0 | 0.138 |
| ENSP00000372955 | 4.76E-06 | -0.1046 | 0 | 0.000 |
| ENSP00000340820 | 3.27E-05 | -0.1046 | 974 | 0.898 |
| ENSP00000335500 | 1.21E-05 | -0.1046 | 0 | 0.132 |
| ENSP00000384211 | 1.67E-05 | -0.1046 | 172 | 0.306 |
| ENSP00000314414 | 5.02E-06 | -0.1046 | 0 | 0.000 |
| ENSP00000365898 | 3.31E-06 | -0.1047 | 0 | 0.000 |
| ENSP00000359727 | 1.69E-05 | -0.1047 | 384 | 0.389 |
| ENSP00000328732 | 4.03E-06 | -0.1047 | 0 | 0.000 |
| ENSP00000244020 | 1.77E-05 | -0.1047 | 400 | 0.948 |
| ENSP00000338860 | 4.36E-06 | -0.1047 | 0 | 0.690 |
| ENSP00000331214 | 7.65E-06 | -0.1047 | 0 | 0.000 |
| ENSP00000220062 | 9.80E-06 | -0.1047 | 504 | 0.613 |
| ENSP00000284898 | 4.83E-06 | -0.1047 | 0 | 0.474 |
| ENSP00000352185 | 3.88E-06 | -0.1047 | 0 | 0.000 |
| ENSP00000361635 | 8.43E-06 | -0.1047 | 191 | 0.458 |
| ENSP00000379839 | 2.45E-05 | -0.1047 | 213 | 0.288 |
| ENSP00000300209 | 6.26E-05 | -0.1047 | 348 | 0.177 |
| ENSP00000372326 | 1.26E-05 | -0.1047 | 236 | 0.479 |
| ENSP00000429986 | 4.08E-06 | -0.1048 | 0 | 0.530 |
| ENSP00000276590 | 2.83E-06 | -0.1048 | 0 | 0.000 |
| ENSP00000401701 | 5.61E-06 | -0.1048 | 0 | 0.130 |
| ENSP00000256996 | 5.93E-06 | -0.1048 | 0 | 0.796 |
| ENSP00000291182 | 4.49E-06 | -0.1048 | 0 | 0.698 |
| ENSP00000370119 | 2.41E-05 | -0.1048 | 197 | 0.717 |
| ENSP00000294172 | 3.54E-05 | -0.1048 | 398 | 0.835 |
| ENSP00000341243 | 2.49E-05 | -0.1048 | 325 | 0.900 |
| ENSP00000411372 | 1.09E-07 | -0.1048 | 0 | 0.215 |
| ENSP00000360664 | 1.59E-05 | -0.1048 | 324 | 0.727 |
| ENSP00000263694 | 4.80E-05 | -0.1048 | 183 | 0.819 |
| ENSP00000342235 | 3.28E-05 | -0.1048 | 640 | 0.958 |
| ENSP00000344635 | 4.31E-05 | -0.1049 | 246 | 0.706 |
| ENSP00000321209 | 5.76E-06 | -0.1049 | 0 | 0.747 |
| ENSP00000296220 | 4.19E-05 | -0.1049 | 427 | 0.151 |
| ENSP00000355963 | 8.03E-06 | -0.1049 | 418 | 0.284 |
| ENSP00000284878 | 4.00E-05 | -0.1049 | 159 | 0.310 |
| ENSP00000253794 | 3.36E-05 | -0.1049 | 325 | 0.299 |
| ENSP00000263038 | 1.93E-05 | -0.1050 | 0 | 0.239 |
| ENSP00000264515 | 3.50E-05 | -0.1050 | 279 | 0.888 |
| ENSP00000352821 | 1.76E-05 | -0.1050 | 0 | 0.094 |
| ENSP00000292616 | 5.16E-06 | -0.1050 | 0 | 0.636 |

| | | | | |
|---|---|---|---|---|
| ENSP00000448689 | 7.51E-06 | -0.1050 | 0 | 0.399 |
| ENSP00000359070 | 2.68E-05 | -0.1050 | 836 | 0.110 |
| ENSP00000219069 | 3.74E-06 | -0.1050 | 0 | 0.664 |
| ENSP00000354441 | 5.26E-06 | -0.1050 | 0 | 0.175 |
| ENSP00000356410 | 6.99E-06 | -0.1050 | 249 | 0.356 |
| ENSP00000263125 | 8.68E-06 | -0.1050 | 900 | 0.662 |
| ENSP00000370034 | 2.09E-05 | -0.1050 | 242 | 0.886 |
| ENSP00000376965 | 2.82E-05 | -0.1050 | 211 | 0.644 |
| ENSP00000321388 | 8.59E-06 | -0.1051 | 202 | 0.708 |
| ENSP00000391592 | 1.60E-05 | -0.1051 | 0 | 0.835 |
| ENSP00000353739 | 9.00E-06 | -0.1051 | 208 | 0.489 |
| ENSP00000233078 | 4.33E-05 | -0.1052 | 389 | 0.830 |
| ENSP00000273261 | 5.69E-06 | -0.1052 | 0 | 0.695 |
| ENSP00000261908 | 3.58E-05 | -0.1052 | 198 | 0.450 |
| ENSP00000276461 | 3.94E-05 | -0.1052 | 394 | 0.184 |
| ENSP00000269856 | 2.26E-05 | -0.1052 | 279 | 0.600 |
| ENSP00000319851 | 8.41E-06 | -0.1052 | 0 | 0.091 |
| ENSP00000272223 | 4.36E-06 | -0.1052 | 0 | 0.786 |
| ENSP00000263238 | 1.53E-05 | -0.1052 | 378 | 0.667 |
| ENSP00000265087 | 4.40E-05 | -0.1052 | 235 | 0.334 |
| ENSP00000256722 | 3.97E-06 | -0.1052 | 0 | 0.442 |
| ENSP00000301310 | 4.47E-06 | -0.1053 | 0 | 0.697 |
| ENSP00000315931 | 1.87E-05 | -0.1053 | 198 | 0.316 |
| ENSP00000378089 | 1.88E-05 | -0.1053 | 229 | 0.564 |
| ENSP00000211936 | 3.89E-06 | -0.1053 | 0 | 0.703 |
| ENSP00000299575 | 3.78E-06 | -0.1053 | 0 | 0.660 |
| ENSP00000301788 | 4.49E-06 | -0.1053 | 0 | 0.751 |
| ENSP00000296755 | 3.53E-05 | -0.1053 | 296 | 0.720 |
| ENSP00000385746 | 1.91E-05 | -0.1053 | 250 | 0.313 |
| ENSP00000229971 | 2.09E-06 | -0.1054 | 0 | 0.000 |
| ENSP00000256689 | 3.17E-05 | -0.1054 | 151 | 0.082 |
| ENSP00000355251 | 8.64E-06 | -0.1054 | 0 | 0.121 |
| ENSP00000360329 | 1.79E-05 | -0.1054 | 343 | 0.693 |
| ENSP00000261483 | 2.02E-05 | -0.1054 | 211 | 0.168 |
| ENSP00000204517 | 4.47E-05 | -0.1054 | 150 | 0.745 |
| ENSP00000349356 | 3.27E-05 | -0.1055 | 272 | 0.384 |
| ENSP00000285979 | 8.52E-06 | -0.1055 | 160 | 0.229 |
| ENSP00000374529 | 7.59E-06 | -0.1055 | 0 | 0.769 |
| ENSP00000365439 | 2.80E-05 | -0.1055 | 739 | 0.793 |
| ENSP00000376472 | 1.32E-05 | -0.1055 | 189 | 0.265 |
| ENSP00000399718 | 6.36E-06 | -0.1055 | 220 | 0.306 |
| ENSP00000325655 | 1.58E-06 | -0.1055 | 0 | 0.164 |
| ENSP00000340691 | 2.50E-05 | -0.1055 | 419 | 0.860 |
| ENSP00000358765 | 5.96E-06 | -0.1055 | 0 | 0.692 |
| ENSP00000287598 | 3.93E-06 | -0.1055 | 0 | 0.544 |
| ENSP00000368135 | 7.13E-06 | -0.1056 | 0 | 0.652 |

| | | | | |
|---|---|---|---|---|
| ENSP00000230122 | 3.57E-06 | -0.1056 | 0 | 0.670 |
| ENSP00000367851 | 1.37E-05 | -0.1056 | 198 | 0.666 |
| ENSP00000284000 | 2.98E-05 | -0.1056 | 582 | 0.772 |
| ENSP00000305976 | 4.78E-06 | -0.1056 | 0 | 0.706 |
| ENSP00000282296 | 5.08E-06 | -0.1056 | 0 | 0.689 |
| ENSP00000357132 | 7.90E-06 | -0.1057 | 0 | 0.159 |
| ENSP00000320295 | 2.58E-05 | -0.1057 | 420 | 0.000 |
| ENSP00000313983 | 1.32E-05 | -0.1057 | 244 | 0.936 |
| ENSP00000372155 | 9.46E-06 | -0.1057 | 215 | 0.480 |
| ENSP00000287916 | 5.05E-05 | -0.1057 | 373 | 0.154 |
| ENSP00000218652 | 4.53E-05 | -0.1057 | 203 | 0.071 |
| ENSP00000342137 | 2.35E-04 | -0.1058 | 0 | 0.130 |
| ENSP00000343314 | 4.66E-06 | -0.1058 | 0 | 0.165 |
| ENSP00000263918 | 3.59E-05 | -0.1058 | 163 | 0.237 |
| ENSP00000293725 | 4.21E-06 | -0.1058 | 0 | 0.682 |
| ENSP00000327314 | 4.79E-06 | -0.1058 | 0 | 0.678 |
| ENSP00000346160 | 5.32E-06 | -0.1058 | 0 | 0.621 |
| ENSP00000304769 | 4.60E-06 | -0.1058 | 0 | 0.731 |
| ENSP00000470580 | 1.92E-06 | -0.1058 | 0 | 0.000 |
| ENSP00000292562 | 5.39E-06 | -0.1058 | 0 | 0.692 |
| ENSP00000216727 | 3.22E-05 | -0.1058 | 186 | 0.879 |
| ENSP00000262710 | 4.81E-05 | -0.1058 | 382 | 0.744 |
| ENSP00000321195 | 1.52E-05 | -0.1058 | 151 | 0.386 |
| ENSP00000376135 | 4.03E-06 | -0.1059 | 0 | 0.686 |
| ENSP00000339767 | 4.22E-06 | -0.1059 | 0 | 0.690 |
| ENSP00000328625 | 5.67E-06 | -0.1059 | 0 | 0.000 |
| ENSP00000225430 | 2.16E-05 | -0.1059 | 186 | 0.715 |
| ENSP00000321656 | 2.30E-05 | -0.1059 | 198 | 0.775 |
| ENSP00000337501 | 1.28E-05 | -0.1059 | 216 | 0.000 |
| ENSP00000344162 | 3.48E-06 | -0.1059 | 0 | 0.688 |
| ENSP00000313881 | 9.19E-06 | -0.1059 | 0 | 0.209 |
| ENSP00000284273 | 2.02E-05 | -0.1059 | 485 | 0.869 |
| ENSP00000246337 | 1.21E-05 | -0.1059 | 359 | 0.658 |
| ENSP00000324204 | 1.58E-04 | -0.1059 | 0 | 0.000 |
| ENSP00000378792 | 3.57E-06 | -0.1059 | 0 | 0.707 |
| ENSP00000353089 | 1.93E-05 | -0.1060 | 318 | 0.787 |
| ENSP00000262288 | 3.85E-05 | -0.1060 | 161 | 0.169 |
| ENSP00000269025 | 6.25E-06 | -0.1060 | 0 | 0.495 |
| ENSP00000354553 | 5.24E-06 | -0.1060 | 0 | 0.000 |
| ENSP00000326879 | 1.81E-04 | -0.1060 | 0 | 0.169 |
| ENSP00000328187 | 3.68E-05 | -0.1060 | 0 | 0.097 |
| ENSP00000371070 | 1.98E-05 | -0.1060 | 165 | 0.151 |
| ENSP00000371084 | 1.22E-05 | -0.1060 | 0 | 0.124 |
| ENSP00000009041 | 8.41E-05 | -0.1060 | 0 | 0.139 |
| ENSP00000295959 | 2.82E-04 | -0.1060 | 0 | 0.129 |
| ENSP00000336866 | 4.65E-05 | -0.1060 | 150 | 0.180 |

| | | | | |
|---|---|---|---|---|
| ENSP00000275227 | 1.97E-04 | -0.1061 | 0 | 0.145 |
| ENSP00000233047 | 2.58E-04 | -0.1061 | 0 | 0.155 |
| ENSP00000305804 | 3.76E-06 | -0.1061 | 0 | 0.699 |
| ENSP00000358716 | 2.12E-05 | -0.1061 | 348 | 0.893 |
| ENSP00000299687 | 3.23E-06 | -0.1061 | 0 | 0.675 |
| ENSP00000289153 | 1.67E-05 | -0.1061 | 197 | 0.861 |
| ENSP00000261973 | 2.83E-05 | -0.1061 | 550 | 0.730 |
| ENSP00000364430 | 9.99E-06 | -0.1061 | 328 | 0.807 |
| ENSP00000340519 | 9.77E-05 | -0.1061 | 0 | 0.114 |
| ENSP00000245960 | 2.61E-05 | -0.1061 | 181 | 0.732 |
| ENSP00000338533 | 1.76E-04 | -0.1061 | 0 | 0.146 |
| ENSP00000378307 | 1.97E-05 | -0.1062 | 308 | 0.921 |
| ENSP00000364277 | 1.44E-05 | -0.1062 | 179 | 0.585 |
| ENSP00000237281 | 1.87E-04 | -0.1062 | 0 | 0.203 |
| ENSP00000313158 | 4.71E-06 | -0.1062 | 0 | 0.698 |
| ENSP00000383986 | 1.48E-05 | -0.1062 | 165 | 0.549 |
| ENSP00000181796 | 2.10E-04 | -0.1062 | 0 | 0.157 |
| ENSP00000216218 | 4.12E-05 | -0.1062 | 237 | 0.160 |
| ENSP00000339280 | 8.44E-06 | -0.1062 | 0 | 0.133 |
| ENSP00000274457 | 1.99E-05 | -0.1062 | 279 | 0.559 |
| ENSP00000366693 | 2.94E-06 | -0.1062 | 0 | 0.000 |
| ENSP00000244520 | 2.68E-05 | -0.1062 | 546 | 0.894 |
| ENSP00000217130 | 5.81E-06 | -0.1062 | 0 | 0.654 |
| ENSP00000322439 | 1.14E-05 | -0.1062 | 416 | 0.717 |
| ENSP00000290310 | 9.05E-06 | -0.1062 | 0 | 0.243 |
| ENSP00000375795 | 2.32E-05 | -0.1062 | 576 | 0.639 |
| ENSP00000349748 | 1.39E-05 | -0.1063 | 270 | 0.746 |
| ENSP00000376943 | 2.76E-05 | -0.1063 | 265 | 0.897 |
| ENSP00000333266 | 1.18E-05 | -0.1063 | 282 | 0.501 |
| ENSP00000413405 | 1.96E-05 | -0.1063 | 171 | 0.067 |
| ENSP00000310182 | 1.28E-04 | -0.1063 | 0 | 0.164 |
| ENSP00000270459 | 3.58E-06 | -0.1063 | 0 | 0.000 |
| ENSP00000272638 | 4.19E-05 | -0.1063 | 198 | 0.110 |
| ENSP00000266529 | 9.16E-05 | -0.1063 | 0 | 0.376 |
| ENSP00000471593 | 1.98E-06 | -0.1063 | 0 | 0.697 |
| ENSP00000471607 | 1.98E-06 | -0.1063 | 0 | 0.000 |
| ENSP00000471613 | 1.98E-06 | -0.1063 | 0 | 0.000 |
| ENSP00000472160 | 1.98E-06 | -0.1063 | 0 | 0.000 |
| ENSP00000472277 | 1.98E-06 | -0.1063 | 0 | 0.000 |
| ENSP00000472894 | 1.98E-06 | -0.1063 | 0 | 0.000 |
| ENSP00000473043 | 1.98E-06 | -0.1063 | 0 | 0.000 |
| ENSP00000473166 | 1.98E-06 | -0.1063 | 0 | 0.000 |
| ENSP00000473243 | 1.98E-06 | -0.1063 | 0 | 0.697 |
| ENSP00000302768 | 3.83E-05 | -0.1063 | 292 | 0.655 |
| ENSP00000268616 | 2.41E-05 | -0.1063 | 245 | 0.907 |
| ENSP00000304941 | 9.48E-05 | -0.1063 | 0 | 0.100 |

| | | | | |
|---|---|---|---|---|
| ENSP00000302021 | 1.92E-05 | -0.1063 | 300 | 0.080 |
| ENSP00000417509 | 6.88E-06 | -0.1063 | 0 | 0.142 |
| ENSP00000228850 | 5.51E-06 | -0.1063 | 0 | 0.041 |
| ENSP00000295958 | 1.12E-04 | -0.1063 | 0 | 0.117 |
| ENSP00000367545 | 9.94E-06 | -0.1063 | 0 | 0.807 |
| ENSP00000327589 | 5.07E-06 | -0.1063 | 0 | 0.443 |
| ENSP00000361467 | 1.11E-05 | -0.1063 | 190 | 0.465 |
| ENSP00000331106 | 8.79E-05 | -0.1063 | 0 | 0.052 |
| ENSP00000468271 | 1.98E-06 | -0.1063 | 0 | 0.000 |
| ENSP00000295598 | 7.68E-06 | -0.1063 | 211 | 0.442 |
| ENSP00000262291 | 2.73E-05 | -0.1063 | 198 | 0.685 |
| ENSP00000466387 | 1.99E-06 | -0.1064 | 0 | 0.000 |
| ENSP00000465960 | 1.99E-06 | -0.1064 | 0 | 0.671 |
| ENSP00000280560 | 1.07E-05 | -0.1064 | 160 | 0.374 |
| ENSP00000364166 | 1.92E-06 | -0.1064 | 0 | 0.113 |
| ENSP00000309570 | 4.09E-06 | -0.1064 | 0 | 0.759 |
| ENSP00000305995 | 2.12E-05 | -0.1064 | 364 | 0.602 |
| ENSP00000261991 | 1.15E-05 | -0.1064 | 303 | 0.000 |
| ENSP00000269834 | 5.16E-06 | -0.1064 | 0 | 0.707 |
| ENSP00000309640 | 7.76E-05 | -0.1064 | 0 | 0.148 |
| ENSP00000380921 | 8.96E-06 | -0.1064 | 0 | 0.606 |
| ENSP00000458124 | 1.99E-06 | -0.1064 | 0 | 0.000 |
| ENSP00000455659 | 1.99E-06 | -0.1064 | 0 | 0.000 |
| ENSP00000456213 | 1.99E-06 | -0.1064 | 0 | 0.000 |
| ENSP00000342148 | 8.81E-05 | -0.1064 | 0 | 0.081 |
| ENSP00000319726 | 7.42E-05 | -0.1064 | 0 | 0.119 |
| ENSP00000368984 | 6.69E-06 | -0.1064 | 0 | 0.869 |
| ENSP00000339208 | 3.22E-05 | -0.1064 | 584 | 0.259 |
| ENSP00000253159 | 1.30E-05 | -0.1064 | 195 | 0.676 |
| ENSP00000282388 | 2.51E-05 | -0.1064 | 227 | 0.654 |
| ENSP00000375859 | 3.46E-06 | -0.1065 | 0 | 0.130 |
| ENSP00000349250 | 2.17E-05 | -0.1065 | 274 | 0.497 |
| ENSP00000363500 | 1.32E-05 | -0.1065 | 805 | 0.198 |
| ENSP00000219551 | 1.57E-05 | -0.1065 | 288 | 0.388 |
| ENSP00000325448 | 9.97E-06 | -0.1065 | 334 | 0.768 |
| ENSP00000338290 | 4.59E-06 | -0.1065 | 0 | 0.700 |
| ENSP00000369820 | 7.58E-06 | -0.1065 | 0 | 0.411 |
| ENSP00000417686 | 2.76E-05 | -0.1065 | 211 | 0.843 |
| ENSP00000362824 | 1.61E-05 | -0.1065 | 451 | 0.000 |
| ENSP00000301945 | 3.99E-05 | -0.1065 | 482 | 0.360 |
| ENSP00000337797 | 1.98E-05 | -0.1065 | 471 | 0.000 |
| ENSP00000284503 | 4.20E-05 | -0.1065 | 0 | 0.284 |
| ENSP00000447931 | 2.00E-06 | -0.1065 | 0 | 0.697 |
| ENSP00000447987 | 2.00E-06 | -0.1065 | 0 | 0.000 |
| ENSP00000251864 | 2.18E-05 | -0.1065 | 273 | 0.295 |
| ENSP00000349669 | 1.80E-05 | -0.1065 | 291 | 0.000 |

| | | | | |
|---|---|---|---|---|
| ENSP00000306822 | 2.94E-05 | -0.1065 | 501 | 0.450 |
| ENSP00000008527 | 1.87E-05 | -0.1065 | 259 | 0.727 |
| ENSP00000285697 | 8.88E-05 | -0.1065 | 0 | 0.545 |
| ENSP00000255006 | 4.87E-05 | -0.1065 | 0 | 0.203 |
| ENSP00000274255 | 2.38E-05 | -0.1065 | 242 | 0.806 |
| ENSP00000441995 | 2.01E-06 | -0.1065 | 0 | 0.000 |
| ENSP00000230099 | 6.25E-05 | -0.1065 | 0 | 0.000 |
| ENSP00000292450 | 4.54E-06 | -0.1066 | 0 | 0.718 |
| ENSP00000250124 | 3.09E-05 | -0.1066 | 216 | 0.200 |
| ENSP00000219204 | 7.30E-05 | -0.1066 | 0 | 0.142 |
| ENSP00000296255 | 2.30E-05 | -0.1066 | 704 | 0.571 |
| ENSP00000285737 | 2.78E-05 | -0.1066 | 303 | 0.340 |
| ENSP00000396308 | 3.22E-05 | -0.1066 | 425 | 0.797 |
| ENSP00000298492 | 7.37E-05 | -0.1066 | 0 | 0.307 |
| ENSP00000215829 | 2.03E-05 | -0.1066 | 580 | 0.872 |
| ENSP00000307549 | 6.60E-05 | -0.1066 | 0 | 0.092 |
| ENSP00000326933 | 7.77E-05 | -0.1066 | 0 | 0.269 |
| ENSP00000307858 | 5.41E-06 | -0.1066 | 0 | 0.710 |
| ENSP00000322926 | 3.87E-05 | -0.1066 | 0 | 0.135 |
| ENSP00000357189 | 1.25E-05 | -0.1066 | 214 | 0.721 |
| ENSP00000278314 | 4.79E-06 | -0.1066 | 0 | 0.719 |
| ENSP00000358217 | 3.81E-06 | -0.1067 | 0 | 0.700 |
| ENSP00000295987 | 2.44E-05 | -0.1067 | 546 | 0.688 |
| ENSP00000334642 | 4.32E-05 | -0.1067 | 0 | 0.140 |
| ENSP00000238936 | 7.67E-05 | -0.1067 | 0 | 0.134 |
| ENSP00000261880 | 7.64E-05 | -0.1067 | 0 | 0.120 |
| ENSP00000323527 | 4.38E-05 | -0.1067 | 0 | 0.102 |
| ENSP00000265909 | 5.78E-05 | -0.1067 | 0 | 0.100 |
| ENSP00000204549 | 5.60E-05 | -0.1067 | 0 | 0.121 |
| ENSP00000198767 | 2.91E-05 | -0.1067 | 260 | 0.769 |
| ENSP00000223190 | 3.10E-05 | -0.1067 | 195 | 0.708 |
| ENSP00000285243 | 3.77E-05 | -0.1067 | 0 | 0.134 |
| ENSP00000280706 | 1.74E-05 | -0.1067 | 329 | 0.497 |
| ENSP00000410360 | 1.17E-06 | -0.1067 | 0 | 0.000 |
| ENSP00000262726 | 6.21E-06 | -0.1067 | 153 | 0.000 |
| ENSP00000332716 | 1.03E-04 | -0.1067 | 0 | 0.156 |
| ENSP00000330432 | 6.16E-05 | -0.1067 | 0 | 0.103 |
| ENSP00000345728 | 3.00E-05 | -0.1067 | 919 | 0.580 |
| ENSP00000354592 | 1.13E-05 | -0.1067 | 576 | 0.759 |
| ENSP00000413964 | 1.77E-05 | -0.1067 | 256 | 0.782 |
| ENSP00000337461 | 1.21E-05 | -0.1067 | 0 | 0.151 |
| ENSP00000341597 | 1.32E-04 | -0.1067 | 0 | 0.173 |
| ENSP00000421652 | 2.05E-06 | -0.1068 | 0 | 0.000 |
| ENSP00000250113 | 3.16E-05 | -0.1068 | 250 | 0.581 |
| ENSP00000438895 | 1.44E-07 | -0.1068 | 0 | 0.181 |
| ENSP00000263026 | 5.66E-05 | -0.1068 | 0 | 0.568 |

| | | | | |
|---|---|---|---|---|
| ENSP00000010299 | 5.63E-05 | -0.1068 | 0 | 0.313 |
| ENSP00000263549 | 3.63E-05 | -0.1068 | 0 | 0.227 |
| ENSP00000075503 | 7.30E-06 | -0.1068 | 568 | 0.682 |
| ENSP00000365730 | 2.95E-05 | -0.1068 | 520 | 0.652 |
| ENSP00000358575 | 2.91E-05 | -0.1068 | 197 | 0.000 |
| ENSP00000300146 | 2.28E-05 | -0.1068 | 220 | 0.752 |
| ENSP00000471714 | 1.98E-06 | -0.1068 | 0 | 0.000 |
| ENSP00000289431 | 4.20E-05 | -0.1068 | 0 | 0.167 |
| ENSP00000263849 | 5.94E-05 | -0.1068 | 0 | 0.163 |
| ENSP00000350576 | 4.30E-06 | -0.1068 | 0 | 0.693 |
| ENSP00000286719 | 1.18E-05 | -0.1068 | 713 | 0.652 |
| ENSP00000215794 | 6.22E-06 | -0.1068 | 0 | 0.270 |
| ENSP00000275732 | 3.84E-05 | -0.1068 | 0 | 0.355 |
| ENSP00000282441 | 2.46E-05 | -0.1068 | 983 | 0.833 |
| ENSP00000301329 | 4.73E-05 | -0.1068 | 0 | 0.114 |
| ENSP00000353246 | 5.48E-06 | -0.1068 | 0 | 0.866 |
| ENSP00000331577 | 3.83E-06 | -0.1068 | 0 | 0.678 |
| ENSP00000356520 | 2.87E-05 | -0.1069 | 368 | 0.874 |
| ENSP00000313829 | 3.45E-05 | -0.1069 | 357 | 0.791 |
| ENSP00000259818 | 1.45E-05 | -0.1069 | 406 | 0.616 |
| ENSP00000339933 | 1.31E-05 | -0.1069 | 311 | 0.767 |
| ENSP00000326119 | 3.35E-05 | -0.1069 | 158 | 0.756 |
| ENSP00000254624 | 5.80E-05 | -0.1069 | 0 | 0.141 |
| ENSP00000376767 | 7.57E-06 | -0.1069 | 0 | 0.164 |
| ENSP00000299157 | 3.92E-05 | -0.1069 | 0 | 0.106 |
| ENSP00000344786 | 3.13E-05 | -0.1069 | 914 | 0.441 |
| ENSP00000308845 | 9.18E-06 | -0.1069 | 360 | 0.641 |
| ENSP00000274000 | 4.04E-05 | -0.1069 | 0 | 0.072 |
| ENSP00000233055 | 7.98E-06 | -0.1069 | 0 | 0.401 |
| ENSP00000292672 | 3.73E-05 | -0.1069 | 0 | 0.164 |
| ENSP00000431759 | 4.38E-06 | -0.1069 | 0 | 0.000 |
| ENSP00000264312 | 3.78E-05 | -0.1069 | 0 | 0.125 |
| ENSP00000354414 | 2.97E-06 | -0.1069 | 0 | 0.685 |
| ENSP00000419397 | 2.07E-06 | -0.1069 | 0 | 0.697 |
| ENSP00000316007 | 4.52E-05 | -0.1069 | 0 | 0.124 |
| ENSP00000361835 | 3.10E-05 | -0.1069 | 237 | 0.000 |
| ENSP00000001146 | 3.37E-05 | -0.1069 | 493 | 0.313 |
| ENSP00000361672 | 3.04E-06 | -0.1069 | 0 | 0.000 |
| ENSP00000284274 | 6.76E-05 | -0.1070 | 0 | 0.000 |
| ENSP00000303248 | 1.22E-05 | -0.1070 | 357 | 0.677 |
| ENSP00000264993 | 4.36E-05 | -0.1070 | 0 | 0.179 |
| ENSP00000379607 | 4.70E-06 | -0.1070 | 0 | 0.213 |
| ENSP00000323879 | 3.66E-05 | -0.1070 | 0 | 0.112 |
| ENSP00000232905 | 2.51E-05 | -0.1070 | 311 | 0.695 |
| ENSP00000261520 | 5.89E-05 | -0.1070 | 0 | 0.000 |
| ENSP00000246551 | 3.21E-05 | -0.1070 | 0 | 0.444 |

| | | | | |
|---|---|---|---|---|
| ENSP00000348838 | 8.17E-06 | -0.1070 | 0 | 0.539 |
| ENSP00000288680 | 3.12E-05 | -0.1070 | 0 | 0.166 |
| ENSP00000301096 | 3.80E-06 | -0.1070 | 0 | 0.706 |
| ENSP00000383587 | 3.26E-06 | -0.1070 | 0 | 0.352 |
| ENSP00000348459 | 4.17E-06 | -0.1070 | 0 | 0.035 |
| ENSP00000367835 | 3.66E-06 | -0.1070 | 0 | 0.806 |
| ENSP00000309432 | 4.55E-05 | -0.1070 | 0 | 0.087 |
| ENSP00000234301 | 5.12E-05 | -0.1071 | 0 | 0.120 |
| ENSP00000346964 | 1.11E-05 | -0.1071 | 203 | 0.416 |
| ENSP00000342322 | 3.39E-05 | -0.1071 | 0 | 0.138 |
| ENSP00000258439 | 4.81E-05 | -0.1071 | 0 | 0.064 |
| ENSP00000329117 | 2.42E-05 | -0.1071 | 0 | 0.633 |
| ENSP00000355353 | 1.95E-05 | -0.1071 | 242 | 0.098 |
| ENSP00000256649 | 3.53E-05 | -0.1071 | 0 | 0.152 |
| ENSP00000315182 | 3.36E-05 | -0.1071 | 0 | 0.388 |
| ENSP00000255759 | 3.87E-05 | -0.1071 | 0 | 0.090 |
| ENSP00000314949 | 2.65E-05 | -0.1071 | 383 | 0.000 |
| ENSP00000001008 | 1.60E-05 | -0.1071 | 392 | 0.794 |
| ENSP00000318089 | 3.14E-05 | -0.1071 | 644 | 0.000 |
| ENSP00000274217 | 5.58E-05 | -0.1071 | 0 | 0.092 |
| ENSP00000228437 | 4.14E-06 | -0.1072 | 0 | 0.728 |
| ENSP00000301335 | 3.16E-05 | -0.1072 | 0 | 0.082 |
| ENSP00000239231 | 3.65E-05 | -0.1072 | 0 | 0.161 |
| ENSP00000358698 | 2.58E-05 | -0.1072 | 622 | 0.914 |
| ENSP00000357459 | 1.60E-05 | -0.1072 | 198 | 0.703 |
| ENSP00000300850 | 4.45E-06 | -0.1072 | 0 | 0.686 |
| ENSP00000343002 | 2.85E-05 | -0.1072 | 0 | 0.000 |
| ENSP00000309175 | 2.71E-05 | -0.1072 | 0 | 0.375 |
| ENSP00000341961 | 5.52E-06 | -0.1072 | 0 | 0.000 |
| ENSP00000251809 | 8.94E-06 | -0.1072 | 358 | 0.705 |
| ENSP00000263519 | 5.82E-06 | -0.1072 | 0 | 0.630 |
| ENSP00000337222 | 3.21E-05 | -0.1072 | 0 | 0.092 |
| ENSP00000411132 | 2.09E-06 | -0.1072 | 0 | 0.697 |
| ENSP00000316898 | 2.28E-05 | -0.1072 | 0 | 0.191 |
| ENSP00000323313 | 7.14E-06 | -0.1072 | 516 | 0.554 |
| ENSP00000300952 | 3.72E-05 | -0.1072 | 0 | 0.093 |
| ENSP00000314077 | 9.29E-06 | -0.1072 | 153 | 0.000 |
| ENSP00000293879 | 5.12E-06 | -0.1072 | 242 | 0.000 |
| ENSP00000262370 | 3.04E-05 | -0.1072 | 0 | 0.061 |
| ENSP00000372105 | 3.22E-06 | -0.1072 | 0 | 0.144 |
| ENSP00000376824 | 7.27E-07 | -0.1072 | 0 | 0.172 |
| ENSP00000410545 | 2.09E-06 | -0.1072 | 0 | 0.697 |
| ENSP00000260648 | 3.28E-05 | -0.1073 | 676 | 0.094 |
| ENSP00000234831 | 3.83E-05 | -0.1073 | 0 | 0.190 |
| ENSP00000276569 | 4.31E-05 | -0.1073 | 0 | 0.105 |
| ENSP00000340281 | 3.36E-05 | -0.1073 | 445 | 0.550 |

| | | | | |
|---|---|---|---|---|
| ENSP00000363418 | 8.60E-06 | -0.1073 | 0 | 0.206 |
| ENSP00000264447 | 2.29E-05 | -0.1073 | 0 | 0.548 |
| ENSP00000204726 | 3.73E-05 | -0.1073 | 0 | 0.082 |
| ENSP00000355922 | 4.10E-06 | -0.1073 | 0 | 0.431 |
| ENSP00000363054 | 2.50E-05 | -0.1073 | 257 | 0.220 |
| ENSP00000259746 | 3.30E-05 | -0.1073 | 0 | 0.102 |
| ENSP00000332744 | 1.11E-05 | -0.1073 | 169 | 0.848 |
| ENSP00000414887 | 2.09E-06 | -0.1073 | 0 | 0.698 |
| ENSP00000328694 | 7.64E-06 | -0.1073 | 171 | 0.658 |
| ENSP00000308179 | 3.80E-06 | -0.1073 | 0 | 0.865 |
| ENSP00000378359 | 1.57E-05 | -0.1074 | 432 | 0.293 |
| ENSP00000291458 | 2.96E-05 | -0.1074 | 0 | 0.126 |
| ENSP00000259939 | 3.26E-05 | -0.1074 | 0 | 0.208 |
| ENSP00000321706 | 3.59E-05 | -0.1074 | 163 | 0.619 |
| ENSP00000409652 | 2.10E-06 | -0.1074 | 0 | 0.697 |
| ENSP00000265350 | 2.79E-05 | -0.1074 | 0 | 0.681 |
| ENSP00000319482 | 3.41E-05 | -0.1074 | 0 | 0.226 |
| ENSP00000286307 | 2.97E-05 | -0.1074 | 0 | 0.508 |
| ENSP00000361705 | 5.23E-06 | -0.1074 | 0 | 0.303 |
| ENSP00000380315 | 6.37E-06 | -0.1074 | 0 | 0.686 |
| ENSP00000335397 | 5.97E-06 | -0.1074 | 0 | 0.720 |
| ENSP00000301264 | 8.67E-06 | -0.1074 | 800 | 0.672 |
| ENSP00000316042 | 3.53E-05 | -0.1074 | 246 | 0.862 |
| ENSP00000340427 | 2.49E-05 | -0.1074 | 0 | 0.092 |
| ENSP00000312397 | 2.54E-05 | -0.1074 | 0 | 0.087 |
| ENSP00000328178 | 2.52E-05 | -0.1074 | 0 | 0.000 |
| ENSP00000280756 | 4.73E-05 | -0.1074 | 0 | 0.000 |
| ENSP00000373715 | 7.21E-06 | -0.1074 | 195 | 0.744 |
| ENSP00000258739 | 1.98E-05 | -0.1074 | 276 | 0.147 |
| ENSP00000286091 | 1.85E-05 | -0.1074 | 949 | 0.629 |
| ENSP00000314655 | 1.87E-05 | -0.1074 | 0 | 0.761 |
| ENSP00000265843 | 3.78E-05 | -0.1074 | 0 | 0.114 |
| ENSP00000238892 | 2.54E-05 | -0.1074 | 369 | 0.237 |
| ENSP00000320663 | 4.86E-05 | -0.1075 | 198 | 0.625 |
| ENSP00000231512 | 3.23E-05 | -0.1075 | 0 | 0.099 |
| ENSP00000338727 | 3.52E-05 | -0.1075 | 0 | 0.123 |
| ENSP00000322373 | 2.18E-05 | -0.1075 | 0 | 0.118 |
| ENSP00000320940 | 2.60E-05 | -0.1075 | 900 | 0.804 |
| ENSP00000292433 | 1.13E-04 | -0.1075 | 0 | 0.434 |
| ENSP00000258648 | 2.40E-05 | -0.1075 | 571 | 0.693 |
| ENSP00000205636 | 3.15E-05 | -0.1075 | 0 | 0.130 |
| ENSP00000299192 | 5.93E-05 | -0.1075 | 0 | 0.643 |
| ENSP00000272748 | 2.92E-05 | -0.1075 | 0 | 0.154 |
| ENSP00000407653 | 3.58E-07 | -0.1075 | 0 | 0.203 |
| ENSP00000242810 | 2.86E-05 | -0.1075 | 0 | 0.080 |
| ENSP00000238875 | 3.05E-05 | -0.1075 | 0 | 0.101 |

| | | | | |
|---|---|---|---|---|
| ENSP00000248706 | 9.51E-06 | -0.1075 | 448 | 0.596 |
| ENSP00000267294 | 4.30E-06 | -0.1075 | 0 | 0.806 |
| ENSP00000156825 | 3.17E-05 | -0.1075 | 0 | 0.786 |
| ENSP00000367193 | 2.96E-08 | -0.1075 | 0 | 0.217 |
| ENSP00000261739 | 3.87E-05 | -0.1075 | 0 | 0.112 |
| ENSP00000306220 | 2.98E-05 | -0.1075 | 0 | 0.093 |
| ENSP00000309509 | 2.77E-05 | -0.1075 | 0 | 0.139 |
| ENSP00000173527 | 3.14E-05 | -0.1075 | 0 | 0.117 |
| ENSP00000312678 | 3.03E-05 | -0.1075 | 0 | 0.253 |
| ENSP00000294353 | 2.81E-05 | -0.1075 | 0 | 0.232 |
| ENSP00000352035 | 2.21E-05 | -0.1075 | 900 | 0.370 |
| ENSP00000335201 | 2.35E-05 | -0.1076 | 0 | 0.188 |
| ENSP00000300231 | 2.36E-05 | -0.1076 | 0 | 0.277 |
| ENSP00000257787 | 2.95E-05 | -0.1076 | 0 | 0.154 |
| ENSP00000438863 | 3.13E-06 | -0.1076 | 0 | 0.158 |
| ENSP00000293760 | 2.92E-05 | -0.1076 | 221 | 0.522 |
| ENSP00000252599 | 1.35E-04 | -0.1076 | 0 | 0.120 |
| ENSP00000305556 | 2.26E-05 | -0.1076 | 352 | 0.877 |
| ENSP00000265388 | 4.20E-05 | -0.1076 | 323 | 0.467 |
| ENSP00000455300 | 1.98E-06 | -0.1076 | 0 | 0.000 |
| ENSP00000342830 | 2.64E-05 | -0.1076 | 307 | 0.775 |
| ENSP00000289382 | 3.45E-05 | -0.1076 | 0 | 0.283 |
| ENSP00000315263 | 2.39E-05 | -0.1076 | 0 | 0.096 |
| ENSP00000314724 | 2.94E-05 | -0.1076 | 0 | 0.119 |
| ENSP00000390661 | 9.53E-07 | -0.1076 | 0 | 0.147 |
| ENSP00000253363 | 1.88E-05 | -0.1076 | 221 | 0.883 |
| ENSP00000272995 | 2.81E-05 | -0.1076 | 0 | 0.313 |
| ENSP00000225983 | 1.58E-05 | -0.1077 | 323 | 0.000 |
| ENSP00000349496 | 9.40E-06 | -0.1077 | 0 | 0.111 |
| ENSP00000275072 | 3.66E-05 | -0.1077 | 0 | 0.121 |
| ENSP00000281950 | 3.12E-05 | -0.1077 | 0 | 0.616 |
| ENSP00000273986 | 2.07E-05 | -0.1077 | 179 | 0.099 |
| ENSP00000344544 | 2.10E-05 | -0.1077 | 199 | 0.833 |
| ENSP00000355308 | 1.35E-05 | -0.1077 | 284 | 0.000 |
| ENSP00000371722 | 1.02E-06 | -0.1077 | 0 | 0.118 |
| ENSP00000297578 | 2.14E-05 | -0.1077 | 0 | 0.093 |
| ENSP00000253458 | 2.35E-05 | -0.1077 | 0 | 0.186 |
| ENSP00000257909 | 2.58E-05 | -0.1077 | 0 | 0.204 |
| ENSP00000336856 | 3.10E-05 | -0.1077 | 269 | 0.934 |
| ENSP00000246151 | 4.59E-05 | -0.1077 | 0 | 0.096 |
| ENSP00000297857 | 6.16E-05 | -0.1077 | 184 | 0.530 |
| ENSP00000260956 | 1.74E-05 | -0.1077 | 518 | 0.934 |
| ENSP00000292432 | 5.88E-06 | -0.1077 | 202 | 0.654 |
| ENSP00000373726 | 2.17E-06 | -0.1077 | 0 | 0.114 |
| ENSP00000293883 | 5.24E-06 | -0.1077 | 0 | 0.090 |
| ENSP00000325868 | 3.80E-06 | -0.1077 | 0 | 0.074 |

| | | | | |
|---|---|---|---|---|
| ENSP00000255305 | 3.21E-05 | -0.1078 | 0 | 0.238 |
| ENSP00000355621 | 8.45E-06 | -0.1078 | 316 | 0.557 |
| ENSP00000249064 | 2.74E-05 | -0.1078 | 0 | 0.208 |
| ENSP00000318804 | 2.69E-05 | -0.1078 | 0 | 0.090 |
| ENSP00000321724 | 1.88E-05 | -0.1078 | 167 | 0.345 |
| ENSP00000296666 | 4.30E-05 | -0.1078 | 0 | 0.132 |
| ENSP00000255416 | 2.43E-05 | -0.1078 | 0 | 0.167 |
| ENSP00000360824 | 2.73E-06 | -0.1078 | 0 | 0.143 |
| ENSP00000445446 | 2.56E-06 | -0.1078 | 0 | 0.118 |
| ENSP00000233084 | 1.63E-05 | -0.1078 | 305 | 0.930 |
| ENSP00000263960 | 2.01E-05 | -0.1078 | 0 | 0.086 |
| ENSP00000366156 | 3.59E-06 | -0.1078 | 0 | 0.340 |
| ENSP00000291592 | 4.95E-06 | -0.1078 | 0 | 0.675 |
| ENSP00000296682 | 3.73E-06 | -0.1078 | 0 | 0.698 |
| ENSP00000306524 | 5.81E-06 | -0.1079 | 0 | 0.673 |
| ENSP00000304985 | 7.15E-06 | -0.1079 | 150 | 0.724 |
| ENSP00000356363 | 1.40E-05 | -0.1079 | 0 | 0.209 |
| ENSP00000306788 | 2.63E-05 | -0.1079 | 0 | 0.070 |
| ENSP00000292879 | 2.14E-05 | -0.1079 | 0 | 0.747 |
| ENSP00000310471 | 6.48E-05 | -0.1079 | 0 | 0.414 |
| ENSP00000231487 | 1.25E-05 | -0.1079 | 319 | 0.846 |
| ENSP00000416097 | 1.93E-05 | -0.1079 | 313 | 0.280 |
| ENSP00000263377 | 2.49E-05 | -0.1079 | 340 | 0.920 |
| ENSP00000271620 | 3.51E-05 | -0.1079 | 0 | 0.058 |
| ENSP00000337443 | 2.18E-05 | -0.1079 | 0 | 0.121 |
| ENSP00000306123 | 2.23E-05 | -0.1079 | 0 | 0.439 |
| ENSP00000283006 | 2.05E-05 | -0.1079 | 0 | 0.387 |
| ENSP00000258821 | 2.55E-05 | -0.1079 | 0 | 0.164 |
| ENSP00000338258 | 4.11E-05 | -0.1079 | 0 | 0.066 |
| ENSP00000216144 | 4.34E-06 | -0.1079 | 153 | 0.000 |
| ENSP00000247178 | 2.00E-05 | -0.1079 | 0 | 0.240 |
| ENSP00000261858 | 2.02E-05 | -0.1080 | 0 | 0.524 |
| ENSP00000372724 | 9.09E-06 | -0.1080 | 0 | 0.181 |
| ENSP00000437878 | 2.03E-06 | -0.1080 | 0 | 0.000 |
| ENSP00000345708 | 1.79E-05 | -0.1080 | 264 | 0.782 |
| ENSP00000299381 | 2.96E-05 | -0.1080 | 0 | 0.169 |
| ENSP00000011898 | 2.07E-05 | -0.1080 | 0 | 0.121 |
| ENSP00000348596 | 4.86E-06 | -0.1080 | 0 | 0.857 |
| ENSP00000270257 | 2.54E-05 | -0.1080 | 0 | 0.573 |
| ENSP00000312143 | 5.61E-06 | -0.1080 | 0 | 0.753 |
| ENSP00000333948 | 2.06E-05 | -0.1080 | 0 | 0.498 |
| ENSP00000295470 | 3.26E-05 | -0.1080 | 425 | 0.000 |
| ENSP00000344424 | 2.88E-05 | -0.1080 | 0 | 0.000 |
| ENSP00000332247 | 2.18E-05 | -0.1080 | 215 | 0.196 |
| ENSP00000337226 | 2.79E-05 | -0.1080 | 0 | 0.251 |
| ENSP00000343581 | 4.53E-06 | -0.1080 | 0 | 0.690 |

| | | | | |
|---|---|---|---|---|
| ENSP00000264193 | 2.04E-05 | -0.1080 | 294 | 0.546 |
| ENSP00000229595 | 1.58E-05 | -0.1080 | 427 | 0.946 |
| ENSP00000261441 | 1.95E-05 | -0.1080 | 0 | 0.089 |
| ENSP00000375598 | 3.37E-06 | -0.1080 | 0 | 0.688 |
| ENSP00000252329 | 2.18E-05 | -0.1081 | 0 | 0.161 |
| ENSP00000258198 | 1.97E-05 | -0.1081 | 0 | 0.167 |
| ENSP00000221200 | 2.04E-05 | -0.1081 | 0 | 0.090 |
| ENSP00000381290 | 8.77E-07 | -0.1081 | 0 | 0.341 |
| ENSP00000340329 | 1.84E-05 | -0.1081 | 0 | 0.682 |
| ENSP00000273963 | 2.13E-05 | -0.1081 | 0 | 0.114 |
| ENSP00000338629 | 3.60E-05 | -0.1081 | 364 | 0.817 |
| ENSP00000333255 | 2.00E-05 | -0.1081 | 0 | 0.103 |
| ENSP00000377873 | 5.39E-06 | -0.1081 | 0 | 0.701 |
| ENSP00000333019 | 4.72E-06 | -0.1081 | 0 | 0.461 |
| ENSP00000360154 | 2.10E-05 | -0.1081 | 227 | 0.363 |
| ENSP00000229395 | 2.43E-05 | -0.1081 | 0 | 0.155 |
| ENSP00000361186 | 1.43E-05 | -0.1081 | 0 | 0.367 |
| ENSP00000262992 | 2.01E-05 | -0.1081 | 0 | 0.115 |
| ENSP00000005340 | 1.27E-05 | -0.1082 | 652 | 0.898 |
| ENSP00000281419 | 4.80E-05 | -0.1082 | 242 | 0.297 |
| ENSP00000268483 | 2.03E-05 | -0.1082 | 172 | 0.582 |
| ENSP00000287461 | 4.62E-06 | -0.1082 | 0 | 0.698 |
| ENSP00000340118 | 2.11E-05 | -0.1082 | 0 | 0.324 |
| ENSP00000216513 | 8.12E-06 | -0.1082 | 0 | 0.794 |
| ENSP00000316779 | 2.46E-05 | -0.1082 | 535 | 0.430 |
| ENSP00000296135 | 2.74E-05 | -0.1082 | 0 | 0.103 |
| ENSP00000265069 | 1.75E-05 | -0.1082 | 0 | 0.466 |
| ENSP00000269701 | 5.99E-05 | -0.1082 | 0 | 0.499 |
| ENSP00000242209 | 1.86E-05 | -0.1082 | 304 | 0.413 |
| ENSP00000314901 | 4.87E-06 | -0.1082 | 0 | 0.656 |
| ENSP00000362390 | 6.08E-06 | -0.1082 | 0 | 0.757 |
| ENSP00000405726 | 2.27E-05 | -0.1082 | 228 | 0.799 |
| ENSP00000342267 | 2.00E-05 | -0.1082 | 0 | 0.171 |
| ENSP00000326563 | 4.96E-05 | -0.1082 | 0 | 0.495 |
| ENSP00000221086 | 1.98E-05 | -0.1082 | 0 | 0.148 |
| ENSP00000323264 | 2.09E-05 | -0.1082 | 0 | 0.146 |
| ENSP00000193322 | 2.01E-05 | -0.1082 | 0 | 0.071 |
| ENSP00000278319 | 3.85E-06 | -0.1082 | 0 | 0.702 |
| ENSP00000349415 | 4.82E-06 | -0.1082 | 0 | 0.183 |
| ENSP00000309831 | 2.25E-05 | -0.1082 | 0 | 0.524 |
| ENSP00000342755 | 1.84E-05 | -0.1082 | 0 | 0.428 |
| ENSP00000411032 | 2.13E-06 | -0.1082 | 0 | 0.696 |
| ENSP00000304565 | 2.41E-05 | -0.1082 | 178 | 0.000 |
| ENSP00000258796 | 2.52E-05 | -0.1082 | 0 | 0.063 |
| ENSP00000301454 | 2.25E-05 | -0.1082 | 0 | 0.116 |
| ENSP00000262844 | 1.92E-05 | -0.1083 | 0 | 0.052 |

| | | | | |
|---|---|---|---|---|
| ENSP00000355323 | 4.93E-06 | -0.1083 | 0 | 0.000 |
| ENSP00000236137 | 1.78E-05 | -0.1083 | 0 | 0.056 |
| ENSP00000309610 | 1.93E-05 | -0.1083 | 0 | 0.246 |
| ENSP00000326581 | 1.00E-05 | -0.1083 | 202 | 0.787 |
| ENSP00000440207 | 3.43E-06 | -0.1083 | 0 | 0.218 |
| ENSP00000338523 | 2.30E-05 | -0.1083 | 0 | 0.100 |
| ENSP00000294066 | 5.74E-06 | -0.1083 | 246 | 0.638 |
| ENSP00000364188 | 2.07E-05 | -0.1083 | 326 | 0.498 |
| ENSP00000340836 | 1.89E-05 | -0.1083 | 217 | 0.406 |
| ENSP00000309539 | 1.99E-05 | -0.1083 | 202 | 0.530 |
| ENSP00000254799 | 2.17E-05 | -0.1083 | 0 | 0.497 |
| ENSP00000359128 | 3.45E-06 | -0.1083 | 0 | 0.223 |
| ENSP00000282003 | 7.63E-05 | -0.1083 | 0 | 0.328 |
| ENSP00000301318 | 4.11E-06 | -0.1083 | 0 | 0.703 |
| ENSP00000301281 | 2.08E-05 | -0.1083 | 0 | 0.102 |
| ENSP00000324628 | 1.83E-05 | -0.1083 | 0 | 0.211 |
| ENSP00000216039 | 1.87E-05 | -0.1083 | 0 | 0.076 |
| ENSP00000220507 | 7.60E-06 | -0.1084 | 204 | 0.567 |
| ENSP00000297524 | 1.66E-05 | -0.1084 | 0 | 0.075 |
| ENSP00000298912 | 1.81E-05 | -0.1084 | 347 | 0.441 |
| ENSP00000261402 | 2.35E-05 | -0.1084 | 0 | 0.450 |
| ENSP00000328103 | 1.68E-05 | -0.1084 | 0 | 0.395 |
| ENSP00000348673 | 5.72E-06 | -0.1084 | 0 | 0.692 |
| ENSP00000336729 | 2.43E-05 | -0.1084 | 0 | 0.066 |
| ENSP00000399664 | 4.18E-06 | -0.1084 | 0 | 0.704 |
| ENSP00000321573 | 2.34E-05 | -0.1084 | 0 | 0.128 |
| ENSP00000350719 | 2.30E-05 | -0.1084 | 347 | 0.476 |
| ENSP00000317300 | 1.61E-05 | -0.1084 | 0 | 0.143 |
| ENSP00000308369 | 2.36E-05 | -0.1084 | 221 | 0.430 |
| ENSP00000309790 | 3.12E-05 | -0.1084 | 0 | 0.366 |
| ENSP00000335808 | 3.27E-05 | -0.1084 | 0 | 0.131 |
| ENSP00000270001 | 4.56E-06 | -0.1084 | 0 | 0.688 |
| ENSP00000346255 | 2.42E-05 | -0.1084 | 345 | 0.499 |
| ENSP00000300835 | 1.98E-05 | -0.1084 | 0 | 0.123 |
| ENSP00000369757 | 2.56E-05 | -0.1084 | 311 | 0.819 |
| ENSP00000377496 | 1.80E-05 | -0.1084 | 311 | 0.305 |
| ENSP00000334140 | 3.98E-06 | -0.1084 | 0 | 0.165 |
| ENSP00000264409 | 1.72E-05 | -0.1084 | 155 | 0.000 |
| ENSP00000238647 | 4.17E-05 | -0.1084 | 0 | 0.111 |
| ENSP00000256495 | 1.80E-05 | -0.1084 | 195 | 0.840 |
| ENSP00000313691 | 1.82E-05 | -0.1084 | 225 | 0.322 |
| ENSP00000261263 | 2.15E-05 | -0.1085 | 0 | 0.126 |
| ENSP00000260926 | 1.75E-05 | -0.1085 | 163 | 0.859 |
| ENSP00000256496 | 1.86E-05 | -0.1085 | 0 | 0.076 |
| ENSP00000247956 | 3.58E-06 | -0.1085 | 0 | 0.649 |
| ENSP00000349796 | 3.83E-06 | -0.1085 | 0 | 0.674 |

| | | | | |
|---|---|---|---|---|
| ENSP00000381960 | 5.75E-06 | -0.1085 | 0 | 0.000 |
| ENSP00000325285 | 2.17E-05 | -0.1085 | 0 | 0.070 |
| ENSP00000329212 | 9.02E-06 | -0.1085 | 233 | 0.000 |
| ENSP00000312277 | 4.38E-06 | -0.1085 | 0 | 0.675 |
| ENSP00000438590 | 4.34E-06 | -0.1085 | 0 | 0.121 |
| ENSP00000261893 | 1.90E-05 | -0.1085 | 205 | 0.178 |
| ENSP00000387911 | 2.23E-05 | -0.1085 | 161 | 0.707 |
| ENSP00000266735 | 2.07E-05 | -0.1085 | 402 | 0.811 |
| ENSP00000265260 | 1.96E-04 | -0.1085 | 252 | 0.143 |
| ENSP00000334625 | 1.39E-05 | -0.1085 | 0 | 0.095 |
| ENSP00000264451 | 1.52E-05 | -0.1085 | 0 | 0.419 |
| ENSP00000280665 | 1.95E-05 | -0.1085 | 0 | 0.757 |
| ENSP00000262198 | 1.45E-05 | -0.1085 | 0 | 0.253 |
| ENSP00000269195 | 1.65E-05 | -0.1085 | 0 | 0.157 |
| ENSP00000383396 | 3.94E-07 | -0.1085 | 0 | 0.168 |
| ENSP00000470691 | 2.09E-06 | -0.1085 | 0 | 0.000 |
| ENSP00000322341 | 1.80E-05 | -0.1085 | 333 | 0.658 |
| ENSP00000371419 | 1.90E-05 | -0.1086 | 204 | 0.581 |
| ENSP00000264254 | 1.32E-05 | -0.1086 | 0 | 0.111 |
| ENSP00000388920 | 3.69E-06 | -0.1086 | 0 | 0.577 |
| ENSP00000358501 | 2.26E-05 | -0.1086 | 581 | 0.800 |
| ENSP00000263102 | 5.21E-05 | -0.1086 | 196 | 0.547 |
| ENSP00000225792 | 1.18E-05 | -0.1086 | 398 | 0.888 |
| ENSP00000297792 | 2.81E-06 | -0.1086 | 0 | 0.000 |
| ENSP00000423630 | 1.90E-05 | -0.1086 | 0 | 0.096 |
| ENSP00000276594 | 3.94E-06 | -0.1086 | 0 | 0.772 |
| ENSP00000265322 | 1.85E-05 | -0.1086 | 161 | 0.314 |
| ENSP00000329794 | 2.00E-06 | -0.1086 | 0 | 0.135 |
| ENSP00000289004 | 1.83E-05 | -0.1086 | 0 | 0.192 |
| ENSP00000222345 | 2.02E-05 | -0.1086 | 0 | 0.080 |
| ENSP00000258349 | 1.56E-05 | -0.1086 | 0 | 0.404 |
| ENSP00000273286 | 4.60E-06 | -0.1086 | 0 | 0.582 |
| ENSP00000372410 | 3.26E-07 | -0.1086 | 0 | 0.192 |
| ENSP00000326531 | 3.61E-05 | -0.1087 | 0 | 0.565 |
| ENSP00000344220 | 2.49E-05 | -0.1087 | 205 | 0.886 |
| ENSP00000361882 | 1.93E-05 | -0.1087 | 0 | 0.000 |
| ENSP00000262225 | 2.00E-05 | -0.1087 | 276 | 0.170 |
| ENSP00000463832 | 8.37E-07 | -0.1087 | 0 | 0.000 |
| ENSP00000331111 | 1.91E-05 | -0.1087 | 222 | 0.760 |
| ENSP00000331787 | 2.05E-05 | -0.1087 | 0 | 0.084 |
| ENSP00000370376 | 3.53E-05 | -0.1087 | 225 | 0.720 |
| ENSP00000305918 | 2.14E-05 | -0.1087 | 246 | 0.931 |
| ENSP00000224807 | 3.37E-05 | -0.1087 | 0 | 0.086 |
| ENSP00000257177 | 3.08E-05 | -0.1087 | 159 | 0.770 |
| ENSP00000309653 | 4.70E-06 | -0.1087 | 0 | 0.757 |
| ENSP00000216271 | 6.47E-06 | -0.1087 | 339 | 0.000 |

| | | | | |
|---|---|---|---|---|
| ENSP00000198765 | 1.51E-05 | -0.1087 | 0 | 0.215 |
| ENSP00000319974 | 3.14E-05 | -0.1087 | 0 | 0.309 |
| ENSP00000399392 | 1.03E-05 | -0.1087 | 357 | 0.126 |
| ENSP00000260803 | 1.72E-05 | -0.1087 | 0 | 0.713 |
| ENSP00000359073 | 2.06E-05 | -0.1088 | 150 | 0.750 |
| ENSP00000262056 | 1.72E-05 | -0.1088 | 255 | 0.936 |
| ENSP00000297375 | 1.79E-05 | -0.1088 | 382 | 0.864 |
| ENSP00000242351 | 2.82E-05 | -0.1088 | 0 | 0.352 |
| ENSP00000266069 | 2.54E-05 | -0.1088 | 0 | 0.209 |
| ENSP00000337053 | 1.96E-05 | -0.1088 | 811 | 0.643 |
| ENSP00000261332 | 4.49E-06 | -0.1088 | 0 | 0.707 |
| ENSP00000261596 | 8.72E-06 | -0.1088 | 163 | 0.662 |
| ENSP00000419057 | 2.08E-06 | -0.1088 | 0 | 0.000 |
| ENSP00000320043 | 1.73E-05 | -0.1088 | 0 | 0.147 |
| ENSP00000308368 | 4.89E-05 | -0.1088 | 0 | 0.160 |
| ENSP00000331057 | 2.13E-05 | -0.1088 | 235 | 0.840 |
| ENSP00000374455 | 2.47E-05 | -0.1088 | 466 | 0.794 |
| ENSP00000418001 | 1.80E-05 | -0.1089 | 305 | 0.345 |
| ENSP00000343617 | 4.91E-06 | -0.1089 | 0 | 0.697 |
| ENSP00000384979 | 5.50E-06 | -0.1089 | 0 | 0.000 |
| ENSP00000334499 | 3.73E-05 | -0.1089 | 666 | 0.717 |
| ENSP00000260327 | 1.48E-05 | -0.1089 | 0 | 0.324 |
| ENSP00000317614 | 3.77E-06 | -0.1089 | 0 | 0.675 |
| ENSP00000249356 | 1.40E-05 | -0.1089 | 979 | 0.386 |
| ENSP00000258729 | 1.25E-05 | -0.1089 | 163 | 0.568 |
| ENSP00000256637 | 1.86E-05 | -0.1089 | 381 | 0.237 |
| ENSP00000205557 | 2.22E-05 | -0.1089 | 203 | 0.480 |
| ENSP00000295971 | 2.26E-05 | -0.1089 | 0 | 0.274 |
| ENSP00000272647 | 2.22E-05 | -0.1089 | 0 | 0.052 |
| ENSP00000332756 | 1.19E-05 | -0.1089 | 151 | 0.392 |
| ENSP00000370128 | 1.20E-05 | -0.1089 | 186 | 0.410 |
| ENSP00000267938 | 1.45E-05 | -0.1089 | 0 | 0.096 |
| ENSP00000254998 | 2.31E-05 | -0.1089 | 0 | 0.643 |
| ENSP00000245407 | 1.54E-05 | -0.1089 | 0 | 0.163 |
| ENSP00000222382 | 6.18E-06 | -0.1089 | 0 | 0.210 |
| ENSP00000286523 | 2.79E-05 | -0.1090 | 0 | 0.583 |
| ENSP00000007699 | 1.58E-05 | -0.1090 | 302 | 0.724 |
| ENSP00000240139 | 7.50E-06 | -0.1090 | 224 | 0.652 |
| ENSP00000304198 | 6.11E-06 | -0.1090 | 0 | 0.694 |
| ENSP00000361626 | 2.27E-05 | -0.1090 | 850 | 0.783 |
| ENSP00000314733 | 2.09E-05 | -0.1090 | 0 | 0.388 |
| ENSP00000216554 | 2.02E-05 | -0.1090 | 459 | 0.880 |
| ENSP00000268655 | 3.44E-06 | -0.1090 | 0 | 0.644 |
| ENSP00000320503 | 1.65E-05 | -0.1090 | 0 | 0.573 |
| ENSP00000282878 | 5.08E-06 | -0.1090 | 0 | 0.181 |
| ENSP00000308753 | 2.57E-05 | -0.1090 | 0 | 0.190 |

| | | | | |
|---|---|---|---|---|
| ENSP00000220669 | 1.67E-05 | -0.1090 | 0 | 0.121 |
| ENSP00000265028 | 1.62E-05 | -0.1090 | 974 | 0.436 |
| ENSP00000247225 | 1.88E-05 | -0.1090 | 0 | 0.115 |
| ENSP00000268533 | 1.77E-05 | -0.1090 | 195 | 0.262 |
| ENSP00000162391 | 8.68E-06 | -0.1090 | 159 | 0.000 |
| ENSP00000318298 | 2.63E-05 | -0.1090 | 0 | 0.110 |
| ENSP00000275780 | 1.68E-05 | -0.1090 | 0 | 0.374 |
| ENSP00000266594 | 3.59E-05 | -0.1090 | 0 | 0.432 |
| ENSP00000296161 | 2.54E-05 | -0.1090 | 0 | 0.194 |
| ENSP00000234296 | 2.21E-06 | -0.1090 | 0 | 0.000 |
| ENSP00000293677 | 2.52E-05 | -0.1090 | 0 | 0.628 |
| ENSP00000350364 | 1.32E-05 | -0.1091 | 258 | 0.105 |
| ENSP00000249822 | 2.07E-05 | -0.1091 | 0 | 0.202 |
| ENSP00000376568 | 4.82E-06 | -0.1091 | 0 | 0.499 |
| ENSP00000303706 | 2.24E-05 | -0.1091 | 200 | 0.772 |
| ENSP00000337839 | 2.16E-05 | -0.1091 | 0 | 0.345 |
| ENSP00000312664 | 1.42E-05 | -0.1091 | 0 | 0.740 |
| ENSP00000262053 | 1.51E-05 | -0.1091 | 200 | 0.871 |
| ENSP00000329748 | 1.78E-05 | -0.1091 | 0 | 0.080 |
| ENSP00000439182 | 1.65E-05 | -0.1091 | 244 | 0.330 |
| ENSP00000431049 | 1.44E-05 | -0.1091 | 392 | 0.095 |
| ENSP00000261745 | 1.61E-05 | -0.1091 | 0 | 0.090 |
| ENSP00000228251 | 1.36E-05 | -0.1091 | 306 | 0.715 |
| ENSP00000278823 | 2.23E-05 | -0.1091 | 496 | 0.799 |
| ENSP00000261413 | 2.77E-05 | -0.1091 | 0 | 0.368 |
| ENSP00000292123 | 2.56E-05 | -0.1091 | 258 | 0.752 |
| ENSP00000352444 | 3.63E-06 | -0.1091 | 0 | 0.689 |
| ENSP00000253727 | 1.82E-05 | -0.1092 | 482 | 0.711 |
| ENSP00000334448 | 1.32E-05 | -0.1092 | 906 | 0.646 |
| ENSP00000229769 | 2.62E-05 | -0.1092 | 0 | 0.657 |
| ENSP00000327436 | 1.75E-05 | -0.1092 | 0 | 0.609 |
| ENSP00000335083 | 4.40E-06 | -0.1092 | 0 | 0.448 |
| ENSP00000310670 | 2.18E-05 | -0.1092 | 0 | 0.000 |
| ENSP00000307774 | 4.05E-06 | -0.1092 | 0 | 0.703 |
| ENSP00000347451 | 5.57E-06 | -0.1092 | 0 | 0.764 |
| ENSP00000368766 | 1.06E-05 | -0.1092 | 0 | 0.605 |
| ENSP00000265395 | 1.41E-05 | -0.1092 | 0 | 0.275 |
| ENSP00000263239 | 1.70E-05 | -0.1092 | 405 | 0.925 |
| ENSP00000380237 | 3.62E-06 | -0.1093 | 0 | 0.000 |
| ENSP00000262605 | 1.38E-05 | -0.1093 | 0 | 0.510 |
| ENSP00000210313 | 1.24E-05 | -0.1093 | 0 | 0.497 |
| ENSP00000221138 | 6.19E-06 | -0.1093 | 224 | 0.688 |
| ENSP00000334229 | 3.05E-05 | -0.1093 | 0 | 0.104 |
| ENSP00000350512 | 3.10E-05 | -0.1093 | 739 | 0.721 |
| ENSP00000318902 | 8.49E-06 | -0.1093 | 159 | 0.000 |
| ENSP00000369150 | 2.01E-06 | -0.1093 | 0 | 0.174 |

| | | | | |
|---|---|---|---|---|
| ENSP00000229812 | 7.41E-06 | -0.1093 | 490 | 0.667 |
| ENSP00000266544 | 1.21E-05 | -0.1093 | 0 | 0.221 |
| ENSP00000239878 | 2.32E-05 | -0.1093 | 0 | 0.227 |
| ENSP00000386146 | 8.18E-08 | -0.1094 | 0 | 0.212 |
| ENSP00000341504 | 1.40E-05 | -0.1094 | 262 | 0.277 |
| ENSP00000351410 | 3.13E-05 | -0.1094 | 901 | 0.666 |
| ENSP00000216274 | 7.34E-06 | -0.1094 | 300 | 0.689 |
| ENSP00000300069 | 1.95E-05 | -0.1094 | 0 | 0.066 |
| ENSP00000226796 | 1.33E-05 | -0.1094 | 418 | 0.923 |
| ENSP00000359340 | 6.06E-06 | -0.1094 | 0 | 0.131 |
| ENSP00000269973 | 4.97E-06 | -0.1094 | 0 | 0.697 |
| ENSP00000330221 | 1.14E-05 | -0.1095 | 0 | 0.076 |
| ENSP00000218343 | 1.33E-05 | -0.1095 | 0 | 0.000 |
| ENSP00000343552 | 1.15E-05 | -0.1095 | 0 | 0.156 |
| ENSP00000306752 | 6.00E-06 | -0.1095 | 0 | 0.627 |
| ENSP00000282059 | 3.26E-05 | -0.1095 | 0 | 0.000 |
| ENSP00000353652 | 5.29E-06 | -0.1095 | 0 | 0.691 |
| ENSP00000262213 | 1.24E-05 | -0.1095 | 0 | 0.446 |
| ENSP00000278198 | 4.03E-06 | -0.1095 | 0 | 0.642 |
| ENSP00000343629 | 1.61E-05 | -0.1095 | 0 | 0.174 |
| ENSP00000321537 | 1.19E-05 | -0.1095 | 0 | 0.533 |
| ENSP00000265495 | 1.87E-05 | -0.1095 | 0 | 0.000 |
| ENSP00000472469 | 1.18E-05 | -0.1095 | 0 | 0.831 |
| ENSP00000260404 | 1.02E-05 | -0.1096 | 151 | 0.671 |
| ENSP00000386870 | 2.51E-05 | -0.1096 | 0 | 0.220 |
| ENSP00000281031 | 1.09E-05 | -0.1096 | 0 | 0.205 |
| ENSP00000253031 | 1.72E-05 | -0.1096 | 0 | 0.101 |
| ENSP00000263063 | 2.94E-05 | -0.1096 | 159 | 0.760 |
| ENSP00000386461 | 1.82E-05 | -0.1096 | 244 | 0.323 |
| ENSP00000448165 | 4.29E-05 | -0.1096 | 536 | 0.767 |
| ENSP00000428657 | 2.71E-05 | -0.1096 | 226 | 0.676 |
| ENSP00000345629 | 7.02E-06 | -0.1097 | 199 | 0.605 |
| ENSP00000381824 | 2.12E-05 | -0.1097 | 165 | 0.393 |
| ENSP00000304994 | 1.24E-05 | -0.1097 | 0 | 0.000 |
| ENSP00000285873 | 1.97E-05 | -0.1097 | 0 | 0.725 |
| ENSP00000344742 | 1.34E-05 | -0.1097 | 198 | 0.522 |
| ENSP00000358374 | 6.60E-06 | -0.1097 | 400 | 0.384 |
| ENSP00000274140 | 3.12E-05 | -0.1097 | 338 | 0.355 |
| ENSP00000298248 | 1.56E-05 | -0.1097 | 0 | 0.226 |
| ENSP00000257181 | 1.53E-05 | -0.1097 | 0 | 0.669 |
| ENSP00000389414 | 2.23E-05 | -0.1097 | 330 | 0.524 |
| ENSP00000216277 | 2.19E-05 | -0.1097 | 192 | 0.892 |
| ENSP00000273064 | 1.50E-05 | -0.1097 | 232 | 0.181 |
| ENSP00000288368 | 3.65E-06 | -0.1097 | 0 | 0.209 |
| ENSP00000263753 | 1.20E-05 | -0.1097 | 0 | 0.357 |
| ENSP00000339090 | 2.31E-05 | -0.1097 | 338 | 0.857 |

| | | | | |
|---|---|---|---|---|
| ENSP00000349508 | 2.40E-05 | -0.1097 | 361 | 0.896 |
| ENSP00000337363 | 3.79E-06 | -0.1097 | 0 | 0.671 |
| ENSP00000351769 | 4.64E-06 | -0.1098 | 0 | 0.000 |
| ENSP00000311521 | 4.99E-06 | -0.1098 | 0 | 0.704 |
| ENSP00000326128 | 1.41E-05 | -0.1098 | 0 | 0.158 |
| ENSP00000344364 | 2.14E-06 | -0.1098 | 0 | 0.000 |
| ENSP00000192788 | 1.69E-05 | -0.1098 | 0 | 0.409 |
| ENSP00000282077 | 4.06E-05 | -0.1098 | 193 | 0.579 |
| ENSP00000299608 | 2.06E-05 | -0.1098 | 355 | 0.477 |
| ENSP00000347836 | 9.60E-06 | -0.1098 | 0 | 0.318 |
| ENSP00000233099 | 1.39E-05 | -0.1098 | 0 | 0.095 |
| ENSP00000311825 | 9.73E-06 | -0.1098 | 296 | 0.777 |
| ENSP00000331879 | 2.42E-05 | -0.1099 | 205 | 0.876 |
| ENSP00000311713 | 2.31E-05 | -0.1099 | 0 | 0.296 |
| ENSP00000355657 | 1.94E-05 | -0.1099 | 702 | 0.952 |
| ENSP00000268184 | 8.16E-06 | -0.1099 | 0 | 0.642 |
| ENSP00000286049 | 6.37E-06 | -0.1099 | 0 | 0.000 |
| ENSP00000238994 | 1.18E-05 | -0.1099 | 0 | 0.306 |
| ENSP00000299927 | 4.20E-06 | -0.1099 | 0 | 0.684 |
| ENSP00000305480 | 7.91E-06 | -0.1099 | 0 | 0.789 |
| ENSP00000265148 | 3.32E-06 | -0.1099 | 0 | 0.382 |
| ENSP00000250351 | 1.88E-05 | -0.1099 | 499 | 0.175 |
| ENSP00000392678 | 3.46E-06 | -0.1099 | 0 | 0.145 |
| ENSP00000344173 | 2.05E-05 | -0.1100 | 344 | 0.675 |
| ENSP00000255858 | 1.19E-05 | -0.1100 | 0 | 0.500 |
| ENSP00000269383 | 1.28E-04 | -0.1100 | 0 | 0.219 |
| ENSP00000327764 | 1.09E-05 | -0.1100 | 0 | 0.000 |
| ENSP00000264668 | 2.92E-05 | -0.1100 | 489 | 0.381 |
| ENSP00000387219 | 1.17E-05 | -0.1100 | 290 | 0.155 |
| ENSP00000368459 | 2.19E-05 | -0.1100 | 196 | 0.529 |
| ENSP00000265073 | 1.79E-05 | -0.1100 | 227 | 0.772 |
| ENSP00000358464 | 8.80E-06 | -0.1100 | 210 | 0.714 |
| ENSP00000260382 | 4.75E-06 | -0.1100 | 0 | 0.421 |
| ENSP00000273359 | 1.42E-05 | -0.1100 | 0 | 0.103 |
| ENSP00000388872 | 1.08E-05 | -0.1100 | 301 | 0.317 |
| ENSP00000268489 | 1.61E-05 | -0.1100 | 0 | 0.708 |
| ENSP00000424058 | 3.07E-08 | -0.1101 | 0 | 0.000 |
| ENSP00000280557 | 1.59E-05 | -0.1101 | 165 | 0.811 |
| ENSP00000321711 | 4.78E-06 | -0.1101 | 0 | 0.764 |
| ENSP00000290341 | 1.47E-05 | -0.1101 | 163 | 0.722 |
| ENSP00000299667 | 4.73E-06 | -0.1101 | 0 | 0.695 |
| ENSP00000278935 | 9.65E-06 | -0.1101 | 0 | 0.127 |
| ENSP00000314806 | 3.41E-06 | -0.1101 | 0 | 0.000 |
| ENSP00000295087 | 2.35E-05 | -0.1101 | 274 | 0.481 |
| ENSP00000221855 | 1.47E-05 | -0.1101 | 0 | 0.160 |
| ENSP00000341947 | 1.29E-05 | -0.1101 | 0 | 0.721 |

| | | | | |
|---|---|---|---|---|
| ENSP00000454153 | 1.26E-06 | -0.1101 | 0 | 0.180 |
| ENSP00000358714 | 1.67E-05 | -0.1101 | 173 | 0.076 |
| ENSP00000399938 | 3.62E-06 | -0.1101 | 0 | 0.140 |
| ENSP00000301730 | 2.10E-05 | -0.1101 | 214 | 0.000 |
| ENSP00000361949 | 1.93E-05 | -0.1101 | 516 | 0.933 |
| ENSP00000363055 | 1.44E-05 | -0.1101 | 589 | 0.300 |
| ENSP00000287025 | 3.84E-06 | -0.1102 | 0 | 0.000 |
| ENSP00000355185 | 2.23E-05 | -0.1102 | 153 | 0.635 |
| ENSP00000299084 | 9.20E-05 | -0.1102 | 267 | 0.771 |
| ENSP00000283179 | 1.82E-05 | -0.1102 | 264 | 0.777 |
| ENSP00000347329 | 5.82E-06 | -0.1102 | 153 | 0.212 |
| ENSP00000262366 | 3.64E-06 | -0.1102 | 0 | 0.778 |
| ENSP00000250454 | 3.89E-05 | -0.1102 | 0 | 0.192 |
| ENSP00000345029 | 7.52E-06 | -0.1102 | 0 | 0.184 |
| ENSP00000377381 | 4.49E-05 | -0.1102 | 0 | 0.192 |
| ENSP00000343701 | 2.44E-05 | -0.1102 | 207 | 0.704 |
| ENSP00000316543 | 1.64E-05 | -0.1103 | 0 | 0.179 |
| ENSP00000363226 | 1.39E-06 | -0.1103 | 0 | 0.171 |
| ENSP00000467889 | 3.37E-06 | -0.1103 | 0 | 0.711 |
| ENSP00000310126 | 5.53E-06 | -0.1103 | 0 | 0.650 |
| ENSP00000333657 | 1.87E-05 | -0.1103 | 293 | 0.500 |
| ENSP00000278816 | 4.82E-06 | -0.1103 | 0 | 0.000 |
| ENSP00000365014 | 5.91E-06 | -0.1103 | 0 | 0.101 |
| ENSP00000256682 | 1.76E-05 | -0.1103 | 274 | 0.533 |
| ENSP00000357861 | 1.59E-05 | -0.1103 | 191 | 0.214 |
| ENSP00000316905 | 1.84E-05 | -0.1103 | 0 | 0.088 |
| ENSP00000263915 | 6.73E-06 | -0.1103 | 0 | 0.691 |
| ENSP00000391879 | 2.90E-05 | -0.1103 | 207 | 0.322 |
| ENSP00000323670 | 9.64E-06 | -0.1104 | 330 | 0.792 |
| ENSP00000277570 | 1.00E-05 | -0.1104 | 0 | 0.157 |
| ENSP00000264710 | 1.45E-05 | -0.1104 | 177 | 0.240 |
| ENSP00000233944 | 6.42E-06 | -0.1104 | 256 | 0.682 |
| ENSP00000325425 | 1.91E-05 | -0.1104 | 0 | 0.182 |
| ENSP00000373825 | 3.98E-06 | -0.1104 | 0 | 0.122 |
| ENSP00000299001 | 9.18E-06 | -0.1104 | 0 | 0.666 |
| ENSP00000317224 | 1.31E-05 | -0.1104 | 0 | 0.253 |
| ENSP00000296847 | 1.76E-05 | -0.1104 | 0 | 0.000 |
| ENSP00000370984 | 2.49E-05 | -0.1104 | 0 | 0.099 |
| ENSP00000355205 | 1.99E-05 | -0.1104 | 449 | 0.951 |
| ENSP00000328524 | 1.62E-05 | -0.1105 | 0 | 0.082 |
| ENSP00000357409 | 1.22E-05 | -0.1105 | 0 | 0.178 |
| ENSP00000267569 | 8.31E-06 | -0.1105 | 265 | 0.000 |
| ENSP00000328789 | 1.06E-05 | -0.1105 | 0 | 0.221 |
| ENSP00000261464 | 2.72E-05 | -0.1105 | 0 | 0.770 |
| ENSP00000414034 | 1.49E-06 | -0.1105 | 0 | 0.194 |
| ENSP00000314837 | 1.10E-05 | -0.1105 | 316 | 0.604 |

| | | | | |
|---|---|---|---|---|
| ENSP00000053468 | 1.03E-05 | -0.1105 | 355 | 0.743 |
| ENSP00000301744 | 4.92E-06 | -0.1105 | 0 | 0.676 |
| ENSP00000356918 | 2.56E-05 | -0.1105 | 196 | 0.219 |
| ENSP00000270617 | 3.78E-06 | -0.1105 | 0 | 0.662 |
| ENSP00000365107 | 3.50E-06 | -0.1105 | 0 | 0.238 |
| ENSP00000285420 | 1.13E-05 | -0.1105 | 0 | 0.255 |
| ENSP00000358391 | 3.34E-06 | -0.1105 | 0 | 0.141 |
| ENSP00000428281 | 4.00E-06 | -0.1105 | 0 | 0.074 |
| ENSP00000303696 | 4.36E-06 | -0.1106 | 0 | 0.694 |
| ENSP00000296417 | 1.51E-05 | -0.1106 | 422 | 0.912 |
| ENSP00000386076 | 2.60E-06 | -0.1106 | 0 | 0.135 |
| ENSP00000216639 | 6.02E-06 | -0.1106 | 175 | 0.000 |
| ENSP00000323678 | 1.22E-05 | -0.1106 | 0 | 0.089 |
| ENSP00000413728 | 5.12E-06 | -0.1106 | 0 | 0.139 |
| ENSP00000430338 | 3.34E-06 | -0.1106 | 0 | 0.158 |
| ENSP00000263934 | 1.40E-05 | -0.1106 | 358 | 0.375 |
| ENSP00000341938 | 3.57E-05 | -0.1106 | 721 | 0.592 |
| ENSP00000344488 | 1.50E-05 | -0.1106 | 0 | 0.117 |
| ENSP00000344331 | 5.43E-06 | -0.1106 | 0 | 0.184 |
| ENSP00000341483 | 1.79E-05 | -0.1107 | 0 | 0.281 |
| ENSP00000290649 | 2.38E-05 | -0.1107 | 820 | 0.551 |
| ENSP00000309117 | 2.28E-05 | -0.1107 | 404 | 0.405 |
| ENSP00000325369 | 1.24E-05 | -0.1107 | 0 | 0.115 |
| ENSP00000328070 | 6.15E-06 | -0.1107 | 0 | 0.122 |
| ENSP00000395244 | 1.88E-07 | -0.1108 | 0 | 0.119 |
| ENSP00000263736 | 1.55E-05 | -0.1108 | 178 | 0.754 |
| ENSP00000269349 | 1.84E-05 | -0.1108 | 679 | 0.000 |
| ENSP00000321674 | 6.23E-06 | -0.1108 | 0 | 0.454 |
| ENSP00000293362 | 2.09E-05 | -0.1108 | 224 | 0.542 |
| ENSP00000369075 | 3.17E-06 | -0.1108 | 0 | 0.691 |
| ENSP00000349437 | 2.44E-05 | -0.1108 | 271 | 0.681 |
| ENSP00000310015 | 2.57E-05 | -0.1109 | 0 | 0.273 |
| ENSP00000329140 | 1.64E-05 | -0.1109 | 284 | 0.660 |
| ENSP00000301459 | 5.93E-06 | -0.1109 | 0 | 0.759 |
| ENSP00000344166 | 1.20E-05 | -0.1109 | 0 | 0.537 |
| ENSP00000391218 | 1.55E-06 | -0.1109 | 0 | 0.192 |
| ENSP00000245255 | 8.42E-06 | -0.1110 | 0 | 0.668 |
| ENSP00000261842 | 9.86E-06 | -0.1110 | 0 | 0.197 |
| ENSP00000469046 | 2.49E-07 | -0.1110 | 0 | 0.000 |
| ENSP00000300413 | 2.44E-05 | -0.1110 | 613 | 0.851 |
| ENSP00000262519 | 9.71E-06 | -0.1110 | 244 | 0.910 |
| ENSP00000268607 | 2.86E-05 | -0.1110 | 576 | 0.628 |
| ENSP00000325634 | 4.02E-06 | -0.1110 | 0 | 0.695 |
| ENSP00000291759 | 3.07E-05 | -0.1111 | 0 | 0.213 |
| ENSP00000470409 | 2.99E-07 | -0.1111 | 0 | 0.000 |
| ENSP00000320488 | 1.09E-05 | -0.1111 | 326 | 0.000 |

| | | | | |
|---|---|---|---|---|
| ENSP00000267064 | 1.53E-05 | -0.1111 | 161 | 0.875 |
| ENSP00000345555 | 1.85E-05 | -0.1111 | 460 | 0.690 |
| ENSP00000322716 | 1.29E-05 | -0.1111 | 0 | 0.183 |
| ENSP00000311747 | 1.48E-05 | -0.1111 | 0 | 0.781 |
| ENSP00000350263 | 4.96E-06 | -0.1111 | 0 | 0.000 |
| ENSP00000364502 | 1.13E-05 | -0.1111 | 0 | 0.127 |
| ENSP00000380718 | 6.87E-06 | -0.1112 | 163 | 0.000 |
| ENSP00000285518 | 9.54E-06 | -0.1112 | 0 | 0.281 |
| ENSP00000386655 | 2.83E-06 | -0.1112 | 0 | 0.000 |
| ENSP00000389709 | 4.24E-06 | -0.1112 | 0 | 0.104 |
| ENSP00000354686 | 3.80E-06 | -0.1112 | 0 | 0.689 |
| ENSP00000286175 | 1.45E-05 | -0.1112 | 244 | 0.720 |
| ENSP00000368552 | 2.13E-05 | -0.1112 | 195 | 0.874 |
| ENSP00000337736 | 1.09E-05 | -0.1112 | 0 | 0.393 |
| ENSP00000333223 | 4.12E-06 | -0.1112 | 0 | 0.686 |
| ENSP00000307411 | 9.52E-06 | -0.1112 | 0 | 0.757 |
| ENSP00000381992 | 6.32E-06 | -0.1112 | 0 | 0.457 |
| ENSP00000295797 | 9.68E-06 | -0.1112 | 0 | 0.737 |
| ENSP00000366519 | 1.51E-05 | -0.1112 | 325 | 0.882 |
| ENSP00000273179 | 1.09E-05 | -0.1113 | 0 | 0.340 |
| ENSP00000378503 | 3.98E-06 | -0.1113 | 0 | 0.669 |
| ENSP00000383199 | 2.63E-05 | -0.1113 | 202 | 0.703 |
| ENSP00000263620 | 1.10E-05 | -0.1113 | 0 | 0.830 |
| ENSP00000283943 | 9.94E-06 | -0.1113 | 172 | 0.568 |
| ENSP00000309823 | 3.69E-06 | -0.1113 | 0 | 0.000 |
| ENSP00000379931 | 2.40E-05 | -0.1113 | 296 | 0.645 |
| ENSP00000343741 | 2.03E-05 | -0.1113 | 877 | 0.768 |
| ENSP00000283033 | 6.75E-06 | -0.1113 | 569 | 0.538 |
| ENSP00000302310 | 4.84E-06 | -0.1114 | 0 | 0.691 |
| ENSP00000264932 | 7.64E-06 | -0.1114 | 377 | 0.643 |
| ENSP00000301180 | 1.18E-05 | -0.1114 | 0 | 0.209 |
| ENSP00000381240 | 2.13E-05 | -0.1114 | 303 | 0.000 |
| ENSP00000273146 | 2.98E-05 | -0.1114 | 0 | 0.159 |
| ENSP00000342019 | 3.93E-06 | -0.1114 | 0 | 0.694 |
| ENSP00000439493 | 2.01E-05 | -0.1114 | 734 | 0.000 |
| ENSP00000298767 | 1.64E-05 | -0.1114 | 345 | 0.000 |
| ENSP00000339521 | 2.56E-05 | -0.1114 | 477 | 0.558 |
| ENSP00000339256 | 4.39E-06 | -0.1114 | 0 | 0.104 |
| ENSP00000336868 | 1.08E-05 | -0.1114 | 729 | 0.947 |
| ENSP00000324064 | 4.20E-06 | -0.1114 | 0 | 0.714 |
| ENSP00000298068 | 1.41E-05 | -0.1115 | 192 | 0.426 |
| ENSP00000358814 | 1.37E-05 | -0.1115 | 685 | 0.352 |
| ENSP00000357791 | 6.54E-06 | -0.1115 | 0 | 0.109 |
| ENSP00000344401 | 9.53E-06 | -0.1115 | 302 | 0.698 |
| ENSP00000362592 | 2.68E-05 | -0.1115 | 200 | 0.923 |
| ENSP00000346120 | 2.17E-05 | -0.1115 | 358 | 0.913 |

| | | | | |
|---|---|---|---|---|
| ENSP00000314491 | 1.68E-05 | -0.1115 | 206 | 0.000 |
| ENSP00000422344 | 4.16E-06 | -0.1115 | 0 | 0.705 |
| ENSP00000308759 | 4.34E-06 | -0.1116 | 0 | 0.679 |
| ENSP00000277865 | 1.35E-05 | -0.1116 | 193 | 0.581 |
| ENSP00000279873 | 1.80E-05 | -0.1116 | 0 | 0.547 |
| ENSP00000321997 | 8.36E-06 | -0.1116 | 0 | 0.917 |
| ENSP00000417354 | 6.64E-06 | -0.1116 | 0 | 0.115 |
| ENSP00000361577 | 1.95E-05 | -0.1116 | 393 | 0.792 |
| ENSP00000308405 | 3.18E-05 | -0.1116 | 422 | 0.927 |
| ENSP00000306010 | 2.52E-05 | -0.1116 | 470 | 0.556 |
| ENSP00000472384 | 1.87E-06 | -0.1116 | 0 | 0.487 |
| ENSP00000366637 | 3.02E-06 | -0.1116 | 0 | 0.000 |
| ENSP00000321507 | 1.47E-05 | -0.1116 | 320 | 0.513 |
| ENSP00000037243 | 1.63E-05 | -0.1116 | 326 | 0.483 |
| ENSP00000340944 | 2.95E-05 | -0.1117 | 309 | 0.940 |
| ENSP00000370420 | 3.98E-06 | -0.1117 | 0 | 0.178 |
| ENSP00000260947 | 1.66E-05 | -0.1117 | 228 | 0.750 |
| ENSP00000381599 | 3.58E-05 | -0.1117 | 293 | 0.829 |
| ENSP00000354032 | 2.13E-06 | -0.1117 | 0 | 0.196 |
| ENSP00000470752 | 2.83E-06 | -0.1118 | 0 | 0.000 |
| ENSP00000384179 | 1.14E-05 | -0.1118 | 197 | 0.846 |
| ENSP00000230085 | 1.10E-05 | -0.1118 | 0 | 0.153 |
| ENSP00000261353 | 8.04E-06 | -0.1118 | 0 | 0.123 |
| ENSP00000367406 | 6.40E-06 | -0.1118 | 200 | 0.000 |
| ENSP00000344470 | 8.86E-06 | -0.1118 | 508 | 0.588 |
| ENSP00000332706 | 2.41E-05 | -0.1118 | 153 | 0.622 |
| ENSP00000332576 | 1.84E-05 | -0.1118 | 576 | 0.105 |
| ENSP00000304802 | 4.29E-06 | -0.1118 | 0 | 0.533 |
| ENSP00000381304 | 5.94E-06 | -0.1118 | 0 | 0.710 |
| ENSP00000370526 | 2.74E-05 | -0.1118 | 361 | 0.524 |
| ENSP00000362122 | 1.71E-05 | -0.1118 | 301 | 0.494 |
| ENSP00000258886 | 1.10E-05 | -0.1118 | 300 | 0.657 |
| ENSP00000321951 | 2.12E-05 | -0.1118 | 201 | 0.116 |
| ENSP00000375717 | 1.32E-05 | -0.1119 | 414 | 0.175 |
| ENSP00000326200 | 5.74E-06 | -0.1119 | 0 | 0.675 |
| ENSP00000365663 | 1.97E-05 | -0.1119 | 177 | 0.720 |
| ENSP00000346694 | 2.02E-05 | -0.1119 | 539 | 0.946 |
| ENSP00000362870 | 1.47E-05 | -0.1119 | 280 | 0.104 |
| ENSP00000326249 | 4.45E-06 | -0.1119 | 0 | 0.689 |
| ENSP00000297767 | 7.64E-06 | -0.1119 | 0 | 0.110 |
| ENSP00000337122 | 4.86E-06 | -0.1119 | 0 | 0.689 |
| ENSP00000352704 | 1.38E-05 | -0.1120 | 0 | 0.000 |
| ENSP00000361264 | 2.30E-05 | -0.1120 | 196 | 0.806 |
| ENSP00000283875 | 7.80E-06 | -0.1120 | 0 | 0.791 |
| ENSP00000326261 | 2.09E-05 | -0.1120 | 183 | 0.740 |
| ENSP00000348769 | 5.34E-06 | -0.1120 | 0 | 0.333 |

| | | | | |
|---|---|---|---|---|
| ENSP00000337852 | 4.94E-06 | -0.1121 | 0 | 0.692 |
| ENSP00000340879 | 8.85E-06 | -0.1121 | 0 | 0.593 |
| ENSP00000309161 | 4.64E-06 | -0.1121 | 0 | 0.704 |
| ENSP00000263360 | 1.51E-05 | -0.1121 | 345 | 0.802 |
| ENSP00000412150 | 1.38E-07 | -0.1121 | 0 | 0.143 |
| ENSP00000359793 | 2.11E-05 | -0.1121 | 463 | 0.520 |
| ENSP00000317902 | 1.23E-05 | -0.1121 | 0 | 0.120 |
| ENSP00000215115 | 4.79E-06 | -0.1121 | 0 | 0.331 |
| ENSP00000351207 | 2.11E-05 | -0.1121 | 0 | 0.129 |
| ENSP00000229390 | 1.50E-05 | -0.1121 | 234 | 0.943 |
| ENSP00000342002 | 4.46E-06 | -0.1122 | 0 | 0.680 |
| ENSP00000297540 | 1.27E-05 | -0.1122 | 0 | 0.673 |
| ENSP00000354869 | 7.36E-06 | -0.1122 | 0 | 0.000 |
| ENSP00000347507 | 2.07E-05 | -0.1122 | 434 | 0.612 |
| ENSP00000397163 | 2.98E-06 | -0.1122 | 0 | 0.688 |
| ENSP00000352671 | 5.73E-06 | -0.1122 | 0 | 0.257 |
| ENSP00000366814 | 3.23E-06 | -0.1122 | 0 | 0.000 |
| ENSP00000309555 | 2.27E-05 | -0.1122 | 207 | 0.833 |
| ENSP00000262803 | 1.25E-05 | -0.1122 | 273 | 0.907 |
| ENSP00000433721 | 1.09E-05 | -0.1122 | 0 | 0.086 |
| ENSP00000316121 | 1.13E-05 | -0.1122 | 0 | 0.386 |
| ENSP00000250863 | 1.12E-05 | -0.1122 | 152 | 0.910 |
| ENSP00000274711 | 4.86E-06 | -0.1122 | 0 | 0.611 |
| ENSP00000354061 | 3.05E-06 | -0.1122 | 0 | 0.101 |
| ENSP00000326579 | 1.58E-05 | -0.1122 | 0 | 0.131 |
| ENSP00000323046 | 1.65E-05 | -0.1123 | 153 | 0.848 |
| ENSP00000336702 | 2.17E-05 | -0.1123 | 274 | 0.000 |
| ENSP00000362768 | 2.41E-05 | -0.1123 | 216 | 0.814 |
| ENSP00000366630 | 3.23E-06 | -0.1123 | 0 | 0.000 |
| ENSP00000325136 | 1.84E-05 | -0.1123 | 803 | 0.480 |
| ENSP00000470209 | 2.33E-06 | -0.1123 | 0 | 0.694 |
| ENSP00000457715 | 2.26E-06 | -0.1123 | 0 | 0.693 |
| ENSP00000418767 | 6.02E-06 | -0.1123 | 0 | 0.137 |
| ENSP00000385491 | 6.13E-06 | -0.1123 | 0 | 0.000 |
| ENSP00000325203 | 6.92E-06 | -0.1124 | 0 | 0.000 |
| ENSP00000304858 | 1.05E-05 | -0.1124 | 0 | 0.308 |
| ENSP00000420213 | 2.83E-05 | -0.1124 | 382 | 0.608 |
| ENSP00000278568 | 1.23E-05 | -0.1124 | 177 | 0.774 |
| ENSP00000250448 | 1.51E-05 | -0.1124 | 314 | 0.000 |
| ENSP00000343535 | 1.14E-05 | -0.1124 | 206 | 0.703 |
| ENSP00000371512 | 8.24E-06 | -0.1124 | 0 | 0.101 |
| ENSP00000242827 | 6.01E-06 | -0.1124 | 0 | 0.083 |
| ENSP00000254480 | 1.25E-05 | -0.1124 | 161 | 0.866 |
| ENSP00000232978 | 1.15E-05 | -0.1124 | 524 | 0.709 |
| ENSP00000296702 | 4.37E-05 | -0.1125 | 667 | 0.719 |
| ENSP00000400932 | 3.21E-06 | -0.1125 | 0 | 0.069 |

| | | | | |
|---|---|---|---|---|
| ENSP00000388606 | 1.60E-06 | -0.1125 | 0 | 0.000 |
| ENSP00000262971 | 2.18E-05 | -0.1125 | 150 | 0.824 |
| ENSP00000396918 | 1.40E-05 | -0.1125 | 644 | 0.161 |
| ENSP00000364798 | 5.92E-06 | -0.1125 | 0 | 0.732 |
| ENSP00000276123 | 3.35E-06 | -0.1125 | 0 | 0.668 |
| ENSP00000364295 | 4.96E-06 | -0.1125 | 0 | 0.116 |
| ENSP00000341247 | 1.53E-05 | -0.1125 | 440 | 0.860 |
| ENSP00000415106 | 2.83E-06 | -0.1125 | 0 | 0.208 |
| ENSP00000265062 | 1.67E-05 | -0.1125 | 722 | 0.312 |
| ENSP00000253039 | 1.28E-05 | -0.1125 | 249 | 0.808 |
| ENSP00000329170 | 1.32E-05 | -0.1125 | 0 | 0.866 |
| ENSP00000264018 | 1.31E-05 | -0.1126 | 287 | 0.000 |
| ENSP00000272930 | 2.24E-05 | -0.1126 | 0 | 0.380 |
| ENSP00000315410 | 1.31E-05 | -0.1126 | 0 | 0.089 |
| ENSP00000329466 | 1.48E-05 | -0.1126 | 313 | 0.820 |
| ENSP00000308944 | 1.80E-05 | -0.1126 | 160 | 0.926 |
| ENSP00000359206 | 1.95E-05 | -0.1126 | 574 | 0.851 |
| ENSP00000352157 | 2.61E-05 | -0.1126 | 665 | 0.921 |
| ENSP00000395449 | 1.91E-05 | -0.1127 | 150 | 0.756 |
| ENSP00000308430 | 8.13E-06 | -0.1127 | 0 | 0.298 |
| ENSP00000411097 | 3.47E-06 | -0.1127 | 0 | 0.000 |
| ENSP00000332595 | 4.16E-06 | -0.1127 | 0 | 0.684 |
| ENSP00000447650 | 2.67E-06 | -0.1127 | 0 | 0.000 |
| ENSP00000375582 | 3.25E-06 | -0.1127 | 0 | 0.682 |
| ENSP00000341274 | 7.84E-08 | -0.1127 | 0 | 0.000 |
| ENSP00000369581 | 2.52E-05 | -0.1127 | 815 | 0.875 |
| ENSP00000315370 | 4.84E-06 | -0.1128 | 0 | 0.135 |
| ENSP00000366331 | 3.37E-07 | -0.1128 | 0 | 0.192 |
| ENSP00000407154 | 5.42E-06 | -0.1128 | 0 | 0.238 |
| ENSP00000256257 | 1.45E-05 | -0.1128 | 0 | 0.412 |
| ENSP00000399324 | 1.63E-05 | -0.1128 | 170 | 0.881 |
| ENSP00000310551 | 9.19E-06 | -0.1128 | 184 | 0.289 |
| ENSP00000264893 | 7.01E-06 | -0.1128 | 0 | 0.310 |
| ENSP00000432472 | 1.00E-05 | -0.1129 | 215 | 0.480 |
| ENSP00000215570 | 7.79E-06 | -0.1129 | 0 | 0.617 |
| ENSP00000369373 | 5.99E-06 | -0.1129 | 0 | 0.438 |
| ENSP00000382021 | 8.18E-06 | -0.1129 | 0 | 0.130 |
| ENSP00000406162 | 3.43E-05 | -0.1129 | 205 | 0.833 |
| ENSP00000369497 | 5.63E-05 | -0.1129 | 254 | 0.835 |
| ENSP00000378601 | 2.42E-06 | -0.1129 | 0 | 0.000 |
| ENSP00000389128 | 1.13E-05 | -0.1130 | 580 | 0.000 |
| ENSP00000313454 | 3.04E-05 | -0.1130 | 266 | 0.545 |
| ENSP00000357336 | 2.30E-05 | -0.1130 | 217 | 0.273 |
| ENSP00000330141 | 8.20E-06 | -0.1130 | 0 | 0.063 |
| ENSP00000262300 | 3.37E-06 | -0.1130 | 0 | 0.637 |
| ENSP00000238561 | 4.01E-06 | -0.1130 | 0 | 0.069 |

| | | | | |
|---|---|---|---|---|
| ENSP00000363377 | 2.14E-05 | -0.1130 | 905 | 0.000 |
| ENSP00000000233 | 1.29E-05 | -0.1131 | 274 | 0.532 |
| ENSP00000299163 | 1.36E-05 | -0.1131 | 0 | 0.529 |
| ENSP00000264711 | 6.19E-06 | -0.1131 | 204 | 0.527 |
| ENSP00000418081 | 1.59E-05 | -0.1131 | 202 | 0.259 |
| ENSP00000362036 | 6.54E-06 | -0.1131 | 315 | 0.590 |
| ENSP00000298875 | 7.91E-06 | -0.1132 | 0 | 0.836 |
| ENSP00000307863 | 2.03E-05 | -0.1132 | 464 | 0.864 |
| ENSP00000436455 | 1.61E-05 | -0.1132 | 574 | 0.194 |
| ENSP00000365402 | 4.59E-05 | -0.1132 | 902 | 0.704 |
| ENSP00000362778 | 1.75E-05 | -0.1132 | 158 | 0.134 |
| ENSP00000361878 | 2.45E-05 | -0.1132 | 525 | 0.667 |
| ENSP00000295317 | 1.24E-05 | -0.1132 | 0 | 0.269 |
| ENSP00000244040 | 9.23E-06 | -0.1132 | 0 | 0.212 |
| ENSP00000388942 | 2.26E-05 | -0.1132 | 196 | 0.069 |
| ENSP00000220913 | 3.72E-06 | -0.1133 | 0 | 0.000 |
| ENSP00000295488 | 1.82E-05 | -0.1133 | 325 | 0.800 |
| ENSP00000378350 | 7.12E-06 | -0.1133 | 0 | 0.454 |
| ENSP00000316649 | 1.29E-05 | -0.1133 | 0 | 0.091 |
| ENSP00000265100 | 1.16E-05 | -0.1133 | 297 | 0.834 |
| ENSP00000357651 | 2.27E-05 | -0.1133 | 465 | 0.563 |
| ENSP00000292055 | 4.46E-06 | -0.1133 | 0 | 0.000 |
| ENSP00000347979 | 2.88E-05 | -0.1133 | 292 | 0.917 |
| ENSP00000395706 | 3.25E-06 | -0.1133 | 0 | 0.085 |
| ENSP00000242839 | 2.16E-05 | -0.1133 | 809 | 0.586 |
| ENSP00000309103 | 1.23E-05 | -0.1134 | 0 | 0.536 |
| ENSP00000263212 | 5.74E-06 | -0.1134 | 0 | 0.601 |
| ENSP00000354663 | 3.39E-06 | -0.1134 | 0 | 0.689 |
| ENSP00000327968 | 4.96E-06 | -0.1134 | 0 | 0.817 |
| ENSP00000368927 | 5.27E-06 | -0.1134 | 0 | 0.822 |
| ENSP00000343313 | 1.11E-05 | -0.1134 | 554 | 0.588 |
| ENSP00000315476 | 8.32E-06 | -0.1134 | 163 | 0.825 |
| ENSP00000362014 | 3.59E-05 | -0.1135 | 300 | 0.662 |
| ENSP00000467556 | 3.89E-06 | -0.1135 | 0 | 0.268 |
| ENSP00000312999 | 1.65E-05 | -0.1135 | 508 | 0.647 |
| ENSP00000346809 | 4.74E-06 | -0.1135 | 0 | 0.114 |
| ENSP00000365773 | 1.68E-05 | -0.1135 | 186 | 0.264 |
| ENSP00000253144 | 4.31E-06 | -0.1135 | 0 | 0.673 |
| ENSP00000221233 | 6.11E-06 | -0.1135 | 0 | 0.858 |
| ENSP00000317980 | 2.15E-05 | -0.1135 | 222 | 0.371 |
| ENSP00000272645 | 9.37E-06 | -0.1135 | 0 | 0.733 |
| ENSP00000364976 | 1.10E-05 | -0.1136 | 169 | 0.585 |
| ENSP00000309198 | 8.91E-06 | -0.1136 | 0 | 0.347 |
| ENSP00000336740 | 1.40E-05 | -0.1136 | 273 | 0.675 |
| ENSP00000469432 | 2.86E-06 | -0.1136 | 0 | 0.000 |
| ENSP00000342411 | 1.60E-05 | -0.1136 | 0 | 0.152 |

| | | | | |
|---|---|---|---|---|
| ENSP00000464100 | 1.34E-06 | -0.1136 | 0 | 0.000 |
| ENSP00000009105 | 2.55E-06 | -0.1136 | 0 | 0.658 |
| ENSP00000419599 | 1.41E-05 | -0.1136 | 197 | 0.115 |
| ENSP00000354453 | 3.62E-06 | -0.1136 | 0 | 0.701 |
| ENSP00000368976 | 4.69E-06 | -0.1136 | 0 | 0.270 |
| ENSP00000382840 | 1.63E-05 | -0.1137 | 396 | 0.905 |
| ENSP00000443194 | 1.11E-05 | -0.1137 | 159 | 0.315 |
| ENSP00000432412 | 3.26E-06 | -0.1137 | 0 | 0.138 |
| ENSP00000259750 | 7.38E-06 | -0.1137 | 175 | 0.688 |
| ENSP00000305065 | 1.08E-05 | -0.1137 | 0 | 0.189 |
| ENSP00000325690 | 2.62E-05 | -0.1137 | 900 | 0.794 |
| ENSP00000296513 | 5.25E-06 | -0.1137 | 0 | 0.145 |
| ENSP00000358718 | 2.34E-07 | -0.1137 | 0 | 0.000 |
| ENSP00000293831 | 1.60E-05 | -0.1137 | 967 | 0.000 |
| ENSP00000338283 | 2.45E-05 | -0.1138 | 206 | 0.769 |
| ENSP00000359025 | 2.29E-05 | -0.1138 | 204 | 0.252 |
| ENSP00000351255 | 2.84E-05 | -0.1138 | 225 | 0.929 |
| ENSP00000378286 | 5.56E-06 | -0.1138 | 0 | 0.709 |
| ENSP00000267890 | 7.32E-06 | -0.1139 | 175 | 0.686 |
| ENSP00000469964 | 1.34E-06 | -0.1139 | 0 | 0.000 |
| ENSP00000341138 | 2.85E-05 | -0.1139 | 232 | 0.277 |
| ENSP00000297324 | 1.80E-05 | -0.1139 | 0 | 0.180 |
| ENSP00000334375 | 8.31E-06 | -0.1139 | 0 | 0.559 |
| ENSP00000342087 | 1.74E-05 | -0.1139 | 301 | 0.740 |
| ENSP00000274695 | 1.19E-05 | -0.1139 | 378 | 0.494 |
| ENSP00000319501 | 1.08E-05 | -0.1139 | 0 | 0.330 |
| ENSP00000369777 | 5.51E-06 | -0.1139 | 0 | 0.683 |
| ENSP00000461425 | 2.70E-06 | -0.1140 | 0 | 0.693 |
| ENSP00000315955 | 1.98E-05 | -0.1140 | 212 | 0.000 |
| ENSP00000397552 | 3.54E-05 | -0.1140 | 316 | 0.750 |
| ENSP00000342780 | 2.42E-06 | -0.1140 | 0 | 0.000 |
| ENSP00000361707 | 9.84E-06 | -0.1140 | 158 | 0.145 |
| ENSP00000401371 | 1.83E-05 | -0.1141 | 560 | 0.801 |
| ENSP00000263559 | 8.51E-06 | -0.1141 | 0 | 0.136 |
| ENSP00000229243 | 3.95E-06 | -0.1141 | 0 | 0.076 |
| ENSP00000326424 | 5.44E-06 | -0.1141 | 0 | 0.195 |
| ENSP00000269844 | 3.62E-06 | -0.1141 | 0 | 0.666 |
| ENSP00000324175 | 1.19E-05 | -0.1141 | 0 | 0.126 |
| ENSP00000264071 | 1.31E-05 | -0.1141 | 639 | 0.698 |
| ENSP00000380602 | 7.67E-06 | -0.1142 | 0 | 0.203 |
| ENSP00000365494 | 1.16E-05 | -0.1142 | 270 | 0.540 |
| ENSP00000344087 | 8.33E-06 | -0.1142 | 0 | 0.145 |
| ENSP00000315569 | 4.88E-06 | -0.1142 | 0 | 0.731 |
| ENSP00000267339 | 8.40E-06 | -0.1142 | 202 | 0.611 |
| ENSP00000293842 | 1.16E-05 | -0.1142 | 312 | 0.000 |
| ENSP00000247986 | 8.85E-06 | -0.1142 | 300 | 0.292 |

| | | | | |
|---|---|---|---|---|
| ENSP00000400878 | 9.61E-06 | -0.1143 | 0 | 0.688 |
| ENSP00000355402 | 4.07E-06 | -0.1144 | 0 | 0.636 |
| ENSP00000334394 | 4.33E-06 | -0.1144 | 0 | 0.664 |
| ENSP00000325402 | 2.48E-07 | -0.1144 | 0 | 0.196 |
| ENSP00000415634 | 2.75E-05 | -0.1144 | 189 | 0.000 |
| ENSP00000391116 | 1.89E-06 | -0.1144 | 0 | 0.000 |
| ENSP00000362556 | 5.78E-06 | -0.1144 | 0 | 0.696 |
| ENSP00000345968 | 1.82E-05 | -0.1144 | 480 | 0.157 |
| ENSP00000330631 | 4.82E-06 | -0.1144 | 0 | 0.694 |
| ENSP00000262189 | 1.62E-05 | -0.1144 | 244 | 0.000 |
| ENSP00000378720 | 2.09E-05 | -0.1145 | 199 | 0.099 |
| ENSP00000353491 | 4.78E-06 | -0.1145 | 0 | 0.695 |
| ENSP00000332674 | 6.02E-06 | -0.1145 | 0 | 0.610 |
| ENSP00000363018 | 6.06E-06 | -0.1145 | 419 | 0.817 |
| ENSP00000341874 | 9.12E-06 | -0.1145 | 0 | 0.791 |
| ENSP00000354952 | 2.09E-05 | -0.1146 | 877 | 0.842 |
| ENSP00000420182 | 5.19E-06 | -0.1146 | 0 | 0.098 |
| ENSP00000336741 | 1.70E-05 | -0.1146 | 357 | 0.945 |
| ENSP00000370968 | 5.16E-06 | -0.1146 | 0 | 0.823 |
| ENSP00000256460 | 2.44E-06 | -0.1147 | 0 | 0.000 |
| ENSP00000274031 | 1.23E-05 | -0.1147 | 244 | 0.873 |
| ENSP00000300738 | 1.69E-05 | -0.1147 | 316 | 0.676 |
| ENSP00000384370 | 4.53E-06 | -0.1147 | 0 | 0.180 |
| ENSP00000325546 | 4.89E-06 | -0.1147 | 0 | 0.762 |
| ENSP00000267807 | 3.57E-06 | -0.1147 | 0 | 0.724 |
| ENSP00000386143 | 9.01E-07 | -0.1147 | 0 | 0.088 |
| ENSP00000319141 | 7.97E-06 | -0.1147 | 0 | 0.202 |
| ENSP00000301042 | 4.83E-06 | -0.1147 | 0 | 0.687 |
| ENSP00000229922 | 1.74E-05 | -0.1148 | 525 | 0.647 |
| ENSP00000333024 | 1.39E-05 | -0.1148 | 244 | 0.913 |
| ENSP00000381132 | 2.55E-06 | -0.1148 | 0 | 0.170 |
| ENSP00000226004 | 5.99E-06 | -0.1148 | 0 | 0.671 |
| ENSP00000440832 | 2.04E-06 | -0.1148 | 0 | 0.170 |
| ENSP00000342974 | 4.81E-06 | -0.1148 | 0 | 0.696 |
| ENSP00000247207 | 1.06E-05 | -0.1149 | 204 | 0.836 |
| ENSP00000367705 | 3.87E-06 | -0.1149 | 0 | 0.734 |
| ENSP00000403910 | 5.73E-06 | -0.1149 | 0 | 0.142 |
| ENSP00000443985 | 1.64E-05 | -0.1149 | 185 | 0.286 |
| ENSP00000263857 | 7.07E-06 | -0.1149 | 388 | 0.821 |
| ENSP00000272542 | 1.33E-05 | -0.1149 | 183 | 0.081 |
| ENSP00000426909 | 3.07E-05 | -0.1149 | 456 | 0.834 |
| ENSP00000261531 | 2.14E-05 | -0.1150 | 524 | 0.756 |
| ENSP00000468348 | 7.38E-06 | -0.1150 | 170 | 0.819 |
| ENSP00000319716 | 4.53E-06 | -0.1150 | 0 | 0.689 |
| ENSP00000334696 | 1.47E-05 | -0.1150 | 0 | 0.180 |
| ENSP00000290921 | 1.82E-05 | -0.1150 | 215 | 0.807 |

| | | | | |
|---|---|---|---|---|
| ENSP00000363944 | 3.05E-06 | -0.1150 | 0 | 0.821 |
| ENSP00000428824 | 7.68E-06 | -0.1150 | 0 | 0.395 |
| ENSP00000285928 | 8.87E-06 | -0.1150 | 450 | 0.669 |
| ENSP00000326052 | 2.40E-05 | -0.1150 | 0 | 0.150 |
| ENSP00000264357 | 2.11E-05 | -0.1150 | 199 | 0.000 |
| ENSP00000311430 | 3.62E-05 | -0.1151 | 505 | 0.859 |
| ENSP00000285896 | 1.15E-05 | -0.1151 | 284 | 0.405 |
| ENSP00000318646 | 9.11E-06 | -0.1151 | 270 | 0.831 |
| ENSP00000272102 | 2.19E-05 | -0.1151 | 315 | 0.566 |
| ENSP00000465655 | 1.23E-05 | -0.1151 | 150 | 0.792 |
| ENSP00000260443 | 5.24E-06 | -0.1151 | 0 | 0.906 |
| ENSP00000313756 | 9.61E-06 | -0.1151 | 202 | 0.000 |
| ENSP00000303887 | 3.68E-06 | -0.1151 | 0 | 0.778 |
| ENSP00000368632 | 3.34E-05 | -0.1151 | 304 | 0.000 |
| ENSP00000304782 | 9.22E-06 | -0.1152 | 158 | 0.332 |
| ENSP00000360200 | 1.15E-05 | -0.1152 | 190 | 0.584 |
| ENSP00000380598 | 3.27E-06 | -0.1152 | 0 | 0.080 |
| ENSP00000394227 | 1.05E-05 | -0.1152 | 242 | 0.717 |
| ENSP00000272227 | 1.14E-05 | -0.1152 | 933 | 0.598 |
| ENSP00000380033 | 1.61E-05 | -0.1152 | 398 | 0.926 |
| ENSP00000262525 | 3.52E-06 | -0.1153 | 0 | 0.722 |
| ENSP00000259915 | 1.10E-05 | -0.1153 | 220 | 0.864 |
| ENSP00000365253 | 5.12E-06 | -0.1153 | 0 | 0.643 |
| ENSP00000345656 | 2.27E-05 | -0.1153 | 214 | 0.284 |
| ENSP00000249647 | 2.79E-05 | -0.1153 | 210 | 0.269 |
| ENSP00000262426 | 8.71E-06 | -0.1153 | 311 | 0.000 |
| ENSP00000335416 | 7.32E-06 | -0.1153 | 0 | 0.207 |
| ENSP00000379951 | 1.01E-05 | -0.1153 | 0 | 0.000 |
| ENSP00000003302 | 8.51E-06 | -0.1154 | 0 | 0.313 |
| ENSP00000354586 | 2.62E-05 | -0.1154 | 345 | 0.892 |
| ENSP00000240316 | 8.61E-06 | -0.1154 | 0 | 0.651 |
| ENSP00000261233 | 1.00E-05 | -0.1154 | 205 | 0.685 |
| ENSP00000334685 | 4.37E-06 | -0.1154 | 0 | 0.695 |
| ENSP00000371529 | 3.07E-06 | -0.1154 | 0 | 0.194 |
| ENSP00000327251 | 2.81E-05 | -0.1154 | 347 | 0.888 |
| ENSP00000254942 | 8.84E-06 | -0.1154 | 0 | 0.686 |
| ENSP00000331487 | 1.31E-05 | -0.1155 | 395 | 0.442 |
| ENSP00000263847 | 1.07E-05 | -0.1155 | 300 | 0.260 |
| ENSP00000366487 | 2.28E-05 | -0.1155 | 274 | 0.487 |
| ENSP00000351539 | 5.75E-06 | -0.1155 | 0 | 0.720 |
| ENSP00000369703 | 3.93E-05 | -0.1155 | 466 | 0.671 |
| ENSP00000357733 | 1.04E-05 | -0.1156 | 0 | 0.149 |
| ENSP00000219168 | 8.44E-06 | -0.1156 | 0 | 0.080 |
| ENSP00000262259 | 4.24E-06 | -0.1156 | 0 | 0.675 |
| ENSP00000364023 | 5.49E-06 | -0.1156 | 0 | 0.673 |
| ENSP00000377576 | 5.09E-07 | -0.1156 | 0 | 0.192 |

| | | | | |
|---|---|---|---|---|
| ENSP00000297265 | 2.97E-06 | -0.1157 | 0 | 0.000 |
| ENSP00000360406 | 2.23E-05 | -0.1157 | 216 | 0.576 |
| ENSP00000314067 | 2.11E-05 | -0.1157 | 598 | 0.781 |
| ENSP00000308472 | 1.87E-05 | -0.1157 | 303 | 0.687 |
| ENSP00000335147 | 8.81E-06 | -0.1157 | 202 | 0.631 |
| ENSP00000324560 | 9.10E-06 | -0.1157 | 207 | 0.514 |
| ENSP00000413411 | 2.40E-06 | -0.1158 | 0 | 0.690 |
| ENSP00000415840 | 5.60E-06 | -0.1158 | 231 | 0.000 |
| ENSP00000263331 | 8.01E-06 | -0.1158 | 466 | 0.808 |
| ENSP00000261302 | 3.54E-06 | -0.1158 | 0 | 0.000 |
| ENSP00000333547 | 1.98E-05 | -0.1158 | 244 | 0.571 |
| ENSP00000299138 | 1.02E-05 | -0.1158 | 160 | 0.229 |
| ENSP00000384084 | 2.35E-05 | -0.1159 | 334 | 0.251 |
| ENSP00000377892 | 2.19E-05 | -0.1159 | 400 | 0.951 |
| ENSP00000349017 | 6.21E-06 | -0.1159 | 160 | 0.364 |
| ENSP00000349503 | 4.37E-06 | -0.1160 | 0 | 0.718 |
| ENSP00000304051 | 1.34E-05 | -0.1160 | 251 | 0.528 |
| ENSP00000371572 | 8.44E-06 | -0.1160 | 0 | 0.169 |
| ENSP00000261942 | 2.03E-05 | -0.1160 | 762 | 0.290 |
| ENSP00000276816 | 5.75E-06 | -0.1160 | 0 | 0.693 |
| ENSP00000275635 | 1.75E-05 | -0.1160 | 0 | 0.206 |
| ENSP00000411672 | 1.64E-05 | -0.1160 | 0 | 0.053 |
| ENSP00000300433 | 1.08E-05 | -0.1161 | 0 | 0.248 |
| ENSP00000384625 | 2.03E-04 | -0.1161 | 317 | 0.767 |
| ENSP00000334373 | 1.84E-05 | -0.1161 | 154 | 0.698 |
| ENSP00000265351 | 1.09E-05 | -0.1161 | 172 | 0.715 |
| ENSP00000355637 | 9.21E-06 | -0.1161 | 160 | 0.389 |
| ENSP00000448598 | 8.48E-06 | -0.1161 | 0 | 0.620 |
| ENSP00000360464 | 3.18E-06 | -0.1161 | 0 | 0.566 |
| ENSP00000216225 | 1.19E-05 | -0.1162 | 185 | 0.633 |
| ENSP00000409107 | 2.13E-06 | -0.1162 | 0 | 0.695 |
| ENSP00000469018 | 2.71E-06 | -0.1162 | 0 | 0.000 |
| ENSP00000417674 | 2.41E-06 | -0.1162 | 0 | 0.091 |
| ENSP00000344241 | 1.93E-05 | -0.1162 | 512 | 0.851 |
| ENSP00000296273 | 7.50E-06 | -0.1163 | 201 | 0.731 |
| ENSP00000261683 | 4.68E-06 | -0.1163 | 0 | 0.000 |
| ENSP00000366923 | 5.68E-06 | -0.1163 | 0 | 0.129 |
| ENSP00000338766 | 8.42E-06 | -0.1164 | 316 | 0.596 |
| ENSP00000435188 | 3.85E-07 | -0.1164 | 0 | 0.000 |
| ENSP00000360671 | 7.43E-06 | -0.1164 | 256 | 0.573 |
| ENSP00000326813 | 1.24E-05 | -0.1164 | 0 | 0.147 |
| ENSP00000361777 | 2.59E-05 | -0.1164 | 288 | 0.000 |
| ENSP00000319690 | 2.27E-05 | -0.1165 | 287 | 0.000 |
| ENSP00000270722 | 3.92E-06 | -0.1165 | 0 | 0.805 |
| ENSP00000405699 | 2.12E-06 | -0.1165 | 0 | 0.691 |
| ENSP00000379369 | 3.13E-06 | -0.1165 | 0 | 0.000 |

| | | | | |
|---|---|---|---|---|
| ENSP00000372067 | 2.53E-05 | -0.1165 | 205 | 0.870 |
| ENSP00000344547 | 4.74E-05 | -0.1165 | 0 | 0.425 |
| ENSP00000322191 | 3.91E-07 | -0.1165 | 0 | 0.172 |
| ENSP00000417864 | 8.04E-06 | -0.1165 | 0 | 0.669 |
| ENSP00000459216 | 7.69E-06 | -0.1165 | 175 | 0.690 |
| ENSP00000217289 | 9.04E-06 | -0.1166 | 0 | 0.263 |
| ENSP00000254260 | 2.84E-06 | -0.1166 | 0 | 0.000 |
| ENSP00000359024 | 6.17E-06 | -0.1166 | 0 | 0.851 |
| ENSP00000384273 | 3.44E-05 | -0.1166 | 948 | 0.887 |
| ENSP00000431202 | 2.07E-06 | -0.1166 | 0 | 0.692 |
| ENSP00000358857 | 1.81E-06 | -0.1166 | 0 | 0.145 |
| ENSP00000286835 | 2.68E-05 | -0.1166 | 270 | 0.703 |
| ENSP00000361418 | 6.94E-06 | -0.1167 | 418 | 0.296 |
| ENSP00000364912 | 1.82E-05 | -0.1167 | 216 | 0.678 |
| ENSP00000320885 | 1.79E-05 | -0.1167 | 272 | 0.404 |
| ENSP00000252996 | 2.94E-05 | -0.1167 | 163 | 0.770 |
| ENSP00000412788 | 1.84E-05 | -0.1167 | 675 | 0.758 |
| ENSP00000370083 | 3.16E-05 | -0.1167 | 195 | 0.668 |
| ENSP00000331465 | 4.09E-06 | -0.1168 | 0 | 0.000 |
| ENSP00000391457 | 3.27E-06 | -0.1168 | 0 | 0.615 |
| ENSP00000468720 | 2.01E-06 | -0.1168 | 0 | 0.687 |
| ENSP00000287647 | 8.44E-06 | -0.1168 | 0 | 0.735 |
| ENSP00000365407 | 3.23E-06 | -0.1168 | 0 | 0.688 |
| ENSP00000470652 | 5.85E-06 | -0.1168 | 0 | 0.101 |
| ENSP00000404127 | 3.26E-06 | -0.1168 | 0 | 0.000 |
| ENSP00000335287 | 9.92E-06 | -0.1168 | 201 | 0.527 |
| ENSP00000248984 | 1.02E-05 | -0.1169 | 0 | 0.000 |
| ENSP00000366070 | 1.27E-05 | -0.1169 | 213 | 0.097 |
| ENSP00000013125 | 7.06E-06 | -0.1169 | 246 | 0.639 |
| ENSP00000314030 | 1.20E-05 | -0.1169 | 938 | 0.472 |
| ENSP00000454268 | 1.90E-07 | -0.1170 | 0 | 0.206 |
| ENSP00000458954 | 7.54E-06 | -0.1170 | 167 | 0.579 |
| ENSP00000260753 | 6.82E-06 | -0.1170 | 313 | 0.312 |
| ENSP00000346659 | 7.43E-06 | -0.1170 | 0 | 0.292 |
| ENSP00000353770 | 1.16E-05 | -0.1170 | 354 | 0.683 |
| ENSP00000261609 | 8.69E-06 | -0.1170 | 177 | 0.704 |
| ENSP00000284049 | 1.53E-05 | -0.1171 | 196 | 0.920 |
| ENSP00000460475 | 5.28E-06 | -0.1171 | 0 | 0.606 |
| ENSP00000265335 | 8.77E-06 | -0.1171 | 208 | 0.000 |
| ENSP00000229314 | 2.69E-05 | -0.1171 | 0 | 0.095 |
| ENSP00000325313 | 2.60E-05 | -0.1171 | 0 | 0.087 |
| ENSP00000341285 | 1.82E-05 | -0.1171 | 469 | 0.000 |
| ENSP00000340688 | 1.49E-05 | -0.1171 | 150 | 0.000 |
| ENSP00000283684 | 1.95E-05 | -0.1171 | 242 | 0.491 |
| ENSP00000263710 | 6.28E-06 | -0.1171 | 0 | 0.167 |
| ENSP00000451261 | 2.52E-05 | -0.1171 | 696 | 0.604 |

| | | | | |
|---|---|---|---|---|
| ENSP00000349430 | 1.64E-05 | -0.1172 | 885 | 0.954 |
| ENSP00000276326 | 1.61E-04 | -0.1172 | 299 | 0.354 |
| ENSP00000311679 | 4.07E-06 | -0.1172 | 0 | 0.656 |
| ENSP00000246000 | 4.00E-05 | -0.1172 | 0 | 0.000 |
| ENSP00000353904 | 9.27E-06 | -0.1172 | 0 | 0.336 |
| ENSP00000258281 | 7.41E-06 | -0.1172 | 0 | 0.835 |
| ENSP00000353863 | 1.59E-05 | -0.1172 | 200 | 0.777 |
| ENSP00000262455 | 7.13E-06 | -0.1172 | 599 | 0.527 |
| ENSP00000452706 | 9.07E-07 | -0.1173 | 0 | 0.000 |
| ENSP00000386960 | 1.80E-06 | -0.1173 | 0 | 0.000 |
| ENSP00000261303 | 5.90E-06 | -0.1173 | 0 | 0.584 |
| ENSP00000351042 | 4.46E-06 | -0.1173 | 0 | 0.681 |
| ENSP00000287143 | 2.52E-05 | -0.1173 | 608 | 0.163 |
| ENSP00000287437 | 7.45E-06 | -0.1173 | 0 | 0.666 |
| ENSP00000306223 | 1.75E-05 | -0.1173 | 427 | 0.907 |
| ENSP00000329558 | 6.80E-06 | -0.1173 | 987 | 0.610 |
| ENSP00000471077 | 1.99E-06 | -0.1174 | 0 | 0.689 |
| ENSP00000338173 | 2.18E-05 | -0.1174 | 225 | 0.685 |
| ENSP00000470948 | 3.58E-07 | -0.1174 | 0 | 0.000 |
| ENSP00000344233 | 4.92E-06 | -0.1174 | 0 | 0.480 |
| ENSP00000383093 | 2.42E-06 | -0.1175 | 0 | 0.162 |
| ENSP00000356448 | 2.31E-05 | -0.1175 | 283 | 0.682 |
| ENSP00000362527 | 5.05E-06 | -0.1175 | 0 | 0.690 |
| ENSP00000282541 | 7.83E-06 | -0.1175 | 0 | 0.283 |
| ENSP00000271732 | 1.90E-05 | -0.1175 | 0 | 0.083 |
| ENSP00000409898 | 2.60E-06 | -0.1175 | 0 | 0.000 |
| ENSP00000356375 | 8.55E-06 | -0.1175 | 0 | 0.664 |
| ENSP00000348302 | 1.63E-06 | -0.1175 | 0 | 0.000 |
| ENSP00000407130 | 1.27E-05 | -0.1175 | 171 | 0.703 |
| ENSP00000375133 | 5.33E-06 | -0.1175 | 0 | 0.683 |
| ENSP00000367440 | 7.37E-06 | -0.1176 | 0 | 0.364 |
| ENSP00000401802 | 3.33E-05 | -0.1176 | 360 | 0.828 |
| ENSP00000396857 | 5.84E-06 | -0.1176 | 0 | 0.680 |
| ENSP00000438875 | 5.04E-06 | -0.1176 | 0 | 0.637 |
| ENSP00000338671 | 2.09E-05 | -0.1176 | 205 | 0.558 |
| ENSP00000320485 | 2.91E-05 | -0.1176 | 452 | 0.766 |
| ENSP00000261173 | 2.42E-06 | -0.1176 | 0 | 0.626 |
| ENSP00000347821 | 4.21E-06 | -0.1176 | 0 | 0.116 |
| ENSP00000381703 | 6.89E-08 | -0.1176 | 0 | 0.104 |
| ENSP00000378917 | 1.67E-05 | -0.1176 | 292 | 0.640 |
| ENSP00000277554 | 4.12E-06 | -0.1177 | 0 | 0.690 |
| ENSP00000388902 | 3.01E-06 | -0.1177 | 0 | 0.000 |
| ENSP00000333188 | 9.04E-06 | -0.1177 | 226 | 0.000 |
| ENSP00000166244 | 6.46E-06 | -0.1177 | 229 | 0.678 |
| ENSP00000215659 | 6.70E-06 | -0.1178 | 430 | 0.664 |
| ENSP00000265965 | 5.01E-06 | -0.1178 | 0 | 0.133 |

| | | | | |
|---|---|---|---|---|
| ENSP00000331602 | 2.12E-05 | -0.1178 | 906 | 0.667 |
| ENSP00000420269 | 1.50E-05 | -0.1178 | 431 | 0.278 |
| ENSP00000374501 | 1.83E-06 | -0.1178 | 0 | 0.393 |
| ENSP00000299596 | 2.67E-05 | -0.1178 | 0 | 0.067 |
| ENSP00000326806 | 1.87E-05 | -0.1178 | 386 | 0.897 |
| ENSP00000357586 | 5.33E-06 | -0.1179 | 0 | 0.177 |
| ENSP00000339016 | 1.27E-05 | -0.1179 | 272 | 0.318 |
| ENSP00000377969 | 1.49E-05 | -0.1179 | 218 | 0.798 |
| ENSP00000306864 | 8.63E-06 | -0.1179 | 0 | 0.661 |
| ENSP00000404042 | 8.99E-06 | -0.1179 | 162 | 0.142 |
| ENSP00000290573 | 1.17E-05 | -0.1179 | 255 | 0.680 |
| ENSP00000412272 | 2.06E-06 | -0.1180 | 0 | 0.000 |
| ENSP00000272444 | 2.03E-05 | -0.1180 | 0 | 0.400 |
| ENSP00000414893 | 5.55E-06 | -0.1180 | 0 | 0.000 |
| ENSP00000336842 | 2.08E-05 | -0.1180 | 334 | 0.704 |
| ENSP00000284481 | 8.87E-07 | -0.1180 | 0 | 0.000 |
| ENSP00000265773 | 1.66E-05 | -0.1180 | 501 | 0.000 |
| ENSP00000360290 | 1.40E-05 | -0.1180 | 240 | 0.739 |
| ENSP00000343001 | 9.82E-06 | -0.1180 | 0 | 0.546 |
| ENSP00000359378 | 7.17E-06 | -0.1180 | 0 | 0.124 |
| ENSP00000344801 | 1.05E-05 | -0.1181 | 191 | 0.172 |
| ENSP00000410890 | 2.17E-06 | -0.1181 | 0 | 0.681 |
| ENSP00000350990 | 3.82E-06 | -0.1181 | 0 | 0.297 |
| ENSP00000352336 | 1.74E-05 | -0.1181 | 256 | 0.758 |
| ENSP00000342374 | 2.00E-05 | -0.1181 | 341 | 0.861 |
| ENSP00000349401 | 2.87E-06 | -0.1181 | 0 | 0.671 |
| ENSP00000419119 | 1.08E-07 | -0.1182 | 0 | 0.206 |
| ENSP00000446132 | 5.04E-06 | -0.1182 | 0 | 0.096 |
| ENSP00000322887 | 1.24E-05 | -0.1182 | 159 | 0.976 |
| ENSP00000356143 | 4.77E-06 | -0.1182 | 0 | 0.672 |
| ENSP00000348984 | 1.30E-05 | -0.1182 | 0 | 0.577 |
| ENSP00000391723 | 1.94E-05 | -0.1182 | 225 | 0.683 |
| ENSP00000251372 | 3.07E-05 | -0.1182 | 0 | 0.216 |
| ENSP00000232564 | 5.29E-06 | -0.1183 | 397 | 0.653 |
| ENSP00000264808 | 4.26E-06 | -0.1183 | 0 | 0.764 |
| ENSP00000249923 | 7.37E-06 | -0.1183 | 0 | 0.328 |
| ENSP00000352111 | 5.77E-06 | -0.1183 | 0 | 0.124 |
| ENSP00000360798 | 1.35E-05 | -0.1183 | 338 | 0.636 |
| ENSP00000449500 | 2.48E-06 | -0.1183 | 0 | 0.160 |
| ENSP00000310572 | 8.56E-06 | -0.1183 | 342 | 0.678 |
| ENSP00000433459 | 6.78E-06 | -0.1183 | 0 | 0.415 |
| ENSP00000287139 | 1.86E-05 | -0.1183 | 150 | 0.873 |
| ENSP00000370447 | 1.62E-06 | -0.1184 | 0 | 0.172 |
| ENSP00000325863 | 1.42E-05 | -0.1184 | 193 | 0.839 |
| ENSP00000440847 | 1.01E-05 | -0.1184 | 270 | 0.602 |
| ENSP00000324450 | 4.49E-06 | -0.1184 | 0 | 0.000 |

| | | | | |
|---|---|---|---|---|
| ENSP00000230461 | 1.47E-05 | -0.1184 | 0 | 0.101 |
| ENSP00000003084 | 9.50E-06 | -0.1184 | 829 | 0.749 |
| ENSP00000005257 | 8.78E-06 | -0.1184 | 523 | 0.608 |
| ENSP00000362609 | 3.18E-06 | -0.1185 | 0 | 0.677 |
| ENSP00000355465 | 3.23E-06 | -0.1185 | 0 | 0.384 |
| ENSP00000429266 | 2.35E-06 | -0.1185 | 0 | 0.696 |
| ENSP00000469337 | 3.36E-06 | -0.1185 | 0 | 0.663 |
| ENSP00000453973 | 4.28E-06 | -0.1185 | 0 | 0.000 |
| ENSP00000344259 | 2.08E-05 | -0.1185 | 216 | 0.692 |
| ENSP00000334501 | 3.13E-06 | -0.1186 | 0 | 0.202 |
| ENSP00000307023 | 1.34E-05 | -0.1186 | 300 | 0.760 |
| ENSP00000359925 | 1.15E-05 | -0.1186 | 510 | 0.615 |
| ENSP00000267085 | 1.10E-05 | -0.1186 | 218 | 0.256 |
| ENSP00000337478 | 2.09E-05 | -0.1186 | 274 | 0.416 |
| ENSP00000353826 | 1.17E-05 | -0.1186 | 307 | 0.447 |
| ENSP00000400717 | 3.18E-05 | -0.1187 | 508 | 0.648 |
| ENSP00000301067 | 1.41E-05 | -0.1187 | 244 | 0.000 |
| ENSP00000424183 | 4.76E-06 | -0.1187 | 0 | 0.109 |
| ENSP00000385361 | 1.56E-05 | -0.1187 | 265 | 0.593 |
| ENSP00000468862 | 4.96E-06 | -0.1187 | 0 | 0.000 |
| ENSP00000320949 | 1.71E-05 | -0.1188 | 272 | 0.601 |
| ENSP00000398880 | 2.15E-05 | -0.1188 | 172 | 0.868 |
| ENSP00000419475 | 1.62E-06 | -0.1188 | 0 | 0.129 |
| ENSP00000372347 | 2.35E-05 | -0.1188 | 244 | 0.894 |
| ENSP00000470969 | 1.97E-06 | -0.1188 | 0 | 0.000 |
| ENSP00000269391 | 6.00E-06 | -0.1188 | 0 | 0.105 |
| ENSP00000250559 | 8.36E-06 | -0.1188 | 933 | 0.600 |
| ENSP00000470770 | 1.91E-07 | -0.1188 | 0 | 0.000 |
| ENSP00000400958 | 2.86E-06 | -0.1189 | 0 | 0.131 |
| ENSP00000397566 | 8.22E-06 | -0.1189 | 0 | 0.384 |
| ENSP00000367498 | 1.62E-05 | -0.1189 | 188 | 0.835 |
| ENSP00000335493 | 2.75E-06 | -0.1190 | 0 | 0.000 |
| ENSP00000347232 | 1.45E-05 | -0.1190 | 150 | 0.802 |
| ENSP00000309591 | 1.84E-05 | -0.1191 | 900 | 0.642 |
| ENSP00000360411 | 2.21E-05 | -0.1191 | 0 | 0.096 |
| ENSP00000379047 | 3.22E-06 | -0.1191 | 0 | 0.131 |
| ENSP00000353864 | 9.40E-06 | -0.1191 | 151 | 0.731 |
| ENSP00000456894 | 1.56E-06 | -0.1191 | 0 | 0.151 |
| ENSP00000261182 | 9.62E-06 | -0.1191 | 326 | 0.000 |
| ENSP00000216297 | 1.44E-05 | -0.1191 | 445 | 0.924 |
| ENSP00000302898 | 7.13E-06 | -0.1191 | 186 | 0.454 |
| ENSP00000463645 | 1.96E-05 | -0.1192 | 244 | 0.000 |
| ENSP00000355899 | 3.94E-06 | -0.1192 | 0 | 0.854 |
| ENSP00000336721 | 7.25E-06 | -0.1192 | 0 | 0.925 |
| ENSP00000386796 | 3.36E-06 | -0.1192 | 0 | 0.065 |
| ENSP00000303147 | 9.18E-06 | -0.1192 | 414 | 0.638 |

| | | | | |
|---|---|---|---|---|
| ENSP00000406885 | 2.51E-06 | -0.1192 | 0 | 0.077 |
| ENSP00000352645 | 7.37E-06 | -0.1192 | 204 | 0.517 |
| ENSP00000365057 | 5.42E-06 | -0.1193 | 0 | 0.172 |
| ENSP00000302855 | 4.16E-06 | -0.1193 | 0 | 0.733 |
| ENSP00000403975 | 3.05E-06 | -0.1193 | 0 | 0.697 |
| ENSP00000301727 | 4.69E-06 | -0.1193 | 0 | 0.780 |
| ENSP00000243914 | 3.74E-06 | -0.1194 | 0 | 0.736 |
| ENSP00000363064 | 6.19E-06 | -0.1194 | 0 | 0.672 |
| ENSP00000211287 | 6.26E-06 | -0.1194 | 389 | 0.665 |
| ENSP00000264110 | 1.56E-05 | -0.1194 | 406 | 0.000 |
| ENSP00000326381 | 1.31E-05 | -0.1194 | 961 | 0.000 |
| ENSP00000378165 | 1.65E-05 | -0.1194 | 276 | 0.687 |
| ENSP00000370253 | 2.73E-05 | -0.1194 | 260 | 0.874 |
| ENSP00000338413 | 2.59E-05 | -0.1194 | 394 | 0.741 |
| ENSP00000453364 | 1.81E-06 | -0.1194 | 0 | 0.189 |
| ENSP00000380178 | 1.72E-05 | -0.1194 | 178 | 0.659 |
| ENSP00000429562 | 2.12E-05 | -0.1195 | 196 | 0.337 |
| ENSP00000418910 | 4.33E-06 | -0.1195 | 0 | 0.000 |
| ENSP00000294702 | 8.34E-06 | -0.1195 | 193 | 0.832 |
| ENSP00000357425 | 6.73E-06 | -0.1195 | 0 | 0.099 |
| ENSP00000330633 | 1.37E-05 | -0.1195 | 163 | 0.818 |
| ENSP00000336606 | 8.03E-06 | -0.1195 | 160 | 0.000 |
| ENSP00000325377 | 2.95E-05 | -0.1196 | 161 | 0.651 |
| ENSP00000307540 | 2.14E-05 | -0.1196 | 215 | 0.187 |
| ENSP00000325376 | 9.58E-06 | -0.1196 | 196 | 0.946 |
| ENSP00000274606 | 1.06E-05 | -0.1196 | 231 | 0.000 |
| ENSP00000375593 | 3.82E-06 | -0.1196 | 0 | 0.691 |
| ENSP00000292614 | 6.48E-06 | -0.1196 | 0 | 0.828 |
| ENSP00000332513 | 4.84E-05 | -0.1196 | 392 | 0.078 |
| ENSP00000457881 | 1.66E-05 | -0.1196 | 242 | 0.000 |
| ENSP00000471000 | 2.16E-06 | -0.1196 | 0 | 0.694 |
| ENSP00000264972 | 1.21E-05 | -0.1196 | 365 | 0.675 |
| ENSP00000317468 | 2.57E-06 | -0.1197 | 0 | 0.000 |
| ENSP00000324636 | 8.35E-06 | -0.1197 | 175 | 0.000 |
| ENSP00000262646 | 7.72E-06 | -0.1197 | 231 | 0.590 |
| ENSP00000322304 | 1.35E-05 | -0.1197 | 251 | 0.518 |
| ENSP00000446872 | 1.75E-06 | -0.1197 | 0 | 0.000 |
| ENSP00000353893 | 2.33E-05 | -0.1198 | 300 | 0.125 |
| ENSP00000375073 | 3.52E-06 | -0.1198 | 0 | 0.512 |
| ENSP00000324740 | 2.72E-05 | -0.1198 | 504 | 0.656 |
| ENSP00000264335 | 1.32E-05 | -0.1198 | 649 | 0.697 |
| ENSP00000417281 | 3.69E-05 | -0.1198 | 800 | 0.826 |
| ENSP00000394394 | 5.91E-06 | -0.1198 | 0 | 0.408 |
| ENSP00000378323 | 3.50E-05 | -0.1199 | 375 | 0.657 |
| ENSP00000310042 | 5.70E-06 | -0.1199 | 0 | 0.834 |
| ENSP00000431971 | 3.58E-06 | -0.1199 | 0 | 0.123 |

| | | | | |
|---|---|---|---|---|
| ENSP00000229328 | 3.88E-06 | -0.1199 | 0 | 0.000 |
| ENSP00000298428 | 6.72E-06 | -0.1199 | 516 | 0.569 |
| ENSP00000384675 | 2.94E-05 | -0.1199 | 905 | 0.812 |
| ENSP00000418668 | 5.24E-06 | -0.1200 | 0 | 0.240 |
| ENSP00000254940 | 4.04E-06 | -0.1200 | 0 | 0.907 |
| ENSP00000237654 | 1.22E-05 | -0.1200 | 195 | 0.630 |
| ENSP00000331385 | 1.77E-05 | -0.1200 | 0 | 0.163 |
| ENSP00000319053 | 1.04E-05 | -0.1200 | 150 | 0.736 |
| ENSP00000331310 | 2.01E-05 | -0.1201 | 339 | 0.934 |
| ENSP00000336687 | 8.29E-06 | -0.1201 | 556 | 0.860 |
| ENSP00000222725 | 1.77E-05 | -0.1201 | 271 | 0.850 |
| ENSP00000262085 | 1.33E-05 | -0.1202 | 181 | 0.000 |
| ENSP00000342082 | 8.37E-05 | -0.1202 | 407 | 0.770 |
| ENSP00000333667 | 1.12E-05 | -0.1202 | 851 | 0.631 |
| ENSP00000261205 | 1.11E-05 | -0.1202 | 170 | 0.315 |
| ENSP00000376808 | 2.11E-05 | -0.1202 | 330 | 0.714 |
| ENSP00000369915 | 1.95E-05 | -0.1202 | 326 | 0.000 |
| ENSP00000315265 | 2.72E-05 | -0.1203 | 0 | 0.125 |
| ENSP00000304308 | 1.48E-05 | -0.1203 | 197 | 0.759 |
| ENSP00000265728 | 3.95E-06 | -0.1203 | 0 | 0.675 |
| ENSP00000385610 | 3.75E-06 | -0.1203 | 0 | 0.519 |
| ENSP00000244769 | 1.59E-05 | -0.1203 | 198 | 0.692 |
| ENSP00000375557 | 1.62E-05 | -0.1204 | 165 | 0.000 |
| ENSP00000360757 | 5.33E-06 | -0.1204 | 0 | 0.148 |
| ENSP00000259605 | 2.11E-05 | -0.1204 | 0 | 0.447 |
| ENSP00000264010 | 8.60E-06 | -0.1204 | 207 | 0.807 |
| ENSP00000343709 | 1.37E-05 | -0.1205 | 0 | 0.127 |
| ENSP00000323740 | 1.91E-05 | -0.1205 | 330 | 0.658 |
| ENSP00000346148 | 7.84E-06 | -0.1205 | 279 | 0.762 |
| ENSP00000319336 | 9.93E-06 | -0.1205 | 0 | 0.162 |
| ENSP00000382327 | 3.55E-06 | -0.1205 | 0 | 0.113 |
| ENSP00000338020 | 1.73E-05 | -0.1206 | 959 | 0.785 |
| ENSP00000265734 | 1.77E-05 | -0.1206 | 339 | 0.000 |
| ENSP00000376103 | 2.30E-06 | -0.1206 | 0 | 0.141 |
| ENSP00000444788 | 1.52E-05 | -0.1206 | 356 | 0.139 |
| ENSP00000429374 | 7.61E-06 | -0.1206 | 406 | 0.768 |
| ENSP00000380460 | 2.28E-05 | -0.1206 | 231 | 0.619 |
| ENSP00000310205 | 1.27E-05 | -0.1207 | 216 | 0.668 |
| ENSP00000358491 | 5.58E-06 | -0.1207 | 0 | 0.111 |
| ENSP00000351284 | 1.59E-05 | -0.1207 | 205 | 0.874 |
| ENSP00000363640 | 8.38E-06 | -0.1207 | 358 | 0.757 |
| ENSP00000364859 | 1.84E-05 | -0.1207 | 342 | 0.619 |
| ENSP00000295101 | 2.06E-05 | -0.1207 | 299 | 0.485 |
| ENSP00000379704 | 5.61E-06 | -0.1207 | 0 | 0.237 |
| ENSP00000372160 | 2.06E-05 | -0.1207 | 0 | 0.068 |
| ENSP00000365514 | 1.49E-05 | -0.1208 | 863 | 0.862 |

| | | | | |
|---|---|---|---|---|
| ENSP00000356357 | 1.13E-05 | -0.1208 | 0 | 0.838 |
| ENSP00000370330 | 5.35E-06 | -0.1208 | 0 | 0.740 |
| ENSP00000455307 | 2.79E-05 | -0.1208 | 900 | 0.924 |
| ENSP00000321132 | 6.83E-06 | -0.1209 | 0 | 0.698 |
| ENSP00000333926 | 6.42E-06 | -0.1209 | 160 | 0.392 |
| ENSP00000307288 | 1.26E-05 | -0.1210 | 171 | 0.000 |
| ENSP00000265428 | 4.68E-06 | -0.1210 | 0 | 0.608 |
| ENSP00000340998 | 2.41E-05 | -0.1210 | 0 | 0.260 |
| ENSP00000263642 | 1.10E-05 | -0.1210 | 157 | 0.662 |
| ENSP00000217244 | 6.57E-06 | -0.1210 | 920 | 0.000 |
| ENSP00000384330 | 2.58E-05 | -0.1210 | 903 | 0.512 |
| ENSP00000424038 | 1.82E-05 | -0.1210 | 921 | 0.799 |
| ENSP00000264818 | 1.32E-05 | -0.1211 | 251 | 0.660 |
| ENSP00000302603 | 8.35E-06 | -0.1211 | 0 | 0.690 |
| ENSP00000256797 | 7.19E-06 | -0.1211 | 873 | 0.678 |
| ENSP00000368477 | 5.66E-06 | -0.1212 | 232 | 0.115 |
| ENSP00000367408 | 1.14E-05 | -0.1212 | 350 | 0.646 |
| ENSP00000466799 | 6.17E-06 | -0.1212 | 0 | 0.373 |
| ENSP00000387966 | 8.31E-06 | -0.1212 | 0 | 0.000 |
| ENSP00000339830 | 4.55E-06 | -0.1212 | 0 | 0.155 |
| ENSP00000220003 | 1.24E-05 | -0.1212 | 923 | 0.666 |
| ENSP00000314776 | 2.22E-06 | -0.1213 | 0 | 0.000 |
| ENSP00000303915 | 7.11E-06 | -0.1213 | 0 | 0.702 |
| ENSP00000412922 | 4.30E-06 | -0.1213 | 0 | 0.495 |
| ENSP00000454241 | 2.10E-06 | -0.1213 | 0 | 0.000 |
| ENSP00000383402 | 2.37E-06 | -0.1213 | 0 | 0.422 |
| ENSP00000316809 | 3.06E-06 | -0.1213 | 0 | 0.000 |
| ENSP00000264318 | 4.31E-06 | -0.1213 | 0 | 0.318 |
| ENSP00000336591 | 1.30E-05 | -0.1214 | 287 | 0.549 |
| ENSP00000271555 | 8.37E-06 | -0.1214 | 227 | 0.000 |
| ENSP00000472801 | 2.06E-06 | -0.1214 | 0 | 0.000 |
| ENSP00000419038 | 3.43E-05 | -0.1214 | 431 | 0.803 |
| ENSP00000349486 | 5.65E-06 | -0.1214 | 0 | 0.740 |
| ENSP00000398290 | 2.61E-06 | -0.1214 | 0 | 0.150 |
| ENSP00000337641 | 8.99E-06 | -0.1214 | 0 | 0.604 |
| ENSP00000263955 | 4.22E-06 | -0.1215 | 0 | 0.673 |
| ENSP00000471539 | 1.39E-07 | -0.1215 | 0 | 0.000 |
| ENSP00000347648 | 3.88E-06 | -0.1215 | 0 | 0.672 |
| ENSP00000349175 | 9.40E-06 | -0.1215 | 0 | 0.000 |
| ENSP00000379658 | 2.16E-05 | -0.1215 | 284 | 0.856 |
| ENSP00000417147 | 3.95E-06 | -0.1215 | 0 | 0.130 |
| ENSP00000300145 | 3.36E-06 | -0.1216 | 0 | 0.000 |
| ENSP00000381081 | 5.91E-06 | -0.1216 | 0 | 0.108 |
| ENSP00000420384 | 1.91E-05 | -0.1216 | 161 | 0.114 |
| ENSP00000392189 | 3.29E-06 | -0.1216 | 0 | 0.122 |
| ENSP00000325091 | 5.20E-06 | -0.1216 | 0 | 0.561 |

| | | | | |
|---|---|---|---|---|
| ENSP00000395007 | 3.36E-06 | -0.1216 | 0 | 0.665 |
| ENSP00000371003 | 2.36E-06 | -0.1216 | 0 | 0.506 |
| ENSP00000379895 | 9.03E-06 | -0.1216 | 0 | 0.165 |
| ENSP00000280326 | 1.37E-05 | -0.1217 | 772 | 0.722 |
| ENSP00000365807 | 9.54E-06 | -0.1217 | 240 | 0.696 |
| ENSP00000334738 | 4.03E-06 | -0.1217 | 0 | 0.000 |
| ENSP00000282572 | 3.47E-06 | -0.1217 | 0 | 0.628 |
| ENSP00000334008 | 1.43E-05 | -0.1218 | 0 | 0.407 |
| ENSP00000234310 | 7.30E-06 | -0.1218 | 207 | 0.658 |
| ENSP00000394249 | 2.18E-06 | -0.1218 | 0 | 0.530 |
| ENSP00000305449 | 1.39E-05 | -0.1218 | 189 | 0.130 |
| ENSP00000353238 | 2.09E-05 | -0.1218 | 231 | 0.790 |
| ENSP00000335388 | 3.33E-06 | -0.1218 | 0 | 0.000 |
| ENSP00000359845 | 2.98E-08 | -0.1218 | 0 | 0.214 |
| ENSP00000283357 | 1.40E-05 | -0.1218 | 0 | 0.184 |
| ENSP00000311505 | 1.92E-05 | -0.1218 | 304 | 0.364 |
| ENSP00000271715 | 6.26E-06 | -0.1218 | 159 | 0.762 |
| ENSP00000443070 | 8.57E-07 | -0.1218 | 0 | 0.000 |
| ENSP00000374265 | 2.14E-05 | -0.1219 | 242 | 0.339 |
| ENSP00000305373 | 3.83E-06 | -0.1219 | 0 | 0.692 |
| ENSP00000370194 | 5.15E-06 | -0.1219 | 0 | 0.144 |
| ENSP00000354855 | 4.88E-06 | -0.1219 | 0 | 0.761 |
| ENSP00000237264 | 1.12E-05 | -0.1219 | 330 | 0.000 |
| ENSP00000265827 | 6.79E-06 | -0.1219 | 0 | 0.682 |
| ENSP00000365877 | 2.57E-05 | -0.1220 | 244 | 0.000 |
| ENSP00000350332 | 1.64E-05 | -0.1220 | 900 | 0.204 |
| ENSP00000420821 | 1.47E-05 | -0.1220 | 0 | 0.678 |
| ENSP00000296127 | 1.41E-05 | -0.1220 | 0 | 0.089 |
| ENSP00000385028 | 1.26E-05 | -0.1220 | 0 | 0.080 |
| ENSP00000439228 | 1.22E-06 | -0.1220 | 0 | 0.148 |
| ENSP00000448536 | 2.50E-06 | -0.1220 | 0 | 0.130 |
| ENSP00000369907 | 4.09E-06 | -0.1221 | 0 | 0.000 |
| ENSP00000457423 | 4.25E-06 | -0.1221 | 0 | 0.696 |
| ENSP00000349853 | 3.61E-06 | -0.1221 | 0 | 0.000 |
| ENSP00000430684 | 2.73E-05 | -0.1221 | 447 | 0.887 |
| ENSP00000264864 | 3.41E-06 | -0.1222 | 0 | 0.000 |
| ENSP00000408411 | 2.40E-05 | -0.1222 | 378 | 0.392 |
| ENSP00000424737 | 3.61E-06 | -0.1222 | 0 | 0.000 |
| ENSP00000252799 | 6.66E-06 | -0.1222 | 0 | 0.687 |
| ENSP00000283122 | 5.29E-06 | -0.1222 | 213 | 0.669 |
| ENSP00000413035 | 1.34E-05 | -0.1222 | 518 | 0.721 |
| ENSP00000378414 | 2.03E-05 | -0.1222 | 188 | 0.913 |
| ENSP00000273398 | 1.35E-05 | -0.1222 | 200 | 0.256 |
| ENSP00000311449 | 2.08E-05 | -0.1223 | 232 | 0.248 |
| ENSP00000425561 | 2.33E-05 | -0.1223 | 716 | 0.766 |
| ENSP00000470117 | 1.07E-07 | -0.1223 | 0 | 0.000 |

| | | | | |
|---|---|---|---|---|
| ENSP00000326841 | 6.03E-06 | -0.1223 | 0 | 0.150 |
| ENSP00000373167 | 4.21E-06 | -0.1223 | 0 | 0.107 |
| ENSP00000401399 | 3.56E-05 | -0.1223 | 499 | 0.947 |
| ENSP00000413520 | 1.16E-05 | -0.1223 | 298 | 0.087 |
| ENSP00000379880 | 4.76E-06 | -0.1224 | 0 | 0.691 |
| ENSP00000348168 | 3.68E-06 | -0.1224 | 0 | 0.786 |
| ENSP00000328515 | 5.63E-06 | -0.1224 | 0 | 0.700 |
| ENSP00000371973 | 2.56E-05 | -0.1224 | 374 | 0.829 |
| ENSP00000357669 | 1.23E-05 | -0.1224 | 159 | 0.666 |
| ENSP00000354620 | 3.91E-06 | -0.1224 | 0 | 0.000 |
| ENSP00000465578 | 3.07E-06 | -0.1225 | 0 | 0.698 |
| ENSP00000362900 | 2.02E-05 | -0.1225 | 400 | 0.948 |
| ENSP00000420477 | 1.86E-05 | -0.1225 | 312 | 0.715 |
| ENSP00000363275 | 9.62E-06 | -0.1225 | 475 | 0.630 |
| ENSP00000366955 | 1.65E-06 | -0.1225 | 0 | 0.273 |
| ENSP00000362352 | 9.20E-06 | -0.1225 | 198 | 0.967 |
| ENSP00000262238 | 1.57E-05 | -0.1226 | 951 | 0.817 |
| ENSP00000344844 | 1.60E-05 | -0.1226 | 215 | 0.418 |
| ENSP00000330842 | 6.58E-06 | -0.1226 | 160 | 0.711 |
| ENSP00000363330 | 1.36E-05 | -0.1227 | 203 | 0.452 |
| ENSP00000364613 | 1.83E-06 | -0.1227 | 0 | 0.000 |
| ENSP00000319192 | 3.64E-06 | -0.1227 | 0 | 0.000 |
| ENSP00000372689 | 3.51E-06 | -0.1227 | 0 | 0.671 |
| ENSP00000360412 | 2.39E-05 | -0.1227 | 366 | 0.873 |
| ENSP00000324804 | 5.67E-06 | -0.1228 | 0 | 0.622 |
| ENSP00000433361 | 4.46E-06 | -0.1228 | 0 | 0.121 |
| ENSP00000406318 | 2.10E-06 | -0.1228 | 0 | 0.697 |
| ENSP00000308610 | 5.46E-06 | -0.1228 | 203 | 0.667 |
| ENSP00000352420 | 2.93E-06 | -0.1228 | 0 | 0.197 |
| ENSP00000443411 | 1.29E-05 | -0.1228 | 197 | 0.347 |
| ENSP00000361280 | 8.76E-06 | -0.1228 | 414 | 0.000 |
| ENSP00000348996 | 2.67E-06 | -0.1228 | 0 | 0.202 |
| ENSP00000354525 | 5.16E-06 | -0.1229 | 240 | 0.766 |
| ENSP00000345144 | 1.41E-05 | -0.1229 | 0 | 0.240 |
| ENSP00000468046 | 1.93E-06 | -0.1229 | 0 | 0.000 |
| ENSP00000349204 | 1.82E-05 | -0.1229 | 181 | 0.179 |
| ENSP00000371138 | 2.66E-05 | -0.1229 | 306 | 0.803 |
| ENSP00000357858 | 7.49E-06 | -0.1229 | 0 | 0.650 |
| ENSP00000360727 | 2.13E-05 | -0.1230 | 284 | 0.242 |
| ENSP00000373215 | 1.18E-05 | -0.1230 | 313 | 0.158 |
| ENSP00000440920 | 1.91E-07 | -0.1230 | 0 | 0.000 |
| ENSP00000339356 | 2.11E-06 | -0.1230 | 0 | 0.190 |
| ENSP00000437563 | 2.51E-07 | -0.1230 | 0 | 0.000 |
| ENSP00000392147 | 5.33E-06 | -0.1231 | 0 | 0.227 |
| ENSP00000363998 | 1.47E-05 | -0.1231 | 219 | 0.730 |
| ENSP00000383938 | 1.81E-05 | -0.1231 | 439 | 0.095 |

| | | | | |
|---|---|---|---|---|
| ENSP00000334051 | 4.30E-06 | -0.1231 | 205 | 0.000 |
| ENSP00000262105 | 9.29E-06 | -0.1231 | 236 | 0.000 |
| ENSP00000349722 | 2.03E-05 | -0.1231 | 328 | 0.644 |
| ENSP00000367655 | 2.84E-06 | -0.1232 | 0 | 0.126 |
| ENSP00000345731 | 1.40E-05 | -0.1232 | 310 | 0.613 |
| ENSP00000366604 | 3.33E-05 | -0.1232 | 644 | 0.872 |
| ENSP00000460850 | 1.84E-05 | -0.1232 | 645 | 0.056 |
| ENSP00000364893 | 2.21E-05 | -0.1232 | 268 | 0.858 |
| ENSP00000261951 | 9.65E-06 | -0.1232 | 250 | 0.684 |
| ENSP00000366628 | 5.40E-06 | -0.1232 | 0 | 0.108 |
| ENSP00000270162 | 1.05E-05 | -0.1233 | 151 | 0.772 |
| ENSP00000387307 | 1.87E-05 | -0.1233 | 196 | 0.232 |
| ENSP00000262854 | 6.58E-06 | -0.1233 | 361 | 0.732 |
| ENSP00000358554 | 1.92E-05 | -0.1233 | 275 | 0.722 |
| ENSP00000380313 | 5.65E-06 | -0.1233 | 0 | 0.693 |
| ENSP00000391067 | 5.41E-06 | -0.1233 | 0 | 0.683 |
| ENSP00000473193 | 1.60E-06 | -0.1234 | 0 | 0.000 |
| ENSP00000413660 | 1.97E-06 | -0.1234 | 0 | 0.690 |
| ENSP00000393324 | 1.58E-05 | -0.1234 | 188 | 0.822 |
| ENSP00000269051 | 5.63E-06 | -0.1234 | 153 | 0.665 |
| ENSP00000315644 | 9.16E-06 | -0.1234 | 235 | 0.652 |
| ENSP00000323913 | 5.10E-06 | -0.1235 | 0 | 0.210 |
| ENSP00000420959 | 2.22E-06 | -0.1235 | 0 | 0.000 |
| ENSP00000340874 | 2.39E-05 | -0.1235 | 430 | 0.000 |
| ENSP00000469474 | 1.53E-07 | -0.1235 | 0 | 0.000 |
| ENSP00000419417 | 1.20E-06 | -0.1235 | 0 | 0.134 |
| ENSP00000316176 | 2.15E-05 | -0.1235 | 228 | 0.710 |
| ENSP00000402414 | 3.64E-06 | -0.1236 | 0 | 0.251 |
| ENSP00000440896 | 3.68E-06 | -0.1236 | 0 | 0.101 |
| ENSP00000417078 | 2.06E-05 | -0.1236 | 227 | 0.899 |
| ENSP00000238497 | 9.86E-06 | -0.1236 | 0 | 0.175 |
| ENSP00000369798 | 4.02E-06 | -0.1236 | 0 | 0.126 |
| ENSP00000362485 | 6.06E-06 | -0.1236 | 0 | 0.131 |
| ENSP00000385722 | 4.88E-06 | -0.1236 | 0 | 0.282 |
| ENSP00000417183 | 6.62E-06 | -0.1237 | 0 | 0.699 |
| ENSP00000346389 | 2.76E-05 | -0.1237 | 452 | 0.000 |
| ENSP00000368030 | 2.41E-05 | -0.1237 | 198 | 0.440 |
| ENSP00000380960 | 6.35E-06 | -0.1237 | 0 | 0.000 |
| ENSP00000282356 | 3.64E-06 | -0.1237 | 0 | 0.000 |
| ENSP00000256458 | 9.07E-06 | -0.1237 | 205 | 0.693 |
| ENSP00000427514 | 4.34E-05 | -0.1237 | 198 | 0.866 |
| ENSP00000379651 | 2.31E-06 | -0.1237 | 0 | 0.168 |
| ENSP00000333775 | 6.02E-06 | -0.1237 | 0 | 0.199 |
| ENSP00000300619 | 5.96E-06 | -0.1237 | 0 | 0.699 |
| ENSP00000245663 | 5.75E-06 | -0.1238 | 0 | 0.706 |
| ENSP00000316578 | 7.34E-06 | -0.1238 | 0 | 0.820 |

| | | | | |
|---|---|---|---|---|
| ENSP00000258091 | 1.14E-05 | -0.1238 | 795 | 0.728 |
| ENSP00000416897 | 7.83E-08 | -0.1238 | 0 | 0.000 |
| ENSP00000410083 | 5.62E-06 | -0.1238 | 0 | 0.115 |
| ENSP00000366267 | 3.07E-05 | -0.1238 | 917 | 0.614 |
| ENSP00000325776 | 4.85E-06 | -0.1238 | 0 | 0.176 |
| ENSP00000338862 | 1.48E-05 | -0.1239 | 331 | 0.924 |
| ENSP00000416240 | 3.85E-06 | -0.1239 | 0 | 0.000 |
| ENSP00000426120 | 1.50E-05 | -0.1239 | 0 | 0.058 |
| ENSP00000294740 | 6.13E-06 | -0.1239 | 0 | 0.716 |
| ENSP00000431391 | 5.33E-06 | -0.1239 | 0 | 0.290 |
| ENSP00000319140 | 8.14E-06 | -0.1239 | 0 | 0.170 |
| ENSP00000353472 | 3.43E-05 | -0.1239 | 281 | 0.708 |
| ENSP00000360525 | 1.99E-05 | -0.1239 | 996 | 0.892 |
| ENSP00000339047 | 7.37E-06 | -0.1239 | 0 | 0.464 |
| ENSP00000351967 | 6.01E-06 | -0.1240 | 0 | 0.000 |
| ENSP00000369424 | 2.77E-05 | -0.1240 | 201 | 0.926 |
| ENSP00000293276 | 1.28E-05 | -0.1240 | 0 | 0.000 |
| ENSP00000259324 | 6.05E-06 | -0.1241 | 0 | 0.598 |
| ENSP00000366275 | 7.62E-06 | -0.1241 | 189 | 0.107 |
| ENSP00000155926 | 3.98E-06 | -0.1241 | 205 | 0.680 |
| ENSP00000394085 | 2.55E-05 | -0.1241 | 214 | 0.759 |
| ENSP00000385470 | 5.28E-06 | -0.1242 | 0 | 0.488 |
| ENSP00000350789 | 2.14E-05 | -0.1242 | 225 | 0.000 |
| ENSP00000354522 | 2.55E-05 | -0.1242 | 195 | 0.830 |
| ENSP00000349459 | 4.54E-06 | -0.1242 | 0 | 0.071 |
| ENSP00000261839 | 1.19E-05 | -0.1242 | 434 | 0.531 |
| ENSP00000387982 | 1.97E-05 | -0.1242 | 200 | 0.933 |
| ENSP00000350098 | 4.76E-06 | -0.1242 | 0 | 0.535 |
| ENSP00000322791 | 5.24E-06 | -0.1242 | 0 | 0.224 |
| ENSP00000261427 | 8.79E-06 | -0.1243 | 242 | 0.725 |
| ENSP00000346879 | 2.26E-05 | -0.1243 | 230 | 0.883 |
| ENSP00000260731 | 4.45E-06 | -0.1243 | 0 | 0.426 |
| ENSP00000339001 | 2.65E-05 | -0.1243 | 434 | 0.653 |
| ENSP00000332194 | 1.39E-05 | -0.1243 | 216 | 0.000 |
| ENSP00000318753 | 7.44E-06 | -0.1243 | 0 | 0.150 |
| ENSP00000260983 | 6.78E-06 | -0.1243 | 0 | 0.500 |
| ENSP00000413929 | 4.47E-06 | -0.1244 | 0 | 0.703 |
| ENSP00000348010 | 2.00E-05 | -0.1244 | 474 | 0.704 |
| ENSP00000392330 | 1.98E-05 | -0.1244 | 170 | 0.179 |
| ENSP00000360608 | 2.30E-05 | -0.1244 | 200 | 0.691 |
| ENSP00000423567 | 2.41E-06 | -0.1244 | 0 | 0.116 |
| ENSP00000350275 | 1.48E-05 | -0.1244 | 911 | 0.000 |
| ENSP00000364978 | 1.21E-06 | -0.1244 | 0 | 0.175 |
| ENSP00000339764 | 3.69E-06 | -0.1244 | 0 | 0.736 |
| ENSP00000359693 | 1.92E-05 | -0.1244 | 284 | 0.796 |
| ENSP00000455169 | 8.62E-06 | -0.1244 | 0 | 0.049 |

| | | | | |
|---|---|---|---|---|
| ENSP00000293525 | 1.50E-06 | -0.1245 | 0 | 0.000 |
| ENSP00000357597 | 2.09E-05 | -0.1245 | 153 | 0.000 |
| ENSP00000356022 | 3.17E-05 | -0.1245 | 408 | 0.670 |
| ENSP00000265074 | 3.31E-06 | -0.1245 | 0 | 0.663 |
| ENSP00000346886 | 5.22E-06 | -0.1245 | 0 | 0.762 |
| ENSP00000283195 | 1.62E-05 | -0.1245 | 426 | 0.000 |
| ENSP00000377769 | 2.52E-05 | -0.1245 | 316 | 0.211 |
| ENSP00000319169 | 1.49E-05 | -0.1245 | 204 | 0.873 |
| ENSP00000364289 | 2.16E-05 | -0.1246 | 427 | 0.903 |
| ENSP00000365395 | 1.55E-05 | -0.1246 | 151 | 0.149 |
| ENSP00000417492 | 3.80E-06 | -0.1246 | 0 | 0.139 |
| ENSP00000313582 | 3.74E-06 | -0.1246 | 0 | 0.726 |
| ENSP00000334430 | 3.44E-06 | -0.1246 | 0 | 0.155 |
| ENSP00000352233 | 3.13E-06 | -0.1246 | 0 | 0.686 |
| ENSP00000417175 | 3.55E-05 | -0.1246 | 197 | 0.331 |
| ENSP00000418748 | 6.17E-06 | -0.1246 | 0 | 0.308 |
| ENSP00000347140 | 9.35E-06 | -0.1246 | 681 | 0.117 |
| ENSP00000363322 | 5.17E-06 | -0.1246 | 0 | 0.425 |
| ENSP00000345064 | 2.03E-05 | -0.1247 | 335 | 0.698 |
| ENSP00000350011 | 4.05E-06 | -0.1247 | 0 | 0.651 |
| ENSP00000326272 | 8.88E-06 | -0.1247 | 202 | 0.000 |
| ENSP00000327179 | 1.41E-05 | -0.1247 | 204 | 0.853 |
| ENSP00000313007 | 1.67E-05 | -0.1247 | 672 | 0.917 |
| ENSP00000382218 | 7.75E-06 | -0.1247 | 573 | 0.364 |
| ENSP00000302530 | 8.00E-06 | -0.1247 | 198 | 0.000 |
| ENSP00000281830 | 3.55E-06 | -0.1247 | 0 | 0.111 |
| ENSP00000353074 | 1.97E-05 | -0.1248 | 901 | 0.949 |
| ENSP00000351777 | 1.87E-05 | -0.1248 | 798 | 0.761 |
| ENSP00000370669 | 8.03E-06 | -0.1248 | 279 | 0.141 |
| ENSP00000327255 | 1.02E-05 | -0.1248 | 173 | 0.773 |
| ENSP00000364802 | 2.97E-05 | -0.1248 | 271 | 0.897 |
| ENSP00000386280 | 3.15E-06 | -0.1248 | 0 | 0.082 |
| ENSP00000371126 | 2.41E-06 | -0.1248 | 0 | 0.412 |
| ENSP00000380920 | 4.86E-07 | -0.1249 | 0 | 0.169 |
| ENSP00000319474 | 1.02E-05 | -0.1249 | 242 | 0.000 |
| ENSP00000333012 | 4.63E-06 | -0.1249 | 0 | 0.595 |
| ENSP00000389630 | 4.28E-06 | -0.1249 | 0 | 0.072 |
| ENSP00000265709 | 8.67E-06 | -0.1249 | 998 | 0.682 |
| ENSP00000370151 | 9.38E-06 | -0.1249 | 201 | 0.756 |
| ENSP00000361811 | 8.73E-07 | -0.1251 | 0 | 0.180 |
| ENSP00000337451 | 1.42E-05 | -0.1251 | 240 | 0.670 |
| ENSP00000387361 | 1.08E-05 | -0.1251 | 0 | 0.173 |
| ENSP00000356853 | 4.54E-06 | -0.1251 | 0 | 0.325 |
| ENSP00000291295 | 5.16E-06 | -0.1251 | 153 | 0.000 |
| ENSP00000433343 | 2.14E-06 | -0.1251 | 0 | 0.121 |
| ENSP00000347532 | 3.19E-06 | -0.1251 | 0 | 0.000 |

| | | | | |
|---|---|---|---|---|
| ENSP00000410734 | 3.19E-06 | -0.1251 | 0 | 0.697 |
| ENSP00000361219 | 4.19E-06 | -0.1251 | 0 | 0.669 |
| ENSP00000311005 | 1.99E-05 | -0.1251 | 846 | 0.680 |
| ENSP00000252797 | 6.07E-06 | -0.1251 | 0 | 0.692 |
| ENSP00000369351 | 1.48E-05 | -0.1252 | 181 | 0.786 |
| ENSP00000316940 | 1.13E-05 | -0.1252 | 0 | 0.187 |
| ENSP00000248996 | 8.25E-06 | -0.1252 | 508 | 0.000 |
| ENSP00000428966 | 3.50E-06 | -0.1252 | 0 | 0.000 |
| ENSP00000300101 | 5.87E-06 | -0.1252 | 0 | 0.683 |
| ENSP00000306637 | 3.21E-06 | -0.1253 | 0 | 0.711 |
| ENSP00000341139 | 6.17E-07 | -0.1253 | 0 | 0.205 |
| ENSP00000260600 | 8.55E-06 | -0.1253 | 457 | 0.648 |
| ENSP00000262608 | 7.96E-06 | -0.1253 | 400 | 0.000 |
| ENSP00000375108 | 5.19E-07 | -0.1253 | 0 | 0.186 |
| ENSP00000304355 | 2.03E-05 | -0.1253 | 191 | 0.110 |
| ENSP00000346729 | 3.62E-06 | -0.1253 | 0 | 0.692 |
| ENSP00000463741 | 2.30E-06 | -0.1253 | 0 | 0.694 |
| ENSP00000376500 | 2.04E-05 | -0.1254 | 196 | 0.847 |
| ENSP00000389182 | 5.56E-06 | -0.1254 | 0 | 0.630 |
| ENSP00000295756 | 1.19E-05 | -0.1254 | 392 | 0.641 |
| ENSP00000341658 | 7.96E-06 | -0.1254 | 0 | 0.651 |
| ENSP00000264951 | 8.40E-06 | -0.1254 | 419 | 0.000 |
| ENSP00000358160 | 2.23E-05 | -0.1254 | 808 | 0.931 |
| ENSP00000381504 | 6.62E-06 | -0.1255 | 241 | 0.656 |
| ENSP00000340176 | 2.78E-05 | -0.1255 | 412 | 0.375 |
| ENSP00000451320 | 2.58E-05 | -0.1255 | 214 | 0.720 |
| ENSP00000297338 | 4.80E-06 | -0.1255 | 0 | 0.729 |
| ENSP00000378368 | 5.51E-06 | -0.1255 | 0 | 0.252 |
| ENSP00000401721 | 1.34E-05 | -0.1256 | 432 | 0.896 |
| ENSP00000353741 | 2.20E-05 | -0.1256 | 321 | 0.875 |
| ENSP00000372136 | 2.20E-06 | -0.1256 | 0 | 0.143 |
| ENSP00000263666 | 1.76E-05 | -0.1256 | 200 | 0.544 |
| ENSP00000351047 | 3.52E-06 | -0.1256 | 0 | 0.105 |
| ENSP00000263895 | 1.02E-05 | -0.1256 | 317 | 0.590 |
| ENSP00000311113 | 2.58E-05 | -0.1256 | 721 | 0.871 |
| ENSP00000367069 | 4.56E-06 | -0.1257 | 0 | 0.718 |
| ENSP00000452037 | 1.27E-05 | -0.1257 | 165 | 0.472 |
| ENSP00000217188 | 4.66E-06 | -0.1257 | 205 | 0.679 |
| ENSP00000379372 | 4.04E-06 | -0.1257 | 0 | 0.703 |
| ENSP00000334836 | 1.53E-05 | -0.1258 | 267 | 0.669 |
| ENSP00000393292 | 3.70E-06 | -0.1258 | 0 | 0.113 |
| ENSP00000304065 | 6.48E-08 | -0.1258 | 0 | 0.000 |
| ENSP00000348815 | 1.14E-05 | -0.1258 | 191 | 0.172 |
| ENSP00000335285 | 2.06E-06 | -0.1258 | 0 | 0.185 |
| ENSP00000416959 | 1.23E-05 | -0.1258 | 228 | 0.765 |
| ENSP00000349594 | 1.99E-05 | -0.1258 | 264 | 0.843 |

| | | | | |
|---|---|---|---|---|
| ENSP00000263095 | 6.32E-06 | -0.1258 | 0 | 0.692 |
| ENSP00000387907 | 1.52E-06 | -0.1259 | 0 | 0.131 |
| ENSP00000358165 | 4.47E-06 | -0.1259 | 0 | 0.181 |
| ENSP00000431287 | 1.06E-06 | -0.1259 | 0 | 0.238 |
| ENSP00000382342 | 1.39E-05 | -0.1259 | 183 | 0.508 |
| ENSP00000344071 | 5.95E-06 | -0.1260 | 0 | 0.198 |
| ENSP00000265562 | 5.86E-06 | -0.1260 | 229 | 0.659 |
| ENSP00000431198 | 1.83E-06 | -0.1260 | 0 | 0.000 |
| ENSP00000387176 | 3.95E-05 | -0.1260 | 526 | 0.000 |
| ENSP00000339845 | 1.62E-05 | -0.1260 | 342 | 0.728 |
| ENSP00000337746 | 1.04E-05 | -0.1260 | 158 | 0.000 |
| ENSP00000322957 | 8.77E-06 | -0.1261 | 151 | 0.664 |
| ENSP00000298386 | 8.78E-06 | -0.1261 | 198 | 0.679 |
| ENSP00000322545 | 6.57E-06 | -0.1262 | 0 | 0.691 |
| ENSP00000375726 | 3.58E-06 | -0.1262 | 0 | 0.000 |
| ENSP00000324172 | 4.72E-06 | -0.1262 | 0 | 0.623 |
| ENSP00000318297 | 1.39E-05 | -0.1262 | 303 | 0.856 |
| ENSP00000421364 | 1.10E-05 | -0.1262 | 0 | 0.000 |
| ENSP00000282538 | 9.17E-06 | -0.1262 | 0 | 0.180 |
| ENSP00000369391 | 4.49E-06 | -0.1262 | 0 | 0.277 |
| ENSP00000354856 | 1.77E-05 | -0.1262 | 254 | 0.679 |
| ENSP00000382507 | 1.88E-05 | -0.1263 | 201 | 0.171 |
| ENSP00000418529 | 1.27E-05 | -0.1263 | 162 | 0.179 |
| ENSP00000338217 | 6.12E-06 | -0.1263 | 0 | 0.677 |
| ENSP00000380999 | 2.86E-06 | -0.1263 | 0 | 0.678 |
| ENSP00000378181 | 3.66E-06 | -0.1264 | 0 | 0.703 |
| ENSP00000398824 | 5.02E-06 | -0.1264 | 0 | 0.826 |
| ENSP00000268057 | 8.30E-06 | -0.1265 | 359 | 0.700 |
| ENSP00000304226 | 6.42E-06 | -0.1265 | 189 | 0.680 |
| ENSP00000389951 | 1.71E-05 | -0.1266 | 208 | 0.000 |
| ENSP00000352668 | 4.18E-06 | -0.1266 | 0 | 0.119 |
| ENSP00000264903 | 8.78E-06 | -0.1266 | 250 | 0.000 |
| ENSP00000270590 | 4.15E-05 | -0.1266 | 0 | 0.161 |
| ENSP00000344818 | 1.54E-01 | -0.1266 | 999 | 0.000 |
| ENSP00000339449 | 8.96E-06 | -0.1266 | 242 | 0.000 |
| ENSP00000414920 | 3.79E-06 | -0.1266 | 0 | 0.091 |
| ENSP00000465676 | 1.96E-05 | -0.1267 | 373 | 0.815 |
| ENSP00000381840 | 5.81E-06 | -0.1267 | 0 | 0.685 |
| ENSP00000383270 | 3.90E-06 | -0.1268 | 0 | 0.153 |
| ENSP00000414516 | 3.59E-06 | -0.1268 | 0 | 0.628 |
| ENSP00000292644 | 8.14E-06 | -0.1268 | 260 | 0.538 |
| ENSP00000459789 | 5.39E-06 | -0.1268 | 0 | 0.000 |
| ENSP00000400175 | 5.19E-05 | -0.1268 | 958 | 0.794 |
| ENSP00000423134 | 1.90E-06 | -0.1268 | 0 | 0.000 |
| ENSP00000343488 | 7.28E-06 | -0.1268 | 315 | 0.000 |
| ENSP00000332528 | 1.75E-05 | -0.1269 | 161 | 0.127 |

| | | | | |
|---|---|---|---|---|
| ENSP00000408697 | 2.74E-06 | -0.1269 | 0 | 0.111 |
| ENSP00000254963 | 7.93E-06 | -0.1269 | 195 | 0.771 |
| ENSP00000337320 | 6.63E-06 | -0.1269 | 0 | 0.685 |
| ENSP00000263800 | 1.85E-05 | -0.1269 | 210 | 0.814 |
| ENSP00000280772 | 9.96E-06 | -0.1270 | 201 | 0.671 |
| ENSP00000436604 | 4.30E-06 | -0.1271 | 0 | 0.100 |
| ENSP00000339238 | 7.82E-07 | -0.1271 | 0 | 0.199 |
| ENSP00000276014 | 3.62E-06 | -0.1271 | 0 | 0.648 |
| ENSP00000370047 | 5.25E-06 | -0.1271 | 0 | 0.076 |
| ENSP00000370867 | 1.93E-05 | -0.1272 | 261 | 0.546 |
| ENSP00000340683 | 4.16E-06 | -0.1272 | 0 | 0.684 |
| ENSP00000455911 | 3.05E-06 | -0.1272 | 0 | 0.693 |
| ENSP00000399078 | 2.91E-06 | -0.1272 | 0 | 0.000 |
| ENSP00000262752 | 6.19E-06 | -0.1273 | 289 | 0.684 |
| ENSP00000342510 | 5.11E-06 | -0.1273 | 0 | 0.395 |
| ENSP00000265713 | 9.76E-06 | -0.1273 | 320 | 0.000 |
| ENSP00000270112 | 4.28E-06 | -0.1273 | 205 | 0.676 |
| ENSP00000266743 | 4.55E-06 | -0.1273 | 0 | 0.369 |
| ENSP00000339030 | 5.12E-06 | -0.1274 | 0 | 0.701 |
| ENSP00000384832 | 1.22E-06 | -0.1274 | 0 | 0.165 |
| ENSP00000367570 | 1.87E-05 | -0.1274 | 244 | 0.903 |
| ENSP00000217185 | 4.59E-06 | -0.1274 | 205 | 0.684 |
| ENSP00000412189 | 1.52E-05 | -0.1274 | 257 | 0.468 |
| ENSP00000350267 | 9.00E-06 | -0.1274 | 430 | 0.000 |
| ENSP00000341289 | 1.99E-05 | -0.1275 | 476 | 0.626 |
| ENSP00000355920 | 2.24E-05 | -0.1275 | 160 | 0.077 |
| ENSP00000357149 | 8.65E-06 | -0.1275 | 200 | 0.185 |
| ENSP00000385432 | 8.44E-06 | -0.1275 | 923 | 0.218 |
| ENSP00000347948 | 1.33E-05 | -0.1275 | 290 | 0.685 |
| ENSP00000356912 | 1.61E-06 | -0.1275 | 0 | 0.128 |
| ENSP00000433427 | 1.40E-05 | -0.1276 | 204 | 0.204 |
| ENSP00000430073 | 3.25E-07 | -0.1277 | 0 | 0.194 |
| ENSP00000325525 | 3.18E-05 | -0.1277 | 0 | 0.000 |
| ENSP00000361918 | 1.79E-05 | -0.1277 | 482 | 0.900 |
| ENSP00000315743 | 7.93E-06 | -0.1277 | 150 | 0.691 |
| ENSP00000413152 | 9.03E-06 | -0.1277 | 282 | 0.418 |
| ENSP00000317310 | 1.17E-05 | -0.1277 | 290 | 0.000 |
| ENSP00000341826 | 1.77E-05 | -0.1278 | 491 | 0.000 |
| ENSP00000280325 | 3.46E-06 | -0.1278 | 0 | 0.174 |
| ENSP00000371236 | 4.21E-05 | -0.1278 | 605 | 0.659 |
| ENSP00000418401 | 1.50E-05 | -0.1278 | 907 | 0.473 |
| ENSP00000470609 | 1.38E-05 | -0.1278 | 644 | 0.182 |
| ENSP00000366135 | 8.69E-06 | -0.1279 | 225 | 0.893 |
| ENSP00000248975 | 8.34E-06 | -0.1280 | 333 | 0.678 |
| ENSP00000465517 | 1.51E-06 | -0.1280 | 0 | 0.131 |
| ENSP00000373278 | 1.16E-06 | -0.1280 | 0 | 0.173 |

| | | | | |
|---|---|---|---|---|
| ENSP00000311684 | 1.13E-05 | -0.1280 | 441 | 0.661 |
| ENSP00000332111 | 3.77E-06 | -0.1280 | 153 | 0.000 |
| ENSP00000352941 | 8.54E-06 | -0.1281 | 0 | 0.090 |
| ENSP00000437142 | 6.09E-06 | -0.1281 | 191 | 0.000 |
| ENSP00000386722 | 5.84E-06 | -0.1281 | 299 | 0.120 |
| ENSP00000460571 | 1.65E-06 | -0.1281 | 0 | 0.000 |
| ENSP00000255304 | 4.93E-06 | -0.1282 | 0 | 0.143 |
| ENSP00000351717 | 6.79E-06 | -0.1282 | 160 | 0.378 |
| ENSP00000416707 | 3.93E-06 | -0.1282 | 0 | 0.095 |
| ENSP00000355245 | 5.56E-06 | -0.1282 | 0 | 0.855 |
| ENSP00000456401 | 1.87E-06 | -0.1282 | 0 | 0.107 |
| ENSP00000383115 | 2.17E-05 | -0.1282 | 0 | 0.069 |
| ENSP00000371607 | 1.24E-05 | -0.1283 | 517 | 0.130 |
| ENSP00000411439 | 4.05E-06 | -0.1283 | 0 | 0.092 |
| ENSP00000352498 | 1.86E-05 | -0.1284 | 554 | 0.000 |
| ENSP00000453403 | 2.99E-06 | -0.1284 | 0 | 0.387 |
| ENSP00000275300 | 7.23E-06 | -0.1284 | 0 | 0.059 |
| ENSP00000361603 | 1.06E-05 | -0.1284 | 0 | 0.112 |
| ENSP00000346890 | 1.66E-05 | -0.1284 | 242 | 0.185 |
| ENSP00000296122 | 1.02E-05 | -0.1284 | 194 | 0.545 |
| ENSP00000363734 | 3.81E-06 | -0.1284 | 0 | 0.442 |
| ENSP00000296776 | 1.82E-05 | -0.1285 | 0 | 0.214 |
| ENSP00000326981 | 4.89E-06 | -0.1285 | 399 | 0.897 |
| ENSP00000216101 | 3.84E-06 | -0.1285 | 448 | 0.598 |
| ENSP00000394791 | 7.28E-06 | -0.1285 | 163 | 0.743 |
| ENSP00000255465 | 7.56E-06 | -0.1286 | 211 | 0.716 |
| ENSP00000362353 | 2.27E-05 | -0.1286 | 365 | 0.700 |
| ENSP00000384996 | 2.01E-06 | -0.1286 | 0 | 0.094 |
| ENSP00000472120 | 1.28E-06 | -0.1286 | 0 | 0.000 |
| ENSP00000368005 | 3.93E-06 | -0.1286 | 0 | 0.617 |
| ENSP00000359299 | 4.25E-06 | -0.1286 | 0 | 0.101 |
| ENSP00000396192 | 2.47E-06 | -0.1287 | 0 | 0.000 |
| ENSP00000253055 | 4.40E-06 | -0.1287 | 205 | 0.686 |
| ENSP00000359740 | 3.99E-06 | -0.1287 | 0 | 0.193 |
| ENSP00000349708 | 3.62E-06 | -0.1287 | 0 | 0.238 |
| ENSP00000323183 | 5.69E-06 | -0.1287 | 0 | 0.697 |
| ENSP00000354541 | 4.99E-06 | -0.1287 | 0 | 0.216 |
| ENSP00000326240 | 5.80E-06 | -0.1287 | 0 | 0.695 |
| ENSP00000364110 | 6.04E-06 | -0.1288 | 0 | 0.171 |
| ENSP00000380157 | 1.86E-05 | -0.1288 | 198 | 0.222 |
| ENSP00000366032 | 8.88E-06 | -0.1288 | 0 | 0.653 |
| ENSP00000393316 | 8.97E-06 | -0.1288 | 165 | 0.139 |
| ENSP00000238738 | 7.59E-06 | -0.1288 | 204 | 0.563 |
| ENSP00000362261 | 7.98E-06 | -0.1288 | 0 | 0.158 |
| ENSP00000437635 | 9.13E-07 | -0.1289 | 0 | 0.174 |
| ENSP00000364691 | 1.40E-05 | -0.1289 | 267 | 0.503 |

| | | | | |
|---|---|---|---|---|
| ENSP00000285667 | 1.07E-05 | -0.1289 | 195 | 0.797 |
| ENSP00000338573 | 1.34E-05 | -0.1289 | 264 | 0.000 |
| ENSP00000385636 | 7.54E-06 | -0.1289 | 0 | 0.199 |
| ENSP00000353249 | 1.64E-05 | -0.1289 | 152 | 0.082 |
| ENSP00000306124 | 1.18E-05 | -0.1290 | 906 | 0.666 |
| ENSP00000373627 | 4.12E-06 | -0.1290 | 0 | 0.325 |
| ENSP00000338127 | 4.83E-06 | -0.1290 | 205 | 0.677 |
| ENSP00000313504 | 5.06E-06 | -0.1290 | 0 | 0.203 |
| ENSP00000384573 | 1.40E-05 | -0.1290 | 0 | 0.958 |
| ENSP00000404029 | 2.96E-05 | -0.1291 | 429 | 0.807 |
| ENSP00000297565 | 5.72E-06 | -0.1291 | 0 | 0.807 |
| ENSP00000349296 | 7.76E-06 | -0.1291 | 0 | 0.153 |
| ENSP00000279387 | 7.58E-06 | -0.1291 | 224 | 0.694 |
| ENSP00000468991 | 2.18E-05 | -0.1292 | 213 | 0.510 |
| ENSP00000353643 | 1.98E-05 | -0.1292 | 339 | 0.946 |
| ENSP00000366356 | 1.03E-05 | -0.1292 | 0 | 0.000 |
| ENSP00000473047 | 1.66E-05 | -0.1292 | 472 | 0.205 |
| ENSP00000217233 | 6.55E-06 | -0.1292 | 574 | 0.689 |
| ENSP00000347730 | 3.30E-06 | -0.1292 | 0 | 0.693 |
| ENSP00000373572 | 3.15E-06 | -0.1292 | 0 | 0.000 |
| ENSP00000386485 | 7.15E-06 | -0.1293 | 167 | 0.058 |
| ENSP00000368007 | 5.26E-06 | -0.1293 | 0 | 0.150 |
| ENSP00000335544 | 1.76E-05 | -0.1293 | 179 | 0.657 |
| ENSP00000430242 | 7.81E-06 | -0.1293 | 0 | 0.092 |
| ENSP00000351380 | 3.42E-06 | -0.1293 | 0 | 0.112 |
| ENSP00000342114 | 2.26E-05 | -0.1293 | 362 | 0.192 |
| ENSP00000295757 | 6.41E-06 | -0.1294 | 279 | 0.000 |
| ENSP00000390600 | 1.50E-05 | -0.1294 | 171 | 0.575 |
| ENSP00000350547 | 7.61E-06 | -0.1294 | 0 | 0.593 |
| ENSP00000361104 | 2.51E-06 | -0.1294 | 0 | 0.189 |
| ENSP00000356056 | 8.90E-06 | -0.1294 | 451 | 0.253 |
| ENSP00000428864 | 1.56E-05 | -0.1294 | 304 | 0.166 |
| ENSP00000354251 | 1.01E-05 | -0.1295 | 224 | 0.428 |
| ENSP00000360141 | 3.14E-05 | -0.1295 | 306 | 0.849 |
| ENSP00000394390 | 6.94E-06 | -0.1295 | 195 | 0.131 |
| ENSP00000376583 | 7.48E-06 | -0.1295 | 304 | 0.176 |
| ENSP00000368362 | 1.60E-05 | -0.1295 | 202 | 0.131 |
| ENSP00000393845 | 9.29E-06 | -0.1295 | 272 | 0.458 |
| ENSP00000418593 | 4.02E-06 | -0.1295 | 0 | 0.128 |
| ENSP00000273854 | 7.20E-06 | -0.1295 | 205 | 0.670 |
| ENSP00000332723 | 1.86E-05 | -0.1295 | 0 | 0.113 |
| ENSP00000455385 | 6.46E-08 | -0.1295 | 0 | 0.000 |
| ENSP00000381220 | 2.55E-06 | -0.1296 | 0 | 0.097 |
| ENSP00000264674 | 5.23E-06 | -0.1296 | 0 | 0.804 |
| ENSP00000406229 | 2.26E-05 | -0.1296 | 169 | 0.810 |
| ENSP00000317691 | 4.88E-06 | -0.1296 | 0 | 0.427 |

| | | | | |
|---|---|---|---|---|
| ENSP00000293190 | 4.37E-06 | -0.1296 | 0 | 0.536 |
| ENSP00000343633 | 4.73E-06 | -0.1296 | 0 | 0.000 |
| ENSP00000421592 | 3.17E-05 | -0.1296 | 422 | 0.737 |
| ENSP00000373730 | 8.18E-06 | -0.1296 | 158 | 0.989 |
| ENSP00000363390 | 1.52E-05 | -0.1297 | 158 | 0.834 |
| ENSP00000291860 | 1.69E-05 | -0.1297 | 0 | 0.254 |
| ENSP00000383682 | 5.79E-08 | -0.1297 | 0 | 0.215 |
| ENSP00000453019 | 1.91E-06 | -0.1298 | 0 | 0.318 |
| ENSP00000335618 | 4.89E-06 | -0.1298 | 0 | 0.000 |
| ENSP00000367197 | 2.68E-05 | -0.1298 | 195 | 0.659 |
| ENSP00000449751 | 9.57E-06 | -0.1298 | 277 | 0.435 |
| ENSP00000403954 | 2.23E-05 | -0.1298 | 198 | 0.270 |
| ENSP00000416293 | 3.36E-05 | -0.1298 | 211 | 0.717 |
| ENSP00000331268 | 1.11E-05 | -0.1298 | 251 | 0.750 |
| ENSP00000408910 | 2.41E-05 | -0.1298 | 270 | 0.228 |
| ENSP00000407546 | 2.37E-05 | -0.1299 | 258 | 0.663 |
| ENSP00000324856 | 2.04E-05 | -0.1299 | 329 | 0.700 |
| ENSP00000314023 | 5.23E-06 | -0.1299 | 0 | 0.148 |
| ENSP00000358590 | 3.15E-06 | -0.1299 | 0 | 0.702 |
| ENSP00000348211 | 5.18E-06 | -0.1299 | 0 | 0.133 |
| ENSP00000354608 | 1.53E-05 | -0.1299 | 159 | 0.092 |
| ENSP00000371643 | 1.45E-06 | -0.1300 | 0 | 0.131 |
| ENSP00000312001 | 5.01E-06 | -0.1300 | 0 | 0.617 |
| ENSP00000325748 | 1.57E-05 | -0.1300 | 520 | 0.651 |
| ENSP00000361310 | 8.24E-06 | -0.1300 | 323 | 0.737 |
| ENSP00000389399 | 7.59E-06 | -0.1301 | 0 | 0.057 |
| ENSP00000428756 | 3.73E-06 | -0.1301 | 0 | 0.501 |
| ENSP00000329141 | 4.81E-06 | -0.1301 | 0 | 0.686 |
| ENSP00000439189 | 4.12E-05 | -0.1301 | 526 | 0.000 |
| ENSP00000403712 | 1.20E-05 | -0.1301 | 242 | 0.741 |
| ENSP00000417291 | 1.55E-05 | -0.1302 | 0 | 0.657 |
| ENSP00000446680 | 5.76E-06 | -0.1302 | 0 | 0.765 |
| ENSP00000321927 | 3.84E-06 | -0.1302 | 0 | 0.213 |
| ENSP00000262150 | 2.71E-05 | -0.1302 | 0 | 0.076 |
| ENSP00000362688 | 1.58E-05 | -0.1302 | 211 | 0.864 |
| ENSP00000354610 | 1.86E-05 | -0.1302 | 156 | 0.450 |
| ENSP00000365462 | 1.71E-05 | -0.1302 | 260 | 0.491 |
| ENSP00000370343 | 1.78E-05 | -0.1303 | 904 | 0.833 |
| ENSP00000380514 | 8.47E-06 | -0.1303 | 173 | 0.000 |
| ENSP00000335632 | 4.53E-06 | -0.1303 | 0 | 0.000 |
| ENSP00000345445 | 1.52E-05 | -0.1303 | 301 | 0.232 |
| ENSP00000392718 | 3.99E-05 | -0.1303 | 526 | 0.000 |
| ENSP00000396774 | 1.45E-05 | -0.1304 | 196 | 0.239 |
| ENSP00000356489 | 2.56E-05 | -0.1304 | 570 | 0.775 |
| ENSP00000342162 | 8.38E-06 | -0.1304 | 0 | 0.147 |
| ENSP00000394734 | 1.71E-05 | -0.1305 | 254 | 0.714 |

| | | | | |
|---|---|---|---|---|
| ENSP00000383639 | 2.93E-06 | -0.1305 | 0 | 0.000 |
| ENSP00000319597 | 5.14E-06 | -0.1305 | 0 | 0.497 |
| ENSP00000364260 | 5.30E-06 | -0.1305 | 0 | 0.000 |
| ENSP00000315295 | 2.91E-06 | -0.1305 | 0 | 0.172 |
| ENSP00000360918 | 1.89E-05 | -0.1305 | 301 | 0.357 |
| ENSP00000365145 | 2.91E-06 | -0.1306 | 0 | 0.000 |
| ENSP00000322582 | 1.20E-05 | -0.1306 | 0 | 0.151 |
| ENSP00000450607 | 1.38E-05 | -0.1306 | 235 | 0.705 |
| ENSP00000384109 | 1.98E-05 | -0.1306 | 203 | 0.735 |
| ENSP00000419628 | 7.01E-06 | -0.1307 | 195 | 0.371 |
| ENSP00000338812 | 4.19E-06 | -0.1307 | 0 | 0.132 |
| ENSP00000365776 | 1.84E-06 | -0.1307 | 0 | 0.000 |
| ENSP00000405738 | 1.31E-05 | -0.1307 | 161 | 0.258 |
| ENSP00000373191 | 4.96E-06 | -0.1307 | 0 | 0.126 |
| ENSP00000374359 | 3.78E-06 | -0.1307 | 0 | 0.682 |
| ENSP00000413625 | 6.07E-06 | -0.1307 | 0 | 0.445 |
| ENSP00000384742 | 4.75E-06 | -0.1307 | 0 | 0.000 |
| ENSP00000358997 | 1.73E-05 | -0.1307 | 205 | 0.691 |
| ENSP00000467024 | 4.79E-05 | -0.1308 | 300 | 0.787 |
| ENSP00000310701 | 1.30E-05 | -0.1308 | 914 | 0.000 |
| ENSP00000394936 | 1.28E-05 | -0.1308 | 150 | 0.347 |
| ENSP00000264414 | 8.56E-06 | -0.1308 | 198 | 0.645 |
| ENSP00000356234 | 8.23E-06 | -0.1308 | 551 | 0.000 |
| ENSP00000397453 | 1.91E-05 | -0.1309 | 178 | 0.588 |
| ENSP00000448059 | 8.02E-06 | -0.1309 | 211 | 0.328 |
| ENSP00000364361 | 1.08E-05 | -0.1309 | 210 | 0.000 |
| ENSP00000362744 | 1.89E-05 | -0.1309 | 219 | 0.855 |
| ENSP00000389381 | 5.16E-06 | -0.1310 | 527 | 0.760 |
| ENSP00000378152 | 2.12E-05 | -0.1310 | 161 | 0.309 |
| ENSP00000365272 | 1.45E-05 | -0.1310 | 308 | 0.511 |
| ENSP00000421922 | 1.97E-05 | -0.1310 | 242 | 0.639 |
| ENSP00000273320 | 5.18E-06 | -0.1310 | 0 | 0.743 |
| ENSP00000371170 | 7.13E-06 | -0.1311 | 0 | 0.000 |
| ENSP00000426996 | 2.31E-08 | -0.1311 | 0 | 0.000 |
| ENSP00000422769 | 2.31E-08 | -0.1311 | 0 | 0.212 |
| ENSP00000337386 | 2.55E-05 | -0.1311 | 223 | 0.605 |
| ENSP00000372005 | 1.21E-05 | -0.1312 | 895 | 0.227 |
| ENSP00000347195 | 1.08E-05 | -0.1312 | 0 | 0.153 |
| ENSP00000435926 | 1.09E-05 | -0.1312 | 177 | 0.626 |
| ENSP00000376611 | 5.49E-06 | -0.1312 | 0 | 0.733 |
| ENSP00000421917 | 4.46E-06 | -0.1312 | 0 | 0.139 |
| ENSP00000443339 | 8.61E-08 | -0.1313 | 0 | 0.213 |
| ENSP00000005180 | 1.93E-05 | -0.1313 | 0 | 0.176 |
| ENSP00000469886 | 8.89E-07 | -0.1313 | 0 | 0.164 |
| ENSP00000340995 | 4.84E-06 | -0.1313 | 0 | 0.291 |
| ENSP00000462116 | 3.47E-06 | -0.1313 | 0 | 0.616 |

| | | | | |
|---|---|---|---|---|
| ENSP00000357384 | 4.88E-06 | -0.1313 | 0 | 0.070 |
| ENSP00000356723 | 4.26E-06 | -0.1314 | 0 | 0.087 |
| ENSP00000381115 | 1.65E-06 | -0.1314 | 0 | 0.000 |
| ENSP00000370150 | 3.51E-06 | -0.1314 | 0 | 0.469 |
| ENSP00000358894 | 7.84E-06 | -0.1314 | 189 | 0.111 |
| ENSP00000330381 | 8.15E-06 | -0.1314 | 242 | 0.000 |
| ENSP00000358932 | 2.58E-06 | -0.1315 | 0 | 0.169 |
| ENSP00000381270 | 4.34E-06 | -0.1315 | 0 | 0.000 |
| ENSP00000293201 | 7.58E-06 | -0.1315 | 0 | 0.000 |
| ENSP00000316482 | 3.29E-05 | -0.1315 | 0 | 0.069 |
| ENSP00000287239 | 9.04E-06 | -0.1315 | 320 | 0.000 |
| ENSP00000420914 | 8.06E-06 | -0.1316 | 212 | 0.398 |
| ENSP00000311816 | 5.19E-06 | -0.1316 | 0 | 0.817 |
| ENSP00000383520 | 2.93E-06 | -0.1317 | 0 | 0.000 |
| ENSP00000359198 | 1.21E-06 | -0.1317 | 0 | 0.203 |
| ENSP00000419944 | 2.89E-06 | -0.1317 | 0 | 0.106 |
| ENSP00000366006 | 1.88E-05 | -0.1317 | 847 | 0.115 |
| ENSP00000403987 | 2.85E-06 | -0.1317 | 0 | 0.125 |
| ENSP00000427428 | 1.25E-06 | -0.1317 | 0 | 0.161 |
| ENSP00000284245 | 1.08E-05 | -0.1317 | 0 | 0.145 |
| ENSP00000226230 | 9.91E-06 | -0.1317 | 0 | 0.402 |
| ENSP00000363747 | 4.53E-06 | -0.1317 | 0 | 0.697 |
| ENSP00000353586 | 7.05E-06 | -0.1317 | 0 | 0.000 |
| ENSP00000331681 | 4.15E-06 | -0.1317 | 0 | 0.157 |
| ENSP00000260967 | 8.21E-06 | -0.1318 | 221 | 0.000 |
| ENSP00000466433 | 1.85E-06 | -0.1318 | 0 | 0.000 |
| ENSP00000235307 | 1.13E-05 | -0.1318 | 0 | 0.187 |
| ENSP00000296098 | 7.64E-06 | -0.1319 | 0 | 0.184 |
| ENSP00000419952 | 4.70E-06 | -0.1319 | 0 | 0.168 |
| ENSP00000354573 | 2.37E-05 | -0.1320 | 361 | 0.541 |
| ENSP00000384286 | 1.26E-06 | -0.1320 | 0 | 0.138 |
| ENSP00000261556 | 2.41E-05 | -0.1320 | 0 | 0.177 |
| ENSP00000296786 | 2.76E-06 | -0.1320 | 0 | 0.701 |
| ENSP00000428316 | 5.77E-06 | -0.1320 | 0 | 0.279 |
| ENSP00000355077 | 2.96E-06 | -0.1320 | 0 | 0.105 |
| ENSP00000462754 | 1.78E-06 | -0.1320 | 0 | 0.000 |
| ENSP00000448051 | 6.34E-06 | -0.1320 | 0 | 0.233 |
| ENSP00000363040 | 5.34E-06 | -0.1321 | 0 | 0.135 |
| ENSP00000395723 | 3.68E-06 | -0.1321 | 0 | 0.689 |
| ENSP00000262916 | 1.74E-05 | -0.1322 | 167 | 0.278 |
| ENSP00000368887 | 1.44E-05 | -0.1322 | 156 | 0.861 |
| ENSP00000379514 | 1.89E-05 | -0.1322 | 274 | 0.570 |
| ENSP00000246794 | 2.24E-05 | -0.1322 | 0 | 0.185 |
| ENSP00000316628 | 5.87E-06 | -0.1323 | 0 | 0.102 |
| ENSP00000363124 | 1.95E-05 | -0.1323 | 412 | 0.491 |
| ENSP00000262719 | 8.63E-06 | -0.1323 | 235 | 0.698 |

| | | | | |
|---|---|---|---|---|
| ENSP00000373047 | 6.31E-07 | -0.1324 | 0 | 0.215 |
| ENSP00000440638 | 3.00E-06 | -0.1324 | 0 | 0.155 |
| ENSP00000418897 | 4.67E-06 | -0.1324 | 0 | 0.690 |
| ENSP00000366908 | 3.29E-06 | -0.1324 | 0 | 0.000 |
| ENSP00000364240 | 2.47E-06 | -0.1325 | 0 | 0.131 |
| ENSP00000385643 | 1.05E-06 | -0.1326 | 0 | 0.000 |
| ENSP00000471905 | 2.52E-06 | -0.1326 | 0 | 0.690 |
| ENSP00000305302 | 1.93E-05 | -0.1326 | 182 | 0.085 |
| ENSP00000381098 | 2.05E-05 | -0.1327 | 199 | 0.609 |
| ENSP00000419153 | 1.04E-05 | -0.1327 | 171 | 0.730 |
| ENSP00000467209 | 3.61E-07 | -0.1327 | 0 | 0.000 |
| ENSP00000379684 | 5.80E-06 | -0.1327 | 0 | 0.684 |
| ENSP00000383892 | 2.10E-05 | -0.1328 | 361 | 0.566 |
| ENSP00000298198 | 8.37E-06 | -0.1328 | 309 | 0.509 |
| ENSP00000283547 | 1.55E-05 | -0.1328 | 0 | 0.168 |
| ENSP00000368244 | 8.38E-06 | -0.1329 | 150 | 0.530 |
| ENSP00000371846 | 3.42E-06 | -0.1329 | 0 | 0.489 |
| ENSP00000414687 | 2.10E-06 | -0.1329 | 0 | 0.000 |
| ENSP00000455068 | 8.77E-06 | -0.1329 | 0 | 0.164 |
| ENSP00000322568 | 1.53E-05 | -0.1329 | 184 | 0.837 |
| ENSP00000361662 | 6.26E-06 | -0.1329 | 0 | 0.086 |
| ENSP00000346892 | 5.51E-06 | -0.1329 | 0 | 0.188 |
| ENSP00000361092 | 1.77E-05 | -0.1330 | 242 | 0.874 |
| ENSP00000392617 | 2.05E-05 | -0.1330 | 188 | 0.923 |
| ENSP00000364298 | 1.74E-05 | -0.1330 | 316 | 0.703 |
| ENSP00000356652 | 7.27E-06 | -0.1331 | 196 | 0.381 |
| ENSP00000425166 | 7.34E-06 | -0.1331 | 0 | 0.122 |
| ENSP00000462730 | 6.37E-06 | -0.1331 | 390 | 0.000 |
| ENSP00000369050 | 2.67E-05 | -0.1331 | 265 | 0.785 |
| ENSP00000350509 | 1.68E-05 | -0.1332 | 179 | 0.123 |
| ENSP00000472703 | 5.97E-07 | -0.1332 | 0 | 0.000 |
| ENSP00000361857 | 3.18E-06 | -0.1332 | 0 | 0.681 |
| ENSP00000358478 | 3.48E-06 | -0.1332 | 0 | 0.737 |
| ENSP00000359698 | 2.67E-06 | -0.1332 | 0 | 0.150 |
| ENSP00000386443 | 2.19E-05 | -0.1332 | 482 | 0.109 |
| ENSP00000454748 | 1.08E-05 | -0.1332 | 0 | 0.469 |
| ENSP00000360163 | 2.22E-05 | -0.1332 | 268 | 0.913 |
| ENSP00000339004 | 6.77E-06 | -0.1333 | 179 | 0.000 |
| ENSP00000376765 | 2.44E-05 | -0.1333 | 158 | 0.808 |
| ENSP00000368880 | 2.88E-05 | -0.1334 | 349 | 0.000 |
| ENSP00000420826 | 7.84E-06 | -0.1334 | 158 | 0.137 |
| ENSP00000387170 | 4.13E-06 | -0.1335 | 0 | 0.438 |
| ENSP00000340799 | 4.30E-06 | -0.1335 | 0 | 0.740 |
| ENSP00000330838 | 3.50E-06 | -0.1335 | 0 | 0.658 |
| ENSP00000457704 | 2.25E-06 | -0.1335 | 0 | 0.000 |
| ENSP00000360268 | 2.42E-05 | -0.1335 | 575 | 0.548 |

| | | | | |
|---|---|---|---|---|
| ENSP00000441685 | 1.14E-07 | -0.1335 | 0 | 0.206 |
| ENSP00000332369 | 1.89E-05 | -0.1335 | 428 | 0.432 |
| ENSP00000374467 | 1.71E-05 | -0.1336 | 318 | 0.632 |
| ENSP00000348565 | 1.57E-05 | -0.1336 | 318 | 0.442 |
| ENSP00000375268 | 3.47E-06 | -0.1336 | 0 | 0.186 |
| ENSP00000391200 | 4.52E-06 | -0.1336 | 0 | 0.698 |
| ENSP00000366527 | 2.83E-06 | -0.1336 | 0 | 0.683 |
| ENSP00000366542 | 2.87E-06 | -0.1337 | 0 | 0.674 |
| ENSP00000364864 | 2.11E-05 | -0.1337 | 270 | 0.415 |
| ENSP00000381793 | 1.46E-05 | -0.1337 | 0 | 0.763 |
| ENSP00000409936 | 1.57E-05 | -0.1337 | 244 | 0.000 |
| ENSP00000380444 | 1.65E-05 | -0.1337 | 244 | 0.469 |
| ENSP00000245620 | 3.07E-05 | -0.1337 | 0 | 0.214 |
| ENSP00000251377 | 3.07E-05 | -0.1337 | 0 | 0.216 |
| ENSP00000251390 | 3.07E-05 | -0.1337 | 0 | 0.000 |
| ENSP00000366528 | 5.83E-06 | -0.1337 | 0 | 0.108 |
| ENSP00000246792 | 6.86E-06 | -0.1337 | 448 | 0.606 |
| ENSP00000270221 | 1.99E-05 | -0.1338 | 179 | 0.121 |
| ENSP00000379475 | 1.74E-05 | -0.1338 | 533 | 0.000 |
| ENSP00000375151 | 4.94E-07 | -0.1338 | 0 | 0.200 |
| ENSP00000309597 | 6.78E-06 | -0.1338 | 205 | 0.689 |
| ENSP00000340887 | 3.76E-06 | -0.1338 | 0 | 0.192 |
| ENSP00000417335 | 8.61E-06 | -0.1339 | 0 | 0.675 |
| ENSP00000433352 | 1.00E-05 | -0.1339 | 271 | 0.598 |
| ENSP00000430237 | 8.85E-06 | -0.1339 | 265 | 0.302 |
| ENSP00000380024 | 2.33E-05 | -0.1339 | 203 | 0.908 |
| ENSP00000438346 | 7.52E-06 | -0.1339 | 181 | 0.067 |
| ENSP00000307798 | 3.95E-06 | -0.1339 | 0 | 0.129 |
| ENSP00000244576 | 6.00E-06 | -0.1339 | 0 | 0.703 |
| ENSP00000409932 | 2.13E-06 | -0.1339 | 0 | 0.000 |
| ENSP00000344193 | 5.11E-06 | -0.1340 | 0 | 0.121 |
| ENSP00000377801 | 4.66E-06 | -0.1340 | 0 | 0.197 |
| ENSP00000442736 | 4.76E-06 | -0.1340 | 0 | 0.450 |
| ENSP00000282549 | 1.24E-05 | -0.1340 | 238 | 0.865 |
| ENSP00000339527 | 2.40E-05 | -0.1341 | 358 | 0.000 |
| ENSP00000329880 | 2.03E-05 | -0.1341 | 360 | 0.168 |
| ENSP00000386621 | 5.95E-06 | -0.1341 | 330 | 0.662 |
| ENSP00000339250 | 7.11E-06 | -0.1341 | 0 | 0.446 |
| ENSP00000278601 | 1.85E-08 | -0.1341 | 0 | 0.208 |
| ENSP00000366028 | 1.34E-05 | -0.1343 | 358 | 0.000 |
| ENSP00000329995 | 1.31E-05 | -0.1343 | 0 | 0.198 |
| ENSP00000377867 | 7.60E-06 | -0.1343 | 520 | 0.000 |
| ENSP00000356536 | 1.18E-05 | -0.1343 | 158 | 0.117 |
| ENSP00000355799 | 1.59E-05 | -0.1343 | 280 | 0.380 |
| ENSP00000234040 | 4.80E-06 | -0.1344 | 205 | 0.683 |
| ENSP00000023897 | 4.83E-06 | -0.1344 | 0 | 0.395 |

| | | | | |
|---|---|---|---|---|
| ENSP00000334681 | 2.07E-06 | -0.1344 | 0 | 0.116 |
| ENSP00000455442 | 1.14E-06 | -0.1344 | 0 | 0.127 |
| ENSP00000342381 | 7.82E-06 | -0.1344 | 175 | 0.702 |
| ENSP00000293805 | 3.85E-06 | -0.1344 | 0 | 0.759 |
| ENSP00000345393 | 6.04E-06 | -0.1344 | 0 | 0.685 |
| ENSP00000384004 | 1.13E-05 | -0.1345 | 174 | 0.634 |
| ENSP00000388953 | 7.31E-06 | -0.1345 | 0 | 0.000 |
| ENSP00000324806 | 3.31E-05 | -0.1345 | 414 | 0.000 |
| ENSP00000451866 | 1.00E-06 | -0.1346 | 0 | 0.105 |
| ENSP00000415070 | 3.01E-06 | -0.1346 | 0 | 0.000 |
| ENSP00000401445 | 2.98E-05 | -0.1346 | 999 | 0.689 |
| ENSP00000418191 | 2.21E-05 | -0.1346 | 520 | 0.647 |
| ENSP00000354671 | 4.19E-06 | -0.1346 | 0 | 0.551 |
| ENSP00000372035 | 3.15E-05 | -0.1346 | 490 | 0.685 |
| ENSP00000354621 | 2.43E-05 | -0.1347 | 241 | 0.833 |
| ENSP00000357564 | 1.92E-05 | -0.1347 | 177 | 0.253 |
| ENSP00000361892 | 8.03E-06 | -0.1347 | 520 | 0.648 |
| ENSP00000424198 | 1.20E-05 | -0.1348 | 293 | 0.199 |
| ENSP00000265191 | 1.18E-05 | -0.1348 | 315 | 0.586 |
| ENSP00000310189 | 6.12E-06 | -0.1348 | 171 | 0.659 |
| ENSP00000359478 | 1.06E-05 | -0.1348 | 216 | 0.469 |
| ENSP00000319796 | 6.29E-06 | -0.1348 | 0 | 0.132 |
| ENSP00000392495 | 4.43E-06 | -0.1349 | 0 | 0.108 |
| ENSP00000361454 | 2.01E-05 | -0.1349 | 359 | 0.000 |
| ENSP00000351206 | 1.35E-05 | -0.1349 | 235 | 0.106 |
| ENSP00000372290 | 4.79E-06 | -0.1349 | 0 | 0.095 |
| ENSP00000344242 | 6.97E-06 | -0.1349 | 0 | 0.631 |
| ENSP00000377492 | 2.01E-05 | -0.1349 | 215 | 0.590 |
| ENSP00000315299 | 4.68E-06 | -0.1349 | 153 | 0.652 |
| ENSP00000302400 | 1.50E-05 | -0.1350 | 0 | 0.112 |
| ENSP00000434258 | 3.45E-06 | -0.1350 | 0 | 0.000 |
| ENSP00000310878 | 3.88E-06 | -0.1350 | 0 | 0.000 |
| ENSP00000338774 | 4.91E-06 | -0.1351 | 0 | 0.797 |
| ENSP00000449999 | 1.18E-05 | -0.1352 | 159 | 0.127 |
| ENSP00000345530 | 1.32E-05 | -0.1352 | 379 | 0.755 |
| ENSP00000367841 | 7.10E-06 | -0.1352 | 198 | 0.064 |
| ENSP00000366434 | 9.46E-06 | -0.1352 | 177 | 0.721 |
| ENSP00000425840 | 5.09E-06 | -0.1353 | 0 | 0.376 |
| ENSP00000443539 | 3.57E-07 | -0.1353 | 0 | 0.000 |
| ENSP00000322802 | 8.94E-06 | -0.1353 | 159 | 0.000 |
| ENSP00000348762 | 2.32E-05 | -0.1353 | 325 | 0.393 |
| ENSP00000409964 | 1.25E-06 | -0.1353 | 0 | 0.000 |
| ENSP00000404375 | 3.33E-06 | -0.1353 | 0 | 0.801 |
| ENSP00000420211 | 2.61E-06 | -0.1353 | 0 | 0.142 |
| ENSP00000466950 | 2.12E-06 | -0.1353 | 0 | 0.000 |
| ENSP00000354270 | 7.86E-06 | -0.1353 | 0 | 0.096 |

| | | | | |
|---|---|---|---|---|
| ENSP00000376921 | 5.66E-06 | -0.1354 | 0 | 0.356 |
| ENSP00000392028 | 2.87E-05 | -0.1354 | 210 | 0.901 |
| ENSP00000339179 | 1.24E-05 | -0.1354 | 205 | 0.000 |
| ENSP00000465213 | 5.18E-06 | -0.1355 | 0 | 0.481 |
| ENSP00000265304 | 1.58E-05 | -0.1355 | 153 | 0.718 |
| ENSP00000424261 | 5.73E-06 | -0.1356 | 0 | 0.171 |
| ENSP00000451235 | 2.38E-05 | -0.1356 | 191 | 0.000 |
| ENSP00000472951 | 2.26E-08 | -0.1356 | 0 | 0.000 |
| ENSP00000350386 | 1.54E-05 | -0.1357 | 270 | 0.286 |
| ENSP00000371983 | 2.69E-06 | -0.1357 | 0 | 0.452 |
| ENSP00000392411 | 6.52E-06 | -0.1357 | 0 | 0.064 |
| ENSP00000430548 | 4.23E-06 | -0.1357 | 0 | 0.148 |
| ENSP00000338369 | 1.79E-05 | -0.1357 | 222 | 0.279 |
| ENSP00000357775 | 1.02E-05 | -0.1358 | 260 | 0.290 |
| ENSP00000348827 | 1.97E-05 | -0.1358 | 238 | 0.803 |
| ENSP00000452581 | 2.93E-07 | -0.1358 | 0 | 0.000 |
| ENSP00000370125 | 4.04E-06 | -0.1358 | 0 | 0.662 |
| ENSP00000337825 | 3.04E-05 | -0.1359 | 854 | 0.698 |
| ENSP00000432279 | 3.57E-06 | -0.1359 | 0 | 0.065 |
| ENSP00000324464 | 7.28E-06 | -0.1359 | 182 | 0.703 |
| ENSP00000259808 | 5.34E-06 | -0.1359 | 300 | 0.691 |
| ENSP00000364632 | 1.73E-06 | -0.1359 | 0 | 0.188 |
| ENSP00000362608 | 1.87E-05 | -0.1359 | 259 | 0.689 |
| ENSP00000373640 | 4.15E-06 | -0.1360 | 0 | 0.000 |
| ENSP00000305260 | 6.36E-06 | -0.1360 | 397 | 0.660 |
| ENSP00000429084 | 1.33E-05 | -0.1360 | 197 | 0.676 |
| ENSP00000308479 | 1.15E-05 | -0.1360 | 0 | 0.141 |
| ENSP00000265334 | 1.92E-05 | -0.1360 | 0 | 0.199 |
| ENSP00000364595 | 1.36E-05 | -0.1361 | 632 | 0.194 |
| ENSP00000383578 | 1.62E-06 | -0.1361 | 0 | 0.000 |
| ENSP00000356379 | 3.71E-06 | -0.1361 | 0 | 0.298 |
| ENSP00000397759 | 5.19E-06 | -0.1362 | 0 | 0.164 |
| ENSP00000405289 | 2.78E-06 | -0.1362 | 0 | 0.710 |
| ENSP00000373073 | 3.95E-06 | -0.1362 | 0 | 0.102 |
| ENSP00000356530 | 1.83E-05 | -0.1362 | 586 | 0.699 |
| ENSP00000410396 | 1.50E-05 | -0.1363 | 215 | 0.815 |
| ENSP00000229002 | 4.04E-06 | -0.1363 | 466 | 0.591 |
| ENSP00000393453 | 1.81E-05 | -0.1363 | 244 | 0.917 |
| ENSP00000427690 | 1.29E-05 | -0.1364 | 198 | 0.195 |
| ENSP00000355026 | 4.35E-06 | -0.1364 | 0 | 0.597 |
| ENSP00000379387 | 3.74E-06 | -0.1364 | 0 | 0.679 |
| ENSP00000454445 | 2.04E-05 | -0.1364 | 178 | 0.000 |
| ENSP00000415183 | 2.67E-05 | -0.1364 | 846 | 0.000 |
| ENSP00000354497 | 4.00E-06 | -0.1365 | 0 | 0.283 |
| ENSP00000328054 | 2.24E-06 | -0.1365 | 0 | 0.138 |
| ENSP00000359777 | 7.53E-06 | -0.1365 | 0 | 0.000 |

| | | | | |
|---|---|---|---|---|
| ENSP00000287878 | 7.52E-06 | -0.1365 | 259 | 0.678 |
| ENSP00000367959 | 1.73E-05 | -0.1366 | 296 | 0.718 |
| ENSP00000360762 | 2.38E-05 | -0.1366 | 239 | 0.809 |
| ENSP00000337044 | 2.06E-05 | -0.1366 | 0 | 0.105 |
| ENSP00000377954 | 2.24E-05 | -0.1367 | 441 | 0.723 |
| ENSP00000441929 | 1.64E-05 | -0.1367 | 196 | 0.248 |
| ENSP00000394415 | 5.91E-06 | -0.1367 | 330 | 0.000 |
| ENSP00000370227 | 3.87E-06 | -0.1367 | 0 | 0.000 |
| ENSP00000397582 | 2.09E-05 | -0.1367 | 180 | 0.576 |
| ENSP00000356972 | 3.87E-06 | -0.1367 | 0 | 0.393 |
| ENSP00000355739 | 4.05E-06 | -0.1368 | 0 | 0.099 |
| ENSP00000261890 | 1.09E-05 | -0.1368 | 771 | 0.526 |
| ENSP00000375093 | 2.18E-06 | -0.1368 | 0 | 0.227 |
| ENSP00000394842 | 3.65E-06 | -0.1368 | 0 | 0.081 |
| ENSP00000401946 | 5.11E-06 | -0.1368 | 0 | 0.482 |
| ENSP00000403683 | 4.74E-06 | -0.1369 | 0 | 0.104 |
| ENSP00000381542 | 3.69E-06 | -0.1369 | 0 | 0.117 |
| ENSP00000384197 | 2.72E-05 | -0.1369 | 200 | 0.546 |
| ENSP00000382987 | 3.16E-06 | -0.1369 | 0 | 0.686 |
| ENSP00000387471 | 2.93E-06 | -0.1369 | 0 | 0.248 |
| ENSP00000310661 | 6.43E-06 | -0.1369 | 511 | 0.656 |
| ENSP00000367391 | 1.61E-05 | -0.1370 | 247 | 0.202 |
| ENSP00000306390 | 1.84E-05 | -0.1370 | 230 | 0.132 |
| ENSP00000358884 | 2.59E-06 | -0.1370 | 0 | 0.166 |
| ENSP00000457539 | 6.28E-06 | -0.1370 | 323 | 0.126 |
| ENSP00000353793 | 3.49E-06 | -0.1371 | 0 | 0.675 |
| ENSP00000412130 | 1.78E-05 | -0.1371 | 159 | 0.272 |
| ENSP00000360240 | 3.83E-06 | -0.1371 | 0 | 0.155 |
| ENSP00000397073 | 1.45E-05 | -0.1371 | 207 | 0.269 |
| ENSP00000365735 | 1.64E-05 | -0.1372 | 417 | 0.821 |
| ENSP00000380635 | 2.13E-05 | -0.1372 | 286 | 0.440 |
| ENSP00000308279 | 1.45E-05 | -0.1372 | 0 | 0.186 |
| ENSP00000363980 | 5.62E-06 | -0.1372 | 0 | 0.237 |
| ENSP00000382957 | 3.15E-06 | -0.1373 | 0 | 0.688 |
| ENSP00000379702 | 2.95E-06 | -0.1373 | 0 | 0.682 |
| ENSP00000353422 | 4.64E-06 | -0.1373 | 0 | 0.182 |
| ENSP00000456010 | 9.51E-07 | -0.1373 | 0 | 0.000 |
| ENSP00000368264 | 3.42E-06 | -0.1373 | 0 | 0.229 |
| ENSP00000331127 | 6.49E-06 | -0.1374 | 0 | 0.612 |
| ENSP00000417038 | 4.24E-06 | -0.1374 | 0 | 0.103 |
| ENSP00000360843 | 4.00E-06 | -0.1374 | 0 | 0.470 |
| ENSP00000375639 | 7.74E-06 | -0.1374 | 0 | 0.382 |
| ENSP00000363412 | 5.28E-06 | -0.1375 | 776 | 0.273 |
| ENSP00000346251 | 7.98E-06 | -0.1375 | 0 | 0.109 |
| ENSP00000379183 | 2.25E-06 | -0.1375 | 0 | 0.095 |
| ENSP00000382951 | 1.28E-06 | -0.1376 | 0 | 0.000 |

| | | | | |
|---|---|---|---|---|
| ENSP00000430432 | 3.64E-05 | -0.1376 | 462 | 0.000 |
| ENSP00000394178 | 3.41E-06 | -0.1376 | 0 | 0.100 |
| ENSP00000402915 | 2.29E-07 | -0.1376 | 0 | 0.172 |
| ENSP00000320209 | 8.48E-06 | -0.1376 | 0 | 0.336 |
| ENSP00000348965 | 1.11E-05 | -0.1376 | 179 | 0.300 |
| ENSP00000350249 | 2.27E-05 | -0.1377 | 311 | 0.520 |
| ENSP00000364728 | 2.94E-06 | -0.1377 | 0 | 0.000 |
| ENSP00000335084 | 1.17E-05 | -0.1377 | 415 | 0.616 |
| ENSP00000414208 | 1.71E-06 | -0.1377 | 0 | 0.000 |
| ENSP00000299415 | 5.77E-06 | -0.1377 | 0 | 0.000 |
| ENSP00000222256 | 9.40E-06 | -0.1378 | 271 | 0.233 |
| ENSP00000377218 | 1.40E-05 | -0.1378 | 923 | 0.814 |
| ENSP00000341787 | 1.05E-05 | -0.1378 | 0 | 0.120 |
| ENSP00000388457 | 2.06E-06 | -0.1378 | 0 | 0.000 |
| ENSP00000397415 | 9.09E-06 | -0.1378 | 191 | 0.210 |
| ENSP00000337691 | 1.57E-05 | -0.1379 | 344 | 0.671 |
| ENSP00000298375 | 2.47E-05 | -0.1379 | 243 | 0.160 |
| ENSP00000416445 | 5.13E-06 | -0.1379 | 0 | 0.000 |
| ENSP00000358301 | 2.50E-05 | -0.1379 | 345 | 0.582 |
| ENSP00000434939 | 9.87E-06 | -0.1379 | 158 | 0.000 |
| ENSP00000380603 | 4.00E-06 | -0.1379 | 0 | 0.117 |
| ENSP00000299759 | 1.32E-05 | -0.1380 | 466 | 0.616 |
| ENSP00000358314 | 1.48E-05 | -0.1380 | 166 | 0.000 |
| ENSP00000410466 | 2.49E-06 | -0.1380 | 0 | 0.698 |
| ENSP00000368761 | 3.28E-06 | -0.1380 | 0 | 0.667 |
| ENSP00000367817 | 1.60E-05 | -0.1381 | 152 | 0.400 |
| ENSP00000377384 | 6.80E-06 | -0.1382 | 0 | 0.000 |
| ENSP00000360938 | 1.29E-05 | -0.1382 | 237 | 0.468 |
| ENSP00000404403 | 1.31E-05 | -0.1382 | 198 | 0.710 |
| ENSP00000429824 | 1.27E-05 | -0.1382 | 206 | 0.371 |
| ENSP00000466051 | 2.37E-06 | -0.1382 | 0 | 0.693 |
| ENSP00000422168 | 4.87E-06 | -0.1382 | 0 | 0.332 |
| ENSP00000398798 | 3.15E-06 | -0.1382 | 0 | 0.680 |
| ENSP00000360985 | 2.12E-05 | -0.1383 | 191 | 0.238 |
| ENSP00000417496 | 1.44E-06 | -0.1383 | 0 | 0.166 |
| ENSP00000380037 | 3.53E-06 | -0.1383 | 0 | 0.164 |
| ENSP00000376823 | 1.46E-05 | -0.1383 | 158 | 0.250 |
| ENSP00000366058 | 1.35E-06 | -0.1384 | 0 | 0.132 |
| ENSP00000467018 | 2.87E-06 | -0.1384 | 0 | 0.692 |
| ENSP00000379228 | 2.10E-05 | -0.1384 | 197 | 0.494 |
| ENSP00000343242 | 7.79E-06 | -0.1385 | 0 | 0.000 |
| ENSP00000266674 | 8.33E-06 | -0.1385 | 197 | 0.690 |
| ENSP00000411099 | 3.74E-06 | -0.1385 | 0 | 0.458 |
| ENSP00000375683 | 1.96E-05 | -0.1385 | 504 | 0.216 |
| ENSP00000330188 | 9.06E-06 | -0.1385 | 0 | 0.232 |
| ENSP00000241463 | 4.53E-06 | -0.1385 | 448 | 0.595 |

| | | | | |
|---|---|---|---|---|
| ENSP00000386166 | 1.19E-06 | -0.1385 | 0 | 0.152 |
| ENSP00000362763 | 4.71E-06 | -0.1385 | 0 | 0.718 |
| ENSP00000375057 | 2.27E-05 | -0.1386 | 274 | 0.495 |
| ENSP00000345229 | 1.85E-05 | -0.1386 | 260 | 0.219 |
| ENSP00000356840 | 1.33E-05 | -0.1386 | 163 | 0.840 |
| ENSP00000420465 | 5.42E-06 | -0.1386 | 0 | 0.113 |
| ENSP00000259845 | 3.32E-05 | -0.1387 | 0 | 0.178 |
| ENSP00000304553 | 1.08E-05 | -0.1387 | 0 | 0.083 |
| ENSP00000472005 | 5.57E-07 | -0.1387 | 0 | 0.000 |
| ENSP00000354888 | 1.63E-05 | -0.1387 | 0 | 0.141 |
| ENSP00000425133 | 3.42E-06 | -0.1387 | 0 | 0.768 |
| ENSP00000331614 | 1.23E-05 | -0.1387 | 163 | 0.833 |
| ENSP00000341083 | 4.67E-06 | -0.1388 | 0 | 0.667 |
| ENSP00000458075 | 1.82E-05 | -0.1388 | 165 | 0.000 |
| ENSP00000432688 | 6.25E-06 | -0.1388 | 0 | 0.122 |
| ENSP00000274897 | 8.47E-06 | -0.1388 | 0 | 0.188 |
| ENSP00000349568 | 4.25E-06 | -0.1388 | 0 | 0.000 |
| ENSP00000323867 | 8.39E-06 | -0.1389 | 256 | 0.682 |
| ENSP00000423333 | 3.67E-06 | -0.1389 | 0 | 0.885 |
| ENSP00000387462 | 4.57E-06 | -0.1389 | 0 | 0.708 |
| ENSP00000417498 | 2.18E-05 | -0.1389 | 240 | 0.360 |
| ENSP00000355241 | 2.28E-06 | -0.1389 | 0 | 0.159 |
| ENSP00000389175 | 1.37E-05 | -0.1389 | 159 | 0.161 |
| ENSP00000365441 | 1.05E-05 | -0.1389 | 407 | 0.385 |
| ENSP00000358262 | 1.92E-05 | -0.1390 | 277 | 0.929 |
| ENSP00000366534 | 5.14E-06 | -0.1390 | 0 | 0.000 |
| ENSP00000332592 | 3.06E-06 | -0.1391 | 0 | 0.075 |
| ENSP00000262506 | 5.35E-06 | -0.1391 | 156 | 0.000 |
| ENSP00000462337 | 4.05E-06 | -0.1392 | 0 | 0.744 |
| ENSP00000358224 | 9.12E-06 | -0.1392 | 311 | 0.000 |
| ENSP00000367356 | 5.79E-06 | -0.1392 | 0 | 0.102 |
| ENSP00000363480 | 1.32E-05 | -0.1392 | 302 | 0.542 |
| ENSP00000281382 | 7.06E-06 | -0.1392 | 0 | 0.164 |
| ENSP00000444747 | 3.76E-06 | -0.1392 | 0 | 0.690 |
| ENSP00000355265 | 2.12E-06 | -0.1393 | 0 | 0.080 |
| ENSP00000406164 | 2.35E-06 | -0.1393 | 0 | 0.217 |
| ENSP00000365637 | 4.89E-06 | -0.1394 | 0 | 0.091 |
| ENSP00000378624 | 2.19E-05 | -0.1394 | 158 | 0.610 |
| ENSP00000266712 | 5.79E-06 | -0.1394 | 316 | 0.701 |
| ENSP00000329584 | 7.46E-06 | -0.1394 | 0 | 0.133 |
| ENSP00000245304 | 5.46E-06 | -0.1394 | 452 | 0.608 |
| ENSP00000376293 | 4.50E-06 | -0.1395 | 0 | 0.093 |
| ENSP00000358896 | 1.88E-05 | -0.1395 | 213 | 0.107 |
| ENSP00000351497 | 1.52E-05 | -0.1395 | 165 | 0.674 |
| ENSP00000293405 | 7.62E-06 | -0.1395 | 0 | 0.102 |
| ENSP00000261211 | 7.70E-06 | -0.1395 | 221 | 0.000 |

| | | | | |
|---|---|---|---|---|
| ENSP00000362047 | 3.14E-06 | -0.1395 | 0 | 0.119 |
| ENSP00000354910 | 5.16E-06 | -0.1395 | 0 | 0.656 |
| ENSP00000267205 | 9.41E-06 | -0.1396 | 204 | 0.563 |
| ENSP00000381214 | 5.63E-06 | -0.1396 | 0 | 0.721 |
| ENSP00000316030 | 2.17E-05 | -0.1396 | 227 | 0.000 |
| ENSP00000343204 | 2.51E-05 | -0.1396 | 589 | 0.677 |
| ENSP00000238609 | 8.87E-06 | -0.1396 | 0 | 0.146 |
| ENSP00000324767 | 9.98E-07 | -0.1397 | 0 | 0.000 |
| ENSP00000302716 | 1.82E-05 | -0.1397 | 252 | 0.254 |
| ENSP00000352527 | 3.08E-06 | -0.1397 | 0 | 0.165 |
| ENSP00000249284 | 3.55E-05 | -0.1397 | 290 | 0.480 |
| ENSP00000380318 | 1.57E-05 | -0.1397 | 212 | 0.000 |
| ENSP00000404121 | 1.51E-05 | -0.1398 | 532 | 0.853 |
| ENSP00000454407 | 1.54E-06 | -0.1398 | 0 | 0.099 |
| ENSP00000451145 | 1.29E-05 | -0.1398 | 163 | 0.000 |
| ENSP00000364801 | 1.17E-05 | -0.1399 | 195 | 0.832 |
| ENSP00000242728 | 6.35E-06 | -0.1399 | 0 | 0.793 |
| ENSP00000357116 | 5.85E-06 | -0.1399 | 0 | 0.172 |
| ENSP00000367075 | 3.67E-06 | -0.1399 | 0 | 0.116 |
| ENSP00000370503 | 1.03E-05 | -0.1399 | 226 | 0.700 |
| ENSP00000336701 | 1.10E-05 | -0.1400 | 340 | 0.774 |
| ENSP00000337014 | 2.67E-05 | -0.1400 | 181 | 0.154 |
| ENSP00000398502 | 6.01E-06 | -0.1400 | 304 | 0.000 |
| ENSP00000301407 | 2.04E-05 | -0.1400 | 325 | 0.000 |
| ENSP00000423673 | 1.11E-05 | -0.1401 | 196 | 0.331 |
| ENSP00000371341 | 5.51E-06 | -0.1401 | 0 | 0.549 |
| ENSP00000459566 | 2.01E-06 | -0.1401 | 0 | 0.696 |
| ENSP00000384900 | 5.96E-06 | -0.1401 | 0 | 0.154 |
| ENSP00000427820 | 1.44E-06 | -0.1401 | 0 | 0.121 |
| ENSP00000417424 | 3.48E-06 | -0.1402 | 0 | 0.699 |
| ENSP00000370395 | 2.00E-05 | -0.1402 | 441 | 0.521 |
| ENSP00000311579 | 7.95E-06 | -0.1402 | 202 | 0.696 |
| ENSP00000371067 | 3.66E-05 | -0.1402 | 497 | 0.000 |
| ENSP00000421981 | 3.46E-06 | -0.1403 | 0 | 0.085 |
| ENSP00000356243 | 1.77E-05 | -0.1403 | 178 | 0.666 |
| ENSP00000472121 | 4.66E-07 | -0.1403 | 0 | 0.000 |
| ENSP00000392896 | 4.85E-06 | -0.1403 | 186 | 0.212 |
| ENSP00000293441 | 5.16E-06 | -0.1403 | 211 | 0.677 |
| ENSP00000354688 | 2.48E-06 | -0.1403 | 0 | 0.119 |
| ENSP00000282908 | 6.63E-06 | -0.1404 | 277 | 0.000 |
| ENSP00000363773 | 2.11E-05 | -0.1404 | 613 | 0.568 |
| ENSP00000409378 | 2.37E-05 | -0.1404 | 163 | 0.576 |
| ENSP00000325018 | 4.15E-06 | -0.1405 | 0 | 0.721 |
| ENSP00000317895 | 4.40E-06 | -0.1405 | 0 | 0.117 |
| ENSP00000334998 | 1.96E-05 | -0.1405 | 0 | 0.123 |
| ENSP00000450635 | 5.83E-06 | -0.1405 | 0 | 0.000 |

| | | | | |
|---|---|---|---|---|
| ENSP00000396732 | 2.89E-06 | -0.1406 | 0 | 0.619 |
| ENSP00000348704 | 1.68E-05 | -0.1406 | 154 | 0.643 |
| ENSP00000301466 | 8.13E-06 | -0.1406 | 294 | 0.367 |
| ENSP00000332995 | 1.65E-05 | -0.1406 | 244 | 0.000 |
| ENSP00000291906 | 5.30E-06 | -0.1406 | 158 | 0.653 |
| ENSP00000320532 | 3.61E-06 | -0.1406 | 0 | 0.157 |
| ENSP00000368964 | 2.02E-06 | -0.1406 | 0 | 0.130 |
| ENSP00000458866 | 1.99E-06 | -0.1407 | 0 | 0.000 |
| ENSP00000261170 | 2.66E-06 | -0.1407 | 0 | 0.664 |
| ENSP00000364371 | 2.52E-06 | -0.1407 | 0 | 0.122 |
| ENSP00000360672 | 1.05E-05 | -0.1407 | 159 | 0.364 |
| ENSP00000296046 | 1.73E-05 | -0.1408 | 279 | 0.159 |
| ENSP00000308351 | 5.56E-06 | -0.1408 | 205 | 0.680 |
| ENSP00000392257 | 4.57E-06 | -0.1408 | 496 | 0.540 |
| ENSP00000231887 | 1.04E-05 | -0.1409 | 251 | 0.441 |
| ENSP00000360621 | 1.74E-05 | -0.1409 | 342 | 0.851 |
| ENSP00000357922 | 2.07E-05 | -0.1409 | 203 | 0.269 |
| ENSP00000365624 | 3.54E-06 | -0.1409 | 0 | 0.000 |
| ENSP00000419740 | 2.09E-05 | -0.1410 | 184 | 0.859 |
| ENSP00000384703 | 5.02E-06 | -0.1410 | 201 | 0.000 |
| ENSP00000362392 | 1.57E-06 | -0.1410 | 0 | 0.000 |
| ENSP00000297737 | 8.20E-06 | -0.1410 | 0 | 0.128 |
| ENSP00000466829 | 3.21E-06 | -0.1411 | 0 | 0.000 |
| ENSP00000360540 | 2.49E-05 | -0.1411 | 210 | 0.229 |
| ENSP00000347742 | 4.06E-06 | -0.1411 | 0 | 0.131 |
| ENSP00000264316 | 7.41E-06 | -0.1411 | 205 | 0.682 |
| ENSP00000302790 | 3.74E-06 | -0.1411 | 0 | 0.699 |
| ENSP00000396593 | 3.34E-06 | -0.1411 | 0 | 0.111 |
| ENSP00000334985 | 1.00E-05 | -0.1412 | 0 | 0.142 |
| ENSP00000383020 | 3.24E-06 | -0.1412 | 0 | 0.686 |
| ENSP00000346635 | 7.71E-06 | -0.1412 | 552 | 0.000 |
| ENSP00000366511 | 1.40E-06 | -0.1412 | 0 | 0.000 |
| ENSP00000265563 | 6.64E-06 | -0.1412 | 760 | 0.652 |
| ENSP00000308450 | 1.11E-05 | -0.1412 | 163 | 0.700 |
| ENSP00000340546 | 3.19E-07 | -0.1412 | 0 | 0.194 |
| ENSP00000440385 | 1.02E-07 | -0.1413 | 0 | 0.000 |
| ENSP00000366452 | 1.06E-05 | -0.1413 | 199 | 0.152 |
| ENSP00000311390 | 1.24E-05 | -0.1413 | 0 | 0.000 |
| ENSP00000308176 | 1.21E-05 | -0.1413 | 214 | 0.671 |
| ENSP00000385481 | 4.66E-06 | -0.1414 | 306 | 0.496 |
| ENSP00000360475 | 4.94E-06 | -0.1414 | 0 | 0.094 |
| ENSP00000402551 | 3.14E-06 | -0.1414 | 0 | 0.462 |
| ENSP00000313420 | 2.29E-05 | -0.1415 | 818 | 0.772 |
| ENSP00000331965 | 5.53E-06 | -0.1415 | 0 | 0.134 |
| ENSP00000438400 | 2.15E-06 | -0.1415 | 0 | 0.139 |
| ENSP00000381009 | 2.82E-06 | -0.1416 | 0 | 0.098 |

| | | | | |
|---|---|---|---|---|
| ENSP00000349893 | 1.40E-05 | -0.1416 | 271 | 0.712 |
| ENSP00000380251 | 6.30E-06 | -0.1416 | 0 | 0.091 |
| ENSP00000433157 | 9.13E-06 | -0.1416 | 163 | 0.148 |
| ENSP00000409463 | 2.99E-06 | -0.1416 | 0 | 0.722 |
| ENSP00000384894 | 1.17E-05 | -0.1416 | 0 | 0.152 |
| ENSP00000352182 | 1.14E-05 | -0.1416 | 158 | 0.091 |
| ENSP00000364985 | 3.42E-06 | -0.1416 | 0 | 0.156 |
| ENSP00000312150 | 7.41E-06 | -0.1416 | 284 | 0.689 |
| ENSP00000285116 | 3.39E-06 | -0.1416 | 0 | 0.300 |
| ENSP00000366005 | 2.44E-05 | -0.1417 | 927 | 0.732 |
| ENSP00000364310 | 2.08E-05 | -0.1417 | 643 | 0.895 |
| ENSP00000435591 | 2.60E-05 | -0.1417 | 407 | 0.000 |
| ENSP00000370109 | 1.09E-05 | -0.1417 | 347 | 0.760 |
| ENSP00000451080 | 1.90E-05 | -0.1417 | 218 | 0.435 |
| ENSP00000441365 | 3.28E-06 | -0.1417 | 0 | 0.147 |
| ENSP00000473233 | 4.70E-06 | -0.1417 | 0 | 0.778 |
| ENSP00000390590 | 9.65E-06 | -0.1418 | 0 | 0.097 |
| ENSP00000299766 | 1.23E-05 | -0.1418 | 505 | 0.732 |
| ENSP00000408631 | 4.54E-06 | -0.1418 | 0 | 0.253 |
| ENSP00000286190 | 2.75E-05 | -0.1418 | 0 | 0.132 |
| ENSP00000340514 | 5.77E-06 | -0.1418 | 0 | 0.667 |
| ENSP00000327583 | 5.39E-06 | -0.1419 | 185 | 0.729 |
| ENSP00000386456 | 1.27E-05 | -0.1419 | 198 | 0.666 |
| ENSP00000365063 | 3.07E-06 | -0.1420 | 0 | 0.072 |
| ENSP00000262429 | 2.55E-06 | -0.1420 | 0 | 0.627 |
| ENSP00000370938 | 2.32E-05 | -0.1420 | 262 | 0.000 |
| ENSP00000371932 | 8.89E-06 | -0.1420 | 0 | 0.483 |
| ENSP00000365694 | 1.89E-05 | -0.1421 | 199 | 0.207 |
| ENSP00000348489 | 4.49E-06 | -0.1421 | 0 | 0.216 |
| ENSP00000349358 | 9.43E-06 | -0.1421 | 236 | 0.142 |
| ENSP00000358948 | 1.48E-05 | -0.1421 | 210 | 0.000 |
| ENSP00000382675 | 1.14E-06 | -0.1421 | 0 | 0.150 |
| ENSP00000428982 | 1.71E-05 | -0.1421 | 263 | 0.765 |
| ENSP00000404304 | 3.51E-07 | -0.1421 | 0 | 0.180 |
| ENSP00000391432 | 1.69E-05 | -0.1422 | 599 | 0.861 |
| ENSP00000300131 | 1.01E-05 | -0.1422 | 0 | 0.764 |
| ENSP00000435210 | 2.96E-06 | -0.1423 | 0 | 0.496 |
| ENSP00000469049 | 1.01E-05 | -0.1423 | 278 | 0.424 |
| ENSP00000382561 | 2.12E-06 | -0.1423 | 0 | 0.156 |
| ENSP00000366984 | 8.34E-06 | -0.1423 | 0 | 0.150 |
| ENSP00000391774 | 5.35E-06 | -0.1424 | 0 | 0.736 |
| ENSP00000420270 | 5.77E-06 | -0.1424 | 0 | 0.665 |
| ENSP00000439918 | 1.98E-06 | -0.1424 | 0 | 0.378 |
| ENSP00000384744 | 1.32E-05 | -0.1424 | 545 | 0.316 |
| ENSP00000356744 | 3.59E-06 | -0.1425 | 0 | 0.103 |
| ENSP00000378138 | 2.16E-06 | -0.1425 | 0 | 0.129 |

| | | | | |
|---|---|---|---|---|
| ENSP00000333887 | 1.03E-06 | -0.1425 | 0 | 0.184 |
| ENSP00000457511 | 1.33E-07 | -0.1425 | 0 | 0.149 |
| ENSP00000469468 | 2.16E-05 | -0.1425 | 179 | 0.632 |
| ENSP00000335503 | 1.07E-06 | -0.1426 | 0 | 0.186 |
| ENSP00000364180 | 3.81E-06 | -0.1426 | 0 | 0.088 |
| ENSP00000364089 | 3.12E-06 | -0.1426 | 0 | 0.155 |
| ENSP00000256953 | 4.20E-06 | -0.1426 | 448 | 0.595 |
| ENSP00000335567 | 1.02E-06 | -0.1426 | 0 | 0.184 |
| ENSP00000358407 | 4.99E-06 | -0.1426 | 0 | 0.052 |
| ENSP00000395220 | 2.59E-06 | -0.1427 | 0 | 0.159 |
| ENSP00000326759 | 4.25E-06 | -0.1427 | 0 | 0.679 |
| ENSP00000361558 | 1.59E-05 | -0.1427 | 209 | 0.169 |
| ENSP00000381636 | 3.07E-06 | -0.1427 | 0 | 0.091 |
| ENSP00000351430 | 1.75E-05 | -0.1428 | 301 | 0.180 |
| ENSP00000334364 | 6.14E-07 | -0.1428 | 0 | 0.116 |
| ENSP00000361852 | 1.41E-05 | -0.1428 | 0 | 0.158 |
| ENSP00000373918 | 1.33E-05 | -0.1428 | 175 | 0.703 |
| ENSP00000351732 | 1.02E-05 | -0.1428 | 0 | 0.128 |
| ENSP00000398481 | 4.46E-06 | -0.1428 | 0 | 0.151 |
| ENSP00000386357 | 5.65E-06 | -0.1429 | 0 | 0.608 |
| ENSP00000411397 | 6.22E-06 | -0.1429 | 0 | 0.759 |
| ENSP00000472998 | 2.90E-05 | -0.1429 | 604 | 0.885 |
| ENSP00000402343 | 8.33E-06 | -0.1430 | 157 | 0.689 |
| ENSP00000367668 | 1.37E-05 | -0.1430 | 377 | 0.245 |
| ENSP00000361486 | 3.23E-06 | -0.1430 | 0 | 0.158 |
| ENSP00000385892 | 1.33E-05 | -0.1430 | 202 | 0.484 |
| ENSP00000365588 | 2.12E-05 | -0.1430 | 333 | 0.861 |
| ENSP00000359552 | 7.21E-06 | -0.1431 | 158 | 0.666 |
| ENSP00000371308 | 3.34E-06 | -0.1431 | 0 | 0.168 |
| ENSP00000367439 | 5.70E-06 | -0.1431 | 172 | 0.909 |
| ENSP00000366995 | 2.22E-06 | -0.1431 | 0 | 0.127 |
| ENSP00000367637 | 1.19E-05 | -0.1431 | 211 | 0.164 |
| ENSP00000341549 | 1.48E-05 | -0.1432 | 179 | 0.099 |
| ENSP00000391504 | 2.50E-07 | -0.1432 | 0 | 0.361 |
| ENSP00000356574 | 1.69E-05 | -0.1432 | 197 | 0.136 |
| ENSP00000417330 | 4.74E-06 | -0.1432 | 0 | 0.104 |
| ENSP00000411825 | 1.96E-05 | -0.1432 | 575 | 0.675 |
| ENSP00000421848 | 6.87E-06 | -0.1432 | 351 | 0.093 |
| ENSP00000324920 | 1.95E-05 | -0.1432 | 173 | 0.143 |
| ENSP00000374234 | 6.96E-07 | -0.1433 | 0 | 0.123 |
| ENSP00000273221 | 8.45E-06 | -0.1433 | 0 | 0.201 |
| ENSP00000312735 | 9.18E-06 | -0.1433 | 405 | 0.830 |
| ENSP00000353025 | 1.54E-05 | -0.1433 | 151 | 0.247 |
| ENSP00000467931 | 2.94E-06 | -0.1433 | 0 | 0.713 |
| ENSP00000457601 | 2.87E-06 | -0.1434 | 0 | 0.000 |
| ENSP00000364805 | 1.19E-05 | -0.1434 | 195 | 0.820 |

| | | | | |
|---|---|---|---|---|
| ENSP00000378699 | 4.26E-05 | -0.1434 | 647 | 0.000 |
| ENSP00000345853 | 5.30E-06 | -0.1435 | 0 | 0.704 |
| ENSP00000454411 | 3.84E-06 | -0.1436 | 0 | 0.134 |
| ENSP00000384881 | 8.63E-06 | -0.1436 | 300 | 0.570 |
| ENSP00000272852 | 1.98E-05 | -0.1436 | 199 | 0.072 |
| ENSP00000339681 | 7.45E-06 | -0.1436 | 0 | 0.105 |
| ENSP00000361499 | 5.84E-06 | -0.1436 | 0 | 0.499 |
| ENSP00000298048 | 7.21E-06 | -0.1436 | 205 | 0.702 |
| ENSP00000368315 | 2.12E-06 | -0.1437 | 0 | 0.000 |
| ENSP00000268864 | 5.54E-06 | -0.1437 | 448 | 0.000 |
| ENSP00000359000 | 1.99E-05 | -0.1437 | 267 | 0.659 |
| ENSP00000354588 | 1.74E-05 | -0.1437 | 913 | 0.000 |
| ENSP00000346671 | 2.90E-05 | -0.1437 | 907 | 0.701 |
| ENSP00000279249 | 6.85E-06 | -0.1437 | 0 | 0.066 |
| ENSP00000417573 | 3.70E-06 | -0.1438 | 0 | 0.129 |
| ENSP00000297145 | 1.35E-05 | -0.1438 | 0 | 0.206 |
| ENSP00000420939 | 7.10E-06 | -0.1438 | 164 | 0.072 |
| ENSP00000364839 | 3.77E-06 | -0.1438 | 0 | 0.715 |
| ENSP00000444972 | 2.46E-05 | -0.1438 | 842 | 0.866 |
| ENSP00000409514 | 2.19E-06 | -0.1438 | 0 | 0.000 |
| ENSP00000456566 | 1.57E-06 | -0.1439 | 0 | 0.120 |
| ENSP00000331784 | 6.47E-06 | -0.1439 | 0 | 0.000 |
| ENSP00000355013 | 8.26E-06 | -0.1439 | 316 | 0.751 |
| ENSP00000378289 | 2.42E-06 | -0.1440 | 0 | 0.183 |
| ENSP00000333993 | 1.96E-06 | -0.1440 | 0 | 0.152 |
| ENSP00000367229 | 5.72E-06 | -0.1440 | 0 | 0.121 |
| ENSP00000388220 | 1.85E-06 | -0.1440 | 0 | 0.000 |
| ENSP00000355136 | 1.19E-05 | -0.1440 | 242 | 0.552 |
| ENSP00000418960 | 3.98E-05 | -0.1441 | 349 | 0.825 |
| ENSP00000359404 | 3.39E-06 | -0.1441 | 0 | 0.138 |
| ENSP00000392828 | 1.28E-05 | -0.1441 | 196 | 0.269 |
| ENSP00000431371 | 1.75E-06 | -0.1442 | 0 | 0.221 |
| ENSP00000442365 | 3.07E-06 | -0.1442 | 0 | 0.080 |
| ENSP00000359239 | 2.63E-06 | -0.1442 | 0 | 0.000 |
| ENSP00000290823 | 6.22E-06 | -0.1442 | 0 | 0.000 |
| ENSP00000369270 | 5.19E-06 | -0.1443 | 0 | 0.736 |
| ENSP00000253362 | 1.27E-05 | -0.1443 | 0 | 0.121 |
| ENSP00000370741 | 3.89E-06 | -0.1443 | 0 | 0.279 |
| ENSP00000401335 | 1.23E-06 | -0.1443 | 0 | 0.148 |
| ENSP00000304643 | 4.32E-06 | -0.1444 | 153 | 0.641 |
| ENSP00000364898 | 2.58E-05 | -0.1444 | 943 | 0.665 |
| ENSP00000371644 | 7.63E-07 | -0.1444 | 0 | 0.203 |
| ENSP00000370460 | 4.96E-06 | -0.1444 | 244 | 0.684 |
| ENSP00000330426 | 9.31E-06 | -0.1444 | 0 | 0.125 |
| ENSP00000385939 | 3.50E-06 | -0.1445 | 0 | 0.684 |
| ENSP00000389427 | 7.00E-06 | -0.1445 | 0 | 0.399 |

| | | | | |
|---|---|---|---|---|
| ENSP00000261845 | 8.02E-06 | -0.1445 | 344 | 0.674 |
| ENSP00000297439 | 1.09E-05 | -0.1445 | 192 | 0.495 |
| ENSP00000443189 | 2.41E-05 | -0.1446 | 274 | 0.000 |
| ENSP00000464976 | 2.26E-06 | -0.1446 | 0 | 0.690 |
| ENSP00000392568 | 1.28E-06 | -0.1447 | 0 | 0.161 |
| ENSP00000334061 | 8.59E-06 | -0.1447 | 322 | 0.000 |
| ENSP00000382177 | 2.22E-05 | -0.1448 | 434 | 0.547 |
| ENSP00000328813 | 2.42E-05 | -0.1448 | 188 | 0.127 |
| ENSP00000405620 | 1.64E-05 | -0.1448 | 176 | 0.909 |
| ENSP00000360120 | 8.96E-06 | -0.1449 | 205 | 0.676 |
| ENSP00000295878 | 8.06E-06 | -0.1449 | 0 | 0.000 |
| ENSP00000413937 | 1.70E-05 | -0.1449 | 160 | 0.223 |
| ENSP00000391594 | 4.87E-06 | -0.1449 | 0 | 0.167 |
| ENSP00000355541 | 1.39E-05 | -0.1450 | 221 | 0.893 |
| ENSP00000393571 | 5.03E-06 | -0.1451 | 0 | 0.000 |
| ENSP00000375086 | 1.90E-05 | -0.1451 | 296 | 0.569 |
| ENSP00000266088 | 1.73E-05 | -0.1451 | 284 | 0.242 |
| ENSP00000356560 | 1.16E-05 | -0.1452 | 0 | 0.137 |
| ENSP00000264748 | 5.10E-06 | -0.1452 | 205 | 0.679 |
| ENSP00000445829 | 3.25E-06 | -0.1452 | 0 | 0.000 |
| ENSP00000412566 | 1.77E-05 | -0.1452 | 427 | 0.911 |
| ENSP00000450085 | 3.45E-06 | -0.1453 | 0 | 0.152 |
| ENSP00000331692 | 4.39E-06 | -0.1453 | 0 | 0.000 |
| ENSP00000357229 | 9.75E-06 | -0.1453 | 200 | 0.000 |
| ENSP00000390407 | 6.12E-06 | -0.1453 | 0 | 0.072 |
| ENSP00000281708 | 1.04E-05 | -0.1453 | 224 | 0.810 |
| ENSP00000364403 | 7.44E-06 | -0.1453 | 644 | 0.287 |
| ENSP00000346027 | 1.67E-05 | -0.1454 | 239 | 0.799 |
| ENSP00000384496 | 1.86E-05 | -0.1455 | 282 | 0.000 |
| ENSP00000380505 | 2.16E-05 | -0.1455 | 913 | 0.616 |
| ENSP00000363596 | 1.64E-05 | -0.1455 | 260 | 0.609 |
| ENSP00000368801 | 2.30E-06 | -0.1456 | 0 | 0.722 |
| ENSP00000258200 | 3.68E-06 | -0.1456 | 0 | 0.288 |
| ENSP00000333873 | 4.77E-06 | -0.1456 | 195 | 0.000 |
| ENSP00000272317 | 8.65E-06 | -0.1456 | 269 | 0.857 |
| ENSP00000472802 | 4.86E-06 | -0.1457 | 0 | 0.682 |
| ENSP00000398516 | 5.11E-06 | -0.1457 | 0 | 0.781 |
| ENSP00000420727 | 2.21E-06 | -0.1457 | 0 | 0.686 |
| ENSP00000387019 | 7.53E-06 | -0.1457 | 198 | 0.220 |
| ENSP00000429200 | 5.87E-06 | -0.1458 | 0 | 0.110 |
| ENSP00000312767 | 8.74E-06 | -0.1458 | 0 | 0.000 |
| ENSP00000429803 | 3.35E-06 | -0.1458 | 0 | 0.713 |
| ENSP00000383933 | 2.47E-06 | -0.1458 | 0 | 0.103 |
| ENSP00000443168 | 2.18E-05 | -0.1458 | 266 | 0.510 |
| ENSP00000315017 | 5.71E-06 | -0.1458 | 373 | 0.000 |
| ENSP00000376317 | 1.44E-05 | -0.1459 | 181 | 0.509 |

| | | | | |
|---|---|---|---|---|
| ENSP00000457748 | 1.56E-06 | -0.1459 | 0 | 0.173 |
| ENSP00000267396 | 5.31E-06 | -0.1459 | 448 | 0.600 |
| ENSP00000368102 | 7.27E-06 | -0.1459 | 0 | 0.114 |
| ENSP00000420418 | 4.30E-06 | -0.1459 | 0 | 0.702 |
| ENSP00000385276 | 1.85E-05 | -0.1459 | 188 | 0.134 |
| ENSP00000386921 | 3.20E-06 | -0.1459 | 0 | 0.215 |
| ENSP00000363411 | 3.96E-06 | -0.1460 | 0 | 0.357 |
| ENSP00000405676 | 2.77E-06 | -0.1460 | 0 | 0.000 |
| ENSP00000360190 | 1.88E-06 | -0.1460 | 0 | 0.121 |
| ENSP00000369335 | 8.19E-06 | -0.1461 | 315 | 0.649 |
| ENSP00000403925 | 4.49E-06 | -0.1461 | 0 | 0.113 |
| ENSP00000371825 | 3.06E-06 | -0.1461 | 0 | 0.134 |
| ENSP00000425864 | 7.61E-07 | -0.1462 | 0 | 0.000 |
| ENSP00000354499 | 3.24E-05 | -0.1462 | 242 | 0.698 |
| ENSP00000400168 | 2.57E-06 | -0.1462 | 0 | 0.251 |
| ENSP00000400223 | 1.11E-05 | -0.1462 | 169 | 0.416 |
| ENSP00000252037 | 8.23E-06 | -0.1462 | 271 | 0.583 |
| ENSP00000418719 | 3.35E-06 | -0.1462 | 0 | 0.693 |
| ENSP00000467123 | 3.66E-06 | -0.1462 | 0 | 0.132 |
| ENSP00000409370 | 3.66E-06 | -0.1462 | 0 | 0.129 |
| ENSP00000459753 | 9.14E-07 | -0.1462 | 0 | 0.000 |
| ENSP00000405573 | 2.11E-05 | -0.1462 | 505 | 0.626 |
| ENSP00000388169 | 6.29E-06 | -0.1462 | 0 | 0.374 |
| ENSP00000405812 | 4.75E-06 | -0.1463 | 0 | 0.146 |
| ENSP00000354829 | 1.45E-05 | -0.1463 | 725 | 0.213 |
| ENSP00000338988 | 2.20E-06 | -0.1464 | 0 | 0.000 |
| ENSP00000398131 | 1.57E-05 | -0.1464 | 440 | 0.867 |
| ENSP00000375076 | 7.70E-07 | -0.1464 | 0 | 0.204 |
| ENSP00000359525 | 1.88E-05 | -0.1464 | 178 | 0.000 |
| ENSP00000442521 | 2.43E-06 | -0.1464 | 0 | 0.148 |
| ENSP00000367494 | 1.51E-05 | -0.1464 | 0 | 0.096 |
| ENSP00000355572 | 2.89E-06 | -0.1464 | 0 | 0.229 |
| ENSP00000351767 | 1.77E-05 | -0.1464 | 169 | 0.268 |
| ENSP00000343493 | 4.18E-06 | -0.1464 | 0 | 0.164 |
| ENSP00000311760 | 8.31E-06 | -0.1464 | 0 | 0.181 |
| ENSP00000228928 | 6.10E-06 | -0.1465 | 900 | 0.655 |
| ENSP00000456635 | 6.95E-07 | -0.1465 | 0 | 0.000 |
| ENSP00000462054 | 2.35E-06 | -0.1465 | 0 | 0.698 |
| ENSP00000297436 | 1.97E-05 | -0.1465 | 195 | 0.341 |
| ENSP00000362743 | 9.78E-06 | -0.1466 | 0 | 0.175 |
| ENSP00000354387 | 9.14E-06 | -0.1466 | 277 | 0.153 |
| ENSP00000432172 | 3.97E-06 | -0.1466 | 0 | 0.143 |
| ENSP00000265174 | 3.83E-06 | -0.1466 | 0 | 0.668 |
| ENSP00000292125 | 8.54E-06 | -0.1467 | 0 | 0.209 |
| ENSP00000353656 | 1.39E-05 | -0.1467 | 397 | 0.000 |
| ENSP00000354982 | 1.80E-05 | -0.1467 | 181 | 0.275 |

| | | | | |
|---|---|---|---|---|
| ENSP00000428426 | 3.10E-05 | -0.1467 | 398 | 0.833 |
| ENSP00000362399 | 2.27E-05 | -0.1467 | 204 | 0.265 |
| ENSP00000359085 | 1.16E-05 | -0.1468 | 150 | 0.652 |
| ENSP00000354461 | 4.73E-06 | -0.1468 | 0 | 0.156 |
| ENSP00000471921 | 5.01E-06 | -0.1468 | 0 | 0.000 |
| ENSP00000343463 | 9.40E-06 | -0.1468 | 230 | 0.654 |
| ENSP00000360822 | 1.24E-05 | -0.1469 | 187 | 0.190 |
| ENSP00000275857 | 3.84E-06 | -0.1469 | 0 | 0.206 |
| ENSP00000432614 | 2.29E-06 | -0.1469 | 0 | 0.217 |
| ENSP00000376899 | 1.29E-05 | -0.1469 | 0 | 0.175 |
| ENSP00000396755 | 3.89E-06 | -0.1469 | 0 | 0.612 |
| ENSP00000332404 | 6.21E-06 | -0.1470 | 0 | 0.122 |
| ENSP00000306330 | 1.80E-05 | -0.1470 | 396 | 0.694 |
| ENSP00000393556 | 3.94E-06 | -0.1470 | 0 | 0.000 |
| ENSP00000259089 | 4.84E-06 | -0.1471 | 205 | 0.673 |
| ENSP00000420723 | 2.35E-07 | -0.1471 | 0 | 0.172 |
| ENSP00000310832 | 2.28E-05 | -0.1471 | 252 | 0.262 |
| ENSP00000349154 | 6.97E-06 | -0.1471 | 0 | 0.122 |
| ENSP00000296358 | 2.16E-05 | -0.1471 | 537 | 0.120 |
| ENSP00000454861 | 2.50E-06 | -0.1471 | 0 | 0.000 |
| ENSP00000353785 | 6.56E-06 | -0.1472 | 191 | 0.165 |
| ENSP00000363463 | 7.07E-06 | -0.1472 | 185 | 0.062 |
| ENSP00000325808 | 5.90E-06 | -0.1472 | 0 | 0.102 |
| ENSP00000356224 | 1.98E-05 | -0.1472 | 345 | 0.412 |
| ENSP00000399168 | 2.25E-05 | -0.1472 | 900 | 0.610 |
| ENSP00000338130 | 1.25E-05 | -0.1473 | 0 | 0.636 |
| ENSP00000362508 | 3.68E-06 | -0.1473 | 0 | 0.150 |
| ENSP00000345771 | 1.16E-05 | -0.1473 | 369 | 0.645 |
| ENSP00000290663 | 4.19E-06 | -0.1473 | 0 | 0.654 |
| ENSP00000361998 | 2.04E-05 | -0.1474 | 0 | 0.732 |
| ENSP00000357288 | 4.00E-06 | -0.1474 | 0 | 0.101 |
| ENSP00000352657 | 1.33E-05 | -0.1474 | 309 | 0.000 |
| ENSP00000441459 | 1.08E-06 | -0.1474 | 0 | 0.000 |
| ENSP00000374071 | 1.29E-05 | -0.1475 | 200 | 0.155 |
| ENSP00000306129 | 1.75E-05 | -0.1475 | 175 | 0.641 |
| ENSP00000367911 | 4.50E-06 | -0.1475 | 0 | 0.135 |
| ENSP00000358983 | 2.16E-05 | -0.1476 | 357 | 0.865 |
| ENSP00000366997 | 1.48E-05 | -0.1476 | 300 | 0.748 |
| ENSP00000438468 | 1.17E-05 | -0.1476 | 197 | 0.649 |
| ENSP00000264399 | 4.96E-06 | -0.1476 | 587 | 0.640 |
| ENSP00000377112 | 4.00E-06 | -0.1476 | 0 | 0.092 |
| ENSP00000366618 | 1.98E-05 | -0.1476 | 848 | 0.962 |
| ENSP00000405890 | 9.84E-06 | -0.1476 | 181 | 0.865 |
| ENSP00000466104 | 1.46E-06 | -0.1476 | 0 | 0.000 |
| ENSP00000406490 | 2.40E-05 | -0.1476 | 353 | 0.767 |
| ENSP00000362296 | 7.09E-06 | -0.1477 | 0 | 0.844 |

| | | | | |
|---|---|---|---|---|
| ENSP00000404074 | 3.07E-06 | -0.1477 | 0 | 0.688 |
| ENSP00000367189 | 2.95E-06 | -0.1477 | 0 | 0.000 |
| ENSP00000364578 | 4.67E-06 | -0.1477 | 0 | 0.583 |
| ENSP00000303963 | 1.85E-05 | -0.1478 | 158 | 0.081 |
| ENSP00000296468 | 4.67E-06 | -0.1478 | 0 | 0.108 |
| ENSP00000301665 | 1.15E-05 | -0.1478 | 0 | 0.153 |
| ENSP00000470539 | 2.63E-06 | -0.1478 | 0 | 0.092 |
| ENSP00000419782 | 4.19E-05 | -0.1479 | 450 | 0.000 |
| ENSP00000404220 | 7.83E-06 | -0.1479 | 0 | 0.073 |
| ENSP00000439065 | 2.39E-05 | -0.1480 | 316 | 0.000 |
| ENSP00000358095 | 5.59E-06 | -0.1480 | 0 | 0.168 |
| ENSP00000334285 | 7.34E-06 | -0.1481 | 0 | 0.459 |
| ENSP00000309836 | 2.94E-06 | -0.1481 | 0 | 0.135 |
| ENSP00000394071 | 1.89E-05 | -0.1481 | 202 | 0.230 |
| ENSP00000410818 | 1.49E-06 | -0.1482 | 0 | 0.131 |
| ENSP00000332615 | 7.35E-06 | -0.1482 | 0 | 0.136 |
| ENSP00000384725 | 1.42E-05 | -0.1482 | 167 | 0.735 |
| ENSP00000415200 | 7.80E-06 | -0.1483 | 0 | 0.000 |
| ENSP00000313809 | 1.79E-05 | -0.1483 | 357 | 0.402 |
| ENSP00000357796 | 3.62E-06 | -0.1483 | 0 | 0.186 |
| ENSP00000410858 | 1.82E-06 | -0.1483 | 0 | 0.282 |
| ENSP00000366050 | 3.64E-06 | -0.1483 | 0 | 0.141 |
| ENSP00000262187 | 5.62E-06 | -0.1483 | 457 | 0.635 |
| ENSP00000345772 | 9.39E-06 | -0.1483 | 903 | 0.000 |
| ENSP00000373952 | 5.13E-06 | -0.1484 | 0 | 0.698 |
| ENSP00000280098 | 3.55E-06 | -0.1484 | 0 | 0.451 |
| ENSP00000349467 | 1.33E-05 | -0.1485 | 311 | 0.668 |
| ENSP00000354643 | 9.87E-06 | -0.1485 | 0 | 0.060 |
| ENSP00000376914 | 6.06E-06 | -0.1486 | 222 | 0.000 |
| ENSP00000404658 | 1.86E-05 | -0.1486 | 244 | 0.887 |
| ENSP00000387739 | 6.64E-06 | -0.1486 | 0 | 0.114 |
| ENSP00000342105 | 7.51E-06 | -0.1486 | 446 | 0.692 |
| ENSP00000257515 | 8.37E-06 | -0.1487 | 0 | 0.129 |
| ENSP00000265717 | 5.94E-06 | -0.1487 | 760 | 0.657 |
| ENSP00000471898 | 1.52E-06 | -0.1487 | 0 | 0.000 |
| ENSP00000364965 | 4.04E-06 | -0.1487 | 0 | 0.129 |
| ENSP00000441410 | 2.70E-05 | -0.1488 | 241 | 0.813 |
| ENSP00000467286 | 6.04E-06 | -0.1488 | 161 | 0.000 |
| ENSP00000365585 | 1.76E-06 | -0.1488 | 0 | 0.195 |
| ENSP00000288309 | 1.52E-05 | -0.1488 | 225 | 0.000 |
| ENSP00000304642 | 4.53E-06 | -0.1489 | 0 | 0.127 |
| ENSP00000293892 | 4.91E-06 | -0.1489 | 0 | 0.000 |
| ENSP00000362935 | 1.68E-05 | -0.1489 | 179 | 0.255 |
| ENSP00000354340 | 9.07E-06 | -0.1489 | 266 | 0.509 |
| ENSP00000380531 | 2.20E-06 | -0.1491 | 0 | 0.189 |
| ENSP00000360532 | 1.99E-05 | -0.1491 | 461 | 0.813 |

| | | | | |
|---|---|---|---|---|
| ENSP00000294016 | 7.56E-06 | -0.1491 | 0 | 0.718 |
| ENSP00000367910 | 4.29E-06 | -0.1491 | 0 | 0.687 |
| ENSP00000326804 | 1.40E-05 | -0.1491 | 570 | 0.702 |
| ENSP00000394487 | 3.83E-06 | -0.1491 | 0 | 0.218 |
| ENSP00000443140 | 1.86E-06 | -0.1491 | 0 | 0.000 |
| ENSP00000380453 | 1.17E-05 | -0.1491 | 173 | 0.088 |
| ENSP00000368544 | 1.29E-05 | -0.1492 | 195 | 0.092 |
| ENSP00000346918 | 3.83E-06 | -0.1492 | 0 | 0.000 |
| ENSP00000334834 | 9.12E-07 | -0.1492 | 0 | 0.185 |
| ENSP00000351259 | 3.18E-06 | -0.1493 | 0 | 0.000 |
| ENSP00000357879 | 1.35E-05 | -0.1493 | 370 | 0.623 |
| ENSP00000371870 | 1.77E-05 | -0.1493 | 206 | 0.978 |
| ENSP00000378260 | 4.60E-06 | -0.1493 | 0 | 0.055 |
| ENSP00000370532 | 3.92E-06 | -0.1493 | 0 | 0.732 |
| ENSP00000359385 | 7.44E-06 | -0.1494 | 477 | 0.316 |
| ENSP00000375899 | 3.99E-06 | -0.1494 | 0 | 0.536 |
| ENSP00000363118 | 7.61E-06 | -0.1494 | 0 | 0.105 |
| ENSP00000367346 | 3.82E-06 | -0.1494 | 0 | 0.344 |
| ENSP00000373485 | 1.48E-06 | -0.1494 | 0 | 0.142 |
| ENSP00000451792 | 4.26E-06 | -0.1495 | 0 | 0.000 |
| ENSP00000471505 | 7.88E-06 | -0.1495 | 167 | 0.000 |
| ENSP00000395549 | 7.21E-06 | -0.1495 | 0 | 0.000 |
| ENSP00000471625 | 2.89E-06 | -0.1495 | 0 | 0.000 |
| ENSP00000396538 | 7.18E-06 | -0.1495 | 0 | 0.000 |
| ENSP00000369579 | 6.94E-06 | -0.1495 | 201 | 0.000 |
| ENSP00000334560 | 6.24E-06 | -0.1495 | 0 | 0.109 |
| ENSP00000338293 | 4.03E-06 | -0.1496 | 0 | 0.086 |
| ENSP00000350928 | 2.25E-05 | -0.1496 | 252 | 0.662 |
| ENSP00000398163 | 1.77E-05 | -0.1496 | 153 | 0.000 |
| ENSP00000465845 | 5.40E-06 | -0.1496 | 0 | 0.000 |
| ENSP00000352976 | 2.82E-06 | -0.1497 | 0 | 0.108 |
| ENSP00000301200 | 4.45E-06 | -0.1497 | 0 | 0.159 |
| ENSP00000366221 | 8.04E-06 | -0.1497 | 0 | 0.211 |
| ENSP00000375622 | 2.54E-06 | -0.1497 | 0 | 0.158 |
| ENSP00000393860 | 7.24E-06 | -0.1497 | 0 | 0.000 |
| ENSP00000366926 | 8.07E-06 | -0.1498 | 0 | 0.138 |
| ENSP00000325775 | 4.98E-06 | -0.1499 | 0 | 0.121 |
| ENSP00000362687 | 1.59E-05 | -0.1499 | 364 | 0.934 |
| ENSP00000381844 | 2.74E-06 | -0.1499 | 0 | 0.106 |
| ENSP00000329926 | 2.00E-05 | -0.1499 | 300 | 0.136 |
| ENSP00000312436 | 6.09E-06 | -0.1500 | 0 | 0.771 |
| ENSP00000406478 | 5.03E-07 | -0.1500 | 0 | 0.214 |
| ENSP00000250495 | 6.78E-06 | -0.1501 | 422 | 0.787 |
| ENSP00000284142 | 1.49E-05 | -0.1501 | 0 | 0.180 |
| ENSP00000364603 | 4.38E-06 | -0.1501 | 0 | 0.144 |
| ENSP00000429726 | 4.51E-07 | -0.1501 | 0 | 0.193 |

| | | | | |
|---|---|---|---|---|
| ENSP00000361266 | 2.56E-05 | -0.1501 | 433 | 0.918 |
| ENSP00000357644 | 4.15E-06 | -0.1501 | 0 | 0.381 |
| ENSP00000341031 | 6.34E-06 | -0.1502 | 0 | 0.148 |
| ENSP00000330878 | 1.49E-05 | -0.1502 | 154 | 0.581 |
| ENSP00000359454 | 1.70E-05 | -0.1502 | 427 | 0.856 |
| ENSP00000379098 | 1.19E-05 | -0.1502 | 175 | 0.644 |
| ENSP00000354652 | 1.74E-05 | -0.1502 | 213 | 0.614 |
| ENSP00000352028 | 1.84E-05 | -0.1503 | 161 | 0.227 |
| ENSP00000428878 | 3.88E-06 | -0.1503 | 0 | 0.670 |
| ENSP00000317721 | 5.07E-06 | -0.1503 | 0 | 0.307 |
| ENSP00000356015 | 6.73E-06 | -0.1503 | 295 | 0.427 |
| ENSP00000315136 | 8.31E-06 | -0.1503 | 0 | 0.094 |
| ENSP00000390427 | 5.07E-06 | -0.1503 | 244 | 0.000 |
| ENSP00000367220 | 1.86E-05 | -0.1504 | 316 | 0.682 |
| ENSP00000359334 | 9.34E-06 | -0.1505 | 252 | 0.000 |
| ENSP00000375892 | 2.51E-05 | -0.1505 | 699 | 0.000 |
| ENSP00000359233 | 1.04E-05 | -0.1505 | 230 | 0.253 |
| ENSP00000410395 | 2.98E-06 | -0.1505 | 0 | 0.000 |
| ENSP00000364649 | 1.25E-05 | -0.1505 | 906 | 0.689 |
| ENSP00000378430 | 3.41E-06 | -0.1505 | 0 | 0.000 |
| ENSP00000377430 | 1.60E-05 | -0.1506 | 220 | 0.082 |
| ENSP00000344430 | 3.48E-06 | -0.1506 | 0 | 0.162 |
| ENSP00000424417 | 1.95E-05 | -0.1506 | 464 | 0.808 |
| ENSP00000369127 | 2.16E-05 | -0.1506 | 949 | 0.757 |
| ENSP00000457386 | 1.77E-05 | -0.1506 | 235 | 0.384 |
| ENSP00000380336 | 6.66E-06 | -0.1506 | 0 | 0.560 |
| ENSP00000371398 | 3.52E-06 | -0.1507 | 0 | 0.744 |
| ENSP00000255992 | 1.50E-05 | -0.1507 | 0 | 0.000 |
| ENSP00000368824 | 1.33E-05 | -0.1507 | 294 | 0.271 |
| ENSP00000355173 | 3.81E-06 | -0.1507 | 0 | 0.176 |
| ENSP00000354762 | 3.16E-06 | -0.1508 | 0 | 0.080 |
| ENSP00000339916 | 4.47E-06 | -0.1508 | 210 | 0.675 |
| ENSP00000335677 | 2.84E-06 | -0.1509 | 0 | 0.000 |
| ENSP00000372122 | 3.15E-06 | -0.1509 | 0 | 0.000 |
| ENSP00000355613 | 2.06E-05 | -0.1509 | 501 | 0.290 |
| ENSP00000356515 | 2.22E-05 | -0.1510 | 420 | 0.719 |
| ENSP00000383901 | 1.12E-05 | -0.1510 | 196 | 0.250 |
| ENSP00000297477 | 9.82E-06 | -0.1510 | 0 | 0.166 |
| ENSP00000390783 | 4.97E-06 | -0.1510 | 202 | 0.234 |
| ENSP00000334592 | 5.03E-06 | -0.1510 | 0 | 0.198 |
| ENSP00000387262 | 9.17E-06 | -0.1510 | 252 | 0.498 |
| ENSP00000340667 | 4.98E-06 | -0.1511 | 0 | 0.109 |
| ENSP00000386207 | 1.02E-05 | -0.1511 | 252 | 0.119 |
| ENSP00000368605 | 3.34E-06 | -0.1511 | 0 | 0.098 |
| ENSP00000451605 | 1.54E-05 | -0.1511 | 361 | 0.757 |
| ENSP00000377793 | 5.37E-06 | -0.1511 | 0 | 0.843 |

| | | | | |
|---|---|---|---|---|
| ENSP00000444823 | 1.96E-05 | -0.1511 | 844 | 0.000 |
| ENSP00000389770 | 4.34E-06 | -0.1511 | 0 | 0.109 |
| ENSP00000326309 | 4.74E-06 | -0.1512 | 0 | 0.103 |
| ENSP00000333800 | 1.25E-05 | -0.1512 | 0 | 0.262 |
| ENSP00000380982 | 1.32E-05 | -0.1513 | 200 | 0.000 |
| ENSP00000414144 | 2.00E-06 | -0.1513 | 0 | 0.000 |
| ENSP00000419457 | 1.11E-05 | -0.1514 | 165 | 0.778 |
| ENSP00000389014 | 1.54E-06 | -0.1514 | 0 | 0.092 |
| ENSP00000373669 | 3.51E-06 | -0.1515 | 0 | 0.000 |
| ENSP00000349967 | 1.32E-05 | -0.1515 | 0 | 0.360 |
| ENSP00000378505 | 1.72E-05 | -0.1515 | 272 | 0.552 |
| ENSP00000370242 | 4.20E-06 | -0.1515 | 0 | 0.123 |
| ENSP00000329698 | 5.53E-06 | -0.1516 | 0 | 0.113 |
| ENSP00000366170 | 7.87E-06 | -0.1516 | 0 | 0.164 |
| ENSP00000348234 | 6.52E-06 | -0.1516 | 0 | 0.507 |
| ENSP00000406084 | 1.37E-05 | -0.1516 | 159 | 0.425 |
| ENSP00000362674 | 1.55E-05 | -0.1517 | 459 | 0.000 |
| ENSP00000365169 | 1.28E-06 | -0.1517 | 0 | 0.176 |
| ENSP00000345808 | 4.81E-06 | -0.1517 | 0 | 0.176 |
| ENSP00000433457 | 2.29E-06 | -0.1518 | 0 | 0.678 |
| ENSP00000262211 | 5.44E-06 | -0.1518 | 291 | 0.000 |
| ENSP00000410400 | 1.90E-06 | -0.1519 | 0 | 0.155 |
| ENSP00000412999 | 3.00E-06 | -0.1519 | 0 | 0.682 |
| ENSP00000334437 | 6.78E-06 | -0.1520 | 0 | 0.112 |
| ENSP00000369126 | 1.29E-05 | -0.1520 | 313 | 0.000 |
| ENSP00000375069 | 2.24E-05 | -0.1520 | 169 | 0.236 |
| ENSP00000260361 | 3.63E-06 | -0.1520 | 0 | 0.139 |
| ENSP00000385751 | 8.08E-06 | -0.1521 | 163 | 0.851 |
| ENSP00000432487 | 3.55E-06 | -0.1521 | 0 | 0.146 |
| ENSP00000431162 | 2.24E-06 | -0.1521 | 0 | 0.593 |
| ENSP00000353401 | 1.52E-05 | -0.1522 | 0 | 0.832 |
| ENSP00000360696 | 6.47E-06 | -0.1522 | 0 | 0.143 |
| ENSP00000351049 | 1.15E-05 | -0.1522 | 186 | 0.786 |
| ENSP00000362762 | 3.29E-06 | -0.1523 | 0 | 0.000 |
| ENSP00000351575 | 5.34E-06 | -0.1523 | 0 | 0.621 |
| ENSP00000382390 | 3.66E-06 | -0.1523 | 0 | 0.094 |
| ENSP00000362551 | 3.96E-06 | -0.1523 | 0 | 0.673 |
| ENSP00000356621 | 3.29E-06 | -0.1523 | 0 | 0.484 |
| ENSP00000462046 | 2.62E-06 | -0.1524 | 0 | 0.138 |
| ENSP00000327796 | 7.75E-06 | -0.1524 | 0 | 0.175 |
| ENSP00000323587 | 1.49E-05 | -0.1524 | 241 | 0.349 |
| ENSP00000235933 | 5.51E-06 | -0.1524 | 0 | 0.193 |
| ENSP00000329614 | 1.58E-05 | -0.1525 | 240 | 0.128 |
| ENSP00000349723 | 4.55E-06 | -0.1525 | 0 | 0.805 |
| ENSP00000265741 | 7.53E-06 | -0.1525 | 221 | 0.000 |
| ENSP00000375397 | 1.53E-06 | -0.1525 | 0 | 0.135 |

| | | | | |
|---|---|---|---|---|
| ENSP00000264037 | 1.98E-05 | -0.1525 | 211 | 0.275 |
| ENSP00000331479 | 5.50E-06 | -0.1526 | 0 | 0.135 |
| ENSP00000406293 | 2.01E-05 | -0.1526 | 168 | 0.876 |
| ENSP00000265431 | 8.38E-06 | -0.1526 | 374 | 0.665 |
| ENSP00000319323 | 5.41E-06 | -0.1526 | 0 | 0.167 |
| ENSP00000377411 | 3.01E-06 | -0.1527 | 0 | 0.116 |
| ENSP00000256196 | 4.05E-06 | -0.1527 | 451 | 0.614 |
| ENSP00000454561 | 1.87E-07 | -0.1527 | 0 | 0.000 |
| ENSP00000378409 | 2.29E-06 | -0.1527 | 0 | 0.120 |
| ENSP00000366387 | 8.08E-06 | -0.1527 | 153 | 0.147 |
| ENSP00000312770 | 1.93E-05 | -0.1528 | 0 | 0.282 |
| ENSP00000373304 | 2.59E-06 | -0.1528 | 0 | 0.118 |
| ENSP00000379030 | 1.98E-06 | -0.1528 | 0 | 0.158 |
| ENSP00000362456 | 2.66E-06 | -0.1528 | 0 | 0.506 |
| ENSP00000366084 | 7.94E-06 | -0.1528 | 160 | 0.400 |
| ENSP00000354720 | 1.62E-05 | -0.1528 | 185 | 0.797 |
| ENSP00000419471 | 1.40E-05 | -0.1529 | 340 | 0.000 |
| ENSP00000454253 | 4.12E-06 | -0.1529 | 0 | 0.134 |
| ENSP00000342709 | 3.64E-06 | -0.1529 | 0 | 0.265 |
| ENSP00000355084 | 8.83E-06 | -0.1529 | 189 | 0.155 |
| ENSP00000304336 | 2.01E-05 | -0.1530 | 242 | 0.501 |
| ENSP00000414906 | 2.56E-06 | -0.1530 | 0 | 0.215 |
| ENSP00000352413 | 5.98E-06 | -0.1530 | 0 | 0.100 |
| ENSP00000472832 | 1.86E-07 | -0.1530 | 0 | 0.000 |
| ENSP00000307132 | 2.14E-05 | -0.1530 | 301 | 0.121 |
| ENSP00000405032 | 6.23E-07 | -0.1530 | 0 | 0.172 |
| ENSP00000303178 | 4.57E-06 | -0.1531 | 163 | 0.000 |
| ENSP00000470526 | 1.58E-06 | -0.1531 | 0 | 0.000 |
| ENSP00000343129 | 1.21E-05 | -0.1532 | 576 | 0.228 |
| ENSP00000381932 | 1.01E-05 | -0.1532 | 189 | 0.692 |
| ENSP00000357927 | 2.31E-06 | -0.1533 | 0 | 0.113 |
| ENSP00000364037 | 5.58E-06 | -0.1533 | 0 | 0.777 |
| ENSP00000445868 | 1.05E-05 | -0.1533 | 270 | 0.404 |
| ENSP00000325638 | 4.42E-06 | -0.1533 | 0 | 0.107 |
| ENSP00000386212 | 1.24E-05 | -0.1533 | 193 | 0.389 |
| ENSP00000298715 | 1.61E-05 | -0.1533 | 234 | 0.000 |
| ENSP00000396954 | 6.75E-06 | -0.1534 | 345 | 0.747 |
| ENSP00000379201 | 1.78E-06 | -0.1534 | 0 | 0.000 |
| ENSP00000374212 | 1.95E-05 | -0.1535 | 196 | 0.945 |
| ENSP00000345491 | 4.56E-06 | -0.1535 | 0 | 0.127 |
| ENSP00000415774 | 2.65E-06 | -0.1535 | 0 | 0.700 |
| ENSP00000473153 | 2.44E-06 | -0.1535 | 0 | 0.168 |
| ENSP00000373347 | 9.53E-06 | -0.1535 | 153 | 0.565 |
| ENSP00000364782 | 3.53E-06 | -0.1536 | 0 | 0.058 |
| ENSP00000413163 | 3.58E-06 | -0.1536 | 0 | 0.132 |
| ENSP00000339168 | 1.81E-05 | -0.1536 | 0 | 0.112 |

| | | | | |
|---|---|---|---|---|
| ENSP00000467676 | 5.66E-06 | -0.1536 | 450 | 0.814 |
| ENSP00000331581 | 4.36E-06 | -0.1536 | 0 | 0.120 |
| ENSP00000301411 | 1.88E-05 | -0.1536 | 163 | 0.000 |
| ENSP00000373752 | 2.58E-06 | -0.1536 | 0 | 0.120 |
| ENSP00000416583 | 1.59E-05 | -0.1537 | 165 | 0.342 |
| ENSP00000381565 | 1.63E-05 | -0.1537 | 171 | 0.539 |
| ENSP00000365920 | 1.78E-05 | -0.1537 | 584 | 0.123 |
| ENSP00000363616 | 6.75E-06 | -0.1537 | 322 | 0.146 |
| ENSP00000330098 | 3.68E-06 | -0.1537 | 0 | 0.000 |
| ENSP00000403400 | 3.94E-06 | -0.1537 | 0 | 0.092 |
| ENSP00000420357 | 4.99E-06 | -0.1537 | 0 | 0.618 |
| ENSP00000425809 | 2.11E-05 | -0.1538 | 319 | 0.659 |
| ENSP00000286380 | 5.17E-06 | -0.1538 | 0 | 0.193 |
| ENSP00000368391 | 5.90E-06 | -0.1538 | 0 | 0.126 |
| ENSP00000408792 | 4.35E-06 | -0.1538 | 0 | 0.779 |
| ENSP00000370377 | 6.23E-06 | -0.1538 | 0 | 0.159 |
| ENSP00000394033 | 1.81E-05 | -0.1538 | 216 | 0.345 |
| ENSP00000251566 | 4.92E-06 | -0.1539 | 0 | 0.061 |
| ENSP00000362424 | 1.73E-05 | -0.1539 | 487 | 0.290 |
| ENSP00000363313 | 1.57E-05 | -0.1539 | 175 | 0.905 |
| ENSP00000454697 | 3.24E-07 | -0.1539 | 0 | 0.192 |
| ENSP00000362968 | 3.08E-06 | -0.1539 | 0 | 0.118 |
| ENSP00000346037 | 6.51E-06 | -0.1539 | 628 | 0.769 |
| ENSP00000470257 | 1.63E-06 | -0.1540 | 0 | 0.000 |
| ENSP00000372445 | 4.23E-06 | -0.1540 | 0 | 0.493 |
| ENSP00000366237 | 1.25E-05 | -0.1540 | 181 | 0.523 |
| ENSP00000350358 | 1.11E-05 | -0.1540 | 0 | 0.138 |
| ENSP00000464167 | 2.72E-07 | -0.1540 | 0 | 0.000 |
| ENSP00000356848 | 2.94E-06 | -0.1540 | 0 | 0.448 |
| ENSP00000375608 | 3.23E-06 | -0.1540 | 0 | 0.192 |
| ENSP00000365312 | 1.95E-05 | -0.1540 | 900 | 0.660 |
| ENSP00000264669 | 4.83E-06 | -0.1540 | 0 | 0.167 |
| ENSP00000356958 | 7.69E-06 | -0.1540 | 241 | 0.519 |
| ENSP00000430333 | 2.13E-05 | -0.1541 | 172 | 0.885 |
| ENSP00000331201 | 1.21E-05 | -0.1542 | 424 | 0.674 |
| ENSP00000472465 | 1.69E-05 | -0.1542 | 328 | 0.000 |
| ENSP00000343212 | 4.87E-06 | -0.1542 | 0 | 0.131 |
| ENSP00000370736 | 1.10E-05 | -0.1543 | 176 | 0.075 |
| ENSP00000376522 | 1.00E-05 | -0.1543 | 0 | 0.125 |
| ENSP00000345195 | 6.19E-06 | -0.1543 | 233 | 0.746 |
| ENSP00000411070 | 3.84E-07 | -0.1543 | 0 | 0.212 |
| ENSP00000368253 | 1.44E-05 | -0.1544 | 163 | 0.869 |
| ENSP00000358867 | 1.26E-05 | -0.1544 | 509 | 0.626 |
| ENSP00000360365 | 4.28E-06 | -0.1544 | 0 | 0.377 |
| ENSP00000262903 | 7.34E-06 | -0.1544 | 327 | 0.709 |
| ENSP00000354201 | 1.88E-05 | -0.1544 | 178 | 0.589 |

| | | | | |
|---|---|---|---|---|
| ENSP00000372023 | 1.60E-05 | -0.1545 | 163 | 0.803 |
| ENSP00000383216 | 4.23E-07 | -0.1545 | 0 | 0.151 |
| ENSP00000348786 | 1.77E-05 | -0.1545 | 933 | 0.598 |
| ENSP00000311405 | 1.05E-05 | -0.1545 | 0 | 0.644 |
| ENSP00000396620 | 6.49E-06 | -0.1546 | 910 | 0.000 |
| ENSP00000402038 | 1.07E-05 | -0.1547 | 319 | 0.583 |
| ENSP00000378306 | 1.87E-05 | -0.1547 | 224 | 0.657 |
| ENSP00000328487 | 6.20E-06 | -0.1547 | 0 | 0.114 |
| ENSP00000342952 | 1.73E-05 | -0.1547 | 200 | 0.648 |
| ENSP00000386962 | 1.96E-06 | -0.1548 | 0 | 0.124 |
| ENSP00000363242 | 1.29E-06 | -0.1548 | 0 | 0.170 |
| ENSP00000363115 | 2.20E-05 | -0.1548 | 307 | 0.662 |
| ENSP00000326267 | 3.87E-06 | -0.1548 | 153 | 0.652 |
| ENSP00000356549 | 5.86E-06 | -0.1548 | 0 | 0.413 |
| ENSP00000419539 | 1.54E-06 | -0.1548 | 0 | 0.183 |
| ENSP00000386895 | 4.37E-06 | -0.1549 | 0 | 0.118 |
| ENSP00000315635 | 1.14E-05 | -0.1549 | 0 | 0.098 |
| ENSP00000347498 | 3.23E-06 | -0.1549 | 0 | 0.670 |
| ENSP00000309126 | 1.96E-05 | -0.1549 | 204 | 0.830 |
| ENSP00000406970 | 1.17E-05 | -0.1549 | 157 | 0.327 |
| ENSP00000439467 | 1.84E-05 | -0.1549 | 264 | 0.526 |
| ENSP00000279544 | 7.41E-06 | -0.1550 | 0 | 0.000 |
| ENSP00000386139 | 1.64E-06 | -0.1550 | 0 | 0.000 |
| ENSP00000323880 | 6.90E-06 | -0.1550 | 0 | 0.000 |
| ENSP00000423014 | 1.67E-06 | -0.1550 | 0 | 0.131 |
| ENSP00000380270 | 1.99E-06 | -0.1550 | 0 | 0.141 |
| ENSP00000393667 | 3.68E-06 | -0.1550 | 0 | 0.078 |
| ENSP00000357097 | 1.35E-05 | -0.1551 | 260 | 0.425 |
| ENSP00000354376 | 2.18E-05 | -0.1551 | 313 | 0.576 |
| ENSP00000340594 | 4.87E-06 | -0.1552 | 0 | 0.105 |
| ENSP00000308774 | 5.35E-06 | -0.1552 | 205 | 0.674 |
| ENSP00000347942 | 2.73E-05 | -0.1552 | 342 | 0.707 |
| ENSP00000334767 | 4.85E-06 | -0.1552 | 0 | 0.160 |
| ENSP00000456272 | 1.85E-05 | -0.1552 | 174 | 0.099 |
| ENSP00000343290 | 1.03E-05 | -0.1552 | 0 | 0.000 |
| ENSP00000360317 | 7.71E-06 | -0.1552 | 161 | 0.287 |
| ENSP00000346001 | 1.66E-05 | -0.1552 | 479 | 0.858 |
| ENSP00000361740 | 3.96E-06 | -0.1553 | 0 | 0.158 |
| ENSP00000321519 | 1.24E-05 | -0.1553 | 0 | 0.146 |
| ENSP00000387564 | 1.34E-05 | -0.1553 | 165 | 0.477 |
| ENSP00000359630 | 1.64E-05 | -0.1553 | 195 | 0.000 |
| ENSP00000363573 | 2.58E-06 | -0.1554 | 0 | 0.974 |
| ENSP00000470082 | 2.33E-06 | -0.1554 | 0 | 0.250 |
| ENSP00000361993 | 1.19E-05 | -0.1554 | 0 | 0.350 |
| ENSP00000426978 | 1.70E-05 | -0.1554 | 237 | 0.710 |
| ENSP00000358635 | 7.81E-06 | -0.1554 | 0 | 0.804 |

| | | | | |
|---|---|---|---|---|
| ENSP00000347161 | 1.27E-05 | -0.1555 | 570 | 0.212 |
| ENSP00000376384 | 5.82E-06 | -0.1555 | 0 | 0.000 |
| ENSP00000445920 | 7.72E-06 | -0.1555 | 239 | 0.204 |
| ENSP00000362170 | 2.62E-06 | -0.1556 | 0 | 0.138 |
| ENSP00000370088 | 5.29E-06 | -0.1556 | 0 | 0.098 |
| ENSP00000364501 | 2.58E-06 | -0.1557 | 0 | 0.138 |
| ENSP00000381237 | 1.29E-05 | -0.1557 | 369 | 0.253 |
| ENSP00000375809 | 7.35E-06 | -0.1557 | 201 | 0.798 |
| ENSP00000431443 | 3.72E-06 | -0.1557 | 0 | 0.070 |
| ENSP00000438042 | 3.43E-06 | -0.1557 | 0 | 0.071 |
| ENSP00000378845 | 1.67E-05 | -0.1557 | 181 | 0.697 |
| ENSP00000405574 | 1.17E-05 | -0.1557 | 900 | 0.799 |
| ENSP00000371711 | 3.87E-06 | -0.1557 | 0 | 0.686 |
| ENSP00000379326 | 2.43E-05 | -0.1558 | 173 | 0.062 |
| ENSP00000363270 | 2.98E-06 | -0.1558 | 0 | 0.098 |
| ENSP00000376849 | 8.27E-06 | -0.1558 | 0 | 0.582 |
| ENSP00000414037 | 1.82E-06 | -0.1559 | 0 | 0.000 |
| ENSP00000307852 | 3.82E-06 | -0.1559 | 0 | 0.000 |
| ENSP00000431376 | 6.31E-06 | -0.1560 | 201 | 0.209 |
| ENSP00000334952 | 4.35E-06 | -0.1561 | 0 | 0.000 |
| ENSP00000417303 | 6.26E-06 | -0.1561 | 834 | 0.747 |
| ENSP00000383456 | 4.19E-06 | -0.1562 | 0 | 0.105 |
| ENSP00000314380 | 1.70E-05 | -0.1562 | 210 | 0.112 |
| ENSP00000356355 | 6.39E-06 | -0.1562 | 171 | 0.322 |
| ENSP00000369702 | 1.00E-06 | -0.1562 | 0 | 0.124 |
| ENSP00000379430 | 4.77E-06 | -0.1562 | 0 | 0.220 |
| ENSP00000403310 | 1.77E-05 | -0.1563 | 601 | 0.962 |
| ENSP00000359899 | 5.00E-06 | -0.1564 | 0 | 0.495 |
| ENSP00000314543 | 2.63E-06 | -0.1564 | 0 | 0.103 |
| ENSP00000382328 | 2.21E-06 | -0.1564 | 0 | 0.103 |
| ENSP00000469270 | 4.23E-08 | -0.1565 | 0 | 0.000 |
| ENSP00000387310 | 5.29E-06 | -0.1565 | 0 | 0.826 |
| ENSP00000348751 | 4.63E-06 | -0.1565 | 0 | 0.207 |
| ENSP00000309515 | 7.60E-06 | -0.1566 | 0 | 0.081 |
| ENSP00000370614 | 5.09E-06 | -0.1566 | 0 | 0.568 |
| ENSP00000430505 | 5.42E-06 | -0.1566 | 0 | 0.398 |
| ENSP00000256362 | 1.63E-06 | -0.1566 | 0 | 0.166 |
| ENSP00000371205 | 2.11E-06 | -0.1566 | 0 | 0.000 |
| ENSP00000455908 | 1.36E-06 | -0.1566 | 0 | 0.000 |
| ENSP00000388566 | 1.11E-05 | -0.1567 | 195 | 0.616 |
| ENSP00000367330 | 2.34E-06 | -0.1568 | 0 | 0.154 |
| ENSP00000386557 | 5.62E-07 | -0.1568 | 0 | 0.180 |
| ENSP00000300784 | 4.85E-06 | -0.1568 | 0 | 0.094 |
| ENSP00000368884 | 1.85E-05 | -0.1568 | 333 | 0.000 |
| ENSP00000351926 | 1.41E-05 | -0.1568 | 157 | 0.249 |
| ENSP00000362649 | 3.44E-05 | -0.1569 | 602 | 0.000 |

| | | | | |
|---|---|---|---|---|
| ENSP00000378735 | 8.50E-06 | -0.1569 | 668 | 0.706 |
| ENSP00000369600 | 1.36E-05 | -0.1569 | 860 | 0.254 |
| ENSP00000354511 | 2.09E-05 | -0.1569 | 509 | 0.711 |
| ENSP00000353953 | 4.16E-06 | -0.1570 | 0 | 0.061 |
| ENSP00000404132 | 4.28E-06 | -0.1570 | 0 | 0.000 |
| ENSP00000330612 | 9.79E-06 | -0.1570 | 0 | 0.175 |
| ENSP00000348419 | 3.72E-06 | -0.1570 | 0 | 0.723 |
| ENSP00000393393 | 3.25E-06 | -0.1571 | 0 | 0.804 |
| ENSP00000347733 | 1.40E-05 | -0.1571 | 206 | 0.819 |
| ENSP00000216445 | 2.87E-05 | -0.1571 | 0 | 0.137 |
| ENSP00000358400 | 3.69E-06 | -0.1572 | 0 | 0.781 |
| ENSP00000362578 | 6.72E-06 | -0.1572 | 163 | 0.805 |
| ENSP00000347271 | 3.59E-06 | -0.1572 | 0 | 0.790 |
| ENSP00000449428 | 1.87E-05 | -0.1572 | 150 | 0.328 |
| ENSP00000308575 | 3.39E-06 | -0.1573 | 0 | 0.110 |
| ENSP00000355865 | 2.89E-05 | -0.1573 | 455 | 0.880 |
| ENSP00000384183 | 1.21E-06 | -0.1573 | 0 | 0.146 |
| ENSP00000350673 | 5.24E-06 | -0.1573 | 0 | 0.137 |
| ENSP00000400513 | 2.45E-06 | -0.1573 | 0 | 0.134 |
| ENSP00000242275 | 4.80E-06 | -0.1574 | 0 | 0.101 |
| ENSP00000349679 | 1.06E-05 | -0.1574 | 163 | 0.085 |
| ENSP00000362018 | 3.57E-06 | -0.1574 | 0 | 0.512 |
| ENSP00000346017 | 1.55E-05 | -0.1574 | 0 | 0.447 |
| ENSP00000431265 | 2.85E-06 | -0.1575 | 0 | 0.688 |
| ENSP00000362758 | 3.36E-06 | -0.1575 | 0 | 0.651 |
| ENSP00000380544 | 9.22E-06 | -0.1576 | 0 | 0.089 |
| ENSP00000348337 | 2.83E-06 | -0.1576 | 0 | 0.000 |
| ENSP00000335307 | 2.43E-06 | -0.1576 | 0 | 0.090 |
| ENSP00000435634 | 1.71E-06 | -0.1576 | 0 | 0.152 |
| ENSP00000327197 | 3.34E-06 | -0.1576 | 0 | 0.443 |
| ENSP00000282507 | 4.04E-06 | -0.1576 | 0 | 0.068 |
| ENSP00000393511 | 9.38E-06 | -0.1577 | 0 | 0.134 |
| ENSP00000377971 | 3.92E-06 | -0.1577 | 0 | 0.164 |
| ENSP00000364764 | 2.54E-06 | -0.1577 | 0 | 0.104 |
| ENSP00000274766 | 6.01E-06 | -0.1577 | 0 | 0.221 |
| ENSP00000346697 | 5.81E-06 | -0.1577 | 0 | 0.321 |
| ENSP00000376871 | 1.96E-05 | -0.1577 | 244 | 0.000 |
| ENSP00000410925 | 9.92E-06 | -0.1578 | 302 | 0.187 |
| ENSP00000369257 | 1.80E-05 | -0.1578 | 301 | 0.392 |
| ENSP00000379847 | 8.89E-06 | -0.1578 | 206 | 0.708 |
| ENSP00000420140 | 8.38E-07 | -0.1578 | 0 | 0.176 |
| ENSP00000350856 | 5.40E-06 | -0.1579 | 0 | 0.117 |
| ENSP00000371219 | 2.81E-05 | -0.1579 | 240 | 0.584 |
| ENSP00000371940 | 3.64E-06 | -0.1579 | 0 | 0.000 |
| ENSP00000336861 | 3.34E-06 | -0.1580 | 0 | 0.182 |
| ENSP00000405635 | 3.08E-06 | -0.1580 | 0 | 0.065 |

| | | | | |
|---|---|---|---|---|
| ENSP00000446514 | 8.25E-08 | -0.1580 | 0 | 0.205 |
| ENSP00000328444 | 3.06E-06 | -0.1580 | 0 | 0.174 |
| ENSP00000340672 | 4.18E-06 | -0.1581 | 0 | 0.131 |
| ENSP00000359818 | 5.35E-06 | -0.1581 | 0 | 0.561 |
| ENSP00000363559 | 1.57E-05 | -0.1581 | 167 | 0.903 |
| ENSP00000379108 | 1.29E-05 | -0.1581 | 243 | 0.485 |
| ENSP00000264218 | 1.85E-05 | -0.1581 | 213 | 0.560 |
| ENSP00000381577 | 3.72E-06 | -0.1582 | 0 | 0.429 |
| ENSP00000380308 | 8.48E-06 | -0.1582 | 414 | 0.184 |
| ENSP00000335481 | 7.53E-06 | -0.1583 | 0 | 0.156 |
| ENSP00000421655 | 3.78E-06 | -0.1583 | 0 | 0.632 |
| ENSP00000384296 | 1.29E-05 | -0.1583 | 364 | 0.354 |
| ENSP00000384770 | 1.70E-06 | -0.1584 | 0 | 0.186 |
| ENSP00000400500 | 6.24E-06 | -0.1584 | 163 | 0.172 |
| ENSP00000310110 | 1.83E-05 | -0.1584 | 0 | 0.213 |
| ENSP00000462333 | 2.17E-06 | -0.1584 | 0 | 0.090 |
| ENSP00000345412 | 3.55E-06 | -0.1584 | 0 | 0.762 |
| ENSP00000415273 | 1.41E-06 | -0.1585 | 0 | 0.000 |
| ENSP00000377040 | 4.69E-06 | -0.1585 | 0 | 0.224 |
| ENSP00000328983 | 1.58E-05 | -0.1585 | 974 | 0.080 |
| ENSP00000420195 | 1.10E-05 | -0.1586 | 0 | 0.000 |
| ENSP00000350633 | 6.37E-06 | -0.1586 | 0 | 0.516 |
| ENSP00000382834 | 4.53E-06 | -0.1586 | 0 | 0.579 |
| ENSP00000367605 | 1.87E-05 | -0.1586 | 270 | 0.000 |
| ENSP00000382485 | 1.35E-05 | -0.1586 | 0 | 0.086 |
| ENSP00000376417 | 1.43E-05 | -0.1586 | 0 | 0.095 |
| ENSP00000452454 | 1.78E-05 | -0.1587 | 359 | 0.706 |
| ENSP00000351957 | 3.98E-06 | -0.1587 | 0 | 0.268 |
| ENSP00000242109 | 7.58E-06 | -0.1587 | 0 | 0.000 |
| ENSP00000301920 | 1.39E-05 | -0.1587 | 163 | 0.112 |
| ENSP00000358335 | 7.80E-06 | -0.1588 | 348 | 0.693 |
| ENSP00000382476 | 3.36E-06 | -0.1588 | 0 | 0.136 |
| ENSP00000300714 | 2.25E-05 | -0.1588 | 169 | 0.134 |
| ENSP00000304414 | 1.41E-05 | -0.1588 | 191 | 0.535 |
| ENSP00000409952 | 5.22E-06 | -0.1589 | 0 | 0.157 |
| ENSP00000329127 | 1.24E-05 | -0.1589 | 917 | 0.666 |
| ENSP00000412886 | 2.49E-06 | -0.1589 | 0 | 0.188 |
| ENSP00000246100 | 1.34E-05 | -0.1589 | 0 | 0.124 |
| ENSP00000399863 | 7.83E-08 | -0.1590 | 0 | 0.244 |
| ENSP00000375081 | 1.78E-05 | -0.1590 | 456 | 0.000 |
| ENSP00000333685 | 1.67E-05 | -0.1590 | 691 | 0.662 |
| ENSP00000442291 | 1.86E-06 | -0.1590 | 0 | 0.152 |
| ENSP00000323275 | 7.33E-06 | -0.1591 | 316 | 0.701 |
| ENSP00000317170 | 6.20E-06 | -0.1591 | 0 | 0.094 |
| ENSP00000394183 | 3.54E-06 | -0.1591 | 0 | 0.065 |
| ENSP00000464383 | 2.27E-05 | -0.1592 | 346 | 0.000 |

| | | | | |
|---|---|---|---|---|
| ENSP00000366829 | 4.52E-06 | -0.1592 | 0 | 0.343 |
| ENSP00000361124 | 4.02E-06 | -0.1593 | 0 | 0.000 |
| ENSP00000347206 | 1.62E-05 | -0.1593 | 191 | 0.091 |
| ENSP00000436376 | 2.27E-05 | -0.1593 | 281 | 0.468 |
| ENSP00000341171 | 1.61E-05 | -0.1593 | 159 | 0.129 |
| ENSP00000361790 | 3.57E-06 | -0.1593 | 0 | 0.658 |
| ENSP00000439444 | 9.20E-06 | -0.1593 | 272 | 0.411 |
| ENSP00000377655 | 3.18E-06 | -0.1594 | 0 | 0.491 |
| ENSP00000300187 | 6.84E-06 | -0.1594 | 0 | 0.113 |
| ENSP00000350876 | 1.95E-05 | -0.1594 | 293 | 0.000 |
| ENSP00000362617 | 1.91E-05 | -0.1594 | 326 | 0.000 |
| ENSP00000416290 | 2.43E-06 | -0.1594 | 0 | 0.124 |
| ENSP00000402962 | 5.18E-06 | -0.1594 | 0 | 0.000 |
| ENSP00000370021 | 2.42E-06 | -0.1595 | 0 | 0.156 |
| ENSP00000376127 | 7.08E-06 | -0.1595 | 416 | 0.000 |
| ENSP00000361982 | 1.25E-05 | -0.1595 | 355 | 0.221 |
| ENSP00000363667 | 6.56E-06 | -0.1595 | 270 | 0.505 |
| ENSP00000395772 | 1.35E-05 | -0.1595 | 295 | 0.000 |
| ENSP00000272519 | 5.37E-06 | -0.1595 | 448 | 0.598 |
| ENSP00000416662 | 4.63E-06 | -0.1596 | 0 | 0.669 |
| ENSP00000415662 | 9.42E-07 | -0.1596 | 0 | 0.158 |
| ENSP00000445508 | 9.55E-06 | -0.1596 | 167 | 0.551 |
| ENSP00000335357 | 3.16E-06 | -0.1597 | 0 | 0.000 |
| ENSP00000261643 | 7.90E-06 | -0.1597 | 307 | 0.323 |
| ENSP00000333018 | 7.48E-06 | -0.1597 | 316 | 0.702 |
| ENSP00000349132 | 9.76E-06 | -0.1597 | 0 | 0.000 |
| ENSP00000405210 | 2.43E-06 | -0.1597 | 0 | 0.175 |
| ENSP00000405700 | 4.02E-06 | -0.1597 | 0 | 0.000 |
| ENSP00000360112 | 2.56E-06 | -0.1597 | 0 | 0.239 |
| ENSP00000415517 | 7.21E-07 | -0.1597 | 0 | 0.118 |
| ENSP00000380869 | 8.41E-06 | -0.1597 | 204 | 0.502 |
| ENSP00000402515 | 1.69E-05 | -0.1598 | 165 | 0.744 |
| ENSP00000367276 | 4.88E-06 | -0.1598 | 0 | 0.258 |
| ENSP00000328939 | 1.27E-05 | -0.1598 | 316 | 0.740 |
| ENSP00000356832 | 1.69E-05 | -0.1598 | 416 | 0.674 |
| ENSP00000354398 | 1.28E-05 | -0.1598 | 191 | 0.413 |
| ENSP00000371546 | 6.12E-06 | -0.1599 | 0 | 0.091 |
| ENSP00000384889 | 7.31E-06 | -0.1599 | 0 | 0.688 |
| ENSP00000406861 | 1.21E-05 | -0.1599 | 168 | 0.456 |
| ENSP00000379008 | 3.61E-06 | -0.1599 | 0 | 0.101 |
| ENSP00000360245 | 7.58E-06 | -0.1599 | 0 | 0.000 |
| ENSP00000298630 | 6.97E-06 | -0.1599 | 277 | 0.000 |
| ENSP00000355264 | 7.10E-06 | -0.1600 | 0 | 0.234 |
| ENSP00000355064 | 2.01E-06 | -0.1600 | 0 | 0.197 |
| ENSP00000418707 | 3.58E-06 | -0.1600 | 0 | 0.135 |
| ENSP00000305355 | 7.87E-06 | -0.1600 | 168 | 0.671 |

| | | | | |
|---|---|---|---|---|
| ENSP00000397947 | 1.03E-05 | -0.1600 | 194 | 0.119 |
| ENSP00000363787 | 1.14E-05 | -0.1601 | 270 | 0.759 |
| ENSP00000378201 | 3.74E-06 | -0.1602 | 0 | 0.000 |
| ENSP00000406579 | 4.04E-07 | -0.1602 | 0 | 0.000 |
| ENSP00000354622 | 3.54E-06 | -0.1602 | 0 | 0.186 |
| ENSP00000380855 | 1.01E-05 | -0.1602 | 216 | 0.631 |
| ENSP00000454014 | 2.17E-06 | -0.1602 | 0 | 0.063 |
| ENSP00000350415 | 6.61E-06 | -0.1602 | 0 | 0.136 |
| ENSP00000388741 | 1.58E-06 | -0.1602 | 0 | 0.198 |
| ENSP00000232496 | 4.05E-05 | -0.1603 | 0 | 0.213 |
| ENSP00000320757 | 3.81E-06 | -0.1604 | 0 | 0.146 |
| ENSP00000286355 | 4.56E-06 | -0.1604 | 0 | 0.641 |
| ENSP00000352929 | 8.98E-06 | -0.1604 | 175 | 0.707 |
| ENSP00000419235 | 1.37E-06 | -0.1605 | 0 | 0.000 |
| ENSP00000362344 | 5.48E-06 | -0.1605 | 175 | 0.482 |
| ENSP00000450461 | 1.69E-06 | -0.1605 | 0 | 0.125 |
| ENSP00000354532 | 6.25E-06 | -0.1605 | 0 | 0.181 |
| ENSP00000360860 | 7.83E-06 | -0.1605 | 320 | 0.703 |
| ENSP00000307155 | 6.27E-06 | -0.1606 | 0 | 0.271 |
| ENSP00000409231 | 1.92E-06 | -0.1606 | 0 | 0.104 |
| ENSP00000333900 | 6.01E-06 | -0.1606 | 0 | 0.146 |
| ENSP00000432768 | 7.46E-06 | -0.1606 | 806 | 0.384 |
| ENSP00000368856 | 1.70E-05 | -0.1606 | 0 | 0.761 |
| ENSP00000384863 | 1.44E-05 | -0.1607 | 593 | 0.000 |
| ENSP00000418803 | 5.35E-06 | -0.1607 | 0 | 0.095 |
| ENSP00000371169 | 1.83E-05 | -0.1607 | 452 | 0.874 |
| ENSP00000370421 | 1.48E-05 | -0.1608 | 335 | 0.870 |
| ENSP00000289104 | 5.65E-06 | -0.1608 | 448 | 0.590 |
| ENSP00000414982 | 2.58E-05 | -0.1608 | 345 | 0.741 |
| ENSP00000356247 | 7.14E-06 | -0.1609 | 319 | 0.682 |
| ENSP00000274278 | 3.97E-06 | -0.1609 | 0 | 0.063 |
| ENSP00000359680 | 5.14E-06 | -0.1609 | 173 | 0.381 |
| ENSP00000375091 | 7.95E-07 | -0.1609 | 0 | 0.141 |
| ENSP00000395219 | 3.46E-06 | -0.1609 | 0 | 0.151 |
| ENSP00000332591 | 1.86E-05 | -0.1610 | 167 | 0.598 |
| ENSP00000387547 | 1.83E-06 | -0.1610 | 0 | 0.000 |
| ENSP00000317027 | 1.23E-05 | -0.1610 | 0 | 0.090 |
| ENSP00000363023 | 2.08E-06 | -0.1610 | 0 | 0.072 |
| ENSP00000333956 | 1.36E-05 | -0.1610 | 643 | 0.000 |
| ENSP00000406220 | 5.89E-06 | -0.1610 | 0 | 0.063 |
| ENSP00000308413 | 9.38E-06 | -0.1611 | 307 | 0.000 |
| ENSP00000383421 | 1.73E-06 | -0.1611 | 0 | 0.451 |
| ENSP00000433681 | 2.98E-06 | -0.1611 | 0 | 0.383 |
| ENSP00000315878 | 2.04E-05 | -0.1611 | 472 | 0.000 |
| ENSP00000345502 | 1.24E-05 | -0.1611 | 511 | 0.653 |
| ENSP00000364665 | 2.28E-06 | -0.1611 | 0 | 0.000 |

| | | | | |
|---|---|---|---|---|
| ENSP00000415836 | 4.68E-06 | -0.1612 | 0 | 0.690 |
| ENSP00000368305 | 9.38E-06 | -0.1613 | 245 | 0.103 |
| ENSP00000363768 | 1.46E-05 | -0.1613 | 222 | 0.158 |
| ENSP00000252032 | 4.40E-06 | -0.1613 | 0 | 0.247 |
| ENSP00000453089 | 1.80E-05 | -0.1613 | 242 | 0.000 |
| ENSP00000358695 | 1.16E-05 | -0.1613 | 283 | 0.486 |
| ENSP00000396527 | 4.92E-06 | -0.1614 | 167 | 0.000 |
| ENSP00000384040 | 4.31E-06 | -0.1614 | 0 | 0.087 |
| ENSP00000413861 | 3.01E-06 | -0.1614 | 0 | 0.131 |
| ENSP00000361036 | 2.62E-06 | -0.1614 | 0 | 0.172 |
| ENSP00000266031 | 1.62E-05 | -0.1614 | 0 | 0.086 |
| ENSP00000355050 | 1.04E-05 | -0.1614 | 158 | 0.239 |
| ENSP00000456832 | 3.68E-06 | -0.1614 | 0 | 0.256 |
| ENSP00000346762 | 3.70E-06 | -0.1614 | 0 | 0.659 |
| ENSP00000327467 | 1.02E-05 | -0.1615 | 0 | 0.136 |
| ENSP00000347322 | 3.33E-06 | -0.1615 | 0 | 0.164 |
| ENSP00000304697 | 1.59E-05 | -0.1615 | 477 | 0.691 |
| ENSP00000464872 | 2.19E-06 | -0.1615 | 0 | 0.694 |
| ENSP00000438526 | 4.17E-06 | -0.1615 | 0 | 0.118 |
| ENSP00000418744 | 1.68E-05 | -0.1616 | 482 | 0.229 |
| ENSP00000307853 | 3.28E-06 | -0.1616 | 0 | 0.709 |
| ENSP00000363433 | 4.89E-06 | -0.1616 | 0 | 0.345 |
| ENSP00000406145 | 4.65E-06 | -0.1616 | 0 | 0.151 |
| ENSP00000364589 | 1.86E-05 | -0.1617 | 218 | 0.748 |
| ENSP00000329200 | 2.63E-06 | -0.1617 | 0 | 0.244 |
| ENSP00000455258 | 2.82E-06 | -0.1617 | 0 | 0.000 |
| ENSP00000355518 | 1.25E-05 | -0.1617 | 369 | 0.637 |
| ENSP00000377374 | 4.66E-06 | -0.1617 | 0 | 0.114 |
| ENSP00000360379 | 3.35E-06 | -0.1617 | 0 | 0.189 |
| ENSP00000351416 | 1.74E-05 | -0.1618 | 202 | 0.649 |
| ENSP00000262848 | 4.15E-06 | -0.1618 | 587 | 0.647 |
| ENSP00000367248 | 1.04E-05 | -0.1618 | 179 | 0.000 |
| ENSP00000373298 | 2.15E-05 | -0.1618 | 420 | 0.640 |
| ENSP00000361048 | 2.30E-06 | -0.1618 | 0 | 0.138 |
| ENSP00000316590 | 1.63E-05 | -0.1618 | 201 | 0.000 |
| ENSP00000356000 | 1.56E-05 | -0.1618 | 207 | 0.627 |
| ENSP00000471914 | 1.52E-05 | -0.1619 | 179 | 0.000 |
| ENSP00000347778 | 3.28E-06 | -0.1619 | 0 | 0.062 |
| ENSP00000421985 | 1.11E-05 | -0.1619 | 0 | 0.122 |
| ENSP00000399511 | 1.61E-05 | -0.1619 | 314 | 0.452 |
| ENSP00000440485 | 6.72E-06 | -0.1620 | 340 | 0.557 |
| ENSP00000347872 | 1.24E-05 | -0.1620 | 0 | 0.140 |
| ENSP00000346599 | 4.59E-06 | -0.1620 | 0 | 0.632 |
| ENSP00000351325 | 6.86E-06 | -0.1620 | 0 | 0.146 |
| ENSP00000430276 | 1.86E-05 | -0.1620 | 318 | 0.757 |
| ENSP00000405176 | 1.66E-05 | -0.1620 | 230 | 0.871 |

| | | | | |
|---|---|---|---|---|
| ENSP00000347244 | 1.38E-05 | -0.1622 | 172 | 0.491 |
| ENSP00000334910 | 6.89E-06 | -0.1622 | 511 | 0.651 |
| ENSP00000446137 | 1.62E-09 | -0.1622 | 0 | 0.000 |
| ENSP00000387380 | 2.45E-06 | -0.1623 | 0 | 0.076 |
| ENSP00000433378 | 1.83E-06 | -0.1623 | 0 | 0.101 |
| ENSP00000369292 | 3.54E-06 | -0.1624 | 0 | 0.158 |
| ENSP00000368245 | 4.12E-06 | -0.1624 | 0 | 0.157 |
| ENSP00000370410 | 1.00E-05 | -0.1624 | 191 | 0.513 |
| ENSP00000373979 | 5.00E-06 | -0.1624 | 0 | 0.068 |
| ENSP00000357073 | 1.64E-05 | -0.1624 | 222 | 0.094 |
| ENSP00000358703 | 4.77E-06 | -0.1624 | 0 | 0.238 |
| ENSP00000312789 | 7.01E-06 | -0.1625 | 189 | 0.685 |
| ENSP00000352833 | 1.85E-05 | -0.1625 | 0 | 0.847 |
| ENSP00000362282 | 5.25E-06 | -0.1626 | 152 | 0.314 |
| ENSP00000356263 | 3.46E-06 | -0.1626 | 0 | 0.000 |
| ENSP00000411145 | 1.12E-05 | -0.1626 | 259 | 0.779 |
| ENSP00000386717 | 4.58E-06 | -0.1627 | 170 | 0.812 |
| ENSP00000323686 | 9.13E-06 | -0.1627 | 0 | 0.000 |
| ENSP00000384792 | 1.80E-05 | -0.1627 | 191 | 0.103 |
| ENSP00000390331 | 3.71E-06 | -0.1627 | 188 | 0.408 |
| ENSP00000368646 | 1.96E-05 | -0.1628 | 494 | 0.628 |
| ENSP00000441000 | 1.26E-05 | -0.1628 | 490 | 0.629 |
| ENSP00000436318 | 5.08E-06 | -0.1628 | 311 | 0.406 |
| ENSP00000354677 | 6.40E-06 | -0.1628 | 684 | 0.238 |
| ENSP00000376307 | 3.30E-06 | -0.1628 | 0 | 0.140 |
| ENSP00000337209 | 2.47E-06 | -0.1629 | 0 | 0.146 |
| ENSP00000380041 | 3.46E-06 | -0.1629 | 0 | 0.000 |
| ENSP00000445905 | 1.92E-06 | -0.1629 | 0 | 0.106 |
| ENSP00000425634 | 1.67E-05 | -0.1629 | 387 | 0.755 |
| ENSP00000311778 | 6.06E-06 | -0.1629 | 0 | 0.746 |
| ENSP00000345917 | 3.55E-06 | -0.1629 | 0 | 0.864 |
| ENSP00000375691 | 3.38E-06 | -0.1629 | 0 | 0.000 |
| ENSP00000321424 | 1.68E-05 | -0.1629 | 270 | 0.226 |
| ENSP00000357697 | 1.79E-05 | -0.1630 | 150 | 0.226 |
| ENSP00000346437 | 7.85E-06 | -0.1630 | 508 | 0.628 |
| ENSP00000436042 | 3.23E-06 | -0.1630 | 0 | 0.078 |
| ENSP00000328111 | 8.81E-06 | -0.1631 | 0 | 0.600 |
| ENSP00000438204 | 2.67E-06 | -0.1631 | 0 | 0.103 |
| ENSP00000363942 | 1.92E-05 | -0.1631 | 169 | 0.187 |
| ENSP00000353717 | 4.23E-06 | -0.1632 | 0 | 0.110 |
| ENSP00000343244 | 4.76E-06 | -0.1632 | 0 | 0.673 |
| ENSP00000465432 | 2.43E-06 | -0.1632 | 0 | 0.137 |
| ENSP00000348145 | 1.32E-05 | -0.1632 | 0 | 0.236 |
| ENSP00000368165 | 3.05E-06 | -0.1632 | 0 | 0.107 |
| ENSP00000367991 | 5.84E-06 | -0.1632 | 0 | 0.300 |
| ENSP00000440045 | 1.79E-05 | -0.1632 | 417 | 0.641 |

| | | | | |
|---|---|---|---|---|
| ENSP00000319052 | 3.15E-06 | -0.1633 | 0 | 0.643 |
| ENSP00000466420 | 1.60E-05 | -0.1633 | 179 | 0.000 |
| ENSP00000295622 | 2.81E-06 | -0.1633 | 0 | 0.227 |
| ENSP00000383594 | 1.95E-06 | -0.1633 | 0 | 0.123 |
| ENSP00000401191 | 2.20E-05 | -0.1633 | 225 | 0.685 |
| ENSP00000358719 | 1.29E-05 | -0.1633 | 284 | 0.459 |
| ENSP00000348442 | 8.34E-06 | -0.1633 | 150 | 0.523 |
| ENSP00000356096 | 7.93E-06 | -0.1634 | 0 | 0.133 |
| ENSP00000403067 | 4.28E-06 | -0.1634 | 0 | 0.066 |
| ENSP00000396995 | 8.72E-06 | -0.1634 | 0 | 0.862 |
| ENSP00000246020 | 5.53E-05 | -0.1634 | 0 | 0.239 |
| ENSP00000357838 | 1.10E-05 | -0.1635 | 197 | 0.501 |
| ENSP00000361345 | 3.58E-06 | -0.1635 | 0 | 0.117 |
| ENSP00000333896 | 7.24E-06 | -0.1635 | 289 | 0.000 |
| ENSP00000359042 | 4.17E-06 | -0.1635 | 0 | 0.513 |
| ENSP00000449784 | 4.74E-06 | -0.1635 | 0 | 0.141 |
| ENSP00000466519 | 2.97E-06 | -0.1636 | 0 | 0.000 |
| ENSP00000359240 | 4.72E-06 | -0.1636 | 0 | 0.128 |
| ENSP00000357175 | 1.34E-05 | -0.1636 | 167 | 0.779 |
| ENSP00000423563 | 7.55E-06 | -0.1636 | 201 | 0.964 |
| ENSP00000418693 | 4.03E-06 | -0.1636 | 0 | 0.141 |
| ENSP00000448323 | 1.41E-05 | -0.1637 | 330 | 0.000 |
| ENSP00000365255 | 9.17E-06 | -0.1637 | 202 | 0.646 |
| ENSP00000450909 | 1.32E-05 | -0.1637 | 0 | 0.969 |
| ENSP00000371318 | 2.43E-06 | -0.1637 | 0 | 0.149 |
| ENSP00000427108 | 2.76E-06 | -0.1638 | 0 | 0.322 |
| ENSP00000364363 | 2.77E-06 | -0.1638 | 0 | 0.133 |
| ENSP00000354645 | 5.28E-06 | -0.1638 | 0 | 0.100 |
| ENSP00000442411 | 4.03E-07 | -0.1638 | 0 | 0.000 |
| ENSP00000388648 | 1.27E-05 | -0.1638 | 830 | 0.741 |
| ENSP00000361076 | 1.72E-05 | -0.1638 | 297 | 0.916 |
| ENSP00000299732 | 4.86E-06 | -0.1639 | 0 | 0.140 |
| ENSP00000379712 | 3.91E-06 | -0.1639 | 0 | 0.135 |
| ENSP00000419279 | 2.31E-06 | -0.1639 | 0 | 0.063 |
| ENSP00000419449 | 1.75E-05 | -0.1639 | 614 | 0.790 |
| ENSP00000348054 | 8.38E-06 | -0.1640 | 297 | 0.098 |
| ENSP00000365005 | 5.91E-06 | -0.1640 | 230 | 0.647 |
| ENSP00000356541 | 3.95E-06 | -0.1640 | 0 | 0.841 |
| ENSP00000380675 | 4.38E-06 | -0.1640 | 0 | 0.663 |
| ENSP00000351214 | 3.54E-06 | -0.1641 | 0 | 0.159 |
| ENSP00000378367 | 4.33E-06 | -0.1641 | 0 | 0.327 |
| ENSP00000406541 | 3.37E-07 | -0.1641 | 0 | 0.159 |
| ENSP00000409318 | 4.16E-06 | -0.1641 | 0 | 0.685 |
| ENSP00000293404 | 1.65E-05 | -0.1642 | 181 | 0.427 |
| ENSP00000432353 | 2.46E-06 | -0.1642 | 0 | 0.115 |
| ENSP00000332293 | 5.72E-06 | -0.1643 | 0 | 0.822 |

| | | | | |
|---|---|---|---|---|
| ENSP00000351981 | 1.03E-05 | -0.1643 | 241 | 0.102 |
| ENSP00000254627 | 2.18E-05 | -0.1643 | 292 | 0.000 |
| ENSP00000354193 | 4.52E-06 | -0.1643 | 0 | 0.000 |
| ENSP00000384683 | 7.62E-07 | -0.1644 | 0 | 0.137 |
| ENSP00000339826 | 1.28E-05 | -0.1644 | 0 | 0.641 |
| ENSP00000460885 | 4.22E-06 | -0.1644 | 0 | 0.068 |
| ENSP00000359034 | 1.70E-06 | -0.1644 | 0 | 0.142 |
| ENSP00000415436 | 3.51E-06 | -0.1644 | 0 | 0.222 |
| ENSP00000316909 | 5.04E-06 | -0.1644 | 0 | 0.259 |
| ENSP00000318999 | 9.28E-06 | -0.1645 | 0 | 0.106 |
| ENSP00000358417 | 4.78E-06 | -0.1645 | 0 | 0.624 |
| ENSP00000346283 | 4.40E-06 | -0.1645 | 0 | 0.175 |
| ENSP00000332215 | 5.49E-06 | -0.1645 | 0 | 0.218 |
| ENSP00000326031 | 1.23E-05 | -0.1645 | 194 | 0.529 |
| ENSP00000451211 | 3.98E-06 | -0.1645 | 0 | 0.119 |
| ENSP00000319897 | 3.49E-06 | -0.1645 | 0 | 0.099 |
| ENSP00000279168 | 1.25E-05 | -0.1645 | 0 | 0.161 |
| ENSP00000358474 | 8.94E-06 | -0.1646 | 288 | 0.602 |
| ENSP00000370479 | 3.15E-06 | -0.1646 | 0 | 0.176 |
| ENSP00000369739 | 3.80E-06 | -0.1647 | 0 | 0.208 |
| ENSP00000399259 | 2.32E-06 | -0.1647 | 0 | 0.167 |
| ENSP00000364860 | 1.65E-05 | -0.1647 | 287 | 0.820 |
| ENSP00000351789 | 4.95E-06 | -0.1647 | 0 | 0.368 |
| ENSP00000265403 | 4.61E-06 | -0.1647 | 0 | 0.062 |
| ENSP00000342520 | 1.84E-05 | -0.1647 | 197 | 0.419 |
| ENSP00000381488 | 3.15E-07 | -0.1648 | 0 | 0.159 |
| ENSP00000422293 | 2.23E-06 | -0.1648 | 0 | 0.169 |
| ENSP00000343027 | 1.16E-05 | -0.1648 | 511 | 0.623 |
| ENSP00000369131 | 1.63E-05 | -0.1649 | 285 | 0.000 |
| ENSP00000418511 | 1.68E-06 | -0.1649 | 0 | 0.232 |
| ENSP00000389813 | 2.00E-05 | -0.1649 | 179 | 0.133 |
| ENSP00000320303 | 5.06E-06 | -0.1650 | 0 | 0.093 |
| ENSP00000362550 | 8.07E-06 | -0.1650 | 0 | 0.000 |
| ENSP00000402389 | 2.84E-06 | -0.1650 | 0 | 0.675 |
| ENSP00000399903 | 2.71E-06 | -0.1650 | 0 | 0.113 |
| ENSP00000359035 | 1.39E-05 | -0.1650 | 201 | 0.645 |
| ENSP00000473036 | 1.72E-05 | -0.1651 | 497 | 0.531 |
| ENSP00000320247 | 1.54E-05 | -0.1651 | 193 | 0.218 |
| ENSP00000331556 | 6.73E-06 | -0.1651 | 0 | 0.086 |
| ENSP00000368266 | 4.86E-06 | -0.1651 | 0 | 0.254 |
| ENSP00000296343 | 5.37E-06 | -0.1651 | 0 | 0.000 |
| ENSP00000404923 | 7.37E-06 | -0.1651 | 0 | 0.080 |
| ENSP00000431987 | 2.44E-06 | -0.1651 | 0 | 0.264 |
| ENSP00000357086 | 3.52E-06 | -0.1652 | 0 | 0.153 |
| ENSP00000398930 | 1.98E-05 | -0.1652 | 224 | 0.307 |
| ENSP00000361392 | 8.52E-06 | -0.1652 | 0 | 0.126 |

| | | | | |
|---|---|---|---|---|
| ENSP00000395281 | 3.31E-06 | -0.1652 | 0 | 0.145 |
| ENSP00000387654 | 1.53E-05 | -0.1652 | 0 | 0.090 |
| ENSP00000356231 | 2.78E-06 | -0.1653 | 0 | 0.219 |
| ENSP00000417003 | 2.03E-05 | -0.1653 | 167 | 0.250 |
| ENSP00000307951 | 1.58E-05 | -0.1653 | 0 | 0.161 |
| ENSP00000421689 | 2.67E-05 | -0.1653 | 256 | 0.000 |
| ENSP00000415564 | 4.23E-06 | -0.1654 | 0 | 0.143 |
| ENSP00000354255 | 1.39E-05 | -0.1654 | 178 | 0.126 |
| ENSP00000385036 | 4.59E-06 | -0.1655 | 0 | 0.112 |
| ENSP00000369134 | 1.64E-05 | -0.1655 | 262 | 0.445 |
| ENSP00000355090 | 3.24E-06 | -0.1655 | 0 | 0.291 |
| ENSP00000362616 | 1.93E-05 | -0.1655 | 326 | 0.000 |
| ENSP00000346155 | 1.53E-05 | -0.1655 | 578 | 0.520 |
| ENSP00000377570 | 3.10E-06 | -0.1656 | 0 | 0.090 |
| ENSP00000277526 | 1.65E-05 | -0.1656 | 0 | 0.126 |
| ENSP00000375810 | 1.08E-05 | -0.1656 | 316 | 0.524 |
| ENSP00000391676 | 1.88E-05 | -0.1656 | 281 | 0.671 |
| ENSP00000295569 | 1.20E-05 | -0.1657 | 0 | 0.136 |
| ENSP00000359529 | 2.76E-06 | -0.1657 | 0 | 0.000 |
| ENSP00000371475 | 1.85E-05 | -0.1658 | 484 | 0.881 |
| ENSP00000298999 | 1.40E-05 | -0.1658 | 325 | 0.148 |
| ENSP00000432931 | 2.29E-06 | -0.1659 | 0 | 0.107 |
| ENSP00000367319 | 2.15E-06 | -0.1659 | 0 | 0.107 |
| ENSP00000387209 | 1.87E-06 | -0.1659 | 0 | 0.131 |
| ENSP00000368758 | 1.72E-06 | -0.1659 | 0 | 0.113 |
| ENSP00000258776 | 4.03E-06 | -0.1659 | 0 | 0.000 |
| ENSP00000360017 | 4.32E-06 | -0.1659 | 0 | 0.740 |
| ENSP00000381476 | 5.01E-06 | -0.1659 | 0 | 0.099 |
| ENSP00000432870 | 2.14E-06 | -0.1660 | 0 | 0.129 |
| ENSP00000387535 | 3.25E-06 | -0.1660 | 0 | 0.152 |
| ENSP00000265068 | 8.87E-06 | -0.1660 | 0 | 0.195 |
| ENSP00000469305 | 1.17E-06 | -0.1660 | 0 | 0.000 |
| ENSP00000432034 | 4.85E-06 | -0.1660 | 0 | 0.000 |
| ENSP00000360497 | 1.54E-05 | -0.1660 | 230 | 0.772 |
| ENSP00000469582 | 3.51E-06 | -0.1661 | 0 | 0.686 |
| ENSP00000374125 | 1.57E-06 | -0.1661 | 0 | 0.172 |
| ENSP00000394484 | 1.36E-05 | -0.1661 | 508 | 0.747 |
| ENSP00000264705 | 8.91E-06 | -0.1661 | 681 | 0.000 |
| ENSP00000357701 | 1.22E-05 | -0.1662 | 0 | 0.162 |
| ENSP00000370222 | 4.80E-06 | -0.1662 | 0 | 0.348 |
| ENSP00000414712 | 4.99E-06 | -0.1662 | 0 | 0.088 |
| ENSP00000454699 | 1.70E-05 | -0.1662 | 343 | 0.000 |
| ENSP00000404432 | 5.07E-06 | -0.1662 | 0 | 0.248 |
| ENSP00000368797 | 2.86E-06 | -0.1663 | 0 | 0.132 |
| ENSP00000456333 | 9.24E-07 | -0.1663 | 0 | 0.158 |
| ENSP00000380888 | 1.58E-05 | -0.1663 | 216 | 0.707 |

| | | | | |
|---|---|---|---|---|
| ENSP00000395232 | 1.88E-06 | -0.1663 | 0 | 0.084 |
| ENSP00000461728 | 2.64E-06 | -0.1663 | 0 | 0.000 |
| ENSP00000304004 | 4.10E-06 | -0.1663 | 0 | 0.000 |
| ENSP00000355086 | 1.34E-05 | -0.1664 | 213 | 0.413 |
| ENSP00000371085 | 1.70E-06 | -0.1664 | 0 | 0.150 |
| ENSP00000315662 | 4.95E-06 | -0.1664 | 0 | 0.000 |
| ENSP00000309285 | 8.25E-06 | -0.1664 | 0 | 0.154 |
| ENSP00000356925 | 7.86E-06 | -0.1664 | 0 | 0.129 |
| ENSP00000367869 | 3.29E-05 | -0.1664 | 928 | 0.000 |
| ENSP00000416829 | 2.73E-06 | -0.1665 | 0 | 0.136 |
| ENSP00000232607 | 4.43E-06 | -0.1665 | 551 | 0.000 |
| ENSP00000328017 | 2.56E-06 | -0.1665 | 0 | 0.161 |
| ENSP00000375479 | 3.88E-07 | -0.1666 | 0 | 0.161 |
| ENSP00000346598 | 5.39E-06 | -0.1666 | 452 | 0.000 |
| ENSP00000269582 | 1.75E-05 | -0.1667 | 216 | 0.538 |
| ENSP00000406738 | 1.58E-05 | -0.1667 | 167 | 0.000 |
| ENSP00000316609 | 5.02E-06 | -0.1667 | 0 | 0.100 |
| ENSP00000370258 | 1.46E-05 | -0.1667 | 429 | 0.913 |
| ENSP00000340274 | 7.05E-06 | -0.1667 | 448 | 0.597 |
| ENSP00000361084 | 3.88E-06 | -0.1668 | 0 | 0.464 |
| ENSP00000358854 | 1.49E-05 | -0.1668 | 150 | 0.852 |
| ENSP00000359583 | 1.43E-05 | -0.1668 | 459 | 0.105 |
| ENSP00000379057 | 1.15E-06 | -0.1668 | 0 | 0.000 |
| ENSP00000410157 | 5.24E-06 | -0.1668 | 0 | 0.180 |
| ENSP00000317445 | 8.90E-06 | -0.1668 | 0 | 0.188 |
| ENSP00000410833 | 7.47E-06 | -0.1668 | 174 | 0.359 |
| ENSP00000373860 | 4.52E-06 | -0.1668 | 0 | 0.177 |
| ENSP00000413697 | 1.79E-05 | -0.1668 | 197 | 0.215 |
| ENSP00000392726 | 8.54E-07 | -0.1668 | 0 | 0.125 |
| ENSP00000285273 | 1.73E-05 | -0.1669 | 268 | 0.115 |
| ENSP00000410481 | 2.57E-06 | -0.1669 | 0 | 0.149 |
| ENSP00000262283 | 1.83E-05 | -0.1669 | 224 | 0.092 |
| ENSP00000386306 | 5.19E-06 | -0.1669 | 0 | 0.405 |
| ENSP00000381468 | 1.73E-06 | -0.1670 | 0 | 0.073 |
| ENSP00000363524 | 3.23E-06 | -0.1670 | 0 | 0.306 |
| ENSP00000360437 | 4.29E-06 | -0.1670 | 0 | 0.152 |
| ENSP00000415032 | 4.52E-06 | -0.1670 | 0 | 0.000 |
| ENSP00000347744 | 6.73E-06 | -0.1670 | 0 | 0.000 |
| ENSP00000358953 | 1.26E-05 | -0.1671 | 0 | 0.084 |
| ENSP00000364207 | 4.40E-06 | -0.1672 | 0 | 0.214 |
| ENSP00000265362 | 1.64E-05 | -0.1672 | 271 | 0.785 |
| ENSP00000442159 | 1.87E-06 | -0.1672 | 0 | 0.141 |
| ENSP00000349658 | 1.25E-05 | -0.1672 | 343 | 0.607 |
| ENSP00000382883 | 1.98E-06 | -0.1673 | 0 | 0.258 |
| ENSP00000381005 | 2.13E-06 | -0.1673 | 0 | 0.168 |
| ENSP00000331857 | 6.46E-06 | -0.1674 | 0 | 0.088 |

| | | | | |
|---|---|---|---|---|
| ENSP00000416683 | 8.54E-06 | -0.1674 | 0 | 0.729 |
| ENSP00000430620 | 1.04E-05 | -0.1674 | 178 | 0.223 |
| ENSP00000263182 | 6.93E-06 | -0.1674 | 163 | 0.674 |
| ENSP00000349076 | 6.02E-06 | -0.1674 | 0 | 0.785 |
| ENSP00000451382 | 1.92E-06 | -0.1674 | 0 | 0.000 |
| ENSP00000367024 | 4.64E-06 | -0.1675 | 0 | 0.340 |
| ENSP00000308926 | 3.74E-06 | -0.1675 | 0 | 0.107 |
| ENSP00000366582 | 1.01E-06 | -0.1675 | 0 | 0.111 |
| ENSP00000417580 | 1.23E-06 | -0.1675 | 0 | 0.097 |
| ENSP00000360247 | 1.12E-05 | -0.1675 | 841 | 0.260 |
| ENSP00000312673 | 1.84E-05 | -0.1676 | 191 | 0.686 |
| ENSP00000381008 | 6.19E-06 | -0.1677 | 0 | 0.118 |
| ENSP00000425845 | 1.20E-05 | -0.1678 | 227 | 0.824 |
| ENSP00000357025 | 1.59E-05 | -0.1678 | 158 | 0.740 |
| ENSP00000358851 | 5.85E-06 | -0.1678 | 0 | 0.120 |
| ENSP00000358957 | 1.50E-05 | -0.1678 | 200 | 0.000 |
| ENSP00000357904 | 5.19E-06 | -0.1678 | 0 | 0.144 |
| ENSP00000254235 | 2.63E-06 | -0.1678 | 0 | 0.645 |
| ENSP00000353650 | 8.76E-06 | -0.1678 | 0 | 0.290 |
| ENSP00000406327 | 3.29E-06 | -0.1678 | 0 | 0.095 |
| ENSP00000395848 | 8.40E-07 | -0.1679 | 0 | 0.000 |
| ENSP00000350911 | 1.31E-05 | -0.1679 | 314 | 0.228 |
| ENSP00000371393 | 1.45E-05 | -0.1680 | 467 | 0.585 |
| ENSP00000355045 | 3.74E-06 | -0.1680 | 0 | 0.094 |
| ENSP00000375063 | 2.41E-06 | -0.1680 | 0 | 0.154 |
| ENSP00000417128 | 1.14E-06 | -0.1680 | 0 | 0.121 |
| ENSP00000355652 | 2.54E-05 | -0.1681 | 725 | 0.574 |
| ENSP00000289921 | 4.46E-06 | -0.1681 | 0 | 0.101 |
| ENSP00000370607 | 1.02E-05 | -0.1681 | 181 | 0.166 |
| ENSP00000409346 | 2.49E-05 | -0.1681 | 216 | 0.917 |
| ENSP00000382863 | 2.05E-05 | -0.1682 | 196 | 0.922 |
| ENSP00000362330 | 1.79E-05 | -0.1682 | 196 | 0.928 |
| ENSP00000433282 | 1.96E-06 | -0.1682 | 0 | 0.000 |
| ENSP00000367638 | 1.80E-06 | -0.1682 | 0 | 0.272 |
| ENSP00000334197 | 5.87E-06 | -0.1682 | 0 | 0.151 |
| ENSP00000360372 | 7.70E-06 | -0.1682 | 241 | 0.307 |
| ENSP00000402608 | 1.21E-05 | -0.1682 | 504 | 0.716 |
| ENSP00000346810 | 5.11E-06 | -0.1684 | 0 | 0.788 |
| ENSP00000432786 | 3.62E-06 | -0.1684 | 0 | 0.000 |
| ENSP00000371787 | 3.92E-06 | -0.1684 | 0 | 0.000 |
| ENSP00000368720 | 4.82E-06 | -0.1684 | 0 | 0.174 |
| ENSP00000472066 | 6.09E-07 | -0.1684 | 0 | 0.000 |
| ENSP00000366093 | 1.00E-05 | -0.1685 | 159 | 0.806 |
| ENSP00000348380 | 4.08E-06 | -0.1686 | 0 | 0.180 |
| ENSP00000371682 | 2.21E-06 | -0.1686 | 0 | 0.173 |
| ENSP00000320849 | 6.72E-06 | -0.1686 | 0 | 0.000 |

| | | | | |
|---|---|---|---|---|
| ENSP00000453153 | 4.12E-06 | -0.1687 | 0 | 0.160 |
| ENSP00000355299 | 1.27E-06 | -0.1687 | 0 | 0.086 |
| ENSP00000357594 | 1.53E-05 | -0.1687 | 161 | 0.137 |
| ENSP00000357615 | 2.01E-05 | -0.1687 | 248 | 0.000 |
| ENSP00000385706 | 1.80E-06 | -0.1689 | 0 | 0.176 |
| ENSP00000360869 | 1.58E-05 | -0.1689 | 360 | 0.706 |
| ENSP00000386105 | 3.34E-06 | -0.1689 | 0 | 0.620 |
| ENSP00000406027 | 3.57E-06 | -0.1690 | 0 | 0.112 |
| ENSP00000349824 | 3.32E-06 | -0.1690 | 0 | 0.080 |
| ENSP00000405932 | 3.83E-06 | -0.1690 | 0 | 0.000 |
| ENSP00000410447 | 4.42E-06 | -0.1690 | 0 | 0.711 |
| ENSP00000417569 | 1.07E-05 | -0.1690 | 0 | 0.134 |
| ENSP00000431254 | 9.49E-06 | -0.1691 | 254 | 0.393 |
| ENSP00000333208 | 1.51E-05 | -0.1691 | 0 | 0.112 |
| ENSP00000385215 | 6.27E-06 | -0.1692 | 239 | 0.000 |
| ENSP00000366819 | 4.33E-06 | -0.1692 | 0 | 0.451 |
| ENSP00000355533 | 1.83E-05 | -0.1692 | 234 | 0.584 |
| ENSP00000258807 | 1.18E-05 | -0.1692 | 158 | 0.260 |
| ENSP00000391950 | 1.09E-05 | -0.1692 | 202 | 0.606 |
| ENSP00000362513 | 4.42E-06 | -0.1692 | 0 | 0.154 |
| ENSP00000332313 | 3.59E-06 | -0.1693 | 0 | 0.000 |
| ENSP00000331242 | 1.81E-05 | -0.1693 | 290 | 0.542 |
| ENSP00000411645 | 1.91E-06 | -0.1694 | 0 | 0.123 |
| ENSP00000388299 | 2.61E-06 | -0.1694 | 0 | 0.264 |
| ENSP00000409667 | 2.75E-05 | -0.1694 | 414 | 0.401 |
| ENSP00000463517 | 3.29E-06 | -0.1694 | 0 | 0.148 |
| ENSP00000378328 | 5.11E-06 | -0.1694 | 0 | 0.084 |
| ENSP00000418018 | 9.80E-06 | -0.1694 | 216 | 0.180 |
| ENSP00000349732 | 4.32E-06 | -0.1694 | 0 | 0.106 |
| ENSP00000374274 | 3.68E-06 | -0.1694 | 0 | 0.183 |
| ENSP00000356113 | 5.05E-06 | -0.1695 | 0 | 0.240 |
| ENSP00000301057 | 1.01E-05 | -0.1695 | 0 | 0.124 |
| ENSP00000300811 | 7.70E-06 | -0.1696 | 0 | 0.104 |
| ENSP00000419765 | 6.34E-06 | -0.1696 | 0 | 0.505 |
| ENSP00000457522 | 2.28E-05 | -0.1696 | 327 | 0.236 |
| ENSP00000354927 | 1.66E-05 | -0.1696 | 343 | 0.664 |
| ENSP00000349298 | 8.81E-06 | -0.1696 | 215 | 0.263 |
| ENSP00000382544 | 1.85E-05 | -0.1697 | 482 | 0.676 |
| ENSP00000367172 | 5.05E-06 | -0.1697 | 0 | 0.097 |
| ENSP00000418735 | 1.60E-05 | -0.1697 | 286 | 0.319 |
| ENSP00000251203 | 1.63E-05 | -0.1697 | 198 | 0.812 |
| ENSP00000303153 | 1.80E-05 | -0.1697 | 227 | 0.706 |
| ENSP00000398342 | 2.72E-06 | -0.1697 | 0 | 0.124 |
| ENSP00000426225 | 3.16E-06 | -0.1698 | 0 | 0.000 |
| ENSP00000397679 | 5.81E-06 | -0.1698 | 0 | 0.100 |
| ENSP00000400088 | 2.43E-05 | -0.1698 | 271 | 0.000 |

| | | | | |
|---|---|---|---|---|
| ENSP00000364467 | 3.78E-06 | -0.1698 | 0 | 0.081 |
| ENSP00000361894 | 5.86E-06 | -0.1698 | 0 | 0.239 |
| ENSP00000433290 | 1.58E-05 | -0.1699 | 284 | 0.522 |
| ENSP00000300093 | 1.67E-05 | -0.1699 | 244 | 0.000 |
| ENSP00000371813 | 2.57E-06 | -0.1699 | 0 | 0.170 |
| ENSP00000390941 | 1.59E-06 | -0.1699 | 0 | 0.114 |
| ENSP00000372192 | 2.43E-07 | -0.1699 | 0 | 0.000 |
| ENSP00000364265 | 4.88E-06 | -0.1699 | 0 | 0.000 |
| ENSP00000360252 | 8.10E-06 | -0.1699 | 0 | 0.048 |
| ENSP00000411658 | 2.11E-05 | -0.1700 | 544 | 0.635 |
| ENSP00000340608 | 9.19E-06 | -0.1700 | 289 | 0.681 |
| ENSP00000428459 | 3.18E-06 | -0.1701 | 0 | 0.119 |
| ENSP00000399475 | 3.00E-06 | -0.1701 | 0 | 0.275 |
| ENSP00000450710 | 2.14E-05 | -0.1701 | 213 | 0.000 |
| ENSP00000335660 | 4.56E-06 | -0.1701 | 0 | 0.184 |
| ENSP00000377771 | 1.33E-05 | -0.1701 | 0 | 0.196 |
| ENSP00000355938 | 5.63E-06 | -0.1701 | 0 | 0.108 |
| ENSP00000431748 | 1.92E-06 | -0.1702 | 0 | 0.101 |
| ENSP00000371927 | 7.07E-06 | -0.1702 | 510 | 0.132 |
| ENSP00000348573 | 4.35E-06 | -0.1702 | 0 | 0.413 |
| ENSP00000364107 | 5.62E-06 | -0.1702 | 0 | 0.082 |
| ENSP00000361254 | 1.21E-05 | -0.1703 | 358 | 0.123 |
| ENSP00000303148 | 5.72E-06 | -0.1703 | 0 | 0.142 |
| ENSP00000371715 | 3.02E-06 | -0.1703 | 0 | 0.115 |
| ENSP00000470383 | 2.53E-06 | -0.1704 | 0 | 0.000 |
| ENSP00000382767 | 5.61E-06 | -0.1704 | 247 | 0.455 |
| ENSP00000355651 | 1.72E-05 | -0.1704 | 235 | 0.275 |
| ENSP00000240079 | 5.47E-06 | -0.1704 | 0 | 0.086 |
| ENSP00000384876 | 4.04E-06 | -0.1704 | 0 | 0.294 |
| ENSP00000358421 | 1.45E-05 | -0.1704 | 202 | 0.500 |
| ENSP00000367752 | 3.42E-06 | -0.1705 | 0 | 0.128 |
| ENSP00000377823 | 3.79E-06 | -0.1705 | 0 | 0.058 |
| ENSP00000365596 | 8.78E-06 | -0.1705 | 293 | 0.370 |
| ENSP00000450675 | 3.26E-06 | -0.1705 | 0 | 0.000 |
| ENSP00000359571 | 2.94E-06 | -0.1705 | 0 | 0.162 |
| ENSP00000370589 | 1.43E-05 | -0.1705 | 243 | 0.000 |
| ENSP00000311181 | 4.36E-06 | -0.1705 | 0 | 0.000 |
| ENSP00000364620 | 1.33E-05 | -0.1706 | 270 | 0.068 |
| ENSP00000359361 | 1.71E-05 | -0.1707 | 192 | 0.411 |
| ENSP00000347626 | 1.11E-05 | -0.1707 | 0 | 0.162 |
| ENSP00000358170 | 7.52E-06 | -0.1707 | 0 | 0.145 |
| ENSP00000317743 | 7.76E-06 | -0.1708 | 0 | 0.131 |
| ENSP00000401502 | 2.89E-06 | -0.1708 | 0 | 0.122 |
| ENSP00000370568 | 4.42E-06 | -0.1708 | 0 | 0.140 |
| ENSP00000364270 | 5.02E-06 | -0.1708 | 0 | 0.759 |
| ENSP00000305913 | 2.10E-05 | -0.1708 | 0 | 0.362 |

| | | | | |
|---|---|---|---|---|
| ENSP00000373509 | 4.39E-06 | -0.1709 | 0 | 0.124 |
| ENSP00000423391 | 3.95E-06 | -0.1709 | 0 | 0.228 |
| ENSP00000378958 | 1.96E-05 | -0.1709 | 244 | 0.000 |
| ENSP00000450995 | 2.46E-06 | -0.1709 | 0 | 0.000 |
| ENSP00000404705 | 5.28E-06 | -0.1709 | 0 | 0.077 |
| ENSP00000379242 | 4.41E-06 | -0.1709 | 0 | 0.152 |
| ENSP00000343392 | 5.82E-06 | -0.1709 | 340 | 0.777 |
| ENSP00000377370 | 6.32E-06 | -0.1710 | 0 | 0.127 |
| ENSP00000360381 | 1.48E-06 | -0.1710 | 0 | 0.135 |
| ENSP00000458130 | 1.75E-06 | -0.1711 | 0 | 0.112 |
| ENSP00000314976 | 5.82E-06 | -0.1711 | 0 | 0.502 |
| ENSP00000222275 | 1.58E-05 | -0.1711 | 0 | 0.146 |
| ENSP00000354607 | 1.83E-05 | -0.1711 | 227 | 0.900 |
| ENSP00000272367 | 7.73E-06 | -0.1711 | 0 | 0.100 |
| ENSP00000363513 | 4.33E-06 | -0.1711 | 0 | 0.112 |
| ENSP00000414750 | 3.79E-06 | -0.1711 | 0 | 0.494 |
| ENSP00000265732 | 1.38E-05 | -0.1711 | 0 | 0.199 |
| ENSP00000358708 | 4.73E-06 | -0.1711 | 0 | 0.255 |
| ENSP00000470478 | 1.49E-06 | -0.1711 | 0 | 0.000 |
| ENSP00000377577 | 6.46E-07 | -0.1711 | 0 | 0.000 |
| ENSP00000378736 | 1.21E-05 | -0.1711 | 0 | 0.300 |
| ENSP00000446479 | 1.57E-05 | -0.1712 | 242 | 0.467 |
| ENSP00000355428 | 4.15E-06 | -0.1713 | 0 | 0.137 |
| ENSP00000377007 | 3.17E-06 | -0.1713 | 0 | 0.342 |
| ENSP00000362376 | 4.71E-06 | -0.1714 | 0 | 0.090 |
| ENSP00000381698 | 3.81E-06 | -0.1714 | 0 | 0.264 |
| ENSP00000392094 | 2.27E-05 | -0.1714 | 541 | 0.899 |
| ENSP00000414068 | 1.27E-05 | -0.1715 | 908 | 0.000 |
| ENSP00000432799 | 1.33E-05 | -0.1715 | 746 | 0.342 |
| ENSP00000376304 | 4.32E-06 | -0.1715 | 0 | 0.107 |
| ENSP00000454322 | 1.77E-06 | -0.1715 | 0 | 0.112 |
| ENSP00000380969 | 1.21E-05 | -0.1715 | 905 | 0.601 |
| ENSP00000357226 | 2.22E-06 | -0.1715 | 0 | 0.160 |
| ENSP00000297596 | 6.07E-06 | -0.1715 | 448 | 0.601 |
| ENSP00000369774 | 3.93E-06 | -0.1716 | 0 | 0.719 |
| ENSP00000264380 | 6.57E-06 | -0.1716 | 771 | 0.670 |
| ENSP00000401197 | 5.16E-06 | -0.1716 | 0 | 0.171 |
| ENSP00000451542 | 1.45E-06 | -0.1717 | 0 | 0.000 |
| ENSP00000424381 | 3.30E-06 | -0.1717 | 0 | 0.100 |
| ENSP00000399947 | 6.11E-06 | -0.1717 | 0 | 0.000 |
| ENSP00000402505 | 6.12E-06 | -0.1718 | 0 | 0.116 |
| ENSP00000361138 | 2.35E-06 | -0.1718 | 0 | 0.099 |
| ENSP00000379363 | 3.92E-06 | -0.1718 | 0 | 0.725 |
| ENSP00000356966 | 5.02E-06 | -0.1718 | 156 | 0.325 |
| ENSP00000421258 | 1.13E-05 | -0.1718 | 265 | 0.697 |
| ENSP00000363571 | 1.15E-05 | -0.1718 | 209 | 0.673 |

| | | | | |
|---|---|---|---|---|
| ENSP00000321805 | 1.21E-05 | -0.1718 | 503 | 0.605 |
| ENSP00000396976 | 4.12E-06 | -0.1718 | 0 | 0.495 |
| ENSP00000447149 | 9.76E-06 | -0.1719 | 456 | 0.686 |
| ENSP00000371985 | 1.64E-05 | -0.1719 | 313 | 0.523 |
| ENSP00000428612 | 1.70E-06 | -0.1720 | 0 | 0.076 |
| ENSP00000367185 | 6.05E-06 | -0.1720 | 0 | 0.095 |
| ENSP00000357737 | 5.17E-06 | -0.1721 | 0 | 0.104 |
| ENSP00000357920 | 1.10E-05 | -0.1721 | 206 | 0.245 |
| ENSP00000362171 | 1.79E-05 | -0.1721 | 326 | 0.000 |
| ENSP00000376440 | 2.64E-06 | -0.1721 | 0 | 0.173 |
| ENSP00000291971 | 3.81E-06 | -0.1721 | 0 | 0.170 |
| ENSP00000348300 | 6.66E-06 | -0.1722 | 0 | 0.413 |
| ENSP00000405577 | 2.91E-06 | -0.1722 | 0 | 0.172 |
| ENSP00000359600 | 5.80E-06 | -0.1722 | 0 | 0.103 |
| ENSP00000401535 | 4.26E-06 | -0.1723 | 0 | 0.000 |
| ENSP00000361721 | 2.59E-06 | -0.1723 | 0 | 0.221 |
| ENSP00000365682 | 1.21E-05 | -0.1725 | 214 | 0.809 |
| ENSP00000379946 | 3.41E-06 | -0.1725 | 0 | 0.547 |
| ENSP00000429243 | 8.65E-06 | -0.1725 | 196 | 0.369 |
| ENSP00000311427 | 5.03E-06 | -0.1726 | 0 | 0.101 |
| ENSP00000364204 | 1.24E-05 | -0.1726 | 662 | 0.620 |
| ENSP00000428646 | 4.73E-06 | -0.1726 | 0 | 0.149 |
| ENSP00000363642 | 1.21E-05 | -0.1727 | 288 | 0.903 |
| ENSP00000365686 | 1.27E-05 | -0.1727 | 517 | 0.132 |
| ENSP00000311436 | 1.52E-05 | -0.1727 | 270 | 0.231 |
| ENSP00000329904 | 1.11E-05 | -0.1727 | 0 | 0.168 |
| ENSP00000457628 | 3.38E-06 | -0.1727 | 0 | 0.000 |
| ENSP00000391944 | 1.25E-05 | -0.1727 | 560 | 0.802 |
| ENSP00000451476 | 2.14E-06 | -0.1727 | 0 | 0.000 |
| ENSP00000374574 | 4.74E-06 | -0.1727 | 0 | 0.893 |
| ENSP00000243189 | 7.59E-06 | -0.1727 | 0 | 0.000 |
| ENSP00000358293 | 2.16E-06 | -0.1728 | 0 | 0.000 |
| ENSP00000326253 | 1.52E-05 | -0.1729 | 197 | 0.129 |
| ENSP00000377192 | 6.52E-06 | -0.1729 | 220 | 0.437 |
| ENSP00000374408 | 2.90E-06 | -0.1729 | 0 | 0.161 |
| ENSP00000346534 | 9.08E-06 | -0.1730 | 205 | 0.533 |
| ENSP00000423820 | 1.10E-05 | -0.1731 | 204 | 0.735 |
| ENSP00000362810 | 5.24E-06 | -0.1731 | 0 | 0.575 |
| ENSP00000442436 | 3.86E-06 | -0.1732 | 0 | 0.000 |
| ENSP00000356946 | 1.59E-05 | -0.1732 | 242 | 0.708 |
| ENSP00000416040 | 1.87E-06 | -0.1732 | 0 | 0.312 |
| ENSP00000376246 | 3.46E-06 | -0.1733 | 0 | 0.133 |
| ENSP00000358159 | 1.59E-05 | -0.1734 | 543 | 0.907 |
| ENSP00000316224 | 6.94E-06 | -0.1734 | 0 | 0.000 |
| ENSP00000367309 | 1.90E-05 | -0.1735 | 265 | 0.000 |
| ENSP00000362115 | 1.62E-05 | -0.1735 | 320 | 0.430 |

| | | | | |
|---|---|---|---|---|
| ENSP00000381102 | 2.96E-06 | -0.1736 | 0 | 0.077 |
| ENSP00000321108 | 1.25E-05 | -0.1736 | 769 | 0.063 |
| ENSP00000412388 | 4.24E-06 | -0.1736 | 0 | 0.111 |
| ENSP00000353157 | 5.31E-06 | -0.1737 | 0 | 0.463 |
| ENSP00000356202 | 1.15E-05 | -0.1737 | 290 | 0.572 |
| ENSP00000367446 | 1.61E-05 | -0.1737 | 177 | 0.600 |
| ENSP00000319590 | 3.65E-06 | -0.1737 | 0 | 0.107 |
| ENSP00000391465 | 2.09E-06 | -0.1738 | 0 | 0.000 |
| ENSP00000370023 | 2.16E-05 | -0.1738 | 301 | 0.504 |
| ENSP00000369099 | 1.14E-05 | -0.1738 | 201 | 0.183 |
| ENSP00000420610 | 1.52E-06 | -0.1739 | 0 | 0.190 |
| ENSP00000361658 | 4.17E-06 | -0.1739 | 0 | 0.358 |
| ENSP00000257570 | 6.48E-06 | -0.1739 | 901 | 0.654 |
| ENSP00000254336 | 5.39E-06 | -0.1739 | 0 | 0.110 |
| ENSP00000300575 | 8.58E-06 | -0.1739 | 0 | 0.129 |
| ENSP00000344860 | 1.95E-06 | -0.1739 | 0 | 0.183 |
| ENSP00000383923 | 3.33E-06 | -0.1740 | 0 | 0.189 |
| ENSP00000305731 | 1.46E-05 | -0.1740 | 167 | 0.330 |
| ENSP00000455075 | 1.19E-06 | -0.1740 | 0 | 0.000 |
| ENSP00000364405 | 3.77E-06 | -0.1740 | 0 | 0.679 |
| ENSP00000406751 | 1.12E-05 | -0.1740 | 243 | 0.712 |
| ENSP00000377486 | 9.54E-06 | -0.1740 | 401 | 0.636 |
| ENSP00000451369 | 4.18E-06 | -0.1741 | 0 | 0.000 |
| ENSP00000378485 | 2.42E-05 | -0.1741 | 274 | 0.681 |
| ENSP00000346921 | 8.79E-06 | -0.1741 | 480 | 0.550 |
| ENSP00000437073 | 2.22E-05 | -0.1742 | 244 | 0.434 |
| ENSP00000350310 | 1.53E-05 | -0.1743 | 175 | 0.622 |
| ENSP00000395461 | 1.69E-06 | -0.1743 | 0 | 0.150 |
| ENSP00000352606 | 1.40E-05 | -0.1743 | 0 | 0.134 |
| ENSP00000395328 | 5.30E-06 | -0.1743 | 0 | 0.108 |
| ENSP00000311010 | 1.76E-05 | -0.1744 | 200 | 0.839 |
| ENSP00000347602 | 1.09E-05 | -0.1744 | 195 | 0.000 |
| ENSP00000310856 | 1.31E-05 | -0.1745 | 165 | 0.148 |
| ENSP00000362946 | 8.75E-06 | -0.1745 | 671 | 0.608 |
| ENSP00000324292 | 4.83E-06 | -0.1745 | 0 | 0.120 |
| ENSP00000375428 | 1.18E-06 | -0.1745 | 0 | 0.183 |
| ENSP00000361423 | 1.99E-05 | -0.1746 | 310 | 0.694 |
| ENSP00000346142 | 1.55E-05 | -0.1746 | 337 | 0.365 |
| ENSP00000357692 | 2.29E-06 | -0.1746 | 0 | 0.121 |
| ENSP00000374292 | 4.28E-06 | -0.1746 | 0 | 0.134 |
| ENSP00000363046 | 3.27E-06 | -0.1747 | 0 | 0.083 |
| ENSP00000440509 | 9.89E-07 | -0.1747 | 0 | 0.144 |
| ENSP00000445626 | 4.73E-06 | -0.1747 | 0 | 0.770 |
| ENSP00000323217 | 2.77E-06 | -0.1748 | 0 | 0.160 |
| ENSP00000471799 | 1.99E-06 | -0.1748 | 0 | 0.000 |
| ENSP00000362481 | 1.32E-05 | -0.1749 | 583 | 0.539 |

| | | | | |
|---|---|---|---|---|
| ENSP00000363458 | 1.92E-05 | -0.1749 | 930 | 0.660 |
| ENSP00000429808 | 2.66E-06 | -0.1749 | 0 | 0.113 |
| ENSP00000377380 | 1.36E-05 | -0.1750 | 172 | 0.391 |
| ENSP00000340361 | 1.59E-05 | -0.1750 | 196 | 0.893 |
| ENSP00000246105 | 2.95E-06 | -0.1750 | 0 | 0.144 |
| ENSP00000429896 | 6.40E-07 | -0.1750 | 0 | 0.161 |
| ENSP00000356476 | 3.83E-06 | -0.1750 | 0 | 0.835 |
| ENSP00000222462 | 1.49E-05 | -0.1751 | 573 | 0.927 |
| ENSP00000376992 | 3.46E-06 | -0.1751 | 0 | 0.112 |
| ENSP00000362702 | 8.25E-06 | -0.1751 | 150 | 0.444 |
| ENSP00000429190 | 6.65E-06 | -0.1751 | 0 | 0.291 |
| ENSP00000367797 | 4.72E-06 | -0.1751 | 0 | 0.737 |
| ENSP00000368813 | 5.39E-06 | -0.1751 | 0 | 0.165 |
| ENSP00000367013 | 1.77E-05 | -0.1751 | 205 | 0.860 |
| ENSP00000287748 | 1.23E-05 | -0.1752 | 0 | 0.171 |
| ENSP00000428924 | 2.69E-05 | -0.1752 | 306 | 0.656 |
| ENSP00000462883 | 3.64E-07 | -0.1752 | 0 | 0.156 |
| ENSP00000310149 | 5.35E-06 | -0.1752 | 0 | 0.143 |
| ENSP00000262637 | 1.01E-05 | -0.1753 | 0 | 0.000 |
| ENSP00000387875 | 2.97E-06 | -0.1753 | 0 | 0.242 |
| ENSP00000353622 | 1.61E-05 | -0.1754 | 285 | 0.000 |
| ENSP00000375599 | 1.64E-06 | -0.1754 | 0 | 0.000 |
| ENSP00000363360 | 3.02E-06 | -0.1754 | 0 | 0.107 |
| ENSP00000425446 | 5.84E-06 | -0.1755 | 0 | 0.000 |
| ENSP00000349588 | 1.06E-05 | -0.1755 | 912 | 0.675 |
| ENSP00000370522 | 1.75E-05 | -0.1755 | 265 | 0.208 |
| ENSP00000364650 | 3.73E-06 | -0.1756 | 0 | 0.090 |
| ENSP00000422591 | 1.04E-05 | -0.1756 | 168 | 0.850 |
| ENSP00000365891 | 1.20E-05 | -0.1756 | 195 | 0.463 |
| ENSP00000319096 | 6.44E-06 | -0.1756 | 448 | 0.590 |
| ENSP00000358531 | 1.18E-05 | -0.1756 | 0 | 0.376 |
| ENSP00000343140 | 1.07E-05 | -0.1756 | 150 | 0.101 |
| ENSP00000340644 | 8.99E-06 | -0.1757 | 0 | 0.118 |
| ENSP00000328879 | 4.79E-06 | -0.1757 | 0 | 0.786 |
| ENSP00000441802 | 1.63E-05 | -0.1757 | 150 | 0.101 |
| ENSP00000313572 | 5.18E-06 | -0.1757 | 205 | 0.000 |
| ENSP00000366395 | 1.21E-05 | -0.1757 | 940 | 0.568 |
| ENSP00000258042 | 7.03E-06 | -0.1758 | 0 | 0.502 |
| ENSP00000357311 | 3.52E-06 | -0.1758 | 0 | 0.180 |
| ENSP00000386787 | 7.98E-06 | -0.1759 | 0 | 0.113 |
| ENSP00000386133 | 2.97E-06 | -0.1759 | 0 | 0.141 |
| ENSP00000354028 | 5.08E-06 | -0.1759 | 0 | 0.101 |
| ENSP00000318264 | 3.11E-06 | -0.1760 | 0 | 0.162 |
| ENSP00000386414 | 3.41E-06 | -0.1760 | 0 | 0.582 |
| ENSP00000423600 | 1.10E-05 | -0.1760 | 220 | 0.060 |
| ENSP00000257894 | 1.33E-05 | -0.1760 | 0 | 0.151 |

| | | | | |
|---|---|---|---|---|
| ENSP00000348395 | 1.59E-05 | -0.1760 | 209 | 0.361 |
| ENSP00000362937 | 4.53E-06 | -0.1761 | 0 | 0.680 |
| ENSP00000269967 | 3.56E-06 | -0.1761 | 0 | 0.234 |
| ENSP00000421169 | 4.21E-06 | -0.1761 | 0 | 0.102 |
| ENSP00000294600 | 1.24E-05 | -0.1761 | 0 | 0.165 |
| ENSP00000372482 | 4.78E-06 | -0.1761 | 0 | 0.099 |
| ENSP00000369341 | 1.84E-06 | -0.1762 | 0 | 0.119 |
| ENSP00000443772 | 2.99E-06 | -0.1762 | 0 | 0.000 |
| ENSP00000336817 | 1.15E-05 | -0.1762 | 0 | 0.118 |
| ENSP00000355231 | 2.01E-05 | -0.1762 | 505 | 0.675 |
| ENSP00000346802 | 6.95E-06 | -0.1762 | 0 | 0.138 |
| ENSP00000359819 | 2.43E-06 | -0.1763 | 0 | 0.113 |
| ENSP00000427123 | 8.86E-06 | -0.1763 | 201 | 0.635 |
| ENSP00000354468 | 6.22E-06 | -0.1763 | 193 | 0.325 |
| ENSP00000306490 | 1.72E-05 | -0.1764 | 185 | 0.708 |
| ENSP00000280082 | 6.54E-06 | -0.1764 | 0 | 0.000 |
| ENSP00000270517 | 4.88E-06 | -0.1764 | 0 | 0.312 |
| ENSP00000391311 | 9.28E-06 | -0.1764 | 208 | 0.456 |
| ENSP00000332488 | 1.57E-05 | -0.1764 | 264 | 0.095 |
| ENSP00000402825 | 1.77E-05 | -0.1764 | 153 | 0.000 |
| ENSP00000361042 | 3.22E-06 | -0.1764 | 0 | 0.146 |
| ENSP00000354566 | 1.63E-05 | -0.1765 | 175 | 0.675 |
| ENSP00000354991 | 3.98E-06 | -0.1765 | 0 | 0.123 |
| ENSP00000368881 | 1.08E-05 | -0.1765 | 156 | 0.327 |
| ENSP00000335281 | 1.53E-06 | -0.1765 | 0 | 0.125 |
| ENSP00000391227 | 2.79E-06 | -0.1765 | 0 | 0.129 |
| ENSP00000380734 | 3.86E-06 | -0.1765 | 0 | 0.177 |
| ENSP00000289749 | 9.60E-06 | -0.1766 | 0 | 0.109 |
| ENSP00000373998 | 7.83E-06 | -0.1766 | 0 | 0.135 |
| ENSP00000357106 | 1.09E-05 | -0.1766 | 158 | 0.085 |
| ENSP00000392553 | 3.72E-06 | -0.1766 | 0 | 0.000 |
| ENSP00000367164 | 1.02E-06 | -0.1766 | 0 | 0.131 |
| ENSP00000364687 | 1.87E-05 | -0.1766 | 411 | 0.000 |
| ENSP00000347748 | 1.02E-05 | -0.1766 | 0 | 0.166 |
| ENSP00000434516 | 1.83E-05 | -0.1766 | 604 | 0.726 |
| ENSP00000252318 | 1.46E-05 | -0.1766 | 0 | 0.104 |
| ENSP00000407107 | 1.98E-06 | -0.1767 | 0 | 0.196 |
| ENSP00000377583 | 3.24E-06 | -0.1767 | 0 | 0.181 |
| ENSP00000372202 | 2.66E-06 | -0.1767 | 0 | 0.196 |
| ENSP00000363745 | 4.33E-06 | -0.1767 | 0 | 0.852 |
| ENSP00000318437 | 1.25E-05 | -0.1768 | 0 | 0.112 |
| ENSP00000251507 | 4.55E-06 | -0.1768 | 0 | 0.164 |
| ENSP00000313423 | 9.11E-06 | -0.1768 | 0 | 0.136 |
| ENSP00000390153 | 3.58E-06 | -0.1768 | 0 | 0.000 |
| ENSP00000344831 | 8.50E-06 | -0.1768 | 242 | 0.073 |
| ENSP00000387298 | 1.03E-06 | -0.1768 | 0 | 0.192 |

| | | | | |
|---|---|---|---|---|
| ENSP00000333534 | 5.57E-06 | -0.1768 | 0 | 0.164 |
| ENSP00000256925 | 4.95E-06 | -0.1768 | 0 | 0.188 |
| ENSP00000315214 | 1.80E-05 | -0.1769 | 0 | 0.000 |
| ENSP00000389466 | 3.63E-06 | -0.1769 | 0 | 0.132 |
| ENSP00000378326 | 4.73E-06 | -0.1769 | 0 | 0.086 |
| ENSP00000360593 | 5.19E-06 | -0.1769 | 247 | 0.000 |
| ENSP00000380271 | 6.02E-06 | -0.1769 | 0 | 0.502 |
| ENSP00000374069 | 2.13E-05 | -0.1770 | 264 | 0.630 |
| ENSP00000358810 | 3.58E-06 | -0.1770 | 0 | 0.092 |
| ENSP00000388723 | 3.94E-06 | -0.1771 | 0 | 0.087 |
| ENSP00000386810 | 1.19E-05 | -0.1771 | 270 | 0.000 |
| ENSP00000373810 | 2.84E-06 | -0.1772 | 0 | 0.000 |
| ENSP00000380929 | 1.73E-06 | -0.1772 | 0 | 0.182 |
| ENSP00000359869 | 8.13E-06 | -0.1772 | 223 | 0.199 |
| ENSP00000415430 | 3.71E-06 | -0.1772 | 0 | 0.230 |
| ENSP00000427687 | 1.22E-05 | -0.1772 | 720 | 0.344 |
| ENSP00000424768 | 2.61E-06 | -0.1773 | 0 | 0.122 |
| ENSP00000401932 | 6.14E-06 | -0.1773 | 0 | 0.105 |
| ENSP00000268043 | 3.64E-06 | -0.1773 | 0 | 0.703 |
| ENSP00000352408 | 1.23E-05 | -0.1773 | 292 | 0.446 |
| ENSP00000364543 | 5.58E-06 | -0.1773 | 0 | 0.892 |
| ENSP00000354812 | 3.07E-06 | -0.1773 | 0 | 0.800 |
| ENSP00000366748 | 6.93E-06 | -0.1773 | 748 | 0.303 |
| ENSP00000358497 | 1.43E-05 | -0.1774 | 282 | 0.411 |
| ENSP00000343325 | 2.15E-05 | -0.1774 | 734 | 0.667 |
| ENSP00000374387 | 3.13E-06 | -0.1774 | 0 | 0.544 |
| ENSP00000391409 | 4.65E-06 | -0.1774 | 0 | 0.177 |
| ENSP00000334922 | 3.57E-06 | -0.1774 | 0 | 0.110 |
| ENSP00000353198 | 1.71E-05 | -0.1775 | 209 | 0.598 |
| ENSP00000365256 | 1.28E-05 | -0.1776 | 360 | 0.088 |
| ENSP00000319126 | 4.12E-06 | -0.1776 | 0 | 0.135 |
| ENSP00000299432 | 1.11E-05 | -0.1776 | 0 | 0.148 |
| ENSP00000361289 | 5.13E-06 | -0.1776 | 0 | 0.284 |
| ENSP00000371347 | 8.23E-06 | -0.1776 | 0 | 0.500 |
| ENSP00000456645 | 3.03E-06 | -0.1777 | 0 | 0.000 |
| ENSP00000240691 | 1.01E-05 | -0.1777 | 0 | 0.151 |
| ENSP00000444856 | 7.27E-06 | -0.1777 | 178 | 0.687 |
| ENSP00000358742 | 2.98E-06 | -0.1778 | 0 | 0.154 |
| ENSP00000353375 | 3.99E-06 | -0.1778 | 0 | 0.236 |
| ENSP00000436426 | 1.71E-06 | -0.1778 | 0 | 0.118 |
| ENSP00000414786 | 3.11E-06 | -0.1778 | 0 | 0.076 |
| ENSP00000378145 | 1.17E-05 | -0.1778 | 0 | 0.082 |
| ENSP00000355031 | 3.95E-06 | -0.1778 | 0 | 0.690 |
| ENSP00000362105 | 3.46E-06 | -0.1778 | 0 | 0.862 |
| ENSP00000431445 | 1.20E-05 | -0.1779 | 0 | 0.503 |
| ENSP00000215061 | 7.14E-06 | -0.1779 | 0 | 0.124 |

| | | | | |
|---|---|---|---|---|
| ENSP00000361990 | 7.95E-06 | -0.1780 | 167 | 0.200 |
| ENSP00000325836 | 5.86E-06 | -0.1780 | 448 | 0.594 |
| ENSP00000415556 | 3.38E-06 | -0.1781 | 0 | 0.093 |
| ENSP00000310008 | 9.45E-06 | -0.1781 | 0 | 0.379 |
| ENSP00000377523 | 1.65E-05 | -0.1781 | 268 | 0.930 |
| ENSP00000374328 | 4.13E-06 | -0.1781 | 0 | 0.135 |
| ENSP00000321309 | 3.01E-06 | -0.1782 | 0 | 0.177 |
| ENSP00000340988 | 8.51E-06 | -0.1782 | 0 | 0.163 |
| ENSP00000371535 | 3.93E-06 | -0.1782 | 0 | 0.256 |
| ENSP00000366662 | 8.88E-06 | -0.1783 | 0 | 0.103 |
| ENSP00000439287 | 1.93E-06 | -0.1783 | 0 | 0.151 |
| ENSP00000361761 | 1.27E-05 | -0.1783 | 150 | 0.083 |
| ENSP00000360645 | 6.58E-06 | -0.1783 | 279 | 0.155 |
| ENSP00000305875 | 5.97E-06 | -0.1783 | 0 | 0.176 |
| ENSP00000368565 | 5.72E-06 | -0.1783 | 642 | 0.824 |
| ENSP00000377385 | 1.44E-05 | -0.1783 | 213 | 0.875 |
| ENSP00000350965 | 6.22E-06 | -0.1783 | 257 | 0.000 |
| ENSP00000364035 | 5.33E-06 | -0.1784 | 417 | 0.183 |
| ENSP00000328270 | 1.35E-06 | -0.1784 | 0 | 0.172 |
| ENSP00000346045 | 3.37E-06 | -0.1784 | 0 | 0.815 |
| ENSP00000408730 | 1.45E-06 | -0.1785 | 0 | 0.113 |
| ENSP00000359772 | 3.91E-06 | -0.1785 | 0 | 0.000 |
| ENSP00000351669 | 1.17E-05 | -0.1786 | 262 | 0.073 |
| ENSP00000371328 | 4.31E-06 | -0.1786 | 0 | 0.137 |
| ENSP00000398617 | 2.56E-06 | -0.1786 | 0 | 0.163 |
| ENSP00000357307 | 1.01E-05 | -0.1786 | 313 | 0.244 |
| ENSP00000442339 | 1.35E-06 | -0.1786 | 0 | 0.564 |
| ENSP00000316012 | 3.31E-06 | -0.1786 | 0 | 0.114 |
| ENSP00000284382 | 5.31E-06 | -0.1786 | 0 | 0.488 |
| ENSP00000340125 | 7.95E-06 | -0.1787 | 197 | 0.167 |
| ENSP00000363482 | 1.79E-06 | -0.1788 | 0 | 0.109 |
| ENSP00000319388 | 1.47E-05 | -0.1788 | 630 | 0.131 |
| ENSP00000302657 | 5.50E-06 | -0.1788 | 0 | 0.126 |
| ENSP00000438262 | 1.40E-05 | -0.1788 | 254 | 0.693 |
| ENSP00000377865 | 1.68E-05 | -0.1788 | 332 | 0.766 |
| ENSP00000359170 | 4.04E-06 | -0.1789 | 0 | 0.166 |
| ENSP00000303522 | 1.77E-05 | -0.1790 | 283 | 0.661 |
| ENSP00000368389 | 3.46E-06 | -0.1790 | 0 | 0.161 |
| ENSP00000368965 | 3.54E-06 | -0.1790 | 0 | 0.105 |
| ENSP00000357829 | 3.33E-06 | -0.1790 | 0 | 0.424 |
| ENSP00000375853 | 1.32E-05 | -0.1790 | 197 | 0.384 |
| ENSP00000352926 | 9.28E-06 | -0.1790 | 918 | 0.000 |
| ENSP00000359645 | 4.29E-06 | -0.1791 | 0 | 0.873 |
| ENSP00000327168 | 3.52E-06 | -0.1791 | 0 | 0.139 |
| ENSP00000331302 | 2.87E-06 | -0.1791 | 0 | 0.341 |
| ENSP00000355315 | 3.89E-06 | -0.1792 | 0 | 0.768 |

| | | | | |
|---|---|---|---|---|
| ENSP00000295718 | 1.36E-05 | -0.1792 | 220 | 0.648 |
| ENSP00000389277 | 5.44E-06 | -0.1792 | 361 | 0.212 |
| ENSP00000430307 | 5.66E-06 | -0.1793 | 0 | 0.156 |
| ENSP00000371923 | 1.14E-05 | -0.1793 | 235 | 0.206 |
| ENSP00000455906 | 1.80E-06 | -0.1793 | 0 | 0.125 |
| ENSP00000221978 | 1.29E-05 | -0.1793 | 0 | 0.130 |
| ENSP00000409197 | 4.43E-06 | -0.1793 | 0 | 0.000 |
| ENSP00000295290 | 1.03E-05 | -0.1794 | 0 | 0.105 |
| ENSP00000368931 | 1.48E-06 | -0.1794 | 0 | 0.181 |
| ENSP00000443007 | 3.53E-06 | -0.1794 | 0 | 0.000 |
| ENSP00000407497 | 3.35E-06 | -0.1794 | 0 | 0.127 |
| ENSP00000452871 | 1.56E-05 | -0.1794 | 202 | 0.805 |
| ENSP00000378508 | 6.28E-06 | -0.1794 | 811 | 0.250 |
| ENSP00000337146 | 4.18E-06 | -0.1794 | 0 | 0.157 |
| ENSP00000353824 | 7.92E-06 | -0.1795 | 805 | 0.684 |
| ENSP00000243938 | 3.18E-06 | -0.1795 | 0 | 0.143 |
| ENSP00000364398 | 1.21E-05 | -0.1795 | 0 | 0.642 |
| ENSP00000358967 | 1.96E-05 | -0.1795 | 303 | 0.437 |
| ENSP00000369438 | 7.91E-07 | -0.1796 | 0 | 0.170 |
| ENSP00000265993 | 5.76E-06 | -0.1796 | 0 | 0.130 |
| ENSP00000367746 | 1.97E-06 | -0.1797 | 0 | 0.117 |
| ENSP00000367407 | 4.49E-06 | -0.1797 | 0 | 0.165 |
| ENSP00000385695 | 5.73E-06 | -0.1797 | 0 | 0.517 |
| ENSP00000297537 | 4.58E-06 | -0.1797 | 0 | 0.799 |
| ENSP00000406144 | 1.19E-06 | -0.1797 | 0 | 0.139 |
| ENSP00000360210 | 3.82E-06 | -0.1797 | 0 | 0.118 |
| ENSP00000354569 | 1.44E-05 | -0.1798 | 515 | 0.000 |
| ENSP00000376786 | 9.46E-06 | -0.1798 | 159 | 0.736 |
| ENSP00000409423 | 2.96E-06 | -0.1798 | 0 | 0.230 |
| ENSP00000472867 | 2.08E-06 | -0.1798 | 0 | 0.694 |
| ENSP00000355133 | 6.65E-06 | -0.1798 | 208 | 0.235 |
| ENSP00000355760 | 4.99E-06 | -0.1798 | 0 | 0.378 |
| ENSP00000447547 | 1.56E-06 | -0.1799 | 0 | 0.381 |
| ENSP00000371936 | 5.09E-06 | -0.1799 | 0 | 0.560 |
| ENSP00000368169 | 2.64E-05 | -0.1799 | 748 | 0.927 |
| ENSP00000410862 | 9.19E-06 | -0.1799 | 203 | 0.193 |
| ENSP00000255448 | 3.17E-06 | -0.1799 | 0 | 0.724 |
| ENSP00000352175 | 2.82E-06 | -0.1800 | 0 | 0.136 |
| ENSP00000426296 | 1.76E-06 | -0.1800 | 0 | 0.137 |
| ENSP00000302552 | 5.89E-06 | -0.1800 | 0 | 0.104 |
| ENSP00000425658 | 7.43E-06 | -0.1800 | 202 | 0.593 |
| ENSP00000359286 | 2.44E-06 | -0.1800 | 0 | 0.000 |
| ENSP00000355961 | 1.03E-05 | -0.1800 | 0 | 0.217 |
| ENSP00000256443 | 4.33E-06 | -0.1800 | 0 | 0.817 |
| ENSP00000263468 | 7.28E-06 | -0.1800 | 0 | 0.000 |
| ENSP00000348394 | 9.15E-06 | -0.1801 | 0 | 0.087 |

| | | | | |
|---|---|---|---|---|
| ENSP00000365200 | 3.21E-06 | -0.1801 | 0 | 0.129 |
| ENSP00000381333 | 1.38E-05 | -0.1801 | 359 | 0.749 |
| ENSP00000351832 | 1.55E-05 | -0.1802 | 156 | 0.122 |
| ENSP00000375629 | 3.71E-06 | -0.1802 | 0 | 0.231 |
| ENSP00000346151 | 3.85E-06 | -0.1802 | 0 | 0.481 |
| ENSP00000305138 | 1.89E-06 | -0.1802 | 0 | 0.166 |
| ENSP00000354883 | 3.38E-06 | -0.1803 | 0 | 0.115 |
| ENSP00000339136 | 1.03E-05 | -0.1803 | 472 | 0.616 |
| ENSP00000390651 | 1.80E-05 | -0.1804 | 205 | 0.688 |
| ENSP00000374447 | 9.66E-06 | -0.1804 | 480 | 0.693 |
| ENSP00000414055 | 3.99E-06 | -0.1804 | 0 | 0.142 |
| ENSP00000414334 | 1.81E-05 | -0.1805 | 204 | 0.915 |
| ENSP00000395473 | 1.28E-05 | -0.1805 | 231 | 0.073 |
| ENSP00000399563 | 2.46E-06 | -0.1805 | 0 | 0.000 |
| ENSP00000360376 | 6.50E-06 | -0.1805 | 0 | 0.153 |
| ENSP00000377015 | 1.53E-05 | -0.1806 | 721 | 0.232 |
| ENSP00000362283 | 3.93E-06 | -0.1806 | 0 | 0.382 |
| ENSP00000253461 | 1.74E-05 | -0.1807 | 0 | 0.126 |
| ENSP00000398526 | 3.20E-06 | -0.1807 | 0 | 0.467 |
| ENSP00000452296 | 1.16E-05 | -0.1807 | 201 | 0.000 |
| ENSP00000376309 | 1.88E-05 | -0.1807 | 772 | 0.969 |
| ENSP00000430955 | 5.50E-06 | -0.1807 | 236 | 0.314 |
| ENSP00000277491 | 2.29E-05 | -0.1808 | 165 | 0.000 |
| ENSP00000329860 | 4.58E-06 | -0.1808 | 0 | 0.150 |
| ENSP00000406201 | 2.88E-06 | -0.1808 | 0 | 0.688 |
| ENSP00000356886 | 7.28E-06 | -0.1808 | 0 | 0.000 |
| ENSP00000436767 | 1.10E-05 | -0.1808 | 240 | 0.838 |
| ENSP00000350878 | 1.51E-05 | -0.1808 | 191 | 0.588 |
| ENSP00000417980 | 2.39E-05 | -0.1808 | 411 | 0.000 |
| ENSP00000318029 | 3.47E-06 | -0.1809 | 0 | 0.071 |
| ENSP00000454380 | 4.60E-06 | -0.1809 | 0 | 0.381 |
| ENSP00000362634 | 1.55E-05 | -0.1809 | 232 | 0.678 |
| ENSP00000354841 | 2.94E-06 | -0.1809 | 0 | 0.081 |
| ENSP00000417696 | 3.17E-06 | -0.1809 | 0 | 0.701 |
| ENSP00000362287 | 1.73E-05 | -0.1809 | 270 | 0.710 |
| ENSP00000392709 | 3.93E-06 | -0.1811 | 0 | 0.367 |
| ENSP00000356437 | 4.86E-06 | -0.1811 | 0 | 0.078 |
| ENSP00000363533 | 1.20E-05 | -0.1811 | 313 | 0.434 |
| ENSP00000468389 | 1.44E-06 | -0.1812 | 0 | 0.000 |
| ENSP00000327647 | 6.25E-06 | -0.1812 | 0 | 0.503 |
| ENSP00000335616 | 3.60E-06 | -0.1813 | 0 | 0.141 |
| ENSP00000349884 | 9.32E-06 | -0.1813 | 167 | 0.315 |
| ENSP00000359371 | 1.49E-05 | -0.1813 | 167 | 0.697 |
| ENSP00000351937 | 3.59E-06 | -0.1813 | 0 | 0.131 |
| ENSP00000379032 | 1.05E-05 | -0.1814 | 232 | 0.539 |
| ENSP00000419371 | 2.33E-06 | -0.1814 | 0 | 0.380 |

| | | | | |
|---|---|---|---|---|
| ENSP00000355377 | 5.79E-06 | -0.1814 | 0 | 0.080 |
| ENSP00000274532 | 8.57E-06 | -0.1814 | 0 | 0.209 |
| ENSP00000364847 | 3.56E-06 | -0.1814 | 0 | 0.273 |
| ENSP00000377083 | 1.14E-05 | -0.1815 | 226 | 0.299 |
| ENSP00000394546 | 1.63E-06 | -0.1815 | 0 | 0.139 |
| ENSP00000350540 | 8.25E-06 | -0.1815 | 0 | 0.000 |
| ENSP00000442688 | 2.41E-06 | -0.1815 | 0 | 0.110 |
| ENSP00000381184 | 2.75E-06 | -0.1815 | 0 | 0.000 |
| ENSP00000384442 | 7.10E-06 | -0.1815 | 174 | 0.650 |
| ENSP00000400815 | 1.69E-06 | -0.1816 | 0 | 0.119 |
| ENSP00000355385 | 3.16E-06 | -0.1817 | 0 | 0.140 |
| ENSP00000461518 | 6.64E-06 | -0.1818 | 0 | 0.266 |
| ENSP00000381937 | 2.09E-06 | -0.1818 | 0 | 0.000 |
| ENSP00000456827 | 3.33E-06 | -0.1819 | 0 | 0.000 |
| ENSP00000368025 | 2.82E-06 | -0.1819 | 0 | 0.135 |
| ENSP00000376215 | 1.81E-05 | -0.1819 | 178 | 0.671 |
| ENSP00000332565 | 1.71E-05 | -0.1819 | 216 | 0.342 |
| ENSP00000357906 | 7.02E-06 | -0.1819 | 0 | 0.097 |
| ENSP00000454374 | 1.21E-06 | -0.1819 | 0 | 0.000 |
| ENSP00000411532 | 3.28E-05 | -0.1820 | 711 | 0.784 |
| ENSP00000387641 | 1.01E-05 | -0.1820 | 151 | 0.228 |
| ENSP00000367623 | 1.80E-05 | -0.1820 | 195 | 0.789 |
| ENSP00000437523 | 2.95E-06 | -0.1820 | 0 | 0.000 |
| ENSP00000365380 | 2.90E-05 | -0.1820 | 933 | 0.000 |
| ENSP00000217428 | 4.43E-06 | -0.1820 | 0 | 0.155 |
| ENSP00000303999 | 6.67E-06 | -0.1821 | 0 | 0.087 |
| ENSP00000452521 | 9.54E-06 | -0.1821 | 0 | 0.221 |
| ENSP00000472922 | 5.47E-07 | -0.1821 | 0 | 0.000 |
| ENSP00000402634 | 5.26E-06 | -0.1821 | 0 | 0.185 |
| ENSP00000365318 | 1.34E-05 | -0.1821 | 270 | 0.587 |
| ENSP00000360255 | 1.00E-05 | -0.1821 | 196 | 0.177 |
| ENSP00000385006 | 5.02E-06 | -0.1822 | 0 | 0.169 |
| ENSP00000334042 | 1.01E-05 | -0.1823 | 0 | 0.096 |
| ENSP00000339960 | 1.65E-05 | -0.1823 | 216 | 0.454 |
| ENSP00000312273 | 7.07E-06 | -0.1823 | 0 | 0.110 |
| ENSP00000363993 | 1.51E-05 | -0.1823 | 639 | 0.399 |
| ENSP00000461388 | 3.15E-06 | -0.1823 | 0 | 0.000 |
| ENSP00000427329 | 4.27E-06 | -0.1824 | 0 | 0.000 |
| ENSP00000355583 | 4.44E-06 | -0.1824 | 205 | 0.000 |
| ENSP00000219162 | 1.78E-05 | -0.1825 | 0 | 0.103 |
| ENSP00000435310 | 1.43E-06 | -0.1825 | 0 | 0.000 |
| ENSP00000373354 | 1.58E-05 | -0.1825 | 244 | 0.000 |
| ENSP00000421180 | 2.02E-06 | -0.1825 | 0 | 0.575 |
| ENSP00000383219 | 2.06E-06 | -0.1826 | 0 | 0.169 |
| ENSP00000390722 | 5.87E-06 | -0.1826 | 221 | 0.178 |
| ENSP00000391835 | 2.05E-06 | -0.1827 | 0 | 0.000 |

| | | | | |
|---|---|---|---|---|
| ENSP00000362174 | 1.01E-05 | -0.1828 | 810 | 0.000 |
| ENSP00000452007 | 5.69E-06 | -0.1828 | 398 | 0.169 |
| ENSP00000384848 | 1.00E-05 | -0.1829 | 0 | 0.068 |
| ENSP00000358753 | 8.09E-06 | -0.1829 | 187 | 0.274 |
| ENSP00000362549 | 3.46E-06 | -0.1829 | 0 | 0.119 |
| ENSP00000352601 | 8.10E-06 | -0.1829 | 561 | 0.115 |
| ENSP00000387362 | 4.33E-06 | -0.1829 | 0 | 0.343 |
| ENSP00000410403 | 2.50E-05 | -0.1829 | 284 | 0.000 |
| ENSP00000350720 | 1.72E-05 | -0.1829 | 836 | 0.000 |
| ENSP00000371243 | 3.44E-06 | -0.1830 | 0 | 0.149 |
| ENSP00000370517 | 1.04E-05 | -0.1830 | 333 | 0.651 |
| ENSP00000343838 | 4.15E-06 | -0.1830 | 0 | 0.165 |
| ENSP00000347978 | 5.48E-06 | -0.1830 | 0 | 0.758 |
| ENSP00000301068 | 9.01E-06 | -0.1830 | 448 | 0.592 |
| ENSP00000424328 | 2.57E-06 | -0.1830 | 0 | 0.170 |
| ENSP00000301391 | 3.74E-06 | -0.1831 | 0 | 0.059 |
| ENSP00000372449 | 3.40E-06 | -0.1831 | 0 | 0.000 |
| ENSP00000327832 | 5.78E-06 | -0.1832 | 0 | 0.150 |
| ENSP00000211413 | 8.66E-06 | -0.1832 | 0 | 0.110 |
| ENSP00000366608 | 2.91E-06 | -0.1833 | 0 | 0.314 |
| ENSP00000336524 | 3.03E-06 | -0.1833 | 0 | 0.141 |
| ENSP00000421191 | 4.98E-06 | -0.1833 | 0 | 0.127 |
| ENSP00000457330 | 2.08E-06 | -0.1834 | 0 | 0.000 |
| ENSP00000332773 | 1.41E-05 | -0.1834 | 749 | 0.649 |
| ENSP00000327763 | 4.47E-06 | -0.1834 | 0 | 0.295 |
| ENSP00000361009 | 9.94E-06 | -0.1834 | 0 | 0.310 |
| ENSP00000281722 | 6.27E-06 | -0.1834 | 0 | 0.205 |
| ENSP00000283871 | 1.56E-05 | -0.1834 | 172 | 0.108 |
| ENSP00000334805 | 1.10E-05 | -0.1834 | 0 | 0.273 |
| ENSP00000450832 | 8.44E-06 | -0.1835 | 0 | 0.338 |
| ENSP00000463999 | 2.82E-05 | -0.1835 | 902 | 0.586 |
| ENSP00000384302 | 9.86E-06 | -0.1835 | 403 | 0.832 |
| ENSP00000363211 | 4.37E-06 | -0.1835 | 185 | 0.169 |
| ENSP00000403293 | 2.50E-06 | -0.1835 | 0 | 0.361 |
| ENSP00000360583 | 3.43E-06 | -0.1835 | 0 | 0.138 |
| ENSP00000378360 | 1.11E-05 | -0.1836 | 159 | 0.000 |
| ENSP00000363128 | 4.29E-06 | -0.1836 | 0 | 0.210 |
| ENSP00000358763 | 1.58E-05 | -0.1836 | 151 | 0.134 |
| ENSP00000266991 | 1.89E-05 | -0.1836 | 281 | 0.888 |
| ENSP00000412315 | 4.00E-06 | -0.1836 | 0 | 0.071 |
| ENSP00000261486 | 7.09E-06 | -0.1837 | 0 | 0.076 |
| ENSP00000382707 | 1.18E-05 | -0.1837 | 156 | 0.486 |
| ENSP00000356785 | 1.14E-05 | -0.1837 | 315 | 0.569 |
| ENSP00000470004 | 1.29E-05 | -0.1838 | 212 | 0.796 |
| ENSP00000364404 | 5.25E-06 | -0.1838 | 0 | 0.121 |
| ENSP00000428325 | 4.57E-06 | -0.1838 | 0 | 0.115 |

| | | | | |
|---|---|---|---|---|
| ENSP00000402151 | 2.04E-06 | -0.1838 | 0 | 0.000 |
| ENSP00000348455 | 3.52E-06 | -0.1838 | 0 | 0.213 |
| ENSP00000395253 | 9.38E-06 | -0.1839 | 231 | 0.000 |
| ENSP00000403495 | 4.37E-06 | -0.1839 | 0 | 0.615 |
| ENSP00000355510 | 1.51E-05 | -0.1839 | 273 | 0.600 |
| ENSP00000396304 | 2.37E-06 | -0.1840 | 0 | 0.127 |
| ENSP00000315997 | 1.65E-05 | -0.1841 | 0 | 0.110 |
| ENSP00000296694 | 1.32E-05 | -0.1841 | 0 | 0.152 |
| ENSP00000351492 | 4.61E-06 | -0.1842 | 0 | 0.504 |
| ENSP00000367802 | 3.26E-06 | -0.1842 | 0 | 0.000 |
| ENSP00000423933 | 2.88E-06 | -0.1842 | 0 | 0.590 |
| ENSP00000388996 | 4.10E-06 | -0.1843 | 0 | 0.211 |
| ENSP00000379754 | 1.60E-06 | -0.1843 | 0 | 0.167 |
| ENSP00000462963 | 2.44E-06 | -0.1844 | 0 | 0.160 |
| ENSP00000383178 | 5.11E-06 | -0.1844 | 167 | 0.410 |
| ENSP00000357475 | 1.04E-05 | -0.1845 | 151 | 0.387 |
| ENSP00000365505 | 1.11E-05 | -0.1845 | 197 | 0.178 |
| ENSP00000417470 | 2.44E-06 | -0.1846 | 0 | 0.681 |
| ENSP00000373343 | 3.82E-06 | -0.1846 | 0 | 0.101 |
| ENSP00000324870 | 1.47E-05 | -0.1846 | 407 | 0.105 |
| ENSP00000363891 | 3.15E-06 | -0.1846 | 0 | 0.051 |
| ENSP00000421871 | 4.79E-06 | -0.1846 | 0 | 0.000 |
| ENSP00000284818 | 1.80E-05 | -0.1846 | 258 | 0.645 |
| ENSP00000362566 | 1.17E-05 | -0.1847 | 349 | 0.302 |
| ENSP00000450685 | 5.57E-07 | -0.1847 | 0 | 0.000 |
| ENSP00000317232 | 1.47E-05 | -0.1847 | 372 | 0.427 |
| ENSP00000416658 | 1.14E-05 | -0.1847 | 315 | 0.594 |
| ENSP00000371559 | 3.99E-06 | -0.1847 | 0 | 0.172 |
| ENSP00000390158 | 1.69E-05 | -0.1848 | 158 | 0.711 |
| ENSP00000385814 | 2.14E-06 | -0.1848 | 0 | 0.300 |
| ENSP00000349493 | 6.81E-06 | -0.1848 | 0 | 0.099 |
| ENSP00000402460 | 8.34E-06 | -0.1850 | 0 | 0.164 |
| ENSP00000386541 | 1.05E-05 | -0.1850 | 167 | 0.537 |
| ENSP00000383840 | 2.56E-06 | -0.1851 | 0 | 0.091 |
| ENSP00000370373 | 1.65E-05 | -0.1851 | 260 | 0.708 |
| ENSP00000371517 | 1.22E-05 | -0.1852 | 151 | 0.275 |
| ENSP00000402527 | 4.08E-06 | -0.1852 | 0 | 0.590 |
| ENSP00000259983 | 2.01E-05 | -0.1852 | 0 | 0.204 |
| ENSP00000343118 | 5.57E-06 | -0.1852 | 0 | 0.125 |
| ENSP00000379310 | 1.74E-05 | -0.1852 | 216 | 0.120 |
| ENSP00000432845 | 2.11E-06 | -0.1852 | 0 | 0.179 |
| ENSP00000296721 | 1.51E-05 | -0.1852 | 720 | 0.090 |
| ENSP00000328090 | 1.81E-05 | -0.1853 | 161 | 0.112 |
| ENSP00000392549 | 4.14E-06 | -0.1853 | 0 | 0.060 |
| ENSP00000367770 | 5.24E-06 | -0.1854 | 0 | 0.097 |
| ENSP00000334668 | 4.46E-06 | -0.1854 | 0 | 0.140 |

| | | | | |
|---|---|---|---|---|
| ENSP00000391751 | 2.19E-05 | -0.1854 | 274 | 0.436 |
| ENSP00000470123 | 2.29E-06 | -0.1854 | 0 | 0.675 |
| ENSP00000367353 | 2.15E-06 | -0.1855 | 0 | 0.102 |
| ENSP00000365012 | 1.58E-05 | -0.1855 | 242 | 0.664 |
| ENSP00000349143 | 5.85E-06 | -0.1855 | 0 | 0.148 |
| ENSP00000344729 | 5.11E-06 | -0.1855 | 0 | 0.135 |
| ENSP00000375783 | 1.88E-06 | -0.1856 | 0 | 0.139 |
| ENSP00000424474 | 3.28E-06 | -0.1856 | 0 | 0.095 |
| ENSP00000381823 | 5.98E-06 | -0.1856 | 196 | 0.182 |
| ENSP00000421853 | 3.11E-06 | -0.1856 | 0 | 0.106 |
| ENSP00000374443 | 1.15E-05 | -0.1858 | 159 | 0.144 |
| ENSP00000366976 | 4.96E-06 | -0.1858 | 0 | 0.124 |
| ENSP00000381795 | 4.27E-06 | -0.1858 | 0 | 0.078 |
| ENSP00000308057 | 1.15E-05 | -0.1858 | 270 | 0.218 |
| ENSP00000443989 | 8.89E-06 | -0.1859 | 311 | 0.000 |
| ENSP00000438285 | 1.54E-06 | -0.1859 | 0 | 0.209 |
| ENSP00000429865 | 1.70E-06 | -0.1859 | 0 | 0.107 |
| ENSP00000378292 | 2.28E-06 | -0.1859 | 0 | 0.128 |
| ENSP00000417935 | 5.25E-06 | -0.1859 | 181 | 0.000 |
| ENSP00000384801 | 2.05E-06 | -0.1860 | 0 | 0.123 |
| ENSP00000351740 | 4.86E-06 | -0.1860 | 0 | 0.090 |
| ENSP00000469533 | 3.35E-06 | -0.1860 | 0 | 0.000 |
| ENSP00000361032 | 6.65E-06 | -0.1860 | 0 | 0.154 |
| ENSP00000276570 | 1.59E-05 | -0.1861 | 920 | 0.242 |
| ENSP00000224600 | 3.02E-05 | -0.1861 | 0 | 0.000 |
| ENSP00000371824 | 1.11E-06 | -0.1861 | 0 | 0.161 |
| ENSP00000429148 | 2.36E-06 | -0.1861 | 0 | 0.698 |
| ENSP00000369349 | 7.39E-06 | -0.1861 | 0 | 0.529 |
| ENSP00000366203 | 2.28E-06 | -0.1861 | 0 | 0.081 |
| ENSP00000378295 | 2.61E-06 | -0.1861 | 0 | 0.427 |
| ENSP00000302579 | 1.52E-05 | -0.1862 | 151 | 0.182 |
| ENSP00000368020 | 1.88E-05 | -0.1862 | 416 | 0.546 |
| ENSP00000291691 | 5.12E-06 | -0.1862 | 0 | 0.128 |
| ENSP00000342576 | 3.56E-06 | -0.1863 | 0 | 0.118 |
| ENSP00000408108 | 2.77E-06 | -0.1863 | 0 | 0.152 |
| ENSP00000353030 | 1.69E-05 | -0.1863 | 294 | 0.817 |
| ENSP00000434849 | 3.58E-06 | -0.1863 | 0 | 0.621 |
| ENSP00000381654 | 5.87E-06 | -0.1864 | 235 | 0.430 |
| ENSP00000272134 | 1.71E-05 | -0.1864 | 161 | 0.872 |
| ENSP00000356632 | 5.45E-06 | -0.1864 | 0 | 0.094 |
| ENSP00000439856 | 3.04E-06 | -0.1864 | 0 | 0.455 |
| ENSP00000364742 | 3.40E-06 | -0.1865 | 0 | 0.250 |
| ENSP00000354886 | 4.35E-06 | -0.1865 | 0 | 0.088 |
| ENSP00000417556 | 1.01E-06 | -0.1865 | 0 | 0.000 |
| ENSP00000307552 | 1.98E-05 | -0.1866 | 256 | 0.120 |
| ENSP00000357907 | 1.06E-05 | -0.1866 | 551 | 0.000 |

| | | | | |
|---|---|---|---|---|
| ENSP00000470473 | 2.84E-07 | -0.1866 | 0 | 0.000 |
| ENSP00000380824 | 4.79E-06 | -0.1867 | 0 | 0.270 |
| ENSP00000354871 | 2.10E-05 | -0.1867 | 963 | 0.654 |
| ENSP00000355721 | 1.53E-05 | -0.1868 | 216 | 0.229 |
| ENSP00000275275 | 1.00E-05 | -0.1868 | 0 | 0.166 |
| ENSP00000383692 | 7.13E-07 | -0.1868 | 0 | 0.212 |
| ENSP00000355632 | 8.81E-06 | -0.1869 | 268 | 0.198 |
| ENSP00000377747 | 1.97E-05 | -0.1869 | 196 | 0.937 |
| ENSP00000317289 | 8.25E-06 | -0.1870 | 0 | 0.122 |
| ENSP00000410626 | 4.29E-06 | -0.1871 | 0 | 0.456 |
| ENSP00000278780 | 5.28E-06 | -0.1871 | 0 | 0.452 |
| ENSP00000389934 | 9.40E-06 | -0.1871 | 0 | 0.237 |
| ENSP00000473172 | 2.33E-05 | -0.1871 | 403 | 0.870 |
| ENSP00000370658 | 3.06E-06 | -0.1872 | 0 | 0.173 |
| ENSP00000388293 | 2.84E-06 | -0.1872 | 0 | 0.249 |
| ENSP00000400587 | 2.32E-06 | -0.1872 | 0 | 0.000 |
| ENSP00000329292 | 5.08E-06 | -0.1873 | 0 | 0.115 |
| ENSP00000408860 | 4.11E-06 | -0.1873 | 0 | 0.270 |
| ENSP00000333697 | 4.06E-06 | -0.1873 | 0 | 0.184 |
| ENSP00000471635 | 5.89E-07 | -0.1874 | 0 | 0.315 |
| ENSP00000359392 | 2.91E-06 | -0.1874 | 0 | 0.210 |
| ENSP00000333142 | 1.32E-05 | -0.1874 | 214 | 0.704 |
| ENSP00000348316 | 4.06E-06 | -0.1874 | 0 | 0.083 |
| ENSP00000417895 | 4.39E-06 | -0.1874 | 0 | 0.301 |
| ENSP00000363021 | 3.75E-06 | -0.1874 | 0 | 0.743 |
| ENSP00000360195 | 7.82E-06 | -0.1874 | 201 | 0.609 |
| ENSP00000472467 | 2.51E-06 | -0.1875 | 0 | 0.114 |
| ENSP00000382239 | 2.55E-06 | -0.1875 | 0 | 0.263 |
| ENSP00000216471 | 1.24E-05 | -0.1875 | 0 | 0.204 |
| ENSP00000368119 | 7.45E-06 | -0.1876 | 0 | 0.097 |
| ENSP00000401277 | 3.06E-06 | -0.1876 | 0 | 0.297 |
| ENSP00000275200 | 8.37E-06 | -0.1876 | 0 | 0.089 |
| ENSP00000306241 | 6.41E-06 | -0.1876 | 0 | 0.000 |
| ENSP00000452237 | 1.87E-06 | -0.1877 | 0 | 0.121 |
| ENSP00000367428 | 9.46E-06 | -0.1877 | 625 | 0.568 |
| ENSP00000356430 | 1.04E-05 | -0.1878 | 188 | 0.576 |
| ENSP00000466298 | 4.08E-06 | -0.1878 | 0 | 0.220 |
| ENSP00000365569 | 5.49E-06 | -0.1878 | 0 | 0.152 |
| ENSP00000426741 | 3.05E-06 | -0.1878 | 0 | 0.000 |
| ENSP00000380661 | 4.13E-06 | -0.1879 | 0 | 0.368 |
| ENSP00000374268 | 3.20E-06 | -0.1880 | 0 | 0.064 |
| ENSP00000346643 | 4.45E-06 | -0.1880 | 202 | 0.654 |
| ENSP00000377978 | 1.50E-05 | -0.1880 | 197 | 0.000 |
| ENSP00000383225 | 1.37E-06 | -0.1880 | 0 | 0.163 |
| ENSP00000405290 | 5.94E-06 | -0.1881 | 0 | 0.772 |
| ENSP00000257637 | 1.48E-05 | -0.1881 | 0 | 0.095 |

| | | | | |
|---|---|---|---|---|
| ENSP00000457071 | 6.98E-07 | -0.1881 | 0 | 0.085 |
| ENSP00000343686 | 6.12E-06 | -0.1882 | 0 | 0.182 |
| ENSP00000429064 | 1.08E-05 | -0.1882 | 181 | 0.457 |
| ENSP00000302327 | 5.67E-06 | -0.1882 | 0 | 0.756 |
| ENSP00000264094 | 1.64E-05 | -0.1882 | 0 | 0.123 |
| ENSP00000358696 | 5.68E-06 | -0.1883 | 0 | 0.520 |
| ENSP00000372982 | 4.03E-06 | -0.1883 | 0 | 0.129 |
| ENSP00000370419 | 4.11E-06 | -0.1883 | 0 | 0.143 |
| ENSP00000455755 | 1.67E-06 | -0.1883 | 0 | 0.000 |
| ENSP00000384408 | 4.59E-06 | -0.1883 | 0 | 0.447 |
| ENSP00000310622 | 6.21E-06 | -0.1884 | 0 | 0.086 |
| ENSP00000318409 | 6.68E-06 | -0.1884 | 0 | 0.168 |
| ENSP00000401018 | 2.59E-06 | -0.1884 | 0 | 0.375 |
| ENSP00000374423 | 1.15E-05 | -0.1885 | 0 | 0.000 |
| ENSP00000367318 | 1.90E-06 | -0.1885 | 0 | 0.158 |
| ENSP00000355022 | 4.96E-06 | -0.1885 | 0 | 0.113 |
| ENSP00000361871 | 2.82E-06 | -0.1885 | 0 | 0.132 |
| ENSP00000467041 | 1.39E-06 | -0.1886 | 0 | 0.000 |
| ENSP00000431538 | 9.28E-06 | -0.1886 | 181 | 0.593 |
| ENSP00000420174 | 5.07E-06 | -0.1886 | 0 | 0.120 |
| ENSP00000310547 | 2.67E-06 | -0.1887 | 0 | 0.055 |
| ENSP00000366475 | 1.09E-05 | -0.1887 | 183 | 0.051 |
| ENSP00000309786 | 4.80E-06 | -0.1887 | 0 | 0.000 |
| ENSP00000384597 | 2.12E-06 | -0.1887 | 0 | 0.545 |
| ENSP00000331466 | 9.01E-06 | -0.1887 | 0 | 0.137 |
| ENSP00000369716 | 2.05E-05 | -0.1888 | 196 | 0.923 |
| ENSP00000354090 | 8.23E-06 | -0.1888 | 0 | 0.117 |
| ENSP00000357360 | 4.52E-06 | -0.1888 | 0 | 0.053 |
| ENSP00000353677 | 3.50E-06 | -0.1888 | 0 | 0.099 |
| ENSP00000330475 | 6.91E-06 | -0.1889 | 0 | 0.137 |
| ENSP00000361158 | 4.07E-06 | -0.1889 | 205 | 0.674 |
| ENSP00000347427 | 1.02E-05 | -0.1890 | 0 | 0.462 |
| ENSP00000384700 | 1.72E-05 | -0.1890 | 192 | 0.818 |
| ENSP00000468743 | 2.51E-06 | -0.1890 | 0 | 0.000 |
| ENSP00000335185 | 1.04E-05 | -0.1891 | 402 | 0.583 |
| ENSP00000472249 | 5.64E-06 | -0.1891 | 0 | 0.100 |
| ENSP00000376946 | 2.06E-05 | -0.1891 | 760 | 0.694 |
| ENSP00000357591 | 3.00E-06 | -0.1891 | 0 | 0.000 |
| ENSP00000361097 | 8.48E-06 | -0.1892 | 0 | 0.131 |
| ENSP00000370611 | 4.93E-06 | -0.1892 | 0 | 0.191 |
| ENSP00000398089 | 1.36E-06 | -0.1892 | 0 | 0.222 |
| ENSP00000260386 | 4.03E-06 | -0.1892 | 0 | 0.140 |
| ENSP00000342840 | 4.80E-06 | -0.1892 | 0 | 0.158 |
| ENSP00000216211 | 1.39E-05 | -0.1892 | 0 | 0.177 |
| ENSP00000424040 | 1.86E-06 | -0.1892 | 0 | 0.132 |
| ENSP00000354360 | 9.00E-06 | -0.1893 | 0 | 0.276 |

| | | | | |
|---|---|---|---|---|
| ENSP00000438378 | 4.28E-06 | -0.1893 | 0 | 0.174 |
| ENSP00000368790 | 1.10E-05 | -0.1893 | 290 | 0.583 |
| ENSP00000371847 | 2.04E-06 | -0.1893 | 0 | 0.134 |
| ENSP00000355966 | 8.76E-06 | -0.1894 | 169 | 0.419 |
| ENSP00000438205 | 1.23E-05 | -0.1894 | 0 | 0.112 |
| ENSP00000367227 | 2.69E-06 | -0.1894 | 0 | 0.073 |
| ENSP00000367422 | 3.73E-06 | -0.1895 | 0 | 0.091 |
| ENSP00000366876 | 5.72E-06 | -0.1895 | 0 | 0.154 |
| ENSP00000332162 | 4.46E-06 | -0.1895 | 0 | 0.132 |
| ENSP00000407487 | 4.50E-06 | -0.1895 | 0 | 0.208 |
| ENSP00000357625 | 1.25E-05 | -0.1895 | 173 | 0.514 |
| ENSP00000418823 | 1.48E-05 | -0.1895 | 323 | 0.606 |
| ENSP00000253410 | 8.05E-06 | -0.1896 | 0 | 0.123 |
| ENSP00000290015 | 1.61E-05 | -0.1896 | 507 | 0.935 |
| ENSP00000363019 | 1.83E-05 | -0.1896 | 322 | 0.692 |
| ENSP00000335255 | 8.81E-06 | -0.1896 | 0 | 0.131 |
| ENSP00000334276 | 2.91E-06 | -0.1897 | 0 | 0.057 |
| ENSP00000366939 | 1.08E-05 | -0.1897 | 159 | 0.208 |
| ENSP00000329303 | 7.47E-06 | -0.1897 | 0 | 0.000 |
| ENSP00000363205 | 5.32E-06 | -0.1898 | 413 | 0.314 |
| ENSP00000223795 | 1.19E-05 | -0.1898 | 0 | 0.479 |
| ENSP00000341013 | 6.32E-06 | -0.1898 | 0 | 0.114 |
| ENSP00000366390 | 6.83E-06 | -0.1898 | 0 | 0.128 |
| ENSP00000364526 | 4.46E-06 | -0.1899 | 0 | 0.100 |
| ENSP00000355840 | 2.54E-06 | -0.1899 | 0 | 0.000 |
| ENSP00000389787 | 4.09E-06 | -0.1899 | 0 | 0.084 |
| ENSP00000355551 | 3.98E-06 | -0.1899 | 0 | 0.100 |
| ENSP00000300258 | 7.80E-06 | -0.1899 | 0 | 0.158 |
| ENSP00000422581 | 2.76E-06 | -0.1899 | 0 | 0.310 |
| ENSP00000315945 | 5.69E-06 | -0.1899 | 0 | 0.101 |
| ENSP00000368468 | 1.01E-05 | -0.1900 | 0 | 0.072 |
| ENSP00000458021 | 1.30E-06 | -0.1900 | 0 | 0.000 |
| ENSP00000387347 | 5.06E-06 | -0.1900 | 272 | 0.327 |
| ENSP00000375259 | 7.98E-07 | -0.1900 | 0 | 0.000 |
| ENSP00000394405 | 2.52E-06 | -0.1901 | 0 | 0.072 |
| ENSP00000430031 | 1.32E-05 | -0.1901 | 250 | 0.557 |
| ENSP00000352173 | 4.48E-06 | -0.1901 | 0 | 0.139 |
| ENSP00000298943 | 6.87E-06 | -0.1901 | 0 | 0.142 |
| ENSP00000397050 | 3.35E-06 | -0.1902 | 0 | 0.619 |
| ENSP00000428209 | 1.09E-05 | -0.1903 | 569 | 0.682 |
| ENSP00000362121 | 6.77E-06 | -0.1903 | 211 | 0.128 |
| ENSP00000379901 | 5.93E-06 | -0.1904 | 159 | 0.095 |
| ENSP00000366299 | 6.49E-06 | -0.1904 | 205 | 0.103 |
| ENSP00000368062 | 2.40E-06 | -0.1904 | 0 | 0.484 |
| ENSP00000361110 | 1.04E-05 | -0.1904 | 191 | 0.179 |
| ENSP00000300303 | 1.95E-05 | -0.1905 | 0 | 0.134 |

| | | | | |
|---|---|---|---|---|
| ENSP00000386846 | 1.02E-06 | -0.1905 | 0 | 0.000 |
| ENSP00000223273 | 1.16E-05 | -0.1905 | 0 | 0.162 |
| ENSP00000346340 | 1.03E-05 | -0.1905 | 408 | 0.335 |
| ENSP00000261669 | 1.45E-05 | -0.1905 | 588 | 0.088 |
| ENSP00000367049 | 2.26E-06 | -0.1905 | 0 | 0.072 |
| ENSP00000378965 | 1.01E-05 | -0.1905 | 195 | 0.199 |
| ENSP00000348632 | 3.39E-06 | -0.1906 | 0 | 0.349 |
| ENSP00000256897 | 3.86E-06 | -0.1906 | 0 | 0.775 |
| ENSP00000416549 | 2.47E-10 | -0.1906 | 0 | 0.000 |
| ENSP00000410198 | 2.47E-06 | -0.1906 | 0 | 0.694 |
| ENSP00000282849 | 1.69E-05 | -0.1906 | 167 | 0.315 |
| ENSP00000361813 | 1.76E-06 | -0.1907 | 0 | 0.135 |
| ENSP00000229570 | 1.51E-05 | -0.1907 | 0 | 0.217 |
| ENSP00000352222 | 1.05E-05 | -0.1907 | 334 | 0.711 |
| ENSP00000359567 | 6.58E-06 | -0.1907 | 0 | 0.838 |
| ENSP00000354669 | 7.50E-06 | -0.1908 | 223 | 0.398 |
| ENSP00000378635 | 2.73E-06 | -0.1908 | 0 | 0.118 |
| ENSP00000379026 | 2.75E-06 | -0.1909 | 0 | 0.000 |
| ENSP00000369614 | 2.64E-06 | -0.1909 | 0 | 0.071 |
| ENSP00000357448 | 3.18E-06 | -0.1909 | 0 | 0.197 |
| ENSP00000360968 | 3.79E-06 | -0.1909 | 0 | 0.160 |
| ENSP00000386134 | 6.10E-06 | -0.1910 | 169 | 0.847 |
| ENSP00000223364 | 1.45E-05 | -0.1910 | 178 | 0.446 |
| ENSP00000432695 | 2.73E-06 | -0.1910 | 0 | 0.529 |
| ENSP00000379568 | 2.63E-06 | -0.1910 | 0 | 0.095 |
| ENSP00000377558 | 1.92E-06 | -0.1910 | 0 | 0.082 |
| ENSP00000449795 | 7.04E-06 | -0.1911 | 150 | 0.240 |
| ENSP00000346997 | 8.08E-06 | -0.1912 | 244 | 0.000 |
| ENSP00000410768 | 3.23E-06 | -0.1912 | 0 | 0.170 |
| ENSP00000457780 | 1.93E-06 | -0.1912 | 0 | 0.000 |
| ENSP00000409279 | 7.77E-06 | -0.1913 | 0 | 0.097 |
| ENSP00000397441 | 4.60E-06 | -0.1913 | 0 | 0.152 |
| ENSP00000355250 | 2.19E-06 | -0.1913 | 0 | 0.146 |
| ENSP00000300576 | 4.41E-06 | -0.1913 | 0 | 0.160 |
| ENSP00000348463 | 7.55E-06 | -0.1913 | 0 | 0.000 |
| ENSP00000364217 | 5.88E-06 | -0.1914 | 0 | 0.320 |
| ENSP00000373331 | 4.30E-06 | -0.1914 | 236 | 0.659 |
| ENSP00000361095 | 3.01E-06 | -0.1914 | 0 | 0.099 |
| ENSP00000356389 | 4.97E-06 | -0.1914 | 0 | 0.106 |
| ENSP00000434676 | 9.84E-06 | -0.1914 | 223 | 0.219 |
| ENSP00000370930 | 8.56E-06 | -0.1914 | 0 | 0.560 |
| ENSP00000355560 | 6.49E-06 | -0.1914 | 212 | 0.322 |
| ENSP00000358909 | 9.16E-06 | -0.1915 | 0 | 0.080 |
| ENSP00000358120 | 3.99E-06 | -0.1915 | 0 | 0.127 |
| ENSP00000281171 | 1.09E-05 | -0.1916 | 206 | 0.656 |
| ENSP00000394510 | 2.59E-06 | -0.1916 | 0 | 0.104 |

| | | | | |
|---|---|---|---|---|
| ENSP00000366612 | 3.33E-06 | -0.1916 | 0 | 0.331 |
| ENSP00000322609 | 6.12E-06 | -0.1917 | 0 | 0.000 |
| ENSP00000417970 | 2.28E-06 | -0.1917 | 0 | 0.330 |
| ENSP00000352785 | 1.23E-05 | -0.1917 | 158 | 0.158 |
| ENSP00000419542 | 3.89E-06 | -0.1918 | 0 | 0.614 |
| ENSP00000412215 | 1.94E-06 | -0.1918 | 0 | 0.127 |
| ENSP00000347635 | 5.64E-06 | -0.1918 | 0 | 0.135 |
| ENSP00000258106 | 1.02E-05 | -0.1918 | 0 | 0.845 |
| ENSP00000331704 | 4.56E-06 | -0.1918 | 0 | 0.131 |
| ENSP00000418802 | 3.73E-06 | -0.1919 | 0 | 0.000 |
| ENSP00000365251 | 1.41E-05 | -0.1919 | 261 | 0.071 |
| ENSP00000259938 | 1.48E-05 | -0.1919 | 311 | 0.605 |
| ENSP00000446264 | 8.11E-07 | -0.1920 | 0 | 0.000 |
| ENSP00000360988 | 1.79E-06 | -0.1920 | 0 | 0.110 |
| ENSP00000378890 | 1.43E-05 | -0.1920 | 394 | 0.550 |
| ENSP00000312126 | 4.83E-06 | -0.1921 | 0 | 0.645 |
| ENSP00000311218 | 7.88E-06 | -0.1921 | 0 | 0.127 |
| ENSP00000361083 | 8.37E-06 | -0.1922 | 0 | 0.149 |
| ENSP00000378605 | 1.26E-05 | -0.1922 | 776 | 0.144 |
| ENSP00000438465 | 8.10E-06 | -0.1922 | 0 | 0.219 |
| ENSP00000368464 | 3.27E-06 | -0.1923 | 0 | 0.228 |
| ENSP00000354481 | 6.26E-06 | -0.1923 | 0 | 0.000 |
| ENSP00000308716 | 8.58E-06 | -0.1923 | 0 | 0.835 |
| ENSP00000419088 | 4.71E-06 | -0.1924 | 0 | 0.120 |
| ENSP00000393854 | 4.91E-06 | -0.1924 | 0 | 0.137 |
| ENSP00000373575 | 5.38E-06 | -0.1924 | 0 | 0.169 |
| ENSP00000378072 | 1.07E-05 | -0.1924 | 189 | 0.114 |
| ENSP00000366418 | 2.80E-06 | -0.1924 | 0 | 0.112 |
| ENSP00000425048 | 1.71E-06 | -0.1925 | 0 | 0.129 |
| ENSP00000361800 | 2.19E-06 | -0.1925 | 0 | 0.220 |
| ENSP00000356634 | 8.81E-06 | -0.1925 | 0 | 0.144 |
| ENSP00000357153 | 1.77E-05 | -0.1925 | 224 | 0.728 |
| ENSP00000371805 | 3.94E-06 | -0.1926 | 0 | 0.129 |
| ENSP00000324834 | 1.09E-05 | -0.1926 | 202 | 0.000 |
| ENSP00000366656 | 2.90E-06 | -0.1927 | 0 | 0.000 |
| ENSP00000361725 | 1.26E-05 | -0.1927 | 328 | 0.542 |
| ENSP00000350854 | 1.18E-05 | -0.1927 | 375 | 0.000 |
| ENSP00000358320 | 3.15E-06 | -0.1928 | 0 | 0.234 |
| ENSP00000405674 | 2.51E-06 | -0.1928 | 0 | 0.419 |
| ENSP00000416193 | 3.62E-06 | -0.1928 | 0 | 0.080 |
| ENSP00000345774 | 1.02E-05 | -0.1929 | 256 | 0.346 |
| ENSP00000362116 | 8.29E-06 | -0.1929 | 231 | 0.262 |
| ENSP00000386772 | 3.70E-06 | -0.1930 | 0 | 0.288 |
| ENSP00000360876 | 1.60E-05 | -0.1930 | 326 | 0.704 |
| ENSP00000361875 | 5.48E-06 | -0.1930 | 0 | 0.108 |
| ENSP00000248600 | 3.95E-06 | -0.1930 | 0 | 0.623 |

| | | | | |
|---|---|---|---|---|
| ENSP00000305151 | 1.58E-05 | -0.1930 | 0 | 0.144 |
| ENSP00000360530 | 1.19E-05 | -0.1930 | 165 | 0.716 |
| ENSP00000329638 | 2.24E-06 | -0.1931 | 0 | 0.164 |
| ENSP00000415978 | 1.36E-06 | -0.1931 | 0 | 0.000 |
| ENSP00000301039 | 1.39E-05 | -0.1932 | 0 | 0.508 |
| ENSP00000353820 | 9.08E-06 | -0.1933 | 358 | 0.597 |
| ENSP00000328232 | 3.09E-06 | -0.1933 | 0 | 0.377 |
| ENSP00000348205 | 5.58E-06 | -0.1933 | 161 | 0.445 |
| ENSP00000347454 | 5.22E-06 | -0.1933 | 0 | 0.192 |
| ENSP00000361336 | 6.89E-06 | -0.1933 | 0 | 0.126 |
| ENSP00000458290 | 1.95E-06 | -0.1933 | 0 | 0.085 |
| ENSP00000406546 | 2.94E-06 | -0.1934 | 0 | 0.082 |
| ENSP00000409799 | 2.90E-06 | -0.1934 | 0 | 0.229 |
| ENSP00000382717 | 8.19E-06 | -0.1934 | 0 | 0.068 |
| ENSP00000365692 | 1.01E-05 | -0.1935 | 810 | 0.687 |
| ENSP00000402644 | 9.76E-07 | -0.1935 | 0 | 0.103 |
| ENSP00000376465 | 1.01E-05 | -0.1935 | 165 | 0.165 |
| ENSP00000362403 | 1.41E-05 | -0.1935 | 169 | 0.544 |
| ENSP00000400113 | 1.92E-05 | -0.1936 | 206 | 0.000 |
| ENSP00000358812 | 1.42E-05 | -0.1936 | 356 | 0.910 |
| ENSP00000309402 | 4.31E-06 | -0.1936 | 0 | 0.136 |
| ENSP00000349351 | 3.04E-06 | -0.1936 | 0 | 0.150 |
| ENSP00000284486 | 3.81E-06 | -0.1936 | 0 | 0.177 |
| ENSP00000411593 | 1.22E-05 | -0.1937 | 200 | 0.421 |
| ENSP00000418070 | 4.69E-06 | -0.1937 | 0 | 0.235 |
| ENSP00000355237 | 5.06E-06 | -0.1937 | 205 | 0.676 |
| ENSP00000375267 | 2.54E-06 | -0.1938 | 0 | 0.130 |
| ENSP00000320207 | 3.26E-06 | -0.1938 | 0 | 0.000 |
| ENSP00000355014 | 4.82E-06 | -0.1938 | 0 | 0.419 |
| ENSP00000421586 | 3.65E-06 | -0.1938 | 0 | 0.100 |
| ENSP00000347924 | 2.80E-06 | -0.1939 | 0 | 0.096 |
| ENSP00000440704 | 1.83E-06 | -0.1939 | 0 | 0.077 |
| ENSP00000296506 | 1.03E-05 | -0.1939 | 0 | 0.118 |
| ENSP00000455585 | 2.96E-06 | -0.1940 | 0 | 0.675 |
| ENSP00000345773 | 1.85E-06 | -0.1940 | 0 | 0.211 |
| ENSP00000333212 | 4.93E-06 | -0.1941 | 0 | 0.214 |
| ENSP00000358424 | 6.83E-06 | -0.1941 | 198 | 0.443 |
| ENSP00000295759 | 6.62E-06 | -0.1941 | 0 | 0.079 |
| ENSP00000365714 | 2.62E-06 | -0.1941 | 0 | 0.151 |
| ENSP00000382713 | 5.41E-06 | -0.1941 | 0 | 0.082 |
| ENSP00000348639 | 6.91E-06 | -0.1941 | 0 | 0.161 |
| ENSP00000358813 | 7.89E-06 | -0.1941 | 178 | 0.270 |
| ENSP00000278853 | 3.40E-06 | -0.1942 | 0 | 0.083 |
| ENSP00000355198 | 5.30E-06 | -0.1942 | 0 | 0.209 |
| ENSP00000358233 | 5.65E-06 | -0.1943 | 0 | 0.087 |
| ENSP00000471531 | 1.06E-07 | -0.1943 | 0 | 0.000 |

| | | | | |
|---|---|---|---|---|
| ENSP00000365492 | 8.50E-06 | -0.1943 | 0 | 0.124 |
| ENSP00000384651 | 3.02E-06 | -0.1943 | 0 | 0.077 |
| ENSP00000350541 | 1.26E-05 | -0.1943 | 546 | 0.000 |
| ENSP00000356828 | 3.55E-06 | -0.1944 | 0 | 0.147 |
| ENSP00000360806 | 4.46E-06 | -0.1944 | 0 | 0.332 |
| ENSP00000352584 | 1.92E-05 | -0.1944 | 301 | 0.366 |
| ENSP00000406598 | 2.50E-06 | -0.1945 | 0 | 0.178 |
| ENSP00000362532 | 3.03E-06 | -0.1945 | 0 | 0.442 |
| ENSP00000437968 | 2.01E-06 | -0.1945 | 0 | 0.096 |
| ENSP00000442349 | 1.09E-05 | -0.1945 | 198 | 0.000 |
| ENSP00000300399 | 4.87E-06 | -0.1945 | 0 | 0.121 |
| ENSP00000462664 | 1.46E-05 | -0.1946 | 215 | 0.818 |
| ENSP00000364699 | 7.27E-06 | -0.1946 | 215 | 0.431 |
| ENSP00000435517 | 3.15E-06 | -0.1946 | 0 | 0.130 |
| ENSP00000242729 | 1.78E-05 | -0.1947 | 177 | 0.076 |
| ENSP00000347464 | 6.37E-06 | -0.1947 | 0 | 0.367 |
| ENSP00000347601 | 3.52E-06 | -0.1947 | 0 | 0.000 |
| ENSP00000300184 | 1.05E-05 | -0.1948 | 0 | 0.189 |
| ENSP00000381486 | 1.28E-05 | -0.1948 | 167 | 0.622 |
| ENSP00000366901 | 3.19E-06 | -0.1948 | 0 | 0.089 |
| ENSP00000361917 | 8.44E-06 | -0.1948 | 0 | 0.195 |
| ENSP00000410216 | 1.89E-06 | -0.1948 | 0 | 0.085 |
| ENSP00000387426 | 1.18E-05 | -0.1949 | 0 | 0.190 |
| ENSP00000261070 | 3.39E-06 | -0.1949 | 0 | 0.178 |
| ENSP00000367715 | 3.81E-06 | -0.1949 | 0 | 0.096 |
| ENSP00000357776 | 4.38E-06 | -0.1949 | 0 | 0.135 |
| ENSP00000319170 | 9.63E-06 | -0.1950 | 353 | 0.457 |
| ENSP00000368237 | 2.66E-06 | -0.1951 | 0 | 0.111 |
| ENSP00000359609 | 8.97E-06 | -0.1951 | 163 | 0.115 |
| ENSP00000369611 | 3.35E-06 | -0.1951 | 0 | 0.208 |
| ENSP00000362525 | 5.00E-06 | -0.1951 | 0 | 0.161 |
| ENSP00000347586 | 7.32E-06 | -0.1952 | 0 | 0.158 |
| ENSP00000346600 | 4.55E-06 | -0.1952 | 0 | 0.000 |
| ENSP00000357748 | 4.96E-06 | -0.1952 | 163 | 0.812 |
| ENSP00000404676 | 5.27E-06 | -0.1953 | 0 | 0.221 |
| ENSP00000472810 | 3.68E-07 | -0.1953 | 0 | 0.000 |
| ENSP00000369346 | 6.23E-06 | -0.1953 | 0 | 0.131 |
| ENSP00000329668 | 1.67E-05 | -0.1953 | 158 | 0.733 |
| ENSP00000348852 | 3.50E-06 | -0.1953 | 0 | 0.133 |
| ENSP00000417587 | 1.18E-05 | -0.1953 | 282 | 0.582 |
| ENSP00000424363 | 1.02E-05 | -0.1954 | 163 | 0.000 |
| ENSP00000367615 | 1.11E-05 | -0.1954 | 367 | 0.573 |
| ENSP00000351484 | 7.47E-06 | -0.1954 | 0 | 0.281 |
| ENSP00000406925 | 3.59E-06 | -0.1955 | 0 | 0.157 |
| ENSP00000334708 | 3.63E-06 | -0.1955 | 0 | 0.150 |
| ENSP00000315111 | 9.54E-06 | -0.1956 | 0 | 0.000 |

| | | | | |
|---|---|---|---|---|
| ENSP00000386203 | 1.59E-06 | -0.1957 | 0 | 0.000 |
| ENSP00000439146 | 9.02E-06 | -0.1957 | 288 | 0.000 |
| ENSP00000315203 | 1.16E-05 | -0.1957 | 0 | 0.191 |
| ENSP00000357484 | 1.17E-05 | -0.1957 | 323 | 0.209 |
| ENSP00000360299 | 5.77E-06 | -0.1957 | 0 | 0.000 |
| ENSP00000289820 | 6.64E-06 | -0.1957 | 0 | 0.347 |
| ENSP00000383431 | 2.60E-06 | -0.1957 | 0 | 0.475 |
| ENSP00000386764 | 3.42E-06 | -0.1958 | 0 | 0.562 |
| ENSP00000394757 | 3.62E-06 | -0.1958 | 0 | 0.690 |
| ENSP00000378851 | 3.02E-06 | -0.1958 | 0 | 0.100 |
| ENSP00000354884 | 1.04E-05 | -0.1959 | 174 | 0.168 |
| ENSP00000386359 | 4.19E-06 | -0.1959 | 0 | 0.139 |
| ENSP00000312942 | 4.00E-06 | -0.1959 | 0 | 0.073 |
| ENSP00000464146 | 1.27E-05 | -0.1960 | 199 | 0.000 |
| ENSP00000358146 | 2.48E-06 | -0.1960 | 0 | 0.139 |
| ENSP00000340474 | 6.46E-06 | -0.1960 | 0 | 0.344 |
| ENSP00000360644 | 1.03E-05 | -0.1960 | 229 | 0.513 |
| ENSP00000295682 | 5.10E-06 | -0.1961 | 0 | 0.118 |
| ENSP00000430706 | 1.38E-05 | -0.1961 | 266 | 0.000 |
| ENSP00000333833 | 6.06E-06 | -0.1961 | 0 | 0.145 |
| ENSP00000354223 | 3.30E-06 | -0.1961 | 0 | 0.125 |
| ENSP00000469958 | 5.70E-06 | -0.1961 | 0 | 0.665 |
| ENSP00000352522 | 5.13E-06 | -0.1962 | 0 | 0.162 |
| ENSP00000356982 | 4.02E-06 | -0.1962 | 0 | 0.076 |
| ENSP00000386190 | 3.61E-06 | -0.1962 | 0 | 0.130 |
| ENSP00000402697 | 7.26E-06 | -0.1962 | 169 | 0.000 |
| ENSP00000354432 | 6.67E-06 | -0.1963 | 202 | 0.653 |
| ENSP00000419970 | 1.03E-05 | -0.1963 | 178 | 0.377 |
| ENSP00000349887 | 7.03E-06 | -0.1963 | 202 | 0.165 |
| ENSP00000366672 | 3.13E-06 | -0.1964 | 0 | 0.236 |
| ENSP00000289448 | 3.22E-06 | -0.1964 | 0 | 0.172 |
| ENSP00000361731 | 1.77E-06 | -0.1964 | 0 | 0.219 |
| ENSP00000381717 | 2.46E-05 | -0.1964 | 322 | 0.678 |
| ENSP00000418721 | 4.19E-06 | -0.1964 | 0 | 0.208 |
| ENSP00000366677 | 2.79E-06 | -0.1964 | 0 | 0.000 |
| ENSP00000364261 | 2.74E-06 | -0.1965 | 0 | 0.107 |
| ENSP00000378312 | 1.30E-06 | -0.1965 | 0 | 0.105 |
| ENSP00000454657 | 5.51E-06 | -0.1966 | 175 | 0.000 |
| ENSP00000353013 | 3.43E-06 | -0.1966 | 0 | 0.113 |
| ENSP00000362041 | 1.03E-05 | -0.1966 | 718 | 0.402 |
| ENSP00000448182 | 7.77E-06 | -0.1967 | 0 | 0.187 |
| ENSP00000370394 | 1.78E-06 | -0.1968 | 0 | 0.091 |
| ENSP00000366506 | 5.81E-06 | -0.1968 | 0 | 0.459 |
| ENSP00000381785 | 8.14E-06 | -0.1968 | 476 | 0.313 |
| ENSP00000355895 | 7.07E-06 | -0.1968 | 0 | 0.060 |
| ENSP00000394954 | 3.76E-06 | -0.1968 | 0 | 0.083 |

| | | | | |
|---|---|---|---|---|
| ENSP00000382157 | 9.52E-07 | -0.1969 | 0 | 0.000 |
| ENSP00000357219 | 7.04E-06 | -0.1969 | 316 | 0.702 |
| ENSP00000421915 | 2.40E-06 | -0.1969 | 0 | 0.680 |
| ENSP00000417548 | 8.72E-06 | -0.1969 | 803 | 0.223 |
| ENSP00000320416 | 7.74E-06 | -0.1969 | 0 | 0.080 |
| ENSP00000340434 | 5.97E-06 | -0.1969 | 0 | 0.109 |
| ENSP00000365388 | 8.60E-06 | -0.1969 | 167 | 0.355 |
| ENSP00000353670 | 3.29E-06 | -0.1969 | 0 | 0.532 |
| ENSP00000298223 | 5.23E-06 | -0.1970 | 0 | 0.077 |
| ENSP00000359675 | 8.04E-06 | -0.1970 | 0 | 0.430 |
| ENSP00000354039 | 3.58E-06 | -0.1971 | 0 | 0.178 |
| ENSP00000250825 | 1.19E-05 | -0.1971 | 0 | 0.213 |
| ENSP00000349785 | 3.83E-06 | -0.1971 | 0 | 0.253 |
| ENSP00000369981 | 1.28E-05 | -0.1971 | 260 | 0.586 |
| ENSP00000366178 | 6.26E-06 | -0.1971 | 0 | 0.121 |
| ENSP00000433821 | 1.09E-05 | -0.1972 | 787 | 0.850 |
| ENSP00000446280 | 3.23E-05 | -0.1972 | 468 | 0.632 |
| ENSP00000340590 | 6.43E-06 | -0.1972 | 0 | 0.092 |
| ENSP00000414517 | 8.29E-06 | -0.1972 | 0 | 0.197 |
| ENSP00000355955 | 8.07E-06 | -0.1972 | 0 | 0.086 |
| ENSP00000384917 | 1.00E-05 | -0.1973 | 684 | 0.117 |
| ENSP00000357008 | 1.05E-05 | -0.1973 | 0 | 0.084 |
| ENSP00000272324 | 2.00E-05 | -0.1973 | 159 | 0.350 |
| ENSP00000386156 | 9.99E-06 | -0.1973 | 213 | 0.000 |
| ENSP00000355499 | 4.76E-06 | -0.1974 | 0 | 0.057 |
| ENSP00000430128 | 1.06E-05 | -0.1974 | 0 | 0.978 |
| ENSP00000301332 | 3.45E-06 | -0.1974 | 0 | 0.257 |
| ENSP00000369513 | 1.36E-06 | -0.1975 | 0 | 0.060 |
| ENSP00000353769 | 3.57E-06 | -0.1976 | 0 | 0.219 |
| ENSP00000341867 | 6.77E-06 | -0.1976 | 0 | 0.110 |
| ENSP00000303777 | 2.19E-06 | -0.1976 | 0 | 0.098 |
| ENSP00000429367 | 3.99E-06 | -0.1976 | 0 | 0.102 |
| ENSP00000377148 | 1.81E-05 | -0.1976 | 634 | 0.188 |
| ENSP00000254072 | 1.27E-06 | -0.1976 | 0 | 0.170 |
| ENSP00000350869 | 3.63E-06 | -0.1976 | 0 | 0.253 |
| ENSP00000362560 | 2.49E-06 | -0.1977 | 0 | 0.190 |
| ENSP00000354218 | 4.73E-06 | -0.1978 | 0 | 0.637 |
| ENSP00000386487 | 3.27E-06 | -0.1978 | 0 | 0.089 |
| ENSP00000418379 | 1.60E-05 | -0.1978 | 429 | 0.000 |
| ENSP00000369500 | 2.32E-06 | -0.1978 | 0 | 0.107 |
| ENSP00000358747 | 1.20E-05 | -0.1978 | 467 | 0.392 |
| ENSP00000318770 | 1.30E-05 | -0.1979 | 0 | 0.201 |
| ENSP00000316457 | 2.43E-06 | -0.1979 | 0 | 0.000 |
| ENSP00000182290 | 1.36E-05 | -0.1980 | 0 | 0.125 |
| ENSP00000394049 | 2.57E-06 | -0.1980 | 0 | 0.283 |
| ENSP00000386218 | 2.46E-06 | -0.1981 | 0 | 0.000 |

| | | | | |
|---|---|---|---|---|
| ENSP00000271357 | 9.55E-06 | -0.1982 | 171 | 0.319 |
| ENSP00000346576 | 4.53E-06 | -0.1982 | 0 | 0.000 |
| ENSP00000346022 | 5.36E-06 | -0.1982 | 410 | 0.799 |
| ENSP00000429168 | 2.61E-06 | -0.1982 | 0 | 0.106 |
| ENSP00000393154 | 5.08E-06 | -0.1982 | 0 | 0.700 |
| ENSP00000392353 | 3.23E-06 | -0.1983 | 0 | 0.113 |
| ENSP00000455681 | 1.71E-06 | -0.1983 | 0 | 0.000 |
| ENSP00000357583 | 3.05E-06 | -0.1983 | 0 | 0.150 |
| ENSP00000377055 | 1.54E-05 | -0.1983 | 501 | 0.229 |
| ENSP00000375475 | 6.83E-07 | -0.1984 | 0 | 0.146 |
| ENSP00000357297 | 6.79E-06 | -0.1984 | 575 | 0.280 |
| ENSP00000355493 | 5.80E-06 | -0.1984 | 0 | 0.519 |
| ENSP00000403302 | 6.54E-06 | -0.1985 | 202 | 0.619 |
| ENSP00000385025 | 1.89E-06 | -0.1985 | 0 | 0.122 |
| ENSP00000369318 | 2.23E-05 | -0.1985 | 220 | 0.565 |
| ENSP00000389160 | 1.52E-06 | -0.1985 | 0 | 0.162 |
| ENSP00000356001 | 5.83E-06 | -0.1985 | 0 | 0.423 |
| ENSP00000379678 | 7.58E-06 | -0.1986 | 153 | 0.278 |
| ENSP00000334287 | 1.91E-06 | -0.1986 | 0 | 0.251 |
| ENSP00000371587 | 1.73E-05 | -0.1986 | 370 | 0.422 |
| ENSP00000352265 | 7.51E-06 | -0.1987 | 0 | 0.083 |
| ENSP00000355492 | 1.96E-06 | -0.1987 | 0 | 0.068 |
| ENSP00000355228 | 5.15E-06 | -0.1987 | 0 | 0.593 |
| ENSP00000349313 | 4.50E-06 | -0.1987 | 0 | 0.562 |
| ENSP00000363794 | 4.47E-06 | -0.1987 | 0 | 0.190 |
| ENSP00000454926 | 3.89E-06 | -0.1988 | 0 | 0.135 |
| ENSP00000355731 | 1.15E-05 | -0.1988 | 315 | 0.652 |
| ENSP00000350353 | 7.87E-06 | -0.1988 | 415 | 0.122 |
| ENSP00000371994 | 2.74E-06 | -0.1988 | 0 | 0.343 |
| ENSP00000363614 | 6.58E-06 | -0.1989 | 235 | 0.457 |
| ENSP00000350352 | 3.21E-06 | -0.1989 | 0 | 0.820 |
| ENSP00000362555 | 3.99E-06 | -0.1989 | 0 | 0.089 |
| ENSP00000369855 | 1.35E-05 | -0.1990 | 307 | 0.677 |
| ENSP00000445768 | 2.48E-06 | -0.1990 | 0 | 0.098 |
| ENSP00000355121 | 6.09E-06 | -0.1990 | 0 | 0.130 |
| ENSP00000360718 | 6.98E-06 | -0.1990 | 0 | 0.199 |
| ENSP00000377075 | 1.51E-06 | -0.1990 | 0 | 0.352 |
| ENSP00000361640 | 4.55E-06 | -0.1990 | 0 | 0.223 |
| ENSP00000351345 | 1.94E-06 | -0.1991 | 0 | 0.106 |
| ENSP00000370949 | 2.98E-06 | -0.1991 | 0 | 0.163 |
| ENSP00000359396 | 2.56E-06 | -0.1991 | 0 | 0.290 |
| ENSP00000326391 | 7.90E-06 | -0.1992 | 207 | 0.833 |
| ENSP00000252675 | 1.59E-05 | -0.1992 | 360 | 0.065 |
| ENSP00000361518 | 5.27E-06 | -0.1993 | 0 | 0.180 |
| ENSP00000416033 | 2.31E-06 | -0.1993 | 0 | 0.092 |
| ENSP00000283921 | 1.62E-05 | -0.1993 | 204 | 0.871 |

| | | | | |
|---|---|---|---|---|
| ENSP00000384192 | 4.33E-06 | -0.1994 | 0 | 0.352 |
| ENSP00000311343 | 1.01E-05 | -0.1994 | 0 | 0.144 |
| ENSP00000392270 | 2.81E-06 | -0.1994 | 0 | 0.571 |
| ENSP00000453399 | 2.89E-06 | -0.1994 | 0 | 0.116 |
| ENSP00000373244 | 1.14E-05 | -0.1994 | 301 | 0.407 |
| ENSP00000359297 | 6.24E-06 | -0.1995 | 198 | 0.278 |
| ENSP00000355046 | 1.23E-05 | -0.1995 | 214 | 0.538 |
| ENSP00000424870 | 4.69E-06 | -0.1995 | 158 | 0.663 |
| ENSP00000377958 | 2.05E-05 | -0.1997 | 785 | 0.742 |
| ENSP00000372437 | 6.55E-06 | -0.1997 | 0 | 0.000 |
| ENSP00000401566 | 4.30E-06 | -0.1997 | 0 | 0.666 |
| ENSP00000363763 | 2.10E-05 | -0.1997 | 632 | 0.670 |
| ENSP00000291481 | 2.98E-06 | -0.1997 | 0 | 0.158 |
| ENSP00000413064 | 2.97E-06 | -0.1997 | 0 | 0.072 |
| ENSP00000394494 | 2.71E-06 | -0.1997 | 0 | 0.220 |
| ENSP00000371553 | 4.84E-06 | -0.1997 | 0 | 0.452 |
| ENSP00000339801 | 1.27E-05 | -0.1997 | 214 | 0.117 |
| ENSP00000366179 | 7.36E-06 | -0.1997 | 990 | 0.244 |
| ENSP00000360613 | 1.73E-05 | -0.1998 | 223 | 0.869 |
| ENSP00000454454 | 5.07E-06 | -0.1998 | 182 | 0.000 |
| ENSP00000271835 | 1.34E-05 | -0.1998 | 0 | 0.149 |
| ENSP00000359557 | 4.60E-06 | -0.1998 | 0 | 0.259 |
| ENSP00000373565 | 7.04E-06 | -0.1998 | 0 | 0.146 |
| ENSP00000365687 | 1.06E-05 | -0.1998 | 156 | 0.841 |
| ENSP00000381377 | 1.73E-06 | -0.1999 | 0 | 0.129 |
| ENSP00000428343 | 9.84E-06 | -0.1999 | 188 | 0.368 |
| ENSP00000361524 | 3.11E-06 | -0.2000 | 0 | 0.135 |
| ENSP00000362727 | 9.10E-06 | -0.2000 | 256 | 0.074 |
| ENSP00000352425 | 1.12E-05 | -0.2000 | 208 | 0.630 |
| ENSP00000357511 | 6.06E-06 | -0.2000 | 0 | 0.184 |
| ENSP00000359782 | 4.64E-06 | -0.2000 | 0 | 0.169 |
| ENSP00000274181 | 1.68E-05 | -0.2000 | 167 | 0.127 |
| ENSP00000347538 | 4.13E-06 | -0.2000 | 0 | 0.107 |
| ENSP00000402084 | 5.13E-06 | -0.2001 | 0 | 0.708 |
| ENSP00000381167 | 7.99E-06 | -0.2001 | 235 | 0.152 |
| ENSP00000384053 | 1.27E-05 | -0.2001 | 215 | 0.691 |
| ENSP00000369440 | 8.59E-06 | -0.2001 | 0 | 0.119 |
| ENSP00000265404 | 6.21E-06 | -0.2001 | 0 | 0.211 |
| ENSP00000285871 | 1.24E-05 | -0.2001 | 0 | 0.153 |
| ENSP00000358059 | 3.99E-06 | -0.2002 | 0 | 0.141 |
| ENSP00000349768 | 1.47E-05 | -0.2002 | 173 | 0.682 |
| ENSP00000315137 | 5.35E-06 | -0.2002 | 0 | 0.080 |
| ENSP00000267484 | 4.31E-06 | -0.2003 | 0 | 0.103 |
| ENSP00000457258 | 2.89E-06 | -0.2003 | 0 | 0.597 |
| ENSP00000356156 | 5.92E-06 | -0.2003 | 183 | 0.229 |
| ENSP00000457656 | 4.85E-06 | -0.2003 | 193 | 0.000 |

| | | | | |
|---|---|---|---|---|
| ENSP00000433415 | 2.08E-06 | -0.2003 | 0 | 0.183 |
| ENSP00000364643 | 3.33E-06 | -0.2003 | 0 | 0.092 |
| ENSP00000255695 | 1.26E-05 | -0.2004 | 0 | 0.089 |
| ENSP00000358784 | 4.79E-06 | -0.2004 | 0 | 0.378 |
| ENSP00000350686 | 2.15E-06 | -0.2004 | 0 | 0.069 |
| ENSP00000221797 | 1.54E-05 | -0.2004 | 0 | 0.110 |
| ENSP00000346478 | 9.12E-06 | -0.2004 | 0 | 0.181 |
| ENSP00000372112 | 3.14E-06 | -0.2004 | 0 | 0.106 |
| ENSP00000417914 | 8.56E-06 | -0.2005 | 202 | 0.667 |
| ENSP00000311695 | 4.08E-06 | -0.2005 | 0 | 0.274 |
| ENSP00000428220 | 1.62E-05 | -0.2007 | 175 | 0.566 |
| ENSP00000395995 | 3.21E-06 | -0.2007 | 0 | 0.135 |
| ENSP00000362748 | 1.04E-05 | -0.2007 | 150 | 0.263 |
| ENSP00000376410 | 3.15E-06 | -0.2007 | 0 | 0.286 |
| ENSP00000371693 | 1.20E-05 | -0.2008 | 290 | 0.771 |
| ENSP00000360191 | 3.11E-06 | -0.2008 | 0 | 0.122 |
| ENSP00000351632 | 3.41E-06 | -0.2009 | 0 | 0.100 |
| ENSP00000382670 | 5.10E-06 | -0.2009 | 0 | 0.101 |
| ENSP00000412361 | 3.56E-06 | -0.2010 | 0 | 0.122 |
| ENSP00000368124 | 3.35E-06 | -0.2010 | 0 | 0.000 |
| ENSP00000360316 | 1.97E-05 | -0.2010 | 349 | 0.398 |
| ENSP00000332163 | 1.03E-05 | -0.2010 | 0 | 0.121 |
| ENSP00000405252 | 3.95E-06 | -0.2010 | 0 | 0.182 |
| ENSP00000329565 | 3.61E-06 | -0.2012 | 0 | 0.150 |
| ENSP00000368144 | 1.72E-06 | -0.2012 | 0 | 0.093 |
| ENSP00000354575 | 7.28E-06 | -0.2012 | 159 | 0.108 |
| ENSP00000408405 | 2.47E-06 | -0.2013 | 0 | 0.090 |
| ENSP00000281772 | 1.13E-05 | -0.2013 | 0 | 0.141 |
| ENSP00000337450 | 4.50E-06 | -0.2013 | 0 | 0.294 |
| ENSP00000356465 | 6.40E-06 | -0.2013 | 0 | 0.150 |
| ENSP00000380785 | 3.89E-06 | -0.2013 | 0 | 0.106 |
| ENSP00000344668 | 1.59E-05 | -0.2014 | 175 | 0.584 |
| ENSP00000265801 | 1.49E-05 | -0.2014 | 242 | 0.368 |
| ENSP00000298298 | 7.45E-06 | -0.2014 | 0 | 0.000 |
| ENSP00000430025 | 2.88E-06 | -0.2015 | 0 | 0.148 |
| ENSP00000358762 | 7.74E-06 | -0.2015 | 0 | 0.118 |
| ENSP00000323421 | 3.56E-06 | -0.2015 | 0 | 0.767 |
| ENSP00000361569 | 3.86E-06 | -0.2016 | 0 | 0.109 |
| ENSP00000362387 | 3.60E-06 | -0.2017 | 0 | 0.119 |
| ENSP00000373370 | 3.65E-06 | -0.2018 | 0 | 0.113 |
| ENSP00000328410 | 6.44E-06 | -0.2018 | 0 | 0.127 |
| ENSP00000364324 | 2.31E-06 | -0.2018 | 0 | 0.150 |
| ENSP00000055682 | 6.79E-06 | -0.2018 | 0 | 0.196 |
| ENSP00000452252 | 9.09E-07 | -0.2018 | 0 | 0.200 |
| ENSP00000458062 | 3.58E-06 | -0.2019 | 0 | 0.000 |
| ENSP00000352639 | 1.50E-05 | -0.2019 | 150 | 0.120 |

| | | | | |
|---|---|---|---|---|
| ENSP00000356429 | 1.66E-05 | -0.2019 | 242 | 0.721 |
| ENSP00000357196 | 4.37E-06 | -0.2020 | 0 | 0.711 |
| ENSP00000312672 | 1.03E-05 | -0.2020 | 0 | 0.112 |
| ENSP00000288022 | 1.51E-05 | -0.2020 | 361 | 0.605 |
| ENSP00000431482 | 3.37E-06 | -0.2021 | 0 | 0.436 |
| ENSP00000323191 | 3.38E-06 | -0.2022 | 0 | 0.150 |
| ENSP00000354848 | 4.60E-06 | -0.2022 | 0 | 0.000 |
| ENSP00000473005 | 1.35E-06 | -0.2023 | 0 | 0.000 |
| ENSP00000351618 | 5.10E-06 | -0.2024 | 0 | 0.224 |
| ENSP00000429301 | 3.60E-06 | -0.2024 | 0 | 0.144 |
| ENSP00000431245 | 4.94E-06 | -0.2024 | 0 | 0.458 |
| ENSP00000436557 | 1.11E-06 | -0.2024 | 0 | 0.123 |
| ENSP00000452762 | 1.43E-05 | -0.2024 | 277 | 0.389 |
| ENSP00000385527 | 3.01E-06 | -0.2024 | 0 | 0.073 |
| ENSP00000360244 | 2.01E-06 | -0.2024 | 0 | 0.132 |
| ENSP00000297290 | 6.16E-06 | -0.2025 | 0 | 0.140 |
| ENSP00000427336 | 4.32E-06 | -0.2025 | 0 | 0.272 |
| ENSP00000422338 | 1.99E-06 | -0.2025 | 0 | 0.149 |
| ENSP00000377566 | 5.28E-06 | -0.2026 | 0 | 0.190 |
| ENSP00000356182 | 3.56E-06 | -0.2026 | 0 | 0.000 |
| ENSP00000419975 | 3.47E-06 | -0.2026 | 0 | 0.151 |
| ENSP00000340703 | 6.25E-06 | -0.2027 | 0 | 0.000 |
| ENSP00000332340 | 1.40E-05 | -0.2029 | 213 | 0.890 |
| ENSP00000417401 | 2.12E-05 | -0.2029 | 900 | 0.748 |
| ENSP00000299866 | 4.92E-06 | -0.2030 | 0 | 0.088 |
| ENSP00000346080 | 5.44E-06 | -0.2030 | 156 | 0.766 |
| ENSP00000451030 | 1.28E-05 | -0.2030 | 188 | 0.562 |
| ENSP00000318119 | 4.08E-06 | -0.2030 | 0 | 0.000 |
| ENSP00000443191 | 2.23E-06 | -0.2030 | 0 | 0.148 |
| ENSP00000448888 | 2.18E-06 | -0.2030 | 0 | 0.000 |
| ENSP00000402537 | 4.36E-06 | -0.2030 | 0 | 0.131 |
| ENSP00000423159 | 1.02E-06 | -0.2031 | 0 | 0.160 |
| ENSP00000398454 | 1.02E-06 | -0.2031 | 0 | 0.160 |
| ENSP00000362386 | 7.01E-06 | -0.2031 | 316 | 0.702 |
| ENSP00000436901 | 4.83E-06 | -0.2031 | 0 | 0.277 |
| ENSP00000335062 | 1.67E-05 | -0.2031 | 163 | 0.796 |
| ENSP00000349396 | 5.26E-06 | -0.2032 | 0 | 0.179 |
| ENSP00000450731 | 9.56E-06 | -0.2032 | 187 | 0.222 |
| ENSP00000346240 | 1.10E-05 | -0.2032 | 377 | 0.139 |
| ENSP00000305533 | 8.04E-06 | -0.2032 | 0 | 0.165 |
| ENSP00000266560 | 1.28E-05 | -0.2033 | 0 | 0.293 |
| ENSP00000358211 | 1.18E-05 | -0.2033 | 195 | 0.767 |
| ENSP00000451484 | 2.01E-06 | -0.2033 | 0 | 0.000 |
| ENSP00000437464 | 4.20E-06 | -0.2033 | 0 | 0.000 |
| ENSP00000359489 | 6.10E-06 | -0.2033 | 0 | 0.144 |
| ENSP00000387091 | 1.04E-05 | -0.2034 | 198 | 0.253 |

| | | | | |
|---|---|---|---|---|
| ENSP00000377061 | 5.78E-06 | -0.2034 | 0 | 0.609 |
| ENSP00000328511 | 8.63E-06 | -0.2034 | 224 | 0.327 |
| ENSP00000362273 | 3.85E-06 | -0.2034 | 0 | 0.178 |
| ENSP00000379156 | 3.66E-06 | -0.2035 | 0 | 0.165 |
| ENSP00000410852 | 3.92E-06 | -0.2035 | 0 | 0.128 |
| ENSP00000359370 | 8.17E-06 | -0.2035 | 152 | 0.104 |
| ENSP00000379566 | 2.72E-06 | -0.2035 | 0 | 0.154 |
| ENSP00000369518 | 5.56E-06 | -0.2036 | 0 | 0.170 |
| ENSP00000357251 | 4.81E-06 | -0.2036 | 0 | 0.000 |
| ENSP00000349364 | 6.18E-06 | -0.2036 | 0 | 0.123 |
| ENSP00000349359 | 4.03E-06 | -0.2036 | 0 | 0.152 |
| ENSP00000341045 | 3.34E-06 | -0.2036 | 0 | 0.113 |
| ENSP00000369014 | 1.05E-06 | -0.2037 | 0 | 0.155 |
| ENSP00000381631 | 2.72E-06 | -0.2037 | 0 | 0.198 |
| ENSP00000247706 | 1.85E-05 | -0.2037 | 0 | 0.161 |
| ENSP00000355593 | 1.07E-05 | -0.2037 | 306 | 0.372 |
| ENSP00000365840 | 3.39E-06 | -0.2037 | 0 | 0.117 |
| ENSP00000298699 | 7.26E-06 | -0.2038 | 0 | 0.160 |
| ENSP00000384416 | 2.86E-06 | -0.2038 | 0 | 0.144 |
| ENSP00000358312 | 6.49E-06 | -0.2038 | 0 | 0.119 |
| ENSP00000366826 | 7.02E-06 | -0.2038 | 202 | 0.648 |
| ENSP00000434593 | 1.09E-06 | -0.2039 | 0 | 0.170 |
| ENSP00000368920 | 1.10E-06 | -0.2039 | 0 | 0.175 |
| ENSP00000355800 | 3.13E-06 | -0.2039 | 0 | 0.087 |
| ENSP00000315357 | 2.45E-06 | -0.2040 | 0 | 0.198 |
| ENSP00000425556 | 5.65E-06 | -0.2040 | 0 | 0.235 |
| ENSP00000384551 | 3.46E-06 | -0.2041 | 0 | 0.083 |
| ENSP00000362656 | 5.23E-06 | -0.2041 | 235 | 0.151 |
| ENSP00000354001 | 8.57E-07 | -0.2042 | 0 | 0.158 |
| ENSP00000447537 | 3.98E-06 | -0.2042 | 0 | 0.085 |
| ENSP00000434995 | 5.12E-06 | -0.2042 | 284 | 0.732 |
| ENSP00000435509 | 5.70E-06 | -0.2043 | 205 | 0.793 |
| ENSP00000438685 | 2.61E-06 | -0.2043 | 0 | 0.000 |
| ENSP00000395574 | 3.24E-06 | -0.2043 | 0 | 0.521 |
| ENSP00000367454 | 6.17E-06 | -0.2043 | 0 | 0.279 |
| ENSP00000370781 | 6.13E-06 | -0.2043 | 0 | 0.474 |
| ENSP00000343890 | 5.53E-06 | -0.2043 | 0 | 0.000 |
| ENSP00000424048 | 2.88E-06 | -0.2043 | 0 | 0.000 |
| ENSP00000333680 | 7.83E-06 | -0.2044 | 0 | 0.251 |
| ENSP00000383365 | 9.24E-06 | -0.2044 | 275 | 0.648 |
| ENSP00000045083 | 6.46E-06 | -0.2044 | 0 | 0.097 |
| ENSP00000379571 | 8.95E-06 | -0.2044 | 191 | 0.111 |
| ENSP00000382271 | 5.14E-06 | -0.2045 | 0 | 0.099 |
| ENSP00000403984 | 2.01E-06 | -0.2046 | 0 | 0.127 |
| ENSP00000345156 | 6.09E-06 | -0.2046 | 0 | 0.825 |
| ENSP00000351075 | 9.68E-06 | -0.2047 | 0 | 0.100 |

| | | | | |
|---|---|---|---|---|
| ENSP00000443988 | 3.77E-06 | -0.2047 | 0 | 0.850 |
| ENSP00000468098 | 1.31E-06 | -0.2047 | 0 | 0.000 |
| ENSP00000418447 | 2.84E-05 | -0.2047 | 629 | 0.000 |
| ENSP00000349305 | 5.26E-06 | -0.2048 | 214 | 0.273 |
| ENSP00000263266 | 9.82E-06 | -0.2048 | 0 | 0.095 |
| ENSP00000446205 | 3.62E-06 | -0.2048 | 0 | 0.702 |
| ENSP00000418259 | 4.51E-06 | -0.2048 | 0 | 0.728 |
| ENSP00000357754 | 1.07E-05 | -0.2048 | 252 | 0.066 |
| ENSP00000324551 | 4.66E-06 | -0.2048 | 0 | 0.113 |
| ENSP00000349560 | 9.43E-06 | -0.2049 | 150 | 0.148 |
| ENSP00000364320 | 1.32E-05 | -0.2049 | 400 | 0.911 |
| ENSP00000308024 | 1.93E-05 | -0.2049 | 467 | 0.637 |
| ENSP00000344563 | 4.69E-06 | -0.2050 | 0 | 0.137 |
| ENSP00000436926 | 4.84E-06 | -0.2050 | 0 | 0.506 |
| ENSP00000365401 | 1.91E-06 | -0.2050 | 0 | 0.103 |
| ENSP00000423422 | 1.06E-06 | -0.2050 | 0 | 0.000 |
| ENSP00000427130 | 2.47E-06 | -0.2051 | 0 | 0.135 |
| ENSP00000293261 | 5.85E-06 | -0.2051 | 0 | 0.122 |
| ENSP00000357341 | 4.34E-06 | -0.2051 | 0 | 0.369 |
| ENSP00000361206 | 5.68E-06 | -0.2051 | 302 | 0.634 |
| ENSP00000328347 | 4.93E-06 | -0.2051 | 0 | 0.117 |
| ENSP00000362085 | 3.16E-06 | -0.2051 | 0 | 0.161 |
| ENSP00000357178 | 1.84E-05 | -0.2051 | 259 | 0.690 |
| ENSP00000427407 | 1.06E-06 | -0.2051 | 0 | 0.086 |
| ENSP00000356951 | 6.04E-06 | -0.2052 | 0 | 0.000 |
| ENSP00000323096 | 6.37E-06 | -0.2052 | 0 | 0.148 |
| ENSP00000414721 | 3.20E-06 | -0.2052 | 0 | 0.105 |
| ENSP00000381064 | 4.75E-06 | -0.2052 | 0 | 0.158 |
| ENSP00000402020 | 6.78E-06 | -0.2053 | 0 | 0.102 |
| ENSP00000357158 | 5.58E-06 | -0.2054 | 0 | 0.114 |
| ENSP00000434508 | 6.11E-06 | -0.2054 | 0 | 0.000 |
| ENSP00000377944 | 4.34E-06 | -0.2055 | 0 | 0.874 |
| ENSP00000373698 | 2.27E-06 | -0.2056 | 0 | 0.000 |
| ENSP00000356214 | 1.85E-06 | -0.2056 | 0 | 0.144 |
| ENSP00000381982 | 8.30E-06 | -0.2057 | 0 | 0.125 |
| ENSP00000304822 | 1.39E-05 | -0.2057 | 295 | 0.454 |
| ENSP00000362413 | 1.63E-05 | -0.2057 | 417 | 0.829 |
| ENSP00000378782 | 3.40E-06 | -0.2057 | 0 | 0.307 |
| ENSP00000457544 | 1.09E-06 | -0.2058 | 0 | 0.000 |
| ENSP00000363189 | 6.05E-06 | -0.2058 | 0 | 0.099 |
| ENSP00000367882 | 4.16E-06 | -0.2058 | 0 | 0.117 |
| ENSP00000429065 | 3.44E-06 | -0.2058 | 0 | 0.389 |
| ENSP00000356130 | 4.96E-06 | -0.2059 | 205 | 0.687 |
| ENSP00000352839 | 1.87E-05 | -0.2059 | 263 | 0.661 |
| ENSP00000398105 | 5.13E-06 | -0.2059 | 214 | 0.058 |
| ENSP00000419378 | 4.58E-06 | -0.2059 | 0 | 0.069 |

| | | | | |
|---|---|---|---|---|
| ENSP00000246646 | 3.98E-06 | -0.2059 | 0 | 0.126 |
| ENSP00000415178 | 4.95E-06 | -0.2059 | 0 | 0.000 |
| ENSP00000421566 | 1.27E-05 | -0.2059 | 734 | 0.671 |
| ENSP00000361929 | 1.00E-05 | -0.2060 | 0 | 0.093 |
| ENSP00000403438 | 2.89E-06 | -0.2060 | 0 | 0.131 |
| ENSP00000472929 | 1.96E-05 | -0.2060 | 207 | 0.898 |
| ENSP00000328923 | 7.61E-06 | -0.2060 | 201 | 0.531 |
| ENSP00000360689 | 9.22E-06 | -0.2060 | 201 | 0.696 |
| ENSP00000471312 | 1.03E-06 | -0.2060 | 0 | 0.000 |
| ENSP00000359417 | 6.59E-06 | -0.2061 | 163 | 0.225 |
| ENSP00000357900 | 4.37E-06 | -0.2061 | 0 | 0.106 |
| ENSP00000399547 | 7.72E-06 | -0.2061 | 202 | 0.667 |
| ENSP00000424176 | 1.20E-05 | -0.2062 | 252 | 0.084 |
| ENSP00000354730 | 1.63E-06 | -0.2062 | 0 | 0.174 |
| ENSP00000352706 | 3.62E-06 | -0.2062 | 0 | 0.336 |
| ENSP00000324672 | 9.81E-06 | -0.2062 | 0 | 0.084 |
| ENSP00000460441 | 5.71E-06 | -0.2063 | 0 | 0.000 |
| ENSP00000417085 | 1.29E-05 | -0.2063 | 464 | 0.093 |
| ENSP00000344749 | 5.64E-06 | -0.2063 | 0 | 0.000 |
| ENSP00000326500 | 9.11E-06 | -0.2063 | 0 | 0.132 |
| ENSP00000348577 | 4.63E-06 | -0.2064 | 0 | 0.766 |
| ENSP00000273905 | 1.49E-05 | -0.2064 | 153 | 0.062 |
| ENSP00000349016 | 3.43E-06 | -0.2064 | 0 | 0.214 |
| ENSP00000356563 | 2.87E-06 | -0.2064 | 0 | 0.054 |
| ENSP00000415203 | 4.07E-06 | -0.2064 | 0 | 0.178 |
| ENSP00000385021 | 1.35E-05 | -0.2064 | 157 | 0.549 |
| ENSP00000359384 | 5.16E-06 | -0.2064 | 0 | 0.092 |
| ENSP00000386175 | 2.82E-06 | -0.2065 | 0 | 0.110 |
| ENSP00000347504 | 5.16E-06 | -0.2065 | 0 | 0.344 |
| ENSP00000311657 | 8.46E-06 | -0.2065 | 0 | 0.108 |
| ENSP00000375053 | 1.27E-06 | -0.2065 | 0 | 0.124 |
| ENSP00000460871 | 1.67E-05 | -0.2065 | 287 | 0.904 |
| ENSP00000328150 | 3.80E-06 | -0.2065 | 0 | 0.094 |
| ENSP00000370262 | 1.36E-05 | -0.2066 | 803 | 0.099 |
| ENSP00000264771 | 6.07E-06 | -0.2067 | 0 | 0.158 |
| ENSP00000456881 | 3.60E-06 | -0.2067 | 0 | 0.186 |
| ENSP00000360296 | 6.11E-06 | -0.2067 | 0 | 0.000 |
| ENSP00000266546 | 5.91E-06 | -0.2067 | 0 | 0.154 |
| ENSP00000335575 | 5.70E-06 | -0.2068 | 213 | 0.000 |
| ENSP00000387124 | 1.07E-06 | -0.2068 | 0 | 0.163 |
| ENSP00000398106 | 1.60E-06 | -0.2068 | 0 | 0.121 |
| ENSP00000366030 | 2.69E-06 | -0.2068 | 0 | 0.000 |
| ENSP00000361298 | 7.01E-06 | -0.2068 | 200 | 0.280 |
| ENSP00000381553 | 3.62E-06 | -0.2069 | 0 | 0.000 |
| ENSP00000301698 | 5.21E-06 | -0.2069 | 0 | 0.165 |
| ENSP00000223208 | 5.85E-06 | -0.2069 | 0 | 0.102 |

| | | | | |
|---|---|---|---|---|
| ENSP00000411084 | 3.67E-06 | -0.2070 | 0 | 0.684 |
| ENSP00000354580 | 3.55E-06 | -0.2070 | 0 | 0.458 |
| ENSP00000361628 | 1.96E-06 | -0.2071 | 0 | 0.140 |
| ENSP00000376827 | 8.56E-06 | -0.2071 | 150 | 0.301 |
| ENSP00000369810 | 2.97E-06 | -0.2072 | 0 | 0.172 |
| ENSP00000357048 | 1.04E-05 | -0.2072 | 156 | 0.245 |
| ENSP00000424757 | 3.51E-06 | -0.2073 | 0 | 0.104 |
| ENSP00000358358 | 4.88E-06 | -0.2074 | 0 | 0.114 |
| ENSP00000468267 | 7.58E-06 | -0.2074 | 202 | 0.000 |
| ENSP00000409075 | 4.19E-06 | -0.2075 | 193 | 0.000 |
| ENSP00000324701 | 7.92E-06 | -0.2075 | 0 | 0.385 |
| ENSP00000362948 | 4.79E-06 | -0.2075 | 0 | 0.644 |
| ENSP00000422431 | 4.64E-06 | -0.2075 | 0 | 0.093 |
| ENSP00000438455 | 1.23E-05 | -0.2076 | 0 | 0.391 |
| ENSP00000295908 | 4.05E-06 | -0.2076 | 0 | 0.526 |
| ENSP00000278505 | 4.34E-06 | -0.2076 | 0 | 0.130 |
| ENSP00000337240 | 6.05E-06 | -0.2076 | 0 | 0.200 |
| ENSP00000359891 | 3.31E-06 | -0.2077 | 0 | 0.115 |
| ENSP00000264735 | 5.24E-06 | -0.2077 | 0 | 0.111 |
| ENSP00000369802 | 2.57E-06 | -0.2077 | 0 | 0.000 |
| ENSP00000306900 | 7.55E-06 | -0.2077 | 0 | 0.102 |
| ENSP00000358203 | 3.24E-06 | -0.2077 | 0 | 0.000 |
| ENSP00000375236 | 1.18E-06 | -0.2077 | 0 | 0.156 |
| ENSP00000350102 | 5.01E-06 | -0.2078 | 0 | 0.143 |
| ENSP00000413234 | 1.20E-05 | -0.2078 | 154 | 0.239 |
| ENSP00000362221 | 5.53E-06 | -0.2078 | 205 | 0.679 |
| ENSP00000367564 | 4.48E-06 | -0.2078 | 0 | 0.114 |
| ENSP00000354168 | 1.22E-05 | -0.2078 | 205 | 0.000 |
| ENSP00000347038 | 1.14E-05 | -0.2078 | 0 | 0.087 |
| ENSP00000346768 | 5.88E-06 | -0.2079 | 0 | 0.165 |
| ENSP00000386226 | 6.73E-06 | -0.2079 | 153 | 0.971 |
| ENSP00000384369 | 4.41E-06 | -0.2079 | 311 | 0.581 |
| ENSP00000381589 | 2.42E-06 | -0.2079 | 0 | 0.150 |
| ENSP00000365530 | 2.60E-06 | -0.2080 | 0 | 0.168 |
| ENSP00000354571 | 5.09E-06 | -0.2080 | 0 | 0.107 |
| ENSP00000361052 | 5.46E-06 | -0.2080 | 0 | 0.161 |
| ENSP00000401437 | 2.11E-05 | -0.2081 | 387 | 0.646 |
| ENSP00000398410 | 7.21E-06 | -0.2082 | 0 | 0.421 |
| ENSP00000466121 | 2.44E-06 | -0.2082 | 0 | 0.000 |
| ENSP00000387841 | 4.52E-06 | -0.2082 | 307 | 0.000 |
| ENSP00000300659 | 1.79E-05 | -0.2082 | 0 | 0.726 |
| ENSP00000330596 | 2.32E-06 | -0.2082 | 0 | 0.130 |
| ENSP00000456594 | 5.39E-07 | -0.2083 | 0 | 0.162 |
| ENSP00000454746 | 3.10E-06 | -0.2083 | 0 | 0.000 |
| ENSP00000407401 | 8.32E-06 | -0.2083 | 163 | 0.242 |
| ENSP00000342278 | 1.95E-05 | -0.2083 | 903 | 0.655 |

| | | | | |
|---|---|---|---|---|
| ENSP00000362718 | 5.55E-06 | -0.2084 | 0 | 0.147 |
| ENSP00000401328 | 3.87E-06 | -0.2084 | 0 | 0.133 |
| ENSP00000380256 | 6.28E-06 | -0.2085 | 201 | 0.686 |
| ENSP00000302978 | 1.48E-05 | -0.2085 | 727 | 0.382 |
| ENSP00000360147 | 1.26E-05 | -0.2085 | 761 | 0.621 |
| ENSP00000347754 | 1.29E-05 | -0.2085 | 591 | 0.571 |
| ENSP00000297623 | 1.13E-05 | -0.2085 | 0 | 0.118 |
| ENSP00000379891 | 5.75E-06 | -0.2086 | 0 | 0.260 |
| ENSP00000365837 | 4.55E-06 | -0.2086 | 317 | 0.778 |
| ENSP00000363250 | 3.44E-06 | -0.2086 | 0 | 0.082 |
| ENSP00000380354 | 1.24E-06 | -0.2086 | 0 | 0.124 |
| ENSP00000401878 | 1.94E-06 | -0.2086 | 0 | 0.130 |
| ENSP00000365426 | 8.58E-06 | -0.2086 | 0 | 0.199 |
| ENSP00000354227 | 1.33E-06 | -0.2087 | 0 | 0.160 |
| ENSP00000383394 | 4.56E-06 | -0.2087 | 0 | 0.097 |
| ENSP00000280245 | 1.64E-05 | -0.2087 | 0 | 0.121 |
| ENSP00000307129 | 6.06E-06 | -0.2087 | 0 | 0.127 |
| ENSP00000361151 | 1.17E-05 | -0.2088 | 284 | 0.302 |
| ENSP00000428845 | 8.03E-06 | -0.2088 | 161 | 0.388 |
| ENSP00000450730 | 4.59E-06 | -0.2088 | 205 | 0.000 |
| ENSP00000367923 | 5.43E-06 | -0.2089 | 280 | 0.621 |
| ENSP00000387187 | 2.56E-06 | -0.2089 | 0 | 0.194 |
| ENSP00000429601 | 1.76E-06 | -0.2089 | 0 | 0.235 |
| ENSP00000306564 | 4.23E-06 | -0.2089 | 0 | 0.135 |
| ENSP00000297001 | 7.49E-06 | -0.2089 | 0 | 0.122 |
| ENSP00000359532 | 2.18E-05 | -0.2089 | 398 | 0.653 |
| ENSP00000420716 | 2.64E-06 | -0.2089 | 0 | 0.155 |
| ENSP00000362249 | 1.44E-05 | -0.2089 | 216 | 0.524 |
| ENSP00000364777 | 3.73E-06 | -0.2090 | 0 | 0.399 |
| ENSP00000406909 | 3.19E-06 | -0.2090 | 0 | 0.090 |
| ENSP00000239906 | 1.20E-05 | -0.2090 | 0 | 0.200 |
| ENSP00000259371 | 4.85E-06 | -0.2091 | 0 | 0.606 |
| ENSP00000370671 | 3.56E-06 | -0.2091 | 0 | 0.107 |
| ENSP00000301190 | 5.28E-06 | -0.2091 | 0 | 0.146 |
| ENSP00000334540 | 1.11E-05 | -0.2092 | 662 | 0.153 |
| ENSP00000452885 | 4.32E-06 | -0.2092 | 173 | 0.132 |
| ENSP00000216071 | 1.45E-05 | -0.2093 | 0 | 0.192 |
| ENSP00000390478 | 8.22E-06 | -0.2093 | 0 | 0.000 |
| ENSP00000295588 | 4.44E-06 | -0.2093 | 0 | 0.066 |
| ENSP00000359211 | 1.91E-05 | -0.2093 | 618 | 0.673 |
| ENSP00000360488 | 2.77E-06 | -0.2093 | 0 | 0.509 |
| ENSP00000341282 | 3.69E-06 | -0.2093 | 0 | 0.173 |
| ENSP00000375857 | 4.06E-06 | -0.2093 | 0 | 0.254 |
| ENSP00000355877 | 5.35E-06 | -0.2093 | 0 | 0.102 |
| ENSP00000346196 | 4.50E-06 | -0.2094 | 0 | 0.200 |
| ENSP00000278222 | 1.85E-05 | -0.2094 | 0 | 0.171 |

| | | | | |
|---|---|---|---|---|
| ENSP00000366936 | 1.10E-05 | -0.2094 | 181 | 0.575 |
| ENSP00000307297 | 1.63E-05 | -0.2094 | 202 | 0.492 |
| ENSP00000354675 | 3.06E-06 | -0.2094 | 0 | 0.167 |
| ENSP00000394051 | 5.34E-06 | -0.2095 | 0 | 0.131 |
| ENSP00000444617 | 2.46E-06 | -0.2095 | 0 | 0.000 |
| ENSP00000419502 | 1.82E-06 | -0.2095 | 0 | 0.000 |
| ENSP00000356134 | 1.03E-05 | -0.2095 | 361 | 0.830 |
| ENSP00000338067 | 9.77E-06 | -0.2096 | 0 | 0.132 |
| ENSP00000360660 | 3.91E-06 | -0.2096 | 0 | 0.171 |
| ENSP00000363683 | 3.70E-06 | -0.2096 | 0 | 0.284 |
| ENSP00000378364 | 1.02E-05 | -0.2097 | 0 | 0.279 |
| ENSP00000384690 | 1.12E-05 | -0.2097 | 0 | 0.074 |
| ENSP00000420294 | 8.90E-06 | -0.2097 | 313 | 0.778 |
| ENSP00000363714 | 1.52E-06 | -0.2098 | 0 | 0.186 |
| ENSP00000356290 | 1.24E-05 | -0.2098 | 485 | 0.632 |
| ENSP00000363638 | 4.90E-06 | -0.2098 | 0 | 0.102 |
| ENSP00000451700 | 1.11E-05 | -0.2098 | 187 | 0.000 |
| ENSP00000451798 | 1.11E-05 | -0.2098 | 187 | 0.000 |
| ENSP00000366248 | 1.77E-05 | -0.2099 | 165 | 0.841 |
| ENSP00000406933 | 2.67E-06 | -0.2099 | 0 | 0.105 |
| ENSP00000363386 | 4.45E-06 | -0.2099 | 0 | 0.133 |
| ENSP00000265239 | 6.02E-06 | -0.2100 | 0 | 0.112 |
| ENSP00000361599 | 2.59E-06 | -0.2100 | 0 | 0.000 |
| ENSP00000269720 | 7.24E-06 | -0.2100 | 0 | 0.000 |
| ENSP00000317671 | 3.03E-06 | -0.2100 | 0 | 0.180 |
| ENSP00000420267 | 1.53E-05 | -0.2101 | 325 | 0.725 |
| ENSP00000329890 | 1.83E-05 | -0.2101 | 185 | 0.406 |
| ENSP00000454565 | 3.22E-06 | -0.2101 | 0 | 0.000 |
| ENSP00000355944 | 4.89E-06 | -0.2101 | 0 | 0.184 |
| ENSP00000386292 | 2.55E-06 | -0.2102 | 0 | 0.000 |
| ENSP00000348986 | 1.07E-05 | -0.2102 | 163 | 0.599 |
| ENSP00000385461 | 8.18E-06 | -0.2102 | 194 | 0.744 |
| ENSP00000393987 | 5.50E-06 | -0.2102 | 0 | 0.109 |
| ENSP00000391085 | 1.07E-06 | -0.2103 | 0 | 0.160 |
| ENSP00000361411 | 4.17E-06 | -0.2103 | 0 | 0.136 |
| ENSP00000327599 | 7.80E-06 | -0.2104 | 0 | 0.124 |
| ENSP00000347919 | 7.83E-06 | -0.2104 | 0 | 0.145 |
| ENSP00000427975 | 3.42E-06 | -0.2105 | 0 | 0.184 |
| ENSP00000256103 | 1.97E-05 | -0.2106 | 165 | 0.147 |
| ENSP00000375893 | 8.74E-06 | -0.2106 | 416 | 0.505 |
| ENSP00000415011 | 4.22E-06 | -0.2106 | 0 | 0.295 |
| ENSP00000452959 | 3.49E-06 | -0.2106 | 0 | 0.000 |
| ENSP00000310146 | 1.63E-05 | -0.2107 | 295 | 0.000 |
| ENSP00000363392 | 3.18E-06 | -0.2107 | 0 | 0.163 |
| ENSP00000352456 | 3.65E-06 | -0.2108 | 0 | 0.106 |
| ENSP00000353373 | 2.45E-06 | -0.2108 | 0 | 0.101 |

| | | | | |
|---|---|---|---|---|
| ENSP00000347443 | 3.09E-06 | -0.2108 | 0 | 0.000 |
| ENSP00000369895 | 3.88E-06 | -0.2108 | 0 | 0.283 |
| ENSP00000292357 | 8.55E-06 | -0.2108 | 210 | 0.171 |
| ENSP00000412064 | 5.69E-06 | -0.2110 | 197 | 0.144 |
| ENSP00000363388 | 8.56E-07 | -0.2110 | 0 | 0.172 |
| ENSP00000350570 | 4.10E-06 | -0.2110 | 0 | 0.092 |
| ENSP00000249044 | 7.98E-06 | -0.2111 | 0 | 0.098 |
| ENSP00000427015 | 2.74E-05 | -0.2111 | 303 | 0.692 |
| ENSP00000369897 | 9.54E-06 | -0.2111 | 403 | 0.708 |
| ENSP00000265007 | 6.50E-06 | -0.2111 | 0 | 0.125 |
| ENSP00000270077 | 4.72E-06 | -0.2111 | 0 | 0.172 |
| ENSP00000354689 | 4.71E-06 | -0.2112 | 0 | 0.087 |
| ENSP00000417706 | 1.69E-06 | -0.2112 | 0 | 0.195 |
| ENSP00000365908 | 3.07E-06 | -0.2112 | 0 | 0.095 |
| ENSP00000434236 | 5.61E-06 | -0.2113 | 671 | 0.000 |
| ENSP00000386532 | 7.73E-06 | -0.2114 | 202 | 0.641 |
| ENSP00000301246 | 4.67E-06 | -0.2114 | 0 | 0.171 |
| ENSP00000439056 | 2.67E-06 | -0.2114 | 0 | 0.102 |
| ENSP00000243457 | 1.46E-05 | -0.2114 | 163 | 0.451 |
| ENSP00000428205 | 1.35E-05 | -0.2114 | 181 | 0.808 |
| ENSP00000354968 | 2.26E-06 | -0.2114 | 0 | 0.000 |
| ENSP00000380247 | 8.53E-06 | -0.2115 | 363 | 0.521 |
| ENSP00000395086 | 2.51E-06 | -0.2115 | 0 | 0.269 |
| ENSP00000360958 | 4.64E-06 | -0.2115 | 0 | 0.206 |
| ENSP00000386845 | 3.48E-06 | -0.2115 | 0 | 0.180 |
| ENSP00000337477 | 3.13E-06 | -0.2115 | 0 | 0.614 |
| ENSP00000386141 | 3.60E-06 | -0.2115 | 0 | 0.080 |
| ENSP00000367697 | 3.97E-06 | -0.2115 | 0 | 0.103 |
| ENSP00000299578 | 3.08E-06 | -0.2115 | 0 | 0.182 |
| ENSP00000365529 | 5.97E-06 | -0.2116 | 0 | 0.114 |
| ENSP00000398124 | 7.16E-06 | -0.2116 | 163 | 0.810 |
| ENSP00000401867 | 2.50E-06 | -0.2116 | 0 | 0.265 |
| ENSP00000367631 | 6.53E-06 | -0.2116 | 201 | 0.000 |
| ENSP00000376390 | 6.67E-06 | -0.2116 | 0 | 0.087 |
| ENSP00000371802 | 7.08E-06 | -0.2116 | 0 | 0.979 |
| ENSP00000362285 | 5.46E-06 | -0.2117 | 0 | 0.086 |
| ENSP00000359778 | 1.50E-05 | -0.2117 | 242 | 0.000 |
| ENSP00000416935 | 5.36E-06 | -0.2117 | 0 | 0.000 |
| ENSP00000369456 | 1.11E-05 | -0.2118 | 226 | 0.551 |
| ENSP00000380779 | 1.68E-06 | -0.2118 | 0 | 0.073 |
| ENSP00000006724 | 1.61E-05 | -0.2118 | 0 | 0.169 |
| ENSP00000297354 | 1.46E-05 | -0.2119 | 0 | 0.146 |
| ENSP00000406046 | 1.50E-05 | -0.2119 | 314 | 0.773 |
| ENSP00000351379 | 8.47E-06 | -0.2119 | 0 | 0.098 |
| ENSP00000395650 | 1.11E-05 | -0.2119 | 201 | 0.000 |
| ENSP00000345974 | 4.49E-06 | -0.2119 | 0 | 0.069 |

| | | | | |
|---|---|---|---|---|
| ENSP00000412365 | 2.43E-06 | -0.2119 | 0 | 0.363 |
| ENSP00000357759 | 4.86E-06 | -0.2120 | 0 | 0.114 |
| ENSP00000357882 | 3.84E-06 | -0.2120 | 0 | 0.083 |
| ENSP00000351346 | 6.48E-06 | -0.2120 | 163 | 0.197 |
| ENSP00000343676 | 8.86E-06 | -0.2120 | 217 | 0.355 |
| ENSP00000392812 | 3.75E-06 | -0.2120 | 0 | 0.716 |
| ENSP00000370200 | 3.26E-06 | -0.2120 | 0 | 0.512 |
| ENSP00000373413 | 2.21E-06 | -0.2121 | 0 | 0.092 |
| ENSP00000345044 | 4.82E-06 | -0.2121 | 0 | 0.122 |
| ENSP00000375476 | 8.77E-07 | -0.2121 | 0 | 0.146 |
| ENSP00000366493 | 1.02E-05 | -0.2121 | 208 | 0.559 |
| ENSP00000371220 | 2.09E-06 | -0.2121 | 0 | 0.000 |
| ENSP00000216268 | 3.87E-06 | -0.2122 | 0 | 0.187 |
| ENSP00000386488 | 4.06E-06 | -0.2122 | 0 | 0.598 |
| ENSP00000378313 | 2.69E-06 | -0.2122 | 0 | 0.243 |
| ENSP00000449960 | 4.35E-06 | -0.2123 | 0 | 0.109 |
| ENSP00000345494 | 8.23E-06 | -0.2123 | 0 | 0.339 |
| ENSP00000367794 | 4.17E-06 | -0.2123 | 0 | 0.144 |
| ENSP00000320509 | 6.27E-06 | -0.2124 | 0 | 0.424 |
| ENSP00000354204 | 2.82E-06 | -0.2124 | 0 | 0.164 |
| ENSP00000342118 | 3.18E-06 | -0.2124 | 0 | 0.098 |
| ENSP00000367792 | 4.87E-06 | -0.2125 | 0 | 0.090 |
| ENSP00000356607 | 5.70E-06 | -0.2125 | 0 | 0.092 |
| ENSP00000417214 | 6.81E-06 | -0.2125 | 0 | 0.187 |
| ENSP00000246081 | 1.41E-05 | -0.2125 | 0 | 0.136 |
| ENSP00000362139 | 4.87E-06 | -0.2125 | 205 | 0.674 |
| ENSP00000232854 | 1.00E-05 | -0.2125 | 313 | 0.588 |
| ENSP00000358595 | 8.83E-06 | -0.2126 | 0 | 0.487 |
| ENSP00000445077 | 4.81E-06 | -0.2126 | 226 | 0.000 |
| ENSP00000286808 | 3.94E-06 | -0.2126 | 0 | 0.124 |
| ENSP00000363575 | 2.63E-06 | -0.2126 | 0 | 0.000 |
| ENSP00000360515 | 4.12E-06 | -0.2126 | 0 | 0.874 |
| ENSP00000302319 | 8.11E-06 | -0.2127 | 0 | 0.186 |
| ENSP00000397002 | 6.06E-06 | -0.2127 | 0 | 0.122 |
| ENSP00000238508 | 9.88E-06 | -0.2127 | 0 | 0.318 |
| ENSP00000340271 | 3.70E-06 | -0.2127 | 0 | 0.000 |
| ENSP00000349971 | 1.02E-05 | -0.2127 | 0 | 0.198 |
| ENSP00000323087 | 7.97E-06 | -0.2127 | 0 | 0.184 |
| ENSP00000385422 | 8.92E-06 | -0.2128 | 150 | 0.243 |
| ENSP00000386385 | 2.67E-06 | -0.2128 | 0 | 0.090 |
| ENSP00000382204 | 2.29E-06 | -0.2128 | 0 | 0.570 |
| ENSP00000362546 | 2.66E-06 | -0.2129 | 0 | 0.147 |
| ENSP00000376758 | 2.51E-06 | -0.2129 | 0 | 0.483 |
| ENSP00000357122 | 3.07E-06 | -0.2130 | 0 | 0.128 |
| ENSP00000335623 | 6.44E-06 | -0.2130 | 0 | 0.162 |
| ENSP00000327509 | 3.72E-06 | -0.2130 | 0 | 0.113 |

| | | | | |
|---|---|---|---|---|
| ENSP00000378166 | 4.09E-06 | -0.2130 | 0 | 0.164 |
| ENSP00000349142 | 7.46E-06 | -0.2131 | 343 | 0.496 |
| ENSP00000398729 | 4.14E-06 | -0.2131 | 0 | 0.000 |
| ENSP00000454770 | 7.51E-06 | -0.2131 | 202 | 0.675 |
| ENSP00000355444 | 9.82E-07 | -0.2131 | 0 | 0.173 |
| ENSP00000379441 | 2.72E-06 | -0.2131 | 0 | 0.421 |
| ENSP00000465204 | 2.49E-06 | -0.2131 | 0 | 0.144 |
| ENSP00000425679 | 1.24E-06 | -0.2132 | 0 | 0.000 |
| ENSP00000349654 | 1.03E-05 | -0.2132 | 211 | 0.298 |
| ENSP00000366240 | 6.10E-06 | -0.2133 | 0 | 0.104 |
| ENSP00000367228 | 4.03E-06 | -0.2134 | 0 | 0.000 |
| ENSP00000369411 | 5.16E-06 | -0.2134 | 201 | 0.716 |
| ENSP00000386239 | 7.35E-06 | -0.2134 | 202 | 0.645 |
| ENSP00000386531 | 2.80E-06 | -0.2134 | 0 | 0.054 |
| ENSP00000367815 | 2.39E-06 | -0.2134 | 0 | 0.056 |
| ENSP00000359904 | 5.23E-06 | -0.2134 | 0 | 0.130 |
| ENSP00000358423 | 6.26E-06 | -0.2134 | 0 | 0.428 |
| ENSP00000371364 | 3.00E-06 | -0.2134 | 0 | 0.141 |
| ENSP00000360803 | 6.60E-06 | -0.2135 | 363 | 0.102 |
| ENSP00000381340 | 1.36E-05 | -0.2135 | 900 | 0.246 |
| ENSP00000335382 | 1.85E-06 | -0.2135 | 0 | 0.119 |
| ENSP00000387851 | 8.94E-07 | -0.2136 | 0 | 0.000 |
| ENSP00000359307 | 2.62E-06 | -0.2136 | 0 | 0.000 |
| ENSP00000345895 | 3.66E-06 | -0.2136 | 0 | 0.531 |
| ENSP00000366863 | 1.04E-05 | -0.2136 | 215 | 0.569 |
| ENSP00000346536 | 4.91E-07 | -0.2137 | 0 | 0.143 |
| ENSP00000417806 | 1.82E-06 | -0.2137 | 0 | 0.144 |
| ENSP00000346335 | 4.03E-06 | -0.2137 | 0 | 0.275 |
| ENSP00000423822 | 1.14E-05 | -0.2137 | 208 | 0.188 |
| ENSP00000335320 | 1.17E-05 | -0.2137 | 172 | 0.752 |
| ENSP00000394472 | 2.41E-06 | -0.2137 | 0 | 0.172 |
| ENSP00000386163 | 1.72E-06 | -0.2138 | 0 | 0.000 |
| ENSP00000316740 | 1.85E-06 | -0.2138 | 0 | 0.128 |
| ENSP00000420608 | 3.90E-06 | -0.2139 | 0 | 0.118 |
| ENSP00000451253 | 1.13E-05 | -0.2139 | 187 | 0.000 |
| ENSP00000370912 | 5.34E-06 | -0.2139 | 205 | 0.677 |
| ENSP00000359982 | 3.41E-06 | -0.2139 | 0 | 0.107 |
| ENSP00000420840 | 4.43E-06 | -0.2139 | 0 | 0.095 |
| ENSP00000385927 | 4.47E-06 | -0.2140 | 0 | 0.095 |
| ENSP00000378441 | 4.86E-06 | -0.2140 | 0 | 0.094 |
| ENSP00000441032 | 2.86E-06 | -0.2140 | 0 | 0.105 |
| ENSP00000290552 | 4.68E-06 | -0.2140 | 0 | 0.815 |
| ENSP00000353518 | 1.24E-05 | -0.2140 | 201 | 0.642 |
| ENSP00000381097 | 1.17E-05 | -0.2141 | 206 | 0.671 |
| ENSP00000253801 | 1.31E-05 | -0.2141 | 163 | 0.317 |
| ENSP00000273610 | 1.28E-05 | -0.2141 | 0 | 0.334 |

| | | | | |
|---|---|---|---|---|
| ENSP00000314914 | 1.10E-05 | -0.2142 | 0 | 0.133 |
| ENSP00000346103 | 8.82E-06 | -0.2142 | 235 | 0.313 |
| ENSP00000358060 | 4.74E-06 | -0.2142 | 405 | 0.710 |
| ENSP00000372857 | 1.75E-06 | -0.2142 | 0 | 0.149 |
| ENSP00000365301 | 5.54E-06 | -0.2143 | 0 | 0.883 |
| ENSP00000301917 | 3.05E-06 | -0.2143 | 0 | 0.205 |
| ENSP00000357080 | 2.34E-06 | -0.2144 | 0 | 0.151 |
| ENSP00000395259 | 7.04E-06 | -0.2144 | 165 | 0.000 |
| ENSP00000468633 | 1.63E-05 | -0.2144 | 332 | 0.463 |
| ENSP00000413493 | 9.62E-06 | -0.2144 | 201 | 0.566 |
| ENSP00000412436 | 7.19E-07 | -0.2144 | 0 | 0.202 |
| ENSP00000361025 | 3.84E-06 | -0.2145 | 0 | 0.000 |
| ENSP00000240619 | 2.70E-06 | -0.2145 | 0 | 0.143 |
| ENSP00000456200 | 1.44E-06 | -0.2146 | 0 | 0.054 |
| ENSP00000354778 | 6.18E-06 | -0.2146 | 0 | 0.267 |
| ENSP00000411010 | 4.85E-06 | -0.2147 | 0 | 0.788 |
| ENSP00000402595 | 2.72E-06 | -0.2147 | 0 | 0.244 |
| ENSP00000367360 | 4.18E-06 | -0.2148 | 0 | 0.103 |
| ENSP00000470441 | 2.20E-08 | -0.2148 | 0 | 0.000 |
| ENSP00000380321 | 8.77E-06 | -0.2148 | 358 | 0.220 |
| ENSP00000418734 | 1.46E-05 | -0.2148 | 186 | 0.831 |
| ENSP00000371152 | 7.96E-06 | -0.2148 | 154 | 0.137 |
| ENSP00000360891 | 8.86E-06 | -0.2148 | 333 | 0.710 |
| ENSP00000348283 | 5.15E-06 | -0.2148 | 0 | 0.482 |
| ENSP00000379680 | 5.12E-06 | -0.2148 | 0 | 0.154 |
| ENSP00000282641 | 6.67E-06 | -0.2149 | 0 | 0.263 |
| ENSP00000158009 | 7.63E-06 | -0.2149 | 0 | 0.182 |
| ENSP00000361900 | 7.42E-06 | -0.2149 | 338 | 0.707 |
| ENSP00000384524 | 5.01E-06 | -0.2149 | 0 | 0.782 |
| ENSP00000354590 | 2.71E-06 | -0.2150 | 0 | 0.125 |
| ENSP00000414964 | 6.94E-06 | -0.2151 | 0 | 0.095 |
| ENSP00000386499 | 2.16E-07 | -0.2151 | 0 | 0.000 |
| ENSP00000367727 | 2.38E-06 | -0.2151 | 0 | 0.086 |
| ENSP00000358919 | 7.98E-06 | -0.2151 | 240 | 0.205 |
| ENSP00000419901 | 4.65E-06 | -0.2152 | 0 | 0.103 |
| ENSP00000330433 | 1.31E-05 | -0.2152 | 457 | 0.127 |
| ENSP00000375413 | 3.21E-06 | -0.2152 | 0 | 0.000 |
| ENSP00000354876 | 1.10E-05 | -0.2152 | 232 | 0.534 |
| ENSP00000346693 | 6.45E-06 | -0.2153 | 0 | 0.389 |
| ENSP00000405533 | 8.09E-06 | -0.2153 | 197 | 0.000 |
| ENSP00000417654 | 2.23E-06 | -0.2153 | 0 | 0.072 |
| ENSP00000369045 | 5.40E-06 | -0.2153 | 0 | 0.088 |
| ENSP00000348657 | 3.03E-06 | -0.2154 | 0 | 0.378 |
| ENSP00000354900 | 8.50E-06 | -0.2154 | 0 | 0.544 |
| ENSP00000411514 | 1.85E-06 | -0.2154 | 0 | 0.140 |
| ENSP00000354733 | 6.08E-06 | -0.2154 | 0 | 0.140 |

| | | | | |
|---|---|---|---|---|
| ENSP00000229563 | 8.05E-06 | -0.2154 | 0 | 0.114 |
| ENSP00000372093 | 5.07E-06 | -0.2154 | 0 | 0.543 |
| ENSP00000316532 | 2.47E-06 | -0.2155 | 0 | 0.138 |
| ENSP00000365477 | 4.76E-06 | -0.2155 | 0 | 0.133 |
| ENSP00000368304 | 2.61E-06 | -0.2155 | 0 | 0.138 |
| ENSP00000339971 | 3.94E-06 | -0.2155 | 0 | 0.121 |
| ENSP00000228515 | 4.04E-06 | -0.2156 | 0 | 0.095 |
| ENSP00000359345 | 1.28E-05 | -0.2156 | 335 | 0.000 |
| ENSP00000363435 | 1.17E-05 | -0.2156 | 328 | 0.762 |
| ENSP00000379034 | 3.43E-06 | -0.2157 | 0 | 0.090 |
| ENSP00000431040 | 5.96E-06 | -0.2157 | 265 | 0.731 |
| ENSP00000369038 | 1.15E-05 | -0.2157 | 344 | 0.702 |
| ENSP00000359729 | 3.81E-06 | -0.2158 | 0 | 0.122 |
| ENSP00000356842 | 6.32E-06 | -0.2158 | 0 | 0.116 |
| ENSP00000293274 | 7.89E-06 | -0.2158 | 0 | 0.000 |
| ENSP00000423897 | 2.89E-06 | -0.2159 | 0 | 0.154 |
| ENSP00000367453 | 4.00E-06 | -0.2159 | 0 | 0.143 |
| ENSP00000359682 | 6.64E-06 | -0.2159 | 0 | 0.292 |
| ENSP00000404381 | 5.69E-06 | -0.2159 | 862 | 0.290 |
| ENSP00000356329 | 5.44E-06 | -0.2160 | 0 | 0.133 |
| ENSP00000387683 | 2.68E-06 | -0.2160 | 0 | 0.070 |
| ENSP00000359988 | 3.57E-06 | -0.2160 | 0 | 0.818 |
| ENSP00000376204 | 1.57E-05 | -0.2160 | 616 | 0.000 |
| ENSP00000436005 | 3.77E-06 | -0.2160 | 0 | 0.162 |
| ENSP00000361650 | 3.20E-06 | -0.2161 | 0 | 0.106 |
| ENSP00000341819 | 1.29E-05 | -0.2161 | 489 | 0.185 |
| ENSP00000327557 | 1.53E-05 | -0.2161 | 201 | 0.000 |
| ENSP00000302951 | 5.49E-06 | -0.2162 | 0 | 0.288 |
| ENSP00000401450 | 6.37E-06 | -0.2162 | 173 | 0.000 |
| ENSP00000385551 | 9.01E-06 | -0.2162 | 207 | 0.871 |
| ENSP00000450811 | 2.35E-06 | -0.2162 | 0 | 0.000 |
| ENSP00000280057 | 4.46E-06 | -0.2163 | 0 | 0.135 |
| ENSP00000298527 | 1.23E-05 | -0.2163 | 325 | 0.300 |
| ENSP00000384291 | 3.24E-06 | -0.2163 | 0 | 0.121 |
| ENSP00000386738 | 3.39E-06 | -0.2163 | 0 | 0.156 |
| ENSP00000378588 | 3.87E-06 | -0.2163 | 0 | 0.586 |
| ENSP00000345575 | 5.71E-06 | -0.2164 | 870 | 0.292 |
| ENSP00000371051 | 4.47E-06 | -0.2164 | 0 | 0.093 |
| ENSP00000362208 | 5.60E-06 | -0.2164 | 359 | 0.808 |
| ENSP00000398803 | 5.36E-06 | -0.2164 | 198 | 0.183 |
| ENSP00000429633 | 1.85E-05 | -0.2165 | 277 | 0.000 |
| ENSP00000453129 | 1.00E-05 | -0.2165 | 157 | 0.202 |
| ENSP00000363654 | 2.03E-06 | -0.2165 | 0 | 0.121 |
| ENSP00000368869 | 2.30E-06 | -0.2165 | 0 | 0.144 |
| ENSP00000420333 | 1.84E-06 | -0.2166 | 0 | 0.098 |
| ENSP00000328083 | 4.04E-06 | -0.2166 | 0 | 0.128 |

| | | | | |
|---|---|---|---|---|
| ENSP00000258662 | 1.31E-05 | -0.2167 | 195 | 0.114 |
| ENSP00000376162 | 3.45E-06 | -0.2167 | 0 | 0.398 |
| ENSP00000354293 | 3.12E-06 | -0.2167 | 0 | 0.000 |
| ENSP00000372126 | 4.35E-06 | -0.2167 | 0 | 0.113 |
| ENSP00000384182 | 1.65E-06 | -0.2167 | 0 | 0.154 |
| ENSP00000358232 | 4.55E-06 | -0.2168 | 0 | 0.150 |
| ENSP00000289707 | 1.17E-05 | -0.2168 | 0 | 0.153 |
| ENSP00000355870 | 3.51E-06 | -0.2169 | 0 | 0.830 |
| ENSP00000386538 | 1.80E-06 | -0.2169 | 0 | 0.109 |
| ENSP00000403802 | 2.53E-06 | -0.2169 | 0 | 0.113 |
| ENSP00000426638 | 3.46E-06 | -0.2169 | 0 | 0.234 |
| ENSP00000335392 | 2.43E-06 | -0.2169 | 0 | 0.103 |
| ENSP00000348752 | 5.81E-06 | -0.2170 | 0 | 0.137 |
| ENSP00000406037 | 1.94E-05 | -0.2170 | 320 | 0.000 |
| ENSP00000365167 | 2.59E-06 | -0.2170 | 0 | 0.000 |
| ENSP00000457168 | 2.92E-06 | -0.2170 | 0 | 0.108 |
| ENSP00000350050 | 2.67E-06 | -0.2170 | 0 | 0.112 |
| ENSP00000359401 | 3.60E-06 | -0.2170 | 0 | 0.116 |
| ENSP00000263084 | 8.21E-06 | -0.2170 | 0 | 0.241 |
| ENSP00000359333 | 4.81E-06 | -0.2171 | 0 | 0.135 |
| ENSP00000358309 | 8.70E-06 | -0.2171 | 205 | 0.675 |
| ENSP00000248643 | 4.03E-06 | -0.2171 | 0 | 0.000 |
| ENSP00000295201 | 3.98E-06 | -0.2171 | 0 | 0.072 |
| ENSP00000302938 | 1.34E-05 | -0.2171 | 0 | 0.103 |
| ENSP00000375829 | 7.57E-06 | -0.2171 | 216 | 0.486 |
| ENSP00000344936 | 9.85E-06 | -0.2171 | 0 | 0.640 |
| ENSP00000428338 | 9.32E-07 | -0.2172 | 0 | 0.101 |
| ENSP00000378733 | 4.60E-06 | -0.2172 | 0 | 0.167 |
| ENSP00000270452 | 8.89E-06 | -0.2172 | 0 | 0.179 |
| ENSP00000333593 | 4.52E-06 | -0.2172 | 0 | 0.133 |
| ENSP00000369604 | 1.79E-06 | -0.2173 | 0 | 0.150 |
| ENSP00000413632 | 4.93E-06 | -0.2173 | 0 | 0.642 |
| ENSP00000452085 | 1.70E-05 | -0.2173 | 330 | 0.000 |
| ENSP00000344909 | 4.33E-06 | -0.2173 | 0 | 0.108 |
| ENSP00000265529 | 1.15E-05 | -0.2175 | 720 | 0.170 |
| ENSP00000420443 | 4.73E-06 | -0.2175 | 0 | 0.136 |
| ENSP00000360874 | 7.17E-06 | -0.2175 | 316 | 0.703 |
| ENSP00000357393 | 1.32E-05 | -0.2175 | 191 | 0.737 |
| ENSP00000253262 | 1.13E-05 | -0.2176 | 0 | 0.000 |
| ENSP00000361646 | 3.56E-06 | -0.2176 | 0 | 0.112 |
| ENSP00000450399 | 2.67E-05 | -0.2176 | 323 | 0.540 |
| ENSP00000411012 | 2.88E-06 | -0.2176 | 0 | 0.106 |
| ENSP00000384222 | 3.18E-06 | -0.2176 | 0 | 0.298 |
| ENSP00000261047 | 1.58E-05 | -0.2177 | 483 | 0.288 |
| ENSP00000345359 | 5.67E-06 | -0.2178 | 0 | 0.124 |
| ENSP00000345193 | 6.12E-06 | -0.2178 | 255 | 0.677 |

| | | | | |
|---|---|---|---|---|
| ENSP00000322097 | 6.23E-06 | -0.2178 | 0 | 0.104 |
| ENSP00000349640 | 3.40E-06 | -0.2178 | 0 | 0.740 |
| ENSP00000293303 | 3.05E-06 | -0.2178 | 0 | 0.079 |
| ENSP00000354958 | 9.53E-06 | -0.2179 | 0 | 0.139 |
| ENSP00000384604 | 5.17E-06 | -0.2179 | 0 | 0.101 |
| ENSP00000418667 | 3.49E-06 | -0.2179 | 0 | 0.134 |
| ENSP00000468007 | 9.39E-07 | -0.2180 | 0 | 0.159 |
| ENSP00000381567 | 4.56E-06 | -0.2180 | 0 | 0.411 |
| ENSP00000354945 | 3.03E-06 | -0.2181 | 0 | 0.000 |
| ENSP00000359512 | 6.08E-06 | -0.2181 | 0 | 0.162 |
| ENSP00000364370 | 3.52E-06 | -0.2182 | 0 | 0.097 |
| ENSP00000415890 | 3.61E-06 | -0.2183 | 0 | 0.120 |
| ENSP00000418210 | 1.43E-05 | -0.2183 | 0 | 0.249 |
| ENSP00000470087 | 1.34E-05 | -0.2183 | 229 | 0.000 |
| ENSP00000361199 | 3.40E-06 | -0.2183 | 0 | 0.124 |
| ENSP00000359224 | 2.42E-06 | -0.2183 | 0 | 0.169 |
| ENSP00000380880 | 3.00E-06 | -0.2184 | 0 | 0.135 |
| ENSP00000315098 | 8.75E-06 | -0.2184 | 206 | 0.060 |
| ENSP00000318077 | 1.11E-05 | -0.2184 | 212 | 0.411 |
| ENSP00000436897 | 2.51E-06 | -0.2184 | 0 | 0.146 |
| ENSP00000354718 | 8.27E-06 | -0.2185 | 150 | 0.656 |
| ENSP00000372059 | 7.09E-06 | -0.2185 | 0 | 0.148 |
| ENSP00000390084 | 2.54E-06 | -0.2185 | 0 | 0.070 |
| ENSP00000387617 | 7.15E-07 | -0.2186 | 0 | 0.106 |
| ENSP00000377696 | 5.02E-06 | -0.2186 | 0 | 0.211 |
| ENSP00000335306 | 4.51E-06 | -0.2186 | 0 | 0.338 |
| ENSP00000312706 | 5.63E-06 | -0.2186 | 0 | 0.074 |
| ENSP00000293604 | 9.87E-06 | -0.2186 | 0 | 0.224 |
| ENSP00000373734 | 3.06E-06 | -0.2187 | 0 | 0.084 |
| ENSP00000364512 | 3.44E-06 | -0.2188 | 0 | 0.299 |
| ENSP00000421489 | 1.95E-06 | -0.2188 | 0 | 0.144 |
| ENSP00000405963 | 3.61E-06 | -0.2189 | 0 | 0.625 |
| ENSP00000338964 | 1.45E-05 | -0.2189 | 900 | 0.284 |
| ENSP00000297866 | 4.43E-06 | -0.2190 | 0 | 0.000 |
| ENSP00000411418 | 4.91E-06 | -0.2190 | 0 | 0.082 |
| ENSP00000349856 | 2.38E-06 | -0.2191 | 0 | 0.176 |
| ENSP00000297323 | 5.59E-06 | -0.2191 | 0 | 0.636 |
| ENSP00000424151 | 2.76E-06 | -0.2192 | 0 | 0.125 |
| ENSP00000396615 | 5.61E-06 | -0.2192 | 0 | 0.081 |
| ENSP00000378857 | 3.20E-06 | -0.2194 | 0 | 0.720 |
| ENSP00000389998 | 9.54E-06 | -0.2194 | 576 | 0.258 |
| ENSP00000333441 | 8.14E-06 | -0.2194 | 0 | 0.518 |
| ENSP00000386869 | 2.88E-06 | -0.2194 | 0 | 0.436 |
| ENSP00000371345 | 6.01E-06 | -0.2194 | 340 | 0.149 |
| ENSP00000328992 | 3.04E-06 | -0.2195 | 0 | 0.296 |
| ENSP00000266688 | 1.41E-05 | -0.2195 | 160 | 0.000 |

| | | | | |
|---|---|---|---|---|
| ENSP00000355720 | 2.97E-06 | -0.2196 | 0 | 0.546 |
| ENSP00000401859 | 1.93E-06 | -0.2196 | 0 | 0.000 |
| ENSP00000369979 | 1.22E-05 | -0.2196 | 152 | 0.132 |
| ENSP00000360360 | 1.96E-06 | -0.2196 | 0 | 0.166 |
| ENSP00000464814 | 9.54E-06 | -0.2196 | 414 | 0.481 |
| ENSP00000356991 | 4.32E-06 | -0.2196 | 0 | 0.084 |
| ENSP00000311712 | 1.31E-05 | -0.2197 | 235 | 0.121 |
| ENSP00000328149 | 4.58E-06 | -0.2197 | 0 | 0.137 |
| ENSP00000374507 | 3.46E-06 | -0.2197 | 0 | 0.135 |
| ENSP00000433816 | 6.79E-08 | -0.2197 | 0 | 0.215 |
| ENSP00000381489 | 6.41E-07 | -0.2197 | 0 | 0.167 |
| ENSP00000419325 | 1.12E-05 | -0.2198 | 471 | 0.689 |
| ENSP00000360773 | 7.24E-06 | -0.2198 | 0 | 0.154 |
| ENSP00000332454 | 1.26E-05 | -0.2199 | 375 | 0.715 |
| ENSP00000384288 | 5.47E-06 | -0.2200 | 0 | 0.000 |
| ENSP00000343799 | 4.04E-06 | -0.2200 | 0 | 0.145 |
| ENSP00000329553 | 1.30E-05 | -0.2200 | 246 | 0.264 |
| ENSP00000365625 | 1.60E-05 | -0.2200 | 299 | 0.938 |
| ENSP00000266542 | 9.83E-06 | -0.2200 | 0 | 0.115 |
| ENSP00000398789 | 3.02E-06 | -0.2201 | 0 | 0.091 |
| ENSP00000416125 | 1.57E-06 | -0.2201 | 0 | 0.141 |
| ENSP00000359799 | 9.91E-06 | -0.2202 | 914 | 0.367 |
| ENSP00000375986 | 5.72E-06 | -0.2202 | 0 | 0.628 |
| ENSP00000376333 | 4.06E-06 | -0.2202 | 0 | 0.182 |
| ENSP00000376443 | 3.63E-06 | -0.2202 | 0 | 0.100 |
| ENSP00000354415 | 8.24E-06 | -0.2202 | 0 | 0.300 |
| ENSP00000365172 | 2.50E-06 | -0.2202 | 0 | 0.093 |
| ENSP00000342991 | 4.16E-06 | -0.2202 | 0 | 0.162 |
| ENSP00000259365 | 1.40E-05 | -0.2203 | 904 | 0.233 |
| ENSP00000321179 | 3.74E-06 | -0.2203 | 0 | 0.146 |
| ENSP00000334866 | 4.11E-06 | -0.2203 | 0 | 0.129 |
| ENSP00000439594 | 3.71E-06 | -0.2203 | 0 | 0.000 |
| ENSP00000308699 | 3.75E-06 | -0.2203 | 0 | 0.000 |
| ENSP00000409315 | 1.16E-06 | -0.2204 | 0 | 0.237 |
| ENSP00000356189 | 6.33E-06 | -0.2204 | 0 | 0.123 |
| ENSP00000311257 | 5.05E-06 | -0.2205 | 0 | 0.104 |
| ENSP00000380378 | 1.76E-05 | -0.2205 | 198 | 0.753 |
| ENSP00000382777 | 2.59E-06 | -0.2205 | 0 | 0.117 |
| ENSP00000364902 | 9.62E-06 | -0.2205 | 319 | 0.389 |
| ENSP00000349297 | 4.76E-06 | -0.2205 | 0 | 0.475 |
| ENSP00000381473 | 2.38E-06 | -0.2206 | 0 | 0.142 |
| ENSP00000379739 | 3.50E-06 | -0.2207 | 0 | 0.206 |
| ENSP00000370557 | 2.76E-06 | -0.2207 | 0 | 0.395 |
| ENSP00000366800 | 5.35E-06 | -0.2207 | 776 | 0.243 |
| ENSP00000453502 | 2.48E-06 | -0.2208 | 0 | 0.077 |
| ENSP00000325618 | 8.43E-06 | -0.2208 | 0 | 0.255 |

| | | | | |
|---|---|---|---|---|
| ENSP00000339251 | 1.25E-05 | -0.2209 | 0 | 0.131 |
| ENSP00000381480 | 3.32E-06 | -0.2209 | 0 | 0.086 |
| ENSP00000388107 | 2.67E-05 | -0.2210 | 285 | 0.826 |
| ENSP00000381605 | 2.09E-06 | -0.2210 | 0 | 0.133 |
| ENSP00000342570 | 5.55E-06 | -0.2210 | 0 | 0.121 |
| ENSP00000384474 | 3.65E-06 | -0.2211 | 0 | 0.313 |
| ENSP00000349727 | 5.40E-06 | -0.2211 | 0 | 0.688 |
| ENSP00000357012 | 3.85E-06 | -0.2211 | 0 | 0.697 |
| ENSP00000363676 | 9.55E-06 | -0.2212 | 406 | 0.819 |
| ENSP00000432481 | 3.92E-06 | -0.2212 | 0 | 0.078 |
| ENSP00000385142 | 8.58E-06 | -0.2212 | 197 | 0.341 |
| ENSP00000359783 | 4.01E-06 | -0.2212 | 0 | 0.276 |
| ENSP00000353465 | 4.24E-06 | -0.2212 | 0 | 0.097 |
| ENSP00000461295 | 2.50E-06 | -0.2213 | 0 | 0.000 |
| ENSP00000316842 | 9.51E-06 | -0.2213 | 181 | 0.749 |
| ENSP00000386200 | 8.49E-06 | -0.2214 | 207 | 0.000 |
| ENSP00000360215 | 9.33E-06 | -0.2214 | 210 | 0.499 |
| ENSP00000270593 | 6.55E-06 | -0.2214 | 0 | 0.148 |
| ENSP00000334186 | 1.14E-05 | -0.2214 | 438 | 0.416 |
| ENSP00000424400 | 2.30E-06 | -0.2215 | 0 | 0.105 |
| ENSP00000349270 | 4.27E-06 | -0.2215 | 0 | 0.198 |
| ENSP00000466680 | 2.29E-05 | -0.2215 | 211 | 0.601 |
| ENSP00000368528 | 2.36E-06 | -0.2215 | 0 | 0.104 |
| ENSP00000379834 | 1.07E-05 | -0.2215 | 157 | 0.392 |
| ENSP00000405514 | 4.96E-06 | -0.2216 | 0 | 0.224 |
| ENSP00000430497 | 4.70E-06 | -0.2216 | 0 | 0.562 |
| ENSP00000374135 | 8.69E-06 | -0.2216 | 359 | 0.633 |
| ENSP00000380597 | 1.21E-05 | -0.2216 | 336 | 0.000 |
| ENSP00000294889 | 2.10E-06 | -0.2217 | 0 | 0.144 |
| ENSP00000345826 | 3.98E-06 | -0.2218 | 0 | 0.099 |
| ENSP00000361602 | 3.18E-06 | -0.2218 | 0 | 0.286 |
| ENSP00000370297 | 5.19E-06 | -0.2218 | 0 | 0.785 |
| ENSP00000380184 | 4.50E-06 | -0.2219 | 0 | 0.000 |
| ENSP00000346550 | 8.12E-06 | -0.2220 | 164 | 0.365 |
| ENSP00000360021 | 4.13E-06 | -0.2220 | 0 | 0.444 |
| ENSP00000366953 | 1.90E-06 | -0.2221 | 0 | 0.109 |
| ENSP00000352270 | 7.27E-06 | -0.2221 | 0 | 0.091 |
| ENSP00000369531 | 5.59E-06 | -0.2221 | 183 | 0.707 |
| ENSP00000385576 | 6.72E-06 | -0.2222 | 157 | 0.105 |
| ENSP00000374237 | 3.38E-06 | -0.2223 | 0 | 0.101 |
| ENSP00000417161 | 1.35E-05 | -0.2223 | 256 | 0.165 |
| ENSP00000251776 | 1.48E-05 | -0.2223 | 0 | 0.152 |
| ENSP00000389087 | 3.70E-06 | -0.2223 | 0 | 0.073 |
| ENSP00000246070 | 1.32E-05 | -0.2223 | 0 | 0.142 |
| ENSP00000357762 | 4.16E-06 | -0.2224 | 0 | 0.000 |
| ENSP00000285013 | 2.40E-06 | -0.2224 | 0 | 0.135 |

| | | | | |
|---|---|---|---|---|
| ENSP00000325954 | 8.83E-06 | -0.2224 | 0 | 0.113 |
| ENSP00000447731 | 2.78E-06 | -0.2225 | 0 | 0.086 |
| ENSP00000368688 | 2.36E-06 | -0.2226 | 0 | 0.105 |
| ENSP00000430897 | 1.89E-06 | -0.2226 | 0 | 0.128 |
| ENSP00000355902 | 4.11E-06 | -0.2226 | 0 | 0.098 |
| ENSP00000358062 | 3.57E-06 | -0.2226 | 0 | 0.108 |
| ENSP00000349365 | 4.48E-06 | -0.2227 | 0 | 0.479 |
| ENSP00000377980 | 6.44E-06 | -0.2227 | 0 | 0.082 |
| ENSP00000398064 | 4.02E-06 | -0.2228 | 0 | 0.096 |
| ENSP00000358019 | 2.60E-06 | -0.2228 | 0 | 0.388 |
| ENSP00000307636 | 8.41E-06 | -0.2229 | 0 | 0.193 |
| ENSP00000368799 | 3.06E-06 | -0.2229 | 0 | 0.158 |
| ENSP00000353048 | 3.53E-06 | -0.2229 | 0 | 0.645 |
| ENSP00000362936 | 7.02E-06 | -0.2229 | 0 | 0.951 |
| ENSP00000368858 | 2.61E-06 | -0.2229 | 0 | 0.121 |
| ENSP00000357072 | 3.43E-06 | -0.2230 | 0 | 0.144 |
| ENSP00000301031 | 3.67E-06 | -0.2230 | 0 | 0.000 |
| ENSP00000357772 | 3.93E-06 | -0.2230 | 0 | 0.138 |
| ENSP00000262074 | 9.05E-06 | -0.2231 | 0 | 0.109 |
| ENSP00000357150 | 1.09E-05 | -0.2231 | 207 | 0.464 |
| ENSP00000358272 | 2.08E-06 | -0.2231 | 0 | 0.113 |
| ENSP00000377047 | 1.42E-05 | -0.2231 | 223 | 0.772 |
| ENSP00000375478 | 5.29E-06 | -0.2232 | 0 | 0.076 |
| ENSP00000374323 | 5.06E-06 | -0.2232 | 205 | 0.674 |
| ENSP00000455643 | 4.80E-06 | -0.2232 | 200 | 0.153 |
| ENSP00000361932 | 1.01E-05 | -0.2232 | 185 | 0.101 |
| ENSP00000297784 | 4.99E-06 | -0.2232 | 0 | 0.164 |
| ENSP00000424077 | 1.86E-06 | -0.2232 | 0 | 0.583 |
| ENSP00000418112 | 9.25E-06 | -0.2233 | 270 | 0.738 |
| ENSP00000350881 | 2.85E-06 | -0.2233 | 0 | 0.119 |
| ENSP00000270642 | 8.50E-06 | -0.2234 | 0 | 0.162 |
| ENSP00000363478 | 3.25E-06 | -0.2234 | 0 | 0.104 |
| ENSP00000390423 | 2.34E-06 | -0.2235 | 0 | 0.068 |
| ENSP00000441024 | 3.82E-06 | -0.2235 | 0 | 0.000 |
| ENSP00000345868 | 4.37E-06 | -0.2235 | 0 | 0.107 |
| ENSP00000286732 | 3.47E-06 | -0.2236 | 0 | 0.000 |
| ENSP00000350036 | 3.45E-06 | -0.2237 | 0 | 0.694 |
| ENSP00000457512 | 1.71E-06 | -0.2237 | 0 | 0.000 |
| ENSP00000408464 | 8.35E-06 | -0.2237 | 177 | 0.341 |
| ENSP00000383737 | 2.80E-06 | -0.2237 | 0 | 0.073 |
| ENSP00000360371 | 4.97E-06 | -0.2237 | 0 | 0.547 |
| ENSP00000249079 | 1.47E-05 | -0.2238 | 0 | 0.150 |
| ENSP00000364721 | 4.67E-06 | -0.2238 | 0 | 0.333 |
| ENSP00000391942 | 4.33E-06 | -0.2238 | 0 | 0.123 |
| ENSP00000376506 | 4.30E-06 | -0.2239 | 0 | 0.382 |
| ENSP00000384164 | 4.96E-06 | -0.2239 | 0 | 0.192 |

| | | | | |
|---|---|---|---|---|
| ENSP00000402239 | 3.56E-06 | -0.2239 | 0 | 0.111 |
| ENSP00000401362 | 1.96E-06 | -0.2239 | 0 | 0.120 |
| ENSP00000453969 | 2.78E-06 | -0.2239 | 0 | 0.161 |
| ENSP00000389140 | 1.16E-05 | -0.2239 | 190 | 0.609 |
| ENSP00000361180 | 2.86E-06 | -0.2240 | 0 | 0.577 |
| ENSP00000363384 | 5.73E-06 | -0.2240 | 0 | 0.839 |
| ENSP00000350265 | 9.26E-06 | -0.2240 | 169 | 0.170 |
| ENSP00000448012 | 1.52E-05 | -0.2240 | 359 | 0.297 |
| ENSP00000426159 | 8.77E-07 | -0.2241 | 0 | 0.163 |
| ENSP00000244296 | 2.41E-06 | -0.2241 | 0 | 0.124 |
| ENSP00000342931 | 5.80E-06 | -0.2242 | 0 | 0.853 |
| ENSP00000369434 | 6.57E-06 | -0.2242 | 202 | 0.638 |
| ENSP00000397598 | 4.53E-06 | -0.2242 | 0 | 0.296 |
| ENSP00000404438 | 1.49E-06 | -0.2242 | 0 | 0.361 |
| ENSP00000343656 | 8.51E-06 | -0.2242 | 157 | 0.275 |
| ENSP00000393559 | 5.98E-06 | -0.2242 | 0 | 0.080 |
| ENSP00000405482 | 3.98E-06 | -0.2243 | 0 | 0.103 |
| ENSP00000370546 | 3.57E-06 | -0.2243 | 0 | 0.051 |
| ENSP00000272907 | 1.49E-05 | -0.2243 | 0 | 0.151 |
| ENSP00000368517 | 3.43E-06 | -0.2244 | 0 | 0.111 |
| ENSP00000385000 | 4.77E-06 | -0.2244 | 0 | 0.222 |
| ENSP00000392262 | 1.94E-05 | -0.2244 | 261 | 0.661 |
| ENSP00000416696 | 4.14E-06 | -0.2245 | 0 | 0.000 |
| ENSP00000333374 | 5.12E-06 | -0.2246 | 0 | 0.066 |
| ENSP00000272164 | 1.46E-05 | -0.2247 | 677 | 0.934 |
| ENSP00000306407 | 5.74E-06 | -0.2247 | 0 | 0.102 |
| ENSP00000458307 | 1.37E-05 | -0.2247 | 900 | 0.272 |
| ENSP00000391596 | 3.47E-06 | -0.2247 | 0 | 0.144 |
| ENSP00000451131 | 7.53E-06 | -0.2248 | 316 | 0.702 |
| ENSP00000286733 | 1.53E-05 | -0.2248 | 349 | 0.184 |
| ENSP00000321386 | 4.39E-06 | -0.2248 | 0 | 0.117 |
| ENSP00000470240 | 1.41E-06 | -0.2248 | 0 | 0.000 |
| ENSP00000381666 | 4.12E-06 | -0.2249 | 0 | 0.092 |
| ENSP00000341206 | 1.68E-05 | -0.2249 | 459 | 0.049 |
| ENSP00000385616 | 3.98E-06 | -0.2249 | 0 | 0.324 |
| ENSP00000380109 | 7.45E-06 | -0.2249 | 202 | 0.691 |
| ENSP00000361397 | 1.42E-06 | -0.2250 | 0 | 0.000 |
| ENSP00000359759 | 1.69E-06 | -0.2250 | 0 | 0.000 |
| ENSP00000382688 | 1.32E-05 | -0.2250 | 270 | 0.000 |
| ENSP00000341051 | 1.22E-05 | -0.2252 | 176 | 0.104 |
| ENSP00000387928 | 2.58E-06 | -0.2252 | 0 | 0.000 |
| ENSP00000403760 | 2.10E-06 | -0.2252 | 0 | 0.594 |
| ENSP00000414558 | 5.09E-06 | -0.2253 | 0 | 0.174 |
| ENSP00000354739 | 4.43E-06 | -0.2253 | 421 | 0.804 |
| ENSP00000450436 | 5.01E-06 | -0.2253 | 159 | 0.191 |
| ENSP00000389585 | 1.13E-05 | -0.2253 | 187 | 0.000 |

| | | | | |
|---|---|---|---|---|
| ENSP00000299492 | 1.23E-05 | -0.2253 | 182 | 0.121 |
| ENSP00000363092 | 1.77E-05 | -0.2253 | 587 | 0.634 |
| ENSP00000360992 | 3.77E-06 | -0.2254 | 0 | 0.325 |
| ENSP00000300961 | 4.58E-06 | -0.2254 | 0 | 0.101 |
| ENSP00000387266 | 1.29E-05 | -0.2255 | 0 | 0.157 |
| ENSP00000362863 | 3.59E-06 | -0.2255 | 0 | 0.111 |
| ENSP00000423463 | 8.77E-06 | -0.2255 | 0 | 0.377 |
| ENSP00000348129 | 9.03E-06 | -0.2255 | 242 | 0.000 |
| ENSP00000422375 | 3.24E-06 | -0.2255 | 0 | 0.174 |
| ENSP00000384081 | 4.40E-06 | -0.2256 | 0 | 0.107 |
| ENSP00000369407 | 2.89E-06 | -0.2256 | 0 | 0.084 |
| ENSP00000307954 | 5.41E-06 | -0.2257 | 0 | 0.167 |
| ENSP00000345243 | 6.40E-06 | -0.2257 | 0 | 0.208 |
| ENSP00000434655 | 3.14E-06 | -0.2257 | 0 | 0.108 |
| ENSP00000351155 | 4.92E-06 | -0.2257 | 0 | 0.085 |
| ENSP00000380702 | 1.98E-06 | -0.2258 | 0 | 0.317 |
| ENSP00000321242 | 8.55E-06 | -0.2259 | 0 | 0.167 |
| ENSP00000367486 | 5.34E-06 | -0.2260 | 0 | 0.097 |
| ENSP00000415434 | 2.69E-06 | -0.2260 | 0 | 0.160 |
| ENSP00000297819 | 4.89E-06 | -0.2260 | 0 | 0.183 |
| ENSP00000366342 | 1.19E-06 | -0.2260 | 0 | 0.000 |
| ENSP00000258180 | 1.46E-05 | -0.2261 | 152 | 0.070 |
| ENSP00000357167 | 9.24E-06 | -0.2261 | 0 | 0.119 |
| ENSP00000397773 | 2.60E-06 | -0.2262 | 0 | 0.106 |
| ENSP00000355880 | 2.86E-06 | -0.2262 | 0 | 0.050 |
| ENSP00000350704 | 4.07E-06 | -0.2262 | 0 | 0.130 |
| ENSP00000369465 | 7.47E-06 | -0.2263 | 900 | 0.143 |
| ENSP00000349543 | 3.90E-06 | -0.2263 | 0 | 0.183 |
| ENSP00000377862 | 1.59E-05 | -0.2263 | 173 | 0.743 |
| ENSP00000338171 | 9.18E-06 | -0.2263 | 0 | 0.389 |
| ENSP00000239451 | 5.69E-06 | -0.2263 | 0 | 0.074 |
| ENSP00000368699 | 3.47E-05 | -0.2263 | 366 | 0.675 |
| ENSP00000353699 | 3.49E-06 | -0.2263 | 0 | 0.097 |
| ENSP00000347277 | 1.88E-06 | -0.2263 | 0 | 0.370 |
| ENSP00000450337 | 2.33E-06 | -0.2263 | 0 | 0.099 |
| ENSP00000426906 | 4.06E-06 | -0.2263 | 158 | 0.000 |
| ENSP00000259988 | 7.16E-06 | -0.2263 | 0 | 0.000 |
| ENSP00000348849 | 5.64E-06 | -0.2264 | 198 | 0.781 |
| ENSP00000354782 | 8.51E-06 | -0.2264 | 195 | 0.743 |
| ENSP00000364567 | 3.35E-06 | -0.2264 | 0 | 0.145 |
| ENSP00000456927 | 1.88E-06 | -0.2264 | 0 | 0.153 |
| ENSP00000412178 | 3.05E-06 | -0.2265 | 0 | 0.145 |
| ENSP00000420487 | 4.36E-06 | -0.2265 | 0 | 0.271 |
| ENSP00000407193 | 4.50E-06 | -0.2265 | 0 | 0.082 |
| ENSP00000426234 | 2.90E-06 | -0.2265 | 0 | 0.000 |
| ENSP00000433433 | 3.91E-06 | -0.2266 | 0 | 0.194 |

| | | | | |
|---|---|---|---|---|
| ENSP00000402698 | 3.10E-06 | -0.2266 | 0 | 0.000 |
| ENSP00000450244 | 3.45E-06 | -0.2267 | 0 | 0.414 |
| ENSP00000470381 | 3.05E-06 | -0.2267 | 0 | 0.702 |
| ENSP00000353259 | 4.23E-06 | -0.2268 | 0 | 0.115 |
| ENSP00000335384 | 4.77E-06 | -0.2268 | 0 | 0.092 |
| ENSP00000383295 | 2.87E-06 | -0.2268 | 0 | 0.078 |
| ENSP00000348554 | 1.65E-05 | -0.2268 | 0 | 0.505 |
| ENSP00000324274 | 3.63E-06 | -0.2269 | 0 | 0.217 |
| ENSP00000330877 | 1.03E-05 | -0.2269 | 0 | 0.000 |
| ENSP00000333627 | 6.32E-06 | -0.2269 | 0 | 0.000 |
| ENSP00000305766 | 1.29E-05 | -0.2270 | 181 | 0.136 |
| ENSP00000356230 | 5.27E-06 | -0.2270 | 0 | 0.386 |
| ENSP00000355932 | 3.46E-06 | -0.2271 | 0 | 0.199 |
| ENSP00000356436 | 9.78E-06 | -0.2271 | 597 | 0.523 |
| ENSP00000343087 | 3.43E-06 | -0.2271 | 0 | 0.102 |
| ENSP00000238044 | 3.93E-06 | -0.2272 | 0 | 0.089 |
| ENSP00000419786 | 4.63E-06 | -0.2272 | 0 | 0.135 |
| ENSP00000333737 | 4.93E-06 | -0.2272 | 0 | 0.100 |
| ENSP00000354152 | 4.93E-06 | -0.2273 | 200 | 0.170 |
| ENSP00000268595 | 1.20E-05 | -0.2273 | 0 | 0.115 |
| ENSP00000381436 | 3.33E-06 | -0.2273 | 0 | 0.133 |
| ENSP00000373808 | 4.02E-06 | -0.2273 | 0 | 0.154 |
| ENSP00000360323 | 1.98E-06 | -0.2273 | 0 | 0.155 |
| ENSP00000368411 | 3.92E-06 | -0.2273 | 0 | 0.148 |
| ENSP00000396160 | 1.35E-06 | -0.2273 | 0 | 0.225 |
| ENSP00000302297 | 6.54E-06 | -0.2274 | 0 | 0.147 |
| ENSP00000301831 | 7.91E-06 | -0.2274 | 193 | 0.139 |
| ENSP00000344545 | 3.09E-06 | -0.2274 | 0 | 0.123 |
| ENSP00000318445 | 1.41E-05 | -0.2274 | 900 | 0.000 |
| ENSP00000380942 | 1.43E-05 | -0.2275 | 205 | 0.696 |
| ENSP00000358798 | 6.92E-06 | -0.2275 | 0 | 0.149 |
| ENSP00000330375 | 9.30E-06 | -0.2275 | 198 | 0.207 |
| ENSP00000304331 | 4.59E-06 | -0.2276 | 0 | 0.799 |
| ENSP00000381594 | 3.17E-06 | -0.2276 | 0 | 0.123 |
| ENSP00000336887 | 3.33E-06 | -0.2276 | 0 | 0.084 |
| ENSP00000016913 | 1.24E-05 | -0.2277 | 0 | 0.216 |
| ENSP00000359050 | 3.09E-06 | -0.2277 | 0 | 0.110 |
| ENSP00000334044 | 1.71E-05 | -0.2277 | 295 | 0.653 |
| ENSP00000382250 | 5.44E-06 | -0.2277 | 340 | 0.640 |
| ENSP00000355028 | 6.89E-06 | -0.2278 | 0 | 0.322 |
| ENSP00000295992 | 6.60E-06 | -0.2278 | 0 | 0.141 |
| ENSP00000323872 | 5.16E-06 | -0.2278 | 0 | 0.096 |
| ENSP00000445859 | 6.05E-06 | -0.2279 | 154 | 0.113 |
| ENSP00000219301 | 1.46E-05 | -0.2279 | 0 | 0.139 |
| ENSP00000358430 | 1.15E-05 | -0.2280 | 292 | 0.113 |
| ENSP00000369959 | 2.45E-06 | -0.2280 | 0 | 0.201 |

| | | | | |
|---|---|---|---|---|
| ENSP00000319062 | 1.08E-05 | -0.2280 | 345 | 0.260 |
| ENSP00000278520 | 3.51E-06 | -0.2281 | 0 | 0.181 |
| ENSP00000380494 | 9.77E-06 | -0.2281 | 197 | 0.000 |
| ENSP00000368981 | 3.12E-06 | -0.2281 | 0 | 0.057 |
| ENSP00000349456 | 5.13E-06 | -0.2281 | 0 | 0.118 |
| ENSP00000310031 | 8.27E-06 | -0.2281 | 0 | 0.000 |
| ENSP00000378897 | 3.06E-06 | -0.2281 | 0 | 0.071 |
| ENSP00000388275 | 4.59E-06 | -0.2282 | 0 | 0.133 |
| ENSP00000351520 | 4.58E-06 | -0.2282 | 0 | 0.099 |
| ENSP00000377796 | 3.73E-06 | -0.2282 | 0 | 0.088 |
| ENSP00000353203 | 6.45E-06 | -0.2282 | 0 | 0.116 |
| ENSP00000370761 | 4.56E-06 | -0.2282 | 0 | 0.161 |
| ENSP00000422644 | 3.10E-06 | -0.2282 | 0 | 0.086 |
| ENSP00000332455 | 1.74E-05 | -0.2282 | 183 | 0.726 |
| ENSP00000342169 | 4.13E-06 | -0.2282 | 0 | 0.115 |
| ENSP00000232744 | 1.55E-05 | -0.2283 | 201 | 0.639 |
| ENSP00000226460 | 1.04E-05 | -0.2283 | 0 | 0.118 |
| ENSP00000376553 | 5.01E-06 | -0.2283 | 0 | 0.108 |
| ENSP00000377910 | 4.72E-06 | -0.2283 | 0 | 0.000 |
| ENSP00000357823 | 2.35E-06 | -0.2284 | 0 | 0.498 |
| ENSP00000329137 | 5.92E-06 | -0.2284 | 0 | 0.075 |
| ENSP00000338648 | 1.52E-05 | -0.2285 | 240 | 0.155 |
| ENSP00000354632 | 7.25E-06 | -0.2285 | 244 | 0.604 |
| ENSP00000361170 | 5.13E-06 | -0.2285 | 198 | 0.604 |
| ENSP00000373937 | 3.87E-06 | -0.2286 | 0 | 0.310 |
| ENSP00000357292 | 9.60E-06 | -0.2286 | 631 | 0.721 |
| ENSP00000302046 | 9.43E-06 | -0.2286 | 0 | 0.109 |
| ENSP00000378288 | 1.32E-05 | -0.2286 | 167 | 0.657 |
| ENSP00000355827 | 1.76E-06 | -0.2286 | 0 | 0.176 |
| ENSP00000412060 | 1.69E-06 | -0.2287 | 0 | 0.089 |
| ENSP00000361616 | 2.89E-06 | -0.2287 | 0 | 0.093 |
| ENSP00000339484 | 1.65E-05 | -0.2287 | 318 | 0.659 |
| ENSP00000358955 | 3.35E-06 | -0.2287 | 0 | 0.153 |
| ENSP00000364126 | 5.56E-06 | -0.2288 | 156 | 0.441 |
| ENSP00000365175 | 3.76E-06 | -0.2288 | 0 | 0.230 |
| ENSP00000434385 | 4.89E-06 | -0.2288 | 0 | 0.312 |
| ENSP00000192314 | 1.15E-05 | -0.2288 | 0 | 0.102 |
| ENSP00000367848 | 3.70E-06 | -0.2288 | 0 | 0.320 |
| ENSP00000281416 | 3.61E-06 | -0.2289 | 0 | 0.078 |
| ENSP00000350402 | 8.89E-06 | -0.2289 | 0 | 0.089 |
| ENSP00000386121 | 3.89E-06 | -0.2289 | 0 | 0.082 |
| ENSP00000349891 | 7.96E-06 | -0.2289 | 202 | 0.674 |
| ENSP00000345571 | 4.94E-06 | -0.2289 | 0 | 0.792 |
| ENSP00000436500 | 1.93E-06 | -0.2290 | 0 | 0.111 |
| ENSP00000369042 | 9.88E-06 | -0.2290 | 418 | 0.000 |
| ENSP00000430271 | 3.04E-06 | -0.2291 | 0 | 0.336 |

| | | | | |
|---|---|---|---|---|
| ENSP00000303042 | 3.63E-06 | -0.2291 | 0 | 0.069 |
| ENSP00000371054 | 3.36E-06 | -0.2291 | 0 | 0.146 |
| ENSP00000398177 | 6.76E-06 | -0.2292 | 227 | 0.000 |
| ENSP00000347324 | 6.83E-06 | -0.2292 | 189 | 0.060 |
| ENSP00000385741 | 2.17E-06 | -0.2293 | 0 | 0.149 |
| ENSP00000404179 | 4.14E-06 | -0.2294 | 0 | 0.404 |
| ENSP00000349205 | 7.91E-06 | -0.2294 | 0 | 0.257 |
| ENSP00000225550 | 4.45E-06 | -0.2294 | 0 | 0.151 |
| ENSP00000350844 | 1.87E-05 | -0.2294 | 212 | 0.867 |
| ENSP00000340776 | 2.27E-06 | -0.2294 | 0 | 0.128 |
| ENSP00000379888 | 5.59E-06 | -0.2295 | 214 | 0.912 |
| ENSP00000307461 | 7.32E-06 | -0.2295 | 0 | 0.085 |
| ENSP00000364464 | 1.28E-05 | -0.2295 | 271 | 0.281 |
| ENSP00000335040 | 6.69E-06 | -0.2296 | 0 | 0.142 |
| ENSP00000382392 | 1.66E-05 | -0.2296 | 325 | 0.900 |
| ENSP00000302648 | 1.51E-05 | -0.2296 | 187 | 0.630 |
| ENSP00000328307 | 4.44E-06 | -0.2296 | 0 | 0.153 |
| ENSP00000362682 | 3.70E-06 | -0.2296 | 0 | 0.142 |
| ENSP00000400099 | 1.68E-06 | -0.2297 | 0 | 0.124 |
| ENSP00000440765 | 1.64E-06 | -0.2297 | 0 | 0.117 |
| ENSP00000321962 | 8.66E-06 | -0.2297 | 0 | 0.122 |
| ENSP00000358541 | 3.65E-06 | -0.2298 | 0 | 0.163 |
| ENSP00000414202 | 3.13E-06 | -0.2298 | 0 | 0.296 |
| ENSP00000401980 | 3.97E-06 | -0.2298 | 0 | 0.634 |
| ENSP00000355675 | 5.28E-06 | -0.2298 | 0 | 0.317 |
| ENSP00000310814 | 4.69E-06 | -0.2298 | 0 | 0.098 |
| ENSP00000331625 | 5.96E-06 | -0.2299 | 0 | 0.000 |
| ENSP00000364815 | 1.52E-05 | -0.2299 | 588 | 0.694 |
| ENSP00000335565 | 2.98E-06 | -0.2299 | 0 | 0.083 |
| ENSP00000436049 | 3.44E-06 | -0.2300 | 0 | 0.788 |
| ENSP00000355587 | 3.29E-06 | -0.2300 | 0 | 0.270 |
| ENSP00000375248 | 1.31E-06 | -0.2300 | 0 | 0.370 |
| ENSP00000328998 | 5.13E-06 | -0.2301 | 0 | 0.068 |
| ENSP00000355991 | 5.21E-06 | -0.2301 | 0 | 0.163 |
| ENSP00000355614 | 4.51E-06 | -0.2302 | 0 | 0.131 |
| ENSP00000357588 | 2.46E-06 | -0.2302 | 0 | 0.150 |
| ENSP00000356058 | 7.48E-06 | -0.2302 | 0 | 0.000 |
| ENSP00000400376 | 1.37E-05 | -0.2302 | 675 | 0.412 |
| ENSP00000354774 | 5.57E-06 | -0.2303 | 0 | 0.106 |
| ENSP00000454591 | 1.39E-06 | -0.2303 | 0 | 0.000 |
| ENSP00000297107 | 4.28E-06 | -0.2303 | 0 | 0.136 |
| ENSP00000264819 | 4.15E-06 | -0.2304 | 0 | 0.136 |
| ENSP00000313088 | 4.61E-06 | -0.2304 | 0 | 0.076 |
| ENSP00000407763 | 2.60E-06 | -0.2304 | 0 | 0.171 |
| ENSP00000386348 | 5.01E-06 | -0.2304 | 0 | 0.136 |
| ENSP00000472710 | 1.78E-06 | -0.2305 | 0 | 0.000 |

| | | | | |
|---|---|---|---|---|
| ENSP00000345216 | 3.93E-06 | -0.2306 | 0 | 0.074 |
| ENSP00000366690 | 2.52E-06 | -0.2306 | 0 | 0.083 |
| ENSP00000357480 | 4.75E-06 | -0.2306 | 0 | 0.373 |
| ENSP00000351737 | 1.79E-06 | -0.2307 | 0 | 0.107 |
| ENSP00000454303 | 2.89E-06 | -0.2307 | 0 | 0.359 |
| ENSP00000417176 | 4.80E-06 | -0.2307 | 0 | 0.236 |
| ENSP00000359098 | 1.84E-06 | -0.2307 | 0 | 0.128 |
| ENSP00000368732 | 2.79E-06 | -0.2308 | 0 | 0.000 |
| ENSP00000390500 | 8.11E-06 | -0.2308 | 520 | 0.653 |
| ENSP00000414237 | 1.99E-06 | -0.2308 | 0 | 0.140 |
| ENSP00000343348 | 3.74E-06 | -0.2308 | 0 | 0.098 |
| ENSP00000344866 | 3.26E-06 | -0.2309 | 0 | 0.122 |
| ENSP00000398391 | 3.94E-06 | -0.2309 | 0 | 0.000 |
| ENSP00000296657 | 3.80E-06 | -0.2310 | 0 | 0.167 |
| ENSP00000321038 | 4.76E-06 | -0.2311 | 0 | 0.129 |
| ENSP00000369863 | 3.00E-06 | -0.2311 | 0 | 0.122 |
| ENSP00000377265 | 5.42E-06 | -0.2312 | 0 | 0.752 |
| ENSP00000370215 | 2.78E-06 | -0.2312 | 0 | 0.373 |
| ENSP00000304032 | 4.47E-06 | -0.2312 | 0 | 0.124 |
| ENSP00000356444 | 3.66E-06 | -0.2312 | 0 | 0.164 |
| ENSP00000352319 | 5.36E-06 | -0.2313 | 0 | 0.116 |
| ENSP00000371451 | 4.98E-06 | -0.2313 | 329 | 0.276 |
| ENSP00000450924 | 3.60E-06 | -0.2313 | 0 | 0.000 |
| ENSP00000356071 | 4.62E-06 | -0.2314 | 175 | 0.080 |
| ENSP00000400895 | 3.54E-06 | -0.2314 | 0 | 0.099 |
| ENSP00000441823 | 4.29E-06 | -0.2314 | 0 | 0.743 |
| ENSP00000399696 | 6.90E-06 | -0.2315 | 0 | 0.000 |
| ENSP00000347802 | 7.76E-06 | -0.2316 | 201 | 0.614 |
| ENSP00000361276 | 3.61E-06 | -0.2316 | 0 | 0.115 |
| ENSP00000351318 | 4.81E-06 | -0.2317 | 0 | 0.111 |
| ENSP00000251691 | 4.47E-06 | -0.2317 | 0 | 0.000 |
| ENSP00000388431 | 4.93E-06 | -0.2317 | 0 | 0.123 |
| ENSP00000372576 | 3.72E-06 | -0.2317 | 0 | 0.110 |
| ENSP00000332823 | 1.35E-05 | -0.2317 | 0 | 0.666 |
| ENSP00000365830 | 8.30E-06 | -0.2317 | 202 | 0.644 |
| ENSP00000371505 | 4.93E-06 | -0.2318 | 0 | 0.403 |
| ENSP00000353270 | 3.32E-06 | -0.2318 | 0 | 0.103 |
| ENSP00000355162 | 2.64E-06 | -0.2318 | 0 | 0.156 |
| ENSP00000373964 | 1.55E-05 | -0.2318 | 338 | 0.382 |
| ENSP00000256039 | 1.16E-05 | -0.2318 | 0 | 0.165 |
| ENSP00000354086 | 3.30E-06 | -0.2319 | 0 | 0.228 |
| ENSP00000349124 | 3.60E-06 | -0.2321 | 0 | 0.241 |
| ENSP00000303779 | 1.02E-05 | -0.2321 | 0 | 0.000 |
| ENSP00000324763 | 1.49E-05 | -0.2321 | 163 | 0.092 |
| ENSP00000315070 | 5.96E-06 | -0.2321 | 0 | 0.106 |
| ENSP00000373300 | 3.22E-06 | -0.2321 | 0 | 0.063 |

| | | | | |
|---|---|---|---|---|
| ENSP00000264968 | 7.22E-06 | -0.2322 | 0 | 0.100 |
| ENSP00000330269 | 5.48E-06 | -0.2322 | 0 | 0.119 |
| ENSP00000459356 | 1.20E-06 | -0.2322 | 0 | 0.000 |
| ENSP00000383226 | 1.81E-06 | -0.2322 | 0 | 0.108 |
| ENSP00000396068 | 3.81E-06 | -0.2322 | 0 | 0.203 |
| ENSP00000385903 | 1.90E-06 | -0.2322 | 0 | 0.088 |
| ENSP00000365844 | 3.50E-06 | -0.2323 | 0 | 0.115 |
| ENSP00000362836 | 3.56E-06 | -0.2323 | 0 | 0.135 |
| ENSP00000414390 | 5.09E-06 | -0.2324 | 0 | 0.112 |
| ENSP00000357892 | 7.17E-06 | -0.2324 | 0 | 0.131 |
| ENSP00000358563 | 1.37E-05 | -0.2324 | 443 | 0.907 |
| ENSP00000370719 | 1.34E-05 | -0.2325 | 199 | 0.579 |
| ENSP00000416050 | 3.67E-06 | -0.2325 | 0 | 0.118 |
| ENSP00000331086 | 5.52E-06 | -0.2325 | 0 | 0.107 |
| ENSP00000367125 | 4.28E-06 | -0.2326 | 0 | 0.133 |
| ENSP00000359174 | 4.85E-06 | -0.2326 | 0 | 0.059 |
| ENSP00000376776 | 7.81E-06 | -0.2326 | 195 | 0.622 |
| ENSP00000452844 | 1.05E-06 | -0.2326 | 0 | 0.000 |
| ENSP00000263284 | 2.61E-06 | -0.2326 | 0 | 0.000 |
| ENSP00000369473 | 6.23E-06 | -0.2326 | 0 | 0.099 |
| ENSP00000397911 | 2.06E-06 | -0.2326 | 0 | 0.373 |
| ENSP00000440374 | 9.21E-06 | -0.2326 | 150 | 0.147 |
| ENSP00000232519 | 5.14E-06 | -0.2327 | 0 | 0.114 |
| ENSP00000264257 | 1.08E-05 | -0.2327 | 0 | 0.217 |
| ENSP00000308873 | 6.38E-06 | -0.2327 | 0 | 0.115 |
| ENSP00000275988 | 9.99E-06 | -0.2327 | 0 | 0.322 |
| ENSP00000357768 | 4.34E-06 | -0.2328 | 0 | 0.134 |
| ENSP00000359115 | 1.44E-06 | -0.2329 | 0 | 0.140 |
| ENSP00000312272 | 2.43E-06 | -0.2329 | 0 | 0.154 |
| ENSP00000382269 | 4.55E-06 | -0.2329 | 0 | 0.434 |
| ENSP00000371388 | 3.13E-06 | -0.2330 | 0 | 0.098 |
| ENSP00000365486 | 4.99E-06 | -0.2330 | 0 | 0.089 |
| ENSP00000389543 | 2.82E-06 | -0.2330 | 0 | 0.000 |
| ENSP00000331298 | 7.43E-06 | -0.2331 | 0 | 0.117 |
| ENSP00000379203 | 2.87E-06 | -0.2331 | 0 | 0.120 |
| ENSP00000344420 | 7.41E-06 | -0.2331 | 0 | 0.197 |
| ENSP00000301272 | 8.75E-06 | -0.2332 | 0 | 0.190 |
| ENSP00000384198 | 3.10E-06 | -0.2332 | 0 | 0.435 |
| ENSP00000469716 | 1.80E-06 | -0.2333 | 0 | 0.000 |
| ENSP00000373420 | 1.65E-06 | -0.2333 | 0 | 0.097 |
| ENSP00000338228 | 5.15E-06 | -0.2333 | 0 | 0.074 |
| ENSP00000375387 | 5.29E-06 | -0.2333 | 0 | 0.000 |
| ENSP00000442046 | 1.56E-05 | -0.2333 | 800 | 0.703 |
| ENSP00000396627 | 7.28E-06 | -0.2334 | 0 | 0.000 |
| ENSP00000272521 | 2.48E-06 | -0.2334 | 0 | 0.159 |
| ENSP00000334798 | 2.27E-06 | -0.2334 | 0 | 0.147 |

| | | | | |
|---|---|---|---|---|
| ENSP00000453573 | 1.02E-06 | -0.2334 | 0 | 0.000 |
| ENSP00000379500 | 1.05E-05 | -0.2335 | 329 | 0.652 |
| ENSP00000448219 | 2.18E-06 | -0.2335 | 0 | 0.112 |
| ENSP00000378324 | 8.09E-06 | -0.2335 | 934 | 0.440 |
| ENSP00000390849 | 7.42E-06 | -0.2336 | 175 | 0.409 |
| ENSP00000417656 | 2.15E-06 | -0.2336 | 0 | 0.162 |
| ENSP00000308976 | 6.25E-06 | -0.2336 | 0 | 0.068 |
| ENSP00000416558 | 2.79E-06 | -0.2336 | 0 | 0.161 |
| ENSP00000420616 | 3.18E-06 | -0.2336 | 0 | 0.806 |
| ENSP00000409267 | 1.73E-05 | -0.2337 | 153 | 0.000 |
| ENSP00000331746 | 1.46E-05 | -0.2337 | 0 | 0.573 |
| ENSP00000359305 | 2.30E-06 | -0.2337 | 0 | 0.124 |
| ENSP00000368612 | 2.02E-06 | -0.2337 | 0 | 0.085 |
| ENSP00000363317 | 1.09E-05 | -0.2337 | 272 | 0.305 |
| ENSP00000320634 | 1.17E-05 | -0.2338 | 173 | 0.051 |
| ENSP00000426528 | 4.05E-06 | -0.2338 | 0 | 0.081 |
| ENSP00000362991 | 3.21E-06 | -0.2338 | 0 | 0.069 |
| ENSP00000403117 | 5.28E-06 | -0.2338 | 0 | 0.036 |
| ENSP00000351856 | 7.70E-06 | -0.2339 | 201 | 0.608 |
| ENSP00000221954 | 1.51E-05 | -0.2339 | 0 | 0.198 |
| ENSP00000420714 | 4.16E-06 | -0.2340 | 0 | 0.639 |
| ENSP00000311184 | 7.42E-06 | -0.2340 | 0 | 0.106 |
| ENSP00000399637 | 1.16E-06 | -0.2340 | 0 | 0.000 |
| ENSP00000413496 | 1.13E-05 | -0.2340 | 157 | 0.898 |
| ENSP00000407323 | 1.38E-05 | -0.2340 | 330 | 0.913 |
| ENSP00000305596 | 3.13E-06 | -0.2340 | 0 | 0.135 |
| ENSP00000431603 | 5.69E-06 | -0.2340 | 0 | 0.771 |
| ENSP00000360782 | 7.17E-06 | -0.2341 | 198 | 0.348 |
| ENSP00000306459 | 4.37E-06 | -0.2341 | 0 | 0.073 |
| ENSP00000350874 | 3.13E-06 | -0.2341 | 0 | 0.084 |
| ENSP00000331643 | 4.16E-06 | -0.2342 | 0 | 0.134 |
| ENSP00000296121 | 2.20E-06 | -0.2342 | 0 | 0.159 |
| ENSP00000468308 | 3.76E-06 | -0.2343 | 0 | 0.123 |
| ENSP00000354313 | 3.99E-06 | -0.2343 | 0 | 0.100 |
| ENSP00000254250 | 5.20E-06 | -0.2344 | 0 | 0.343 |
| ENSP00000360065 | 4.27E-06 | -0.2345 | 0 | 0.118 |
| ENSP00000346954 | 3.79E-06 | -0.2345 | 0 | 0.142 |
| ENSP00000354583 | 2.71E-06 | -0.2346 | 0 | 0.122 |
| ENSP00000259392 | 1.13E-05 | -0.2346 | 0 | 0.078 |
| ENSP00000435342 | 2.54E-06 | -0.2346 | 0 | 0.303 |
| ENSP00000377369 | 1.67E-06 | -0.2346 | 0 | 0.000 |
| ENSP00000418194 | 6.00E-06 | -0.2347 | 0 | 0.224 |
| ENSP00000328118 | 1.29E-05 | -0.2347 | 318 | 0.317 |
| ENSP00000401770 | 3.13E-06 | -0.2347 | 0 | 0.092 |
| ENSP00000390630 | 1.62E-06 | -0.2347 | 0 | 0.110 |
| ENSP00000397394 | 2.36E-06 | -0.2348 | 0 | 0.131 |

| | | | | |
|---|---|---|---|---|
| ENSP00000389009 | 4.77E-06 | -0.2348 | 0 | 0.417 |
| ENSP00000378812 | 3.68E-06 | -0.2348 | 0 | 0.150 |
| ENSP00000360797 | 1.17E-05 | -0.2348 | 256 | 0.702 |
| ENSP00000425955 | 1.97E-06 | -0.2348 | 0 | 0.601 |
| ENSP00000380858 | 4.64E-06 | -0.2348 | 0 | 0.000 |
| ENSP00000360676 | 3.94E-06 | -0.2349 | 0 | 0.249 |
| ENSP00000316845 | 6.67E-06 | -0.2349 | 0 | 0.631 |
| ENSP00000375087 | 1.63E-06 | -0.2349 | 0 | 0.142 |
| ENSP00000375220 | 1.88E-06 | -0.2349 | 0 | 0.160 |
| ENSP00000363338 | 1.28E-05 | -0.2349 | 209 | 0.351 |
| ENSP00000363394 | 6.19E-06 | -0.2350 | 0 | 0.083 |
| ENSP00000373586 | 1.91E-06 | -0.2350 | 0 | 0.139 |
| ENSP00000351132 | 4.46E-06 | -0.2351 | 0 | 0.196 |
| ENSP00000367029 | 3.40E-06 | -0.2352 | 0 | 0.834 |
| ENSP00000356256 | 6.42E-06 | -0.2352 | 231 | 0.197 |
| ENSP00000300175 | 7.82E-06 | -0.2352 | 0 | 0.294 |
| ENSP00000374565 | 3.52E-06 | -0.2352 | 0 | 0.108 |
| ENSP00000385899 | 6.32E-06 | -0.2352 | 0 | 0.108 |
| ENSP00000347301 | 2.04E-06 | -0.2352 | 0 | 0.142 |
| ENSP00000383672 | 9.13E-06 | -0.2352 | 214 | 0.000 |
| ENSP00000427264 | 1.96E-06 | -0.2352 | 0 | 0.597 |
| ENSP00000423503 | 1.96E-06 | -0.2352 | 0 | 0.597 |
| ENSP00000425582 | 1.96E-06 | -0.2352 | 0 | 0.597 |
| ENSP00000463847 | 4.14E-06 | -0.2353 | 297 | 0.837 |
| ENSP00000457904 | 9.03E-07 | -0.2353 | 0 | 0.160 |
| ENSP00000366565 | 3.25E-06 | -0.2354 | 0 | 0.092 |
| ENSP00000427926 | 2.23E-05 | -0.2354 | 204 | 0.567 |
| ENSP00000360498 | 3.54E-06 | -0.2354 | 0 | 0.292 |
| ENSP00000284776 | 3.86E-06 | -0.2354 | 0 | 0.201 |
| ENSP00000345849 | 1.10E-05 | -0.2355 | 254 | 0.063 |
| ENSP00000351790 | 4.05E-06 | -0.2355 | 0 | 0.318 |
| ENSP00000376421 | 5.19E-06 | -0.2355 | 0 | 0.184 |
| ENSP00000354929 | 3.93E-06 | -0.2355 | 0 | 0.069 |
| ENSP00000440190 | 2.48E-06 | -0.2355 | 0 | 0.657 |
| ENSP00000407552 | 8.83E-06 | -0.2355 | 177 | 0.456 |
| ENSP00000344095 | 2.52E-06 | -0.2355 | 0 | 0.149 |
| ENSP00000422621 | 1.96E-06 | -0.2356 | 0 | 0.597 |
| ENSP00000422216 | 1.96E-06 | -0.2356 | 0 | 0.597 |
| ENSP00000379802 | 6.42E-06 | -0.2356 | 0 | 0.182 |
| ENSP00000366144 | 3.56E-06 | -0.2356 | 0 | 0.600 |
| ENSP00000411409 | 3.59E-06 | -0.2356 | 0 | 0.297 |
| ENSP00000406209 | 3.77E-06 | -0.2356 | 0 | 0.289 |
| ENSP00000250366 | 8.59E-06 | -0.2356 | 0 | 0.000 |
| ENSP00000359892 | 6.82E-06 | -0.2356 | 0 | 0.110 |
| ENSP00000368496 | 4.58E-06 | -0.2357 | 0 | 0.126 |
| ENSP00000340568 | 5.72E-06 | -0.2357 | 0 | 0.178 |

| | | | | |
|---|---|---|---|---|
| ENSP00000355529 | 4.90E-06 | -0.2357 | 0 | 0.082 |
| ENSP00000457733 | 5.55E-06 | -0.2358 | 0 | 0.130 |
| ENSP00000283946 | 4.64E-06 | -0.2358 | 0 | 0.171 |
| ENSP00000428765 | 9.85E-06 | -0.2358 | 0 | 0.063 |
| ENSP00000311648 | 4.66E-06 | -0.2359 | 0 | 0.043 |
| ENSP00000386609 | 2.93E-06 | -0.2359 | 0 | 0.073 |
| ENSP00000373411 | 2.29E-06 | -0.2359 | 0 | 0.134 |
| ENSP00000392936 | 2.62E-06 | -0.2359 | 0 | 0.432 |
| ENSP00000370003 | 9.71E-06 | -0.2361 | 198 | 0.617 |
| ENSP00000343331 | 5.41E-06 | -0.2361 | 0 | 0.161 |
| ENSP00000383392 | 1.93E-06 | -0.2362 | 0 | 0.150 |
| ENSP00000346874 | 4.08E-06 | -0.2362 | 0 | 0.074 |
| ENSP00000311153 | 2.90E-06 | -0.2362 | 0 | 0.000 |
| ENSP00000370744 | 9.83E-06 | -0.2362 | 313 | 0.532 |
| ENSP00000331500 | 7.29E-06 | -0.2363 | 0 | 0.091 |
| ENSP00000359127 | 4.80E-06 | -0.2363 | 174 | 0.787 |
| ENSP00000296882 | 3.86E-06 | -0.2364 | 0 | 0.106 |
| ENSP00000374562 | 2.11E-06 | -0.2364 | 0 | 0.156 |
| ENSP00000414922 | 5.00E-06 | -0.2364 | 0 | 0.150 |
| ENSP00000289373 | 8.84E-06 | -0.2365 | 0 | 0.195 |
| ENSP00000356121 | 1.74E-06 | -0.2365 | 0 | 0.138 |
| ENSP00000361776 | 4.95E-06 | -0.2365 | 0 | 0.182 |
| ENSP00000430269 | 1.33E-05 | -0.2365 | 293 | 0.344 |
| ENSP00000463094 | 3.07E-06 | -0.2366 | 0 | 0.086 |
| ENSP00000416289 | 3.48E-06 | -0.2367 | 0 | 0.121 |
| ENSP00000327889 | 3.50E-06 | -0.2367 | 0 | 0.188 |
| ENSP00000366234 | 6.36E-06 | -0.2367 | 0 | 0.529 |
| ENSP00000341128 | 3.71E-06 | -0.2368 | 0 | 0.097 |
| ENSP00000359520 | 3.09E-06 | -0.2369 | 0 | 0.078 |
| ENSP00000378784 | 3.70E-06 | -0.2369 | 0 | 0.815 |
| ENSP00000357218 | 5.65E-06 | -0.2369 | 192 | 0.253 |
| ENSP00000321360 | 7.16E-06 | -0.2370 | 0 | 0.157 |
| ENSP00000375956 | 7.04E-06 | -0.2370 | 0 | 0.841 |
| ENSP00000356272 | 2.63E-06 | -0.2370 | 0 | 0.120 |
| ENSP00000363522 | 1.96E-06 | -0.2370 | 0 | 0.099 |
| ENSP00000349970 | 3.08E-06 | -0.2370 | 0 | 0.118 |
| ENSP00000305334 | 6.45E-06 | -0.2371 | 0 | 0.116 |
| ENSP00000332062 | 5.28E-06 | -0.2371 | 0 | 0.082 |
| ENSP00000322108 | 4.25E-06 | -0.2371 | 0 | 0.112 |
| ENSP00000355470 | 6.59E-06 | -0.2371 | 0 | 0.263 |
| ENSP00000453042 | 3.60E-06 | -0.2371 | 0 | 0.000 |
| ENSP00000280571 | 1.10E-05 | -0.2371 | 0 | 0.109 |
| ENSP00000462795 | 3.52E-06 | -0.2372 | 0 | 0.000 |
| ENSP00000423665 | 2.42E-05 | -0.2372 | 221 | 0.000 |
| ENSP00000376403 | 4.01E-06 | -0.2372 | 0 | 0.110 |
| ENSP00000317595 | 5.03E-06 | -0.2372 | 0 | 0.122 |

| | | | | |
|---|---|---|---|---|
| ENSP00000333358 | 4.27E-06 | -0.2373 | 0 | 0.105 |
| ENSP00000360045 | 3.67E-06 | -0.2373 | 0 | 0.122 |
| ENSP00000374390 | 1.40E-05 | -0.2374 | 330 | 0.914 |
| ENSP00000369899 | 4.22E-06 | -0.2375 | 0 | 0.457 |
| ENSP00000387365 | 1.41E-05 | -0.2375 | 591 | 0.877 |
| ENSP00000361005 | 5.10E-06 | -0.2375 | 0 | 0.174 |
| ENSP00000286349 | 3.23E-06 | -0.2375 | 0 | 0.095 |
| ENSP00000322832 | 9.76E-06 | -0.2376 | 0 | 0.140 |
| ENSP00000343279 | 4.23E-06 | -0.2376 | 0 | 0.263 |
| ENSP00000403459 | 9.16E-06 | -0.2376 | 0 | 0.805 |
| ENSP00000318674 | 5.24E-06 | -0.2377 | 0 | 0.181 |
| ENSP00000328069 | 8.41E-06 | -0.2377 | 0 | 0.109 |
| ENSP00000449334 | 1.49E-06 | -0.2377 | 0 | 0.000 |
| ENSP00000449223 | 1.49E-06 | -0.2377 | 0 | 0.000 |
| ENSP00000357036 | 2.65E-06 | -0.2377 | 0 | 0.267 |
| ENSP00000388550 | 3.86E-06 | -0.2377 | 0 | 0.239 |
| ENSP00000364515 | 1.68E-06 | -0.2377 | 0 | 0.095 |
| ENSP00000382770 | 1.34E-05 | -0.2377 | 701 | 0.243 |
| ENSP00000291672 | 3.58E-06 | -0.2377 | 0 | 0.139 |
| ENSP00000362195 | 5.33E-06 | -0.2378 | 0 | 0.790 |
| ENSP00000365678 | 5.28E-06 | -0.2378 | 0 | 0.085 |
| ENSP00000351813 | 3.67E-06 | -0.2378 | 0 | 0.094 |
| ENSP00000352904 | 2.36E-06 | -0.2378 | 0 | 0.104 |
| ENSP00000326018 | 1.12E-05 | -0.2378 | 0 | 0.106 |
| ENSP00000307801 | 5.40E-06 | -0.2378 | 0 | 0.128 |
| ENSP00000438970 | 7.12E-07 | -0.2379 | 0 | 0.000 |
| ENSP00000361275 | 6.72E-06 | -0.2379 | 191 | 0.502 |
| ENSP00000351147 | 5.51E-06 | -0.2379 | 0 | 0.121 |
| ENSP00000468367 | 1.60E-05 | -0.2379 | 401 | 0.235 |
| ENSP00000433757 | 4.93E-06 | -0.2380 | 671 | 0.208 |
| ENSP00000270560 | 2.65E-05 | -0.2380 | 0 | 0.453 |
| ENSP00000362562 | 2.18E-06 | -0.2380 | 0 | 0.152 |
| ENSP00000456850 | 2.73E-06 | -0.2380 | 0 | 0.100 |
| ENSP00000360828 | 1.42E-05 | -0.2381 | 313 | 0.931 |
| ENSP00000360642 | 2.80E-06 | -0.2381 | 0 | 0.078 |
| ENSP00000399235 | 1.33E-06 | -0.2382 | 0 | 0.000 |
| ENSP00000451812 | 2.34E-06 | -0.2383 | 0 | 0.533 |
| ENSP00000291823 | 6.19E-06 | -0.2383 | 189 | 0.674 |
| ENSP00000362268 | 2.25E-06 | -0.2383 | 0 | 0.079 |
| ENSP00000351536 | 5.54E-06 | -0.2383 | 0 | 0.086 |
| ENSP00000356988 | 5.74E-06 | -0.2383 | 212 | 0.230 |
| ENSP00000309521 | 5.96E-06 | -0.2384 | 0 | 0.129 |
| ENSP00000362507 | 3.21E-06 | -0.2384 | 0 | 0.111 |
| ENSP00000366083 | 1.70E-06 | -0.2385 | 0 | 0.000 |
| ENSP00000416126 | 2.61E-06 | -0.2385 | 0 | 0.086 |
| ENSP00000357706 | 2.59E-06 | -0.2385 | 0 | 0.168 |

| | | | | |
|---|---|---|---|---|
| ENSP00000293756 | 6.81E-06 | -0.2385 | 0 | 0.071 |
| ENSP00000349770 | 1.16E-05 | -0.2385 | 912 | 0.820 |
| ENSP00000359505 | 1.82E-06 | -0.2386 | 0 | 0.000 |
| ENSP00000327386 | 7.98E-06 | -0.2386 | 0 | 0.133 |
| ENSP00000374219 | 5.68E-06 | -0.2386 | 248 | 0.548 |
| ENSP00000370196 | 2.60E-06 | -0.2386 | 0 | 0.059 |
| ENSP00000371144 | 3.68E-06 | -0.2386 | 0 | 0.000 |
| ENSP00000371117 | 3.68E-06 | -0.2386 | 0 | 0.186 |
| ENSP00000344403 | 6.32E-06 | -0.2387 | 0 | 0.122 |
| ENSP00000248089 | 5.80E-06 | -0.2387 | 0 | 0.177 |
| ENSP00000369141 | 5.76E-06 | -0.2387 | 196 | 0.509 |
| ENSP00000391524 | 2.01E-06 | -0.2388 | 0 | 0.132 |
| ENSP00000419512 | 3.74E-06 | -0.2388 | 0 | 0.000 |
| ENSP00000362329 | 6.20E-06 | -0.2388 | 162 | 0.762 |
| ENSP00000392204 | 3.65E-07 | -0.2389 | 0 | 0.160 |
| ENSP00000363349 | 3.80E-06 | -0.2389 | 0 | 0.137 |
| ENSP00000429931 | 1.55E-05 | -0.2390 | 262 | 0.846 |
| ENSP00000365570 | 2.05E-06 | -0.2390 | 0 | 0.000 |
| ENSP00000433919 | 6.11E-06 | -0.2390 | 416 | 0.464 |
| ENSP00000265720 | 1.34E-05 | -0.2390 | 241 | 0.612 |
| ENSP00000457868 | 1.10E-05 | -0.2390 | 201 | 0.632 |
| ENSP00000455507 | 1.60E-05 | -0.2391 | 220 | 0.642 |
| ENSP00000375237 | 1.93E-06 | -0.2391 | 0 | 0.154 |
| ENSP00000316496 | 1.07E-05 | -0.2392 | 0 | 0.181 |
| ENSP00000424707 | 1.36E-06 | -0.2392 | 0 | 0.159 |
| ENSP00000379691 | 3.87E-06 | -0.2392 | 0 | 0.112 |
| ENSP00000310585 | 1.30E-05 | -0.2392 | 193 | 0.200 |
| ENSP00000291495 | 1.66E-05 | -0.2392 | 284 | 0.198 |
| ENSP00000415822 | 3.62E-06 | -0.2393 | 0 | 0.377 |
| ENSP00000432816 | 4.27E-06 | -0.2393 | 0 | 0.358 |
| ENSP00000371471 | 5.25E-06 | -0.2393 | 0 | 0.462 |
| ENSP00000351327 | 6.75E-06 | -0.2393 | 0 | 0.310 |
| ENSP00000347834 | 4.02E-06 | -0.2394 | 0 | 0.337 |
| ENSP00000365782 | 5.85E-06 | -0.2394 | 0 | 0.093 |
| ENSP00000408236 | 4.20E-06 | -0.2394 | 0 | 0.201 |
| ENSP00000423115 | 2.03E-06 | -0.2394 | 0 | 0.595 |
| ENSP00000352424 | 2.06E-05 | -0.2395 | 0 | 0.709 |
| ENSP00000419000 | 5.46E-06 | -0.2396 | 0 | 0.102 |
| ENSP00000281146 | 4.73E-06 | -0.2396 | 0 | 0.133 |
| ENSP00000334289 | 1.95E-06 | -0.2396 | 0 | 0.227 |
| ENSP00000410621 | 2.08E-06 | -0.2396 | 0 | 0.601 |
| ENSP00000293826 | 7.78E-06 | -0.2396 | 0 | 0.126 |
| ENSP00000367879 | 1.26E-05 | -0.2396 | 0 | 0.167 |
| ENSP00000239444 | 1.20E-05 | -0.2397 | 0 | 0.130 |
| ENSP00000417132 | 7.73E-06 | -0.2397 | 209 | 0.682 |
| ENSP00000355735 | 3.24E-06 | -0.2397 | 0 | 0.000 |

| | | | | |
|---|---|---|---|---|
| ENSP00000400880 | 2.14E-06 | -0.2397 | 0 | 0.601 |
| ENSP00000442464 | 6.14E-06 | -0.2398 | 360 | 0.164 |
| ENSP00000390759 | 2.18E-06 | -0.2398 | 0 | 0.000 |
| ENSP00000352219 | 6.58E-06 | -0.2398 | 362 | 0.603 |
| ENSP00000307722 | 7.31E-06 | -0.2398 | 0 | 0.000 |
| ENSP00000446058 | 1.51E-06 | -0.2398 | 0 | 0.108 |
| ENSP00000362092 | 3.11E-06 | -0.2398 | 0 | 0.426 |
| ENSP00000225873 | 5.46E-06 | -0.2398 | 0 | 0.075 |
| ENSP00000420176 | 4.83E-06 | -0.2399 | 0 | 0.781 |
| ENSP00000353292 | 7.44E-06 | -0.2399 | 384 | 0.403 |
| ENSP00000383505 | 4.93E-06 | -0.2399 | 0 | 0.086 |
| ENSP00000409256 | 1.46E-06 | -0.2399 | 0 | 0.000 |
| ENSP00000444052 | 9.48E-06 | -0.2400 | 0 | 0.078 |
| ENSP00000454670 | 5.03E-06 | -0.2400 | 183 | 0.092 |
| ENSP00000365643 | 8.89E-06 | -0.2400 | 0 | 0.286 |
| ENSP00000412324 | 9.41E-06 | -0.2400 | 0 | 0.568 |
| ENSP00000352962 | 2.84E-06 | -0.2401 | 0 | 0.000 |
| ENSP00000294829 | 4.23E-06 | -0.2401 | 0 | 0.114 |
| ENSP00000364749 | 6.91E-06 | -0.2401 | 448 | 0.595 |
| ENSP00000455298 | 9.48E-07 | -0.2401 | 0 | 0.152 |
| ENSP00000418741 | 4.96E-06 | -0.2401 | 0 | 0.080 |
| ENSP00000328977 | 4.73E-06 | -0.2402 | 0 | 0.099 |
| ENSP00000468678 | 5.72E-06 | -0.2402 | 0 | 0.126 |
| ENSP00000365926 | 6.74E-06 | -0.2402 | 287 | 0.275 |
| ENSP00000438949 | 2.56E-06 | -0.2402 | 0 | 0.292 |
| ENSP00000241041 | 6.77E-06 | -0.2402 | 0 | 0.051 |
| ENSP00000341803 | 1.10E-05 | -0.2402 | 0 | 0.084 |
| ENSP00000386878 | 2.09E-06 | -0.2403 | 0 | 0.117 |
| ENSP00000376848 | 2.99E-06 | -0.2403 | 0 | 0.078 |
| ENSP00000375232 | 1.82E-06 | -0.2403 | 0 | 0.139 |
| ENSP00000352603 | 5.18E-06 | -0.2403 | 0 | 0.166 |
| ENSP00000378161 | 2.07E-06 | -0.2403 | 0 | 0.114 |
| ENSP00000320886 | 9.53E-06 | -0.2403 | 390 | 0.293 |
| ENSP00000356579 | 3.67E-06 | -0.2403 | 0 | 0.058 |
| ENSP00000376794 | 2.36E-06 | -0.2403 | 0 | 0.140 |
| ENSP00000288048 | 2.46E-06 | -0.2404 | 0 | 0.000 |
| ENSP00000242249 | 1.88E-05 | -0.2405 | 0 | 0.105 |
| ENSP00000451263 | 5.28E-06 | -0.2405 | 205 | 0.683 |
| ENSP00000462701 | 3.85E-06 | -0.2405 | 0 | 0.208 |
| ENSP00000386711 | 2.37E-06 | -0.2405 | 0 | 0.096 |
| ENSP00000449770 | 1.47E-05 | -0.2405 | 230 | 0.905 |
| ENSP00000350447 | 2.18E-06 | -0.2406 | 0 | 0.269 |
| ENSP00000325296 | 1.32E-05 | -0.2406 | 202 | 0.204 |
| ENSP00000361926 | 3.23E-06 | -0.2407 | 0 | 0.073 |
| ENSP00000348959 | 3.99E-06 | -0.2407 | 0 | 0.132 |
| ENSP00000465894 | 2.66E-06 | -0.2407 | 0 | 0.077 |

| | | | | |
|---|---|---|---|---|
| ENSP00000297459 | 3.50E-06 | -0.2407 | 0 | 0.105 |
| ENSP00000399668 | 1.62E-05 | -0.2408 | 153 | 0.000 |
| ENSP00000451995 | 1.08E-05 | -0.2408 | 187 | 0.000 |
| ENSP00000386971 | 7.74E-06 | -0.2408 | 201 | 0.000 |
| ENSP00000348602 | 6.62E-06 | -0.2408 | 150 | 0.477 |
| ENSP00000389760 | 3.65E-06 | -0.2408 | 0 | 0.128 |
| ENSP00000338613 | 6.85E-06 | -0.2409 | 0 | 0.131 |
| ENSP00000310841 | 4.46E-06 | -0.2409 | 0 | 0.105 |
| ENSP00000402355 | 1.21E-06 | -0.2409 | 0 | 0.118 |
| ENSP00000356218 | 4.89E-06 | -0.2409 | 193 | 0.331 |
| ENSP00000348593 | 4.51E-06 | -0.2410 | 0 | 0.000 |
| ENSP00000389443 | 2.17E-06 | -0.2410 | 0 | 0.597 |
| ENSP00000367130 | 3.74E-06 | -0.2410 | 0 | 0.337 |
| ENSP00000304811 | 4.81E-06 | -0.2410 | 0 | 0.210 |
| ENSP00000374592 | 7.20E-06 | -0.2412 | 487 | 0.000 |
| ENSP00000361331 | 2.84E-06 | -0.2412 | 0 | 0.175 |
| ENSP00000389913 | 3.02E-06 | -0.2413 | 0 | 0.108 |
| ENSP00000355752 | 1.35E-06 | -0.2414 | 0 | 0.096 |
| ENSP00000263863 | 1.57E-05 | -0.2414 | 202 | 0.563 |
| ENSP00000264728 | 9.87E-06 | -0.2414 | 0 | 0.095 |
| ENSP00000352676 | 3.45E-06 | -0.2414 | 0 | 0.303 |
| ENSP00000347839 | 3.77E-06 | -0.2415 | 0 | 0.155 |
| ENSP00000371101 | 7.54E-06 | -0.2415 | 0 | 0.832 |
| ENSP00000382610 | 6.22E-06 | -0.2415 | 202 | 0.633 |
| ENSP00000400592 | 3.57E-06 | -0.2415 | 0 | 0.000 |
| ENSP00000395249 | 2.55E-06 | -0.2415 | 0 | 0.088 |
| ENSP00000321464 | 7.95E-06 | -0.2415 | 0 | 0.188 |
| ENSP00000471653 | 6.45E-07 | -0.2416 | 0 | 0.000 |
| ENSP00000361755 | 3.36E-06 | -0.2417 | 0 | 0.200 |
| ENSP00000320520 | 1.94E-06 | -0.2417 | 0 | 0.000 |
| ENSP00000397239 | 6.56E-06 | -0.2417 | 163 | 0.000 |
| ENSP00000364591 | 1.64E-05 | -0.2418 | 153 | 0.000 |
| ENSP00000318912 | 6.10E-06 | -0.2418 | 0 | 0.119 |
| ENSP00000384887 | 3.15E-06 | -0.2420 | 0 | 0.144 |
| ENSP00000353742 | 2.54E-06 | -0.2420 | 0 | 0.117 |
| ENSP00000370697 | 3.21E-06 | -0.2420 | 0 | 0.410 |
| ENSP00000310829 | 7.70E-06 | -0.2420 | 0 | 0.000 |
| ENSP00000414904 | 3.48E-06 | -0.2420 | 0 | 0.093 |
| ENSP00000333823 | 1.65E-05 | -0.2421 | 0 | 0.000 |
| ENSP00000362873 | 1.88E-06 | -0.2421 | 0 | 0.226 |
| ENSP00000287275 | 1.28E-05 | -0.2421 | 0 | 0.217 |
| ENSP00000370057 | 2.00E-06 | -0.2421 | 0 | 0.105 |
| ENSP00000349954 | 1.15E-05 | -0.2421 | 208 | 0.554 |
| ENSP00000379154 | 2.31E-06 | -0.2421 | 0 | 0.076 |
| ENSP00000341141 | 5.20E-06 | -0.2421 | 0 | 0.391 |
| ENSP00000362588 | 4.52E-06 | -0.2422 | 0 | 0.821 |

| | | | | |
|---|---|---|---|---|
| ENSP00000369618 | 1.81E-06 | -0.2422 | 0 | 0.123 |
| ENSP00000312286 | 8.05E-06 | -0.2422 | 0 | 0.300 |
| ENSP00000351885 | 8.86E-06 | -0.2422 | 163 | 0.810 |
| ENSP00000262138 | 3.23E-06 | -0.2422 | 0 | 0.124 |
| ENSP00000318158 | 4.31E-06 | -0.2423 | 0 | 0.400 |
| ENSP00000278826 | 7.05E-06 | -0.2424 | 0 | 0.179 |
| ENSP00000361413 | 3.76E-06 | -0.2424 | 0 | 0.211 |
| ENSP00000423067 | 1.17E-05 | -0.2424 | 260 | 0.833 |
| ENSP00000417182 | 1.20E-06 | -0.2424 | 0 | 0.097 |
| ENSP00000358241 | 6.96E-06 | -0.2424 | 0 | 0.000 |
| ENSP00000359507 | 3.61E-06 | -0.2425 | 0 | 0.110 |
| ENSP00000332805 | 3.86E-06 | -0.2426 | 0 | 0.112 |
| ENSP00000386657 | 9.03E-07 | -0.2426 | 0 | 0.000 |
| ENSP00000380793 | 4.84E-06 | -0.2427 | 0 | 0.222 |
| ENSP00000440273 | 2.26E-06 | -0.2427 | 0 | 0.265 |
| ENSP00000328336 | 2.58E-06 | -0.2427 | 0 | 0.115 |
| ENSP00000326407 | 8.04E-06 | -0.2427 | 0 | 0.121 |
| ENSP00000318395 | 7.81E-06 | -0.2427 | 0 | 0.000 |
| ENSP00000420127 | 7.23E-06 | -0.2428 | 161 | 0.196 |
| ENSP00000386264 | 2.08E-06 | -0.2428 | 0 | 0.097 |
| ENSP00000353114 | 8.77E-06 | -0.2429 | 0 | 0.149 |
| ENSP00000230012 | 1.02E-05 | -0.2429 | 0 | 0.000 |
| ENSP00000456021 | 5.02E-07 | -0.2429 | 0 | 0.000 |
| ENSP00000362746 | 7.87E-06 | -0.2430 | 404 | 0.137 |
| ENSP00000380380 | 6.02E-07 | -0.2430 | 0 | 0.000 |
| ENSP00000373176 | 3.89E-06 | -0.2430 | 191 | 0.308 |
| ENSP00000346466 | 3.54E-06 | -0.2430 | 0 | 0.122 |
| ENSP00000353910 | 5.52E-06 | -0.2430 | 0 | 0.100 |
| ENSP00000357581 | 7.56E-06 | -0.2431 | 163 | 0.833 |
| ENSP00000333779 | 3.81E-06 | -0.2431 | 0 | 0.176 |
| ENSP00000345716 | 5.80E-06 | -0.2431 | 0 | 0.243 |
| ENSP00000317818 | 4.78E-06 | -0.2431 | 0 | 0.157 |
| ENSP00000308708 | 3.23E-06 | -0.2432 | 0 | 0.121 |
| ENSP00000368686 | 9.73E-06 | -0.2432 | 195 | 0.775 |
| ENSP00000273695 | 6.51E-06 | -0.2432 | 0 | 0.116 |
| ENSP00000355672 | 3.17E-06 | -0.2432 | 0 | 0.126 |
| ENSP00000426174 | 1.42E-06 | -0.2433 | 0 | 0.146 |
| ENSP00000386398 | 7.37E-06 | -0.2433 | 202 | 0.677 |
| ENSP00000378356 | 4.18E-06 | -0.2433 | 0 | 0.318 |
| ENSP00000355177 | 2.86E-06 | -0.2434 | 0 | 0.087 |
| ENSP00000464258 | 1.92E-06 | -0.2434 | 0 | 0.274 |
| ENSP00000349041 | 1.26E-05 | -0.2434 | 313 | 0.855 |
| ENSP00000356115 | 3.19E-06 | -0.2434 | 0 | 0.118 |
| ENSP00000362704 | 3.56E-06 | -0.2434 | 0 | 0.195 |
| ENSP00000323068 | 4.27E-06 | -0.2434 | 0 | 0.111 |
| ENSP00000266534 | 2.38E-06 | -0.2435 | 0 | 0.088 |

| | | | | |
|---|---|---|---|---|
| ENSP00000427802 | 5.20E-06 | -0.2435 | 312 | 0.762 |
| ENSP00000336832 | 6.54E-06 | -0.2435 | 0 | 0.000 |
| ENSP00000359935 | 3.21E-06 | -0.2435 | 0 | 0.083 |
| ENSP00000348496 | 2.40E-06 | -0.2436 | 0 | 0.087 |
| ENSP00000431083 | 5.78E-06 | -0.2436 | 0 | 0.142 |
| ENSP00000384144 | 1.28E-05 | -0.2436 | 877 | 0.000 |
| ENSP00000352216 | 5.68E-06 | -0.2436 | 0 | 0.081 |
| ENSP00000327786 | 4.91E-06 | -0.2437 | 0 | 0.093 |
| ENSP00000366645 | 2.96E-06 | -0.2437 | 0 | 0.129 |
| ENSP00000370826 | 3.47E-06 | -0.2438 | 0 | 0.305 |
| ENSP00000405708 | 7.04E-06 | -0.2438 | 0 | 0.050 |
| ENSP00000414670 | 2.17E-06 | -0.2438 | 0 | 0.141 |
| ENSP00000364243 | 5.80E-06 | -0.2438 | 0 | 0.188 |
| ENSP00000323246 | 3.29E-06 | -0.2438 | 0 | 0.604 |
| ENSP00000378529 | 5.79E-06 | -0.2439 | 0 | 0.576 |
| ENSP00000386918 | 6.18E-06 | -0.2439 | 233 | 0.129 |
| ENSP00000406026 | 2.21E-06 | -0.2439 | 0 | 0.077 |
| ENSP00000309132 | 1.13E-05 | -0.2440 | 158 | 0.441 |
| ENSP00000323435 | 2.13E-06 | -0.2440 | 0 | 0.107 |
| ENSP00000392859 | 6.64E-06 | -0.2440 | 329 | 0.615 |
| ENSP00000319673 | 4.09E-06 | -0.2441 | 0 | 0.088 |
| ENSP00000420227 | 2.86E-06 | -0.2442 | 0 | 0.000 |
| ENSP00000398655 | 1.00E-05 | -0.2442 | 205 | 0.678 |
| ENSP00000406577 | 5.82E-06 | -0.2443 | 347 | 0.182 |
| ENSP00000190165 | 3.60E-06 | -0.2443 | 0 | 0.519 |
| ENSP00000355958 | 3.27E-06 | -0.2444 | 0 | 0.603 |
| ENSP00000387252 | 1.19E-06 | -0.2444 | 0 | 0.136 |
| ENSP00000398688 | 2.57E-06 | -0.2444 | 0 | 0.517 |
| ENSP00000407978 | 4.41E-06 | -0.2444 | 0 | 0.457 |
| ENSP00000384582 | 1.20E-05 | -0.2444 | 347 | 0.000 |
| ENSP00000365007 | 5.36E-06 | -0.2445 | 815 | 0.216 |
| ENSP00000390621 | 1.49E-05 | -0.2445 | 330 | 0.925 |
| ENSP00000462945 | 2.99E-06 | -0.2445 | 0 | 0.441 |
| ENSP00000352955 | 3.22E-06 | -0.2445 | 0 | 0.110 |
| ENSP00000416095 | 1.00E-06 | -0.2446 | 0 | 0.000 |
| ENSP00000370443 | 1.29E-05 | -0.2446 | 237 | 0.149 |
| ENSP00000369198 | 1.32E-05 | -0.2446 | 167 | 0.000 |
| ENSP00000351596 | 2.94E-06 | -0.2447 | 0 | 0.638 |
| ENSP00000334330 | 1.23E-06 | -0.2447 | 0 | 0.133 |
| ENSP00000350409 | 4.19E-06 | -0.2447 | 0 | 0.101 |
| ENSP00000355923 | 3.64E-06 | -0.2447 | 0 | 0.698 |
| ENSP00000349525 | 5.78E-06 | -0.2447 | 277 | 0.217 |
| ENSP00000306381 | 3.76E-06 | -0.2448 | 0 | 0.000 |
| ENSP00000380996 | 1.76E-06 | -0.2448 | 0 | 0.153 |
| ENSP00000363545 | 2.70E-06 | -0.2448 | 0 | 0.138 |
| ENSP00000290079 | 3.23E-06 | -0.2448 | 0 | 0.165 |

| | | | | |
|---|---|---|---|---|
| ENSP00000387303 | 1.50E-05 | -0.2449 | 181 | 0.858 |
| ENSP00000363387 | 6.09E-06 | -0.2450 | 0 | 0.521 |
| ENSP00000307640 | 3.15E-06 | -0.2450 | 0 | 0.102 |
| ENSP00000376123 | 2.52E-06 | -0.2450 | 0 | 0.393 |
| ENSP00000325978 | 5.87E-06 | -0.2450 | 0 | 0.101 |
| ENSP00000356319 | 5.71E-06 | -0.2450 | 0 | 0.238 |
| ENSP00000323178 | 1.23E-05 | -0.2451 | 0 | 0.000 |
| ENSP00000261592 | 7.73E-06 | -0.2451 | 0 | 0.156 |
| ENSP00000401435 | 5.00E-06 | -0.2452 | 163 | 0.137 |
| ENSP00000202625 | 1.23E-05 | -0.2452 | 0 | 0.387 |
| ENSP00000364476 | 1.62E-06 | -0.2452 | 0 | 0.102 |
| ENSP00000370105 | 3.21E-06 | -0.2452 | 0 | 0.740 |
| ENSP00000348161 | 4.83E-06 | -0.2452 | 0 | 0.127 |
| ENSP00000307096 | 5.67E-06 | -0.2452 | 0 | 0.000 |
| ENSP00000311528 | 7.17E-06 | -0.2453 | 0 | 0.228 |
| ENSP00000293889 | 5.71E-06 | -0.2453 | 0 | 0.110 |
| ENSP00000382379 | 9.14E-06 | -0.2453 | 202 | 0.699 |
| ENSP00000357020 | 3.03E-06 | -0.2453 | 0 | 0.000 |
| ENSP00000330190 | 1.29E-05 | -0.2453 | 151 | 0.683 |
| ENSP00000368174 | 3.75E-06 | -0.2454 | 0 | 0.698 |
| ENSP00000313513 | 6.49E-06 | -0.2454 | 0 | 0.101 |
| ENSP00000347486 | 3.67E-06 | -0.2454 | 0 | 0.080 |
| ENSP00000282226 | 6.36E-06 | -0.2454 | 0 | 0.091 |
| ENSP00000354772 | 3.55E-06 | -0.2454 | 0 | 0.084 |
| ENSP00000332690 | 3.30E-06 | -0.2456 | 0 | 0.133 |
| ENSP00000341677 | 5.33E-06 | -0.2456 | 0 | 0.125 |
| ENSP00000388137 | 1.37E-06 | -0.2456 | 0 | 0.091 |
| ENSP00000338532 | 4.74E-06 | -0.2456 | 0 | 0.108 |
| ENSP00000422533 | 1.45E-05 | -0.2456 | 196 | 0.000 |
| ENSP00000372394 | 3.92E-06 | -0.2457 | 0 | 0.174 |
| ENSP00000382714 | 3.59E-06 | -0.2457 | 0 | 0.532 |
| ENSP00000361923 | 2.13E-06 | -0.2457 | 0 | 0.299 |
| ENSP00000306335 | 3.54E-06 | -0.2457 | 0 | 0.732 |
| ENSP00000357844 | 3.49E-06 | -0.2457 | 0 | 0.092 |
| ENSP00000442477 | 4.12E-06 | -0.2457 | 0 | 0.150 |
| ENSP00000288098 | 9.82E-06 | -0.2457 | 0 | 0.235 |
| ENSP00000337759 | 4.01E-06 | -0.2457 | 169 | 0.000 |
| ENSP00000387536 | 4.49E-06 | -0.2458 | 197 | 0.442 |
| ENSP00000308606 | 4.50E-06 | -0.2458 | 0 | 0.116 |
| ENSP00000400471 | 4.00E-06 | -0.2458 | 0 | 0.124 |
| ENSP00000357739 | 4.37E-06 | -0.2458 | 0 | 0.120 |
| ENSP00000367459 | 2.14E-06 | -0.2458 | 0 | 0.116 |
| ENSP00000368908 | 1.11E-05 | -0.2458 | 0 | 0.080 |
| ENSP00000453436 | 8.86E-07 | -0.2459 | 0 | 0.000 |
| ENSP00000307831 | 6.84E-06 | -0.2460 | 0 | 0.000 |
| ENSP00000295083 | 4.17E-06 | -0.2460 | 0 | 0.092 |

| | | | | |
|---|---|---|---|---|
| ENSP00000307741 | 4.27E-06 | -0.2460 | 0 | 0.118 |
| ENSP00000335158 | 9.74E-06 | -0.2461 | 662 | 0.202 |
| ENSP00000361064 | 4.07E-06 | -0.2462 | 0 | 0.156 |
| ENSP00000413049 | 1.86E-06 | -0.2463 | 0 | 0.073 |
| ENSP00000351446 | 8.14E-06 | -0.2463 | 242 | 0.000 |
| ENSP00000342336 | 7.60E-06 | -0.2464 | 0 | 0.146 |
| ENSP00000367203 | 1.72E-05 | -0.2464 | 316 | 0.000 |
| ENSP00000340220 | 2.77E-06 | -0.2465 | 0 | 0.148 |
| ENSP00000367065 | 3.46E-06 | -0.2465 | 0 | 0.768 |
| ENSP00000382508 | 1.58E-06 | -0.2465 | 0 | 0.132 |
| ENSP00000365534 | 5.29E-06 | -0.2466 | 440 | 0.574 |
| ENSP00000382104 | 4.93E-06 | -0.2466 | 0 | 0.067 |
| ENSP00000392057 | 6.37E-06 | -0.2466 | 0 | 0.068 |
| ENSP00000361726 | 3.39E-06 | -0.2466 | 0 | 0.152 |
| ENSP00000347883 | 3.92E-06 | -0.2467 | 0 | 0.114 |
| ENSP00000369862 | 6.25E-06 | -0.2467 | 0 | 0.121 |
| ENSP00000367490 | 1.94E-06 | -0.2468 | 0 | 0.166 |
| ENSP00000383155 | 5.48E-06 | -0.2468 | 0 | 0.000 |
| ENSP00000356057 | 2.74E-06 | -0.2468 | 0 | 0.109 |
| ENSP00000291539 | 8.63E-06 | -0.2469 | 511 | 0.658 |
| ENSP00000338457 | 3.57E-06 | -0.2470 | 0 | 0.072 |
| ENSP00000368876 | 3.29E-06 | -0.2470 | 0 | 0.121 |
| ENSP00000355146 | 2.94E-06 | -0.2471 | 0 | 0.191 |
| ENSP00000417602 | 2.65E-06 | -0.2471 | 0 | 0.605 |
| ENSP00000428780 | 1.38E-06 | -0.2472 | 0 | 0.144 |
| ENSP00000398615 | 1.32E-06 | -0.2472 | 0 | 0.000 |
| ENSP00000338783 | 1.18E-05 | -0.2472 | 974 | 0.061 |
| ENSP00000445162 | 3.87E-06 | -0.2472 | 0 | 0.744 |
| ENSP00000417050 | 1.38E-06 | -0.2474 | 0 | 0.000 |
| ENSP00000469517 | 2.59E-06 | -0.2474 | 0 | 0.000 |
| ENSP00000377078 | 1.55E-06 | -0.2474 | 0 | 0.144 |
| ENSP00000359375 | 5.00E-06 | -0.2474 | 0 | 0.099 |
| ENSP00000362395 | 3.39E-06 | -0.2474 | 0 | 0.071 |
| ENSP00000360286 | 7.88E-06 | -0.2474 | 156 | 0.564 |
| ENSP00000356700 | 5.20E-06 | -0.2474 | 0 | 0.102 |
| ENSP00000365103 | 8.92E-06 | -0.2475 | 211 | 0.234 |
| ENSP00000432545 | 3.98E-06 | -0.2476 | 0 | 0.000 |
| ENSP00000364627 | 2.08E-06 | -0.2476 | 0 | 0.128 |
| ENSP00000381921 | 3.28E-06 | -0.2476 | 0 | 0.176 |
| ENSP00000361238 | 3.81E-06 | -0.2476 | 0 | 0.063 |
| ENSP00000343900 | 3.19E-06 | -0.2477 | 0 | 0.561 |
| ENSP00000374183 | 3.86E-06 | -0.2477 | 0 | 0.115 |
| ENSP00000393541 | 3.94E-06 | -0.2478 | 0 | 0.101 |
| ENSP00000435412 | 1.86E-05 | -0.2478 | 308 | 0.000 |
| ENSP00000325564 | 7.27E-06 | -0.2478 | 0 | 0.094 |
| ENSP00000239450 | 5.74E-06 | -0.2478 | 0 | 0.136 |

| | | | | |
|---|---|---|---|---|
| ENSP00000409581 | 2.09E-05 | -0.2479 | 972 | 0.859 |
| ENSP00000292574 | 6.04E-06 | -0.2479 | 0 | 0.107 |
| ENSP00000361642 | 3.35E-06 | -0.2479 | 0 | 0.504 |
| ENSP00000366641 | 1.29E-05 | -0.2480 | 151 | 0.273 |
| ENSP00000367398 | 9.33E-06 | -0.2480 | 303 | 0.271 |
| ENSP00000356432 | 6.31E-06 | -0.2480 | 0 | 0.000 |
| ENSP00000348064 | 5.10E-06 | -0.2480 | 0 | 0.108 |
| ENSP00000361302 | 5.36E-06 | -0.2481 | 0 | 0.202 |
| ENSP00000304078 | 4.00E-06 | -0.2481 | 0 | 0.110 |
| ENSP00000396915 | 1.77E-06 | -0.2482 | 0 | 0.146 |
| ENSP00000390637 | 7.58E-06 | -0.2482 | 155 | 0.267 |
| ENSP00000396622 | 5.23E-06 | -0.2482 | 0 | 0.134 |
| ENSP00000355374 | 3.29E-06 | -0.2483 | 0 | 0.115 |
| ENSP00000384967 | 4.08E-06 | -0.2483 | 0 | 0.120 |
| ENSP00000350331 | 7.01E-06 | -0.2483 | 202 | 0.662 |
| ENSP00000442518 | 7.14E-06 | -0.2485 | 202 | 0.000 |
| ENSP00000341794 | 7.25E-06 | -0.2485 | 0 | 0.128 |
| ENSP00000337854 | 5.11E-06 | -0.2485 | 0 | 0.093 |
| ENSP00000448035 | 4.57E-06 | -0.2485 | 340 | 0.763 |
| ENSP00000359049 | 3.92E-06 | -0.2486 | 0 | 0.202 |
| ENSP00000239125 | 3.80E-06 | -0.2486 | 0 | 0.000 |
| ENSP00000354891 | 5.14E-06 | -0.2486 | 0 | 0.105 |
| ENSP00000347930 | 3.92E-06 | -0.2486 | 0 | 0.000 |
| ENSP00000377170 | 5.99E-06 | -0.2486 | 202 | 0.618 |
| ENSP00000376188 | 3.48E-06 | -0.2486 | 0 | 0.249 |
| ENSP00000391447 | 2.80E-06 | -0.2486 | 0 | 0.768 |
| ENSP00000381494 | 2.32E-06 | -0.2486 | 0 | 0.105 |
| ENSP00000264387 | 1.15E-05 | -0.2486 | 0 | 0.118 |
| ENSP00000358307 | 4.51E-06 | -0.2486 | 0 | 0.128 |
| ENSP00000350078 | 5.69E-06 | -0.2487 | 0 | 0.838 |
| ENSP00000347188 | 3.07E-06 | -0.2487 | 0 | 0.123 |
| ENSP00000429018 | 1.59E-06 | -0.2487 | 0 | 0.125 |
| ENSP00000352672 | 5.80E-06 | -0.2488 | 0 | 0.245 |
| ENSP00000346931 | 3.33E-06 | -0.2489 | 0 | 0.100 |
| ENSP00000378756 | 3.50E-06 | -0.2489 | 0 | 0.055 |
| ENSP00000359531 | 1.42E-05 | -0.2489 | 271 | 0.000 |
| ENSP00000436585 | 5.34E-06 | -0.2489 | 0 | 0.249 |
| ENSP00000328737 | 1.01E-05 | -0.2489 | 700 | 0.000 |
| ENSP00000354777 | 2.03E-06 | -0.2490 | 0 | 0.105 |
| ENSP00000314709 | 6.11E-06 | -0.2490 | 0 | 0.404 |
| ENSP00000299155 | 1.17E-05 | -0.2491 | 163 | 0.131 |
| ENSP00000348757 | 1.60E-06 | -0.2491 | 0 | 0.078 |
| ENSP00000263765 | 2.88E-06 | -0.2491 | 0 | 0.000 |
| ENSP00000368273 | 2.62E-06 | -0.2492 | 0 | 0.259 |
| ENSP00000357547 | 3.55E-06 | -0.2492 | 0 | 0.370 |
| ENSP00000419879 | 5.86E-06 | -0.2492 | 200 | 0.276 |

| | | | | |
|---|---|---|---|---|
| ENSP00000340017 | 3.08E-06 | -0.2493 | 0 | 0.108 |
| ENSP00000335325 | 5.05E-06 | -0.2493 | 0 | 0.091 |
| ENSP00000046087 | 1.47E-05 | -0.2494 | 0 | 0.053 |
| ENSP00000354691 | 7.30E-06 | -0.2494 | 0 | 0.100 |
| ENSP00000358099 | 8.75E-06 | -0.2494 | 188 | 0.577 |
| ENSP00000362332 | 4.73E-06 | -0.2495 | 0 | 0.162 |
| ENSP00000381821 | 4.79E-06 | -0.2496 | 0 | 0.111 |
| ENSP00000418193 | 3.99E-06 | -0.2496 | 0 | 0.102 |
| ENSP00000389455 | 3.15E-06 | -0.2496 | 0 | 0.324 |
| ENSP00000367965 | 3.63E-06 | -0.2496 | 0 | 0.107 |
| ENSP00000371542 | 4.07E-06 | -0.2496 | 0 | 0.309 |
| ENSP00000185206 | 5.05E-06 | -0.2497 | 0 | 0.053 |
| ENSP00000298622 | 5.92E-06 | -0.2497 | 0 | 0.103 |
| ENSP00000430075 | 7.71E-06 | -0.2497 | 294 | 0.211 |
| ENSP00000450560 | 1.39E-05 | -0.2497 | 485 | 0.659 |
| ENSP00000295440 | 1.51E-05 | -0.2498 | 0 | 0.345 |
| ENSP00000368716 | 2.24E-06 | -0.2498 | 0 | 0.327 |
| ENSP00000341030 | 1.11E-05 | -0.2498 | 0 | 0.227 |
| ENSP00000363590 | 5.16E-06 | -0.2498 | 0 | 0.087 |
| ENSP00000245810 | 1.58E-05 | -0.2499 | 0 | 0.471 |
| ENSP00000328423 | 6.53E-06 | -0.2499 | 0 | 0.000 |
| ENSP00000262765 | 2.11E-06 | -0.2499 | 0 | 0.179 |
| ENSP00000395653 | 3.66E-06 | -0.2499 | 0 | 0.000 |
| ENSP00000347251 | 4.04E-06 | -0.2499 | 0 | 0.000 |
| ENSP00000295297 | 6.85E-06 | -0.2500 | 0 | 0.216 |
| ENSP00000366620 | 6.70E-06 | -0.2500 | 597 | 0.541 |
| ENSP00000330200 | 3.44E-06 | -0.2500 | 0 | 0.098 |
| ENSP00000409016 | 3.57E-06 | -0.2500 | 0 | 0.128 |
| ENSP00000345957 | 4.14E-06 | -0.2500 | 0 | 0.755 |
| ENSP00000353128 | 3.72E-06 | -0.2500 | 0 | 0.105 |
| ENSP00000341412 | 5.66E-06 | -0.2500 | 0 | 0.000 |
| ENSP00000379865 | 1.53E-05 | -0.2501 | 242 | 0.284 |
| ENSP00000418082 | 5.78E-06 | -0.2502 | 171 | 0.826 |
| ENSP00000384869 | 6.93E-06 | -0.2502 | 196 | 0.272 |
| ENSP00000354597 | 2.28E-06 | -0.2503 | 0 | 0.120 |
| ENSP00000431459 | 2.75E-06 | -0.2503 | 0 | 0.128 |
| ENSP00000352167 | 4.42E-06 | -0.2503 | 0 | 0.106 |
| ENSP00000385347 | 2.32E-06 | -0.2503 | 0 | 0.111 |
| ENSP00000316491 | 7.56E-06 | -0.2504 | 0 | 0.086 |
| ENSP00000347999 | 3.08E-06 | -0.2504 | 0 | 0.358 |
| ENSP00000365528 | 3.24E-06 | -0.2504 | 0 | 0.320 |
| ENSP00000256389 | 1.01E-05 | -0.2504 | 0 | 0.069 |
| ENSP00000359464 | 1.36E-06 | -0.2507 | 0 | 0.144 |
| ENSP00000360966 | 5.47E-06 | -0.2507 | 162 | 0.066 |
| ENSP00000363001 | 4.15E-06 | -0.2507 | 0 | 0.073 |
| ENSP00000354724 | 3.40E-06 | -0.2508 | 0 | 0.659 |

| | | | | |
|---|---|---|---|---|
| ENSP00000330442 | 6.26E-06 | -0.2508 | 0 | 0.125 |
| ENSP00000362987 | 1.50E-06 | -0.2510 | 0 | 0.120 |
| ENSP00000387593 | 7.23E-06 | -0.2510 | 211 | 0.712 |
| ENSP00000369312 | 3.98E-06 | -0.2510 | 0 | 0.594 |
| ENSP00000356298 | 3.28E-06 | -0.2511 | 0 | 0.000 |
| ENSP00000351138 | 4.48E-06 | -0.2511 | 0 | 0.119 |
| ENSP00000319118 | 1.05E-05 | -0.2511 | 186 | 0.819 |
| ENSP00000409722 | 2.54E-06 | -0.2511 | 0 | 0.000 |
| ENSP00000370293 | 2.60E-06 | -0.2512 | 0 | 0.425 |
| ENSP00000350377 | 2.72E-06 | -0.2512 | 0 | 0.097 |
| ENSP00000362677 | 4.00E-06 | -0.2512 | 242 | 0.000 |
| ENSP00000358242 | 4.04E-06 | -0.2512 | 0 | 0.752 |
| ENSP00000377807 | 2.48E-06 | -0.2512 | 0 | 0.092 |
| ENSP00000365567 | 4.56E-06 | -0.2513 | 0 | 0.126 |
| ENSP00000295171 | 8.23E-06 | -0.2513 | 0 | 0.101 |
| ENSP00000335292 | 7.97E-06 | -0.2513 | 0 | 0.070 |
| ENSP00000345133 | 3.51E-06 | -0.2514 | 0 | 0.262 |
| ENSP00000391088 | 3.09E-06 | -0.2514 | 0 | 0.089 |
| ENSP00000419199 | 8.60E-06 | -0.2514 | 202 | 0.606 |
| ENSP00000398211 | 7.54E-06 | -0.2515 | 0 | 0.093 |
| ENSP00000339723 | 3.92E-06 | -0.2516 | 0 | 0.367 |
| ENSP00000363139 | 6.67E-06 | -0.2516 | 0 | 0.077 |
| ENSP00000258403 | 5.63E-06 | -0.2516 | 0 | 0.065 |
| ENSP00000244625 | 7.61E-06 | -0.2516 | 0 | 0.000 |
| ENSP00000284617 | 5.86E-06 | -0.2517 | 0 | 0.106 |
| ENSP00000386171 | 4.21E-06 | -0.2518 | 0 | 0.775 |
| ENSP00000414109 | 2.22E-06 | -0.2518 | 0 | 0.341 |
| ENSP00000430024 | 1.40E-06 | -0.2518 | 0 | 0.145 |
| ENSP00000362814 | 1.17E-05 | -0.2519 | 167 | 0.099 |
| ENSP00000279024 | 5.76E-06 | -0.2519 | 0 | 0.159 |
| ENSP00000349629 | 9.87E-06 | -0.2519 | 169 | 0.241 |
| ENSP00000341360 | 4.66E-06 | -0.2519 | 0 | 0.162 |
| ENSP00000332613 | 1.21E-05 | -0.2520 | 169 | 0.226 |
| ENSP00000369446 | 4.18E-06 | -0.2520 | 0 | 0.154 |
| ENSP00000364956 | 6.08E-06 | -0.2520 | 0 | 0.089 |
| ENSP00000426103 | 1.10E-05 | -0.2521 | 245 | 0.549 |
| ENSP00000329295 | 5.17E-06 | -0.2521 | 0 | 0.081 |
| ENSP00000345001 | 4.62E-06 | -0.2521 | 0 | 0.817 |
| ENSP00000307519 | 9.35E-06 | -0.2521 | 0 | 0.207 |
| ENSP00000345512 | 1.12E-05 | -0.2521 | 154 | 0.672 |
| ENSP00000299272 | 1.67E-05 | -0.2521 | 167 | 0.000 |
| ENSP00000457689 | 6.69E-06 | -0.2522 | 201 | 0.000 |
| ENSP00000294309 | 7.98E-06 | -0.2522 | 0 | 0.049 |
| ENSP00000354822 | 3.63E-06 | -0.2523 | 0 | 0.385 |
| ENSP00000315106 | 1.06E-05 | -0.2523 | 159 | 0.173 |
| ENSP00000290607 | 2.89E-06 | -0.2524 | 0 | 0.178 |

| | | | | |
|---|---|---|---|---|
| ENSP00000432743 | 4.48E-06 | -0.2524 | 0 | 0.756 |
| ENSP00000358042 | 6.56E-06 | -0.2524 | 252 | 0.500 |
| ENSP00000358105 | 8.41E-06 | -0.2525 | 198 | 0.276 |
| ENSP00000329266 | 8.50E-06 | -0.2525 | 414 | 0.000 |
| ENSP00000349325 | 3.77E-06 | -0.2525 | 0 | 0.381 |
| ENSP00000367378 | 8.44E-06 | -0.2525 | 229 | 0.142 |
| ENSP00000380781 | 5.09E-06 | -0.2525 | 0 | 0.757 |
| ENSP00000364349 | 9.97E-06 | -0.2526 | 506 | 0.564 |
| ENSP00000374218 | 7.37E-06 | -0.2527 | 0 | 0.069 |
| ENSP00000418842 | 1.07E-05 | -0.2527 | 201 | 0.346 |
| ENSP00000350359 | 9.40E-06 | -0.2527 | 225 | 0.000 |
| ENSP00000284881 | 7.34E-06 | -0.2527 | 0 | 0.129 |
| ENSP00000369317 | 5.96E-06 | -0.2527 | 0 | 0.324 |
| ENSP00000370077 | 2.29E-06 | -0.2527 | 0 | 0.122 |
| ENSP00000423049 | 3.92E-06 | -0.2528 | 0 | 0.000 |
| ENSP00000364220 | 3.29E-06 | -0.2528 | 0 | 0.532 |
| ENSP00000360054 | 5.71E-06 | -0.2528 | 0 | 0.133 |
| ENSP00000346088 | 6.35E-06 | -0.2529 | 156 | 0.748 |
| ENSP00000393183 | 4.35E-06 | -0.2530 | 0 | 0.466 |
| ENSP00000352734 | 3.27E-06 | -0.2530 | 0 | 0.137 |
| ENSP00000358107 | 4.73E-06 | -0.2530 | 0 | 0.080 |
| ENSP00000354769 | 4.61E-06 | -0.2530 | 0 | 0.000 |
| ENSP00000318429 | 3.03E-06 | -0.2530 | 0 | 0.056 |
| ENSP00000231484 | 1.15E-05 | -0.2530 | 0 | 0.177 |
| ENSP00000445340 | 1.37E-05 | -0.2530 | 184 | 0.106 |
| ENSP00000335620 | 9.05E-06 | -0.2531 | 159 | 0.347 |
| ENSP00000366988 | 5.20E-06 | -0.2531 | 0 | 0.091 |
| ENSP00000253079 | 1.68E-05 | -0.2531 | 198 | 0.114 |
| ENSP00000360270 | 1.24E-05 | -0.2531 | 340 | 0.285 |
| ENSP00000342143 | 7.85E-06 | -0.2532 | 267 | 0.166 |
| ENSP00000382133 | 4.57E-06 | -0.2532 | 0 | 0.723 |
| ENSP00000465075 | 2.53E-06 | -0.2532 | 0 | 0.267 |
| ENSP00000217320 | 6.30E-06 | -0.2532 | 0 | 0.159 |
| ENSP00000345689 | 7.30E-06 | -0.2533 | 184 | 0.000 |
| ENSP00000360973 | 1.11E-05 | -0.2533 | 780 | 0.456 |
| ENSP00000298047 | 1.03E-05 | -0.2533 | 151 | 0.000 |
| ENSP00000360327 | 3.80E-06 | -0.2533 | 0 | 0.493 |
| ENSP00000411584 | 2.14E-06 | -0.2534 | 0 | 0.121 |
| ENSP00000438144 | 1.34E-05 | -0.2534 | 464 | 0.507 |
| ENSP00000356047 | 5.32E-06 | -0.2534 | 174 | 0.311 |
| ENSP00000253934 | 9.72E-06 | -0.2534 | 0 | 0.158 |
| ENSP00000263174 | 7.60E-06 | -0.2534 | 0 | 0.146 |
| ENSP00000280734 | 3.99E-06 | -0.2534 | 0 | 0.117 |
| ENSP00000364737 | 1.75E-06 | -0.2534 | 0 | 0.307 |
| ENSP00000319623 | 5.05E-06 | -0.2534 | 0 | 0.296 |
| ENSP00000237353 | 6.71E-06 | -0.2535 | 0 | 0.211 |

| | | | | |
|---|---|---|---|---|
| ENSP00000438125 | 3.44E-06 | -0.2535 | 0 | 0.365 |
| ENSP00000440053 | 3.82E-06 | -0.2536 | 0 | 0.000 |
| ENSP00000330509 | 3.06E-06 | -0.2536 | 0 | 0.101 |
| ENSP00000422185 | 2.85E-06 | -0.2536 | 0 | 0.174 |
| ENSP00000401597 | 6.17E-07 | -0.2537 | 0 | 0.209 |
| ENSP00000443432 | 1.99E-06 | -0.2537 | 0 | 0.049 |
| ENSP00000248306 | 4.17E-06 | -0.2538 | 0 | 0.136 |
| ENSP00000370213 | 1.03E-05 | -0.2538 | 196 | 0.129 |
| ENSP00000345751 | 6.13E-06 | -0.2538 | 0 | 0.257 |
| ENSP00000227918 | 9.33E-06 | -0.2538 | 0 | 0.104 |
| ENSP00000365811 | 1.24E-05 | -0.2538 | 154 | 0.366 |
| ENSP00000421307 | 7.71E-07 | -0.2539 | 0 | 0.000 |
| ENSP00000377396 | 2.55E-06 | -0.2539 | 0 | 0.152 |
| ENSP00000295980 | 1.43E-05 | -0.2539 | 197 | 0.000 |
| ENSP00000302676 | 3.25E-06 | -0.2539 | 0 | 0.132 |
| ENSP00000394770 | 4.04E-06 | -0.2540 | 0 | 0.086 |
| ENSP00000355500 | 3.46E-06 | -0.2540 | 0 | 0.092 |
| ENSP00000334851 | 1.10E-05 | -0.2540 | 265 | 0.156 |
| ENSP00000245551 | 6.14E-06 | -0.2540 | 0 | 0.313 |
| ENSP00000251921 | 9.23E-06 | -0.2540 | 0 | 0.095 |
| ENSP00000261854 | 1.73E-05 | -0.2540 | 242 | 0.143 |
| ENSP00000418348 | 2.83E-06 | -0.2540 | 0 | 0.116 |
| ENSP00000335223 | 3.84E-06 | -0.2541 | 0 | 0.133 |
| ENSP00000355211 | 2.53E-06 | -0.2541 | 0 | 0.000 |
| ENSP00000328940 | 1.46E-05 | -0.2541 | 191 | 0.419 |
| ENSP00000279101 | 2.89E-06 | -0.2541 | 0 | 0.099 |
| ENSP00000362095 | 5.28E-06 | -0.2543 | 0 | 0.399 |
| ENSP00000245543 | 8.61E-06 | -0.2543 | 0 | 0.147 |
| ENSP00000383411 | 3.93E-06 | -0.2544 | 0 | 0.123 |
| ENSP00000263655 | 9.16E-06 | -0.2544 | 0 | 0.103 |
| ENSP00000364855 | 5.09E-06 | -0.2545 | 317 | 0.567 |
| ENSP00000278360 | 4.86E-06 | -0.2546 | 0 | 0.000 |
| ENSP00000377303 | 3.31E-06 | -0.2546 | 0 | 0.076 |
| ENSP00000397879 | 2.88E-06 | -0.2547 | 0 | 0.000 |
| ENSP00000326708 | 6.63E-06 | -0.2547 | 0 | 0.114 |
| ENSP00000351875 | 7.17E-06 | -0.2547 | 202 | 0.625 |
| ENSP00000289359 | 5.08E-06 | -0.2547 | 0 | 0.073 |
| ENSP00000382349 | 6.06E-06 | -0.2547 | 183 | 0.352 |
| ENSP00000261177 | 5.03E-06 | -0.2548 | 0 | 0.192 |
| ENSP00000408058 | 3.85E-06 | -0.2549 | 0 | 0.095 |
| ENSP00000368516 | 7.93E-06 | -0.2549 | 301 | 0.691 |
| ENSP00000352288 | 4.31E-06 | -0.2549 | 0 | 0.225 |
| ENSP00000359859 | 5.05E-06 | -0.2550 | 340 | 0.000 |
| ENSP00000375429 | 4.34E-06 | -0.2551 | 0 | 0.119 |
| ENSP00000403396 | 1.09E-06 | -0.2551 | 0 | 0.088 |
| ENSP00000385026 | 1.26E-05 | -0.2552 | 784 | 0.330 |

| | | | | |
|---|---|---|---|---|
| ENSP00000240123 | 7.09E-06 | -0.2553 | 0 | 0.333 |
| ENSP00000378408 | 9.13E-06 | -0.2554 | 351 | 0.334 |
| ENSP00000370224 | 6.92E-06 | -0.2554 | 0 | 0.103 |
| ENSP00000385478 | 3.53E-06 | -0.2554 | 0 | 0.216 |
| ENSP00000377934 | 3.62E-06 | -0.2554 | 0 | 0.316 |
| ENSP00000350018 | 3.07E-06 | -0.2554 | 0 | 0.267 |
| ENSP00000363041 | 2.06E-06 | -0.2555 | 0 | 0.082 |
| ENSP00000276173 | 2.00E-06 | -0.2555 | 0 | 0.252 |
| ENSP00000349957 | 4.70E-06 | -0.2555 | 0 | 0.447 |
| ENSP00000363557 | 3.82E-06 | -0.2555 | 0 | 0.088 |
| ENSP00000438788 | 3.25E-06 | -0.2555 | 0 | 0.463 |
| ENSP00000384345 | 5.19E-06 | -0.2556 | 0 | 0.390 |
| ENSP00000373404 | 5.19E-06 | -0.2557 | 375 | 0.795 |
| ENSP00000400010 | 8.84E-06 | -0.2557 | 212 | 0.723 |
| ENSP00000254901 | 3.80E-06 | -0.2557 | 0 | 0.052 |
| ENSP00000360552 | 3.59E-06 | -0.2557 | 0 | 0.770 |
| ENSP00000004103 | 6.02E-06 | -0.2558 | 0 | 0.143 |
| ENSP00000351503 | 4.15E-06 | -0.2558 | 0 | 0.116 |
| ENSP00000457016 | 2.35E-06 | -0.2558 | 0 | 0.351 |
| ENSP00000360129 | 3.80E-06 | -0.2558 | 153 | 0.000 |
| ENSP00000350934 | 4.42E-06 | -0.2559 | 0 | 0.113 |
| ENSP00000330787 | 3.47E-06 | -0.2559 | 0 | 0.108 |
| ENSP00000354536 | 5.61E-06 | -0.2559 | 0 | 0.201 |
| ENSP00000309524 | 7.61E-06 | -0.2559 | 0 | 0.281 |
| ENSP00000445063 | 5.80E-07 | -0.2560 | 0 | 0.000 |
| ENSP00000375095 | 5.54E-06 | -0.2560 | 0 | 0.148 |
| ENSP00000454919 | 5.31E-06 | -0.2561 | 0 | 0.308 |
| ENSP00000395962 | 2.86E-06 | -0.2561 | 0 | 0.000 |
| ENSP00000384774 | 1.31E-05 | -0.2561 | 261 | 0.000 |
| ENSP00000289989 | 4.80E-06 | -0.2561 | 0 | 0.162 |
| ENSP00000367934 | 3.11E-06 | -0.2562 | 0 | 0.193 |
| ENSP00000386389 | 1.13E-05 | -0.2562 | 0 | 0.172 |
| ENSP00000419194 | 2.10E-06 | -0.2562 | 0 | 0.090 |
| ENSP00000299665 | 1.26E-05 | -0.2562 | 0 | 0.083 |
| ENSP00000379709 | 2.18E-06 | -0.2562 | 0 | 0.143 |
| ENSP00000265634 | 1.39E-05 | -0.2563 | 0 | 0.204 |
| ENSP00000380762 | 2.50E-06 | -0.2563 | 0 | 0.108 |
| ENSP00000425107 | 5.93E-06 | -0.2563 | 397 | 0.498 |
| ENSP00000380488 | 2.00E-06 | -0.2563 | 0 | 0.177 |
| ENSP00000442068 | 1.51E-06 | -0.2563 | 0 | 0.294 |
| ENSP00000381634 | 2.76E-06 | -0.2564 | 0 | 0.069 |
| ENSP00000360472 | 4.30E-06 | -0.2564 | 0 | 0.353 |
| ENSP00000253807 | 6.45E-06 | -0.2564 | 0 | 0.087 |
| ENSP00000253381 | 6.79E-06 | -0.2564 | 0 | 0.114 |
| ENSP00000331849 | 2.75E-06 | -0.2565 | 0 | 0.427 |
| ENSP00000396452 | 3.15E-06 | -0.2565 | 0 | 0.000 |

| | | | | |
|---|---|---|---|---|
| ENSP00000342313 | 4.44E-06 | -0.2565 | 0 | 0.139 |
| ENSP00000382058 | 2.50E-06 | -0.2565 | 0 | 0.396 |
| ENSP00000352463 | 6.68E-06 | -0.2566 | 0 | 0.284 |
| ENSP00000362135 | 4.89E-06 | -0.2566 | 0 | 0.453 |
| ENSP00000295694 | 7.39E-06 | -0.2566 | 0 | 0.091 |
| ENSP00000326247 | 5.59E-06 | -0.2567 | 0 | 0.102 |
| ENSP00000333490 | 5.68E-06 | -0.2567 | 0 | 0.000 |
| ENSP00000259456 | 7.58E-06 | -0.2567 | 0 | 0.425 |
| ENSP00000377051 | 1.03E-05 | -0.2567 | 609 | 0.696 |
| ENSP00000265272 | 4.17E-06 | -0.2567 | 0 | 0.132 |
| ENSP00000296444 | 6.44E-06 | -0.2568 | 0 | 0.096 |
| ENSP00000447000 | 4.22E-06 | -0.2568 | 0 | 0.000 |
| ENSP00000378167 | 1.93E-06 | -0.2569 | 0 | 0.134 |
| ENSP00000304327 | 6.02E-06 | -0.2569 | 0 | 0.225 |
| ENSP00000383719 | 3.06E-06 | -0.2569 | 0 | 0.110 |
| ENSP00000010132 | 5.22E-06 | -0.2569 | 189 | 0.667 |
| ENSP00000225899 | 2.47E-06 | -0.2570 | 0 | 0.117 |
| ENSP00000364389 | 3.36E-06 | -0.2570 | 0 | 0.000 |
| ENSP00000363709 | 5.12E-06 | -0.2570 | 0 | 0.000 |
| ENSP00000434279 | 7.06E-06 | -0.2570 | 0 | 0.243 |
| ENSP00000332530 | 4.45E-06 | -0.2571 | 0 | 0.119 |
| ENSP00000359640 | 7.66E-06 | -0.2571 | 0 | 0.091 |
| ENSP00000323663 | 2.67E-06 | -0.2572 | 0 | 0.584 |
| ENSP00000369299 | 4.33E-06 | -0.2572 | 0 | 0.366 |
| ENSP00000376945 | 1.65E-06 | -0.2572 | 0 | 0.136 |
| ENSP00000315949 | 1.16E-05 | -0.2572 | 151 | 0.816 |
| ENSP00000249887 | 1.57E-05 | -0.2572 | 0 | 0.000 |
| ENSP00000368207 | 4.51E-06 | -0.2572 | 225 | 0.230 |
| ENSP00000380679 | 6.27E-06 | -0.2573 | 0 | 0.059 |
| ENSP00000425786 | 1.42E-06 | -0.2573 | 0 | 0.108 |
| ENSP00000385450 | 4.63E-06 | -0.2573 | 0 | 0.465 |
| ENSP00000396040 | 3.90E-06 | -0.2573 | 0 | 0.109 |
| ENSP00000454786 | 2.77E-06 | -0.2574 | 0 | 0.000 |
| ENSP00000271064 | 9.93E-06 | -0.2574 | 0 | 0.121 |
| ENSP00000415823 | 6.43E-06 | -0.2575 | 0 | 0.127 |
| ENSP00000392452 | 8.29E-07 | -0.2575 | 0 | 0.163 |
| ENSP00000366249 | 2.42E-05 | -0.2575 | 295 | 0.656 |
| ENSP00000348901 | 4.18E-06 | -0.2575 | 0 | 0.079 |
| ENSP00000297977 | 1.11E-05 | -0.2575 | 169 | 0.088 |
| ENSP00000383939 | 1.91E-06 | -0.2576 | 0 | 0.326 |
| ENSP00000402338 | 1.38E-05 | -0.2576 | 170 | 0.918 |
| ENSP00000393198 | 5.87E-06 | -0.2576 | 0 | 0.771 |
| ENSP00000356087 | 3.45E-06 | -0.2576 | 0 | 0.000 |
| ENSP00000360149 | 3.98E-06 | -0.2577 | 0 | 0.142 |
| ENSP00000360652 | 2.76E-06 | -0.2577 | 0 | 0.000 |
| ENSP00000259211 | 1.30E-05 | -0.2577 | 0 | 0.206 |

| | | | | |
|---|---|---|---|---|
| ENSP00000308901 | 2.56E-06 | -0.2578 | 0 | 0.000 |
| ENSP00000404011 | 1.51E-06 | -0.2578 | 0 | 0.000 |
| ENSP00000378254 | 2.83E-06 | -0.2578 | 0 | 0.104 |
| ENSP00000414851 | 4.28E-06 | -0.2578 | 0 | 0.680 |
| ENSP00000363162 | 1.65E-06 | -0.2578 | 0 | 0.047 |
| ENSP00000287474 | 4.48E-06 | -0.2579 | 0 | 0.102 |
| ENSP00000319817 | 5.05E-06 | -0.2579 | 0 | 0.067 |
| ENSP00000434466 | 1.99E-06 | -0.2579 | 0 | 0.111 |
| ENSP00000377840 | 3.00E-06 | -0.2579 | 0 | 0.247 |
| ENSP00000324741 | 8.76E-06 | -0.2580 | 0 | 0.168 |
| ENSP00000371201 | 1.64E-05 | -0.2580 | 184 | 0.835 |
| ENSP00000363899 | 4.25E-06 | -0.2581 | 0 | 0.091 |
| ENSP00000374484 | 3.61E-06 | -0.2581 | 0 | 0.070 |
| ENSP00000364938 | 4.20E-06 | -0.2581 | 0 | 0.162 |
| ENSP00000370849 | 1.81E-06 | -0.2582 | 0 | 0.146 |
| ENSP00000363582 | 1.39E-06 | -0.2582 | 0 | 0.000 |
| ENSP00000354107 | 4.47E-06 | -0.2582 | 0 | 0.000 |
| ENSP00000323926 | 7.26E-06 | -0.2582 | 0 | 0.307 |
| ENSP00000417601 | 6.57E-06 | -0.2583 | 226 | 0.315 |
| ENSP00000359939 | 3.85E-06 | -0.2583 | 0 | 0.856 |
| ENSP00000389039 | 3.97E-06 | -0.2583 | 0 | 0.000 |
| ENSP00000370270 | 4.72E-06 | -0.2583 | 0 | 0.822 |
| ENSP00000355927 | 3.20E-06 | -0.2584 | 0 | 0.171 |
| ENSP00000416673 | 1.42E-06 | -0.2584 | 0 | 0.168 |
| ENSP00000356587 | 4.28E-06 | -0.2584 | 0 | 0.187 |
| ENSP00000328016 | 2.83E-06 | -0.2584 | 0 | 0.110 |
| ENSP00000315731 | 7.87E-06 | -0.2585 | 0 | 0.105 |
| ENSP00000348278 | 7.51E-06 | -0.2585 | 0 | 0.334 |
| ENSP00000352447 | 4.12E-06 | -0.2585 | 0 | 0.186 |
| ENSP00000338556 | 2.90E-06 | -0.2586 | 0 | 0.088 |
| ENSP00000354518 | 2.41E-06 | -0.2586 | 0 | 0.575 |
| ENSP00000405038 | 4.04E-06 | -0.2586 | 205 | 0.000 |
| ENSP00000301488 | 6.40E-06 | -0.2587 | 0 | 0.205 |
| ENSP00000271139 | 6.87E-06 | -0.2587 | 0 | 0.126 |
| ENSP00000370022 | 5.85E-06 | -0.2587 | 0 | 0.581 |
| ENSP00000370718 | 3.87E-06 | -0.2587 | 0 | 0.127 |
| ENSP00000404079 | 1.08E-05 | -0.2588 | 905 | 0.333 |
| ENSP00000329199 | 2.90E-06 | -0.2589 | 0 | 0.241 |
| ENSP00000362328 | 2.53E-06 | -0.2589 | 0 | 0.092 |
| ENSP00000251303 | 1.34E-05 | -0.2589 | 191 | 0.426 |
| ENSP00000355384 | 3.49E-06 | -0.2590 | 0 | 0.066 |
| ENSP00000420449 | 6.68E-06 | -0.2591 | 173 | 0.000 |
| ENSP00000349291 | 2.39E-06 | -0.2592 | 0 | 0.146 |
| ENSP00000457654 | 1.26E-07 | -0.2592 | 0 | 0.000 |
| ENSP00000449629 | 9.00E-06 | -0.2593 | 201 | 0.610 |
| ENSP00000357306 | 1.49E-05 | -0.2593 | 485 | 0.614 |

| | | | | |
|---|---|---|---|---|
| ENSP00000310770 | 7.76E-06 | -0.2593 | 0 | 0.098 |
| ENSP00000331608 | 1.56E-05 | -0.2594 | 191 | 0.812 |
| ENSP00000358529 | 3.44E-06 | -0.2594 | 0 | 0.124 |
| ENSP00000205386 | 1.57E-05 | -0.2594 | 0 | 0.167 |
| ENSP00000391564 | 2.86E-06 | -0.2595 | 0 | 0.000 |
| ENSP00000348107 | 4.96E-06 | -0.2596 | 0 | 0.732 |
| ENSP00000371036 | 2.16E-06 | -0.2596 | 0 | 0.161 |
| ENSP00000356936 | 3.39E-06 | -0.2598 | 0 | 0.214 |
| ENSP00000296877 | 5.78E-06 | -0.2598 | 0 | 0.092 |
| ENSP00000257264 | 6.94E-06 | -0.2598 | 0 | 0.107 |
| ENSP00000331678 | 1.02E-05 | -0.2599 | 169 | 0.556 |
| ENSP00000327234 | 3.56E-06 | -0.2599 | 0 | 0.143 |
| ENSP00000388631 | 6.57E-06 | -0.2599 | 0 | 0.428 |
| ENSP00000458791 | 8.66E-06 | -0.2599 | 292 | 0.199 |
| ENSP00000462747 | 1.24E-06 | -0.2599 | 0 | 0.000 |
| ENSP00000405424 | 2.32E-06 | -0.2599 | 0 | 0.070 |
| ENSP00000335675 | 6.46E-06 | -0.2599 | 0 | 0.133 |
| ENSP00000367950 | 4.31E-06 | -0.2600 | 0 | 0.140 |
| ENSP00000324403 | 3.75E-06 | -0.2600 | 0 | 0.161 |
| ENSP00000365630 | 3.43E-06 | -0.2601 | 0 | 0.225 |
| ENSP00000345487 | 4.36E-06 | -0.2601 | 0 | 0.468 |
| ENSP00000371594 | 4.32E-06 | -0.2602 | 0 | 0.376 |
| ENSP00000274710 | 3.45E-06 | -0.2602 | 0 | 0.125 |
| ENSP00000422473 | 3.37E-06 | -0.2602 | 0 | 0.176 |
| ENSP00000367284 | 2.10E-06 | -0.2603 | 0 | 0.149 |
| ENSP00000393776 | 2.68E-06 | -0.2604 | 0 | 0.270 |
| ENSP00000361681 | 2.12E-06 | -0.2604 | 0 | 0.000 |
| ENSP00000297029 | 1.38E-05 | -0.2604 | 217 | 0.395 |
| ENSP00000397259 | 2.42E-06 | -0.2604 | 0 | 0.851 |
| ENSP00000354972 | 3.27E-06 | -0.2604 | 0 | 0.138 |
| ENSP00000350491 | 2.62E-06 | -0.2604 | 0 | 0.626 |
| ENSP00000375430 | 4.24E-06 | -0.2604 | 0 | 0.137 |
| ENSP00000264079 | 1.24E-05 | -0.2604 | 242 | 0.191 |
| ENSP00000443900 | 2.83E-06 | -0.2605 | 0 | 0.143 |
| ENSP00000383611 | 4.16E-06 | -0.2605 | 0 | 0.637 |
| ENSP00000311364 | 5.22E-06 | -0.2605 | 0 | 0.100 |
| ENSP00000375238 | 3.64E-06 | -0.2605 | 0 | 0.093 |
| ENSP00000380690 | 5.22E-06 | -0.2605 | 0 | 0.117 |
| ENSP00000417052 | 3.13E-06 | -0.2605 | 0 | 0.079 |
| ENSP00000376372 | 4.65E-06 | -0.2605 | 0 | 0.078 |
| ENSP00000349595 | 7.30E-06 | -0.2605 | 309 | 0.630 |
| ENSP00000355471 | 1.05E-05 | -0.2606 | 388 | 0.835 |
| ENSP00000355157 | 3.22E-06 | -0.2606 | 0 | 0.116 |
| ENSP00000250823 | 4.76E-06 | -0.2606 | 0 | 0.226 |
| ENSP00000254337 | 2.23E-06 | -0.2607 | 0 | 0.176 |
| ENSP00000245923 | 3.74E-06 | -0.2607 | 0 | 0.095 |

| | | | | |
|---|---|---|---|---|
| ENSP00000275884 | 3.87E-06 | -0.2607 | 0 | 0.110 |
| ENSP00000381003 | 8.80E-06 | -0.2607 | 683 | 0.705 |
| ENSP00000392985 | 2.50E-06 | -0.2608 | 0 | 0.364 |
| ENSP00000345659 | 6.03E-06 | -0.2608 | 0 | 0.154 |
| ENSP00000395535 | 4.55E-06 | -0.2608 | 0 | 0.771 |
| ENSP00000363645 | 3.01E-06 | -0.2609 | 0 | 0.166 |
| ENSP00000283285 | 4.28E-06 | -0.2609 | 0 | 0.112 |
| ENSP00000352021 | 2.40E-06 | -0.2609 | 0 | 0.762 |
| ENSP00000347170 | 4.66E-06 | -0.2609 | 0 | 0.350 |
| ENSP00000332875 | 4.44E-06 | -0.2609 | 0 | 0.142 |
| ENSP00000265707 | 7.69E-06 | -0.2609 | 0 | 0.154 |
| ENSP00000456615 | 9.85E-07 | -0.2610 | 0 | 0.000 |
| ENSP00000356133 | 4.66E-06 | -0.2610 | 0 | 0.143 |
| ENSP00000454833 | 1.98E-06 | -0.2611 | 0 | 0.000 |
| ENSP00000348645 | 5.37E-06 | -0.2611 | 0 | 0.135 |
| ENSP00000434724 | 2.27E-06 | -0.2612 | 0 | 0.094 |
| ENSP00000327133 | 3.77E-06 | -0.2612 | 0 | 0.058 |
| ENSP00000296529 | 5.86E-06 | -0.2612 | 0 | 0.096 |
| ENSP00000295452 | 1.18E-05 | -0.2613 | 227 | 0.315 |
| ENSP00000417963 | 5.13E-06 | -0.2613 | 0 | 0.608 |
| ENSP00000360483 | 3.00E-06 | -0.2613 | 0 | 0.000 |
| ENSP00000360133 | 3.02E-06 | -0.2613 | 0 | 0.373 |
| ENSP00000352547 | 3.25E-06 | -0.2614 | 0 | 0.251 |
| ENSP00000260447 | 8.11E-06 | -0.2614 | 0 | 0.081 |
| ENSP00000356807 | 4.87E-06 | -0.2614 | 0 | 0.279 |
| ENSP00000359423 | 3.72E-06 | -0.2614 | 0 | 0.157 |
| ENSP00000370430 | 4.58E-06 | -0.2615 | 0 | 0.118 |
| ENSP00000243286 | 6.84E-06 | -0.2615 | 0 | 0.133 |
| ENSP00000363400 | 5.19E-06 | -0.2616 | 0 | 0.072 |
| ENSP00000381010 | 2.25E-06 | -0.2617 | 0 | 0.179 |
| ENSP00000303206 | 1.15E-05 | -0.2617 | 0 | 0.414 |
| ENSP00000322273 | 8.15E-06 | -0.2617 | 0 | 0.094 |
| ENSP00000387233 | 7.11E-06 | -0.2617 | 202 | 0.640 |
| ENSP00000370299 | 2.97E-06 | -0.2617 | 0 | 0.107 |
| ENSP00000305964 | 3.61E-06 | -0.2618 | 0 | 0.000 |
| ENSP00000358571 | 6.35E-06 | -0.2619 | 189 | 0.679 |
| ENSP00000351933 | 2.81E-06 | -0.2620 | 0 | 0.179 |
| ENSP00000460274 | 1.14E-06 | -0.2620 | 0 | 0.151 |
| ENSP00000387281 | 8.70E-06 | -0.2620 | 359 | 0.000 |
| ENSP00000355559 | 2.66E-06 | -0.2620 | 0 | 0.065 |
| ENSP00000332900 | 6.34E-06 | -0.2620 | 0 | 0.000 |
| ENSP00000373399 | 3.17E-06 | -0.2621 | 0 | 0.404 |
| ENSP00000358888 | 3.19E-06 | -0.2621 | 0 | 0.114 |
| ENSP00000386049 | 1.89E-06 | -0.2622 | 0 | 0.185 |
| ENSP00000216799 | 5.64E-06 | -0.2622 | 0 | 0.175 |
| ENSP00000323034 | 4.70E-06 | -0.2622 | 0 | 0.000 |

| | | | | |
|---|---|---|---|---|
| ENSP00000347408 | 3.31E-06 | -0.2623 | 0 | 0.107 |
| ENSP00000360035 | 2.46E-06 | -0.2623 | 0 | 0.092 |
| ENSP00000429834 | 1.96E-06 | -0.2623 | 0 | 0.154 |
| ENSP00000288710 | 3.60E-06 | -0.2623 | 0 | 0.263 |
| ENSP00000265381 | 7.27E-06 | -0.2624 | 171 | 0.228 |
| ENSP00000334611 | 7.91E-06 | -0.2624 | 0 | 0.174 |
| ENSP00000468690 | 1.05E-05 | -0.2624 | 201 | 0.000 |
| ENSP00000312631 | 5.85E-06 | -0.2625 | 0 | 0.710 |
| ENSP00000318113 | 7.40E-06 | -0.2626 | 0 | 0.133 |
| ENSP00000262995 | 7.31E-06 | -0.2626 | 212 | 0.715 |
| ENSP00000455826 | 9.83E-07 | -0.2626 | 0 | 0.155 |
| ENSP00000451511 | 1.48E-05 | -0.2627 | 202 | 0.000 |
| ENSP00000370710 | 3.26E-06 | -0.2627 | 0 | 0.842 |
| ENSP00000245615 | 7.68E-06 | -0.2627 | 0 | 0.117 |
| ENSP00000363395 | 7.34E-06 | -0.2628 | 153 | 0.000 |
| ENSP00000335094 | 1.86E-06 | -0.2628 | 0 | 0.094 |
| ENSP00000365413 | 3.60E-06 | -0.2628 | 0 | 0.057 |
| ENSP00000262545 | 1.09E-05 | -0.2629 | 498 | 0.634 |
| ENSP00000414635 | 2.79E-06 | -0.2629 | 0 | 0.108 |
| ENSP00000231136 | 6.76E-06 | -0.2629 | 0 | 0.119 |
| ENSP00000361057 | 5.21E-06 | -0.2629 | 279 | 0.179 |
| ENSP00000377640 | 9.31E-06 | -0.2629 | 191 | 0.880 |
| ENSP00000296059 | 2.82E-06 | -0.2630 | 0 | 0.196 |
| ENSP00000334294 | 1.08E-05 | -0.2630 | 150 | 0.392 |
| ENSP00000383599 | 1.84E-06 | -0.2630 | 0 | 0.116 |
| ENSP00000349902 | 4.99E-06 | -0.2630 | 0 | 0.000 |
| ENSP00000265299 | 6.02E-06 | -0.2630 | 0 | 0.164 |
| ENSP00000338276 | 1.14E-05 | -0.2631 | 340 | 0.071 |
| ENSP00000319651 | 1.04E-05 | -0.2632 | 211 | 0.574 |
| ENSP00000345988 | 6.88E-06 | -0.2632 | 0 | 0.373 |
| ENSP00000461862 | 1.60E-06 | -0.2633 | 0 | 0.125 |
| ENSP00000393832 | 3.53E-06 | -0.2633 | 0 | 0.111 |
| ENSP00000408206 | 1.10E-06 | -0.2634 | 0 | 0.000 |
| ENSP00000363216 | 6.09E-06 | -0.2634 | 224 | 0.399 |
| ENSP00000316130 | 8.72E-06 | -0.2635 | 0 | 0.077 |
| ENSP00000408005 | 5.59E-06 | -0.2635 | 0 | 0.563 |
| ENSP00000354498 | 2.96E-06 | -0.2635 | 0 | 0.097 |
| ENSP00000346691 | 5.01E-06 | -0.2636 | 0 | 0.000 |
| ENSP00000299698 | 7.56E-06 | -0.2636 | 0 | 0.090 |
| ENSP00000356470 | 3.90E-06 | -0.2636 | 0 | 0.149 |
| ENSP00000354501 | 3.39E-06 | -0.2637 | 0 | 0.313 |
| ENSP00000369080 | 2.21E-06 | -0.2637 | 0 | 0.104 |
| ENSP00000338260 | 6.88E-06 | -0.2637 | 163 | 0.229 |
| ENSP00000374490 | 3.18E-06 | -0.2638 | 0 | 0.126 |
| ENSP00000313506 | 2.97E-06 | -0.2639 | 0 | 0.259 |
| ENSP00000297788 | 6.42E-06 | -0.2639 | 0 | 0.110 |

| | | | | |
|---|---|---|---|---|
| ENSP00000295927 | 1.39E-05 | -0.2639 | 0 | 0.184 |
| ENSP00000312021 | 8.41E-06 | -0.2640 | 250 | 0.102 |
| ENSP00000341021 | 4.29E-06 | -0.2640 | 0 | 0.521 |
| ENSP00000320038 | 3.70E-06 | -0.2640 | 0 | 0.322 |
| ENSP00000352314 | 5.32E-06 | -0.2642 | 0 | 0.172 |
| ENSP00000320649 | 4.83E-06 | -0.2642 | 0 | 0.133 |
| ENSP00000366121 | 7.99E-06 | -0.2642 | 0 | 0.234 |
| ENSP00000420863 | 3.93E-06 | -0.2642 | 0 | 0.097 |
| ENSP00000359540 | 4.28E-06 | -0.2643 | 0 | 0.130 |
| ENSP00000329318 | 4.50E-06 | -0.2644 | 0 | 0.126 |
| ENSP00000222674 | 1.01E-05 | -0.2644 | 0 | 0.253 |
| ENSP00000224756 | 4.92E-06 | -0.2644 | 0 | 0.104 |
| ENSP00000467494 | 8.88E-06 | -0.2644 | 190 | 0.545 |
| ENSP00000393583 | 4.05E-06 | -0.2644 | 0 | 0.000 |
| ENSP00000432016 | 7.55E-06 | -0.2644 | 202 | 0.665 |
| ENSP00000344855 | 5.05E-06 | -0.2645 | 0 | 0.167 |
| ENSP00000367059 | 7.22E-06 | -0.2645 | 202 | 0.780 |
| ENSP00000371897 | 4.00E-06 | -0.2646 | 0 | 0.586 |
| ENSP00000380495 | 5.56E-06 | -0.2646 | 0 | 0.871 |
| ENSP00000383223 | 2.94E-06 | -0.2646 | 0 | 0.157 |
| ENSP00000449535 | 5.09E-06 | -0.2646 | 183 | 0.336 |
| ENSP00000356104 | 3.62E-06 | -0.2647 | 0 | 0.179 |
| ENSP00000218008 | 7.41E-06 | -0.2649 | 0 | 0.085 |
| ENSP00000349932 | 7.75E-06 | -0.2649 | 0 | 0.700 |
| ENSP00000367137 | 2.36E-06 | -0.2650 | 0 | 0.069 |
| ENSP00000461269 | 2.56E-06 | -0.2650 | 0 | 0.126 |
| ENSP00000455444 | 3.78E-06 | -0.2650 | 0 | 0.624 |
| ENSP00000392066 | 4.12E-06 | -0.2650 | 0 | 0.149 |
| ENSP00000369176 | 2.78E-06 | -0.2650 | 0 | 0.252 |
| ENSP00000428603 | 2.14E-06 | -0.2651 | 0 | 0.143 |
| ENSP00000472933 | 2.41E-06 | -0.2652 | 0 | 0.000 |
| ENSP00000254528 | 8.02E-06 | -0.2652 | 0 | 0.162 |
| ENSP00000364524 | 1.27E-05 | -0.2652 | 201 | 0.865 |
| ENSP00000381968 | 7.38E-06 | -0.2653 | 220 | 0.336 |
| ENSP00000362021 | 3.00E-06 | -0.2653 | 0 | 0.106 |
| ENSP00000372750 | 1.53E-05 | -0.2654 | 185 | 0.498 |
| ENSP00000283233 | 7.64E-06 | -0.2654 | 0 | 0.133 |
| ENSP00000356733 | 6.49E-07 | -0.2655 | 0 | 0.214 |
| ENSP00000339802 | 5.51E-06 | -0.2656 | 0 | 0.111 |
| ENSP00000447001 | 6.68E-06 | -0.2657 | 199 | 0.820 |
| ENSP00000363345 | 1.42E-06 | -0.2657 | 0 | 0.190 |
| ENSP00000420659 | 1.37E-06 | -0.2657 | 0 | 0.196 |
| ENSP00000364271 | 4.07E-06 | -0.2658 | 0 | 0.193 |
| ENSP00000447852 | 1.02E-06 | -0.2659 | 0 | 0.118 |
| ENSP00000362071 | 4.26E-06 | -0.2659 | 0 | 0.126 |
| ENSP00000431876 | 1.94E-06 | -0.2660 | 0 | 0.151 |

| | | | | |
|---|---|---|---|---|
| ENSP00000413001 | 3.13E-06 | -0.2660 | 0 | 0.081 |
| ENSP00000330746 | 2.62E-06 | -0.2660 | 0 | 0.143 |
| ENSP00000295213 | 6.58E-06 | -0.2660 | 0 | 0.109 |
| ENSP00000253110 | 1.60E-06 | -0.2661 | 0 | 0.147 |
| ENSP00000323424 | 1.00E-05 | -0.2661 | 205 | 0.835 |
| ENSP00000354490 | 5.97E-06 | -0.2661 | 189 | 0.387 |
| ENSP00000314484 | 2.29E-06 | -0.2661 | 0 | 0.129 |
| ENSP00000352849 | 4.70E-06 | -0.2661 | 0 | 0.127 |
| ENSP00000420132 | 4.70E-06 | -0.2662 | 0 | 0.069 |
| ENSP00000347767 | 3.32E-06 | -0.2662 | 0 | 0.087 |
| ENSP00000410759 | 2.76E-06 | -0.2663 | 0 | 0.769 |
| ENSP00000362058 | 3.24E-06 | -0.2663 | 0 | 0.072 |
| ENSP00000360944 | 2.92E-06 | -0.2663 | 0 | 0.253 |
| ENSP00000376799 | 1.66E-06 | -0.2663 | 0 | 0.105 |
| ENSP00000351644 | 5.06E-06 | -0.2664 | 313 | 0.583 |
| ENSP00000366886 | 4.64E-06 | -0.2664 | 0 | 0.092 |
| ENSP00000408295 | 6.03E-06 | -0.2665 | 201 | 0.715 |
| ENSP00000431116 | 2.00E-06 | -0.2665 | 0 | 0.000 |
| ENSP00000376548 | 2.39E-06 | -0.2666 | 0 | 0.139 |
| ENSP00000358886 | 3.90E-06 | -0.2666 | 0 | 0.647 |
| ENSP00000299295 | 3.77E-06 | -0.2666 | 0 | 0.099 |
| ENSP00000258456 | 1.10E-05 | -0.2667 | 0 | 0.142 |
| ENSP00000447879 | 3.05E-06 | -0.2668 | 0 | 0.152 |
| ENSP00000322339 | 2.73E-06 | -0.2668 | 0 | 0.155 |
| ENSP00000381628 | 3.02E-06 | -0.2668 | 0 | 0.115 |
| ENSP00000385916 | 2.50E-06 | -0.2669 | 0 | 0.000 |
| ENSP00000403208 | 3.19E-06 | -0.2669 | 0 | 0.048 |
| ENSP00000417061 | 1.62E-06 | -0.2669 | 0 | 0.000 |
| ENSP00000387512 | 1.23E-06 | -0.2670 | 0 | 0.000 |
| ENSP00000358451 | 2.59E-06 | -0.2670 | 0 | 0.218 |
| ENSP00000333592 | 9.63E-06 | -0.2670 | 0 | 0.000 |
| ENSP00000368511 | 1.65E-06 | -0.2670 | 0 | 0.153 |
| ENSP00000349877 | 5.25E-06 | -0.2670 | 177 | 0.377 |
| ENSP00000381856 | 2.19E-06 | -0.2672 | 0 | 0.131 |
| ENSP00000264400 | 1.26E-05 | -0.2672 | 251 | 0.503 |
| ENSP00000416168 | 1.74E-06 | -0.2673 | 0 | 0.139 |
| ENSP00000351086 | 4.06E-06 | -0.2673 | 0 | 0.000 |
| ENSP00000446654 | 1.00E-06 | -0.2673 | 0 | 0.000 |
| ENSP00000350136 | 4.24E-06 | -0.2674 | 221 | 0.118 |
| ENSP00000262932 | 7.96E-06 | -0.2674 | 0 | 0.092 |
| ENSP00000354109 | 3.69E-06 | -0.2674 | 0 | 0.111 |
| ENSP00000365243 | 2.75E-06 | -0.2674 | 0 | 0.302 |
| ENSP00000411471 | 4.65E-06 | -0.2675 | 150 | 0.694 |
| ENSP00000312284 | 1.38E-05 | -0.2675 | 159 | 0.199 |
| ENSP00000358126 | 3.19E-06 | -0.2675 | 0 | 0.235 |
| ENSP00000305899 | 3.27E-06 | -0.2676 | 0 | 0.807 |

| | | | | |
|---|---|---|---|---|
| ENSP00000386588 | 3.94E-06 | -0.2676 | 0 | 0.711 |
| ENSP00000261377 | 1.32E-05 | -0.2676 | 205 | 0.000 |
| ENSP00000367323 | 8.10E-06 | -0.2676 | 195 | 0.160 |
| ENSP00000308354 | 3.68E-06 | -0.2676 | 0 | 0.149 |
| ENSP00000467958 | 2.11E-06 | -0.2677 | 0 | 0.000 |
| ENSP00000302177 | 3.92E-06 | -0.2677 | 0 | 0.122 |
| ENSP00000357112 | 4.16E-06 | -0.2677 | 0 | 0.556 |
| ENSP00000340396 | 6.59E-06 | -0.2677 | 0 | 0.000 |
| ENSP00000366460 | 2.64E-06 | -0.2677 | 0 | 0.111 |
| ENSP00000383143 | 3.82E-06 | -0.2677 | 0 | 0.176 |
| ENSP00000407925 | 1.93E-06 | -0.2677 | 0 | 0.000 |
| ENSP00000438349 | 3.76E-06 | -0.2678 | 0 | 0.861 |
| ENSP00000359788 | 2.73E-06 | -0.2678 | 0 | 0.335 |
| ENSP00000356779 | 4.94E-06 | -0.2678 | 0 | 0.082 |
| ENSP00000348314 | 2.12E-06 | -0.2678 | 0 | 0.092 |
| ENSP00000349705 | 5.42E-06 | -0.2679 | 256 | 0.658 |
| ENSP00000386911 | 4.35E-06 | -0.2679 | 0 | 0.379 |
| ENSP00000427366 | 3.91E-06 | -0.2680 | 0 | 0.564 |
| ENSP00000423777 | 3.91E-06 | -0.2680 | 0 | 0.564 |
| ENSP00000228936 | 1.17E-05 | -0.2680 | 0 | 0.247 |
| ENSP00000423211 | 3.91E-06 | -0.2680 | 0 | 0.564 |
| ENSP00000360485 | 1.89E-06 | -0.2680 | 0 | 0.140 |
| ENSP00000422969 | 3.92E-06 | -0.2681 | 0 | 0.564 |
| ENSP00000422097 | 3.92E-06 | -0.2681 | 0 | 0.564 |
| ENSP00000422887 | 3.92E-06 | -0.2681 | 0 | 0.564 |
| ENSP00000289388 | 1.01E-05 | -0.2681 | 0 | 0.000 |
| ENSP00000216468 | 3.33E-06 | -0.2681 | 0 | 0.181 |
| ENSP00000330267 | 3.57E-06 | -0.2681 | 0 | 0.000 |
| ENSP00000386227 | 3.52E-06 | -0.2681 | 0 | 0.108 |
| ENSP00000360366 | 6.94E-06 | -0.2681 | 211 | 0.139 |
| ENSP00000297109 | 3.94E-06 | -0.2682 | 0 | 0.634 |
| ENSP00000403852 | 9.76E-06 | -0.2682 | 371 | 0.810 |
| ENSP00000345270 | 6.49E-06 | -0.2682 | 0 | 0.186 |
| ENSP00000345580 | 3.05E-06 | -0.2683 | 0 | 0.000 |
| ENSP00000317564 | 5.64E-06 | -0.2683 | 0 | 0.116 |
| ENSP00000265598 | 1.39E-05 | -0.2684 | 569 | 0.440 |
| ENSP00000358265 | 2.96E-06 | -0.2685 | 0 | 0.180 |
| ENSP00000330138 | 1.07E-05 | -0.2685 | 224 | 0.000 |
| ENSP00000352097 | 8.59E-06 | -0.2685 | 0 | 0.078 |
| ENSP00000365991 | 6.42E-06 | -0.2685 | 917 | 0.495 |
| ENSP00000329735 | 5.12E-06 | -0.2685 | 0 | 0.128 |
| ENSP00000312439 | 2.63E-06 | -0.2685 | 0 | 0.126 |
| ENSP00000369251 | 6.08E-06 | -0.2686 | 0 | 0.386 |
| ENSP00000366697 | 6.99E-06 | -0.2686 | 202 | 0.000 |
| ENSP00000341674 | 8.77E-06 | -0.2687 | 153 | 0.000 |
| ENSP00000297203 | 1.93E-06 | -0.2687 | 0 | 0.189 |

| | | | | |
|---|---|---|---|---|
| ENSP00000377401 | 4.09E-06 | -0.2687 | 0 | 0.179 |
| ENSP00000453785 | 8.41E-06 | -0.2687 | 220 | 0.182 |
| ENSP00000443130 | 1.99E-06 | -0.2688 | 0 | 0.000 |
| ENSP00000233027 | 6.21E-06 | -0.2688 | 0 | 0.164 |
| ENSP00000404860 | 2.50E-06 | -0.2688 | 0 | 0.112 |
| ENSP00000284311 | 8.19E-06 | -0.2688 | 0 | 0.216 |
| ENSP00000264231 | 5.94E-06 | -0.2688 | 0 | 0.119 |
| ENSP00000453076 | 6.79E-06 | -0.2688 | 159 | 0.432 |
| ENSP00000381086 | 2.15E-06 | -0.2688 | 0 | 0.157 |
| ENSP00000371193 | 3.78E-06 | -0.2689 | 0 | 0.109 |
| ENSP00000308312 | 3.12E-06 | -0.2689 | 0 | 0.044 |
| ENSP00000318716 | 3.21E-06 | -0.2690 | 0 | 0.123 |
| ENSP00000360076 | 3.08E-06 | -0.2691 | 0 | 0.116 |
| ENSP00000343248 | 7.53E-06 | -0.2691 | 0 | 0.141 |
| ENSP00000280236 | 9.88E-06 | -0.2691 | 0 | 0.104 |
| ENSP00000347710 | 7.20E-06 | -0.2691 | 294 | 0.518 |
| ENSP00000373846 | 4.45E-06 | -0.2691 | 0 | 0.423 |
| ENSP00000401338 | 2.24E-06 | -0.2692 | 0 | 0.130 |
| ENSP00000393596 | 4.72E-06 | -0.2693 | 0 | 0.435 |
| ENSP00000310309 | 4.67E-06 | -0.2693 | 0 | 0.162 |
| ENSP00000425375 | 3.90E-06 | -0.2694 | 0 | 0.558 |
| ENSP00000438513 | 1.05E-05 | -0.2694 | 187 | 0.000 |
| ENSP00000302924 | 5.32E-06 | -0.2694 | 0 | 0.243 |
| ENSP00000361112 | 1.20E-05 | -0.2694 | 156 | 0.466 |
| ENSP00000469872 | 2.78E-06 | -0.2694 | 0 | 0.169 |
| ENSP00000394008 | 9.16E-06 | -0.2695 | 185 | 0.000 |
| ENSP00000264824 | 9.57E-06 | -0.2697 | 0 | 0.801 |
| ENSP00000421686 | 9.55E-07 | -0.2697 | 0 | 0.170 |
| ENSP00000314608 | 4.22E-06 | -0.2697 | 0 | 0.109 |
| ENSP00000360761 | 8.74E-06 | -0.2698 | 301 | 0.452 |
| ENSP00000371786 | 2.64E-06 | -0.2698 | 0 | 0.316 |
| ENSP00000371037 | 1.27E-06 | -0.2698 | 0 | 0.069 |
| ENSP00000347345 | 5.47E-06 | -0.2699 | 451 | 0.698 |
| ENSP00000409382 | 4.52E-06 | -0.2699 | 0 | 0.000 |
| ENSP00000447488 | 4.00E-06 | -0.2700 | 0 | 0.315 |
| ENSP00000441858 | 4.83E-06 | -0.2700 | 0 | 0.057 |
| ENSP00000362807 | 3.51E-06 | -0.2701 | 0 | 0.119 |
| ENSP00000346046 | 3.11E-06 | -0.2701 | 0 | 0.000 |
| ENSP00000362111 | 3.63E-06 | -0.2701 | 0 | 0.092 |
| ENSP00000325508 | 3.57E-06 | -0.2701 | 0 | 0.096 |
| ENSP00000357602 | 2.94E-06 | -0.2702 | 0 | 0.132 |
| ENSP00000360107 | 5.22E-06 | -0.2703 | 0 | 0.092 |
| ENSP00000434797 | 4.13E-06 | -0.2703 | 0 | 0.269 |
| ENSP00000263071 | 1.36E-05 | -0.2703 | 342 | 0.357 |
| ENSP00000414066 | 3.24E-06 | -0.2703 | 0 | 0.000 |
| ENSP00000342216 | 4.02E-06 | -0.2704 | 0 | 0.088 |

| | | | | |
|---|---|---|---|---|
| ENSP00000386908 | 3.78E-06 | -0.2704 | 0 | 0.165 |
| ENSP00000243673 | 1.15E-05 | -0.2704 | 539 | 0.361 |
| ENSP00000228820 | 7.21E-06 | -0.2705 | 0 | 0.237 |
| ENSP00000348472 | 7.03E-06 | -0.2705 | 0 | 0.392 |
| ENSP00000259212 | 1.20E-05 | -0.2705 | 0 | 0.206 |
| ENSP00000344996 | 6.54E-06 | -0.2706 | 0 | 0.121 |
| ENSP00000401508 | 2.89E-06 | -0.2707 | 0 | 0.000 |
| ENSP00000376705 | 2.67E-06 | -0.2707 | 0 | 0.086 |
| ENSP00000314042 | 4.78E-06 | -0.2707 | 0 | 0.146 |
| ENSP00000360998 | 9.20E-06 | -0.2708 | 202 | 0.636 |
| ENSP00000306497 | 4.66E-06 | -0.2708 | 0 | 0.247 |
| ENSP00000343665 | 2.57E-06 | -0.2708 | 0 | 0.000 |
| ENSP00000374354 | 3.68E-06 | -0.2708 | 0 | 0.789 |
| ENSP00000333591 | 4.53E-06 | -0.2710 | 0 | 0.049 |
| ENSP00000313243 | 8.87E-06 | -0.2711 | 153 | 0.000 |
| ENSP00000440915 | 1.34E-06 | -0.2711 | 0 | 0.312 |
| ENSP00000419851 | 1.96E-05 | -0.2711 | 628 | 0.788 |
| ENSP00000276185 | 5.47E-06 | -0.2711 | 0 | 0.158 |
| ENSP00000375080 | 2.19E-06 | -0.2712 | 0 | 0.141 |
| ENSP00000352163 | 1.03E-05 | -0.2712 | 0 | 0.094 |
| ENSP00000355261 | 2.72E-06 | -0.2712 | 0 | 0.737 |
| ENSP00000300079 | 1.17E-05 | -0.2713 | 179 | 0.452 |
| ENSP00000338785 | 7.12E-06 | -0.2714 | 0 | 0.184 |
| ENSP00000326340 | 2.32E-06 | -0.2714 | 0 | 0.098 |
| ENSP00000333003 | 7.46E-06 | -0.2715 | 0 | 0.118 |
| ENSP00000359219 | 2.65E-06 | -0.2715 | 0 | 0.109 |
| ENSP00000310431 | 5.34E-06 | -0.2715 | 0 | 0.101 |
| ENSP00000442057 | 2.18E-06 | -0.2716 | 0 | 0.711 |
| ENSP00000376268 | 6.61E-06 | -0.2716 | 0 | 0.448 |
| ENSP00000357067 | 5.65E-06 | -0.2716 | 0 | 0.152 |
| ENSP00000355719 | 8.08E-06 | -0.2716 | 0 | 0.096 |
| ENSP00000379401 | 6.04E-06 | -0.2717 | 0 | 0.241 |
| ENSP00000352995 | 2.92E-06 | -0.2717 | 0 | 0.000 |
| ENSP00000335060 | 4.59E-06 | -0.2717 | 0 | 0.060 |
| ENSP00000340477 | 3.42E-06 | -0.2717 | 0 | 0.113 |
| ENSP00000269209 | 7.63E-06 | -0.2718 | 0 | 0.119 |
| ENSP00000392398 | 1.37E-05 | -0.2718 | 242 | 0.201 |
| ENSP00000359449 | 3.63E-06 | -0.2718 | 0 | 0.000 |
| ENSP00000378578 | 3.88E-06 | -0.2719 | 0 | 0.720 |
| ENSP00000361512 | 5.04E-06 | -0.2719 | 241 | 0.636 |
| ENSP00000355387 | 2.78E-06 | -0.2719 | 0 | 0.165 |
| ENSP00000464036 | 4.45E-06 | -0.2720 | 0 | 0.584 |
| ENSP00000273173 | 5.67E-06 | -0.2720 | 0 | 0.089 |
| ENSP00000345702 | 7.91E-06 | -0.2721 | 925 | 0.000 |
| ENSP00000235532 | 5.95E-06 | -0.2721 | 0 | 0.073 |
| ENSP00000364412 | 5.21E-06 | -0.2722 | 0 | 0.213 |

| | | | | |
|---|---|---|---|---|
| ENSP00000432104 | 1.69E-06 | -0.2724 | 0 | 0.148 |
| ENSP00000458570 | 1.73E-06 | -0.2724 | 0 | 0.105 |
| ENSP00000350651 | 4.35E-06 | -0.2725 | 0 | 0.298 |
| ENSP00000321221 | 7.67E-06 | -0.2725 | 208 | 0.703 |
| ENSP00000371272 | 3.08E-06 | -0.2725 | 0 | 0.348 |
| ENSP00000417748 | 1.41E-05 | -0.2726 | 216 | 0.000 |
| ENSP00000281523 | 4.62E-06 | -0.2726 | 0 | 0.134 |
| ENSP00000334650 | 2.80E-06 | -0.2726 | 0 | 0.134 |
| ENSP00000461460 | 1.47E-05 | -0.2726 | 167 | 0.000 |
| ENSP00000355736 | 8.05E-06 | -0.2726 | 0 | 0.063 |
| ENSP00000380427 | 4.82E-06 | -0.2728 | 0 | 0.239 |
| ENSP00000366477 | 1.52E-06 | -0.2728 | 0 | 0.000 |
| ENSP00000331474 | 4.40E-06 | -0.2728 | 0 | 0.103 |
| ENSP00000361473 | 3.46E-06 | -0.2728 | 0 | 0.877 |
| ENSP00000361337 | 6.26E-06 | -0.2729 | 0 | 0.126 |
| ENSP00000300658 | 3.94E-06 | -0.2729 | 0 | 0.054 |
| ENSP00000321731 | 8.57E-06 | -0.2729 | 201 | 0.564 |
| ENSP00000340467 | 3.17E-06 | -0.2730 | 0 | 0.104 |
| ENSP00000324196 | 2.87E-06 | -0.2731 | 0 | 0.082 |
| ENSP00000385995 | 2.74E-06 | -0.2731 | 0 | 0.372 |
| ENSP00000370129 | 6.39E-06 | -0.2731 | 243 | 0.197 |
| ENSP00000333183 | 5.06E-06 | -0.2731 | 0 | 0.547 |
| ENSP00000382120 | 5.91E-06 | -0.2732 | 315 | 0.458 |
| ENSP00000451304 | 1.04E-05 | -0.2732 | 187 | 0.000 |
| ENSP00000416797 | 1.97E-06 | -0.2733 | 0 | 0.073 |
| ENSP00000338624 | 5.92E-06 | -0.2734 | 0 | 0.104 |
| ENSP00000369374 | 4.68E-06 | -0.2734 | 0 | 0.651 |
| ENSP00000328495 | 5.66E-06 | -0.2734 | 0 | 0.000 |
| ENSP00000355119 | 4.41E-06 | -0.2734 | 0 | 0.676 |
| ENSP00000312457 | 1.01E-05 | -0.2734 | 0 | 0.379 |
| ENSP00000336783 | 3.63E-06 | -0.2734 | 0 | 0.486 |
| ENSP00000387046 | 2.45E-06 | -0.2735 | 0 | 0.103 |
| ENSP00000457682 | 1.73E-06 | -0.2735 | 0 | 0.000 |
| ENSP00000376570 | 5.05E-06 | -0.2736 | 0 | 0.088 |
| ENSP00000325144 | 2.35E-06 | -0.2736 | 0 | 0.134 |
| ENSP00000281806 | 1.42E-05 | -0.2737 | 210 | 0.530 |
| ENSP00000362125 | 3.45E-06 | -0.2737 | 0 | 0.140 |
| ENSP00000371497 | 5.16E-06 | -0.2737 | 0 | 0.082 |
| ENSP00000357474 | 4.40E-06 | -0.2738 | 0 | 0.130 |
| ENSP00000360020 | 7.36E-06 | -0.2739 | 448 | 0.618 |
| ENSP00000366396 | 1.26E-05 | -0.2739 | 204 | 0.000 |
| ENSP00000362975 | 5.64E-06 | -0.2739 | 0 | 0.000 |
| ENSP00000268281 | 7.86E-06 | -0.2740 | 0 | 0.147 |
| ENSP00000357883 | 3.51E-06 | -0.2740 | 0 | 0.431 |
| ENSP00000263671 | 1.33E-05 | -0.2740 | 359 | 0.452 |
| ENSP00000310880 | 4.31E-06 | -0.2740 | 0 | 0.191 |

| | | | | |
|---|---|---|---|---|
| ENSP00000353462 | 6.10E-06 | -0.2741 | 0 | 0.140 |
| ENSP00000358142 | 4.09E-06 | -0.2741 | 0 | 0.140 |
| ENSP00000287152 | 3.54E-06 | -0.2741 | 196 | 0.162 |
| ENSP00000357631 | 2.84E-06 | -0.2741 | 0 | 0.252 |
| ENSP00000252597 | 3.92E-06 | -0.2741 | 0 | 0.125 |
| ENSP00000362469 | 3.70E-06 | -0.2741 | 0 | 0.094 |
| ENSP00000328352 | 6.64E-06 | -0.2742 | 0 | 0.216 |
| ENSP00000367721 | 3.34E-06 | -0.2742 | 0 | 0.527 |
| ENSP00000302811 | 1.35E-05 | -0.2742 | 304 | 0.655 |
| ENSP00000300226 | 7.54E-06 | -0.2742 | 0 | 0.181 |
| ENSP00000260453 | 5.56E-06 | -0.2742 | 0 | 0.139 |
| ENSP00000370508 | 4.80E-06 | -0.2742 | 324 | 0.724 |
| ENSP00000347548 | 3.27E-06 | -0.2743 | 0 | 0.093 |
| ENSP00000276077 | 4.39E-06 | -0.2744 | 0 | 0.134 |
| ENSP00000324944 | 3.77E-06 | -0.2744 | 0 | 0.132 |
| ENSP00000346378 | 8.72E-06 | -0.2744 | 252 | 0.269 |
| ENSP00000341490 | 4.29E-06 | -0.2745 | 0 | 0.103 |
| ENSP00000352284 | 3.16E-06 | -0.2745 | 0 | 0.147 |
| ENSP00000412957 | 2.99E-06 | -0.2745 | 0 | 0.477 |
| ENSP00000361202 | 5.54E-06 | -0.2746 | 0 | 0.672 |
| ENSP00000242819 | 3.82E-06 | -0.2746 | 0 | 0.179 |
| ENSP00000361935 | 4.40E-06 | -0.2746 | 0 | 0.223 |
| ENSP00000249016 | 1.32E-05 | -0.2746 | 173 | 0.596 |
| ENSP00000354916 | 3.90E-06 | -0.2747 | 0 | 0.000 |
| ENSP00000256367 | 4.57E-06 | -0.2747 | 0 | 0.118 |
| ENSP00000266068 | 6.98E-06 | -0.2748 | 0 | 0.223 |
| ENSP00000411827 | 2.58E-06 | -0.2748 | 0 | 0.128 |
| ENSP00000253699 | 3.58E-06 | -0.2749 | 0 | 0.000 |
| ENSP00000309338 | 8.74E-06 | -0.2749 | 0 | 0.324 |
| ENSP00000407761 | 3.68E-06 | -0.2749 | 0 | 0.208 |
| ENSP00000292586 | 4.82E-06 | -0.2750 | 0 | 0.247 |
| ENSP00000350976 | 3.86E-06 | -0.2750 | 0 | 0.118 |
| ENSP00000419974 | 3.53E-06 | -0.2752 | 0 | 0.196 |
| ENSP00000297814 | 3.23E-06 | -0.2753 | 0 | 0.325 |
| ENSP00000365075 | 8.05E-06 | -0.2753 | 0 | 0.161 |
| ENSP00000418661 | 1.88E-06 | -0.2753 | 0 | 0.073 |
| ENSP00000368818 | 3.20E-06 | -0.2753 | 0 | 0.163 |
| ENSP00000296327 | 1.02E-05 | -0.2754 | 0 | 0.161 |
| ENSP00000237275 | 9.23E-06 | -0.2754 | 0 | 0.137 |
| ENSP00000358931 | 7.48E-06 | -0.2754 | 401 | 0.446 |
| ENSP00000339613 | 4.71E-06 | -0.2755 | 0 | 0.118 |
| ENSP00000356278 | 3.54E-06 | -0.2755 | 0 | 0.089 |
| ENSP00000392879 | 1.50E-06 | -0.2755 | 0 | 0.000 |
| ENSP00000390111 | 3.52E-06 | -0.2755 | 0 | 0.080 |
| ENSP00000226578 | 1.12E-05 | -0.2756 | 239 | 0.215 |
| ENSP00000313384 | 2.82E-06 | -0.2756 | 0 | 0.000 |

| | | | | |
|---|---|---|---|---|
| ENSP00000389289 | 6.80E-06 | -0.2756 | 243 | 0.121 |
| ENSP00000355302 | 3.91E-06 | -0.2756 | 0 | 0.120 |
| ENSP00000427900 | 4.14E-06 | -0.2757 | 0 | 0.201 |
| ENSP00000341346 | 5.08E-06 | -0.2758 | 0 | 0.096 |
| ENSP00000333638 | 3.90E-06 | -0.2758 | 0 | 0.128 |
| ENSP00000356366 | 7.12E-06 | -0.2759 | 278 | 0.085 |
| ENSP00000308893 | 1.27E-05 | -0.2761 | 160 | 0.171 |
| ENSP00000373684 | 1.46E-05 | -0.2761 | 490 | 0.662 |
| ENSP00000394794 | 7.40E-06 | -0.2761 | 0 | 0.824 |
| ENSP00000333234 | 3.24E-06 | -0.2761 | 0 | 0.091 |
| ENSP00000356411 | 4.90E-06 | -0.2761 | 0 | 0.247 |
| ENSP00000364580 | 4.50E-06 | -0.2763 | 0 | 0.229 |
| ENSP00000320017 | 3.98E-06 | -0.2764 | 0 | 0.203 |
| ENSP00000310969 | 3.25E-06 | -0.2764 | 0 | 0.000 |
| ENSP00000348915 | 2.56E-06 | -0.2764 | 0 | 0.230 |
| ENSP00000402935 | 4.40E-06 | -0.2764 | 0 | 0.091 |
| ENSP00000357812 | 2.39E-06 | -0.2766 | 0 | 0.241 |
| ENSP00000281471 | 4.42E-06 | -0.2766 | 0 | 0.158 |
| ENSP00000361338 | 1.90E-06 | -0.2766 | 0 | 0.000 |
| ENSP00000264651 | 8.16E-06 | -0.2767 | 0 | 0.124 |
| ENSP00000378050 | 3.28E-06 | -0.2767 | 0 | 0.134 |
| ENSP00000440594 | 3.98E-06 | -0.2767 | 0 | 0.109 |
| ENSP00000303486 | 8.14E-06 | -0.2767 | 0 | 0.150 |
| ENSP00000259873 | 4.33E-06 | -0.2768 | 0 | 0.412 |
| ENSP00000431822 | 2.88E-05 | -0.2768 | 345 | 0.673 |
| ENSP00000375082 | 3.68E-06 | -0.2768 | 0 | 0.177 |
| ENSP00000354543 | 3.65E-06 | -0.2768 | 0 | 0.139 |
| ENSP00000221462 | 1.01E-05 | -0.2769 | 0 | 0.125 |
| ENSP00000348635 | 5.63E-06 | -0.2769 | 0 | 0.098 |
| ENSP00000414378 | 4.70E-06 | -0.2769 | 0 | 0.664 |
| ENSP00000366402 | 2.77E-06 | -0.2769 | 0 | 0.122 |
| ENSP00000335196 | 6.31E-06 | -0.2770 | 0 | 0.183 |
| ENSP00000320401 | 4.25E-06 | -0.2770 | 0 | 0.131 |
| ENSP00000356992 | 2.08E-06 | -0.2771 | 0 | 0.102 |
| ENSP00000326445 | 2.50E-06 | -0.2773 | 0 | 0.135 |
| ENSP00000444777 | 4.20E-06 | -0.2774 | 205 | 0.000 |
| ENSP00000273390 | 8.33E-06 | -0.2775 | 0 | 0.113 |
| ENSP00000273980 | 2.56E-06 | -0.2776 | 0 | 0.125 |
| ENSP00000385739 | 4.26E-06 | -0.2777 | 0 | 0.126 |
| ENSP00000357507 | 2.59E-06 | -0.2777 | 0 | 0.133 |
| ENSP00000355192 | 4.57E-06 | -0.2777 | 0 | 0.424 |
| ENSP00000463483 | 1.50E-06 | -0.2777 | 0 | 0.125 |
| ENSP00000381566 | 4.04E-06 | -0.2778 | 0 | 0.109 |
| ENSP00000357807 | 3.01E-06 | -0.2779 | 0 | 0.093 |
| ENSP00000417229 | 6.39E-06 | -0.2780 | 206 | 0.861 |
| ENSP00000322486 | 6.66E-06 | -0.2780 | 0 | 0.095 |

| | | | | |
|---|---|---|---|---|
| ENSP00000330051 | 4.13E-06 | -0.2780 | 0 | 0.126 |
| ENSP00000344929 | 3.27E-06 | -0.2781 | 0 | 0.139 |
| ENSP00000305725 | 6.01E-06 | -0.2781 | 0 | 0.095 |
| ENSP00000215912 | 4.88E-06 | -0.2781 | 0 | 0.067 |
| ENSP00000343318 | 3.81E-06 | -0.2782 | 0 | 0.074 |
| ENSP00000303928 | 4.54E-06 | -0.2783 | 0 | 0.221 |
| ENSP00000321617 | 3.87E-06 | -0.2784 | 0 | 0.135 |
| ENSP00000364150 | 4.91E-06 | -0.2784 | 0 | 0.159 |
| ENSP00000201647 | 5.15E-06 | -0.2784 | 0 | 0.111 |
| ENSP00000460823 | 4.19E-06 | -0.2786 | 0 | 0.196 |
| ENSP00000286544 | 6.54E-06 | -0.2787 | 0 | 0.151 |
| ENSP00000296350 | 9.88E-06 | -0.2788 | 0 | 0.119 |
| ENSP00000170150 | 1.08E-05 | -0.2788 | 0 | 0.120 |
| ENSP00000376432 | 4.96E-06 | -0.2788 | 0 | 0.406 |
| ENSP00000356529 | 9.77E-06 | -0.2789 | 188 | 0.485 |
| ENSP00000227065 | 9.93E-06 | -0.2789 | 0 | 0.126 |
| ENSP00000355969 | 7.50E-06 | -0.2791 | 0 | 0.289 |
| ENSP00000216068 | 3.66E-06 | -0.2792 | 0 | 0.120 |
| ENSP00000393912 | 6.49E-06 | -0.2792 | 163 | 0.000 |
| ENSP00000369445 | 2.35E-06 | -0.2792 | 0 | 0.000 |
| ENSP00000362629 | 1.82E-06 | -0.2792 | 0 | 0.000 |
| ENSP00000381970 | 1.86E-06 | -0.2793 | 0 | 0.113 |
| ENSP00000365757 | 4.22E-06 | -0.2793 | 0 | 0.435 |
| ENSP00000239614 | 1.14E-05 | -0.2794 | 0 | 0.137 |
| ENSP00000455510 | 3.94E-06 | -0.2794 | 0 | 0.143 |
| ENSP00000462972 | 6.16E-06 | -0.2794 | 488 | 0.413 |
| ENSP00000373277 | 3.85E-06 | -0.2795 | 0 | 0.137 |
| ENSP00000391742 | 5.82E-06 | -0.2795 | 0 | 0.198 |
| ENSP00000357773 | 3.96E-06 | -0.2795 | 0 | 0.125 |
| ENSP00000339340 | 4.64E-06 | -0.2796 | 0 | 0.120 |
| ENSP00000376397 | 3.59E-06 | -0.2796 | 0 | 0.084 |
| ENSP00000270824 | 3.47E-06 | -0.2796 | 0 | 0.103 |
| ENSP00000334219 | 4.95E-06 | -0.2797 | 0 | 0.098 |
| ENSP00000356792 | 1.40E-05 | -0.2797 | 196 | 0.578 |
| ENSP00000167106 | 1.33E-05 | -0.2798 | 0 | 0.168 |
| ENSP00000292894 | 2.42E-06 | -0.2798 | 0 | 0.153 |
| ENSP00000345667 | 6.00E-06 | -0.2798 | 0 | 0.093 |
| ENSP00000331435 | 5.20E-06 | -0.2799 | 0 | 0.547 |
| ENSP00000415026 | 1.82E-06 | -0.2799 | 0 | 0.097 |
| ENSP00000377878 | 5.06E-06 | -0.2799 | 154 | 0.439 |
| ENSP00000358510 | 2.79E-06 | -0.2800 | 0 | 0.122 |
| ENSP00000380646 | 2.48E-06 | -0.2800 | 0 | 0.141 |
| ENSP00000266395 | 1.21E-05 | -0.2801 | 0 | 0.222 |
| ENSP00000346130 | 3.61E-06 | -0.2801 | 0 | 0.093 |
| ENSP00000304676 | 7.05E-06 | -0.2801 | 0 | 0.345 |
| ENSP00000357769 | 3.35E-06 | -0.2802 | 0 | 0.169 |

| | | | | |
|---|---|---|---|---|
| ENSP00000309792 | 3.13E-06 | -0.2802 | 0 | 0.102 |
| ENSP00000379531 | 4.71E-06 | -0.2803 | 0 | 0.097 |
| ENSP00000365351 | 8.16E-06 | -0.2803 | 0 | 0.247 |
| ENSP00000337804 | 5.13E-06 | -0.2804 | 0 | 0.065 |
| ENSP00000331062 | 8.18E-06 | -0.2804 | 0 | 0.184 |
| ENSP00000328397 | 8.08E-06 | -0.2804 | 0 | 0.109 |
| ENSP00000381611 | 1.83E-06 | -0.2804 | 0 | 0.142 |
| ENSP00000424126 | 7.15E-06 | -0.2804 | 0 | 0.116 |
| ENSP00000359942 | 3.95E-06 | -0.2805 | 0 | 0.107 |
| ENSP00000294664 | 5.17E-06 | -0.2805 | 0 | 0.091 |
| ENSP00000371577 | 2.57E-06 | -0.2806 | 0 | 0.130 |
| ENSP00000309595 | 4.85E-06 | -0.2806 | 163 | 0.000 |
| ENSP00000379908 | 3.87E-06 | -0.2808 | 0 | 0.126 |
| ENSP00000370964 | 4.91E-06 | -0.2809 | 0 | 0.000 |
| ENSP00000429273 | 1.93E-06 | -0.2810 | 0 | 0.149 |
| ENSP00000317439 | 3.93E-06 | -0.2811 | 0 | 0.110 |
| ENSP00000393161 | 3.55E-06 | -0.2814 | 0 | 0.120 |
| ENSP00000267853 | 6.46E-06 | -0.2815 | 0 | 0.264 |
| ENSP00000311122 | 3.76E-06 | -0.2815 | 0 | 0.156 |
| ENSP00000349781 | 2.87E-06 | -0.2816 | 0 | 0.142 |
| ENSP00000349336 | 2.73E-06 | -0.2817 | 0 | 0.149 |
| ENSP00000330148 | 4.21E-06 | -0.2817 | 0 | 0.112 |
| ENSP00000382804 | 1.24E-06 | -0.2817 | 0 | 0.169 |
| ENSP00000280358 | 4.94E-06 | -0.2818 | 0 | 0.238 |
| ENSP00000427401 | 9.41E-07 | -0.2818 | 0 | 0.118 |
| ENSP00000355607 | 3.77E-06 | -0.2818 | 0 | 0.251 |
| ENSP00000407818 | 2.34E-06 | -0.2819 | 0 | 0.181 |
| ENSP00000350336 | 1.40E-05 | -0.2820 | 0 | 0.112 |
| ENSP00000366827 | 4.26E-06 | -0.2820 | 0 | 0.070 |
| ENSP00000402152 | 1.08E-05 | -0.2820 | 159 | 0.179 |
| ENSP00000301419 | 5.34E-06 | -0.2821 | 0 | 0.072 |
| ENSP00000359928 | 7.35E-06 | -0.2821 | 201 | 0.000 |
| ENSP00000388825 | 9.40E-06 | -0.2821 | 177 | 0.570 |
| ENSP00000298050 | 3.62E-06 | -0.2821 | 0 | 0.125 |
| ENSP00000417583 | 8.57E-06 | -0.2822 | 224 | 0.182 |
| ENSP00000299481 | 9.54E-06 | -0.2822 | 0 | 0.115 |
| ENSP00000218056 | 8.13E-06 | -0.2826 | 0 | 0.106 |
| ENSP00000464265 | 2.58E-05 | -0.2826 | 295 | 0.000 |
| ENSP00000358617 | 2.46E-06 | -0.2826 | 0 | 0.460 |
| ENSP00000338164 | 3.10E-06 | -0.2828 | 0 | 0.139 |
| ENSP00000371734 | 8.10E-06 | -0.2828 | 201 | 0.708 |
| ENSP00000368808 | 5.37E-06 | -0.2828 | 0 | 0.222 |
| ENSP00000259213 | 1.05E-05 | -0.2828 | 0 | 0.100 |
| ENSP00000323302 | 1.02E-05 | -0.2828 | 178 | 0.154 |
| ENSP00000288976 | 5.35E-06 | -0.2829 | 165 | 0.000 |
| ENSP00000419503 | 2.92E-06 | -0.2829 | 0 | 0.000 |

| | | | | |
|---|---|---|---|---|
| ENSP00000387185 | 2.64E-06 | -0.2829 | 0 | 0.077 |
| ENSP00000306324 | 6.29E-06 | -0.2829 | 0 | 0.165 |
| ENSP00000253193 | 9.81E-06 | -0.2831 | 0 | 0.154 |
| ENSP00000315626 | 6.59E-06 | -0.2832 | 0 | 0.152 |
| ENSP00000260008 | 3.82E-06 | -0.2832 | 0 | 0.237 |
| ENSP00000353695 | 3.35E-06 | -0.2832 | 0 | 0.075 |
| ENSP00000182096 | 8.31E-06 | -0.2832 | 0 | 0.000 |
| ENSP00000355968 | 4.52E-06 | -0.2832 | 0 | 0.659 |
| ENSP00000272438 | 4.08E-06 | -0.2832 | 0 | 0.114 |
| ENSP00000383234 | 9.50E-06 | -0.2833 | 344 | 0.667 |
| ENSP00000378191 | 1.05E-05 | -0.2834 | 353 | 0.706 |
| ENSP00000380795 | 4.22E-06 | -0.2834 | 0 | 0.000 |
| ENSP00000290722 | 9.49E-06 | -0.2834 | 0 | 0.274 |
| ENSP00000352282 | 2.55E-06 | -0.2835 | 0 | 0.218 |
| ENSP00000396704 | 2.04E-05 | -0.2835 | 697 | 0.740 |
| ENSP00000318641 | 3.54E-06 | -0.2835 | 0 | 0.332 |
| ENSP00000356990 | 3.36E-06 | -0.2836 | 0 | 0.273 |
| ENSP00000442793 | 3.21E-06 | -0.2837 | 0 | 0.118 |
| ENSP00000363999 | 1.61E-06 | -0.2837 | 0 | 0.269 |
| ENSP00000348982 | 5.52E-06 | -0.2837 | 0 | 0.144 |
| ENSP00000356905 | 4.38E-06 | -0.2838 | 0 | 0.145 |
| ENSP00000417919 | 1.68E-05 | -0.2839 | 153 | 0.000 |
| ENSP00000399356 | 1.63E-06 | -0.2839 | 0 | 0.127 |
| ENSP00000323635 | 5.50E-06 | -0.2839 | 0 | 0.095 |
| ENSP00000348831 | 6.49E-06 | -0.2840 | 195 | 0.501 |
| ENSP00000381782 | 2.00E-06 | -0.2840 | 0 | 0.083 |
| ENSP00000273857 | 1.05E-05 | -0.2840 | 0 | 0.680 |
| ENSP00000368173 | 2.49E-06 | -0.2841 | 0 | 0.155 |
| ENSP00000314520 | 1.18E-05 | -0.2841 | 282 | 0.346 |
| ENSP00000430344 | 5.48E-06 | -0.2842 | 0 | 0.516 |
| ENSP00000417901 | 6.51E-06 | -0.2843 | 392 | 0.271 |
| ENSP00000333064 | 2.90E-06 | -0.2844 | 0 | 0.177 |
| ENSP00000307004 | 6.54E-06 | -0.2844 | 0 | 0.486 |
| ENSP00000393963 | 3.76E-06 | -0.2844 | 0 | 0.261 |
| ENSP00000419195 | 3.69E-06 | -0.2845 | 0 | 0.000 |
| ENSP00000357909 | 3.97E-06 | -0.2846 | 0 | 0.441 |
| ENSP00000364092 | 4.78E-06 | -0.2846 | 0 | 0.127 |
| ENSP00000263196 | 5.57E-06 | -0.2846 | 0 | 0.202 |
| ENSP00000391137 | 9.06E-06 | -0.2846 | 202 | 0.671 |
| ENSP00000376349 | 4.82E-06 | -0.2847 | 201 | 0.269 |
| ENSP00000340982 | 3.92E-06 | -0.2847 | 0 | 0.159 |
| ENSP00000429216 | 4.16E-06 | -0.2847 | 0 | 0.090 |
| ENSP00000283916 | 9.95E-06 | -0.2847 | 212 | 0.333 |
| ENSP00000369344 | 2.87E-06 | -0.2848 | 0 | 0.148 |
| ENSP00000450895 | 7.46E-06 | -0.2848 | 201 | 0.598 |
| ENSP00000342197 | 3.93E-06 | -0.2848 | 0 | 0.164 |

| | | | | |
|---|---|---|---|---|
| ENSP00000379090 | 2.83E-06 | -0.2849 | 0 | 0.234 |
| ENSP00000334714 | 6.16E-06 | -0.2849 | 0 | 0.121 |
| ENSP00000434614 | 1.13E-05 | -0.2849 | 320 | 0.661 |
| ENSP00000386181 | 3.23E-06 | -0.2850 | 0 | 0.064 |
| ENSP00000363391 | 9.99E-06 | -0.2850 | 524 | 0.051 |
| ENSP00000274473 | 3.67E-06 | -0.2850 | 0 | 0.182 |
| ENSP00000371397 | 2.17E-06 | -0.2851 | 0 | 0.123 |
| ENSP00000319476 | 9.07E-06 | -0.2852 | 0 | 0.410 |
| ENSP00000350032 | 5.88E-06 | -0.2853 | 0 | 0.116 |
| ENSP00000315152 | 1.02E-05 | -0.2853 | 244 | 0.801 |
| ENSP00000301599 | 1.12E-05 | -0.2853 | 0 | 0.157 |
| ENSP00000300591 | 6.80E-06 | -0.2854 | 0 | 0.111 |
| ENSP00000261007 | 1.25E-05 | -0.2854 | 150 | 0.574 |
| ENSP00000368814 | 4.78E-06 | -0.2856 | 0 | 0.107 |
| ENSP00000338627 | 5.14E-06 | -0.2856 | 0 | 0.072 |
| ENSP00000280606 | 8.05E-06 | -0.2856 | 0 | 0.184 |
| ENSP00000338034 | 7.63E-06 | -0.2857 | 0 | 0.095 |
| ENSP00000468969 | 2.93E-06 | -0.2857 | 0 | 0.163 |
| ENSP00000312753 | 2.57E-06 | -0.2858 | 0 | 0.114 |
| ENSP00000368295 | 7.70E-06 | -0.2858 | 205 | 0.000 |
| ENSP00000364257 | 7.02E-06 | -0.2859 | 0 | 0.129 |
| ENSP00000299340 | 3.68E-06 | -0.2860 | 0 | 0.138 |
| ENSP00000370555 | 3.36E-06 | -0.2860 | 0 | 0.056 |
| ENSP00000378077 | 1.99E-06 | -0.2860 | 0 | 0.156 |
| ENSP00000389649 | 3.74E-06 | -0.2860 | 0 | 0.294 |
| ENSP00000378132 | 8.91E-06 | -0.2860 | 150 | 0.784 |
| ENSP00000313890 | 5.14E-06 | -0.2862 | 0 | 0.000 |
| ENSP00000361965 | 3.79E-06 | -0.2862 | 0 | 0.684 |
| ENSP00000379339 | 5.27E-06 | -0.2862 | 195 | 0.721 |
| ENSP00000386807 | 1.14E-06 | -0.2862 | 0 | 0.186 |
| ENSP00000252939 | 8.81E-06 | -0.2862 | 0 | 0.094 |
| ENSP00000259963 | 1.25E-05 | -0.2863 | 0 | 0.116 |
| ENSP00000435352 | 1.44E-06 | -0.2863 | 0 | 0.000 |
| ENSP00000344041 | 3.00E-06 | -0.2864 | 0 | 0.123 |
| ENSP00000307275 | 6.19E-06 | -0.2864 | 0 | 0.111 |
| ENSP00000267406 | 4.83E-06 | -0.2865 | 0 | 0.122 |
| ENSP00000344551 | 6.93E-06 | -0.2866 | 0 | 0.140 |
| ENSP00000423660 | 3.30E-06 | -0.2866 | 0 | 0.159 |
| ENSP00000426691 | 1.69E-06 | -0.2867 | 0 | 0.115 |
| ENSP00000345767 | 7.44E-06 | -0.2867 | 202 | 0.743 |
| ENSP00000275560 | 7.87E-06 | -0.2867 | 0 | 0.000 |
| ENSP00000281703 | 7.66E-06 | -0.2869 | 0 | 0.142 |
| ENSP00000379364 | 6.29E-06 | -0.2869 | 171 | 0.658 |
| ENSP00000352272 | 5.10E-06 | -0.2870 | 0 | 0.228 |
| ENSP00000319591 | 1.30E-05 | -0.2871 | 514 | 0.322 |
| ENSP00000443824 | 3.56E-06 | -0.2871 | 0 | 0.415 |

| | | | | |
|---|---|---|---|---|
| ENSP00000339867 | 1.12E-05 | -0.2871 | 225 | 0.275 |
| ENSP00000339292 | 5.56E-06 | -0.2872 | 0 | 0.105 |
| ENSP00000280020 | 6.02E-06 | -0.2872 | 0 | 0.189 |
| ENSP00000303584 | 1.08E-05 | -0.2873 | 313 | 0.578 |
| ENSP00000358999 | 2.30E-06 | -0.2874 | 0 | 0.104 |
| ENSP00000206513 | 9.67E-06 | -0.2875 | 0 | 0.734 |
| ENSP00000393539 | 4.09E-06 | -0.2875 | 0 | 0.000 |
| ENSP00000325589 | 8.04E-06 | -0.2875 | 0 | 0.175 |
| ENSP00000318846 | 5.03E-06 | -0.2875 | 0 | 0.000 |
| ENSP00000259407 | 5.11E-06 | -0.2875 | 0 | 0.177 |
| ENSP00000393241 | 4.91E-06 | -0.2876 | 388 | 0.756 |
| ENSP00000437409 | 5.13E-06 | -0.2878 | 0 | 0.792 |
| ENSP00000339621 | 8.12E-06 | -0.2879 | 0 | 0.000 |
| ENSP00000405600 | 7.00E-06 | -0.2880 | 0 | 0.075 |
| ENSP00000264233 | 1.06E-05 | -0.2880 | 274 | 0.000 |
| ENSP00000340088 | 8.36E-06 | -0.2880 | 0 | 0.096 |
| ENSP00000301204 | 3.39E-06 | -0.2880 | 0 | 0.118 |
| ENSP00000429969 | 4.70E-06 | -0.2880 | 0 | 0.176 |
| ENSP00000265294 | 4.98E-06 | -0.2881 | 0 | 0.226 |
| ENSP00000365025 | 4.84E-06 | -0.2881 | 0 | 0.731 |
| ENSP00000373566 | 2.63E-06 | -0.2881 | 0 | 0.121 |
| ENSP00000331691 | 2.64E-06 | -0.2881 | 0 | 0.151 |
| ENSP00000350592 | 2.70E-06 | -0.2883 | 0 | 0.239 |
| ENSP00000257663 | 5.63E-06 | -0.2883 | 0 | 0.112 |
| ENSP00000358316 | 5.41E-06 | -0.2883 | 0 | 0.094 |
| ENSP00000366271 | 3.24E-06 | -0.2884 | 0 | 0.000 |
| ENSP00000160373 | 1.37E-05 | -0.2884 | 201 | 0.603 |
| ENSP00000295645 | 5.15E-06 | -0.2884 | 0 | 0.083 |
| ENSP00000350961 | 1.40E-06 | -0.2884 | 0 | 0.000 |
| ENSP00000409637 | 4.51E-06 | -0.2885 | 0 | 0.000 |
| ENSP00000328677 | 4.80E-06 | -0.2886 | 0 | 0.123 |
| ENSP00000309087 | 4.77E-08 | -0.2886 | 0 | 0.215 |
| ENSP00000358956 | 4.01E-06 | -0.2887 | 0 | 0.094 |
| ENSP00000300022 | 4.41E-06 | -0.2888 | 0 | 0.146 |
| ENSP00000338927 | 3.41E-06 | -0.2889 | 0 | 0.066 |
| ENSP00000223026 | 7.97E-06 | -0.2890 | 0 | 0.061 |
| ENSP00000375066 | 1.25E-05 | -0.2890 | 223 | 0.537 |
| ENSP00000341584 | 7.80E-06 | -0.2890 | 0 | 0.358 |
| ENSP00000359848 | 3.04E-06 | -0.2891 | 0 | 0.070 |
| ENSP00000273588 | 1.18E-05 | -0.2891 | 263 | 0.408 |
| ENSP00000427562 | 6.90E-06 | -0.2891 | 221 | 0.672 |
| ENSP00000257836 | 9.55E-06 | -0.2892 | 0 | 0.166 |
| ENSP00000442770 | 3.87E-06 | -0.2892 | 0 | 0.625 |
| ENSP00000302289 | 6.83E-06 | -0.2893 | 0 | 0.064 |
| ENSP00000387941 | 8.03E-06 | -0.2894 | 216 | 0.241 |
| ENSP00000357804 | 2.75E-06 | -0.2894 | 0 | 0.310 |

| | | | | |
|---|---|---|---|---|
| ENSP00000295966 | 3.05E-06 | -0.2895 | 0 | 0.108 |
| ENSP00000357943 | 4.35E-06 | -0.2898 | 0 | 0.056 |
| ENSP00000410007 | 1.20E-06 | -0.2901 | 0 | 0.085 |
| ENSP00000259989 | 5.40E-06 | -0.2901 | 0 | 0.126 |
| ENSP00000310003 | 3.96E-06 | -0.2903 | 0 | 0.113 |
| ENSP00000263370 | 4.85E-06 | -0.2905 | 0 | 0.107 |
| ENSP00000376227 | 2.80E-06 | -0.2905 | 0 | 0.128 |
| ENSP00000385545 | 2.65E-06 | -0.2906 | 0 | 0.178 |
| ENSP00000377086 | 8.39E-06 | -0.2906 | 247 | 0.000 |
| ENSP00000360492 | 2.71E-06 | -0.2906 | 0 | 0.326 |
| ENSP00000328327 | 1.19E-05 | -0.2907 | 307 | 0.000 |
| ENSP00000386069 | 8.94E-06 | -0.2907 | 173 | 0.540 |
| ENSP00000349989 | 2.46E-06 | -0.2908 | 0 | 0.000 |
| ENSP00000359719 | 1.06E-05 | -0.2908 | 902 | 0.642 |
| ENSP00000348813 | 1.79E-06 | -0.2908 | 0 | 0.109 |
| ENSP00000307860 | 7.33E-06 | -0.2909 | 0 | 0.120 |
| ENSP00000360398 | 3.57E-06 | -0.2910 | 0 | 0.135 |
| ENSP00000314560 | 4.82E-06 | -0.2910 | 0 | 0.100 |
| ENSP00000339182 | 4.36E-06 | -0.2910 | 0 | 0.142 |
| ENSP00000374036 | 2.90E-06 | -0.2910 | 0 | 0.229 |
| ENSP00000472698 | 1.25E-06 | -0.2911 | 0 | 0.000 |
| ENSP00000223864 | 1.22E-05 | -0.2911 | 0 | 0.112 |
| ENSP00000222032 | 7.80E-06 | -0.2911 | 0 | 0.124 |
| ENSP00000283713 | 1.27E-05 | -0.2912 | 583 | 0.254 |
| ENSP00000357022 | 3.70E-06 | -0.2912 | 0 | 0.144 |
| ENSP00000333744 | 2.05E-06 | -0.2913 | 0 | 0.188 |
| ENSP00000397644 | 1.88E-06 | -0.2914 | 0 | 0.000 |
| ENSP00000431184 | 6.20E-06 | -0.2914 | 0 | 0.177 |
| ENSP00000362639 | 2.28E-06 | -0.2914 | 0 | 0.261 |
| ENSP00000332504 | 1.15E-05 | -0.2914 | 0 | 0.538 |
| ENSP00000418532 | 3.34E-06 | -0.2914 | 0 | 0.135 |
| ENSP00000352992 | 3.79E-06 | -0.2915 | 0 | 0.131 |
| ENSP00000410186 | 2.98E-06 | -0.2915 | 0 | 0.371 |
| ENSP00000232508 | 4.61E-06 | -0.2915 | 0 | 0.118 |
| ENSP00000264784 | 3.70E-06 | -0.2915 | 0 | 0.119 |
| ENSP00000344140 | 2.51E-06 | -0.2916 | 0 | 0.106 |
| ENSP00000343636 | 5.36E-06 | -0.2917 | 0 | 0.090 |
| ENSP00000342518 | 3.45E-06 | -0.2917 | 0 | 0.117 |
| ENSP00000370126 | 3.12E-06 | -0.2917 | 0 | 0.153 |
| ENSP00000347915 | 2.34E-06 | -0.2918 | 0 | 0.085 |
| ENSP00000219207 | 5.59E-06 | -0.2918 | 0 | 0.108 |
| ENSP00000229583 | 5.54E-06 | -0.2918 | 0 | 0.139 |
| ENSP00000362841 | 5.08E-06 | -0.2919 | 0 | 0.094 |
| ENSP00000415635 | 4.88E-06 | -0.2919 | 165 | 0.000 |
| ENSP00000355109 | 2.94E-06 | -0.2920 | 0 | 0.118 |
| ENSP00000261369 | 4.39E-06 | -0.2920 | 0 | 0.135 |

| | | | | |
|---|---|---|---|---|
| ENSP00000340914 | 4.85E-06 | -0.2920 | 0 | 0.169 |
| ENSP00000385571 | 8.44E-06 | -0.2921 | 189 | 0.000 |
| ENSP00000378033 | 3.13E-06 | -0.2921 | 0 | 0.149 |
| ENSP00000296051 | 9.42E-06 | -0.2921 | 370 | 0.450 |
| ENSP00000470389 | 1.33E-06 | -0.2922 | 0 | 0.000 |
| ENSP00000472409 | 1.33E-06 | -0.2922 | 0 | 0.302 |
| ENSP00000370801 | 2.77E-06 | -0.2923 | 0 | 0.547 |
| ENSP00000360214 | 2.73E-06 | -0.2923 | 0 | 0.124 |
| ENSP00000381253 | 3.80E-06 | -0.2924 | 0 | 0.712 |
| ENSP00000329507 | 5.08E-06 | -0.2924 | 0 | 0.348 |
| ENSP00000354335 | 8.36E-06 | -0.2925 | 0 | 0.135 |
| ENSP00000371929 | 4.31E-06 | -0.2926 | 0 | 0.273 |
| ENSP00000331418 | 9.98E-06 | -0.2926 | 157 | 0.530 |
| ENSP00000354722 | 3.26E-06 | -0.2927 | 0 | 0.824 |
| ENSP00000329942 | 4.47E-06 | -0.2929 | 0 | 0.000 |
| ENSP00000283025 | 4.07E-06 | -0.2930 | 0 | 0.101 |
| ENSP00000433931 | 1.23E-05 | -0.2930 | 0 | 0.172 |
| ENSP00000360058 | 2.98E-06 | -0.2931 | 0 | 0.503 |
| ENSP00000226951 | 7.57E-06 | -0.2931 | 0 | 0.171 |
| ENSP00000353868 | 1.74E-06 | -0.2931 | 0 | 0.138 |
| ENSP00000324775 | 6.35E-06 | -0.2932 | 0 | 0.106 |
| ENSP00000376504 | 4.46E-06 | -0.2932 | 0 | 0.384 |
| ENSP00000384376 | 2.37E-06 | -0.2932 | 0 | 0.534 |
| ENSP00000335511 | 5.17E-06 | -0.2933 | 0 | 0.335 |
| ENSP00000261058 | 8.19E-06 | -0.2934 | 0 | 0.087 |
| ENSP00000304108 | 3.47E-06 | -0.2936 | 0 | 0.101 |
| ENSP00000355388 | 6.91E-06 | -0.2936 | 202 | 0.000 |
| ENSP00000374205 | 6.13E-06 | -0.2937 | 0 | 0.869 |
| ENSP00000354728 | 4.33E-06 | -0.2937 | 0 | 0.148 |
| ENSP00000368318 | 4.48E-06 | -0.2938 | 317 | 0.636 |
| ENSP00000310801 | 1.10E-05 | -0.2938 | 0 | 0.000 |
| ENSP00000384363 | 5.10E-06 | -0.2938 | 215 | 0.112 |
| ENSP00000318650 | 1.13E-05 | -0.2938 | 287 | 0.639 |
| ENSP00000413445 | 1.54E-06 | -0.2940 | 0 | 0.149 |
| ENSP00000472450 | 1.29E-06 | -0.2940 | 0 | 0.000 |
| ENSP00000362181 | 5.68E-06 | -0.2940 | 0 | 0.526 |
| ENSP00000444350 | 4.01E-06 | -0.2940 | 0 | 0.000 |
| ENSP00000329287 | 1.26E-05 | -0.2942 | 254 | 0.291 |
| ENSP00000317385 | 6.51E-06 | -0.2942 | 0 | 0.099 |
| ENSP00000281087 | 3.86E-06 | -0.2944 | 0 | 0.052 |
| ENSP00000225805 | 8.41E-06 | -0.2945 | 0 | 0.144 |
| ENSP00000454836 | 3.18E-06 | -0.2946 | 0 | 0.879 |
| ENSP00000300618 | 4.89E-06 | -0.2946 | 0 | 0.000 |
| ENSP00000359376 | 4.06E-06 | -0.2947 | 0 | 0.083 |
| ENSP00000391167 | 4.45E-06 | -0.2947 | 0 | 0.073 |
| ENSP00000455547 | 3.20E-06 | -0.2948 | 0 | 0.000 |

| | | | | |
|---|---|---|---|---|
| ENSP00000354813 | 8.79E-06 | -0.2948 | 185 | 0.452 |
| ENSP00000320180 | 1.15E-05 | -0.2949 | 262 | 0.659 |
| ENSP00000332788 | 4.70E-06 | -0.2949 | 0 | 0.087 |
| ENSP00000423602 | 2.78E-06 | -0.2950 | 0 | 0.249 |
| ENSP00000307080 | 8.67E-06 | -0.2951 | 181 | 0.705 |
| ENSP00000249499 | 5.17E-06 | -0.2951 | 0 | 0.818 |
| ENSP00000326846 | 3.10E-06 | -0.2952 | 0 | 0.132 |
| ENSP00000264659 | 6.39E-06 | -0.2953 | 0 | 0.000 |
| ENSP00000335522 | 2.99E-06 | -0.2953 | 0 | 0.239 |
| ENSP00000369849 | 2.76E-06 | -0.2953 | 0 | 0.128 |
| ENSP00000324882 | 3.81E-06 | -0.2953 | 0 | 0.111 |
| ENSP00000350348 | 8.74E-06 | -0.2954 | 251 | 0.568 |
| ENSP00000337475 | 5.01E-06 | -0.2955 | 0 | 0.088 |
| ENSP00000262659 | 6.82E-06 | -0.2956 | 0 | 0.130 |
| ENSP00000321005 | 5.99E-06 | -0.2956 | 0 | 0.084 |
| ENSP00000340526 | 5.14E-06 | -0.2956 | 0 | 0.304 |
| ENSP00000370254 | 6.20E-06 | -0.2957 | 243 | 0.223 |
| ENSP00000295150 | 6.79E-06 | -0.2957 | 0 | 0.137 |
| ENSP00000322617 | 7.04E-06 | -0.2957 | 0 | 0.313 |
| ENSP00000261340 | 4.04E-06 | -0.2957 | 0 | 0.120 |
| ENSP00000372649 | 1.46E-06 | -0.2957 | 0 | 0.142 |
| ENSP00000366233 | 6.30E-06 | -0.2957 | 0 | 0.151 |
| ENSP00000312700 | 5.82E-06 | -0.2957 | 0 | 0.154 |
| ENSP00000365080 | 2.08E-06 | -0.2957 | 0 | 0.108 |
| ENSP00000381298 | 3.37E-06 | -0.2957 | 0 | 0.117 |
| ENSP00000321810 | 8.84E-06 | -0.2958 | 343 | 0.604 |
| ENSP00000347689 | 6.34E-06 | -0.2958 | 511 | 0.659 |
| ENSP00000243314 | 7.31E-06 | -0.2959 | 0 | 0.000 |
| ENSP00000264644 | 4.40E-06 | -0.2960 | 0 | 0.094 |
| ENSP00000392252 | 2.80E-06 | -0.2960 | 0 | 0.081 |
| ENSP00000389465 | 4.85E-06 | -0.2960 | 311 | 0.000 |
| ENSP00000328800 | 4.19E-06 | -0.2960 | 0 | 0.102 |
| ENSP00000375844 | 6.19E-06 | -0.2961 | 284 | 0.000 |
| ENSP00000260276 | 7.08E-06 | -0.2962 | 0 | 0.122 |
| ENSP00000356908 | 1.00E-05 | -0.2962 | 439 | 0.277 |
| ENSP00000230301 | 6.15E-06 | -0.2962 | 0 | 0.118 |
| ENSP00000359100 | 4.47E-06 | -0.2963 | 0 | 0.402 |
| ENSP00000272972 | 6.24E-06 | -0.2966 | 0 | 0.136 |
| ENSP00000307911 | 1.26E-05 | -0.2966 | 941 | 0.051 |
| ENSP00000361311 | 5.10E-06 | -0.2967 | 0 | 0.107 |
| ENSP00000326570 | 7.10E-06 | -0.2967 | 0 | 0.089 |
| ENSP00000461785 | 3.02E-06 | -0.2968 | 0 | 0.250 |
| ENSP00000320828 | 9.62E-06 | -0.2968 | 0 | 0.145 |
| ENSP00000218230 | 7.28E-06 | -0.2968 | 0 | 0.146 |
| ENSP00000250693 | 1.08E-05 | -0.2969 | 0 | 0.208 |
| ENSP00000356708 | 3.77E-06 | -0.2969 | 0 | 0.770 |

| | | | | |
|---|---|---|---|---|
| ENSP00000312054 | 4.80E-06 | -0.2969 | 0 | 0.000 |
| ENSP00000387278 | 9.12E-06 | -0.2970 | 398 | 0.502 |
| ENSP00000330247 | 4.28E-06 | -0.2970 | 0 | 0.100 |
| ENSP00000365938 | 5.35E-06 | -0.2971 | 274 | 0.120 |
| ENSP00000234798 | 1.15E-05 | -0.2971 | 0 | 0.107 |
| ENSP00000473200 | 2.19E-06 | -0.2971 | 0 | 0.000 |
| ENSP00000403946 | 2.32E-06 | -0.2972 | 0 | 0.000 |
| ENSP00000358715 | 7.15E-06 | -0.2972 | 191 | 0.128 |
| ENSP00000420168 | 6.45E-06 | -0.2973 | 0 | 0.322 |
| ENSP00000403636 | 3.40E-06 | -0.2973 | 0 | 0.422 |
| ENSP00000472696 | 2.18E-06 | -0.2975 | 0 | 0.000 |
| ENSP00000332325 | 3.25E-06 | -0.2975 | 0 | 0.272 |
| ENSP00000272425 | 4.05E-06 | -0.2976 | 0 | 0.145 |
| ENSP00000270620 | 4.73E-06 | -0.2976 | 0 | 0.000 |
| ENSP00000357575 | 4.00E-06 | -0.2977 | 0 | 0.000 |
| ENSP00000333711 | 3.99E-06 | -0.2977 | 0 | 0.000 |
| ENSP00000375909 | 4.10E-06 | -0.2977 | 0 | 0.087 |
| ENSP00000392760 | 3.57E-06 | -0.2978 | 0 | 0.337 |
| ENSP00000362932 | 2.77E-06 | -0.2978 | 0 | 0.111 |
| ENSP00000267594 | 6.06E-06 | -0.2981 | 0 | 0.129 |
| ENSP00000348812 | 9.84E-06 | -0.2981 | 169 | 0.757 |
| ENSP00000362442 | 3.94E-06 | -0.2981 | 0 | 0.244 |
| ENSP00000237696 | 1.25E-05 | -0.2982 | 0 | 0.142 |
| ENSP00000263265 | 3.59E-06 | -0.2983 | 0 | 0.198 |
| ENSP00000402297 | 1.63E-06 | -0.2983 | 0 | 0.137 |
| ENSP00000223862 | 6.97E-06 | -0.2984 | 0 | 0.316 |
| ENSP00000354665 | 2.07E-06 | -0.2984 | 0 | 0.130 |
| ENSP00000330264 | 7.65E-06 | -0.2984 | 0 | 0.181 |
| ENSP00000357152 | 9.97E-06 | -0.2985 | 208 | 0.671 |
| ENSP00000260743 | 4.81E-06 | -0.2985 | 0 | 0.103 |
| ENSP00000406157 | 7.50E-06 | -0.2985 | 0 | 0.665 |
| ENSP00000416341 | 7.16E-06 | -0.2985 | 0 | 0.065 |
| ENSP00000329943 | 1.84E-06 | -0.2985 | 0 | 0.131 |
| ENSP00000365176 | 5.12E-06 | -0.2986 | 0 | 0.100 |
| ENSP00000399013 | 4.03E-06 | -0.2987 | 0 | 0.068 |
| ENSP00000273962 | 5.89E-06 | -0.2988 | 0 | 0.740 |
| ENSP00000335447 | 2.23E-06 | -0.2989 | 0 | 0.130 |
| ENSP00000267426 | 9.71E-06 | -0.2989 | 0 | 0.246 |
| ENSP00000306523 | 4.73E-06 | -0.2990 | 0 | 0.319 |
| ENSP00000359665 | 4.30E-06 | -0.2990 | 0 | 0.661 |
| ENSP00000310303 | 5.12E-06 | -0.2991 | 0 | 0.285 |
| ENSP00000229281 | 8.99E-06 | -0.2992 | 0 | 0.117 |
| ENSP00000419704 | 2.27E-06 | -0.2993 | 0 | 0.000 |
| ENSP00000411197 | 2.41E-06 | -0.2994 | 0 | 0.126 |
| ENSP00000299140 | 3.90E-06 | -0.2994 | 0 | 0.110 |
| ENSP00000295226 | 5.18E-06 | -0.2995 | 0 | 0.138 |

| | | | | |
|---|---|---|---|---|
| ENSP00000264072 | 6.26E-06 | -0.2995 | 0 | 0.436 |
| ENSP00000409195 | 3.27E-06 | -0.2997 | 0 | 0.000 |
| ENSP00000243578 | 4.43E-06 | -0.2997 | 0 | 0.159 |
| ENSP00000334153 | 2.92E-06 | -0.2997 | 0 | 0.135 |
| ENSP00000412394 | 2.74E-06 | -0.2998 | 0 | 0.000 |
| ENSP00000341782 | 4.57E-06 | -0.2998 | 0 | 0.099 |
| ENSP00000248879 | 3.94E-06 | -0.2998 | 0 | 0.118 |
| ENSP00000369921 | 8.75E-06 | -0.2999 | 0 | 0.158 |
| ENSP00000364995 | 7.68E-06 | -0.2999 | 0 | 0.785 |
| ENSP00000322181 | 4.68E-06 | -0.2999 | 0 | 0.077 |
| ENSP00000374409 | 5.35E-06 | -0.2999 | 0 | 0.590 |
| ENSP00000417116 | 3.25E-06 | -0.2999 | 0 | 0.491 |
| ENSP00000420954 | 1.02E-06 | -0.3000 | 0 | 0.000 |
| ENSP00000354080 | 5.48E-06 | -0.3000 | 0 | 0.067 |
| ENSP00000382492 | 1.51E-06 | -0.3000 | 0 | 0.190 |
| ENSP00000393066 | 3.23E-06 | -0.3000 | 0 | 0.090 |
| ENSP00000418428 | 6.68E-06 | -0.3003 | 0 | 0.120 |
| ENSP00000369566 | 8.43E-06 | -0.3003 | 0 | 0.418 |
| ENSP00000416753 | 6.26E-06 | -0.3004 | 0 | 0.086 |
| ENSP00000328326 | 3.12E-06 | -0.3004 | 0 | 0.085 |
| ENSP00000284770 | 9.06E-06 | -0.3004 | 0 | 0.403 |
| ENSP00000301656 | 7.81E-06 | -0.3005 | 0 | 0.122 |
| ENSP00000346560 | 4.90E-06 | -0.3006 | 0 | 0.169 |
| ENSP00000381739 | 1.26E-05 | -0.3006 | 829 | 0.895 |
| ENSP00000222122 | 9.37E-06 | -0.3006 | 0 | 0.527 |
| ENSP00000286063 | 5.47E-06 | -0.3007 | 511 | 0.657 |
| ENSP00000454500 | 2.18E-06 | -0.3008 | 0 | 0.000 |
| ENSP00000301263 | 1.06E-05 | -0.3009 | 0 | 0.210 |
| ENSP00000380250 | 3.17E-06 | -0.3010 | 0 | 0.047 |
| ENSP00000227348 | 6.26E-06 | -0.3010 | 0 | 0.093 |
| ENSP00000427772 | 6.03E-06 | -0.3011 | 211 | 0.302 |
| ENSP00000368737 | 1.49E-06 | -0.3011 | 0 | 0.202 |
| ENSP00000363036 | 3.84E-06 | -0.3011 | 0 | 0.000 |
| ENSP00000380692 | 2.79E-06 | -0.3011 | 0 | 0.000 |
| ENSP00000320025 | 2.85E-06 | -0.3013 | 0 | 0.254 |
| ENSP00000340677 | 1.23E-05 | -0.3013 | 835 | 0.875 |
| ENSP00000331915 | 6.76E-06 | -0.3014 | 0 | 0.136 |
| ENSP00000355900 | 3.46E-06 | -0.3014 | 0 | 0.118 |
| ENSP00000272133 | 4.42E-06 | -0.3014 | 0 | 0.078 |
| ENSP00000292114 | 3.73E-06 | -0.3014 | 0 | 0.100 |
| ENSP00000456434 | 5.98E-06 | -0.3015 | 246 | 0.000 |
| ENSP00000358515 | 4.62E-06 | -0.3015 | 0 | 0.073 |
| ENSP00000247194 | 3.69E-06 | -0.3016 | 0 | 0.103 |
| ENSP00000345972 | 4.91E-06 | -0.3016 | 0 | 0.114 |
| ENSP00000357682 | 6.39E-06 | -0.3017 | 202 | 0.636 |
| ENSP00000374482 | 2.01E-06 | -0.3017 | 0 | 0.134 |

| | | | | |
|---|---|---|---|---|
| ENSP00000295092 | 4.79E-06 | -0.3017 | 0 | 0.084 |
| ENSP00000361557 | 1.92E-06 | -0.3018 | 0 | 0.231 |
| ENSP00000309538 | 9.04E-06 | -0.3018 | 151 | 0.105 |
| ENSP00000353791 | 3.91E-06 | -0.3019 | 0 | 0.075 |
| ENSP00000303518 | 6.25E-06 | -0.3019 | 201 | 0.541 |
| ENSP00000216177 | 6.18E-06 | -0.3019 | 0 | 0.136 |
| ENSP00000307617 | 3.67E-06 | -0.3020 | 0 | 0.000 |
| ENSP00000406674 | 3.39E-06 | -0.3020 | 0 | 0.362 |
| ENSP00000359940 | 4.48E-06 | -0.3020 | 0 | 0.124 |
| ENSP00000356094 | 8.30E-06 | -0.3021 | 303 | 0.284 |
| ENSP00000290871 | 6.94E-06 | -0.3021 | 0 | 0.082 |
| ENSP00000355577 | 2.68E-06 | -0.3022 | 0 | 0.098 |
| ENSP00000339750 | 3.51E-06 | -0.3022 | 0 | 0.140 |
| ENSP00000355467 | 3.54E-06 | -0.3024 | 0 | 0.070 |
| ENSP00000388001 | 2.75E-05 | -0.3025 | 903 | 0.667 |
| ENSP00000308870 | 5.34E-06 | -0.3025 | 0 | 0.103 |
| ENSP00000441949 | 8.13E-07 | -0.3025 | 0 | 0.156 |
| ENSP00000324901 | 6.17E-06 | -0.3025 | 0 | 0.097 |
| ENSP00000363634 | 5.58E-06 | -0.3025 | 355 | 0.521 |
| ENSP00000342535 | 2.46E-06 | -0.3026 | 0 | 0.096 |
| ENSP00000364486 | 5.64E-06 | -0.3026 | 153 | 0.669 |
| ENSP00000452776 | 3.64E-06 | -0.3026 | 0 | 0.097 |
| ENSP00000352101 | 8.41E-06 | -0.3026 | 153 | 0.000 |
| ENSP00000272369 | 1.02E-05 | -0.3027 | 163 | 0.821 |
| ENSP00000275767 | 8.89E-06 | -0.3029 | 0 | 0.107 |
| ENSP00000245812 | 3.57E-06 | -0.3030 | 0 | 0.078 |
| ENSP00000349085 | 2.17E-06 | -0.3030 | 0 | 0.495 |
| ENSP00000311035 | 7.95E-06 | -0.3030 | 0 | 0.197 |
| ENSP00000345420 | 4.64E-06 | -0.3031 | 0 | 0.557 |
| ENSP00000400157 | 2.18E-06 | -0.3031 | 0 | 0.069 |
| ENSP00000365040 | 6.80E-06 | -0.3032 | 162 | 0.000 |
| ENSP00000365817 | 4.28E-06 | -0.3033 | 0 | 0.438 |
| ENSP00000360690 | 7.12E-06 | -0.3033 | 208 | 0.000 |
| ENSP00000359795 | 5.32E-06 | -0.3033 | 0 | 0.233 |
| ENSP00000244241 | 6.66E-06 | -0.3033 | 0 | 0.374 |
| ENSP00000435061 | 1.10E-06 | -0.3034 | 0 | 0.175 |
| ENSP00000280481 | 9.54E-06 | -0.3034 | 206 | 0.186 |
| ENSP00000341610 | 4.68E-06 | -0.3034 | 0 | 0.161 |
| ENSP00000253435 | 7.10E-06 | -0.3035 | 0 | 0.000 |
| ENSP00000286760 | 3.68E-06 | -0.3036 | 0 | 0.148 |
| ENSP00000363727 | 5.27E-06 | -0.3037 | 0 | 0.374 |
| ENSP00000286122 | 3.66E-06 | -0.3038 | 0 | 0.459 |
| ENSP00000302274 | 7.98E-06 | -0.3038 | 0 | 0.099 |
| ENSP00000381049 | 2.90E-06 | -0.3038 | 0 | 0.000 |
| ENSP00000368982 | 4.54E-06 | -0.3040 | 0 | 0.252 |
| ENSP00000373873 | 3.60E-06 | -0.3040 | 0 | 0.240 |

| | | | | |
|---|---|---|---|---|
| ENSP00000272321 | 7.05E-06 | -0.3041 | 0 | 0.255 |
| ENSP00000368994 | 2.26E-06 | -0.3041 | 0 | 0.256 |
| ENSP00000348775 | 5.10E-06 | -0.3041 | 0 | 0.317 |
| ENSP00000326538 | 1.28E-05 | -0.3041 | 158 | 0.085 |
| ENSP00000356123 | 3.35E-06 | -0.3041 | 0 | 0.106 |
| ENSP00000456318 | 1.80E-06 | -0.3042 | 0 | 0.000 |
| ENSP00000358016 | 2.64E-06 | -0.3044 | 0 | 0.201 |
| ENSP00000358786 | 2.69E-06 | -0.3044 | 0 | 0.097 |
| ENSP00000249075 | 9.93E-06 | -0.3044 | 0 | 0.775 |
| ENSP00000402219 | 2.18E-06 | -0.3045 | 0 | 0.107 |
| ENSP00000326110 | 6.49E-06 | -0.3047 | 0 | 0.101 |
| ENSP00000428480 | 5.27E-06 | -0.3047 | 0 | 0.869 |
| ENSP00000393379 | 4.17E-06 | -0.3047 | 0 | 0.289 |
| ENSP00000264703 | 9.49E-06 | -0.3048 | 0 | 0.105 |
| ENSP00000355001 | 5.98E-06 | -0.3049 | 0 | 0.824 |
| ENSP00000374399 | 3.22E-06 | -0.3050 | 0 | 0.815 |
| ENSP00000244333 | 9.00E-06 | -0.3051 | 0 | 0.186 |
| ENSP00000264992 | 7.16E-06 | -0.3051 | 0 | 0.077 |
| ENSP00000358460 | 7.67E-06 | -0.3052 | 271 | 0.152 |
| ENSP00000417658 | 5.60E-06 | -0.3052 | 0 | 0.067 |
| ENSP00000330278 | 5.69E-06 | -0.3052 | 0 | 0.093 |
| ENSP00000275358 | 6.25E-06 | -0.3054 | 0 | 0.088 |
| ENSP00000381781 | 3.40E-06 | -0.3056 | 0 | 0.000 |
| ENSP00000331470 | 5.64E-06 | -0.3057 | 0 | 0.720 |
| ENSP00000413228 | 2.69E-06 | -0.3057 | 0 | 0.000 |
| ENSP00000417378 | 2.82E-06 | -0.3057 | 0 | 0.135 |
| ENSP00000267260 | 5.74E-06 | -0.3058 | 0 | 0.228 |
| ENSP00000389252 | 5.95E-06 | -0.3058 | 0 | 0.120 |
| ENSP00000265727 | 5.89E-06 | -0.3059 | 0 | 0.248 |
| ENSP00000347716 | 4.92E-06 | -0.3059 | 0 | 0.558 |
| ENSP00000310193 | 6.02E-06 | -0.3060 | 0 | 0.114 |
| ENSP00000374013 | 2.89E-06 | -0.3060 | 0 | 0.607 |
| ENSP00000253812 | 3.85E-06 | -0.3061 | 0 | 0.149 |
| ENSP00000347003 | 1.86E-06 | -0.3061 | 0 | 0.000 |
| ENSP00000320346 | 3.73E-06 | -0.3061 | 0 | 0.514 |
| ENSP00000405969 | 2.24E-06 | -0.3062 | 0 | 0.118 |
| ENSP00000312834 | 5.45E-06 | -0.3062 | 0 | 0.274 |
| ENSP00000344579 | 3.61E-06 | -0.3063 | 0 | 0.112 |
| ENSP00000362187 | 2.46E-06 | -0.3064 | 0 | 0.118 |
| ENSP00000221847 | 7.42E-06 | -0.3064 | 0 | 0.243 |
| ENSP00000399588 | 1.48E-06 | -0.3064 | 0 | 0.253 |
| ENSP00000377547 | 2.72E-06 | -0.3066 | 0 | 0.130 |
| ENSP00000357556 | 4.22E-06 | -0.3067 | 0 | 0.606 |
| ENSP00000253354 | 9.62E-06 | -0.3069 | 0 | 0.107 |
| ENSP00000368684 | 3.76E-06 | -0.3069 | 0 | 0.370 |
| ENSP00000386770 | 3.81E-06 | -0.3069 | 0 | 0.105 |

| | | | | |
|---|---|---|---|---|
| ENSP00000184183 | 7.09E-06 | -0.3069 | 0 | 0.093 |
| ENSP00000307264 | 1.88E-06 | -0.3070 | 0 | 0.164 |
| ENSP00000355523 | 1.33E-05 | -0.3070 | 252 | 0.614 |
| ENSP00000455561 | 1.18E-06 | -0.3070 | 0 | 0.000 |
| ENSP00000369943 | 3.19E-06 | -0.3071 | 0 | 0.082 |
| ENSP00000363624 | 2.92E-06 | -0.3073 | 0 | 0.160 |
| ENSP00000256652 | 8.63E-06 | -0.3074 | 0 | 0.129 |
| ENSP00000381399 | 8.59E-06 | -0.3074 | 0 | 0.065 |
| ENSP00000347997 | 2.43E-06 | -0.3076 | 0 | 0.139 |
| ENSP00000308591 | 4.06E-06 | -0.3076 | 0 | 0.084 |
| ENSP00000242315 | 3.11E-06 | -0.3076 | 0 | 0.000 |
| ENSP00000334594 | 4.31E-06 | -0.3076 | 0 | 0.075 |
| ENSP00000357540 | 5.59E-06 | -0.3076 | 0 | 0.104 |
| ENSP00000340839 | 5.35E-06 | -0.3077 | 487 | 0.106 |
| ENSP00000286482 | 6.28E-06 | -0.3077 | 0 | 0.216 |
| ENSP00000366977 | 3.99E-06 | -0.3077 | 0 | 0.185 |
| ENSP00000423541 | 2.67E-06 | -0.3077 | 0 | 0.074 |
| ENSP00000365473 | 2.71E-06 | -0.3079 | 0 | 0.085 |
| ENSP00000468977 | 3.97E-06 | -0.3080 | 0 | 0.836 |
| ENSP00000250894 | 5.54E-06 | -0.3080 | 0 | 0.425 |
| ENSP00000249116 | 7.13E-06 | -0.3080 | 0 | 0.092 |
| ENSP00000346412 | 2.10E-06 | -0.3081 | 0 | 0.105 |
| ENSP00000274565 | 7.41E-06 | -0.3081 | 0 | 0.126 |
| ENSP00000284136 | 1.36E-05 | -0.3081 | 0 | 0.502 |
| ENSP00000225275 | 1.53E-05 | -0.3082 | 0 | 0.175 |
| ENSP00000361483 | 5.12E-06 | -0.3082 | 0 | 0.138 |
| ENSP00000402861 | 4.74E-06 | -0.3082 | 0 | 0.532 |
| ENSP00000353142 | 4.80E-06 | -0.3083 | 0 | 0.080 |
| ENSP00000367756 | 3.11E-06 | -0.3083 | 0 | 0.296 |
| ENSP00000334564 | 3.22E-06 | -0.3084 | 0 | 0.740 |
| ENSP00000326652 | 2.99E-06 | -0.3084 | 0 | 0.148 |
| ENSP00000259205 | 1.18E-05 | -0.3084 | 0 | 0.318 |
| ENSP00000246896 | 7.62E-06 | -0.3084 | 0 | 0.099 |
| ENSP00000421380 | 3.51E-06 | -0.3084 | 0 | 0.256 |
| ENSP00000366586 | 2.13E-06 | -0.3084 | 0 | 0.097 |
| ENSP00000345055 | 4.35E-06 | -0.3084 | 0 | 0.186 |
| ENSP00000398277 | 2.08E-06 | -0.3086 | 0 | 0.000 |
| ENSP00000318128 | 5.58E-06 | -0.3087 | 0 | 0.095 |
| ENSP00000436714 | 9.32E-06 | -0.3088 | 206 | 0.504 |
| ENSP00000367747 | 8.41E-06 | -0.3088 | 0 | 0.446 |
| ENSP00000293636 | 9.56E-06 | -0.3089 | 0 | 0.205 |
| ENSP00000355775 | 1.10E-05 | -0.3090 | 158 | 0.866 |
| ENSP00000276708 | 2.75E-06 | -0.3090 | 0 | 0.107 |
| ENSP00000353542 | 2.75E-06 | -0.3090 | 0 | 0.111 |
| ENSP00000420037 | 2.25E-06 | -0.3090 | 0 | 0.090 |
| ENSP00000358207 | 3.86E-06 | -0.3091 | 0 | 0.750 |

| | | | | |
|---|---|---|---|---|
| ENSP00000237201 | 5.64E-06 | -0.3091 | 0 | 0.130 |
| ENSP00000365601 | 2.86E-06 | -0.3092 | 0 | 0.076 |
| ENSP00000347294 | 5.84E-06 | -0.3093 | 0 | 0.750 |
| ENSP00000292199 | 4.19E-06 | -0.3093 | 0 | 0.361 |
| ENSP00000414624 | 5.15E-06 | -0.3093 | 201 | 0.569 |
| ENSP00000289429 | 7.62E-06 | -0.3094 | 0 | 0.131 |
| ENSP00000370639 | 7.29E-06 | -0.3094 | 198 | 0.105 |
| ENSP00000303192 | 5.68E-06 | -0.3094 | 0 | 0.434 |
| ENSP00000298251 | 5.80E-06 | -0.3094 | 0 | 0.170 |
| ENSP00000336769 | 4.12E-06 | -0.3095 | 0 | 0.159 |
| ENSP00000340979 | 4.27E-06 | -0.3096 | 0 | 0.097 |
| ENSP00000248701 | 4.44E-06 | -0.3097 | 0 | 0.127 |
| ENSP00000335247 | 3.45E-06 | -0.3097 | 0 | 0.099 |
| ENSP00000324633 | 1.91E-06 | -0.3098 | 0 | 0.077 |
| ENSP00000309767 | 7.47E-06 | -0.3098 | 0 | 0.122 |
| ENSP00000440272 | 4.10E-06 | -0.3099 | 179 | 0.000 |
| ENSP00000334854 | 6.40E-06 | -0.3099 | 0 | 0.097 |
| ENSP00000340811 | 5.67E-06 | -0.3099 | 0 | 0.084 |
| ENSP00000355419 | 4.06E-06 | -0.3099 | 0 | 0.106 |
| ENSP00000286479 | 9.89E-06 | -0.3100 | 216 | 0.382 |
| ENSP00000419361 | 9.47E-06 | -0.3101 | 0 | 0.646 |
| ENSP00000429275 | 2.26E-06 | -0.3103 | 0 | 0.088 |
| ENSP00000387888 | 2.99E-06 | -0.3103 | 0 | 0.000 |
| ENSP00000329920 | 8.59E-06 | -0.3103 | 0 | 0.000 |
| ENSP00000319531 | 4.40E-06 | -0.3105 | 0 | 0.275 |
| ENSP00000384290 | 4.93E-06 | -0.3105 | 0 | 0.084 |
| ENSP00000354579 | 4.25E-06 | -0.3105 | 0 | 0.134 |
| ENSP00000370936 | 2.92E-06 | -0.3106 | 0 | 0.865 |
| ENSP00000369574 | 3.48E-06 | -0.3106 | 0 | 0.427 |
| ENSP00000305200 | 9.60E-06 | -0.3108 | 271 | 0.128 |
| ENSP00000370800 | 3.05E-06 | -0.3110 | 0 | 0.669 |
| ENSP00000386733 | 2.53E-06 | -0.3110 | 0 | 0.000 |
| ENSP00000290122 | 8.67E-06 | -0.3110 | 0 | 0.159 |
| ENSP00000261530 | 9.15E-06 | -0.3112 | 0 | 0.073 |
| ENSP00000295461 | 2.88E-06 | -0.3112 | 0 | 0.093 |
| ENSP00000323568 | 1.12E-05 | -0.3113 | 253 | 0.664 |
| ENSP00000220940 | 1.04E-05 | -0.3114 | 0 | 0.158 |
| ENSP00000277309 | 1.04E-06 | -0.3115 | 0 | 0.000 |
| ENSP00000341765 | 9.41E-06 | -0.3116 | 0 | 0.114 |
| ENSP00000340369 | 3.76E-06 | -0.3116 | 0 | 0.000 |
| ENSP00000329454 | 4.08E-06 | -0.3117 | 0 | 0.278 |
| ENSP00000298892 | 3.66E-06 | -0.3118 | 0 | 0.236 |
| ENSP00000390761 | 1.76E-06 | -0.3118 | 0 | 0.000 |
| ENSP00000400325 | 2.34E-06 | -0.3118 | 0 | 0.000 |
| ENSP00000397181 | 1.46E-05 | -0.3118 | 360 | 0.767 |
| ENSP00000353942 | 3.63E-06 | -0.3119 | 0 | 0.000 |

| | | | | |
|---|---|---|---|---|
| ENSP00000359521 | 2.66E-06 | -0.3119 | 0 | 0.080 |
| ENSP00000420927 | 4.58E-06 | -0.3120 | 185 | 0.492 |
| ENSP00000330601 | 5.89E-06 | -0.3120 | 0 | 0.131 |
| ENSP00000352927 | 5.55E-06 | -0.3121 | 0 | 0.000 |
| ENSP00000290374 | 3.24E-06 | -0.3121 | 0 | 0.123 |
| ENSP00000273371 | 7.55E-06 | -0.3122 | 0 | 0.071 |
| ENSP00000262095 | 6.06E-06 | -0.3124 | 0 | 0.000 |
| ENSP00000328563 | 1.09E-06 | -0.3124 | 0 | 0.000 |
| ENSP00000302724 | 5.38E-06 | -0.3125 | 0 | 0.133 |
| ENSP00000320234 | 4.49E-06 | -0.3125 | 0 | 0.093 |
| ENSP00000312368 | 7.09E-06 | -0.3127 | 0 | 0.112 |
| ENSP00000369238 | 4.74E-06 | -0.3127 | 0 | 0.120 |
| ENSP00000326693 | 2.18E-06 | -0.3128 | 0 | 0.064 |
| ENSP00000297468 | 2.98E-06 | -0.3128 | 0 | 0.161 |
| ENSP00000364520 | 8.23E-06 | -0.3129 | 396 | 0.415 |
| ENSP00000428288 | 2.23E-06 | -0.3129 | 0 | 0.111 |
| ENSP00000328402 | 1.60E-05 | -0.3130 | 159 | 0.000 |
| ENSP00000359607 | 4.94E-06 | -0.3131 | 0 | 0.107 |
| ENSP00000218197 | 7.26E-06 | -0.3131 | 0 | 0.104 |
| ENSP00000404464 | 4.20E-06 | -0.3131 | 0 | 0.175 |
| ENSP00000302144 | 3.49E-06 | -0.3132 | 0 | 0.179 |
| ENSP00000260403 | 3.31E-06 | -0.3132 | 0 | 0.105 |
| ENSP00000364249 | 6.65E-06 | -0.3133 | 176 | 0.249 |
| ENSP00000286809 | 6.34E-06 | -0.3134 | 0 | 0.000 |
| ENSP00000303712 | 4.48E-06 | -0.3135 | 0 | 0.520 |
| ENSP00000374145 | 8.76E-06 | -0.3136 | 207 | 0.219 |
| ENSP00000289272 | 9.87E-06 | -0.3137 | 0 | 0.095 |
| ENSP00000370844 | 8.97E-06 | -0.3137 | 230 | 0.334 |
| ENSP00000364597 | 6.45E-06 | -0.3137 | 0 | 0.455 |
| ENSP00000303686 | 8.38E-06 | -0.3138 | 159 | 0.496 |
| ENSP00000351068 | 1.65E-06 | -0.3139 | 0 | 0.134 |
| ENSP00000233615 | 7.79E-06 | -0.3140 | 0 | 0.137 |
| ENSP00000312002 | 6.78E-06 | -0.3141 | 0 | 0.464 |
| ENSP00000364145 | 6.84E-06 | -0.3141 | 174 | 0.304 |
| ENSP00000303532 | 2.96E-06 | -0.3142 | 0 | 0.100 |
| ENSP00000339377 | 4.73E-06 | -0.3142 | 0 | 0.503 |
| ENSP00000320951 | 2.37E-06 | -0.3142 | 0 | 0.088 |
| ENSP00000386229 | 1.36E-05 | -0.3143 | 188 | 0.564 |
| ENSP00000358777 | 3.42E-06 | -0.3143 | 0 | 0.055 |
| ENSP00000344125 | 3.32E-06 | -0.3143 | 0 | 0.000 |
| ENSP00000198536 | 9.00E-06 | -0.3144 | 0 | 0.168 |
| ENSP00000334463 | 6.78E-06 | -0.3146 | 0 | 0.111 |
| ENSP00000355167 | 3.68E-06 | -0.3147 | 0 | 0.341 |
| ENSP00000278243 | 7.77E-06 | -0.3147 | 0 | 0.106 |
| ENSP00000258436 | 4.72E-06 | -0.3147 | 0 | 0.074 |
| ENSP00000360312 | 4.17E-06 | -0.3147 | 0 | 0.138 |

| | | | | |
|---|---|---|---|---|
| ENSP00000219689 | 2.81E-06 | -0.3148 | 0 | 0.080 |
| ENSP00000278187 | 7.02E-06 | -0.3149 | 0 | 0.330 |
| ENSP00000350475 | 7.06E-06 | -0.3149 | 0 | 0.000 |
| ENSP00000429553 | 3.87E-06 | -0.3149 | 0 | 0.655 |
| ENSP00000358902 | 1.28E-06 | -0.3151 | 0 | 0.105 |
| ENSP00000296736 | 4.15E-06 | -0.3151 | 0 | 0.165 |
| ENSP00000268389 | 8.26E-06 | -0.3151 | 0 | 0.122 |
| ENSP00000304437 | 5.23E-06 | -0.3151 | 0 | 0.098 |
| ENSP00000282611 | 4.17E-06 | -0.3152 | 0 | 0.064 |
| ENSP00000369578 | 4.76E-06 | -0.3152 | 0 | 0.156 |
| ENSP00000251642 | 3.83E-06 | -0.3152 | 0 | 0.648 |
| ENSP00000323328 | 3.06E-06 | -0.3153 | 0 | 0.121 |
| ENSP00000362869 | 2.83E-06 | -0.3153 | 0 | 0.090 |
| ENSP00000356320 | 4.68E-06 | -0.3153 | 0 | 0.341 |
| ENSP00000362206 | 3.40E-06 | -0.3154 | 0 | 0.120 |
| ENSP00000305852 | 7.53E-06 | -0.3154 | 0 | 0.082 |
| ENSP00000349174 | 4.44E-06 | -0.3154 | 0 | 0.093 |
| ENSP00000361010 | 7.22E-06 | -0.3154 | 205 | 0.830 |
| ENSP00000363329 | 3.35E-06 | -0.3155 | 0 | 0.089 |
| ENSP00000281441 | 6.11E-06 | -0.3157 | 0 | 0.128 |
| ENSP00000332772 | 2.16E-06 | -0.3159 | 0 | 0.159 |
| ENSP00000466399 | 6.66E-06 | -0.3163 | 340 | 0.000 |
| ENSP00000330070 | 9.03E-06 | -0.3163 | 158 | 0.507 |
| ENSP00000368080 | 3.86E-06 | -0.3163 | 0 | 0.282 |
| ENSP00000470059 | 6.46E-06 | -0.3163 | 169 | 0.000 |
| ENSP00000421172 | 4.46E-06 | -0.3163 | 0 | 0.149 |
| ENSP00000403536 | 5.64E-06 | -0.3164 | 0 | 0.716 |
| ENSP00000348897 | 2.64E-06 | -0.3164 | 0 | 0.257 |
| ENSP00000344353 | 1.04E-05 | -0.3164 | 210 | 0.480 |
| ENSP00000310891 | 6.05E-06 | -0.3164 | 0 | 0.000 |
| ENSP00000369785 | 5.13E-06 | -0.3165 | 0 | 0.202 |
| ENSP00000354574 | 3.47E-06 | -0.3166 | 0 | 0.093 |
| ENSP00000216487 | 6.21E-06 | -0.3166 | 0 | 0.140 |
| ENSP00000329803 | 2.67E-06 | -0.3168 | 0 | 0.217 |
| ENSP00000259351 | 4.21E-06 | -0.3169 | 0 | 0.095 |
| ENSP00000362255 | 2.91E-06 | -0.3170 | 0 | 0.064 |
| ENSP00000309673 | 8.39E-07 | -0.3170 | 0 | 0.000 |
| ENSP00000304846 | 9.10E-07 | -0.3171 | 0 | 0.000 |
| ENSP00000296980 | 7.03E-06 | -0.3171 | 0 | 0.478 |
| ENSP00000302543 | 8.45E-06 | -0.3172 | 0 | 0.223 |
| ENSP00000267499 | 6.77E-06 | -0.3172 | 0 | 0.000 |
| ENSP00000363031 | 3.59E-06 | -0.3174 | 0 | 0.115 |
| ENSP00000310657 | 1.07E-05 | -0.3174 | 213 | 0.346 |
| ENSP00000328698 | 3.67E-06 | -0.3176 | 0 | 0.088 |
| ENSP00000334879 | 4.52E-06 | -0.3176 | 0 | 0.165 |
| ENSP00000269503 | 6.71E-06 | -0.3177 | 0 | 0.132 |

| | | | | |
|---|---|---|---|---|
| ENSP00000256585 | 1.05E-05 | -0.3180 | 0 | 0.104 |
| ENSP00000303575 | 9.99E-06 | -0.3180 | 163 | 0.337 |
| ENSP00000272641 | 5.77E-06 | -0.3180 | 0 | 0.264 |
| ENSP00000357060 | 6.12E-06 | -0.3181 | 211 | 0.414 |
| ENSP00000348132 | 2.42E-06 | -0.3181 | 0 | 0.111 |
| ENSP00000361072 | 4.90E-06 | -0.3181 | 0 | 0.116 |
| ENSP00000388223 | 3.87E-06 | -0.3182 | 0 | 0.000 |
| ENSP00000361281 | 3.15E-06 | -0.3183 | 0 | 0.063 |
| ENSP00000272203 | 2.68E-06 | -0.3184 | 0 | 0.198 |
| ENSP00000388207 | 1.97E-06 | -0.3184 | 0 | 0.119 |
| ENSP00000366488 | 8.32E-06 | -0.3185 | 587 | 0.634 |
| ENSP00000325561 | 4.29E-06 | -0.3185 | 0 | 0.107 |
| ENSP00000419395 | 2.72E-06 | -0.3186 | 0 | 0.135 |
| ENSP00000293218 | 7.03E-06 | -0.3186 | 0 | 0.000 |
| ENSP00000322649 | 3.72E-06 | -0.3187 | 0 | 0.103 |
| ENSP00000326598 | 6.34E-06 | -0.3187 | 0 | 0.154 |
| ENSP00000459775 | 6.58E-06 | -0.3187 | 202 | 0.687 |
| ENSP00000295542 | 6.14E-06 | -0.3188 | 0 | 0.160 |
| ENSP00000298815 | 3.07E-06 | -0.3189 | 0 | 0.181 |
| ENSP00000324510 | 3.50E-06 | -0.3190 | 0 | 0.084 |
| ENSP00000419751 | 8.62E-06 | -0.3190 | 165 | 0.312 |
| ENSP00000358853 | 3.40E-06 | -0.3190 | 0 | 0.153 |
| ENSP00000364919 | 6.66E-06 | -0.3191 | 448 | 0.591 |
| ENSP00000216338 | 7.64E-06 | -0.3191 | 0 | 0.196 |
| ENSP00000395902 | 5.11E-06 | -0.3191 | 0 | 0.000 |
| ENSP00000244669 | 5.00E-06 | -0.3192 | 0 | 0.083 |
| ENSP00000297581 | 1.21E-05 | -0.3193 | 0 | 0.118 |
| ENSP00000329165 | 3.42E-06 | -0.3193 | 0 | 0.121 |
| ENSP00000369135 | 2.02E-06 | -0.3193 | 0 | 0.061 |
| ENSP00000364013 | 2.48E-06 | -0.3194 | 0 | 0.115 |
| ENSP00000218516 | 9.02E-06 | -0.3194 | 0 | 0.141 |
| ENSP00000395983 | 4.32E-06 | -0.3194 | 0 | 0.097 |
| ENSP00000429473 | 3.91E-06 | -0.3198 | 0 | 0.058 |
| ENSP00000442778 | 5.07E-06 | -0.3199 | 0 | 0.000 |
| ENSP00000377616 | 1.64E-06 | -0.3199 | 0 | 0.000 |
| ENSP00000301159 | 3.29E-06 | -0.3200 | 0 | 0.300 |
| ENSP00000231721 | 7.01E-06 | -0.3201 | 0 | 0.339 |
| ENSP00000362524 | 6.42E-06 | -0.3201 | 0 | 0.268 |
| ENSP00000217299 | 6.83E-06 | -0.3201 | 0 | 0.000 |
| ENSP00000342560 | 7.99E-06 | -0.3201 | 197 | 0.504 |
| ENSP00000339390 | 5.79E-06 | -0.3202 | 0 | 0.091 |
| ENSP00000255039 | 8.23E-06 | -0.3202 | 0 | 0.092 |
| ENSP00000331867 | 8.82E-06 | -0.3202 | 403 | 0.383 |
| ENSP00000334940 | 7.34E-06 | -0.3203 | 0 | 0.392 |
| ENSP00000367462 | 2.03E-06 | -0.3204 | 0 | 0.054 |
| ENSP00000306888 | 5.22E-06 | -0.3204 | 0 | 0.081 |

| | | | | |
|---|---|---|---|---|
| ENSP00000344577 | 2.47E-06 | -0.3205 | 0 | 0.187 |
| ENSP00000371548 | 4.93E-06 | -0.3205 | 0 | 0.103 |
| ENSP00000215917 | 9.00E-06 | -0.3207 | 0 | 0.081 |
| ENSP00000400312 | 2.91E-06 | -0.3207 | 0 | 0.343 |
| ENSP00000346442 | 3.35E-06 | -0.3208 | 0 | 0.155 |
| ENSP00000456953 | 8.40E-06 | -0.3208 | 201 | 0.571 |
| ENSP00000308361 | 9.35E-06 | -0.3208 | 207 | 0.470 |
| ENSP00000457718 | 1.59E-06 | -0.3209 | 0 | 0.000 |
| ENSP00000216024 | 2.60E-06 | -0.3209 | 340 | 0.000 |
| ENSP00000268271 | 6.49E-06 | -0.3212 | 0 | 0.000 |
| ENSP00000337757 | 2.91E-06 | -0.3212 | 0 | 0.139 |
| ENSP00000223528 | 7.79E-06 | -0.3214 | 0 | 0.184 |
| ENSP00000434045 | 4.26E-06 | -0.3215 | 0 | 0.423 |
| ENSP00000276943 | 5.61E-06 | -0.3215 | 0 | 0.207 |
| ENSP00000266086 | 1.01E-05 | -0.3215 | 200 | 0.145 |
| ENSP00000233331 | 2.37E-06 | -0.3216 | 0 | 0.200 |
| ENSP00000321077 | 3.20E-06 | -0.3217 | 0 | 0.115 |
| ENSP00000311687 | 3.71E-06 | -0.3219 | 0 | 0.072 |
| ENSP00000359121 | 6.03E-06 | -0.3219 | 0 | 0.089 |
| ENSP00000344226 | 4.16E-06 | -0.3219 | 0 | 0.000 |
| ENSP00000415332 | 2.64E-06 | -0.3219 | 0 | 0.083 |
| ENSP00000335038 | 4.48E-06 | -0.3220 | 0 | 0.112 |
| ENSP00000355866 | 7.52E-06 | -0.3222 | 158 | 0.757 |
| ENSP00000237642 | 5.36E-06 | -0.3222 | 0 | 0.000 |
| ENSP00000377717 | 9.35E-06 | -0.3225 | 900 | 0.128 |
| ENSP00000279058 | 6.74E-06 | -0.3226 | 0 | 0.124 |
| ENSP00000247992 | 7.37E-06 | -0.3227 | 0 | 0.179 |
| ENSP00000284224 | 3.51E-06 | -0.3228 | 0 | 0.066 |
| ENSP00000310593 | 6.33E-06 | -0.3228 | 301 | 0.155 |
| ENSP00000399970 | 3.69E-06 | -0.3228 | 0 | 0.440 |
| ENSP00000268695 | 6.22E-06 | -0.3229 | 0 | 0.092 |
| ENSP00000394168 | 4.54E-06 | -0.3230 | 0 | 0.160 |
| ENSP00000353346 | 1.30E-05 | -0.3231 | 582 | 0.191 |
| ENSP00000377043 | 3.18E-06 | -0.3231 | 0 | 0.080 |
| ENSP00000358739 | 3.06E-06 | -0.3232 | 0 | 0.209 |
| ENSP00000296978 | 6.50E-06 | -0.3233 | 0 | 0.149 |
| ENSP00000307041 | 7.37E-06 | -0.3233 | 0 | 0.112 |
| ENSP00000302260 | 2.75E-06 | -0.3235 | 0 | 0.160 |
| ENSP00000252453 | 1.03E-05 | -0.3237 | 0 | 0.094 |
| ENSP00000364348 | 3.12E-06 | -0.3239 | 0 | 0.000 |
| ENSP00000384396 | 2.61E-06 | -0.3239 | 0 | 0.000 |
| ENSP00000259030 | 8.53E-06 | -0.3240 | 0 | 0.096 |
| ENSP00000275198 | 9.06E-06 | -0.3241 | 209 | 0.174 |
| ENSP00000327533 | 3.42E-06 | -0.3241 | 0 | 0.118 |
| ENSP00000334996 | 4.05E-06 | -0.3242 | 0 | 0.110 |
| ENSP00000332886 | 6.15E-06 | -0.3242 | 0 | 0.125 |

| | | | | |
|---|---|---|---|---|
| ENSP00000344260 | 4.02E-06 | -0.3242 | 0 | 0.085 |
| ENSP00000356846 | 2.09E-06 | -0.3242 | 0 | 0.129 |
| ENSP00000342889 | 5.27E-06 | -0.3242 | 187 | 0.778 |
| ENSP00000248072 | 1.55E-05 | -0.3243 | 207 | 0.000 |
| ENSP00000261588 | 4.74E-06 | -0.3243 | 0 | 0.111 |
| ENSP00000254853 | 5.20E-06 | -0.3244 | 0 | 0.080 |
| ENSP00000330031 | 3.72E-06 | -0.3245 | 0 | 0.509 |
| ENSP00000359594 | 2.89E-06 | -0.3246 | 0 | 0.093 |
| ENSP00000417659 | 6.83E-06 | -0.3246 | 196 | 0.675 |
| ENSP00000294484 | 4.19E-06 | -0.3246 | 0 | 0.086 |
| ENSP00000356308 | 4.98E-06 | -0.3246 | 0 | 0.291 |
| ENSP00000328359 | 5.11E-06 | -0.3247 | 0 | 0.143 |
| ENSP00000195455 | 7.85E-06 | -0.3247 | 0 | 0.000 |
| ENSP00000258749 | 9.20E-06 | -0.3247 | 0 | 0.000 |
| ENSP00000370531 | 3.08E-06 | -0.3249 | 0 | 0.095 |
| ENSP00000319166 | 5.21E-06 | -0.3249 | 0 | 0.135 |
| ENSP00000303276 | 6.54E-06 | -0.3249 | 0 | 0.383 |
| ENSP00000441462 | 3.33E-06 | -0.3249 | 0 | 0.055 |
| ENSP00000370192 | 1.99E-06 | -0.3251 | 0 | 0.114 |
| ENSP00000309270 | 1.01E-05 | -0.3251 | 974 | 0.069 |
| ENSP00000350914 | 7.09E-06 | -0.3252 | 0 | 0.300 |
| ENSP00000255189 | 1.05E-05 | -0.3252 | 235 | 0.399 |
| ENSP00000321026 | 4.84E-06 | -0.3253 | 0 | 0.115 |
| ENSP00000300113 | 2.70E-06 | -0.3253 | 0 | 0.000 |
| ENSP00000344942 | 3.63E-06 | -0.3254 | 0 | 0.103 |
| ENSP00000240615 | 5.32E-06 | -0.3254 | 0 | 0.171 |
| ENSP00000359131 | 3.70E-06 | -0.3254 | 0 | 0.070 |
| ENSP00000354950 | 8.00E-06 | -0.3254 | 329 | 0.334 |
| ENSP00000256737 | 4.83E-06 | -0.3254 | 0 | 0.102 |
| ENSP00000418994 | 4.25E-06 | -0.3256 | 0 | 0.164 |
| ENSP00000357779 | 4.21E-06 | -0.3256 | 0 | 0.103 |
| ENSP00000306328 | 4.37E-06 | -0.3256 | 0 | 0.076 |
| ENSP00000350718 | 7.32E-06 | -0.3257 | 914 | 0.076 |
| ENSP00000343507 | 3.20E-06 | -0.3257 | 0 | 0.158 |
| ENSP00000358532 | 2.86E-06 | -0.3258 | 0 | 0.129 |
| ENSP00000222033 | 6.35E-06 | -0.3258 | 0 | 0.106 |
| ENSP00000323978 | 4.04E-06 | -0.3259 | 0 | 0.094 |
| ENSP00000261275 | 3.66E-06 | -0.3259 | 0 | 0.164 |
| ENSP00000427888 | 4.09E-06 | -0.3260 | 0 | 0.103 |
| ENSP00000333845 | 4.36E-06 | -0.3260 | 0 | 0.200 |
| ENSP00000248948 | 9.35E-06 | -0.3261 | 0 | 0.106 |
| ENSP00000256969 | 7.33E-06 | -0.3262 | 0 | 0.000 |
| ENSP00000274368 | 6.55E-06 | -0.3262 | 0 | 0.238 |
| ENSP00000271532 | 5.30E-06 | -0.3263 | 0 | 0.244 |
| ENSP00000300557 | 4.47E-06 | -0.3263 | 0 | 0.089 |
| ENSP00000311827 | 3.59E-06 | -0.3265 | 0 | 0.388 |

| | | | | |
|---|---|---|---|---|
| ENSP00000260502 | 6.41E-06 | -0.3266 | 0 | 0.101 |
| ENSP00000354623 | 8.67E-06 | -0.3267 | 349 | 0.244 |
| ENSP00000335261 | 4.14E-06 | -0.3268 | 0 | 0.097 |
| ENSP00000366522 | 3.50E-06 | -0.3269 | 0 | 0.353 |
| ENSP00000400258 | 5.31E-06 | -0.3269 | 0 | 0.071 |
| ENSP00000257776 | 9.48E-06 | -0.3270 | 0 | 0.077 |
| ENSP00000269394 | 5.55E-06 | -0.3271 | 0 | 0.106 |
| ENSP00000305107 | 6.66E-06 | -0.3271 | 0 | 0.085 |
| ENSP00000354478 | 1.10E-05 | -0.3271 | 162 | 0.859 |
| ENSP00000216133 | 4.10E-06 | -0.3272 | 0 | 0.744 |
| ENSP00000363229 | 5.58E-06 | -0.3273 | 0 | 0.507 |
| ENSP00000462023 | 4.89E-06 | -0.3273 | 305 | 0.788 |
| ENSP00000307821 | 5.24E-06 | -0.3274 | 0 | 0.000 |
| ENSP00000342832 | 2.58E-06 | -0.3274 | 0 | 0.105 |
| ENSP00000358064 | 3.45E-06 | -0.3275 | 0 | 0.620 |
| ENSP00000363972 | 4.11E-06 | -0.3276 | 0 | 0.078 |
| ENSP00000329403 | 5.88E-06 | -0.3276 | 0 | 0.075 |
| ENSP00000302629 | 4.24E-06 | -0.3276 | 0 | 0.059 |
| ENSP00000324323 | 6.85E-06 | -0.3276 | 0 | 0.083 |
| ENSP00000292330 | 1.78E-06 | -0.3278 | 0 | 0.127 |
| ENSP00000251646 | 3.75E-06 | -0.3279 | 0 | 0.104 |
| ENSP00000342538 | 5.22E-06 | -0.3279 | 0 | 0.123 |
| ENSP00000303231 | 7.66E-06 | -0.3280 | 0 | 0.699 |
| ENSP00000424424 | 2.90E-06 | -0.3280 | 0 | 0.102 |
| ENSP00000357915 | 2.74E-06 | -0.3281 | 0 | 0.053 |
| ENSP00000342924 | 2.92E-06 | -0.3282 | 0 | 0.488 |
| ENSP00000410732 | 3.42E-06 | -0.3282 | 0 | 0.333 |
| ENSP00000410111 | 2.18E-06 | -0.3282 | 0 | 0.649 |
| ENSP00000289407 | 3.85E-06 | -0.3282 | 0 | 0.000 |
| ENSP00000356172 | 5.41E-06 | -0.3282 | 0 | 0.673 |
| ENSP00000378920 | 4.68E-06 | -0.3283 | 0 | 0.100 |
| ENSP00000362570 | 3.66E-06 | -0.3283 | 0 | 0.138 |
| ENSP00000361446 | 6.30E-06 | -0.3284 | 383 | 0.798 |
| ENSP00000261862 | 7.54E-06 | -0.3286 | 0 | 0.124 |
| ENSP00000345295 | 2.24E-06 | -0.3286 | 0 | 0.144 |
| ENSP00000362601 | 1.93E-06 | -0.3287 | 0 | 0.079 |
| ENSP00000273037 | 4.00E-06 | -0.3288 | 0 | 0.091 |
| ENSP00000312774 | 4.07E-06 | -0.3288 | 0 | 0.071 |
| ENSP00000296125 | 9.71E-06 | -0.3290 | 0 | 0.117 |
| ENSP00000310241 | 3.98E-06 | -0.3290 | 0 | 0.054 |
| ENSP00000379503 | 2.88E-06 | -0.3290 | 0 | 0.562 |
| ENSP00000284437 | 5.88E-06 | -0.3293 | 0 | 0.129 |
| ENSP00000303549 | 7.29E-06 | -0.3294 | 206 | 0.166 |
| ENSP00000364005 | 2.22E-06 | -0.3294 | 0 | 0.000 |
| ENSP00000341942 | 2.67E-06 | -0.3295 | 0 | 0.152 |
| ENSP00000334869 | 4.63E-06 | -0.3295 | 0 | 0.102 |

| | | | | |
|---|---|---|---|---|
| ENSP00000332511 | 3.80E-06 | -0.3295 | 0 | 0.087 |
| ENSP00000377948 | 5.06E-06 | -0.3295 | 0 | 0.449 |
| ENSP00000359410 | 5.70E-06 | -0.3296 | 0 | 0.097 |
| ENSP00000260257 | 6.08E-06 | -0.3297 | 0 | 0.285 |
| ENSP00000218894 | 8.34E-06 | -0.3297 | 0 | 0.571 |
| ENSP00000342711 | 2.55E-06 | -0.3298 | 0 | 0.129 |
| ENSP00000308535 | 2.27E-06 | -0.3298 | 0 | 0.089 |
| ENSP00000202677 | 4.00E-06 | -0.3298 | 0 | 0.103 |
| ENSP00000354912 | 3.54E-06 | -0.3299 | 0 | 0.090 |
| ENSP00000331734 | 5.13E-06 | -0.3299 | 0 | 0.000 |
| ENSP00000434359 | 2.95E-06 | -0.3301 | 0 | 0.836 |
| ENSP00000282670 | 8.77E-06 | -0.3301 | 0 | 0.107 |
| ENSP00000378451 | 3.07E-06 | -0.3302 | 0 | 0.000 |
| ENSP00000289932 | 7.47E-06 | -0.3302 | 0 | 0.183 |
| ENSP00000359204 | 2.10E-06 | -0.3303 | 0 | 0.000 |
| ENSP00000359688 | 2.99E-06 | -0.3303 | 0 | 0.881 |
| ENSP00000272452 | 6.18E-06 | -0.3303 | 0 | 0.081 |
| ENSP00000417764 | 6.47E-06 | -0.3304 | 150 | 0.372 |
| ENSP00000375907 | 3.46E-06 | -0.3306 | 0 | 0.130 |
| ENSP00000310255 | 4.88E-06 | -0.3307 | 0 | 0.093 |
| ENSP00000408979 | 4.36E-06 | -0.3308 | 0 | 0.364 |
| ENSP00000007735 | 5.32E-06 | -0.3309 | 0 | 0.087 |
| ENSP00000391498 | 2.45E-06 | -0.3309 | 0 | 0.057 |
| ENSP00000383210 | 6.08E-06 | -0.3311 | 0 | 0.000 |
| ENSP00000307713 | 1.28E-05 | -0.3312 | 242 | 0.000 |
| ENSP00000435777 | 5.50E-06 | -0.3312 | 401 | 0.865 |
| ENSP00000297267 | 4.45E-06 | -0.3312 | 0 | 0.225 |
| ENSP00000255087 | 3.45E-06 | -0.3312 | 0 | 0.582 |
| ENSP00000297130 | 5.91E-06 | -0.3312 | 0 | 0.096 |
| ENSP00000262207 | 3.98E-06 | -0.3313 | 0 | 0.081 |
| ENSP00000319250 | 4.00E-06 | -0.3313 | 0 | 0.300 |
| ENSP00000334314 | 3.06E-06 | -0.3313 | 0 | 0.189 |
| ENSP00000309782 | 6.89E-06 | -0.3313 | 0 | 0.156 |
| ENSP00000360967 | 4.84E-06 | -0.3315 | 0 | 0.000 |
| ENSP00000367432 | 2.79E-06 | -0.3315 | 0 | 0.111 |
| ENSP00000356518 | 1.98E-06 | -0.3315 | 0 | 0.151 |
| ENSP00000228887 | 2.50E-06 | -0.3316 | 0 | 0.169 |
| ENSP00000359787 | 3.93E-06 | -0.3316 | 0 | 0.177 |
| ENSP00000394700 | 2.68E-06 | -0.3317 | 0 | 0.074 |
| ENSP00000366036 | 3.98E-06 | -0.3318 | 0 | 0.151 |
| ENSP00000325738 | 4.16E-06 | -0.3319 | 0 | 0.295 |
| ENSP00000393275 | 7.70E-06 | -0.3319 | 0 | 0.305 |
| ENSP00000321343 | 4.91E-06 | -0.3319 | 0 | 0.084 |
| ENSP00000470318 | 5.66E-06 | -0.3322 | 216 | 0.000 |
| ENSP00000289877 | 1.27E-05 | -0.3324 | 0 | 0.095 |
| ENSP00000455047 | 6.31E-06 | -0.3324 | 315 | 0.391 |

| | | | | |
|---|---|---|---|---|
| ENSP00000309818 | 1.10E-05 | -0.3324 | 231 | 0.573 |
| ENSP00000246801 | 7.52E-06 | -0.3324 | 0 | 0.120 |
| ENSP00000362026 | 2.80E-06 | -0.3325 | 0 | 0.699 |
| ENSP00000362094 | 2.51E-06 | -0.3325 | 0 | 0.111 |
| ENSP00000464034 | 4.92E-06 | -0.3327 | 0 | 0.000 |
| ENSP00000409000 | 6.56E-06 | -0.3329 | 208 | 0.451 |
| ENSP00000307449 | 4.73E-06 | -0.3330 | 0 | 0.090 |
| ENSP00000413074 | 3.59E-06 | -0.3331 | 0 | 0.664 |
| ENSP00000274487 | 7.52E-06 | -0.3332 | 0 | 0.171 |
| ENSP00000220244 | 5.94E-06 | -0.3332 | 0 | 0.000 |
| ENSP00000358965 | 2.02E-06 | -0.3332 | 0 | 0.160 |
| ENSP00000299709 | 5.65E-06 | -0.3332 | 0 | 0.052 |
| ENSP00000384610 | 8.85E-06 | -0.3335 | 452 | 0.637 |
| ENSP00000384115 | 2.76E-06 | -0.3337 | 0 | 0.199 |
| ENSP00000335024 | 9.09E-06 | -0.3338 | 0 | 0.311 |
| ENSP00000341539 | 1.69E-06 | -0.3339 | 0 | 0.197 |
| ENSP00000336984 | 4.22E-06 | -0.3339 | 0 | 0.415 |
| ENSP00000333496 | 1.18E-05 | -0.3340 | 292 | 0.505 |
| ENSP00000344219 | 2.83E-06 | -0.3340 | 0 | 0.112 |
| ENSP00000341564 | 3.60E-06 | -0.3341 | 0 | 0.078 |
| ENSP00000341481 | 1.75E-04 | -0.3341 | 0 | 0.000 |
| ENSP00000342790 | 1.75E-04 | -0.3341 | 0 | 0.213 |
| ENSP00000308292 | 3.74E-06 | -0.3342 | 0 | 0.078 |
| ENSP00000464272 | 2.99E-07 | -0.3343 | 0 | 0.000 |
| ENSP00000407749 | 2.99E-07 | -0.3343 | 0 | 0.213 |
| ENSP00000413896 | 2.99E-07 | -0.3343 | 0 | 0.000 |
| ENSP00000333157 | 6.54E-06 | -0.3343 | 0 | 0.371 |
| ENSP00000323075 | 2.77E-06 | -0.3343 | 0 | 0.132 |
| ENSP00000358963 | 1.16E-05 | -0.3344 | 323 | 0.673 |
| ENSP00000431605 | 3.34E-06 | -0.3345 | 0 | 0.342 |
| ENSP00000311833 | 3.83E-06 | -0.3345 | 0 | 0.098 |
| ENSP00000252729 | 3.78E-06 | -0.3345 | 0 | 0.137 |
| ENSP00000273158 | 3.41E-06 | -0.3346 | 0 | 0.073 |
| ENSP00000270238 | 3.30E-06 | -0.3346 | 0 | 0.109 |
| ENSP00000371327 | 2.11E-06 | -0.3346 | 0 | 0.210 |
| ENSP00000323549 | 4.48E-06 | -0.3349 | 0 | 0.091 |
| ENSP00000451998 | 2.79E-06 | -0.3349 | 0 | 0.097 |
| ENSP00000446743 | 3.31E-06 | -0.3350 | 0 | 0.106 |
| ENSP00000273352 | 4.93E-06 | -0.3350 | 0 | 0.000 |
| ENSP00000255226 | 6.20E-06 | -0.3351 | 0 | 0.153 |
| ENSP00000350266 | 5.76E-06 | -0.3351 | 0 | 0.129 |
| ENSP00000341914 | 3.16E-06 | -0.3352 | 0 | 0.072 |
| ENSP00000245105 | 8.34E-06 | -0.3352 | 0 | 0.130 |
| ENSP00000462196 | 1.79E-05 | -0.3353 | 195 | 0.000 |
| ENSP00000323895 | 1.93E-05 | -0.3353 | 0 | 0.205 |
| ENSP00000256246 | 4.01E-06 | -0.3354 | 0 | 0.148 |

| | | | | |
|---|---|---|---|---|
| ENSP00000318258 | 2.53E-06 | -0.3355 | 0 | 0.105 |
| ENSP00000294435 | 7.80E-06 | -0.3356 | 0 | 0.113 |
| ENSP00000328207 | 6.55E-06 | -0.3357 | 0 | 0.610 |
| ENSP00000291934 | 8.40E-06 | -0.3357 | 0 | 0.121 |
| ENSP00000348668 | 7.66E-06 | -0.3358 | 576 | 0.113 |
| ENSP00000346265 | 3.76E-06 | -0.3359 | 0 | 0.201 |
| ENSP00000317000 | 1.94E-06 | -0.3362 | 0 | 0.133 |
| ENSP00000263177 | 4.21E-06 | -0.3362 | 0 | 0.000 |
| ENSP00000303028 | 4.24E-06 | -0.3363 | 0 | 0.112 |
| ENSP00000295049 | 2.70E-06 | -0.3363 | 0 | 0.145 |
| ENSP00000244565 | 7.54E-06 | -0.3363 | 0 | 0.102 |
| ENSP00000373066 | 6.38E-06 | -0.3363 | 0 | 0.000 |
| ENSP00000355155 | 4.76E-06 | -0.3363 | 0 | 0.317 |
| ENSP00000383558 | 1.33E-05 | -0.3364 | 522 | 0.648 |
| ENSP00000300051 | 5.27E-06 | -0.3366 | 0 | 0.114 |
| ENSP00000355260 | 4.13E-06 | -0.3366 | 0 | 0.095 |
| ENSP00000281142 | 2.85E-06 | -0.3368 | 0 | 0.098 |
| ENSP00000339692 | 3.50E-06 | -0.3369 | 0 | 0.223 |
| ENSP00000361087 | 4.33E-06 | -0.3370 | 0 | 0.190 |
| ENSP00000253008 | 4.04E-06 | -0.3370 | 0 | 0.428 |
| ENSP00000221573 | 4.73E-06 | -0.3370 | 0 | 0.493 |
| ENSP00000406022 | 4.25E-06 | -0.3371 | 0 | 0.531 |
| ENSP00000296785 | 6.72E-06 | -0.3372 | 201 | 0.606 |
| ENSP00000405165 | 3.48E-06 | -0.3373 | 0 | 0.103 |
| ENSP00000357535 | 2.49E-04 | -0.3373 | 801 | 0.366 |
| ENSP00000318415 | 3.27E-06 | -0.3375 | 0 | 0.749 |
| ENSP00000367743 | 4.40E-06 | -0.3375 | 0 | 0.082 |
| ENSP00000229332 | 9.98E-06 | -0.3375 | 0 | 0.291 |
| ENSP00000241274 | 3.03E-06 | -0.3376 | 0 | 0.114 |
| ENSP00000322530 | 5.05E-06 | -0.3376 | 0 | 0.000 |
| ENSP00000333097 | 7.71E-06 | -0.3377 | 282 | 0.688 |
| ENSP00000264428 | 4.19E-06 | -0.3377 | 0 | 0.108 |
| ENSP00000378160 | 3.97E-06 | -0.3380 | 222 | 0.000 |
| ENSP00000298231 | 5.06E-06 | -0.3380 | 0 | 0.782 |
| ENSP00000336693 | 2.58E-06 | -0.3380 | 0 | 0.062 |
| ENSP00000356076 | 6.16E-06 | -0.3381 | 189 | 0.675 |
| ENSP00000350676 | 2.62E-06 | -0.3381 | 0 | 0.000 |
| ENSP00000320794 | 4.97E-06 | -0.3381 | 0 | 0.000 |
| ENSP00000330606 | 5.22E-06 | -0.3383 | 0 | 0.048 |
| ENSP00000300515 | 3.29E-06 | -0.3383 | 0 | 0.000 |
| ENSP00000450353 | 5.69E-06 | -0.3383 | 216 | 0.000 |
| ENSP00000161006 | 8.43E-06 | -0.3386 | 0 | 0.133 |
| ENSP00000414019 | 4.87E-06 | -0.3387 | 215 | 0.109 |
| ENSP00000296484 | 9.13E-06 | -0.3390 | 226 | 0.156 |
| ENSP00000253680 | 2.64E-06 | -0.3390 | 0 | 0.000 |
| ENSP00000369146 | 3.66E-06 | -0.3390 | 0 | 0.083 |

| | | | | |
|---|---|---|---|---|
| ENSP00000337732 | 5.35E-06 | -0.3393 | 0 | 0.105 |
| ENSP00000353165 | 3.68E-06 | -0.3395 | 0 | 0.339 |
| ENSP00000276127 | 3.15E-06 | -0.3397 | 0 | 0.118 |
| ENSP00000369558 | 3.96E-06 | -0.3398 | 0 | 0.375 |
| ENSP00000383698 | 4.86E-06 | -0.3400 | 0 | 0.160 |
| ENSP00000266980 | 3.27E-06 | -0.3405 | 0 | 0.094 |
| ENSP00000369009 | 2.28E-06 | -0.3406 | 0 | 0.131 |
| ENSP00000338607 | 5.55E-06 | -0.3406 | 0 | 0.103 |
| ENSP00000259053 | 5.26E-06 | -0.3408 | 0 | 0.117 |
| ENSP00000444293 | 4.82E-06 | -0.3408 | 0 | 0.000 |
| ENSP00000227618 | 2.06E-06 | -0.3410 | 0 | 0.150 |
| ENSP00000316670 | 5.11E-06 | -0.3411 | 0 | 0.000 |
| ENSP00000340943 | 1.04E-05 | -0.3411 | 212 | 0.656 |
| ENSP00000354213 | 5.84E-06 | -0.3411 | 0 | 0.061 |
| ENSP00000362123 | 4.09E-06 | -0.3411 | 0 | 0.202 |
| ENSP00000435619 | 2.31E-06 | -0.3411 | 0 | 0.404 |
| ENSP00000262103 | 2.56E-06 | -0.3413 | 0 | 0.285 |
| ENSP00000230568 | 9.90E-06 | -0.3413 | 0 | 0.153 |
| ENSP00000374353 | 2.38E-06 | -0.3413 | 0 | 0.000 |
| ENSP00000349874 | 2.45E-06 | -0.3413 | 0 | 0.146 |
| ENSP00000363643 | 8.98E-06 | -0.3414 | 271 | 0.522 |
| ENSP00000281924 | 4.58E-06 | -0.3414 | 0 | 0.069 |
| ENSP00000329482 | 4.34E-06 | -0.3415 | 0 | 0.000 |
| ENSP00000341637 | 4.10E-06 | -0.3416 | 0 | 0.158 |
| ENSP00000262376 | 3.74E-06 | -0.3416 | 0 | 0.258 |
| ENSP00000462799 | 1.45E-06 | -0.3417 | 0 | 0.205 |
| ENSP00000404078 | 2.41E-06 | -0.3418 | 0 | 0.000 |
| ENSP00000382646 | 1.79E-06 | -0.3420 | 0 | 0.108 |
| ENSP00000321929 | 4.36E-06 | -0.3424 | 0 | 0.052 |
| ENSP00000396896 | 6.10E-06 | -0.3425 | 776 | 0.192 |
| ENSP00000376623 | 3.31E-06 | -0.3426 | 0 | 0.288 |
| ENSP00000156084 | 4.76E-06 | -0.3427 | 0 | 0.177 |
| ENSP00000380605 | 4.15E-06 | -0.3428 | 0 | 0.135 |
| ENSP00000309036 | 3.10E-06 | -0.3428 | 0 | 0.080 |
| ENSP00000313816 | 1.79E-06 | -0.3428 | 0 | 0.000 |
| ENSP00000373272 | 3.07E-06 | -0.3429 | 0 | 0.204 |
| ENSP00000357840 | 4.90E-06 | -0.3429 | 0 | 0.093 |
| ENSP00000257940 | 5.76E-06 | -0.3430 | 0 | 0.334 |
| ENSP00000295886 | 8.20E-06 | -0.3431 | 163 | 0.835 |
| ENSP00000301336 | 5.24E-06 | -0.3432 | 0 | 0.223 |
| ENSP00000296595 | 4.08E-06 | -0.3435 | 0 | 0.095 |
| ENSP00000310405 | 1.15E-05 | -0.3437 | 0 | 0.544 |
| ENSP00000466248 | 6.00E-06 | -0.3438 | 191 | 0.387 |
| ENSP00000312411 | 4.54E-06 | -0.3438 | 0 | 0.594 |
| ENSP00000274361 | 5.32E-06 | -0.3438 | 0 | 0.068 |
| ENSP00000363298 | 1.85E-06 | -0.3439 | 0 | 0.133 |

| | | | | |
|---|---|---|---|---|
| ENSP00000353557 | 3.26E-06 | -0.3439 | 0 | 0.075 |
| ENSP00000306461 | 7.16E-06 | -0.3439 | 0 | 0.328 |
| ENSP00000317404 | 7.77E-06 | -0.3440 | 0 | 0.067 |
| ENSP00000376957 | 3.75E-06 | -0.3440 | 0 | 0.152 |
| ENSP00000385727 | 2.09E-06 | -0.3441 | 0 | 0.138 |
| ENSP00000412897 | 4.20E-06 | -0.3441 | 0 | 0.554 |
| ENSP00000243501 | 7.49E-06 | -0.3441 | 0 | 0.156 |
| ENSP00000368591 | 4.80E-06 | -0.3442 | 0 | 0.060 |
| ENSP00000302413 | 4.82E-06 | -0.3442 | 0 | 0.111 |
| ENSP00000273936 | 5.15E-06 | -0.3442 | 0 | 0.102 |
| ENSP00000401513 | 3.47E-06 | -0.3443 | 0 | 0.121 |
| ENSP00000404063 | 2.90E-06 | -0.3443 | 0 | 0.263 |
| ENSP00000354830 | 2.97E-06 | -0.3444 | 0 | 0.236 |
| ENSP00000344937 | 3.94E-06 | -0.3444 | 0 | 0.090 |
| ENSP00000303468 | 2.92E-06 | -0.3447 | 0 | 0.089 |
| ENSP00000355893 | 2.70E-06 | -0.3448 | 0 | 0.659 |
| ENSP00000370979 | 4.30E-06 | -0.3448 | 0 | 0.662 |
| ENSP00000455952 | 7.99E-06 | -0.3449 | 216 | 0.000 |
| ENSP00000381425 | 8.53E-06 | -0.3450 | 302 | 0.483 |
| ENSP00000419101 | 3.70E-06 | -0.3450 | 0 | 0.281 |
| ENSP00000343428 | 7.86E-06 | -0.3450 | 0 | 0.432 |
| ENSP00000363452 | 3.31E-06 | -0.3452 | 0 | 0.075 |
| ENSP00000344961 | 2.29E-06 | -0.3452 | 0 | 0.107 |
| ENSP00000328672 | 3.09E-06 | -0.3453 | 0 | 0.130 |
| ENSP00000352414 | 6.81E-06 | -0.3454 | 0 | 0.912 |
| ENSP00000280758 | 5.33E-06 | -0.3456 | 0 | 0.276 |
| ENSP00000309493 | 1.09E-05 | -0.3457 | 252 | 0.475 |
| ENSP00000338967 | 4.52E-06 | -0.3457 | 0 | 0.354 |
| ENSP00000304147 | 3.07E-06 | -0.3459 | 0 | 0.137 |
| ENSP00000410603 | 2.91E-06 | -0.3460 | 0 | 0.109 |
| ENSP00000304707 | 5.65E-06 | -0.3460 | 0 | 0.181 |
| ENSP00000323816 | 3.98E-06 | -0.3462 | 0 | 0.623 |
| ENSP00000350162 | 5.80E-06 | -0.3462 | 0 | 0.291 |
| ENSP00000362894 | 3.47E-06 | -0.3463 | 0 | 0.088 |
| ENSP00000183605 | 7.78E-06 | -0.3463 | 0 | 0.098 |
| ENSP00000367173 | 2.00E-06 | -0.3464 | 0 | 0.095 |
| ENSP00000443246 | 6.49E-06 | -0.3464 | 262 | 0.000 |
| ENSP00000298854 | 6.53E-06 | -0.3464 | 0 | 0.351 |
| ENSP00000255152 | 4.70E-06 | -0.3465 | 0 | 0.171 |
| ENSP00000321627 | 5.31E-06 | -0.3465 | 0 | 0.152 |
| ENSP00000441093 | 4.42E-06 | -0.3465 | 535 | 0.000 |
| ENSP00000288025 | 6.45E-06 | -0.3466 | 0 | 0.128 |
| ENSP00000361214 | 3.02E-06 | -0.3468 | 0 | 0.422 |
| ENSP00000390011 | 5.97E-06 | -0.3469 | 201 | 0.450 |
| ENSP00000359563 | 5.34E-06 | -0.3470 | 0 | 0.127 |
| ENSP00000396103 | 4.92E-06 | -0.3471 | 0 | 0.544 |

| | | | | |
|---|---|---|---|---|
| ENSP00000215906 | 4.81E-06 | -0.3471 | 0 | 0.144 |
| ENSP00000359096 | 8.60E-06 | -0.3471 | 0 | 0.382 |
| ENSP00000364992 | 7.12E-06 | -0.3473 | 0 | 0.209 |
| ENSP00000333395 | 4.77E-06 | -0.3473 | 0 | 0.072 |
| ENSP00000366629 | 2.85E-06 | -0.3473 | 0 | 0.000 |
| ENSP00000421076 | 1.48E-06 | -0.3474 | 0 | 0.172 |
| ENSP00000309741 | 4.35E-06 | -0.3477 | 0 | 0.051 |
| ENSP00000319636 | 8.24E-06 | -0.3477 | 900 | 0.197 |
| ENSP00000343234 | 4.92E-06 | -0.3478 | 0 | 0.062 |
| ENSP00000326003 | 3.88E-06 | -0.3478 | 0 | 0.287 |
| ENSP00000261234 | 2.88E-06 | -0.3482 | 0 | 0.072 |
| ENSP00000301972 | 3.51E-06 | -0.3484 | 0 | 0.130 |
| ENSP00000264956 | 9.91E-06 | -0.3486 | 0 | 0.345 |
| ENSP00000387694 | 5.41E-06 | -0.3487 | 0 | 0.224 |
| ENSP00000306918 | 4.15E-06 | -0.3487 | 0 | 0.121 |
| ENSP00000472299 | 2.02E-06 | -0.3487 | 0 | 0.000 |
| ENSP00000352544 | 3.62E-06 | -0.3488 | 0 | 0.194 |
| ENSP00000313318 | 3.50E-06 | -0.3488 | 0 | 0.047 |
| ENSP00000357453 | 5.50E-06 | -0.3489 | 237 | 0.190 |
| ENSP00000363894 | 3.28E-06 | -0.3491 | 0 | 0.108 |
| ENSP00000276480 | 4.61E-06 | -0.3492 | 0 | 0.097 |
| ENSP00000344976 | 6.07E-06 | -0.3492 | 0 | 0.098 |
| ENSP00000355692 | 1.86E-06 | -0.3495 | 0 | 0.218 |
| ENSP00000259555 | 6.16E-06 | -0.3495 | 0 | 0.000 |
| ENSP00000379353 | 7.53E-06 | -0.3496 | 313 | 0.064 |
| ENSP00000417710 | 1.50E-06 | -0.3497 | 0 | 0.243 |
| ENSP00000342935 | 4.29E-06 | -0.3497 | 0 | 0.084 |
| ENSP00000274134 | 3.07E-06 | -0.3498 | 0 | 0.066 |
| ENSP00000368320 | 2.05E-06 | -0.3499 | 0 | 0.243 |
| ENSP00000296545 | 7.58E-06 | -0.3500 | 0 | 0.749 |
| ENSP00000248933 | 3.07E-06 | -0.3501 | 0 | 0.106 |
| ENSP00000255476 | 6.70E-06 | -0.3501 | 0 | 0.550 |
| ENSP00000260723 | 6.12E-06 | -0.3504 | 0 | 0.114 |
| ENSP00000322218 | 5.52E-06 | -0.3506 | 206 | 0.000 |
| ENSP00000255681 | 3.87E-06 | -0.3506 | 0 | 0.171 |
| ENSP00000290894 | 8.22E-06 | -0.3506 | 0 | 0.078 |
| ENSP00000345152 | 4.09E-06 | -0.3509 | 0 | 0.103 |
| ENSP00000288167 | 3.68E-06 | -0.3509 | 0 | 0.190 |
| ENSP00000417190 | 2.68E-05 | -0.3510 | 0 | 0.160 |
| ENSP00000360167 | 2.05E-06 | -0.3510 | 0 | 0.146 |
| ENSP00000290216 | 5.62E-06 | -0.3510 | 0 | 0.160 |
| ENSP00000271643 | 8.09E-06 | -0.3511 | 0 | 0.167 |
| ENSP00000379999 | 6.79E-06 | -0.3511 | 188 | 0.194 |
| ENSP00000268699 | 3.91E-06 | -0.3511 | 0 | 0.061 |
| ENSP00000219022 | 8.04E-06 | -0.3514 | 0 | 0.150 |
| ENSP00000373169 | 1.01E-05 | -0.3514 | 323 | 0.577 |

| | | | | |
|---|---|---|---|---|
| ENSP00000333952 | 3.97E-06 | -0.3515 | 0 | 0.115 |
| ENSP00000296318 | 6.31E-06 | -0.3516 | 0 | 0.258 |
| ENSP00000359668 | 4.63E-06 | -0.3519 | 0 | 0.236 |
| ENSP00000369666 | 3.47E-06 | -0.3520 | 0 | 0.000 |
| ENSP00000259951 | 8.51E-06 | -0.3522 | 0 | 0.171 |
| ENSP00000260191 | 7.09E-06 | -0.3522 | 0 | 0.317 |
| ENSP00000354877 | 2.86E-06 | -0.3524 | 0 | 0.316 |
| ENSP00000269385 | 5.26E-06 | -0.3524 | 0 | 0.686 |
| ENSP00000341698 | 3.47E-06 | -0.3525 | 0 | 0.667 |
| ENSP00000223398 | 6.10E-06 | -0.3525 | 0 | 0.094 |
| ENSP00000386272 | 1.76E-06 | -0.3526 | 0 | 0.046 |
| ENSP00000281317 | 5.34E-06 | -0.3526 | 0 | 0.113 |
| ENSP00000333919 | 5.55E-06 | -0.3527 | 0 | 0.481 |
| ENSP00000364550 | 3.61E-06 | -0.3527 | 0 | 0.000 |
| ENSP00000234816 | 1.02E-05 | -0.3530 | 0 | 0.166 |
| ENSP00000303246 | 3.72E-06 | -0.3532 | 0 | 0.133 |
| ENSP00000357123 | 5.93E-06 | -0.3533 | 0 | 0.509 |
| ENSP00000300057 | 5.78E-06 | -0.3535 | 0 | 0.759 |
| ENSP00000328133 | 3.43E-06 | -0.3538 | 0 | 0.371 |
| ENSP00000314196 | 4.30E-06 | -0.3541 | 0 | 0.198 |
| ENSP00000329548 | 7.66E-06 | -0.3541 | 189 | 0.477 |
| ENSP00000305974 | 7.13E-06 | -0.3541 | 206 | 0.195 |
| ENSP00000390784 | 4.19E-06 | -0.3541 | 0 | 0.125 |
| ENSP00000304956 | 5.13E-06 | -0.3541 | 0 | 0.713 |
| ENSP00000278559 | 8.74E-06 | -0.3542 | 191 | 0.000 |
| ENSP00000411932 | 4.59E-06 | -0.3544 | 0 | 0.200 |
| ENSP00000253247 | 4.78E-06 | -0.3544 | 0 | 0.417 |
| ENSP00000234800 | 3.58E-06 | -0.3547 | 0 | 0.000 |
| ENSP00000299564 | 3.71E-06 | -0.3547 | 163 | 0.000 |
| ENSP00000353072 | 5.73E-06 | -0.3547 | 309 | 0.624 |
| ENSP00000260113 | 9.51E-06 | -0.3548 | 0 | 0.074 |
| ENSP00000296218 | 4.40E-06 | -0.3548 | 0 | 0.045 |
| ENSP00000316794 | 2.15E-06 | -0.3549 | 0 | 0.154 |
| ENSP00000265136 | 6.91E-06 | -0.3549 | 0 | 0.094 |
| ENSP00000288861 | 2.29E-06 | -0.3554 | 0 | 0.000 |
| ENSP00000284288 | 5.91E-06 | -0.3555 | 0 | 0.090 |
| ENSP00000335358 | 5.44E-06 | -0.3555 | 0 | 0.085 |
| ENSP00000356033 | 3.03E-06 | -0.3555 | 0 | 0.223 |
| ENSP00000266077 | 9.05E-06 | -0.3556 | 0 | 0.518 |
| ENSP00000321498 | 5.33E-06 | -0.3557 | 0 | 0.105 |
| ENSP00000295898 | 4.47E-06 | -0.3558 | 0 | 0.210 |
| ENSP00000297186 | 5.11E-06 | -0.3558 | 0 | 0.093 |
| ENSP00000395323 | 1.06E-05 | -0.3559 | 177 | 0.381 |
| ENSP00000264444 | 4.95E-06 | -0.3559 | 0 | 0.522 |
| ENSP00000241312 | 3.33E-06 | -0.3561 | 0 | 0.066 |
| ENSP00000302276 | 4.20E-06 | -0.3562 | 0 | 0.462 |

| | | | | |
|---|---|---|---|---|
| ENSP00000366927 | 6.34E-06 | -0.3563 | 274 | 0.358 |
| ENSP00000221538 | 4.55E-06 | -0.3564 | 0 | 0.085 |
| ENSP00000298032 | 4.78E-06 | -0.3564 | 0 | 0.079 |
| ENSP00000261226 | 4.51E-06 | -0.3565 | 0 | 0.060 |
| ENSP00000301457 | 2.74E-06 | -0.3565 | 0 | 0.000 |
| ENSP00000292079 | 7.09E-06 | -0.3568 | 0 | 0.094 |
| ENSP00000450040 | 8.87E-06 | -0.3569 | 237 | 0.693 |
| ENSP00000331167 | 3.70E-06 | -0.3570 | 0 | 0.000 |
| ENSP00000262961 | 4.37E-06 | -0.3571 | 0 | 0.352 |
| ENSP00000371434 | 3.99E-06 | -0.3571 | 0 | 0.698 |
| ENSP00000361173 | 2.47E-06 | -0.3571 | 0 | 0.000 |
| ENSP00000301293 | 4.31E-06 | -0.3572 | 0 | 0.000 |
| ENSP00000299045 | 3.67E-06 | -0.3573 | 0 | 0.063 |
| ENSP00000020926 | 8.25E-06 | -0.3573 | 0 | 0.149 |
| ENSP00000289619 | 4.51E-06 | -0.3574 | 0 | 0.105 |
| ENSP00000255082 | 9.71E-06 | -0.3574 | 0 | 0.121 |
| ENSP00000325562 | 2.71E-06 | -0.3576 | 0 | 0.075 |
| ENSP00000231749 | 5.75E-06 | -0.3576 | 0 | 0.071 |
| ENSP00000266254 | 2.28E-06 | -0.3577 | 0 | 0.051 |
| ENSP00000295890 | 8.62E-06 | -0.3577 | 340 | 0.277 |
| ENSP00000305975 | 2.62E-06 | -0.3578 | 0 | 0.164 |
| ENSP00000380066 | 7.11E-06 | -0.3578 | 246 | 0.642 |
| ENSP00000311447 | 4.64E-06 | -0.3579 | 0 | 0.097 |
| ENSP00000385143 | 5.00E-06 | -0.3580 | 0 | 0.396 |
| ENSP00000330484 | 8.84E-06 | -0.3580 | 0 | 0.383 |
| ENSP00000313172 | 4.07E-06 | -0.3581 | 0 | 0.088 |
| ENSP00000367476 | 2.49E-06 | -0.3582 | 0 | 0.075 |
| ENSP00000356404 | 3.10E-06 | -0.3582 | 0 | 0.072 |
| ENSP00000353129 | 3.99E-06 | -0.3583 | 0 | 0.123 |
| ENSP00000217254 | 8.05E-06 | -0.3585 | 0 | 0.174 |
| ENSP00000296921 | 5.41E-06 | -0.3586 | 0 | 0.786 |
| ENSP00000471569 | 3.70E-06 | -0.3587 | 0 | 0.000 |
| ENSP00000332208 | 4.17E-06 | -0.3588 | 0 | 0.671 |
| ENSP00000300105 | 3.22E-06 | -0.3591 | 0 | 0.392 |
| ENSP00000216075 | 8.88E-06 | -0.3593 | 0 | 0.098 |
| ENSP00000084798 | 7.47E-06 | -0.3595 | 0 | 0.136 |
| ENSP00000261038 | 8.41E-06 | -0.3596 | 0 | 0.424 |
| ENSP00000221730 | 6.58E-06 | -0.3596 | 0 | 0.130 |
| ENSP00000358547 | 5.86E-06 | -0.3597 | 302 | 0.529 |
| ENSP00000358559 | 2.45E-06 | -0.3597 | 0 | 0.000 |
| ENSP00000244314 | 9.40E-06 | -0.3599 | 0 | 0.130 |
| ENSP00000293851 | 8.19E-06 | -0.3600 | 0 | 0.137 |
| ENSP00000286749 | 4.64E-06 | -0.3601 | 0 | 0.058 |
| ENSP00000252998 | 5.95E-06 | -0.3602 | 0 | 0.135 |
| ENSP00000164640 | 9.37E-06 | -0.3603 | 0 | 0.096 |
| ENSP00000222125 | 8.03E-06 | -0.3604 | 158 | 0.649 |

| | | | | |
|---|---|---|---|---|
| ENSP00000384391 | 4.15E-06 | -0.3604 | 0 | 0.072 |
| ENSP00000234392 | 1.07E-05 | -0.3607 | 0 | 0.768 |
| ENSP00000280191 | 3.47E-06 | -0.3610 | 0 | 0.117 |
| ENSP00000461324 | 4.09E-06 | -0.3611 | 0 | 0.156 |
| ENSP00000156109 | 4.22E-06 | -0.3611 | 0 | 0.326 |
| ENSP00000321874 | 7.74E-06 | -0.3611 | 900 | 0.130 |
| ENSP00000274867 | 8.87E-06 | -0.3612 | 184 | 0.664 |
| ENSP00000379065 | 6.13E-06 | -0.3612 | 0 | 0.000 |
| ENSP00000267383 | 4.87E-06 | -0.3613 | 0 | 0.085 |
| ENSP00000337144 | 3.93E-06 | -0.3616 | 0 | 0.216 |
| ENSP00000296088 | 3.55E-06 | -0.3616 | 0 | 0.109 |
| ENSP00000243878 | 2.86E-06 | -0.3616 | 0 | 0.079 |
| ENSP00000259782 | 7.42E-06 | -0.3616 | 0 | 0.216 |
| ENSP00000215886 | 1.17E-05 | -0.3617 | 0 | 0.116 |
| ENSP00000248564 | 1.01E-05 | -0.3617 | 0 | 0.431 |
| ENSP00000256452 | 8.76E-06 | -0.3617 | 0 | 0.628 |
| ENSP00000354660 | 3.12E-06 | -0.3617 | 0 | 0.326 |
| ENSP00000417581 | 8.88E-08 | -0.3618 | 0 | 0.212 |
| ENSP00000283243 | 7.70E-06 | -0.3618 | 0 | 0.113 |
| ENSP00000359329 | 7.09E-06 | -0.3619 | 159 | 0.000 |
| ENSP00000357778 | 2.43E-06 | -0.3620 | 0 | 0.135 |
| ENSP00000366970 | 2.39E-06 | -0.3621 | 0 | 0.146 |
| ENSP00000330240 | 6.46E-06 | -0.3622 | 0 | 0.087 |
| ENSP00000269298 | 4.36E-06 | -0.3622 | 0 | 0.129 |
| ENSP00000352820 | 2.36E-06 | -0.3623 | 0 | 0.149 |
| ENSP00000331766 | 2.94E-06 | -0.3624 | 0 | 0.133 |
| ENSP00000433967 | 2.67E-06 | -0.3624 | 0 | 0.000 |
| ENSP00000415904 | 4.63E-06 | -0.3624 | 0 | 0.173 |
| ENSP00000315988 | 5.53E-06 | -0.3625 | 0 | 0.054 |
| ENSP00000466834 | 3.79E-06 | -0.3626 | 0 | 0.000 |
| ENSP00000348911 | 4.81E-06 | -0.3627 | 0 | 0.074 |
| ENSP00000348648 | 4.87E-06 | -0.3630 | 0 | 0.117 |
| ENSP00000280979 | 4.81E-06 | -0.3630 | 0 | 0.468 |
| ENSP00000370381 | 7.17E-06 | -0.3631 | 0 | 0.268 |
| ENSP00000264601 | 3.14E-06 | -0.3633 | 0 | 0.225 |
| ENSP00000348074 | 5.02E-06 | -0.3633 | 0 | 0.354 |
| ENSP00000042931 | 6.21E-06 | -0.3634 | 0 | 0.128 |
| ENSP00000301671 | 7.49E-06 | -0.3636 | 0 | 0.596 |
| ENSP00000259339 | 7.41E-06 | -0.3640 | 0 | 0.239 |
| ENSP00000275764 | 7.58E-06 | -0.3640 | 0 | 0.419 |
| ENSP00000239347 | 7.79E-06 | -0.3641 | 0 | 0.339 |
| ENSP00000347916 | 5.16E-06 | -0.3641 | 0 | 0.194 |
| ENSP00000318270 | 6.31E-06 | -0.3644 | 0 | 0.083 |
| ENSP00000417763 | 3.61E-06 | -0.3644 | 0 | 0.373 |
| ENSP00000345294 | 3.31E-06 | -0.3645 | 0 | 0.000 |
| ENSP00000369553 | 3.97E-06 | -0.3646 | 0 | 0.323 |

| | | | | |
|---|---|---|---|---|
| ENSP00000333639 | 5.79E-06 | -0.3647 | 0 | 0.448 |
| ENSP00000258613 | 4.79E-06 | -0.3647 | 0 | 0.111 |
| ENSP00000218224 | 7.71E-06 | -0.3648 | 0 | 0.390 |
| ENSP00000256324 | 3.50E-06 | -0.3648 | 0 | 0.090 |
| ENSP00000281961 | 2.37E-06 | -0.3648 | 0 | 0.170 |
| ENSP00000357342 | 4.24E-06 | -0.3650 | 0 | 0.209 |
| ENSP00000343957 | 5.47E-06 | -0.3651 | 0 | 0.670 |
| ENSP00000328079 | 2.73E-06 | -0.3652 | 0 | 0.120 |
| ENSP00000356658 | 7.33E-06 | -0.3654 | 601 | 0.255 |
| ENSP00000294794 | 5.55E-06 | -0.3655 | 0 | 0.122 |
| ENSP00000293780 | 5.38E-06 | -0.3656 | 0 | 0.113 |
| ENSP00000320813 | 2.73E-06 | -0.3656 | 0 | 0.105 |
| ENSP00000314103 | 4.55E-06 | -0.3656 | 0 | 0.000 |
| ENSP00000264363 | 5.06E-06 | -0.3657 | 0 | 0.257 |
| ENSP00000230256 | 6.92E-06 | -0.3661 | 0 | 0.156 |
| ENSP00000299663 | 8.24E-06 | -0.3661 | 0 | 0.396 |
| ENSP00000247829 | 5.62E-06 | -0.3661 | 0 | 0.565 |
| ENSP00000367629 | 9.81E-06 | -0.3662 | 179 | 0.576 |
| ENSP00000006777 | 5.19E-06 | -0.3667 | 0 | 0.112 |
| ENSP00000362613 | 5.81E-06 | -0.3669 | 0 | 0.856 |
| ENSP00000357057 | 4.69E-06 | -0.3670 | 0 | 0.199 |
| ENSP00000329214 | 5.04E-06 | -0.3676 | 0 | 0.068 |
| ENSP00000296862 | 5.55E-06 | -0.3676 | 0 | 0.000 |
| ENSP00000386029 | 8.36E-06 | -0.3676 | 200 | 0.246 |
| ENSP00000362183 | 1.00E-05 | -0.3678 | 202 | 0.269 |
| ENSP00000379176 | 2.24E-06 | -0.3679 | 0 | 0.271 |
| ENSP00000353078 | 2.98E-06 | -0.3679 | 0 | 0.159 |
| ENSP00000288065 | 2.92E-06 | -0.3681 | 0 | 0.077 |
| ENSP00000283309 | 6.73E-06 | -0.3684 | 0 | 0.252 |
| ENSP00000303092 | 2.74E-06 | -0.3688 | 0 | 0.055 |
| ENSP00000264833 | 5.36E-06 | -0.3689 | 0 | 0.066 |
| ENSP00000255224 | 6.29E-06 | -0.3689 | 0 | 0.184 |
| ENSP00000006658 | 4.89E-06 | -0.3693 | 0 | 0.092 |
| ENSP00000274569 | 3.36E-06 | -0.3693 | 0 | 0.084 |
| ENSP00000309504 | 4.68E-06 | -0.3694 | 0 | 0.087 |
| ENSP00000226798 | 4.89E-06 | -0.3694 | 0 | 0.320 |
| ENSP00000450742 | 2.87E-06 | -0.3695 | 0 | 0.660 |
| ENSP00000332879 | 4.65E-06 | -0.3695 | 0 | 0.121 |
| ENSP00000385019 | 3.04E-06 | -0.3696 | 0 | 0.190 |
| ENSP00000314223 | 4.83E-06 | -0.3696 | 0 | 0.310 |
| ENSP00000348828 | 4.00E-06 | -0.3696 | 0 | 0.578 |
| ENSP00000292907 | 4.15E-06 | -0.3697 | 0 | 0.088 |
| ENSP00000245957 | 3.19E-06 | -0.3697 | 0 | 0.000 |
| ENSP00000406052 | 3.10E-06 | -0.3697 | 0 | 0.477 |
| ENSP00000420040 | 6.93E-06 | -0.3697 | 375 | 0.373 |
| ENSP00000316237 | 3.86E-06 | -0.3700 | 0 | 0.127 |

| | | | | |
|---|---|---|---|---|
| ENSP00000308750 | 4.63E-06 | -0.3700 | 0 | 0.746 |
| ENSP00000258526 | 6.73E-06 | -0.3700 | 0 | 0.385 |
| ENSP00000345477 | 8.77E-06 | -0.3700 | 900 | 0.151 |
| ENSP00000383145 | 5.43E-06 | -0.3701 | 0 | 0.049 |
| ENSP00000260637 | 4.87E-06 | -0.3702 | 0 | 0.000 |
| ENSP00000187762 | 5.16E-06 | -0.3704 | 0 | 0.079 |
| ENSP00000351118 | 6.15E-06 | -0.3707 | 0 | 0.085 |
| ENSP00000332448 | 2.52E-06 | -0.3707 | 0 | 0.389 |
| ENSP00000228567 | 5.14E-06 | -0.3710 | 0 | 0.117 |
| ENSP00000349238 | 8.21E-06 | -0.3713 | 162 | 0.618 |
| ENSP00000372316 | 2.47E-06 | -0.3713 | 0 | 0.500 |
| ENSP00000297325 | 3.90E-06 | -0.3715 | 0 | 0.063 |
| ENSP00000305973 | 8.04E-06 | -0.3715 | 0 | 0.797 |
| ENSP00000468280 | 7.98E-06 | -0.3715 | 277 | 0.000 |
| ENSP00000353500 | 7.38E-06 | -0.3716 | 0 | 0.585 |
| ENSP00000352268 | 4.28E-06 | -0.3717 | 0 | 0.085 |
| ENSP00000416453 | 1.73E-06 | -0.3718 | 0 | 0.000 |
| ENSP00000308318 | 5.05E-06 | -0.3719 | 0 | 0.142 |
| ENSP00000363003 | 3.34E-06 | -0.3720 | 0 | 0.784 |
| ENSP00000317214 | 6.81E-06 | -0.3720 | 224 | 0.000 |
| ENSP00000403932 | 4.68E-06 | -0.3722 | 153 | 0.000 |
| ENSP00000362799 | 2.29E-06 | -0.3723 | 0 | 0.161 |
| ENSP00000318684 | 3.37E-06 | -0.3724 | 0 | 0.135 |
| ENSP00000245121 | 3.19E-06 | -0.3725 | 0 | 0.179 |
| ENSP00000315465 | 6.48E-06 | -0.3726 | 195 | 0.136 |
| ENSP00000217901 | 8.99E-06 | -0.3726 | 175 | 0.478 |
| ENSP00000302851 | 3.93E-06 | -0.3728 | 0 | 0.072 |
| ENSP00000316193 | 6.41E-06 | -0.3733 | 0 | 0.000 |
| ENSP00000362136 | 2.15E-06 | -0.3735 | 0 | 0.120 |
| ENSP00000282753 | 1.01E-05 | -0.3736 | 571 | 0.717 |
| ENSP00000234142 | 7.15E-06 | -0.3738 | 0 | 0.248 |
| ENSP00000354581 | 6.88E-06 | -0.3740 | 158 | 0.204 |
| ENSP00000299290 | 7.63E-06 | -0.3740 | 0 | 0.104 |
| ENSP00000317836 | 4.31E-06 | -0.3743 | 0 | 0.069 |
| ENSP00000262305 | 5.33E-06 | -0.3743 | 0 | 0.105 |
| ENSP00000261374 | 6.70E-06 | -0.3749 | 0 | 0.141 |
| ENSP00000453793 | 7.51E-06 | -0.3750 | 177 | 0.800 |
| ENSP00000274382 | 7.19E-06 | -0.3751 | 0 | 0.127 |
| ENSP00000344432 | 6.89E-06 | -0.3751 | 0 | 0.123 |
| ENSP00000352064 | 5.48E-06 | -0.3751 | 0 | 0.467 |
| ENSP00000284856 | 4.89E-06 | -0.3754 | 0 | 0.151 |
| ENSP00000336733 | 3.99E-06 | -0.3754 | 0 | 0.072 |
| ENSP00000407952 | 3.40E-06 | -0.3757 | 0 | 0.000 |
| ENSP00000351695 | 5.59E-06 | -0.3758 | 0 | 0.192 |
| ENSP00000264228 | 6.67E-06 | -0.3758 | 0 | 0.141 |
| ENSP00000253109 | 1.33E-05 | -0.3761 | 0 | 0.406 |

| | | | | |
|---|---|---|---|---|
| ENSP00000343877 | 4.22E-06 | -0.3761 | 0 | 0.082 |
| ENSP00000295367 | 6.87E-06 | -0.3767 | 0 | 0.105 |
| ENSP00000275189 | 3.94E-06 | -0.3773 | 0 | 0.000 |
| ENSP00000411904 | 4.36E-06 | -0.3774 | 0 | 0.115 |
| ENSP00000274787 | 4.21E-06 | -0.3775 | 0 | 0.112 |
| ENSP00000349208 | 3.90E-06 | -0.3775 | 0 | 0.490 |
| ENSP00000265340 | 8.60E-06 | -0.3778 | 214 | 0.000 |
| ENSP00000322460 | 3.18E-06 | -0.3779 | 0 | 0.136 |
| ENSP00000215838 | 6.60E-06 | -0.3779 | 0 | 0.121 |
| ENSP00000365152 | 4.01E-06 | -0.3780 | 0 | 0.661 |
| ENSP00000290431 | 7.03E-06 | -0.3780 | 0 | 0.167 |
| ENSP00000275461 | 5.60E-06 | -0.3781 | 0 | 0.419 |
| ENSP00000287585 | 7.75E-06 | -0.3782 | 0 | 0.090 |
| ENSP00000419081 | 5.52E-06 | -0.3782 | 153 | 0.000 |
| ENSP00000263707 | 4.63E-06 | -0.3783 | 0 | 0.662 |
| ENSP00000165524 | 6.77E-06 | -0.3783 | 0 | 0.303 |
| ENSP00000267549 | 5.83E-06 | -0.3787 | 0 | 0.427 |
| ENSP00000273347 | 5.36E-06 | -0.3789 | 0 | 0.120 |
| ENSP00000349478 | 7.93E-06 | -0.3792 | 188 | 0.000 |
| ENSP00000281834 | 6.87E-06 | -0.3794 | 0 | 0.791 |
| ENSP00000315835 | 3.50E-06 | -0.3794 | 0 | 0.000 |
| ENSP00000229955 | 5.62E-06 | -0.3795 | 0 | 0.202 |
| ENSP00000347152 | 3.35E-06 | -0.3797 | 0 | 0.091 |
| ENSP00000284110 | 6.38E-06 | -0.3800 | 0 | 0.087 |
| ENSP00000216775 | 5.20E-06 | -0.3804 | 0 | 0.000 |
| ENSP00000233545 | 3.05E-06 | -0.3806 | 0 | 0.091 |
| ENSP00000390354 | 4.17E-06 | -0.3807 | 0 | 0.000 |
| ENSP00000369409 | 3.22E-06 | -0.3809 | 0 | 0.278 |
| ENSP00000273668 | 5.41E-06 | -0.3809 | 0 | 0.451 |
| ENSP00000466514 | 2.18E-06 | -0.3812 | 0 | 0.000 |
| ENSP00000329008 | 5.71E-06 | -0.3812 | 0 | 0.124 |
| ENSP00000381030 | 7.24E-06 | -0.3814 | 277 | 0.000 |
| ENSP00000347546 | 6.04E-06 | -0.3814 | 0 | 0.413 |
| ENSP00000303515 | 3.27E-06 | -0.3814 | 0 | 0.730 |
| ENSP00000355206 | 5.93E-06 | -0.3816 | 185 | 0.421 |
| ENSP00000381247 | 3.65E-06 | -0.3817 | 153 | 0.000 |
| ENSP00000330389 | 2.35E-06 | -0.3818 | 0 | 0.118 |
| ENSP00000302441 | 5.13E-06 | -0.3819 | 0 | 0.075 |
| ENSP00000400882 | 3.97E-06 | -0.3819 | 0 | 0.085 |
| ENSP00000336607 | 6.07E-06 | -0.3820 | 0 | 0.524 |
| ENSP00000296498 | 4.23E-06 | -0.3820 | 0 | 0.097 |
| ENSP00000346577 | 3.56E-06 | -0.3822 | 0 | 0.402 |
| ENSP00000321346 | 3.93E-06 | -0.3822 | 0 | 0.085 |
| ENSP00000327315 | 3.64E-06 | -0.3822 | 0 | 0.516 |
| ENSP00000318182 | 1.83E-06 | -0.3824 | 0 | 0.000 |
| ENSP00000441269 | 5.24E-06 | -0.3828 | 0 | 0.000 |

| | | | | |
|---|---|---|---|---|
| ENSP00000351706 | 4.77E-06 | -0.3828 | 305 | 0.382 |
| ENSP00000295190 | 3.12E-06 | -0.3831 | 0 | 0.054 |
| ENSP00000275954 | 3.77E-06 | -0.3832 | 0 | 0.121 |
| ENSP00000219197 | 6.98E-06 | -0.3838 | 0 | 0.134 |
| ENSP00000321987 | 6.84E-06 | -0.3839 | 0 | 0.189 |
| ENSP00000363832 | 7.45E-06 | -0.3839 | 0 | 0.220 |
| ENSP00000356729 | 4.49E-06 | -0.3842 | 0 | 0.122 |
| ENSP00000361636 | 9.03E-06 | -0.3843 | 907 | 0.000 |
| ENSP00000371297 | 4.21E-06 | -0.3844 | 0 | 0.128 |
| ENSP00000355787 | 8.03E-07 | -0.3845 | 0 | 0.000 |
| ENSP00000254765 | 3.40E-06 | -0.3847 | 0 | 0.113 |
| ENSP00000302537 | 6.68E-06 | -0.3848 | 0 | 0.287 |
| ENSP00000319744 | 8.01E-06 | -0.3850 | 165 | 0.476 |
| ENSP00000260126 | 4.51E-06 | -0.3854 | 0 | 0.053 |
| ENSP00000421315 | 5.73E-06 | -0.3855 | 193 | 0.000 |
| ENSP00000350552 | 3.89E-06 | -0.3855 | 0 | 0.323 |
| ENSP00000258385 | 9.02E-06 | -0.3857 | 0 | 0.146 |
| ENSP00000287777 | 7.97E-06 | -0.3857 | 0 | 0.114 |
| ENSP00000327417 | 4.42E-06 | -0.3857 | 0 | 0.367 |
| ENSP00000281882 | 6.57E-06 | -0.3860 | 0 | 0.161 |
| ENSP00000289292 | 5.65E-06 | -0.3860 | 0 | 0.099 |
| ENSP00000349477 | 4.62E-06 | -0.3860 | 0 | 0.484 |
| ENSP00000304250 | 5.51E-06 | -0.3862 | 0 | 0.074 |
| ENSP00000252087 | 4.97E-06 | -0.3862 | 0 | 0.108 |
| ENSP00000272433 | 3.47E-06 | -0.3865 | 0 | 0.083 |
| ENSP00000268919 | 2.57E-06 | -0.3868 | 0 | 0.214 |
| ENSP00000232892 | 5.80E-06 | -0.3875 | 0 | 0.150 |
| ENSP00000384892 | 9.13E-06 | -0.3876 | 0 | 0.211 |
| ENSP00000308339 | 3.62E-06 | -0.3876 | 0 | 0.082 |
| ENSP00000357637 | 4.39E-06 | -0.3877 | 0 | 0.657 |
| ENSP00000250699 | 4.44E-06 | -0.3881 | 0 | 0.184 |
| ENSP00000346667 | 4.83E-06 | -0.3881 | 0 | 0.133 |
| ENSP00000347358 | 3.17E-06 | -0.3882 | 0 | 0.219 |
| ENSP00000411940 | 4.15E-06 | -0.3886 | 0 | 0.566 |
| ENSP00000344549 | 8.57E-06 | -0.3886 | 302 | 0.467 |
| ENSP00000357835 | 1.61E-06 | -0.3891 | 0 | 0.088 |
| ENSP00000394653 | 2.92E-08 | -0.3891 | 0 | 0.150 |
| ENSP00000356251 | 4.50E-06 | -0.3892 | 0 | 0.269 |
| ENSP00000265840 | 3.02E-06 | -0.3895 | 0 | 0.117 |
| ENSP00000307674 | 6.12E-06 | -0.3895 | 0 | 0.173 |
| ENSP00000240618 | 4.01E-06 | -0.3896 | 0 | 0.000 |
| ENSP00000262593 | 6.71E-06 | -0.3896 | 0 | 0.189 |
| ENSP00000430000 | 2.64E-06 | -0.3897 | 0 | 0.197 |
| ENSP00000254325 | 4.29E-06 | -0.3898 | 0 | 0.763 |
| ENSP00000289957 | 4.77E-06 | -0.3900 | 0 | 0.201 |
| ENSP00000229729 | 6.28E-06 | -0.3900 | 0 | 0.126 |

| | | | | |
|---|---|---|---|---|
| ENSP00000280684 | 9.31E-06 | -0.3900 | 513 | 0.000 |
| ENSP00000264990 | 4.43E-06 | -0.3901 | 0 | 0.130 |
| ENSP00000359581 | 3.58E-06 | -0.3901 | 0 | 0.132 |
| ENSP00000298296 | 2.51E-06 | -0.3901 | 0 | 0.248 |
| ENSP00000346298 | 4.60E-06 | -0.3902 | 0 | 0.106 |
| ENSP00000296449 | 2.45E-06 | -0.3903 | 0 | 0.106 |
| ENSP00000256958 | 8.85E-06 | -0.3904 | 301 | 0.249 |
| ENSP00000312399 | 3.74E-06 | -0.3906 | 0 | 0.082 |
| ENSP00000263708 | 4.10E-06 | -0.3907 | 0 | 0.515 |
| ENSP00000343490 | 7.48E-06 | -0.3908 | 201 | 0.197 |
| ENSP00000263314 | 7.55E-06 | -0.3909 | 0 | 0.491 |
| ENSP00000355480 | 4.59E-06 | -0.3910 | 153 | 0.000 |
| ENSP00000252804 | 8.79E-06 | -0.3911 | 0 | 0.182 |
| ENSP00000281182 | 3.35E-06 | -0.3914 | 0 | 0.237 |
| ENSP00000338330 | 4.01E-06 | -0.3915 | 0 | 0.073 |
| ENSP00000270235 | 4.92E-06 | -0.3916 | 0 | 0.207 |
| ENSP00000217043 | 6.63E-06 | -0.3917 | 0 | 0.098 |
| ENSP00000155858 | 5.96E-06 | -0.3917 | 0 | 0.267 |
| ENSP00000326244 | 4.72E-06 | -0.3918 | 0 | 0.105 |
| ENSP00000393557 | 8.19E-06 | -0.3920 | 0 | 0.240 |
| ENSP00000303540 | 5.11E-06 | -0.3923 | 0 | 0.388 |
| ENSP00000273598 | 2.52E-06 | -0.3923 | 0 | 0.121 |
| ENSP00000353295 | 2.36E-06 | -0.3924 | 0 | 0.214 |
| ENSP00000265922 | 5.10E-06 | -0.3925 | 0 | 0.000 |
| ENSP00000299333 | 8.34E-06 | -0.3926 | 354 | 0.215 |
| ENSP00000267015 | 5.82E-06 | -0.3929 | 0 | 0.254 |
| ENSP00000385586 | 3.54E-06 | -0.3930 | 0 | 0.813 |
| ENSP00000295824 | 5.57E-06 | -0.3932 | 0 | 0.105 |
| ENSP00000337862 | 3.94E-06 | -0.3932 | 0 | 0.669 |
| ENSP00000262741 | 5.80E-06 | -0.3934 | 0 | 0.769 |
| ENSP00000326432 | 1.05E-05 | -0.3934 | 0 | 0.540 |
| ENSP00000306776 | 4.23E-06 | -0.3934 | 0 | 0.115 |
| ENSP00000313803 | 6.97E-07 | -0.3938 | 0 | 0.000 |
| ENSP00000319705 | 4.23E-06 | -0.3938 | 0 | 0.064 |
| ENSP00000333917 | 4.27E-06 | -0.3940 | 0 | 0.652 |
| ENSP00000064780 | 8.25E-06 | -0.3940 | 0 | 0.158 |
| ENSP00000236709 | 5.30E-06 | -0.3941 | 0 | 0.074 |
| ENSP00000284674 | 3.89E-06 | -0.3942 | 0 | 0.205 |
| ENSP00000298110 | 6.98E-06 | -0.3952 | 0 | 0.247 |
| ENSP00000369564 | 2.85E-06 | -0.3954 | 0 | 0.289 |
| ENSP00000246104 | 6.47E-06 | -0.3956 | 0 | 0.800 |
| ENSP00000283256 | 8.44E-06 | -0.3958 | 163 | 0.533 |
| ENSP00000298974 | 2.75E-06 | -0.3958 | 0 | 0.275 |
| ENSP00000333300 | 4.85E-06 | -0.3959 | 0 | 0.228 |
| ENSP00000321976 | 8.72E-06 | -0.3959 | 201 | 0.605 |
| ENSP00000256404 | 3.48E-06 | -0.3961 | 0 | 0.176 |

| | | | | |
|---|---|---|---|---|
| ENSP00000340609 | 4.10E-06 | -0.3962 | 0 | 0.611 |
| ENSP00000336764 | 1.14E-05 | -0.3963 | 240 | 0.608 |
| ENSP00000328472 | 4.19E-06 | -0.3964 | 0 | 0.461 |
| ENSP00000300778 | 2.01E-06 | -0.3965 | 0 | 0.000 |
| ENSP00000389870 | 7.20E-06 | -0.3968 | 0 | 0.876 |
| ENSP00000327824 | 5.46E-06 | -0.3969 | 0 | 0.537 |
| ENSP00000386201 | 4.84E-06 | -0.3970 | 0 | 0.474 |
| ENSP00000284562 | 6.08E-06 | -0.3972 | 0 | 0.108 |
| ENSP00000450281 | 1.34E-06 | -0.3974 | 0 | 0.113 |
| ENSP00000278947 | 3.44E-06 | -0.3975 | 0 | 0.123 |
| ENSP00000262794 | 3.56E-06 | -0.3976 | 0 | 0.779 |
| ENSP00000323194 | 4.43E-06 | -0.3976 | 0 | 0.563 |
| ENSP00000322300 | 2.97E-06 | -0.3977 | 0 | 0.219 |
| ENSP00000343995 | 2.64E-06 | -0.3979 | 0 | 0.066 |
| ENSP00000301202 | 3.16E-03 | -0.3979 | 0 | 0.189 |
| ENSP00000252997 | 7.52E-06 | -0.3979 | 596 | 0.000 |
| ENSP00000220478 | 6.68E-06 | -0.3980 | 0 | 0.094 |
| ENSP00000377344 | 3.53E-06 | -0.3980 | 0 | 0.000 |
| ENSP00000337446 | 5.01E-06 | -0.3980 | 0 | 0.273 |
| ENSP00000415398 | 4.75E-06 | -0.3986 | 173 | 0.000 |
| ENSP00000345824 | 3.67E-06 | -0.3986 | 0 | 0.068 |
| ENSP00000355273 | 4.16E-06 | -0.3986 | 0 | 0.112 |
| ENSP00000324651 | 5.23E-06 | -0.3987 | 0 | 0.051 |
| ENSP00000346990 | 5.64E-06 | -0.3987 | 0 | 0.123 |
| ENSP00000236166 | 5.07E-06 | -0.3988 | 0 | 0.000 |
| ENSP00000386210 | 3.77E-06 | -0.3989 | 0 | 0.479 |
| ENSP00000316518 | 3.33E-05 | -0.3997 | 0 | 0.219 |
| ENSP00000329990 | 9.29E-06 | -0.4001 | 201 | 0.000 |
| ENSP00000289248 | 3.67E-06 | -0.4001 | 0 | 0.437 |
| ENSP00000361700 | 4.30E-06 | -0.4001 | 0 | 0.260 |
| ENSP00000263726 | 5.18E-06 | -0.4003 | 0 | 0.785 |
| ENSP00000303212 | 5.31E-06 | -0.4004 | 0 | 0.544 |
| ENSP00000358466 | 2.01E-06 | -0.4004 | 0 | 0.066 |
| ENSP00000354347 | 3.17E-03 | -0.4005 | 471 | 0.629 |
| ENSP00000314173 | 3.40E-06 | -0.4007 | 0 | 0.125 |
| ENSP00000261797 | 8.11E-06 | -0.4008 | 196 | 0.342 |
| ENSP00000276646 | 4.51E-06 | -0.4009 | 0 | 0.090 |
| ENSP00000265500 | 3.12E-06 | -0.4010 | 0 | 0.000 |
| ENSP00000239032 | 6.92E-06 | -0.4010 | 0 | 0.417 |
| ENSP00000449253 | 3.75E-06 | -0.4011 | 0 | 0.174 |
| ENSP00000225410 | 4.77E-06 | -0.4011 | 0 | 0.000 |
| ENSP00000296142 | 2.28E-06 | -0.4012 | 0 | 0.106 |
| ENSP00000274629 | 6.40E-06 | -0.4013 | 0 | 0.182 |
| ENSP00000335578 | 5.69E-06 | -0.4013 | 0 | 0.818 |
| ENSP00000293973 | 5.57E-06 | -0.4013 | 0 | 0.512 |
| ENSP00000291839 | 5.25E-06 | -0.4014 | 0 | 0.078 |

| | | | | |
|---|---|---|---|---|
| ENSP00000207157 | 5.70E-06 | -0.4015 | 0 | 0.787 |
| ENSP00000323479 | 5.12E-06 | -0.4015 | 0 | 0.102 |
| ENSP00000316416 | 4.63E-06 | -0.4016 | 0 | 0.254 |
| ENSP00000360310 | 4.72E-06 | -0.4019 | 0 | 0.756 |
| ENSP00000257868 | 7.13E-06 | -0.4022 | 0 | 0.873 |
| ENSP00000316173 | 6.34E-06 | -0.4028 | 0 | 0.132 |
| ENSP00000353796 | 5.82E-06 | -0.4028 | 201 | 0.000 |
| ENSP00000280350 | 3.76E-06 | -0.4029 | 0 | 0.168 |
| ENSP00000300873 | 4.79E-06 | -0.4033 | 0 | 0.525 |
| ENSP00000339973 | 2.09E-06 | -0.4034 | 0 | 0.219 |
| ENSP00000366549 | 3.85E-06 | -0.4035 | 0 | 0.101 |
| ENSP00000331636 | 2.06E-06 | -0.4039 | 0 | 0.000 |
| ENSP00000452854 | 3.29E-06 | -0.4041 | 0 | 0.714 |
| ENSP00000319318 | 3.56E-06 | -0.4041 | 0 | 0.610 |
| ENSP00000432622 | 7.34E-08 | -0.4043 | 0 | 0.000 |
| ENSP00000457849 | 2.17E-06 | -0.4044 | 0 | 0.000 |
| ENSP00000339820 | 4.48E-06 | -0.4046 | 0 | 0.140 |
| ENSP00000282185 | 4.83E-06 | -0.4047 | 0 | 0.072 |
| ENSP00000317619 | 3.96E-06 | -0.4048 | 0 | 0.062 |
| ENSP00000263881 | 5.63E-06 | -0.4048 | 246 | 0.640 |
| ENSP00000242317 | 4.28E-06 | -0.4051 | 0 | 0.151 |
| ENSP00000301887 | 7.78E-06 | -0.4053 | 224 | 0.000 |
| ENSP00000303775 | 7.44E-06 | -0.4053 | 0 | 0.526 |
| ENSP00000349490 | 5.91E-06 | -0.4054 | 0 | 0.744 |
| ENSP00000353151 | 5.14E-06 | -0.4056 | 0 | 0.821 |
| ENSP00000378611 | 3.96E-06 | -0.4056 | 0 | 0.421 |
| ENSP00000262648 | 8.40E-06 | -0.4057 | 465 | 0.000 |
| ENSP00000219320 | 5.08E-06 | -0.4063 | 0 | 0.039 |
| ENSP00000349320 | 4.64E-06 | -0.4063 | 0 | 0.247 |
| ENSP00000252602 | 3.37E-06 | -0.4063 | 0 | 0.553 |
| ENSP00000269197 | 4.77E-06 | -0.4064 | 0 | 0.724 |
| ENSP00000215530 | 6.83E-06 | -0.4066 | 0 | 0.903 |
| ENSP00000355082 | 3.05E-06 | -0.4066 | 0 | 0.102 |
| ENSP00000307126 | 5.98E-06 | -0.4067 | 0 | 0.083 |
| ENSP00000335006 | 3.79E-06 | -0.4067 | 0 | 0.116 |
| ENSP00000260323 | 3.82E-06 | -0.4069 | 0 | 0.186 |
| ENSP00000345014 | 2.13E-06 | -0.4069 | 0 | 0.000 |
| ENSP00000264249 | 4.88E-06 | -0.4070 | 0 | 0.059 |
| ENSP00000383212 | 3.16E-06 | -0.4072 | 0 | 0.134 |
| ENSP00000203629 | 1.10E-05 | -0.4073 | 0 | 0.511 |
| ENSP00000233735 | 8.91E-06 | -0.4073 | 0 | 0.251 |
| ENSP00000430846 | 2.30E-06 | -0.4075 | 0 | 0.049 |
| ENSP00000290332 | 3.43E-06 | -0.4076 | 0 | 0.061 |
| ENSP00000335191 | 4.65E-06 | -0.4076 | 0 | 0.766 |
| ENSP00000338191 | 4.87E-06 | -0.4077 | 0 | 0.097 |
| ENSP00000361914 | 6.73E-06 | -0.4077 | 369 | 0.191 |

| | | | | |
|---|---|---|---|---|
| ENSP00000223145 | 3.26E-06 | -0.4078 | 0 | 0.097 |
| ENSP00000382260 | 3.42E-06 | -0.4078 | 0 | 0.140 |
| ENSP00000332068 | 5.86E-07 | -0.4079 | 0 | 0.000 |
| ENSP00000348793 | 3.44E-06 | -0.4085 | 0 | 0.000 |
| ENSP00000055335 | 6.17E-06 | -0.4086 | 0 | 0.208 |
| ENSP00000017003 | 3.57E-06 | -0.4088 | 0 | 0.051 |
| ENSP00000320431 | 3.81E-06 | -0.4088 | 0 | 0.050 |
| ENSP00000369573 | 9.85E-06 | -0.4088 | 0 | 0.000 |
| ENSP00000325674 | 4.83E-06 | -0.4093 | 0 | 0.269 |
| ENSP00000252677 | 7.10E-06 | -0.4094 | 0 | 0.852 |
| ENSP00000452398 | 4.85E-06 | -0.4094 | 0 | 0.791 |
| ENSP00000417871 | 4.53E-06 | -0.4095 | 317 | 0.568 |
| ENSP00000332124 | 3.80E-06 | -0.4097 | 0 | 0.000 |
| ENSP00000312587 | 3.38E-06 | -0.4102 | 0 | 0.104 |
| ENSP00000363398 | 4.77E-06 | -0.4109 | 0 | 0.147 |
| ENSP00000401536 | 2.30E-06 | -0.4114 | 0 | 0.688 |
| ENSP00000334644 | 4.27E-06 | -0.4118 | 0 | 0.111 |
| ENSP00000279575 | 4.15E-06 | -0.4118 | 0 | 0.152 |
| ENSP00000360811 | 6.35E-06 | -0.4120 | 0 | 0.837 |
| ENSP00000348460 | 5.62E-06 | -0.4122 | 163 | 0.682 |
| ENSP00000261448 | 5.74E-06 | -0.4123 | 0 | 0.236 |
| ENSP00000362398 | 3.65E-06 | -0.4124 | 0 | 0.000 |
| ENSP00000354966 | 3.62E-06 | -0.4127 | 0 | 0.107 |
| ENSP00000356687 | 5.15E-06 | -0.4132 | 558 | 0.098 |
| ENSP00000300688 | 2.74E-06 | -0.4132 | 0 | 0.280 |
| ENSP00000262811 | 3.90E-06 | -0.4136 | 0 | 0.385 |
| ENSP00000299308 | 1.72E-06 | -0.4137 | 0 | 0.206 |
| ENSP00000354963 | 3.28E-06 | -0.4140 | 0 | 0.072 |
| ENSP00000308733 | 4.80E-06 | -0.4141 | 0 | 0.261 |
| ENSP00000377311 | 4.45E-06 | -0.4143 | 535 | 0.246 |
| ENSP00000267731 | 4.44E-06 | -0.4148 | 0 | 0.000 |
| ENSP00000366682 | 3.89E-06 | -0.4149 | 0 | 0.327 |
| ENSP00000258829 | 5.25E-06 | -0.4154 | 0 | 0.814 |
| ENSP00000334547 | 4.51E-06 | -0.4155 | 0 | 0.755 |
| ENSP00000282096 | 5.98E-06 | -0.4155 | 0 | 0.663 |
| ENSP00000209718 | 4.47E-06 | -0.4159 | 0 | 0.147 |
| ENSP00000303740 | 4.42E-06 | -0.4162 | 0 | 0.000 |
| ENSP00000269081 | 4.88E-06 | -0.4166 | 0 | 0.082 |
| ENSP00000359579 | 4.01E-06 | -0.4169 | 0 | 0.000 |
| ENSP00000256343 | 3.84E-06 | -0.4174 | 0 | 0.061 |
| ENSP00000310696 | 6.34E-06 | -0.4175 | 0 | 0.077 |
| ENSP00000198801 | 4.39E-06 | -0.4175 | 0 | 0.190 |
| ENSP00000305824 | 3.48E-06 | -0.4177 | 0 | 0.153 |
| ENSP00000319991 | 3.95E-06 | -0.4181 | 0 | 0.050 |
| ENSP00000262113 | 6.73E-06 | -0.4181 | 0 | 0.212 |
| ENSP00000362131 | 2.67E-06 | -0.4183 | 0 | 0.666 |

| | | | | |
|---|---|---|---|---|
| ENSP00000341682 | 4.10E-06 | -0.4184 | 0 | 0.082 |
| ENSP00000348821 | 3.21E-06 | -0.4185 | 0 | 0.233 |
| ENSP00000359300 | 4.90E-06 | -0.4186 | 0 | 0.661 |
| ENSP00000306678 | 7.96E-06 | -0.4190 | 201 | 0.638 |
| ENSP00000353655 | 4.55E-06 | -0.4192 | 0 | 0.117 |
| ENSP00000363232 | 1.83E-06 | -0.4200 | 0 | 0.182 |
| ENSP00000360577 | 2.98E-06 | -0.4201 | 0 | 0.236 |
| ENSP00000361952 | 2.36E-06 | -0.4204 | 0 | 0.077 |
| ENSP00000218104 | 5.93E-06 | -0.4207 | 0 | 0.191 |
| ENSP00000466174 | 8.69E-06 | -0.4212 | 224 | 0.000 |
| ENSP00000363807 | 3.30E-06 | -0.4212 | 0 | 0.088 |
| ENSP00000334198 | 3.26E-06 | -0.4213 | 0 | 0.297 |
| ENSP00000225609 | 5.38E-06 | -0.4216 | 0 | 0.097 |
| ENSP00000369530 | 4.64E-06 | -0.4226 | 0 | 0.155 |
| ENSP00000352782 | 2.97E-06 | -0.4227 | 0 | 0.125 |
| ENSP00000385632 | 6.06E-06 | -0.4228 | 511 | 0.657 |
| ENSP00000333292 | 3.70E-06 | -0.4229 | 0 | 0.074 |
| ENSP00000307694 | 4.30E-06 | -0.4231 | 0 | 0.110 |
| ENSP00000383023 | 6.25E-06 | -0.4233 | 0 | 0.393 |
| ENSP00000294117 | 5.85E-06 | -0.4238 | 0 | 0.447 |
| ENSP00000359045 | 2.70E-06 | -0.4239 | 0 | 0.091 |
| ENSP00000362103 | 4.28E-06 | -0.4240 | 0 | 0.848 |
| ENSP00000215479 | 6.56E-06 | -0.4244 | 0 | 0.093 |
| ENSP00000451628 | 2.34E-06 | -0.4245 | 0 | 0.000 |
| ENSP00000281474 | 2.63E-06 | -0.4246 | 0 | 0.117 |
| ENSP00000225941 | 3.68E-06 | -0.4254 | 0 | 0.275 |
| ENSP00000268674 | 6.22E-06 | -0.4255 | 0 | 0.000 |
| ENSP00000350248 | 3.08E-07 | -0.4256 | 0 | 0.000 |
| ENSP00000314129 | 7.41E-06 | -0.4259 | 408 | 0.000 |
| ENSP00000369689 | 5.18E-06 | -0.4266 | 153 | 0.000 |
| ENSP00000339221 | 3.33E-06 | -0.4268 | 0 | 0.000 |
| ENSP00000334181 | 5.77E-06 | -0.4270 | 163 | 0.000 |
| ENSP00000355441 | 2.91E-07 | -0.4270 | 0 | 0.000 |
| ENSP00000209929 | 5.25E-06 | -0.4273 | 0 | 0.081 |
| ENSP00000384665 | 1.38E-05 | -0.4274 | 165 | 0.651 |
| ENSP00000295237 | 3.58E-06 | -0.4275 | 0 | 0.121 |
| ENSP00000463576 | 4.12E-06 | -0.4286 | 173 | 0.000 |
| ENSP00000323071 | 3.37E-06 | -0.4287 | 0 | 0.227 |
| ENSP00000262717 | 4.13E-06 | -0.4288 | 0 | 0.097 |
| ENSP00000246515 | 6.63E-06 | -0.4289 | 0 | 0.203 |
| ENSP00000257215 | 5.77E-06 | -0.4290 | 0 | 0.141 |
| ENSP00000324419 | 3.36E-06 | -0.4290 | 0 | 0.173 |
| ENSP00000265825 | 4.30E-06 | -0.4293 | 0 | 0.082 |
| ENSP00000366061 | 9.26E-06 | -0.4298 | 151 | 0.548 |
| ENSP00000356793 | 5.28E-06 | -0.4306 | 0 | 0.482 |
| ENSP00000324857 | 4.04E-06 | -0.4308 | 0 | 0.524 |

| | | | | |
|---|---|---|---|---|
| ENSP00000289753 | 7.23E-06 | -0.4310 | 0 | 0.634 |
| ENSP00000397351 | 3.31E-06 | -0.4311 | 0 | 0.288 |
| ENSP00000319370 | 2.27E-06 | -0.4315 | 0 | 0.098 |
| ENSP00000371377 | 1.49E-06 | -0.4319 | 0 | 0.000 |
| ENSP00000253401 | 4.53E-06 | -0.4322 | 0 | 0.092 |
| ENSP00000242462 | 6.68E-06 | -0.4325 | 0 | 0.847 |
| ENSP00000305696 | 3.46E-06 | -0.4330 | 0 | 0.339 |
| ENSP00000345436 | 7.78E-06 | -0.4332 | 201 | 0.664 |
| ENSP00000379377 | 5.58E-06 | -0.4332 | 0 | 0.113 |
| ENSP00000318974 | 2.32E-06 | -0.4336 | 0 | 0.116 |
| ENSP00000424595 | 5.36E-06 | -0.4340 | 241 | 0.621 |
| ENSP00000230323 | 2.05E-06 | -0.4341 | 0 | 0.000 |
| ENSP00000299989 | 4.59E-06 | -0.4341 | 0 | 0.000 |
| ENSP00000216416 | 3.40E-06 | -0.4342 | 0 | 0.000 |
| ENSP00000265459 | 4.31E-06 | -0.4349 | 0 | 0.148 |
| ENSP00000259895 | 7.04E-06 | -0.4349 | 0 | 0.577 |
| ENSP00000280056 | 4.96E-06 | -0.4352 | 0 | 0.190 |
| ENSP00000364831 | 4.57E-06 | -0.4358 | 0 | 0.132 |
| ENSP00000350022 | 6.76E-07 | -0.4362 | 0 | 0.000 |
| ENSP00000357087 | 2.74E-06 | -0.4364 | 0 | 0.000 |
| ENSP00000331600 | 3.71E-06 | -0.4366 | 0 | 0.229 |
| ENSP00000278882 | 3.63E-06 | -0.4367 | 0 | 0.000 |
| ENSP00000216124 | 6.76E-06 | -0.4368 | 0 | 0.137 |
| ENSP00000225276 | 4.49E-06 | -0.4372 | 0 | 0.085 |
| ENSP00000378690 | 4.05E-06 | -0.4372 | 153 | 0.000 |
| ENSP00000307006 | 4.74E-06 | -0.4373 | 0 | 0.768 |
| ENSP00000269945 | 4.32E-06 | -0.4376 | 0 | 0.507 |
| ENSP00000261381 | 4.01E-06 | -0.4380 | 0 | 0.049 |
| ENSP00000466795 | 2.06E-06 | -0.4381 | 0 | 0.000 |
| ENSP00000347721 | 3.43E-06 | -0.4381 | 0 | 0.065 |
| ENSP00000349577 | 7.42E-06 | -0.4382 | 204 | 0.282 |
| ENSP00000207457 | 3.96E-06 | -0.4382 | 0 | 0.054 |
| ENSP00000334113 | 3.04E-06 | -0.4383 | 0 | 0.201 |
| ENSP00000329991 | 5.59E-06 | -0.4384 | 0 | 0.710 |
| ENSP00000411096 | 5.85E-06 | -0.4387 | 207 | 0.149 |
| ENSP00000229134 | 7.37E-06 | -0.4390 | 0 | 0.554 |
| ENSP00000305613 | 4.15E-06 | -0.4392 | 0 | 0.203 |
| ENSP00000450554 | 2.07E-06 | -0.4393 | 0 | 0.000 |
| ENSP00000335560 | 5.39E-06 | -0.4401 | 0 | 0.592 |
| ENSP00000312385 | 4.30E-06 | -0.4403 | 0 | 0.813 |
| ENSP00000239587 | 3.90E-06 | -0.4413 | 0 | 0.121 |
| ENSP00000324343 | 3.77E-06 | -0.4413 | 0 | 0.089 |
| ENSP00000281047 | 6.28E-06 | -0.4414 | 0 | 0.812 |
| ENSP00000264051 | 4.21E-06 | -0.4418 | 0 | 0.631 |
| ENSP00000332766 | 4.93E-06 | -0.4420 | 0 | 0.514 |
| ENSP00000345147 | 5.66E-06 | -0.4424 | 0 | 0.802 |

| | | | | |
|---|---|---|---|---|
| ENSP00000328598 | 4.37E-06 | -0.4427 | 0 | 0.812 |
| ENSP00000294725 | 3.34E-06 | -0.4433 | 0 | 0.057 |
| ENSP00000318982 | 3.65E-06 | -0.4435 | 0 | 0.179 |
| ENSP00000327465 | 3.73E-06 | -0.4436 | 0 | 0.343 |
| ENSP00000354961 | 3.59E-06 | -0.4441 | 0 | 0.275 |
| ENSP00000365895 | 4.20E-06 | -0.4445 | 0 | 0.060 |
| ENSP00000331933 | 3.57E-06 | -0.4447 | 0 | 0.354 |
| ENSP00000035307 | 4.44E-06 | -0.4450 | 0 | 0.053 |
| ENSP00000430241 | 3.40E-06 | -0.4450 | 0 | 0.766 |
| ENSP00000355354 | 3.06E-06 | -0.4454 | 0 | 0.668 |
| ENSP00000334050 | 4.42E-06 | -0.4462 | 0 | 0.487 |
| ENSP00000296499 | 4.81E-06 | -0.4468 | 0 | 0.285 |
| ENSP00000289575 | 4.94E-06 | -0.4470 | 0 | 0.049 |
| ENSP00000372319 | 5.75E-06 | -0.4473 | 163 | 0.000 |
| ENSP00000262301 | 6.95E-06 | -0.4476 | 215 | 0.164 |
| ENSP00000302194 | 2.70E-06 | -0.4476 | 0 | 0.056 |
| ENSP00000249598 | 9.39E-06 | -0.4482 | 0 | 0.000 |
| ENSP00000309794 | 3.34E-06 | -0.4484 | 0 | 0.173 |
| ENSP00000405095 | 2.90E-06 | -0.4495 | 0 | 0.486 |
| ENSP00000307971 | 5.30E-06 | -0.4497 | 0 | 0.761 |
| ENSP00000320965 | 3.88E-06 | -0.4499 | 0 | 0.057 |
| ENSP00000328818 | 4.97E-06 | -0.4503 | 0 | 0.522 |
| ENSP00000284425 | 8.61E-06 | -0.4503 | 0 | 0.088 |
| ENSP00000303252 | 6.00E-06 | -0.4506 | 255 | 0.262 |
| ENSP00000333310 | 7.87E-06 | -0.4510 | 0 | 0.096 |
| ENSP00000223324 | 2.53E-06 | -0.4511 | 0 | 0.636 |
| ENSP00000283228 | 4.51E-06 | -0.4513 | 0 | 0.669 |
| ENSP00000321345 | 4.81E-06 | -0.4517 | 0 | 0.620 |
| ENSP00000352144 | 3.02E-06 | -0.4524 | 0 | 0.077 |
| ENSP00000380523 | 5.04E-06 | -0.4528 | 0 | 0.646 |
| ENSP00000260283 | 3.02E-06 | -0.4529 | 0 | 0.238 |
| ENSP00000265022 | 3.52E-06 | -0.4534 | 0 | 0.116 |
| ENSP00000304235 | 1.16E-06 | -0.4542 | 0 | 0.000 |
| ENSP00000308334 | 3.20E-06 | -0.4546 | 0 | 0.051 |
| ENSP00000362354 | 5.07E-06 | -0.4550 | 180 | 0.666 |
| ENSP00000381758 | 3.58E-06 | -0.4550 | 0 | 0.000 |
| ENSP00000315006 | 5.00E-06 | -0.4550 | 0 | 0.078 |
| ENSP00000308770 | 5.91E-06 | -0.4556 | 0 | 0.074 |
| ENSP00000264433 | 2.86E-06 | -0.4557 | 0 | 0.100 |
| ENSP00000304502 | 5.61E-06 | -0.4558 | 0 | 0.844 |
| ENSP00000301175 | 4.55E-06 | -0.4558 | 0 | 0.144 |
| ENSP00000301050 | 3.40E-06 | -0.4558 | 0 | 0.177 |
| ENSP00000307766 | 8.81E-06 | -0.4558 | 211 | 0.510 |
| ENSP00000290390 | 3.90E-06 | -0.4563 | 0 | 0.196 |
| ENSP00000330276 | 4.09E-06 | -0.4570 | 0 | 0.477 |
| ENSP00000380053 | 6.12E-06 | -0.4572 | 0 | 0.819 |

| | | | | |
|---|---|---|---|---|
| ENSP00000282146 | 3.33E-06 | -0.4580 | 0 | 0.061 |
| ENSP00000341237 | 4.75E-06 | -0.4582 | 173 | 0.000 |
| ENSP00000287042 | 4.14E-06 | -0.4584 | 0 | 0.060 |
| ENSP00000302836 | 4.87E-06 | -0.4589 | 0 | 0.730 |
| ENSP00000247138 | 4.03E-06 | -0.4594 | 0 | 0.114 |
| ENSP00000264554 | 7.22E-06 | -0.4600 | 0 | 0.804 |
| ENSP00000320219 | 4.03E-06 | -0.4601 | 0 | 0.077 |
| ENSP00000258324 | 4.59E-06 | -0.4604 | 0 | 0.000 |
| ENSP00000264020 | 3.14E-06 | -0.4608 | 0 | 0.071 |
| ENSP00000313661 | 6.00E-06 | -0.4611 | 0 | 0.000 |
| ENSP00000338349 | 4.15E-06 | -0.4613 | 0 | 0.182 |
| ENSP00000431719 | 2.85E-06 | -0.4614 | 0 | 0.497 |
| ENSP00000253122 | 3.73E-06 | -0.4621 | 0 | 0.077 |
| ENSP00000329256 | 2.32E-06 | -0.4637 | 0 | 0.000 |
| ENSP00000329684 | 4.93E-06 | -0.4640 | 0 | 0.490 |
| ENSP00000273814 | 4.35E-06 | -0.4640 | 0 | 0.132 |
| ENSP00000368109 | 4.14E-06 | -0.4647 | 0 | 0.194 |
| ENSP00000423313 | 1.87E-06 | -0.4648 | 0 | 0.123 |
| ENSP00000268989 | 2.85E-06 | -0.4649 | 0 | 0.161 |
| ENSP00000363350 | 2.22E-06 | -0.4652 | 0 | 0.000 |
| ENSP00000322924 | 8.81E-06 | -0.4653 | 234 | 0.589 |
| ENSP00000434241 | 2.56E-06 | -0.4654 | 0 | 0.145 |
| ENSP00000260953 | 3.99E-06 | -0.4655 | 0 | 0.256 |
| ENSP00000288490 | 2.60E-06 | -0.4655 | 0 | 0.103 |
| ENSP00000266383 | 4.24E-06 | -0.4663 | 0 | 0.123 |
| ENSP00000226067 | 6.40E-06 | -0.4665 | 0 | 0.695 |
| ENSP00000359683 | 2.54E-06 | -0.4673 | 0 | 0.000 |
| ENSP00000307726 | 7.03E-07 | -0.4683 | 0 | 0.000 |
| ENSP00000327724 | 4.44E-06 | -0.4684 | 0 | 0.489 |
| ENSP00000419266 | 4.17E-06 | -0.4687 | 0 | 0.828 |
| ENSP00000328230 | 4.83E-06 | -0.4699 | 0 | 0.476 |
| ENSP00000258111 | 3.85E-06 | -0.4707 | 0 | 0.105 |
| ENSP00000380153 | 3.25E-06 | -0.4709 | 0 | 0.000 |
| ENSP00000229335 | 5.61E-06 | -0.4709 | 0 | 0.693 |
| ENSP00000264151 | 5.78E-06 | -0.4716 | 0 | 0.269 |
| ENSP00000331698 | 3.04E-06 | -0.4720 | 0 | 0.167 |
| ENSP00000297146 | 3.88E-06 | -0.4721 | 0 | 0.240 |
| ENSP00000348772 | 6.23E-06 | -0.4722 | 360 | 0.000 |
| ENSP00000230053 | 3.89E-06 | -0.4727 | 0 | 0.078 |
| ENSP00000354416 | 5.74E-06 | -0.4733 | 0 | 0.547 |
| ENSP00000367930 | 5.30E-06 | -0.4735 | 0 | 0.474 |
| ENSP00000297994 | 3.46E-06 | -0.4735 | 0 | 0.743 |
| ENSP00000261196 | 5.80E-06 | -0.4741 | 0 | 0.167 |
| ENSP00000363939 | 3.59E-06 | -0.4744 | 0 | 0.000 |
| ENSP00000405950 | 3.66E-06 | -0.4747 | 0 | 0.000 |
| ENSP00000358802 | 3.32E-06 | -0.4750 | 0 | 0.151 |

| | | | | |
|---|---|---|---|---|
| ENSP00000231228 | 8.44E-06 | -0.4757 | 0 | 0.755 |
| ENSP00000320957 | 4.80E-06 | -0.4762 | 0 | 0.793 |
| ENSP00000386167 | 1.16E-06 | -0.4768 | 0 | 0.000 |
| ENSP00000363459 | 2.28E-06 | -0.4776 | 0 | 0.000 |
| ENSP00000264661 | 4.37E-06 | -0.4777 | 0 | 0.096 |
| ENSP00000301305 | 3.03E-06 | -0.4781 | 0 | 0.110 |
| ENSP00000262895 | 4.98E-06 | -0.4782 | 0 | 0.309 |
| ENSP00000243913 | 4.32E-06 | -0.4783 | 0 | 0.067 |
| ENSP00000263080 | 6.06E-06 | -0.4785 | 0 | 0.082 |
| ENSP00000259241 | 5.30E-06 | -0.4786 | 0 | 0.382 |
| ENSP00000330384 | 9.25E-07 | -0.4788 | 0 | 0.000 |
| ENSP00000341821 | 7.59E-06 | -0.4790 | 0 | 0.476 |
| ENSP00000226021 | 3.98E-06 | -0.4797 | 0 | 0.166 |
| ENSP00000328277 | 3.04E-06 | -0.4797 | 0 | 0.124 |
| ENSP00000452549 | 2.53E-06 | -0.4798 | 0 | 0.267 |
| ENSP00000302166 | 3.56E-06 | -0.4801 | 0 | 0.171 |
| ENSP00000295314 | 6.50E-06 | -0.4808 | 0 | 0.329 |
| ENSP00000355156 | 4.29E-06 | -0.4813 | 0 | 0.464 |
| ENSP00000439425 | 3.53E-06 | -0.4814 | 0 | 0.000 |
| ENSP00000274093 | 5.71E-06 | -0.4818 | 0 | 0.279 |
| ENSP00000259216 | 6.45E-06 | -0.4824 | 0 | 0.567 |
| ENSP00000312129 | 3.72E-06 | -0.4828 | 0 | 0.048 |
| ENSP00000263278 | 5.35E-06 | -0.4833 | 0 | 0.230 |
| ENSP00000302801 | 2.76E-06 | -0.4846 | 0 | 0.799 |
| ENSP00000240361 | 2.85E-06 | -0.4849 | 0 | 0.083 |
| ENSP00000312329 | 3.09E-06 | -0.4849 | 0 | 0.000 |
| ENSP00000363296 | 9.04E-06 | -0.4850 | 324 | 0.574 |
| ENSP00000361475 | 3.56E-06 | -0.4850 | 0 | 0.292 |
| ENSP00000341479 | 3.13E-06 | -0.4853 | 0 | 0.194 |
| ENSP00000369960 | 5.95E-06 | -0.4855 | 0 | 0.497 |
| ENSP00000283936 | 3.74E-06 | -0.4864 | 0 | 0.197 |
| ENSP00000385247 | 3.16E-06 | -0.4865 | 0 | 0.000 |
| ENSP00000287490 | 3.23E-06 | -0.4871 | 0 | 0.249 |
| ENSP00000378492 | 9.02E-06 | -0.4878 | 426 | 0.615 |
| ENSP00000401514 | 2.62E-06 | -0.4884 | 0 | 0.118 |
| ENSP00000308908 | 4.46E-06 | -0.4884 | 0 | 0.147 |
| ENSP00000249601 | 4.74E-06 | -0.4885 | 0 | 0.452 |
| ENSP00000355837 | 3.29E-06 | -0.4889 | 0 | 0.000 |
| ENSP00000276211 | 3.22E-06 | -0.4892 | 0 | 0.281 |
| ENSP00000295055 | 2.48E-06 | -0.4895 | 0 | 0.631 |
| ENSP00000350836 | 8.21E-07 | -0.4905 | 0 | 0.000 |
| ENSP00000332812 | 5.89E-06 | -0.4905 | 0 | 0.529 |
| ENSP00000251108 | 3.57E-06 | -0.4908 | 0 | 0.279 |
| ENSP00000230582 | 6.28E-06 | -0.4908 | 0 | 0.110 |
| ENSP00000247883 | 6.16E-06 | -0.4909 | 0 | 0.493 |
| ENSP00000373193 | 3.62E-06 | -0.4924 | 0 | 0.000 |

| | | | | |
|---|---|---|---|---|
| ENSP00000264977 | 3.75E-06 | -0.4932 | 0 | 0.227 |
| ENSP00000262622 | 4.07E-06 | -0.4936 | 0 | 0.105 |
| ENSP00000355316 | 7.96E-06 | -0.4944 | 316 | 0.634 |
| ENSP00000248150 | 5.63E-06 | -0.4953 | 0 | 0.544 |
| ENSP00000243103 | 3.45E-06 | -0.4954 | 0 | 0.630 |
| ENSP00000322898 | 5.44E-06 | -0.4954 | 0 | 0.849 |
| ENSP00000229554 | 2.92E-06 | -0.4957 | 0 | 0.034 |
| ENSP00000263160 | 6.48E-06 | -0.4972 | 0 | 0.542 |
| ENSP00000465737 | 2.26E-06 | -0.4978 | 0 | 0.000 |
| ENSP00000302968 | 4.21E-06 | -0.4978 | 163 | 0.000 |
| ENSP00000297404 | 2.73E-06 | -0.4989 | 0 | 0.091 |
| ENSP00000244174 | 5.41E-06 | -0.4995 | 0 | 0.448 |
| ENSP00000215368 | 5.69E-06 | -0.4996 | 0 | 0.832 |
| ENSP00000318502 | 4.25E-06 | -0.4998 | 0 | 0.682 |
| ENSP00000274849 | 2.80E-06 | -0.5003 | 0 | 0.853 |
| ENSP00000452702 | 4.15E-06 | -0.5013 | 0 | 0.652 |
| ENSP00000286639 | 7.25E-06 | -0.5029 | 273 | 0.000 |
| ENSP00000243440 | 6.34E-06 | -0.5032 | 224 | 0.000 |
| ENSP00000256861 | 4.34E-06 | -0.5036 | 0 | 0.000 |
| ENSP00000310490 | 2.99E-06 | -0.5036 | 0 | 0.093 |
| ENSP00000296777 | 6.79E-06 | -0.5037 | 0 | 0.557 |
| ENSP00000270458 | 3.18E-06 | -0.5037 | 0 | 0.114 |
| ENSP00000329539 | 4.74E-06 | -0.5044 | 0 | 0.716 |
| ENSP00000257974 | 4.95E-06 | -0.5049 | 0 | 0.145 |
| ENSP00000315654 | 2.92E-06 | -0.5053 | 0 | 0.095 |
| ENSP00000351884 | 3.01E-06 | -0.5079 | 0 | 0.152 |
| ENSP00000307822 | 4.96E-06 | -0.5084 | 0 | 0.438 |
| ENSP00000247879 | 6.39E-06 | -0.5093 | 0 | 0.492 |
| ENSP00000249501 | 5.88E-06 | -0.5101 | 0 | 0.842 |
| ENSP00000347646 | 3.11E-06 | -0.5108 | 0 | 0.655 |
| ENSP00000332737 | 4.14E-06 | -0.5112 | 0 | 0.485 |
| ENSP00000298440 | 5.13E-06 | -0.5132 | 0 | 0.452 |
| ENSP00000269080 | 4.36E-06 | -0.5146 | 0 | 0.128 |
| ENSP00000346401 | 2.39E-06 | -0.5149 | 0 | 0.243 |
| ENSP00000250838 | 4.17E-06 | -0.5160 | 163 | 0.000 |
| ENSP00000356331 | 1.39E-05 | -0.5166 | 242 | 0.833 |
| ENSP00000401966 | 3.40E-07 | -0.5175 | 0 | 0.000 |
| ENSP00000431914 | 1.57E-06 | -0.5193 | 0 | 0.000 |
| ENSP00000387523 | 1.32E-06 | -0.5196 | 0 | 0.000 |
| ENSP00000304127 | 3.65E-06 | -0.5226 | 0 | 0.074 |
| ENSP00000358783 | 3.99E-06 | -0.5228 | 0 | 0.461 |
| ENSP00000327585 | 4.70E-06 | -0.5232 | 158 | 0.000 |
| ENSP00000232766 | 3.93E-06 | -0.5237 | 0 | 0.598 |
| ENSP00000274400 | 3.54E-06 | -0.5241 | 0 | 0.784 |
| ENSP00000327611 | 3.29E-06 | -0.5254 | 0 | 0.057 |
| ENSP00000303325 | 5.86E-06 | -0.5254 | 0 | 0.620 |

| | | | | |
|---|---|---|---|---|
| ENSP00000331938 | 3.17E-06 | -0.5278 | 0 | 0.000 |
| ENSP00000277942 | 5.66E-06 | -0.5281 | 0 | 0.469 |
| ENSP00000271971 | 2.38E-06 | -0.5297 | 0 | 0.000 |
| ENSP00000215882 | 3.98E-06 | -0.5305 | 0 | 0.159 |
| ENSP00000265104 | 2.44E-06 | -0.5319 | 0 | 0.118 |
| ENSP00000218176 | 4.41E-06 | -0.5320 | 0 | 0.264 |
| ENSP00000351466 | 2.67E-06 | -0.5322 | 0 | 0.000 |
| ENSP00000354957 | 3.72E-06 | -0.5330 | 0 | 0.798 |
| ENSP00000227256 | 6.61E-06 | -0.5339 | 0 | 0.000 |
| ENSP00000257549 | 4.49E-06 | -0.5344 | 0 | 0.421 |
| ENSP00000316955 | 9.10E-07 | -0.5350 | 0 | 0.000 |
| ENSP00000312554 | 2.77E-06 | -0.5354 | 0 | 0.126 |
| ENSP00000267377 | 5.90E-06 | -0.5363 | 0 | 0.642 |
| ENSP00000381036 | 2.35E-06 | -0.5374 | 0 | 0.000 |
| ENSP00000301037 | 5.43E-06 | -0.5417 | 158 | 0.000 |
| ENSP00000226091 | 4.69E-06 | -0.5465 | 0 | 0.711 |
| ENSP00000225235 | 3.25E-06 | -0.5473 | 0 | 0.220 |
| ENSP00000240335 | 5.50E-06 | -0.5474 | 0 | 0.846 |
| ENSP00000428619 | 2.82E-06 | -0.5489 | 0 | 0.855 |
| ENSP00000005284 | 2.83E-06 | -0.5497 | 0 | 0.137 |
| ENSP00000262442 | 2.33E-06 | -0.5498 | 0 | 0.114 |
| ENSP00000359643 | 7.43E-06 | -0.5523 | 150 | 0.635 |
| ENSP00000396747 | 6.67E-06 | -0.5524 | 201 | 0.000 |
| ENSP00000306095 | 6.41E-07 | -0.5524 | 0 | 0.000 |
| ENSP00000246115 | 5.77E-06 | -0.5526 | 0 | 0.481 |
| ENSP00000246549 | 6.00E-06 | -0.5531 | 0 | 0.472 |
| ENSP00000249041 | 6.16E-06 | -0.5566 | 0 | 0.501 |
| ENSP00000373702 | 2.35E-06 | -0.5572 | 0 | 0.583 |
| ENSP00000221444 | 3.42E-06 | -0.5590 | 0 | 0.081 |
| ENSP00000386502 | 6.52E-06 | -0.5590 | 201 | 0.000 |
| ENSP00000330284 | 4.43E-06 | -0.5622 | 0 | 0.496 |
| ENSP00000445688 | 6.57E-06 | -0.5668 | 201 | 0.000 |
| ENSP00000355435 | 7.81E-07 | -0.5682 | 0 | 0.000 |
| ENSP00000471417 | 6.65E-06 | -0.5699 | 201 | 0.000 |
| ENSP00000444054 | 4.66E-07 | -0.5702 | 0 | 0.000 |
| ENSP00000240306 | 5.89E-06 | -0.5708 | 0 | 0.844 |
| ENSP00000263776 | 4.27E-06 | -0.5714 | 0 | 0.185 |
| ENSP00000241256 | 6.79E-06 | -0.5761 | 0 | 0.656 |
| ENSP00000386160 | 1.58E-06 | -0.5762 | 0 | 0.000 |
| ENSP00000269466 | 4.18E-06 | -0.5808 | 0 | 0.729 |
| ENSP00000301908 | 5.91E-06 | -0.5821 | 0 | 0.539 |
| ENSP00000234389 | 3.75E-06 | -0.5857 | 0 | 0.348 |
| ENSP00000287907 | 4.86E-06 | -0.5882 | 0 | 0.528 |
| ENSP00000367219 | 6.58E-07 | -0.5890 | 0 | 0.000 |
| ENSP00000271385 | 4.12E-06 | -0.5916 | 0 | 0.655 |
| ENSP00000291041 | 1.84E-06 | -0.5930 | 0 | 0.000 |

| | | | | |
|---|---|---|---|---|
| ENSP00000301532 | 1.07E-06 | -0.5931 | 0 | 0.000 |
| ENSP00000326232 | 4.50E-06 | -0.5938 | 214 | 0.000 |
| ENSP00000303096 | 3.97E-07 | -0.5944 | 0 | 0.000 |
| ENSP00000265440 | 2.08E-06 | -0.5956 | 0 | 0.000 |
| ENSP00000271751 | 4.61E-06 | -0.5964 | 0 | 0.259 |
| ENSP00000299727 | 5.32E-06 | -0.5975 | 0 | 0.553 |
| ENSP00000321419 | 6.03E-07 | -0.5987 | 0 | 0.000 |
| ENSP00000328215 | 5.49E-07 | -0.6033 | 0 | 0.000 |
| ENSP00000386195 | 3.80E-07 | -0.6068 | 0 | 0.000 |
| ENSP00000255008 | 4.70E-06 | -0.6076 | 0 | 0.516 |
| ENSP00000365451 | 1.48E-06 | -0.6081 | 0 | 0.000 |
| ENSP00000274643 | 2.75E-06 | -0.6089 | 0 | 0.659 |
| ENSP00000276616 | 2.60E-06 | -0.6091 | 0 | 0.000 |
| ENSP00000175756 | 4.19E-06 | -0.6100 | 0 | 0.617 |
| ENSP00000324913 | 8.94E-07 | -0.6115 | 0 | 0.000 |
| ENSP00000342697 | 4.89E-07 | -0.6116 | 0 | 0.000 |
| ENSP00000380155 | 1.99E-06 | -0.6119 | 0 | 0.000 |
| ENSP00000305424 | 6.26E-07 | -0.6119 | 0 | 0.000 |
| ENSP00000376633 | 4.62E-07 | -0.6137 | 0 | 0.000 |
| ENSP00000305877 | 5.02E-06 | -0.6139 | 0 | 0.540 |
| ENSP00000362133 | 7.13E-05 | -0.6165 | 0 | 0.208 |
| ENSP00000327875 | 4.46E-06 | -0.6180 | 0 | 0.513 |
| ENSP00000261891 | 2.68E-06 | -0.6184 | 0 | 0.000 |
| ENSP00000386959 | 2.14E-06 | -0.6198 | 0 | 0.000 |
| ENSP00000445383 | 3.59E-07 | -0.6211 | 0 | 0.000 |
| ENSP00000302437 | 4.42E-07 | -0.6211 | 0 | 0.000 |
| ENSP00000326259 | 7.71E-07 | -0.6245 | 0 | 0.000 |
| ENSP00000322435 | 4.51E-07 | -0.6254 | 0 | 0.000 |
| ENSP00000331545 | 1.16E-06 | -0.6256 | 0 | 0.000 |
| ENSP00000258930 | 3.19E-06 | -0.6258 | 0 | 0.276 |
| ENSP00000384563 | 2.31E-06 | -0.6266 | 0 | 0.000 |
| ENSP00000384434 | 3.25E-06 | -0.6310 | 0 | 0.000 |
| ENSP00000254101 | 2.74E-06 | -0.6421 | 0 | 0.000 |
| ENSP00000269878 | 3.08E-06 | -0.6480 | 0 | 0.221 |
| ENSP00000261383 | 2.26E-06 | -0.6481 | 0 | 0.108 |
| ENSP00000452401 | 6.53E-06 | -0.6527 | 158 | 0.000 |
| ENSP00000393889 | 5.35E-07 | -0.6560 | 0 | 0.000 |
| ENSP00000349719 | 9.06E-07 | -0.6597 | 0 | 0.000 |
| ENSP00000303151 | 7.83E-07 | -0.6625 | 0 | 0.000 |
| ENSP00000308764 | 4.69E-07 | -0.6667 | 0 | 0.000 |
| ENSP00000341291 | 4.41E-07 | -0.6696 | 0 | 0.000 |
| ENSP00000324769 | 1.64E-06 | -0.6726 | 0 | 0.000 |
| ENSP00000334115 | 1.92E-06 | -0.6733 | 0 | 0.000 |
| ENSP00000347418 | 7.63E-07 | -0.6735 | 0 | 0.000 |
| ENSP00000436004 | 8.70E-07 | -0.6742 | 0 | 0.000 |
| ENSP00000334441 | 8.14E-07 | -0.6760 | 0 | 0.000 |

| | | | | |
|---|---|---|---|---|
| ENSP00000313936 | 1.41E-06 | -0.6785 | 0 | 0.000 |
| ENSP00000431165 | 6.14E-06 | -0.6801 | 158 | 0.000 |
| ENSP00000367649 | 4.32E-07 | -0.6813 | 0 | 0.000 |
| ENSP00000363913 | 4.50E-06 | -0.6824 | 167 | 0.000 |
| ENSP00000228862 | 3.96E-06 | -0.6842 | 0 | 0.758 |
| ENSP00000304419 | 5.80E-07 | -0.6856 | 0 | 0.000 |
| ENSP00000307447 | 4.39E-06 | -0.6919 | 325 | 0.000 |
| ENSP00000310632 | 1.81E-06 | -0.6949 | 0 | 0.000 |
| ENSP00000255262 | 5.29E-06 | -0.6952 | 0 | 0.544 |
| ENSP00000373196 | 7.91E-07 | -0.6957 | 0 | 0.000 |
| ENSP00000305640 | 5.15E-07 | -0.6961 | 0 | 0.000 |
| ENSP00000368990 | 4.30E-06 | -0.7001 | 235 | 0.000 |
| ENSP00000330049 | 2.49E-06 | -0.7015 | 0 | 0.000 |
| ENSP00000341581 | 5.22E-07 | -0.7033 | 0 | 0.000 |
| ENSP00000432011 | 4.86E-07 | -0.7057 | 0 | 0.000 |
| ENSP00000331823 | 1.64E-06 | -0.7092 | 0 | 0.000 |
| ENSP00000389625 | 9.47E-07 | -0.7122 | 0 | 0.000 |
| ENSP00000350474 | 7.76E-07 | -0.7165 | 0 | 0.000 |
| ENSP00000346345 | 7.26E-06 | -0.7179 | 0 | 0.000 |
| ENSP00000441354 | 5.83E-07 | -0.7198 | 0 | 0.000 |
| ENSP00000305207 | 5.93E-07 | -0.7214 | 0 | 0.000 |
| ENSP00000325381 | 1.20E-06 | -0.7225 | 0 | 0.000 |
| ENSP00000325682 | 4.51E-07 | -0.7303 | 0 | 0.000 |
| ENSP00000256261 | 3.57E-06 | -0.7325 | 0 | 0.636 |
| ENSP00000244623 | 5.19E-07 | -0.7331 | 0 | 0.000 |
| ENSP00000326349 | 5.59E-07 | -0.7331 | 0 | 0.000 |
| ENSP00000304188 | 5.32E-07 | -0.7359 | 0 | 0.000 |
| ENSP00000357134 | 7.74E-07 | -0.7378 | 0 | 0.000 |
| ENSP00000386174 | 1.77E-06 | -0.7395 | 0 | 0.000 |
| ENSP00000355429 | 8.23E-07 | -0.7444 | 0 | 0.000 |
| ENSP00000342448 | 3.39E-07 | -0.7465 | 0 | 0.000 |
| ENSP00000355430 | 3.43E-07 | -0.7469 | 0 | 0.000 |
| ENSP00000344040 | 1.59E-06 | -0.7487 | 0 | 0.000 |
| ENSP00000310337 | 2.40E-06 | -0.7496 | 0 | 0.000 |
| ENSP00000328934 | 3.23E-07 | -0.7507 | 0 | 0.000 |
| ENSP00000386209 | 4.57E-07 | -0.7509 | 0 | 0.000 |
| ENSP00000367664 | 4.76E-07 | -0.7527 | 0 | 0.000 |
| ENSP00000302863 | 7.00E-07 | -0.7566 | 0 | 0.000 |
| ENSP00000347965 | 6.99E-07 | -0.7583 | 0 | 0.000 |
| ENSP00000317482 | 8.30E-07 | -0.7662 | 0 | 0.000 |
| ENSP00000369729 | 5.56E-07 | -0.7684 | 0 | 0.000 |
| ENSP00000435416 | 5.32E-07 | -0.7688 | 0 | 0.000 |
| ENSP00000334393 | 7.62E-07 | -0.7698 | 0 | 0.000 |
| ENSP00000409094 | 3.01E-07 | -0.7701 | 0 | 0.000 |
| ENSP00000353044 | 3.09E-07 | -0.7717 | 0 | 0.000 |
| ENSP00000386151 | 4.43E-07 | -0.7736 | 0 | 0.000 |

| | | | | |
|---|---|---|---|---|
| ENSP00000310773 | 9.41E-07 | -0.7755 | 0 | 0.000 |
| ENSP00000369728 | 1.04E-06 | -0.7760 | 0 | 0.000 |
| ENSP00000322088 | 4.63E-07 | -0.7768 | 0 | 0.000 |
| ENSP00000330338 | 6.29E-07 | -0.7782 | 0 | 0.000 |
| ENSP00000364164 | 3.74E-07 | -0.7785 | 0 | 0.000 |
| ENSP00000259357 | 5.26E-07 | -0.7800 | 0 | 0.000 |
| ENSP00000412752 | 3.67E-07 | -0.7836 | 0 | 0.000 |
| ENSP00000284287 | 5.23E-07 | -0.7852 | 0 | 0.000 |
| ENSP00000314324 | 5.75E-07 | -0.7857 | 0 | 0.000 |
| ENSP00000324958 | 5.12E-07 | -0.7881 | 0 | 0.000 |
| ENSP00000329056 | 7.07E-07 | -0.7888 | 0 | 0.000 |
| ENSP00000324557 | 1.22E-06 | -0.7910 | 0 | 0.000 |
| ENSP00000328144 | 5.85E-07 | -0.7920 | 0 | 0.000 |
| ENSP00000326301 | 3.27E-07 | -0.7942 | 0 | 0.000 |
| ENSP00000331789 | 9.14E-07 | -0.7950 | 0 | 0.000 |
| ENSP00000298642 | 5.59E-07 | -0.7953 | 0 | 0.000 |
| ENSP00000353537 | 4.28E-07 | -0.7963 | 0 | 0.000 |
| ENSP00000355431 | 7.88E-07 | -0.7984 | 0 | 0.000 |
| ENSP00000352710 | 7.23E-07 | -0.7987 | 0 | 0.000 |
| ENSP00000378516 | 4.42E-07 | -0.7993 | 0 | 0.000 |
| ENSP00000319511 | 5.03E-07 | -0.7995 | 0 | 0.000 |
| ENSP00000355432 | 7.15E-07 | -0.8028 | 0 | 0.000 |
| ENSP00000306657 | 4.58E-07 | -0.8043 | 0 | 0.000 |
| ENSP00000333684 | 6.70E-07 | -0.8046 | 0 | 0.000 |
| ENSP00000333196 | 3.88E-07 | -0.8050 | 0 | 0.000 |
| ENSP00000318878 | 7.41E-07 | -0.8057 | 0 | 0.000 |
| ENSP00000344101 | 8.55E-07 | -0.8060 | 0 | 0.000 |
| ENSP00000302199 | 4.84E-07 | -0.8062 | 0 | 0.000 |
| ENSP00000248073 | 4.71E-07 | -0.8078 | 0 | 0.000 |
| ENSP00000335596 | 3.76E-07 | -0.8084 | 0 | 0.000 |
| ENSP00000343521 | 5.51E-07 | -0.8100 | 0 | 0.000 |
| ENSP00000322724 | 3.42E-07 | -0.8102 | 0 | 0.000 |
| ENSP00000323354 | 3.47E-07 | -0.8112 | 0 | 0.000 |
| ENSP00000323982 | 3.61E-07 | -0.8122 | 0 | 0.000 |
| ENSP00000317357 | 8.60E-07 | -0.8138 | 0 | 0.000 |
| ENSP00000386138 | 7.99E-07 | -0.8152 | 0 | 0.000 |
| ENSP00000322784 | 4.65E-07 | -0.8159 | 0 | 0.000 |
| ENSP00000334418 | 4.09E-07 | -0.8176 | 0 | 0.000 |
| ENSP00000310704 | 3.72E-07 | -0.8186 | 0 | 0.000 |
| ENSP00000368989 | 2.79E-07 | -0.8194 | 0 | 0.000 |
| ENSP00000308270 | 5.85E-07 | -0.8202 | 0 | 0.000 |
| ENSP00000352604 | 4.30E-07 | -0.8212 | 0 | 0.000 |
| ENSP00000332185 | 4.57E-07 | -0.8213 | 0 | 0.000 |
| ENSP00000315047 | 4.36E-07 | -0.8214 | 0 | 0.000 |
| ENSP00000319601 | 7.41E-07 | -0.8222 | 0 | 0.000 |
| ENSP00000321729 | 6.05E-07 | -0.8226 | 0 | 0.000 |

| | | | | |
|---|---|---|---|---|
| ENSP00000354800 | 4.43E-07 | -0.8237 | 0 | 0.000 |
| ENSP00000340748 | 6.65E-07 | -0.8242 | 0 | 0.000 |
| ENSP00000332473 | 3.08E-07 | -0.8247 | 0 | 0.000 |
| ENSP00000323952 | 4.80E-07 | -0.8248 | 0 | 0.000 |
| ENSP00000346611 | 5.13E-07 | -0.8251 | 0 | 0.000 |
| ENSP00000401706 | 2.64E-07 | -0.8253 | 0 | 0.000 |
| ENSP00000345163 | 3.58E-07 | -0.8254 | 0 | 0.000 |
| ENSP00000368986 | 2.73E-07 | -0.8255 | 0 | 0.000 |
| ENSP00000353343 | 2.91E-07 | -0.8256 | 0 | 0.000 |
| ENSP00000420502 | 2.91E-07 | -0.8260 | 0 | 0.000 |
| ENSP00000369738 | 2.77E-07 | -0.8260 | 0 | 0.000 |
| ENSP00000259362 | 3.40E-07 | -0.8261 | 0 | 0.000 |
| ENSP00000355436 | 4.34E-07 | -0.8263 | 0 | 0.000 |
| ENSP00000343062 | 2.92E-07 | -0.8264 | 0 | 0.000 |
| ENSP00000334934 | 3.02E-07 | -0.8265 | 0 | 0.000 |
| ENSP00000436424 | 3.67E-07 | -0.8266 | 0 | 0.000 |
| ENSP00000350222 | 2.92E-07 | -0.8266 | 0 | 0.000 |
| ENSP00000302057 | 6.43E-07 | -0.8266 | 0 | 0.000 |
| ENSP00000377799 | 2.72E-07 | -0.8267 | 0 | 0.000 |
| ENSP00000307734 | 3.18E-07 | -0.8267 | 0 | 0.000 |
| ENSP00000302606 | 3.21E-07 | -0.8269 | 0 | 0.000 |
| ENSP00000322546 | 3.56E-07 | -0.8270 | 0 | 0.000 |
| ENSP00000373195 | 2.65E-07 | -0.8271 | 0 | 0.000 |
| ENSP00000334721 | 5.62E-07 | -0.8273 | 0 | 0.000 |
| ENSP00000362792 | 2.81E-07 | -0.8274 | 0 | 0.000 |
| ENSP00000369559 | 2.73E-07 | -0.8274 | 0 | 0.000 |
| ENSP00000404102 | 3.56E-07 | -0.8275 | 0 | 0.000 |
| ENSP00000334068 | 3.28E-07 | -0.8275 | 0 | 0.000 |
| ENSP00000323423 | 3.16E-07 | -0.8276 | 0 | 0.000 |
| ENSP00000335535 | 3.02E-07 | -0.8280 | 0 | 0.000 |
| ENSP00000335025 | 3.02E-07 | -0.8280 | 0 | 0.000 |
| ENSP00000321338 | 4.03E-07 | -0.8280 | 0 | 0.000 |
| ENSP00000342836 | 2.92E-07 | -0.8281 | 0 | 0.000 |
| ENSP00000333184 | 3.55E-07 | -0.8281 | 0 | 0.000 |
| ENSP00000318834 | 3.16E-07 | -0.8281 | 0 | 0.000 |
| ENSP00000322939 | 3.16E-07 | -0.8281 | 0 | 0.000 |
| ENSP00000352305 | 2.92E-07 | -0.8281 | 0 | 0.000 |
| ENSP00000367650 | 5.41E-07 | -0.8283 | 0 | 0.000 |
| ENSP00000347428 | 2.92E-07 | -0.8283 | 0 | 0.000 |
| ENSP00000373194 | 2.94E-07 | -0.8283 | 0 | 0.000 |
| ENSP00000285600 | 6.73E-07 | -0.8284 | 0 | 0.000 |
| ENSP00000432417 | 2.93E-07 | -0.8284 | 0 | 0.000 |
| ENSP00000348449 | 2.92E-07 | -0.8284 | 0 | 0.000 |
| ENSP00000348368 | 2.92E-07 | -0.8284 | 0 | 0.000 |
| ENSP00000346575 | 2.92E-07 | -0.8285 | 0 | 0.000 |
| ENSP00000283225 | 3.14E-07 | -0.8287 | 0 | 0.000 |

| | | | | |
|---|---|---|---|---|
| ENSP00000448811 | 2.71E-07 | -0.8288 | 0 | 0.000 |
| ENSP00000438815 | 2.71E-07 | -0.8288 | 0 | 0.000 |
| ENSP00000389072 | 2.71E-07 | -0.8289 | 0 | 0.000 |
| ENSP00000362784 | 2.81E-07 | -0.8289 | 0 | 0.000 |
| ENSP00000357126 | 2.81E-07 | -0.8289 | 0 | 0.000 |
| ENSP00000303834 | 7.04E-07 | -0.8290 | 0 | 0.000 |
| ENSP00000386180 | 2.74E-07 | -0.8290 | 0 | 0.000 |
| ENSP00000386208 | 2.71E-07 | -0.8291 | 0 | 0.000 |
| ENSP00000386137 | 2.71E-07 | -0.8291 | 0 | 0.000 |
| ENSP00000335529 | 3.02E-07 | -0.8292 | 0 | 0.000 |
| ENSP00000326225 | 3.16E-07 | -0.8292 | 0 | 0.000 |
| ENSP00000322593 | 3.16E-07 | -0.8295 | 0 | 0.000 |
| ENSP00000308689 | 3.18E-07 | -0.8295 | 0 | 0.000 |
| ENSP00000331774 | 4.19E-07 | -0.8297 | 0 | 0.000 |
| ENSP00000306607 | 3.19E-07 | -0.8298 | 0 | 0.000 |
| ENSP00000319217 | 3.16E-07 | -0.8298 | 0 | 0.000 |
| ENSP00000342008 | 2.92E-07 | -0.8299 | 0 | 0.000 |
| ENSP00000303864 | 3.19E-07 | -0.8300 | 0 | 0.000 |
| ENSP00000326718 | 3.33E-07 | -0.8300 | 0 | 0.000 |
| ENSP00000355443 | 6.16E-07 | -0.8300 | 0 | 0.000 |
| ENSP00000320560 | 3.76E-07 | -0.8302 | 0 | 0.000 |
| ENSP00000364152 | 2.82E-07 | -0.8303 | 0 | 0.000 |
| ENSP00000328878 | 3.14E-07 | -0.8304 | 0 | 0.000 |
| ENSP00000306974 | 3.19E-07 | -0.8304 | 0 | 0.000 |
| ENSP00000302422 | 3.21E-07 | -0.8304 | 0 | 0.000 |
| ENSP00000324369 | 3.24E-07 | -0.8306 | 0 | 0.000 |
| ENSP00000327525 | 3.15E-07 | -0.8306 | 0 | 0.000 |
| ENSP00000325078 | 3.75E-07 | -0.8307 | 0 | 0.000 |
| ENSP00000330904 | 3.14E-07 | -0.8310 | 0 | 0.000 |
| ENSP00000349930 | 2.98E-07 | -0.8311 | 0 | 0.000 |
| ENSP00000323928 | 3.16E-07 | -0.8311 | 0 | 0.000 |
| ENSP00000311688 | 3.90E-07 | -0.8311 | 0 | 0.000 |
| ENSP00000322823 | 3.16E-07 | -0.8312 | 0 | 0.000 |
| ENSP00000329467 | 6.25E-07 | -0.8313 | 0 | 0.000 |
| ENSP00000308714 | 3.18E-07 | -0.8313 | 0 | 0.000 |
| ENSP00000363911 | 5.02E-07 | -0.8313 | 0 | 0.000 |
| ENSP00000209540 | 3.45E-07 | -0.8313 | 0 | 0.000 |
| ENSP00000321447 | 3.16E-07 | -0.8314 | 0 | 0.000 |
| ENSP00000444134 | 4.11E-07 | -0.8314 | 0 | 0.000 |
| ENSP00000307751 | 3.59E-07 | -0.8315 | 0 | 0.000 |
| ENSP00000323606 | 3.21E-07 | -0.8317 | 0 | 0.000 |
| ENSP00000313110 | 5.47E-07 | -0.8317 | 0 | 0.000 |
| ENSP00000311038 | 3.18E-07 | -0.8319 | 0 | 0.000 |
| ENSP00000311477 | 3.18E-07 | -0.8319 | 0 | 0.000 |
| ENSP00000312208 | 3.18E-07 | -0.8319 | 0 | 0.000 |
| ENSP00000321246 | 4.06E-07 | -0.8319 | 0 | 0.000 |

| | | | | |
|---|---|---|---|---|
| ENSP00000324111 | 6.07E-07 | -0.8320 | 0 | 0.000 |
| ENSP00000324583 | 6.26E-07 | -0.8321 | 0 | 0.000 |
| ENSP00000318956 | 3.16E-07 | -0.8325 | 0 | 0.000 |
| ENSP00000259466 | 3.40E-07 | -0.8326 | 0 | 0.000 |
| ENSP00000322801 | 1.23E-06 | -0.8328 | 0 | 0.000 |
| ENSP00000334452 | 5.36E-07 | -0.8332 | 0 | 0.000 |
| ENSP00000279791 | 3.32E-07 | -0.8332 | 0 | 0.000 |
| ENSP00000297913 | 3.92E-07 | -0.8333 | 0 | 0.000 |
| ENSP00000320077 | 3.20E-07 | -0.8333 | 0 | 0.000 |
| ENSP00000348033 | 2.97E-07 | -0.8333 | 0 | 0.000 |
| ENSP00000324534 | 8.73E-07 | -0.8334 | 0 | 0.000 |
| ENSP00000437629 | 3.51E-07 | -0.8335 | 0 | 0.000 |
| ENSP00000305469 | 4.47E-07 | -0.8335 | 0 | 0.000 |
| ENSP00000303469 | 3.58E-07 | -0.8337 | 0 | 0.000 |
| ENSP00000307130 | 8.84E-07 | -0.8338 | 0 | 0.000 |
| ENSP00000331572 | 4.04E-07 | -0.8339 | 0 | 0.000 |
| ENSP00000305011 | 4.95E-07 | -0.8339 | 0 | 0.000 |
| ENSP00000321426 | 4.37E-07 | -0.8347 | 0 | 0.000 |
| ENSP00000329210 | 4.43E-07 | -0.8348 | 0 | 0.000 |
| ENSP00000303862 | 3.36E-07 | -0.8349 | 0 | 0.000 |
| ENSP00000329153 | 4.22E-07 | -0.8351 | 0 | 0.000 |
| ENSP00000351211 | 2.93E-07 | -0.8351 | 0 | 0.000 |
| ENSP00000305970 | 3.23E-07 | -0.8352 | 0 | 0.000 |
| ENSP00000319071 | 3.19E-07 | -0.8353 | 0 | 0.000 |
| ENSP00000325128 | 4.93E-07 | -0.8353 | 0 | 0.000 |
| ENSP00000248058 | 4.47E-07 | -0.8355 | 0 | 0.000 |
| ENSP00000325065 | 3.33E-07 | -0.8357 | 0 | 0.000 |
| ENSP00000300773 | 5.71E-07 | -0.8359 | 0 | 0.000 |
| ENSP00000307159 | 3.22E-07 | -0.8359 | 0 | 0.000 |
| ENSP00000299454 | 3.48E-07 | -0.8359 | 0 | 0.000 |
| ENSP00000320302 | 3.38E-07 | -0.8359 | 0 | 0.000 |
| ENSP00000319546 | 5.11E-07 | -0.8361 | 0 | 0.000 |
| ENSP00000277216 | 5.03E-07 | -0.8368 | 0 | 0.000 |
| ENSP00000318997 | 3.19E-07 | -0.8369 | 0 | 0.000 |
| ENSP00000322866 | 4.81E-07 | -0.8369 | 0 | 0.000 |
| ENSP00000311605 | 4.22E-07 | -0.8372 | 0 | 0.000 |
| ENSP00000368987 | 4.89E-07 | -0.8374 | 0 | 0.000 |
| ENSP00000321506 | 3.47E-07 | -0.8375 | 0 | 0.000 |
| ENSP00000322754 | 4.43E-07 | -0.8380 | 0 | 0.000 |
| ENSP00000307598 | 3.81E-07 | -0.8381 | 0 | 0.000 |
| ENSP00000303076 | 5.88E-07 | -0.8383 | 0 | 0.000 |
| ENSP00000304077 | 3.58E-07 | -0.8390 | 0 | 0.000 |
| ENSP00000324831 | 8.28E-07 | -0.8393 | 0 | 0.000 |
| ENSP00000332110 | 4.01E-07 | -0.8393 | 0 | 0.000 |
| ENSP00000369157 | 3.37E-07 | -0.8396 | 0 | 0.000 |
| ENSP00000321196 | 5.07E-07 | -0.8398 | 0 | 0.000 |

| | | | | |
|---|---|---|---|---|
| ENSP00000304807 | 8.49E-07 | -0.8399 | 0 | 0.000 |
| ENSP00000299459 | 3.50E-07 | -0.8407 | 0 | 0.000 |
| ENSP00000325076 | 4.30E-07 | -0.8416 | 0 | 0.000 |
| ENSP00000369731 | 3.67E-07 | -0.8418 | 0 | 0.000 |
| ENSP00000322156 | 3.51E-07 | -0.8419 | 0 | 0.000 |
| ENSP00000353988 | 9.82E-07 | -0.8421 | 0 | 0.000 |
| ENSP00000327540 | 4.77E-07 | -0.8430 | 0 | 0.000 |
| ENSP00000301529 | 3.98E-07 | -0.8430 | 0 | 0.000 |
| ENSP00000369568 | 3.89E-07 | -0.8432 | 0 | 0.000 |
| ENSP00000353516 | 7.01E-07 | -0.8440 | 0 | 0.000 |
| ENSP00000324687 | 3.93E-07 | -0.8440 | 0 | 0.000 |
| ENSP00000386222 | 1.40E-06 | -0.8442 | 0 | 0.000 |
| ENSP00000323224 | 6.39E-07 | -0.8443 | 0 | 0.000 |
| ENSP00000308082 | 5.31E-07 | -0.8446 | 0 | 0.000 |
| ENSP00000352626 | 5.36E-07 | -0.8448 | 0 | 0.000 |
| ENSP00000330280 | 3.65E-07 | -0.8452 | 0 | 0.000 |
| ENSP00000319197 | 3.16E-07 | -0.8461 | 0 | 0.000 |
| ENSP00000357127 | 5.46E-07 | -0.8467 | 0 | 0.000 |
| ENSP00000332500 | 7.09E-07 | -0.8474 | 0 | 0.000 |
| ENSP00000319322 | 8.39E-07 | -0.8477 | 0 | 0.000 |
| ENSP00000291231 | 5.57E-07 | -0.8480 | 0 | 0.000 |
| ENSP00000289451 | 4.81E-07 | -0.8490 | 0 | 0.000 |
| ENSP00000312470 | 3.35E-07 | -0.8498 | 0 | 0.000 |
| ENSP00000305055 | 4.53E-07 | -0.8499 | 0 | 0.000 |
| ENSP00000345563 | 4.65E-07 | -0.8500 | 0 | 0.000 |
| ENSP00000306651 | 7.27E-07 | -0.8502 | 0 | 0.000 |
| ENSP00000349945 | 3.51E-07 | -0.8506 | 0 | 0.000 |
| ENSP00000278409 | 4.01E-07 | -0.8506 | 0 | 0.000 |
| ENSP00000308595 | 4.13E-07 | -0.8517 | 0 | 0.000 |
| ENSP00000319654 | 5.77E-07 | -0.8522 | 0 | 0.000 |
| ENSP00000248384 | 5.08E-07 | -0.8540 | 0 | 0.000 |
| ENSP00000306688 | 4.24E-07 | -0.8550 | 0 | 0.000 |
| ENSP00000303822 | 5.34E-07 | -0.8566 | 0 | 0.000 |
| ENSP00000324251 | 6.57E-07 | -0.8573 | 0 | 0.000 |
| ENSP00000312403 | 7.93E-07 | -0.8599 | 0 | 0.000 |
| ENSP00000310788 | 4.83E-07 | -0.8609 | 0 | 0.000 |
| ENSP00000329689 | 4.87E-07 | -0.8685 | 0 | 0.000 |
| ENSP00000310488 | 7.10E-07 | -0.8745 | 0 | 0.000 |
| ENSP00000252835 | 5.53E-07 | -0.8761 | 0 | 0.000 |
